# Insight of a Metabolic Prognostic Model to Identify Tumor Environment and Drug Vulnerability for Lung Adenocarcinoma

**DOI:** 10.3389/fimmu.2022.872910

**Published:** 2022-06-23

**Authors:** Shun-Li Peng, Rong Wang, Yu-Ling Zhou, Wei Wei, Gui-Hua Zhong, Xiao-Tao Huang, Shuai Yang, Qiao-Dan Liu, Zhi-Gang Liu

**Affiliations:** ^1^ The Cancer Center of the Fifth Affiliated Hospital of Sun Yat-sen University, Zhuhai, China; ^2^ Guangdong Provincial Key Laboratory of Biomedical Imaging, The Fifth Affiliated Hospital of Sun Yat-sen University, Zhuhai, China

**Keywords:** metabolic signature, prognostic model, radiosensitivity, immune response, non-small cell lung cancer

## Abstract

Metabolic reprogramming is a novel method for the treatment of malignant tumors. The exploration of metabolism procedures between radiosensitive and radioresistant tumors may provide novel perspectives for lung adenocarcinoma (LUAD) patients after radiation therapy. In our study, metabolic reprogramming and immune response changes were found between radioresistant cell line (A549RR) and its parent cells (A549) using gene ontology and Kyoto Encyclopedia of Genes and Genomes (KEGG) pathway analysis. Nucleotide/amino acid, lipid, and glucose metabolic process, including Alanine, aspartate and glutamate metabolism, Tryptophan/Tyrosine metabolism, Butanoate metabolism, Purine/Pyrimidine metabolism, were screened out. Then molecular signatures database and The Cancer Genome Atlas Program (TCGA) lung adenocarcinoma datasets were used to identify metabolism-related genes (MRGs) between radiosensitive and radioresistant lung adenocarcinoma (LUAD) cells. A metabolism-based prognostic model, receiver operating characteristic (ROC) curve and nomogram were constructed using Metabolism Score calculated by 14 metabolism-related genes (MRGs). Three independent public datasets, (GSE72094, GSE3141, GSE8894) and one immunotherapy cohort (IMvigor210) were used as external validation cohorts. Expression of 14 hub genes in cells, normal and LUAD specimens were explored by Human Protein Atlas, TIMER2.0 and RT-qPCR. Patients with low-Metabolism Scores were correlated with longer survival times, higher response rates to immune checkpoint inhibitors (ICIs), different immune cell infiltrations and drug vulnerability. Our study demonstrated a comprehensive landscape between radiosensitive and radioresistant LUAD, and provide novel targets for NSCLC, especially those patients received radiation therapy. Moreover, this metabolism-based prognostic model may help to investigate connections between radiosensitivity, immune response, metabolic reprogramming, and patients’ prognosis.

## Introduction

As an important local treatment technique, radiation therapy (RT) is recommended as a standard treatment (1, 2) for locally advanced unresectable recurring and metastatic non-small lung cancer (NSCLC) (3, 4). Unfortunately, not all patients could achieve complete response after standard first-line treatment, especially those with local advanced and metastatic NSCLC (5, 6). Owing to the emergence of radiation resistance and the tolerant dose range of normal tissue, re-radiation for residual and relapsed tumors that have received RT before is cautiously. Therefore, choosing an appropriate treatment scheme for NSCLC patients after RT is a practical clinical problem. Despite a series of pre-clinical research and clinical trials have been develop to explore novel strategies for NSCLC, there has been a lack of illustration on metabolic changes before and after radiation.

During the process of tumorigenesis and development, heterogeneous metabolic phenotypes develop in tumor cells to withstand complex challenges (7). Therefore, metabolic reprogramming has been found to provide novel insights for the treatment of malignant tumors (8). As a key enzyme in the uronic acid pathway, Uridine diphosphate (UDP)-glucose 6-dehydrogenase (9) has been reported to promote metastasis of lung cancer through converting UDP-glucose to UDP-glucuronic acid. Glycolysis flux becomes an important therapeutic target for those glucose-dependent cancers (10). Meanwhile, increasing dependence on glutamine was found to promote growth and progression of normal airway epithelial cells and non-small cell lung cancer (NSCLC) (11, 12). Those studies indicates that there is a close relationship between metabolism reprograming and malignant biological behavior of tumors. Previous studies have shown that targeting glucose and hydroperoxide metabolism (13) were important strategies to improve radiation response of cancer cells. Meanwhile, lipid oxidation and ferroptosis (14) were reported to involved in radiotherapy efficacy. Our study found that there are great differences in DNA binding transcription, metabolic process, and immune response between radiosensitive and radioresistant lung adenocarcinoma (LUAD) cells. Moreover, we constructed a protein-protein interaction regulator network to visualize the regulator network. These findings may illustrate a comprehensive molecular landscape between radiosensitive and radioresistant LUAD, and may provide a series of molecules and pathways for the treatment of NSCLC, especially for those recurrence and metastatic LUAD after radiation therapy.

Alterations in the tumor environmental metabolic characteristics also affect treatment outcomes (15). The determination of the abscopal effect helps pave the way for combinations of RT with immunotherapy (16). Adequate metabolic alterations between tumor and the microenvironment were reported to format a reciprocal regulator model involving host immune cells and microbiota (17). Therefore, clarifying potential connection between tumor and environment may also provide novel insights for treatments of NSCLC. In our study, we explored potential connections between metabolism reprogramming, immune cell infiltrations, immune checkpoint inhibitors (ICIs) response, drug vulnerability and radiosensitivity, and provide a metabolism-based prognostic model and novel targets for NSCLC from the perspective of metabolism reprogramming.

## Methods

### Construction of Radioresistant Cells and Cell Culture

A549 cells were intermittently exposed to 6 Gy X-ray to establish radioresistant cells A549RR according to the method previously described (18). A radioresistant cell line (A549RR) and its parent cell (A549) were cultured in RPMI-1640 medium supplemented with 10% foetal bovine serum (FBS; Gibco, NY, USA) in a standard environment as described previously (19).

### Bioinformatics Mining Between Radiosensitive and Radioresistant Lung Adenocarcinoma Cells

A549 and A549RR cells were collected and determined by RNA sequencing to obtain gene expression profiling at the level of the transcriptome (Shanghai Genechem Co., Ltd., China). Then, differentially expressed genes (DEGs) between radioresistant (A549RR) and parent cells(A549) were identified as found in **Supplementary Table S1** (log2FC ≥ 1, and p < 0.05). The Kyoto Encyclopedia of Genes and Genomes (KEGG) pathways analysis of DEGs was performed with the cluster Profiler R package (20) (http://bioconductor.org/packages/release/bioc/html/clusterProfiler.html), and metabolic pathway was the top pathway with 294 DEGs enriched (**Supplementary Table S2**, p < 0.05). To further explore the metabolic changes, the above 294 DEGs were further performed by KEGG pathways analysis, and significant pathway were divided and mapped according to nucleotide, amino acid, lipid, and glucose metabolism.

Gene ontology (GO) and immune system process were analyzed and visualized with Cytoscape ClueGO (**Supplementary Table S3**, two-sided hypergeometric test, adjusted p-value < 0.05 corrected with the Benjamini-Hochberg procedure) and R package circlize. In addition, a protein-protein interaction (PPI) network (21) was constructed with STRING database analysis (22) to evaluate interactive associations among all the DEGs (**Supplementary Table S4**, **Supplementary Figure S1**).

### Construction of Metabolism-Based Prognostic Model and Metabolism Score

The list of 944 metabolism-related genes (MRGs) was downloaded from the Molecular Signatures Database (https://www.gsea-msigdb.org/gsea/msigdb/index.jsp). And 192 common genes were screened out based on the overlap between ‘MRGs’ and ‘DEGs’ (**Supplementary Table S5**). Then univariate Cox regression model was further used to identify 53 survival-related MRGs (p < 0.05, **Supplementary Table S6**) based on 502 lung adenocarcinoma (LUAD) expression profiles with clinical characteristics (TCGA database, https://portal.gdc.cancer.gov/). In addition, lasso regression was used to construct a prognostic model to calculate risk score based on the above survival-related MRGs (HR < 0.75 or HR > 1.25 and p < 0.05), and the risk score was named as Metabolism Score. In this study, 14 hub gene were involved, and Metabolism Score = mRNA_gene1_ × coefficients_gene1_ + mRNA_gene2_ × coefficients_gene2_ + mRNA_gene3_ × coefficients_gene3_ +…+ mRNA_gene14_ × coefficients_gene14_.

502 LUAD patients were divided into two groups according to the median value of Metabolism Score (**Supplementary Table S7**), and Kaplan-Meier method was used to evaluate the availability of this prognostic model. The receiver operating characteristic (ROC) curve was used to test the measurement of classifications, and clinic correlations were analysed using the pheatmap R package (http://bioconductor.org/packages/release/bioc/html/heatmaps.html; **Supplementary Table S8**; **Supplementary Figure S2**). A Metabolism Score assessment nomogram was also established to evaluate prognosis in LUAD patients (1-, 3- and 5-year survival rates).

### The Validation of Metabolism-Based Prognostic Model and Metabolism Score

Gene Expression Omnibus (https://www.ncbi.nlm.nih.gov/geo/) was used to download the clinical information and gene expression data of three public lung cancer datasets (GSE72094, GSE3141 and GSE8894). Metabolism Score of specimens were calculated by metabolism-based prognostic model, and Kaplan-Meier and Chi-squared analysis were used to evaluate survival and immune response status. Two public websites, Human Protein Atlas (https://www.proteinatlas.org/) and TIMER2.0 (http://timer.cistrome.org/), were used to analyzed expression of 14 hub genes in normal and LUAD tissues.

### Evaluation of the Immune Microenvironment Based on Metabolism Score

CIBERSORT and ssGSEA algorithm were used to discriminate immune cell phenotypes in LUAD. The corrplot and ggplot R packages were used to map the distribution of immune cells (https://stackoverflow.com/questions/14753344/corrplot-parameters-in-r, **Supplementary Table S9**). ESTIMATE scores were calculated to estimate stromal and immune cells by tumor purity score and immune score (https://bioinformatics.mdanderson.org/estimate/rpackage.html, **Supplementary Table S10**), and a higher score reflected a larger ratio of the corresponding component in the tumor microenvironment. The correlation between ESTIMATE and Metabolism Scores was evaluated by Spearman’s analysis (p < 0.05). Tumor Immune Dysfunction and Exclusion (TIDE) (http://tide.dfci.harvard.edu/) and urothelial cancer (mUC) immune checkpoint cohort (IMvigor210) were used to evaluate response to immunotherapy (**Supplementary Table S11**). The genomic, transcriptomic, with matched clinical information of IMvigor210 cohort were downloaded under the Creative Commons 3.0 license (http://research-pub.gene.com/IMvigor210CoreBiologies). Chi-squared analysis was used to analyzed the difference between the high- and low-Metabolism Score groups. Statistical significance was set as p < 0.05.

To explore differences of tumor environment between high- and low-Metabolism Score groups, immune-related genes (IRGs) (https://www.immport.org/shared/home) were downloaded from the ImmPort database, and the limma package (http://bioconductor.org/packages/release/bioc/html/limma.html) was used for the extraction of differently expressed immune-related genes (DEIRGs). False discovery rate (FDR), p-value < 0.05, filter of 0.05 and log (fold change) filter ≥ 0.58 were applied as the threshold to select DEIRGs (**Supplementary Table S12**). Finally, DEIRGs were drawn with ggpubr (https://www.rdocumentation.org/packages/ggpubr/versions/0.1.4).

### Associations Between Metabolism Score and Drug Sensitivity

CellMiner (https://discover.nci.nih.gov/cellminer/) was used to explore transcript and drug patterns in the NCI-60 cell line set developed by the Developmental Therapeutics Program of the US National Cancer Institute. The associations between gene expressions and drug susceptibility were determined using the corrplot R package with Spearman’s method (p < 0.05, **Supplementary Table S13**, https://stackoverflow.com/questions/14753344/corrplot-parameters-in-r).

### Western Blot and Quantitative Real-Time PCR

Extraction of total protein and RNA were performed according to standard protocols (19, 23). The primary antibodies used in this study included CPS1 (Proteintech, #18703-1-AP), AOX1 (Proteintech, # 19495-1-AP), OXCT1 (Proteintech, #12175-1-AP), NME4 (Bioworld, #BS71176) and β-Actin (CST, #4967). Oligonucleotide primers used for detection of human-TRDMT1, SMS, UAP1, WARS2, PLOR3G, NNT, GAPDH (21) were described in **Supplementary Figure S3**. Cycle threshold (Ct) values of target gene cDNA were normalized to GAPDH using the −2ΔΔCt method. All the reactions were performed in triplicate for each sample.

### Statistical Analysis

For between-group comparisons, the p-value was calculated with unpaired Student’s t-tests. And for non-normally distributed variables, the p-value was calculated with Mann-Whitney U tests. Statistical significance was set as p < 0.05. FDR, and Benjamini-Hochberg were used for multiple tests to correct the p-value in DEGs, KEGG and GO analysis. The Kaplan-Meier method was used for generation of survival curves, while the log-rank (Mantel-Cox) test was used to evaluate differences; statistical significance was determined to be p < 0.05. The predicted response to immunotherapy was statistically analysed with Chi-squared test between high- and low-Metabolism Score groups (p < 0.05).

## Results

### Metabolism Reprogramming Characterisation in Radioresistant Lung Adenocarcinoma Cells

**Figure 1:**
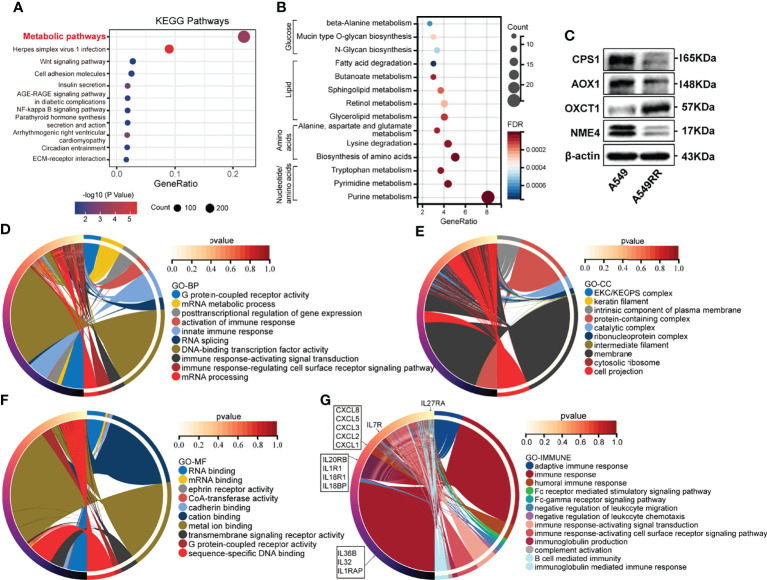
The function enrichment of differentially expressed genes (DEGs) between radioresistant (A549RR) and its parent cells (A549). **(A, B)** Results of Kyoto Encyclopedia of Genes and Genomes (KEGG) pathway enrichment (p < 0.05). The color of the node was counted by p-value, and more significant enrichment was shown with greater node size. **(C)** Expression of metabolic-related protein in A549 and A549RR cells measured by western blot. **(D–F)** Results of gene ontology, including biological process (BP), molecular function (MF), and cellular component (CC) were mapped using R package circlize (p < 0.05). Among the groups, the representative term and lag were highlighted by different colors. **(G)** Differences immune system process and related genes were mapped using R package circlize. The representative term and lag were highlighted by different colors. (p < 0.05).

To evaluate differences before and after radiation, A549 cells were exposed to radiation intermittently to establish the radio-resistant cell A549RR, according to the method previously described (18). Then A549RR and its parent cells, A549, were collected and measured using RNA sequencing to clarify the differences between radioresistant and radiosensitive NSCLC cells. As shown in **Supplementary Table S1**, a total of 3694 DEGs between A549 and A549RR cells were identified with log_2_FC ≥ 1 and FDR < 0.05. Then, KEGG pathway analysis was performed to explore changes in signal pathways (**Supplementary Table S2**), and 294 DEGs were closely associated with the TOP1 metabolic pathway (**Figure 1A**). To further explore the metabolic process in detail, 294 DEGs were performed by KEGG analysis, and a series of nucleotide, amino acid, lipid, glucose metabolic pathways were screened out (**Figure 1B**). In addition, we further detected expression of rate limiting enzymes (CPS1, AOX1, OXCT1, NME4) that enriched in Alanine, aspartate and glutamate metabolism, Tryptophan/Tyrosine metabolism, Butanoate metabolism, Purine/Pyrimidine metabolism in A549 and A549RR cells by western blot (**Figure 1C**). Those results indicate metabolism reprogramming in LUAD cells after acquire radiation resistance.

Meanwhile, a series of DEGs were enriched in cancer-related pathways (Wnt pathway, NF-kappa B pathway, ECM-receptor interaction, PPAR pathway, etc.) (**Figure 1A**). Transcription factors (NF-kappa B) (24) was found to be activated by ionizing radiation (IR) and may promote resistance to RT. Wnt pathway (25) was found to be involved in radio-resistance by promoting DNA damage repair. Furthermore, the crosstalk (26) between PPAR and canonical WNT/β-catenin pathway has been deeply clarified during the process of carcinogenesis. Those results indicated potential radioresistant mechanism of A549RR cells.

### Comprehensive Molecular Characterisation of Radioresistant LUAD Based on Gene Ontology Enrichment Analysis and Protein-Protein Interaction Regulator Network

To explore the relationship between DEGs and the malignant phenotype of LUAD, GO enrichment analysis and PPI network were performed. As shown in **Figures 1D-F** and **Supplementary Table S3**, DEGs are mainly distributed in nucleic acids transcriptional regulation and immune response process according to BP enrichment analysis. Furthermore, the results of the cellular component (CC) screened out a series of DEGs related to the intrinsic component of the plasma membrane, plasma membrane-bounded cell projection, and extracellular space. Moreover, DEGs distributed in RNA binding, CoA-transferase activity, cadherin binding, transmembrane pathway receptor activity, and DNA-binding activity were found through MF analysis.

Among the above GO results, DEGs involved in immune response processes were frequently screened out, indicating potential immune-related changes between A549 and A549RR cells. Therefore, the immune system process was investigated and mapped, including humoral immune response, Fc receptor-mediated stimulatory signalling pathway, negative regulation of leukocyte chemotaxis, immune response-activating signal transduction, B cell-mediated immunity and immunoglobulin-mediated immune response (**Figure 1G**). These results indicated a different immune status of NSCLC after acquired radio-resistance.

To further explore the potential molecular regulator network in radioresistant LUAD cells, DEGs were uploaded to STRING (22) to establish the PPI network (**Supplementary Table S4** and **Supplementary Figure S1**). Among the regulator network, a series of DEGs containing high experimentally determined interaction scores (> 0.9) with high combined scores (> 0.9) and high co-expression coefficients (> 0.9) were screened out, including PSMA1 and PSMA2, RPL9 and RPS26, RPL9 and RPS28, BYSL and PNO1, POLA1 and PRIM2, RPS26 and RPS28, RPL9 and RPS10, RPS10 and RPS26, FGA and FGB, PSMB8 and PSMB9, RPS10 and RPS28, UTP15 and WDR43 as well as NDUFB8 and NDUFS7. These protein molecules with high interaction scores could provide target candidates for radioresistant NSCLC, especially for recurrent and metastatic NSCLC after received RT.

### Optimisation of the Metabolism-Based Prognostic Model for LUAD

Previous studies (7) have shown that metabolism reprogramming could promote malignant phenotypes of tumor cells. However, only a few studies explored the differences between radiosensitive and radioresistant lung cancers. Metabolic changes in radioresistant lung cancers remain to be clarified. Since metabolic pathways have been screened out in previous results (**Figures 1A-C**), we established a metabolic signature-based prognostic model to explore the connection between metabolic reprogramming and prognosis of NSCLC.

**Figure 2:**
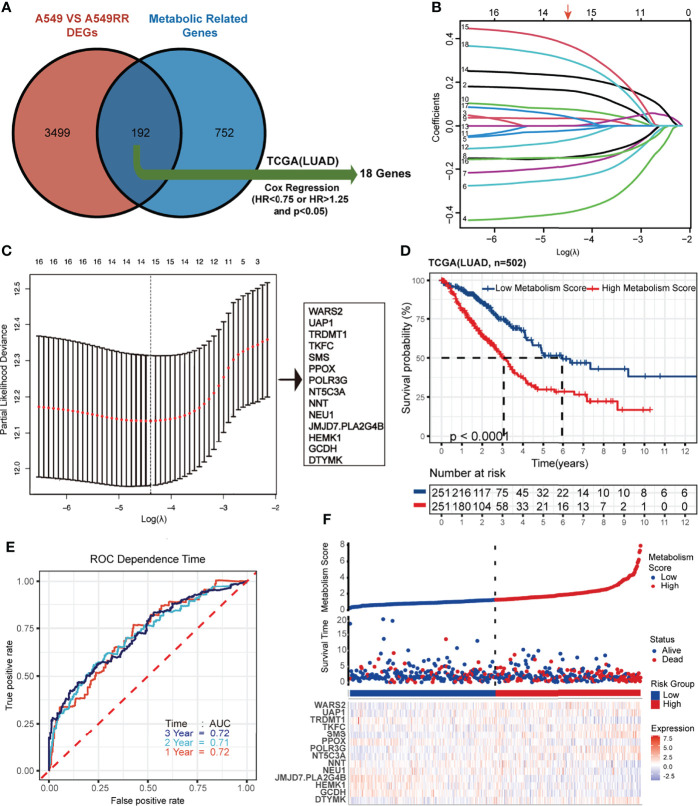
Construction and verification of Lasso regression based on metabolic-related DEGs (MRGs). **(A-C)** construction process based on lasso regression. 192 common genes in DEGs and MRGs were mapped by Venn plot, and 18 genes were screened out using Cox regression based on the TCGA database (lung adenocarcinoma, LUAD). Then lasso regression complexity was controlled by lambda using the glmnet R package, and a metabolic-related prognostic model was constructed to calculate Metabolism Scores of tumors based on expression level of 14 hub genes. **(D)** Overall survival status of LUAD patients (N=502) with high and low Metabolism Score using Kaplan–Meier plotter analysis. **(E)** ROC curve based on Metabolism Score using metabolic-related prognostic model. **(F)** LUAD patients with survival status (middle) were ranked according to Metabolic Score (top), and expression levels genes were plotted with a heat map (below).

A total of 944 MRGs were downloaded from the molecular signatures database, and 192 genes were obtained based on the overlap of MRGs and previously identified DEGs (**Supplementary Tables S5**; **Figure 2A**). Then, univariate Cox regression analysis was used (**Supplementary Tables S6**), and 18 MRGs were selected with a threshold of HR < 0.75 or HR > 1.25 for further study. Finally, lasso regression analysis indicated that best prognostic model was contributed when log (lambda) was between -4 and -5, and the Metabolism Score was calculated based on 14 hub genes (*DTYMK, GCDH, HEMK1, JMJD7.PLA2G4B, NEU1, NNT, NT5C3A, POLR3G, PPOX, SMS, TKFC, TRDMT1, UAP1 and WARS2*) (**Supplementary Table S7**; **Figures 2B, C**).

### Metabolism Score Possibly Acts as an Independent Risk Factor in LUAD

Using the above 14 MRG-based prognostic model, LUAD specimens were divided into high- and low-Metabolism Score groups (**Supplementary Table S7**). As shown in **Figure 2D**, patients with high-Metabolism Scores seemed to have shorter overall survival times than those with low-Metabolism Scores. In subgroup of patients who received radiation therapy, similar results were found in **Supplementary Figure S2A**. Additionally, ROC curve was calculated with area under the curve (AUC) = 0.72 in 1-3 years (**Figure 2E**; **Supplementary Figure S2B**). The risk score map of LUAD patients including Metabolic Score and expression of 14 hub genes (DTYMK, GCDH, HEMK1, JMJD7.PLA2G4B, NEU1, NNT, NT5C3A, POLR3G, PPOX, SMS, TKFC, TRDMT1, UAP1 and WARS2) was showed in **Figure 2F**.

**Figure 3:**
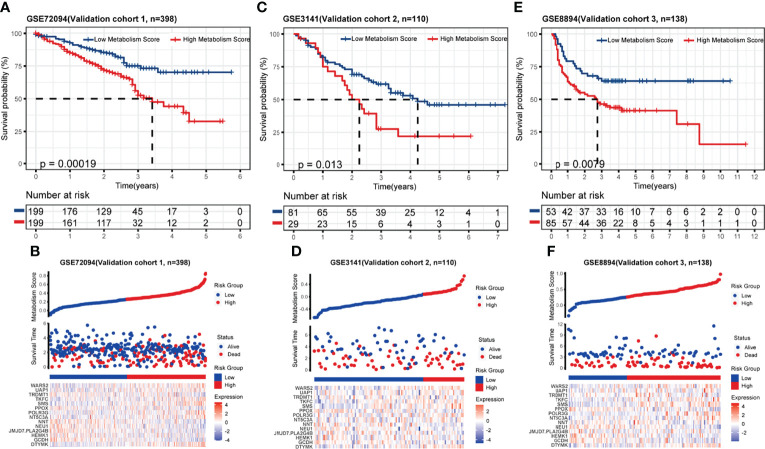
Validation of metabolic-related prognostic model. **(A-F)** Survival status, risk and heatmap of NSCLC patients in low- and high- Metabolism Score subgroups based on 3 independent public datasets, three public datasets (GSE72094, lung adenocarcinoma, N=398; GSE3141, lung cancer, N=110; GSE8894, lung cancer, N=138).

To test the accuracy and universality of this metabolic-based model, three public datasets (GSE72094, lung adenocarcinoma, N=398; GSE3141, lung cancer, N=110; GSE8894, lung cancer, N=138) were performed as validation cohorts. As shown in **Figures 3A-F**, the survival curve, status risk and heatmap of patients with low- and high- Metabolism Score were mapped. Patients with high Metabolism Scores seems to have shorter survival time and higher survival risk than those with low Metabolism Scores (**Figures 3A-F**). These results indicated the successful establishment of a stable predictive model with a metabolic signature and good accuracy.

### The Association Between Metabolism Score and Clinical Characteristics

**Figure 4:**
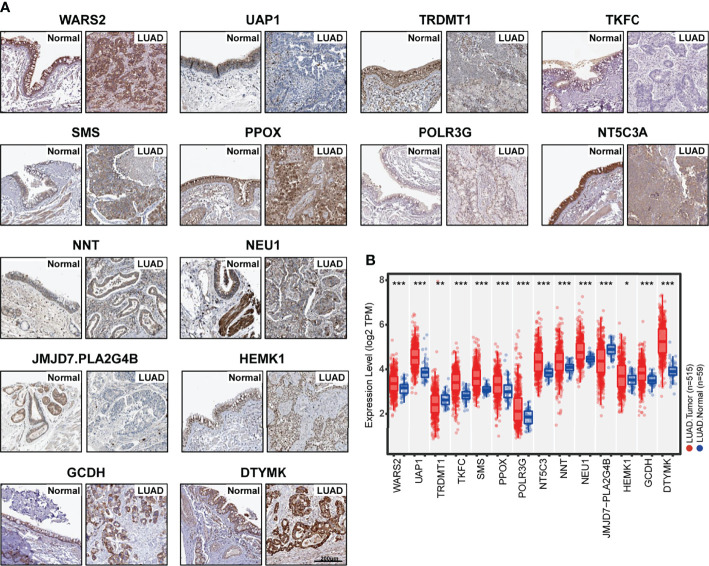
Expression of 14 hub genes in normal and lung adenocarcinoma tissues. **(A)** Protein expression level and localization of 14 hub genes in normal bronchus tissue and lung adenocarcinoma specimens measured by IHC staining based on Human Protein Atlas (bars = 200 μm). **(B)** mRNA expression levels of 14 hub genes in normal (N=59) and lung adenocarcinoma (N=515) tissues were mapped with boxplots based on TIMER 20. and TCGA database. *p < 0.05, **p < 0.01 and ***p < 0.001.

Since the Metabolism Score was calculated by expression level of 14 hub genes (DTYMK, GCDH, HEMK1, JMJD7.PLA2G4B, NEU1, NNT, NT5C3A, POLR3G, PPOX, SMS, TKFC, TRDMT1, UAP1 and WARS2), we further evaluated expression level and localization of the above 14 genes using two public websites, Human Protein Atlas, TIMER 2.0 (**Figures 4A, B**). As shown in **Figure 4B**, TRDMT1 and JMJD7.PLA2G4B genes were downregulated, while the other 10 genes were significantly overexpressed in lung adenocarcinomas compared with normal tissues. Meanwhile, TRDMT1 were upregulated in A549RR cells, while SMS, UAP1, WARS2, PLOR2G and NNT expression were decreased measured by RT-qPCR (**Supplementary Figure S3**).

**Figure 5:**
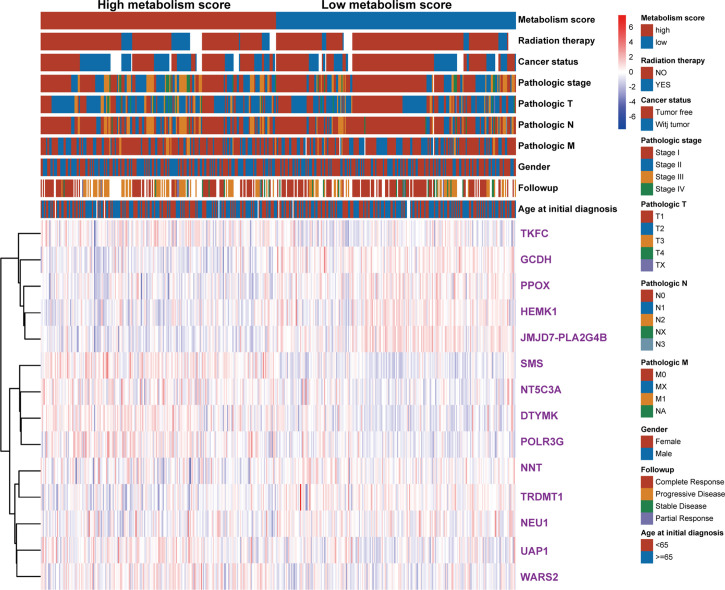
The heat map of clinical characteristics and gene expression level between high- and low-Metabolism Score groups. LUAD specimens were divided into high- and low-Metabolism Score groups, clinical characteristics (age_at_initial_diagnosis, follow-up results, gender, pathologic_M, pathologic_N, pathologic_T, pathologic_stage, cancer_status, radiation_therapy, survival time and survival status), and 14 MRGs expression levels were mapped.

**Figure 6:**
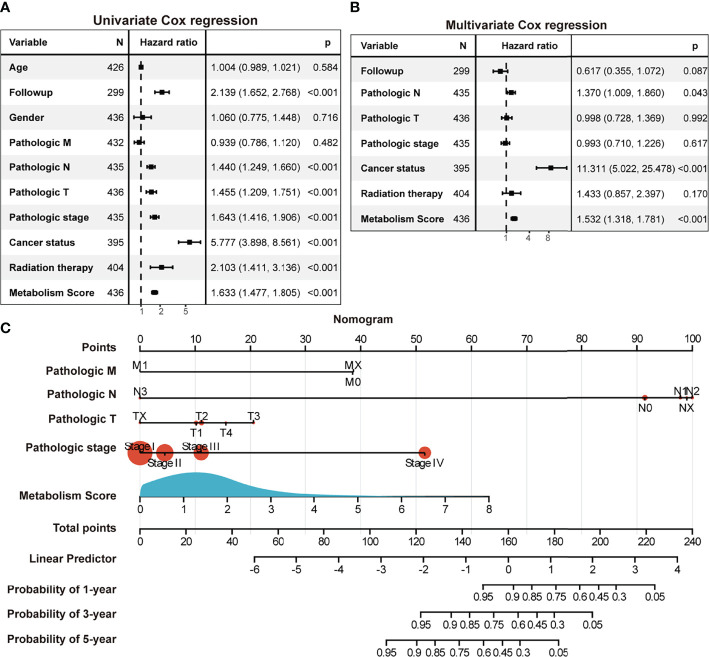
The Metabolism Score based on a metabolic prognostic model possibly acted as an independent risk factor in LUAD. **(A)** Univariate Cox regression analysis of risk factors in LUAD. **(B)** Multivariate Cox regression analysis of risk factors in LUAD. **(C)** Assessment nomogram with Metabolism Score to evaluate prognosis of LUAD (1-, 3- and 5-year survival rates).

To further explore the relationship between Metabolism Score, clinical characters, and survival risk of LUAD patients, heatmap were performed in **Figure 5**. And the results of univariate and multivariate cox regression indicated that follow-up, pathologic N, pathologic T, pathologic stage, cancer status, radiation therapy and Metabolism Score were significantly correlated with overall survival of LUAD patients (**Figures 6A, B**), which indicates that Metabolism Score may act as independent risk factors for LUAD. In addition, a nomogram was made to provide a simple and convenient method for clinical application in the treatment of NSCLC (**Figure 6C**).

### Exploration Between Metabolism Score and Immune Microenvironment

**Figure 7:**
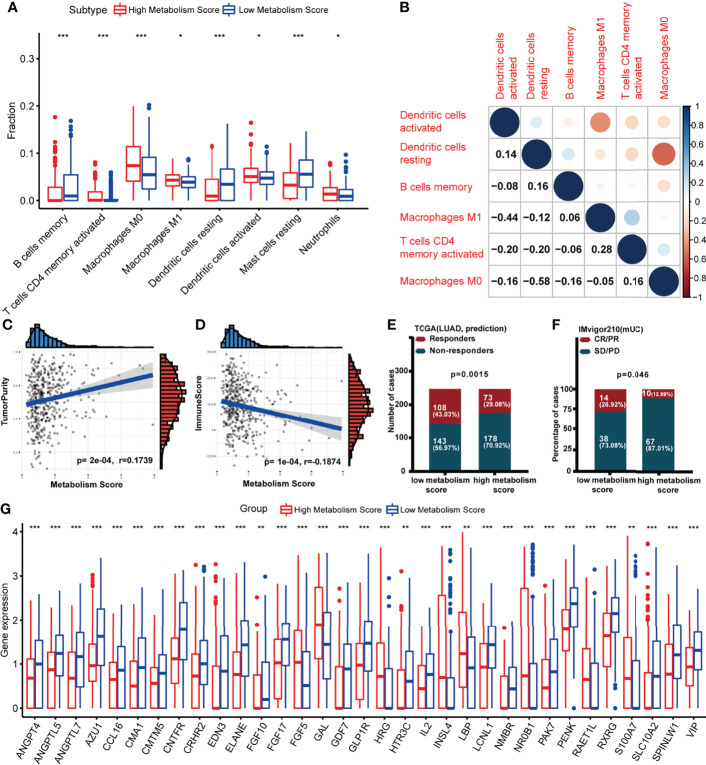
Association between Metabolism Score and immune microenvironment. **(A)** Boxplot showing ratio infiltration differences of 8 immune cells between high- and low-Metabolism Score groups in LUAD. The Wilcoxon rank-sum test was used for the significance test. **(B)** The infiltration correlation between the above eight immune cells. **(C)** Correlation between tumor purity score and Metabolism Score. **(D)** Correlation between immune score and Metabolism Score. **(E, F)** Response rate to immune checkpoint inhibitors (ICIs) between high- and low-Metabolism Score groups based on TIDE scores and immune checkpoint cohort (IMvigor210). **(G)** Differently expressed immune-related genes (DEIRGs) in tumor environments between LUAD with high and low Metabolism Scores were mapped with boxplots. *p < 0.05, **p < 0.01 and ***p < 0.001.

Since we found a close connection between metabolic changes and immune response processes (**Figures 1D, G**), we further explored differences of immune microenvironment between LUAD patients with low and high Metabolism Score. Different distribution of immune cells was found between high- and low-Metabolism Score groups neither using either CIBERSORT and ssGSEA algorithm (**Figure 7A**; **Supplementary Figure S4**). And immune subtypes with significant difference including B cell memory, T cell CD4 memory activation, macrophage M0, macrophage M1, dendritic cell resting, dendritic cell activation, mast cell resting and neutrophils based on CIBERSORT algorithm (**Figure 7A**). The interactions between the above immune cells were carried out using corrplot R in **Figure 7B**. And positive correlations were found in the infiltration of M1 macrophage and CD4^+^ memory T cells (Cor = 0.28, p = 0.043). However, a negative correlation was found between M1 macrophage and activated dendritic cell (Cor = -0.44, p = 0.033), as well as M0 macrophages and resting dendritic cell (Cor = -0.58, p = 0.011). ESTIMATE scores were performed to estimate tumor components in tissues, and specimens with higher Metabolism Scores seemed to have higher tumor purity scores (p = 0.0002) and lower immune scores (p < 0.0001) (**Figures 7C, D**). These results highly suggest significant differences in the immune microenvironment between LUAD with low- and high-Metabolism Scores.

Nowadays, immune checkpoint inhibitors (ICIs) (27, 28) have been demonstrated as promising treatments aimed at reconstructing immunosurveillance capabilities. Patients who responded to ICIs were found to have significantly longer survival times than those without a response. However, the lack of effective biomarkers to predict response limits the efficacy and clinical benefits of ICIs (27, 28). To explore the connection between Metabolism Scores and ICIs response status, TIDE and immune checkpoint cohort (IMvigor210) were used (**Figures 7E, F**; **Supplementary Table S11**). The distribution of immune responders, non-responders, CR/PR, and PD/SD were plotted in **Figures 7E, F**, and patients with low-Metabolism Scores seemed to have higher response rates than those with high-Metabolism Scores (p = 0.0015).

Since significant difference of immune microenvironment were found between low- and high-Metabolism Score groups (**Figures 7A-F**), we further explore immune-related DEGs between two groups. A total of 33 immune-related DEGs were screened out, including *ANGPT4, ANGPTL5, ANGPTL7, AZU1, CCL16, CMA1, CMTM5, CNTFR, CRHR2, EDN3, ELANE, FGF10, FGF17, FGF5, GAL, GDF7, GLP1R, HRG, HTR3C, IL2, INSL4, LBP, LCNL1, NMBR, NR0B1, PAK7, PENK, RAET1L, RXRG, S100A7, SLC10A2, SPINLW1* and *VIP* (**Supplementary Table S12**; **Figure 7G**).

### Associations Between MRGs, Metabolism Score and Clinic Drug Vulnerability

**Figure 8:**
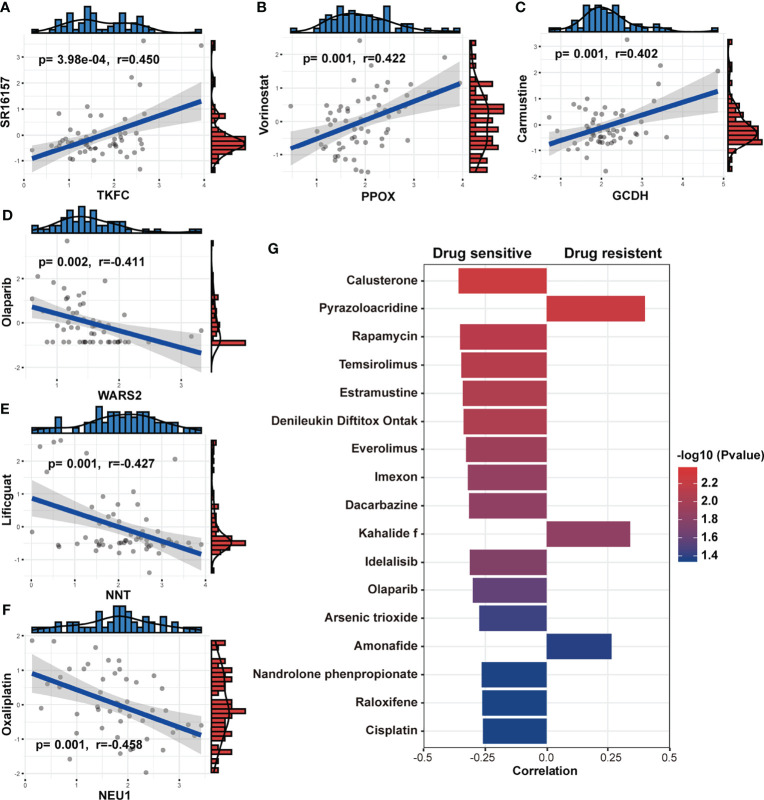
Association between MRGs, Metabolism Score and clinic drug vulnerability. **(A-F)** Gene expression and drug IC50 were downloaded from CellMiner and used to evaluate the correlation coefficient between MRGs and drug vulnerability. **(G)** The correlation coefficient was calculated based on the Metabolism Score and drug IC50. Higher coefficients were shown with longer bars, and the color of bars was counted by p-value.

The follow-up treatment of NSCLC patients after RT needs to be carefully considered, especially for those who have undergone multi-line treatments. Since we found that patients with low-Metabolism Score seemed to have higher response rate to ICIs (**Figures 7E, F**), we further explored the connection between the expression of 14 MRG, Metabolism Score and clinic drug vulnerability. A public website (CellMiner) was used, and the correlation between gene expression (*GCDH, TKFC, DTYMK, PPOX, NEU1*, etc.) and drug sensitivity (cyclophosphamide, oxaliplatin, olaparib, vorinostat, etc.) were mapped (**Figures 8A-F**; **Supplementary Table S13**).

In addition, NSCLC cell lines with higher Metabolism Scores were found to be vulnerable to the following drugs (**Figure 8G**): platinum compounds (cisplatin), alkylating agent (dacarbazine), cyanoaziridine derivative (imexon), PARP inhibitor (olaparib), PI3K/mTOR inhibitors (rapamycin, temsirolimus, everolimus and idelalisib) and hormone drugs (calusterone and raloxifene). Meanwhile, NSCLC cell lines may be resistant to topoisomerase inhibitors (pyrazoloacridine and amonafide) and natural marine extracts (kahalide f). These findings show the prognostic value of the metabolism-related model in improving drug vulnerability, which could provide a practical tool for the treatment of lung cancer, especially for those patients who have received RT.

## Discussion

A series of prognostic models (28) have been constructed to predict survival and prognosis of patients with malignant tumors. In our study, a prognostic model with metabolic signature was successfully established. Patients who received radiation therapy with higher Metabolism Scores in tumor tissues seem to have shorter survival rates than those with lower Metabolism Scores (**Figures 2D**, **3A–D**), which indicate that this metabolic-based model could be specifically applied for those LUAD acquired radio-resistance. More basic experiment could help us to discover more valuable metabolism genes. And combination of transcriptome, proteomics, and single cell sequencing data from radiosensitive and radioresistant tissues may help to further optimize this metabolic prognosis model. Our study also found that most DEGs between radio-sensitive and radio-resistant NSCLC cells were involved in metabolic pathways, such as tryptophan metabolism, butanoate metabolism, arginine biosynthesis.

L-tryptophan (Trp) (29) metabolism in the kynurenine pathway (KP) was reported to regulate immunity, the microbiota (30) and intestinal homeostasis by affecting the activity of rate-limiting enzymes, indoleamine-2,3-dioxygenase 1 (IDO1), IDO2, tryptophan-2,3-dioxygenase (TDO) and kynurenine monooxygenase (KMO). As a transport substrate in butanoate metabolism, butyrate (31) seems to play a paradoxical role in normal colonocyte growth and the cell differentiation of colorectal cancer cells. In addition, post-translational arginine methylation is responsible for the regulation of stem cell biology, alternative splicing (32), epigenetics and immune surveillance (33). These studies suggest complex interactions between metabolism, TME and host immunity response. More experiments should be conducted to identify the connection between abnormal metabolic processes and malignant phenotypes of lung cancer, such as tumor proliferation, migration, radiosensitivity, etc.

There are emerging evidences that RT may trigger host responses not only in the field with radiation exposure but also in the remote out-of-field arena (16). Therefore, clarifying the difference between tumors and tumor microenvironment (TME) between radiosensitive and radioresistant tissues may provide novel targets to increase the efficacy of RT in NSCLC. We found significant differences in TME between high and low-Metabolism Score groups, including immune cell infiltrations, ICIs response status and immune factors expression (**Figures 7A-G**). As a well-known pleiotropic cytokine, interleukin-2 (IL-2) (34) regulates the proliferation/differentiation of effector lymphocytes and the expansion/survival of regulatory T cells. The application of IL-2-based therapeutics has brought great benefits to tumor patients, such as renal cell carcinoma and melanoma patients (35, 36). As shown in **Figure 6F**, lower expression level of IL-2 in the high-Metabolism Score group compared with low-Metabolism Score group, which indicates less effective T cells and worse anti-tumor response in high-Metabolism Score subgroups. Fibroblast growth factor 5 (FGF5) (37) was reported to induce resistance to HER2-targeted therapies in breast cancer. Moreover, downregulation of FGF5 could inhibit cell growth and invasion of NSCLC cells (38, 39). We found higher expression of FGF5 in the high-Metabolism Score subgroups, which indicated more immune suppressive environment in this groups. Immune factors may affect proliferation of immune cells and immune escape capabilities of NSCLC cells, ultimately leading the survival of NSCLC patients.

During the process of modelling, 14 metabolic-related genes were screened out, including PPOX, DTYMK, GCDH, HEMK1, JMJD7.PLA2G4B, NEU1, NT5C3A, NNT, TKFC, POLR3G, TRDMT1, SMS, UAP1 and WARS2. However, the functions of those genes on radiosensitivity, tumorigenesis, differentiation, migration, metastasis, immunological tolerance, and drug vulnerability remain to be clarified. As a penultimate enzyme of heme biosynthesis, PPOX catalyzes the 6-electron oxidation of protoporphyrinogen IX to form protoporphyrin IX. Inhibition of PPOX was reported to significantly reduce the growth of colon cancer cells *in vitro* and *in vivo* (40). We found that high expression of PPOX was positively correlated with the effect of vorinostat (41), a promising histone deacetylase inhibitor that could selectively ablate drug-resistant tumor cells in MAPK inhibitor-resistant melanomas. However, the connection between PPOX, radiosensitivity and immune systems remains unclear.

As a thymidylate kinase involved in nucleotide biosynthetic process, DTYMK could positively promote proliferation of hepatocellular carcinoma through regulating the cell cycle (42). Meanwhile, LKB1 mutant lung cancers have deficits in nucleotide metabolism that confer hypersensitivity to DTYMK inhibition (43). We found that high expression of DTYMK in tumors was positively correlated with the activity of asparaginase (**Supplementary Table S13**). However, the influence of DTYMK on aspartate synthesis was rarely reported. Moreover, aspartate synthesis plays an important role in the proliferation of cancer cells when respiration is impaired (44), while hypoxia (45) may promote radiation resistance of tumor cells. More experiments could be developed to explore the connection between DTYMK, aspartate synthesis and radiosensitivity in NSCLC.

In summary, we demonstrated a comprehensive landscape of differences between radiosensitive and radio-resistant LUAD cells, and screened out a series of core genes that may be involved in radiosensitivity, metabolism reprogramming and clinical characteristics of NSCLC patients. A metabolism-based prognostic model using Metabolism Scores was constructed to predict immune infiltration, ICIs response, drug vulnerability and prognosis of LUAD patients. Our study provides novel targets and prognostic models for LUAD from the perspective of metabolism, and predicts the potential connection between radiosensitivity, metabolism reprogramming, and immune response activity.

## Data Availability Statement

The datasets presented in this study can be found in online repositories. The names of the repository/repositories and accession number(s) can be found in the article/supplementary material.

## Ethics Statement

This work was reviewed and approved by the Medical Ethics Committee of the Fifth Affiliated Hospital of Sun Yat-sen University, and the experimental procedures were conducted in accordance with the Declaration of Helsinki.

## Author Contributions

S-LP and RW concepted and designed the manuscript. S-LP, RW and Y-LZ developed the methodology and analyzed the data. S-LP drafted the manuscript. S-LP, RW, WW, G-HZ, X-TH, SY and Q-DL collected the data and reviewed the manuscript. Z-GL was responsible for project administration and supervision. All authors contributed to the article and approved the submitted version.

## Funding

This work was supported by Guangdong Basic and Applied Basic Research Foundation (Grant 2020A1515110599 to S-LP) and China Postdoctoral Science Foundation (Grant 2020M683066 to S-LP), Zhuhai City Medical and Health Technology Plan (Grant No. ZH2202200013HJL, awarded to Z-GL), The Fundamental Research Funds for the Central Universities (Grant No. 19ykzd07, awarded to Z-GL).

## Conflict of Interest

The authors declare that the research was conducted in the absence of any commercial or financial relationships that could be construed as a potential conflict of interest.

## Publisher’s Note

All claims expressed in this article are solely those of the authors and do not necessarily represent those of their affiliated organizations, or those of the publisher, the editors and the reviewers. Any product that may be evaluated in this article, or claim that may be made by its manufacturer, is not guaranteed or endorsed by the publisher.

## Supplementary Material

Supplementary Figure 1 The construction of a protein-protein interaction (PPI) network. DEGs between radio-resistant (A549RR) and parent (A549) cells were analyzed to evaluate PPI using the STRING database. Molecules and connections were mapped as nodes and lines. Proteins with high combined scores (> 0.9) and high co-expression coefficient (> 0.9) were screened out and mapped.

Supplementary Figure 2 Validation of metabolic-based prognostic models in lung adenocarcinoma (LUAD) patients received radiation therapy. **(A).** Metabolism Score and overall survival status of LUAD patients received radiation therapy using metabolic prognostic model and Kaplan–Meier plotter analysis. **(B).** Prediction and observed of ROC curve based on Metabolism Score.

Supplementary Figure 3 Expression of DTYMK, GCDH, HEMK1 in A549 and A549RR cells measured by RT-qPCR. **p < 0.01.

Supplementary Figure 4 Percentages of immune subtypes in LUAD with low and high Metabolism Score calculated by ssGSEA algorithm. Immune subtypes with significant difference between low and high Metabolism Score groups were marked. **p < 0.01 and ***p < 0.001.
