# Supplementary material for: Insight of a Metabolic Prognostic Model to Identify Tumor Environment and Drug Vulnerability for Lung Adenocarcinoma
Source: Front Immunol. 2022 Jun 23;13:872910. doi: 10.3389/fimmu.2022.872910 (PMC9262104; doi:10.3389/fimmu.2022.872910)

## Supplementary Figure 1

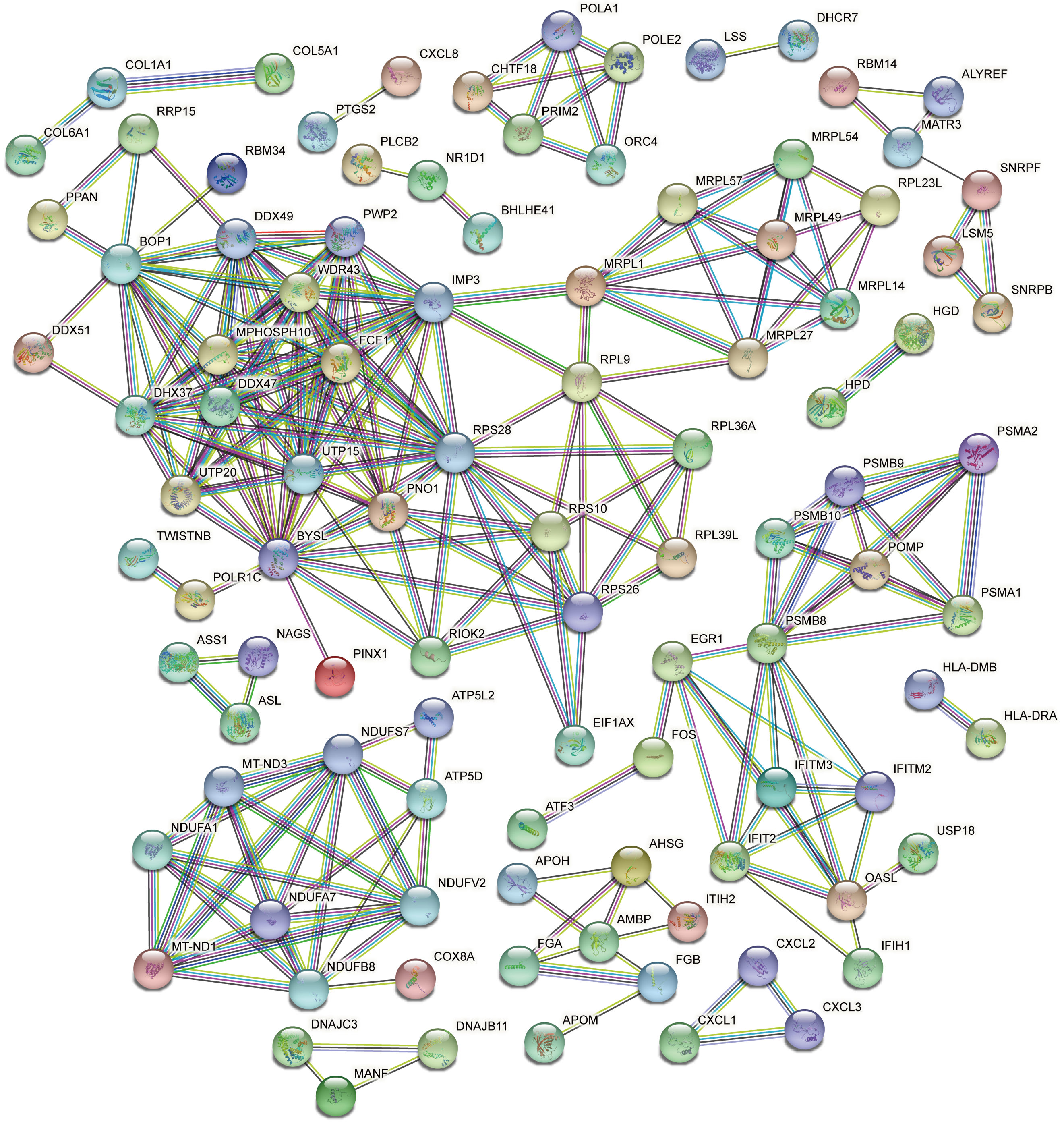

**Supplementary Figure 2**

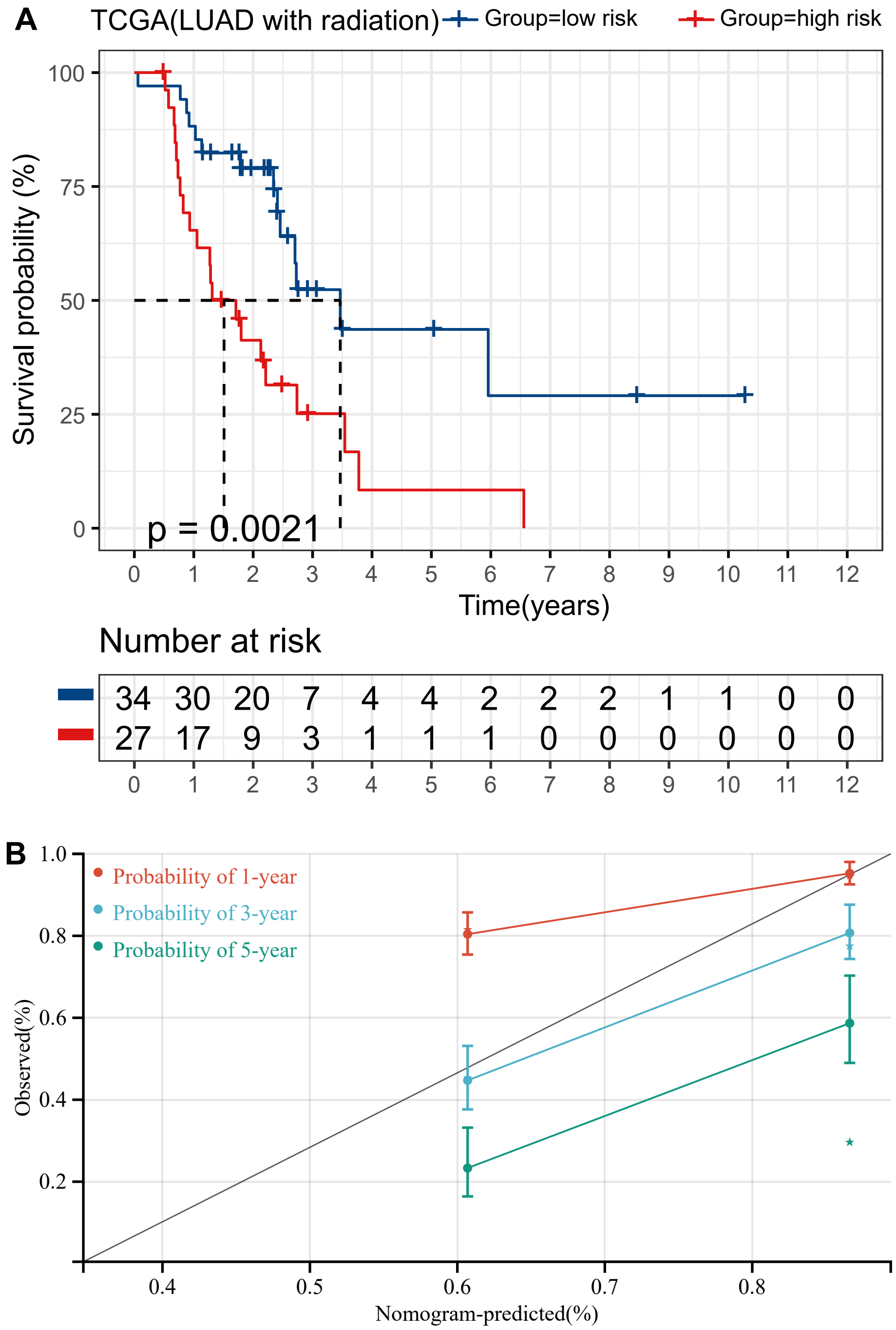

# Supplementary Figure 3

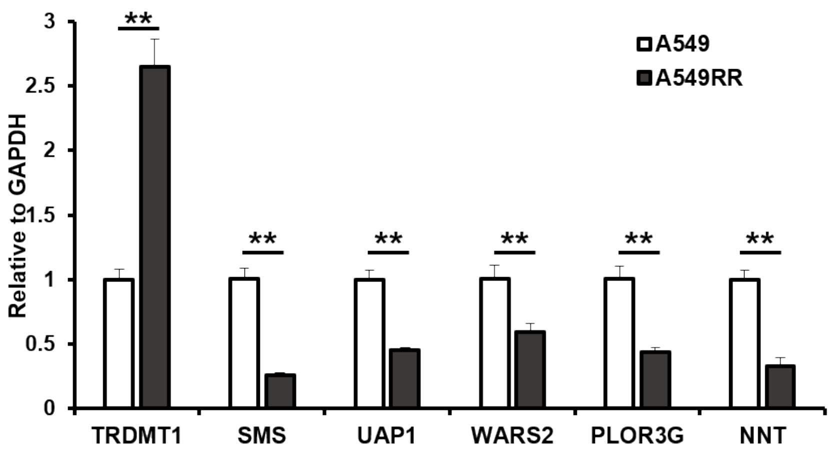


## Primer Sequence

**TRDMT1 Forward: CGGGTGCTGGAGCTATACAG**

**TRDMT1 Reverse: CGACAGTGTTGACATCAATGGC**

**SMS Forward: TGGGCGGGTGAAACGATTAC**

**SMS Reverse: CCAAACTGCTTCGAGTGTAGAA**

**UAP1 Forward: AATGACCTCAAACTCACGTTGT**

**UAP1 Reverse: GCTCTGCATAAAGTTCTACCTGT**

**WARS2 Forward: CTGCACTCAATGCGGAAAGC**

**WARS2 Reverse: GAATGCCGGAAAATACTCGCT**

**PLOR3G Forward: CACTTCGGCTGCAGAGTTTT**

**PLOR3G Reverse: AGTGGGCAAATTCTGAAAG**

**NNT Forward: GGGGTCCTGTAAGGGTCTAC**

**NNT Reverse: ATGCCACTCGCTTCTCATTTT**

# Supplementary Figure 4

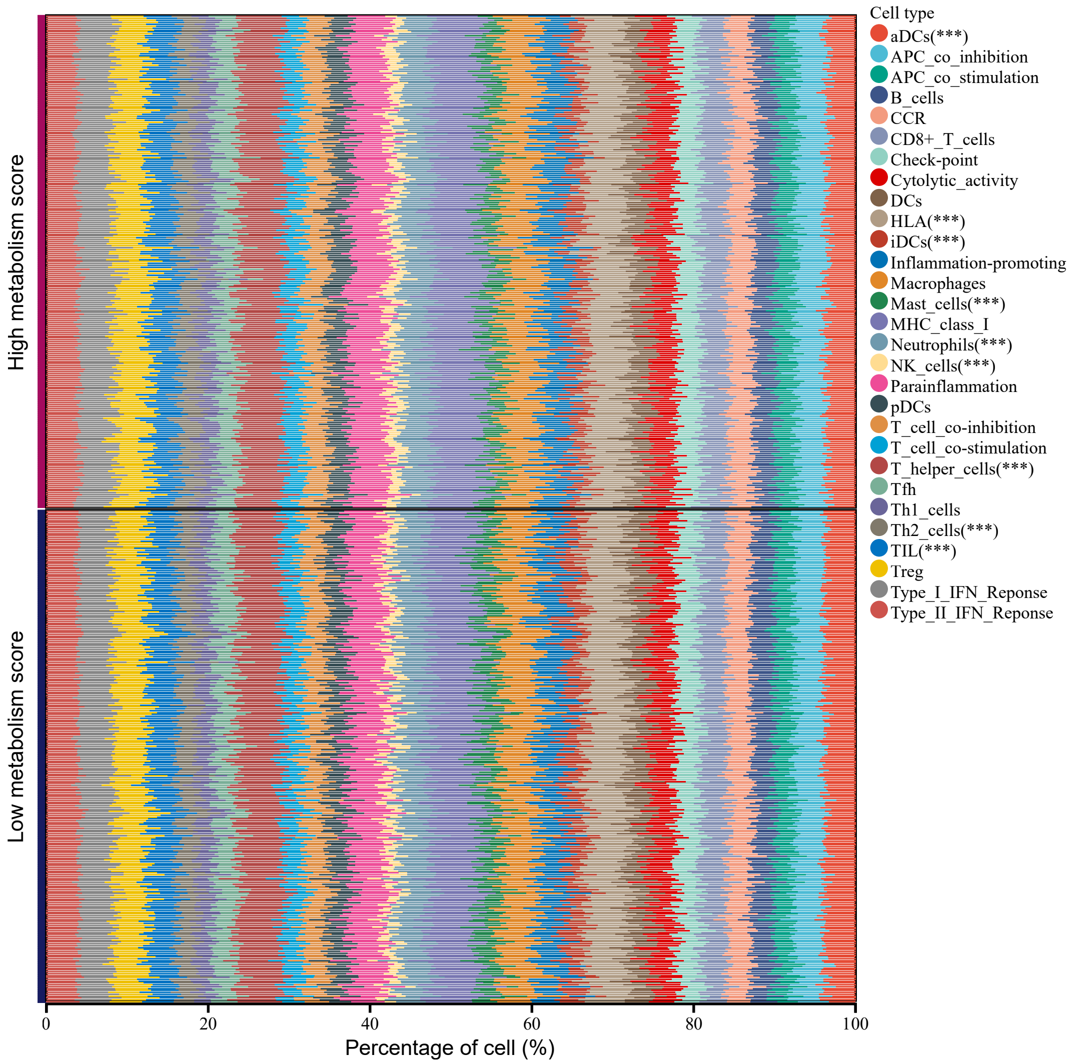

Supplement: Supplementary Figure 1 — The construction of a protein-protein interaction (PPI) network. DEGs between radio-resistant (A549RR) and parent (A549) cells were analyzed to evaluate PPI using the STRING database. Molecules and connections were mapped as nodes and lines. Proteins with high combined scores (> 0.9) and high co-expression coefficient (> 0.9) were screened out and mapped. [file DataSheet_14.pdf]
