# Supplementary material for: Insight of a Metabolic Prognostic Model to Identify Tumor Environment and Drug Vulnerability for Lung Adenocarcinoma
Source: Front Immunol. 2022 Jun 23;13:872910. doi: 10.3389/fimmu.2022.872910 (PMC9262104; doi:10.3389/fimmu.2022.872910)
Supplement: Supplementary file 2 [file DataSheet_1.pdf]

Supplementary Table S1: The differentially expressed genes (DEGs) between A549 and A549RR cells.

ensembl_gene_id	external_gene_name	gene_biotype	log2FC	Pvalue	FDR	Style
ENSG00000115414	FN1	protein_coding	1.812974433	0	0	up
ENSG00000198888	MT-ND1	protein_coding	-1.149081251	0	0	down
ENSG00000151632	AKR1C2	protein_coding	1.440208362	0	0	up
ENSG00000242265	PEG10	protein_coding	-1.445619907	0	0	down
ENSG00000164741	DLC1	protein_coding	5.230839535	0	0	up
ENSG00000146674	IGFBP3	protein_coding	3.305185721	0	0	up
ENSG00000145632	PLK2	protein_coding	-2.208480014	0	0	down
ENSG00000079805	DNM2	protein_coding	1.935355429	0	0	up
ENSG00000187134	AKR1C1	protein_coding	1.151458072	0	0	up
ENSG00000096696	DSP	protein_coding	1.250156915	0	0	up
ENSG00000101871	MID1	protein_coding	-1.253442434	0	0	down
ENSG00000038427	VCAN	protein_coding	2.868039708	9.79E-308	4.89E-305	up
ENSG00000168209	DDIT4	protein_coding	1.459208225	3.48E-268	1.55E-265	up
ENSG00000104419	NDRG1	protein_coding	2.915950188	1.95E-264	8.22E-262	up
ENSG00000170004	CHD3	protein_coding	1.018535388	8.49E-247	3.41E-244	up
ENSG00000170477	KRT4	protein_coding	6.087528109	9.95E-230	3.64E-227	up
ENSG00000073756	PTGS2	protein_coding	-1.961459707	9.89E-222	3.47E-219	down
ENSG00000064651	SLC12A2	protein_coding	-1.350901239	9.38E-210	3.15E-207	down
ENSG00000131462	TUBG1	protein_coding	-1.318632715	2.00E-208	6.58E-206	down
ENSG00000018236	CNTN1	protein_coding	-2.788665689	4.79E-207	1.55E-204	down
ENSG00000163735	CXCL5	protein_coding	-1.630301375	1.75E-206	5.55E-204	down
ENSG00000170381	SEMA3E	protein_coding	2.645138019	1.21E-201	3.75E-199	up
ENSG00000128524	ATP6V1F	protein_coding	-1.035644921	5.95E-200	1.82E-197	down
ENSG00000215182	MUC5AC	protein_coding	1.100288534	1.36E-198	4.07E-196	up
ENSG00000169851	PCDH7	protein_coding	3.463465789	3.86E-196	1.14E-193	up
ENSG00000140538	NTRK3	protein_coding	-2.382451434	4.14E-191	1.14E-188	down
ENSG00000086061	DNAJA1	protein_coding	-1.292122653	9.86E-185	2.62E-182	down
ENSG00000004799	PDK4	protein_coding	-2.199842764	3.22E-183	8.30E-181	down
ENSG00000091129	NRCAM	protein_coding	-1.172750607	2.54E-182	6.45E-180	down
ENSG00000228253	MT-ATP8	protein_coding	-1.760330989	2.35E-167	5.24E-165	down
ENSG00000089289	IGBP1	protein_coding	1.422093029	5.33E-155	1.07E-152	up
ENSG00000006453	BAIAP2L1	protein_coding	1.561904658	7.32E-155	1.45E-152	up
ENSG00000003436	TFPI	protein_coding	1.537969731	2.22E-153	4.35E-151	up
ENSG00000183684	ALYREF	protein_coding	-1.532420265	1.69E-152	3.24E-150	down
ENSG00000106780	MEGF9	protein_coding	1.167308609	1.16E-149	2.15E-147	up
ENSG00000000971	CFH	protein_coding	2.544592673	3.66E-148	6.70E-146	up
ENSG00000167106	FAM102A	protein_coding	1.112703401	5.22E-142	9.14E-140	up
ENSG00000171345	KRT19	protein_coding	-11.11129468	2.35E-141	4.08E-139	down
ENSG00000167468	GPX4	protein_coding	1.047790352	1.65E-138	2.81E-136	up
ENSG00000075426	FOSL2	protein_coding	-1.748148526	9.70E-134	1.57E-131	down
ENSG00000104805	NUCB1	protein_coding	1.048088872	2.35E-130	3.76E-128	up
ENSG00000024422	EHD2	protein_coding	1.364238836	1.45E-129	2.28E-127	up
ENSG00000275395	FCGBP	protein_coding	2.287678397	1.22E-128	1.88E-126	up
ENSG00000066468	FGFR2	protein_coding	5.05342897	1.48E-128	2.26E-126	up
ENSG00000139289	PHLDA1	protein_coding	-1.25575449	1.25E-127	1.89E-125	down
ENSG00000108106	UBE2S	protein_coding	-1.457048091	1.26E-127	1.88E-125	down
ENSG00000171867	PRNP	protein_coding	-1.40532443	1.37E-127	2.03E-125	down
ENSG00000124429	POF1B	protein_coding	1.878842227	2.26E-127	3.32E-125	up
ENSG00000142798	HSPG2	protein_coding	1.088696756	2.59E-124	3.74E-122	up
ENSG00000105825	TFPI2	protein_coding	-1.389093962	3.62E-124	5.19E-122	down
ENSG00000146374	RSPO3	protein_coding	-2.050445578	3.66E-123	5.20E-121	down

ENSG00000184226	PCDH9	protein_coding	1.949503663	1.26E-120	1.73E-118	up
ENSG00000118898	PPL	protein_coding	2.594773046	1.35E-120	1.84E-118	up
ENSG00000103275	UBE2I	protein_coding	-1.322440801	7.82E-117	9.98E-115	down
ENSG00000168453	HR	protein_coding	2.614675975	1.71E-115	2.13E-113	up
ENSG00000005889	ZFX	protein_coding	-2.487167295	1.74E-114	2.16E-112	down
ENSG00000272398	CD24	protein_coding	1.226573797	3.61E-114	4.44E-112	up
ENSG00000065054	SLC9A3R2	protein_coding	2.385492093	4.03E-113	4.81E-111	up
ENSG00000076864	RAP1GAP	protein_coding	1.279363366	1.54E-112	1.81E-110	up
ENSG00000163811	WDR43	protein_coding	-1.208142621	5.20E-112	6.03E-110	down
ENSG00000130985	UBA1	protein_coding	-1.00818327	8.80E-111	1.01E-108	down
ENSG00000146166	LGSN	protein_coding	2.567108557	7.51E-107	8.31E-105	up
ENSG00000138061	CYP1B1	protein_coding	-1.728914064	1.75E-106	1.90E-104	down
ENSG00000102172	SMS	protein_coding	-1.195905401	1.07E-105	1.15E-103	down
ENSG00000152818	UTRN	protein_coding	1.072703956	3.00E-105	3.19E-103	up
ENSG00000171617	ENC1	protein_coding	-2.066302674	9.57E-105	1.01E-102	down
ENSG00000107984	DKK1	protein_coding	1.148211919	7.57E-104	7.89E-102	up
ENSG00000164032	H2AFZ	protein_coding	-1.058405687	2.40E-103	2.49E-101	down
ENSG00000102317	RBM3	protein_coding	-1.206260673	6.83E-103	7.04E-101	down
ENSG00000182809	CRIP2	protein_coding	3.778664372	4.53E-102	4.63E-100	up
ENSG00000131470	PSMC3IP	protein_coding	-2.526515456	5.19E-102	5.28E-100	down
ENSG00000156510	HKDC1	protein_coding	-1.438468212	4.41E-101	4.46E-99	down
ENSG00000153922	CHD1	protein_coding	-1.130413145	9.75E-101	9.80E-99	down
ENSG00000132561	MATN2	protein_coding	2.585834245	1.27E-100	1.27E-98	up
ENSG00000158856	DMTN	protein_coding	1.730801274	1.27E-99	1.26E-97	up
ENSG00000100504	PYGL	protein_coding	1.165935641	1.74E-99	1.70E-97	up
ENSG00000112877	CEP72	protein_coding	-3.271132791	2.48E-99	2.42E-97	down
ENSG00000182621	PLCB1	protein_coding	2.900262882	3.50E-98	3.39E-96	up
ENSG00000108691	CCL2	protein_coding	-2.760606138	3.65E-98	3.51E-96	down
ENSG00000152661	GJA1	protein_coding	1.401894225	5.63E-98	5.36E-96	up
ENSG00000130204	TOMM40	protein_coding	-1.037499525	6.75E-97	6.35E-95	down
ENSG00000171848	RRM2	protein_coding	-1.812273407	1.22E-95	1.14E-93	down
ENSG00000123384	LRP1	protein_coding	1.201921001	3.38E-93	3.09E-91	up
ENSG00000144057	ST6GAL2	protein_coding	5.142744802	4.11E-92	3.74E-90	up
ENSG00000169925	BRD3	protein_coding	1.170146237	7.54E-92	6.82E-90	up
ENSG00000198840	MT-ND3	protein_coding	-1.397059793	4.43E-91	3.99E-89	down
ENSG00000111799	COL12A1	protein_coding	2.25710107	4.10E-90	3.63E-88	up
ENSG00000157214	STEAP2	protein_coding	-1.851767939	1.34E-88	1.16E-86	down
ENSG00000242372	EIF6	protein_coding	-1.511975547	1.60E-88	1.38E-86	down
ENSG00000131018	SYNE1	protein_coding	1.284372877	1.73E-88	1.49E-86	up
ENSG00000033170	FUT8	protein_coding	-1.879003085	4.12E-88	3.52E-86	down
ENSG00000072422	RHOBTB1	protein_coding	1.458890739	1.44E-87	1.22E-85	up
ENSG00000197170	PSMD12	protein_coding	-1.126830491	2.23E-86	1.87E-84	down
ENSG00000141564	RPTOR	protein_coding	-1.335902446	4.30E-86	3.56E-84	down
ENSG00000130725	UBE2M	protein_coding	-1.337517162	8.50E-86	7.00E-84	down
ENSG00000111275	ALDH2	protein_coding	1.100351203	3.90E-85	3.19E-83	up
ENSG00000168646	AXIN2	protein_coding	-2.17842378	7.56E-85	6.13E-83	down
ENSG00000173848	NET1	protein_coding	1.055808406	1.48E-84	1.19E-82	up
ENSG00000138162	TACC2	protein_coding	1.197638077	2.61E-84	2.08E-82	up
ENSG00000178996	SNX18	protein_coding	-2.292768139	4.42E-84	3.50E-82	down
ENSG00000240694	PNMA2	protein_coding	1.5356245	5.17E-83	4.01E-81	up
ENSG00000117983	MUC5B	protein_coding	1.85084503	6.34E-83	4.90E-81	up
ENSG00000088247	KHSRP	protein_coding	-1.094316712	3.41E-82	2.61E-80	down
ENSG00000186350	RXRA	protein_coding	1.353522892	4.50E-82	3.42E-80	up
ENSG00000088826	SMOX	protein_coding	-1.570974857	5.11E-82	3.86E-80	down

ENSG00000117139	KDM5B	protein_coding	1.057079741	2.29E-81	1.69E-79	up
ENSG00000169410	PTPN9	protein_coding	1.347525887	2.30E-81	1.69E-79	up
ENSG00000175756	AURKAIP1	protein_coding	-1.325195525	3.09E-81	2.27E-79	down
ENSG00000006118	TMEM132A	protein_coding	-2.073666487	3.65E-81	2.66E-79	down
ENSG00000164338	UTP15	protein_coding	-2.085022362	1.25E-80	8.87E-79	down
ENSG00000090924	PLEKHG2	protein_coding	1.689283552	1.52E-79	1.06E-77	up
ENSG00000101040	ZMYND8	protein_coding	1.319603084	1.23E-78	8.41E-77	up
ENSG00000103888	CEMIP	protein_coding	-1.343430334	1.46E-78	9.88E-77	down
ENSG00000141510	TP53	protein_coding	1.17395472	2.69E-78	1.81E-76	up
ENSG00000101004	NINL	protein_coding	1.346276624	7.26E-78	4.84E-76	up
ENSG00000088038	CNOT3	protein_coding	1.018996461	2.54E-77	1.68E-75	up
ENSG00000151892	GFRA1	protein_coding	2.76257467	3.35E-77	2.20E-75	up
ENSG00000187634	SAMD11	protein_coding	2.618894224	5.14E-77	3.36E-75	up
ENSG00000121073	SLC35B1	protein_coding	-2.043477118	7.54E-77	4.91E-75	down
ENSG00000141013	GAS8	protein_coding	1.468097009	3.58E-76	2.31E-74	up
ENSG00000115946	PNO1	protein_coding	-1.534101149	8.63E-76	5.51E-74	down
ENSG00000154305	MIA3	protein_coding	1.12519653	1.49E-74	9.36E-73	up
ENSG00000138193	PLCE1	protein_coding	1.796102759	5.25E-74	3.28E-72	up
ENSG00000103111	MON1B	protein_coding	1.26625193	7.89E-73	4.85E-71	up
ENSG00000167306	MYO5B	protein_coding	-3.622748507	1.03E-72	6.30E-71	down
ENSG00000130508	PXDN	protein_coding	-9.841388793	2.30E-72	1.39E-70	down
ENSG00000108375	RNF43	protein_coding	-1.386936613	3.83E-72	2.30E-70	down
ENSG00000119812	FAM98A	protein_coding	-1.675177294	5.20E-72	3.10E-70	down
ENSG00000155545	MIER3	protein_coding	-2.598117642	2.53E-71	1.50E-69	down
ENSG00000115993	TRAK2	protein_coding	1.668948042	3.65E-71	2.15E-69	up
ENSG00000041353	RAB27B	protein_coding	-1.385976368	5.61E-71	3.28E-69	down
ENSG00000092853	CLSPN	protein_coding	-1.623399178	1.43E-70	8.31E-69	down
ENSG00000165323	FAT3	protein_coding	3.755287128	2.69E-70	1.55E-68	up
ENSG00000074416	MGLL	protein_coding	1.329678033	1.27E-69	7.29E-68	up
ENSG00000081041	CXCL2	protein_coding	-4.868457036	1.36E-69	7.75E-68	down
ENSG00000171604	CXXC5	protein_coding	1.592173118	4.31E-69	2.45E-67	up
ENSG00000088992	TESC	protein_coding	-1.354746553	7.02E-69	3.95E-67	down
ENSG00000198722	UNC13B	protein_coding	1.350015936	1.07E-68	5.99E-67	up
ENSG00000162627	SNX7	protein_coding	1.944000798	2.92E-68	1.62E-66	up
ENSG00000164647	STEAP1	protein_coding	-1.187822478	5.42E-68	3.01E-66	down
ENSG00000119326	CTNNAL1	protein_coding	-1.424026241	5.73E-68	3.17E-66	down
ENSG00000184347	SLIT3	protein_coding	2.481452111	7.24E-68	3.99E-66	up
ENSG00000117394	SLC2A1	protein_coding	1.184286129	8.74E-68	4.80E-66	up
ENSG00000125356	NDUFA1	protein_coding	-1.222249177	1.86E-67	1.02E-65	down
ENSG00000177200	CHD9	protein_coding	1.077881863	4.27E-67	2.32E-65	up
ENSG00000072071	ADGRL1	protein_coding	2.313269452	6.19E-67	3.35E-65	up
ENSG00000186395	KRT10	protein_coding	-1.825646056	1.03E-66	5.56E-65	down
ENSG00000125835	SNRPB	protein_coding	-1.011371559	1.94E-66	1.04E-64	down
ENSG00000172927	MYEOV	protein_coding	-1.989142413	2.78E-66	1.48E-64	down
ENSG00000114374	USP9Y	protein_coding	1.73470647	3.65E-66	1.94E-64	up
ENSG00000078061	ARAF	protein_coding	-2.592533322	3.95E-66	2.09E-64	down
ENSG00000174446	SNAPC5	protein_coding	-2.499282816	4.08E-66	2.15E-64	down
ENSG00000079215	SLC1A3	protein_coding	-3.176373041	5.09E-66	2.67E-64	down
ENSG00000116574	RHOU	protein_coding	4.977193384	1.01E-65	5.26E-64	up
ENSG00000196526	AFAP1	protein_coding	1.32731823	3.69E-65	1.92E-63	up
ENSG00000159593	NAE1	protein_coding	-1.219448434	2.30E-64	1.17E-62	down
ENSG00000166938	DIS3L	protein_coding	-1.083175465	2.97E-64	1.51E-62	down
ENSG00000012174	MBTPS2	protein_coding	-1.491202438	3.14E-64	1.59E-62	down
ENSG00000186871	ERCC6L	protein_coding	-2.178267229	5.72E-64	2.88E-62	down

ENSG00000106355	LSM5	protein_coding	-1.122556109	6.81E-64	3.41E-62	down
ENSG00000163683	SMIM14	protein_coding	1.605312267	2.74E-63	1.36E-61	up
ENSG00000132639	SNAP25	protein_coding	2.000675783	1.51E-62	7.38E-61	up
ENSG00000123131	PRDX4	protein_coding	-1.611190969	1.71E-62	8.37E-61	down
ENSG00000196704	AMZ2	protein_coding	-1.080900476	1.80E-62	8.77E-61	down
ENSG00000162734	PEA15	protein_coding	-1.544228603	3.75E-62	1.82E-60	down
ENSG00000065882	TBC1D1	protein_coding	1.093553109	3.79E-62	1.84E-60	up
ENSG00000078018	MAP2	protein_coding	2.629819993	4.01E-62	1.94E-60	up
ENSG00000169174	PCSK9	protein_coding	2.110746819	6.19E-62	2.97E-60	up
ENSG00000067798	NAV3	protein_coding	-1.696683782	6.68E-62	3.20E-60	down
ENSG00000155097	ATP6V1C1	protein_coding	-1.448066358	1.25E-61	5.91E-60	down
ENSG00000104760	FGL1	protein_coding	2.342778131	1.76E-61	8.29E-60	up
ENSG00000141698	NT5C3B	protein_coding	-1.033737356	1.80E-61	8.49E-60	down
ENSG00000120063	GNA13	protein_coding	-1.193495037	2.68E-61	1.25E-59	down
ENSG00000103202	NME4	protein_coding	-1.312399931	3.16E-61	1.47E-59	down
ENSG00000100292	HMOX1	protein_coding	-1.395951341	5.70E-61	2.64E-59	down
ENSG00000186660	ZFP91	protein_coding	-1.428533559	6.34E-61	2.93E-59	down
ENSG00000058729	RIOK2	protein_coding	-1.964428403	7.42E-61	3.41E-59	down
ENSG00000115183	TANC1	protein_coding	1.374874923	9.16E-61	4.19E-59	up
ENSG00000146574	CCZ1B	protein_coding	2.462391955	1.05E-60	4.78E-59	up
ENSG00000173918	C1QTNF1	protein_coding	-8.300820412	1.13E-60	5.13E-59	down
ENSG00000112992	NNT	protein_coding	-3.087013521	1.31E-60	5.91E-59	down
ENSG00000109133	TMEM33	protein_coding	-1.243715241	1.47E-60	6.65E-59	down
ENSG00000130433	CACNG6	protein_coding	3.028027097	1.69E-60	7.61E-59	up
ENSG00000095321	CRAT	protein_coding	1.389243468	6.81E-59	2.98E-57	up
ENSG00000213918	DNASE1	protein_coding	1.914651482	1.14E-58	5.00E-57	up
ENSG00000140284	SLC27A2	protein_coding	-1.787219818	1.27E-58	5.55E-57	down
ENSG00000136950	ARPC5L	protein_coding	-2.798575709	1.85E-58	8.03E-57	down
ENSG00000130158	DOCK6	protein_coding	1.663867008	1.89E-58	8.17E-57	up
ENSG00000164244	PRRC1	protein_coding	-1.35211926	3.65E-58	1.57E-56	down
ENSG00000186472	PCLO	protein_coding	1.566831124	3.81E-58	1.63E-56	up
ENSG00000104765	BNIP3L	protein_coding	1.08454425	4.06E-58	1.74E-56	up
ENSG00000163170	BOLA3	protein_coding	-1.888694508	5.98E-58	2.55E-56	down
ENSG00000143315	PIGM	protein_coding	-8.210622603	1.25E-57	5.28E-56	down
ENSG00000141456	PELP1	protein_coding	-1.099700789	7.81E-57	3.27E-55	down
ENSG00000123358	NR4A1	protein_coding	-2.335652983	1.20E-56	4.99E-55	down
ENSG00000130165	ELOF1	protein_coding	-2.86477172	3.73E-56	1.54E-54	down
ENSG00000130707	ASS1	protein_coding	1.520682343	4.64E-56	1.91E-54	up
ENSG00000168090	COPS6	protein_coding	-1.251764915	6.65E-56	2.72E-54	down
ENSG00000264364	DYNLL2	protein_coding	-1.204587361	7.35E-56	3.00E-54	down
ENSG00000164638	SLC29A4	protein_coding	2.223721303	7.43E-56	3.02E-54	up
ENSG00000196872	KIAA1211L	protein_coding	3.444013426	8.92E-56	3.61E-54	up
ENSG00000120800	UTP20	protein_coding	-1.019933674	1.51E-55	6.04E-54	down
ENSG00000158528	PPP1R9A	protein_coding	1.045023868	1.53E-55	6.11E-54	up
ENSG00000081087	OSTM1	protein_coding	-2.456471282	1.54E-55	6.15E-54	down
ENSG00000175592	FOSL1	protein_coding	-2.37190351	2.21E-55	8.77E-54	down
ENSG00000119048	UBE2B	protein_coding	-2.991751528	3.03E-55	1.20E-53	down
ENSG00000172175	MALT1	protein_coding	-1.080802502	6.13E-55	2.41E-53	down
ENSG00000132326	PER2	protein_coding	1.542734063	8.35E-55	3.28E-53	up
ENSG00000125434	SLC25A35	protein_coding	-3.029015796	8.97E-55	3.51E-53	down
ENSG00000118596	SLC16A7	protein_coding	1.994117169	1.02E-54	3.99E-53	up
ENSG00000173674	EIF1AX	protein_coding	-1.186246643	1.66E-54	6.41E-53	down
ENSG00000049167	ERCC8	protein_coding	-2.494056153	2.27E-54	8.77E-53	down
ENSG00000141232	TOB1	protein_coding	-1.187301932	2.59E-54	9.97E-53	down

ENSG00000090905	TNRC6A	protein_coding	-1.095619935	3.08E-54	1.18E-52	down
ENSG00000145050	MANF	protein_coding	-1.269662119	4.49E-54	1.72E-52	down
ENSG00000178971	CTC1	protein_coding	1.135601229	7.02E-54	2.68E-52	up
ENSG00000144290	SLC4A10	protein_coding	8.977193384	8.60E-54	3.26E-52	up
ENSG00000107890	ANKRD26	protein_coding	-1.838523459	1.79E-53	6.76E-52	down
ENSG00000086548	CEACAM6	protein_coding	-2.186036964	2.65E-53	9.98E-52	down
ENSG00000072832	CRMP1	protein_coding	1.764233178	3.08E-53	1.16E-51	up
ENSG00000124882	EREG	protein_coding	-1.225062121	3.83E-53	1.44E-51	down
ENSG00000150990	DHX37	protein_coding	-1.209070483	3.87E-53	1.45E-51	down
ENSG00000111880	RNGTT	protein_coding	-2.307605016	4.14E-53	1.54E-51	down
ENSG00000163739	CXCL1	protein_coding	-3.09596162	4.79E-53	1.77E-51	down
ENSG00000141560	FN3KRP	protein_coding	-1.906764802	5.94E-53	2.19E-51	down
ENSG00000241119	UGT1A9	protein_coding	3.300521832	6.74E-53	2.48E-51	up
ENSG00000130635	COL5A1	protein_coding	1.377445809	8.89E-53	3.26E-51	up
ENSG00000100139	MICALL1	protein_coding	1.408753992	9.94E-53	3.64E-51	up
ENSG00000121749	TBC1D15	protein_coding	-1.396283956	1.23E-52	4.50E-51	down
ENSG00000168393	DTYMK	protein_coding	-1.237017546	1.47E-52	5.33E-51	down
ENSG00000101811	CSTF2	protein_coding	-1.910701884	1.49E-52	5.38E-51	down
ENSG00000108826	MRPL27	protein_coding	-1.401856732	1.59E-52	5.70E-51	down
ENSG00000103326	CAPN15	protein_coding	-2.057007986	1.81E-52	6.49E-51	down
ENSG00000136925	TSTD2	protein_coding	-2.560760327	2.20E-52	7.88E-51	down
ENSG00000004468	CD38	protein_coding	1.653118757	3.21E-52	1.14E-50	up
ENSG00000110104	CCDC86	protein_coding	-1.132085066	4.16E-52	1.47E-50	down
ENSG00000124766	SOX4	protein_coding	-1.290206278	4.96E-52	1.75E-50	down
ENSG00000139625	MAP3K12	protein_coding	1.474540561	5.23E-52	1.84E-50	up
ENSG00000163814	CDCP1	protein_coding	-1.586780665	5.52E-52	1.94E-50	down
ENSG00000181143	MUC16	protein_coding	2.525393538	6.20E-52	2.17E-50	up
ENSG00000116096	SPR	protein_coding	-1.721960978	8.18E-52	2.85E-50	down
ENSG00000106541	AGR2	protein_coding	1.40653872	8.66E-52	3.01E-50	up
ENSG00000224877	NDUFAF8	protein_coding	-1.204979527	9.15E-52	3.18E-50	down
ENSG00000181381	DDX60L	protein_coding	-2.665231838	9.95E-52	3.45E-50	down
ENSG00000183098	GPC6	protein_coding	2.178395859	1.05E-51	3.64E-50	up
ENSG00000113657	DPYSL3	protein_coding	1.13180867	2.15E-51	7.40E-50	up
ENSG00000148677	ANKRD1	protein_coding	2.240692859	2.32E-51	7.96E-50	up
ENSG00000166619	BLCAP	protein_coding	-1.859885137	5.94E-51	2.04E-49	down
ENSG00000174840	PDE12	protein_coding	-1.916733918	6.41E-51	2.19E-49	down
ENSG00000171453	POLR1C	protein_coding	-2.155899416	6.70E-51	2.28E-49	down
ENSG00000048649	RSF1	protein_coding	1.036620683	6.71E-51	2.28E-49	up
ENSG00000147475	ERLIN2	protein_coding	1.177447329	8.13E-51	2.75E-49	up
ENSG00000163870	TPRA1	protein_coding	-2.60181336	9.39E-51	3.17E-49	down
ENSG00000111775	COX6A1	protein_coding	1.513403936	4.56E-50	1.53E-48	up
ENSG00000135338	LCA5	protein_coding	2.887267168	5.08E-50	1.70E-48	up
ENSG00000117528	ABCD3	protein_coding	1.162100193	9.31E-50	3.10E-48	up
ENSG00000136854	STXBP1	protein_coding	1.078346603	1.38E-49	4.57E-48	up
ENSG00000169231	THBS3	protein_coding	1.95373584	2.68E-49	8.78E-48	up
ENSG00000164543	STK17A	protein_coding	-1.346881243	3.92E-49	1.28E-47	down
ENSG00000140961	OSGIN1	protein_coding	-1.476526902	4.77E-49	1.54E-47	down
ENSG00000135111	TBX3	protein_coding	-1.168117874	4.82E-49	1.56E-47	down
ENSG00000137492	THAP12	protein_coding	-1.169340676	5.77E-49	1.85E-47	down
ENSG00000106804	C5	protein_coding	2.384679286	7.43E-49	2.38E-47	up
ENSG00000113916	BCL6	protein_coding	1.96022976	7.51E-49	2.40E-47	up
ENSG00000117155	SSX2IP	protein_coding	-1.267453314	1.08E-48	3.44E-47	down
ENSG00000173599	PC	protein_coding	1.39412455	1.59E-48	5.04E-47	up
ENSG00000103528	SYT17	protein_coding	2.405293407	1.74E-48	5.52E-47	up

ENSG00000079112	CDH17	protein_coding	4.166785138	2.55E-48	8.06E-47	up
ENSG00000134762	DSC3	protein_coding	2.709626376	3.29E-48	1.04E-46	up
ENSG00000184992	BRI3BP	protein_coding	-1.022618752	4.62E-48	1.45E-46	down
ENSG00000163346	PBXIP1	protein_coding	1.296546094	7.78E-48	2.44E-46	up
ENSG00000113621	TXNDC15	protein_coding	-1.18292848	9.29E-48	2.89E-46	down
ENSG00000069011	PITX1	protein_coding	-1.518965862	1.81E-47	5.60E-46	down
ENSG00000127324	TSPAN8	protein_coding	8.743573708	2.77E-47	8.57E-46	up
ENSG00000100154	TTC28	protein_coding	1.278186233	2.79E-47	8.62E-46	up
ENSG00000126767	ELK1	protein_coding	-1.871762442	2.85E-47	8.79E-46	down
ENSG00000152700	SAR1B	protein_coding	-1.324793623	3.04E-47	9.34E-46	down
ENSG00000123178	SPRYD7	protein_coding	-2.180791854	4.10E-47	1.25E-45	down
ENSG00000233276	GPX1	protein_coding	-1.287961866	5.96E-47	1.81E-45	down
ENSG00000065559	MAP2K4	protein_coding	-1.342071319	6.37E-47	1.93E-45	down
ENSG00000184922	FMNL1	protein_coding	-1.032273413	6.76E-47	2.05E-45	down
ENSG00000101911	PRPS2	protein_coding	-1.525029979	8.61E-47	2.60E-45	down
ENSG00000185344	ATP6V0A2	protein_coding	-1.773701985	1.39E-46	4.18E-45	down
ENSG00000114861	FOXP1	protein_coding	1.524533912	1.95E-46	5.87E-45	up
ENSG00000169896	ITGAM	protein_coding	7.537989574	2.55E-46	7.63E-45	up
ENSG00000101868	POLA1	protein_coding	-1.133866977	3.03E-46	9.04E-45	down
ENSG00000172716	SLFN11	protein_coding	-9.011313794	3.55E-46	1.06E-44	down
ENSG00000151690	MFSD6	protein_coding	1.22869197	3.63E-46	1.08E-44	up
ENSG00000177971	IMP3	protein_coding	-1.278240654	5.34E-46	1.58E-44	down
ENSG00000049323	LTBP1	protein_coding	-7.818668717	5.41E-46	1.59E-44	down
ENSG00000167302	TEPSIN	protein_coding	-3.511240191	7.25E-46	2.13E-44	down
ENSG00000141741	MIEN1	protein_coding	-1.725911576	7.47E-46	2.19E-44	down
ENSG00000269343	ZNF587B	protein_coding	-1.815157251	8.21E-46	2.41E-44	down
ENSG00000112110	MRPL18	protein_coding	-1.22705744	8.35E-46	2.44E-44	down
ENSG00000135241	PNPLA8	protein_coding	1.61335113	9.23E-46	2.70E-44	up
ENSG00000173141	MRPL57	protein_coding	-1.33490035	1.20E-45	3.46E-44	down
ENSG00000196562	SULF2	protein_coding	1.200372327	1.30E-45	3.75E-44	up
ENSG00000221823	PPP3R1	protein_coding	-1.050364072	2.02E-45	5.79E-44	down
ENSG00000150347	ARID5B	protein_coding	2.096454731	3.71E-45	1.06E-43	up
ENSG00000102753	KPNA3	protein_coding	-1.434763534	4.06E-45	1.15E-43	down
ENSG00000198157	HMGN5	protein_coding	2.238675596	5.63E-45	1.59E-43	up
ENSG00000103534	TMC5	protein_coding	-2.518441486	6.76E-45	1.90E-43	down
ENSG00000257365	FNTB	protein_coding	-3.063781214	6.77E-45	1.90E-43	down
ENSG00000004866	ST7	protein_coding	-3.003660222	7.96E-45	2.23E-43	down
ENSG00000167767	KRT80	protein_coding	1.206880416	8.63E-45	2.41E-43	up
ENSG00000144560	VGLL4	protein_coding	1.070013249	2.03E-44	5.63E-43	up
ENSG00000160953	MUM1	protein_coding	-1.589736656	2.06E-44	5.69E-43	down
ENSG00000103494	RPGRIP1L	protein_coding	-1.732864336	3.31E-44	9.10E-43	down
ENSG00000179348	GATA2	protein_coding	-3.980452831	3.36E-44	9.24E-43	down
ENSG00000126107	HECTD3	protein_coding	1.814540229	4.94E-44	1.35E-42	up
ENSG00000068438	FTSJ1	protein_coding	-1.772909055	5.16E-44	1.40E-42	down
ENSG00000133739	LRRCC1	protein_coding	2.503903295	7.55E-44	2.05E-42	up
ENSG00000166548	TK2	protein_coding	1.859050925	8.60E-44	2.33E-42	up
ENSG00000174442	ZWILCH	protein_coding	-1.250419226	1.09E-43	2.94E-42	down
ENSG00000166333	ILK	protein_coding	1.277005908	2.22E-43	5.90E-42	up
ENSG00000101493	ZNF516	protein_coding	2.320498889	2.69E-43	7.12E-42	up
ENSG00000048028	USP28	protein_coding	-1.098828161	2.74E-43	7.25E-42	down
ENSG00000196782	MAML3	protein_coding	1.155119808	3.16E-43	8.36E-42	up
ENSG00000102287	GABRE	protein_coding	1.711393302	3.17E-43	8.36E-42	up
ENSG00000100124	ANKRD54	protein_coding	-4.352350712	5.47E-43	1.43E-41	down
ENSG00000095015	MAP3K1	protein_coding	-1.721620467	5.63E-43	1.47E-41	down

ENSG00000143512	HHIPL2	protein_coding	1.048200789	6.18E-43	1.61E-41	up
ENSG00000132963	POMP	protein_coding	-1.238278946	1.36E-42	3.53E-41	down
ENSG00000166165	CKB	protein_coding	1.668510759	1.37E-42	3.53E-41	up
ENSG00000065361	ERBB3	protein_coding	4.246074048	2.03E-42	5.22E-41	up
ENSG00000197555	SIPA1L1	protein_coding	1.338199306	2.20E-42	5.66E-41	up
ENSG00000117114	ADGRL2	protein_coding	1.211282429	2.41E-42	6.18E-41	up
ENSG00000099977	DDT	protein_coding	1.821240201	2.72E-42	6.97E-41	up
ENSG00000161249	DMKN	protein_coding	2.88740948	3.35E-42	8.56E-41	up
ENSG00000100889	PCK2	protein_coding	1.831800891	3.48E-42	8.87E-41	up
ENSG00000155465	SLC7A7	protein_coding	1.903848539	4.28E-42	1.09E-40	up
ENSG00000143971	ETAA1	protein_coding	-1.498184038	4.31E-42	1.09E-40	down
ENSG00000167394	ZNF668	protein_coding	-2.764975677	4.49E-42	1.14E-40	down
ENSG00000182197	EXT1	protein_coding	1.30447531	4.56E-42	1.16E-40	up
ENSG00000083544	TDRD3	protein_coding	1.821915159	5.68E-42	1.44E-40	up
ENSG00000108984	MAP2K6	protein_coding	-2.442292838	6.12E-42	1.54E-40	down
ENSG00000184232	OAF	protein_coding	-3.99893007	8.64E-42	2.16E-40	down
ENSG00000117751	PPP1R8	protein_coding	-1.611091174	9.68E-42	2.41E-40	down
ENSG00000121931	LRIF1	protein_coding	-2.143044493	9.82E-42	2.44E-40	down
ENSG00000235162	C12orf75	protein_coding	-1.549499524	1.02E-41	2.52E-40	down
ENSG00000171492	LRRC8D	protein_coding	-1.452491431	1.35E-41	3.35E-40	down
ENSG00000162409	PRKAA2	protein_coding	1.477498848	1.42E-41	3.50E-40	up
ENSG00000197296	FITM2	protein_coding	1.773568448	1.42E-41	3.50E-40	up
ENSG00000107815	TWNK	protein_coding	-1.273966874	1.66E-41	4.09E-40	down
ENSG00000188375	H3F3C	protein_coding	-1.743940453	1.97E-41	4.85E-40	down
ENSG00000185561	TLCD2	protein_coding	2.295558886	2.18E-41	5.35E-40	up
ENSG00000214706	IFRD2	protein_coding	-1.768457271	3.10E-41	7.58E-40	down
ENSG00000122643	NT5C3A	protein_coding	-1.849001977	3.92E-41	9.51E-40	down
ENSG00000121064	SCPEP1	protein_coding	-2.60171838	4.12E-41	9.98E-40	down
ENSG00000187678	SPRY4	protein_coding	-2.887401191	4.47E-41	1.08E-39	down
ENSG00000184220	CMSS1	protein_coding	-1.241672526	4.90E-41	1.18E-39	down
ENSG00000160190	SLC37A1	protein_coding	-5.909868531	6.95E-41	1.67E-39	down
ENSG00000090565	RAB11FIP3	protein_coding	-1.298246468	1.04E-40	2.49E-39	down
ENSG00000065485	PDIA5	protein_coding	1.338688925	1.88E-40	4.49E-39	up
ENSG00000072135	PTPN18	protein_coding	-1.865040061	1.92E-40	4.57E-39	down
ENSG00000167112	TRUB2	protein_coding	1.06762706	2.14E-40	5.10E-39	up
ENSG00000164307	ERAP1	protein_coding	-2.584230954	2.38E-40	5.66E-39	down
ENSG00000151726	ACSL1	protein_coding	-1.368969072	3.39E-40	8.03E-39	down
ENSG00000137700	SLC37A4	protein_coding	1.578584973	3.70E-40	8.74E-39	up
ENSG00000163882	POLR2H	protein_coding	-2.633731144	4.87E-40	1.15E-38	down
ENSG00000107331	ABCA2	protein_coding	1.117171933	4.87E-40	1.15E-38	up
ENSG00000064309	CDON	protein_coding	1.457455689	4.96E-40	1.17E-38	up
ENSG00000149182	ARFGAP2	protein_coding	-2.154981029	5.48E-40	1.28E-38	down
ENSG00000115902	SLC1A4	protein_coding	-2.037227795	8.03E-40	1.87E-38	down
ENSG00000247077	PGAM5	protein_coding	-1.174188031	1.46E-39	3.39E-38	down
ENSG00000188158	NHS	protein_coding	-2.463751266	1.61E-39	3.72E-38	down
ENSG00000225973	PIGBOS1	protein_coding	-3.343889134	1.66E-39	3.83E-38	down
ENSG00000255112	CHMP1B	protein_coding	-1.111859788	2.15E-39	4.93E-38	down
ENSG00000149212	SESN3	protein_coding	3.203005326	2.29E-39	5.25E-38	up
ENSG00000136828	RALGPS1	protein_coding	2.26497108	2.78E-39	6.34E-38	up
ENSG00000083099	LYRM2	protein_coding	-2.112036383	2.82E-39	6.44E-38	down
ENSG00000065325	GLP2R	protein_coding	1.792373006	3.01E-39	6.85E-38	up
ENSG00000115289	PCGF1	protein_coding	-3.336153485	3.13E-39	7.12E-38	down
ENSG00000163898	LIPH	protein_coding	2.337581348	3.26E-39	7.42E-38	up
ENSG00000095794	CREM	protein_coding	-1.792479193	3.39E-39	7.69E-38	down

ENSG00000264230	ANXA8L1	protein_coding	1.496297553	3.50E-39	7.92E-38	up
ENSG00000116809	ZBTB17	protein_coding	-4.011313794	4.25E-39	9.59E-38	down
ENSG00000100836	PABPN1	protein_coding	-1.735260442	4.58E-39	1.03E-37	down
ENSG00000176406	RIMS2	protein_coding	2.979771929	4.68E-39	1.05E-37	up
ENSG00000067533	RRP15	protein_coding	-1.217607043	1.14E-38	2.55E-37	down
ENSG00000166106	ADAMTS15	protein_coding	4.436064673	1.26E-38	2.82E-37	up
ENSG00000146670	CDCA5	protein_coding	-1.479804498	1.44E-38	3.21E-37	down
ENSG00000086200	IPO11	protein_coding	-1.340388603	1.61E-38	3.58E-37	down
ENSG00000119673	ACOT2	protein_coding	-2.173405706	1.78E-38	3.95E-37	down
ENSG00000145908	ZNF300	protein_coding	1.786741567	2.94E-38	6.50E-37	up
ENSG00000169519	METTL15	protein_coding	-2.590845331	2.96E-38	6.53E-37	down
ENSG00000136897	MRPL50	protein_coding	-1.037464776	3.80E-38	8.35E-37	down
ENSG00000088356	PDRG1	protein_coding	-3.020497117	4.73E-38	1.04E-36	down
ENSG00000006327	TNFRSF12A	protein_coding	-1.813528989	5.52E-38	1.21E-36	down
ENSG00000197696	NMB	protein_coding	-4.19188604	6.22E-38	1.36E-36	down
ENSG00000183421	RIPK4	protein_coding	2.357105361	6.29E-38	1.37E-36	up
ENSG00000185201	IFITM2	protein_coding	-6.395977645	6.49E-38	1.41E-36	down
ENSG00000011638	TMEM159	protein_coding	2.808226666	8.95E-38	1.94E-36	up
ENSG00000086504	MRPL28	protein_coding	-1.345526513	9.37E-38	2.02E-36	down
ENSG00000167720	SRR	protein_coding	-3.202845252	9.84E-38	2.13E-36	down
ENSG00000122696	SLC25A51	protein_coding	-2.617173636	1.05E-37	2.25E-36	down
ENSG00000082074	FYB1	protein_coding	1.445851644	1.11E-37	2.38E-36	up
ENSG00000118985	ELL2	protein_coding	-1.48801765	1.18E-37	2.54E-36	down
ENSG00000166046	TCP11L2	protein_coding	2.419684096	1.36E-37	2.90E-36	up
ENSG00000163125	RPRD2	protein_coding	1.067027657	1.36E-37	2.90E-36	up
ENSG00000161204	ABCF3	protein_coding	-1.588502343	1.40E-37	2.97E-36	down
ENSG00000128274	A4GALT	protein_coding	1.680642452	1.54E-37	3.27E-36	up
ENSG00000185989	RASA3	protein_coding	1.30555289	1.61E-37	3.41E-36	up
ENSG00000139133	ALG10	protein_coding	2.077815064	1.78E-37	3.77E-36	up
ENSG00000143553	SNAPIN	protein_coding	-1.824994355	2.21E-37	4.68E-36	down
ENSG00000111412	C12orf49	protein_coding	-1.147154224	2.59E-37	5.46E-36	down
ENSG00000187187	ZNF546	protein_coding	2.828107218	2.59E-37	5.46E-36	up
ENSG00000123892	RAB38	protein_coding	3.572843406	3.22E-37	6.77E-36	up
ENSG00000059145	UNKL	protein_coding	-1.477798059	3.27E-37	6.87E-36	down
ENSG00000175221	MED16	protein_coding	-2.604349596	3.38E-37	7.10E-36	down
ENSG00000239306	RBM14	protein_coding	-1.067943505	3.80E-37	7.96E-36	down
ENSG00000106077	ABHD11	protein_coding	-4.608942707	3.96E-37	8.27E-36	down
ENSG00000172893	DHCR7	protein_coding	-1.022995599	4.18E-37	8.73E-36	down
ENSG00000178695	KCTD12	protein_coding	2.925777039	4.76E-37	9.91E-36	up
ENSG00000158122	AAED1	protein_coding	-2.405803916	4.79E-37	9.95E-36	down
ENSG00000102098	SCML2	protein_coding	-2.523212833	4.84E-37	1.01E-35	down
ENSG00000133731	IMPA1	protein_coding	-1.726505155	5.21E-37	1.08E-35	down
ENSG00000171365	CLCN5	protein_coding	-2.682691047	6.97E-37	1.44E-35	down
ENSG00000225828	FAM229A	protein_coding	2.997814995	8.02E-37	1.65E-35	up
ENSG00000106066	CPVL	protein_coding	1.530579984	8.41E-37	1.73E-35	up
ENSG00000125734	GPR108	protein_coding	1.285096947	9.33E-37	1.91E-35	up
ENSG00000073350	LLGL2	protein_coding	1.373513117	1.08E-36	2.21E-35	up
ENSG00000113790	EHHADH	protein_coding	7.144453144	1.11E-36	2.27E-35	up
ENSG00000102265	TIMP1	protein_coding	-1.022135288	1.12E-36	2.29E-35	down
ENSG00000107281	NPDC1	protein_coding	1.882550121	1.35E-36	2.75E-35	up
ENSG00000181638	ZFP41	protein_coding	-3.832110979	1.89E-36	3.82E-35	down
ENSG00000182568	SATB1	protein_coding	8.274955168	2.04E-36	4.11E-35	up
ENSG00000109321	AREG	protein_coding	-1.952234562	2.04E-36	4.11E-35	down
ENSG00000111860	CEP85L	protein_coding	1.875170591	3.10E-36	6.22E-35	up

ENSG00000184897	H1FX	protein_coding	-1.059110783	3.20E-36	6.40E-35	down
ENSG00000102103	PQBP1	protein_coding	-1.024444469	3.27E-36	6.52E-35	down
ENSG00000005249	PRKAR2B	protein_coding	-6.322515483	3.38E-36	6.72E-35	down
ENSG00000164284	GRPEL2	protein_coding	-1.411704518	3.40E-36	6.76E-35	down
ENSG00000170836	PPM1D	protein_coding	-2.122240945	3.77E-36	7.49E-35	down
ENSG00000165175	MID1IP1	protein_coding	-1.468424612	3.95E-36	7.83E-35	down
ENSG00000139344	AMDHD1	protein_coding	-2.871136136	4.08E-36	8.07E-35	down
ENSG00000182512	GLRX5	protein_coding	-1.12401231	4.55E-36	8.99E-35	down
ENSG00000038295	TLL1	protein_coding	-8.56020704	4.92E-36	9.71E-35	down
ENSG00000204304	PBX2	protein_coding	1.652808244	4.97E-36	9.79E-35	up
ENSG00000129521	EGLN3	protein_coding	1.647358392	5.13E-36	1.01E-34	up
ENSG00000148339	SLC25A25	protein_coding	1.421645613	5.23E-36	1.03E-34	up
ENSG00000123395	ATG101	protein_coding	-1.630550598	5.32E-36	1.05E-34	down
ENSG00000090520	DNAJB11	protein_coding	-1.221322491	5.61E-36	1.10E-34	down
ENSG00000012171	SEMA3B	protein_coding	2.932607532	6.27E-36	1.23E-34	up
ENSG00000127152	BCL11B	protein_coding	2.49846121	6.73E-36	1.31E-34	up
ENSG00000104472	CHRAC1	protein_coding	-1.452177281	7.41E-36	1.44E-34	down
ENSG00000170500	LONRF2	protein_coding	-1.097227697	7.59E-36	1.48E-34	down
ENSG00000110427	KIAA1549L	protein_coding	-8.541828511	1.14E-35	2.20E-34	down
ENSG00000056998	GYG2	protein_coding	-3.890632312	1.92E-35	3.70E-34	down
ENSG00000137038	DMAC1	protein_coding	-2.980452831	2.10E-35	4.03E-34	down
ENSG00000169136	ATF5	protein_coding	-4.104423199	2.12E-35	4.06E-34	down
ENSG00000168944	CEP120	protein_coding	-1.57129963	2.28E-35	4.35E-34	down
ENSG00000132612	VPS4A	protein_coding	-1.985264006	2.29E-35	4.37E-34	down
ENSG00000152413	HOMER1	protein_coding	-7.364950749	2.72E-35	5.18E-34	down
ENSG00000104886	PLEKHJ1	protein_coding	-2.496253307	2.96E-35	5.62E-34	down
ENSG00000074855	ANO8	protein_coding	4.743573708	2.99E-35	5.66E-34	up
ENSG00000197779	ZNF81	protein_coding	-3.706459213	3.22E-35	6.10E-34	down
ENSG00000184831	APOO	protein_coding	-3.706459213	3.22E-35	6.10E-34	down
ENSG00000139278	GLIPR1	protein_coding	-1.918608295	3.46E-35	6.53E-34	down
ENSG00000151883	PARP8	protein_coding	1.791119292	4.69E-35	8.84E-34	up
ENSG00000105879	CBLL1	protein_coding	-1.125554067	5.23E-35	9.84E-34	down
ENSG00000254093	PINX1	protein_coding	-2.311334633	5.37E-35	1.01E-33	down
ENSG00000074590	NUAK1	protein_coding	1.72320591	5.78E-35	1.08E-33	up
ENSG00000151304	SRFBP1	protein_coding	-1.952032857	5.83E-35	1.09E-33	down
ENSG00000261236	BOP1	protein_coding	-1.023062753	5.95E-35	1.11E-33	down
ENSG00000164442	CITED2	protein_coding	1.941987266	6.36E-35	1.19E-33	up
ENSG00000156304	SCAF4	protein_coding	-1.109043859	6.72E-35	1.25E-33	down
ENSG00000101076	HNF4A	protein_coding	2.556642281	1.38E-34	2.57E-33	up
ENSG00000072840	EVC	protein_coding	1.247499636	1.39E-34	2.58E-33	up
ENSG00000138031	ADCY3	protein_coding	-1.042830819	1.58E-34	2.92E-33	down
ENSG00000095303	PTGS1	protein_coding	8.178201935	1.71E-34	3.18E-33	up
ENSG00000186193	SAPCD2	protein_coding	1.174972179	1.89E-34	3.49E-33	up
ENSG00000178074	C2orf69	protein_coding	-1.845961523	2.02E-34	3.72E-33	down
ENSG00000124333	VAMP7	protein_coding	-1.310022301	2.04E-34	3.76E-33	down
ENSG00000033327	GAB2	protein_coding	1.903569592	2.07E-34	3.81E-33	up
ENSG00000168679	SLC16A4	protein_coding	1.278064162	2.20E-34	4.05E-33	up
ENSG00000155592	ZKSCAN2	protein_coding	-2.079961141	2.61E-34	4.79E-33	down
ENSG00000142784	WDTC1	protein_coding	1.833894315	2.91E-34	5.33E-33	up
ENSG00000159314	ARHGAP27	protein_coding	-1.761800528	3.11E-34	5.68E-33	down
ENSG00000273611	ZNHIT3	protein_coding	-2.199842764	3.20E-34	5.85E-33	down
ENSG00000117748	RPA2	protein_coding	-1.874664948	3.67E-34	6.69E-33	down
ENSG00000124243	BCAS4	protein_coding	-3.015418193	3.99E-34	7.26E-33	down
ENSG00000160799	CCDC12	protein_coding	-2.347028841	4.60E-34	8.36E-33	down

ENSG00000188807	TMEM201	protein_coding	-2.183575727	4.73E-34	8.57E-33	down
ENSG00000104142	VPS18	protein_coding	-1.554866034	7.00E-34	1.26E-32	down
ENSG00000179912	R3HDM2	protein_coding	1.181565192	8.35E-34	1.50E-32	up
ENSG00000008513	ST3GAL1	protein_coding	1.144035205	9.19E-34	1.65E-32	up
ENSG00000125731	SH2D3A	protein_coding	3.459780742	9.98E-34	1.79E-32	up
ENSG00000189180	ZNF33A	protein_coding	-1.393308099	1.01E-33	1.81E-32	down
ENSG00000087266	SH3BP2	protein_coding	1.963140085	1.04E-33	1.87E-32	up
ENSG00000058668	ATP2B4	protein_coding	1.203005326	1.39E-33	2.47E-32	up
ENSG00000172671	ZFAND4	protein_coding	-3.648743715	1.54E-33	2.73E-32	down
ENSG00000087510	TFAP2C	protein_coding	1.285195074	1.71E-33	3.02E-32	up
ENSG00000172795	DCP2	protein_coding	-1.510999119	1.72E-33	3.04E-32	down
ENSG00000106537	TSPAN13	protein_coding	1.996993025	2.24E-33	3.93E-32	up
ENSG00000125375	ATP5S	protein_coding	1.926098746	2.28E-33	4.00E-32	up
ENSG00000134490	TMEM241	protein_coding	-4.900282482	2.40E-33	4.19E-32	down
ENSG00000117305	HMGCL	protein_coding	1.845935425	2.43E-33	4.25E-32	up
ENSG00000115457	IGFBP2	protein_coding	-1.264600224	2.89E-33	5.04E-32	down
ENSG00000150977	RILPL2	protein_coding	-3.362625696	3.48E-33	6.05E-32	down
ENSG00000198920	KIAA0753	protein_coding	1.097819523	4.31E-33	7.49E-32	up
ENSG00000122490	PQLC1	protein_coding	3.173561548	4.48E-33	7.78E-32	up
ENSG00000121964	GTDC1	protein_coding	-6.17528953	5.67E-33	9.81E-32	down
ENSG00000075407	ZNF37A	protein_coding	-2.329405738	8.48E-33	1.46E-31	down
ENSG00000165240	ATP7A	protein_coding	1.046022795	8.98E-33	1.55E-31	up
ENSG00000132359	RAP1GAP2	protein_coding	1.948688138	1.19E-32	2.04E-31	up
ENSG00000110200	ANAPC15	protein_coding	1.4585422	1.38E-32	2.36E-31	up
ENSG00000124383	MPHOSPH10	protein_coding	-1.117263545	1.46E-32	2.49E-31	down
ENSG00000249992	TMEM158	protein_coding	-8.375367365	1.58E-32	2.69E-31	down
ENSG00000162852	CNST	protein_coding	-1.409161532	1.89E-32	3.21E-31	down
ENSG00000174989	FBXW8	protein_coding	1.448330721	1.90E-32	3.23E-31	up
ENSG00000178502	KLHL11	protein_coding	-2.075185978	2.12E-32	3.59E-31	down
ENSG00000005194	CIAPIN1	protein_coding	-1.368217118	2.60E-32	4.37E-31	down
ENSG00000079308	TNS1	protein_coding	4.203005326	2.82E-32	4.75E-31	up
ENSG00000111885	MAN1A1	protein_coding	-2.416082958	2.84E-32	4.78E-31	down
ENSG00000205413	SAMD9	protein_coding	-5.151244056	3.22E-32	5.39E-31	down
ENSG00000015532	XYLT2	protein_coding	-1.171750985	3.23E-32	5.42E-31	down
ENSG00000134108	ARL8B	protein_coding	-1.057458216	3.34E-32	5.58E-31	down
ENSG00000100625	SIX4	protein_coding	1.870245511	3.84E-32	6.41E-31	up
ENSG00000129925	TMEM8A	protein_coding	-2.046972927	4.21E-32	7.00E-31	down
ENSG00000154240	CEP112	protein_coding	-1.444630027	4.31E-32	7.16E-31	down
ENSG00000006757	PNPLA4	protein_coding	-8.343889134	5.78E-32	9.58E-31	down
ENSG00000162378	ZYG11B	protein_coding	-1.314818087	5.98E-32	9.89E-31	down
ENSG00000146067	FAM193B	protein_coding	-2.666302086	6.14E-32	1.01E-30	down
ENSG00000181004	BBS12	protein_coding	2.458932846	6.35E-32	1.05E-30	up
ENSG00000143850	PLEKHA6	protein_coding	1.182315468	6.52E-32	1.08E-30	up
ENSG00000095777	MYO3A	protein_coding	2.980612905	6.72E-32	1.10E-30	up
ENSG00000005486	RHBDD2	protein_coding	-1.431512999	6.79E-32	1.12E-30	down
ENSG00000170653	ATF7	protein_coding	1.352515071	7.55E-32	1.24E-30	up
ENSG00000136159	NUDT15	protein_coding	-2.303292897	7.87E-32	1.29E-30	down
ENSG00000112651	MRPL2	protein_coding	-1.478818714	8.18E-32	1.34E-30	down
ENSG00000185499	MUC1	protein_coding	4.184146299	8.36E-32	1.37E-30	up
ENSG00000088876	ZNF343	protein_coding	-3.256426292	9.66E-32	1.57E-30	down
ENSG00000104447	TRPS1	protein_coding	2.001976665	9.69E-32	1.58E-30	up
ENSG00000088538	DOCK3	protein_coding	1.650464303	9.85E-32	1.60E-30	up
ENSG00000166526	ZNF3	protein_coding	1.397248959	1.22E-31	1.98E-30	up
ENSG00000172273	HINFP	protein_coding	1.904807749	1.23E-31	2.00E-30	up

ENSG00000197043	ANXA6	protein_coding	1.22308691	1.36E-31	2.19E-30	up
ENSG00000198331	HYLS1	protein_coding	-1.444053295	1.45E-31	2.35E-30	down
ENSG00000142039	CCDC97	protein_coding	-1.289386491	1.54E-31	2.49E-30	down
ENSG00000033011	ALG1	protein_coding	-2.395324398	1.56E-31	2.51E-30	down
ENSG00000186814	ZSCAN30	protein_coding	2.300950377	1.71E-31	2.75E-30	up
ENSG00000166166	TRMT61A	protein_coding	-1.580370733	1.72E-31	2.77E-30	down
ENSG00000134070	IRAK2	protein_coding	-1.352310727	1.94E-31	3.11E-30	down
ENSG00000162522	KIAA1522	protein_coding	1.075803466	2.01E-31	3.23E-30	up
ENSG00000101945	SUV39H1	protein_coding	-1.746529607	2.40E-31	3.83E-30	down
ENSG00000167118	URM1	protein_coding	1.176749427	2.49E-31	3.97E-30	up
ENSG00000121957	GPSM2	protein_coding	1.258146881	2.54E-31	4.05E-30	up
ENSG00000144645	OSBPL10	protein_coding	1.373361408	2.92E-31	4.64E-30	up
ENSG00000163743	RCHY1	protein_coding	-2.20033173	3.09E-31	4.91E-30	down
ENSG00000135365	PHF21A	protein_coding	1.065501803	3.17E-31	5.03E-30	up
ENSG00000160113	NR2F6	protein_coding	1.239410318	3.28E-31	5.19E-30	up
ENSG00000176371	ZSCAN2	protein_coding	-2.961298257	3.29E-31	5.20E-30	down
ENSG00000127580	WDR24	protein_coding	-8.300820412	3.29E-31	5.21E-30	down
ENSG00000005108	THSD7A	protein_coding	1.2276073	3.38E-31	5.33E-30	up
ENSG00000205208	C4orf46	protein_coding	2.022997074	3.58E-31	5.65E-30	up
ENSG00000137100	DCTN3	protein_coding	-1.399384246	3.73E-31	5.87E-30	down
ENSG00000149476	TKFC	protein_coding	1.199638475	4.18E-31	6.56E-30	up
ENSG00000171103	TRMT61B	protein_coding	-1.721649549	4.87E-31	7.63E-30	down
ENSG00000142327	RNPEPL1	protein_coding	1.392408392	5.08E-31	7.95E-30	up
ENSG00000107951	MTPAP	protein_coding	-1.494005117	5.69E-31	8.88E-30	down
ENSG00000165757	JCAD	protein_coding	-6.076605255	5.81E-31	9.04E-30	down
ENSG00000166582	CENPV	protein_coding	-1.775155095	6.06E-31	9.42E-30	down
ENSG00000086712	TXLNG	protein_coding	-1.324292323	7.01E-31	1.09E-29	down
ENSG00000188343	FAM92A	protein_coding	-3.172902937	7.28E-31	1.13E-29	down
ENSG00000005075	POLR2J	protein_coding	-1.976648623	7.33E-31	1.14E-29	down
ENSG00000136933	RABEPK	protein_coding	-1.93123541	7.37E-31	1.14E-29	down
ENSG00000185404	SP140L	protein_coding	-4.792479193	7.38E-31	1.14E-29	down
ENSG00000163950	SLBP	protein_coding	-1.043732172	1.35E-30	2.07E-29	down
ENSG00000154122	ANKH	protein_coding	1.307702705	1.56E-30	2.38E-29	up
ENSG00000135698	MPHOSPH6	protein_coding	-1.332636859	1.61E-30	2.46E-29	down
ENSG00000119862	LGALSL	protein_coding	-4.541828511	1.64E-30	2.50E-29	down
ENSG00000047457	CP	protein_coding	1.125821272	1.67E-30	2.54E-29	up
ENSG00000146950	SHROOM2	protein_coding	-8.256426292	1.90E-30	2.89E-29	down
ENSG00000139437	TCHP	protein_coding	-1.611020576	2.32E-30	3.50E-29	down
ENSG00000135315	CEP162	protein_coding	2.390463958	2.40E-30	3.61E-29	up
ENSG00000197961	ZNF121	protein_coding	-1.180546327	2.43E-30	3.66E-29	down
ENSG00000008282	SYPL1	protein_coding	-1.082609476	2.49E-30	3.75E-29	down
ENSG00000198856	OSTC	protein_coding	-1.010921811	2.55E-30	3.83E-29	down
ENSG00000185920	PTCH1	protein_coding	1.649530218	2.67E-30	4.00E-29	up
ENSG00000112294	ALDH5A1	protein_coding	-2.098244624	2.92E-30	4.36E-29	down
ENSG00000160446	ZDHHC12	protein_coding	-1.580458975	3.04E-30	4.54E-29	down
ENSG00000175854	SWI5	protein_coding	1.387792794	3.06E-30	4.56E-29	up
ENSG00000161980	POLR3K	protein_coding	-2.145867917	3.36E-30	5.00E-29	down
ENSG00000102109	PCSK1N	protein_coding	-3.008988741	3.39E-30	5.04E-29	down
ENSG00000163611	SPICE1	protein_coding	1.140178489	3.63E-30	5.40E-29	up
ENSG00000162664	ZNF326	protein_coding	-1.374189873	3.78E-30	5.61E-29	down
ENSG00000187051	RPS19BP1	protein_coding	-1.041311436	3.89E-30	5.76E-29	down
ENSG00000166398	KIAA0355	protein_coding	1.060732558	4.54E-30	6.72E-29	up
ENSG00000152078	TMEM56	protein_coding	1.203005326	4.87E-30	7.18E-29	up
ENSG00000164938	TP53INP1	protein_coding	1.527469657	5.32E-30	7.82E-29	up

ENSG00000007047	MARK4	protein_coding	2.220011752	6.82E-30	9.99E-29	up
ENSG00000140006	WDR89	protein_coding	-1.961183306	6.92E-30	1.01E-28	down
ENSG00000160199	PKNOX1	protein_coding	-3.188407869	7.93E-30	1.16E-28	down
ENSG00000181472	ZBTB2	protein_coding	-2.502405534	9.73E-30	1.42E-28	down
ENSG00000184368	MAP7D2	protein_coding	-8.210622603	1.11E-29	1.62E-28	down
ENSG00000198298	ZNF485	protein_coding	-4.504353806	1.14E-29	1.65E-28	down
ENSG00000140326	CDAN1	protein_coding	-5.426351294	1.22E-29	1.78E-28	down
ENSG00000213246	SUPT4H1	protein_coding	-1.227397244	1.32E-29	1.91E-28	down
ENSG00000148481	MINDY3	protein_coding	1.615566095	1.33E-29	1.93E-28	up
ENSG00000108439	PNPO	protein_coding	-1.171537395	1.61E-29	2.33E-28	down
ENSG00000117143	UAP1	protein_coding	-1.728178183	1.72E-29	2.47E-28	down
ENSG00000180884	ZNF792	protein_coding	2.281993623	1.73E-29	2.49E-28	up
ENSG00000146834	MEPCE	protein_coding	-1.01525132	1.78E-29	2.55E-28	down
ENSG00000263528	IKBKE	protein_coding	-1.904498837	1.87E-29	2.68E-28	down
ENSG00000166313	APBB1	protein_coding	1.674311045	2.13E-29	3.04E-28	up
ENSG00000135537	AFG1L	protein_coding	4.267135664	2.56E-29	3.65E-28	up
ENSG00000086015	MAST2	protein_coding	-1.229899998	2.68E-29	3.82E-28	down
ENSG00000204264	PSMB8	protein_coding	-8.18716363	2.70E-29	3.84E-28	down
ENSG00000124787	RPP40	protein_coding	-1.710992156	2.73E-29	3.87E-28	down
ENSG00000163082	SGPP2	protein_coding	-1.576183288	3.22E-29	4.57E-28	down
ENSG00000165521	EML5	protein_coding	-3.865235535	3.31E-29	4.69E-28	down
ENSG00000164221	CCDC112	protein_coding	-3.063781214	3.43E-29	4.85E-28	down
ENSG00000100479	POLE2	protein_coding	-2.400017146	3.82E-29	5.39E-28	down
ENSG00000133619	KRBA1	protein_coding	-8.17528953	4.21E-29	5.92E-28	down
ENSG00000157625	TAB3	protein_coding	-1.285638213	4.40E-29	6.17E-28	down
ENSG00000048471	SNX29	protein_coding	1.731082764	4.57E-29	6.42E-28	up
ENSG00000092094	OSGEP	protein_coding	1.120784238	5.08E-29	7.11E-28	up
ENSG00000162757	C1orf74	protein_coding	3.773321051	5.35E-29	7.48E-28	up
ENSG00000174136	RGMB	protein_coding	-2.357030233	5.44E-29	7.60E-28	down
ENSG00000105849	TWISTNB	protein_coding	-1.521129518	6.34E-29	8.84E-28	down
ENSG00000163993	S100P	protein_coding	2.000482011	6.56E-29	9.14E-28	up
ENSG00000155893	PXYLP1	protein_coding	-8.163316888	6.57E-29	9.15E-28	down
ENSG00000186767	SPIN4	protein_coding	-1.830541411	7.96E-29	1.10E-27	down
ENSG00000113739	STC2	protein_coding	-1.094903732	7.96E-29	1.10E-27	down
ENSG00000140481	CCDC33	protein_coding	1.756551562	8.07E-29	1.12E-27	up
ENSG00000104369	JPH1	protein_coding	2.831036549	8.50E-29	1.17E-27	up
ENSG00000179085	DPM3	protein_coding	-3.205442364	8.63E-29	1.19E-27	down
ENSG00000130881	LRP3	protein_coding	-1.65111073	9.94E-29	1.37E-27	down
ENSG00000232388	SMIM26	protein_coding	-8.151244056	1.03E-28	1.41E-27	down
ENSG00000114631	PODXL2	protein_coding	-1.416002605	1.03E-28	1.42E-27	down
ENSG00000122687	MRM2	protein_coding	-1.421600894	1.16E-28	1.59E-27	down
ENSG00000136237	RAPGEF5	protein_coding	3.893320827	1.25E-28	1.71E-27	up
ENSG00000074047	GLI2	protein_coding	-1.05251705	1.49E-28	2.02E-27	down
ENSG00000103249	CLCN7	protein_coding	-1.019250054	1.60E-28	2.18E-27	down
ENSG00000166225	FRS2	protein_coding	-1.012152815	1.67E-28	2.26E-27	down
ENSG00000122741	DCAF10	protein_coding	1.222896033	2.03E-28	2.75E-27	up
ENSG00000172977	KAT5	protein_coding	-1.611091174	2.16E-28	2.92E-27	down
ENSG00000014914	MTMR11	protein_coding	2.230198289	2.18E-28	2.95E-27	up
ENSG00000273542	HIST1H4K	protein_coding	-1.940496938	2.20E-28	2.98E-27	down
ENSG00000181751	C5orf30	protein_coding	-1.698430839	2.88E-28	3.88E-27	down
ENSG00000181481	RNF135	protein_coding	1.069647481	3.01E-28	4.06E-27	up
ENSG00000117620	SLC35A3	protein_coding	-1.98512427	3.60E-28	4.82E-27	down
ENSG00000153485	TMEM251	protein_coding	-5.928851634	3.66E-28	4.90E-27	down
ENSG00000112130	RNF8	protein_coding	1.162577927	3.81E-28	5.09E-27	up

ENSG00000119686	FLVCR2	protein_coding	-4.95686601	4.09E-28	5.45E-27	down
ENSG00000104332	SFRP1	protein_coding	-2.569842887	4.37E-28	5.80E-27	down
ENSG00000062725	APPBP2	protein_coding	-1.135473945	4.65E-28	6.18E-27	down
ENSG00000188811	NHLRC3	protein_coding	2.341475264	5.23E-28	6.92E-27	up
ENSG00000138073	PREB	protein_coding	-1.250028521	5.76E-28	7.62E-27	down
ENSG00000156374	PCGF6	protein_coding	-2.467930397	5.93E-28	7.81E-27	down
ENSG00000002549	LAP3	protein_coding	-1.120149029	6.26E-28	8.23E-27	down
ENSG00000164597	COG5	protein_coding	-1.262969138	6.44E-28	8.45E-27	down
ENSG00000273079	GRIN2B	protein_coding	1.913498709	6.69E-28	8.77E-27	up
ENSG00000122707	RECK	protein_coding	-2.220625571	6.95E-28	9.11E-27	down
ENSG00000119231	SENP5	protein_coding	-1.189582543	7.03E-28	9.19E-27	down
ENSG00000205903	ZNF316	protein_coding	-1.739748531	7.66E-28	1.00E-26	down
ENSG00000189057	FAM111B	protein_coding	-2.613978297	8.64E-28	1.13E-26	down
ENSG00000203950	RTL8A	protein_coding	-2.142558328	9.46E-28	1.23E-26	down
ENSG00000130054	FAM155B	protein_coding	-3.691812437	1.09E-27	1.41E-26	down
ENSG00000157191	NECAP2	protein_coding	1.387031858	1.09E-27	1.42E-26	up
ENSG00000140365	COMMD4	protein_coding	-2.057117484	1.64E-27	2.11E-26	down
ENSG00000049883	PTCD2	protein_coding	-1.853070598	1.65E-27	2.12E-26	down
ENSG00000169129	AFAP1L2	protein_coding	1.800906883	1.66E-27	2.13E-26	up
ENSG00000135919	SERPINE2	protein_coding	-1.000245557	1.70E-27	2.19E-26	down
ENSG00000101935	AMMECR1	protein_coding	1.409836311	1.86E-27	2.39E-26	up
ENSG00000144034	TPRKB	protein_coding	-1.646885769	2.16E-27	2.75E-26	down
ENSG00000131094	C1QL1	protein_coding	-8.063781214	2.40E-27	3.05E-26	down
ENSG00000152782	PANK1	protein_coding	-3.338220383	2.46E-27	3.13E-26	down
ENSG00000164144	ARFIP1	protein_coding	-1.706739266	2.47E-27	3.14E-26	down
ENSG00000197959	DNM3	protein_coding	-2.544002882	2.51E-27	3.19E-26	down
ENSG00000144802	NFKBIZ	protein_coding	1.487734803	2.54E-27	3.22E-26	up
ENSG00000089199	CHGB	protein_coding	-3.267653548	2.88E-27	3.64E-26	down
ENSG00000148926	ADM	protein_coding	1.070895061	2.89E-27	3.65E-26	up
ENSG00000001167	NFYA	protein_coding	-1.240234259	3.07E-27	3.88E-26	down
ENSG00000130304	SLC27A1	protein_coding	2.818861835	3.12E-27	3.93E-26	up
ENSG00000149260	CAPN5	protein_coding	1.991501221	3.27E-27	4.11E-26	up
ENSG00000156650	KAT6B	protein_coding	-1.005916719	4.45E-27	5.58E-26	down
ENSG00000176454	LPCAT4	protein_coding	1.197178863	4.45E-27	5.57E-26	up
ENSG00000169756	LIMS1	protein_coding	-1.092163016	4.59E-27	5.74E-26	down
ENSG00000142408	CACNG8	protein_coding	1.167808367	5.12E-27	6.41E-26	up
ENSG00000169696	ASPSCR1	protein_coding	-1.993391886	6.18E-27	7.70E-26	down
ENSG00000100982	PCIF1	protein_coding	1.041689288	6.68E-27	8.31E-26	up
ENSG00000165449	SLC16A9	protein_coding	1.995636996	6.76E-27	8.40E-26	up
ENSG00000275221	HIST1H2AK	protein_coding	-1.307565141	7.27E-27	9.02E-26	down
ENSG00000156103	MMP16	protein_coding	2.573648706	7.80E-27	9.64E-26	up
ENSG00000149792	MRPL49	protein_coding	-1.015972259	7.90E-27	9.76E-26	down
ENSG00000219545	UMAD1	protein_coding	-4.885782912	9.80E-27	1.21E-25	down
ENSG00000119616	FCF1	protein_coding	-1.096249267	1.07E-26	1.32E-25	down
ENSG00000166377	ATP9B	protein_coding	1.24420769	1.12E-26	1.37E-25	up
ENSG00000139835	GRTP1	protein_coding	3.958586599	1.30E-26	1.59E-25	up
ENSG00000145740	SLC30A5	protein_coding	-1.148244641	1.36E-26	1.66E-25	down
ENSG00000168268	NT5DC2	protein_coding	1.117355179	1.36E-26	1.67E-25	up
ENSG00000147316	MCPH1	protein_coding	-1.337780987	1.56E-26	1.90E-25	down
ENSG00000167384	ZNF180	protein_coding	-1.878863498	1.64E-26	2.00E-25	down
ENSG00000187098	MITF	protein_coding	-2.397288828	1.66E-26	2.02E-25	down
ENSG00000179562	GCC1	protein_coding	1.192474659	1.82E-26	2.22E-25	up
ENSG00000182472	CAPN12	protein_coding	1.872856725	1.91E-26	2.32E-25	up
ENSG00000119950	MXI1	protein_coding	1.615249611	2.00E-26	2.42E-25	up

ENSG00000113593	PPWD1	protein_coding	-1.424267979	2.23E-26	2.70E-25	down
ENSG00000197483	ZNF628	protein_coding	-7.997893279	2.34E-26	2.82E-25	down
ENSG00000176896	TCEANC	protein_coding	4.35238295	2.34E-26	2.82E-25	up
ENSG00000130958	SLC35D2	protein_coding	1.243242457	2.34E-26	2.82E-25	up
ENSG00000105520	PLPPR2	protein_coding	-3.997893279	2.41E-26	2.90E-25	down
ENSG00000164187	LMBRD2	protein_coding	1.304288662	2.45E-26	2.94E-25	up
ENSG00000167969	ECI1	protein_coding	1.245599867	2.47E-26	2.97E-25	up
ENSG00000117115	PADI2	protein_coding	1.654829496	2.48E-26	2.98E-25	up
ENSG00000064652	SNX24	protein_coding	-1.997893279	2.56E-26	3.07E-25	down
ENSG00000186318	BACE1	protein_coding	1.270820711	2.89E-26	3.46E-25	up
ENSG00000196182	STK40	protein_coding	-2.710992156	3.49E-26	4.16E-25	down
ENSG00000204389	HSPA1A	protein_coding	-1.141866528	3.60E-26	4.30E-25	down
ENSG00000164086	DUSP7	protein_coding	-7.984346747	3.70E-26	4.41E-25	down
ENSG00000171729	TMEM51	protein_coding	-1.470021765	3.74E-26	4.45E-25	down
ENSG00000110057	UNC93B1	protein_coding	1.121115778	3.76E-26	4.47E-25	up
ENSG00000177103	DSCAML1	protein_coding	7.686088213	4.09E-26	4.85E-25	up
ENSG00000139977	NAA30	protein_coding	-1.169145967	4.10E-26	4.86E-25	down
ENSG00000239264	TXNDC5	protein_coding	-2.47587143	4.23E-26	5.00E-25	down
ENSG00000131732	ZCCHC9	protein_coding	-2.1963053	4.24E-26	5.01E-25	down
ENSG00000182952	HMGN4	protein_coding	1.407118232	4.98E-26	5.87E-25	up
ENSG00000018699	TTC27	protein_coding	-1.270710518	5.22E-26	6.15E-25	down
ENSG00000139618	BRCA2	protein_coding	-1.029332768	5.25E-26	6.19E-25	down
ENSG00000179532	DNHD1	protein_coding	-1.835288593	6.15E-26	7.25E-25	down
ENSG00000147905	ZCCHC7	protein_coding	1.015378323	6.24E-26	7.34E-25	up
ENSG00000167447	SMG8	protein_coding	-1.318155282	6.55E-26	7.69E-25	down
ENSG00000099956	SMARCB1	protein_coding	-1.242686236	7.20E-26	8.45E-25	down
ENSG00000173805	HAP1	protein_coding	-4.549208042	7.67E-26	8.99E-25	down
ENSG00000101052	IFT52	protein_coding	-1.389825418	8.00E-26	9.35E-25	down
ENSG00000168591	TMUB2	protein_coding	-2.490891546	8.14E-26	9.51E-25	down
ENSG00000115947	ORC4	protein_coding	-1.10213955	8.16E-26	9.52E-25	down
ENSG00000158234	FAIM	protein_coding	-1.805764883	8.32E-26	9.70E-25	down
ENSG00000136770	DNAJC1	protein_coding	-1.354829996	9.20E-26	1.07E-24	down
ENSG00000177548	RABEP2	protein_coding	-2.0026697	1.06E-25	1.23E-24	down
ENSG00000157107	FCHO2	protein_coding	1.026436323	1.09E-25	1.26E-24	up
ENSG00000198799	LRIG2	protein_coding	-1.761850028	1.11E-25	1.29E-24	down
ENSG00000151287	TEX30	protein_coding	-2.762317222	1.17E-25	1.35E-24	down
ENSG00000161847	RAVER1	protein_coding	-1.29409671	1.34E-25	1.54E-24	down
ENSG00000204271	SPIN3	protein_coding	-2.089776423	1.36E-25	1.56E-24	down
ENSG00000120334	CENPL	protein_coding	-1.782972107	1.39E-25	1.59E-24	down
ENSG00000146083	RNF44	protein_coding	1.186091943	1.56E-25	1.79E-24	up
ENSG00000213213	CCDC183	protein_coding	2.463021541	1.72E-25	1.96E-24	up
ENSG00000170439	METTL7B	protein_coding	2.463021541	1.72E-25	1.96E-24	up
ENSG00000034677	RNF19A	protein_coding	1.048823061	1.74E-25	1.99E-24	up
ENSG00000119681	LTBP2	protein_coding	1.381388091	1.80E-25	2.06E-24	up
ENSG00000142252	GEMIN7	protein_coding	-1.938529951	1.82E-25	2.07E-24	down
ENSG00000135253	KCP	protein_coding	7.638391471	2.05E-25	2.33E-24	up
ENSG00000042317	SPATA7	protein_coding	2.255219531	2.22E-25	2.51E-24	up
ENSG00000157036	EXOG	protein_coding	-1.946741793	2.23E-25	2.53E-24	down
ENSG00000115363	EVA1A	protein_coding	-7.928851634	2.34E-25	2.65E-24	down
ENSG00000111087	GLI1	protein_coding	1.636020567	2.41E-25	2.73E-24	up
ENSG00000198780	FAM169A	protein_coding	-1.665988661	2.67E-25	3.02E-24	down
ENSG00000173163	COMMD1	protein_coding	-2.093995827	2.74E-25	3.10E-24	down
ENSG00000180992	MRPL14	protein_coding	-1.519460698	2.93E-25	3.30E-24	down
ENSG00000167771	RCOR2	protein_coding	7.626216757	3.06E-25	3.45E-24	up

ENSG00000148908	RGS10	protein_coding	1.141415592	3.25E-25	3.65E-24	up
ENSG00000116641	DOCK7	protein_coding	-1.0516065	3.43E-25	3.86E-24	down
ENSG00000102543	CDADC1	protein_coding	2.941513085	3.52E-25	3.95E-24	up
ENSG00000196776	CD47	protein_coding	-1.064474985	3.64E-25	4.08E-24	down
ENSG00000125398	SOX9	protein_coding	1.004497367	3.69E-25	4.13E-24	up
ENSG00000104936	DMPK	protein_coding	-1.594007651	4.10E-25	4.59E-24	down
ENSG00000162512	SDC3	protein_coding	1.574597536	4.12E-25	4.61E-24	up
ENSG00000127528	KLF2	protein_coding	7.613938427	4.59E-25	5.13E-24	up
ENSG00000145919	BOD1	protein_coding	-1.191134832	4.63E-25	5.16E-24	down
ENSG00000156928	MALSU1	protein_coding	-1.202987033	5.44E-25	6.05E-24	down
ENSG00000146833	TRIM4	protein_coding	1.148901996	6.61E-25	7.33E-24	up
ENSG00000135333	EPHA7	protein_coding	7.601554703	6.89E-25	7.64E-24	up
ENSG00000117009	KMO	protein_coding	6.49846121	7.98E-25	8.82E-24	up
ENSG00000240184	PCDHGC3	protein_coding	-2.256426292	9.01E-25	9.94E-24	down
ENSG00000187522	HSPA14	protein_coding	-1.184440179	9.60E-25	1.06E-23	down
ENSG00000166575	TMEM135	protein_coding	-1.427471834	9.70E-25	1.07E-23	down
ENSG00000056558	TRAF1	protein_coding	-6.764220932	1.07E-24	1.17E-23	down
ENSG00000184203	PPP1R2	protein_coding	1.093078047	1.30E-24	1.42E-23	up
ENSG00000084764	MAPRE3	protein_coding	-1.181081167	1.38E-24	1.51E-23	down
ENSG00000179761	PIPOX	protein_coding	7.576463722	1.56E-24	1.69E-23	up
ENSG00000188959	C9orf152	protein_coding	7.576463722	1.56E-24	1.69E-23	up
ENSG00000204435	CSNK2B	protein_coding	-5.147197223	1.65E-24	1.79E-23	down
ENSG00000183605	SFXN4	protein_coding	-1.500095373	1.66E-24	1.80E-23	down
ENSG00000106031	HOXA13	protein_coding	4.254535627	1.80E-24	1.96E-23	up
ENSG00000196976	LAGE3	protein_coding	-1.726372133	1.94E-24	2.10E-23	down
ENSG00000178966	RMI1	protein_coding	-1.660148478	2.24E-24	2.42E-23	down
ENSG00000108963	DPH1	protein_coding	-1.815853701	2.28E-24	2.46E-23	down
ENSG00000171877	FRMD5	protein_coding	-2.710602185	2.35E-24	2.53E-23	down
ENSG00000137338	PGBD1	protein_coding	-7.856339134	2.39E-24	2.58E-23	down
ENSG00000130193	THEM6	protein_coding	-2.435749992	2.55E-24	2.75E-23	down
ENSG00000120149	MSX2	protein_coding	-2.435749992	2.55E-24	2.75E-23	down
ENSG00000001461	NIPAL3	protein_coding	1.476526239	2.56E-24	2.75E-23	up
ENSG00000165169	DYNLT3	protein_coding	-1.935906392	2.56E-24	2.75E-23	down
ENSG00000100304	TTLL12	protein_coding	-1.157551042	2.82E-24	3.02E-23	down
ENSG00000137764	MAP2K5	protein_coding	1.145090067	2.86E-24	3.06E-23	up
ENSG00000107821	KAZALD1	protein_coding	1.8534914	2.90E-24	3.10E-23	up
ENSG00000139926	FRMD6	protein_coding	1.197164448	3.40E-24	3.63E-23	up
ENSG00000125347	IRF1	protein_coding	-1.032461451	3.44E-24	3.67E-23	down
ENSG00000197362	ZNF786	protein_coding	-1.978892317	3.45E-24	3.68E-23	down
ENSG00000130224	LRCH2	protein_coding	7.55092863	3.52E-24	3.75E-23	up
ENSG00000206190	ATP10A	protein_coding	1.494345101	3.80E-24	4.04E-23	up
ENSG00000142347	MYO1F	protein_coding	4.886531662	3.91E-24	4.16E-23	up
ENSG00000102580	DNAJC3	protein_coding	-1.050354125	3.99E-24	4.24E-23	down
ENSG00000185504	FAAP100	protein_coding	-2.819362487	4.26E-24	4.53E-23	down
ENSG00000138030	KHK	protein_coding	1.155834122	4.31E-24	4.57E-23	up
ENSG00000166261	ZNF202	protein_coding	1.117871542	4.55E-24	4.83E-23	up
ENSG00000128596	CCDC136	protein_coding	-1.90551913	4.63E-24	4.91E-23	down
ENSG00000165821	SALL2	protein_coding	3.016592202	4.65E-24	4.92E-23	up
ENSG00000197299	BLM	protein_coding	-1.20093118	4.77E-24	5.05E-23	down
ENSG00000140104	CLBA1	protein_coding	-3.195025748	4.88E-24	5.17E-23	down
ENSG00000198522	GPN1	protein_coding	-1.492054541	5.10E-24	5.39E-23	down
ENSG00000117461	PIK3R3	protein_coding	-5.682691047	5.17E-24	5.46E-23	down
ENSG00000170293	CMTM8	protein_coding	-6.715857911	5.44E-24	5.74E-23	down
ENSG00000157350	ST3GAL2	protein_coding	-1.191228026	6.34E-24	6.68E-23	down

ENSG00000171560	FGA	protein_coding	2.182146721	6.76E-24	7.12E-23	up
ENSG00000145781	COMMD10	protein_coding	-1.921762209	7.05E-24	7.41E-23	down
ENSG00000165376	CLDN2	protein_coding	1.064273454	7.28E-24	7.64E-23	up
ENSG00000170852	KBTBD2	protein_coding	-1.076558488	7.52E-24	7.89E-23	down
ENSG00000135473	PAN2	protein_coding	1.361703072	8.85E-24	9.25E-23	up
ENSG00000128283	CDC42EP1	protein_coding	-2.239148301	9.35E-24	9.77E-23	down
ENSG00000140983	RHOT2	protein_coding	-1.339639661	1.02E-23	1.06E-22	down
ENSG00000145780	FEM1C	protein_coding	-1.27632585	1.07E-23	1.11E-22	down
ENSG00000121297	TSHZ3	protein_coding	2.26344118	1.12E-23	1.17E-22	up
ENSG00000103274	NUBP1	protein_coding	-1.144284787	1.17E-23	1.22E-22	down
ENSG00000188643	S100A16	protein_coding	-1.276436253	1.40E-23	1.45E-22	down
ENSG00000171067	C11orf24	protein_coding	-1.401299922	1.52E-23	1.58E-22	down
ENSG00000176532	PRR15	protein_coding	-7.795585103	1.57E-23	1.62E-22	down
ENSG00000180537	RNF182	protein_coding	-7.795585103	1.57E-23	1.62E-22	down
ENSG00000104825	NFKBIB	protein_coding	-1.052123009	1.60E-23	1.66E-22	down
ENSG00000159167	STC1	protein_coding	1.818460919	1.64E-23	1.69E-22	up
ENSG00000101333	PLCB4	protein_coding	-1.212924654	1.65E-23	1.70E-22	down
ENSG00000130558	OLFM1	protein_coding	1.291585072	1.72E-23	1.77E-22	up
ENSG00000073169	SELENOO	protein_coding	-2.173964132	1.78E-23	1.83E-22	down
ENSG00000165152	TMEM246	protein_coding	1.033611215	1.94E-23	1.99E-22	up
ENSG00000204681	GABBR1	protein_coding	-2.53799665	2.13E-23	2.18E-22	down
ENSG00000114200	BCHE	protein_coding	2.865564232	2.13E-23	2.18E-22	up
ENSG00000132825	PPP1R3D	protein_coding	6.40178519	2.13E-23	2.18E-22	up
ENSG00000161999	JMJD8	protein_coding	-1.280810451	2.15E-23	2.20E-22	down
ENSG00000118894	EEF2KMT	protein_coding	-1.241159536	2.16E-23	2.21E-22	down
ENSG00000196605	ZNF846	protein_coding	4.471494162	2.18E-23	2.22E-22	up
ENSG00000141759	TXNL4A	protein_coding	-1.18349477	2.26E-23	2.30E-22	down
ENSG00000125352	RNF113A	protein_coding	-2.205200969	2.42E-23	2.46E-22	down
ENSG00000142396	ERVK3-1	protein_coding	-2.283907029	2.44E-23	2.48E-22	down
ENSG00000184470	TXNRD2	protein_coding	-2.704886793	2.60E-23	2.64E-22	down
ENSG00000141994	DUS3L	protein_coding	-1.567424827	2.61E-23	2.65E-22	down
ENSG00000075234	TTC38	protein_coding	-2.303119528	2.78E-23	2.81E-22	down
ENSG00000180773	SLC36A4	protein_coding	-1.260384306	2.87E-23	2.90E-22	down
ENSG00000198947	DMD	protein_coding	2.542315499	2.96E-23	2.99E-22	up
ENSG00000106392	C1GALT1	protein_coding	-1.347191756	3.46E-23	3.49E-22	down
ENSG00000169093	ASMTL	protein_coding	1.350904021	3.52E-23	3.56E-22	up
ENSG00000115421	PAPOLG	protein_coding	-1.147554228	3.81E-23	3.84E-22	down
ENSG00000104154	SLC30A4	protein_coding	-3.385709309	3.91E-23	3.94E-22	down
ENSG00000189144	ZNF573	protein_coding	-7.764220932	4.04E-23	4.06E-22	down
ENSG00000109065	NAT9	protein_coding	-1.776683104	4.20E-23	4.22E-22	down
ENSG00000103148	NPRL3	protein_coding	-1.431200693	4.23E-23	4.25E-22	down
ENSG00000204220	PFDN6	protein_coding	-1.607500733	5.25E-23	5.26E-22	down
ENSG00000126062	TMEM115	protein_coding	-1.782495104	5.30E-23	5.30E-22	down
ENSG00000168286	THAP11	protein_coding	-1.468729896	5.64E-23	5.64E-22	down
ENSG00000129535	NRL	protein_coding	7.457819225	6.29E-23	6.25E-22	up
ENSG00000134668	SPOCD1	protein_coding	7.457819225	6.29E-23	6.25E-22	up
ENSG00000137726	FXYD6	protein_coding	7.457819225	6.29E-23	6.25E-22	up
ENSG00000104856	RELB	protein_coding	-2.028351725	6.31E-23	6.27E-22	down
ENSG00000184304	PRKD1	protein_coding	1.132345552	6.65E-23	6.60E-22	up
ENSG00000134285	FKBP11	protein_coding	-2.51738637	6.80E-23	6.73E-22	down
ENSG00000125848	FLRT3	protein_coding	1.48342181	6.95E-23	6.88E-22	up
ENSG00000166897	ELFN2	protein_coding	1.553242496	7.07E-23	6.99E-22	up
ENSG00000164219	PGGT1B	protein_coding	-1.420312377	7.44E-23	7.34E-22	down
ENSG00000185730	ZNF696	protein_coding	-4.665817228	7.62E-23	7.51E-22	down

ENSG00000133302	SLF1	protein_coding	-1.455548934	7.74E-23	7.62E-22	down
ENSG00000139343	SNRPF	protein_coding	-1.166444925	8.01E-23	7.89E-22	down
ENSG00000198824	CHAMP1	protein_coding	-1.104423199	8.07E-23	7.94E-22	down
ENSG00000068078	FGFR3	protein_coding	4.163112599	8.14E-23	7.99E-22	up
ENSG00000113838	TBCCD1	protein_coding	1.783730834	8.99E-23	8.81E-22	up
ENSG00000239857	GET4	protein_coding	2.559861755	9.06E-23	8.88E-22	up
ENSG00000125247	TMTC4	protein_coding	-2.097728546	9.24E-23	9.05E-22	down
ENSG00000144677	CTDSPL	protein_coding	1.380137405	9.40E-23	9.20E-22	up
ENSG00000109771	LRP2BP	protein_coding	7.444013426	9.52E-23	9.31E-22	up
ENSG00000038532	CLEC16A	protein_coding	-1.0630091	1.02E-22	9.96E-22	down
ENSG00000011198	ABHD5	protein_coding	-7.732159723	1.05E-22	1.02E-21	down
ENSG00000058866	DGKG	protein_coding	-1.415462978	1.13E-22	1.10E-21	down
ENSG00000187123	LYPD6	protein_coding	2.679033456	1.30E-22	1.27E-21	up
ENSG00000135093	USP30	protein_coding	1.901114985	1.31E-22	1.27E-21	up
ENSG00000128059	PPAT	protein_coding	-1.09596162	1.32E-22	1.29E-21	down
ENSG00000114315	HES1	protein_coding	1.203799981	1.37E-22	1.33E-21	up
ENSG00000137273	FOXF2	protein_coding	-1.162417732	1.50E-22	1.46E-21	down
ENSG00000151151	IPMK	protein_coding	1.036981763	1.54E-22	1.49E-21	up
ENSG00000148153	INIP	protein_coding	1.243839044	1.56E-22	1.51E-21	up
ENSG00000100342	APOL1	protein_coding	1.871329255	1.60E-22	1.54E-21	up
ENSG00000170684	ZNF296	protein_coding	-7.715857911	1.68E-22	1.62E-21	down
ENSG00000243147	MRPL33	protein_coding	-1.0952715	1.73E-22	1.67E-21	down
ENSG00000184564	SLITRK6	protein_coding	3.926795532	1.81E-22	1.73E-21	up
ENSG00000141562	NARF	protein_coding	-1.252368076	1.82E-22	1.75E-21	down
ENSG00000141569	TRIM65	protein_coding	-1.537327119	1.84E-22	1.76E-21	down
ENSG00000143772	ITPKB	protein_coding	-5.578354387	1.97E-22	1.89E-21	down
ENSG00000103343	ZNF174	protein_coding	1.701138441	1.99E-22	1.91E-21	up
ENSG00000186951	PPARA	protein_coding	1.470404925	2.03E-22	1.94E-21	up
ENSG00000079257	LXN	protein_coding	1.879839398	2.20E-22	2.10E-21	up
ENSG00000095539	SEMA4G	protein_coding	1.011952334	2.23E-22	2.13E-21	up
ENSG00000132275	RRP8	protein_coding	-1.129734287	2.91E-22	2.77E-21	down
ENSG00000012211	PRICKLE3	protein_coding	-2.004239269	3.00E-22	2.85E-21	down
ENSG00000163053	SLC16A14	protein_coding	7.40178519	3.32E-22	3.15E-21	up
ENSG00000119508	NR4A3	protein_coding	-4.343889134	3.46E-22	3.27E-21	down
ENSG00000086827	ZW10	protein_coding	1.143753248	3.49E-22	3.30E-21	up
ENSG00000163703	CRELD1	protein_coding	1.974899254	3.56E-22	3.36E-21	up
ENSG00000174917	C19orf70	protein_coding	-2.458060153	3.56E-22	3.36E-21	down
ENSG00000178307	TMEM11	protein_coding	-1.1963053	3.67E-22	3.46E-21	down
ENSG00000168282	MGAT2	protein_coding	-1.192795186	4.03E-22	3.80E-21	down
ENSG00000165655	ZNF503	protein_coding	-1.473524414	4.23E-22	3.98E-21	down
ENSG00000197763	TXNRD3	protein_coding	1.473159772	4.25E-22	4.00E-21	up
ENSG00000104643	MTMR9	protein_coding	-1.358649801	4.54E-22	4.26E-21	down
ENSG00000156860	FBRS	protein_coding	-1.120503804	4.86E-22	4.56E-21	down
ENSG00000186897	C1QL4	protein_coding	-2.866479774	5.14E-22	4.82E-21	down
ENSG00000276234	TADA2A	protein_coding	-1.451305221	5.22E-22	4.90E-21	down
ENSG00000169242	EFNA1	protein_coding	1.968204601	5.46E-22	5.11E-21	up
ENSG00000006756	ARSD	protein_coding	-1.395900711	5.87E-22	5.48E-21	down
ENSG00000135842	FAM129A	protein_coding	-1.569584178	6.01E-22	5.60E-21	down
ENSG00000124098	FAM210B	protein_coding	1.382358908	6.04E-22	5.62E-21	up
ENSG00000218891	ZNF579	protein_coding	-1.939236116	6.30E-22	5.86E-21	down
ENSG00000120833	SOCS2	protein_coding	2.05135762	6.44E-22	5.99E-21	up
ENSG00000162959	MEMO1	protein_coding	-7.665817228	7.10E-22	6.59E-21	down
ENSG00000053108	FSTL4	protein_coding	-7.665817228	7.10E-22	6.59E-21	down
ENSG00000180354	MTURN	protein_coding	3.226656594	7.22E-22	6.69E-21	up

ENSG00000171823	FBXL14	protein_coding	-2.167889618	7.32E-22	6.78E-21	down
ENSG00000090097	PCBP4	protein_coding	-6.56020704	7.63E-22	7.06E-21	down
ENSG00000138942	RNF185	protein_coding	-3.764220932	7.82E-22	7.22E-21	down
ENSG00000141873	SLC39A3	protein_coding	-3.764220932	7.82E-22	7.22E-21	down
ENSG00000099203	TMED1	protein_coding	-1.446087512	7.96E-22	7.35E-21	down
ENSG00000196659	TTC30B	protein_coding	1.336334579	8.64E-22	7.98E-21	up
ENSG00000107537	PHYH	protein_coding	-1.105804429	9.39E-22	8.66E-21	down
ENSG00000164306	PRIMPOL	protein_coding	-2.758926633	9.74E-22	8.96E-21	down
ENSG00000021574	SPAST	protein_coding	-1.11353425	9.74E-22	8.95E-21	down
ENSG00000197785	ATAD3A	protein_coding	-1.041220412	1.04E-21	9.55E-21	down
ENSG00000189339	SLC35E2B	protein_coding	1.075226312	1.08E-21	9.88E-21	up
ENSG00000103381	CPPED1	protein_coding	-1.038960042	1.11E-21	1.02E-20	down
ENSG00000144591	GMPPA	protein_coding	1.111588716	1.15E-21	1.05E-20	up
ENSG00000204967	PCDHA4	protein_coding	-2.218951587	1.16E-21	1.06E-20	down
ENSG00000183873	SCN5A	protein_coding	7.358283552	1.17E-21	1.07E-20	up
ENSG00000154743	TSEN2	protein_coding	-1.297195898	1.18E-21	1.08E-20	down
ENSG00000197565	COL4A6	protein_coding	-2.055168084	1.30E-21	1.18E-20	down
ENSG00000153294	ADGRF4	protein_coding	-6.541828511	1.33E-21	1.21E-20	down
ENSG00000164970	FAM219A	protein_coding	-1.517190524	1.37E-21	1.24E-20	down
ENSG00000177380	PPFIA3	protein_coding	1.743573708	1.50E-21	1.36E-20	up
ENSG00000115594	IL1R1	protein_coding	1.13934118	1.51E-21	1.36E-20	up
ENSG00000173275	ZNF449	protein_coding	1.863312952	1.61E-21	1.45E-20	up
ENSG00000158805	ZNF276	protein_coding	1.156512741	1.81E-21	1.63E-20	up
ENSG00000021826	CPS1	protein_coding	-7.631465724	1.86E-21	1.68E-20	down
ENSG00000102078	SLC25A14	protein_coding	2.541529932	1.96E-21	1.76E-20	up
ENSG00000213762	ZNF134	protein_coding	-1.401084535	2.25E-21	2.02E-20	down
ENSG00000173898	SPTBN2	protein_coding	-1.906239937	2.33E-21	2.09E-20	down
ENSG00000008324	SS18L2	protein_coding	-3.489087049	2.36E-21	2.11E-20	down
ENSG00000132740	IGHMBP2	protein_coding	-2.34131519	2.55E-21	2.28E-20	down
ENSG00000167216	KATNAL2	protein_coding	2.391862144	2.60E-21	2.33E-20	up
ENSG00000185753	CXorf38	protein_coding	-2.109877629	2.63E-21	2.35E-20	down
ENSG00000078596	ITM2A	protein_coding	7.328536208	2.72E-21	2.42E-20	up
ENSG00000198517	MAFK	protein_coding	-1.219693741	2.80E-21	2.50E-20	down
ENSG00000084636	COL16A1	protein_coding	-3.732159723	2.86E-21	2.55E-20	down
ENSG00000186638	KIF24	protein_coding	1.002578237	2.86E-21	2.55E-20	up
ENSG00000102984	ZNF821	protein_coding	4.34348655	2.88E-21	2.56E-20	up
ENSG00000178538	CA8	protein_coding	-1.495892227	2.97E-21	2.64E-20	down
ENSG00000182544	MFSD5	protein_coding	-1.382874628	3.44E-21	3.04E-20	down
ENSG00000197852	FAM212B	protein_coding	3.677685772	3.44E-21	3.04E-20	up
ENSG00000169439	SDC2	protein_coding	2.294983649	3.59E-21	3.17E-20	up
ENSG00000142619	PADI3	protein_coding	2.058084331	4.24E-21	3.74E-20	up
ENSG00000167272	POP5	protein_coding	-1.464153326	4.39E-21	3.87E-20	down
ENSG00000183778	B3GALT5	protein_coding	-7.596276295	4.92E-21	4.32E-20	down
ENSG00000108379	WNT3	protein_coding	-7.596276295	4.92E-21	4.32E-20	down
ENSG00000211445	GPX3	protein_coding	-7.596276295	4.92E-21	4.32E-20	down
ENSG00000081791	KIAA0141	protein_coding	-1.215321089	5.33E-21	4.67E-20	down
ENSG00000141447	OSBPL1A	protein_coding	1.111795455	6.11E-21	5.34E-20	up
ENSG00000125454	SLC25A19	protein_coding	-1.696998884	6.19E-21	5.40E-20	down
ENSG00000178700	DHFR2	protein_coding	1.661735416	6.41E-21	5.59E-20	up
ENSG00000115825	PRKD3	protein_coding	-1.05219324	6.70E-21	5.84E-20	down
ENSG00000176978	DPP7	protein_coding	1.125821272	7.05E-21	6.14E-20	up
ENSG00000077152	UBE2T	protein_coding	-1.049754329	7.16E-21	6.23E-20	down
ENSG00000149636	DSN1	protein_coding	-1.161851479	7.32E-21	6.36E-20	down
ENSG00000198026	ZNF335	protein_coding	-1.012292092	7.70E-21	6.69E-20	down

ENSG00000105793	GTPBP10	protein_coding	-1.796994674	7.73E-21	6.71E-20	down
ENSG00000176399	DMRTA1	protein_coding	5.219307139	7.80E-21	6.77E-20	up
ENSG00000165997	ARL5B	protein_coding	-1.06293282	8.13E-21	7.05E-20	down
ENSG00000283526	PRRT1B	protein_coding	3.831036549	8.15E-21	7.05E-20	up
ENSG00000142655	PEX14	protein_coding	-2.163316888	8.74E-21	7.56E-20	down
ENSG00000183323	CCDC125	protein_coding	-1.936167018	8.93E-21	7.71E-20	down
ENSG00000241635	UGT1A1	protein_coding	3.267135664	9.32E-21	8.05E-20	up
ENSG00000165490	DDIAS	protein_coding	-1.462010426	1.14E-20	9.81E-20	down
ENSG00000135643	KCNMB4	protein_coding	-6.465879658	1.24E-20	1.06E-19	down
ENSG00000165675	ENOX2	protein_coding	-1.712308844	1.28E-20	1.10E-19	down
ENSG00000137727	ARHGAP20	protein_coding	-7.56020704	1.30E-20	1.12E-19	down
ENSG00000169031	COL4A3	protein_coding	2.328536208	1.41E-20	1.20E-19	up
ENSG00000148090	AUH	protein_coding	1.422556118	1.42E-20	1.22E-19	up
ENSG00000076053	RBM7	protein_coding	1.495387066	1.70E-20	1.45E-19	up
ENSG00000101940	WDR13	protein_coding	-1.530459697	1.72E-20	1.46E-19	down
ENSG00000180694	TMEM64	protein_coding	-1.077102593	1.89E-20	1.61E-19	down
ENSG00000119938	PPP1R3C	protein_coding	1.664963873	1.91E-20	1.63E-19	up
ENSG00000101928	MOSPD1	protein_coding	-1.369320349	2.00E-20	1.70E-19	down
ENSG00000213096	ZNF254	protein_coding	-1.521887436	2.05E-20	1.74E-19	down
ENSG00000136870	ZNF189	protein_coding	1.320362277	2.10E-20	1.78E-19	up
ENSG00000187556	NANOS3	protein_coding	7.251368348	2.27E-20	1.92E-19	up
ENSG00000108001	EBF3	protein_coding	7.251368348	2.27E-20	1.92E-19	up
ENSG00000164615	CAMLG	protein_coding	-2.437756057	2.36E-20	1.99E-19	down
ENSG00000130511	SSBP4	protein_coding	1.470889132	2.37E-20	2.00E-19	up
ENSG00000135439	AGAP2	protein_coding	-4.504353806	2.54E-20	2.14E-19	down
ENSG00000162065	TBC1D24	protein_coding	1.7691088	2.57E-20	2.17E-19	up
ENSG00000116885	OSCP1	protein_coding	-5.426351294	2.67E-20	2.25E-19	down
ENSG00000144659	SLC25A38	protein_coding	-1.270410522	2.71E-20	2.28E-19	down
ENSG00000158458	NRG2	protein_coding	-3.07842799	2.85E-20	2.39E-19	down
ENSG00000185085	INTS5	protein_coding	1.09011127	2.97E-20	2.49E-19	up
ENSG00000196814	MVB12B	protein_coding	-1.648743715	3.02E-20	2.53E-19	down
ENSG00000234602	MCIDAS	protein_coding	7.235426804	3.48E-20	2.90E-19	up
ENSG00000159674	SPON2	protein_coding	7.235426804	3.48E-20	2.90E-19	up
ENSG00000110237	ARHGEF17	protein_coding	1.059660924	3.63E-20	3.03E-19	up
ENSG00000136826	KLF4	protein_coding	-3.32055663	3.66E-20	3.05E-19	down
ENSG00000174106	LEMD3	protein_coding	-1.340084221	3.86E-20	3.21E-19	down
ENSG00000121067	SPOP	protein_coding	-1.004118293	3.94E-20	3.28E-19	down
ENSG00000136878	USP20	protein_coding	1.826035868	4.03E-20	3.35E-19	up
ENSG00000166435	XRRA1	protein_coding	2.123716417	4.08E-20	3.38E-19	up
ENSG00000184979	USP18	protein_coding	-4.86128835	4.16E-20	3.45E-19	down
ENSG00000177888	ZBTB41	protein_coding	-1.132851607	4.26E-20	3.53E-19	down
ENSG00000154832	CXXC1	protein_coding	-1.413967569	4.33E-20	3.59E-19	down
ENSG00000183401	CCDC159	protein_coding	4.267135664	4.38E-20	3.62E-19	up
ENSG00000035115	SH3YL1	protein_coding	1.725116803	4.57E-20	3.78E-19	up
ENSG00000007376	RPUSD1	protein_coding	-1.950323165	4.58E-20	3.78E-19	down
ENSG00000256087	ZNF432	protein_coding	2.098106468	4.86E-20	4.01E-19	up
ENSG00000168116	KIAA1586	protein_coding	2.098106468	4.86E-20	4.01E-19	up
ENSG00000087995	METTL2A	protein_coding	-1.209261184	5.19E-20	4.27E-19	down
ENSG00000132570	PCBD2	protein_coding	-2.09292756	5.33E-20	4.38E-19	down
ENSG00000171033	PKIA	protein_coding	3.991501221	5.63E-20	4.62E-19	up
ENSG00000111554	MDM1	protein_coding	-1.578354387	6.06E-20	4.97E-19	down
ENSG00000107614	TRDMT1	protein_coding	1.875485383	6.46E-20	5.29E-19	up
ENSG00000162236	STX5	protein_coding	1.438034573	6.55E-20	5.36E-19	up
ENSG00000149564	ESAM	protein_coding	2.04982018	6.75E-20	5.53E-19	up

ENSG00000140092	FBLN5	protein_coding	2.754800963	7.60E-20	6.21E-19	up
ENSG00000069188	SDK2	protein_coding	1.226742207	7.64E-20	6.24E-19	up
ENSG00000103932	RPAP1	protein_coding	-1.650504173	7.88E-20	6.42E-19	down
ENSG00000188641	DPYD	protein_coding	2.55092863	8.02E-20	6.54E-19	up
ENSG00000106123	EPHB6	protein_coding	7.203005326	8.20E-20	6.67E-19	up
ENSG00000178927	C17orf62	protein_coding	-1.008845539	8.91E-20	7.23E-19	down
ENSG00000152684	PELO	protein_coding	-1.265949066	9.02E-20	7.31E-19	down
ENSG00000185000	DGAT1	protein_coding	-7.485244983	9.32E-20	7.53E-19	down
ENSG00000173581	CCDC106	protein_coding	-2.324597795	9.39E-20	7.59E-19	down
ENSG00000257335	MGAM	protein_coding	1.7126797	1.01E-19	8.14E-19	up
ENSG00000130584	ZBTB46	protein_coding	-2.349535697	1.06E-19	8.50E-19	down
ENSG00000086300	SNX10	protein_coding	1.497817293	1.06E-19	8.57E-19	up
ENSG00000185347	TEDC1	protein_coding	-1.786941009	1.08E-19	8.66E-19	down
ENSG00000132773	TOE1	protein_coding	-1.963644543	1.14E-19	9.19E-19	down
ENSG00000108852	MPP2	protein_coding	-2.855463978	1.18E-19	9.44E-19	down
ENSG00000178935	ZNF552	protein_coding	-6.385709309	1.18E-19	9.46E-19	down
ENSG00000133687	TMTC1	protein_coding	6.118613139	1.19E-19	9.57E-19	up
ENSG00000155256	ZFYVE27	protein_coding	-1.216533565	1.26E-19	1.01E-18	down
ENSG00000107077	KDM4C	protein_coding	1.165665965	1.31E-19	1.05E-18	up
ENSG00000197467	COL13A1	protein_coding	-7.465879658	1.53E-19	1.22E-18	down
ENSG00000166321	NUDT13	protein_coding	2.144453144	1.70E-19	1.35E-18	up
ENSG00000213066	FGFR1OP	protein_coding	-5.364950749	1.71E-19	1.36E-18	down
ENSG00000198551	ZNF627	protein_coding	-5.364950749	1.71E-19	1.36E-18	down
ENSG00000188070	C11orf95	protein_coding	-1.757620435	1.77E-19	1.41E-18	down
ENSG00000107443	CCNJ	protein_coding	-1.620998725	1.89E-19	1.50E-18	down
ENSG00000175895	PLEKHF2	protein_coding	-2.964245541	1.99E-19	1.58E-18	down
ENSG00000037757	MRI1	protein_coding	-1.455070111	2.01E-19	1.59E-18	down
ENSG00000114423	CBLB	protein_coding	1.600833536	2.07E-19	1.63E-18	up
ENSG00000125744	RTN2	protein_coding	2.732258394	2.08E-19	1.65E-18	up
ENSG00000266714	MYO15B	protein_coding	-1.669024746	2.13E-19	1.68E-18	down
ENSG00000126756	UXT	protein_coding	-1.028581315	2.22E-19	1.76E-18	down
ENSG00000240857	RDH14	protein_coding	-1.558545906	2.31E-19	1.82E-18	down
ENSG00000105671	DDX49	protein_coding	-1.246965963	2.40E-19	1.89E-18	down
ENSG00000232112	TMA7	protein_coding	-1.338575334	2.93E-19	2.30E-18	down
ENSG00000162813	BPNT1	protein_coding	1.0848239	3.03E-19	2.38E-18	up
ENSG00000128694	OSGEPL1	protein_coding	-2.025813364	3.04E-19	2.39E-18	down
ENSG00000006459	KDM7A	protein_coding	1.482276442	3.14E-19	2.46E-18	up
ENSG00000164045	CDC25A	protein_coding	-1.052892898	3.15E-19	2.47E-18	down
ENSG00000158092	NCK1	protein_coding	1.220516338	3.18E-19	2.49E-18	up
ENSG00000116455	WDR77	protein_coding	-2.224245887	3.32E-19	2.60E-18	down
ENSG00000166188	ZNF319	protein_coding	-1.735594129	3.63E-19	2.83E-18	down
ENSG00000090776	EFNB1	protein_coding	-6.343889134	3.67E-19	2.86E-18	down
ENSG00000108511	HOXB6	protein_coding	-1.287014612	3.86E-19	3.01E-18	down
ENSG00000186889	TMEM17	protein_coding	-2.773001818	4.03E-19	3.14E-18	down
ENSG00000122574	WIPF3	protein_coding	-7.426351294	4.13E-19	3.21E-18	down
ENSG00000172661	WASHC2C	protein_coding	-1.153773115	4.25E-19	3.30E-18	down
ENSG00000196557	CACNA1H	protein_coding	1.219672733	4.61E-19	3.56E-18	up
ENSG00000007237	GAS7	protein_coding	7.13589113	4.61E-19	3.56E-18	up
ENSG00000146205	ANO7	protein_coding	7.13589113	4.61E-19	3.56E-18	up
ENSG00000146233	CYP39A1	protein_coding	7.13589113	4.61E-19	3.56E-18	up
ENSG00000198719	DLL1	protein_coding	7.13589113	4.61E-19	3.56E-18	up
ENSG00000137814	HAUS2	protein_coding	-1.554949783	4.66E-19	3.59E-18	down
ENSG00000135002	RFK	protein_coding	-1.056401684	4.69E-19	3.62E-18	down
ENSG00000141012	GALNS	protein_coding	-1.046579124	4.69E-19	3.62E-18	down

ENSG00000122033	MTIF3	protein_coding	1.141166073	4.75E-19	3.66E-18	up
ENSG00000079482	OPHN1	protein_coding	1.687479146	4.87E-19	3.74E-18	up
ENSG00000121864	ZNF639	protein_coding	1.392230884	4.91E-19	3.77E-18	up
ENSG00000071575	TRIB2	protein_coding	-2.438629624	4.96E-19	3.81E-18	down
ENSG00000154146	NRGN	protein_coding	-2.290914669	5.02E-19	3.85E-18	down
ENSG00000157538	DSCR3	protein_coding	-1.637855399	5.16E-19	3.96E-18	down
ENSG00000079931	MOXD1	protein_coding	6.065501803	5.17E-19	3.96E-18	up
ENSG00000152763	WDR78	protein_coding	3.40178519	5.25E-19	4.01E-18	up
ENSG00000085433	WDR47	protein_coding	1.206857652	5.25E-19	4.02E-18	up
ENSG00000172264	MACROD2	protein_coding	4.55092863	5.35E-19	4.09E-18	up
ENSG00000188315	C3orf62	protein_coding	-4.779988248	5.39E-19	4.11E-18	down
ENSG00000160753	RUSC1	protein_coding	-1.816297812	5.51E-19	4.20E-18	down
ENSG00000005059	MCUB	protein_coding	1.42065441	5.58E-19	4.26E-18	up
ENSG00000036054	TBC1D23	protein_coding	1.121400128	5.59E-19	4.26E-18	up
ENSG00000122390	NAA60	protein_coding	-1.373783243	5.87E-19	4.47E-18	down
ENSG00000147799	ARHGAP39	protein_coding	-1.558081992	6.04E-19	4.59E-18	down
ENSG00000167165	UGT1A6	protein_coding	4.186517204	6.68E-19	5.07E-18	up
ENSG00000170681	CAVIN4	protein_coding	2.901114985	6.79E-19	5.14E-18	up
ENSG00000131845	ZNF304	protein_coding	-7.406173412	6.80E-19	5.15E-18	down
ENSG00000080224	EPHA6	protein_coding	7.118613139	7.12E-19	5.38E-18	up
ENSG00000012124	CD22	protein_coding	-2.758926633	7.38E-19	5.58E-18	down
ENSG00000198774	RASSF9	protein_coding	2.379883088	8.68E-19	6.53E-18	up
ENSG00000105053	VRK3	protein_coding	-1.100410136	9.05E-19	6.80E-18	down
ENSG00000137075	RNF38	protein_coding	-1.151988882	9.58E-19	7.19E-18	down
ENSG00000185414	MRPL30	protein_coding	-1.496253307	1.03E-18	7.72E-18	down
ENSG00000241484	ARHGAP8	protein_coding	7.101125712	1.10E-18	8.22E-18	up
ENSG00000167487	KLHL26	protein_coding	-7.385709309	1.12E-18	8.39E-18	down
ENSG00000275004	ZNF280B	protein_coding	-2.988230181	1.19E-18	8.90E-18	down
ENSG00000214046	SMIM7	protein_coding	1.059146308	1.21E-18	8.99E-18	up
ENSG00000166669	ATF7IP2	protein_coding	1.435056532	1.22E-18	9.11E-18	up
ENSG00000141738	GRB7	protein_coding	-4.385709309	1.25E-18	9.27E-18	down
ENSG00000112578	BYSL	protein_coding	-1.151380268	1.26E-18	9.34E-18	down
ENSG00000171488	LRRC8C	protein_coding	-2.355961966	1.26E-18	9.39E-18	down
ENSG00000163026	WDCP	protein_coding	-1.335652983	1.29E-18	9.60E-18	down
ENSG00000130940	CASZ1	protein_coding	3.521181286	1.32E-18	9.76E-18	up
ENSG00000125967	NECAB3	protein_coding	1.504693164	1.39E-18	1.03E-17	up
ENSG00000173465	SSSCA1	protein_coding	-2.384181839	1.41E-18	1.04E-17	down
ENSG00000116874	WARS2	protein_coding	-2.384181839	1.41E-18	1.04E-17	down
ENSG00000116670	MAD2L2	protein_coding	-1.227667299	1.42E-18	1.05E-17	down
ENSG00000020129	NCDN	protein_coding	-1.674037735	1.45E-18	1.07E-17	down
ENSG00000007255	TRAPPC6A	protein_coding	-4.104423199	1.63E-18	1.21E-17	down
ENSG00000140406	TLNRD1	protein_coding	-1.57129963	1.70E-18	1.25E-17	down
ENSG00000188177	ZC3H6	protein_coding	2.806583505	1.81E-18	1.33E-17	up
ENSG00000169016	E2F6	protein_coding	-1.180594879	1.83E-18	1.34E-17	down
ENSG00000107175	CREB3	protein_coding	-1.123159761	1.86E-18	1.37E-17	down
ENSG00000140057	AK7	protein_coding	1.879551377	1.88E-18	1.38E-17	up
ENSG00000128408	RIBC2	protein_coding	2.008276934	1.93E-18	1.41E-17	up
ENSG00000156050	FAM161B	protein_coding	-4.737552982	1.95E-18	1.43E-17	down
ENSG00000138613	APH1B	protein_coding	1.164902953	2.10E-18	1.54E-17	up
ENSG00000166780	C16orf45	protein_coding	-1.063781214	2.15E-18	1.58E-17	down
ENSG00000109107	ALDOC	protein_coding	1.985331454	2.21E-18	1.62E-17	up
ENSG00000169570	DTWD2	protein_coding	-4.364950749	2.39E-18	1.74E-17	down
ENSG00000137310	TCF19	protein_coding	-1.305894969	2.40E-18	1.75E-17	down
ENSG00000099624	ATP5D	protein_coding	-1.016533966	2.54E-18	1.85E-17	down

ENSG00000143502	SUSD4	protein_coding	7.065501803	2.63E-18	1.92E-17	up
ENSG00000154040	CABYR	protein_coding	-1.272136375	2.79E-18	2.03E-17	down
ENSG00000196110	ZNF699	protein_coding	-7.343889134	3.07E-18	2.22E-17	down
ENSG00000266338	NBPF15	protein_coding	1.035883036	3.24E-18	2.34E-17	up
ENSG00000154803	FLCN	protein_coding	-1.251337416	3.29E-18	2.38E-17	down
ENSG00000197951	ZNF71	protein_coding	-1.530601256	3.53E-18	2.54E-17	down
ENSG00000181404	WASHC1	protein_coding	-6.256426292	3.61E-18	2.60E-17	down
ENSG00000189283	FHIT	protein_coding	-4.715857911	3.71E-18	2.66E-17	down
ENSG00000108799	EZH1	protein_coding	1.084383268	3.89E-18	2.79E-17	up
ENSG00000198556	ZNF789	protein_coding	-2.33171442	3.90E-18	2.79E-17	down
ENSG00000124466	LYPD3	protein_coding	7.047354456	4.08E-18	2.92E-17	up
ENSG00000111328	CDK2AP1	protein_coding	-1.005464719	4.19E-18	2.99E-17	down
ENSG00000081059	TCF7	protein_coding	-1.628844158	4.34E-18	3.10E-17	down
ENSG00000175183	CSRP2	protein_coding	1.864203413	4.34E-18	3.10E-17	up
ENSG00000198108	CHSY3	protein_coding	5.010360248	4.40E-18	3.14E-17	up
ENSG00000140451	PIF1	protein_coding	1.396378854	4.59E-18	3.27E-17	up
ENSG00000095637	SORBS1	protein_coding	4.480539302	4.69E-18	3.34E-17	up
ENSG00000114904	NEK4	protein_coding	-1.000587388	4.70E-18	3.35E-17	down
ENSG00000173653	RCE1	protein_coding	-1.990032874	4.96E-18	3.52E-17	down
ENSG00000103489	XYLT1	protein_coding	-1.990032874	4.96E-18	3.52E-17	down
ENSG00000177943	MAMDC4	protein_coding	2.781708837	5.00E-18	3.55E-17	up
ENSG00000175344	CHRNA7	protein_coding	-7.322515483	5.09E-18	3.60E-17	down
ENSG00000216937	CCDC7	protein_coding	1.224005625	5.09E-18	3.61E-17	up
ENSG00000006015	REX1BD	protein_coding	-2.102255362	5.29E-18	3.74E-17	down
ENSG00000147100	SLC16A2	protein_coding	-1.266995534	5.38E-18	3.81E-17	down
ENSG00000132716	DCAF8	protein_coding	-1.031866034	5.80E-18	4.10E-17	down
ENSG00000154429	CCSAP	protein_coding	-1.390282039	5.82E-18	4.11E-17	down
ENSG00000148450	MSRB2	protein_coding	1.374339898	5.90E-18	4.16E-17	up
ENSG00000154822	PLCL2	protein_coding	-1.707637404	6.29E-18	4.43E-17	down
ENSG00000074964	ARHGEF10L	protein_coding	1.04919999	6.70E-18	4.71E-17	up
ENSG00000254004	ZNF260	protein_coding	-1.109584904	6.95E-18	4.87E-17	down
ENSG00000074370	ATP2A3	protein_coding	1.983039642	7.08E-18	4.96E-17	up
ENSG00000106003	LFNG	protein_coding	2.047907743	7.12E-18	4.99E-17	up
ENSG00000165443	PHYHIPL	protein_coding	-5.233706216	7.22E-18	5.06E-17	down
ENSG00000136169	SETDB2	protein_coding	-1.932536681	7.55E-18	5.28E-17	down
ENSG00000102048	ASB9	protein_coding	-1.061778861	7.63E-18	5.34E-17	down
ENSG00000196458	ZNF605	protein_coding	-1.361059833	7.67E-18	5.36E-17	down
ENSG00000188878	FBF1	protein_coding	-3.841388793	7.93E-18	5.54E-17	down
ENSG00000163040	CCDC74A	protein_coding	-2.811015144	8.19E-18	5.70E-17	down
ENSG00000139737	SLAIN1	protein_coding	2.7691088	8.30E-18	5.77E-17	up
ENSG00000170915	PAQR8	protein_coding	3.203005326	8.40E-18	5.84E-17	up
ENSG00000178726	THBD	protein_coding	-7.300820412	8.44E-18	5.86E-17	down
ENSG00000120057	SFRP5	protein_coding	-7.300820412	8.44E-18	5.86E-17	down
ENSG00000243678	NME2	protein_coding	-7.300820412	8.44E-18	5.86E-17	down
ENSG00000169548	ZNF280A	protein_coding	-7.300820412	8.44E-18	5.86E-17	down
ENSG00000171723	GPHN	protein_coding	-1.231159534	8.65E-18	6.00E-17	down
ENSG00000126768	TIMM17B	protein_coding	-2.157648138	8.73E-18	6.05E-17	down
ENSG00000164684	ZNF704	protein_coding	1.117698014	8.94E-18	6.19E-17	up
ENSG00000185100	ADSSL1	protein_coding	2.328536208	9.22E-18	6.37E-17	up
ENSG00000157764	BRAF	protein_coding	-1.010849084	9.47E-18	6.54E-17	down
ENSG00000115718	PROC	protein_coding	3.634344638	9.50E-18	6.56E-17	up
ENSG00000007384	RHBDF1	protein_coding	-2.406985969	9.54E-18	6.58E-17	down
ENSG00000116128	BCL9	protein_coding	-1.426351294	9.56E-18	6.59E-17	down
ENSG00000172530	BANP	protein_coding	1.107694239	9.60E-18	6.62E-17	up

ENSG00000129932	DOHH	protein_coding	-3.658524736	1.02E-17	7.03E-17	down
ENSG00000057704	TMCC3	protein_coding	4.101125712	1.02E-17	7.04E-17	up
ENSG00000221886	ZBED8	protein_coding	1.758220484	1.04E-17	7.13E-17	up
ENSG00000024526	DEPDC1	protein_coding	-1.034033871	1.13E-17	7.75E-17	down
ENSG00000197223	C1D	protein_coding	-4.043022654	1.16E-17	7.91E-17	down
ENSG00000152953	STK32B	protein_coding	-1.647747723	1.18E-17	8.09E-17	down
ENSG00000197037	ZSCAN25	protein_coding	-2.198940798	1.19E-17	8.15E-17	down
ENSG00000167566	NCKAP5L	protein_coding	1.126577379	1.19E-17	8.15E-17	up
ENSG00000100221	JOSD1	protein_coding	-1.051841601	1.22E-17	8.33E-17	down
ENSG00000204175	GPRIN2	protein_coding	1.544854115	1.25E-17	8.52E-17	up
ENSG00000106004	HOXA5	protein_coding	-3.504353806	1.28E-17	8.69E-17	down
ENSG00000214087	ARL16	protein_coding	-1.393929816	1.31E-17	8.89E-17	down
ENSG00000120616	EPC1	protein_coding	-1.004590093	1.39E-17	9.41E-17	down
ENSG00000138641	HERC3	protein_coding	-7.278794105	1.40E-17	9.52E-17	down
ENSG00000183172	SMDT1	protein_coding	-7.278794105	1.40E-17	9.52E-17	down
ENSG00000236782	AL391650.1	protein_coding	-7.278794105	1.40E-17	9.52E-17	down
ENSG00000179841	AKAP5	protein_coding	-7.278794105	1.40E-17	9.52E-17	down
ENSG00000102967	DHODH	protein_coding	-1.076344676	1.46E-17	9.89E-17	down
ENSG00000089847	ANKRD24	protein_coding	6.991501221	1.53E-17	1.03E-16	up
ENSG00000170703	TTLL6	protein_coding	5.933398267	1.63E-17	1.10E-16	up
ENSG00000156687	UNC5D	protein_coding	-1.733239989	1.65E-17	1.11E-16	down
ENSG00000132950	ZMYM5	protein_coding	1.278705052	1.67E-17	1.12E-16	up
ENSG00000115042	FAHD2A	protein_coding	-1.04783967	1.70E-17	1.15E-16	down
ENSG00000177981	ASB8	protein_coding	1.370205388	1.75E-17	1.18E-16	up
ENSG00000156136	DCK	protein_coding	-1.290373624	1.75E-17	1.18E-16	down
ENSG00000221988	PPT2	protein_coding	4.083423711	1.77E-17	1.19E-16	up
ENSG00000174652	ZNF266	protein_coding	2.626216757	1.87E-17	1.26E-16	up
ENSG00000099998	GGT5	protein_coding	1.86983748	1.91E-17	1.28E-16	up
ENSG00000147535	PLPP5	protein_coding	-1.841388793	1.91E-17	1.28E-16	down
ENSG00000067992	PDK3	protein_coding	-1.284549893	1.97E-17	1.32E-16	down
ENSG00000119242	CCDC92	protein_coding	1.019547169	1.97E-17	1.32E-16	up
ENSG00000096092	TMEM14A	protein_coding	-1.813908057	1.99E-17	1.33E-16	down
ENSG00000066735	KIF26A	protein_coding	1.851102172	2.09E-17	1.40E-16	up
ENSG00000215252	GOLGA8B	protein_coding	1.13206942	2.13E-17	1.42E-16	up
ENSG00000185070	FLRT2	protein_coding	3.813963036	2.27E-17	1.51E-16	up
ENSG00000131196	NFATC1	protein_coding	1.134449156	2.29E-17	1.53E-16	up
ENSG00000168306	ACOX2	protein_coding	-7.256426292	2.34E-17	1.56E-16	down
ENSG00000105499	PLA2G4C	protein_coding	-7.256426292	2.34E-17	1.56E-16	down
ENSG00000073670	ADAM11	protein_coding	-7.256426292	2.34E-17	1.56E-16	down
ENSG00000244274	DBNDD2	protein_coding	6.972392398	2.38E-17	1.59E-16	up
ENSG00000205754	SLCO1B7	protein_coding	6.972392398	2.38E-17	1.59E-16	up
ENSG00000011009	LYPLA2	protein_coding	-1.423076162	2.48E-17	1.65E-16	down
ENSG00000147471	PLPBP	protein_coding	-1.095732239	2.56E-17	1.70E-16	down
ENSG00000119328	FAM206A	protein_coding	-2.35040244	2.67E-17	1.77E-16	down
ENSG00000126821	SGPP1	protein_coding	-1.105308559	2.67E-17	1.77E-16	down
ENSG00000187994	RINL	protein_coding	5.913498709	2.69E-17	1.78E-16	up
ENSG00000171262	FAM98B	protein_coding	-1.003524992	2.87E-17	1.90E-16	down
ENSG00000091157	WDR7	protein_coding	-1.136317626	2.92E-17	1.94E-16	down
ENSG00000214029	ZNF891	protein_coding	-1.631717613	2.97E-17	1.97E-16	down
ENSG00000104774	MAN2B1	protein_coding	1.195641938	3.09E-17	2.04E-16	up
ENSG00000125089	SH3TC1	protein_coding	1.419444705	3.19E-17	2.11E-16	up
ENSG00000143469	SYT14	protein_coding	-4.278794105	3.23E-17	2.13E-16	down
ENSG00000175155	YPEL2	protein_coding	1.354438072	3.33E-17	2.20E-16	up
ENSG00000175866	BAIAP2	protein_coding	-1.284995444	3.40E-17	2.24E-16	down

ENSG00000116678	LEPR	protein_coding	2.669573126	3.44E-17	2.27E-16	up
ENSG00000106484	MEST	protein_coding	-1.602201129	3.45E-17	2.28E-16	down
ENSG00000180318	ALX1	protein_coding	1.583793264	3.62E-17	2.38E-16	up
ENSG00000009709	PAX7	protein_coding	1.892799716	3.62E-17	2.38E-16	up
ENSG00000159885	ZNF222	protein_coding	-6.163316888	3.64E-17	2.39E-16	down
ENSG00000123297	TSFM	protein_coding	-1.871136136	3.70E-17	2.43E-16	down
ENSG00000149591	TAGLN	protein_coding	6.953027073	3.72E-17	2.44E-16	up
ENSG00000198753	PLXNB3	protein_coding	6.953027073	3.72E-17	2.44E-16	up
ENSG00000059378	PARP12	protein_coding	-7.233706216	3.90E-17	2.56E-16	down
ENSG00000189114	BLOC1S3	protein_coding	1.172417007	4.13E-17	2.70E-16	up
ENSG00000178188	SH2B1	protein_coding	1.142843891	4.37E-17	2.86E-16	up
ENSG00000185112	FAM43A	protein_coding	-5.163316888	4.76E-17	3.11E-16	down
ENSG00000197119	SLC25A29	protein_coding	1.308973942	4.89E-17	3.19E-16	up
ENSG00000132024	CC2D1A	protein_coding	-1.134816845	5.02E-17	3.28E-16	down
ENSG00000165118	C9orf64	protein_coding	-1.109877629	5.13E-17	3.34E-16	down
ENSG00000176108	CHMP6	protein_coding	-1.745464373	5.44E-17	3.54E-16	down
ENSG00000157542	KCNJ6	protein_coding	-3.334428804	5.55E-17	3.61E-16	down
ENSG00000163462	TRIM46	protein_coding	6.933398267	5.80E-17	3.76E-16	up
ENSG00000203724	C1orf53	protein_coding	2.218658591	5.98E-17	3.87E-16	up
ENSG00000185163	DDX51	protein_coding	-1.297902928	6.09E-17	3.94E-16	down
ENSG00000137166	FOXP4	protein_coding	-1.076876872	6.32E-17	4.09E-16	down
ENSG00000133863	TEX15	protein_coding	-7.210622603	6.51E-17	4.20E-16	down
ENSG00000155729	KCTD18	protein_coding	1.41495096	6.55E-17	4.23E-16	up
ENSG00000163171	CDC42EP3	protein_coding	-1.209966981	6.65E-17	4.29E-16	down
ENSG00000166920	C15orf48	protein_coding	3.143504315	6.95E-17	4.48E-16	up
ENSG00000167645	YIF1B	protein_coding	-1.989780633	7.09E-17	4.56E-16	down
ENSG00000166311	SMPD1	protein_coding	-3.59881849	7.13E-17	4.59E-16	down
ENSG00000204149	AGAP6	protein_coding	1.896525631	7.58E-17	4.86E-16	up
ENSG00000159917	ZNF235	protein_coding	-1.78589368	8.60E-17	5.50E-16	down
ENSG00000198715	GLMP	protein_coding	-1.602876706	9.05E-17	5.79E-16	down
ENSG00000092208	GEMIN2	protein_coding	1.775282567	9.06E-17	5.79E-16	up
ENSG00000149922	TBX6	protein_coding	6.913498709	9.06E-17	5.79E-16	up
ENSG00000134049	IER3IP1	protein_coding	-1.620174563	9.54E-17	6.09E-16	down
ENSG00000145362	ANK2	protein_coding	1.115083831	9.94E-17	6.34E-16	up
ENSG00000197982	C1orf122	protein_coding	-1.462330591	1.02E-16	6.49E-16	down
ENSG00000141441	GAREM1	protein_coding	1.606691901	1.05E-16	6.68E-16	up
ENSG00000165698	SPACA9	protein_coding	-7.18716363	1.09E-16	6.92E-16	down
ENSG00000163389	POGLUT1	protein_coding	-1.387357162	1.15E-16	7.29E-16	down
ENSG00000104490	NCALD	protein_coding	1.378576891	1.17E-16	7.42E-16	up
ENSG00000124279	FASTKD3	protein_coding	1.03462249	1.17E-16	7.44E-16	up
ENSG00000065802	ASB1	protein_coding	-1.048002402	1.17E-16	7.44E-16	down
ENSG00000189050	RNFT1	protein_coding	-3.201284738	1.22E-16	7.71E-16	down
ENSG00000138336	TET1	protein_coding	-2.455206156	1.25E-16	7.91E-16	down
ENSG00000136010	ALDH1L2	protein_coding	1.113462841	1.43E-16	9.04E-16	up
ENSG00000009780	FAM76A	protein_coding	-3.011313794	1.49E-16	9.40E-16	down
ENSG00000137413	TAF8	protein_coding	-1.0026697	1.52E-16	9.58E-16	down
ENSG00000204128	C2orf72	protein_coding	1.493030246	1.62E-16	1.02E-15	up
ENSG00000178467	P4HTM	protein_coding	1.318267873	1.64E-16	1.03E-15	up
ENSG00000171931	FBXW10	protein_coding	1.810687904	1.65E-16	1.04E-15	up
ENSG00000163867	ZMYM6	protein_coding	-2.792479193	1.69E-16	1.06E-15	down
ENSG00000164463	CREBRF	protein_coding	1.204125866	1.74E-16	1.09E-15	up
ENSG00000123552	USP45	protein_coding	1.016876449	1.76E-16	1.10E-15	up
ENSG00000273604	EPOP	protein_coding	-1.539880239	1.80E-16	1.13E-15	down
ENSG00000114166	KAT2B	protein_coding	1.072976923	1.82E-16	1.14E-15	up

ENSG00000105270	CLIP3	protein_coding	-7.163316888	1.82E-16	1.14E-15	down
ENSG00000138400	MDH1B	protein_coding	-7.163316888	1.82E-16	1.14E-15	down
ENSG00000245680	ZNF585B	protein_coding	-7.163316888	1.82E-16	1.14E-15	down
ENSG00000196967	ZNF585A	protein_coding	-7.163316888	1.82E-16	1.14E-15	down
ENSG00000170837	GPR27	protein_coding	-7.163316888	1.82E-16	1.14E-15	down
ENSG00000166925	TSC22D4	protein_coding	-1.180343899	2.03E-16	1.27E-15	down
ENSG00000166896	ATP23	protein_coding	-3.737552982	2.05E-16	1.28E-15	down
ENSG00000121406	ZNF549	protein_coding	-3.737552982	2.05E-16	1.28E-15	down
ENSG00000214226	C17orf67	protein_coding	-6.089316306	2.09E-16	1.30E-15	down
ENSG00000104859	CLASRP	protein_coding	1.037419261	2.14E-16	1.33E-15	up
ENSG00000164758	MED30	protein_coding	1.743573708	2.16E-16	1.34E-15	up
ENSG00000070759	TESK2	protein_coding	6.872856725	2.22E-16	1.38E-15	up
ENSG00000147676	MAL2	protein_coding	6.872856725	2.22E-16	1.38E-15	up
ENSG00000182957	SPATA13	protein_coding	-1.695711338	2.24E-16	1.39E-15	down
ENSG00000146038	DCDC2	protein_coding	-1.766910577	2.26E-16	1.40E-15	down
ENSG00000153767	GTF2E1	protein_coding	-1.050842159	2.28E-16	1.41E-15	down
ENSG00000198131	ZNF544	protein_coding	-2.182425711	2.48E-16	1.53E-15	down
ENSG00000152402	GUCY1A2	protein_coding	1.01487573	2.51E-16	1.55E-15	up
ENSG00000102901	CENPT	protein_coding	-1.55845483	2.52E-16	1.56E-15	down
ENSG00000153157	SYCP2L	protein_coding	1.784914503	2.67E-16	1.65E-15	up
ENSG00000176542	USF3	protein_coding	-1.019889689	2.71E-16	1.67E-15	down
ENSG00000242612	DECR2	protein_coding	-2.563087631	2.77E-16	1.71E-15	down
ENSG00000164603	BMT2	protein_coding	-1.802394661	2.80E-16	1.72E-15	down
ENSG00000198088	NUP62CL	protein_coding	1.001224917	2.83E-16	1.74E-15	up
ENSG00000073050	XRCC1	protein_coding	-1.17512619	2.92E-16	1.80E-15	down
ENSG00000170537	TMC7	protein_coding	2.213893642	3.03E-16	1.86E-15	up
ENSG00000183072	NKX2-5	protein_coding	-7.139069342	3.06E-16	1.88E-15	down
ENSG00000158156	XKR8	protein_coding	-7.139069342	3.06E-16	1.88E-15	down
ENSG00000186020	ZNF529	protein_coding	-7.139069342	3.06E-16	1.88E-15	down
ENSG00000136842	TMOD1	protein_coding	-7.139069342	3.06E-16	1.88E-15	down
ENSG00000100099	HPS4	protein_coding	-1.145208814	3.14E-16	1.93E-15	down
ENSG00000260027	HOXB7	protein_coding	-1.268300393	3.22E-16	1.98E-15	down
ENSG00000104884	ERCC2	protein_coding	1.056283273	3.35E-16	2.05E-15	up
ENSG00000124802	EEF1E1	protein_coding	1.368064573	3.36E-16	2.06E-15	up
ENSG00000185477	GPRIN3	protein_coding	-1.20788244	3.41E-16	2.09E-15	down
ENSG00000106789	CORO2A	protein_coding	2.117969222	3.47E-16	2.12E-15	up
ENSG00000157326	DHRS4	protein_coding	-1.233883724	3.56E-16	2.18E-15	down
ENSG00000152061	RABGAP1L	protein_coding	1.058084331	3.63E-16	2.21E-15	up
ENSG00000181649	PHLDA2	protein_coding	-1.535402242	3.63E-16	2.22E-15	down
ENSG00000006625	GGCT	protein_coding	-2.147720033	3.64E-16	2.22E-15	down
ENSG00000169249	ZRSR2	protein_coding	-1.708938497	3.65E-16	2.22E-15	down
ENSG00000165914	TTC7B	protein_coding	1.201864405	3.66E-16	2.23E-15	up
ENSG00000186017	ZNF566	protein_coding	-1.256426292	4.02E-16	2.45E-15	down
ENSG00000158006	PAFAH2	protein_coding	1.307702705	4.61E-16	2.79E-15	up
ENSG00000134215	VAV3	protein_coding	1.822525049	4.83E-16	2.92E-15	up
ENSG00000153832	FBXO36	protein_coding	2.055517714	4.86E-16	2.95E-15	up
ENSG00000243943	ZNF512	protein_coding	-1.228192612	5.18E-16	3.14E-15	down
ENSG00000131944	FAAP24	protein_coding	-1.862605279	5.26E-16	3.18E-15	down
ENSG00000131981	LGALS3	protein_coding	1.147206444	5.61E-16	3.39E-15	up
ENSG00000027075	PRKCH	protein_coding	-1.20714539	5.89E-16	3.55E-15	down
ENSG00000171097	KYAT1	protein_coding	1.643046832	6.13E-16	3.69E-15	up
ENSG00000171428	NAT1	protein_coding	1.853198199	6.15E-16	3.70E-15	up
ENSG00000103024	NME3	protein_coding	-6.037786006	6.76E-16	4.06E-15	down
ENSG00000109680	TBC1D19	protein_coding	2.591570614	6.78E-16	4.08E-15	up

ENSG00000100505	TRIM9	protein_coding	1.530844384	7.11E-16	4.26E-15	up
ENSG00000152784	PRDM8	protein_coding	2.650464303	7.65E-16	4.58E-15	up
ENSG00000165886	UBTD1	protein_coding	-1.262475337	8.18E-16	4.89E-15	down
ENSG00000166856	GPR182	protein_coding	6.809662898	8.59E-16	5.13E-15	up
ENSG00000014257	ACPP	protein_coding	6.809662898	8.59E-16	5.13E-15	up
ENSG00000176182	MYPOP	protein_coding	-2.179805011	8.61E-16	5.14E-15	down
ENSG00000136002	ARHGEF4	protein_coding	-1.852036037	8.65E-16	5.16E-15	down
ENSG00000204410	MSH5	protein_coding	-7.089316306	8.68E-16	5.17E-15	down
ENSG00000100726	TELO2	protein_coding	-1.64761705	8.81E-16	5.24E-15	down
ENSG00000099139	PCSK5	protein_coding	4.809662898	9.26E-16	5.50E-15	up
ENSG00000113356	POLR3G	protein_coding	-1.494586029	9.45E-16	5.61E-15	down
ENSG00000186577	SMIM29	protein_coding	-2.578354387	9.50E-16	5.63E-15	down
ENSG00000141524	TMC6	protein_coding	-1.713471318	9.51E-16	5.64E-15	down
ENSG00000172183	ISG20	protein_coding	1.922897407	9.52E-16	5.64E-15	up
ENSG00000213949	ITGA1	protein_coding	2.960804424	9.77E-16	5.79E-15	up
ENSG00000179023	KLHDC7A	protein_coding	1.469892058	9.88E-16	5.85E-15	up
ENSG00000179546	HTR1D	protein_coding	1.469892058	9.88E-16	5.85E-15	up
ENSG00000136895	GARNL3	protein_coding	1.478384417	9.99E-16	5.91E-15	up
ENSG00000137802	MAPKBP1	protein_coding	1.50512494	1.02E-15	6.03E-15	up
ENSG00000154719	MRPL39	protein_coding	-1.158666244	1.13E-15	6.67E-15	down
ENSG00000103449	SALL1	protein_coding	-1.948303997	1.14E-15	6.70E-15	down
ENSG00000240065	PSMB9	protein_coding	-6.011313794	1.22E-15	7.15E-15	down
ENSG00000198915	RASGEF1A	protein_coding	2.249926374	1.23E-15	7.22E-15	up
ENSG00000196937	FAM3C	protein_coding	-1.154064575	1.24E-15	7.26E-15	down
ENSG00000137766	UNC13C	protein_coding	6.787967827	1.35E-15	7.92E-15	up
ENSG00000169862	CTNND2	protein_coding	6.787967827	1.35E-15	7.92E-15	up
ENSG00000119042	SATB2	protein_coding	1.057786837	1.35E-15	7.93E-15	up
ENSG00000197279	ZNF165	protein_coding	3.933398267	1.40E-15	8.21E-15	up
ENSG00000174669	SLC29A2	protein_coding	1.527081888	1.48E-15	8.65E-15	up
ENSG00000104915	STX10	protein_coding	-1.244453651	1.49E-15	8.67E-15	down
ENSG00000196227	FAM217B	protein_coding	-1.227279947	1.55E-15	9.01E-15	down
ENSG00000132205	EMILIN2	protein_coding	-4.139069342	1.62E-15	9.46E-15	down
ENSG00000161647	MPP3	protein_coding	-1.087041121	1.63E-15	9.47E-15	down
ENSG00000197766	CFD	protein_coding	-2.190538357	1.74E-15	1.01E-14	down
ENSG00000136425	CIB2	protein_coding	1.778339126	1.77E-15	1.03E-14	up
ENSG00000204386	NEU1	protein_coding	-2.938250332	1.78E-15	1.03E-14	down
ENSG00000000457	SCYL3	protein_coding	1.291474114	1.79E-15	1.04E-14	up
ENSG00000068394	GPKOW	protein_coding	-1.007818957	1.82E-15	1.05E-14	down
ENSG00000150967	ABCB9	protein_coding	1.646611978	1.84E-15	1.06E-14	up
ENSG00000204531	POU5F1	protein_coding	4.267135664	1.88E-15	1.09E-14	up
ENSG00000153487	ING1	protein_coding	1.433030376	1.91E-15	1.11E-14	up
ENSG00000162971	TYW5	protein_coding	-1.340490557	1.96E-15	1.13E-14	down
ENSG00000188997	KCTD21	protein_coding	1.60811029	1.99E-15	1.15E-14	up
ENSG00000213903	LTB4R	protein_coding	-1.898874288	2.07E-15	1.19E-14	down
ENSG00000167523	SPATA33	protein_coding	1.473966648	2.07E-15	1.19E-14	up
ENSG00000108932	SLC16A6	protein_coding	-1.012403855	2.10E-15	1.21E-14	down
ENSG00000184898	RBM43	protein_coding	1.561735385	2.11E-15	1.21E-14	up
ENSG00000249115	HAUS5	protein_coding	-1.205200969	2.31E-15	1.32E-14	down
ENSG00000047365	ARAP2	protein_coding	-7.037786006	2.48E-15	1.42E-14	down
ENSG00000163923	RPL39L	protein_coding	-1.170269649	2.58E-15	1.47E-14	down
ENSG00000175137	SH3BP5L	protein_coding	1.042540654	2.66E-15	1.52E-14	up
ENSG00000175105	ZNF654	protein_coding	-1.12654601	2.68E-15	1.53E-14	down
ENSG00000164978	NUDT2	protein_coding	1.638876329	2.71E-15	1.54E-14	up
ENSG00000166529	ZSCAN21	protein_coding	1.625737217	2.78E-15	1.58E-14	up

ENSG00000100532	CGRRF1	protein_coding	-1.385256273	2.98E-15	1.69E-14	down
ENSG00000128739	SNRPN	protein_coding	1.735161468	3.00E-15	1.71E-14	up
ENSG00000105072	C19orf44	protein_coding	-1.582786633	3.12E-15	1.77E-14	down
ENSG00000149328	GLB1L2	protein_coding	-1.752121455	3.16E-15	1.79E-14	down
ENSG00000132434	LANCL2	protein_coding	-1.435975713	3.23E-15	1.83E-14	down
ENSG00000244045	TMEM199	protein_coding	-1.536534211	3.24E-15	1.84E-14	down
ENSG00000160285	LSS	protein_coding	-1.130014913	3.24E-15	1.84E-14	down
ENSG00000257341	AL928654.4	protein_coding	6.743573708	3.36E-15	1.90E-14	up
ENSG00000137171	KLC4	protein_coding	1.894515606	3.39E-15	1.92E-14	up
ENSG00000251664	PCDHA12	protein_coding	-2.707087701	3.46E-15	1.95E-14	down
ENSG00000139190	VAMP1	protein_coding	1.798021492	3.48E-15	1.96E-14	up
ENSG00000100258	LMF2	protein_coding	-1.151073292	3.67E-15	2.07E-14	down
ENSG00000172197	MBOAT1	protein_coding	-2.117720021	3.70E-15	2.09E-14	down
ENSG00000145743	FBXL17	protein_coding	-1.661941017	3.80E-15	2.14E-14	down
ENSG00000163482	STK36	protein_coding	1.179392769	3.80E-15	2.14E-14	up
ENSG00000198832	SELENOM	protein_coding	1.097682208	3.81E-15	2.14E-14	up
ENSG00000173535	TNFRSF10C	protein_coding	-3.322515483	4.13E-15	2.32E-14	down
ENSG00000064787	BCAS1	protein_coding	2.743573708	4.17E-15	2.34E-14	up
ENSG00000108813	DLX4	protein_coding	-7.011313794	4.20E-15	2.36E-14	down
ENSG00000103742	IGDCC4	protein_coding	-7.011313794	4.20E-15	2.36E-14	down
ENSG00000141642	ELAC1	protein_coding	-7.011313794	4.20E-15	2.36E-14	down
ENSG00000240972	MIF	protein_coding	-1.168963451	4.44E-15	2.49E-14	down
ENSG00000133640	LRRIQ1	protein_coding	2.8233009	4.46E-15	2.49E-14	up
ENSG00000159184	HOXB13	protein_coding	1.726699889	4.47E-15	2.50E-14	up
ENSG00000126005	MMP24OS	protein_coding	-1.377988272	4.49E-15	2.51E-14	down
ENSG00000144741	SLC25A26	protein_coding	-1.556659316	4.55E-15	2.54E-14	down
ENSG00000117791	2-Mar	protein_coding	1.908632954	4.66E-15	2.60E-14	up
ENSG00000111252	SH2B3	protein_coding	-1.256426292	4.70E-15	2.62E-14	down
ENSG00000010704	HFE	protein_coding	-1.019250054	4.76E-15	2.65E-14	down
ENSG00000159263	SIM2	protein_coding	1.008846586	4.97E-15	2.77E-14	up
ENSG00000167685	ZNF444	protein_coding	-1.351906601	5.12E-15	2.85E-14	down
ENSG00000213799	ZNF845	protein_coding	-2.48423221	5.14E-15	2.86E-14	down
ENSG00000183475	ASB7	protein_coding	-1.039397056	5.21E-15	2.90E-14	down
ENSG00000198223	CSF2RA	protein_coding	-3.625660102	5.29E-15	2.94E-14	down
ENSG00000249222	ATP5L2	protein_coding	6.720853631	5.31E-15	2.95E-14	up
ENSG00000106328	FSCN3	protein_coding	6.720853631	5.31E-15	2.95E-14	up
ENSG00000196459	TRAPPC2	protein_coding	-1.476104329	5.86E-15	3.24E-14	down
ENSG00000066923	STAG3	protein_coding	1.23315816	6.18E-15	3.42E-14	up
ENSG00000076716	GPC4	protein_coding	3.425397748	6.24E-15	3.45E-14	up
ENSG00000275111	ZNF2	protein_coding	1.562126836	6.34E-15	3.50E-14	up
ENSG00000133597	ADCK2	protein_coding	1.23738832	6.54E-15	3.61E-14	up
ENSG00000124067	SLC12A4	protein_coding	-1.092196897	6.55E-15	3.62E-14	down
ENSG00000243335	KCTD7	protein_coding	-1.40927778	6.61E-15	3.65E-14	down
ENSG00000165055	METTL2B	protein_coding	-1.283143631	6.83E-15	3.77E-14	down
ENSG00000132563	REEP2	protein_coding	-6.984346747	7.13E-15	3.92E-14	down
ENSG00000188785	ZNF548	protein_coding	-6.984346747	7.13E-15	3.92E-14	down
ENSG00000149292	TTC12	protein_coding	1.799426942	7.32E-15	4.02E-14	up
ENSG00000198873	GRK5	protein_coding	-4.95686601	7.62E-15	4.18E-14	down
ENSG00000181396	OGFOD3	protein_coding	-1.380605704	7.72E-15	4.23E-14	down
ENSG00000272031	ANKRD34A	protein_coding	4.720853631	8.01E-15	4.39E-14	up
ENSG00000114388	NPRL2	protein_coding	-1.256426292	8.13E-15	4.45E-14	down
ENSG00000138785	INTS12	protein_coding	-1.705733694	8.15E-15	4.46E-14	down
ENSG00000166275	BORCS7	protein_coding	-1.731511175	8.22E-15	4.50E-14	down
ENSG00000100593	ISM2	protein_coding	6.697770018	8.40E-15	4.59E-14	up

ENSG00000124785	NRN1	protein_coding	6.697770018	8.40E-15	4.59E-14	up
ENSG00000169252	ADRB2	protein_coding	6.697770018	8.40E-15	4.59E-14	up
ENSG00000182287	AP1S2	protein_coding	-1.492866488	8.44E-15	4.61E-14	down
ENSG00000007520	TSR3	protein_coding	-1.021497224	9.31E-15	5.07E-14	down
ENSG00000134248	LAMTOR5	protein_coding	-1.197532603	9.95E-15	5.40E-14	down
ENSG00000181315	ZNF322	protein_coding	2.643046832	1.04E-14	5.62E-14	up
ENSG00000198841	KTI12	protein_coding	-2.173964132	1.04E-14	5.65E-14	down
ENSG00000168061	SAC3D1	protein_coding	-1.915389374	1.08E-14	5.84E-14	down
ENSG00000066583	ISOC1	protein_coding	-1.321196548	1.12E-14	6.07E-14	down
ENSG00000153933	DGKE	protein_coding	-1.627263988	1.18E-14	6.39E-14	down
ENSG00000168970	JMJD7-PLA2G	protein_coding	3.239999534	1.20E-14	6.49E-14	up
ENSG00000204947	ZNF425	protein_coding	-6.95686601	1.21E-14	6.55E-14	down
ENSG00000020181	ADGRA2	protein_coding	-3.426351294	1.25E-14	6.75E-14	down
ENSG00000153904	DDAH1	protein_coding	-1.748279389	1.33E-14	7.15E-14	down
ENSG00000197147	LRRC8B	protein_coding	-1.929615976	1.33E-14	7.16E-14	down
ENSG00000138650	PCDH10	protein_coding	6.674311045	1.33E-14	7.17E-14	up
ENSG00000128710	HOXD10	protein_coding	1.514949333	1.34E-14	7.22E-14	up
ENSG00000105173	CCNE1	protein_coding	-1.077969657	1.40E-14	7.51E-14	down
ENSG00000188428	BLOC1S5	protein_coding	-4.928851634	1.44E-14	7.75E-14	down
ENSG00000099256	PRTFDC1	protein_coding	-4.928851634	1.44E-14	7.75E-14	down
ENSG00000184305	CCSER1	protein_coding	2.397577853	1.48E-14	7.93E-14	up
ENSG00000147255	IGSF1	protein_coding	2.303001116	1.57E-14	8.41E-14	up
ENSG00000146143	PRIM2	protein_coding	-1.170696418	1.58E-14	8.48E-14	down
ENSG00000196369	SRGAP2B	protein_coding	1.781048413	1.66E-14	8.86E-14	up
ENSG00000143224	PPOX	protein_coding	2.112807517	1.81E-14	9.65E-14	up
ENSG00000121690	DEPDC7	protein_coding	1.45093284	1.82E-14	9.69E-14	up
ENSG00000184060	ADAP2	protein_coding	1.855783211	1.82E-14	9.69E-14	up
ENSG00000196284	SUPT3H	protein_coding	2.022109207	1.88E-14	1.00E-13	up
ENSG00000103811	CTSH	protein_coding	-1.865235535	1.92E-14	1.02E-13	down
ENSG00000128242	GAL3ST1	protein_coding	1.833459211	1.98E-14	1.06E-13	up
ENSG00000164162	ANAPC10	protein_coding	-1.284171283	2.04E-14	1.08E-13	down
ENSG00000154760	SLFN13	protein_coding	-6.928851634	2.07E-14	1.10E-13	down
ENSG00000118263	KLF7	protein_coding	1.165213942	2.12E-14	1.12E-13	up
ENSG00000173511	VEGFB	protein_coding	1.187792232	2.17E-14	1.15E-13	up
ENSG00000155158	TTC39B	protein_coding	1.692348384	2.18E-14	1.15E-13	up
ENSG00000180287	PLD5	protein_coding	1.216326705	2.30E-14	1.22E-13	up
ENSG00000188372	ZP3	protein_coding	1.328536208	2.31E-14	1.22E-13	up
ENSG00000077585	GPR137B	protein_coding	-1.291701767	2.39E-14	1.26E-13	down
ENSG00000142669	SH3BGRL3	protein_coding	-1.551047041	2.42E-14	1.27E-13	down
ENSG00000179397	CATSPERE	protein_coding	2.55092863	2.45E-14	1.29E-13	up
ENSG00000196353	CPNE4	protein_coding	2.20923728	2.49E-14	1.31E-13	up
ENSG00000104885	DOT1L	protein_coding	-1.114407287	2.53E-14	1.33E-13	down
ENSG00000186907	RTN4RL2	protein_coding	1.120218972	2.57E-14	1.35E-13	up
ENSG00000125249	RAP2A	protein_coding	1.012963609	2.60E-14	1.36E-13	up
ENSG00000170412	GPRC5C	protein_coding	-2.121496712	2.61E-14	1.37E-13	down
ENSG00000150456	EEF1AKMT1	protein_coding	1.578943006	2.72E-14	1.43E-13	up
ENSG00000168301	KCTD6	protein_coding	-1.931991342	2.73E-14	1.43E-13	down
ENSG00000152439	ZNF773	protein_coding	-4.900282482	2.74E-14	1.44E-13	down
ENSG00000196368	NUDT11	protein_coding	2.75987552	3.42E-14	1.79E-13	up
ENSG00000173040	EVC2	protein_coding	-6.900282482	3.53E-14	1.84E-13	down
ENSG00000115137	DNAJC27	protein_coding	-2.578354387	3.63E-14	1.89E-13	down
ENSG00000085563	ABCB1	protein_coding	3.065501803	3.67E-14	1.92E-13	up
ENSG00000110801	PSMD9	protein_coding	-2.304789314	3.70E-14	1.93E-13	down
ENSG00000170417	TMEM182	protein_coding	-3.554106841	3.71E-14	1.93E-13	down

ENSG00000198435	NRARP	protein_coding	2.948688138	3.72E-14	1.94E-13	up
ENSG00000076706	MCAM	protein_coding	2.948688138	3.72E-14	1.94E-13	up
ENSG00000115998	C2orf42	protein_coding	-1.795945822	4.29E-14	2.23E-13	down
ENSG00000113812	ACTR8	protein_coding	-1.095672937	4.52E-14	2.34E-13	down
ENSG00000137502	RAB30	protein_coding	1.999913461	4.52E-14	2.34E-13	up
ENSG00000198960	ARMCX6	protein_coding	-1.598117642	4.64E-14	2.41E-13	down
ENSG00000139826	ABHD13	protein_coding	-2.377441693	4.67E-14	2.42E-13	down
ENSG00000066651	TRMT11	protein_coding	-1.091367046	4.87E-14	2.52E-13	down
ENSG00000143630	HCN3	protein_coding	1.792817227	4.89E-14	2.53E-13	up
ENSG00000113083	LOX	protein_coding	1.65918152	4.97E-14	2.57E-13	up
ENSG00000021645	NRXN3	protein_coding	4.13589113	5.04E-14	2.60E-13	up
ENSG00000125703	ATG4C	protein_coding	-1.841388793	5.19E-14	2.68E-13	down
ENSG00000179242	CDH4	protein_coding	5.576463722	5.28E-14	2.72E-13	up
ENSG00000092421	SEMA6A	protein_coding	5.576463722	5.28E-14	2.72E-13	up
ENSG00000144847	IGSF11	protein_coding	6.601554703	5.34E-14	2.75E-13	up
ENSG00000283632	EXOC3L2	protein_coding	6.601554703	5.34E-14	2.75E-13	up
ENSG00000166822	TMEM170A	protein_coding	-1.205800219	5.55E-14	2.85E-13	down
ENSG00000168528	SERINC2	protein_coding	1.55092863	5.80E-14	2.98E-13	up
ENSG00000130827	PLXNA3	protein_coding	1.940875148	5.84E-14	3.00E-13	up
ENSG00000134253	TRIM45	protein_coding	-6.871136136	6.03E-14	3.09E-13	down
ENSG00000221916	C19orf73	protein_coding	-6.871136136	6.03E-14	3.09E-13	down
ENSG00000204611	ZNF616	protein_coding	-6.871136136	6.03E-14	3.09E-13	down
ENSG00000174851	YIF1A	protein_coding	-1.03620497	6.14E-14	3.14E-13	down
ENSG00000136866	ZFP37	protein_coding	3.047354456	6.21E-14	3.17E-13	up
ENSG00000173409	ARV1	protein_coding	-1.007989263	6.29E-14	3.21E-13	down
ENSG00000175764	TTLL11	protein_coding	3.787967827	6.46E-14	3.30E-13	up
ENSG00000137731	FXYD2	protein_coding	2.521181286	6.54E-14	3.34E-13	up
ENSG00000156463	SH3RF2	protein_coding	-2.90551913	6.91E-14	3.53E-13	down
ENSG00000174132	FAM174A	protein_coding	-2.323540488	7.34E-14	3.74E-13	down
ENSG00000169184	MN1	protein_coding	2.042232023	8.11E-14	4.12E-13	up
ENSG00000137825	ITPKA	protein_coding	-3.984346747	8.25E-14	4.18E-13	down
ENSG00000116717	GADD45A	protein_coding	-1.506652922	8.32E-14	4.22E-13	down
ENSG00000130363	RSPH3	protein_coding	1.509108454	8.43E-14	4.27E-13	up
ENSG00000185453	ZSWIM9	protein_coding	-1.654018657	8.48E-14	4.29E-13	down
ENSG00000165091	TMC1	protein_coding	6.576463722	8.50E-14	4.30E-13	up
ENSG00000273802	HIST1H2BG	protein_coding	6.576463722	8.50E-14	4.30E-13	up
ENSG00000115828	QPCT	protein_coding	-1.073303988	8.52E-14	4.31E-13	down
ENSG00000077454	LRCH4	protein_coding	-3.355961966	8.59E-14	4.34E-13	down
ENSG00000169991	IFFO2	protein_coding	1.050235046	8.67E-14	4.38E-13	up
ENSG00000153443	UBALD1	protein_coding	-1.10103261	8.72E-14	4.40E-13	down
ENSG00000106009	BRAT1	protein_coding	-1.176766843	8.91E-14	4.48E-13	down
ENSG00000158715	SLC45A3	protein_coding	3.328536208	9.17E-14	4.61E-13	up
ENSG00000099377	HSD3B7	protein_coding	-1.921559142	9.32E-14	4.69E-13	down
ENSG00000152942	RAD17	protein_coding	-1.050677627	9.85E-14	4.95E-13	down
ENSG00000214776	AC092821.1	protein_coding	-4.841388793	9.90E-14	4.97E-13	down
ENSG00000163393	SLC22A15	protein_coding	2.250533696	1.02E-13	5.10E-13	up
ENSG00000023608	SNAPC1	protein_coding	1.18567945	1.02E-13	5.11E-13	up
ENSG00000157617	C2CD2	protein_coding	-1.384181839	1.02E-13	5.11E-13	down
ENSG00000197457	STMN3	protein_coding	-6.841388793	1.03E-13	5.18E-13	down
ENSG00000197757	HOXC6	protein_coding	-6.841388793	1.03E-13	5.18E-13	down
ENSG00000219438	FAM19A5	protein_coding	-6.841388793	1.03E-13	5.18E-13	down
ENSG00000165171	METTL27	protein_coding	-6.841388793	1.03E-13	5.18E-13	down
ENSG00000127564	PKMYT1	protein_coding	-1.076176761	1.04E-13	5.19E-13	down
ENSG00000097046	CDC7	protein_coding	-1.033300608	1.05E-13	5.22E-13	down

ENSG00000140807	NKD1	protein_coding	-1.741853119	1.15E-13	5.71E-13	down
ENSG00000153574	RPIA	protein_coding	-1.578354387	1.15E-13	5.72E-13	down
ENSG00000266173	STRADA	protein_coding	1.470555213	1.18E-13	5.89E-13	up
ENSG00000164181	ELOVL7	protein_coding	-2.978892317	1.22E-13	6.06E-13	down
ENSG00000220205	VAMP2	protein_coding	1.534987086	1.22E-13	6.07E-13	up
ENSG00000112511	PHF1	protein_coding	-2.59746321	1.24E-13	6.18E-13	down
ENSG00000135828	RNASEL	protein_coding	-2.59746321	1.24E-13	6.18E-13	down
ENSG00000232434	C9orf172	protein_coding	1.734050934	1.25E-13	6.19E-13	up
ENSG00000140987	ZSCAN32	protein_coding	1.36248354	1.25E-13	6.22E-13	up
ENSG00000273748	AL592183.1	protein_coding	2.203005326	1.27E-13	6.30E-13	up
ENSG00000146476	ARMT1	protein_coding	-1.186961473	1.28E-13	6.34E-13	down
ENSG00000168447	SCNN1B	protein_coding	6.55092863	1.36E-13	6.73E-13	up
ENSG00000105963	ADAP1	protein_coding	6.55092863	1.36E-13	6.73E-13	up
ENSG00000142409	ZNF787	protein_coding	-1.074878923	1.36E-13	6.73E-13	down
ENSG00000204315	FKBPL	protein_coding	1.9973303	1.45E-13	7.19E-13	up
ENSG00000105204	DYRK1B	protein_coding	1.052122773	1.46E-13	7.22E-13	up
ENSG00000173175	ADCY5	protein_coding	5.524933421	1.48E-13	7.31E-13	up
ENSG00000174938	SEZ6L2	protein_coding	1.163905507	1.54E-13	7.59E-13	up
ENSG00000153234	NR4A2	protein_coding	-3.95686601	1.59E-13	7.82E-13	down
ENSG00000115526	CHST10	protein_coding	-1.124921509	1.61E-13	7.91E-13	down
ENSG00000116793	PHTF1	protein_coding	-1.458060153	1.61E-13	7.92E-13	down
ENSG00000112394	SLC16A10	protein_coding	2.237113181	1.62E-13	7.96E-13	up
ENSG00000234616	JRK	protein_coding	1.023066536	1.74E-13	8.55E-13	up
ENSG00000162804	SNED1	protein_coding	2.895576801	1.76E-13	8.62E-13	up
ENSG00000120318	ARAP3	protein_coding	1.526949767	1.76E-13	8.65E-13	up
ENSG00000108387	4-Sep	protein_coding	-6.811015144	1.78E-13	8.71E-13	down
ENSG00000152766	ANKRD22	protein_coding	-6.811015144	1.78E-13	8.71E-13	down
ENSG00000172007	RAB33B	protein_coding	-1.701837441	1.83E-13	8.96E-13	down
ENSG00000099860	GADD45B	protein_coding	1.841605791	1.85E-13	9.04E-13	up
ENSG00000203727	SAMD5	protein_coding	1.136533042	1.99E-13	9.69E-13	up
ENSG00000115657	ABCB6	protein_coding	6.524933421	2.17E-13	1.06E-12	up
ENSG00000147027	TMEM47	protein_coding	-4.286173636	2.31E-13	1.12E-12	down
ENSG00000178694	NSUN3	protein_coding	-1.521398502	2.39E-13	1.16E-12	down
ENSG00000115540	MOB4	protein_coding	1.079857096	2.42E-13	1.18E-12	up
ENSG00000158796	DEDD	protein_coding	-1.114033193	2.47E-13	1.20E-12	down
ENSG00000126882	FAM78A	protein_coding	5.49846121	2.48E-13	1.20E-12	up
ENSG00000214300	SPDYE3	protein_coding	5.49846121	2.48E-13	1.20E-12	up
ENSG00000026950	BTN3A1	protein_coding	1.855466588	2.57E-13	1.24E-12	up
ENSG00000196655	TRAPPC4	protein_coding	-1.096269067	2.57E-13	1.24E-12	down
ENSG00000127124	HIVEP3	protein_coding	-3.478818714	2.58E-13	1.25E-12	down
ENSG00000132688	NES	protein_coding	4.065501803	2.61E-13	1.26E-12	up
ENSG00000277462	ZNF670	protein_coding	-5.748279389	2.64E-13	1.28E-12	down
ENSG00000197620	CXorf40A	protein_coding	-2.086501291	2.65E-13	1.28E-12	down
ENSG00000173264	GPR137	protein_coding	1.309170884	2.81E-13	1.36E-12	up
ENSG00000197557	TTC30A	protein_coding	-1.256426292	2.86E-13	1.38E-12	down
ENSG00000179152	TCAIM	protein_coding	-1.312074322	2.90E-13	1.40E-12	down
ENSG00000155744	FAM126B	protein_coding	1.267909849	2.92E-13	1.40E-12	up
ENSG00000101197	BIRC7	protein_coding	-6.779988248	3.06E-13	1.47E-12	down
ENSG00000142677	IL22RA1	protein_coding	-6.779988248	3.06E-13	1.47E-12	down
ENSG00000155265	GOLGA7B	protein_coding	-6.779988248	3.06E-13	1.47E-12	down
ENSG00000184357	HIST1H1B	protein_coding	-6.779988248	3.06E-13	1.47E-12	down
ENSG00000082458	DLG3	protein_coding	-1.082659225	3.21E-13	1.54E-12	down
ENSG00000241127	YAE1D1	protein_coding	-2.239938169	3.44E-13	1.65E-12	down
ENSG00000046647	GEMIN8	protein_coding	-2.136132059	3.73E-13	1.78E-12	down

ENSG00000158850	B4GALT3	protein_coding	-1.727732011	3.79E-13	1.81E-12	down
ENSG00000155621	C9orf85	protein_coding	2.209891712	4.07E-13	1.94E-12	up
ENSG00000007341	ST7L	protein_coding	-1.287453188	4.14E-13	1.97E-12	down
ENSG00000109063	MYH3	protein_coding	5.471494162	4.17E-13	1.98E-12	up
ENSG00000168938	PPIC	protein_coding	-1.45436567	4.21E-13	2.00E-12	down
ENSG00000167186	COQ7	protein_coding	-1.11174654	4.25E-13	2.02E-12	down
ENSG00000256188	TAS2R30	protein_coding	2.676459512	4.27E-13	2.03E-12	up
ENSG00000143995	MEIS1	protein_coding	-2.163316888	4.40E-13	2.09E-12	down
ENSG00000149548	CCDC15	protein_coding	1.205916922	4.53E-13	2.15E-12	up
ENSG00000135482	ZC3H10	protein_coding	1.205916922	4.53E-13	2.15E-12	up
ENSG00000134575	ACP2	protein_coding	-1.241926723	4.66E-13	2.21E-12	down
ENSG00000112282	MED23	protein_coding	-1.014608372	4.74E-13	2.25E-12	down
ENSG00000170234	PWWP2A	protein_coding	1.0361272	4.82E-13	2.28E-12	up
ENSG00000196437	ZNF569	protein_coding	-5.715857911	4.85E-13	2.29E-12	down
ENSG00000204305	AGER	protein_coding	2.972392398	5.03E-13	2.37E-12	up
ENSG00000174206	C12orf66	protein_coding	-1.084539775	5.11E-13	2.41E-12	down
ENSG00000185798	WDR53	protein_coding	1.753842043	5.23E-13	2.47E-12	up
ENSG00000168389	MFSD2A	protein_coding	-6.748279389	5.28E-13	2.49E-12	down
ENSG00000168765	GSTM4	protein_coding	-6.748279389	5.28E-13	2.49E-12	down
ENSG00000163485	ADORA1	protein_coding	-6.748279389	5.28E-13	2.49E-12	down
ENSG00000186469	GNG2	protein_coding	-6.748279389	5.28E-13	2.49E-12	down
ENSG00000008277	ADAM22	protein_coding	1.502565608	5.30E-13	2.49E-12	up
ENSG00000278023	RDM1	protein_coding	2.518867421	5.41E-13	2.54E-12	up
ENSG00000123838	C4BPA	protein_coding	6.471494162	5.56E-13	2.61E-12	up
ENSG00000135519	KCNH3	protein_coding	-2.390282039	5.66E-13	2.65E-12	down
ENSG00000112039	FANCE	protein_coding	-3.281961384	5.87E-13	2.75E-12	down
ENSG00000114698	PLSCR4	protein_coding	1.388345604	6.18E-13	2.89E-12	up
ENSG00000163328	GPR155	protein_coding	2.391271964	6.19E-13	2.90E-12	up
ENSG00000034693	PEX3	protein_coding	-2.435749992	6.24E-13	2.92E-12	down
ENSG00000149016	TUT1	protein_coding	-1.912471891	6.54E-13	3.06E-12	down
ENSG00000132031	MATN3	protein_coding	-4.748279389	6.85E-13	3.20E-12	down
ENSG00000104081	BMF	protein_coding	1.78664243	6.89E-13	3.21E-12	up
ENSG00000105668	UPK1A	protein_coding	5.444013426	7.00E-13	3.26E-12	up
ENSG00000270629	NBPF14	protein_coding	-1.868610262	7.03E-13	3.28E-12	down
ENSG00000132846	ZBED3	protein_coding	1.244002698	7.24E-13	3.37E-12	up
ENSG00000109103	UNC119	protein_coding	-1.22757143	7.55E-13	3.51E-12	down
ENSG00000130294	KIF1A	protein_coding	2.743573708	7.74E-13	3.60E-12	up
ENSG00000250120	PCDHA10	protein_coding	2.743573708	7.74E-13	3.60E-12	up
ENSG00000178662	CSRNP3	protein_coding	3.246074048	7.80E-13	3.62E-12	up
ENSG00000271303	SRXN1	protein_coding	-2.292050202	7.89E-13	3.66E-12	down
ENSG00000116922	C1orf109	protein_coding	-1.104423199	7.89E-13	3.66E-12	down
ENSG00000156398	SFXN2	protein_coding	-1.928851634	8.08E-13	3.74E-12	down
ENSG00000139636	LMBR1L	protein_coding	1.950024585	8.23E-13	3.80E-12	up
ENSG00000057294	PKP2	protein_coding	1.026967201	8.37E-13	3.87E-12	up
ENSG00000126705	AHDC1	protein_coding	1.077992747	8.42E-13	3.89E-12	up
ENSG00000037965	HOXC8	protein_coding	-4.226052643	8.57E-13	3.96E-12	down
ENSG00000241978	AKAP2	protein_coding	6.444013426	8.93E-13	4.11E-12	up
ENSG00000170775	GPR37	protein_coding	6.444013426	8.93E-13	4.11E-12	up
ENSG00000160219	GAB3	protein_coding	6.444013426	8.93E-13	4.11E-12	up
ENSG00000184408	KCND2	protein_coding	6.444013426	8.93E-13	4.11E-12	up
ENSG00000159640	ACE	protein_coding	6.444013426	8.93E-13	4.11E-12	up
ENSG00000134533	RERG	protein_coding	-6.715857911	9.14E-13	4.19E-12	down
ENSG00000167984	NLRC3	protein_coding	-6.715857911	9.14E-13	4.19E-12	down
ENSG00000177125	ZBTB34	protein_coding	-1.213781955	9.25E-13	4.24E-12	down

ENSG00000108423	TUBD1	protein_coding	-1.213781955	9.25E-13	4.24E-12	down
ENSG00000151729	SLC25A4	protein_coding	-1.176255944	9.49E-13	4.34E-12	down
ENSG00000177570	SAMD12	protein_coding	2.004101258	9.77E-13	4.46E-12	up
ENSG00000167130	DOLPP1	protein_coding	1.083266082	9.83E-13	4.49E-12	up
ENSG00000122641	INHBA	protein_coding	3.674311045	9.92E-13	4.53E-12	up
ENSG00000186205	1-Mar	protein_coding	1.055851633	1.00E-12	4.56E-12	up
ENSG00000075399	VPS9D1	protein_coding	1.602711172	1.05E-12	4.81E-12	up
ENSG00000170425	ADORA2B	protein_coding	1.102428372	1.08E-12	4.91E-12	up
ENSG00000129158	SERGEF	protein_coding	1.486077485	1.10E-12	4.99E-12	up
ENSG00000151689	INPP1	protein_coding	1.509108454	1.11E-12	5.05E-12	up
ENSG00000092098	RNF31	protein_coding	-1.147612805	1.13E-12	5.12E-12	down
ENSG00000162396	PARS2	protein_coding	1.301569161	1.20E-12	5.47E-12	up
ENSG00000196296	ATP2A1	protein_coding	2.428071882	1.21E-12	5.48E-12	up
ENSG00000198910	L1CAM	protein_coding	1.713200059	1.23E-12	5.59E-12	up
ENSG00000246922	UBAP1L	protein_coding	-3.114407287	1.26E-12	5.71E-12	down
ENSG00000004766	VPS50	protein_coding	-1.226982514	1.31E-12	5.92E-12	down
ENSG00000135917	SLC19A3	protein_coding	2.82157622	1.38E-12	6.23E-12	up
ENSG00000115267	IFIH1	protein_coding	6.41599905	1.44E-12	6.49E-12	up
ENSG00000164651	SP8	protein_coding	6.41599905	1.44E-12	6.49E-12	up
ENSG00000171450	CDK5R2	protein_coding	6.41599905	1.44E-12	6.49E-12	up
ENSG00000198870	STKLD1	protein_coding	6.41599905	1.44E-12	6.49E-12	up
ENSG00000111713	GYS2	protein_coding	6.41599905	1.44E-12	6.49E-12	up
ENSG00000163590	PPM1L	protein_coding	1.640814133	1.44E-12	6.50E-12	up
ENSG00000105085	MED26	protein_coding	-1.869403169	1.45E-12	6.53E-12	down
ENSG00000177679	SRRM3	protein_coding	-3.606923539	1.46E-12	6.57E-12	down
ENSG00000155970	MICU3	protein_coding	1.206052202	1.47E-12	6.64E-12	up
ENSG00000171246	NPTX1	protein_coding	2.092723271	1.49E-12	6.69E-12	up
ENSG00000263956	NBPF11	protein_coding	-1.827968277	1.52E-12	6.86E-12	down
ENSG00000185187	SIGIRR	protein_coding	-2.063781214	1.56E-12	7.04E-12	down
ENSG00000141391	PRELID3A	protein_coding	-6.682691047	1.58E-12	7.12E-12	down
ENSG00000170396	ZNF804A	protein_coding	-6.682691047	1.58E-12	7.12E-12	down
ENSG00000105393	BABAM1	protein_coding	1.53771301	1.61E-12	7.20E-12	up
ENSG00000111224	PARP11	protein_coding	2.168071536	1.61E-12	7.23E-12	up
ENSG00000115266	APC2	protein_coding	-5.648743715	1.64E-12	7.35E-12	down
ENSG00000175787	ZNF169	protein_coding	2.55092863	1.68E-12	7.52E-12	up
ENSG00000197980	LEKR1	protein_coding	3.650464303	1.71E-12	7.66E-12	up
ENSG00000167515	TRAPPC2L	protein_coding	-1.09563008	1.92E-12	8.55E-12	down
ENSG00000251493	FOXD1	protein_coding	1.925777039	1.94E-12	8.65E-12	up
ENSG00000115257	PCSK4	protein_coding	5.387429897	1.99E-12	8.85E-12	up
ENSG00000049283	EPN3	protein_coding	5.387429897	1.99E-12	8.85E-12	up
ENSG00000171045	TSNARE1	protein_coding	5.387429897	1.99E-12	8.85E-12	up
ENSG00000254685	FPGT	protein_coding	-1.139912284	2.11E-12	9.39E-12	down
ENSG00000127423	AUNIP	protein_coding	-2.025100746	2.20E-12	9.77E-12	down
ENSG00000070610	GBA2	protein_coding	-1.118922769	2.24E-12	9.92E-12	down
ENSG00000239305	RNF103	protein_coding	-1.118922769	2.24E-12	9.92E-12	down
ENSG00000186310	NAP1L3	protein_coding	6.387429897	2.31E-12	1.02E-11	up
ENSG00000129514	FOXA1	protein_coding	-1.625660102	2.34E-12	1.04E-11	down
ENSG00000139187	KLRG1	protein_coding	-4.682691047	2.50E-12	1.11E-11	down
ENSG00000139438	FAM222A	protein_coding	-4.682691047	2.50E-12	1.11E-11	down
ENSG00000113119	TMCO6	protein_coding	1.366806225	2.52E-12	1.11E-11	up
ENSG00000188549	C15orf52	protein_coding	1.057954994	2.62E-12	1.15E-11	up
ENSG00000155816	FMN2	protein_coding	-2.865235535	2.69E-12	1.19E-11	down
ENSG00000092929	UNC13D	protein_coding	-1.58400095	2.70E-12	1.19E-11	down
ENSG00000114737	CISH	protein_coding	-6.648743715	2.75E-12	1.21E-11	down

ENSG00000144134	RABL2A	protein_coding	-6.648743715	2.75E-12	1.21E-11	down
ENSG00000168243	GNG4	protein_coding	-6.648743715	2.75E-12	1.21E-11	down
ENSG00000005981	ASB4	protein_coding	-6.648743715	2.75E-12	1.21E-11	down
ENSG00000137878	GCOM1	protein_coding	-6.648743715	2.75E-12	1.21E-11	down
ENSG00000117472	TSPAN1	protein_coding	-6.648743715	2.75E-12	1.21E-11	down
ENSG00000092295	TGM1	protein_coding	-6.648743715	2.75E-12	1.21E-11	down
ENSG00000158966	CACHD1	protein_coding	-1.870243656	2.98E-12	1.30E-11	down
ENSG00000100926	TM9SF1	protein_coding	1.429819544	3.06E-12	1.34E-11	up
ENSG00000115935	WIPF1	protein_coding	1.274848338	3.06E-12	1.34E-11	up
ENSG00000129084	PSMA1	protein_coding	-1.185343194	3.10E-12	1.35E-11	down
ENSG00000114841	DNAH1	protein_coding	1.495186078	3.34E-12	1.46E-11	up
ENSG00000114735	HEMK1	protein_coding	1.54634136	3.35E-12	1.46E-11	up
ENSG00000152219	ARL14EP	protein_coding	-1.074752332	3.37E-12	1.47E-11	down
ENSG00000186197	EDARADD	protein_coding	-1.0577067	3.40E-12	1.48E-11	down
ENSG00000178202	KDELC2	protein_coding	-1.087920568	3.55E-12	1.54E-11	down
ENSG00000151276	MAGI1	protein_coding	1.005668553	3.56E-12	1.55E-11	up
ENSG00000073910	FRY	protein_coding	2.065501803	3.63E-12	1.58E-11	up
ENSG00000049246	PER3	protein_coding	-1.154546678	3.70E-12	1.61E-11	down
ENSG00000169814	BTD	protein_coding	-1.885782912	3.71E-12	1.61E-11	down
ENSG00000104313	EYA1	protein_coding	6.358283552	3.73E-12	1.62E-11	up
ENSG00000070915	SLC12A3	protein_coding	6.358283552	3.73E-12	1.62E-11	up
ENSG00000165309	ARMC3	protein_coding	6.358283552	3.73E-12	1.62E-11	up
ENSG00000149243	KLHL35	protein_coding	6.358283552	3.73E-12	1.62E-11	up
ENSG00000159588	CCDC17	protein_coding	6.358283552	3.73E-12	1.62E-11	up
ENSG00000186364	NUDT17	protein_coding	3.18097902	3.86E-12	1.67E-11	up
ENSG00000198203	SULT1C2	protein_coding	3.18097902	3.86E-12	1.67E-11	up
ENSG00000170185	USP38	protein_coding	-1.13203543	3.96E-12	1.71E-11	down
ENSG00000188234	AGAP4	protein_coding	2.139502384	4.01E-12	1.73E-11	up
ENSG00000116785	CFHR3	protein_coding	3.939970921	4.07E-12	1.76E-11	up
ENSG00000135074	ADAM19	protein_coding	-1.728914064	4.18E-12	1.80E-11	down
ENSG00000144583	4-Mar	protein_coding	-1.953034149	4.20E-12	1.81E-11	down
ENSG00000174628	IQCK	protein_coding	-2.478818714	4.22E-12	1.82E-11	down
ENSG00000111052	LIN7A	protein_coding	1.196746336	4.24E-12	1.83E-11	up
ENSG00000167136	ENDOG	protein_coding	-2.536534211	4.54E-12	1.95E-11	down
ENSG00000138378	STAT4	protein_coding	-2.125181759	4.54E-12	1.95E-11	down
ENSG00000135821	GLUL	protein_coding	1.611470172	4.57E-12	1.97E-11	up
ENSG00000095383	TBC1D2	protein_coding	-1.361222482	4.62E-12	1.98E-11	down
ENSG00000146802	TMEM168	protein_coding	-1.0516065	4.76E-12	2.04E-11	down
ENSG00000171100	MTM1	protein_coding	-1.620700678	4.78E-12	2.05E-11	down
ENSG00000158079	PTPDC1	protein_coding	1.565236466	4.79E-12	2.06E-11	up
ENSG00000111424	VDR	protein_coding	-2.600380694	4.79E-12	2.06E-11	down
ENSG00000119699	TGFB3	protein_coding	-6.613978297	4.80E-12	2.06E-11	down
ENSG00000197208	SLC22A4	protein_coding	-6.613978297	4.80E-12	2.06E-11	down
ENSG00000158125	XDH	protein_coding	-6.613978297	4.80E-12	2.06E-11	down
ENSG00000260456	C16orf95	protein_coding	-6.613978297	4.80E-12	2.06E-11	down
ENSG00000005243	COPZ2	protein_coding	-6.613978297	4.80E-12	2.06E-11	down
ENSG00000254221	PCDHGB1	protein_coding	-6.613978297	4.80E-12	2.06E-11	down
ENSG00000137404	NRM	protein_coding	1.988196076	4.88E-12	2.09E-11	up
ENSG00000154917	RAB6B	protein_coding	1.754800963	4.93E-12	2.11E-11	up
ENSG00000099785	2-Mar	protein_coding	1.754800963	4.93E-12	2.11E-11	up
ENSG00000168939	SPRY3	protein_coding	3.601554703	5.10E-12	2.18E-11	up
ENSG00000277586	NEFL	protein_coding	1.052677762	5.39E-12	2.30E-11	up
ENSG00000235376	RPEL1	protein_coding	-5.578354387	5.60E-12	2.39E-11	down
ENSG00000122481	RWDD3	protein_coding	5.328536208	5.66E-12	2.41E-11	up

ENSG00000162624	LHX8	protein_coding	6.328536208	6.03E-12	2.56E-11	up
ENSG00000189157	FAM47E	protein_coding	6.328536208	6.03E-12	2.56E-11	up
ENSG00000106571	GLI3	protein_coding	1.433505768	6.47E-12	2.74E-11	up
ENSG00000160606	TLCD1	protein_coding	-1.310197549	6.66E-12	2.82E-11	down
ENSG00000197822	OCLN	protein_coding	-1.426351294	6.73E-12	2.85E-11	down
ENSG00000073464	CLCN4	protein_coding	-1.426351294	6.73E-12	2.85E-11	down
ENSG00000164542	KIAA0895	protein_coding	-1.749965765	6.76E-12	2.86E-11	down
ENSG00000128564	VGF	protein_coding	-2.406173412	6.87E-12	2.91E-11	down
ENSG00000156973	PDE6D	protein_coding	-1.264789765	6.89E-12	2.91E-11	down
ENSG00000135469	COQ10A	protein_coding	-2.219900416	7.31E-12	3.08E-11	down
ENSG00000100228	RAB36	protein_coding	-1.441781334	7.57E-12	3.19E-11	down
ENSG00000104957	CCDC130	protein_coding	-1.608728036	7.57E-12	3.18E-11	down
ENSG00000197728	RPS26	protein_coding	-2.015418193	7.63E-12	3.21E-11	down
ENSG00000149970	CNKSR2	protein_coding	1.037635009	8.01E-12	3.37E-11	up
ENSG00000278318	ZNF229	protein_coding	2.36248354	8.24E-12	3.46E-11	up
ENSG00000110002	VWA5A	protein_coding	-6.578354387	8.38E-12	3.51E-11	down
ENSG00000069493	CLEC2D	protein_coding	-6.578354387	8.38E-12	3.51E-11	down
ENSG00000117226	GBP3	protein_coding	-6.578354387	8.38E-12	3.51E-11	down
ENSG00000161040	FBXL13	protein_coding	-6.578354387	8.38E-12	3.51E-11	down
ENSG00000131378	RFTN1	protein_coding	-6.578354387	8.38E-12	3.51E-11	down
ENSG00000204131	NHSL2	protein_coding	-6.578354387	8.38E-12	3.51E-11	down
ENSG00000170214	ADRA1B	protein_coding	1.831036549	8.38E-12	3.51E-11	up
ENSG00000244242	IFITM10	protein_coding	2.251368348	8.51E-12	3.56E-11	up
ENSG00000148082	SHC3	protein_coding	2.569544308	8.52E-12	3.56E-11	up
ENSG00000230667	SETSIP	protein_coding	2.569544308	8.52E-12	3.56E-11	up
ENSG00000241322	CDRT1	protein_coding	-2.919391305	8.90E-12	3.71E-11	down
ENSG00000164011	ZNF691	protein_coding	1.321002536	9.01E-12	3.76E-11	up
ENSG00000176340	COX8A	protein_coding	-1.264887871	9.06E-12	3.78E-11	down
ENSG00000196912	ANKRD36B	protein_coding	1.016592202	9.18E-12	3.82E-11	up
ENSG00000167550	RHEBL1	protein_coding	-4.613978297	9.19E-12	3.83E-11	down
ENSG00000156239	N6AMT1	protein_coding	-1.904124548	9.47E-12	3.94E-11	down
ENSG00000110921	MVK	protein_coding	1.198139571	9.51E-12	3.96E-11	up
ENSG00000121454	LHX4	protein_coding	-1.528505838	9.59E-12	3.99E-11	down
ENSG00000239590	OR1J4	protein_coding	6.298162559	9.76E-12	4.05E-11	up
ENSG00000004838	ZMYND10	protein_coding	6.298162559	9.76E-12	4.05E-11	up
ENSG00000152463	OLAH	protein_coding	6.298162559	9.76E-12	4.05E-11	up
ENSG00000136014	USP44	protein_coding	6.298162559	9.76E-12	4.05E-11	up
ENSG00000122691	TWIST1	protein_coding	1.125622795	9.77E-12	4.05E-11	up
ENSG00000197008	ZNF138	protein_coding	2.110356038	9.90E-12	4.10E-11	up
ENSG00000174796	THAP6	protein_coding	1.278905441	9.94E-12	4.12E-11	up
ENSG00000138735	PDE5A	protein_coding	2.418173421	1.00E-11	4.15E-11	up
ENSG00000112238	PRDM13	protein_coding	1.546127644	1.01E-11	4.20E-11	up
ENSG00000066248	NGEF	protein_coding	1.518507152	1.02E-11	4.21E-11	up
ENSG00000163704	PRRT3	protein_coding	-3.519460698	1.03E-11	4.24E-11	down
ENSG00000198546	ZNF511	protein_coding	-1.773001818	1.09E-11	4.49E-11	down
ENSG00000166436	TRIM66	protein_coding	1.360776545	1.10E-11	4.52E-11	up
ENSG00000171311	EXOSC1	protein_coding	-1.150557657	1.10E-11	4.55E-11	down
ENSG00000157399	ARSE	protein_coding	-1.127346343	1.18E-11	4.84E-11	down
ENSG00000229809	ZNF688	protein_coding	-4.097728546	1.19E-11	4.88E-11	down
ENSG00000100300	TSPO	protein_coding	-1.264988306	1.19E-11	4.91E-11	down
ENSG00000105556	MIER2	protein_coding	-1.202270522	1.20E-11	4.95E-11	down
ENSG00000111785	RIC8B	protein_coding	-1.054237442	1.22E-11	5.00E-11	down
ENSG00000258429	PDF	protein_coding	-3.315319981	1.24E-11	5.08E-11	down
ENSG00000142149	HUNK	protein_coding	1.05220498	1.30E-11	5.36E-11	up

ENSG00000161270	NPHS1	protein_coding	-1.826742017	1.33E-11	5.47E-11	down
ENSG00000196639	HRH1	protein_coding	-2.436998538	1.35E-11	5.53E-11	down
ENSG00000151500	THYN1	protein_coding	1.054775396	1.41E-11	5.80E-11	up
ENSG00000154710	RABGEF1	protein_coding	-6.541828511	1.47E-11	6.00E-11	down
ENSG00000167578	RAB4B	protein_coding	-6.541828511	1.47E-11	6.00E-11	down
ENSG00000010803	SCMH1	protein_coding	1.088402205	1.50E-11	6.12E-11	up
ENSG00000140025	EFCAB11	protein_coding	-1.312279527	1.52E-11	6.19E-11	down
ENSG00000007541	PIGQ	protein_coding	-2.555986574	1.57E-11	6.39E-11	down
ENSG00000116667	C1orf21	protein_coding	1.073881468	1.58E-11	6.44E-11	up
ENSG00000113924	HGD	protein_coding	2.400685994	1.62E-11	6.59E-11	up
ENSG00000139626	ITGB7	protein_coding	5.267135664	1.62E-11	6.59E-11	up
ENSG00000150054	MPP7	protein_coding	5.267135664	1.62E-11	6.59E-11	up
ENSG00000213930	GALT	protein_coding	-2.625660102	1.65E-11	6.68E-11	down
ENSG00000228696	ARL17B	protein_coding	4.358283552	1.65E-11	6.69E-11	up
ENSG00000171303	KCNK3	protein_coding	-2.893856213	1.65E-11	6.71E-11	down
ENSG00000176428	VPS37D	protein_coding	2.179672823	1.68E-11	6.81E-11	up
ENSG00000147912	FBXO10	protein_coding	-4.578354387	1.76E-11	7.14E-11	down
ENSG00000131480	AOC2	protein_coding	-4.578354387	1.76E-11	7.14E-11	down
ENSG00000163516	ANKZF1	protein_coding	1.036620683	1.94E-11	7.83E-11	up
ENSG00000196208	GREB1	protein_coding	-1.521770859	1.94E-11	7.84E-11	down
ENSG00000105516	DBP	protein_coding	1.2276073	1.99E-11	8.01E-11	up
ENSG00000129911	KLF16	protein_coding	-1.313009821	2.00E-11	8.05E-11	down
ENSG00000100577	GSTZ1	protein_coding	-1.408429386	2.04E-11	8.23E-11	down
ENSG00000116954	RRAGC	protein_coding	-1.63751646	2.10E-11	8.44E-11	down
ENSG00000121716	PILRB	protein_coding	-1.486293834	2.10E-11	8.45E-11	down
ENSG00000171130	ATP6V0E2	protein_coding	-1.180075406	2.11E-11	8.48E-11	down
ENSG00000126561	STAT5A	protein_coding	2.328536208	2.13E-11	8.58E-11	up
ENSG00000163251	FZD5	protein_coding	1.085760642	2.16E-11	8.69E-11	up
ENSG00000146386	ABRACL	protein_coding	-1.02954089	2.20E-11	8.85E-11	down
ENSG00000080561	MID2	protein_coding	1.284142089	2.22E-11	8.90E-11	up
ENSG00000163235	TGFA	protein_coding	-1.256426292	2.31E-11	9.26E-11	down
ENSG00000005882	PDK2	protein_coding	-1.058826332	2.37E-11	9.52E-11	down
ENSG00000080031	PTPRH	protein_coding	1.380066509	2.48E-11	9.93E-11	up
ENSG00000166595	FAM96B	protein_coding	-1.186416571	2.52E-11	1.01E-10	down
ENSG00000095596	CYP26A1	protein_coding	6.235426804	2.57E-11	1.03E-10	up
ENSG00000081479	LRP2	protein_coding	6.235426804	2.57E-11	1.03E-10	up
ENSG00000187824	TMEM220	protein_coding	6.235426804	2.57E-11	1.03E-10	up
ENSG00000158023	WDR66	protein_coding	-6.504353806	2.58E-11	1.03E-10	down
ENSG00000125462	C1orf61	protein_coding	-6.504353806	2.58E-11	1.03E-10	down
ENSG00000243279	PRAF2	protein_coding	-6.504353806	2.58E-11	1.03E-10	down
ENSG00000267508	ZNF285	protein_coding	-6.504353806	2.58E-11	1.03E-10	down
ENSG00000225663	MCRIP1	protein_coding	-1.439872433	2.60E-11	1.03E-10	down
ENSG00000131080	EDA2R	protein_coding	-1.140949075	2.73E-11	1.08E-10	down
ENSG00000253846	PCDHGA10	protein_coding	5.235426804	2.75E-11	1.09E-10	up
ENSG00000181444	ZNF467	protein_coding	1.934257269	2.76E-11	1.09E-10	up
ENSG00000213123	TCTEX1D2	protein_coding	4.328536208	2.85E-11	1.13E-10	up
ENSG00000173013	CCDC96	protein_coding	4.328536208	2.85E-11	1.13E-10	up
ENSG00000235750	KIAA0040	protein_coding	4.328536208	2.85E-11	1.13E-10	up
ENSG00000088881	EBF4	protein_coding	4.328536208	2.85E-11	1.13E-10	up
ENSG00000163126	ANKRD23	protein_coding	1.610935939	2.91E-11	1.15E-10	up
ENSG00000122335	SERAC1	protein_coding	1.049076255	2.91E-11	1.15E-10	up
ENSG00000159713	TPPP3	protein_coding	2.702931723	2.93E-11	1.16E-10	up
ENSG00000116106	EPHA4	protein_coding	3.279626608	3.00E-11	1.19E-10	up
ENSG00000103044	HAS3	protein_coding	-1.510182885	3.01E-11	1.19E-10	down

ENSG00000184702	5-Sep	protein_coding	1.105281705	3.41E-11	1.34E-10	up
ENSG00000002587	HS3ST1	protein_coding	-5.465879658	3.58E-11	1.41E-10	down
ENSG00000151468	CCDC3	protein_coding	-5.465879658	3.58E-11	1.41E-10	down
ENSG00000155755	TMEM237	protein_coding	-1.17842378	3.64E-11	1.43E-10	down
ENSG00000168350	DEGS2	protein_coding	3.831036549	3.67E-11	1.44E-10	up
ENSG00000006042	TMEM98	protein_coding	-2.343889134	3.82E-11	1.50E-10	down
ENSG00000143409	MINDY1	protein_coding	-2.054792431	3.87E-11	1.51E-10	down
ENSG00000135540	NHSL1	protein_coding	-2.054792431	3.87E-11	1.51E-10	down
ENSG00000130714	POMT1	protein_coding	-1.214847189	3.90E-11	1.53E-10	down
ENSG00000114745	GORASP1	protein_coding	1.133520226	3.94E-11	1.54E-10	up
ENSG00000188092	GPR89B	protein_coding	1.808865168	4.05E-11	1.59E-10	up
ENSG00000169429	CXCL8	protein_coding	-1.373609832	4.10E-11	1.60E-10	down
ENSG00000001460	STPG1	protein_coding	1.432644612	4.12E-11	1.61E-10	up
ENSG00000183773	AIFM3	protein_coding	6.203005326	4.18E-11	1.63E-10	up
ENSG00000123999	INHA	protein_coding	6.203005326	4.18E-11	1.63E-10	up
ENSG00000127366	TAS2R5	protein_coding	6.203005326	4.18E-11	1.63E-10	up
ENSG00000011143	MKS1	protein_coding	-1.632293194	4.29E-11	1.67E-10	down
ENSG00000130962	PRRG1	protein_coding	-1.206957616	4.31E-11	1.68E-10	down
ENSG00000163689	C3orf67	protein_coding	2.591570614	4.32E-11	1.68E-10	up
ENSG00000284691	AC073111.5	protein_coding	-4.029015796	4.42E-11	1.72E-10	down
ENSG00000136943	CTSV	protein_coding	1.55092863	4.43E-11	1.72E-10	up
ENSG00000135205	CCDC146	protein_coding	1.535768823	4.45E-11	1.73E-10	up
ENSG00000146426	TIAM2	protein_coding	-3.256426292	4.46E-11	1.73E-10	down
ENSG00000167619	TMEM145	protein_coding	-6.465879658	4.53E-11	1.76E-10	down
ENSG00000162975	KCNF1	protein_coding	-6.465879658	4.53E-11	1.76E-10	down
ENSG00000004777	ARHGAP33	protein_coding	-6.465879658	4.53E-11	1.76E-10	down
ENSG00000111837	MAK	protein_coding	-6.465879658	4.53E-11	1.76E-10	down
ENSG00000225362	CT62	protein_coding	5.203005326	4.68E-11	1.81E-10	up
ENSG00000283486	FAM95C	protein_coding	1.36309343	4.93E-11	1.91E-10	up
ENSG00000130413	STK33	protein_coding	-2.510182885	5.09E-11	1.97E-10	down
ENSG00000079150	FKBP7	protein_coding	-2.23624841	5.16E-11	1.99E-10	down
ENSG00000105699	LSR	protein_coding	-1.191575148	5.23E-11	2.02E-10	down
ENSG00000181061	HIGD1A	protein_coding	-1.32681562	5.28E-11	2.04E-10	down
ENSG00000204228	HSD17B8	protein_coding	-1.874178728	5.37E-11	2.07E-10	down
ENSG00000146530	VWDE	protein_coding	-1.108869104	5.37E-11	2.07E-10	down
ENSG00000168826	ZBTB49	protein_coding	-2.578354387	5.41E-11	2.09E-10	down
ENSG00000186665	C17orf58	protein_coding	-2.011313794	5.43E-11	2.09E-10	down
ENSG00000111801	BTN3A3	protein_coding	-2.112036383	5.53E-11	2.13E-10	down
ENSG00000138629	UBL7	protein_coding	-1.087299021	5.56E-11	2.14E-10	down
ENSG00000158321	AUTS2	protein_coding	-1.143951563	5.68E-11	2.18E-10	down
ENSG00000130021	PUDP	protein_coding	-1.156359337	6.28E-11	2.41E-10	down
ENSG00000123353	ORMDL2	protein_coding	1.01749643	6.34E-11	2.43E-10	up
ENSG00000161395	PGAP3	protein_coding	1.483814434	6.36E-11	2.44E-10	up
ENSG00000182700	IGIP	protein_coding	-4.504353806	6.52E-11	2.50E-10	down
ENSG00000215183	MSMP	protein_coding	-4.504353806	6.52E-11	2.50E-10	down
ENSG00000165887	ANKRD2	protein_coding	-4.504353806	6.52E-11	2.50E-10	down
ENSG00000096093	EFHC1	protein_coding	1.345609722	6.69E-11	2.56E-10	up
ENSG00000143278	F13B	protein_coding	6.169838462	6.82E-11	2.60E-10	up
ENSG00000123096	SSPN	protein_coding	6.169838462	6.82E-11	2.60E-10	up
ENSG00000179104	TMTC2	protein_coding	-1.256426292	6.94E-11	2.65E-10	down
ENSG00000099338	CATSPERG	protein_coding	2.571392732	7.10E-11	2.71E-10	up
ENSG00000172830	SSH3	protein_coding	1.083802619	7.54E-11	2.87E-10	up
ENSG00000142556	ZNF614	protein_coding	-1.295420424	7.63E-11	2.90E-10	down
ENSG00000120738	EGR1	protein_coding	-6.426351294	8.00E-11	3.03E-10	down

ENSG00000178404	CEP295NL	protein_coding	-6.426351294	8.00E-11	3.03E-10	down
ENSG00000090339	ICAM1	protein_coding	-6.426351294	8.00E-11	3.03E-10	down
ENSG00000143590	EFNA3	protein_coding	-6.426351294	8.00E-11	3.03E-10	down
ENSG00000113070	HBEGF	protein_coding	-6.426351294	8.00E-11	3.03E-10	down
ENSG00000160233	LRRC3	protein_coding	-6.426351294	8.00E-11	3.03E-10	down
ENSG00000257950	P2RX5-TAX1B	protein_coding	-6.426351294	8.00E-11	3.03E-10	down
ENSG00000184731	FAM110C	protein_coding	-6.426351294	8.00E-11	3.03E-10	down
ENSG00000153029	MR1	protein_coding	1.087057946	8.15E-11	3.08E-10	up
ENSG00000177042	TMEM80	protein_coding	2.40653872	8.32E-11	3.14E-10	up
ENSG00000197329	PELI1	protein_coding	-2.426351294	8.38E-11	3.16E-10	down
ENSG00000082146	STRADB	protein_coding	-1.055095486	8.50E-11	3.21E-10	down
ENSG00000167861	HID1	protein_coding	1.425397748	8.54E-11	3.22E-10	up
ENSG00000100890	KIAA0391	protein_coding	-1.529444787	8.68E-11	3.27E-10	down
ENSG00000115286	NDUFS7	protein_coding	1.110690576	8.89E-11	3.35E-10	up
ENSG00000226742	HSBP1L1	protein_coding	-1.148366546	9.00E-11	3.39E-10	down
ENSG00000182389	CACNB4	protein_coding	3.041254256	9.27E-11	3.48E-10	up
ENSG00000139910	NOVA1	protein_coding	-1.810024622	9.28E-11	3.49E-10	down
ENSG00000163900	TMEM41A	protein_coding	-1.044922187	9.72E-11	3.65E-10	down
ENSG00000181873	IBA57	protein_coding	-1.605575856	1.08E-10	4.03E-10	down
ENSG00000275895	U2AF1L5	protein_coding	-3.648743715	1.11E-10	4.15E-10	down
ENSG00000167646	DNAAF3	protein_coding	6.13589113	1.11E-10	4.16E-10	up
ENSG00000169894	MUC3A	protein_coding	6.13589113	1.11E-10	4.16E-10	up
ENSG00000198354	DCAF12L2	protein_coding	6.13589113	1.11E-10	4.16E-10	up
ENSG00000086619	ERO1B	protein_coding	-1.285770254	1.13E-10	4.21E-10	down
ENSG00000135124	P2RX4	protein_coding	-1.212837024	1.17E-10	4.36E-10	down
ENSG00000182870	GALNT9	protein_coding	-1.666710262	1.18E-10	4.38E-10	down
ENSG00000070182	SPTB	protein_coding	-1.189312096	1.19E-10	4.44E-10	down
ENSG00000153975	ZUFSP	protein_coding	-1.738819059	1.21E-10	4.52E-10	down
ENSG00000145088	EAF2	protein_coding	1.951169127	1.22E-10	4.52E-10	up
ENSG00000163734	CXCL3	protein_coding	-1.558989062	1.22E-10	4.55E-10	down
ENSG00000214114	MYCBP	protein_coding	-1.558989062	1.22E-10	4.55E-10	down
ENSG00000182795	C1orf116	protein_coding	1.641694094	1.23E-10	4.58E-10	up
ENSG00000261794	GOLGA8H	protein_coding	-1.893856213	1.38E-10	5.10E-10	down
ENSG00000091583	APOH	protein_coding	1.770045919	1.39E-10	5.14E-10	up
ENSG00000176410	DNAJC30	protein_coding	-1.342582936	1.39E-10	5.15E-10	down
ENSG00000186326	RGS9BP	protein_coding	-6.385709309	1.42E-10	5.23E-10	down
ENSG00000135838	NPL	protein_coding	-6.385709309	1.42E-10	5.23E-10	down
ENSG00000160886	LY6K	protein_coding	-6.385709309	1.42E-10	5.23E-10	down
ENSG00000186222	BLOC1S4	protein_coding	-1.676329547	1.51E-10	5.56E-10	down
ENSG00000106268	NUDT1	protein_coding	-1.028157305	1.54E-10	5.68E-10	down
ENSG00000114771	AADAC	protein_coding	-1.751190984	1.54E-10	5.69E-10	down
ENSG00000243667	WDR92	protein_coding	-1.97524454	1.54E-10	5.69E-10	down
ENSG00000157379	DHRS1	protein_coding	-2.195025748	1.56E-10	5.74E-10	down
ENSG00000189298	ZKSCAN3	protein_coding	-2.195025748	1.56E-10	5.74E-10	down
ENSG00000169288	MRPL1	protein_coding	-1.330973246	1.58E-10	5.83E-10	down
ENSG00000180815	MAP3K15	protein_coding	-2.073562235	1.62E-10	5.96E-10	down
ENSG00000168661	ZNF30	protein_coding	2.099054362	1.63E-10	5.99E-10	up
ENSG00000165512	ZNF22	protein_coding	1.845111734	1.67E-10	6.12E-10	up
ENSG00000048052	HDAC9	protein_coding	-1.522016403	1.76E-10	6.45E-10	down
ENSG00000177352	CCDC71	protein_coding	1.177976532	1.76E-10	6.46E-10	up
ENSG00000101298	SNPH	protein_coding	-1.074979421	1.76E-10	6.46E-10	down
ENSG00000123992	DNPEP	protein_coding	-1.247027594	1.77E-10	6.48E-10	down
ENSG00000104432	IL7	protein_coding	6.101125712	1.82E-10	6.67E-10	up
ENSG00000128714	HOXD13	protein_coding	6.101125712	1.82E-10	6.67E-10	up

ENSG00000162738	VANGL2	protein_coding	6.101125712	1.82E-10	6.67E-10	up
ENSG00000117971	CHRNB4	protein_coding	6.101125712	1.82E-10	6.67E-10	up
ENSG00000162148	PPP1R32	protein_coding	6.101125712	1.82E-10	6.67E-10	up
ENSG00000170873	MTSS1	protein_coding	6.101125712	1.82E-10	6.67E-10	up
ENSG00000197372	ZNF675	protein_coding	-1.398445297	1.83E-10	6.68E-10	down
ENSG00000171763	SPATA5L1	protein_coding	-1.370083074	1.85E-10	6.74E-10	down
ENSG00000131668	BARX1	protein_coding	-2.604349596	1.87E-10	6.82E-10	down
ENSG00000166405	RIC3	protein_coding	-2.900282482	1.89E-10	6.90E-10	down
ENSG00000145882	PCYOX1L	protein_coding	1.467056073	1.91E-10	6.94E-10	up
ENSG00000186496	ZNF396	protein_coding	2.53017007	1.91E-10	6.96E-10	up
ENSG00000204438	GPANK1	protein_coding	-1.723552303	1.96E-10	7.14E-10	down
ENSG00000197302	ZNF720	protein_coding	-1.237810614	1.97E-10	7.15E-10	down
ENSG00000135736	CCDC102A	protein_coding	-1.447369075	2.01E-10	7.30E-10	down
ENSG00000103152	MPG	protein_coding	-1.144780936	2.05E-10	7.43E-10	down
ENSG00000170917	NUDT6	protein_coding	-1.357512417	2.11E-10	7.67E-10	down
ENSG00000217930	PAM16	protein_coding	-3.613978297	2.13E-10	7.75E-10	down
ENSG00000126970	ZC4H2	protein_coding	1.356016945	2.15E-10	7.80E-10	up
ENSG00000189319	FAM53B	protein_coding	-1.058299149	2.18E-10	7.89E-10	down
ENSG00000171368	TPPP	protein_coding	1.730634652	2.21E-10	8.01E-10	up
ENSG00000173598	NUDT4	protein_coding	-1.000086539	2.22E-10	8.02E-10	down
ENSG00000130810	PPAN	protein_coding	3.41599905	2.30E-10	8.31E-10	up
ENSG00000176422	SPRYD4	protein_coding	-1.073204468	2.31E-10	8.35E-10	down
ENSG00000088543	C3orf18	protein_coding	-6.343889134	2.51E-10	9.06E-10	down
ENSG00000171435	KSR2	protein_coding	-6.343889134	2.51E-10	9.06E-10	down
ENSG00000204822	MRPL53	protein_coding	-6.343889134	2.51E-10	9.06E-10	down
ENSG00000131409	LRRC4B	protein_coding	-6.343889134	2.51E-10	9.06E-10	down
ENSG00000204316	MRPL38	protein_coding	-6.343889134	2.51E-10	9.06E-10	down
ENSG00000166126	AMN	protein_coding	1.831036549	2.53E-10	9.11E-10	up
ENSG00000138794	CASP6	protein_coding	1.175800968	2.56E-10	9.21E-10	up
ENSG00000158055	GRHL3	protein_coding	4.203005326	2.58E-10	9.29E-10	up
ENSG00000178229	ZNF543	protein_coding	-1.143532236	2.69E-10	9.65E-10	down
ENSG00000178127	NDUFV2	protein_coding	-2.173964132	2.70E-10	9.69E-10	down
ENSG00000113209	PCDHB5	protein_coding	1.800157236	2.74E-10	9.82E-10	up
ENSG00000103245	NARFL	protein_coding	-1.099700789	2.74E-10	9.83E-10	down
ENSG00000140682	TGFB1I1	protein_coding	1.019515597	2.83E-10	1.01E-09	up
ENSG00000130270	ATP8B3	protein_coding	1.085041989	2.85E-10	1.02E-09	up
ENSG00000254901	BORCS8	protein_coding	1.337581348	2.91E-10	1.04E-09	up
ENSG00000158480	SPATA2	protein_coding	-1.050957174	3.05E-10	1.08E-09	down
ENSG00000153551	CMTM7	protein_coding	-1.246776122	3.07E-10	1.09E-09	down
ENSG00000121989	ACVR2A	protein_coding	1.191719519	3.11E-10	1.11E-09	up
ENSG00000164039	BDH2	protein_coding	-3.919391305	3.19E-10	1.13E-09	down
ENSG00000106479	ZNF862	protein_coding	1.717578499	3.30E-10	1.17E-09	up
ENSG00000278384	AL354822.1	protein_coding	3.713200059	3.30E-10	1.17E-09	up
ENSG00000008441	NFIX	protein_coding	-1.237317469	3.41E-10	1.21E-09	down
ENSG00000105607	GCDH	protein_coding	-1.021961039	3.43E-10	1.22E-09	down
ENSG00000172432	GTPBP2	protein_coding	-1.122674903	3.45E-10	1.23E-09	down
ENSG00000181544	FANCB	protein_coding	-1.122674903	3.45E-10	1.23E-09	down
ENSG00000256537	SMIM10L1	protein_coding	-1.040292861	3.48E-10	1.23E-09	down
ENSG00000160746	ANO10	protein_coding	-1.040292861	3.48E-10	1.23E-09	down
ENSG00000158104	HPD	protein_coding	1.849368372	3.52E-10	1.25E-09	up
ENSG00000198934	MAGEE1	protein_coding	-1.614880263	3.68E-10	1.30E-09	down
ENSG00000188818	ZDHHC11	protein_coding	-1.474606462	3.83E-10	1.36E-09	down
ENSG00000113522	RAD50	protein_coding	-1.201978508	3.89E-10	1.37E-09	down
ENSG00000107738	VSIR	protein_coding	1.151379638	4.11E-10	1.45E-09	up

ENSG00000198105	ZNF248	protein_coding	1.046965851	4.15E-10	1.46E-09	up
ENSG00000182362	YBEY	protein_coding	-1.063781214	4.25E-10	1.50E-09	down
ENSG00000119737	GPR75	protein_coding	-6.300820412	4.48E-10	1.57E-09	down
ENSG00000183801	OLFML1	protein_coding	-6.300820412	4.48E-10	1.57E-09	down
ENSG00000236609	ZNF853	protein_coding	-6.300820412	4.48E-10	1.57E-09	down
ENSG00000122477	LRRC39	protein_coding	-6.300820412	4.48E-10	1.57E-09	down
ENSG00000214290	COLCA2	protein_coding	-6.300820412	4.48E-10	1.57E-09	down
ENSG00000214534	ZNF705E	protein_coding	-6.300820412	4.48E-10	1.57E-09	down
ENSG00000111012	CYP27B1	protein_coding	-6.300820412	4.48E-10	1.57E-09	down
ENSG00000282936	AC004706.4	protein_coding	-6.300820412	4.48E-10	1.57E-09	down
ENSG00000213347	MXD3	protein_coding	-1.058097576	4.56E-10	1.60E-09	down
ENSG00000148426	PROSER2	protein_coding	1.357105361	4.58E-10	1.60E-09	up
ENSG00000182141	ZNF708	protein_coding	6.028975927	4.91E-10	1.72E-09	up
ENSG00000169760	NLGN1	protein_coding	6.028975927	4.91E-10	1.72E-09	up
ENSG00000125409	TEKT3	protein_coding	6.028975927	4.91E-10	1.72E-09	up
ENSG00000178919	FOXE1	protein_coding	-1.656356899	4.95E-10	1.73E-09	down
ENSG00000137691	C11orf70	protein_coding	1.423693441	5.46E-10	1.90E-09	up
ENSG00000154889	MPPE1	protein_coding	1.040733897	5.49E-10	1.91E-09	up
ENSG00000186272	ZNF17	protein_coding	-2.478818714	5.72E-10	1.99E-09	down
ENSG00000169105	CHST14	protein_coding	1.176533115	5.76E-10	2.00E-09	up
ENSG00000148848	ADAM12	protein_coding	-1.393929816	6.29E-10	2.18E-09	down
ENSG00000064687	ABCA7	protein_coding	1.082375621	6.40E-10	2.22E-09	up
ENSG00000128510	CPA4	protein_coding	-1.79076272	6.48E-10	2.24E-09	down
ENSG00000162999	DUSP19	protein_coding	-1.79076272	6.48E-10	2.24E-09	down
ENSG00000172985	SH3RF3	protein_coding	-2.841388793	6.53E-10	2.26E-09	down
ENSG00000155542	SETD9	protein_coding	-2.841388793	6.53E-10	2.26E-09	down
ENSG00000143919	CAMKMT	protein_coding	-1.745173478	6.56E-10	2.27E-09	down
ENSG00000159423	ALDH4A1	protein_coding	2.674311045	6.71E-10	2.32E-09	up
ENSG00000143942	CHAC2	protein_coding	-1.200573058	6.74E-10	2.33E-09	down
ENSG00000156959	LHFPL4	protein_coding	3.358283552	6.79E-10	2.34E-09	up
ENSG00000162227	TAF6L	protein_coding	-1.445460117	6.92E-10	2.39E-09	down
ENSG00000009950	MLXIPL	protein_coding	1.71660666	7.00E-10	2.41E-09	up
ENSG00000165060	FXN	protein_coding	-1.125181759	7.22E-10	2.49E-09	down
ENSG00000223573	TINCR	protein_coding	2.794199781	7.29E-10	2.51E-09	up
ENSG00000180448	ARHGAP45	protein_coding	3.122085331	7.42E-10	2.55E-09	up
ENSG00000164002	EXO5	protein_coding	2.939970921	7.57E-10	2.60E-09	up
ENSG00000182324	KCNJ14	protein_coding	-1.859767322	7.82E-10	2.68E-09	down
ENSG00000170270	GON7	protein_coding	-1.641090143	7.90E-10	2.71E-09	down
ENSG00000188321	ZNF559	protein_coding	-6.256426292	8.00E-10	2.74E-09	down
ENSG00000100206	DMC1	protein_coding	-6.256426292	8.00E-10	2.74E-09	down
ENSG00000183018	SPNS2	protein_coding	-6.256426292	8.00E-10	2.74E-09	down
ENSG00000137491	SLCO2B1	protein_coding	-6.256426292	8.00E-10	2.74E-09	down
ENSG00000137460	FHDC1	protein_coding	-6.256426292	8.00E-10	2.74E-09	down
ENSG00000166278	C2	protein_coding	-6.256426292	8.00E-10	2.74E-09	down
ENSG00000112149	CD83	protein_coding	-6.256426292	8.00E-10	2.74E-09	down
ENSG00000155324	GRAMD2B	protein_coding	-6.256426292	8.00E-10	2.74E-09	down
ENSG00000165810	BTNL9	protein_coding	-6.256426292	8.00E-10	2.74E-09	down
ENSG00000137821	LRRC49	protein_coding	-1.138244866	8.04E-10	2.75E-09	down
ENSG00000168843	FSTL5	protein_coding	5.991501221	8.09E-10	2.76E-09	up
ENSG00000145147	SLIT2	protein_coding	5.991501221	8.09E-10	2.76E-09	up
ENSG00000170236	USP50	protein_coding	5.991501221	8.09E-10	2.76E-09	up
ENSG00000116254	CHD5	protein_coding	1.438719126	8.13E-10	2.77E-09	up
ENSG00000104497	SNX16	protein_coding	1.253248081	8.20E-10	2.79E-09	up
ENSG00000171970	ZNF57	protein_coding	-5.256426292	8.27E-10	2.81E-09	down

ENSG00000037042	TUBG2	protein_coding	1.217504896	8.97E-10	3.04E-09	up
ENSG00000160963	COL26A1	protein_coding	-4.343889134	9.01E-10	3.06E-09	down
ENSG00000181450	ZNF678	protein_coding	-1.413967569	9.38E-10	3.18E-09	down
ENSG00000226763	SRRM5	protein_coding	-2.168963451	9.50E-10	3.22E-09	down
ENSG00000139405	RITA1	protein_coding	-1.037309003	9.53E-10	3.23E-09	down
ENSG00000135596	MICAL1	protein_coding	1.358283552	9.74E-10	3.29E-09	up
ENSG00000168026	TTC21A	protein_coding	3.650464303	9.91E-10	3.35E-09	up
ENSG00000214954	LRRC69	protein_coding	3.650464303	9.91E-10	3.35E-09	up
ENSG00000168916	ZNF608	protein_coding	1.729634517	9.93E-10	3.35E-09	up
ENSG00000198951	NAGA	protein_coding	1.065501803	1.05E-09	3.54E-09	up
ENSG00000108176	DNAJC12	protein_coding	1.161185151	1.06E-09	3.58E-09	up
ENSG00000254470	AP5B1	protein_coding	-1.046340766	1.09E-09	3.68E-09	down
ENSG00000187193	MT1X	protein_coding	-1.563854817	1.11E-09	3.74E-09	down
ENSG00000198062	POTEH	protein_coding	4.991501221	1.16E-09	3.89E-09	up
ENSG00000062282	DGAT2	protein_coding	-2.940924467	1.17E-09	3.92E-09	down
ENSG00000135045	C9orf40	protein_coding	-1.59346128	1.19E-09	3.99E-09	down
ENSG00000144681	STAC	protein_coding	-2.811015144	1.21E-09	4.05E-09	down
ENSG00000130675	MNX1	protein_coding	1.533153928	1.25E-09	4.18E-09	up
ENSG00000138738	PRDM5	protein_coding	-1.055832461	1.25E-09	4.19E-09	down
ENSG00000123095	BHLHE41	protein_coding	-2.256426292	1.28E-09	4.29E-09	down
ENSG00000146409	SLC18B1	protein_coding	-1.165660828	1.29E-09	4.32E-09	down
ENSG00000082196	C1QTNF3	protein_coding	5.953027073	1.33E-09	4.45E-09	up
ENSG00000198125	MB	protein_coding	-1.788921373	1.34E-09	4.46E-09	down
ENSG00000197562	RAB40C	protein_coding	-1.23596219	1.36E-09	4.51E-09	down
ENSG00000149927	DOC2A	protein_coding	2.444013426	1.36E-09	4.52E-09	up
ENSG00000170458	CD14	protein_coding	2.444013426	1.36E-09	4.52E-09	up
ENSG00000168672	FAM84B	protein_coding	1.122085331	1.36E-09	4.53E-09	up
ENSG00000143412	ANXA9	protein_coding	-6.210622603	1.43E-09	4.75E-09	down
ENSG00000162591	MEGF6	protein_coding	-6.210622603	1.43E-09	4.75E-09	down
ENSG00000137561	TTPA	protein_coding	-6.210622603	1.43E-09	4.75E-09	down
ENSG00000102935	ZNF423	protein_coding	-6.210622603	1.43E-09	4.75E-09	down
ENSG00000167220	HDHD2	protein_coding	1.715559332	1.48E-09	4.91E-09	up
ENSG00000171227	TMEM37	protein_coding	1.23597009	1.56E-09	5.14E-09	up
ENSG00000112139	MDGA1	protein_coding	-5.210622603	1.56E-09	5.15E-09	down
ENSG00000119574	ZBTB45	protein_coding	-1.302229982	1.58E-09	5.21E-09	down
ENSG00000167772	ANGPTL4	protein_coding	1.66413924	1.62E-09	5.33E-09	up
ENSG00000137501	SYTL2	protein_coding	-1.076344676	1.62E-09	5.35E-09	down
ENSG00000168517	HEXIM2	protein_coding	-1.120542864	1.64E-09	5.40E-09	down
ENSG00000135605	TEC	protein_coding	-1.355961966	1.65E-09	5.43E-09	down
ENSG00000177427	MIEF2	protein_coding	-2.363341496	1.65E-09	5.44E-09	down
ENSG00000276045	ORAI1	protein_coding	1.309553105	1.70E-09	5.57E-09	up
ENSG00000175906	ARL4D	protein_coding	-4.300820412	1.74E-09	5.73E-09	down
ENSG00000198089	SFI1	protein_coding	1.169337614	1.77E-09	5.80E-09	up
ENSG00000151655	ITIH2	protein_coding	1.576463722	1.78E-09	5.84E-09	up
ENSG00000132394	EEFSEC	protein_coding	1.557160583	1.80E-09	5.90E-09	up
ENSG00000150667	FSIP1	protein_coding	-3.256426292	1.81E-09	5.94E-09	down
ENSG00000198848	CES1	protein_coding	-3.256426292	1.81E-09	5.94E-09	down
ENSG00000153814	JAZF1	protein_coding	1.78986736	1.86E-09	6.09E-09	up
ENSG00000181894	ZNF329	protein_coding	-2.18716363	1.93E-09	6.32E-09	down
ENSG00000132274	TRIM22	protein_coding	-2.18716363	1.93E-09	6.32E-09	down
ENSG00000169752	NRG4	protein_coding	2.047354456	2.03E-09	6.64E-09	up
ENSG00000089639	GMIP	protein_coding	-1.682691047	2.16E-09	7.04E-09	down
ENSG00000283930	AL117339.5	protein_coding	5.913498709	2.21E-09	7.18E-09	up
ENSG00000198723	TEX45	protein_coding	5.913498709	2.21E-09	7.18E-09	up

ENSG00000184271	POU6F1	protein_coding	5.913498709	2.21E-09	7.18E-09	up
ENSG00000160999	SH2B2	protein_coding	2.421645613	2.21E-09	7.18E-09	up
ENSG00000205423	CNEP1R1	protein_coding	-1.140233275	2.21E-09	7.18E-09	down
ENSG00000240891	PLCXD2	protein_coding	1.299967056	2.36E-09	7.67E-09	up
ENSG00000186462	NAP1L2	protein_coding	1.083649149	2.42E-09	7.86E-09	up
ENSG00000095397	WHRN	protein_coding	1.432872868	2.46E-09	7.97E-09	up
ENSG00000262209	PCDHGB3	protein_coding	-6.163316888	2.57E-09	8.33E-09	down
ENSG00000095752	IL11	protein_coding	-6.163316888	2.57E-09	8.33E-09	down
ENSG00000140022	STON2	protein_coding	-6.163316888	2.57E-09	8.33E-09	down
ENSG00000178623	GPR35	protein_coding	-6.163316888	2.57E-09	8.33E-09	down
ENSG00000213906	LTB4R2	protein_coding	-6.163316888	2.57E-09	8.33E-09	down
ENSG00000197748	CFAP43	protein_coding	-6.163316888	2.57E-09	8.33E-09	down
ENSG00000103647	CORO2B	protein_coding	-6.163316888	2.57E-09	8.33E-09	down
ENSG00000165046	LETM2	protein_coding	-6.163316888	2.57E-09	8.33E-09	down
ENSG00000250644	AC068580.4	protein_coding	1.318688422	2.59E-09	8.35E-09	up
ENSG00000109255	NMU	protein_coding	1.492511944	2.61E-09	8.41E-09	up
ENSG00000172167	MTBP	protein_coding	-1.196720046	2.68E-09	8.63E-09	down
ENSG00000175104	TRAF6	protein_coding	-1.303732007	2.74E-09	8.84E-09	down
ENSG00000119865	CNRIP1	protein_coding	-3.465879658	2.91E-09	9.37E-09	down
ENSG00000175193	PARL	protein_coding	-5.163316888	2.95E-09	9.49E-09	down
ENSG00000183971	NPW	protein_coding	3.584875962	2.97E-09	9.56E-09	up
ENSG00000204977	TRIM13	protein_coding	-1.109877629	3.06E-09	9.85E-09	down
ENSG00000226784	PGAM4	protein_coding	1.714427362	3.15E-09	1.01E-08	up
ENSG00000127586	CHTF18	protein_coding	-1.153666718	3.22E-09	1.03E-08	down
ENSG00000174456	C12orf76	protein_coding	1.383398143	3.24E-09	1.04E-08	up
ENSG00000118369	USP35	protein_coding	1.290316886	3.29E-09	1.06E-08	up
ENSG00000267796	LIN37	protein_coding	-4.256426292	3.37E-09	1.08E-08	down
ENSG00000083750	RRAGB	protein_coding	1.129990529	3.55E-09	1.14E-08	up
ENSG00000065717	TLE2	protein_coding	1.978038961	3.65E-09	1.17E-08	up
ENSG00000206561	COLQ	protein_coding	5.872856725	3.66E-09	1.17E-08	up
ENSG00000166828	SCNN1G	protein_coding	5.872856725	3.66E-09	1.17E-08	up
ENSG00000170909	OSCAR	protein_coding	5.872856725	3.66E-09	1.17E-08	up
ENSG00000135709	KIAA0513	protein_coding	1.061935188	3.67E-09	1.17E-08	up
ENSG00000100307	CBX7	protein_coding	1.203005326	3.79E-09	1.21E-08	up
ENSG00000137496	IL18BP	protein_coding	-1.951571711	3.82E-09	1.22E-08	down
ENSG00000204237	OXLD1	protein_coding	-1.088481655	3.86E-09	1.23E-08	down
ENSG00000146054	TRIM7	protein_coding	-1.451073723	4.11E-09	1.30E-08	down
ENSG00000206535	LNP1	protein_coding	-1.638296928	4.30E-09	1.36E-08	down
ENSG00000118655	DCLRE1B	protein_coding	-1.378416817	4.31E-09	1.37E-08	down
ENSG00000250091	DNAH10OS	protein_coding	-3.758926633	4.45E-09	1.41E-08	down
ENSG00000105427	CNFN	protein_coding	-6.114407287	4.64E-09	1.46E-08	down
ENSG00000237651	C2orf74	protein_coding	-6.114407287	4.64E-09	1.46E-08	down
ENSG00000197608	ZNF841	protein_coding	-6.114407287	4.64E-09	1.46E-08	down
ENSG00000167895	TMC8	protein_coding	-6.114407287	4.64E-09	1.46E-08	down
ENSG00000175920	DOK7	protein_coding	-6.114407287	4.64E-09	1.46E-08	down
ENSG00000185909	KLHDC8B	protein_coding	-6.114407287	4.64E-09	1.46E-08	down
ENSG00000215126	CBWD6	protein_coding	-6.114407287	4.64E-09	1.46E-08	down
ENSG00000092200	RPGRIP1	protein_coding	-6.114407287	4.64E-09	1.46E-08	down
ENSG00000175206	NPPA	protein_coding	-6.114407287	4.64E-09	1.46E-08	down
ENSG00000125618	PAX8	protein_coding	-6.114407287	4.64E-09	1.46E-08	down
ENSG00000204381	LAYN	protein_coding	-6.114407287	4.64E-09	1.46E-08	down
ENSG00000106404	CLDN15	protein_coding	-2.099700789	4.87E-09	1.53E-08	down
ENSG00000163818	LZTFL1	protein_coding	-1.113821897	4.89E-09	1.54E-08	down
ENSG00000164176	EDIL3	protein_coding	1.847910368	5.09E-09	1.60E-08	up

ENSG00000172828	CES3	protein_coding	1.238338399	5.11E-09	1.60E-08	up
ENSG00000140830	TXNL4B	protein_coding	-1.038834857	5.14E-09	1.61E-08	down
ENSG00000189431	RASSF10	protein_coding	-1.90551913	5.18E-09	1.63E-08	down
ENSG00000005189	REXO5	protein_coding	1.246074048	5.40E-09	1.69E-08	up
ENSG00000177432	NAP1L5	protein_coding	-5.114407287	5.59E-09	1.75E-08	down
ENSG00000076513	ANKRD13A	protein_coding	1.009307263	5.78E-09	1.80E-08	up
ENSG00000166866	MYO1A	protein_coding	4.872856725	5.86E-09	1.83E-08	up
ENSG00000196150	ZNF250	protein_coding	-1.478818714	5.99E-09	1.87E-08	down
ENSG00000073711	PPP2R3A	protein_coding	-1.478818714	5.99E-09	1.87E-08	down
ENSG00000144891	AGTR1	protein_coding	5.831036549	6.08E-09	1.89E-08	up
ENSG00000123570	RAB9B	protein_coding	5.831036549	6.08E-09	1.89E-08	up
ENSG00000181322	NME9	protein_coding	5.831036549	6.08E-09	1.89E-08	up
ENSG00000181418	DDN	protein_coding	5.831036549	6.08E-09	1.89E-08	up
ENSG00000156140	ADAMTS3	protein_coding	5.831036549	6.08E-09	1.89E-08	up
ENSG00000101695	RNF125	protein_coding	5.831036549	6.08E-09	1.89E-08	up
ENSG00000256223	ZNF10	protein_coding	-4.210622603	6.54E-09	2.03E-08	down
ENSG00000248483	POU5F2	protein_coding	-4.210622603	6.54E-09	2.03E-08	down
ENSG00000146416	AIG1	protein_coding	-1.26820346	6.77E-09	2.10E-08	down
ENSG00000197016	ZNF470	protein_coding	-2.610063247	7.39E-09	2.28E-08	down
ENSG00000178531	CTXN1	protein_coding	-2.715857911	7.60E-09	2.35E-08	down
ENSG00000235109	ZSCAN31	protein_coding	-2.715857911	7.60E-09	2.35E-08	down
ENSG00000122971	ACADS	protein_coding	1.586032431	7.83E-09	2.41E-08	up
ENSG00000138380	CARF	protein_coding	-1.562234722	7.83E-09	2.41E-08	down
ENSG00000122694	GLIPR2	protein_coding	1.544264899	7.98E-09	2.46E-08	up
ENSG00000197128	ZNF772	protein_coding	-6.063781214	8.40E-09	2.58E-08	down
ENSG00000134538	SLCO1B1	protein_coding	-6.063781214	8.40E-09	2.58E-08	down
ENSG00000110675	ELMOD1	protein_coding	-6.063781214	8.40E-09	2.58E-08	down
ENSG00000109738	GLRB	protein_coding	-6.063781214	8.40E-09	2.58E-08	down
ENSG00000143369	ECM1	protein_coding	-6.063781214	8.40E-09	2.58E-08	down
ENSG00000163467	TSACC	protein_coding	-6.063781214	8.40E-09	2.58E-08	down
ENSG00000156384	SFR1	protein_coding	-6.063781214	8.40E-09	2.58E-08	down
ENSG00000117010	ZNF684	protein_coding	-6.063781214	8.40E-09	2.58E-08	down
ENSG00000004848	ARX	protein_coding	-6.063781214	8.40E-09	2.58E-08	down
ENSG00000171564	FGB	protein_coding	-6.063781214	8.40E-09	2.58E-08	down
ENSG00000156042	CFAP70	protein_coding	-6.063781214	8.40E-09	2.58E-08	down
ENSG00000179674	ARL14	protein_coding	-6.063781214	8.40E-09	2.58E-08	down
ENSG00000198929	NOS1AP	protein_coding	-6.063781214	8.40E-09	2.58E-08	down
ENSG00000184949	FAM227A	protein_coding	1.328536208	8.41E-09	2.57E-08	up
ENSG00000171984	C20orf196	protein_coding	-3.715857911	8.60E-09	2.63E-08	down
ENSG00000167333	TRIM68	protein_coding	-1.212704915	8.74E-09	2.67E-08	down
ENSG00000105497	ZNF175	protein_coding	1.339424525	8.75E-09	2.68E-08	up
ENSG00000171885	AQP4	protein_coding	3.516163212	8.89E-09	2.72E-08	up
ENSG00000128185	DGCR6L	protein_coding	1.350731955	9.10E-09	2.78E-08	up
ENSG00000196083	IL1RAP	protein_coding	1.350731955	9.10E-09	2.78E-08	up
ENSG00000139044	B4GALNT3	protein_coding	2.35238295	9.40E-09	2.87E-08	up
ENSG00000184381	PLA2G6	protein_coding	2.203005326	9.50E-09	2.90E-08	up
ENSG00000172339	ALG14	protein_coding	-1.148050131	9.67E-09	2.95E-08	down
ENSG00000147231	CXorf57	protein_coding	3.203005326	1.01E-08	3.07E-08	up
ENSG00000162772	ATF3	protein_coding	4.831036549	1.01E-08	3.07E-08	up
ENSG00000138606	SHF	protein_coding	5.787967827	1.01E-08	3.07E-08	up
ENSG00000177182	CLVS1	protein_coding	5.787967827	1.01E-08	3.07E-08	up
ENSG00000196421	C20orf204	protein_coding	5.787967827	1.01E-08	3.07E-08	up
ENSG00000149972	CNTN5	protein_coding	5.787967827	1.01E-08	3.07E-08	up
ENSG00000145040	UCN2	protein_coding	5.787967827	1.01E-08	3.07E-08	up

ENSG00000182359	KBTBD3	protein_coding	-2.343889134	1.03E-08	3.13E-08	down
ENSG00000104881	PPP1R13L	protein_coding	1.069343869	1.05E-08	3.18E-08	up
ENSG00000258366	RTEL1	protein_coding	-5.063781214	1.06E-08	3.22E-08	down
ENSG00000179988	PSTK	protein_coding	-3.385709309	1.07E-08	3.24E-08	down
ENSG00000157429	ZNF19	protein_coding	1.618042826	1.11E-08	3.35E-08	up
ENSG00000075461	CACNG4	protein_coding	1.459780742	1.13E-08	3.42E-08	up
ENSG00000130772	MED18	protein_coding	1.594430268	1.13E-08	3.42E-08	up
ENSG00000130052	STARD8	protein_coding	-2.017238628	1.18E-08	3.56E-08	down
ENSG00000255423	EBLN2	protein_coding	1.972392398	1.19E-08	3.60E-08	up
ENSG00000126522	ASL	protein_coding	-1.730357481	1.19E-08	3.61E-08	down
ENSG00000142235	LMTK3	protein_coding	1.203005326	1.25E-08	3.76E-08	up
ENSG00000213722	DDAH2	protein_coding	1.130596831	1.25E-08	3.77E-08	up
ENSG00000254469	AP002495.1	protein_coding	-2.489087049	1.26E-08	3.79E-08	down
ENSG00000143479	DYRK3	protein_coding	-1.426351294	1.27E-08	3.81E-08	down
ENSG00000155906	RMND1	protein_coding	1.019585968	1.27E-08	3.81E-08	up
ENSG00000204520	MICA	protein_coding	-1.201564357	1.27E-08	3.83E-08	down
ENSG00000183826	BTBD9	protein_coding	-1.146548675	1.27E-08	3.83E-08	down
ENSG00000063438	AHRR	protein_coding	1.13589113	1.34E-08	4.04E-08	up
ENSG00000163472	TMEM79	protein_coding	-2.578354387	1.34E-08	4.04E-08	down
ENSG00000120093	HOXB3	protein_coding	-2.203958872	1.38E-08	4.15E-08	down
ENSG00000256683	ZNF350	protein_coding	-2.0516065	1.43E-08	4.28E-08	down
ENSG00000174684	B4GAT1	protein_coding	-1.129191104	1.51E-08	4.51E-08	down
ENSG00000170430	MGMT	protein_coding	1.40253679	1.51E-08	4.53E-08	up
ENSG00000160460	SPTBN4	protein_coding	-1.745811133	1.52E-08	4.55E-08	down
ENSG00000154678	PDE1C	protein_coding	-6.011313794	1.52E-08	4.55E-08	down
ENSG00000173267	SNCG	protein_coding	-6.011313794	1.52E-08	4.55E-08	down
ENSG00000069712	KIAA1107	protein_coding	-6.011313794	1.52E-08	4.55E-08	down
ENSG00000152454	ZNF256	protein_coding	-6.011313794	1.52E-08	4.55E-08	down
ENSG00000155980	KIF5A	protein_coding	-6.011313794	1.52E-08	4.55E-08	down
ENSG00000150773	PIH1D2	protein_coding	-6.011313794	1.52E-08	4.55E-08	down
ENSG00000008517	IL32	protein_coding	-6.011313794	1.52E-08	4.55E-08	down
ENSG00000076662	ICAM3	protein_coding	-6.011313794	1.52E-08	4.55E-08	down
ENSG00000277399	GPR179	protein_coding	-6.011313794	1.52E-08	4.55E-08	down
ENSG00000145246	ATP10D	protein_coding	-1.473657009	1.59E-08	4.71E-08	down
ENSG00000104870	FCGRT	protein_coding	1.069448987	1.64E-08	4.86E-08	up
ENSG00000013619	MAMLD1	protein_coding	1.517297852	1.68E-08	5.00E-08	up
ENSG00000147145	LPAR4	protein_coding	5.743573708	1.69E-08	5.00E-08	up
ENSG00000249931	GOLGA8K	protein_coding	5.743573708	1.69E-08	5.00E-08	up
ENSG00000136457	CHAD	protein_coding	5.743573708	1.69E-08	5.00E-08	up
ENSG00000120696	KBTBD7	protein_coding	-1.171537395	1.69E-08	5.01E-08	down
ENSG00000197808	ZNF461	protein_coding	-1.171537395	1.69E-08	5.01E-08	down
ENSG00000148655	LRMDA	protein_coding	-2.089316306	1.71E-08	5.07E-08	down
ENSG00000135185	TMEM243	protein_coding	-1.256426292	1.74E-08	5.14E-08	down
ENSG00000174871	CNIH2	protein_coding	4.787967827	1.74E-08	5.15E-08	up
ENSG00000171680	PLEKHG5	protein_coding	1.249464637	1.78E-08	5.25E-08	up
ENSG00000164056	SPRY1	protein_coding	1.200618734	1.82E-08	5.36E-08	up
ENSG00000170624	SGCD	protein_coding	-1.08327739	1.83E-08	5.41E-08	down
ENSG00000164742	ADCY1	protein_coding	-1.453463139	1.87E-08	5.50E-08	down
ENSG00000120913	PDLIM2	protein_coding	1.258146881	1.87E-08	5.53E-08	up
ENSG00000256043	CTSO	protein_coding	1.760647221	1.91E-08	5.62E-08	up
ENSG00000183114	FAM43B	protein_coding	1.760647221	1.91E-08	5.62E-08	up
ENSG00000163686	ABHD6	protein_coding	-1.411704518	1.93E-08	5.69E-08	down
ENSG00000198948	MFAP3L	protein_coding	-1.710992156	1.93E-08	5.69E-08	down
ENSG00000078967	UBE2D4	protein_coding	-1.026420687	1.97E-08	5.80E-08	down

ENSG00000019485	PRDM11	protein_coding	-1.026420687	1.97E-08	5.80E-08	down
ENSG00000102547	CAB39L	protein_coding	-1.993391886	2.00E-08	5.88E-08	down
ENSG00000065413	ANKRD44	protein_coding	1.727085585	2.02E-08	5.95E-08	up
ENSG00000159214	CCDC24	protein_coding	-5.011313794	2.02E-08	5.95E-08	down
ENSG00000118402	ELOVL4	protein_coding	-2.13089541	2.04E-08	5.99E-08	down
ENSG00000188266	HYKK	protein_coding	1.042540654	2.27E-08	6.64E-08	up
ENSG00000115317	HTRA2	protein_coding	-1.008038662	2.37E-08	6.94E-08	down
ENSG00000178814	OPLAH	protein_coding	-2.545932909	2.44E-08	7.13E-08	down
ENSG00000185924	RTN4RL1	protein_coding	2.608644128	2.57E-08	7.48E-08	up
ENSG00000161277	THAP8	protein_coding	1.123845789	2.63E-08	7.66E-08	up
ENSG00000134363	FST	protein_coding	3.444013426	2.66E-08	7.74E-08	up
ENSG00000120458	MSANTD2	protein_coding	-1.151073292	2.67E-08	7.77E-08	down
ENSG00000163623	NKX6-1	protein_coding	-5.95686601	2.78E-08	8.07E-08	down
ENSG00000187957	DNER	protein_coding	-5.95686601	2.78E-08	8.07E-08	down
ENSG00000256235	SMIM3	protein_coding	-5.95686601	2.78E-08	8.07E-08	down
ENSG00000124743	KLHL31	protein_coding	-5.95686601	2.78E-08	8.07E-08	down
ENSG00000109654	TRIM2	protein_coding	-5.95686601	2.78E-08	8.07E-08	down
ENSG00000186354	C9orf47	protein_coding	-5.95686601	2.78E-08	8.07E-08	down
ENSG00000162650	ATXN7L2	protein_coding	-5.95686601	2.78E-08	8.07E-08	down
ENSG00000015592	STMN4	protein_coding	-5.95686601	2.78E-08	8.07E-08	down
ENSG00000136630	HLX	protein_coding	-5.95686601	2.78E-08	8.07E-08	down
ENSG00000131471	AOC3	protein_coding	-5.95686601	2.78E-08	8.07E-08	down
ENSG00000204962	PCDHA8	protein_coding	-5.95686601	2.78E-08	8.07E-08	down
ENSG00000151117	TMEM86A	protein_coding	-5.95686601	2.78E-08	8.07E-08	down
ENSG00000008311	AASS	protein_coding	-5.95686601	2.78E-08	8.07E-08	down
ENSG00000135617	PRADC1	protein_coding	-5.95686601	2.78E-08	8.07E-08	down
ENSG00000164604	GPR85	protein_coding	5.697770018	2.82E-08	8.16E-08	up
ENSG00000074317	SNCB	protein_coding	5.697770018	2.82E-08	8.16E-08	up
ENSG00000085741	WNT11	protein_coding	2.743573708	2.85E-08	8.23E-08	up
ENSG00000185513	L3MBTL1	protein_coding	2.743573708	2.85E-08	8.23E-08	up
ENSG00000168228	ZCCHC4	protein_coding	-1.168963451	2.94E-08	8.48E-08	down
ENSG00000113205	PCDHB3	protein_coding	3.13589113	2.95E-08	8.51E-08	up
ENSG00000169903	TM4SF4	protein_coding	-1.86410887	2.97E-08	8.57E-08	down
ENSG00000187609	EXD3	protein_coding	1.884929557	3.18E-08	9.12E-08	up
ENSG00000183137	CEP57L1	protein_coding	-1.46287717	3.21E-08	9.21E-08	down
ENSG00000137274	BPHL	protein_coding	-1.46287717	3.21E-08	9.21E-08	down
ENSG00000133627	ACTR3B	protein_coding	-1.544002882	3.27E-08	9.38E-08	down
ENSG00000041982	TNC	protein_coding	-1.418697721	3.34E-08	9.58E-08	down
ENSG00000130518	KIAA1683	protein_coding	-1.96914434	3.38E-08	9.70E-08	down
ENSG00000163297	ANTXR2	protein_coding	1.146332877	3.51E-08	1.00E-07	up
ENSG00000256061	DNAAF4	protein_coding	2.471494162	3.64E-08	1.04E-07	up
ENSG00000186648	CARMIL3	protein_coding	3.872856725	3.77E-08	1.08E-07	up
ENSG00000243244	STON1	protein_coding	3.872856725	3.77E-08	1.08E-07	up
ENSG00000170260	ZNF212	protein_coding	1.246074048	3.79E-08	1.08E-07	up
ENSG00000139132	FGD4	protein_coding	1.195562343	3.86E-08	1.10E-07	up
ENSG00000277363	SRCIN1	protein_coding	1.195562343	3.86E-08	1.10E-07	up
ENSG00000175564	UCP3	protein_coding	-4.95686601	3.87E-08	1.10E-07	down
ENSG00000244165	P2RY11	protein_coding	-4.95686601	3.87E-08	1.10E-07	down
ENSG00000099974	DDTL	protein_coding	2.279626608	3.93E-08	1.12E-07	up
ENSG00000060642	PIGV	protein_coding	-1.705733694	4.00E-08	1.14E-07	down
ENSG00000233954	UQCRHL	protein_coding	-2.149511088	4.18E-08	1.19E-07	down
ENSG00000130821	SLC6A8	protein_coding	1.366010914	4.30E-08	1.22E-07	up
ENSG00000128805	ARHGAP22	protein_coding	1.165037476	4.31E-08	1.23E-07	up
ENSG00000278535	DHRS11	protein_coding	-1.313009821	4.36E-08	1.24E-07	down

ENSG00000196378	ZNF34	protein_coding	-2.512766046	4.42E-08	1.25E-07	down
ENSG00000109743	BST1	protein_coding	-3.063781214	4.44E-08	1.26E-07	down
ENSG00000187726	DNAJB13	protein_coding	2.065501803	4.53E-08	1.28E-07	up
ENSG00000165548	TMEM63C	protein_coding	3.40653872	4.59E-08	1.30E-07	up
ENSG00000167930	FAM234A	protein_coding	-1.208332004	4.61E-08	1.31E-07	down
ENSG00000169860	P2RY1	protein_coding	-1.915389374	4.61E-08	1.31E-07	down
ENSG00000175322	ZNF519	protein_coding	1.078363954	4.68E-08	1.33E-07	up
ENSG00000006740	ARHGAP44	protein_coding	1.662955248	4.71E-08	1.33E-07	up
ENSG00000144749	LRIG1	protein_coding	-2.880917157	4.73E-08	1.34E-07	down
ENSG00000183914	DNAH2	protein_coding	5.650464303	4.73E-08	1.34E-07	up
ENSG00000134532	SOX5	protein_coding	5.650464303	4.73E-08	1.34E-07	up
ENSG00000128645	HOXD1	protein_coding	5.650464303	4.73E-08	1.34E-07	up
ENSG00000186862	PDZD7	protein_coding	5.650464303	4.73E-08	1.34E-07	up
ENSG00000172955	ADH6	protein_coding	5.650464303	4.73E-08	1.34E-07	up
ENSG00000092758	COL9A3	protein_coding	5.650464303	4.73E-08	1.34E-07	up
ENSG00000146453	PNLDC1	protein_coding	5.650464303	4.73E-08	1.34E-07	up
ENSG00000155749	ALS2CR12	protein_coding	5.650464303	4.73E-08	1.34E-07	up
ENSG00000115844	DLX2	protein_coding	5.650464303	4.73E-08	1.34E-07	up
ENSG00000164825	DEFB1	protein_coding	5.650464303	4.73E-08	1.34E-07	up
ENSG00000174448	STARD6	protein_coding	5.650464303	4.73E-08	1.34E-07	up
ENSG00000249471	ZNF324B	protein_coding	1.634344638	4.86E-08	1.37E-07	up
ENSG00000214026	MRPL23	protein_coding	1.226895947	4.93E-08	1.39E-07	up
ENSG00000183513	COA5	protein_coding	-1.005618189	4.98E-08	1.40E-07	down
ENSG00000154025	SLC5A10	protein_coding	-5.900282482	5.08E-08	1.43E-07	down
ENSG00000056736	IL17RB	protein_coding	-5.900282482	5.08E-08	1.43E-07	down
ENSG00000125122	LRRC29	protein_coding	-5.900282482	5.08E-08	1.43E-07	down
ENSG00000110723	EXPH5	protein_coding	-5.900282482	5.08E-08	1.43E-07	down
ENSG00000182836	PLCXD3	protein_coding	-5.900282482	5.08E-08	1.43E-07	down
ENSG00000128965	CHAC1	protein_coding	-5.900282482	5.08E-08	1.43E-07	down
ENSG00000235961	PNMA6A	protein_coding	-5.900282482	5.08E-08	1.43E-07	down
ENSG00000162669	HFM1	protein_coding	-5.900282482	5.08E-08	1.43E-07	down
ENSG00000186832	KRT16	protein_coding	-5.900282482	5.08E-08	1.43E-07	down
ENSG00000162946	DISC1	protein_coding	-5.900282482	5.08E-08	1.43E-07	down
ENSG00000196433	ASMT	protein_coding	-5.900282482	5.08E-08	1.43E-07	down
ENSG00000196456	ZNF775	protein_coding	-5.900282482	5.08E-08	1.43E-07	down
ENSG00000157111	TMEM171	protein_coding	-5.900282482	5.08E-08	1.43E-07	down
ENSG00000172748	ZNF596	protein_coding	-5.900282482	5.08E-08	1.43E-07	down
ENSG00000205923	CEMP1	protein_coding	-5.900282482	5.08E-08	1.43E-07	down
ENSG00000244731	C4A	protein_coding	-5.900282482	5.08E-08	1.43E-07	down
ENSG00000156298	TSPAN7	protein_coding	-5.900282482	5.08E-08	1.43E-07	down
ENSG00000174837	ADGRE1	protein_coding	-5.900282482	5.08E-08	1.43E-07	down
ENSG00000135678	CPM	protein_coding	-5.900282482	5.08E-08	1.43E-07	down
ENSG00000145242	EPHA5	protein_coding	-5.900282482	5.08E-08	1.43E-07	down
ENSG00000170271	FAXDC2	protein_coding	-5.900282482	5.08E-08	1.43E-07	down
ENSG00000161281	COX7A1	protein_coding	4.697770018	5.22E-08	1.46E-07	up
ENSG00000135114	OASL	protein_coding	4.697770018	5.22E-08	1.46E-07	up
ENSG00000068354	TBC1D25	protein_coding	-1.104423199	5.31E-08	1.48E-07	down
ENSG00000104671	DCTN6	protein_coding	-1.07404955	5.45E-08	1.52E-07	down
ENSG00000078487	ZCWPW1	protein_coding	1.149088433	5.47E-08	1.52E-07	up
ENSG00000214021	TTLL3	protein_coding	1.005065949	5.82E-08	1.62E-07	up
ENSG00000142156	COL6A1	protein_coding	-1.119924292	5.96E-08	1.66E-07	down
ENSG00000161920	MED11	protein_coding	-1.119924292	5.96E-08	1.66E-07	down
ENSG00000160993	ALKBH4	protein_coding	2.254535627	6.30E-08	1.75E-07	up
ENSG00000189410	SH2D5	protein_coding	-1.451442275	6.48E-08	1.80E-07	down

ENSG00000154642	C21orf91	protein_coding	-1.533960268	6.67E-08	1.85E-07	down
ENSG00000152217	SETBP1	protein_coding	1.395650404	6.70E-08	1.86E-07	up
ENSG00000242802	AP5Z1	protein_coding	-1.366850282	6.76E-08	1.87E-07	down
ENSG00000153558	FBXL2	protein_coding	1.216326705	6.78E-08	1.88E-07	up
ENSG00000106927	AMBP	protein_coding	1.216326705	6.78E-08	1.88E-07	up
ENSG00000169084	DHRSX	protein_coding	-1.102255362	6.98E-08	1.93E-07	down
ENSG00000112182	BACH2	protein_coding	2.55092863	7.00E-08	1.94E-07	up
ENSG00000034533	ASTE1	protein_coding	2.04313399	7.09E-08	1.96E-07	up
ENSG00000163312	HELQ	protein_coding	-2.121496712	7.22E-08	1.99E-07	down
ENSG00000156853	ZNF689	protein_coding	-1.284440668	7.32E-08	2.02E-07	down
ENSG00000134755	DSC2	protein_coding	1.139502384	7.40E-08	2.04E-07	up
ENSG00000169951	ZNF764	protein_coding	-4.900282482	7.40E-08	2.04E-07	down
ENSG00000169435	RASSF6	protein_coding	-4.900282482	7.40E-08	2.04E-07	down
ENSG00000171631	P2RY6	protein_coding	1.543275057	7.47E-08	2.06E-07	up
ENSG00000274180	NATD1	protein_coding	-3.256426292	7.49E-08	2.06E-07	down
ENSG00000134262	AP4B1	protein_coding	-1.607898663	7.68E-08	2.12E-07	down
ENSG00000055118	KCNH2	protein_coding	2.166785138	7.84E-08	2.15E-07	up
ENSG00000120471	TP53AIP1	protein_coding	5.601554703	7.96E-08	2.18E-07	up
ENSG00000168961	LGALS9	protein_coding	5.601554703	7.96E-08	2.18E-07	up
ENSG00000105549	THEG	protein_coding	5.601554703	7.96E-08	2.18E-07	up
ENSG00000140600	SH3GL3	protein_coding	5.601554703	7.96E-08	2.18E-07	up
ENSG00000188739	RBM34	protein_coding	5.601554703	7.96E-08	2.18E-07	up
ENSG00000183484	GPR132	protein_coding	5.601554703	7.96E-08	2.18E-07	up
ENSG00000166839	ANKDD1A	protein_coding	5.601554703	7.96E-08	2.18E-07	up
ENSG00000170786	SDR16C5	protein_coding	5.601554703	7.96E-08	2.18E-07	up
ENSG00000160224	AIRE	protein_coding	5.601554703	7.96E-08	2.18E-07	up
ENSG00000167840	ZNF232	protein_coding	-1.456363863	8.51E-08	2.33E-07	down
ENSG00000176473	WDR25	protein_coding	-1.456363863	8.51E-08	2.33E-07	down
ENSG00000148384	INPP5E	protein_coding	-1.022936162	8.73E-08	2.39E-07	down
ENSG00000103066	PLA2G15	protein_coding	1.354299305	8.89E-08	2.43E-07	up
ENSG00000150510	FAM124A	protein_coding	1.261422013	9.02E-08	2.47E-07	up
ENSG00000187066	TMEM262	protein_coding	-4.011313794	9.29E-08	2.53E-07	down
ENSG00000203737	GPR52	protein_coding	-5.841388793	9.33E-08	2.54E-07	down
ENSG00000164088	PPM1M	protein_coding	-5.841388793	9.33E-08	2.54E-07	down
ENSG00000187775	DNAH17	protein_coding	-5.841388793	9.33E-08	2.54E-07	down
ENSG00000197106	SLC6A17	protein_coding	-5.841388793	9.33E-08	2.54E-07	down
ENSG00000148832	PAOX	protein_coding	-5.841388793	9.33E-08	2.54E-07	down
ENSG00000242574	HLA-DMB	protein_coding	-5.841388793	9.33E-08	2.54E-07	down
ENSG00000133805	AMPD3	protein_coding	-5.841388793	9.33E-08	2.54E-07	down
ENSG00000166664	CHRFAM7A	protein_coding	-5.841388793	9.33E-08	2.54E-07	down
ENSG00000176024	ZNF613	protein_coding	-5.841388793	9.33E-08	2.54E-07	down
ENSG00000112293	GPLD1	protein_coding	-5.841388793	9.33E-08	2.54E-07	down
ENSG00000170608	FOXA3	protein_coding	-5.841388793	9.33E-08	2.54E-07	down
ENSG00000253731	PCDHGA6	protein_coding	-5.841388793	9.33E-08	2.54E-07	down
ENSG00000180855	ZNF443	protein_coding	-5.841388793	9.33E-08	2.54E-07	down
ENSG00000165972	CCDC38	protein_coding	-5.841388793	9.33E-08	2.54E-07	down
ENSG00000150722	PPP1R1C	protein_coding	-5.841388793	9.33E-08	2.54E-07	down
ENSG00000169071	ROR2	protein_coding	-5.841388793	9.33E-08	2.54E-07	down
ENSG00000169169	CPT1C	protein_coding	-5.841388793	9.33E-08	2.54E-07	down
ENSG00000103089	FA2H	protein_coding	1.213893642	9.89E-08	2.68E-07	up
ENSG00000042429	MED17	protein_coding	1.281993623	1.00E-07	2.71E-07	up
ENSG00000169762	TAPT1	protein_coding	-1.243005777	1.03E-07	2.78E-07	down
ENSG00000143127	ITGA10	protein_coding	1.872856725	1.03E-07	2.80E-07	up
ENSG00000157510	AFAP1L1	protein_coding	-1.515812921	1.04E-07	2.82E-07	down

ENSG00000131370	SH3BP5	protein_coding	-1.002977935	1.05E-07	2.83E-07	down
ENSG00000145569	FAM105A	protein_coding	1.601554703	1.06E-07	2.86E-07	up
ENSG00000182263	FIGN	protein_coding	-1.14276951	1.06E-07	2.86E-07	down
ENSG00000161682	FAM171A2	protein_coding	1.55092863	1.08E-07	2.93E-07	up
ENSG00000178852	EFCAB13	protein_coding	1.506074394	1.09E-07	2.93E-07	up
ENSG00000139173	TMEM117	protein_coding	2.020413913	1.11E-07	2.99E-07	up
ENSG00000186493	C5orf38	protein_coding	1.826035868	1.13E-07	3.05E-07	up
ENSG00000121361	KCNJ8	protein_coding	2.521181286	1.15E-07	3.10E-07	up
ENSG00000064225	ST3GAL6	protein_coding	-1.490891546	1.24E-07	3.34E-07	down
ENSG00000160683	CXCR5	protein_coding	-2.360762952	1.30E-07	3.48E-07	down
ENSG00000174842	GLMN	protein_coding	-1.317826837	1.32E-07	3.55E-07	down
ENSG00000160813	PPP1R35	protein_coding	-1.794846207	1.34E-07	3.60E-07	down
ENSG00000168675	LDLRAD4	protein_coding	5.55092863	1.34E-07	3.59E-07	up
ENSG00000021488	SLC7A9	protein_coding	5.55092863	1.34E-07	3.59E-07	up
ENSG00000147465	STAR	protein_coding	5.55092863	1.34E-07	3.59E-07	up
ENSG00000215262	KCNU1	protein_coding	5.55092863	1.34E-07	3.59E-07	up
ENSG00000132801	ZSWIM3	protein_coding	5.55092863	1.34E-07	3.59E-07	up
ENSG00000167700	MFSD3	protein_coding	-1.393929816	1.34E-07	3.59E-07	down
ENSG00000162458	FBLIM1	protein_coding	-1.023980206	1.41E-07	3.75E-07	down
ENSG00000063180	CA11	protein_coding	1.162075062	1.42E-07	3.80E-07	up
ENSG00000140993	TIGD7	protein_coding	1.902772303	1.43E-07	3.82E-07	up
ENSG00000099330	OCEL1	protein_coding	1.641694094	1.49E-07	3.96E-07	up
ENSG00000145107	TM4SF19	protein_coding	1.451392956	1.53E-07	4.08E-07	up
ENSG00000162413	KLHL21	protein_coding	-1.160210977	1.55E-07	4.11E-07	down
ENSG00000105877	DNAH11	protein_coding	1.584875962	1.55E-07	4.13E-07	up
ENSG00000214694	ARHGEF33	protein_coding	4.601554703	1.57E-07	4.18E-07	up
ENSG00000242028	HYPK	protein_coding	2.387429897	1.58E-07	4.19E-07	up
ENSG00000162526	TSSK3	protein_coding	2.387429897	1.58E-07	4.19E-07	up
ENSG00000149150	SLC43A1	protein_coding	1.852098164	1.58E-07	4.20E-07	up
ENSG00000156299	TIAM1	protein_coding	-1.893856213	1.60E-07	4.25E-07	down
ENSG00000152582	SPEF2	protein_coding	-1.216533565	1.67E-07	4.43E-07	down
ENSG00000115221	ITGB6	protein_coding	-5.779988248	1.72E-07	4.55E-07	down
ENSG00000163565	IFI16	protein_coding	-5.779988248	1.72E-07	4.55E-07	down
ENSG00000134207	SYT6	protein_coding	-5.779988248	1.72E-07	4.55E-07	down
ENSG00000168237	GLYCTK	protein_coding	-5.779988248	1.72E-07	4.55E-07	down
ENSG00000157992	KRTCAP3	protein_coding	-5.779988248	1.72E-07	4.55E-07	down
ENSG00000182993	C12orf60	protein_coding	-5.779988248	1.72E-07	4.55E-07	down
ENSG00000163093	BBS5	protein_coding	-5.779988248	1.72E-07	4.55E-07	down
ENSG00000163376	KBTBD8	protein_coding	-5.779988248	1.72E-07	4.55E-07	down
ENSG00000147041	SYTL5	protein_coding	-5.779988248	1.72E-07	4.55E-07	down
ENSG00000064205	WISP2	protein_coding	-5.779988248	1.72E-07	4.55E-07	down
ENSG00000147144	CCDC120	protein_coding	-5.779988248	1.72E-07	4.55E-07	down
ENSG00000186867	QRFPR	protein_coding	-5.779988248	1.72E-07	4.55E-07	down
ENSG00000255872	AL138752.2	protein_coding	-5.779988248	1.72E-07	4.55E-07	down
ENSG00000127364	TAS2R4	protein_coding	-5.779988248	1.72E-07	4.55E-07	down
ENSG00000185670	ZBTB3	protein_coding	-5.779988248	1.72E-07	4.55E-07	down
ENSG00000198829	SUCNR1	protein_coding	-5.779988248	1.72E-07	4.55E-07	down
ENSG00000204052	LRRC73	protein_coding	-5.779988248	1.72E-07	4.55E-07	down
ENSG00000277224	HIST1H2BF	protein_coding	-5.779988248	1.72E-07	4.55E-07	down
ENSG00000253958	CLDN23	protein_coding	1.184146299	1.74E-07	4.59E-07	up
ENSG00000088451	TGDS	protein_coding	-1.242487101	1.79E-07	4.71E-07	down
ENSG00000185420	SMYD3	protein_coding	1.031150298	1.79E-07	4.72E-07	up
ENSG00000096654	ZNF184	protein_coding	1.248046291	1.83E-07	4.81E-07	up
ENSG00000151917	BEND6	protein_coding	1.724464885	1.96E-07	5.14E-07	up

ENSG00000102043	MTMR8	protein_coding	2.117032103	1.97E-07	5.16E-07	up
ENSG00000181035	SLC25A42	protein_coding	3.743573708	1.99E-07	5.23E-07	up
ENSG00000124257	NEURL2	protein_coding	2.274088424	2.05E-07	5.38E-07	up
ENSG00000146021	KLHL3	protein_coding	2.274088424	2.05E-07	5.38E-07	up
ENSG00000184489	PTP4A3	protein_coding	1.436595954	2.20E-07	5.75E-07	up
ENSG00000163449	TMEM169	protein_coding	1.595051183	2.25E-07	5.87E-07	up
ENSG00000119866	BCL11A	protein_coding	5.49846121	2.27E-07	5.92E-07	up
ENSG00000105467	SYNGR4	protein_coding	5.49846121	2.27E-07	5.92E-07	up
ENSG00000139364	TMEM132B	protein_coding	5.49846121	2.27E-07	5.92E-07	up
ENSG00000139597	N4BP2L1	protein_coding	5.49846121	2.27E-07	5.92E-07	up
ENSG00000186854	TRABD2A	protein_coding	5.49846121	2.27E-07	5.92E-07	up
ENSG00000136352	NKX2-1	protein_coding	5.49846121	2.27E-07	5.92E-07	up
ENSG00000145949	MYLK4	protein_coding	5.49846121	2.27E-07	5.92E-07	up
ENSG00000116194	ANGPTL1	protein_coding	5.49846121	2.27E-07	5.92E-07	up
ENSG00000057593	F7	protein_coding	5.49846121	2.27E-07	5.92E-07	up
ENSG00000135100	HNF1A	protein_coding	5.49846121	2.27E-07	5.92E-07	up
ENSG00000111452	ADGRD1	protein_coding	5.49846121	2.27E-07	5.92E-07	up
ENSG00000169495	HTRA4	protein_coding	5.49846121	2.27E-07	5.92E-07	up
ENSG00000228486	C2orf92	protein_coding	-2.32681562	2.30E-07	6.00E-07	down
ENSG00000253767	PCDHGA8	protein_coding	2.041254256	2.33E-07	6.06E-07	up
ENSG00000206262	FOXL2NB	protein_coding	2.779197617	2.41E-07	6.26E-07	up
ENSG00000124614	RPS10	protein_coding	1.831036549	2.41E-07	6.26E-07	up
ENSG00000109944	JHY	protein_coding	1.047354456	2.50E-07	6.48E-07	up
ENSG00000176046	NUPR1	protein_coding	1.026212671	2.58E-07	6.68E-07	up
ENSG00000153165	RGPD3	protein_coding	4.55092863	2.73E-07	7.06E-07	up
ENSG00000105708	ZNF14	protein_coding	-4.779988248	2.74E-07	7.07E-07	down
ENSG00000100558	PLEK2	protein_coding	-4.779988248	2.74E-07	7.07E-07	down
ENSG00000172780	RAB43	protein_coding	-4.779988248	2.74E-07	7.07E-07	down
ENSG00000102878	HSF4	protein_coding	-4.779988248	2.74E-07	7.07E-07	down
ENSG00000160221	C21orf33	protein_coding	-4.779988248	2.74E-07	7.07E-07	down
ENSG00000137070	IL11RA	protein_coding	-1.792479193	2.79E-07	7.19E-07	down
ENSG00000145214	DGKQ	protein_coding	1.256386423	2.82E-07	7.28E-07	up
ENSG00000114279	FGF12	protein_coding	1.37160493	2.89E-07	7.44E-07	up
ENSG00000182919	C11orf54	protein_coding	-1.215006365	2.91E-07	7.50E-07	down
ENSG00000139160	ETFBKMT	protein_coding	-2.163316888	3.03E-07	7.79E-07	down
ENSG00000184985	SORCS2	protein_coding	1.404087241	3.07E-07	7.87E-07	up
ENSG00000179941	BBS10	protein_coding	1.013662871	3.13E-07	8.02E-07	up
ENSG00000105851	PIK3CG	protein_coding	-5.715857911	3.19E-07	8.14E-07	down
ENSG00000105929	ATP6V0A4	protein_coding	-5.715857911	3.19E-07	8.14E-07	down
ENSG00000164532	TBX20	protein_coding	-5.715857911	3.19E-07	8.14E-07	down
ENSG00000164619	BMPER	protein_coding	-5.715857911	3.19E-07	8.14E-07	down
ENSG00000261934	PCDHGA9	protein_coding	-5.715857911	3.19E-07	8.14E-07	down
ENSG00000163624	CDS1	protein_coding	-5.715857911	3.19E-07	8.14E-07	down
ENSG00000114923	SLC4A3	protein_coding	-5.715857911	3.19E-07	8.14E-07	down
ENSG00000197872	FAM49A	protein_coding	-5.715857911	3.19E-07	8.14E-07	down
ENSG00000100867	DHRS2	protein_coding	-5.715857911	3.19E-07	8.14E-07	down
ENSG00000124813	RUNX2	protein_coding	-5.715857911	3.19E-07	8.14E-07	down
ENSG00000137434	C6orf52	protein_coding	-5.715857911	3.19E-07	8.14E-07	down
ENSG00000206077	ZDHHC11B	protein_coding	-5.715857911	3.19E-07	8.14E-07	down
ENSG00000064692	SNCAIP	protein_coding	-5.715857911	3.19E-07	8.14E-07	down
ENSG00000172824	CES4A	protein_coding	-5.715857911	3.19E-07	8.14E-07	down
ENSG00000166035	LIPC	protein_coding	-5.715857911	3.19E-07	8.14E-07	down
ENSG00000166349	RAG1	protein_coding	-5.715857911	3.19E-07	8.14E-07	down
ENSG00000259571	BLID	protein_coding	-5.715857911	3.19E-07	8.14E-07	down

ENSG00000112137	PHACTR1	protein_coding	-5.715857911	3.19E-07	8.14E-07	down
ENSG00000171462	DLK2	protein_coding	-5.715857911	3.19E-07	8.14E-07	down
ENSG00000106006	HOXA6	protein_coding	-5.715857911	3.19E-07	8.14E-07	down
ENSG00000121743	GJA3	protein_coding	-5.715857911	3.19E-07	8.14E-07	down
ENSG00000214562	NUTM2D	protein_coding	-5.715857911	3.19E-07	8.14E-07	down
ENSG00000165215	CLDN3	protein_coding	-5.715857911	3.19E-07	8.14E-07	down
ENSG00000180875	GREM2	protein_coding	-5.715857911	3.19E-07	8.14E-07	down
ENSG00000140905	GCSH	protein_coding	-5.715857911	3.19E-07	8.14E-07	down
ENSG00000184530	C6orf58	protein_coding	-5.715857911	3.19E-07	8.14E-07	down
ENSG00000184845	DRD1	protein_coding	-5.715857911	3.19E-07	8.14E-07	down
ENSG00000127129	EDN2	protein_coding	2.246074048	3.30E-07	8.39E-07	up
ENSG00000178038	ALS2CL	protein_coding	1.577563756	3.30E-07	8.39E-07	up
ENSG00000164342	TLR3	protein_coding	1.859050925	3.37E-07	8.56E-07	up
ENSG00000169964	TMEM42	protein_coding	-1.304789314	3.45E-07	8.76E-07	down
ENSG00000089558	KCNH4	protein_coding	3.697770018	3.47E-07	8.82E-07	up
ENSG00000019505	SYT13	protein_coding	-3.900282482	3.52E-07	8.93E-07	down
ENSG00000158292	GPR153	protein_coding	2.584875962	3.63E-07	9.19E-07	up
ENSG00000110436	SLC1A2	protein_coding	2.016592202	3.64E-07	9.23E-07	up
ENSG00000187122	SLIT1	protein_coding	5.444013426	3.84E-07	9.71E-07	up
ENSG00000196482	ESRRG	protein_coding	5.444013426	3.84E-07	9.71E-07	up
ENSG00000166816	LDHD	protein_coding	5.444013426	3.84E-07	9.71E-07	up
ENSG00000112936	C7	protein_coding	5.444013426	3.84E-07	9.71E-07	up
ENSG00000097096	SYDE2	protein_coding	-1.408429386	4.04E-07	1.02E-06	down
ENSG00000204248	COL11A2	protein_coding	2.149566067	4.08E-07	1.03E-06	up
ENSG00000198246	SLC29A3	protein_coding	1.004839021	4.12E-07	1.04E-06	up
ENSG00000184792	OSBP2	protein_coding	1.004839021	4.12E-07	1.04E-06	up
ENSG00000113971	NPHP3	protein_coding	1.950024585	4.17E-07	1.05E-06	up
ENSG00000108465	CDK5RAP3	protein_coding	-1.600380694	4.23E-07	1.06E-06	down
ENSG00000148803	FUOM	protein_coding	2.953027073	4.23E-07	1.06E-06	up
ENSG00000280987	MATR3	protein_coding	-2.07954853	4.41E-07	1.11E-06	down
ENSG00000158717	RNF166	protein_coding	-2.37190351	4.61E-07	1.16E-06	down
ENSG00000103253	HAGHL	protein_coding	-1.38682293	4.68E-07	1.17E-06	down
ENSG00000071246	VASH1	protein_coding	1.119949587	4.79E-07	1.20E-06	up
ENSG00000260643	AC092718.3	protein_coding	1.486077485	4.83E-07	1.21E-06	up
ENSG00000164008	C1orf50	protein_coding	2.428071882	5.07E-07	1.27E-06	up
ENSG00000164620	RELL2	protein_coding	-2.465879658	5.09E-07	1.27E-06	down
ENSG00000133678	TMEM254	protein_coding	-1.964245541	5.10E-07	1.27E-06	down
ENSG00000164920	OSR2	protein_coding	-3.114407287	5.18E-07	1.29E-06	down
ENSG00000006025	OSBPL7	protein_coding	1.037304911	5.19E-07	1.30E-06	up
ENSG00000230797	YY2	protein_coding	-4.715857911	5.28E-07	1.32E-06	down
ENSG00000185019	UBOX5	protein_coding	-4.715857911	5.28E-07	1.32E-06	down
ENSG00000132196	HSD17B7	protein_coding	1.097210662	5.55E-07	1.38E-06	up
ENSG00000106733	NMRK1	protein_coding	1.133140519	5.55E-07	1.38E-06	up
ENSG00000142733	MAP3K6	protein_coding	-1.13089541	5.56E-07	1.38E-06	down
ENSG00000131584	ACAP3	protein_coding	-1.092039474	5.81E-07	1.44E-06	down
ENSG00000138622	HCN4	protein_coding	-5.648743715	5.93E-07	1.46E-06	down
ENSG00000187688	TRPV2	protein_coding	-5.648743715	5.93E-07	1.46E-06	down
ENSG00000114812	VIPR1	protein_coding	-5.648743715	5.93E-07	1.46E-06	down
ENSG00000158315	RHBDL2	protein_coding	-5.648743715	5.93E-07	1.46E-06	down
ENSG00000183346	CABCOCO1	protein_coding	-5.648743715	5.93E-07	1.46E-06	down
ENSG00000134627	PIWIL4	protein_coding	-5.648743715	5.93E-07	1.46E-06	down
ENSG00000099957	P2RX6	protein_coding	-5.648743715	5.93E-07	1.46E-06	down
ENSG00000104043	ATP8B4	protein_coding	-5.648743715	5.93E-07	1.46E-06	down
ENSG00000128709	HOXD9	protein_coding	-5.648743715	5.93E-07	1.46E-06	down

ENSG00000163121	NEURL3	protein_coding	-5.648743715	5.93E-07	1.46E-06	down
ENSG00000163406	SLC15A2	protein_coding	-5.648743715	5.93E-07	1.46E-06	down
ENSG00000113361	CDH6	protein_coding	-5.648743715	5.93E-07	1.46E-06	down
ENSG00000182459	TEX19	protein_coding	-5.648743715	5.93E-07	1.46E-06	down
ENSG00000132622	HSPA12B	protein_coding	-5.648743715	5.93E-07	1.46E-06	down
ENSG00000260007	AC107871.1	protein_coding	-5.648743715	5.93E-07	1.46E-06	down
ENSG00000259330	INAFM2	protein_coding	-5.648743715	5.93E-07	1.46E-06	down
ENSG00000185274	GALNT17	protein_coding	-5.648743715	5.93E-07	1.46E-06	down
ENSG00000180044	C3orf80	protein_coding	-5.648743715	5.93E-07	1.46E-06	down
ENSG00000228623	ZNF883	protein_coding	-5.648743715	5.93E-07	1.46E-06	down
ENSG00000170961	HAS2	protein_coding	-5.648743715	5.93E-07	1.46E-06	down
ENSG00000155530	LRGUK	protein_coding	-5.648743715	5.93E-07	1.46E-06	down
ENSG00000180525	PRR26	protein_coding	-5.648743715	5.93E-07	1.46E-06	down
ENSG00000106852	LHX6	protein_coding	-5.648743715	5.93E-07	1.46E-06	down
ENSG00000101230	ISM1	protein_coding	-5.648743715	5.93E-07	1.46E-06	down
ENSG00000170469	SPATA24	protein_coding	-5.648743715	5.93E-07	1.46E-06	down
ENSG00000253293	HOXA10	protein_coding	-5.648743715	5.93E-07	1.46E-06	down
ENSG00000188001	TPRG1	protein_coding	2.55092863	6.00E-07	1.48E-06	up
ENSG00000204859	ZBTB48	protein_coding	-1.440850863	6.01E-07	1.48E-06	down
ENSG00000166401	SERPINB8	protein_coding	-1.307052365	6.02E-07	1.48E-06	down
ENSG00000179542	SLITRK4	protein_coding	3.650464303	6.06E-07	1.49E-06	up
ENSG00000164898	FMC1	protein_coding	3.650464303	6.06E-07	1.49E-06	up
ENSG00000100583	SAMD15	protein_coding	-1.18671732	6.20E-07	1.52E-06	down
ENSG00000174516	PELI3	protein_coding	-2.003660222	6.24E-07	1.53E-06	down
ENSG00000026297	RNASET2	protein_coding	1.702931723	6.27E-07	1.54E-06	up
ENSG00000166153	DEPDC4	protein_coding	1.702931723	6.27E-07	1.54E-06	up
ENSG00000186868	MAPT	protein_coding	1.147295894	6.41E-07	1.57E-06	up
ENSG00000136883	KIF12	protein_coding	1.925777039	6.46E-07	1.58E-06	up
ENSG00000213780	GTF2H4	protein_coding	5.387429897	6.53E-07	1.60E-06	up
ENSG00000129946	SHC2	protein_coding	5.387429897	6.53E-07	1.60E-06	up
ENSG00000187372	PCDHB13	protein_coding	5.387429897	6.53E-07	1.60E-06	up
ENSG00000274810	NPHP3-ACAD	protein_coding	5.387429897	6.53E-07	1.60E-06	up
ENSG00000268041	AC010616.1	protein_coding	5.387429897	6.53E-07	1.60E-06	up
ENSG00000165949	IFI27	protein_coding	5.387429897	6.53E-07	1.60E-06	up
ENSG00000204344	STK19	protein_coding	2.707047832	6.77E-07	1.65E-06	up
ENSG00000143416	SELENBP1	protein_coding	3.203005326	6.99E-07	1.70E-06	up
ENSG00000156968	MPV17L	protein_coding	-1.32681562	7.02E-07	1.71E-06	down
ENSG00000170689	HOXB9	protein_coding	-1.368901022	7.10E-07	1.73E-06	down
ENSG00000102362	SYTL4	protein_coding	-1.198710794	7.43E-07	1.81E-06	down
ENSG00000185437	SH3BGR	protein_coding	2.039029591	7.67E-07	1.87E-06	up
ENSG00000187210	GCNT1	protein_coding	1.314322349	7.77E-07	1.89E-06	up
ENSG00000160325	CACFD1	protein_coding	1.229000535	7.90E-07	1.92E-06	up
ENSG00000159884	CCDC107	protein_coding	-1.446250851	7.90E-07	1.92E-06	down
ENSG00000262814	MRPL12	protein_coding	-1.226052643	8.02E-07	1.95E-06	down
ENSG00000256294	ZNF225	protein_coding	-1.226052643	8.02E-07	1.95E-06	down
ENSG00000100564	PIGH	protein_coding	-1.545932909	8.11E-07	1.97E-06	down
ENSG00000162241	SLC25A45	protein_coding	-1.545932909	8.11E-07	1.97E-06	down
ENSG00000183032	SLC25A21	protein_coding	4.444013426	8.30E-07	2.01E-06	up
ENSG00000174564	IL20RB	protein_coding	4.444013426	8.30E-07	2.01E-06	up
ENSG00000123411	IKZF4	protein_coding	1.34348655	8.45E-07	2.05E-06	up
ENSG00000182010	RTKN2	protein_coding	1.065501803	8.49E-07	2.06E-06	up
ENSG00000165113	GKAP1	protein_coding	-1.008498779	8.71E-07	2.11E-06	down
ENSG00000169193	CCDC126	protein_coding	1.375841923	9.08E-07	2.20E-06	up
ENSG00000240204	SMKR1	protein_coding	-1.639754932	9.19E-07	2.22E-06	down

ENSG00000128604	IRF5	protein_coding	-1.37190351	9.37E-07	2.26E-06	down
ENSG00000172403	SYNPO2	protein_coding	-1.37190351	9.37E-07	2.26E-06	down
ENSG00000261221	ZNF865	protein_coding	-1.240658976	9.54E-07	2.30E-06	down
ENSG00000162642	C1orf52	protein_coding	-1.126365752	9.67E-07	2.33E-06	down
ENSG00000113966	ARL6	protein_coding	-1.273105033	1.01E-06	2.43E-06	down
ENSG00000235453	SMIM27	protein_coding	-4.648743715	1.02E-06	2.46E-06	down
ENSG00000204540	PSORS1C1	protein_coding	-4.648743715	1.02E-06	2.46E-06	down
ENSG00000119640	ACYP1	protein_coding	-1.350753675	1.07E-06	2.58E-06	down
ENSG00000253251	AC008560.1	protein_coding	-2.154546678	1.08E-06	2.60E-06	down
ENSG00000144596	GRIP2	protein_coding	-5.578354387	1.11E-06	2.64E-06	down
ENSG00000105472	CLEC11A	protein_coding	-5.578354387	1.11E-06	2.64E-06	down
ENSG00000197191	CYSRT1	protein_coding	-5.578354387	1.11E-06	2.64E-06	down
ENSG00000100351	GRAP2	protein_coding	-5.578354387	1.11E-06	2.64E-06	down
ENSG00000144214	LYG1	protein_coding	-5.578354387	1.11E-06	2.64E-06	down
ENSG00000060762	MPC1	protein_coding	-5.578354387	1.11E-06	2.64E-06	down
ENSG00000177409	SAMD9L	protein_coding	-5.578354387	1.11E-06	2.64E-06	down
ENSG00000104814	MAP4K1	protein_coding	-5.578354387	1.11E-06	2.64E-06	down
ENSG00000187140	FOXD3	protein_coding	-5.578354387	1.11E-06	2.64E-06	down
ENSG00000162927	PUS10	protein_coding	-5.578354387	1.11E-06	2.64E-06	down
ENSG00000166257	SCN3B	protein_coding	-5.578354387	1.11E-06	2.64E-06	down
ENSG00000117480	FAAH	protein_coding	-5.578354387	1.11E-06	2.64E-06	down
ENSG00000142538	PTH2	protein_coding	-5.578354387	1.11E-06	2.64E-06	down
ENSG00000186105	LRRC70	protein_coding	-5.578354387	1.11E-06	2.64E-06	down
ENSG00000161265	U2AF1L4	protein_coding	-5.578354387	1.11E-06	2.64E-06	down
ENSG00000125388	GRK4	protein_coding	-5.578354387	1.11E-06	2.64E-06	down
ENSG00000174428	GTF2IRD2B	protein_coding	-5.578354387	1.11E-06	2.64E-06	down
ENSG00000101605	MYOM1	protein_coding	-5.578354387	1.11E-06	2.64E-06	down
ENSG00000267221	C17orf113	protein_coding	-5.578354387	1.11E-06	2.64E-06	down
ENSG00000124216	SNAI1	protein_coding	5.328536208	1.11E-06	2.65E-06	up
ENSG00000009694	TENM1	protein_coding	5.328536208	1.11E-06	2.65E-06	up
ENSG00000116014	KISS1R	protein_coding	5.328536208	1.11E-06	2.65E-06	up
ENSG00000149781	FERMT3	protein_coding	5.328536208	1.11E-06	2.65E-06	up
ENSG00000241343	RPL36A	protein_coding	-1.104423199	1.14E-06	2.70E-06	down
ENSG00000188167	TMPPE	protein_coding	1.166785138	1.15E-06	2.73E-06	up
ENSG00000215784	FAM72D	protein_coding	-1.066948493	1.17E-06	2.76E-06	down
ENSG00000129295	LRRC6	protein_coding	3.158611207	1.20E-06	2.84E-06	up
ENSG00000139908	TSSK4	protein_coding	3.158611207	1.20E-06	2.84E-06	up
ENSG00000284526	AC015802.6	protein_coding	-1.240306627	1.26E-06	2.98E-06	down
ENSG00000180626	ZNF594	protein_coding	1.131499361	1.26E-06	2.99E-06	up
ENSG00000181350	LRRC75A	protein_coding	1.24836586	1.30E-06	3.06E-06	up
ENSG00000003147	ICA1	protein_coding	1.184146299	1.32E-06	3.10E-06	up
ENSG00000180914	OXTR	protein_coding	1.377445809	1.33E-06	3.14E-06	up
ENSG00000180806	HOXC9	protein_coding	-3.779988248	1.34E-06	3.15E-06	down
ENSG00000160408	ST6GALNAC6	protein_coding	-1.273499806	1.34E-06	3.15E-06	down
ENSG00000161996	WDR90	protein_coding	-1.566766413	1.38E-06	3.25E-06	down
ENSG00000136542	GALNT5	protein_coding	-1.310874076	1.39E-06	3.27E-06	down
ENSG00000203965	EFCAB7	protein_coding	1.41495096	1.41E-06	3.32E-06	up
ENSG00000165695	AK8	protein_coding	4.387429897	1.45E-06	3.41E-06	up
ENSG00000165983	PTER	protein_coding	-1.030544885	1.53E-06	3.59E-06	down
ENSG00000109323	MANBA	protein_coding	-1.133278062	1.55E-06	3.63E-06	down
ENSG00000151687	ANKAR	protein_coding	1.020801995	1.55E-06	3.64E-06	up
ENSG00000165795	NDRG2	protein_coding	1.154506809	1.57E-06	3.67E-06	up
ENSG00000196911	KPNA5	protein_coding	-1.021961039	1.63E-06	3.81E-06	down
ENSG00000105696	TMEM59L	protein_coding	-3.315319981	1.66E-06	3.87E-06	down

ENSG00000176125	UFSP1	protein_coding	-3.315319981	1.66E-06	3.87E-06	down
ENSG00000104998	IL27RA	protein_coding	1.23456006	1.80E-06	4.19E-06	up
ENSG00000188542	DUSP28	protein_coding	-1.464534488	1.80E-06	4.19E-06	down
ENSG00000166136	NDUFB8	protein_coding	-1.464534488	1.80E-06	4.19E-06	down
ENSG00000181690	PLAG1	protein_coding	1.344477752	1.82E-06	4.24E-06	up
ENSG00000072952	MRVI1	protein_coding	3.55092863	1.84E-06	4.28E-06	up
ENSG00000092036	HAUS4	protein_coding	-1.312279527	1.84E-06	4.29E-06	down
ENSG00000165124	SVEP1	protein_coding	1.126902347	1.84E-06	4.29E-06	up
ENSG00000253250	C8orf88	protein_coding	-3.011313794	1.87E-06	4.35E-06	down
ENSG00000011021	CLCN6	protein_coding	-1.143215682	1.88E-06	4.37E-06	down
ENSG00000119919	NKX2-3	protein_coding	5.267135664	1.90E-06	4.42E-06	up
ENSG00000188199	NUTM2B	protein_coding	5.267135664	1.90E-06	4.42E-06	up
ENSG00000255346	NOX5	protein_coding	5.267135664	1.90E-06	4.42E-06	up
ENSG00000166342	NETO1	protein_coding	5.267135664	1.90E-06	4.42E-06	up
ENSG00000259224	SLC35G6	protein_coding	5.267135664	1.90E-06	4.42E-06	up
ENSG00000243789	JMJD7	protein_coding	5.267135664	1.90E-06	4.42E-06	up
ENSG00000157870	FAM213B	protein_coding	-1.841388793	1.93E-06	4.48E-06	down
ENSG00000127415	IDUA	protein_coding	-4.578354387	1.99E-06	4.60E-06	down
ENSG00000213397	HAUS7	protein_coding	2.831036549	2.06E-06	4.76E-06	up
ENSG00000164142	FAM160A1	protein_coding	2.831036549	2.06E-06	4.76E-06	up
ENSG00000128573	FOXP2	protein_coding	2.831036549	2.06E-06	4.76E-06	up
ENSG00000213199	ASIC3	protein_coding	-5.504353806	2.08E-06	4.79E-06	down
ENSG00000237515	SHISA9	protein_coding	-5.504353806	2.08E-06	4.79E-06	down
ENSG00000188316	ENO4	protein_coding	-5.504353806	2.08E-06	4.79E-06	down
ENSG00000115423	DNAH6	protein_coding	-5.504353806	2.08E-06	4.79E-06	down
ENSG00000188394	GPR21	protein_coding	-5.504353806	2.08E-06	4.79E-06	down
ENSG00000091128	LAMB4	protein_coding	-5.504353806	2.08E-06	4.79E-06	down
ENSG00000101098	RIMS4	protein_coding	-5.504353806	2.08E-06	4.79E-06	down
ENSG00000173210	ABLIM3	protein_coding	-5.504353806	2.08E-06	4.79E-06	down
ENSG00000173809	TDRD12	protein_coding	-5.504353806	2.08E-06	4.79E-06	down
ENSG00000134917	ADAMTS8	protein_coding	-5.504353806	2.08E-06	4.79E-06	down
ENSG00000166592	RRAD	protein_coding	-5.504353806	2.08E-06	4.79E-06	down
ENSG00000171056	SOX7	protein_coding	-5.504353806	2.08E-06	4.79E-06	down
ENSG00000204642	HLA-F	protein_coding	-5.504353806	2.08E-06	4.79E-06	down
ENSG00000204767	FAM196B	protein_coding	-5.504353806	2.08E-06	4.79E-06	down
ENSG00000149380	P4HA3	protein_coding	-5.504353806	2.08E-06	4.79E-06	down
ENSG00000198453	ZNF568	protein_coding	-5.504353806	2.08E-06	4.79E-06	down
ENSG00000204287	HLA-DRA	protein_coding	-5.504353806	2.08E-06	4.79E-06	down
ENSG00000152580	IGSF10	protein_coding	1.065501803	2.09E-06	4.79E-06	up
ENSG00000102359	SRPX2	protein_coding	1.571392732	2.17E-06	4.97E-06	up
ENSG00000160191	PDE9A	protein_coding	1.199638475	2.19E-06	5.02E-06	up
ENSG00000160352	ZNF714	protein_coding	-1.041413401	2.31E-06	5.29E-06	down
ENSG00000250067	YJEFN3	protein_coding	1.01920815	2.43E-06	5.56E-06	up
ENSG00000147813	NAPRT	protein_coding	-1.223259428	2.46E-06	5.61E-06	down
ENSG00000182575	NXPH3	protein_coding	4.328536208	2.54E-06	5.79E-06	up
ENSG00000168000	BSCL2	protein_coding	1.328536208	2.56E-06	5.84E-06	up
ENSG00000143554	SLC27A3	protein_coding	-3.715857911	2.60E-06	5.92E-06	down
ENSG00000167614	TTYH1	protein_coding	-3.715857911	2.60E-06	5.92E-06	down
ENSG00000126368	NR1D1	protein_coding	1.231860189	2.63E-06	5.98E-06	up
ENSG00000089091	DZANK1	protein_coding	1.122085331	2.68E-06	6.09E-06	up
ENSG00000162194	LBHD1	protein_coding	1.743573708	2.73E-06	6.20E-06	up
ENSG00000146859	TMEM140	protein_coding	1.697770018	2.88E-06	6.53E-06	up
ENSG00000257704	INAFM1	protein_coding	-2.343889134	2.94E-06	6.67E-06	down
ENSG00000183155	RABIF	protein_coding	-1.192295955	3.00E-06	6.80E-06	down

ENSG00000109339	MAPK10	protein_coding	1.444013426	3.09E-06	6.99E-06	up
ENSG00000204444	APOM	protein_coding	2.591570614	3.14E-06	7.08E-06	up
ENSG00000149798	CDC42EP2	protein_coding	-1.104423199	3.18E-06	7.17E-06	down
ENSG00000107902	LHPP	protein_coding	1.55092863	3.18E-06	7.17E-06	up
ENSG00000105255	FSD1	protein_coding	-3.256426292	3.19E-06	7.19E-06	down
ENSG00000166262	FAM227B	protein_coding	-3.256426292	3.19E-06	7.19E-06	down
ENSG00000142632	ARHGEF19	protein_coding	-3.256426292	3.19E-06	7.19E-06	down
ENSG00000185055	EFCAB10	protein_coding	-3.256426292	3.19E-06	7.19E-06	down
ENSG00000145022	TCTA	protein_coding	-2.082396893	3.25E-06	7.33E-06	down
ENSG00000164683	HEY1	protein_coding	5.203005326	3.27E-06	7.35E-06	up
ENSG00000123572	NRK	protein_coding	5.203005326	3.27E-06	7.35E-06	up
ENSG00000141485	SLC13A5	protein_coding	5.203005326	3.27E-06	7.35E-06	up
ENSG00000165617	DACT1	protein_coding	5.203005326	3.27E-06	7.35E-06	up
ENSG00000111058	ACSS3	protein_coding	5.203005326	3.27E-06	7.35E-06	up
ENSG00000170379	TCAF2	protein_coding	5.203005326	3.27E-06	7.35E-06	up
ENSG00000183208	GDPGP1	protein_coding	2.097210662	3.39E-06	7.60E-06	up
ENSG00000253953	PCDHGB4	protein_coding	1.297172038	3.44E-06	7.72E-06	up
ENSG00000187566	NHLRC1	protein_coding	-2.578354387	3.52E-06	7.89E-06	down
ENSG00000187554	TLR5	protein_coding	2.29377079	3.54E-06	7.93E-06	up
ENSG00000205220	PSMB10	protein_coding	-2.741853119	3.63E-06	8.11E-06	down
ENSG00000138356	AOX1	protein_coding	-4.504353806	3.87E-06	8.63E-06	down
ENSG00000162654	GBP4	protein_coding	-4.504353806	3.87E-06	8.63E-06	down
ENSG00000204179	PTPN20	protein_coding	-4.504353806	3.87E-06	8.63E-06	down
ENSG00000206052	DOK6	protein_coding	-2.143951563	3.88E-06	8.65E-06	down
ENSG00000152669	CCNO	protein_coding	1.7691088	3.88E-06	8.65E-06	up
ENSG00000130783	CCDC62	protein_coding	-5.426351294	3.92E-06	8.70E-06	down
ENSG00000164061	BSN	protein_coding	-5.426351294	3.92E-06	8.70E-06	down
ENSG00000188033	ZNF490	protein_coding	-5.426351294	3.92E-06	8.70E-06	down
ENSG00000173335	CST9	protein_coding	-5.426351294	3.92E-06	8.70E-06	down
ENSG00000197363	ZNF517	protein_coding	-5.426351294	3.92E-06	8.70E-06	down
ENSG00000173947	PIFO	protein_coding	-5.426351294	3.92E-06	8.70E-06	down
ENSG00000134548	SPX	protein_coding	-5.426351294	3.92E-06	8.70E-06	down
ENSG00000120075	HOXB5	protein_coding	-5.426351294	3.92E-06	8.70E-06	down
ENSG00000120278	PLEKHG1	protein_coding	-5.426351294	3.92E-06	8.70E-06	down
ENSG00000153237	CCDC148	protein_coding	-5.426351294	3.92E-06	8.70E-06	down
ENSG00000138172	CALHM2	protein_coding	-5.426351294	3.92E-06	8.70E-06	down
ENSG00000162670	BRINP3	protein_coding	-5.426351294	3.92E-06	8.70E-06	down
ENSG00000137699	TRIM29	protein_coding	-5.426351294	3.92E-06	8.70E-06	down
ENSG00000231887	PRH1	protein_coding	-5.426351294	3.92E-06	8.70E-06	down
ENSG00000107518	ATRNL1	protein_coding	-5.426351294	3.92E-06	8.70E-06	down
ENSG00000142694	EVA1B	protein_coding	-5.426351294	3.92E-06	8.70E-06	down
ENSG00000166922	SCG5	protein_coding	-5.426351294	3.92E-06	8.70E-06	down
ENSG00000087494	PTHLH	protein_coding	-5.426351294	3.92E-06	8.70E-06	down
ENSG00000087589	CASS4	protein_coding	-5.426351294	3.92E-06	8.70E-06	down
ENSG00000253910	PCDHGB2	protein_coding	-5.426351294	3.92E-06	8.70E-06	down
ENSG00000198807	PAX9	protein_coding	-5.426351294	3.92E-06	8.70E-06	down
ENSG00000135835	KIAA1614	protein_coding	-5.426351294	3.92E-06	8.70E-06	down
ENSG00000096088	PGC	protein_coding	-5.426351294	3.92E-06	8.70E-06	down
ENSG00000164099	PRSS12	protein_coding	-1.618996372	3.95E-06	8.73E-06	down
ENSG00000164308	ERAP2	protein_coding	-1.841388793	4.04E-06	8.92E-06	down
ENSG00000067606	PRKCZ	protein_coding	-1.024610617	4.25E-06	9.38E-06	down
ENSG00000049192	ADAMTS6	protein_coding	2.40653872	4.38E-06	9.65E-06	up
ENSG00000184185	KCNJ12	protein_coding	2.40653872	4.38E-06	9.65E-06	up
ENSG00000135363	LMO2	protein_coding	4.267135664	4.45E-06	9.79E-06	up

ENSG00000145506	NKD2	protein_coding	2.169838462	4.45E-06	9.79E-06	up
ENSG00000073150	PANX2	protein_coding	-1.578354387	4.84E-06	1.06E-05	down
ENSG00000147118	ZNF182	protein_coding	-1.07842799	4.88E-06	1.07E-05	down
ENSG00000169740	ZNF32	protein_coding	-1.34131519	4.95E-06	1.09E-05	down
ENSG00000119725	ZNF410	protein_coding	-3.648743715	5.08E-06	1.11E-05	down
ENSG00000185386	MAPK11	protein_coding	-3.648743715	5.08E-06	1.11E-05	down
ENSG00000114405	C3orf14	protein_coding	-1.238278946	5.13E-06	1.12E-05	down
ENSG00000175768	TOMM5	protein_coding	-1.098361446	5.53E-06	1.21E-05	down
ENSG00000103485	QPRT	protein_coding	3.444013426	5.58E-06	1.22E-05	up
ENSG00000118640	VAMP8	protein_coding	-2.044922187	5.60E-06	1.22E-05	down
ENSG00000168477	TNXB	protein_coding	5.13589113	5.62E-06	1.22E-05	up
ENSG00000069812	HES2	protein_coding	5.13589113	5.62E-06	1.22E-05	up
ENSG00000231672	DIRC3	protein_coding	5.13589113	5.62E-06	1.22E-05	up
ENSG00000166863	TAC3	protein_coding	5.13589113	5.62E-06	1.22E-05	up
ENSG00000230510	PPP5D1	protein_coding	5.13589113	5.62E-06	1.22E-05	up
ENSG00000149527	PLCH2	protein_coding	5.13589113	5.62E-06	1.22E-05	up
ENSG00000155085	AK9	protein_coding	1.111724819	5.68E-06	1.23E-05	up
ENSG00000007866	TEAD3	protein_coding	-1.133569544	5.72E-06	1.24E-05	down
ENSG00000148950	IMMP1L	protein_coding	-1.173964132	5.79E-06	1.26E-05	down
ENSG00000141933	TPGS1	protein_coding	-1.173964132	5.79E-06	1.26E-05	down
ENSG00000174370	C11orf45	protein_coding	-1.173964132	5.79E-06	1.26E-05	down
ENSG00000174586	ZNF497	protein_coding	-1.540219258	5.86E-06	1.27E-05	down
ENSG00000171126	KCNG3	protein_coding	3.016592202	6.06E-06	1.31E-05	up
ENSG00000185332	TMEM105	protein_coding	-3.195025748	6.12E-06	1.32E-05	down
ENSG00000146411	SLC2A12	protein_coding	1.694664107	6.19E-06	1.34E-05	up
ENSG00000182208	MOB2	protein_coding	-1.120364743	6.22E-06	1.34E-05	down
ENSG00000162620	LRRIQ3	protein_coding	1.40653872	6.36E-06	1.37E-05	up
ENSG00000185261	KIAA0825	protein_coding	1.40653872	6.36E-06	1.37E-05	up
ENSG00000102128	RAB40AL	protein_coding	1.650464303	6.43E-06	1.39E-05	up
ENSG00000171174	RBKS	protein_coding	1.084610626	6.55E-06	1.41E-05	up
ENSG00000184545	DUSP8	protein_coding	1.610307177	6.61E-06	1.42E-05	up
ENSG00000171346	KRT15	protein_coding	1.265110828	6.71E-06	1.44E-05	up
ENSG00000243725	TTC4	protein_coding	1.898851933	7.02E-06	1.51E-05	up
ENSG00000160360	GPSM1	protein_coding	1.025344676	7.11E-06	1.53E-05	up
ENSG00000105409	ATP1A3	protein_coding	1.294983649	7.43E-06	1.59E-05	up
ENSG00000213023	SYT3	protein_coding	-5.343889134	7.43E-06	1.59E-05	down
ENSG00000213204	AL049697.1	protein_coding	-5.343889134	7.43E-06	1.59E-05	down
ENSG00000203985	LDLRAD1	protein_coding	-5.343889134	7.43E-06	1.59E-05	down
ENSG00000124602	UNC5CL	protein_coding	-5.343889134	7.43E-06	1.59E-05	down
ENSG00000007171	NOS2	protein_coding	-5.343889134	7.43E-06	1.59E-05	down
ENSG00000158683	PKD1L1	protein_coding	-5.343889134	7.43E-06	1.59E-05	down
ENSG00000222047	C10orf55	protein_coding	-5.343889134	7.43E-06	1.59E-05	down
ENSG00000138347	MYPN	protein_coding	-5.343889134	7.43E-06	1.59E-05	down
ENSG00000271092	TMEM56-RWI	protein_coding	-5.343889134	7.43E-06	1.59E-05	down
ENSG00000137265	IRF4	protein_coding	-5.343889134	7.43E-06	1.59E-05	down
ENSG00000186188	FFAR4	protein_coding	-5.343889134	7.43E-06	1.59E-05	down
ENSG00000280109	PLAC4	protein_coding	-5.343889134	7.43E-06	1.59E-05	down
ENSG00000182557	SPNS3	protein_coding	-5.343889134	7.43E-06	1.59E-05	down
ENSG00000259529	AL136295.5	protein_coding	-5.343889134	7.43E-06	1.59E-05	down
ENSG00000175874	CREG2	protein_coding	-5.343889134	7.43E-06	1.59E-05	down
ENSG00000268104	SLC6A14	protein_coding	-5.343889134	7.43E-06	1.59E-05	down
ENSG00000214193	SH3D21	protein_coding	-5.343889134	7.43E-06	1.59E-05	down
ENSG00000179846	NKPD1	protein_coding	-5.343889134	7.43E-06	1.59E-05	down
ENSG00000116774	OLFML3	protein_coding	-5.343889134	7.43E-06	1.59E-05	down

ENSG00000184507	NUTM1	protein_coding	-5.343889134	7.43E-06	1.59E-05	down
ENSG00000111664	GNB3	protein_coding	-5.343889134	7.43E-06	1.59E-05	down
ENSG00000243709	LEFTY1	protein_coding	-5.343889134	7.43E-06	1.59E-05	down
ENSG00000100299	ARSA	protein_coding	-1.219900416	7.55E-06	1.61E-05	down
ENSG00000057657	PRDM1	protein_coding	1.831036549	7.73E-06	1.64E-05	up
ENSG00000105583	WDR83OS	protein_coding	-1.007066823	7.79E-06	1.66E-05	down
ENSG00000100100	PIK3IP1	protein_coding	4.203005326	7.81E-06	1.66E-05	up
ENSG00000143816	WNT9A	protein_coding	-2.173964132	8.00E-06	1.70E-05	down
ENSG00000184005	ST6GALNAC3	protein_coding	-2.173964132	8.00E-06	1.70E-05	down
ENSG00000100294	MCAT	protein_coding	-1.256426292	8.07E-06	1.71E-05	down
ENSG00000173376	NDNF	protein_coding	2.033080325	8.43E-06	1.79E-05	up
ENSG00000125319	C17orf53	protein_coding	-1.298246468	8.46E-06	1.79E-05	down
ENSG00000167920	TMEM99	protein_coding	-1.403267681	8.65E-06	1.83E-05	down
ENSG00000163682	RPL9	protein_coding	-1.142255272	9.17E-06	1.94E-05	down
ENSG00000132004	FBXW9	protein_coding	-2.256426292	9.31E-06	1.96E-05	down
ENSG00000078687	TNRC6C	protein_coding	-1.276890395	9.54E-06	2.01E-05	down
ENSG00000169981	ZNF35	protein_coding	-1.276890395	9.54E-06	2.01E-05	down
ENSG00000163071	SPATA18	protein_coding	1.433889209	9.56E-06	2.01E-05	up
ENSG00000275464	FP565260.1	protein_coding	1.945207569	9.69E-06	2.04E-05	up
ENSG00000164690	SHH	protein_coding	5.065501803	9.70E-06	2.04E-05	up
ENSG00000187486	KCNJ11	protein_coding	5.065501803	9.70E-06	2.04E-05	up
ENSG00000187642	PERM1	protein_coding	5.065501803	9.70E-06	2.04E-05	up
ENSG00000197479	PCDHB11	protein_coding	5.065501803	9.70E-06	2.04E-05	up
ENSG00000205978	NYNRIN	protein_coding	5.065501803	9.70E-06	2.04E-05	up
ENSG00000149418	ST14	protein_coding	5.065501803	9.70E-06	2.04E-05	up
ENSG00000169026	MFSD7	protein_coding	5.065501803	9.70E-06	2.04E-05	up
ENSG00000072182	ASIC4	protein_coding	5.065501803	9.70E-06	2.04E-05	up
ENSG00000095587	TLL2	protein_coding	3.387429897	9.71E-06	2.04E-05	up
ENSG00000164330	EBF1	protein_coding	-1.438629624	9.80E-06	2.06E-05	down
ENSG00000158987	RAPGEF6	protein_coding	-1.218951587	1.00E-05	2.10E-05	down
ENSG00000280670	CCDC163	protein_coding	2.965966129	1.04E-05	2.17E-05	up
ENSG00000160207	HSF2BP	protein_coding	2.965966129	1.04E-05	2.17E-05	up
ENSG00000151790	TDO2	protein_coding	-2.355961966	1.06E-05	2.22E-05	down
ENSG00000111215	PRR4	protein_coding	-1.877914669	1.06E-05	2.22E-05	down
ENSG00000151881	TMEM267	protein_coding	-1.623208623	1.07E-05	2.23E-05	down
ENSG00000242259	C22orf39	protein_coding	-1.256426292	1.07E-05	2.24E-05	down
ENSG00000121900	TMEM54	protein_coding	-1.775800451	1.11E-05	2.31E-05	down
ENSG00000062370	ZNF112	protein_coding	1.092723271	1.11E-05	2.33E-05	up
ENSG00000135697	BCO1	protein_coding	-1.067664649	1.12E-05	2.33E-05	down
ENSG00000154556	SORBS2	protein_coding	2.101125712	1.13E-05	2.35E-05	up
ENSG00000167971	CASKIN1	protein_coding	-2.063781214	1.17E-05	2.43E-05	down
ENSG00000100263	RHBDD3	protein_coding	-1.017639433	1.19E-05	2.47E-05	down
ENSG00000198478	SH3BGRL2	protein_coding	-1.445460117	1.29E-05	2.68E-05	down
ENSG00000179361	ARID3B	protein_coding	1.232320893	1.29E-05	2.69E-05	up
ENSG00000103196	CRISPLD2	protein_coding	-1.919391305	1.33E-05	2.75E-05	down
ENSG00000222009	BTBD19	protein_coding	1.389936753	1.33E-05	2.77E-05	up
ENSG00000010361	FUZ	protein_coding	1.006608114	1.36E-05	2.81E-05	up
ENSG00000111850	SMIM8	protein_coding	1.413425106	1.37E-05	2.84E-05	up
ENSG00000105605	CACNG7	protein_coding	4.13589113	1.37E-05	2.85E-05	up
ENSG00000183828	NUDT14	protein_coding	-1.806623375	1.41E-05	2.91E-05	down
ENSG00000178809	TRIM73	protein_coding	-5.256426292	1.42E-05	2.91E-05	down
ENSG00000213865	C8orf44	protein_coding	-5.256426292	1.42E-05	2.91E-05	down
ENSG00000163794	UCN	protein_coding	-5.256426292	1.42E-05	2.91E-05	down
ENSG00000100626	GALNT16	protein_coding	-5.256426292	1.42E-05	2.91E-05	down

ENSG00000157884	CIB4	protein_coding	-5.256426292	1.42E-05	2.91E-05	down
ENSG00000167588	GPD1	protein_coding	-5.256426292	1.42E-05	2.91E-05	down
ENSG00000153064	BANK1	protein_coding	-5.256426292	1.42E-05	2.91E-05	down
ENSG00000070669	ASNS	protein_coding	-5.256426292	1.42E-05	2.91E-05	down
ENSG00000162444	RBP7	protein_coding	-5.256426292	1.42E-05	2.91E-05	down
ENSG00000172123	SLFN12	protein_coding	-5.256426292	1.42E-05	2.91E-05	down
ENSG00000147437	GNRH1	protein_coding	-5.256426292	1.42E-05	2.91E-05	down
ENSG00000142185	TRPM2	protein_coding	-5.256426292	1.42E-05	2.91E-05	down
ENSG00000103154	NECAB2	protein_coding	-5.256426292	1.42E-05	2.91E-05	down
ENSG00000136305	CIDEB	protein_coding	-5.256426292	1.42E-05	2.91E-05	down
ENSG00000077616	NAALAD2	protein_coding	-5.256426292	1.42E-05	2.91E-05	down
ENSG00000185664	PMEL	protein_coding	-5.256426292	1.42E-05	2.91E-05	down
ENSG00000204839	MROH6	protein_coding	-5.256426292	1.42E-05	2.91E-05	down
ENSG00000189184	PCDH18	protein_coding	-5.256426292	1.42E-05	2.91E-05	down
ENSG00000165591	FAAH2	protein_coding	-5.256426292	1.42E-05	2.91E-05	down
ENSG00000116690	PRG4	protein_coding	-5.256426292	1.42E-05	2.91E-05	down
ENSG00000125650	PSPN	protein_coding	-5.256426292	1.42E-05	2.91E-05	down
ENSG00000160200	CBS	protein_coding	-5.256426292	1.42E-05	2.91E-05	down
ENSG00000135549	PKIB	protein_coding	-5.256426292	1.42E-05	2.91E-05	down
ENSG00000027869	SH2D2A	protein_coding	-5.256426292	1.42E-05	2.91E-05	down
ENSG00000145087	STXBP5L	protein_coding	-5.256426292	1.42E-05	2.91E-05	down
ENSG00000169247	SH3TC2	protein_coding	-5.256426292	1.42E-05	2.91E-05	down
ENSG00000181790	ADGRB1	protein_coding	2.466039732	1.42E-05	2.91E-05	up
ENSG00000126453	BCL2L12	protein_coding	-1.111228377	1.43E-05	2.94E-05	down
ENSG00000144061	NPHP1	protein_coding	-1.487751838	1.44E-05	2.95E-05	down
ENSG00000171160	MORN4	protein_coding	1.260632144	1.45E-05	2.97E-05	up
ENSG00000158164	TMSB15A	protein_coding	2.184146299	1.48E-05	3.03E-05	up
ENSG00000188761	BCL2L15	protein_coding	-4.343889134	1.48E-05	3.03E-05	down
ENSG00000142623	PADI1	protein_coding	-4.343889134	1.48E-05	3.03E-05	down
ENSG00000131979	GCH1	protein_coding	1.046136478	1.57E-05	3.20E-05	up
ENSG00000135587	SMPD2	protein_coding	1.046136478	1.57E-05	3.20E-05	up
ENSG00000205593	DENND6B	protein_coding	1.838730941	1.66E-05	3.39E-05	up
ENSG00000081237	PTPRC	protein_coding	4.991501221	1.68E-05	3.42E-05	up
ENSG00000101134	DOK5	protein_coding	4.991501221	1.68E-05	3.42E-05	up
ENSG00000197361	FBXL22	protein_coding	4.991501221	1.68E-05	3.42E-05	up
ENSG00000104413	ESRP1	protein_coding	4.991501221	1.68E-05	3.42E-05	up
ENSG00000153896	ZNF599	protein_coding	4.991501221	1.68E-05	3.42E-05	up
ENSG00000171954	CYP4F22	protein_coding	4.991501221	1.68E-05	3.42E-05	up
ENSG00000112599	GUCA1B	protein_coding	4.991501221	1.68E-05	3.42E-05	up
ENSG00000253485	PCDHGA5	protein_coding	4.991501221	1.68E-05	3.42E-05	up
ENSG00000243284	VSIG8	protein_coding	4.991501221	1.68E-05	3.42E-05	up
ENSG00000138449	SLC40A1	protein_coding	1.30992053	1.68E-05	3.42E-05	up
ENSG00000164161	HHIP	protein_coding	3.328536208	1.69E-05	3.43E-05	up
ENSG00000170160	CCDC144A	protein_coding	-1.452823505	1.70E-05	3.46E-05	down
ENSG00000115255	REEP6	protein_coding	1.094070955	1.75E-05	3.56E-05	up
ENSG00000130653	PNPLA7	protein_coding	-1.216897928	1.76E-05	3.57E-05	down
ENSG00000115361	ACADL	protein_coding	2.913498709	1.77E-05	3.58E-05	up
ENSG00000106772	PRUNE2	protein_coding	2.913498709	1.77E-05	3.58E-05	up
ENSG00000141665	FBXO15	protein_coding	2.913498709	1.77E-05	3.58E-05	up
ENSG00000170364	SETMAR	protein_coding	-1.256426292	1.88E-05	3.81E-05	down
ENSG00000101213	PTK6	protein_coding	-3.504353806	1.93E-05	3.90E-05	down
ENSG00000145335	SNCA	protein_coding	-3.504353806	1.93E-05	3.90E-05	down
ENSG00000130720	FIBCD1	protein_coding	-1.094154863	2.05E-05	4.14E-05	down
ENSG00000112357	PEX7	protein_coding	1.618042826	2.06E-05	4.15E-05	up

ENSG00000115232	ITGA4	protein_coding	1.965966129	2.07E-05	4.18E-05	up
ENSG00000100592	DAAM1	protein_coding	-1.04783967	2.08E-05	4.19E-05	down
ENSG00000171121	KCNMB3	protein_coding	-2.426351294	2.13E-05	4.29E-05	down
ENSG00000106588	PSMA2	protein_coding	-1.180477439	2.15E-05	4.32E-05	down
ENSG00000197050	ZNF420	protein_coding	1.174913019	2.16E-05	4.35E-05	up
ENSG00000241399	CD302	protein_coding	-1.080854728	2.21E-05	4.45E-05	down
ENSG00000156453	PCDH1	protein_coding	-3.063781214	2.25E-05	4.52E-05	down
ENSG00000105991	HOXA1	protein_coding	-2.578354387	2.30E-05	4.62E-05	down
ENSG00000278570	NR2E3	protein_coding	-2.578354387	2.30E-05	4.62E-05	down
ENSG00000197062	ZSCAN26	protein_coding	-1.118922769	2.31E-05	4.64E-05	down
ENSG00000106333	PCOLCE	protein_coding	4.065501803	2.42E-05	4.85E-05	up
ENSG00000131848	ZSCAN5A	protein_coding	-1.090416341	2.71E-05	5.41E-05	down
ENSG00000154096	THY1	protein_coding	-5.163316888	2.71E-05	5.39E-05	down
ENSG00000213462	ERV3-1	protein_coding	-5.163316888	2.71E-05	5.39E-05	down
ENSG00000105649	RAB3A	protein_coding	-5.163316888	2.71E-05	5.39E-05	down
ENSG00000105982	RNF32	protein_coding	-5.163316888	2.71E-05	5.39E-05	down
ENSG00000101017	CD40	protein_coding	-5.163316888	2.71E-05	5.39E-05	down
ENSG00000187699	C2orf88	protein_coding	-5.163316888	2.71E-05	5.39E-05	down
ENSG00000242110	AMACR	protein_coding	-5.163316888	2.71E-05	5.39E-05	down
ENSG00000158427	TMSB15B	protein_coding	-5.163316888	2.71E-05	5.39E-05	down
ENSG00000075886	TUBA3D	protein_coding	-5.163316888	2.71E-05	5.39E-05	down
ENSG00000109846	CRYAB	protein_coding	-5.163316888	2.71E-05	5.39E-05	down
ENSG00000183722	LHFPL6	protein_coding	-5.163316888	2.71E-05	5.39E-05	down
ENSG00000157613	CREB3L1	protein_coding	-5.163316888	2.71E-05	5.39E-05	down
ENSG00000133962	CATSPERB	protein_coding	-5.163316888	2.71E-05	5.39E-05	down
ENSG00000187094	CCK	protein_coding	-5.163316888	2.71E-05	5.39E-05	down
ENSG00000127928	GNGT1	protein_coding	-5.163316888	2.71E-05	5.39E-05	down
ENSG00000137393	RNF144B	protein_coding	-5.163316888	2.71E-05	5.39E-05	down
ENSG00000196218	RYR1	protein_coding	-5.163316888	2.71E-05	5.39E-05	down
ENSG00000186952	TMEM232	protein_coding	-5.163316888	2.71E-05	5.39E-05	down
ENSG00000221818	EBF2	protein_coding	-5.163316888	2.71E-05	5.39E-05	down
ENSG00000172594	SMPDL3A	protein_coding	-5.163316888	2.71E-05	5.39E-05	down
ENSG00000133083	DCLK1	protein_coding	-5.163316888	2.71E-05	5.39E-05	down
ENSG00000196724	ZNF418	protein_coding	-5.163316888	2.71E-05	5.39E-05	down
ENSG00000196793	ZNF239	protein_coding	-5.163316888	2.71E-05	5.39E-05	down
ENSG00000058673	ZC3H11A	protein_coding	-5.163316888	2.71E-05	5.39E-05	down
ENSG00000103056	SMPD3	protein_coding	-5.163316888	2.71E-05	5.39E-05	down
ENSG00000176358	TAC4	protein_coding	-5.163316888	2.71E-05	5.39E-05	down
ENSG00000175697	GPR156	protein_coding	-5.163316888	2.71E-05	5.39E-05	down
ENSG00000102890	ELMO3	protein_coding	-5.163316888	2.71E-05	5.39E-05	down
ENSG00000180953	ST20	protein_coding	-5.163316888	2.71E-05	5.39E-05	down
ENSG00000174951	FUT1	protein_coding	-5.163316888	2.71E-05	5.39E-05	down
ENSG00000111405	ENDOU	protein_coding	-5.163316888	2.71E-05	5.39E-05	down
ENSG00000170049	KCNAB3	protein_coding	-5.163316888	2.71E-05	5.39E-05	down
ENSG00000159753	CARMIL2	protein_coding	-5.163316888	2.71E-05	5.39E-05	down
ENSG00000184786	TCTE3	protein_coding	-5.163316888	2.71E-05	5.39E-05	down
ENSG00000159708	LRRC36	protein_coding	-5.163316888	2.71E-05	5.39E-05	down
ENSG00000169228	RAB24	protein_coding	-5.163316888	2.71E-05	5.39E-05	down
ENSG00000204323	SMIM5	protein_coding	-5.163316888	2.71E-05	5.39E-05	down
ENSG00000196502	SULT1A1	protein_coding	1.743573708	2.73E-05	5.39E-05	up
ENSG00000106415	GLCCI1	protein_coding	1.019904936	2.77E-05	5.46E-05	up
ENSG00000176170	SPHK1	protein_coding	-1.62839507	2.89E-05	5.69E-05	down
ENSG00000188897	AC099489.1	protein_coding	-4.256426292	2.91E-05	5.72E-05	down
ENSG00000144668	ITGA9	protein_coding	4.913498709	2.92E-05	5.74E-05	up

ENSG00000262576	PCDHGA4	protein_coding	4.913498709	2.92E-05	5.74E-05	up
ENSG00000140379	BCL2A1	protein_coding	4.913498709	2.92E-05	5.74E-05	up
ENSG00000099958	DERL3	protein_coding	4.913498709	2.92E-05	5.74E-05	up
ENSG00000162687	KCNT2	protein_coding	4.913498709	2.92E-05	5.74E-05	up
ENSG00000215612	HMX1	protein_coding	4.913498709	2.92E-05	5.74E-05	up
ENSG00000137098	SPAG8	protein_coding	4.913498709	2.92E-05	5.74E-05	up
ENSG00000146151	HMGCLL1	protein_coding	4.913498709	2.92E-05	5.74E-05	up
ENSG00000145911	N4BP3	protein_coding	4.913498709	2.92E-05	5.74E-05	up
ENSG00000164932	CTHRC1	protein_coding	4.913498709	2.92E-05	5.74E-05	up
ENSG00000259316	AC087632.1	protein_coding	3.267135664	2.93E-05	5.75E-05	up
ENSG00000166923	GREM1	protein_coding	-1.803914088	2.96E-05	5.80E-05	down
ENSG00000144730	IL17RD	protein_coding	1.421645613	2.97E-05	5.82E-05	up
ENSG00000105717	PBX4	protein_coding	1.449842505	3.03E-05	5.93E-05	up
ENSG00000186684	CYP27C1	protein_coding	1.480539302	3.07E-05	6.01E-05	up
ENSG00000237489	C10orf143	protein_coding	1.55092863	3.08E-05	6.03E-05	up
ENSG00000111653	ING4	protein_coding	1.169838462	3.17E-05	6.19E-05	up
ENSG00000011132	APBA3	protein_coding	1.931200711	3.23E-05	6.30E-05	up
ENSG00000198205	ZXDA	protein_coding	1.031554471	3.29E-05	6.41E-05	up
ENSG00000067836	ROGDI	protein_coding	-1.256426292	3.31E-05	6.46E-05	down
ENSG00000146826	C7orf43	protein_coding	-1.100775422	3.31E-05	6.46E-05	down
ENSG00000151150	ANK3	protein_coding	1.194235117	3.62E-05	7.05E-05	up
ENSG00000116815	CD58	protein_coding	-1.234399986	3.70E-05	7.19E-05	down
ENSG00000204348	DXO	protein_coding	-1.841388793	3.74E-05	7.28E-05	down
ENSG00000177576	C18orf32	protein_coding	-3.426351294	3.76E-05	7.31E-05	down
ENSG00000158813	EDA	protein_coding	-2.37190351	3.84E-05	7.46E-05	down
ENSG00000162300	ZFPL1	protein_coding	-1.478818714	3.90E-05	7.56E-05	down
ENSG00000187626	ZKSCAN4	protein_coding	-1.280273034	3.92E-05	7.59E-05	down
ENSG00000107872	FBXL15	protein_coding	1.35023128	3.98E-05	7.71E-05	up
ENSG00000169629	RGPD8	protein_coding	1.35023128	3.98E-05	7.71E-05	up
ENSG00000213337	ANKRD39	protein_coding	1.142123084	4.03E-05	7.80E-05	up
ENSG00000243364	EFNA4	protein_coding	1.142123084	4.03E-05	7.80E-05	up
ENSG00000213020	ZNF611	protein_coding	-1.334428804	4.05E-05	7.84E-05	down
ENSG00000166762	CATSPER2	protein_coding	1.713200059	4.13E-05	7.99E-05	up
ENSG00000103490	PYCARD	protein_coding	1.051002233	4.23E-05	8.18E-05	up
ENSG00000183617	MRPL54	protein_coding	1.097210662	4.36E-05	8.41E-05	up
ENSG00000168772	CXXC4	protein_coding	1.524933421	4.53E-05	8.71E-05	up
ENSG00000204923	FBXO48	protein_coding	1.524933421	4.53E-05	8.71E-05	up
ENSG00000132970	WASF3	protein_coding	-1.193232466	4.55E-05	8.75E-05	down
ENSG00000112812	PRSS16	protein_coding	-1.884457515	4.71E-05	9.05E-05	down
ENSG00000226124	FTCDNL1	protein_coding	1.106143787	4.71E-05	9.05E-05	up
ENSG00000204516	MICB	protein_coding	1.106143787	4.71E-05	9.05E-05	up
ENSG00000175395	ZNF25	protein_coding	-1.082396893	4.73E-05	9.09E-05	down
ENSG00000181016	LSMEM1	protein_coding	-1.671463792	4.85E-05	9.32E-05	down
ENSG00000183091	NEB	protein_coding	1.269642519	4.88E-05	9.36E-05	up
ENSG00000186792	HYAL3	protein_coding	1.269642519	4.88E-05	9.36E-05	up
ENSG00000103740	ACSBG1	protein_coding	-1.124322756	4.93E-05	9.46E-05	down
ENSG00000162390	ACOT11	protein_coding	-2.114407287	5.07E-05	9.71E-05	down
ENSG00000183426	NPIPA1	protein_coding	4.831036549	5.10E-05	9.76E-05	up
ENSG00000177301	KCNA2	protein_coding	4.831036549	5.10E-05	9.76E-05	up
ENSG00000172508	CARNS1	protein_coding	4.831036549	5.10E-05	9.76E-05	up
ENSG00000259305	ZHX1-C8orf76	protein_coding	4.831036549	5.10E-05	9.76E-05	up
ENSG00000136696	IL36B	protein_coding	4.831036549	5.10E-05	9.76E-05	up
ENSG00000066382	MPPED2	protein_coding	4.831036549	5.10E-05	9.76E-05	up
ENSG00000135423	GLS2	protein_coding	4.831036549	5.10E-05	9.76E-05	up

ENSG00000184995	IFNE	protein_coding	4.831036549	5.10E-05	9.76E-05	up
ENSG00000213139	CRYGS	protein_coding	-5.063781214	5.23E-05	9.94E-05	down
ENSG00000140450	ARRDC4	protein_coding	-5.063781214	5.23E-05	9.94E-05	down
ENSG00000140678	ITGAX	protein_coding	-5.063781214	5.23E-05	9.94E-05	down
ENSG00000163633	C4orf36	protein_coding	-5.063781214	5.23E-05	9.94E-05	down
ENSG00000100116	GCAT	protein_coding	-5.063781214	5.23E-05	9.94E-05	down
ENSG00000271503	CCL5	protein_coding	-5.063781214	5.23E-05	9.94E-05	down
ENSG00000065618	COL17A1	protein_coding	-5.063781214	5.23E-05	9.94E-05	down
ENSG00000110076	NRXN2	protein_coding	-5.063781214	5.23E-05	9.94E-05	down
ENSG00000242114	MTFP1	protein_coding	-5.063781214	5.23E-05	9.94E-05	down
ENSG00000276017	AC007325.1	protein_coding	-5.063781214	5.23E-05	9.94E-05	down
ENSG00000167157	PRRX2	protein_coding	-5.063781214	5.23E-05	9.94E-05	down
ENSG00000118514	ALDH8A1	protein_coding	-5.063781214	5.23E-05	9.94E-05	down
ENSG00000162426	SLC45A1	protein_coding	-5.063781214	5.23E-05	9.94E-05	down
ENSG00000162913	OBSCN-AS1	protein_coding	-5.063781214	5.23E-05	9.94E-05	down
ENSG00000196653	ZNF502	protein_coding	-5.063781214	5.23E-05	9.94E-05	down
ENSG00000196860	TOMM20L	protein_coding	-5.063781214	5.23E-05	9.94E-05	down
ENSG00000215440	NPEPL1	protein_coding	-5.063781214	5.23E-05	9.94E-05	down
ENSG00000107611	CUBN	protein_coding	-5.063781214	5.23E-05	9.94E-05	down
ENSG00000010295	IFFO1	protein_coding	-5.063781214	5.23E-05	9.94E-05	down
ENSG00000166823	MESP1	protein_coding	-5.063781214	5.23E-05	9.94E-05	down
ENSG00000068615	REEP1	protein_coding	-5.063781214	5.23E-05	9.94E-05	down
ENSG00000181240	SLC25A41	protein_coding	-5.063781214	5.23E-05	9.94E-05	down
ENSG00000215018	COL28A1	protein_coding	-5.063781214	5.23E-05	9.94E-05	down
ENSG00000179083	FAM133A	protein_coding	-5.063781214	5.23E-05	9.94E-05	down
ENSG00000253598	SLC10A5	protein_coding	-5.063781214	5.23E-05	9.94E-05	down
ENSG00000131620	ANO1	protein_coding	-5.063781214	5.23E-05	9.94E-05	down
ENSG00000180767	CHST13	protein_coding	-5.063781214	5.23E-05	9.94E-05	down
ENSG00000141748	ARL5C	protein_coding	-5.063781214	5.23E-05	9.94E-05	down
ENSG00000175426	PCSK1	protein_coding	-5.063781214	5.23E-05	9.94E-05	down
ENSG00000198324	FAM109A	protein_coding	-5.063781214	5.23E-05	9.94E-05	down
ENSG00000174514	MFSD4A	protein_coding	-5.063781214	5.23E-05	9.94E-05	down
ENSG00000149596	JPH2	protein_coding	-5.063781214	5.23E-05	9.94E-05	down
ENSG00000198610	AKR1C4	protein_coding	-5.063781214	5.23E-05	9.94E-05	down
ENSG00000174899	PQLC2L	protein_coding	-5.063781214	5.23E-05	9.94E-05	down
ENSG00000233757	AC092835.1	protein_coding	-5.063781214	5.23E-05	9.94E-05	down
ENSG00000008323	PLEKHG6	protein_coding	-5.063781214	5.23E-05	9.94E-05	down
ENSG00000267534	S1PR2	protein_coding	-5.063781214	5.23E-05	9.94E-05	down
ENSG00000135929	CYP27A1	protein_coding	-5.063781214	5.23E-05	9.94E-05	down
ENSG00000160961	ZNF333	protein_coding	-5.063781214	5.23E-05	9.94E-05	down
ENSG00000214756	METTL12	protein_coding	1.189373461	5.32E-05	0.000100497	up
ENSG00000171612	SLC25A33	protein_coding	1.813963036	5.51E-05	0.0001039	up
ENSG00000126217	MCF2L	protein_coding	1.328536208	5.64E-05	0.00010619	up
ENSG00000188981	MSANTD1	protein_coding	-4.163316888	5.73E-05	0.000107799	down
ENSG00000164077	MON1A	protein_coding	-1.256426292	5.84E-05	0.000109847	down
ENSG00000181449	SOX2	protein_coding	1.743573708	5.91E-05	0.000111102	up
ENSG00000183196	CHST6	protein_coding	-1.800746809	6.24E-05	0.000116992	down
ENSG00000173083	HPSE	protein_coding	1.40253679	6.27E-05	0.0001175	up
ENSG00000249459	ZNF286B	protein_coding	2.328536208	6.28E-05	0.000117647	up
ENSG00000166133	RPUSD2	protein_coding	1.53712283	6.63E-05	0.000123881	up
ENSG00000172986	GXYLT2	protein_coding	-1.281961384	6.91E-05	0.000129106	down
ENSG00000167377	ZNF23	protein_coding	1.953027073	6.94E-05	0.000129565	up
ENSG00000172548	NIPAL4	protein_coding	1.170994931	7.31E-05	0.000136171	up
ENSG00000214357	NEURL1B	protein_coding	1.170994931	7.31E-05	0.000136171	up

ENSG00000066032	CTNNA2	protein_coding	3.913498709	7.54E-05	0.000140147	up
ENSG00000080493	SLC4A4	protein_coding	1.286100942	7.58E-05	0.000140917	up
ENSG00000008516	MMP25	protein_coding	2.49846121	7.64E-05	0.000141967	up
ENSG00000143494	VASH2	protein_coding	2.158611207	7.94E-05	0.00014738	up
ENSG00000160602	NEK8	protein_coding	-1.134435768	7.96E-05	0.000147578	down
ENSG00000157353	FUK	protein_coding	-1.310874076	8.13E-05	0.000150646	down
ENSG00000131015	ULBP2	protein_coding	-2.919391305	8.17E-05	0.000151425	down
ENSG00000104518	GSDMD	protein_coding	-1.073562235	8.27E-05	0.00015312	down
ENSG00000101115	SALL4	protein_coding	1.198139571	8.36E-05	0.000154726	up
ENSG00000197530	MIB2	protein_coding	1.1292274	8.65E-05	0.000159973	up
ENSG00000165730	STOX1	protein_coding	-1.232178746	8.67E-05	0.000160413	down
ENSG00000169508	GPR183	protein_coding	2.743573708	8.68E-05	0.000160504	up
ENSG00000058091	CDK14	protein_coding	-1.512766046	8.91E-05	0.000164594	down
ENSG00000140009	ESR2	protein_coding	4.743573708	8.94E-05	0.000164871	up
ENSG00000137968	SLC44A5	protein_coding	4.743573708	8.94E-05	0.000164871	up
ENSG00000172350	ABCG4	protein_coding	4.743573708	8.94E-05	0.000164871	up
ENSG00000196549	MME	protein_coding	4.743573708	8.94E-05	0.000164871	up
ENSG00000198520	C1orf228	protein_coding	4.743573708	8.94E-05	0.000164871	up
ENSG00000121380	BCL2L14	protein_coding	4.743573708	8.94E-05	0.000164871	up
ENSG00000180155	LYNX1	protein_coding	1.710406844	8.96E-05	0.00016502	up
ENSG00000102409	BEX4	protein_coding	1.710406844	8.96E-05	0.00016502	up
ENSG00000154330	PGM5	protein_coding	1.40653872	9.25E-05	0.000170286	up
ENSG00000183474	GTF2H2C	protein_coding	-1.418697721	9.42E-05	0.000173143	down
ENSG00000155974	GRIP1	protein_coding	-1.418697721	9.42E-05	0.000173143	down
ENSG00000100218	RSPH14	protein_coding	2.023681627	9.48E-05	0.000174276	up
ENSG00000135424	ITGA7	protein_coding	-1.888694508	9.94E-05	0.000182525	down
ENSG00000137474	MYO7A	protein_coding	1.246074048	0.000100459	0.000184355	up
ENSG00000006071	ABCC8	protein_coding	1.246074048	0.000100459	0.000184355	up
ENSG00000203780	FANK1	protein_coding	-4.95686601	0.000101536	0.000185064	down
ENSG00000105538	RASIP1	protein_coding	-4.95686601	0.000101536	0.000185064	down
ENSG00000164089	ETNPPL	protein_coding	-4.95686601	0.000101536	0.000185064	down
ENSG00000139445	FOXN4	protein_coding	-4.95686601	0.000101536	0.000185064	down
ENSG00000139567	ACVRL1	protein_coding	-4.95686601	0.000101536	0.000185064	down
ENSG00000188649	CC2D2B	protein_coding	-4.95686601	0.000101536	0.000185064	down
ENSG00000140795	MYLK3	protein_coding	-4.95686601	0.000101536	0.000185064	down
ENSG00000089169	RPH3A	protein_coding	-4.95686601	0.000101536	0.000185064	down
ENSG00000173917	HOXB2	protein_coding	-4.95686601	0.000101536	0.000185064	down
ENSG00000075891	PAX2	protein_coding	-4.95686601	0.000101536	0.000185064	down
ENSG00000167207	NOD2	protein_coding	-4.95686601	0.000101536	0.000185064	down
ENSG00000143355	LHX9	protein_coding	-4.95686601	0.000101536	0.000185064	down
ENSG00000143786	CNIH3	protein_coding	-4.95686601	0.000101536	0.000185064	down
ENSG00000167968	DNASE1L2	protein_coding	-4.95686601	0.000101536	0.000185064	down
ENSG00000187144	SPATA21	protein_coding	-4.95686601	0.000101536	0.000185064	down
ENSG00000128918	ALDH1A2	protein_coding	-4.95686601	0.000101536	0.000185064	down
ENSG00000187391	MAGI2	protein_coding	-4.95686601	0.000101536	0.000185064	down
ENSG00000186352	ANKRD37	protein_coding	-4.95686601	0.000101536	0.000185064	down
ENSG00000280071	FP565260.6	protein_coding	-4.95686601	0.000101536	0.000185064	down
ENSG00000172247	C1QTNF4	protein_coding	-4.95686601	0.000101536	0.000185064	down
ENSG00000182379	NXPH4	protein_coding	-4.95686601	0.000101536	0.000185064	down
ENSG00000182584	ACTL10	protein_coding	-4.95686601	0.000101536	0.000185064	down
ENSG00000205861	PCOTH	protein_coding	-4.95686601	0.000101536	0.000185064	down
ENSG00000181577	C6orf223	protein_coding	-4.95686601	0.000101536	0.000185064	down
ENSG00000142089	IFITM3	protein_coding	-4.95686601	0.000101536	0.000185064	down
ENSG00000152049	KCNE4	protein_coding	-4.95686601	0.000101536	0.000185064	down

ENSG00000176472	ZNF575	protein_coding	-4.95686601	0.000101536	0.000185064	down
ENSG00000284461	RABGEF1	protein_coding	-4.95686601	0.000101536	0.000185064	down
ENSG00000176732	PFN4	protein_coding	-4.95686601	0.000101536	0.000185064	down
ENSG00000175536	LIPT2	protein_coding	-4.95686601	0.000101536	0.000185064	down
ENSG00000205089	CCNI2	protein_coding	-4.95686601	0.000101536	0.000185064	down
ENSG00000132000	PODNL1	protein_coding	-4.95686601	0.000101536	0.000185064	down
ENSG00000179292	TMEM151A	protein_coding	-4.95686601	0.000101536	0.000185064	down
ENSG00000228727	SAPCD1	protein_coding	-4.95686601	0.000101536	0.000185064	down
ENSG00000131398	KCNC3	protein_coding	-4.95686601	0.000101536	0.000185064	down
ENSG00000273398	AC017083.3	protein_coding	-4.95686601	0.000101536	0.000185064	down
ENSG00000165731	RET	protein_coding	-4.95686601	0.000101536	0.000185064	down
ENSG00000151023	ENKUR	protein_coding	-4.95686601	0.000101536	0.000185064	down
ENSG00000149968	MMP3	protein_coding	-4.95686601	0.000101536	0.000185064	down
ENSG00000184619	KRBA2	protein_coding	-4.95686601	0.000101536	0.000185064	down
ENSG00000165474	GJB2	protein_coding	-1.658524736	0.000102026	0.000184771	down
ENSG00000137571	SLCO5A1	protein_coding	-2.149511088	0.00010564	0.000191169	down
ENSG00000161267	BDH1	protein_coding	1.165037476	0.000107445	0.000194327	up
ENSG00000172572	PDE3A	protein_coding	1.101125712	0.000108697	0.000196549	up
ENSG00000197249	SERPINA1	protein_coding	-4.063781214	0.000113214	0.000204392	down
ENSG00000105088	OLFM2	protein_coding	-1.231335311	0.000115361	0.00020812	down
ENSG00000167705	RILP	protein_coding	-1.005618189	0.000115695	0.000208677	down
ENSG00000212124	TAS2R19	protein_coding	1.82157622	0.000119578	0.000215467	up
ENSG00000100246	DNAL4	protein_coding	1.328536208	0.000123012	0.000221558	up
ENSG00000104324	CPQ	protein_coding	-1.944482286	0.000123923	0.000223078	down
ENSG00000171806	METTL18	protein_coding	-1.09596162	0.000124347	0.000223792	down
ENSG00000119922	IFIT2	protein_coding	-1.34757418	0.000125854	0.00022638	down
ENSG00000118849	RARRES1	protein_coding	1.208241975	0.000131299	0.000235711	up
ENSG00000186976	EFCAB6	protein_coding	3.831036549	0.000133299	0.000239106	up
ENSG00000188747	NOXA1	protein_coding	1.410998369	0.000136619	0.000244901	up
ENSG00000162542	TMCO4	protein_coding	-2.393929816	0.000140445	0.000251459	down
ENSG00000172456	FGGY	protein_coding	-2.393929816	0.000140445	0.000251459	down
ENSG00000204946	ZNF783	protein_coding	1.033080325	0.000141084	0.000252535	up
ENSG00000185519	FAM131C	protein_coding	-1.387670826	0.000145133	0.000259387	down
ENSG00000204514	ZNF814	protein_coding	1.980612905	0.000149431	0.000266564	up
ENSG00000189143	CLDN4	protein_coding	-1.230431084	0.000153497	0.000273417	down
ENSG00000243660	ZNF487	protein_coding	1.040555445	0.00015366	0.000273677	up
ENSG00000242616	GNG10	protein_coding	4.650464303	0.000157176	0.0002795	up
ENSG00000206559	ZCWPW2	protein_coding	4.650464303	0.000157176	0.0002795	up
ENSG00000171790	SLFNL1	protein_coding	4.650464303	0.000157176	0.0002795	up
ENSG00000082497	SERTAD4	protein_coding	1.093158146	0.000159	0.000282302	up
ENSG00000167037	SGSM1	protein_coding	-1.618996372	0.000165076	0.000292855	down
ENSG00000204390	HSPA1L	protein_coding	-1.618996372	0.000165076	0.000292855	down
ENSG00000157927	RADIL	protein_coding	-1.205800219	0.000170492	0.000302072	down
ENSG00000254827	SLC22A18AS	protein_coding	-1.205800219	0.000170492	0.000302072	down
ENSG00000101463	SYNDIG1	protein_coding	1.783102072	0.000183498	0.000324401	up
ENSG00000166250	CLMP	protein_coding	-1.006448039	0.000188744	0.000333139	down
ENSG00000012504	NR1H4	protein_coding	-4.841388793	0.000198334	0.000347404	down
ENSG00000144476	ACKR3	protein_coding	-4.841388793	0.000198334	0.000347404	down
ENSG00000119888	EPCAM	protein_coding	-4.841388793	0.000198334	0.000347404	down
ENSG00000105261	OVOL3	protein_coding	-4.841388793	0.000198334	0.000347404	down
ENSG00000213420	GPC2	protein_coding	-4.841388793	0.000198334	0.000347404	down
ENSG00000140090	SLC24A4	protein_coding	-4.841388793	0.000198334	0.000347404	down
ENSG00000164076	CAMKV	protein_coding	-4.841388793	0.000198334	0.000347404	down
ENSG00000115252	PDE1A	protein_coding	-4.841388793	0.000198334	0.000347404	down

ENSG00000091010	POU4F3	protein_coding	-4.841388793	0.000198334	0.000347404	down
ENSG00000164736	SOX17	protein_coding	-4.841388793	0.000198334	0.000347404	down
ENSG00000114541	FRMD4B	protein_coding	-4.841388793	0.000198334	0.000347404	down
ENSG00000007516	BAIAP3	protein_coding	-4.841388793	0.000198334	0.000347404	down
ENSG00000197776	KLHDC1	protein_coding	-4.841388793	0.000198334	0.000347404	down
ENSG00000158445	KCNB1	protein_coding	-4.841388793	0.000198334	0.000347404	down
ENSG00000134470	IL15RA	protein_coding	-4.841388793	0.000198334	0.000347404	down
ENSG00000217555	CKLF	protein_coding	-4.841388793	0.000198334	0.000347404	down
ENSG00000167183	PRR15L	protein_coding	-4.841388793	0.000198334	0.000347404	down
ENSG00000133937	GSC	protein_coding	-4.841388793	0.000198334	0.000347404	down
ENSG00000177688	SUMO4	protein_coding	-4.841388793	0.000198334	0.000347404	down
ENSG00000172031	EPHX4	protein_coding	-4.841388793	0.000198334	0.000347404	down
ENSG00000162745	OLFML2B	protein_coding	-4.841388793	0.000198334	0.000347404	down
ENSG00000172738	TMEM217	protein_coding	-4.841388793	0.000198334	0.000347404	down
ENSG00000254996	ANKHD1-EIF4	protein_coding	-4.841388793	0.000198334	0.000347404	down
ENSG00000117266	CDK18	protein_coding	-4.841388793	0.000198334	0.000347404	down
ENSG00000132965	ALOX5AP	protein_coding	-4.841388793	0.000198334	0.000347404	down
ENSG00000083454	P2RX5	protein_coding	-4.841388793	0.000198334	0.000347404	down
ENSG00000176244	ACBD7	protein_coding	-4.841388793	0.000198334	0.000347404	down
ENSG00000259207	ITGB3	protein_coding	-4.841388793	0.000198334	0.000347404	down
ENSG00000103460	TOX3	protein_coding	-4.841388793	0.000198334	0.000347404	down
ENSG00000204956	PCDHGA1	protein_coding	-4.841388793	0.000198334	0.000347404	down
ENSG00000072818	ACAP1	protein_coding	-4.841388793	0.000198334	0.000347404	down
ENSG00000165269	AQP7	protein_coding	-4.841388793	0.000198334	0.000347404	down
ENSG00000102104	RS1	protein_coding	-4.841388793	0.000198334	0.000347404	down
ENSG00000198133	TMEM229B	protein_coding	-4.841388793	0.000198334	0.000347404	down
ENSG00000160188	RSPH1	protein_coding	-4.841388793	0.000198334	0.000347404	down
ENSG00000174808	BTC	protein_coding	-4.841388793	0.000198334	0.000347404	down
ENSG00000174844	DNAH12	protein_coding	-4.841388793	0.000198334	0.000347404	down
ENSG00000184378	ACTRT3	protein_coding	-4.841388793	0.000198334	0.000347404	down
ENSG00000121101	TEX14	protein_coding	-4.841388793	0.000198334	0.000347404	down
ENSG00000170231	FABP6	protein_coding	-4.841388793	0.000198334	0.000347404	down
ENSG00000121207	LRAT	protein_coding	-4.841388793	0.000198334	0.000347404	down
ENSG00000145287	PLAC8	protein_coding	-4.841388793	0.000198334	0.000347404	down
ENSG00000159915	ZNF233	protein_coding	-4.841388793	0.000198334	0.000347404	down
ENSG00000267855	NDUFA7	protein_coding	-4.841388793	0.000198334	0.000347404	down
ENSG00000181982	CCDC149	protein_coding	-1.043022654	0.000205066	0.000356598	down
ENSG00000026652	AGPAT4	protein_coding	1.490807637	0.000208862	0.000362816	up
ENSG00000152377	SPOCK1	protein_coding	-1.643449415	0.000215039	0.000373015	down
ENSG00000179455	MKRN3	protein_coding	-1.286173636	0.000216562	0.000375558	down
ENSG00000131187	F12	protein_coding	-2.195025748	0.000220166	0.000381568	down
ENSG00000243477	NAT6	protein_coding	-2.195025748	0.000220166	0.000381568	down
ENSG00000130203	APOE	protein_coding	-3.95686601	0.000224421	0.000388043	down
ENSG00000072858	SIDT1	protein_coding	-3.95686601	0.000224421	0.000388043	down
ENSG00000159958	TNFRSF13C	protein_coding	-3.95686601	0.000224421	0.000388043	down
ENSG00000123610	TNFAIP6	protein_coding	3.743573708	0.000236074	0.000406974	up
ENSG00000160396	HIPK4	protein_coding	3.743573708	0.000236074	0.000406974	up
ENSG00000179862	CITED4	protein_coding	-1.504353806	0.000243939	0.000419678	down
ENSG00000144821	MYH15	protein_coding	-1.052892898	0.000253022	0.000434713	down
ENSG00000166676	TVP23A	protein_coding	1.303001116	0.000257517	0.000442113	up
ENSG00000144355	DLX1	protein_coding	-1.95686601	0.000262659	0.00045066	down
ENSG00000212916	MAP10	protein_coding	2.991501221	0.000264746	0.000454169	up
ENSG00000179528	LBX2	protein_coding	-1.578354387	0.000265449	0.000455162	down
ENSG00000111879	FAM184A	protein_coding	-1.578354387	0.000265449	0.000455162	down

ENSG00000177932	ZNF354C	protein_coding	1.18097902	0.000267448	0.000458352	up
ENSG00000205572	SERF1B	protein_coding	1.328536208	0.000269494	0.000461521	up
ENSG00000167363	FN3K	protein_coding	-1.037113264	0.000271066	0.000464068	down
ENSG00000187860	CCDC157	protein_coding	-3.163316888	0.000277209	0.000474143	down
ENSG00000269313	MAGIX	protein_coding	-3.163316888	0.000277209	0.000474143	down
ENSG00000183979	NPB	protein_coding	4.55092863	0.000277647	0.000474152	up
ENSG00000148123	PLPPR1	protein_coding	4.55092863	0.000277647	0.000474152	up
ENSG00000173662	TAS1R1	protein_coding	4.55092863	0.000277647	0.000474152	up
ENSG00000241945	PWP2	protein_coding	4.55092863	0.000277647	0.000474152	up
ENSG00000261652	C15orf65	protein_coding	4.55092863	0.000277647	0.000474152	up
ENSG00000006606	CCL26	protein_coding	4.55092863	0.000277647	0.000474152	up
ENSG00000145687	SSBP2	protein_coding	-1.792479193	0.00028055	0.000478268	down
ENSG00000205323	SARNP	protein_coding	1.197291675	0.000285926	0.000486929	up
ENSG00000128536	CDHR3	protein_coding	-1.081339586	0.000289551	0.000492847	down
ENSG00000154914	USP43	protein_coding	1.387429897	0.00029043	0.000494292	up
ENSG00000254505	CHMP4A	protein_coding	-2.758926633	0.000293739	0.00049964	down
ENSG00000185269	NOTUM	protein_coding	1.117969222	0.00029456	0.000500959	up
ENSG00000084110	HAL	protein_coding	1.606070184	0.000303384	0.000515594	up
ENSG00000256060	TRAPPC2B	protein_coding	1.214879427	0.000304907	0.00051786	up
ENSG00000130349	C6orf203	protein_coding	-1.256426292	0.000324713	0.000550649	down
ENSG00000119138	KLF9	protein_coding	-1.256426292	0.000324713	0.000550649	down
ENSG00000106025	TSPAN12	protein_coding	-1.256426292	0.000324713	0.000550649	down
ENSG00000115507	OTX1	protein_coding	1.254535627	0.000343662	0.000581644	up
ENSG00000149743	TRPT1	protein_coding	-1.841388793	0.000356466	0.000602695	down
ENSG00000159079	C21orf59	protein_coding	-1.075854047	0.000384032	0.000647973	down
ENSG00000129007	CALML4	protein_coding	-4.715857911	0.000390163	0.000654563	down
ENSG00000071242	RPS6KA2	protein_coding	-4.715857911	0.000390163	0.000654563	down
ENSG00000154252	GAL3ST2	protein_coding	-4.715857911	0.000390163	0.000654563	down
ENSG00000105679	GAPDHS	protein_coding	-4.715857911	0.000390163	0.000654563	down
ENSG00000130700	GATA5	protein_coding	-4.715857911	0.000390163	0.000654563	down
ENSG00000188051	TMEM221	protein_coding	-4.715857911	0.000390163	0.000654563	down
ENSG00000164185	ZNF474	protein_coding	-4.715857911	0.000390163	0.000654563	down
ENSG00000115318	LOXL3	protein_coding	-4.715857911	0.000390163	0.000654563	down
ENSG00000140465	CYP1A1	protein_coding	-4.715857911	0.000390163	0.000654563	down
ENSG00000081842	PCDHA6	protein_coding	-4.715857911	0.000390163	0.000654563	down
ENSG00000138944	KIAA1644	protein_coding	-4.715857911	0.000390163	0.000654563	down
ENSG00000163995	ABLIM2	protein_coding	-4.715857911	0.000390163	0.000654563	down
ENSG00000197415	VEPH1	protein_coding	-4.715857911	0.000390163	0.000654563	down
ENSG00000158113	LRRC43	protein_coding	-4.715857911	0.000390163	0.000654563	down
ENSG00000256806	C17orf100	protein_coding	-4.715857911	0.000390163	0.000654563	down
ENSG00000109705	NKX3-2	protein_coding	-4.715857911	0.000390163	0.000654563	down
ENSG00000099953	MMP11	protein_coding	-4.715857911	0.000390163	0.000654563	down
ENSG00000133878	DUSP26	protein_coding	-4.715857911	0.000390163	0.000654563	down
ENSG00000128203	ASPHD2	protein_coding	-4.715857911	0.000390163	0.000654563	down
ENSG00000177359	AC024940.1	protein_coding	-4.715857911	0.000390163	0.000654563	down
ENSG00000176884	GRIN1	protein_coding	-4.715857911	0.000390163	0.000654563	down
ENSG00000221923	ZNF880	protein_coding	-4.715857911	0.000390163	0.000654563	down
ENSG00000171403	KRT9	protein_coding	-4.715857911	0.000390163	0.000654563	down
ENSG00000180113	TDRD6	protein_coding	-4.715857911	0.000390163	0.000654563	down
ENSG00000131379	C3orf20	protein_coding	-4.715857911	0.000390163	0.000654563	down
ENSG00000179520	SLC17A8	protein_coding	-4.715857911	0.000390163	0.000654563	down
ENSG00000003096	KLHL13	protein_coding	-4.715857911	0.000390163	0.000654563	down
ENSG00000116990	MYCL	protein_coding	-4.715857911	0.000390163	0.000654563	down
ENSG00000174343	CHRNA9	protein_coding	-4.715857911	0.000390163	0.000654563	down

ENSG00000198440	ZNF583	protein_coding	-4.715857911	0.000390163	0.000654563	down
ENSG00000160097	FNDC5	protein_coding	-4.715857911	0.000390163	0.000654563	down
ENSG00000198835	GJC2	protein_coding	-4.715857911	0.000390163	0.000654563	down
ENSG00000008226	DLEC1	protein_coding	-4.715857911	0.000390163	0.000654563	down
ENSG00000135747	ZNF670-ZNF6	protein_coding	-4.715857911	0.000390163	0.000654563	down
ENSG00000145192	AHSG	protein_coding	-4.715857911	0.000390163	0.000654563	down
ENSG00000111863	ADTRP	protein_coding	-4.715857911	0.000390163	0.000654563	down
ENSG00000141448	GATA6	protein_coding	1.328536208	0.000399603	0.00066643	up
ENSG00000116039	ATP6V1B1	protein_coding	1.358283552	0.000416042	0.000692339	up
ENSG00000107159	CA9	protein_coding	2.55092863	0.000416508	0.000692905	up
ENSG00000160226	C21orf2	protein_coding	2.55092863	0.000416508	0.000692905	up
ENSG00000269743	SLC25A53	protein_coding	-1.109584904	0.000435877	0.000723664	down
ENSG00000172687	ZNF738	protein_coding	1.631098978	0.00044281	0.000734841	up
ENSG00000006747	SCIN	protein_coding	-3.841388793	0.000446157	0.000739913	down
ENSG00000260691	ANKRD20A1	protein_coding	-3.841388793	0.000446157	0.000739913	down
ENSG00000126460	PRRG2	protein_coding	-3.841388793	0.000446157	0.000739913	down
ENSG00000233927	RPS28	protein_coding	-3.841388793	0.000446157	0.000739913	down
ENSG00000146700	SSC4D	protein_coding	1.469398744	0.000450115	0.000745874	up
ENSG00000173890	GPR160	protein_coding	-1.32681562	0.00045165	0.000748155	down
ENSG00000183023	SLC8A1	protein_coding	1.516163212	0.000453618	0.000751227	up
ENSG00000244219	TMEM225B	protein_coding	2.913498709	0.000457031	0.000756726	up
ENSG00000182580	EPHB3	protein_coding	1.122085331	0.000467529	0.000773098	up
ENSG00000179583	CIITA	protein_coding	1.229000535	0.000480142	0.000793556	up
ENSG00000178947	SMIM10L2A	protein_coding	4.444013426	0.000492612	0.000812496	up
ENSG00000188738	FSIP2	protein_coding	4.444013426	0.000492612	0.000812496	up
ENSG00000173585	CCR9	protein_coding	4.444013426	0.000492612	0.000812496	up
ENSG00000143603	KCNN3	protein_coding	4.444013426	0.000492612	0.000812496	up
ENSG00000137841	PLCB2	protein_coding	4.444013426	0.000492612	0.000812496	up
ENSG00000044524	EPHA3	protein_coding	4.444013426	0.000492612	0.000812496	up
ENSG00000258555	SPECC1L-ADC	protein_coding	4.444013426	0.000492612	0.000812496	up
ENSG00000062038	CDH3	protein_coding	4.444013426	0.000492612	0.000812496	up
ENSG00000175182	FAM131A	protein_coding	-1.290373624	0.000513142	0.000843611	down
ENSG00000186204	CYP4F12	protein_coding	-3.063781214	0.000537972	0.000883242	down
ENSG00000149571	KIRREL3	protein_coding	1.274088424	0.000538421	0.000883583	up
ENSG00000165879	FRAT1	protein_coding	1.076149047	0.000541259	0.000887798	up
ENSG00000178301	AQP11	protein_coding	-1.972633326	0.000558925	0.00091518	down
ENSG00000247746	USP51	protein_coding	1.299967056	0.000566628	0.000927285	up
ENSG00000047597	XK	protein_coding	1.843109381	0.000569655	0.00093187	up
ENSG00000091262	ABCC6	protein_coding	-1.654975669	0.000592904	0.000967978	down
ENSG00000171791	BCL2	protein_coding	-1.654975669	0.000592904	0.000967978	down
ENSG00000176945	MUC20	protein_coding	1.328536208	0.000593343	0.000968455	up
ENSG00000113211	PCDHB6	protein_coding	1.360245068	0.000617727	0.001006361	up
ENSG00000185046	ANKS1B	protein_coding	1.360245068	0.000617727	0.001006361	up
ENSG00000159882	ZNF230	protein_coding	-1.017238628	0.000626707	0.001020687	down
ENSG00000184492	FOXD4L1	protein_coding	1.532069603	0.00066884	0.001086622	up
ENSG00000196466	ZNF799	protein_coding	-1.195025748	0.000717289	0.001162354	down
ENSG00000134864	GGACT	protein_coding	-1.578354387	0.000734545	0.001188738	down
ENSG00000187266	EPOR	protein_coding	-1.841388793	0.000762156	0.001231728	down
ENSG00000152503	TRIM36	protein_coding	-1.256426292	0.000772199	0.00124759	down
ENSG00000168685	IL7R	protein_coding	-4.578354387	0.000773344	0.001242009	down
ENSG00000139269	INHBE	protein_coding	-4.578354387	0.000773344	0.001242009	down
ENSG00000213999	MEF2B	protein_coding	-4.578354387	0.000773344	0.001242009	down
ENSG00000115604	IL18R1	protein_coding	-4.578354387	0.000773344	0.001242009	down
ENSG00000089692	LAG3	protein_coding	-4.578354387	0.000773344	0.001242009	down

ENSG00000148584	A1CF	protein_coding	-4.578354387	0.000773344	0.001242009	down
ENSG00000158220	ESYT3	protein_coding	-4.578354387	0.000773344	0.001242009	down
ENSG00000261408	TEN1-CDK3	protein_coding	-4.578354387	0.000773344	0.001242009	down
ENSG00000114115	RBP1	protein_coding	-4.578354387	0.000773344	0.001242009	down
ENSG00000104951	IL4I1	protein_coding	-4.578354387	0.000773344	0.001242009	down
ENSG00000104901	DKKL1	protein_coding	-4.578354387	0.000773344	0.001242009	down
ENSG00000196388	INCA1	protein_coding	-4.578354387	0.000773344	0.001242009	down
ENSG00000172543	CTSW	protein_coding	-4.578354387	0.000773344	0.001242009	down
ENSG00000280537	AC068946.1	protein_coding	-4.578354387	0.000773344	0.001242009	down
ENSG00000172803	SNX32	protein_coding	-4.578354387	0.000773344	0.001242009	down
ENSG00000103023	PRSS54	protein_coding	-4.578354387	0.000773344	0.001242009	down
ENSG00000250510	GPR162	protein_coding	-4.578354387	0.000773344	0.001242009	down
ENSG00000166793	YPEL4	protein_coding	-4.578354387	0.000773344	0.001242009	down
ENSG00000185291	IL3RA	protein_coding	-4.578354387	0.000773344	0.001242009	down
ENSG00000053702	NRIP2	protein_coding	-4.578354387	0.000773344	0.001242009	down
ENSG00000161653	NAGS	protein_coding	-4.578354387	0.000773344	0.001242009	down
ENSG00000205464	ATP6AP1L	protein_coding	-4.578354387	0.000773344	0.001242009	down
ENSG00000106327	TFR2	protein_coding	-4.578354387	0.000773344	0.001242009	down
ENSG00000121966	CXCR4	protein_coding	-4.578354387	0.000773344	0.001242009	down
ENSG00000253873	PCDHGA11	protein_coding	-4.578354387	0.000773344	0.001242009	down
ENSG00000180878	C11orf42	protein_coding	-4.578354387	0.000773344	0.001242009	down
ENSG00000180999	C1orf105	protein_coding	-4.578354387	0.000773344	0.001242009	down
ENSG00000141854	MISP3	protein_coding	-4.578354387	0.000773344	0.001242009	down
ENSG00000042781	USH2A	protein_coding	-4.578354387	0.000773344	0.001242009	down
ENSG00000150628	SPATA4	protein_coding	-4.578354387	0.000773344	0.001242009	down
ENSG00000159399	HK2	protein_coding	-4.578354387	0.000773344	0.001242009	down
ENSG00000076826	CAMSAP3	protein_coding	-4.578354387	0.000773344	0.001242009	down
ENSG00000204086	RPA4	protein_coding	-4.578354387	0.000773344	0.001242009	down
ENSG00000213782	DDX47	protein_coding	2.831036549	0.000787252	0.00125675	up
ENSG00000164674	SYTL3	protein_coding	1.270820711	0.000799865	0.001275896	up
ENSG00000198754	OXCT2	protein_coding	-2.182425711	0.000819602	0.001306051	down
ENSG00000166578	IQCD	protein_coding	-1.077456151	0.000833845	0.001327912	down
ENSG00000149021	SCGB1A1	protein_coding	4.328536208	0.000878033	0.001393703	up
ENSG00000158428	CATIP	protein_coding	4.328536208	0.000878033	0.001393703	up
ENSG00000153347	FAM81B	protein_coding	4.328536208	0.000878033	0.001393703	up
ENSG00000186399	GOLGA8R	protein_coding	4.328536208	0.000878033	0.001393703	up
ENSG00000121871	SLITRK3	protein_coding	4.328536208	0.000878033	0.001393703	up
ENSG00000180332	KCTD4	protein_coding	4.328536208	0.000878033	0.001393703	up
ENSG00000135960	EDAR	protein_coding	4.328536208	0.000878033	0.001393703	up
ENSG00000136235	GPNMB	protein_coding	-3.715857911	0.000889479	0.00140895	down
ENSG00000185862	EVI2B	protein_coding	-3.715857911	0.000889479	0.00140895	down
ENSG00000101222	SPEF1	protein_coding	-3.715857911	0.000889479	0.00140895	down
ENSG00000198914	POU3F3	protein_coding	2.203005326	0.000920952	0.001457053	up
ENSG00000160201	U2AF1	protein_coding	-1.908502989	0.000960942	0.001517626	down
ENSG00000162994	CLHC1	protein_coding	-1.037786006	0.000963195	0.001520892	down
ENSG00000179965	ZNF771	protein_coding	-1.037786006	0.000963195	0.001520892	down
ENSG00000104361	NIPAL2	protein_coding	1.618042826	0.000971244	0.001533087	up
ENSG00000151470	C4orf33	protein_coding	1.55092863	0.000986327	0.00155615	up
ENSG00000188295	ZNF669	protein_coding	-1.730357481	0.000997761	0.001573135	down
ENSG00000254402	LRRC24	protein_coding	1.102654801	0.001013902	0.001598049	up
ENSG00000214050	FBXO16	protein_coding	1.102654801	0.001013902	0.001598049	up
ENSG00000128849	CGNL1	protein_coding	-2.578354387	0.00103977	0.001636785	down
ENSG00000121270	ABCC11	protein_coding	-2.95686601	0.001041742	0.001639497	down
ENSG00000205436	EXOC3L4	protein_coding	-1.163316888	0.001056331	0.001660712	down

ENSG00000015568	RGPD5	protein_coding	2.40653872	0.001164866	0.00182577	up
ENSG00000126583	PRKCG	protein_coding	1.132615998	0.001184369	0.001855456	up
ENSG00000133874	RNF122	protein_coding	1.002307976	0.00119742	0.001874118	up
ENSG00000109072	VTN	protein_coding	-1.393929816	0.001242514	0.001940732	down
ENSG00000086717	PPEF1	protein_coding	-1.779988248	0.001284133	0.002003175	down
ENSG00000126259	KIRREL2	protein_coding	1.743573708	0.001356055	0.00210848	up
ENSG00000167034	NKX3-1	protein_coding	-1.086501291	0.001367294	0.002124852	down
ENSG00000154479	CCDC173	protein_coding	1.365062084	0.001368293	0.002126204	up
ENSG00000248144	ADH1C	protein_coding	-1.256426292	0.001381714	0.002146047	down
ENSG00000239779	WBP1	protein_coding	-1.46287717	0.001410097	0.002188067	down
ENSG00000164675	IQUB	protein_coding	1.454067091	0.001445118	0.002240194	up
ENSG00000170345	FOS	protein_coding	1.573648706	0.001454388	0.002254247	up
ENSG00000163521	GLB1L	protein_coding	1.210699718	0.001571652	0.002431084	up
ENSG00000108821	COL1A1	protein_coding	1.210699718	0.001571652	0.002431084	up
ENSG00000155849	ELMO1	protein_coding	1.210699718	0.001571652	0.002431084	up
ENSG00000128165	ADM2	protein_coding	4.203005326	0.001572559	0.002429066	up
ENSG00000177614	PGBD5	protein_coding	4.203005326	0.001572559	0.002429066	up
ENSG00000163141	BNIPL	protein_coding	4.203005326	0.001572559	0.002429066	up
ENSG00000171989	LDHAL6B	protein_coding	4.203005326	0.001572559	0.002429066	up
ENSG00000142765	SYTL1	protein_coding	4.203005326	0.001572559	0.002429066	up
ENSG00000162040	HS3ST6	protein_coding	4.203005326	0.001572559	0.002429066	up
ENSG00000174945	AMZ1	protein_coding	4.203005326	0.001572559	0.002429066	up
ENSG00000154310	TNIK	protein_coding	-1.042301487	0.001584957	0.00244409	down
ENSG00000141314	RHBDL3	protein_coding	-1.042301487	0.001584957	0.00244409	down
ENSG00000109220	CHIC2	protein_coding	1.106143787	0.001619701	0.002497083	up
ENSG00000186523	FAM86B1	protein_coding	-2.256426292	0.00172296	0.002652553	down
ENSG00000185869	ZNF829	protein_coding	-2.256426292	0.00172296	0.002652553	down
ENSG00000271447	MMP28	protein_coding	1.945207569	0.001758245	0.002705991	up
ENSG00000154188	ANGPT1	protein_coding	-3.578354387	0.00177804	0.002733901	down
ENSG00000272196	HIST2H2AA4	protein_coding	-3.578354387	0.00177804	0.002733901	down
ENSG00000163864	NMNAT3	protein_coding	-3.578354387	0.00177804	0.002733901	down
ENSG00000136197	C7orf25	protein_coding	-3.578354387	0.00177804	0.002733901	down
ENSG00000204219	TCEA3	protein_coding	-3.578354387	0.00177804	0.002733901	down
ENSG00000164687	FABP5	protein_coding	-1.0026697	0.001807969	0.002776939	down
ENSG00000160179	ABCG1	protein_coding	1.29377079	0.001871223	0.002870614	up
ENSG00000172318	B3GALT1	protein_coding	-1.154546678	0.001888164	0.002895255	down
ENSG00000181274	FRAT2	protein_coding	-1.056127642	0.001963934	0.003004308	down
ENSG00000104368	PLAT	protein_coding	-2.841388793	0.002011561	0.003073454	down
ENSG00000164414	SLC35A1	protein_coding	1.158611207	0.002036568	0.003109211	up
ENSG00000183150	GPR19	protein_coding	1.368064573	0.002042119	0.003117252	up
ENSG00000211448	DIO2	protein_coding	-1.578354387	0.002054968	0.003135413	down
ENSG00000112309	B3GAT2	protein_coding	-1.919391305	0.002069705	0.003155999	down
ENSG00000136371	MTHFS	protein_coding	-1.919391305	0.002069705	0.003155999	down
ENSG00000139679	LPAR6	protein_coding	1.691106288	0.002074802	0.003162454	up
ENSG00000142700	DMRTA2	protein_coding	1.413425106	0.002107018	0.003210221	up
ENSG00000188763	FZD9	protein_coding	1.601554703	0.002143499	0.003263388	up
ENSG00000166924	NYAP1	protein_coding	-1.715857911	0.002146256	0.003266981	down
ENSG00000171443	ZNF524	protein_coding	-1.300820412	0.002196668	0.003340323	down
ENSG00000180096	1-Sep	protein_coding	1.016592202	0.002292462	0.00348149	up
ENSG00000189420	ZFP92	protein_coding	2.650464303	0.002316951	0.003515602	up
ENSG00000280789	PAGR1	protein_coding	3.328536208	0.002347041	0.003557818	up
ENSG00000154548	SRSF12	protein_coding	1.094070955	0.002393988	0.003624148	up
ENSG00000182749	PAQR7	protein_coding	1.110356038	0.002594601	0.003912924	up
ENSG00000204965	PCDHA5	protein_coding	1.110356038	0.002594601	0.003912924	up

ENSG00000162981	FAM84A	protein_coding	1.258146881	0.002649698	0.0039909	up
ENSG00000175279	CENPS	protein_coding	-1.610063247	0.002707064	0.004073578	down
ENSG00000131773	KHDRBS3	protein_coding	1.039029591	0.002738076	0.004117988	up
ENSG00000153993	SEMA3D	protein_coding	1.881077231	0.002774048	0.004169424	up
ENSG00000126016	AMOT	protein_coding	1.128237558	0.002806198	0.004212941	up
ENSG00000163823	CCR1	protein_coding	4.065501803	0.002830739	0.004244369	up
ENSG00000011590	ZBTB32	protein_coding	4.065501803	0.002830739	0.004244369	up
ENSG00000133328	HRASLS2	protein_coding	4.065501803	0.002830739	0.004244369	up
ENSG00000166343	MSS51	protein_coding	4.065501803	0.002830739	0.004244369	up
ENSG00000277870	FAM230A	protein_coding	4.065501803	0.002830739	0.004244369	up
ENSG00000125285	SOX21	protein_coding	4.065501803	0.002830739	0.004244369	up
ENSG00000111254	AKAP3	protein_coding	4.065501803	0.002830739	0.004244369	up
ENSG00000115556	PLCD4	protein_coding	-1.003660222	0.002990491	0.004471688	down
ENSG00000262621	AC025283.2	protein_coding	1.147963963	0.003027745	0.004524725	up
ENSG00000091428	RAPGEF4	protein_coding	-2.163316888	0.003108289	0.004637729	down
ENSG00000129355	CDKN2D	protein_coding	1.421645613	0.003148224	0.004694974	up
ENSG00000183111	ARHGEF37	protein_coding	2.246074048	0.003201452	0.004768096	up
ENSG00000120658	ENOX1	protein_coding	1.480539302	0.003205357	0.004773048	up
ENSG00000144362	PHOSPHO2	protein_coding	1.55092863	0.003212899	0.004783198	up
ENSG00000249158	PCDHA11	protein_coding	-1.519460698	0.003313642	0.00492739	down
ENSG00000228439	TSTD3	protein_coding	-1.256426292	0.003334479	0.004957032	down
ENSG00000130997	POLN	protein_coding	-1.143951563	0.003388488	0.005032099	down
ENSG00000175600	SUGCT	protein_coding	-1.143951563	0.003388488	0.005032099	down
ENSG00000212123	PRR22	protein_coding	-1.648743715	0.003556194	0.005264777	down
ENSG00000188626	GOLGA8M	protein_coding	-3.426351294	0.003562943	0.005270506	down
ENSG00000182325	FBXL6	protein_coding	-3.426351294	0.003562943	0.005270506	down
ENSG00000136167	LCP1	protein_coding	-3.426351294	0.003562943	0.005270506	down
ENSG00000076344	RGS11	protein_coding	-2.37190351	0.003603672	0.005324778	down
ENSG00000139865	TTC6	protein_coding	1.221621005	0.003731308	0.005505225	up
ENSG00000240053	LY6G5B	protein_coding	2.55092863	0.00395486	0.005819681	up
ENSG00000120051	CFAP58	protein_coding	1.115542485	0.004169716	0.006121629	up
ENSG00000117477	CCDC181	protein_coding	1.115542485	0.004169716	0.006121629	up
ENSG00000158423	RIBC1	protein_coding	3.203005326	0.004174601	0.006127162	up
ENSG00000162746	FCRLB	protein_coding	3.203005326	0.004174601	0.006127162	up
ENSG00000176040	TMPRSS7	protein_coding	3.203005326	0.004174601	0.006127162	up
ENSG00000151623	NR3C2	protein_coding	1.813963036	0.004349601	0.006371807	up
ENSG00000167105	TMEM92	protein_coding	1.328536208	0.004401548	0.006443895	up
ENSG00000147689	FAM83A	protein_coding	-1.934498197	0.004493853	0.006573479	down
ENSG00000188613	NANOS1	protein_coding	-1.137781796	0.004547213	0.006647107	down
ENSG00000164093	PITX2	protein_coding	-1.696998884	0.004652418	0.006791855	down
ENSG00000110693	SOX6	protein_coding	1.431629701	0.00471468	0.00687848	up
ENSG00000144339	TMEFF2	protein_coding	1.580074975	0.004767942	0.006953416	up
ENSG00000111348	ARHGDIB	protein_coding	1.580074975	0.004767942	0.006953416	up
ENSG00000005421	PON1	protein_coding	1.49846121	0.004785189	0.006975789	up
ENSG00000204961	PCDHA9	protein_coding	1.49846121	0.004785189	0.006975789	up
ENSG00000100167	3-Sep	protein_coding	1.158611207	0.004861559	0.00707929	up
ENSG00000197568	HHLA3	protein_coding	-1.004887525	0.004966511	0.007224136	down
ENSG00000100271	TTLL1	protein_coding	-1.210622603	0.005020208	0.007298375	down
ENSG00000271425	NBPF10	protein_coding	1.184146299	0.005224279	0.007585012	up
ENSG00000100346	CACNA1I	protein_coding	-1.458060153	0.005294862	0.007684104	down
ENSG00000052850	ALX4	protein_coding	-1.044922187	0.005792074	0.008372118	down
ENSG00000108786	HSD17B1	protein_coding	1.006608114	0.005929292	0.008561818	up
ENSG00000212747	RTL8B	protein_coding	1.246074048	0.00595452	0.008595611	up
ENSG00000141449	GREB1L	protein_coding	-1.256426292	0.006041369	0.008714108	down

ENSG00000099260	PALMD	protein_coding	1.284142089	0.006302894	0.009069494	up
ENSG00000177076	ACER2	protein_coding	1.328536208	0.006621186	0.009507981	up
ENSG00000135604	STX11	protein_coding	1.328536208	0.006621186	0.009507981	up
ENSG00000251537	AC005324.3	protein_coding	-2.256426292	0.006640859	0.009533321	down
ENSG00000145736	GTF2H2	protein_coding	-2.256426292	0.006640859	0.009533321	down
ENSG00000180998	GPR137C	protein_coding	-2.256426292	0.006640859	0.009533321	down
ENSG00000120068	HOXB8	protein_coding	1.122085331	0.006726684	0.009653584	up
ENSG00000172006	ZNF554	protein_coding	1.122085331	0.006726684	0.009653584	up
ENSG00000144182	LIPT1	protein_coding	1.743573708	0.006774254	0.009718466	up
ENSG00000136206	SPDYE1	protein_coding	1.743573708	0.006774254	0.009718466	up
ENSG00000124593	AL365205.1	protein_coding	1.521181286	0.0071554	0.01023896	up
ENSG00000170099	SERPINA6	protein_coding	-2.578354387	0.007412257	0.010590409	down
ENSG00000011332	DPF1	protein_coding	-1.618996372	0.007725786	0.011013533	down
ENSG00000205085	FAM71F2	protein_coding	-1.034033871	0.007750177	0.01104687	down
ENSG00000105672	ETV2	protein_coding	-1.841388793	0.007786222	0.011093447	down
ENSG00000131650	KREMEN2	protein_coding	-1.841388793	0.007786222	0.011093447	down
ENSG00000183048	SLC25A10	protein_coding	-1.006448039	0.008288967	0.011766483	down
ENSG00000197978	GOLGA6L9	protein_coding	-1.006448039	0.008288967	0.011766483	down
ENSG00000133134	BEX2	protein_coding	-1.203958872	0.0091322	0.012903992	down
ENSG00000113396	SLC27A6	protein_coding	1.279626608	0.00950644	0.013408073	up
ENSG00000141540	TTYH2	protein_coding	1.328536208	0.0099957	0.014071028	up
ENSG00000125430	HS3ST3B1	protein_coding	1.459780742	0.010660062	0.014961507	up
ENSG00000083720	OXCT1	protein_coding	1.55092863	0.010715915	0.015029012	up
ENSG00000102003	SYP	protein_coding	1.55092863	0.010715915	0.015029012	up
ENSG00000162551	ALPL	protein_coding	1.130596831	0.0109016	0.015280979	up
ENSG00000176058	TPRN	protein_coding	-1.114407287	0.011083942	0.015516766	down
ENSG00000185038	MROH2A	protein_coding	2.328536208	0.011371779	0.015904174	up
ENSG00000188163	FAM166A	protein_coding	-2.13089541	0.012135703	0.01691012	down
ENSG00000081181	ARG2	protein_coding	-1.199842764	0.012354395	0.017198846	down
ENSG00000105613	MAST1	protein_coding	-1.199842764	0.012354395	0.017198846	down
ENSG00000167723	TRPV3	protein_coding	1.191032685	0.012667473	0.017616824	up
ENSG00000167981	ZNF597	protein_coding	-1.741853119	0.013349602	0.018506879	down
ENSG00000275793	RIMBP3	protein_coding	1.965966129	0.013977161	0.019336198	up
ENSG00000101460	MAP1LC3A	protein_coding	1.965966129	0.013977161	0.019336198	up
ENSG00000196420	S100A5	protein_coding	-2.426351294	0.014113454	0.019519829	down
ENSG00000185761	ADAMTSL5	protein_coding	-2.426351294	0.014113454	0.019519829	down
ENSG00000259431	THTPA	protein_coding	1.274088424	0.014396811	0.019885851	up
ENSG00000197857	ZNF44	protein_coding	-1.104423199	0.014968636	0.020632445	down
ENSG00000214128	TMEM213	protein_coding	1.087528109	0.015071839	0.020762536	up
ENSG00000197889	MEIG1	protein_coding	1.328536208	0.01515494	0.020871777	up
ENSG00000151617	EDNRA	protein_coding	1.328536208	0.01515494	0.020871777	up
ENSG00000156265	MAP3K7CL	protein_coding	-1.426351294	0.015244326	0.020982602	down
ENSG00000235863	B3GALT4	protein_coding	1.591570614	0.01606526	0.022057262	up
ENSG00000135362	PRR5L	protein_coding	1.591570614	0.01606526	0.022057262	up
ENSG00000127951	FGL2	protein_coding	1.480539302	0.016107066	0.022103607	up
ENSG00000164841	TMEM74	protein_coding	1.480539302	0.016107066	0.022103607	up
ENSG00000123243	ITIH5	protein_coding	1.112807517	0.016392149	0.022481714	up
ENSG00000167103	PIP5KL1	protein_coding	-1.195025748	0.016754185	0.022956265	down
ENSG00000254732	AP001931.1	protein_coding	-1.195025748	0.016754185	0.022956265	down
ENSG00000167608	TMC4	protein_coding	-1.140949075	0.01852778	0.025244675	down
ENSG00000204311	PJVK	protein_coding	1.023681627	0.018864694	0.025684611	up
ENSG00000180938	ZNF572	protein_coding	2.203005326	0.019124991	0.026027178	up
ENSG00000178922	HYI	protein_coding	-1.09292756	0.02025787	0.02748603	down
ENSG00000125246	CLYBL	protein_coding	1.217504896	0.020574298	0.027882062	up

ENSG00000154309	DISP1	protein_coding	1.04313399	0.020691884	0.028020667	up
ENSG00000128268	MGAT3	protein_coding	1.267135664	0.021911704	0.029583764	up
ENSG00000188368	PRR19	protein_coding	-1.993391886	0.021958875	0.029634092	down
ENSG00000107485	GATA3	protein_coding	-1.993391886	0.021958875	0.029634092	down
ENSG00000129757	CDKN1C	protein_coding	-1.634937916	0.022619591	0.030470828	down
ENSG00000226174	TEX22	protein_coding	-1.634937916	0.022619591	0.030470828	down
ENSG00000261308	FIGNL2	protein_coding	1.065501803	0.022650615	0.03050389	up
ENSG00000064655	EYA2	protein_coding	-1.189312096	0.022785293	0.030658945	down
ENSG00000135740	SLC9A5	protein_coding	-1.011313794	0.02355881	0.031639025	down
ENSG00000204618	RNF39	protein_coding	1.650464303	0.024079784	0.032303129	up
ENSG00000119121	TRPM6	protein_coding	-1.343889134	0.024208787	0.032461648	down
ENSG00000102904	TSNAXIP1	protein_coding	1.091497011	0.024731278	0.03312855	up
ENSG00000147174	GCNA	protein_coding	-2.256426292	0.026674618	0.035589923	down
ENSG00000162572	SCNN1D	protein_coding	-1.715857911	0.029987262	0.039728178	down
ENSG00000197558	SSPO	protein_coding	1.203005326	0.031408835	0.041521366	up
ENSG00000183691	NOG	protein_coding	2.065501803	0.031943873	0.042181276	up
ENSG00000259030	FPGT-TNNI3K	protein_coding	-1.355961966	0.033037243	0.043576148	down
ENSG00000163536	SERPINI1	protein_coding	1.258146881	0.033557419	0.04422154	up
ENSG00000010310	GIPR	protein_coding	-1.118922769	0.034321073	0.045175473	down
ENSG00000160051	IQCC	protein_coding	-1.118922769	0.034321073	0.045175473	down
ENSG00000205609	EIF3CL	protein_coding	1.743573708	0.035957475	0.047180492	up
ENSG00000182612	TSPAN10	protein_coding	1.421645613	0.036797664	0.0482253	up
ENSG00000099904	ZDHHC8	protein_coding	-1.519460698	0.037820188	0.049461095	down
ENSG00000115239	ASB3	protein_coding	-1.256426292	0.037947358	0.049613622	down
