# Supplementary material for: Insight of a Metabolic Prognostic Model to Identify Tumor Environment and Drug Vulnerability for Lung Adenocarcinoma
Source: Front Immunol. 2022 Jun 23;13:872910. doi: 10.3389/fimmu.2022.872910 (PMC9262104; doi:10.3389/fimmu.2022.872910)
Supplement: Supplementary file 3 [file DataSheet_2.pdf]

Supplementary Table S2:The Kyoto Encyclopedia of Genes and Genomes (KEGG) pathways analysis of DEGs between A549 and A549RR cells.

ID	Description	GeneRatio	BgRatio	pvalue	geneID
hsa01100	Metabolic pathways	295/1347	1499/8095	0.00032673	PTGS2/ATP6V1F/GPX4/SMS/HKDC1/PYGL/PLCB1/RRM2/ST6GAL2/FUT8/ALDH2/SMOX/PLCE1/TRAK2/MGLL/NDUFA1/ATP6V1C1/NT5C3B/NME4/HMOX1/NNT/PIGM/ASS1/UGT1A9/DTYMK/CD38/SPR/COX6A1/PC/GPX1/PRPS2/ATP6V0A2/TK2/CKB/PCK2/EXT1/NT5C3A/ACSL1/ACOT2/SRR/A4GALT/ALG10/DHCR7/IMPA1/EHHADH/AMDHD1/GYG2/ADCY3/PTGS1/ST3GAL1/HMGCL/MAN1A1/XYLT2/ALG1/SUV39H1/TKFC/ALDH5A1/PNPO/UAP1/SGPP2/DPM3/LAP3/PANK1/LPCAT4/KMO/PIPOX/KHK/ST3GAL2/PLCB4/C1GALT1/DGKG/PPAT/IPMK/ITPKB/MGAT2/GMPPA/CPS1/CA8/B3GALT5/GPX3/DHFR2/UGT1A1/AUH/PCBD2/DPYD/DGAT1/MGAM/MRI1/BPNT1/RFK/GALNS/UGT1A6/AK7/ALDOC/FHIT/EZH1/CHSY3/XYLT1/SETDB2/NME2/GPHN/DHODH/DCK/PPT2/GGT5/ACOX2/PLA2G4C/SGPP1/SMPD1/GUCY1A2/DHRS4/GGCT/PAFAH2/KYAT1/NAT1/NME3/NEU1/NUDT2/LSS/MBOAT1/MIF/DGKE/JMJD7-PLA2G4B/PPOX/GAL3ST1/DOT1L/ITPKA/HSD3B7/RPIA/ELOVL7/ADCY5/CHST10/B4GALT3/COQ7/ACP2/GSTM4/INPP1/GYS2/FPGT/GBA2/BTD/GLUL/MTM1/XDH/RPEL1/PDE6D/COX8A/MVK/OLAH/PDE5A/PIGQ/HGD/GALT/AOC2/GSTZ1/ATP6V0E2/CYP26A1/DEGS2/POMT1/HSD17B8/NDUFS7/GALNT9/NPL/NDUFV2/BDH2/GCDH/HPD/CYP27B1/MPPE1/CAMKMT/ALDH4A1/CHAC2/NAGA/DGAT2/PGAM4/ACADS/B4GALNT3/PLA2G6/ALG14/ASL/B4GAT1/PDE1C/ADCY1/ELOVL4/HYKK/OPLAH/AOC3/AASS/PIGV/UQCRHL/BST1/ADH6/CHAC1/ASMT/COX7A1/INPP5E/AMPD3/GPLD1/ST3GAL6/GLYCTK/TGDS/SMYD3/MTMR8/DGKQ/PIK3CG/ATP6V0A4/CDS1/LIPC/GCSH/LDHD/HSD17B7/NMRK1/GALNT17/SELENBP1/GCNT1/PIGH/ACYP1/ST6GALNAC6/GALNT5/AK8/NDUFB8/IDUA/ENO4/P4HA3/PDE9A/NAPRT/ACSS3/AOX1/QPRT/PLCH2/AK9/RBKS/NOS2/ST6GALNAC3/MCAT/TDO2/BCO1/GALNT16/ASNS/CBS/GCH1/SMPD2/ACADL/SETMAR/AMACR/SMPD3/FUT1/SPHK1/HMGCLL1/HYAL3/ACSBG1/CARNS1/GLS2/GCAT/ALDH8A1/AKR1C4/CYP27A1/HPSE/ETNPPL/ALDH1A2/LIPT2/BDH1/PDE3A/PDE1A/NDUFA7/AGPAT4/HAL/GAPDHS/CYP1A1/ATP6V1B1/CA9/PLCB2/IL4I1/NAGS/HK2/OXCT2/ADH1C/LDHAL6B/NMNAT3/B3GALT1/B3GAT2/MTHFS/PLCD4/PHOSPHO2/HSD17B1/ACER2/LIPT1/OXCT1/ALPL/ARG2/THTPA/B3GALT4/PIP5KL1/HYI/MGAT3

hsa05168	Herpes simplex virus 1 infection	121/1347	497/8095	3.60E-06	ALYREF/CCL2/TP53/C5/ZNF300/ZNF546/ZNF81/ZNF33A/ZNF37A/ZNF343/ZNF3/ZNF792/IKBKE/ZNF316/ZNF180/ZNF786/PIK3R3/ZNF846/ZNF573/ZNF254/ZNF189/ZNF432/ZNF552/ZNF627/ZNF304/ZNF699/ZNF605/ZNF891/ZNF222/ZNF235/ZNF585B/ZNF585A/ZNF549/ZNF544/ZNF529/ZNF566/ZNF845/ZNF2/ZNF548/ZNF425/ZNF773/ZNF616/ZFP37/RNASEL/ZSCAN32/ZNF670/ZNF569/IFIH1/ZNF169/ZNF229/ZNF688/PILRB/ZNF285/ZNF614/ZNF30/ZNF675/ZNF543/ZNF248/ZNF853/ZNF705E/ZNF708/ZNF17/ZNF559/ZNF57/TRAF6/ZNF841/ZNF250/ZNF10/ZNF470/ZNF772/ZNF684/ZNF175/ZNF19/ZNF350/ZNF256/ZNF461/ZNF212/ZNF34/ZNF519/ZNF324B/ZNF596/ZNF689/ZNF764/HLA-DMB/ZNF613/ZNF443/ZNF184/ZNF14/TLR3/ZNF225/HLA-F/ZNF568/HLA-DRA/ZNF714/ZNF490/ZNF517/ZNF182/ZNF112/ZNF599/ZNF420/ZNF418/ZNF611/ZNF25/CCL5/ZNF333/ZNF23/ZNF783/ZNF814/ITGB3/ZNF233/ZNF354C/ZNF583/BCL2/ZNF230/ZNF799/ZNF669/ZNF829/ZFP92/ZNF554/ZNF597/ZNF44
hsa04310	Wnt signaling pathway	37/1347	166/8095	0.03425616	RSPO3/DKK1/PLCB1/AXIN2/TP53/RNF43/FOSL1/PPP3R1/SFRP1/CSNK2B/PLCB4/PRICKLE3/WNT3/TCF7/SFRP5/NFATC1/CTNND2/GPC4/NKD1/APC2/FZD5/VANGL2/TLE2/WNT11/ROR2/MAPK10/NKD2/WNT9A/DAAM1/CXXC4/SOX17/NOTUM/PLCB2/FRAT1/PRKCG/FRAT2/FZD9
hsa04514	Cell adhesion molecules	34/1347	149/8095	0.03018036	VCAN/CNTN1/NRCAM/ITGAM/SDC3/CLDN2/SDC2/ESAM/CD22/NRXN3/CDH4/IGSF11/L1CAM/OCLN/ITGB7/ICAM1/LRRC4B/VSIR/NLGN1/CLDN15/ICAM3/HLA-DMB/CLDN23/CLDN3/HLA-F/HLA-DRA/PTPRC/ITGA4/CD40/ITGA9/CD58/NRXN2/CLDN4/CDH3
hsa04911	Insulin secretion	25/1347	86/8095	0.00271041	PLCB1/SLC2A1/SNAP25/PCLO/RIMS2/ADCY3/PLCB4/KCNMB4/CREB3/FXYD2/VAMP2/ADCY5/ADCY1/KCNU1/ATP1A3/KCNJ11/KCNMB3/RAB3A/CREB3L1/CCK/ABCC8/KCNN3/PLCB2/PRKCG/RAPGEF4
hsa04933	AGE-RAGE signaling pathway in diabetic complications	25/1347	100/8095	0.02063831	FN1/PLCB1/CCL2/PLCE1/PIK3R3/PLCB4/COL4A6/COL4A3/THBD/NFATC1/VEGFB/AGER/TGFB3/STAT5A/CXCL8/EGR1/ICAM1/AGTR1/MAPK10/PRKCZ/MAPK11/PLCB2/BCL2/COL1A1/PLCD4
hsa04064	NF-kappa B signaling pathway	25/1347	104/8095	0.03254761	PTGS2/UBE2I/CXCL2/MALT1/CXCL1/TAB3/TRAF1/CSNK2B/RELB/IL1R1/GADD45A/GADD45B/EDARADD/EDA2R/CXCL8/ICAM1/CXCL3/CD14/TRAF6/CD40/BCL2A1/EDA/TNFRSF13C/BCL2/EDAR
hsa04928	Parathyroid hormone synthesis, secretion and action	25/1347	106/8095	0.04016722	PLCB1/RXRA/ARAF/GNA13/ADCY3/MMP16/PLCB4/CREB3/BRAF/ADCY5/NR4A2/VDR/EGR1/HBEGF/CYP27B1/ADCY1/RUNX2/PTHLH/CREB3L1/MMP25/PLCB2/BCL2/PRKCG/FOS/GATA3
hsa05412	Arrhythmogenic right ventricular cardiomyopathy	24/1347	77/8095	0.00114952	DSP/GJA1/CACNG6/CACNG8/DMD/TCF7/ATP2A3/ITGA1/PKP2/ATP2A1/ITGB7/CACNB4/CACNG4/SGCD/DSC2/ITGA10/ITGB6/CACNG7/ITGA4/ITGA9/CTNNA2/ITGA7/ITGB3/SLC8A1

hsa04713	Circadian entrainment	24/1347	97/8095	0.02591914	PLCB1/PER2/ADCY3/GRIN2B/PLCB4/CACNA1H/KCNJ6/GUCY1A2/ADCY5/GNG2/GNG4/PER3/NOS1AP/ADCY1/GNB3/GNGT1/RYR1/GNG10/CALML4/GRIN1/PLCB2/PRKCG/FOS/CACNA1I
hsa04512	ECM-receptor interaction	22/1347	88/8095	0.02867432	FN1/HSPG2/THBS3/CD47/COL4A6/COL4A3/ITGA1/ITGB7/CHAD/TNC/COL9A3/COL6A1/ITGA10/ITGB6/LAMB4/TNXB/ITGA4/ITGA9/ITGA7/ITGB3/VTN/COL1A1
hsa04146	Peroxisome	21/1347	82/8095	0.0249248	CRAT/SLC27A2/ABCD3/ACSL1/EHHADH/HMGCL/PIPOX/PHYH/PEX14/ACOX2/DECR2/DHRS4/PEX3/XDH/MVK/PAOX/MPV17L/NOS2/PEX7/AMACR/HMGCLL1
hsa04610	Complement and coagulation cascades	21/1347	85/8095	0.0361096	TFPI/CFH/C5/ITGAM/FGA/THBD/PROC/CFD/C4BPA/CFHR3/F13B/C2/FGB/C4A/F7/C7/ITGAX/SERPINA1/F12/VTN/PLAT
hsa03320	PPAR signaling pathway	19/1347	76/8095	0.04005881	RXRA/SLC27A2/ILK/PCK2/ACSL1/EHHADH/SLC27A1/PPARA/SORBS1/ACOX2/ANGPTL4/CPT1C/ACADL/ACSBG1/CYP27A1/AQP7/FABP6/FABP5/SLC27A6
hsa04924	Renin secretion	18/1347	69/8095	0.03036528	PLCB1/PPP3R1/PLCB4/GUCY1A2/ADRB2/ADCY5/ADORA1/ACE/ORAI1/NPPA/AGTR1/PDE1C/EDN2/PDE3A/PDE1A/CALML4/PLCB2/EDNRA
hsa05217	Basal cell carcinoma	17/1347	63/8095	0.02544866	AXIN2/TP53/PTCH1/GLI2/GLI1/WNT3/TCF7/GADD45A/GADD45B/APC2/GLI3/FZD5/WNT11/WNT9A/SHH/HHIP/FZD9
hsa00380	Tryptophan metabolism	12/1347	42/8095	0.03682376	CYP1B1/ALDH2/EHHADH/KMO/KYAT1/GCDH/ASMT/AOX1/TDO2/ALDH8A1/CYP1A1/IL4I1
hsa00650	Butanoate metabolism	9/1347	28/8095	0.03270476	EHHADH/HMGCL/ALDH5A1/BDH2/ACADS/HMGCLL1/BDH1/OXCT2/OXCT1
hsa00220	Arginine biosynthesis	8/1347	22/8095	0.02061618	ASS1/CPS1/GLUL/ASL/NOS2/GLS2/NAGS/ARG2
hsa00533	Glycosaminoglycan biosynthesis - keratan sulfate	5/1347	14/8095	0.04840584	FUT8/ST3GAL1/ST3GAL2/B4GALT3/CHST6
