# Supplementary material for: Insight of a Metabolic Prognostic Model to Identify Tumor Environment and Drug Vulnerability for Lung Adenocarcinoma
Source: Front Immunol. 2022 Jun 23;13:872910. doi: 10.3389/fimmu.2022.872910 (PMC9262104; doi:10.3389/fimmu.2022.872910)
Supplement: Supplementary file 4 [file DataSheet_3.pdf]

Supplementary Table S3: The Gene Ontology (GO) analysis of DEGs between A549 and A549RR cells.

GOID	GOTerm	Ontology Source	Group PValue	Associated Genes Found
GO:0000981	DNA-binding transcription factor activity, RNA polymerase II-specific	BiologicalProcess	0.00	[AHRR, AIRE, ALX1, ALX4, AREG, ARID3B, ARID5B, ARX, ATF3, ATF5, ATF7, BACH2, BARX1, BCL11A, BCL11B, BCL6, BHLHE41, CARF, CC2D1A, CREB3, CREB3L1, CREBRF, CREM, CSRNP3, CYP1A1, CYP27B1, DBP, DDN, DLX1, DLX2, DLX4, DMRTA1, DMRTA2, E2F6, EAF2, EBF1, EBF2, EBF3, EBF4, EGR1, ELK1, ELOF1, ESR2, ESRRG, ETV2, EZH1, FOS, FOSL1, FOSL2, FOXA1, FOXA3, FOXD1, FOXD3, FOXD4L1, FOXE1, FOXF2, FOXN4, FOXP1, FOXP2, FOXP4, GATA2, GATA3, GATA5, GATA6, GATD3A, GLI1, GLI2, GLI3, GRHL3, GSC, HES1, HES2, HEY1, HINFP, HLX, HMX1, HNF1A, HNF4A, HOXA1, HOXA10, HOXA13, HOXA5, HOXA6, HOXB13, HOXB2, HOXB3, HOXB5, HOXB6, HOXB7, HOXB8, HOXB9, HOXC6, HOXC8, HOXC9, HOXD1, HOXD10, HOXD13, HOXD9, HSF4, IFI16, IRF1, IRF4, IRF5, KCNH4, KLF16, KLF2, KLF4, KLF7, KLF9, KLRG1, LBX2, LHX4, LHX6, LHX8, LHX9, LMO2, MAFK, MAPRE3, MEF2B, MEIS1, MESP1, MGAM, MIA3, MIER3, MITF, MLXIPL, MNX1, MSX2, MXD3, MXI1, MYCL, MYPOP, NFATC1, NFIX, NFYA, NKX2-1, NKX2-3, NKX2-5, NKX3-1, NKX3-2, NKX6-1, NR1D1, NR1H4, NR2E3, NR2F6, NR3C2, NR4A1, NR4A2, NR4A3, NRL, OTX1, OVOL3, PAX2, PAX7, PAX8, PAX9, PBX2, PBX4, PCGF6, PITX1, PITX2, PKMYT1, PKNOX1, PLAG1, POU3F3, POU4F3, POU5F1, POU5F2, POU6F1, PPARA, PRDM1, PRDM5, PRRX2, RCOR2, RELB, RUNX2, RXRA, SALL1, SALL2, SARNP, SATB1, SATB2, SIM2, SIX4, SNAI1, SOX17, SOX2, SOX21, SOX4, SOX5, SOX6, SOX7, SOX9, SP140L, SP8, STAT4, STAT5A, TBX20, TBX3, TBX6, TCF7, TEAD3, TFAP2C, THAP11, THAP12, THAP6, THAP8, TP53, TRPS1, TSC22D4, TSHZ3, TWIST1, USF3, VDR, YY2, ZBED3, ZBTB17, ZBTB2, ZBTB32, ZBTB34, ZBTB45, ZBTB46, ZC3H6, ZFX, ZKSCAN2, ZKSCAN3, ZMYM5, ZMYM6, ZNF174, ZNF175, ZNF202, ZNF239, ZNF300, ZNF335, ZNF350, ZNF396, ZNF487, ZNF511, ZNF524, ZNF639, ZNF654, ZNF668, ZSCAN21]
GO:0001228	DNA-binding transcription activator activity, RNA polymerase II-specific	BiologicalProcess	0.01	[AIRE, ALX1, ALX4, AREG, ARID3B, ATF3, ATF5, BARX1, BCL11B, CARF, CREB3, CREB3L1, CREBRF, CSRNP3, DBP, DLX2, EAF2, EBF1, EBF2, EBF3, EBF4, EGR1, ELK1, ELOF1, ESRRG, ETV2, FOS, FOSL1, FOSL2, FOXA1, FOXD1, FOXF2, GATA2, GATA3, GLI2, GRHL3, HNF1A, HNF4A, HOXA1, HOXA10, HOXA5, HOXB2, HOXB3, HOXB5, HOXB7, HOXD10, HOXD13, IRF1, IRF4, IRF5, KCNH4, KLF4, KLF7, KLRG1, LHX4, LMO2, MAPRE3, MEF2B, MEIS1, MESP1, MGAM, MITF, MYCL, NFATC1, NFYA, NKX2-5, NR1H4, NR2E3, NR4A1, NR4A2, NR4A3, NRL, OTX1, OVOL3, PAX8, PAX9, PBX2, PBX4, PITX1, PLAG1, POU3F3, POU4F3, POU5F1, POU5F2, POU6F1, PPARA, RUNX2, SALL2, SATB2, SIX4, SOX17, SOX2, SOX21, SOX4, SOX9, STAT4, STAT5A, TBX20, TBX3, TBX6, TCF7, TEAD3, TFAP2C, TP53, YY2, ZBTB17, ZNF175, ZNF300, ZNF639, ZNF654, ZSCAN21]
GO:0017157	regulation of exocytosis	BiologicalProcess	0.01	[ADCY1, ADORA2B, BACE1, BAIAP3, BRAF, CACNA1H, CACNA1I, CDK5R2, DOC2A, DRD1, EXPH5, FGA, FGB, GAB2, GATA2, HAP1, HLA-F, HMOX1, HYAL3, ITGAM, KCNB1, LGALS9, MICAL1, NLGN1, NPPA, NRN1, NRXN3, P2RY1, PCDHA4, PCLO, PLA2G6, PRKCG, RAB27B, RAB33B, RAB3A, RAPGEF4, RIMS2, RIMS4, SMPD3, SNAPIN, SNCA, STXBP1, STXBP5L, SYP, SYT13, SYT17, SYT3, SYT6, SYTL4, TRIM9, UNC13B, UNC13D, VAMP1, VAMP2, VAMP7, VAMP8, VPS18, VPS4A, ZP3]
GO:0021953	central nervous system neuron differentiation	BiologicalProcess	0.00	[ARX, ATF5, ATP7A, BCL11B, C21orf91, CHD5, DCLK1, DISC1, DKK1, DLX1, DLX2, DRD1, EPHA4, EPHB3, FGFR2, FOXN4, GATA2, GATD3A, GBA2, GLI2, GLI3, HES1, HOXD10, INHBA, LHX4, LHX6, LHX8, MAP2, MAPT, MDGA1, MNX1, NANOS1, NKX2-1, NKX6-1, NR4A2, OPHN1, PAX7, PLXNA3, PTCH1, SALL1, SATB2, SCYL3, SFRP1, SHH, SLC4A10, SLIT2, SMOX, SOX4, SPOCK1, SPTBN4, TBX20, TTLL1, UNC5D, WNT3, ZC4H2, ZNF335]
GO:0043009	chordate embryonic development	BiologicalProcess	0.00	[ACVR2A, ACVRL1, ADAMTS3, ADM, AKAP3, ALDH1A2, ALX1, ALX4, AMOT, ANGPT1, APBA3, AREG, ATP7A, AXIN2, BRCA2, BTN3A3, BYSL, CAMSAP3, CCDC24, CCDC62, CITED2, CNOT3, COL1A1, CTHRC1, DACT1, DKK1, DLC1, DLEC1, DLL1, DLX1, DLX2, DSC2, DSC3, DSCAML1, EDNRA, ENDOG, ERCC2, ETV2, EXT1, EYA1, FBXW8, FGFR2, FLCN, FOSL1, FOXA1, FOXD3, FUT8, FUZ, FZD5, FZD9, GATA2, GATA3, GATA6, GATD3A, GLI2, GLI3, GLMN, GNA13, GRHL3, GSC, HES1, HEY1, HINFP, HNF1A, HOXA1, HOXA5, HOXA6, HOXB2, HOXB3, HOXB5, HOXB6, HOXB7, HOXB8, HOXB9, HOXC6, HOXC9, HOXD1, HOXD10, HOXD9, HS3ST6, HSD17B7, HYAL1, IFT52, KBTBD8, KDM4C, KLF2, KRT19, LFNG, LPAR6, LRP2, MESP1, MIA3, MKS1, MMP16, MUC1, NANOS3, NKD1, NKX2-5, NKX3-1, NKX3-2, NOG, NPHP3, NPHP3-ACAD11, NRARP, NRK, OSR2, PAX2, PAX7, PCSK5, PELO, PHLDA2, PNLDC1, POGLUT1, POU5F1, PRDM1, PTCH1, PTPN18, ROR2, RPGRIP1L, RSPO3, RUNX2, RXRA, SALL2, SALL4, SATB2, SFRP1, SHH, SIX4, SLC35E2B, SLC39A3, SMARCB1, SMIM14, SMOX, SNAI1, SOX17, SOX4, SOX6, SOX9, ST14, STMN3, SULF2, TAF8, TAPT1, TBX3, TBX6, TCF7, TET1, TGFB3, TP53, TRAF6, TTPA, TWIST1, UBE2B, VANGL2, VASH1, VASH2, WDTC1, WNT11, WNT9A, XYLT1, ZNF335, ZNF568, ZP3]
GO:0043632	modification-dependent macromolecule catabolic process	BiologicalProcess	0.00	[ANAPC10, ANAPC15, ANKZF1, ARAF, CAMLG, CLEC16A, COMMD1, DERL3, DISC1, ERCC8, ERLIN2, FBXL13, FBXL14, FBXL15, FBXL17, FBXL2, FBXL22, FBXL6, FBXO10, FBXO48, FBXW8, FEM1C, FHIT, FOXF2, GLMN, HECTD3, HFE, HSPA1A, IFI27, KAT5, KCTD21, KCTD6, KLHL3, LRRC29, MAGI1, MRPL23, MTM1, MVB12B, NHLRC1, NHLRC3, NKD2, PCYOX1L, PELI1, PLK2, PSMA1, PSMA2, PSMB10, PSMB8, PSMB9, PSMD12, PSMD9, RCHY1, RHBDD2, RNF103, RNF122, RNF125, RNF144B, RNF166, RNF185, RNF19A, RNF43, RNF8, SH3RF2, SHH, SPOP, TGFB1I1, TMUB2, TRIB2, TRIM13, TRIM2, TRIM9, UBA1, UBAP1L, UBE2B, UBE2D4, UBE2I, UBE2S, UBL7, USP18, USP20, USP28, USP30, USP35, USP38, USP43, USP44, USP45, USP50, USP51, USP9Y, VPS37D, VPS4A, ZYG11B]
GO:0045087	innate immune response	BiologicalProcess	0.00	[ADGRB1, AGER, AKR1C1, APOE, APOL1, AQP4, ARG2, ASS1, C2, C4A, C4BPA, C5, C7, CCL2, CCL26, CCL5, CD14, CD40, CD47, CD58, CDC42EP2, CFD, CFH, CHAMP1, CIITA, CYBC1, CYP27B1, DEFB1, EGR1, EHHADH, ERAP1, EREG, F12, FGA, FGB, GATA3, GBP3, GBP4, GCH1, GSDMD, HFE, HLA-DRA, HLA-F, ICAM1, ICAM3, IFI16, IFI27, IFIH1, IFIT2, IFITM2, IFITM3, IKBKE, IL1RAP, IL36B, IRF1, IRF4, IRF5, ISG20, ITGAM, KLRG1, KRT16, LAG3, LGALS3, LGALS9, MALT1, MATR3, MICA, MID1, MID2, MIF, MR1, MUC1, MUC16, MUC20, MUC3A, MUC5AC, MUC5B, NLRC3, NOD2, NOS2, NR1H4, OASL, PDE12, PIK3CG, POLR3G, POLR3K, PQBP1, PRDM1, PRKD1, PSMA1, PSMA2, PSMB10, PSMB8, PSMB9, PSMD12, PSMD9, PYCARD, RAB43, RBM14, RELB, RNASEL, RNASET2, RNF135, RNF166, SNCA, SPON2, STAR, STXBP1, SUSD4, TAB3, TAC4, TKFC, TLR3, TLR5, TP53, TRAF6, TRIM13, TRIM22, TRIM29, TRIM4, TRIM68, TTC4, TTLL12, ULBP2, UNC13D, UNC93B1, USP18, VAMP7, ZDHHC11]
GO:0048562	embryonic organ morphogenesis	BiologicalProcess	0.00	[ALX1, ALX4, ATP6V1B1, BBS5, CHRNA9, CITED2, CTHRC1, DLL1, DLX2, DSCAML1, EFNA1, EYA1, FGFR2, FOXE1, FOXF2, FOXN4, FRS2, FUZ, FZD5, FZD9, GATA2, GATA3, GATD3A, GLI1, GLI2, GLI3, GRHL3, GSC, HES1, HLX, HOXA1, HOXA5, HOXA6, HOXB2, HOXB3, HOXB5, HOXB6, HOXB7, HOXB8, HOXB9, HOXC9, HOXD10, HOXD9, HYAL1, IFT52, LRIG1, MESP1, MMP16, MYO3A, MYO7A, NKX2-5, NKX3-2, NOG, NPHP3, NPHP3-ACAD11, NR4A3, OSR2, OTX1, PAX2, PAX8, PDZD7, POU4F3, ROR2, RUNX2, SALL1, SATB2, SETDB2, SHH, SIX4, SLC39A3, SLITRK6, SMOX, SOX17, SOX9, STOX1, TBX20, TBX3, TBX6, TCF7, TGFB3, TPRN, TWIST1, VANGL2, WHRN, WNT11, WNT9A]
GO:0048568	embryonic organ development	BiologicalProcess	0.00	[ADM, ALDH1A2, ALX1, ALX4, ATP6V1B1, BBS5, CHRNA9, CITED2, CTHRC1, CXCL8, DLL1, DLX2, DSCAML1, EFNA1, ERCC2, EYA1, FBXW8, FGFR2, FOXE1, FOXF2, FOXN4, FRS2, FUZ, FZD5, FZD9, GATA2, GATA3, GATD3A, GLI1, GLI2, GLI3, GRHL3, GSC, HES1, HEY1, HLX, HOXA1, HOXA5, HOXA6, HOXB2, HOXB3, HOXB5, HOXB6, HOXB7, HOXB8, HOXB9, HOXC9, HOXD10, HOXD9, HYAL1, IFT52, KRT19, LRIG1, MESP1, MIA3, MKS1, MMP16, MYO3A, MYO7A, NES, NKX2-5, NKX3-2, NOG, NPHP3, NPHP3-ACAD11, NR4A3, NRK, OSR2, OTX1, PAX2, PAX8, PBX2, PBX4, PCSK5, PDZD7, PHLDA2, POU4F3, PPP1R13L, PRDM1, PTCH1, ROR2, RSPO3, RUNX2, SALL1, SATB2, SETDB2, SH2B3, SHH, SIX4, SLC39A3, SLITRK6, SMOX, SNAI1, SOX17, SOX9, ST14, STOX1, TBC1D23, TBX20, TBX3, TBX6, TCF7, TEAD3, TGFB3, TP53, TPRN, TTPA, TWIST1, VANGL2, VASH1, VASH2, WHRN, WNT11, WNT9A, ZNF568]
GO:0048608	reproductive structure development	BiologicalProcess	0.00	[ACVR2A, ADAM19, ADM, AREG, ARID5B, ASB1, BCL2, BRCA2, CITED2, CNRIP1, COL9A3, CTSV, CXXC1, CYP27B1, DEDD, DMC1, DMRTA1, EAF2, EPOR, EREG, ETV2, FBXW8, FGFR2, FKBPL, FOSL1, FOXA1, FOXF2, FRS2, FST, FZD5, GATA2, GATA3, GATA6, GATD3A, GJA1, GJB2, GLI1, GLI2, GLI3, GNRH1, GREB1L, HES1, HEY1, HID1, HNF1A, HOXA10, HOXB13, HOXD13, HYAL3, ICAM1, INHA, INHBA, KDM5B, KRT19, LFNG, LHX4, LHX8, LHX9, LRP2, LRRC6, MAMLD1, MIA3, MME, NANOS3, NKX2-1, NKX3-1, NOG, NRK, NUDT1, NUPR1, PEG10, PHLDA2, PLAG1, PRDM1, PRDX4, PTCH1, PTGS2, RNF38, ROR2, RSPO3, RXRA, SALL1, SERPINE2, SFRP1, SHH, SIX4, SLIT1, SLIT2, SLIT3, SNAI1, SOX9, ST14, STAR, STC1, STC2, TBX3, TCF7, TEX19, TFAP2C, TLR3, TLR5, TNC, TPPP3, TTPA, VASH1, VASH2, VDR, VGF, WDR77, ZNF568, ZP3]
GO:0006396	RNA processing	BiologicalProcess	0.00	[A1CF, ALYREF, AURKAIP1, BMT2, BOP1, BYSL, C1D, C9orf64, CCDC130, CLASRP, COQ7, CSTF2, DDX47, DDX49, DDX51, DHX37, DTWD2, DUS3L, EIF6, ELAC1, ERCC2, ESRP1, EXOSC1, FAM98B, FCF1, FOXE1, FTSJ1, GEMIN2, GEMIN7, GEMIN8, GPKOW, GTF2H2, GTF2H4, HELQ, HSPA1A, IMP3, INTS12, INTS5, ISG20, KAT2B, KHDRBS3, KHSRP, KTI12, LAGE3, LGALS3, LSM5, METTL15, METTL2A, METTL2B, MPHOSPH10, MPHOSPH6, MRM2, MRPL1, MTPAP, NOVA1, NSUN3, OSGEP, OSGEPL1, PABPN1, PAN2, PAPOLG, PCBP4, PDE12, PELP1, PIH1D2, POLR2H, POLR2J, POLR3K, POP5, PPP1R8, PPWD1, PQBP1, PRORP, PTCD2, PUS10, PWP2, RAVER1, RBM3, RBM7, RDH14, RIOK2, RNASEL, RNF113A, RNGTT, RPP40, RPRD2, RPS26, RPS28, RPUSD1, RPUSD2, RRAGC, RRP15, RRP8, SARNP, SCAF4, SLBP, SNRPB, SNRPF, SNRPN, SRFBP1, SRSF12, SUPT4H1, SUV39H1, TELO2, TMTC1, TOE1, TP53, TPRKB, TRDMT1, TRMT11, TRMT61A, TRMT61B, TRPT1, TRUB2, TSEN2, TSR3, TUT1, TXNL4A, TXNL4B, TYW5, U2AF1, U2AF1L4, U2AF1L5, URM1, UTP15, UTP20, WDR43, WDR77, YBEY, ZC3H10, ZC3H11A, ZCCHC4, ZNF326, ZNHIT3, ZRSR2]
GO:0048705	skeletal system morphogenesis	BiologicalProcess	0.00	[ALPL, ALX1, ALX4, ANXA6, ARID5B, AXIN2, CHAD, CITED2, COL11A2, COL12A1, COL13A1, COL1A1, COL6A1, DLX2, DSCAML1, EYA1, FGFR2, FGFR3, FUZ, GLI3, GREM1, GSC, HAS2, HHIP, HOXA1, HOXA5, HOXA6, HOXB2, HOXB3, HOXB5, HOXB6, HOXB7, HOXB8, HOXB9, HOXC8, HOXC9, HOXD10, HOXD9, HYAL1, LTBP2, MATN2, MATN3, MMP16, MSX2, NKX3-2, NOG, OSR2, PEX7, RAB33B, ROR2, RUNX2, SATB2, SFRP1, SIX4, SLC39A3, SMPD3, SOX5, SOX6, SOX9, SPEF2, STC1, TGFB3, THBS3, TWIST1, WNT9A]
GO:0048762	mesenchymal cell differentiation	BiologicalProcess	0.01	[ALDH1A2, ALX1, ANXA6, AXIN2, BCL2, CITED2, COL1A1, EDNRA, EFNA1, EFNB1, EPHA3, FBXL17, FGFR2, FN1, FOXA1, FOXF2, FUZ, GATA3, GATA5, GATD3A, GLIPR2, GREM1, GSC, HAS2, HES1, HEY1, IL17RD, KBTBD8, LDLRAD4, LOXL3, MAD2L2, MCRIP1, MSX2, NKX2-1, NOG, OLFM1, PAX2, PITX2, RET, SEMA3B, SEMA3D, SEMA3E, SEMA4G, SEMA6A, SFRP1, SHH, SMOX, SNAI1, SOX9, SPRY1, SRFBP1, TAPT1, TGFB1I1, TGFB3, TIAM1, TWIST1, USF3, WNT11]
GO:0051960	regulation of nervous system development	BiologicalProcess	0.00	[ABCC8, ACAP3, ADGRA2, ADGRB1, ADGRL1, ANKRD1, APBB1, APOE, ARHGAP33, ARHGAP44, BAIAP2, BCL11A, BCL11B, BCL2, BCL6, BEND6, BHLHE41, BMERB1, BRAF, BRINP3, C1D, C21orf91, CACNG7, CASZ1, CD38, CDH4, CDK5RAP3, CDON, CNTN1, COLQ, CRMP1, CTNNA2, CXCR4, CXXC1, DISC1, DKK1, DLL1, DLX1, DLX2, DMD, DMRTA2, DNAAF3, DNM2, DNM3, DOCK7, DPYSL3, EFNA1, EFNA3, ENC1, EPHA3, EPHA4, EPHA7, EPHB3, ESRP1, EYA1, FAIM, FBXW8, FKBPL, FLRT2, FLRT3, FN1, FOXA1, FSTL4, FUOM, FZD9, GATA2, GATA3, GATD3A, GJC2, GLI1, GLI2, GLI3, GNRH1, GORASP1, GPC2, GPC4, GPC6, GPR183, GRHL3, GRIN1, GRIP1, HAP1, HES1, HES2, HEY1, HOXB3, IGSF10, IL1RAP, ILK, ITGAX, ITPKA, KDM4C, KIF1A, KLF4, L1CAM, LHFPL4, LRCH4, LRIG2, LRP1, LRP2, LRRC24, LRRC4B, MAGI2, MAP2, MAPT, MDGA1, MEIS1, MFSD2A, MKS1, MME, MOB2, MYCL, NANOS1, NAP1L2, NCK1, NDNF, NEFL, NGEF, NKX2-5, NKX6-1, NLGN1, NME2, NOG, NPHP3, NPHP3-ACAD11, NR1D1, NRCAM, NTRK3, OLFM1, OXTR, P2RX4, PAX2, PCDHA4, PER2, PHACTR1, PLAG1, PLK2, PLXNA3, PLXNB3, PPP1R9A, PQBP1, PRKCH, PRKD1, PTK6, PTPN9, RAP1GAP, RAP1GAP2, RAP2A, RASSF10, RECK, RET, RIMS2, ROR2, RTN4RL1, RTN4RL2, SDC2, SEMA3B, SEMA3D, SEMA3E, SEMA4G, SEMA6A, SERPINE2, SERPINI1, SFRP1, SH3GL3, SHH, SIPA1L1, SIX4, SLC45A3, SLIT1, SLIT2, SLIT3, SLITRK3, SLITRK4, SLITRK6, SMOX, SNAP25, SNAPIN, SNCA, SOCS2, SOX2, SOX9, SPOCK1, SRCIN1, SRPX2, SS18L2, STAR, SYNDIG1, SYT17, SYT3, TBX6, THY1, TIAM1, TIAM2, TIMP1, TMEM98, TNFRSF12A, TNIK, TP53, TPPP, TRAK2, TRIM46, TRPV2, TSPO, TTPA, UNC5D, UXT, VAMP7, VANGL2, WASF3, WNT3, XK, ZC4H2, ZFYVE27, ZMYND8, ZNF335, ZNF804A]
GO:0060562	epithelial tube morphogenesis	BiologicalProcess	0.00	[ACVRL1, ADM, ALX1, AREG, BBS5, BCL2, CITED2, CSNK2B, CTHRC1, CTSH, CXCR4, CYP27B1, DLC1, DLEC1, DLL1, EDNRA, EPHA4, EPHA7, EYA1, FGFR2, FKBPL, FOXA1, FOXD1, FOXN4, FOXP1, FUZ, FZD9, GATA3, GATD3A, GLI2, GLI3, GLMN, GNA13, GREB1L, GREM1, GRHL3, HES1, HHIP, HOXA5, HOXB7, IFT52, ILK, ITGAX, KDM5B, KLHL3, LRP2, MESP1, MKS1, MSX2, MTSS1, NKX2-1, NKX2-5, NKX3-1, NOG, NPHP3, NPHP3-ACAD11, NRARP, PAX2, PAX8, PTCH1, RASIP1, RET, SALL1, SALL4, SEMA3E, SETDB2, SFRP1, SHH, SIX4, SLIT2, SMOX, SOX17, SOX4, SOX9, SPRY1, ST14, STOX1, TBX20, TBX3, TBX6, TNC, TRAF6, TWIST1, VANGL2, VDR, WNT11]
GO:0061138	morphogenesis of a branching epithelium	BiologicalProcess	0.01	[ADM, AREG, BCL2, CTSH, CYP27B1, EDNRA, EYA1, FGFR2, FKBPL, FOXA1, FOXD1, FRS2, FZD5, GLI2, GLI3, GNA13, GREB1L, GREM1, HHIP, HOXA5, HOXB13, HOXB7, HOXD13, ILK, KDM5B, MKS1, MSX2, NKX2-1, NKX3-1, NOG, NRARP, PAX2, PAX8, PTCH1, RASIP1, RSPO3, RXRA, SALL1, SEMA3E, SFRP1, SHH, SIX4, SLIT2, SMOX, SOX9, SPRY1, ST14, TBX20, TBX3, TNC, VANGL2, VDR]
GO:0070647	protein modification by small protein conjugation or removal	BiologicalProcess	0.00	[ACTR8, ADGRB1, ADRB2, ANAPC10, ANGPT1, AREG, ARRDC4, ASB1, ASB3, ASB4, ASB7, ASB8, ASB9, BABAM1, BCL11A, BCL2, BIRC7, CAMLG, CBLB, CBLL1, CDC25A, CDK5RAP3, CENPS, CISH, CLSPN, COMMD1, COPS6, DCAF10, DCAF8, DNAJA1, EGR1, ENC1, EPOP, ERCC8, EYA1, FBXL13, FBXL14, FBXL15, FBXL17, FBXL2, FBXL22, FBXL6, FBXO10, FBXO15, FBXW10, FBXW8, FBXW9, FEM1C, FOXF2, GATA3, GLMN, GORASP1, H2BC5, HECTD3, HERC3, HSPA1A, IFI27, IFIH1, JOSD1, KAT2B, KBTBD8, KCTD21, KCTD6, KIAA1586, KLHL13, KLHL21, KLHL3, MAD2L2, MAGI1, MALT1, MED11, MED30, MIA3, MIB2, MID2, MINDY1, MINDY3, MKRN3, MRPL23, MTBP, NAE1, NEURL1B, NEURL2, NEURL3, NHLRC1, NHLRC3, NOD2, PCGF1, PCGF6, PELI1, PELI3, PER2, PEX14, PINX1, PRKCG, PROC, PSMA1, PSMA2, PSMB10, PSMB8, PSMB9, PSMD12, PSMD9, RAB40AL, RAB40C, RAG1, RCE1, RCHY1, RNF103, RNF113A, RNF122, RNF125, RNF135, RNF144B, RNF166, RNF182, RNF185, RNF19A, RNF31, RNF38, RNF39, RNF43, RNF8, RNFT1, RUSC1, RWDD3, SENP5, SH3RF2, SH3RF3, SOCS2, SOX4, SPATA2, SPHK1, SPOP, SUMO4, SUPT3H, TP53, TRAF1, TRAF6, TRIB2, TRIM13, TRIM2, TRIM22, TRIM36, TRIM4, TRIM45, TRIM68, TRIM7, TRIM73, TRIM9, TSPO, UBA1, UBE2B, UBE2D4, UBE2I, UBE2M, UBE2S, UBE2T, UBOX5, UNKL, URM1, USP18, USP20, USP28, USP30, USP35, USP38, USP43, USP44, USP45, USP50, USP51, USP9Y, WASHC1, WDTC1, ZFP91, ZNF738]
GO:0006397	mRNA processing	BiologicalProcess	0.00	[A1CF, ALYREF, AURKAIP1, CLASRP, CSTF2, DDX47, ERCC2, ESRP1, FOXE1, GEMIN2, GEMIN7, GEMIN8, GPKOW, GTF2H2, GTF2H4, KHDRBS3, KHSRP, LGALS3, LSM5, MTPAP, NOVA1, PABPN1, PAN2, PAPOLG, PCBP4, PDE12, POLR2H, POLR2J, PPP1R8, PPWD1, PQBP1, PTCD2, RAVER1, RBM3, RBM7, RDH14, RNASEL, RNF113A, RNGTT, RPRD2, SARNP, SCAF4, SLBP, SNRPB, SNRPF, SNRPN, SRFBP1, SRSF12, SUPT4H1, TRUB2, TSEN2, TUT1, TXNL4A, TXNL4B, U2AF1, U2AF1L4, U2AF1L5, WDR77, ZC3H10, ZC3H11A, ZNF326, ZRSR2]
GO:0003700	DNA-binding transcription factor activity	BiologicalProcess	0.00	[ADCY1, AGER, AHRR, AIRE, ALX1, ALX4, AREG, ARID3B, ARID5B, ARX, ATF3, ATF5, ATF7, BACH2, BARX1, BCL11A, BCL11B, BCL6, BEX2, BHLHE41, CARF, CC2D1A, CD40, CDK5RAP3, COMMD1, CRAT, CREB3, CREB3L1, CREBRF, CREM, CSRNP3, CYP1A1, CYP1B1, CYP27B1, DBP, DDN, DLX1, DLX2, DLX4, DMRTA1, DMRTA2, DNPEP, DXO, E2F6, EAF2, EBF1, EBF2, EBF3, EBF4, EDA, EDA2R, EGR1, ELK1, ELOF1, EPHA5, ESR2, ESRRG, ETV2, EZH1, FANK1, FOS, FOSL1, FOSL2, FOXA1, FOXA3, FOXD1, FOXD3, FOXD4L1, FOXE1, FOXF2, FOXN4, FOXP1, FOXP2, FOXP4, GAS7, GATA2, GATA3, GATA5, GATA6, GATD3A, GLI1, GLI2, GLI3, GLMP, GREM1, GRHL3, GSC, HES1, HES2, HEY1, HINFP, HLX, HMOX1, HMX1, HNF1A, HNF4A, HOXA1, HOXA10, HOXA13, HOXA5, HOXA6, HOXB13, HOXB2, HOXB3, HOXB5, HOXB6, HOXB7, HOXB8, HOXB9, HOXC6, HOXC8, HOXC9, HOXD1, HOXD10, HOXD13, HOXD9, HSF4, HSPA1A, ICAM1, IFI16, IKZF4, IL18R1, IL1RAP, IRAK2, IRF1, IRF4, IRF5, KCNH4, KLF16, KLF2, KLF4, KLF7, KLF9, KLRG1, LAMTOR5, LBX2, LGALS9, LHX4, LHX6, LHX8, LHX9, LMO2, MAD2L2, MAFK, MALT1, MAP2K5, MAPK10, MAPK11, MAPRE3, MBTPS2, MEF2B, MEIS1, MESP1, MGAM, MIA3, MID2, MIER3, MITF, MLXIPL, MNX1, MSX2, MTURN, MXD3, MXI1, MYCL, MYPOP, NFATC1, NFIX, NFKBIB, NFYA, NKX2-1, NKX2-3, NKX2-5, NKX3-1, NKX3-2, NKX6-1, NLRC3, NOD2, NR1D1, NR1H4, NR2E3, NR2F6, NR3C2, NR4A1, NR4A2, NR4A3, NRL, NUPR1, OTX1, OVOL3, PAX2, PAX7, PAX8, PAX9, PBX2, PBX4, PCGF6, PELI1, PEX14, PGBD1, PHF1, PITX1, PITX2, PKMYT1, PKNOX1, PLAG1, POU3F3, POU4F3, POU5F1, POU5F2, POU6F1, PPARA, PRDM1, PRDM5, PRKCH, PRKCZ, PRKD1, PRNP, PRRX2, PTCH1, PYCARD, RCOR2, RELB, RHEBL1, RIPK4, RNF31, RTKN2, RUNX2, RWDD3, RXRA, SALL1, SALL2, SALL4, SARNP, SATB1, SATB2, SFRP5, SHH, SIGIRR, SIM2, SIX4, SMARCB1, SMOX, SNAI1, SOX17, SOX2, SOX21, SOX4, SOX5, SOX6, SOX7, SOX9, SP140L, SP8, SPHK1, STAT4, STAT5A, STK36, SUPT4H1, TAB3, TBX20, TBX3, TBX6, TCF19, TCF7, TEAD3, TFAP2C, THAP11, THAP12, THAP6, THAP8, TLR3, TP53, TRAF1, TRAF6, TRIM13, TRIM22, TRIM29, TRIM4, TRPS1, TSC22D4, TSHZ3, TSSK4, TWIST1, USF3, VDR, YY2, ZBED3, ZBTB17, ZBTB2, ZBTB32, ZBTB34, ZBTB45, ZBTB46, ZBTB48, ZC3H6, ZFP37, ZFP41, ZFX, ZKSCAN2, ZKSCAN3, ZKSCAN4, ZMYM5, ZMYM6, ZNF10, ZNF121, ZNF165, ZNF174, ZNF175, ZNF180, ZNF189, ZNF19, ZNF202, ZNF22, ZNF232, ZNF239, ZNF266, ZNF280A, ZNF280B, ZNF286B, ZNF296, ZNF3, ZNF300, ZNF319, ZNF322, ZNF329, ZNF335, ZNF33A, ZNF35, ZNF350, ZNF37A, ZNF396, ZNF423, ZNF444, ZNF449, ZNF487, ZNF497, ZNF511, ZNF524, ZNF529, ZNF554, ZNF559, ZNF566, ZNF599, ZNF613, ZNF614, ZNF628, ZNF639, ZNF654, ZNF668, ZNF669, ZNF675, ZNF684, ZNF688, ZNF691, ZNF786, ZNF814, ZNF821, ZNF845, ZNF846, ZNF891, ZSCAN2, ZSCAN21, ZSCAN25, ZSCAN26, ZSCAN30, ZSCAN31, ZSCAN32, ZSCAN5A, ZXDA]
GO:0004930	G protein-coupled receptor activity	BiologicalProcess	0.00	[ACKR3, ADGRA2, ADGRB1, ADGRD1, ADGRE1, ADGRF4, ADGRL1, ADGRL2, ADORA1, ADORA2B, ADRA1B, ADRB2, AGTR1, CCR1, CCR9, CXCR4, CXCR5, DRD1, EDNRA, ELOVL4, FFAR4, FZD5, FZD9, GABBR1, GIPR, GLP2R, GPR132, GPR153, GPR156, GPR160, GPR162, GPR179, GPR182, GPR183, GPR19, GPR21, GPR27, GPR35, GPR37, GPR52, GPR75, GPR85, GPRC5C, HRH1, HTR1D, KISS1R, LPAR4, LPAR6, LTB4R, LTB4R2, NPRL3, OR1J4, OXTR, P2RY1, P2RY11, P2RY6, PAX8, PCDHA4, PCDHA6, QRFPR, S1PR2, SMOX, SORCS2, SPHK1, SUCNR1, TAPT1, TAS1R1, TAS2R19, TAS2R30, TAS2R4, TAS2R5, TPRA1, VIPR1]

GO:0006325	chromatin organization	BiologicalProcess	0.00	[ACTR8, ALKBH4, APBB1, AUTS2, BABAM1, BANP, BCL6, BRCA2, BRD3, CBX7, CDAN1, CENPL, CENPS, CENPT, CENPV, CHD1, CHD3, CHD5, CHD9, CHRAC1, CPA4, CXXC1, DOT1L, DPF1, DRD1, ELOF1, EPC1, EPOP, EYA1, EYA2, EZH1, FLCN, FOXA1, FOXA3, GATA2, GATA3, GPX4, H1-10, H1-5, H2AC15, H2AZ1, H2BC5, H4C12, HDAC9, HIPK4, HMGN5, HNF1A, HR, HSF4, ING1, ING4, IRF4, KAT2B, KAT5, KAT6B, KDM4C, KDM5B, KDM7A, L3MBTL1, LRIF1, MAGI1, MAP3K12, MIER2, MIER3, MUC1, NAA60, NANOS1, NAP1L2, NAP1L3, NAP1L5, NR1H4, NR4A2, PADI1, PADI2, PADI3, PAGR1, PAX7, PCGF1, PCGF6, PER2, PHF1, PHF21A, PPM1D, PRDM11, PRDM13, PRDM5, PRKAA2, PRKD1, RAG1, RBM14, RNF8, RRP8, RSF1, SATB1, SATB2, SCMH1, SDR16C5, SETDB2, SETMAR, SETSIP, SMARCB1, SMYD3, SNAI1, SNCA, SOX2, SOX9, SUPT3H, SUPT4H1, SUV39H1, TADA2A, TAF6L, TDRD3, TET1, TMEM98, TP53, TTLL12, TWIST1, UBE2B, UCN, USP51, ZNF304, ZNF335]
GO:0006401	RNA catabolic process	BiologicalProcess	0.00	[AXIN2, CACNG7, CNOT3, DCP2, DDX49, DIS3L, DXO, EXOSC1, FASTKD3, GTPBP2, HSPA1A, ISG20, KHSRP, LSM5, MRPL23, MTPAP, NANOS1, NANOS3, NT5C3B, PAN2, PCBP4, PDE12, PELO, PNLDC1, PPP1R8, PRR5L, PSMA1, PSMA2, PSMB10, PSMB8, PSMB9, PSMD12, PSMD9, RBM7, RDH14, RNASEL, RNASET2, RPL36A, RPL9, RPS10, RPS26, RPS28, SCGB1A1, SLFN11, SLFN13, SMG8, TNRC6A, TNRC6C, TOB1, TRDMT1]
GO:0008380	RNA splicing	BiologicalProcess	0.00	[ALYREF, CCDC130, CLASRP, COQ7, CSTF2, DDX47, ESRP1, FAM98B, FOXE1, GEMIN2, GEMIN7, GEMIN8, GPKOW, HSPA1A, KHDRBS3, KHSRP, LGALS3, LSM5, MPHOSPH10, NOVA1, PABPN1, PCBP4, POLR2H, POLR2J, PPP1R8, PPWD1, PQBP1, RAVER1, RBM3, RBM7, RNF113A, RPS26, RRAGC, SNRPB, SNRPF, SNRPN, SRFBP1, SRSF12, TELO2, TRPT1, TSEN2, TXNL4A, TXNL4B, U2AF1, U2AF1L4, U2AF1L5, WDR77, ZC3H10, ZNF326, ZRSR2]
GO:0009887	animal organ morphogenesis	BiologicalProcess	0.00	[ACVRL1, ADM, ALDH1A2, ALPL, ALX1, ALX4, ANKRD1, ANXA6, AREG, ARHGEF19, ARID5B, ARL4D, ARL6, ATP6V1B1, ATRNL1, AXIN2, BBS5, BCL11B, BCL2, BRAF, CCL2, CDON, CEMP1, CFH, CHAD, CHRNA9, CITED2, CNRIP1, COL11A2, COL12A1, COL13A1, COL1A1, COL5A1, COL6A1, CTHRC1, CTNNA2, CTSH, CYP27B1, DAAM1, DACT1, DGCR6L, DKK1, DLC1, DLEC1, DLL1, DLX1, DLX2, DNAH11, DSCAML1, DSP, EDA, EDAR, EFNA1, EREG, EYA1, FGFR2, FGFR3, FGL1, FLRT2, FOXA1, FOXD1, FOXE1, FOXF2, FOXN4, FOXP2, FRS2, FST, FUZ, FZD5, FZD9, GATA2, GATA3, GATA5, GATA6, GATD3A, GCNT1, GLI1, GLI2, GLI3, GNGT1, GPC4, GPC6, GREB1L, GREM1, GREM2, GRHL3, GSC, HAS2, HES1, HEY1, HHIP, HLX, HNF1A, HOXA1, HOXA5, HOXA6, HOXB13, HOXB2, HOXB3, HOXB5, HOXB6, HOXB7, HOXB8, HOXB9, HOXC8, HOXC9, HOXD10, HOXD13, HOXD9, HSPG2, HYAL1, IFT52, IL7, ILK, INHBA, ITGAX, KDM5B, KLHL3, LAMB4, LEFTY1, LFNG, LHX4, LHX8, LHX9, LRIG1, LRP2, LTBP2, MAGI2, MATN2, MATN3, MEGF9, MEIS1, MESP1, MFSD2A, MKS1, MMP16, MMP25, MSX2, MYO3A, MYO7A, MYOM1, NANOS3, NEK8, NKD1, NKX2-1, NKX2-3, NKX2-5, NKX3-1, NKX3-2, NKX6-1, NOG, NPHP3, NPHP3-ACAD11, NR2E3, NR4A3, NRL, OLFM1, OSR2, OTX1, PAX2, PAX7, PAX8, PAX9, PBX2, PBX4, PDZD7, PEX7, PHLDA2, PITX2, PKP2, PLAG1, POU4F3, PPARA, PPP1R13L, PRDM1, PSMA1, PSMA2, PSMB10, PSMB8, PSMB9, PSMD12, PSMD9, PTCD2, PTCH1, RAB33B, ROGDI, ROR2, RPGRIP1, RS1, RSPO3, RUNX2, RXRA, RYR1, SALL1, SAPCD2, SATB2, SCN5A, SDK2, SEMA6A, SETDB2, SFRP1, SHH, SHROOM2, SIX4, SLC24A4, SLC39A3, SLC40A1, SLC4A10, SLIT1, SLIT2, SLIT3, SLITRK6, SMOX, SMPD3, SNAI1, SNX10, SOX17, SOX4, SOX5, SOX6, SOX9, SPEF2, SPRY1, STC1, STK19, STK40, STOX1, SULF2, TBX20, TBX3, TBX6, TCF7, TGFA, TGFB3, THBS3, THY1, TIAM1, TLE2, TMEFF2, TNC, TP53, TPRN, TRAF6, TSPAN12, TWIST1, USH2A, VANGL2, VDR, WHRN, WNT11, WNT9A, ZNF22, ZNF335]
GO:0010608	posttranscriptional regulation of gene expression	BiologicalProcess	0.00	[A1CF, ACE, AIRE, AXIN2, BANK1, C8orf88, CACNG7, CCL5, CNOT3, DCP2, DDX49, DIO2, DNAJC1, DNAJC3, DPH1, DXO, EEFSEC, EFNA1, EIF1AX, EIF3CL, EIF6, ELOF1, ENC1, EXOSC1, FASTKD3, GRB7, GTPBP2, H4C12, HSPA1A, INPP5E, KBTBD8, KHSRP, MALSU1, MATR3, MTIF3, NANOS1, NANOS3, NCK1, NIBAN1, NSUN3, PCBP4, PCIF1, PDE12, PER2, PIWIL4, POLR2H, POLR2J, POU5F1, PSMA1, PSMA2, PSMB10, PSMB8, PSMB9, PSMD12, PSMD9, PSTK, PUS10, RBM3, RMND1, RNASEL, RXRA, SARNP, SCGB1A1, SOX4, TNRC6A, TNRC6C, TOB1, TP53, TRDMT1, TRUB2, TSFM, UCN, ZC3H10, ZCCHC4]
GO:0016071	mRNA metabolic process	BiologicalProcess	0.00	[A1CF, ALYREF, AURKAIP1, AXIN2, CACNG7, CBLL1, CLASRP, CNOT3, CSTF2, DCP2, DDX47, DXO, ERCC2, EREG, ESRP1, EXOSC1, FASTKD3, FOXE1, GEMIN2, GEMIN7, GEMIN8, GPKOW, GTF2H2, GTF2H4, GTPBP2, HSPA1A, KHDRBS3, KHSRP, LGALS3, LSM5, MRPL23, MTPAP, NANOS1, NANOS3, NOVA1, NT5C3B, PABPN1, PAN2, PAPOLG, PCBP4, PCIF1, PDE12, PELO, PNLDC1, POLR2H, POLR2J, PPP1R8, PPWD1, PQBP1, PRR5L, PSMA1, PSMA2, PSMB10, PSMB8, PSMB9, PSMD12, PSMD9, PTCD2, RAVER1, RBM3, RBM7, RDH14, RNASEL, RNF113A, RNGTT, RPL36A, RPL9, RPRD2, RPS10, RPS26, RPS28, SARNP, SCAF4, SCGB1A1, SLBP, SMG8, SNRPB, SNRPF, SNRPN, SRFBP1, SRSF12, SUPT4H1, TNRC6A, TNRC6C, TOB1, TP53, TRMT61A, TRMT61B, TRUB2, TSEN2, TUT1, TXNL4A, TXNL4B, U2AF1, U2AF1L4, U2AF1L5, WDR77, ZC3H10, ZC3H11A, ZNF326, ZRSR2]
GO:0002253	activation of immune response	BiologicalProcess	0.00	[AKR1C1, BAIAP2, BCL2, BRAF, BTN3A1, BTN3A3, BTNL9, C2, C4A, C4BPA, C5, C7, CD22, CD38, CD47, CFD, CFH, ELMO1, ELOF1, FOXP1, FYB1, GATA3, GPLD1, GRAP2, HLA-DRA, ICAM3, IFI16, LGALS3, MALT1, MATR3, MICB, MUC1, MUC16, MUC20, MUC3A, MUC5AC, MUC5B, NCK1, NFKBIZ, NR4A3, PLA2G6, PLCL2, PQBP1, PRKCH, PRNP, PSMA1, PSMA2, PSMB10, PSMB8, PSMB9, PSMD12, PSMD9, PTPRC, PYCARD, RBM14, RELB, RFTN1, RNF31, SH2B2, SUSD4, TAB3, THY1, TRAF6, UXT, VAV3, VTN, WIPF1, WIPF3]
GO:0043043	peptide biosynthetic process	BiologicalProcess	0.00	[AIRE, ASMT, AURKAIP1, BANK1, BDH2, C8orf88, CARNS1, CCL5, CHAC1, CHAC2, CNOT3, DIO2, DMD, DNAJC1, DNAJC3, DPH1, EEF1E1, EEFSEC, EFNA1, EIF1AX, EIF3CL, EIF6, ELOF1, ENC1, FASTKD3, FTSJ1, GGCT, GGT5, GRB7, GTPBP2, HSPA14, IGHMBP2, INPP5E, KBTBD8, KHSRP, MALSU1, MATR3, MRPL1, MRPL12, MRPL14, MRPL18, MRPL2, MRPL23, MRPL27, MRPL28, MRPL30, MRPL33, MRPL38, MRPL39, MRPL49, MRPL50, MRPL53, MRPL54, MRPL57, MTIF3, N6AMT1, NANOS1, NANOS3, NCK1, NDRG1, NDUFA7, NIBAN1, NSUN3, OPLAH, PARS2, PCIF1, PCSK1, PCSK5, PDF, PER2, PIWIL4, PRNP, PSTK, RBM3, RMND1, RPL36A, RPL39L, RPL9, RPS10, RPS26, RPS28, RXRA, SARNP, SOX4, TMA7, TNRC6A, TNRC6C, TOB1, TRUB2, TSFM, UCN, WARS2, ZCCHC4]
GO:0006412	translation	BiologicalProcess	0.00	[AIRE, ASMT, AURKAIP1, BANK1, C8orf88, CCL5, CNOT3, DIO2, DNAJC1, DNAJC3, DPH1, EEF1E1, EEFSEC, EFNA1, EIF1AX, EIF3CL, EIF6, ELOF1, ENC1, FASTKD3, FTSJ1, GGT5, GRB7, GTPBP2, HSPA14, IGHMBP2, INPP5E, KBTBD8, KHSRP, MALSU1, MATR3, MRPL1, MRPL12, MRPL14, MRPL18, MRPL2, MRPL23, MRPL27, MRPL28, MRPL30, MRPL33, MRPL38, MRPL39, MRPL49, MRPL50, MRPL53, MRPL54, MRPL57, MTIF3, N6AMT1, NANOS1, NANOS3, NCK1, NDRG1, NDUFA7, NIBAN1, NSUN3, PARS2, PCIF1, PDF, PER2, PIWIL4, PSTK, RBM3, RMND1, RPL36A, RPL39L, RPL9, RPS10, RPS26, RPS28, RXRA, SARNP, SOX4, TMA7, TNRC6A, TNRC6C, TOB1, TRUB2, TSFM, UCN, WARS2, ZCCHC4]
GO:0048666	neuron development	BiologicalProcess	0.01	[ABITRAM, ACAP3, ADCY1, ADGRB1, ADM, AGER, ANK3, ANKRD1, APBB1, APOE, AREG, ARHGAP33, ARHGAP44, ARX, ATF5, ATP7A, AUTS2, BAIAP2, BCL11A, BCL11B, BCL2, BLOC1S3, BLOC1S4, BLOC1S5, BRAF, BTBD8, C1D, C1QL1, C21orf91, CACNG7, CAMSAP3, CCK, CD38, CDH4, CHRFAM7A, CHRNA7, CNTN1, CRMP1, CTHRC1, CTNNA2, CTNND2, CXCR4, CXXC1, DCDC2, DCLK1, DGKG, DISC1, DKK1, DMD, DNAAF3, DNM2, DNM3, DOCK7, DOK5, DOK6, DPYSL3, DRD1, DSCAML1, EFNA1, EFNA3, EFNA4, EFNB1, ENC1, EPHA3, EPHA4, EPHA5, EPHA6, EPHA7, EPHB3, EXT1, FBXL15, FBXW8, FGFR2, FKBPL, FLRT2, FLRT3, FN1, FOXD1, FRS2, FRY, FSTL4, FZD9, GAB2, GAS7, GATA2, GATA3, GATD3A, GBA2, GFRA1, GJA1, GLI2, GLI3, GNGT1, GORASP1, GPC2, GRB7, GRIN1, GRIP1, HES1, HOXD10, HOXD9, HTRA2, ILK, ITGA1, ITGA4, ITPKA, KIF1A, KIF5A, KIRREL3, KLF4, KLF7, L1CAM, LHX4, LHX6, LHX8, LHX9, LRIG2, LRP1, LRP2, LYPLA2, MAGI2, MANF, MAP2, MAPT, MATN2, MFSD2A, MICALL1, MKS1, MNX1, MOB2, MYO7A, MYPN, NANOS1, NCDN, NCK1, NDNF, NEFL, NGEF, NKX2-1, NKX6-1, NLGN1, NME2, NOG, NPTX1, NR2E3, NR2F6, NR4A2, NR4A3, NRCAM, NRK, NRL, NRN1, NRXN3, NTRK3, NYAP1, OLFM1, OPHN1, PAX2, PBX2, PBX4, PCDHA4, PDZD7, PHACTR1, PLK2, PLXNA3, PLXNB3, POU4F3, PPP1R9A, PQBP1, PRDM1, PRKCZ, PRKD1, PSPN, PTCH1, PTK6, PTPN9, RAB3A, RAP1GAP, RAP1GAP2, RAP2A, RET, RIMS2, ROR2, RPGRIP1, RTN4RL1, RTN4RL2, SCYL3, SDC2, SEMA3B, SEMA3D, SEMA3E, SEMA4G, SEMA6A, SERPINI1, SFRP1, SHC3, SHH, SIPA1L1, SLC1A3, SLC4A10, SLCO1B1, SLIT1, SLIT2, SLIT3, SLITRK3, SLITRK4, SLITRK6, SMOX, SNAP25, SNAPIN, SPAST, SPOCK1, SPRY3, SPTB, SPTBN2, SPTBN4, SRCIN1, SS18L2, STK19, STMN3, STMN4, STXBP1, SYT17, SYT3, TBC1D23, TBC1D24, TBX6, TENM1, THY1, TIAM1, TIAM2, TIMP1, TMC1, TMEFF2, TNC, TNFRSF12A, TNIK, TPRN, TRAK2, TRAPPC4, TRIM46, TRPV2, TSPO, UCN, UNC5D, VAMP7, VANGL2, VASH2, WHRN, WNT3, XK, ZFYVE27, ZMYND8, ZNF335, ZNF804A]
GO:0031175	neuron projection development	BiologicalProcess	0.01	[ABITRAM, ACAP3, ADCY1, ADGRB1, ADM, AGER, ANK3, ANKRD1, APBB1, APOE, AREG, ARHGAP33, ARHGAP44, ARX, ATP7A, AUTS2, BAIAP2, BCL11A, BCL11B, BCL2, BLOC1S3, BLOC1S4, BLOC1S5, BRAF, BTBD8, C1D, C21orf91, CACNG7, CAMSAP3, CCK, CD38, CDH4, CHRFAM7A, CHRNA7, CNTN1, CRMP1, CTHRC1, CTNNA2, CTNND2, CXCR4, CXXC1, DCDC2, DCLK1, DISC1, DKK1, DMD, DNAAF3, DNM2, DNM3, DOCK7, DOK5, DOK6, DPYSL3, DSCAML1, EFNA1, EFNA3, EFNA4, EFNB1, ENC1, EPHA3, EPHA4, EPHA5, EPHA6, EPHA7, EPHB3, EXT1, FBXL15, FBXW8, FGFR2, FKBPL, FLRT2, FLRT3, FN1, FOXD1, FRS2, FRY, FSTL4, FZD9, GAB2, GAS7, GATA3, GATD3A, GFRA1, GJA1, GLI2, GLI3, GORASP1, GPC2, GRB7, GRIN1, GRIP1, HES1, ILK, ITGA1, ITGA4, ITPKA, KIF1A, KIF5A, KIRREL3, KLF4, KLF7, L1CAM, LHX4, LHX9, LRIG2, LRP1, LRP2, LYPLA2, MAGI2, MANF, MAP2, MAPT, MATN2, MFSD2A, MICALL1, MKS1, MNX1, MOB2, MYO7A, MYPN, NCDN, NCK1, NDNF, NEFL, NGEF, NKX2-1, NKX6-1, NLGN1, NME2, NOG, NPTX1, NR4A2, NR4A3, NRCAM, NRK, NRN1, NRXN3, NTRK3, NYAP1, OLFM1, OPHN1, PAX2, PCDHA4, PDZD7, PHACTR1, PLK2, PLXNA3, PLXNB3, POU4F3, PPP1R9A, PQBP1, PRKCZ, PRKD1, PSPN, PTCH1, PTK6, PTPN9, RAB3A, RAP1GAP, RAP1GAP2, RAP2A, RET, RIMS2, ROR2, RTN4RL1, RTN4RL2, SDC2, SEMA3B, SEMA3D, SEMA3E, SEMA4G, SEMA6A, SERPINI1, SFRP1, SHC3, SHH, SIPA1L1, SLCO1B1, SLIT1, SLIT2, SLIT3, SLITRK3, SLITRK4, SLITRK6, SMOX, SNAP25, SNAPIN, SPAST, SPOCK1, SPRY3, SPTB, SPTBN2, SPTBN4, SRCIN1, SS18L2, STMN3, STMN4, STXBP1, SYT17, SYT3, TBC1D23, TBC1D24, TBX6, THY1, TIAM1, TIAM2, TIMP1, TMEFF2, TNC, TNFRSF12A, TNIK, TPRN, TRAK2, TRAPPC4, TRIM46, TRPV2, TSPO, UCN, UNC5D, VAMP7, VANGL2, VASH2, WHRN, WNT3, XK, ZFYVE27, ZMYND8, ZNF335, ZNF804A]
GO:0061564	axon development	BiologicalProcess	0.00	[ADCY1, ADGRB1, ANK3, APBB1, APOE, ARX, AUTS2, BAIAP2, BCL11B, BCL2, BRAF, C1D, CCK, CDH4, CRMP1, CTNNA2, CXCR4, CXXC1, DCLK1, DISC1, DNAAF3, DNM2, DOCK7, DOK5, DOK6, DSCAML1, EFNA1, EFNA3, EFNA4, EFNB1, EPHA3, EPHA4, EPHA5, EPHA6, EPHA7, EPHB3, EXT1, FGFR2, FLRT2, FLRT3, FN1, FOXD1, FRS2, FSTL4, FZD9, GAB2, GATA3, GFRA1, GLI2, GLI3, GRB7, GRIN1, ILK, ITGA4, KIF5A, KLF4, KLF7, L1CAM, LHX4, LHX9, LRIG2, LRP1, LYPLA2, MAP2, MAPT, MATN2, MYPN, NEFL, NKX2-1, NKX6-1, NOG, NPTX1, NR4A2, NR4A3, NRCAM, NRXN3, NTRK3, OLFM1, OPHN1, PAX2, PCDHA4, PLXNA3, PLXNB3, POU4F3, PSPN, PTCH1, RAB3A, RAP1GAP, RET, RTN4RL1, RTN4RL2, SEMA3B, SEMA3D, SEMA3E, SEMA4G, SEMA6A, SHC3, SHH, SIPA1L1, SLIT1, SLIT2, SLIT3, SLITRK3, SLITRK4, SLITRK6, SMOX, SPAST, SPRY3, SPTB, SPTBN2, SPTBN4, STXBP1, THY1, TIAM1, TIAM2, TMEFF2, TNC, TNFRSF12A, TRAK2, TRIM46, TRPV2, TSPO, UNC5D, VANGL2, VASH2, WNT3, XK, ZFYVE27]
GO:0007409	axonogenesis	BiologicalProcess	0.00	[ADCY1, ADGRB1, ANK3, APBB1, APOE, ARX, AUTS2, BAIAP2, BCL11B, BCL2, BRAF, C1D, CCK, CDH4, CRMP1, CTNNA2, CXCR4, CXXC1, DCLK1, DISC1, DNAAF3, DNM2, DOCK7, DOK5, DOK6, DSCAML1, EFNA1, EFNA3, EFNA4, EFNB1, EPHA3, EPHA4, EPHA5, EPHA6, EPHA7, EPHB3, EXT1, FGFR2, FLRT2, FLRT3, FN1, FOXD1, FRS2, FSTL4, FZD9, GAB2, GATA3, GFRA1, GLI2, GLI3, GRB7, GRIN1, ILK, ITGA4, KIF5A, KLF7, L1CAM, LHX4, LHX9, LRP1, LYPLA2, MAP2, MAPT, MATN2, MYPN, NEFL, NKX2-1, NKX6-1, NOG, NPTX1, NR4A2, NR4A3, NRCAM, NRXN3, NTRK3, OLFM1, OPHN1, PAX2, PLXNA3, PLXNB3, POU4F3, PSPN, PTCH1, RAB3A, RAP1GAP, RET, SEMA3B, SEMA3D, SEMA3E, SEMA4G, SEMA6A, SHC3, SHH, SIPA1L1, SLIT1, SLIT2, SLIT3, SLITRK3, SLITRK4, SLITRK6, SMOX, SPAST, SPTB, SPTBN2, SPTBN4, STXBP1, THY1, TIAM1, TIAM2, TMEFF2, TNFRSF12A, TRAK2, TRIM46, TRPV2, UNC5D, VANGL2, WNT3, XK, ZFYVE27]
GO:2000146	negative regulation of cell motility	BiologicalProcess	0.00	[ABCC8, ABHD6, ACVRL1, ADGRB1, ADORA1, ADTRP, APOE, APOH, ARHGDIB, ATP2B4, BCL2, BMERB1, BRAF, C1D, C5, CCDC125, CCL2, CITED2, CLDN3, CSNK2B, CYP1B1, DDT, DDTL, DLC1, DLEC1, DPYSL3, ENC1, FLCN, FRMD5, FUZ, GADD45A, GATA3, GJA1, GNRH1, GREM1, HMOX1, IGFBP3, IL27RA, ILK, KLF4, KRT16, LDLRAD4, LRP1, MACIR, MAGI2, MAP2K5, MIA3, MIF, MITF, MMP28, NAV3, NKX2-1, NOG, PADI2, PIP5KL1, PLCB1, PLXNB3, RAP2A, RECK, SFRP1, SHH, SLIT2, SRFBP1, SRGAP2B, STC1, THY1, TIMP1, TMEFF2, TP53INP1, UXT, VASH1, WNT11, ZMYND8]
GO:0030336	negative regulation of cell migration	BiologicalProcess	0.00	[ABCC8, ABHD6, ACVRL1, ADGRB1, ADORA1, ADTRP, APOE, APOH, ARHGDIB, ATP2B4, BCL2, BMERB1, BRAF, C1D, C5, CCL2, CITED2, CLDN3, CSNK2B, CYP1B1, DDT, DDTL, DLC1, DLEC1, DPYSL3, ENC1, FLCN, FUZ, GADD45A, GJA1, GNRH1, GREM1, HMOX1, IGFBP3, IL27RA, ILK, KLF4, KRT16, LDLRAD4, LRP1, MACIR, MAGI2, MAP2K5, MIA3, MIF, MITF, MMP28, NAV3, NKX2-1, NOG, PADI2, PIP5KL1, PLCB1, PLXNB3, RAP2A, RECK, SFRP1, SHH, SLIT2, SRFBP1, SRGAP2B, STC1, THY1, TIMP1, TMEFF2, TP53INP1, UXT, VASH1, WNT11, ZMYND8]
GO:0022008	neurogenesis	BiologicalProcess	0.00	[ABCA2, ABCC8, ABITRAM, ACAP3, ADAM22, ADCY1, ADGRB1, ADM, AGER, ALDH1A2, ANK3, ANKRD1, APBB1, APOE, AREG, ARHGAP33, ARHGAP44, ARX, ATF5, ATP2B4, ATP7A, AUTS2, BAIAP2, BCHE, BCL11A, BCL11B, BCL2, BCL6, BEND6, BHLHE41, BLOC1S3, BLOC1S4, BLOC1S5, BMERB1, BRAF, BRINP3, BTBD8, C1D, C1QL1, C21orf91, CACNG7, CAMSAP3, CASZ1, CCK, CCL2, CD38, CDH4, CDK5R2, CDK5RAP3, CDON, CEP120, CHAC1, CHD5, CHRFAM7A, CHRNA7, CNTN1, CRMP1, CTHRC1, CTNNA2, CTNND2, CXCR4, CXXC1, DCDC2, DCLK1, DDIT4, DGKG, DISC1, DKK1, DLL1, DLX1, DLX2, DMD, DMRTA2, DNAAF3, DNAAF4, DNER, DNM2, DNM3, DOCK7, DOK5, DOK6, DPYSL3, DRD1, DSCAML1, EFNA1, EFNA3, EFNA4, EFNB1, ENC1, EPHA3, EPHA4, EPHA5, EPHA6, EPHA7, EPHB3, EPOP, ERBB3, ERCC2, ESRP1, EXT1, EYA1, FA2H, FAIM, FBXL15, FBXW8, FGFR2, FKBPL, FLRT2, FLRT3, FN1, FOXA1, FOXD1, FOXN4, FRS2, FRY, FSTL4, FUOM, FZD5, FZD9, GAB2, GAS7, GATA2, GATA3, GATD3A, GBA2, GFRA1, GJA1, GJC2, GLI2, GLI3, GNGT1, GNRH1, GORASP1, GPC2, GPR183, GRB7, GRIN1, GRIP1, HAP1, HDAC9, HES1, HES2, HEY1, HHIP, HOXB3, HOXC8, HOXD1, HOXD10, HOXD9, HTRA2, IGSF10, ILK, INHBA, ITGA1, ITGA4, ITPKA, KDM4C, KIF1A, KIF5A, KIRREL3, KLF4, KLF7, L1CAM, LEPR, LHX4, LHX6, LHX8, LHX9, LRIG2, LRP1, LRP2, LYPLA2, MAGI2, MANF, MAP2, MAPT, MATN2, MDGA1, MEIS1, MESP1, MFSD2A, MICALL1, MKS1, MME, MNX1, MOB2, MYCL, MYO7A, MYPN, NANOS1, NAP1L2, NAV3, NCDN, NCK1, NDNF, NDRG1, NEFL, NGEF, NHLRC1, NKD1, NKX2-1, NKX2-5, NKX6-1, NLGN1, NME2, NOG, NPTX1, NR1D1, NR2E3, NR2F6, NR4A2, NR4A3, NRCAM, NRK, NRL, NRN1, NRXN3, NTRK3, NYAP1, OLFM1, OPHN1, P2RX4, P2RY1, PAX2, PAX7, PBX2, PBX4, PCDHA4, PCSK9, PDZD7, PER2, PEX7, PHACTR1, PITX2, PLAG1, PLK2, PLXNA3, PLXNB3, POU4F3, PPP1R9A, PQBP1, PRDM1, PRDM13, PRDM5, PRDM8, PRKCH, PRKCZ, PRKD1, PSPN, PTCH1, PTK6, PTPN9, RAB3A, RAP1GAP, RAP1GAP2, RAP2A, RASSF10, RET, RIMS2, RITA1, ROGDI, ROR2, RPGRIP1, RTN4RL1, RTN4RL2, RUNX2, SALL1, SATB2, SCYL3, SDC2, SDK2, SEMA3B, SEMA3D, SEMA3E, SEMA4G, SEMA6A, SERPINE2, SERPINI1, SFRP1, SH3GL3, SH3TC2, SHC3, SHH, SIPA1L1, SIX4, SLC1A3, SLC45A3, SLC4A10, SLCO1B1, SLIT1, SLIT2, SLIT3, SLITRK3, SLITRK4, SLITRK6, SMOX, SNAP25, SNAPIN, SNPH, SOCS2, SOX2, SOX4, SOX5, SOX6, SOX9, SPAST, SPOCK1, SPRY3, SPTB, SPTBN2, SPTBN4, SRCIN1, SS18L2, STAR, STK19, STMN3, STMN4, STXBP1, SYT17, SYT3, TBC1D23, TBC1D24, TBX20, TBX6, TEAD3, TENM1, THY1, TIAM1, TIAM2, TIMP1, TMC1, TMEFF2, TMEM98, TNC, TNFRSF12A, TNIK, TP53, TPPP, TPRN, TRAK2, TRAPPC4, TRIM46, TRPV2, TSPO, TTLL1, TWIST1, UCN, UNC5D, USH2A, UXT, VAMP7, VANGL2, VASH2, VTN, WASF3, WHRN, WNT11, WNT3, WNT9A, XK, ZC4H2, ZFYVE27, ZMYND8, ZNF335, ZNF804A]
GO:0048699	generation of neurons	BiologicalProcess	0.00	[ABCC8, ABITRAM, ACAP3, ADCY1, ADGRB1, ADM, AGER, ALDH1A2, ANK3, ANKRD1, APBB1, APOE, AREG, ARHGAP33, ARHGAP44, ARX, ATF5, ATP2B4, ATP7A, AUTS2, BAIAP2, BCHE, BCL11A, BCL11B, BCL2, BCL6, BEND6, BHLHE41, BLOC1S3, BLOC1S4, BLOC1S5, BMERB1, BRAF, BRINP3, BTBD8, C1D, C1QL1, C21orf91, CACNG7, CAMSAP3, CASZ1, CCK, CD38, CDH4, CDK5R2, CDK5RAP3, CDON, CHD5, CHRFAM7A, CHRNA7, CNTN1, CRMP1, CTHRC1, CTNNA2, CTNND2, CXCR4, CXXC1, DCDC2, DCLK1, DDIT4, DGKG, DISC1, DKK1, DLL1, DLX1, DLX2, DMD, DMRTA2, DNAAF3, DNAAF4, DNER, DNM2, DNM3, DOCK7, DOK5, DOK6, DPYSL3, DRD1, DSCAML1, EFNA1, EFNA3, EFNA4, EFNB1, ENC1, EPHA3, EPHA4, EPHA5, EPHA6, EPHA7, EPHB3, EPOP, ERCC2, ESRP1, EXT1, EYA1, FAIM, FBXL15, FBXW8, FGFR2, FKBPL, FLRT2, FLRT3, FN1, FOXA1, FOXD1, FOXN4, FRS2, FRY, FSTL4, FUOM, FZD5, FZD9, GAB2, GAS7, GATA2, GATA3, GATD3A, GBA2, GFRA1, GJA1, GJC2, GLI2, GLI3, GNGT1, GNRH1, GORASP1, GPC2, GPR183, GRB7, GRIN1, GRIP1, HAP1, HDAC9, HES1, HES2, HEY1, HHIP, HOXB3, HOXC8, HOXD1, HOXD10, HOXD9, HTRA2, IGSF10, ILK, INHBA, ITGA1, ITGA4, ITPKA, KDM4C, KIF1A, KIF5A, KIRREL3, KLF4, KLF7, L1CAM, LHX4, LHX6, LHX8, LHX9, LRIG2, LRP1, LRP2, LYPLA2, MAGI2, MANF, MAP2, MAPT, MATN2, MDGA1, MEIS1, MFSD2A, MICALL1, MKS1, MME, MNX1, MOB2, MYCL, MYO7A, MYPN, NANOS1, NAP1L2, NCDN, NCK1, NDNF, NEFL, NGEF, NKD1, NKX2-1, NKX2-5, NKX6-1, NLGN1, NME2, NOG, NPTX1, NR1D1, NR2E3, NR2F6, NR4A2, NR4A3, NRCAM, NRK, NRL, NRN1, NRXN3, NTRK3, NYAP1, OLFM1, OPHN1, P2RX4, PAX2, PAX7, PBX2, PBX4, PCDHA4, PCSK9, PDZD7, PER2, PEX7, PHACTR1, PITX2, PLAG1, PLK2, PLXNA3, PLXNB3, POU4F3, PPP1R9A, PQBP1, PRDM1, PRKCH, PRKCZ, PRKD1, PSPN, PTCH1, PTK6, PTPN9, RAB3A, RAP1GAP, RAP1GAP2, RAP2A, RASSF10, RET, RIMS2, ROR2, RPGRIP1, RTN4RL1, RTN4RL2, RUNX2, SALL1, SATB2, SCYL3, SDC2, SDK2, SEMA3B, SEMA3D, SEMA3E, SEMA4G, SEMA6A, SERPINE2, SERPINI1, SFRP1, SH3GL3, SHC3, SHH, SIPA1L1, SIX4, SLC1A3, SLC45A3, SLC4A10, SLCO1B1, SLIT1, SLIT2, SLIT3, SLITRK3, SLITRK4, SLITRK6, SMOX, SNAP25, SNAPIN, SNPH, SOCS2, SOX2, SOX4, SOX5, SOX9, SPAST, SPOCK1, SPRY3, SPTB, SPTBN2, SPTBN4, SRCIN1, SS18L2, STAR, STK19, STMN3, STMN4, STXBP1, SYT17, SYT3, TBC1D23, TBC1D24, TBX20, TBX6, TEAD3, TENM1, THY1, TIAM1, TIAM2, TIMP1, TMC1, TMEFF2, TMEM98, TNC, TNFRSF12A, TNIK, TP53, TPRN, TRAK2, TRAPPC4, TRIM46, TRPV2, TSPO, TTLL1, TWIST1, UCN, UNC5D, USH2A, UXT, VAMP7, VANGL2, VASH2, WHRN, WNT11, WNT3, WNT9A, XK, ZC4H2, ZFYVE27, ZMYND8, ZNF335, ZNF804A]

GO:0030182	neuron differentiation	BiologicalProcess	0.00	[ABITRAM, ACAP3, ADCY1, ADGRB1, ADM, AGER, ALDH1A2, ANK3, ANKRD1, APBB1, APOE, AREG, ARHGAP33, ARHGAP44, ARX, ATF5, ATP2B4, ATP7A, AUTS2, BAIAP2, BCL11A, BCL11B, BCL2, BCL6, BEND6, BLOC1S3, BLOC1S4, BLOC1S5, BRAF, BRINP3, BTBD8, C1D, C1QL1, C21orf91, CACNG7, CAMSAP3, CASZ1, CCK, CD38, CDH4, CDK5RAP3, CDON, CHD5, CHRFAM7A, CHRNA7, CNTN1, CRMP1, CTHRC1, CTNNA2, CTNND2, CXCR4, CXXC1, DCDC2, DCLK1, DDIT4, DGKG, DISC1, DKK1, DLL1, DLX1, DLX2, DMD, DMRTA2, DNAAF3, DNM2, DNM3, DOCK7, DOK5, DOK6, DPYSL3, DRD1, DSCAML1, EFNA1, EFNA3, EFNA4, EFNB1, ENC1, EPHA3, EPHA4, EPHA5, EPHA6, EPHA7, EPHB3, EPOP, ERCC2, ESRP1, EXT1, EYA1, FBXL15, FBXW8, FGFR2, FKBPL, FLRT2, FLRT3, FN1, FOXA1, FOXD1, FOXN4, FRS2, FRY, FSTL4, FUOM, FZD5, FZD9, GAB2, GAS7, GATA2, GATA3, GATD3A, GBA2, GFRA1, GJA1, GLI2, GLI3, GNGT1, GORASP1, GPC2, GRB7, GRIN1, GRIP1, HDAC9, HES1, HEY1, HOXC8, HOXD1, HOXD10, HOXD9, HTRA2, ILK, INHBA, ITGA1, ITGA4, ITPKA, KDM4C, KIF1A, KIF5A, KIRREL3, KLF4, KLF7, L1CAM, LHX4, LHX6, LHX8, LHX9, LRIG2, LRP1, LRP2, LYPLA2, MAGI2, MANF, MAP2, MAPT, MATN2, MDGA1, MEIS1, MFSD2A, MICALL1, MKS1, MNX1, MOB2, MYCL, MYO7A, MYPN, NANOS1, NAP1L2, NCDN, NCK1, NDNF, NEFL, NGEF, NKD1, NKX2-1, NKX2-5, NKX6-1, NLGN1, NME2, NOG, NPTX1, NR2E3, NR2F6, NR4A2, NR4A3, NRCAM, NRK, NRL, NRN1, NRXN3, NTRK3, NYAP1, OLFM1, OPHN1, PAX2, PAX7, PBX2, PBX4, PCDHA4, PCSK9, PDZD7, PHACTR1, PITX2, PLK2, PLXNA3, PLXNB3, POU4F3, PPP1R9A, PQBP1, PRDM1, PRKCZ, PRKD1, PSPN, PTCH1, PTK6, PTPN9, RAB3A, RAP1GAP, RAP1GAP2, RAP2A, RET, RIMS2, ROR2, RPGRIP1, RTN4RL1, RTN4RL2, RUNX2, SALL1, SATB2, SCYL3, SDC2, SDK2, SEMA3B, SEMA3D, SEMA3E, SEMA4G, SEMA6A, SERPINI1, SFRP1, SH3GL3, SHC3, SHH, SIPA1L1, SLC1A3, SLC4A10, SLCO1B1, SLIT1, SLIT2, SLIT3, SLITRK3, SLITRK4, SLITRK6, SMOX, SNAP25, SNAPIN, SNPH, SOCS2, SOX2, SOX4, SOX9, SPAST, SPOCK1, SPRY3, SPTB, SPTBN2, SPTBN4, SRCIN1, SS18L2, STK19, STMN3, STMN4, STXBP1, SYT17, SYT3, TBC1D23, TBC1D24, TBX20, TBX6, TENM1, THY1, TIAM1, TIAM2, TIMP1, TMC1, TMEFF2, TNC, TNFRSF12A, TNIK, TPRN, TRAK2, TRAPPC4, TRIM46, TRPV2, TSPO, TTLL1, UCN, UNC5D, USH2A, VAMP7, VANGL2, VASH2, WHRN, WNT11, WNT3, WNT9A, XK, ZC4H2, ZFYVE27, ZMYND8, ZNF335, ZNF804A]
GO:0002757	immune response-activating signal transduction	BiologicalProcess	0.00	[BAIAP2, BCL2, BRAF, BTN3A1, BTN3A3, BTNL9, CD22, CD38, CD47, ELMO1, ELOF1, FOXP1, FYB1, GATA3, GPLD1, GRAP2, HLA-DRA, ICAM3, LGALS3, MALT1, MICB, MUC1, MUC16, MUC20, MUC3A, MUC5AC, MUC5B, NCK1, NFKBIZ, NR4A3, PLA2G6, PLCL2, PRKCH, PRNP, PSMA1, PSMA2, PSMB10, PSMB8, PSMB9, PSMD12, PSMD9, PTPRC, RELB, RFTN1, RNF31, SH2B2, TAB3, THY1, TRAF6, UXT, VAV3, WIPF1, WIPF3]
GO:0002768	immune response-regulating cell surface receptor signaling pathway	BiologicalProcess	0.00	[BAIAP2, BCL2, BRAF, BTN3A1, BTN3A3, BTNL9, CD22, CD24, CD38, CD40, CD47, ELMO1, ELOF1, FOS, FOXP1, FYB1, GAB2, GATA3, GPLD1, GRAP2, HLA-DRA, ICAM3, LGALS3, MALT1, MAP2K4, MAP3K1, MAPK10, MICB, MUC1, MUC16, MUC20, MUC3A, MUC5AC, MUC5B, NCK1, NFATC1, NFKBIZ, NR4A3, OSCAR, PLA2G6, PLCL2, PPP3R1, PRKCH, PRNP, PSMA1, PSMA2, PSMB10, PSMB8, PSMB9, PSMD12, PSMD9, PTPRC, RELB, RFTN1, RNF31, SH2B2, TAB3, THY1, TRAF6, UXT, VAV3, WIPF1, WIPF3]
GO:0002429	immune response-activating cell surface receptor signaling pathway	BiologicalProcess	0.00	[BAIAP2, BCL2, BRAF, BTN3A1, BTN3A3, BTNL9, CD22, CD38, CD47, ELMO1, ELOF1, FOXP1, FYB1, GATA3, GPLD1, GRAP2, HLA-DRA, ICAM3, LGALS3, MALT1, MICB, MUC1, MUC16, MUC20, MUC3A, MUC5AC, MUC5B, NCK1, NFKBIZ, NR4A3, PLA2G6, PLCL2, PRKCH, PRNP, PSMA1, PSMA2, PSMB10, PSMB8, PSMB9, PSMD12, PSMD9, PTPRC, RELB, RFTN1, RNF31, SH2B2, TAB3, THY1, TRAF6, UXT, VAV3, WIPF1, WIPF3]
GO:0048858	cell projection morphogenesis	BiologicalProcess	0.00	[ABITRAM, ADCY1, ADGRB1, ANK3, APBB1, APOE, ARHGAP33, ARHGAP44, ARX, ATP7A, AUTS2, BAIAP2, BCL11A, BCL11B, BCL2, BRAF, C1D, CACNG7, CCK, CDH4, CHRFAM7A, CHRNA7, CRMP1, CTNNA2, CTNND2, CXCR4, CXXC1, DCDC2, DCLK1, DISC1, DMD, DNAAF3, DNM2, DNM3, DOCK7, DOK5, DOK6, DSCAML1, EFNA1, EFNA3, EFNA4, EFNB1, EPHA3, EPHA4, EPHA5, EPHA6, EPHA7, EPHB3, EXT1, FBXW8, FGFR2, FLRT2, FLRT3, FN1, FOXD1, FRS2, FSTL4, FZD9, GAB2, GAS7, GATA3, GFRA1, GJA1, GLI2, GLI3, GORASP1, GRB7, GRIN1, GRIP1, ILK, ITGA1, ITGA4, ITPKA, KIF1A, KIF5A, KIRREL3, KLF7, L1CAM, LHX4, LHX9, LRP1, LRP2, LYPLA2, MAP2, MAPT, MATN2, MFSD2A, MNX1, MYPN, NEFL, NES, NGEF, NKX2-1, NKX6-1, NLGN1, NOG, NPTX1, NR4A2, NR4A3, NRCAM, NRK, NRN1, NRXN3, NTRK3, NYAP1, OLFM1, OPHN1, PAX2, PHACTR1, PLXNA3, PLXNB3, POU4F3, PPP1R9A, PQBP1, PRKCZ, PSPN, PTCH1, RAB3A, RAP1GAP, RAP2A, RET, RIMS2, SDC2, SEMA3B, SEMA3D, SEMA3E, SEMA4G, SEMA6A, SHC3, SHH, SIPA1L1, SLIT1, SLIT2, SLIT3, SLITRK3, SLITRK4, SLITRK6, SMOX, SPAST, SPTB, SPTBN2, SPTBN4, SRCIN1, SS18L2, STXBP1, SYT17, SYT3, THY1, TIAM1, TIAM2, TMEFF2, TNFRSF12A, TNIK, TRAK2, TRIM46, TRPV2, UNC5D, VAMP7, VANGL2, WNT3, XK, ZFYVE27, ZNF335]
GO:0120039	plasma membrane bounded cell projection morphogenesis	BiologicalProcess	0.00	[ABITRAM, ADCY1, ADGRB1, ANK3, APBB1, APOE, ARHGAP33, ARHGAP44, ARX, ATP7A, AUTS2, BAIAP2, BCL11A, BCL11B, BCL2, BRAF, C1D, CACNG7, CCK, CDH4, CHRFAM7A, CHRNA7, CRMP1, CTNNA2, CTNND2, CXCR4, CXXC1, DCDC2, DCLK1, DISC1, DMD, DNAAF3, DNM2, DNM3, DOCK7, DOK5, DOK6, DSCAML1, EFNA1, EFNA3, EFNA4, EFNB1, EPHA3, EPHA4, EPHA5, EPHA6, EPHA7, EPHB3, EXT1, FBXW8, FGFR2, FLRT2, FLRT3, FN1, FOXD1, FRS2, FSTL4, FZD9, GAB2, GAS7, GATA3, GFRA1, GJA1, GLI2, GLI3, GORASP1, GRB7, GRIN1, GRIP1, ILK, ITGA1, ITGA4, ITPKA, KIF1A, KIF5A, KIRREL3, KLF7, L1CAM, LHX4, LHX9, LRP1, LRP2, LYPLA2, MAP2, MAPT, MATN2, MFSD2A, MNX1, MYPN, NEFL, NGEF, NKX2-1, NKX6-1, NLGN1, NOG, NPTX1, NR4A2, NR4A3, NRCAM, NRK, NRN1, NRXN3, NTRK3, NYAP1, OLFM1, OPHN1, PAX2, PHACTR1, PLXNA3, PLXNB3, POU4F3, PPP1R9A, PQBP1, PRKCZ, PSPN, PTCH1, RAB3A, RAP1GAP, RAP2A, RET, RIMS2, SDC2, SEMA3B, SEMA3D, SEMA3E, SEMA4G, SEMA6A, SHC3, SHH, SIPA1L1, SLIT1, SLIT2, SLIT3, SLITRK3, SLITRK4, SLITRK6, SMOX, SPAST, SPTB, SPTBN2, SPTBN4, SRCIN1, SS18L2, STXBP1, SYT17, SYT3, THY1, TIAM1, TIAM2, TMEFF2, TNFRSF12A, TNIK, TRAK2, TRIM46, TRPV2, UNC5D, VAMP7, VANGL2, WNT3, XK, ZFYVE27, ZNF335]
GO:0048667	cell morphogenesis involved in neuron differentiation	BiologicalProcess	0.00	[ABITRAM, ADCY1, ADGRB1, ANK3, APBB1, APOE, ARHGAP33, ARHGAP44, ARX, AUTS2, BAIAP2, BCL11B, BCL2, BRAF, C1D, CCK, CDH4, CHRFAM7A, CHRNA7, CRMP1, CTNNA2, CTNND2, CXCR4, CXXC1, DCDC2, DCLK1, DISC1, DNAAF3, DNM2, DNM3, DOCK7, DOK5, DOK6, DSCAML1, EFNA1, EFNA3, EFNA4, EFNB1, EPHA3, EPHA4, EPHA5, EPHA6, EPHA7, EPHB3, EXT1, FBXW8, FGFR2, FLRT2, FLRT3, FN1, FOXD1, FRS2, FSTL4, FZD9, GAB2, GATA3, GATD3A, GFRA1, GLI2, GLI3, GORASP1, GRB7, GRIN1, HES1, ILK, ITGA4, ITPKA, KIF1A, KIF5A, KLF7, L1CAM, LHX4, LHX9, LRP1, LYPLA2, MAP2, MAPT, MATN2, MFSD2A, MYO7A, MYPN, NEFL, NGEF, NKX2-1, NKX6-1, NLGN1, NOG, NPTX1, NR4A2, NR4A3, NRCAM, NRXN3, NTRK3, OLFM1, OPHN1, PAX2, PDZD7, PHACTR1, PLXNA3, PLXNB3, POU4F3, PPP1R9A, PQBP1, PSPN, PTCH1, RAB3A, RAP1GAP, RAP2A, RET, SDC2, SEMA3B, SEMA3D, SEMA3E, SEMA4G, SEMA6A, SHC3, SHH, SIPA1L1, SLC1A3, SLIT1, SLIT2, SLIT3, SLITRK3, SLITRK4, SLITRK6, SMOX, SPAST, SPTB, SPTBN2, SPTBN4, SRCIN1, SS18L2, STXBP1, THY1, TIAM1, TIAM2, TMEFF2, TNFRSF12A, TNIK, TPRN, TRAK2, TRIM46, TRPV2, UNC5D, VAMP7, VANGL2, WHRN, WNT3, XK, ZFYVE27]
GO:0048812	neuron projection morphogenesis	BiologicalProcess	0.00	[ABITRAM, ADCY1, ADGRB1, ANK3, APBB1, APOE, ARHGAP33, ARHGAP44, ARX, ATP7A, AUTS2, BAIAP2, BCL11A, BCL11B, BCL2, BRAF, C1D, CACNG7, CCK, CDH4, CHRFAM7A, CHRNA7, CRMP1, CTNNA2, CTNND2, CXCR4, CXXC1, DCDC2, DCLK1, DISC1, DMD, DNAAF3, DNM2, DNM3, DOCK7, DOK5, DOK6, DSCAML1, EFNA1, EFNA3, EFNA4, EFNB1, EPHA3, EPHA4, EPHA5, EPHA6, EPHA7, EPHB3, EXT1, FBXW8, FGFR2, FLRT2, FLRT3, FN1, FOXD1, FRS2, FSTL4, FZD9, GAB2, GAS7, GATA3, GFRA1, GJA1, GLI2, GLI3, GORASP1, GRB7, GRIN1, GRIP1, ILK, ITGA1, ITGA4, ITPKA, KIF1A, KIF5A, KIRREL3, KLF7, L1CAM, LHX4, LHX9, LRP1, LRP2, LYPLA2, MAP2, MAPT, MATN2, MFSD2A, MNX1, MYPN, NEFL, NGEF, NKX2-1, NKX6-1, NLGN1, NOG, NPTX1, NR4A2, NR4A3, NRCAM, NRK, NRN1, NRXN3, NTRK3, NYAP1, OLFM1, OPHN1, PAX2, PHACTR1, PLXNA3, PLXNB3, POU4F3, PPP1R9A, PQBP1, PRKCZ, PSPN, PTCH1, RAB3A, RAP1GAP, RAP2A, RET, RIMS2, SDC2, SEMA3B, SEMA3D, SEMA3E, SEMA4G, SEMA6A, SHC3, SHH, SIPA1L1, SLIT1, SLIT2, SLIT3, SLITRK3, SLITRK4, SLITRK6, SMOX, SPAST, SPTB, SPTBN2, SPTBN4, SRCIN1, SS18L2, STXBP1, SYT17, SYT3, THY1, TIAM1, TIAM2, TMEFF2, TNFRSF12A, TNIK, TRAK2, TRIM46, TRPV2, UNC5D, VAMP7, VANGL2, WNT3, XK, ZFYVE27, ZNF335]
GO:0032774	RNA biosynthetic process	BiologicalProcess	0.00	[ABCA2, ABLIM2, ABLIM3, ACTR8, ACVR2A, ACVRL1, ADCY1, ADRB2, AFAP1L2, AGAP2, AGER, AHRR, AIRE, ALX1, ALX4, ALYREF, ANKRD1, ANKRD2, APBB1, APOE, AREG, ARHGEF10L, ARID3B, ARID5B, ARX, ATF3, ATF5, ATF7, ATP2B4, AURKAIP1, AUTS2, BACH2, BANP, BARX1, BCL11A, BCL11B, BCL2L12, BCL6, BCL9, BEND6, BEX2, BHLHE41, BLM, BRCA2, BRD3, BTN3A3, C11orf95, C1D, C1orf61, CA9, CARF, CASZ1, CAVIN4, CBX7, CC2D1A, CCDC62, CCNE1, CD38, CD40, CDK14, CDK18, CDK5RAP3, CDKN1C, CDON, CENPV, CHD1, CHD3, CHD5, CIITA, CITED2, CITED4, CNOT3, COL1A1, COMMD1, CRAT, CREB3, CREB3L1, CREBRF, CREM, CRYAB, CSRNP3, CSTF2, CXXC1, CXXC5, CYP1A1, CYP1B1, CYP27B1, DACT1, DBP, DDN, DEDD, DEPDC1, DGKQ, DKK1, DLL1, DLX1, DLX2, DLX4, DMD, DMRTA1, DMRTA2, DNM2, DNPEP, DOT1L, DPF1, DUSP26, DUSP28, DXO, DYRK1B, E2F6, EAF2, EBF1, EBF2, EBF3, EBF4, ECM1, EDA, EDA2R, EFCAB7, EFNA1, EGLN3, EGR1, ELK1, ELL2, ELOF1, EPC1, EPCAM, EPHA5, EPOP, ERCC2, EREG, ERLIN2, ESR2, ESRRG, ETV2, EYA1, EZH1, FANK1, FBLN5, FGFR2, FLCN, FOS, FOSL1, FOSL2, FOXA1, FOXA3, FOXD1, FOXD3, FOXD4L1, FOXE1, FOXF2, FOXN4, FOXP1, FOXP2, FOXP4, FST, FZD5, GADD45A, GAS7, GATA2, GATA3, GATA5, GATA6, GATD3A, GLI1, GLI2, GLI3, GLMP, GREM1, GRHL3, GRIN1, GRIP1, GSC, GTF2E1, GTF2H2, GTF2H2C, GTF2H4, H1-10, H1-5, H2AC15, H2AZ1, H4C12, HAS3, HDAC9, HES1, HES2, HEXIM2, HEY1, HIGD1A, HINFP, HIVEP3, HLX, HMGN5, HMOX1, HMX1, HNF1A, HNF4A, HOXA1, HOXA10, HOXA13, HOXA5, HOXA6, HOXB13, HOXB2, HOXB3, HOXB5, HOXB6, HOXB7, HOXB8, HOXB9, HOXC6, HOXC8, HOXC9, HOXD1, HOXD10, HOXD13, HOXD9, HR, HSBP1L1, HSF2BP, HSF4, HSPA1A, ICAM1, IFI16, IFI27, IGBP1, IGHMBP2, IGSF1, IKZF4, IL11, IL18R1, IL1RAP, ILK, ING1, ING4, INHBA, INTS12, INTS5, IRAK2, IRF1, IRF4, IRF5, JAZF1, KAT2B, KAT5, KAT6B, KCNH4, KDM4C, KDM5B, KDM7A, KHSRP, KLF16, KLF2, KLF4, KLF7, KLF9, KLRG1, KRBA1, KRBA2, KTI12, L3MBTL1, LAGE3, LAMTOR5, LANCL2, LBHD1, LBX2, LEFTY1, LGALS9, LHX4, LHX6, LHX8, LHX9, LIMS1, LIN37, LMO2, LOXL3, LRIF1, MAD2L2, MAFK, MAGI1, MAK, MALT1, MAML3, MAMLD1, MAP2K5, MAP3K12, MAPK10, MAPK11, MAPRE3, MBTPS2, MCIDAS, MCPH1, MED11, MED16, MED17, MED18, MED23, MED26, MED30, MEF2B, MEIS1, MEPCE, MESP1, MGAM, MIA3, MID2, MIER2, MIER3, MITF, MLXIPL, MN1, MNX1, MOSPD1, MRPL12, MRPL23, MSANTD1, MSX2, MTRES1, MTURN, MUC1, MXD3, MXI1, MYCBP, MYCL, MYPOP, NANOS1, NCK1, NDRG1, NEK4, NFATC1, NFIX, NFKBIB, NFKBIZ, NFYA, NHS, NKX2-1, NKX2-3, NKX2-5, NKX3-1, NKX3-2, NKX6-1, NLRC3, NME2, NOD2, NOG, NPPA, NR1D1, NR1H4, NR2E3, NR2F6, NR3C2, NR4A1, NR4A2, NR4A3, NRARP, NRL, NTRK3, NUPR1, OSR2, OTX1, OVOL3, P2RY1, PABPN1, PADI2, PAGR1, PAX2, PAX7, PAX8, PAX9, PBX2, PBX4, PBXIP1, PCBD2, PCGF1, PCGF6, PELI1, PELP1, PER2, PER3, PERM1, PEX14, PGBD1, PHF1, PHF21A, PITX1, PITX2, PKIA, PKMYT1, PKNOX1, PLAC8, PLAG1, PLCB1, POLA1, POLR1C, POLR2H, POLR2J, POLR3G, POLR3K, POU3F3, POU4F3, POU5F1, POU5F2, POU6F1, PPARA, PPM1D, PPP1R13L, PPP3R1, PQBP1, PRDM1, PRDM11, PRDM13, PRDM5, PRDM8, PRIM2, PRIMPOL, PRKCH, PRKCZ, PRKD1, PRNP, PRRX2, PSMA1, PSMA2, PSMB10, PSMB8, PSMB9, PSMC3IP, PSMD12, PSMD9, PSTK, PTCH1, PTP4A3, PTPN18, PXDN, PYCARD, RBM14, RCOR2, RELB, RET, RGMB, RHEBL1, RIPK4, RITA1, RNASEL, RNF31, RNF8, RNGTT, ROR2, RPAP1, RPRD2, RPTOR, RRAGC, RRM2, RRP8, RSF1, RTKN2, RUNX2, RWDD3, RXRA, SALL1, SALL2, SALL4, SAMD11, SARNP, SATB1, SATB2, SCAF4, SCGB1A1, SCMH1, SCML2, SDR16C5, SETDB2, SETSIP, SFR1, SFRP1, SFRP5, SH2D2A, SHH, SIGIRR, SIM2, SIX4, SLBP, SLC25A33, SLC40A1, SMARCB1, SMIM29, SMOX, SMYD3, SNAI1, SNAPC1, SNAPC5, SNCA, SNRPB, SNRPF, SOX17, SOX2, SOX21, SOX4, SOX5, SOX6, SOX7, SOX9, SP140L, SP8, SPAG8, SPHK1, SPIN3, SPIN4, SPOCD1, SRFBP1, SS18L2, SSBP2, SSBP4, STAT4, STAT5A, STK36, STOX1, SUMO4, SUPT3H, SUPT4H1, SUV39H1, TAB3, TADA2A, TAF6L, TAF8, TBX20, TBX3, TBX6, TCEA3, TCEANC, TCF19, TCF7, TDRD3, TEAD3, TENM1, TESC, TET1, TFAP2C, TFR2, TGFA, TGFB1I1, TGFB3, THAP11, THAP12, THAP6, THAP8, TIMP1, TLE2, TLR3, TMEM98, TOB1, TOX3, TP53, TP53INP1, TRAF1, TRAF6, TRAK2, TRAPPC2, TRIM13, TRIM22, TRIM29, TRIM4, TRIM45, TRIM66, TRPS1, TSC22D4, TSFM, TSHZ3, TSSK4, TWIST1, TWISTNB, TWNK, UBE2I, UCN, UNC13D, USF3, UTP15, UXT, VDR, VGLL4, WDR43, WDR77, WDTC1, WNT11, YY2, ZBED3, ZBTB17, ZBTB2, ZBTB32, ZBTB34, ZBTB45, ZBTB46, ZBTB48, ZBTB49, ZC3H6, ZFP37, ZFP41, ZFP92, ZFPL1, ZFX, ZKSCAN2, ZKSCAN3, ZKSCAN4, ZMYM5, ZMYM6, ZMYND8, ZNF10, ZNF112, ZNF121, ZNF134, ZNF138, ZNF14, ZNF165, ZNF169, ZNF17, ZNF174, ZNF175, ZNF180, ZNF182, ZNF184, ZNF189, ZNF19, ZNF2, ZNF202, ZNF212, ZNF22, ZNF222, ZNF225, ZNF229, ZNF23, ZNF230, ZNF232, ZNF233, ZNF235, ZNF239, ZNF248, ZNF25, ZNF250, ZNF254, ZNF256, ZNF266, ZNF280A, ZNF280B,
GO:2001141	regulation of RNA biosynthetic process	BiologicalProcess	0.00	[ABCA2, ABLIM3, ACTR8, ACVR2A, ACVRL1, ADCY1, ADRB2, AFAP1L2, AGAP2, AGER, AHRR, AIRE, ALX1, ALX4, ALYREF, ANKRD1, ANKRD2, APBB1, APOE, AREG, ARHGEF10L, ARID3B, ARID5B, ARX, ATF3, ATF5, ATF7, ATP2B4, AURKAIP1, AUTS2, BACH2, BANP, BARX1, BCL11A, BCL11B, BCL2L12, BCL6, BCL9, BEND6, BEX2, BHLHE41, BLM, BRCA2, BRD3, C11orf95, C1D, C1orf61, CA9, CARF, CASZ1, CAVIN4, CBX7, CC2D1A, CCDC62, CCNE1, CD38, CD40, CDK14, CDK18, CDK5RAP3, CDKN1C, CDON, CENPV, CHD1, CHD3, CHD5, CIITA, CITED2, CITED4, CNOT3, COL1A1, COMMD1, CRAT, CREB3, CREB3L1, CREBRF, CREM, CRYAB, CSRNP3, CXXC1, CXXC5, CYP1A1, CYP1B1, CYP27B1, DACT1, DBP, DDN, DEDD, DEPDC1, DGKQ, DKK1, DLL1, DLX1, DLX2, DLX4, DMD, DMRTA1, DMRTA2, DNM2, DNPEP, DOT1L, DPF1, DUSP26, DUSP28, DXO, DYRK1B, E2F6, EAF2, EBF1, EBF2, EBF3, EBF4, ECM1, EDA, EDA2R, EFCAB7, EFNA1, EGLN3, EGR1, ELK1, ELOF1, EPC1, EPCAM, EPHA5, EPOP, ERCC2, EREG, ERLIN2, ESR2, ESRRG, ETV2, EYA1, EZH1, FANK1, FBLN5, FGFR2, FLCN, FOS, FOSL1, FOSL2, FOXA1, FOXA3, FOXD1, FOXD3, FOXD4L1, FOXE1, FOXF2, FOXN4, FOXP1, FOXP2, FOXP4, FST, FZD5, GADD45A, GAS7, GATA2, GATA3, GATA5, GATA6, GATD3A, GLI1, GLI2, GLI3, GLMP, GREM1, GRHL3, GRIN1, GRIP1, GSC, GTF2E1, GTF2H2, GTF2H2C, GTF2H4, H1-10, H1-5, H2AC15, H2AZ1, H4C12, HAS3, HDAC9, HES1, HES2, HEXIM2, HEY1, HIGD1A, HINFP, HIVEP3, HLX, HMGN5, HMOX1, HMX1, HNF1A, HNF4A, HOXA1, HOXA10, HOXA13, HOXA5, HOXA6, HOXB13, HOXB2, HOXB3, HOXB5, HOXB6, HOXB7, HOXB8, HOXB9, HOXC6, HOXC8, HOXC9, HOXD1, HOXD10, HOXD13, HOXD9, HR, HSBP1L1, HSF4, HSPA1A, ICAM1, IFI16, IFI27, IGBP1, IGHMBP2, IGSF1, IKZF4, IL11, IL18R1, IL1RAP, ILK, ING1, ING4, INHBA, IRAK2, IRF1, IRF4, IRF5, JAZF1, KAT2B, KAT5, KAT6B, KCNH4, KDM4C, KDM5B, KDM7A, KHSRP, KLF16, KLF2, KLF4, KLF7, KLF9, KLRG1, KRBA1, KRBA2, KTI12, L3MBTL1, LAGE3, LAMTOR5, LANCL2, LBHD1, LBX2, LEFTY1, LGALS9, LHX4, LHX6, LHX8, LHX9, LIMS1, LIN37, LMO2, LOXL3, LRIF1, MAD2L2, MAFK, MAGI1, MAK, MALT1, MAML3, MAMLD1, MAP2K5, MAP3K12, MAPK10, MAPK11, MAPRE3, MBTPS2, MCIDAS, MCPH1, MED11, MED16, MED17, MED18, MED23, MED26, MED30, MEF2B, MEIS1, MEPCE, MESP1, MGAM, MIA3, MID2, MIER2, MIER3, MITF, MLXIPL, MN1, MNX1, MOSPD1, MRPL12, MRPL23, MSANTD1, MSX2, MTRES1, MTURN, MUC1, MXD3, MXI1, MYCBP, MYCL, MYPOP, NANOS1, NCK1, NEK4, NFATC1, NFIX, NFKBIB, NFKBIZ, NFYA, NHS, NKX2-1, NKX2-3, NKX2-5, NKX3-1, NKX3-2, NKX6-1, NLRC3, NME2, NOD2, NOG, NR1D1, NR1H4, NR2E3, NR2F6, NR3C2, NR4A1, NR4A2, NR4A3, NRARP, NRL, NTRK3, NUPR1, OSR2, OTX1, OVOL3, P2RY1, PADI2, PAGR1, PAX2, PAX7, PAX8, PAX9, PBX2, PBX4, PBXIP1, PCBD2, PCGF1, PCGF6, PELI1, PELP1, PER2, PER3, PERM1, PEX14, PGBD1, PHF1, PHF21A, PITX1, PITX2, PKIA, PKMYT1, PKNOX1, PLAC8, PLAG1, PLCB1, POLA1, POLR3G, POU3F3, POU4F3, POU5F1, POU5F2, POU6F1, PPARA, PPM1D, PPP1R13L, PPP3R1, PQBP1, PRDM1, PRDM11, PRDM13, PRDM5, PRDM8, PRKCH, PRKCZ, PRKD1, PRNP, PRRX2, PSMA1, PSMA2, PSMB10, PSMB8, PSMB9, PSMC3IP, PSMD12, PSMD9, PTCH1, PTP4A3, PXDN, PYCARD, RBM14, RCOR2, RELB, RET, RGMB, RHEBL1, RIPK4, RITA1, RNASEL, RNF31, RNF8, ROR2, RPTOR, RRM2, RRP8, RSF1, RTKN2, RUNX2, RWDD3, RXRA, SALL1, SALL2, SALL4, SAMD11, SARNP, SATB1, SATB2, SCAF4, SCGB1A1, SCMH1, SCML2, SDR16C5, SETDB2, SETSIP, SFR1, SFRP1, SFRP5, SH2D2A, SHH, SIGIRR, SIM2, SIX4, SLC40A1, SMARCB1, SMIM29, SMOX, SMYD3, SNAI1, SNCA, SOX17, SOX2, SOX21, SOX4, SOX5, SOX6, SOX7, SOX9, SP140L, SP8, SPAG8, SPHK1, SPIN3, SPIN4, SRFBP1, SS18L2, SSBP2, SSBP4, STAT4, STAT5A, STK36, STOX1, SUMO4, SUPT3H, SUPT4H1, SUV39H1, TAB3, TADA2A, TAF6L, TAF8, TBX20, TBX3, TBX6, TCEA3, TCF19, TCF7, TDRD3, TEAD3, TENM1, TESC, TET1, TFAP2C, TFR2, TGFA, TGFB1I1, TGFB3, THAP11, THAP12, THAP6, THAP8, TIMP1, TLE2, TLR3, TMEM98, TOB1, TOX3, TP53, TP53INP1, TRAF1, TRAF6, TRAK2, TRAPPC2, TRIM13, TRIM22, TRIM29, TRIM4, TRIM45, TRIM66, TRPS1, TSC22D4, TSFM, TSHZ3, TSSK4, TWIST1, UBE2I, UCN, UNC13D, USF3, UTP15, UXT, VDR, VGLL4, WDR43, WDR77, WDTC1, WNT11, YY2, ZBED3, ZBTB17, ZBTB2, ZBTB32, ZBTB34, ZBTB45, ZBTB46, ZBTB48, ZBTB49, ZC3H6, ZFP37, ZFP41, ZFP92, ZFPL1, ZFX, ZKSCAN2, ZKSCAN3, ZKSCAN4, ZMYM5, ZMYM6, ZMYND8, ZNF10, ZNF112, ZNF121, ZNF134, ZNF138, ZNF14, ZNF165, ZNF169, ZNF17, ZNF174, ZNF175, ZNF180, ZNF182, ZNF184, ZNF189, ZNF19, ZNF2, ZNF202, ZNF212, ZNF22, ZNF222, ZNF225, ZNF229, ZNF23, ZNF230, ZNF232, ZNF233, ZNF235, ZNF239, ZNF248, ZNF25, ZNF250, ZNF254, ZNF256, ZNF266, ZNF280A, ZNF280B, ZNF285, ZNF286B, ZNF296, ZNF3, ZNF30, ZNF300, ZNF304, ZNF316, ZNF319, ZNF322, ZNF324B, ZNF326, ZNF329, ZNF333, ZNF335, ZNF33A, ZNF34, ZNF343, ZNF35, ZNF350, ZNF354C, ZNF37A, ZNF396, ZNF418, ZNF420, ZNF423, ZNF425, ZNF432, ZNF44, ZNF443, ZNF444,

GO:0006351	transcription, DNA-templated	BiologicalProcess	0.00	[ABCA2, ABLIM2, ABLIM3, ACTR8, ACVR2A, ACVRL1, ADCY1, ADRB2, AFAP1L2, AGAP2, AGER, AHRR, AIRE, ALX1, ALX4, ALYREF, ANKRD1, ANKRD2, APBB1, APOE, AREG, ARHGEF10L, ARID3B, ARID5B, ARX, ATF3, ATF5, ATF7, ATP2B4, AUTS2, BACH2, BANP, BARX1, BCL11A, BCL11B, BCL2L12, BCL6, BCL9, BEX2, BHLHE41, BLM, BRCA2, BRD3, BTN3A3, C11orf95, C1D, C1orf61, CA9, CARF, CASZ1, CAVIN4, CBX7, CC2D1A, CCDC62, CCNE1, CD38, CD40, CDK14, CDK18, CDK5RAP3, CDKN1C, CDON, CENPV, CHD1, CHD3, CHD5, CIITA, CITED2, CITED4, CNOT3, COL1A1, COMMD1, CRAT, CREB3, CREB3L1, CREBRF, CREM, CRYAB, CSRNP3, CSTF2, CXXC1, CXXC5, CYP1A1, CYP1B1, CYP27B1, DACT1, DBP, DDN, DEDD, DEPDC1, DGKQ, DKK1, DLL1, DLX1, DLX2, DLX4, DMD, DMRTA1, DMRTA2, DNM2, DNPEP, DOT1L, DPF1, DUSP26, DUSP28, DXO, DYRK1B, E2F6, EAF2, EBF1, EBF2, EBF3, EBF4, ECM1, EDA, EDA2R, EFCAB7, EFNA1, EGLN3, EGR1, ELK1, ELL2, ELOF1, EPC1, EPCAM, EPHA5, EPOP, ERCC2, EREG, ERLIN2, ESR2, ESRRG, ETV2, EYA1, EZH1, FANK1, FBLN5, FGFR2, FLCN, FOS, FOSL1, FOSL2, FOXA1, FOXA3, FOXD1, FOXD3, FOXD4L1, FOXE1, FOXF2, FOXN4, FOXP1, FOXP2, FOXP4, FST, FZD5, GADD45A, GAS7, GATA2, GATA3, GATA5, GATA6, GATD3A, GLI1, GLI2, GLI3, GLMP, GREM1, GRHL3, GRIN1, GRIP1, GSC, GTF2E1, GTF2H2, GTF2H2C, GTF2H4, H1-10, H1-5, H2AC15, H2AZ1, H4C12, HAS3, HDAC9, HES1, HES2, HEXIM2, HEY1, HIGD1A, HINFP, HIVEP3, HLX, HMGN5, HMOX1, HMX1, HNF1A, HNF4A, HOXA1, HOXA10, HOXA13, HOXA5, HOXA6, HOXB13, HOXB2, HOXB3, HOXB5, HOXB6, HOXB7, HOXB8, HOXB9, HOXC6, HOXC8, HOXC9, HOXD1, HOXD10, HOXD13, HOXD9, HR, HSF2BP, HSF4, HSPA1A, ICAM1, IFI16, IFI27, IGBP1, IGHMBP2, IGSF1, IKZF4, IL11, IL18R1, IL1RAP, ILK, ING1, ING4, INHBA, INTS12, INTS5, IRAK2, IRF1, IRF4, IRF5, JAZF1, KAT2B, KAT5, KAT6B, KCNH4, KDM4C, KDM5B, KDM7A, KHSRP, KLF16, KLF2, KLF4, KLF7, KLF9, KLRG1, KRBA1, KRBA2, KTI12, L3MBTL1, LAGE3, LAMTOR5, LANCL2, LBHD1, LBX2, LEFTY1, LGALS9, LHX4, LHX6, LHX8, LHX9, LIMS1, LIN37, LMO2, LOXL3, LRIF1, MAD2L2, MAFK, MAGI1, MALT1, MAML3, MAMLD1, MAP2K5, MAP3K12, MAPK10, MAPK11, MAPRE3, MBTPS2, MCIDAS, MCPH1, MED11, MED16, MED17, MED18, MED23, MED26, MED30, MEF2B, MEIS1, MEPCE, MESP1, MGAM, MIA3, MID2, MIER2, MIER3, MITF, MLXIPL, MN1, MNX1, MOSPD1, MRPL12, MRPL23, MSANTD1, MSX2, MTRES1, MTURN, MUC1, MXD3, MXI1, MYCBP, MYCL, MYPOP, NANOS1, NCK1, NDRG1, NEK4, NFATC1, NFIX, NFKBIB, NFKBIZ, NFYA, NHS, NKX2-1, NKX2-3, NKX2-5, NKX3-1, NKX3-2, NKX6-1, NLRC3, NME2, NOD2, NOG, NPPA, NR1D1, NR1H4, NR2E3, NR2F6, NR3C2, NR4A1, NR4A2, NR4A3, NRARP, NRL, NUPR1, OSR2, OTX1, OVOL3, P2RY1, PABPN1, PADI2, PAGR1, PAX2, PAX7, PAX8, PAX9, PBX2, PBX4, PBXIP1, PCBD2, PCGF1, PCGF6, PELI1, PELP1, PER2, PER3, PERM1, PEX14, PGBD1, PHF1, PHF21A, PITX1, PITX2, PKIA, PKMYT1, PKNOX1, PLAC8, PLAG1, PLCB1, POLA1, POLR1C, POLR2H, POLR2J, POLR3G, POLR3K, POU3F3, POU4F3, POU5F1, POU5F2, POU6F1, PPARA, PPM1D, PPP1R13L, PPP3R1, PQBP1, PRDM1, PRDM11, PRDM13, PRDM5, PRDM8, PRKCH, PRKCZ, PRKD1, PRNP, PRRX2, PSMA1, PSMA2, PSMB10, PSMB8, PSMB9, PSMD12, PSMD9, PTCH1, PTP4A3, PTPN18, PYCARD, RBM14, RCOR2, RELB, RET, RGMB, RHEBL1, RIPK4, RITA1, RNASEL, RNF31, RNF8, RNGTT, ROR2, RPAP1, RPRD2, RPTOR, RRAGC, RRM2, RRP8, RSF1, RTKN2, RUNX2, RWDD3, RXRA, SALL1, SALL2, SALL4, SAMD11, SARNP, SATB1, SATB2, SCAF4, SCGB1A1, SCMH1, SCML2, SDR16C5, SETDB2, SETSIP, SFR1, SFRP1, SFRP5, SH2D2A, SHH, SIGIRR, SIM2, SIX4, SLBP, SLC25A33, SLC40A1, SMARCB1, SMIM29, SMOX, SMYD3, SNAI1, SNAPC1, SNAPC5, SNCA, SNRPB, SNRPF, SOX17, SOX2, SOX21, SOX4, SOX5, SOX6, SOX7, SOX9, SP140L, SP8, SPAG8, SPHK1, SPIN3, SPIN4, SPOCD1, SRFBP1, SS18L2, SSBP2, SSBP4, STAT4, STAT5A, STK36, STOX1, SUMO4, SUPT3H, SUPT4H1, SUV39H1, TAB3, TADA2A, TAF6L, TAF8, TBX20, TBX3, TBX6, TCEA3, TCEANC, TCF19, TCF7, TEAD3, TENM1, TESC, TET1, TFAP2C, TFR2, TGFA, TGFB1I1, TGFB3, THAP11, THAP12, THAP6, THAP8, TIMP1, TLE2, TLR3, TMEM98, TOX3, TP53, TP53INP1, TRAF1, TRAF6, TRAK2, TRAPPC2, TRIM13, TRIM22, TRIM29, TRIM4, TRIM45, TRIM66, TRPS1, TSC22D4, TSFM, TSHZ3, TSSK4, TWIST1, TWISTNB, TWNK, UBE2I, UCN, USF3, UTP15, UXT, VDR, VGLL4, WDR43, WDR77, WDTC1, WNT11, YY2, ZBED3, ZBTB17, ZBTB2, ZBTB32, ZBTB34, ZBTB45, ZBTB46, ZBTB48, ZBTB49, ZC3H6, ZFP37, ZFP41, ZFP92, ZFPL1, ZFX, ZKSCAN2, ZKSCAN3, ZKSCAN4, ZMYM5, ZMYM6, ZMYND8, ZNF10, ZNF112, ZNF121, ZNF134, ZNF138, ZNF14, ZNF165, ZNF169, ZNF17, ZNF174, ZNF175, ZNF180, ZNF182, ZNF184, ZNF189, ZNF19, ZNF2, ZNF202, ZNF212, ZNF22, ZNF222, ZNF225, ZNF229, ZNF23, ZNF230, ZNF232, ZNF233, ZNF235, ZNF239, ZNF248, ZNF25, ZNF250, ZNF254, ZNF256, ZNF266, ZNF280A, ZNF280B, ZNF285, ZNF286B, ZNF296, ZNF3, ZNF30, ZNF300, ZNF304, ZNF316, ZNF319, ZNF322, ZNF324B, ZNF326, ZNF329,
GO:0097659	nucleic acid-templated transcription	BiologicalProcess	0.00	[ABCA2, ABLIM2, ABLIM3, ACTR8, ACVR2A, ACVRL1, ADCY1, ADRB2, AFAP1L2, AGAP2, AGER, AHRR, AIRE, ALX1, ALX4, ALYREF, ANKRD1, ANKRD2, APBB1, APOE, AREG, ARHGEF10L, ARID3B, ARID5B, ARX, ATF3, ATF5, ATF7, ATP2B4, AURKAIP1, AUTS2, BACH2, BANP, BARX1, BCL11A, BCL11B, BCL2L12, BCL6, BCL9, BEND6, BEX2, BHLHE41, BLM, BRCA2, BRD3, BTN3A3, C11orf95, C1D, C1orf61, CA9, CARF, CASZ1, CAVIN4, CBX7, CC2D1A, CCDC62, CCNE1, CD38, CD40, CDK14, CDK18, CDK5RAP3, CDKN1C, CDON, CENPV, CHD1, CHD3, CHD5, CIITA, CITED2, CITED4, CNOT3, COL1A1, COMMD1, CRAT, CREB3, CREB3L1, CREBRF, CREM, CRYAB, CSRNP3, CSTF2, CXXC1, CXXC5, CYP1A1, CYP1B1, CYP27B1, DACT1, DBP, DDN, DEDD, DEPDC1, DGKQ, DKK1, DLL1, DLX1, DLX2, DLX4, DMD, DMRTA1, DMRTA2, DNM2, DNPEP, DOT1L, DPF1, DUSP26, DUSP28, DXO, DYRK1B, E2F6, EAF2, EBF1, EBF2, EBF3, EBF4, ECM1, EDA, EDA2R, EFCAB7, EFNA1, EGLN3, EGR1, ELK1, ELL2, ELOF1, EPC1, EPCAM, EPHA5, EPOP, ERCC2, EREG, ERLIN2, ESR2, ESRRG, ETV2, EYA1, EZH1, FANK1, FBLN5, FGFR2, FLCN, FOS, FOSL1, FOSL2, FOXA1, FOXA3, FOXD1, FOXD3, FOXD4L1, FOXE1, FOXF2, FOXN4, FOXP1, FOXP2, FOXP4, FST, FZD5, GADD45A, GAS7, GATA2, GATA3, GATA5, GATA6, GATD3A, GLI1, GLI2, GLI3, GLMP, GREM1, GRHL3, GRIN1, GRIP1, GSC, GTF2E1, GTF2H2, GTF2H2C, GTF2H4, H1-10, H1-5, H2AC15, H2AZ1, H4C12, HAS3, HDAC9, HES1, HES2, HEXIM2, HEY1, HIGD1A, HINFP, HIVEP3, HLX, HMGN5, HMOX1, HMX1, HNF1A, HNF4A, HOXA1, HOXA10, HOXA13, HOXA5, HOXA6, HOXB13, HOXB2, HOXB3, HOXB5, HOXB6, HOXB7, HOXB8, HOXB9, HOXC6, HOXC8, HOXC9, HOXD1, HOXD10, HOXD13, HOXD9, HR, HSBP1L1, HSF2BP, HSF4, HSPA1A, ICAM1, IFI16, IFI27, IGBP1, IGHMBP2, IGSF1, IKZF4, IL11, IL18R1, IL1RAP, ILK, ING1, ING4, INHBA, INTS12, INTS5, IRAK2, IRF1, IRF4, IRF5, JAZF1, KAT2B, KAT5, KAT6B, KCNH4, KDM4C, KDM5B, KDM7A, KHSRP, KLF16, KLF2, KLF4, KLF7, KLF9, KLRG1, KRBA1, KRBA2, KTI12, L3MBTL1, LAGE3, LAMTOR5, LANCL2, LBHD1, LBX2, LEFTY1, LGALS9, LHX4, LHX6, LHX8, LHX9, LIMS1, LIN37, LMO2, LOXL3, LRIF1, MAD2L2, MAFK, MAGI1, MAK, MALT1, MAML3, MAMLD1, MAP2K5, MAP3K12, MAPK10, MAPK11, MAPRE3, MBTPS2, MCIDAS, MCPH1, MED11, MED16, MED17, MED18, MED23, MED26, MED30, MEF2B, MEIS1, MEPCE, MESP1, MGAM, MIA3, MID2, MIER2, MIER3, MITF, MLXIPL, MN1, MNX1, MOSPD1, MRPL12, MRPL23, MSANTD1, MSX2, MTRES1, MTURN, MUC1, MXD3, MXI1, MYCBP, MYCL, MYPOP, NANOS1, NCK1, NDRG1, NEK4, NFATC1, NFIX, NFKBIB, NFKBIZ, NFYA, NHS, NKX2-1, NKX2-3, NKX2-5, NKX3-1, NKX3-2, NKX6-1, NLRC3, NME2, NOD2, NOG, NPPA, NR1D1, NR1H4, NR2E3, NR2F6, NR3C2, NR4A1, NR4A2, NR4A3, NRARP, NRL, NUPR1, OSR2, OTX1, OVOL3, P2RY1, PABPN1, PADI2, PAGR1, PAX2, PAX7, PAX8, PAX9, PBX2, PBX4, PBXIP1, PCBD2, PCGF1, PCGF6, PELI1, PELP1, PER2, PER3, PERM1, PEX14, PGBD1, PHF1, PHF21A, PITX1, PITX2, PKIA, PKMYT1, PKNOX1, PLAC8, PLAG1, PLCB1, POLA1, POLR1C, POLR2H, POLR2J, POLR3G, POLR3K, POU3F3, POU4F3, POU5F1, POU5F2, POU6F1, PPARA, PPM1D, PPP1R13L, PPP3R1, PQBP1, PRDM1, PRDM11, PRDM13, PRDM5, PRDM8, PRKCH, PRKCZ, PRKD1, PRNP, PRRX2, PSMA1, PSMA2, PSMB10, PSMB8, PSMB9, PSMC3IP, PSMD12, PSMD9, PTCH1, PTP4A3, PTPN18, PXDN, PYCARD, RBM14, RCOR2, RELB, RET, RGMB, RHEBL1, RIPK4, RITA1, RNASEL, RNF31, RNF8, RNGTT, ROR2, RPAP1, RPRD2, RPTOR, RRAGC, RRM2, RRP8, RSF1, RTKN2, RUNX2, RWDD3, RXRA, SALL1, SALL2, SALL4, SAMD11, SARNP, SATB1, SATB2, SCAF4, SCGB1A1, SCMH1, SCML2, SDR16C5, SETDB2, SETSIP, SFR1, SFRP1, SFRP5, SH2D2A, SHH, SIGIRR, SIM2, SIX4, SLBP, SLC25A33, SLC40A1, SMARCB1, SMIM29, SMOX, SMYD3, SNAI1, SNAPC1, SNAPC5, SNCA, SNRPB, SNRPF, SOX17, SOX2, SOX21, SOX4, SOX5, SOX6, SOX7, SOX9, SP140L, SP8, SPAG8, SPHK1, SPIN3, SPIN4, SPOCD1, SRFBP1, SS18L2, SSBP2, SSBP4, STAT4, STAT5A, STK36, STOX1, SUMO4, SUPT3H, SUPT4H1, SUV39H1, TAB3, TADA2A, TAF6L, TAF8, TBX20, TBX3, TBX6, TCEA3, TCEANC, TCF19, TCF7, TDRD3, TEAD3, TENM1, TESC, TET1, TFAP2C, TFR2, TGFA, TGFB1I1, TGFB3, THAP11, THAP12, THAP6, THAP8, TIMP1, TLE2, TLR3, TMEM98, TOB1, TOX3, TP53, TP53INP1, TRAF1, TRAF6, TRAK2, TRAPPC2, TRIM13, TRIM22, TRIM29, TRIM4, TRIM45, TRIM66, TRPS1, TSC22D4, TSFM, TSHZ3, TSSK4, TWIST1, TWISTNB, TWNK, UBE2I, UCN, UNC13D, USF3, UTP15, UXT, VDR, VGLL4, WDR43, WDR77, WDTC1, WNT11, YY2, ZBED3, ZBTB17, ZBTB2, ZBTB32, ZBTB34, ZBTB45, ZBTB46, ZBTB48, ZBTB49, ZC3H6, ZFP37, ZFP41, ZFP92, ZFPL1, ZFX, ZKSCAN2, ZKSCAN3, ZKSCAN4, ZMYM5, ZMYM6, ZMYND8, ZNF10, ZNF112, ZNF121, ZNF134, ZNF138, ZNF14, ZNF165, ZNF169, ZNF17, ZNF174, ZNF175, ZNF180, ZNF182, ZNF184, ZNF189, ZNF19, ZNF2, ZNF202, ZNF212, ZNF22, ZNF222, ZNF225, ZNF229, ZNF23, ZNF230, ZNF232, ZNF233, ZNF235, ZNF239, ZNF248, ZNF25, ZNF250, ZNF254, ZNF256, ZNF266, ZNF280A, ZNF280B, ZNF285, ZNF286B,
GO:0006355	regulation of transcription, DNA-templated	BiologicalProcess	0.00	[ABCA2, ABLIM3, ACTR8, ACVR2A, ACVRL1, ADCY1, ADRB2, AFAP1L2, AGAP2, AGER, AHRR, AIRE, ALX1, ALX4, ALYREF, ANKRD1, ANKRD2, APBB1, APOE, AREG, ARHGEF10L, ARID3B, ARID5B, ARX, ATF3, ATF5, ATF7, ATP2B4, AUTS2, BACH2, BANP, BARX1, BCL11A, BCL11B, BCL2L12, BCL6, BCL9, BEX2, BHLHE41, BLM, BRCA2, BRD3, C11orf95, C1D, C1orf61, CA9, CARF, CASZ1, CAVIN4, CBX7, CC2D1A, CCDC62, CCNE1, CD38, CD40, CDK14, CDK18, CDK5RAP3, CDKN1C, CDON, CENPV, CHD1, CHD3, CHD5, CIITA, CITED2, CITED4, CNOT3, COL1A1, COMMD1, CRAT, CREB3, CREB3L1, CREBRF, CREM, CRYAB, CSRNP3, CXXC1, CXXC5, CYP1A1, CYP1B1, CYP27B1, DACT1, DBP, DDN, DEDD, DEPDC1, DGKQ, DKK1, DLL1, DLX1, DLX2, DLX4, DMD, DMRTA1, DMRTA2, DNM2, DNPEP, DOT1L, DPF1, DUSP26, DUSP28, DXO, DYRK1B, E2F6, EAF2, EBF1, EBF2, EBF3, EBF4, ECM1, EDA, EDA2R, EFCAB7, EFNA1, EGLN3, EGR1, ELK1, ELOF1, EPC1, EPCAM, EPHA5, EPOP, ERCC2, EREG, ERLIN2, ESR2, ESRRG, ETV2, EYA1, EZH1, FANK1, FBLN5, FGFR2, FLCN, FOS, FOSL1, FOSL2, FOXA1, FOXA3, FOXD1, FOXD3, FOXD4L1, FOXE1, FOXF2, FOXN4, FOXP1, FOXP2, FOXP4, FST, FZD5, GADD45A, GAS7, GATA2, GATA3, GATA5, GATA6, GATD3A, GLI1, GLI2, GLI3, GLMP, GREM1, GRHL3, GRIN1, GRIP1, GSC, GTF2E1, GTF2H2, GTF2H2C, GTF2H4, H1-10, H1-5, H2AC15, H2AZ1, H4C12, HAS3, HDAC9, HES1, HES2, HEXIM2, HEY1, HIGD1A, HINFP, HIVEP3, HLX, HMGN5, HMOX1, HMX1, HNF1A, HNF4A, HOXA1, HOXA10, HOXA13, HOXA5, HOXA6, HOXB13, HOXB2, HOXB3, HOXB5, HOXB6, HOXB7, HOXB8, HOXB9, HOXC6, HOXC8, HOXC9, HOXD1, HOXD10, HOXD13, HOXD9, HR, HSF4, HSPA1A, ICAM1, IFI16, IFI27, IGBP1, IGHMBP2, IGSF1, IKZF4, IL11, IL18R1, IL1RAP, ILK, ING1, ING4, INHBA, IRAK2, IRF1, IRF4, IRF5, JAZF1, KAT2B, KAT5, KAT6B, KCNH4, KDM4C, KDM5B, KDM7A, KHSRP, KLF16, KLF2, KLF4, KLF7, KLF9, KLRG1, KRBA1, KRBA2, KTI12, L3MBTL1, LAGE3, LAMTOR5, LANCL2, LBHD1, LBX2, LEFTY1, LGALS9, LHX4, LHX6, LHX8, LHX9, LIMS1, LIN37, LMO2, LOXL3, LRIF1, MAD2L2, MAFK, MAGI1, MALT1, MAML3, MAMLD1, MAP2K5, MAP3K12, MAPK10, MAPK11, MAPRE3, MBTPS2, MCIDAS, MCPH1, MED11, MED16, MED17, MED18, MED23, MED26, MED30, MEF2B, MEIS1, MEPCE, MESP1, MGAM, MIA3, MID2, MIER2, MIER3, MITF, MLXIPL, MN1, MNX1, MOSPD1, MRPL12, MRPL23, MSANTD1, MSX2, MTRES1, MTURN, MUC1, MXD3, MXI1, MYCBP, MYCL, MYPOP, NANOS1, NCK1, NEK4, NFATC1, NFIX, NFKBIB, NFKBIZ, NFYA, NHS, NKX2-1, NKX2-3, NKX2-5, NKX3-1, NKX3-2, NKX6-1, NLRC3, NME2, NOD2, NOG, NR1D1, NR1H4, NR2E3, NR2F6, NR3C2, NR4A1, NR4A2, NR4A3, NRARP, NRL, NUPR1, OSR2, OTX1, OVOL3, P2RY1, PADI2, PAGR1, PAX2, PAX7, PAX8, PAX9, PBX2, PBX4, PBXIP1, PCBD2, PCGF1, PCGF6, PELI1, PELP1, PER2, PER3, PERM1, PEX14, PGBD1, PHF1, PHF21A, PITX1, PITX2, PKIA, PKMYT1, PKNOX1, PLAC8, PLAG1, PLCB1, POLA1, POLR3G, POU3F3, POU4F3, POU5F1, POU5F2, POU6F1, PPARA, PPM1D, PPP1R13L, PPP3R1, PQBP1, PRDM1, PRDM11, PRDM13, PRDM5, PRDM8, PRKCH, PRKCZ, PRKD1, PRNP, PRRX2, PSMA1, PSMA2, PSMB10, PSMB8, PSMB9, PSMD12, PSMD9, PTCH1, PTP4A3, PYCARD, RBM14, RCOR2, RELB, RET, RGMB, RHEBL1, RIPK4, RITA1, RNASEL, RNF31, RNF8, ROR2, RPTOR, RRM2, RRP8, RSF1, RTKN2, RUNX2, RWDD3, RXRA, SALL1, SALL2, SALL4, SAMD11, SARNP, SATB1, SATB2, SCAF4, SCGB1A1, SCMH1, SCML2, SDR16C5, SETDB2, SETSIP, SFR1, SFRP1, SFRP5, SH2D2A, SHH, SIGIRR, SIM2, SIX4, SLC40A1, SMARCB1, SMIM29, SMOX, SMYD3, SNAI1, SNCA, SOX17, SOX2, SOX21, SOX4, SOX5, SOX6, SOX7, SOX9, SP140L, SP8, SPAG8, SPHK1, SPIN3, SPIN4, SRFBP1, SS18L2, SSBP2, SSBP4, STAT4, STAT5A, STK36, STOX1, SUMO4, SUPT3H, SUPT4H1, SUV39H1, TAB3, TADA2A, TAF6L, TAF8, TBX20, TBX3, TBX6, TCEA3, TCF19, TCF7, TEAD3, TENM1, TESC, TET1, TFAP2C, TFR2, TGFA, TGFB1I1, TGFB3, THAP11, THAP12, THAP6, THAP8, TIMP1, TLE2, TLR3, TMEM98, TOX3, TP53, TP53INP1, TRAF1, TRAF6, TRAK2, TRAPPC2, TRIM13, TRIM22, TRIM29, TRIM4, TRIM45, TRIM66, TRPS1, TSC22D4, TSFM, TSHZ3, TSSK4, TWIST1, UBE2I, UCN, USF3, UTP15, UXT, VDR, VGLL4, WDR43, WDR77, WDTC1, WNT11, YY2, ZBED3, ZBTB17, ZBTB2, ZBTB32, ZBTB34, ZBTB45, ZBTB46, ZBTB48, ZBTB49, ZC3H6, ZFP37, ZFP41, ZFP92, ZFPL1, ZFX, ZKSCAN2, ZKSCAN3, ZKSCAN4, ZMYM5, ZMYM6, ZMYND8, ZNF10, ZNF112, ZNF121, ZNF134, ZNF138, ZNF14, ZNF165, ZNF169, ZNF17, ZNF174, ZNF175, ZNF180, ZNF182, ZNF184, ZNF189, ZNF19, ZNF2, ZNF202, ZNF212, ZNF22, ZNF222, ZNF225, ZNF229, ZNF23, ZNF230, ZNF232, ZNF233, ZNF235, ZNF239, ZNF248, ZNF25, ZNF250, ZNF254, ZNF256, ZNF266, ZNF280A, ZNF280B, ZNF285, ZNF286B, ZNF296, ZNF3, ZNF30, ZNF300, ZNF304, ZNF316, ZNF319, ZNF322, ZNF324B, ZNF326, ZNF329, ZNF333, ZNF335, ZNF33A, ZNF34, ZNF343, ZNF35, ZNF350, ZNF354C, ZNF37A, ZNF396, ZNF418, ZNF420, ZNF423, ZNF425, ZNF432, ZNF44, ZNF443, ZNF444, ZNF449, ZNF461, ZNF467, ZNF470, ZNF485, ZNF487, ZNF490, ZNF497, ZNF502, ZNF503, ZNF511, ZNF516,
GO:1903506	regulation of nucleic acid-templated transcription	BiologicalProcess	0.00	[ABCA2, ABLIM3, ACTR8, ACVR2A, ACVRL1, ADCY1, ADRB2, AFAP1L2, AGAP2, AGER, AHRR, AIRE, ALX1, ALX4, ALYREF, ANKRD1, ANKRD2, APBB1, APOE, AREG, ARHGEF10L, ARID3B, ARID5B, ARX, ATF3, ATF5, ATF7, ATP2B4, AURKAIP1, AUTS2, BACH2, BANP, BARX1, BCL11A, BCL11B, BCL2L12, BCL6, BCL9, BEND6, BEX2, BHLHE41, BLM, BRCA2, BRD3, C11orf95, C1D, C1orf61, CA9, CARF, CASZ1, CAVIN4, CBX7, CC2D1A, CCDC62, CCNE1, CD38, CD40, CDK14, CDK18, CDK5RAP3, CDKN1C, CDON, CENPV, CHD1, CHD3, CHD5, CIITA, CITED2, CITED4, CNOT3, COL1A1, COMMD1, CRAT, CREB3, CREB3L1, CREBRF, CREM, CRYAB, CSRNP3, CXXC1, CXXC5, CYP1A1, CYP1B1, CYP27B1, DACT1, DBP, DDN, DEDD, DEPDC1, DGKQ, DKK1, DLL1, DLX1, DLX2, DLX4, DMD, DMRTA1, DMRTA2, DNM2, DNPEP, DOT1L, DPF1, DUSP26, DUSP28, DXO, DYRK1B, E2F6, EAF2, EBF1, EBF2, EBF3, EBF4, ECM1, EDA, EDA2R, EFCAB7, EFNA1, EGLN3, EGR1, ELK1, ELOF1, EPC1, EPCAM, EPHA5, EPOP, ERCC2, EREG, ERLIN2, ESR2, ESRRG, ETV2, EYA1, EZH1, FANK1, FBLN5, FGFR2, FLCN, FOS, FOSL1, FOSL2, FOXA1, FOXA3, FOXD1, FOXD3, FOXD4L1, FOXE1, FOXF2, FOXN4, FOXP1, FOXP2, FOXP4, FST, FZD5, GADD45A, GAS7, GATA2, GATA3, GATA5, GATA6, GATD3A, GLI1, GLI2, GLI3, GLMP, GREM1, GRHL3, GRIN1, GRIP1, GSC, GTF2E1, GTF2H2, GTF2H2C, GTF2H4, H1-10, H1-5, H2AC15, H2AZ1, H4C12, HAS3, HDAC9, HES1, HES2, HEXIM2, HEY1, HIGD1A, HINFP, HIVEP3, HLX, HMGN5, HMOX1, HMX1, HNF1A, HNF4A, HOXA1, HOXA10, HOXA13, HOXA5, HOXA6, HOXB13, HOXB2, HOXB3, HOXB5, HOXB6, HOXB7, HOXB8, HOXB9, HOXC6, HOXC8, HOXC9, HOXD1, HOXD10, HOXD13, HOXD9, HR, HSBP1L1, HSF4, HSPA1A, ICAM1, IFI16, IFI27, IGBP1, IGHMBP2, IGSF1, IKZF4, IL11, IL18R1, IL1RAP, ILK, ING1, ING4, INHBA, IRAK2, IRF1, IRF4, IRF5, JAZF1, KAT2B, KAT5, KAT6B, KCNH4, KDM4C, KDM5B, KDM7A, KHSRP, KLF16, KLF2, KLF4, KLF7, KLF9, KLRG1, KRBA1, KRBA2, KTI12, L3MBTL1, LAGE3, LAMTOR5, LANCL2, LBHD1, LBX2, LEFTY1, LGALS9, LHX4, LHX6, LHX8, LHX9, LIMS1, LIN37, LMO2, LOXL3, LRIF1, MAD2L2, MAFK, MAGI1, MAK, MALT1, MAML3, MAMLD1, MAP2K5, MAP3K12, MAPK10, MAPK11, MAPRE3, MBTPS2, MCIDAS, MCPH1, MED11, MED16, MED17, MED18, MED23, MED26, MED30, MEF2B, MEIS1, MEPCE, MESP1, MGAM, MIA3, MID2, MIER2, MIER3, MITF, MLXIPL, MN1, MNX1, MOSPD1, MRPL12, MRPL23, MSANTD1, MSX2, MTRES1, MTURN, MUC1, MXD3, MXI1, MYCBP, MYCL, MYPOP, NANOS1, NCK1, NEK4, NFATC1, NFIX, NFKBIB, NFKBIZ, NFYA, NHS, NKX2-1, NKX2-3, NKX2-5, NKX3-1, NKX3-2, NKX6-1, NLRC3, NME2, NOD2, NOG, NR1D1, NR1H4, NR2E3, NR2F6, NR3C2, NR4A1, NR4A2, NR4A3, NRARP, NRL, NUPR1, OSR2, OTX1, OVOL3, P2RY1, PADI2, PAGR1, PAX2, PAX7, PAX8, PAX9, PBX2, PBX4, PBXIP1, PCBD2, PCGF1, PCGF6, PELI1, PELP1, PER2, PER3, PERM1, PEX14, PGBD1, PHF1, PHF21A, PITX1, PITX2, PKIA, PKMYT1, PKNOX1, PLAC8, PLAG1, PLCB1, POLA1, POLR3G, POU3F3, POU4F3, POU5F1, POU5F2, POU6F1, PPARA, PPM1D, PPP1R13L, PPP3R1, PQBP1, PRDM1, PRDM11, PRDM13, PRDM5, PRDM8, PRKCH, PRKCZ, PRKD1, PRNP, PRRX2, PSMA1, PSMA2, PSMB10, PSMB8, PSMB9, PSMC3IP, PSMD12, PSMD9, PTCH1, PTP4A3, PXDN, PYCARD, RBM14, RCOR2, RELB, RET, RGMB, RHEBL1, RIPK4, RITA1, RNASEL, RNF31, RNF8, ROR2, RPTOR, RRM2, RRP8, RSF1, RTKN2, RUNX2, RWDD3, RXRA, SALL1, SALL2, SALL4, SAMD11, SARNP, SATB1, SATB2, SCAF4, SCGB1A1, SCMH1, SCML2, SDR16C5, SETDB2, SETSIP, SFR1, SFRP1, SFRP5, SH2D2A, SHH, SIGIRR, SIM2, SIX4, SLC40A1, SMARCB1, SMIM29, SMOX, SMYD3, SNAI1, SNCA, SOX17, SOX2, SOX21, SOX4, SOX5, SOX6, SOX7, SOX9, SP140L, SP8, SPAG8, SPHK1, SPIN3, SPIN4, SRFBP1, SS18L2, SSBP2, SSBP4, STAT4, STAT5A, STK36, STOX1, SUMO4, SUPT3H, SUPT4H1, SUV39H1, TAB3, TADA2A, TAF6L, TAF8, TBX20, TBX3, TBX6, TCEA3, TCF19, TCF7, TDRD3, TEAD3, TENM1, TESC, TET1, TFAP2C, TFR2, TGFA, TGFB1I1, TGFB3, THAP11, THAP12, THAP6, THAP8, TIMP1, TLE2, TLR3, TMEM98, TOB1, TOX3, TP53, TP53INP1, TRAF1, TRAF6, TRAK2, TRAPPC2, TRIM13, TRIM22, TRIM29, TRIM4, TRIM45, TRIM66, TRPS1, TSC22D4, TSFM, TSHZ3, TSSK4, TWIST1, UBE2I, UCN, UNC13D, USF3, UTP15, UXT, VDR, VGLL4, WDR43, WDR77, WDTC1, WNT11, YY2, ZBED3, ZBTB17, ZBTB2, ZBTB32, ZBTB34, ZBTB45, ZBTB46, ZBTB48, ZBTB49, ZC3H6, ZFP37, ZFP41, ZFP92, ZFPL1, ZFX, ZKSCAN2, ZKSCAN3, ZKSCAN4, ZMYM5, ZMYM6, ZMYND8, ZNF10, ZNF112, ZNF121, ZNF134, ZNF138, ZNF14, ZNF165, ZNF169, ZNF17, ZNF174, ZNF175, ZNF180, ZNF182, ZNF184, ZNF189, ZNF19, ZNF2, ZNF202, ZNF212, ZNF22, ZNF222, ZNF225, ZNF229, ZNF23, ZNF230, ZNF232, ZNF233, ZNF235, ZNF239, ZNF248, ZNF25, ZNF250, ZNF254, ZNF256, ZNF266, ZNF280A, ZNF280B, ZNF285, ZNF286B, ZNF296, ZNF3, ZNF30, ZNF300, ZNF304, ZNF316, ZNF319, ZNF322, ZNF324B, ZNF326, ZNF329, ZNF333, ZNF335, ZNF33A, ZNF34, ZNF343, ZNF35, ZNF350, ZNF354C, ZNF37A, ZNF396, ZNF418, ZNF420, ZNF423, ZNF425, ZNF432, ZNF44, ZNF443, ZNF444, ZNF449, ZNF461,
GO:0000408	EKC/KEOPS complex	CellularComponent	0.00	[GON7, LAGE3, OSGEP, OSGEPL1, TPRKB]
GO:0045095	keratin filament	CellularComponent	0.00	[FBF1, KRT4, KRT80, TCHP]

GO:0031226	intrinsic component of plasma membrane	CellularComponent	0.00	[ABCC11, ABCC8, ABHD6, ACVR2A, ACVRL1, ADAM22, ADCY1, ADCY3, ADCY5, ADGRB1, ADGRD1, ADGRE1, ADGRL1, ADGRL2, ADORA1, ADORA2B, ADRA1B, ADRB2, AGER, AGTR1, AKR1C1, ANKH, ANO1, APH1B, AQP4, AQP7, ASIC3, ASIC4, ATP1A3, ATP2B4, BACE1, C1D, C1QTNF1, C5, C7, CACNA1H, CACNA1I, CACNG4, CACNG7, CACNG8, CALHM2, CCR1, CCR9, CD14, CD22, CD24, CD40, CD47, CD58, CD83, CDH17, CDH4, CDON, CHRFAM7A, CHRNA7, CHRNA9, CHRNB4, CLCN4, CLCN5, CLDN3, CLDN4, CLEC2D, CNIH2, CNIH3, CNTN1, CNTN5, COL13A1, COL17A1, CPT1C, CSF2RA, CXCR5, DCLK1, DLG3, DLL1, DRD1, EDA, EDA2R, EDNRA, EFNA1, EFNA3, EFNA4, EFNB1, EPHA3, EPHA4, EPHA5, EPHA6, EPHA7, EPHB3, EPHB6, EPOR, ERBB3, EREG, ESYT3, EVI2B, FFAR4, FGFR2, FGFR3, FLRT2, FLRT3, FRS2, FUT1, FXYD2, FXYD6, FZD5, GABBR1, GABRE, GAL3ST1, GGT5, GJA1, GJA3, GJB2, GLRB, GPC2, GPC4, GPC6, GPNMB, GPR137B, GPR156, GPR183, GPR19, GPR35, GPR37, GPR52, GPR75, GPRC5C, GRIN1, GRIN2B, HAS2, HAS3, HBEGF, HCN3, HCN4, HFE, HHIP, HLA-DRA, HRH1, HS3ST3B1, HTR1D, HTRA2, ICAM1, ICAM3, IL11RA, IL17RB, IL18R1, IL1R1, IL1RAP, IL27RA, ITGA1, ITGA10, ITGA4, ITGA7, ITGA9, ITGAM, ITGAX, ITGB3, ITGB6, ITGB7, KCNA2, KCNAB3, KCNB1, KCNC3, KCND2, KCNE4, KCNF1, KCNG3, KCNH2, KCNH3, KCNH4, KCNJ11, KCNJ14, KCNJ6, KCNJ8, KCNK3, KCNMB3, KCNMB4, KISS1R, LMBR1L, LPAR4, LPAR6, LRCH4, LRP1, LRRC4B, LRRC70, LRRC8B, LRRC8C, LRRC8D, LTB4R, LTB4R2, LYPD3, MDGA1, MFSD2A, MICA, MIEN1, MME, MMP16, MPP2, MPP3, MUC1, NETO1, NLGN1, NOD2, NOS1AP, NOX5, NOXA1, NPHS1, NPRL2, NPRL3, NRCAM, NRN1, NRXN3, NTRK3, OLFM2, ORAI1, OXTR, P2RX4, P2RX5, P2RX5-TAX1BP3, P2RX6, P2RY1, P2RY11, P2RY6, PCDH1, PCDH10, PCDH18, PCDH7, PCDH9, PCDHA10, PCDHA11, PCDHA12, PCDHA4, PCDHA5, PCDHA6, PCDHA8, PCDHA9, PCDHB11, PCDHB13, PCDHB3, PCDHB5, PCDHB6, PCDHGA1, PCDHGA10, PCDHGA11, PCDHGA4, PCDHGA5, PCDHGA6, PCDHGA8, PCDHGA9, PCDHGB1, PCDHGB2, PCDHGB3, PCDHGB4, PCDHGC3, PGAP6, PILRB, PLPP5, PLPPR1, PLPPR2, PLXNA3, PLXNB3, PMEL, PODXL2, PRNP, PRRG1, PRRG2, PTPRC, PTPRH, RASA3, RCE1, REEP2, RET, RGMB, RNF31, RNF43, ROR2, RTN4RL1, RTN4RL2, RYR1, SCN3B, SCN5A, SCNN1B, SCNN1G, SEMA3B, SEMA3D, SEMA3E, SEMA4G, SEMA6A, SHISA9, SLC12A2, SLC12A3, SLC12A4, SLC15A2, SLC16A10, SLC16A2, SLC16A4, SLC16A6, SLC16A7, SLC1A2, SLC1A3, SLC1A4, SLC22A4, SLC24A4, SLC25A14, SLC25A4, SLC27A1, SLC29A2, SLC2A1, SLC2A12, SLC30A5, SLC35A1, SLC40A1, SLC43A1, SLC4A3, SLC4A4, SLC6A17, SLC6A8, SLC7A7, SLC7A9, SLC8A1, SLCO1B1, SLCO1B7, SLCO2B1, SLCO5A1, SLITRK3, SLITRK6, SMPD2, SNAP25, SORBS1, SORCS2, SPTB, SSPN, ST14, STEAP1, SYNDIG1, SYPL1, SYT13, SYT6, TAS1R1, TENM1, TFR2, THBD, THY1, TLR3, TLR5, TMC1, TMC4, TMC5, TMC6, TMC7, TMC8, TMEM11, TRABD2A, TRAF6, TRPM2, TRPV2, TRPV3, TSPAN1, TSPAN10, TSPAN12, TSPAN13, TSPAN7, TSPAN8, UGT1A1, UGT1A6, ULBP2, UPK1A, VAMP1, VAMP2, VIPR1]
GO:0032991	protein-containing complex	CellularComponent	0.00	[A1CF, ABCA2, ABCB6, ABCC8, ABHD6, ACTR3B, ACTR8, ACVR2A, ACVRL1, ADGRA2, ADRB2, AKR1C1, ALG14, ALX1, ALX4, ALYREF, AMACR, AMN, AMOT, ANAPC10, ANAPC15, ANKRD1, ANKZF1, ANO1, AP1S2, AP4B1, AP5B1, AP5Z1, APBB1, APC2, APH1B, APOE, APOH, APOL1, APOM, APOO, APPBP2, AREG, ARHGAP33, ARL4D, ARL6, ARPC5L, ASB1, ASB4, ATF3, ATF5, ATP1A3, ATP23, ATP2A1, ATP2B4, ATP5F1D, ATP5MGL, ATP6AP1L, ATP6V0A2, ATP6V0A4, ATP6V0E2, ATP6V1B1, ATP6V1C1, ATP6V1F, AURKAIP1, AXIN2, BABAM1, BBS5, BCAS4, BCL2, BCL9, BEX2, BLM, BLOC1S3, BLOC1S4, BLOC1S5, BMF, BMT2, BOD1, BOP1, BORCS7, BORCS8, BRCA2, BTBD8, BTN3A3, BYSL, C12orf66, C15orf48, C1D, C1QL1, C1QL4, C1QTNF1, C1QTNF3, C5, C7, CACHD1, CACNA1H, CACNA1I, CACNB4, CACNG4, CACNG6, CACNG7, CACNG8, CAMKMT, CATSPER2, CATSPERB, CATSPERE, CATSPERG, CBLL1, CBX7, CCDC102A, CCDC12, CCDC120, CCDC130, CCNE1, CCNI2, CCNJ, CCNO, CCZ1B, CD14, CD40, CDH17, CDH3, CDH4, CDH6, CDHR3, CDK14, CDK5R2, CDK5RAP3, CDKN2D, CENPS, CFAP70, CFH, CGNL1, CHD3, CHD5, CHMP1B, CHMP4A, CHMP6, CHRAC1, CHRFAM7A, CHRNA7, CHRNA9, CHRNB4, CHTF18, CIAO2B, CIAO3, CISH, CITED2, CLBA1, CLDN3, CLDN4, CNEP1R1, CNIH2, CNIH3, CNOT3, CNST, CNTN1, COG5, COL11A2, COL12A1, COL13A1, COL16A1, COL17A1, COL1A1, COL26A1, COL28A1, COL4A3, COL4A6, COL5A1, COL6A1, COL9A3, COLQ, COMMD1, COPS6, COPZ2, CORO2A, COX6A1, COX8A, CPS1, CPT1C, CRAT, CREM, CRYAB, CSF2RA, CSNK2B, CSTF2, CTC1, CTHRC1, CXCR4, CXXC1, CYP27B1, DACT1, DCAF10, DCAF12L2, DCAF8, DCP2, DCTN3, DCTN6, DEPDC1, DERL3, DIS3L, DISC1, DLC1, DLG3, DMAC1, DMAC2L, DMD, DNAH1, DNAH11, DNAH12, DNAH17, DNAH2, DNAH6, DNAL4, DNHD1, DNM2, DNM3, DOCK7, DOT1L, DPF1, DPM3, DPYSL3, DSN1, DYNLL2, DYNLT3, E2F6, EAF2, EDA, EEF1E1, EEF2KMT, EEFSEC, EFCAB7, EFNA1, EIF3CL, EIF6, ELL2, ELMO1, ELOF1, EMILIN2, ENC1, ENO4, EPC1, EPHA3, EPHA4, EPHA5, EPHA6, EPHA7, EPHB3, EPHB6, EPN3, EPOP, ERBB3, ERCC2, ERCC8, ERLIN2, ESAM, ETFBKMT, EVC, EVC2, EXOC3L2, EXOC3L4, EXOG, EXOSC1, EYA1, EZH1, F7, FAAP100, FAAP24, FAM98A, FAM98B, FANCB, FANCE, FBF1, FBXL13, FBXL14, FBXL15, FBXL17, FBXL2, FBXL22, FBXL6, FBXO10, FBXO48, FBXW8, FBXW9, FCF1, FEM1C, FGA, FGB, FGFR2, FGFR3, FGL1, FGL2, FKBPL, FN1, FNTB, FOS, FOXE1, FOXF2, FXN, FXYD2, FYB1, GABBR1, GABRE, GATA2, GATA6, GATD3A, GCH1, GCOM1, GCSH, GEMIN2, GEMIN7, GEMIN8, GET4, GFRA1, GJA1, GJA3, GJB2, GJC2, GLI3, GLMN, GLRB, GLUL, GNA13, GNB3, GNG10, GNG2, GNG4, GNGT1, GOLGA7B, GON7, GPD1, GPKOW, GPR156, GPR160, GPR37, GPRC5C, GPSM1, GPSM2, GPX4, GRIN1, GRIN2B, GRPEL2, GSC, GSDMD, GTF2E1, GTF2H2, GTF2H2C, GTF2H4, H1-10, H1-5, H2AC15, H2AZ1, H2BC5, H3-5, H4C12, HAUS2, HAUS4, HAUS5, HAUS7, HCN4, HDAC9, HES1, HFE, HIGD1A, HLA-DMB, HLA-DRA, HLA-F, HMGCL, HNF1A, HOXA10, HOXB13, HOXB9, HPS4, HSPA14, HSPA1A, HSPA1L, HSPG2, HTRA2, HYPK, IFITM2, IFITM3, IFT52, IGFBP3, IGHMBP2, IKZF4, IL11RA, IL18R1, IL27RA, IL3RA, ILK, IMMP1L, IMP3, ING4, INHA, INHBA, INIP, INTS12, INTS5, IRF4, ITGA1, ITGA10, ITGA4, ITGA7, ITGA9, ITGAM, ITGAX, ITGB3, ITGB6, ITGB7, JAZF1, JPH1, JPH2, JRK, KAT2B, KAT5, KAT6B, KBTBD8, KCNA2, KCNAB3, KCNB1, KCNC3, KCND2, KCNE4, KCNF1, KCNG3, KCNH2, KCNH4, KCNJ11, KCNJ14, KCNJ6, KCNJ8, KCNMB3, KCNMB4, KCTD12, KDM4C, KDM5B, KHDRBS3, KHSRP, KIF12, KIF1A, KIF24, KIF26A, KIF5A, KLC4, KLF4, KLHL13, KLHL21, KLHL3, KPNA3, KPNA5, KRT19, KTI12, LAGE3, LAMB4, LAMTOR5, LCP1, LDHD, LEPR, LGALS3, LIN37, LIN7A, LIPC, LMO2, LOX, LRP1, LRP2, LRRC29, LRRC8B, LRRC8C, LRRC8D, LSM5, LSR, LTBP1, LY6G5B, MAD2L2, MAGI1, MAGI2, MALSU1, MALT1, MAP2, MAPT, MARK4, MB, MCUB, MED11, MED16, MED17, MED18, MED23, MED26, MED30, MEF2B, MEGF9, MEIS1, MEPCE, METTL18, MGAM, MIA3, MIB2, MICALL1, MICOS13, MID1, MIER2, MIER3, MITF, MKRN3, MKS1, MLXIPL, MON1A, MON1B, MPHOSPH10, MPHOSPH6, MPP2, MPP3, MPP7, MR1, MRPL1, MRPL12, MRPL14, MRPL18, MRPL2, MRPL23, MRPL27, MRPL28, MRPL30, MRPL33, MRPL38, MRPL39, MRPL49, MRPL50, MRPL53, MRPL54, MRPL57, MTMR9, MVB12B, MXD3, MXI1, MYH15, MYH3, MYO15B, MYO1A, MYO1F, MYO3A, MYO5B, MYO7A, N6AMT1, NAA30, NAE1, NANOS1, NAT9, NCALD, NCK1, NDRG1, NDUFA1, NDUFA7, NDUFB8, NDUFS7, NDUFV2, NECAP2, NFATC1, NFYA, NHLRC1, NKD1, NKX2-1, NKX2-5, NLGN1, NOD2, NOS1AP, NOX5, NOXA1, NPHP3-ACAD11, NPIPA1, NPPA, NPRL2, NPRL3, NR1D1, NR1H4, NR2E3, NR3C2, NR4A1, NR4A2, NR4A3, NRN1, NRXN3, NSUN3, NTRK3, NUP62CL, NUPR1, OCLN, OLFM2, ORAI1, ORC4, ORMDL2, OSGEP, OSGEPL1, OSTC, P2RX6, PABPN1, PAGR1, PAM16, PAN2, PARP11, PAX2, PBX2, PCGF1, PCGF6, PCSK9, PDK2, PDLIM2, PDRG1, PDZD7, PEA15, PELP1, PEX14, PEX3, PFDN6, PGGT1B, PGM5, PHF1, PHF21A, PIGH, PIGM, PIGQ, PIGV, PIH1D2, PIK3CG, PIK3R3, PITX1, PITX2,
GO:1902494	catalytic complex	CellularComponent	0.00	[ACTR8, ACVR2A, ALYREF, ANAPC10, ANAPC15, ASB1, ASB4, C1D, CBLL1, CBX7, CCNE1, CCNI2, CCNJ, CCNO, CDK14, CDK5R2, CDKN2D, CFAP70, CHD3, CHD5, CHRAC1, CISH, CNEP1R1, CNTN1, COMMD1, CXXC1, DCAF10, DCAF12L2, DCAF8, DERL3, DIS3L, DISC1, DMAC1, DNAH1, DNAH11, DNAH12, DNAH17, DNAH2, DNAH6, DNAL4, DNHD1, DPF1, DPM3, DYNLL2, DYNLT3, E2F6, ENC1, ENO4, EPC1, EPOP, ERCC2, ERCC8, EXOSC1, EZH1, F7, FBXL13, FBXL14, FBXL15, FBXL17, FBXL2, FBXL22, FBXL6, FBXO10, FBXO48, FBXW8, FEM1C, FNTB, GCOM1, GCSH, GLMN, GNA13, GNB3, GNG10, GNG2, GNG4, GNGT1, GOLGA7B, GPD1, GPR37, GTF2E1, GTF2H2, GTF2H2C, GTF2H4, HDAC9, HTRA2, ING4, KAT2B, KAT5, KAT6B, KBTBD8, KDM4C, KDM5B, KHSRP, KLHL13, KLHL21, KLHL3, LDHD, LRRC29, MAD2L2, MED11, MED30, MGAM, MIB2, MPHOSPH6, N6AMT1, NAA30, NCK1, NDUFA1, NDUFA7, NDUFB8, NDUFS7, NDUFV2, NKD1, NOX5, NOXA1, NR4A2, ORMDL2, OSTC, PAGR1, PCGF1, PCGF6, PDK2, PELP1, PGGT1B, PHF1, PHF21A, PIGH, PIGM, PIGQ, PIGV, PIK3CG, PIK3R3, POLA1, POLE2, POLR1C, POLR2H, POLR2J, POLR3G, POLR3K, POP5, PPP1R3C, PPP1R3D, PPP2R3A, PPP3R1, PPWD1, PRIM2, PRORP, PRPS2, PSMA1, PSMA2, PSMB10, PSMB8, PSMB9, PSMD12, PSMD9, PYCARD, QPRT, RAG1, RCHY1, RHBDD2, RNF144B, RNF19A, RNF31, RNF8, RPP40, RPRD2, RRM2, RSF1, SATB2, SMARCB1, SNRPB, SNRPF, SNRPN, SOCS2, SORBS1, SPOP, SUMO4, SUPT3H, TADA2A, TAF6L, TAF8, TCTE3, TCTEX1D2,
GO:1990904	ribonucleoprotein complex	CellularComponent	0.00	[ALYREF, BOP1, BTN3A3, BYSL, CCDC12, CCDC130, DCP2, DERL3, EEFSEC, EFNA1, EIF3CL, EIF6, FBXW9, FCF1, FOXE1, GEMIN2, GPKOW, HSPA14, HSPA1A, IGHMBP2, IMP3, JRK, LGALS3, LSM5, MALSU1, MEPCE, MKRN3, MPHOSPH10, MRPL1, MRPL12, MRPL14, MRPL18, MRPL2, MRPL23, MRPL27, MRPL28, MRPL30, MRPL33, MRPL38, MRPL39, MRPL49, MRPL50, MRPL53, MRPL54, MRPL57, NAA30, NDRG1, NHLRC1, NR4A2, NSUN3, PABPN1, PIH1D2, POP5, PPAN, PPP1R8, PPWD1, PRORP, PSMA1, PWP2, RBM14, RBM3, RIOK2, RNF113A, RNF135, RPL36A, RPL39L, RPL9, RPP40, RPS10, RPS26, RPS28, RPP15, SLBP, SNRPB, SNRPF, SNRPN, SOX9, [illegible], TNRC6A, TXNL4A, TXNL4B, U2AF1, U2AF1L4, U2AF1L5, UTP20, ZNHIT3, ZRSR2]
GO:0005882	intermediate filament	CellularComponent	0.00	[DMD, DSP, EIF6, FBF1, IFFO1, IFFO2, KRT10, KRT15, KRT16, KRT19, KRT4, KRT80, KRT9, MICAL1, NARF, NEFL, NES, NME2, PKP2, PPL, SLC1A4, TCHP]
GO:0016020	membrane	CellularComponent	0.00	[A4GALT, AADAC, ABCA2, ABCA7, ABCB1, ABCB6, ABCB9, ABCC11, ABCC6, ABCC8, ABCD3, ABCF3, ABCG1, ABCG4, ABHD13, ABHD6, ACADL, ACAP1, ACE, ACER2, ACKR3, ACP2, ACP3, ACSBG1, ACSL1, ACVR2A, ACVRL1, ADAM11, ADAM12, ADAM19, ADAM22, ADAP1, ADAP2, ADCK2, ADCY1, ADCY3, ADCY5, ADGRA2, ADGRB1, ADGRD1, ADGRE1, ADGRF4, ADGRL1, ADGRL2, ADH1C, ADORA1, ADORA2B, ADRA1B, ADRB2, ADSS1, ADTRP, AFAP1L2, AFG1L, AGAP2, AGER, AGPAT4, AGTR1, AIFM3, AIG1, AJM1, AK9, AKAP5, AKR1C1, ALG1, ALG10, ALG14, ALOX5AP, ALPL, ALYREF, AMACR, AMBP, AMN, AMOT, ANGPT1, ANK2, ANK3, ANKAR, ANKH, ANKRD13A, ANKRD20A1, ANKS1B, ANKZF1, ANO1, ANO10, ANO7, ANO8, ANTXR2, ANXA6, ANXA8L1, ANXA9, AOC2, AOC3, AP1S2, AP4B1, AP5B1, AP5Z1, APBA3, APBB1, APC2, APH1B, APOE, APOL1, APOO, APPBP2, AQP11, AQP4, AQP7, ARAP3, AREG, ARFGAP2, ARFIP1, ARHGAP27, ARHGAP33, ARHGAP44, ARHGAP45, ARHGDIB, ARHGEF4, ARL14, ARL14EP, ARL17B, ARL4D, ARL6, ARL8B, ARMCX6, ARRDC4, ARSA, ARSL, ARV1, ASB3, ASIC3, ASIC4, ASPHD2, ASPSCR1, ASS1, ATAD3A, ATG101, ATP10A, ATP10D, ATP1A3, ATP23, ATP2A1, ATP2A3, ATP2B4, ATP5F1D, ATP5MGL, ATP6AP1L, ATP6V0A2, ATP6V0A4, ATP6V0E2, ATP6V1B1, ATP6V1C1, ATP6V1F, ATP7A, ATP8B3, ATP8B4, ATP9B, ATRNL1, AURKAIP1, B3GALT1, B3GALT4, B3GALT5, B3GAT2, B4GALNT3, B4GALT3, B4GAT1, BACE1, BAIAP2, BAIAP2L1, BAIAP3, BBS5, BCHE, BCL11B, BCL2, BCL2A1, BCL2L12, BCL2L14, BDH1, BLCAP, BMF, BNIP3L, BORCS7, BORCS8, BPHL, BRAF, BRAT1, BRI3BP, BSCL2, BSN, BST1, BTBD8, BTC, BTN3A1, BTN3A3, BTNL9, BYSL, C11orf24, C12orf66, C12orf76, C15orf48, C17orf113, C1D, C1GALT1, C1QTNF1, C1QTNF3, C1orf116, C2CD2, C2orf42, C2orf74, C2orf88, C2orf92, C3orf18, C3orf20, C3orf80, C4A, C4BPA, C5, C7, CA11, CA9, CACFD1, CACHD1, CACNA1H, CACNA1I, CACNB4, CACNG4, CACNG6, CACNG7, CACNG8, CALHM2, CAMKV, CAMLG, CARMIL2, CARMIL3, CASKIN1, CASS4, CATIP, CATSPER2, CATSPERB, CATSPERE, CATSPERG, CAVIN4, CBLB, CC2D1A, CCDC107, CCDC126, CCDC136, CCDC163, CCDC62, CCR1, CCR9, CCZ1B, CD14, CD22, CD24, CD302, CD38, CD40, CD47, CD58, CD83, CDAN1, CDC42EP1, CDC42EP2, CDC42EP3, CDCP1, CDH17, CDH3, CDH4, CDH6, CDHR3, CDK14, CDK5R2, CDK5RAP3, CDON, CDS1, CEACAM6, CEMIP, CEP112, CFAP410, CFH, CGRRF1, CHD5, CHIC2, CHMP1B, CHMP4A, CHMP6, CHRFAM7A, CHRNA7, CHRNA9, CHRNB4, CHST10, CHST13, CHST14, CHST6, CHSY3, CHTF18, CIB2, CIBAR1, CISH, CKLF, CLBA1, CLCN4, CLCN5, CLCN6, CLCN7, CLDN15, CLDN2, CLDN23, CLDN3, CLDN4, CLEC16A, CLEC2D, CLIP3, CLMP, CLVS1, CLYBL, CMTM7, CMTM8, CNEP1R1, CNFN, CNIH2, CNIH3, CNKSR2, CNRIP1, CNST, CNTN1, CNTN5, COG5, COL13A1, COL17A1, COL26A1, COL6A1, COLQ, COMMD1, COPZ2, COQ10A, COQ7, CORO2B, COX6A1, COX7A1, COX8A, CP, CPM, CPNE4, CPPED1, CPS1, CPT1C, CRAT, CREB3, CREB3L1, CRELD1, CRYAB, CSF2RA, CSNK2B, CTNNA2, CTNNAL1, CTNND2, CTSV, CTSW, CTXN1, CUBN, CXCR4, CXCR5, CYBC1, CYP1A1, CYP1B1, CYP26A1, CYP27A1, CYP27B1, CYP27C1, CYP39A1, CYP4F12, CYP4F22, CYRIA, DAAM1, DCLK1, DCTN3, DDN, DDX47, DDX51, DECR2, DEFB1, DEGS2, DERL3, DGAT1, DGAT2, DGKE, DGKG, DGKQ, DHCR7, DHFR2, DHODH, DHRS4, DIO2, DIS3L, DISP1, DKK1, DLC1, DLEC1, DLG3, DLK2, DLL1, DMAC1, DMAC2L, DMD, DMPK, DMTN, DNAAF3, DNAAF4, DNAJA1, DNAJB11, DNAJC1, DNAJC3, DNAJC30, DNAL4, DNER, DNM2, DNM3, DOC2A, DOK7, DOLPP1, DPM3, DRD1, DSC2, DSC3, DSCAML1, DSP, DXO, DYNLL2, EDA, EDA2R, EDAR, EDNRA, EEF1E1, EFCAB7, EFNA1, EFNA3, EFNA4, EFNB1, EHD2, EHHADH, EIF6, ELFN2, ELMO1, ELOVL4, ELOVL7, ENDOG, ENDOU, ENOX1, ENOX2, EPC1, EPCAM, EPHA3, EPHA4, EPHA5, EPHA6, EPHA7, EPHB3, EPHB6, EPHX4, EPN3, EPOR, ERAP1, ERAP2, ERBB3, ERCC6L, EREG, ERLIN2, ERO1B, ESAM, ESYT3, EVA1A, EVA1B, EVC, EVC2, EVI2B, EXOG, EXT1, F12, F7, FA2H, FAAH, FAAH2, FABP5, FABP6, FAM126B, FAM155B, FAM169A, FAM171A2, FAM174A, FAM210B, FAM234A, FAM72D, FAT3, FAXDC2, FBXL15, FBXL2, FCGRT, FCHO2, FCRLB, FERMT3, FFAR4, FGA, FGB, FGFR2, FGFR3, FHIT, FIBCD1, FITM2, FKBP11, FLCN, FLRT2, FLRT3, FLVCR2, FMN2, FMNL1, FN1, FNDC5, FOS, FOSL1, FRMD5, FRMD6, FRS2, FSCN3, FSD1, FUT1, FUT8, FXYD2, FXYD6, FYB1, FZD5, FZD9, GAB2, GABBR1, GABRE, GAL3ST1, GAL3ST2, GALNT16, GALNT17, GALNT5, GALNT9, GAREM1, GAS7, GATA6, GBA2, GBP3, GBP4, GCAT, GCC1, GCH1, GCNT1, GCOM1, GFRA1, GGT5, GIPR, GJA1, GJA3, GJB2, GJC2, GLI2, GLIPR1, GLIPR2, GLMN, GLMP, GLP2R, GLRB, GLUL, GMIP, GNA13, GNB3, GNG10, GNG2, GNG4, GNGT1, GNRH1, GOLGA7B, GOLGA8B, GOLGA8H, GOLGA8K, GOLGA8M, GOLGA8R, GORASP1, GPC2, GPC4, GPC6, GPHN, GPLD1, GPNMB, GPR108, GPR132, GPR137, GPR137B, GPR137C, GPR153, GPR155, GPR156, GPR160, GPR162, GPR179, GPR182, GPR183, GPR19, GPR21, GPR27, GPR35, GPR37, GPR52, GPR75, GPR85,
GO:0022626	cytosolic ribosome	CellularComponent	0.00	[MRPL23, RPL36A, RPL39L, RPL9, RPS10, RPS26, RPS28]
GO:0042995	cell projection	CellularComponent	0.00	[ABCA7, ABHD13, ABITRAM, ABLIM3, ACAP3, ACP3, ACTR3B, ACVRL1, ADAM22, ADCY3, ADCY5, ADGRA2, ADGRB1, ADGRL1, ADGRL2, ADORA1, AFAP1L1, AJM1, AK7, AK8, AKAP3, AKAP5, AMN, AMOT, ANGPT1, ANK2, ANK3, ANKS1B, AOC3, APBA3, APBB1, APC2, APOE, AQP11, AQP4, ARAP3, ARHGAP33, ARHGAP44, ARHGAP45, ARHGEF4, ARL4D, ARL6, ARL8B, ASS1, ATP1A3, ATP2B4, ATP6V0A4, ATP6V1B1, ATP7A, AUTS2, BACE1, BAIAP2, BBS10, BBS12, BBS5, BCL11B, BLOC1S3, BLOC1S4, BLOC1S5, BRAF, BRINP3, BSN, BST1, BTBD8, C1D, C1QL1, C4A, CA9, CABCOCO1, CABYR, CACNA1H, CACNA1I, CACNG7, CARMIL2, CATSPER2, CATSPERB, CATSPERE, CATSPERG, CCDC120, CCDC181, CCK, CCSAP, CD302, CD40, CDHR3, CDK5R2, CEP162, CEP295NL, CEP43, CFAP298, CFAP300, CFAP410, CFAP43, CFAP58, CFAP70, CHAMP1, CHRFAM7A, CHRNA7, CHRNA9, CHRNB4, CIB2, CIBAR1, CKB, CNIH2, CNIH3, CNKSR2, CPT1C, CRMP1, CRYAB, CSNK2B, CTNNA2, CTNND2, CTSV, CUBN, CXXC1, DAAM1, DCDC2, DDN, DEFB1, DISC1, DLC1, DLEC1, DLG3, DMD, DMTN, DNAAF3, DNAAF4, DNAH1, DNAH11, DNAH12, DNAH17, DNAH2, DNAH6, DNAJB13, DNAL4, DNER, DNHD1, DNM2, DNM3, DOC2A, DOCK7, DPYSL3, DRD1, DSCAML1, DYNLL2, EFCAB7, EFHC1, ELK1, ENKUR, EPHA3, EPHA4, EPHA5, EPHA6, EPHA7, EPHB3, EVC, EVC2, FAM161B, FANK1, FBF1, FBXL13, FBXL15, FERMT3, FFAR4, FGD4, FHDC1, FKBPL, FLACC1, FLCN, FLRT2, FLRT3, FMN2, FMNL1, FOS, FOSL1, FOXA1, FRMD4B, FSCN3, FSIP2, FUZ, FZD5, FZD9, GABBR1, GABRE, GAS8, GCH1, GJB2, GJC2, GLI1, GLI2, GLI3, GLMN, GLRB, GLRX5, GLUL, GNA13, GNB3, GNGT1, GNRH1, GPHN, GPNMB, GPR179, GPR19, GRB7, GRIN1, GRIN2B, GRIP1, GRIP2, GRK4, GUCA1B, HAP1, HCN3, HHIP, HNF1A, HOMER1, HRH1, HTR1D, HYAL3, HYLS1, IFT52, IGHMBP2, ILK, INHA, INPP5E, IQCD, IQUB, ITGA1, ITGA4, ITGB3, ITPKA, JCAD, KCNA2, KCNB1, KCNC3, KCND2, KCNH4, KCNJ11, KCNJ14, KCNN3, KCTD12, KIF1A, KIF5A, KIRREL3, KISS1R, L1CAM, LAYN, LCA5, LCP1, LDHD, LHFPL4, LMTK3, LRGUK, LRIG2, LRP1, LRP2, LRRC4B, LRRC6, LYNX1, LYPD6, MACIR, MAGEE1, MAGI1, MAGI2, MAK, MAP2, MAP2K4, MAP3K12, MAPK10, MAPT, MARK4, MAST1, MB, MICAL1, MKS1, MME, MOB2, MOB4, MORN4, MPP2, MPP3, MRI1, MTM1, MTMR9, MTSS1, MUC1, MUC20, MYO1A, MYO1F, MYO3A, MYO7A, MYPN, N4BP3, NANOS1, NCDN, NDRG2, NDUFS7, NECAB2, NEFL, NEK4, NEK8, NGEF, NHLRC1, NHS, NLGN1, NMB, NME2, NMNAT3, NMU, NPHP1, NPHP3, NPHP3-ACAD11, NPHS1, NPPA, NPTX1, NR1D1, NR4A2, NRCAM, NRGN, NTRK3, NUBP1, OLFM1, OPHN1, OSBP2, OXCT2, OXTR, P2RX4, P2RX6, P2RY1, PALMD, PCDH9, PCDHA4, PCDHA6, PCDHB13, PCDHGB1, PCLO, PCSK1, PDE1C, PDE6D, PDE9A, PDZD7, PGAM4, PIFO, PIP5KL1, PKD1L1, PLCB4, PLCE1, PLEK2, PLEKHG5, PLEKHG6, PLK2, PPP1R9A, PQBP1, PRKAA2, PRKAR2B, PRKCG, PRKCZ, PRNP, PROC, PRSS12, PTCH1, PTGS1, PTGS2, PTK6, PTPN9, PTPRC, PTPRH, PXDN, RAB27B, RAB3A, RABEP2, RAP1GAP, RAP1GAP2, RBM3, REEP6, RET, RGS10, RGS9BP, RHOU, RIC3, RILPL2, RIMS2, RINL, RNF38, ROGDI, ROR2, RPGRIP1, RPGRIP1L, RPH3A, RPTOR, RSPH1, RSPH3, RTN4RL1, RTN4RL2, S100P, SCIN, SCYL3, SDC3, SEMA6A, SH3YL1, SHISA9, SIPA1L1, SLC12A2, SLC17A8, SLC1A2, SLC1A3, SLC1A4, SLC4A10, SLC6A17, SLC6A8, SLC7A9, SLC8A1, SMOX, SNAP25, SNAPIN, SNCA, SNCG, SNPH, SORBS2, SORCS2, SPACA9, SPAST, SPATA13, SPATA4, SPATA7, SPEF1, SPEF2, SPOCK1, SPRY4, SPTBN4, SRCIN1, SSX2IP, STAR, STK19, STMN3, STMN4, STON2, STXBP1, SYNDIG1, SYP, SYT13, SYTL1, TANC1, TAPT1, TAS2R4, TBC1D24, TCHP, TCTEX1D2, TDO2, TEDC1, TEKT3, TENM1, TESC, THSD7A, THY1, TIAM1, TIAM2, TMC1, TMEM17, TMEM237, TMEM80, TNFRSF12A, TPGS1, TPRN, TRAK2, TRAPPC4, TRIM46, TRIM9, TRPM2, TRPM6, TRPV2, TSHZ3, TSSK4, TTC21A, TTC30A, TTC30B, TTLL1, TTLL11, TTLL3, TTLL6, TTYH1, TUBA3D, TUBD1, TUBG1, TXNDC15, UCN, UNC13B, UNC13C, USH2A, USP50, UTRN, VAMP1, VAMP2, VAMP7, VCAN, WASF3, WASHC1, WDR47, WDR66, WDR78, WHRN, WIPF1, ZFYVE27, ZMYND8, ZNF804A]

GO:0120025	plasma membrane bounded cell projection	CellularComponent	0.00	[ABCA7, ABHD13, ABITRAM, ABLIM3, ACAP3, ACP3, ACVRL1, ADAM22, ADCY3, ADCY5, ADGRA2, ADGRB1, ADGRL1, ADGRL2, ADORA1, AFAP1L1, AJM1, AK7, AK8, AKAP3, AKAP5, AMN, AMOT, ANGPT1, ANK2, ANK3, ANKS1B, AOC3, APBA3, APBB1, APC2, APOE, AQP11, AQP4, ARAP3, ARHGAP33, ARHGAP44, ARHGAP45, ARHGEF4, ARL4D, ARL6, ARL8B, ASS1, ATP1A3, ATP2B4, ATP6V0A4, ATP6V1B1, ATP7A, AUTS2, BACE1, BAIAP2, BBS10, BBS12, BBS5, BCL11B, BLOC1S3, BLOC1S4, BLOC1S5, BRAF, BRINP3, BSN, BST1, BTBD8, C1D, C1QL1, C4A, CA9, CABCOCO1, CABYR, CACNA1H, CACNA1I, CACNG7, CARMIL2, CATSPER2, CATSPERB, CATSPERE, CATSPERG, CCDC120, CCDC181, CCK, CCSAP, CD302, CD40, CDHR3, CDK5R2, CEP162, CEP295NL, CFAP298, CFAP300, CFAP410, CFAP43, CFAP58, CFAP70, CHRFAM7A, CHRNA7, CHRNA9, CHRNB4, CIB2, CIBAR1, CKB, CNIH2, CNIH3, CNKSR2, CPT1C, CRMP1, CRYAB, CSNK2B, CTNNA2, CTNND2, CTSV, CUBN, DAAM1, DCDC2, DDN, DEFB1, DISC1, DLC1, DLEC1, DLG3, DMD, DMTN, DNAAF3, DNAAF4, DNAH1, DNAH11, DNAH12, DNAH17, DNAH2, DNAH6, DNAJB13, DNAL4, DNER, DNHD1, DNM2, DNM3, DOC2A, DOCK7, DPYSL3, DRD1, DSCAML1, DYNLL2, EFCAB7, EFHC1, ELK1, ENKUR, EPHA3, EPHA4, EPHA5, EPHA6, EPHA7, EPHB3, EVC, EVC2, FAM161B, FANK1, FBF1, FBXL13, FBXL15, FFAR4, FGD4, FHDC1, FKBPL, FLACC1, FLCN, FLRT2, FLRT3, FMN2, FMNL1, FOS, FOSL1, FOXA1, FRMD4B, FSCN3, FSIP2, FZD5, FZD9, GABBR1, GABRE, GAS8, GCH1, GJB2, GJC2, GLI1, GLI2, GLI3, GLMN, GLRB, GLRX5, GLUL, GNA13, GNB3, GNGT1, GNRH1, GPHN, GPNMB, GPR179, GPR19, GRIN1, GRIN2B, GRIP1, GRIP2, GRK4, GUCA1B, HAP1, HCN3, HHIP, HNF1A, HOMER1, HRH1, HTR1D, HYAL3, HYLS1, IFT52, IGHMBP2, ILK, INHA, INPP5E, IQCD, IQUB, ITGA1, ITGA4, ITGB3, ITPKA, JCAD, KCNA2, KCNB1, KCNC3, KCND2, KCNH4, KCNJ11, KCNJ14, KCNN3, KIF1A, KIF5A, KIRREL3, KISS1R, L1CAM, LAYN, LCA5, LCP1, LDHD, LHFPL4, LMTK3, LRIG2, LRP1, LRP2, LRRC4B, LRRC6, LYNX1, LYPD6, MACIR, MAGEE1, MAGI2, MAK, MAP2, MAP2K4, MAP3K12, MAPK10, MAPT, MARK4, MAST1, MB, MICAL1, MKS1, MME, MOB2, MOB4, MORN4, MPP2, MPP3, MTM1, MTMR9, MTSS1, MUC1, MUC20, MYO1A, MYO1F, MYO3A, MYO7A, MYPN, N4BP3, NANOS1, NCDN, NDRG2, NDUFS7, NECAB2, NEFL, NEK4, NEK8, NGEF, NHLRC1, NHS, NLGN1, NMB, NME2, NMNAT3, NMU, NPHP1, NPHP3, NPHP3-ACAD11, NPTX1, NR1D1, NR4A2, NRCAM, NRGN, NTRK3, NUBP1, OLFM1, OPHN1, OSBP2, OXCT2, OXTR, P2RX4, P2RX6, P2RY1, PALMD, PCDH9, PCDHA4, PCDHA6, PCDHB13, PCDHGB1, PCLO, PCSK1, PDE1C, PDE6D, PDE9A, PDZD7, PGAM4, PIFO, PKD1L1, PLCB4, PLCE1, PLEK2, PLEKHG5, PLEKHG6, PLK2, PPP1R9A, PQBP1, PRKAA2, PRKAR2B, PRKCG, PRKCZ, PRNP, PROC, PRSS12, PTCH1, PTGS1, PTGS2, PTK6, PTPN9, PTPRC, PTPRH, PXDN, RAB27B, RAB3A, RABEP2, RAP1GAP, RAP1GAP2, RBM3, REEP6, RET, RGS10, RGS9BP, RIC3, RILPL2, RINL, RNF38, ROGDI, ROR2, RPGRIP1, RPGRIP1L, RPH3A, RPTOR, RSPH1, RSPH3, RTN4RL2, S100P, SCYL3, SDC3, SEMA6A, SH3YL1, SHISA9, SIPA1L1, SLC12A2, SLC17A8, SLC1A2, SLC1A3, SLC1A4, SLC4A10, SLC6A8, SLC7A9, SLC8A1, SMOX, SNAP25, SNAPIN, SNCA, SNCG, SNPH, SORBS2, SORCS2, SPACA9, SPAST, SPATA13, SPATA4, SPATA7, SPEF1, SPEF2, SPOCK1, SPRY4, SPTBN4, SRCIN1, SSX2IP, STAR, STK19, STMN3, STMN4, STON2, STXBP1, SYNDIG1, SYP, SYT13, SYTL1, TANC1, TAPT1, TAS2R4, TBC1D24, TCHP, TCTEX1D2, TDO2, TEDC1, TEKT3, TENM1, TESC, THY1, TIAM1, TIAM2, TMC1, TMEM17, TMEM237,
GO:0005576	extracellular region	CellularComponent	0.00	[ABCB1, ABCB6, ABCC11, ACE, ACOT11, ACP2, ACP3, ACTR3B, ADAM11, ADAM12, ADAM19, ADAMTS15, ADAMTS3, ADAMTS6, ADAMTS8, ADAMTSL5, ADCY1, ADGRB1, ADH6, ADM, ADM2, AGAP2, AGER, AGR2, AHSG, AKR1C1, AKR1C4, ALDH1L2, ALDH2, ALDH8A1, ALDOC, ALPL, ALYREF, AMBP, AMN, AMPD3, ANGPT1, ANGPTL1, ANGPTL4, ANO1, ANTXR2, ANXA6, ANXA8L1, ANXA9, AOX1, APC2, APOE, APOH, APOL1, APOM, APOO, AQP4, AREG, ARHGAP45, ARHGDIB, ARL4D, ARL6, ARL8B, ARMC3, ARPC5L, ARRDC4, ARSA, ARSL, ASL, ASS1, ATP1A3, ATP2B4, ATP6V0A4, ATP6V1B1, ATP6V1C1, ATP6V1F, B4GALT3, B4GAT1, BAIAP2, BAIAP2L1, BCAS1, BCHE, BDH2, BMPER, BPNT1, BRINP3, BST1, BTC, BTD, C11orf45, C11orf54, C12orf49, C12orf66, C17orf58, C17orf67, C1QL1, C1QL4, C1QTNF1, C1QTNF3, C1QTNF4, C1orf116, C2, C2orf69, C4A, C4BPA, C5, C5orf38, C6orf58, C7, C9orf47, CA11, CABYR, CAPN5, CC2D1A, CCDC126, CCDC3, CCK, CCL2, CCL26, CCL5, CCN5, CD14, CD22, CD38, CD40, CD47, CD58, CDC7, CDCP1, CDH17, CDHR3, CDON, CEACAM6, CEMIP, CES1, CES3, CES4A, CFAP58, CFAP70, CFD, CFH, CFHR3, CHAD, CHAMP1, CHGB, CHMP1B, CHMP4A, CHMP6, CHST14, CIB2, CKB, CKLF, CLEC11A, CMTM7, CMTM8, CNKSR2, CNTN1, CNTN5, COL11A2, COL12A1, COL13A1, COL16A1, COL17A1, COL1A1, COL26A1, COL28A1, COL4A3, COL4A6, COL5A1, COL6A1, COL9A3, COLQ, CP, CPA4, CPM, CPNE4, CPPED1, CPQ, CPVL, CRAT, CREG2, CRELD1, CRISPLD2, CRYAB, CSF2RA, CSNK2B, CST9, CTDSPL, CTHRC1, CTSH, CTSO, CTSV, CTSW, CUBN, CXCL1, CXCL2, CXCL3, CXCL5, CXCL8, CXCR4, CXXC1, CYSRT1, DDAH1, DDAH2, DDT, DDTL, DEFB1, DGCR6L, DHRS11, DHRSX, DKK1, DKKL1, DLC1, DLG3, DLL1, DMKN, DNAAF4, DNAH1, DNAH11, DNAJA1, DNAJC3, DNASE1, DNASE1L2, DNHD1, DNM2, DNM3, DNPEP, DPP7, DPYSL3, DSC2, DSC3, DSCAML1, DSN1, DSP, DUSP26, DUSP28, ECM1, EDA, EDIL3, EDN2, EFNA1, EFNA4, EFNB1, EHD2, EHHADH, EIF6, ELFN2, EMILIN2, EML5, ENC1, ENDOU, ENOX1, ENOX2, EPCAM, EPHA3, EPHB3, EPHB6, EPN3, EPOR, ERAP1, ERBB3, EREG, F12, F13B, F7, FABP5, FAM184A, FAM234A, FAM3C, FBLN5, FCGBP, FCGRT, FERMT3, FGA, FGB, FGF12, FGFR2, FGFR3, FGL1, FGL2, FIBCD1, FKBPL, FLRT2, FLRT3, FMNL1, FN1, FNDC5, FRMD4B, FST, FSTL4, FSTL5, FUT8, FUZ, FXYD2, GABBR1, GALNS, GAS8, GBP4, GCNT1, GFRA1, GGACT, GGCT, GLB1L, GLB1L2, GLIPR1, GLIPR2, GLMN, GLUL, GMPPA, GNA13, GNB3, GNG2, GNG4, GNRH1, GPC2, GPC4, GPC6, GPD1, GPLD1, GPNMB, GPR155, GPRC5C, GPX3, GPX4, GREB1, GREM1, GREM2, GRIN1, GSDMD, GSTM4, GTPBP2, H2AC15, H2AZ1, H2BC5, H4C12, HAS3, HBEGF, HDHD2, HFE, HGD, HHIP, HHIPL2, HID1, HLA-DRA, HLA-F, HMOX1, HPD, HPSE, HSPA1A, HSPA1L, HSPG2, HTRA4, HYAL1, HYAL3, ICAM1, ICAM3, IDUA, IFNE, IGFBP2, IGFBP3, IGIP, IGSF1, IGSF10, IL11, IL15RA, IL17RB, IL18BP, IL1R1, IL1RAP, IL32, IL36B, IL4I1, IL7, IL7R, INHA, INHBA, INHBE, ISM1, ISM2, ITGA1, ITGA4, ITGAM, ITGB3, ITGB7, ITIH2, ITIH5, KAZALD1, KCP, KHK, KHSRP, KIF12, KIRREL3, KLHDC8B, KMO, KRT10, KRT15, KRT16, KRT19, KRT9, L1CAM, LAG3, LAMB4, LAP3, LCP1, LEFTY1, LEPR, LFNG, LGALS3, LGALS9, LGALSL, LIN7A, LIPC, LIPH, LOX, LOXL3, LRCH4, LRIG1, LRIG2, LRP2, LRRC24, LRRC6, LSR, LTBP1, LTBP2, LXN, LY6G5B, LY6K, LYG1, LYPD3, LYPD6, LYPLA2, MAL2, MAN1A1, MAN2B1, MANF, MAPT, MATN2, MATN3, MB, MCAM, MCF2L, MDGA1, MEGF6, MEGF9, MEST, MGAM, MICA, MICAL1, MICB, MID2, MIF, MME, MMP11, MMP16, MMP25, MMP28, MMP3, MOXD1, MPHOSPH6, MPP3, MR1, MRPL18, MRPL23, MSMP, MTMR11, MUC1, MUC16, MUC20, MUC3A, MUC5AC, MUC5B, MVB12B, MYH3, MYO5B, NAGA, NAPRT, NDNF, NDRG1, NDRG2, NEB, NETO1, NEU1, NHLRC3, NIBAN1, NINL, NKX6-1, NLGN1, NMB, NME2, NMU, NOG, NOTUM, NPB, NPHP3, NPHP3-ACAD11, NPHS1, NPPA, NPRL3, NPW, NRCAM, NRG2, NRG4, NRN1, NUCB1, NUDT1, NXPH3, NXPH4, OLFM1, OLFM2, OLFML1, OLFML2B, OLFML3, OSBPL1A, OSCAR, P2RX4, PADI2, PCDHA10, PCDHA6, PCIF1, PCLO, PCOLCE, PCSK1, PCSK1N, PCSK5, PCSK9, PCYOX1L, PDZD7, PGAM4, PGAP6, PGC, PLA2G15, PLA2G6, PLAC8, PLAT, PLCB1, PLPPR2, PMEL, PODNL1, PON1, PPFIA3, PPIC, PPL, PPM1L, PPP1R13L, PPT2, PRADC1, PRDX4, PRG4, PRH1, PRKAR2B, PRKCH, PRKCZ, PRNP, PROC, PRR4, PRRG1, PRRG2, PRSS12, PRSS54, PRXL2B, PSMA1, PSMA2, PSMB8, PSMB9, PSMD12, PSPN, PTER, PTGS1, PTH2, PTHLH, PTPRC, PXDN, PYCARD, PYGL, QPCT, QPRT, RAB11FIP3, RAB27B, RAB33B, RAB3A, RAB43, RARRES1, RASSF9, RECK, RELL2, REXO5, RFTN1, RIMS2, RNASET2, RPS26, RPS28, RS1, RSPO3, RTN4RL1, RTN4RL2, RYR1, S100A16, S100P, SCG5, SCGB1A1, SCIN, SCNN1B, SCNN1G, SCPEP1, SDC2, SDC3, SELENBP1, SEMA3B, SEMA3D, SEMA3E, SEMA4G, SEMA6A, SERAC1, SERINC2, SERPINA1, SERPINA6, SERPINB8, SERPINE2, SERPINI1, SFRP1, SFRP5, SGPP2, SH3BGRL3, SHH, SHROOM2, SLC12A2, SLC12A3, SLC15A2, SLC1A4, SLC27A2, SLC2A1, SLC4A4, SLC5A10, SLC6A14, SLC9A3R2, SLIT1, SLIT2, SLIT3, SMOX, SMPD1, SMPDL3A, SMS,
GO:0005615	extracellular space	CellularComponent	0.00	[ABCB1, ABCB6, ABCC11, ACE, ACOT11, ACP2, ACP3, ACTR3B, ADAMTS15, ADAMTS3, ADCY1, ADGRB1, ADH6, ADM, AGAP2, AGR2, AHSG, AKR1C1, AKR1C4, ALDH1L2, ALDH2, ALDH8A1, ALDOC, ALPL, ALYREF, AMBP, AMN, ANGPT1, ANGPTL1, ANGPTL4, ANO1, ANXA6, ANXA9, AOX1, APOE, APOH, APOL1, APOM, APOO, AREG, ARHGDIB, ARL4D, ARL6, ARL8B, ARMC3, ARPC5L, ARSA, ARSL, ASL, ASS1, ATP1A3, ATP2B4, ATP6V0A4, ATP6V1B1, ATP6V1C1, ATP6V1F, B4GALT3, B4GAT1, BAIAP2, BAIAP2L1, BCAS1, BCHE, BDH2, BMPER, BPNT1, BST1, BTC, BTD, C11orf54, C1QL4, C1QTNF1, C1QTNF3, C1QTNF4, C1orf116, C2, C4A, C4BPA, C5, C6orf58, C7, CAPN5, CC2D1A, CCK, CCL2, CCL26, CCL5, CCN5, CD14, CD22, CD38, CD40, CD47, CD58, CDH17, CEACAM6, CES1, CES3, CES4A, CFAP58, CFAP70, CFD, CFH, CFHR3, CHAD, CHAMP1, CHGB, CHMP1B, CHMP4A, CHMP6, CHST14, CIB2, CKB, CKLF, CLEC11A, CMTM7, CMTM8, CNKSR2, CNTN1, COL11A2, COL12A1, COL13A1, COL16A1, COL17A1, COL1A1, COL28A1, COL4A3, COL4A6, COL5A1, COL6A1, COL9A3, COLQ, CP, CPA4, CPM, CPNE4, CPQ, CPVL, CRAT, CREG2, CRISPLD2, CRYAB, CSNK2B, CST9, CTDSPL, CTHRC1, CTSH, CTSO, CTSV, CTSW, CUBN, CXCL1, CXCL2, CXCL3, CXCL5, CXCL8, CXCR4, CXXC1, CYSRT1, DDAH1, DDAH2, DDT, DDTL, DEFB1, DKK1, DKKL1, DLC1, DLG3, DMKN, DNAJA1, DNAJC3, DNASE1, DNHD1, DNM2, DNM3, DNPEP, DPP7, DPYSL3, DSC2, DSC3, DSCAML1, DSP, DUSP26, DUSP28, ECM1, EDIL3, EDN2, EFNB1, EHD2, EHHADH, EIF6, ELFN2, EML5, ENC1, ENDOU, ENOX1, ENOX2, EPCAM, EPN3, ERAP1, ERBB3, EREG, F12, F7, FABP5, FAM184A, FAM234A, FAM3C, FBLN5, FCGBP, FCGRT, FERMT3, FGA, FGB, FGF12, FGL1, FGL2, FIBCD1, FKBPL, FLRT2, FLRT3, FMNL1, FN1, FRMD4B, FST, FUT8, FUZ, FXYD2, GALNS, GCNT1, GFRA1, GGACT, GGCT, GLIPR1, GLIPR2, GLMN, GLUL, GMPPA, GNA13, GNB3, GNG2, GNG4, GNRH1, GPC2, GPC4, GPC6, GPD1, GPLD1, GPR155, GPRC5C, GPX3, GPX4, GREB1, GREM1, GREM2, GSDMD, H2AC15, H2AZ1, H2BC5, H4C12, HBEGF, HDHD2, HFE, HGD, HID1, HLA-DRA, HLA-F, HMOX1, HPD, HSPA1A, HSPA1L, HSPG2, HYAL1, ICAM1, ICAM3, IDUA, IFNE, IGFBP2, IGFBP3, IL11, IL15RA, IL18BP, IL32, IL36B, IL7, INHA, INHBA, INHBE, ITGA1, ITGA4, ITGAM, ITGB3, ITGB7, ITIH2, KCP, KHK, KHSRP, KIF12, KMO, KRT10, KRT15, KRT16, KRT19, KRT9, LAP3, LCP1, LEFTY1, LGALS3, LGALS9, LGALSL, LIN7A, LIPC, LIPH, LOX, LOXL3, LRCH4, LRIG1, LRIG2, LRP2, LRRC24, LSR, LTBP2, LXN, LYPD3, LYPLA2, MAL2, MAN1A1, MAN2B1, MANF, MATN2, MATN3, MB, MCAM, MCF2L, MDGA1, MEST, MGAM, MICA, MICB, MID2, MIF, MME, MMP11, MMP16, MMP25, MMP28, MMP3, MOXD1, MPHOSPH6, MPP3, MR1, MRPL18, MRPL23, MSMP, MTMR11, MUC1, MUC16, MUC5AC, MUC5B, MVB12B, MYH3, MYO5B, NAGA, NAPRT, NDRG1, NDRG2, NEB, NEU1, NIBAN1, NKX6-1, NME2, NOG, NPHS1, NPPA, NPRL3, NRG2, NRG4, NRN1, NUCB1, NUDT1, OLFM1, OSBPL1A, OSCAR, P2RX4, PADI2, PCLO, PCOLCE, PCSK1, PCSK1N, PCSK5, PCSK9, PDZD7, PGAM4, PGAP6, PGC, PLA2G15, PLA2G6, PLAT, PLCB1, PMEL, PODNL1, PON1, PPIC, PPL, PPM1L, PPT2, PRDX4, PRH1, PRKAR2B, PRKCH, PRKCZ, PRNP, PROC, PRR4, PRRG2, PRSS54, PRXL2B, PSMA1, PSMA2, PSMB8, PSMB9, PSMD12, PSPN, PTER, PTGS1, PTHLH, PTPRC, PXDN, PYGL, QPCT, QPRT, RAB27B, RAB33B, RAB43, RARRES1, RASSF9, REXO5, RFTN1, RIMS2, RNASET2, RPS26, RPS28, RS1, RTN4RL1, RTN4RL2, RYR1, S100A16, S100P, SCGB1A1, SCIN, SCNN1B, SCNN1G, SCPEP1, SELENBP1, SEMA3B, SEMA3D, SEMA3E, SEMA4G, SEMA6A, SERINC2, SERPINA1, SERPINA6, SERPINB8, SERPINE2, SERPINI1, SFRP1, SFRP5, SH3BGRL3, SHH, SHROOM2, SLC12A2, SLC12A3, SLC15A2, SLC1A4, SLC27A2, SLC2A1, SLC4A4, SLC5A10, SLC6A14, SLC9A3R2, SLIT1, SLIT2, SLIT3, SMOX, SMPD1, SMPDL3A, SMS, SNCA, SNCG, SNX18, SP8, SPAST, SPOCK1, SPON2, SPR, SPTBN2, SPTBN4, SPX, SRPX2, SSPOP, ST14, ST3GAL1, ST3GAL6, STAG3, STC1, STC2, STXBP1, SUCNR1, SULF2, SVEP1, SYPL1, SYTL1, TAB3, TAC3, TAC4, TADA2A, TAF6L, TAFA5, TEKT3, TEX14, TFPI, TFPI2, TGFA, TGFB3, TGM1, THBD, THY1, TIAM2, TIMP1, TKFC, TLE2, TLR3, TMC4, TMC5, TMC6, TMC8, TMEM132A, TMEM98, TNC, TNFAIP6, TNIK, TNXB, TSPAN1, TSPAN8, TSPO, TTC38, TXNDC5, UBA1, UCN2, ULBP2, UPK1A, UTRN, VAMP7, VAMP8, VASH1, VCAN, VEGFB, VGF, VPS37D, VPS4A, VPS50, VTN, WASF3, WNT11, WNT3, WNT9A, XDH, XYLT1, XYLT2, ZNF260, ZP3]
GO:0005622	intracellular	CellularComponent	0.00	[A1CF, A4GALT, AADAC, AASS, ABCA2, ABCA7, ABCB6, ABCB9, ABCC11, ABCC6, ABCC8, ABCD3, ABCG1, ABHD11, ABHD13, ABHD5, ABHD6, ABITRAM, ABLIM2, ABLIM3, ACADL, ACADS, ACAP1, ACE, ACER2, ACKR3, ACOT11, ACOT2, ACOX2, ACP2, ACP3, ACSBG1, ACSL1, ACSS3, ACTL10, ACTR3B, ACTR8, ACTRT3, ACVR2A, ADAM12, ADAM19, ADAP1, ADAP2, ADCY1, ADCY3, ADCY5, ADGRB1, ADH1C, ADH6, ADM, ADORA1, ADRA1B, ADRB2, ADSS1, AFAP1, AFAP1L1, AFAP1L2, AFG1L, AGAP2, AGAP4, AGAP6, AGER, AGPAT4, AGR2, AHRR, AHSG, AIFM3, AIRE, AK7, AK8, AK9, AKAP3, AKAP5, AKR1C1, AKR1C2, AKR1C4, ALDH1A2, ALDH1L2, ALDH2, ALDH4A1, ALDH5A1, ALDH8A1, ALDOC, ALG1, ALG10, ALG14, ALKBH4, ALOX5AP, ALS2CL, ALX1, ALX4, ALYREF, AMACR, AMBP, AMDHD1, AMMECR1, AMN, AMOT, AMPD3, ANAPC10, ANAPC15, ANK2, ANK3, ANKRD1, ANKRD13A, ANKRD2, ANKRD23, ANKRD26, ANKRD37, ANKRD54, ANKS1B, ANKZF1, ANO1, ANO10, ANO7, ANO8, ANTXR2, ANXA6, ANXA8L1, ANXA9, AOC2, AOC3, AOX1, AP1S2, AP4B1, AP5B1, AP5Z1, APBA3, APBB1, APC2, APH1B, APOE, APOH, APOL1, APOO, APPBP2, AQP11, AQP4, AQP7, ARAF, ARAP2, ARAP3, AREG, ARFGAP2, ARFIP1, ARG2, ARHGAP20, ARHGAP22, ARHGAP27, ARHGAP33, ARHGAP39, ARHGAP44, ARHGAP45, ARHGAP8, ARHGDIB, ARHGEF10L, ARHGEF17, ARHGEF19, ARHGEF37, ARHGEF4, ARID3B, ARID5B, ARL14, ARL14EP, ARL17B, ARL4D, ARL5B, ARL5C, ARL6, ARL8B, ARMCX6, ARPC5L, ARRDC4, ARSA, ARSD, ARSL, ARV1, ARX, ASB1, ASB3, ASB4, ASB7, ASB8, ASB9, ASIC3, ASL, ASMT, ASMTL, ASNS, ASPSCR1, ASS1, ATAD3A, ATF3, ATF5, ATF7, ATF7IP2, ATG101, ATG4C, ATP10A, ATP10D, ATP1A3, ATP23, ATP2A1, ATP2A3, ATP2B4, ATP5F1D, ATP5MGL, ATP6V0A2, ATP6V0A4, ATP6V0E2, ATP6V1B1, ATP6V1C1, ATP6V1F, ATP7A, ATP8B3, ATP8B4, ATP9B, AUH, AUNIP, AURKAIP1, AUTS2, AXIN2, B3GALT1, B3GALT4, B3GALT5, B3GAT2, B4GALNT3, B4GALT3, B4GAT1, BABAM1, BACE1, BACH2, BAIAP2, BAIAP2L1, BAIAP3, BANP, BARX1, BBS5, BCAS1, BCAS4, BCHE, BCL11A, BCL11B, BCL2, BCL2A1, BCL2L12, BCL2L14, BCL2L15, BCL6, BCL9, BCO1, BDH1, BDH2, BEND6, BEX2, BEX4, BHLHE41, BIRC7, BLID, BLM, BLOC1S3, BLOC1S4, BLOC1S5, BMERB1, BMF, BMT2, BNIP3L, BNIPL, BOD1, BOLA3, BOP1, BORCS7, BORCS8, BPHL, BPNT1, BRAF, BRAT1, BRCA2, BRD3, BRI3BP, BRINP3, BSCL2, BSN, BST1, BTBD8, BTC, BTD, BTN3A3, BYSL, C11orf24, C11orf54, C12orf66, C15orf48, C18orf32, C1D, C1GALT1, C1QL1, C1QTNF1, C1orf109, C1orf116, C1orf52, C1orf61, C2CD2, C2orf42, C3orf20, C4A, C4orf46, C8orf44, C8orf88, CA8, CA9, CAB39L, CABCOCO1, CABYR, CACNB4, CACNG4, CACNG7, CACNG8, CAMKMT, CAMKV, CAMLG, CAMSAP3, CAPN12, CAPN15, CAPN5, CARF, CARMIL2, CARMIL3, CARNS1, CASKIN1, CASP6, CASS4, CASZ1, CATIP, CAVIN4, CBLB, CBLL1, CBS, CBX7, CC2D1A, CCDC102A, CCDC106, CCDC12, CCDC120, CCDC125, CCDC130, CCDC136, CCDC146, CCDC15, CCDC181, CCDC3, CCDC38, CCDC62, CCDC86, CCDC92, CCDC96, CCL5, CCN5, CCNE1, CCNI2, CCNJ, CCNO, CCR1, CCSAP, CCZ1B, CD14, CD22, CD24, CD302, CD38, CD40, CD47, CD58, CDADC1, CDAN1, CDC25A, CDC42EP1, CDC42EP2, CDC42EP3, CDC7, CDCA5, CDH17, CDH3, CDK14, CDK18, CDK2AP1, CDK5R2, CDK5RAP3, CDKN1C, CDKN2D, CDS1, CEACAM6, CEMIP, CEMP1, CENPL, CENPS, CENPT, CENPV, CEP112, CEP120, CEP162, CEP295NL, CEP43, CEP57L1, CEP72, CEP85L, CES1, CES3, CFAP298, CFAP300, CFAP410, CFAP43, CFAP58, CFAP70, CFD, CFH, CGNL1, CGRRF1, CHAC1, CHAC2, CHAMP1, CHD1, CHD3, CHD5, CHD9, CHGB, CHIC2, CHMP1B, CHMP4A, CHMP6, CHRAC1, CHRNB4, CHST10, CHST13, CHST14, CHST6, CHSY3, CHTF18, CIAO2B, CIAO3, CIAPIN1, CIB2, CIBAR1, CIDEB, CIITA, CISH, CITED2, CITED4, CKB, CLASRP, CLBA1, CLCN4, CLCN5, CLCN6, CLCN7, CLDN3, CLEC11A, CLEC16A, CLEC2D, CLIP3, CLMP, CLSPN, CLVS1, CLYBL, CMTM8, CNEP1R1, CNFN, CNIH2, CNIH3, CNKSR2, CNOT3, CNRIP1, CNST, COA5, COG5, COL11A2, COL12A1, COL13A1, COL16A1, COL17A1, COL1A1, COL26A1, COL28A1, COL4A3, COL4A6, COL5A1, COL6A1, COL9A3, COLCA2, COMMD1, COMMD10, COMMD4, COPS6, COPZ2, COQ10A, COQ7, CORO2B, COX6A1, COX7A1, COX8A, CP, CPA4, CPPED1, CPQ, CPS1, CPT1C, CRAT, CREB3, CREB3L1, CREBRF, CREG2, CREM, CRIP2, CRISPLD2, CRMP1, CRYAB, CSKMT, CSNK2B, CSRNP3, CSRP2, CSTF2, CTC1, CTDSPL, CTHRC1, CTNNA2, CTNNAL1, CTNND2, CTSH, CTSO, CTSV, CTSW, CUBN, CXCL1, CXCR4, CXXC1, CXXC4, CXXC5, CYBC1, CYP1A1, CYP1B1, CYP26A1, CYP27A1, CYP27B1, CYP27C1, CYP39A1, CYP4F12, CYP4F22, CYRIA, DAAM1, DACT1, DBNDD2, DBP, DCAF10, DCAF12L2, DCAF8, DCDC2, DCK, DCLRE1B, DCP2, DCTN3, DCTN6, DDAH1, DDAH2, DDIAS, DDIT4, DDN, DDT, DDTL, DDX47, DDX49, DDX51, DECR2, DEDD, DEFB1, DEGS2, DELE1, DENND6B, DEPDC1, DEPDC7, DERL3, DGAT1, DGAT2, DGCR6L, DGKE, DGKG, DGKQ, DHCR7, DHFR2, DHODH, DHRS1, DHRS2, DHRS4, DHX37, DIS3L, DISC1, DKK1, DKKL1, DLC1, DLEC1, DLG3, DLL1, DLX1, DLX2, DLX4, DMAC1,

GO:0005737	cytoplasm	CellularComponent	0.00	[A1CF, A4GALT, AADAC, AASS, ABCA2, ABCA7, ABCB6, ABCB9, ABCC11, ABCC6, ABCC8, ABCD3, ABCG1, ABHD11, ABHD13, ABHD5, ABHD6, ABLIM2, ABLIM3, ACADL, ACADS, ACAP1, ACE, ACER2, ACKR3, ACOT11, ACOT2, ACOX2, ACP2, ACP3, ACSBG1, ACSL1, ACSS3, ACTR3B, ACTRT3, ACVR2A, ADAM19, ADAP1, ADAP2, ADCY1, ADCY3, ADGRB1, ADH1C, ADH6, ADM, ADORA1, ADRA1B, ADRB2, ADSS1, AFAP1, AFAP1L1, AFAP1L2, AFG1L, AGAP2, AGPAT4, AGR2, AHRR, AHSG, AIFM3, AIRE, AK7, AK8, AK9, AKAP3, AKAP5, AKR1C1, AKR1C2, AKR1C4, ALDH1A2, ALDH1L2, ALDH2, ALDH4A1, ALDH5A1, ALDH8A1, ALDOC, ALG1, ALG10, ALG14, ALKBH4, ALOX5AP, ALS2CL, ALX1, ALYREF, AMACR, AMDHD1, AMMECR1, AMN, AMOT, AMPD3, ANAPC10, ANAPC15, ANK2, ANK3, ANKRD1, ANKRD13A, ANKRD2, ANKRD23, ANKRD37, ANKRD54, ANKS1B, ANKZF1, ANO1, ANO7, ANO8, ANTXR2, ANXA6, ANXA8L1, ANXA9, AOC2, AOC3, AOX1, AP1S2, AP4B1, AP5B1, AP5Z1, APBA3, APBB1, APC2, APH1B, APOE, APOH, APOL1, APOO, APPBP2, AQP11, AQP4, AQP7, ARAF, ARAP2, ARAP3, AREG, ARFGAP2, ARFIP1, ARG2, ARHGAP20, ARHGAP22, ARHGAP27, ARHGAP33, ARHGAP39, ARHGAP44, ARHGAP45, ARHGAP8, ARHGDIB, ARHGEF10L, ARHGEF17, ARHGEF19, ARHGEF37, ARHGEF4, ARL14, ARL14EP, ARL17B, ARL4D, ARL5B, ARL5C, ARL6, ARL8B, ARMCX6, ARPC5L, ARRDC4, ARSA, ARSD, ARSL, ARV1, ASB1, ASB3, ASB4, ASB7, ASB8, ASB9, ASIC3, ASL, ASMT, ASMTL, ASNS, ASPSCR1, ASS1, ATAD3A, ATF5, ATF7, ATG101, ATG4C, ATP10A, ATP10D, ATP1A3, ATP23, ATP2A1, ATP2A3, ATP2B4, ATP5F1D, ATP5MGL, ATP6V0A2, ATP6V0A4, ATP6V0E2, ATP6V1B1, ATP6V1C1, ATP6V1F, ATP7A, ATP8B3, ATP8B4, ATP9B, AUH, AUNIP, AURKAIP1, AUTS2, AXIN2, B3GALT1, B3GALT4, B3GALT5, B3GAT2, B4GALNT3, B4GALT3, B4GAT1, BABAM1, BACE1, BACH2, BAIAP2, BAIAP2L1, BAIAP3, BBS5, BCAS1, BCAS4, BCHE, BCL11A, BCL11B, BCL2, BCL2A1, BCL2L14, BCL2L15, BCL6, BCL9, BCO1, BDH1, BDH2, BEX2, BEX4, BIRC7, BLID, BLM, BLOC1S3, BLOC1S4, BLOC1S5, BMF, BNIP3L, BNIPL, BOD1, BOLA3, BORCS7, BORCS8, BPHL, BPNT1, BRAF, BRAT1, BRCA2, BRI3BP, BRINP3, BSCL2, BSN, BST1, BTBD8, BTC, BTD, BTN3A3, BYSL, C11orf24, C12orf66, C15orf48, C18orf32, C1D, C1GALT1, C1QL1, C1QTNF1, C1orf109, C1orf116, C2CD2, C3orf20, C4A, C4orf46, C8orf88, CA8, CAB39L, CABCOCO1, CABYR, CACNB4, CACNG4, CACNG7, CACNG8, CAMKMT, CAMKV, CAMLG, CAMSAP3, CAPN12, CAPN15, CAPN5, CARMIL2, CARMIL3, CARNS1, CASKIN1, CASP6, CASS4, CASZ1, CATIP, CAVIN4, CBLB, CBLL1, CBS, CBX7, CC2D1A, CCDC106, CCDC120, CCDC125, CCDC136, CCDC146, CCDC181, CCDC3, CCDC38, CCDC62, CCDC92, CCDC96, CCL5, CCNE1, CCNI2, CCNJ, CCNO, CCR1, CCSAP, CCZ1B, CD14, CD22, CD302, CD38, CD40, CD47, CD58, CDADC1, CDAN1, CDC25A, CDC42EP1, CDC42EP2, CDC42EP3, CDC7, CDCA5, CDH3, CDK14, CDK18, CDK2AP1, CDK5R2, CDK5RAP3, CDKN1C, CDKN2D, CDS1, CEACAM6, CEMIP, CEMP1, CENPL, CENPS, CENPT, CENPV, CEP112, CEP120, CEP162, CEP295NL, CEP43, CEP57L1, CEP72, CEP85L, CES1, CES3, CFAP298, CFAP300, CFAP410, CFAP43, CFAP70, CFD, CFH, CGRRF1, CHAC1, CHAC2, CHAMP1, CHD1, CHD3, CHD5, CHD9, CHGB, CHIC2, CHMP1B, CHMP4A, CHMP6, CHRNB4, CHST10, CHST13, CHST14, CHST6, CHSY3, CHTF18, CIAO2B, CIAO3, CIAPIN1, CIB2, CIBAR1, CIDEB, CIITA, CISH, CITED4, CKB, CLBA1, CLCN4, CLCN5, CLCN6, CLCN7, CLDN3, CLEC11A, CLEC16A, CLEC2D, CLIP3, CLMP, CLSPN, CLVS1, CLYBL, CMTM8, CNEP1R1, CNFN, CNIH2, CNIH3, CNKSR2, CNOT3, CNRIP1, CNST, COA5, COG5, COL11A2, COL12A1, COL13A1, COL16A1, COL17A1, COL1A1, COL26A1, COL28A1, COL4A3, COL4A6, COL5A1, COL6A1, COL9A3, COLCA2, COMMD1, COMMD10, COMMD4, COPS6, COPZ2, COQ10A, COQ7, CORO2B, COX6A1, COX7A1, COX8A, CP, CPA4, CPPED1, CPQ, CPS1, CPT1C, CRAT, CREB3, CREB3L1, CREG2, CREM, CRIP2, CRISPLD2, CRMP1, CRYAB, CSKMT, CSNK2B, CSRP2, CTHRC1, CTNNA2, CTNNAL1, CTNND2, CTSH, CTSO, CTSV, CTSW, CUBN, CXCL1, CXCR4, CXXC1, CXXC4, CXXC5, CYBC1, CYP1A1, CYP1B1, CYP26A1, CYP27A1, CYP27B1, CYP27C1, CYP39A1, CYP4F12, CYP4F22, DAAM1, DACT1, DBNDD2, DCAF8, DCDC2, DCK, DCLRE1B, DCP2, DCTN3, DCTN6, DDAH1, DDAH2, DDIAS, DDIT4, DDN, DDT, DDTL, DECR2, DEDD, DEFB1, DEGS2, DELE1, DENND6B, DEPDC7, DERL3, DGAT1, DGAT2, DGKE, DGKG, DGKQ, DHCR7, DHFR2, DHODH, DHRS1, DHRS2, DHRS4, DIS3L, DISC1, DKK1, DKKL1, DLC1, DLEC1, DLG3, DLL1, DMAC1, DMAC2L, DMD, DMPK, DMTN, DNAAF3, DNAAF4, DNAH1, DNAH11, DNAH12, DNAH17, DNAH2, DNAH6, DNAJA1, DNAJB11, DNAJB13, DNAJC1, DNAJC12, DNAJC3, DNAJC30, DNAL4, DNASE1L2, DNER, DNHD1, DNM2, DNM3, DNPEP, DOC2A, DOCK3, DOCK6, DOHH, DOK5, DOK6, DOK7, DOLPP1, DPF1, DPH1, DPM3, DPP7, DPYD, DPYSL3, DRD1, DSC2, DSC3, DSN1, DSP, DTYMK, DUSP19, DUSP26, DUSP28, DUSP7, DUSP8, DXO, DYNLL2, DYNLT3, DYRK3, ECI1, ECM1, EDARADD, EEF1AKMT1, EEF1E1, EEF2KMT, EEFSEC,
GO:0043226	organelle	CellularComponent	0.00	[A1CF, A4GALT, AADAC, AASS, ABCA2, ABCA7, ABCB1, ABCB6, ABCB9, ABCC11, ABCC6, ABCC8, ABCD3, ABCG1, ABHD11, ABHD5, ABHD6, ABITRAM, ABLIM2, ABLIM3, ACADL, ACADS, ACAP1, ACE, ACER2, ACKR3, ACOT11, ACOT2, ACOX2, ACP2, ACP3, ACSBG1, ACSL1, ACSS3, ACTL10, ACTR3B, ACTR8, ACTRT3, ADAM12, ADAM19, ADAM22, ADAMTS3, ADAP1, ADAP2, ADCY1, ADCY3, ADCY5, ADGRB1, ADGRL1, ADH1C, ADH6, ADORA1, ADRA1B, ADRB2, AFAP1, AFAP1L1, AFG1L, AGAP2, AGAP4, AGAP6, AGER, AGPAT4, AGR2, AHRR, AHSG, AIFM3, AIRE, AJM1, AK7, AK8, AK9, AKAP3, AKAP5, AKR1C1, AKR1C4, ALDH1L2, ALDH2, ALDH4A1, ALDH5A1, ALDH8A1, ALDOC, ALG1, ALG10, ALG14, ALKBH4, ALOX5AP, ALPL, ALX1, ALX4, ALYREF, AMACR, AMBP, AMMECR1, AMN, AMOT, AMPD3, ANAPC10, ANAPC15, ANGPT1, ANGPTL1, ANK2, ANK3, ANKRD1, ANKRD13A, ANKRD2, ANKRD23, ANKRD26, ANKRD37, ANKRD54, ANKS1B, ANKZF1, ANO1, ANO10, ANO7, ANO8, ANTXR2, ANXA6, ANXA8L1, ANXA9, AOC3, AOX1, AP1S2, AP4B1, AP5B1, AP5Z1, APBB1, APC2, APH1B, APOE, APOH, APOL1, APOO, APPBP2, AQP11, AQP4, AQP7, ARAF, ARAP3, AREG, ARFGAP2, ARFIP1, ARG2, ARHGAP22, ARHGAP39, ARHGAP44, ARHGAP45, ARHGDIB, ARID3B, ARID5B, ARL14, ARL14EP, ARL17B, ARL4D, ARL5B, ARL5C, ARL6, ARL8B, ARMC3, ARMCX6, ARPC5L, ARRDC4, ARSA, ARSD, ARSL, ARV1, ARX, ASB9, ASL, ASPSCR1, ASS1, ATAD3A, ATF3, ATF5, ATF7, ATF7IP2, ATG101, ATP10A, ATP10D, ATP1A3, ATP23, ATP2A1, ATP2A3, ATP2B4, ATP5F1D, ATP5MGL, ATP6V0A2, ATP6V0A4, ATP6V0E2, ATP6V1B1, ATP6V1C1, ATP6V1F, ATP7A, ATP8B3, ATP8B4, ATP9B, AUH, AUNIP, AURKAIP1, AUTS2, AXIN2, B3GALT1, B3GALT4, B3GALT5, B3GAT2, B4GALNT3, B4GALT3, B4GAT1, BABAM1, BACE1, BACH2, BAIAP2, BAIAP2L1, BAIAP3, BANP, BARX1, BBS10, BBS12, BBS5, BCAS1, BCHE, BCL11A, BCL11B, BCL2, BCL2A1, BCL2L12, BCL2L14, BCL2L15, BCL6, BCL9, BDH1, BDH2, BEND6, BEX2, BEX4, BHLHE41, BIRC7, BLID, BLM, BLOC1S3, BLOC1S5, BMERB1, BMF, BMT2, BNIP3L, BNIPL, BOD1, BOLA3, BOP1, BORCS7, BORCS8, BPHL, BPNT1, BRAF, BRAT1, BRCA2, BRD3, BRI3BP, BRINP3, BSCL2, BSN, BST1, BTBD8, BTC, BTD, BTN3A3, BYSL, C11orf24, C11orf54, C12orf66, C15orf48, C18orf32, C1D, C1GALT1, C1QTNF1, C1QTNF3, C1orf109, C1orf116, C1orf52, C1orf61, C2, C2CD2, C2orf42, C4A, C5, C6orf58, C7, C8orf44, CA9, CABCOCO1, CABYR, CACNG4, CACNG7, CACNG8, CAMKMT, CAMKV, CAMLG, CAMSAP3, CAPN5, CARF, CARMIL2, CASP6, CASS4, CASZ1, CATIP, CATSPER2, CATSPERB, CATSPERE, CATSPERG, CAVIN4, CBLB, CBLL1, CBS, CBX7, CC2D1A, CCDC102A, CCDC106, CCDC12, CCDC120, CCDC130, CCDC136, CCDC146, CCDC15, CCDC181, CCDC3, CCDC38, CCDC62, CCDC86, CCDC92, CCDC96, CCN5, CCNE1, CCNI2, CCNJ, CCNO, CCSAP, CCZ1B, CD14, CD22, CD24, CD38, CD40, CD47, CD58, CDADC1, CDAN1, CDC25A, CDC42EP1, CDC42EP2, CDC42EP3, CDC7, CDCA5, CDH17, CDK14, CDK18, CDK2AP1, CDK5RAP3, CDKN1C, CDKN2D, CDS1, CEACAM6, CEMIP, CEMP1, CENPL, CENPS, CENPT, CENPV, CEP112, CEP120, CEP162, CEP295NL, CEP43, CEP57L1, CEP72, CEP85L, CES1, CES3, CFAP298, CFAP300, CFAP410, CFAP43, CFAP58, CFAP70, CFD, CFH, CFHR3, CGNL1, CGRRF1, CHAC1, CHAMP1, CHD1, CHD3, CHD5, CHD9, CHGB, CHIC2, CHMP1B, CHMP4A, CHMP6, CHRAC1, CHRNA9, CHRNB4, CHST10, CHST13, CHST14, CHST6, CHSY3, CHTF18, CIAO2B, CIAPIN1, CIB2, CIBAR1, CIITA, CITED2, CITED4, CKB, CLASRP, CLBA1, CLCN4, CLCN5, CLCN6, CLCN7, CLEC16A, CLEC2D, CLIP3, CLMP, CLSPN, CLVS1, CLYBL, CMTM8, CNEP1R1, CNIH2, CNIH3, CNKSR2, CNOT3, CNST, CNTN1, COA5, COG5, COL11A2, COL12A1, COL13A1, COL16A1, COL17A1, COL1A1, COL26A1, COL28A1, COL4A3, COL4A6, COL5A1, COL6A1, COL9A3, COMMD1, COMMD10, COMMD4, COPS6, COPZ2, COQ10A, COQ7, CORO2B, COX6A1, COX7A1, COX8A, CP, CPA4, CPM, CPNE4, CPPED1, CPQ, CPS1, CPT1C, CPVL, CRAT, CREB3, CREB3L1, CREBRF, CREG2, CREM, CRISPLD2, CRMP1, CRYAB, CSKMT, CSNK2B, CSRNP3, CSRP2, CSTF2, CTC1, CTDSPL, CTNNA2, CTNNAL1, CTNND2, CTSH, CTSO, CTSV, CTSW, CUBN, CXCL1, CXCR4, CXXC1, CXXC4, CXXC5, CYBC1, CYP1A1, CYP1B1, CYP26A1, CYP27A1, CYP27B1, CYP27C1, CYP39A1, CYP4F12, CYP4F22, CYSRT1, DAAM1, DACT1, DBP, DCAF10, DCAF8, DCDC2, DCK, DCLK1, DCLRE1B, DCP2, DCTN3, DCTN6, DDAH1, DDAH2, DDIAS, DDIT4, DDN, DDT, DDTL, DDX47, DDX49, DDX51, DECR2, DEDD, DEFB1, DEGS2, DELE1, DENND6B, DEPDC1, DERL3, DGAT1, DGAT2, DGCR6L, DGKE, DGKQ, DHCR7, DHFR2, DHODH, DHRS1, DHRS2, DHRS4, DHX37, DIS3L, DISC1, DKK1, DKKL1, DLC1, DLEC1, DLG3, DLL1, DLX1, DLX2, DLX4, DMAC1, DMAC2L, DMC1, DMD, DMPK, DMRTA1, DMRTA2, DMTN, DNAAF3, DNAAF4, DNAH1, DNAH11, DNAH12, DNAH17, DNAH2, DNAH6, DNAJA1, DNAJB11, DNAJB13, DNAJC1, DNAJC27, DNAJC3, DNAJC30, DNAL4, DNASE1, DNASE1L2, DNER, DNHD1, DNM2, DNM3, DNPEP, DOC2A, DOCK7, DOK7, DOLPP1, DOT1L, DPF1, DPH1, DPM3, DPP7,
GO:0043229	intracellular organelle	CellularComponent	0.00	[A1CF, A4GALT, AADAC, AASS, ABCA2, ABCA7, ABCB6, ABCB9, ABCC11, ABCC6, ABCC8, ABCD3, ABCG1, ABHD11, ABHD5, ABHD6, ABITRAM, ABLIM2, ABLIM3, ACADL, ACADS, ACAP1, ACE, ACER2, ACKR3, ACOT11, ACOT2, ACOX2, ACP2, ACP3, ACSBG1, ACSL1, ACSS3, ACTL10, ACTR3B, ACTR8, ACTRT3, ADAM12, ADAM19, ADAP1, ADAP2, ADCY1, ADCY3, ADCY5, ADH1C, ADORA1, ADRA1B, ADRB2, AFAP1, AFAP1L1, AFG1L, AGAP2, AGAP4, AGAP6, AGER, AGPAT4, AGR2, AHRR, AHSG, AIFM3, AIRE, AK8, AK9, AKAP3, AKAP5, ALDH1L2, ALDH2, ALDH4A1, ALDH5A1, ALDOC, ALG1, ALG10, ALG14, ALKBH4, ALOX5AP, ALX1, ALX4, ALYREF, AMACR, AMBP, AMMECR1, AMN, AMOT, AMPD3, ANAPC10, ANAPC15, ANK2, ANK3, ANKRD1, ANKRD13A, ANKRD2, ANKRD23, ANKRD26, ANKRD37, ANKRD54, ANKS1B, ANKZF1, ANO1, ANO10, ANO7, ANO8, ANTXR2, ANXA6, ANXA8L1, ANXA9, AOC3, AP1S2, AP4B1, AP5B1, AP5Z1, APBB1, APC2, APH1B, APOE, APOH, APOL1, APOO, APPBP2, AQP11, AQP4, AQP7, ARAF, ARAP3, AREG, ARFGAP2, ARFIP1, ARG2, ARHGAP22, ARHGAP39, ARHGAP44, ARHGAP45, ARHGDIB, ARID3B, ARID5B, ARL14, ARL14EP, ARL17B, ARL4D, ARL5B, ARL5C, ARL6, ARL8B, ARMCX6, ARPC5L, ARRDC4, ARSA, ARSD, ARSL, ARV1, ARX, ASB9, ASPSCR1, ASS1, ATAD3A, ATF3, ATF5, ATF7, ATF7IP2, ATP10A, ATP10D, ATP1A3, ATP23, ATP2A1, ATP2A3, ATP2B4, ATP5F1D, ATP5MGL, ATP6V0A2, ATP6V0A4, ATP6V0E2, ATP6V1B1, ATP6V1C1, ATP6V1F, ATP7A, ATP8B3, ATP8B4, ATP9B, AUH, AUNIP, AURKAIP1, AUTS2, AXIN2, B3GALT1, B3GALT4, B3GALT5, B3GAT2, B4GALNT3, B4GALT3, B4GAT1, BABAM1, BACE1, BACH2, BAIAP2, BAIAP2L1, BAIAP3, BANP, BARX1, BBS5, BCHE, BCL11A, BCL11B, BCL2, BCL2A1, BCL2L12, BCL2L14, BCL2L15, BCL6, BCL9, BDH1, BEND6, BEX2, BEX4, BHLHE41, BIRC7, BLID, BLM, BLOC1S3, BLOC1S5, BMERB1, BMF, BMT2, BNIP3L, BNIPL, BOD1, BOLA3, BOP1, BORCS7, BORCS8, BPHL, BPNT1, BRAF, BRAT1, BRCA2, BRD3, BRI3BP, BRINP3, BSCL2, BSN, BST1, BTBD8, BTC, BTD, BTN3A3, BYSL, C11orf24, C11orf54, C12orf66, C15orf48, C18orf32, C1D, C1GALT1, C1QTNF1, C1orf109, C1orf52, C1orf61, C2CD2, C2orf42, C4A, C8orf44, CA9, CABCOCO1, CABYR, CACNG4, CACNG7, CACNG8, CAMKMT, CAMKV, CAMLG, CAMSAP3, CARF, CARMIL2, CASP6, CASS4, CASZ1, CATIP, CAVIN4, CBLB, CBLL1, CBS, CBX7, CC2D1A, CCDC102A, CCDC106, CCDC12, CCDC120, CCDC130, CCDC136, CCDC146, CCDC15, CCDC181, CCDC3, CCDC38, CCDC62, CCDC86, CCDC92, CCDC96, CCN5, CCNE1, CCNI2, CCNJ, CCNO, CCSAP, CCZ1B, CD14, CD22, CD24, CD38, CD40, CD47, CD58, CDADC1, CDAN1, CDC25A, CDC42EP1, CDC42EP2, CDC42EP3, CDC7, CDCA5, CDH17, CDK14, CDK18, CDK2AP1, CDK5RAP3, CDKN1C, CDKN2D, CDS1, CEACAM6, CEMIP, CEMP1, CENPL, CENPS, CENPT, CENPV, CEP112, CEP120, CEP162, CEP295NL, CEP43, CEP57L1, CEP72, CEP85L, CES1, CES3, CFAP298, CFAP300, CFAP410, CFAP43, CFAP58, CFAP70, CFD, CFH, CGNL1, CGRRF1, CHAC1, CHAMP1, CHD1, CHD3, CHD5, CHD9, CHGB, CHIC2, CHMP1B, CHMP4A, CHMP6, CHRAC1, CHRNB4, CHST10, CHST13, CHST14, CHST6, CHSY3, CHTF18, CIAO2B, CIAPIN1, CIB2, CIBAR1, CIITA, CITED2, CITED4, CKB, CLASRP, CLBA1, CLCN4, CLCN5, CLCN6, CLCN7, CLEC16A, CLEC2D, CLIP3, CLMP, CLSPN, CLVS1, CLYBL, CMTM8, CNEP1R1, CNIH2, CNIH3, CNOT3, CNST, COA5, COG5, COL11A2, COL12A1, COL13A1, COL16A1, COL17A1, COL1A1, COL26A1, COL28A1, COL4A3, COL4A6, COL5A1, COL6A1, COL9A3, COMMD1, COMMD10, COMMD4, COPS6, COPZ2, COQ10A, COQ7, CORO2B, COX6A1, COX7A1, COX8A, CP, CPA4, CPPED1, CPQ, CPS1, CPT1C, CRAT, CREB3, CREB3L1, CREBRF, CREG2, CREM, CRISPLD2, CRMP1, CRYAB, CSKMT, CSNK2B, CSRNP3, CSRP2, CSTF2, CTC1, CTDSPL, CTNNA2, CTNNAL1, CTNND2, CTSH, CTSO, CTSV, CTSW, CUBN, CXCL1, CXCR4, CXXC1, CXXC4, CXXC5, CYBC1, CYP1A1, CYP1B1, CYP26A1, CYP27A1, CYP27B1, CYP27C1, CYP39A1, CYP4F12, CYP4F22, DAAM1, DACT1, DBP, DCAF10, DCAF8, DCDC2, DCK, DCLRE1B, DCP2, DCTN3, DCTN6, DDAH2, DDIAS, DDIT4, DDN, DDX47, DDX49, DDX51, DECR2, DEDD, DEFB1, DEGS2, DELE1, DENND6B, DEPDC1, DERL3, DGAT1, DGAT2, DGCR6L, DGKE, DGKQ, DHCR7, DHFR2, DHODH, DHRS1, DHRS2, DHRS4, DHX37, DIS3L, DISC1, DKK1, DKKL1, DLC1, DLEC1, DLL1, DLX1, DLX2, DLX4, DMAC1, DMAC2L, DMC1, DMD, DMPK, DMRTA1, DMRTA2, DMTN, DNAAF3, DNAAF4, DNAH1, DNAH11, DNAH12, DNAH17, DNAH2, DNAH6, DNAJA1, DNAJB11, DNAJB13, DNAJC1, DNAJC27, DNAJC3, DNAJC30, DNAL4, DNASE1, DNASE1L2, DNER, DNHD1, DNM2, DNM3, DNPEP, DOC2A, DOCK7, DOK7, DOLPP1, DOT1L, DPF1, DPH1, DPM3, DPP7, DPYSL3, DRD1, DSC2, DSC3, DSN1, DSP, DTYMK, DUSP26, DUSP28, DUSP7, DUSP8, DXO, DYNLL2, DYNLT3, DYRK1B, DYRK3, E2F6, EAF2, EBF1, EBF2, EBF3, EBF4, ECI1, ECM1, EDA, EEF1E1, EEFSEC, EFCAB6, EFHC1, EFNB1, EGLN3, EGR1, EHD2, EHHADH, EIF6, ELAC1, ELK1, ELL2, ELOF1, ELOVL4, ELOVL7, EML5, ENC1, ENDOG, ENKUR, EPC1, EPHA3, EPHA4, EPHA5,
GO:0043227	membrane-bounded organelle	CellularComponent	0.00	[A1CF, A4GALT, AADAC, AASS, ABCA2, ABCA7, ABCB1, ABCB6, ABCB9, ABCC11, ABCC6, ABCC8, ABCD3, ABCG1, ABHD11, ABHD6, ABITRAM, ACADL, ACADS, ACAP1, ACE, ACER2, ACKR3, ACOT11, ACOT2, ACOX2, ACP2, ACP3, ACSBG1, ACSL1, ACSS3, ACTR3B, ACTR8, ACTRT3, ADAM12, ADAM19, ADAMTS3, ADAP1, ADAP2, ADCY1, ADCY3, ADH1C, ADH6, ADORA1, ADRA1B, ADRB2, AFG1L, AGAP2, AGAP4, AGAP6, AGER, AGPAT4, AGR2, AHRR, AHSG, AIFM3, AIRE, AK8, AK9, AKAP3, AKAP5, AKR1C1, AKR1C4, ALDH1L2, ALDH2, ALDH4A1, ALDH5A1, ALDH8A1, ALDOC, ALG1, ALG10, ALG14, ALKBH4, ALOX5AP, ALPL, ALX1, ALX4, ALYREF, AMACR, AMBP, AMMECR1, AMN, AMOT, AMPD3, ANAPC10, ANAPC15, ANGPT1, ANGPTL1, ANK2, ANK3, ANKRD1, ANKRD13A, ANKRD2, ANKRD23, ANKRD37, ANKRD54, ANKS1B, ANKZF1, ANO1, ANO10, ANO7, ANO8, ANTXR2, ANXA6, ANXA8L1, ANXA9, AOC3, AOX1, AP1S2, AP4B1, AP5B1, AP5Z1, APBB1, APC2, APH1B, APOE, APOH, APOL1, APOO, APPBP2, AQP11, AQP4, AQP7, ARAF, AREG, ARFGAP2, ARFIP1, ARG2, ARHGAP22, ARHGAP39, ARHGAP44, ARHGAP45, ARHGDIB, ARID3B, ARID5B, ARL14, ARL14EP, ARL17B, ARL4D, ARL5B, ARL5C, ARL6, ARL8B, ARMC3, ARMCX6, ARPC5L, ARRDC4, ARSA, ARSD, ARSL, ARV1, ARX, ASB9, ASL, ASPSCR1, ASS1, ATAD3A, ATF3, ATF5, ATF7, ATF7IP2, ATG101, ATP10A, ATP10D, ATP1A3, ATP23, ATP2A1, ATP2A3, ATP2B4, ATP5F1D, ATP5MGL, ATP6V0A2, ATP6V0A4, ATP6V0E2, ATP6V1B1, ATP6V1C1, ATP6V1F, ATP7A, ATP8B3, ATP8B4, ATP9B, AUH, AUNIP, AURKAIP1, AUTS2, AXIN2, B3GALT1, B3GALT4, B3GALT5, B3GAT2, B4GALNT3, B4GALT3, B4GAT1, BABAM1, BACE1, BACH2, BAIAP2, BAIAP2L1, BAIAP3, BANP, BARX1, BBS5, BCAS1, BCHE, BCL11A, BCL11B, BCL2, BCL2A1, BCL2L12, BCL2L15, BCL6, BCL9, BDH1, BDH2, BEND6, BEX2, BEX4, BHLHE41, BIRC7, BLID, BLM, BLOC1S3, BLOC1S5, BMF, BMT2, BNIP3L, BNIPL, BOLA3, BOP1, BORCS7, BORCS8, BPHL, BPNT1, BRAF, BRAT1, BRCA2, BRD3, BRI3BP, BRINP3, BSCL2, BSN, BST1, BTBD8, BTC, BTD, BTN3A3, BYSL, C11orf24, C11orf54, C12orf66, C15orf48, C18orf32, C1D, C1GALT1, C1QTNF1, C1QTNF3, C1orf109, C1orf116, C1orf52, C1orf61, C2, C2CD2, C2orf42, C4A, C5, C6orf58, C7, C8orf44, CA9, CABYR, CACNG4, CACNG7, CACNG8, CAMKMT, CAMKV, CAMLG, CAMSAP3, CAPN5, CARF, CARMIL2, CASP6, CASZ1, CATIP, CBLB, CBLL1, CBS, CBX7, CC2D1A, CCDC106, CCDC12, CCDC120, CCDC130, CCDC136, CCDC3, CCDC62, CCDC86, CCDC92, CCN5, CCNE1, CCNI2, CCNJ, CCNO, CCZ1B, CD14, CD22, CD24, CD38, CD40, CD47, CD58, CDADC1, CDAN1, CDC25A, CDC42EP2, CDC7, CDCA5, CDH17, CDK14, CDK18, CDK2AP1, CDK5RAP3, CDKN1C, CDKN2D, CDS1, CEACAM6, CEMIP, CEMP1, CENPL, CENPS, CENPT, CENPV, CEP162, CEP43, CES1, CES3, CFAP298, CFAP410, CFAP70, CFD, CFH, CFHR3, CGRRF1, CHAC1, CHAMP1, CHD1, CHD3, CHD5, CHD9, CHGB, CHIC2, CHMP1B, CHMP4A, CHMP6, CHRAC1, CHRNB4, CHST10, CHST13, CHST14, CHST6, CHSY3, CHTF18, CIAO2B, CIAPIN1, CIBAR1, CIITA, CITED2, CITED4, CKB, CLASRP, CLBA1, CLCN4, CLCN5, CLCN6, CLCN7, CLEC16A, CLEC2D, CLIP3, CLSPN, CLVS1, CLYBL, CMTM8, CNEP1R1, CNIH2, CNIH3, CNKSR2, CNOT3, CNST, CNTN1, COA5, COG5, COL11A2, COL12A1, COL13A1, COL16A1, COL17A1, COL1A1, COL26A1, COL28A1, COL4A3, COL4A6, COL5A1, COL6A1, COL9A3, COMMD1, COMMD10, COMMD4, COPS6, COPZ2, COQ10A, COQ7, COX6A1, COX7A1, COX8A, CP, CPA4, CPM, CPNE4, CPPED1, CPQ, CPS1, CPT1C, CPVL, CRAT, CREB3, CREB3L1, CREBRF, CREG2, CREM, CRISPLD2, CRMP1, CRYAB, CSKMT, CSNK2B, CSRNP3, CSRP2, CSTF2, CTC1, CTDSPL, CTNNA2, CTNND2, CTSH, CTSO, CTSV, CTSW, CUBN, CXCL1, CXCR4, CXXC1, CXXC4, CXXC5, CYBC1, CYP1A1, CYP1B1, CYP26A1, CYP27A1, CYP27B1, CYP27C1, CYP39A1, CYP4F12, CYP4F22, CYSRT1, DACT1, DBP, DCAF10, DCAF8, DCDC2, DCK, DCLRE1B, DCP2, DCTN3, DDAH1, DDAH2, DDIAS, DDIT4, DDN, DDT, DDTL, DDX47, DDX49, DDX51, DECR2, DEDD, DEFB1, DEGS2, DELE1, DENND6B, DEPDC1, DERL3, DGAT1, DGAT2, DGCR6L, DGKE, DGKQ, DHCR7, DHFR2, DHODH, DHRS1, DHRS2, DHRS4, DHX37, DISC1, DKK1, DKKL1, DLC1, DLEC1, DLL1, DLX1, DLX2, DLX4, DMAC1, DMAC2L, DMC1, DMD, DMPK, DMRTA1, DMRTA2, DMTN, DNAAF3, DNAAF4, DNAJA1, DNAJB11, DNAJC1, DNAJC27, DNAJC3, DNAJC30, DNASE1, DNASE1L2, DNER, DNHD1, DNM2, DNM3, DNPEP, DOC2A, DOCK7, DOK7, DOLPP1, DOT1L, DPF1, DPH1, DPM3, DPP7, DPYSL3, DRD1, DSC2, DSC3, DSN1, DSP, DTYMK, DUSP26, DUSP28, DUSP7, DUSP8, DXO, DYNLL2, DYNLT3, DYRK1B, DYRK3, E2F6, EAF2, EBF1, EBF2, EBF3, EBF4, ECI1, ECM1, EDA, EDIL3, EEF1E1, EEFSEC, EFCAB6, EFCAB7, EFNB1, EGLN3, EGR1, EHD2, EHHADH, EIF6, ELAC1, ELK1, ELL2, ELOF1, ELOVL4, ELOVL7, EML5, ENC1, ENDOG, ENKUR, EPC1, EPCAM, EPHA3, EPHA4, EPHA5, EPHA6, EPN3, EPOP, EPOR, ERAP1, ERAP2, ERCC2, ERCC8, EREG, ERLIN2, ERO1B, ESR2, ESRP1, ESRRG, ESYT3, ETAA1, ETFBKMT, ETNPPL, ETV2, EVA1A, EVC, EVC2, EXO5, EXOG, EXOSC1, EXPH5,
GO:0003723	RNA binding	MolecularFunction	0.00	[A1CF, AK8, ALYREF, ASS1, AUH, BOP1, BTN3A3, BYSL, C1D, C1orf52, C4BPA, CCDC86, CCDC9B, CHD3, CMSS1, CSTF2, DCP2, DDX47, DDX49, DDX51, DDX60L, DHFR2, DHX37, DIS3L, DLX2, DSP, DUS3L, DXO, EEFSEC, EFNA1, EIF1AX, EIF3CL, EIF6, ELOF1, ENDOU, ENOX2, ESRP1, EXOSC1, EYA1, FAM98A, FAM98B, FASTKD3, FCF1, FKBPL, GPKOW, GRB7, GTPBP10, GTPBP2, H1-10, H1-5, H4C12, HEXIM2, HMGN5, HOXB6, HSPA1A, IBA57, IFI16, IFIH1, IFIT2, IGHMBP2, IMP3, ISG20, JRK, KCTD12, KHDRBS3, KHSRP, LGALS3, LRP1, LSM5, LTB4R2, MANF, MAPT, MATR3, MCAT, MEPCE, MPHOSPH10, MPHOSPH6, MRPL1, MRPL12, MRPL14, MRPL18, MRPL2, MRPL23, MRPL27, MRPL28, MRPL30, MRPL39, MRPL54, MTIF3, MTPAP, MTRES1, NANOS1, NANOS3, NBPF10, NOVA1, NSUN3, NUDT1, NUDT4, OASL, PABPN1, PAPOLG, PARP12, PCBP4, PCDHGA9, PCSK9, PEG10, PELP1, PINX1, PIWIL4, PNLDC1, PNO1, POP5, POU5F1, PPAN, PPP1R8, PSMA1, PSTK, PTCD2, PUS10, PWP2, R3HDM2, RADX, RAVER1, RBM14, RBM3, RBM34, RBM43, RBM7, RDM1, REXO5, RFTN1, RNASEL, RNASET2, RPL36A, RPL9, RPP40, RPS10, RPS19BP1, RPS26, RPS28, RPUSD1, RPUSD2, RRP8, S100A16, SARNP, SCAF4, SIDT1, SLBP, SLFN11, SLFN13, SNRPB, SNRPF, SNRPN, SORBS2, SOX2, SOX4, SOX9, SRFBP1, SRRM3, SRSF12, STXBP1, SUPT4H1, SYNE1, TDRD3, TEX19, TLR3, TNRC6A, TNRC6C, TNS1, TOE1, TP53, TRDMT1, TRMT11, TRUB2, TSFM, TUT1, TYW5, U2AF1, U2AF1L4, U2AF1L5, UBA1, UBE2I, UTP15, UTP20,
GO:0003729	mRNA binding	MolecularFunction	0.00	[A1CF, AUH, CSTF2, DHFR2, DXO, EEFSEC, EIF6, ESRP1, JRK, KHDRBS3, KHSRP, MATR3, NOVA1, PCBP4, PPP1R8, RBM14, RBM3, RPS26, SLBP, SRRM3, TP53, TUT1, ZC3H11A]

GO:0005003	ephrin receptor activity	MolecularFunction	0.00	[EFNA3, EFNA4, EPHA3, EPHA4, EPHA5, EPHA6, EPHA7, EPHB3, EPHB6, NTRK3]
GO:0008410	CoA-transferase activity	MolecularFunction	0.00	[AMACR, CHRFAM7A, CHRNA7, OXCT1, OXCT2, SUGCT, TSPO]
GO:0045296	cadherin binding	MolecularFunction	0.00	[ABCF3, ANK3, BAIAP2, BAIAP2L1, CC2D1A, CDC42EP1, CDH17, CDH3, CDH4, CDH6, CDHR3, CHMP4A, CTNNA2, CTNNAL1, CTNND2, EPCAM, H1-10, HSPA1A, LYPLA2, MICALL1, MPP7, NCK1, NDRG1, P2RX4, PKP2, PPL, PPP1R13L, PTPRH, RAB43, RPS26, S100P, SLC9A3R2, SPTBN2, STX5, TBC1D2, TRIM29]
GO:0043169	cation binding	MolecularFunction	0.00	[ABLIM2, ABLIM3, ACAP1, ACAP3, ACE, ACER2, ACVR2A, ACVRL1, ADAM12, ADAM19, ADAMTS15, ADAMTS3, ADAMTS6, ADAMTS8, ADAP1, ADAP2, ADCY1, ADCY3, ADCY5, ADGRE1, ADH1C, ADH6, ADSS1, AGAP2, AGAP4, AGAP6, AHRR, AIFM3, AIRE, ALKBH4, ALPL, AMDHD1, AMPD3, AMZ1, AMZ2, ANKZF1, ANO1, ANTXR2, ANXA6, ANXA8L1, ANXA9, AOC2, AOC3, AOX1, APOE, ARAF, ARAP2, ARAP3, AREG, ARFGAP2, ARG2, ARHGAP45, ARL4D, ARL6, ARMT1, ARSA, ARSD, ARSL, ASPHD2, ATAD3A, ATF7, ATP10A, ATP10D, ATP1A3, ATP23, ATP2A1, ATP2A3, ATP2B4, ATP7A, ATP8B3, ATP8B4, ATP9B, B3GAT2, B4GALT3, B4GAT1, BAIAP3, BCHE, BCL11A, BCL11B, BCL6, BCO1, BIRC7, BLM, BPNT1, BRAF, BSN, C11orf54, C1D, C1GALT1, C2, CA11, CA8, CA9, CABCOCO1, CABYR, CACNA1H, CALML4, CAPN12, CAPN15, CARNS1, CASZ1, CBLB, CBLL1, CBS, CCDC106, CDADC1, CDC7, CDH17, CDH3, CDH4, CDH6, CDHR3, CENPV, CFH, CGRRF1, CHAMP1, CHD3, CHD5, CHRFAM7A, CHRNA7, CHRNB4, CHSY3, CIAO3, CIAPIN1, CIB2, CIB4, CLYBL, CNKSR2, CNRIP1, COL11A2, COL1A1, COL5A1, COMMD1, COQ7, CP, CPA4, CPM, CPNE4, CPPED1, CPQ, CPS1, CRAT, CRELD1, CRIP2, CRYAB, CSNK2B, CSRP2, CTDSPL, CUBN, CXCR4, CXXC1, CXXC4, CXXC5, CYP1A1, CYP1B1, CYP26A1, CYP27A1, CYP27B1, CYP27C1, CYP39A1, CYP4F12, CYP4F22, DCP2, DDAH1, DGKE, DGKG, DGKQ, DLK2, DLL1, DMAC2L, DMD, DMPK, DMRTA1, DMRTA2, DNAJA1, DNASE1L2, DNER, DNPEP, DOC2A, DOHH, DPF1, DPYD, DRD1, DSC2, DSC3, DUS3L, DXO, DYRK3, DZANK1, EBF1, EBF2, EBF3, EBF4, EDIL3, EFCAB10, EFCAB11, EFCAB6, EFCAB7, EFHC1, EGLN3, EGR1, EHD2, ELAC1, ELOF1, ENDOG, ENDOU, ENO4, ERAP1, ERAP2, ERCC2, ESR2, ESRRG, ESYT3, EXD3, EXO5, EXOG, EXT1, EYA1, EYA2, F12, F7, FA2H, FAHD2A, FAT3, FAXDC2, FBLIM1, FBLN5, FGA, FGD4, FIBCD1, FKBP7, FNTB, FOXE1, FOXP1, FOXP2, FOXP4, FPGT-TNNI3K, FSTL4, FSTL5, FXN, GALNS, GALNT16, GALNT17, GALNT5, GALNT9, GALT, GATA2, GATA3, GATA5, GATA6, GCH1, GJB2, GLI1, GLI2, GLI3, GLRB, GLRX5, GLUL, GMIP, GNA13, GORASP1, GPHN, GRIN1, GRIN2B, GTF2E1, GTF2H2, GTF2H2C, GUCA1B, GYG2, HAGHL, HDAC9, HDHD2, HFE, HGD, HHIP, HINFP, HIVEP3, HMGCL, HMGCLL1, HMOX1, HNF1A, HNF4A, HPD, HR, HSPG2, IFIH1, IGFBP3, IGHMBP2, IKZF4, IMPA1, ING1, ING4, INPP1, INTS12, IPMK, ISG20, ITGA1, ITGA10, ITGA4, ITGA7, ITGA9, ITGAM, ITGAX, ITGB7, JAZF1, JMJD7, KAT5, KAT6B, KCND2, KCNJ11, KDM4C, KDM5B, KDM7A, KHSRP, KLF16, KLF2, KLF4, KLF7, KLF9, KSR2, L3MBTL1, LAP3, LCP1, LFNG, LHPP, LHX4, LHX6, LHX8, LHX9, LIMS1, LIPC, LMO2, LMTK3, LONRF2, LOX, LOXL3, LRIG1, LRP1, LRP2, LTBP1, LTBP2, MAK, MAN1A1, MAN2B1, MAP2K5, MAP3K1, MAP3K15, MAP3K6, MAPRE3, MAST1, MAST2, MATN2, MATN3, MATR3, MB, MBTPS2, MEGF6, MGAT2, MGMT, MIB2, MICAL1, MICALL1, MICU3, MID1, MID2, MKRN3, MME, MMP11, MMP16, MMP25, MMP28, MMP3, MOB2, MOB4, MOXD1, MPPE1, MPPED2, MSRB2, MSS51, MT1X, MTHFS, MTPAP, MVK, NAALAD2, NAGS, NANOS1, NANOS3, NAPRT, NCALD, NDRG1, NDUFS7, NDUFV2, NECAB2, NECAB3, NEK4, NEK8, NEURL1B, NEURL3, NHLRC1, NINL, NKD1, NKD2, NME2, NME3, NME4, NMRK1, NOS2, NOX5, NPEPL1, NPTX1, NR1D1, NR1H4, NR2E3, NR2F6, NR3C2, NR4A1, NR4A2, NR4A3, NRXN2, NRXN3, NT5C3A, NT5C3B, NT5DC2, NUAK1, NUBP1, NUCB1, NUDT1, NUDT11, NUDT13, NUDT14, NUDT15, NUDT17, NUDT4, OGFOD3, OSGEP, OSGEPL1, OSR2, OVOL3, P2RX4, P4HA3, P4HTM, PADI1, PADI2, PADI3, PAN2, PAPOLG, PARP12, PC, PCDH1, PCDH10, PCDH18, PCDH7, PCDH9, PCDHA10, PCDHA11, PCDHA12, PCDHA4, PCDHA5, PCDHA6, PCDHA8, PCDHA9, PCDHB11, PCDHB13, PCDHB3, PCDHB5, PCDHB6, PCDHGA1, PCDHGA10, PCDHGA11, PCDHGA4, PCDHGA5, PCDHGA6, PCDHGA8, PCDHGA9, PCDHGB1, PCDHGB2, PCDHGB3, PCDHGB4, PCDHGC3, PCGF1, PCGF6, PCK2, PCLO, PDE12, PDE1A, PDE1C, PDE3A, PDE5A, PDE9A, PDF, PDLIM2, PEG10, PELO, PGGT1B, PGM5, PHACTR1, PHF1, PHF21A, PHOSPHO2, PHYH, PIF1, PKMYT1, PLA2G4C, PLAG1, PLCB1, PLCB2, PLCB4, PLCD4, PLCE1, PLCH2, PLEKHF2, PLSCR4, PNLDC1, POLA1, POLR3K, POMT1, PON1, PPARA, PPAT, PPEF1, PPM1D, PPM1L, PPM1M, PPP2R3A, PPP3R1, PRDM1, PRDM13, PRDM5, PRDM8, PRICKLE3, PRIM2, PRIMPOL, PRKAA2, PRKCG, PRKCH, PRKCZ, PRKD1, PRKD3, PRNP, PROC, PRORP, PRPS2, PRRG1, PRRG2, PRTFDC1, PRUNE2, PTER, PTGS1, PTGS2, PUDP, PUS10, PXDN, PYGL, QPCT, RAB11FIP3, RABGEF1, RABIF, RAD50, RAG1, RAP2A, RASA3, RBKS, RCHY1, RDH14, RET, RFK, RHBDL3, RHEBL1, RHOT2, RHOU, RIMS2, RIOK2, RNASEL, RNF103, RNF113A, RNF122, RNF125, RNF135, RNF144B, RNF166, RNF182, RNF185, RNF19A, RNF31, RNF32, RNF38, RNF39, RNF43, RNF44, RNF8, RNFT1, RNPEPL1, ROR2, RPEL1, RPH3A, RPS6KA2, RRAGC, RRM2, RSF1, RTEL1, RXRA, RYR1, S100A16, S100A5, S100P, SALL1, SALL2, SALL4, SAR1B, SCIN, SELENOO, SESN3, SETDB2, SETMAR, SH3RF2, SH3RF3, SHH, SLC1A2, SLC1A3, SLC25A25, SLC30A5, SLC8A1, SLFN11, SLFN13, SLIT1, SLIT2, SLIT3,
GO:0046872	metal ion binding	MolecularFunction	0.00	[ABLIM2, ABLIM3, ACAP1, ACAP3, ACE, ACER2, ACVR2A, ACVRL1, ADAM12, ADAM19, ADAMTS15, ADAMTS3, ADAMTS6, ADAMTS8, ADAP1, ADAP2, ADCY1, ADCY3, ADCY5, ADGRE1, ADH1C, ADH6, ADSS1, AGAP2, AGAP4, AGAP6, AHRR, AIFM3, AIRE, ALKBH4, ALPL, AMDHD1, AMPD3, AMZ1, AMZ2, ANKZF1, ANO1, ANTXR2, ANXA6, ANXA8L1, ANXA9, AOC2, AOC3, AOX1, APOE, ARAF, ARAP2, ARAP3, AREG, ARFGAP2, ARG2, ARHGAP45, ARL4D, ARL6, ARMT1, ARSA, ARSD, ARSL, ASPHD2, ATAD3A, ATF7, ATP10A, ATP10D, ATP1A3, ATP23, ATP2A1, ATP2A3, ATP2B4, ATP7A, ATP8B3, ATP8B4, ATP9B, B3GAT2, B4GALT3, B4GAT1, BAIAP3, BCL11A, BCL11B, BCL6, BCO1, BIRC7, BLM, BPNT1, BRAF, BSN, C11orf54, C1D, C1GALT1, C2, CA11, CA8, CA9, CABCOCO1, CABYR, CACNA1H, CALML4, CAPN12, CAPN15, CARNS1, CASZ1, CBLB, CBLL1, CBS, CCDC106, CDADC1, CDC7, CDH17, CDH3, CDH4, CDH6, CDHR3, CENPV, CFH, CGRRF1, CHAMP1, CHD3, CHD5, CHSY3, CIAO3, CIAPIN1, CIB2, CIB4, CLYBL, CNKSR2, CNRIP1, COL11A2, COL1A1, COL5A1, COMMD1, COQ7, CP, CPA4, CPM, CPNE4, CPPED1, CPQ, CPS1, CRAT, CRELD1, CRIP2, CRYAB, CSNK2B, CSRP2, CTDSPL, CUBN, CXCR4, CXXC1, CXXC4, CXXC5, CYP1A1, CYP1B1, CYP26A1, CYP27A1, CYP27B1, CYP27C1, CYP39A1, CYP4F12, CYP4F22, DCP2, DDAH1, DGKE, DGKG, DGKQ, DLK2, DLL1, DMAC2L, DMD, DMPK, DMRTA1, DMRTA2, DNAJA1, DNASE1L2, DNER, DNPEP, DOC2A, DOHH, DPF1, DPYD, DSC2, DSC3, DUS3L, DXO, DYRK3, DZANK1, EBF1, EBF2, EBF3, EBF4, EDIL3, EFCAB10, EFCAB11, EFCAB6, EFCAB7, EFHC1, EGLN3, EGR1, EHD2, ELAC1, ELOF1, ENDOG, ENDOU, ENO4, ERAP1, ERAP2, ERCC2, ESR2, ESRRG, ESYT3, EXD3, EXO5, EXOG, EXT1, EYA1, EYA2, F12, F7, FA2H, FAHD2A, FAT3, FAXDC2, FBLIM1, FBLN5, FGA, FGD4, FIBCD1, FKBP7, FNTB, FOXE1, FOXP1, FOXP2, FOXP4, FPGT-TNNI3K, FSTL4, FSTL5, FXN, GALNS, GALNT16, GALNT17, GALNT5, GALNT9, GALT, GATA2, GATA3, GATA5, GATA6, GCH1, GJB2, GLI1, GLI2, GLI3, GLRX5, GLUL, GMIP, GNA13, GORASP1, GPHN, GRIN1, GRIN2B, GTF2E1, GTF2H2, GTF2H2C, GUCA1B, GYG2, HAGHL, HDAC9, HDHD2, HFE, HGD, HHIP, HINFP, HIVEP3, HMGCL, HMGCLL1, HMOX1, HNF1A, HNF4A, HPD, HR, HSPG2, IFIH1, IGFBP3, IGHMBP2, IKZF4, IMPA1, ING1, ING4, INPP1, INTS12, IPMK, ISG20, ITGA1, ITGA10, ITGA4, ITGA7, ITGA9, ITGAM, ITGAX, ITGB7, JAZF1, JMJD7, KAT5, KAT6B, KCND2, KCNJ11, KDM4C, KDM5B, KDM7A, KHSRP, KLF16, KLF2, KLF4, KLF7, KLF9, KSR2, L3MBTL1, LAP3, LCP1, LFNG, LHPP, LHX4, LHX6, LHX8, LHX9, LIMS1, LIPC, LMO2, LMTK3, LONRF2, LOX, LOXL3, LRIG1, LRP1, LRP2, LTBP1, LTBP2, MAK, MAN1A1, MAN2B1, MAP2K5, MAP3K1, MAP3K15, MAP3K6, MAPRE3, MAST1, MAST2, MATN2, MATN3, MATR3, MB, MBTPS2, MEGF6, MGAT2, MGMT, MIB2, MICAL1, MICALL1, MICU3, MID1, MID2, MKRN3, MME, MMP11, MMP16, MMP25, MMP28, MMP3, MOB2, MOB4, MOXD1, MPPE1, MPPED2, MSRB2, MSS51, MT1X, MTHFS, MTPAP, MVK, NAALAD2, NANOS1, NANOS3, NAPRT, NCALD, NDRG1, NDUFS7, NDUFV2, NECAB2, NECAB3, NEK4, NEK8, NEURL1B, NEURL3, NHLRC1, NINL, NKD1, NKD2, NME2, NME3, NME4, NMRK1, NOS2, NOX5, NPEPL1, NPTX1, NR1D1, NR1H4, NR2E3, NR2F6, NR3C2, NR4A1, NR4A2, NR4A3, NRXN2, NRXN3, NT5C3A, NT5C3B, NT5DC2, NUAK1, NUBP1, NUCB1, NUDT1, NUDT11, NUDT13, NUDT14, NUDT15, NUDT17, NUDT4, OGFOD3, OSGEP, OSGEPL1, OSR2, OVOL3, P2RX4, P4HA3, P4HTM, PADI1, PADI2, PADI3, PAN2, PAPOLG, PARP12, PC, PCDH1, PCDH10, PCDH18, PCDH7, PCDH9, PCDHA10, PCDHA11, PCDHA12, PCDHA4, PCDHA5, PCDHA6, PCDHA8, PCDHA9, PCDHB11, PCDHB13, PCDHB3, PCDHB5, PCDHB6, PCDHGA1, PCDHGA10, PCDHGA11, PCDHGA4, PCDHGA5, PCDHGA6, PCDHGA8, PCDHGA9, PCDHGB1, PCDHGB2, PCDHGB3, PCDHGB4, PCDHGC3, PCGF1, PCGF6, PCK2, PCLO, PDE12, PDE1A, PDE1C, PDE3A, PDE5A, PDE9A, PDF, PDLIM2, PEG10, PELO, PGGT1B, PGM5, PHACTR1, PHF1, PHF21A, PHOSPHO2, PHYH, PIF1, PKMYT1, PLA2G4C, PLAG1, PLCB1, PLCB2, PLCB4, PLCD4, PLCE1, PLCH2, PLEKHF2, PLSCR4, PNLDC1, POLA1, POLR3K, POMT1, PON1, PPARA, PPAT, PPEF1, PPM1D, PPM1L, PPM1M, PPP2R3A, PPP3R1, PRDM1, PRDM13, PRDM5, PRDM8, PRICKLE3, PRIM2, PRIMPOL, PRKAA2, PRKCG, PRKCH, PRKCZ, PRKD1, PRKD3, PRNP, PROC, PRORP, PRPS2, PRRG1, PRRG2, PRTFDC1, PRUNE2, PTER, PTGS1, PTGS2, PUDP, PUS10, PXDN, QPCT, RAB11FIP3, RABGEF1, RABIF, RAD50, RAG1, RAP2A, RASA3, RBKS, RCHY1, RDH14, RET, RFK, RHBDL3, RHEBL1, RHOT2, RHOU, RIMS2, RIOK2, RNASEL, RNF103, RNF113A, RNF122, RNF125, RNF135, RNF144B, RNF166, RNF182, RNF185, RNF19A, RNF31, RNF32, RNF38, RNF39, RNF43, RNF44, RNF8, RNFT1, RNPEPL1, ROR2, RPEL1, RPH3A, RPS6KA2, RRAGC, RRM2, RSF1, RTEL1, RXRA, RYR1, S100A16, S100A5, S100P, SALL1, SALL2, SALL4, SAR1B, SCIN, SELENOO, SETDB2, SETMAR, SH3RF2, SH3RF3, SHH, SLC1A2, SLC1A3, SLC25A25, SLC30A5, SLC8A1, SLFN11, SLFN13, SLIT1, SLIT2, SLIT3, SMPD1, SMPD2, SMPD3, SMPDL3A, SMYD3, SNAI1,
GO:0004888	transmembrane signaling receptor activity	MolecularFunction	0.00	[ABCA7, ABCC8, ACKR3, ACVR2A, ACVRL1, ADGRA2, ADGRB1, ADGRD1, ADGRE1, ADGRF4, ADGRL1, ADGRL2, ADORA1, ADORA2B, ADRA1B, ADRB2, AGER, AGTR1, ANTXR2, ANXA9, AREG, BTC, CACNG4, CACNG7, CACNG8, CBLB, CCL2, CCR1, CCR9, CD14, CHMP6, CHRFAM7A, CHRNA7, CHRNA9, CHRNB4, CLDN3, CLDN4, CLEC2D, CNIH2, CNIH3, CNTN1, CSF2RA, CTSH, CXCR4, CXCR5, DLG3, DNER, DRD1, EDA2R, EDAR, EDNRA, EFNA3, EFNA4, ELOVL4, EPHA3, EPHA4, EPHA5, EPHA6, EPHA7, EPHB3, EPHB6, EPOR, ERBB3, EREG, FFAR4, FGFR2, FGFR3, FZD5, FZD9, GABBR1, GABRE, GFRA1, GIPR, GLP2R, GLRB, GPR132, GPR153, GPR156, GPR160, GPR162, GPR179, GPR182, GPR183, GPR19, GPR21, GPR27, GPR35, GPR37, GPR52, GPR75,
GO:0004930	G protein-coupled receptor activity	MolecularFunction	0.00	[ACKR3, ADGRA2, ADGRB1, ADGRD1, ADGRE1, ADGRF4, ADGRL1, ADGRL2, ADORA1, ADORA2B, ADRA1B, ADRB2, AGTR1, CCR1, CCR9, CXCR4, CXCR5, DRD1, EDNRA, ELOVL4, FFAR4, FZD5, FZD9, GABBR1, GIPR, GLP2R, GPR132, GPR153, GPR156, GPR160, GPR162, GPR179, GPR182, GPR183, GPR19, GPR21, GPR27, GPR35, GPR37, GPR52, GPR75, GPR85, GPRC5C, HRH1, HTR1D, KISS1R, LPAR4, LPAR6, LTB4R, LTB4R2, NPRL3, OR1J4, OXTR, P2RY1, P2RY11, P2RY6, PAX8, PCDHA4, PCDHA6, QRFPR, S1PR2, SMOX, SORCS2, SPHK1, SUCNR1, TAPT1, TAS1R1, TAS2R19, TAS2R30, TAS2R4, TAS2R5,
GO:0000981	DNA-binding transcription factor activity, RNA polymerase II-specific	MolecularFunction	0.00	[AHRR, AIRE, ALX1, ALX4, AREG, ARID3B, ARID5B, ARX, ATF3, ATF5, ATF7, BACH2, BARX1, BCL11A, BCL11B, BCL6, BHLHE41, CARF, CC2D1A, CREB3, CREB3L1, CREBRF, CREM, CSRNP3, CYP1A1, CYP27B1, DBP, DDN, DLX1, DLX2, DLX4, DMRTA1, DMRTA2, E2F6, EAF2, EBF1, EBF2, EBF3, EBF4, EGR1, ELK1, ELOF1, ESR2, ESRRG, ETV2, EZH1, FOS, FOSL1, FOSL2, FOXA1, FOXA3, FOXD1, FOXD3, FOXD4L1, FOXE1, FOXF2, FOXN4, FOXP1, FOXP2, FOXP4, GATA2, GATA3, GATA5, GATA6, GATD3A, GLI1, GLI2, GLI3, GRHL3, GSC, HES1, HES2, HEY1, HINFP, HLX, HMX1, HNF1A, HNF4A, HOXA1, HOXA10, HOXA13, HOXA5, HOXA6, HOXB13, HOXB2, HOXB3, HOXB5, HOXB6, HOXB7, HOXB8, HOXB9, HOXC6, HOXC8, HOXC9, HOXD1, HOXD10, HOXD13, HOXD9, HSF4, IFI16, IRF1, IRF4, IRF5, KCNH4, KLF16, KLF2, KLF4, KLF7, KLF9, KLRG1, LBX2, LHX4, LHX6, LHX8, LHX9, LMO2, MAFK, MAPRE3, MEF2B, MEIS1, MESP1, MGAM, MIA3, MIER3, MITF, MLXIPL, MNX1, MSX2, MXD3, MXI1, MYCL, MYPOP, NFATC1, NFIX, NFYA, NKX2-1, NKX2-3, NKX2-5, NKX3-1, NKX3-2, NKX6-1, NR1D1, NR1H4, NR2E3, NR2F6, NR3C2, NR4A1, NR4A2, NR4A3, NRL, OTX1, OVOL3, PAX2, PAX7, PAX8, PAX9, PBX2, PBX4, PCGF6, PITX1, PITX2, PKMYT1, PKNOX1, PLAG1, POU3F3, POU4F3, POU5F1, POU5F2, POU6F1, PPARA, PRDM1, PRDM5, PRRX2, RCOR2, RELB, RUNX2, RXRA, SALL1, SALL2, SARNP, SATB1, SATB2, SIM2, SIX4, SNAI1, SOX17, SOX2, SOX21, SOX4, SOX5, SOX6, SOX7, SOX9, SP140L, SP8, STAT4, STAT5A, TBX20, TBX3, TBX6, TCF7, TEAD3, TFAP2C, THAP11, THAP12, THAP6, THAP8, TP53, TRPS1, TSC22D4, TSHZ3, TWIST1, USF3, VDR, YY2, ZBED3, ZBTB17, ZBTB2, ZBTB32, ZBTB34, ZBTB45, ZBTB46, ZC3H6, ZFX, ZKSCAN2, ZKSCAN3, ZMYM5, ZMYM6, ZNF174, ZNF175, ZNF202, ZNF239, ZNF300, ZNF335, ZNF350, ZNF396, ZNF487, ZNF511, ZNF524, ZNF639, ZNF654, ZNF668, ZSCAN21]
GO:0043565	sequence-specific DNA binding	MolecularFunction	0.00	[AHRR, AIRE, ALX1, ALX4, AREG, ARID3B, ARID5B, ARX, ATF3, ATF5, BACH2, BARX1, BCL11A, BCL11B, BCL2, BCL6, BHLHE41, BLM, CARF, CC2D1A, CNRIP1, CREB3, CREB3L1, CREBRF, CREM, CSRNP3, CTC1, CXXC1, CXXC4, CXXC5, CYP1A1, CYP27B1, DBP, DDN, DLX1, DLX2, DLX4, DMRTA1, DMRTA2, DOT1L, E2F6, EAF2, EBF2, EBF3, EBF4, EGR1, ELK1, ELOF1, ESR2, ESRRG, ETV2, FOS, FOSL1, FOSL2, FOXA1, FOXA3, FOXD1, FOXD3, FOXD4L1, FOXE1, FOXF2, FOXN4, FOXP1, FOXP2, FOXP4, GATA2, GATA3, GATA5, GATA6, GATD3A, GLI1, GLI2, GLI3, GRHL3, GSC, H2AZ1, HES1, HES2, HEY1, HINFP, HLX, HMX1, HNF1A, HNF4A, HOXA1, HOXA10, HOXA13, HOXA5, HOXA6, HOXB13, HOXB2, HOXB3, HOXB5, HOXB6, HOXB7, HOXB8, HOXB9, HOXC6, HOXC8, HOXC9, HOXD1, HOXD10, HOXD13, HOXD9, HR, HSF4, IFI16, IKZF4, IRF1, IRF4, IRF5, KAT2B, KCNH4, KDM5B, KLF16, KLF4, KLF7, KLRG1, LAMTOR5, LHX4, LHX6, LHX8, LHX9, LMO2, MAD2L2, MAFK, MAPRE3, MAPT, MEF2B, MEIS1, MESP1, MGAM, MIA3, MITF, MLXIPL, MNX1, MSX2, MUC1, MXD3, MXI1, MYCL, MYPOP, NFATC1, NFYA, NKX2-1, NKX2-3, NKX2-5, NKX3-1, NKX3-2, NKX6-1, NR1D1, NR1H4, NR2E3, NR2F6, NR3C2, NR4A1, NR4A2, NR4A3, NRL, ORC4, OSR2, OTX1, OVOL3, PAX2, PAX7, PAX8, PAX9, PBX2, PBX4, PCGF6, PER2, PER3, PIF1, PINX1, PITX1, PITX2, PKMYT1, PKNOX1, PLAG1, POLA1, POU3F3, POU4F3, POU5F1, POU6F1, PPARA, PRDM1, PRDM5, PRRX2, RAD50, RAG1, RELB, RPA2, RUNX2, RXRA, SALL1, SALL2, SARNP, SATB1, SATB2, SIX4, SMARCB1, SMYD3, SNAI1, SNAPC1, SOX17, SOX2, SOX21, SOX4, SOX5, SOX6, SOX7, SOX9, STAT4, STAT5A, STON1, STOX1, SUV39H1, TBX20, TBX3, TBX6, TCF7, TEAD3, TELO2, TEN1-CDK3, TENM1, TET1, TFAP2C, THAP11, TOX3, TP53, TRAF6, TRPS1, TWIST1, VDR, WDR77, YY2, ZBTB17, ZBTB2, ZBTB32, ZBTB34, ZBTB45, ZBTB48, ZBTB49, ZFP37, ZFP41, ZKSCAN3, ZNF10, ZNF121, ZNF134, ZNF138, ZNF14, ZNF174, ZNF175, ZNF180, ZNF19, ZNF202, ZNF22, ZNF239, ZNF266, ZNF286B, ZNF296, ZNF3, ZNF300, ZNF329, ZNF335, ZNF35, ZNF350, ZNF44, ZNF443, ZNF487, ZNF502, ZNF516, ZNF517, ZNF519, ZNF529, ZNF554, ZNF559, ZNF566, ZNF568, ZNF57, ZNF572, ZNF599, ZNF605, ZNF614, ZNF627, ZNF628, ZNF639, ZNF668, ZNF669, ZNF675, ZNF678, ZNF684, ZNF688, ZNF696, ZNF704, ZNF708, ZNF714, ZNF799, ZNF846, ZNF891, ZSCAN21, ZW10]
GO:0002250	adaptive immune response	ImmuneSystemProcess	0.00	[ADGRE1, AGER, AIRE, AKR1C1, ARG2, BACH2, BCL6, BTN3A1, BTN3A3, C2, C4A, C4BPA, C5, C7, CD40, CTSH, EPHB6, ERAP1, ERAP2, FCGRT, FGA, FGB, FGL1, FGL2, FZD5, GATA3, GPR183, HFE, HLA-DMB, HLA-DRA, HLA-F, HLX, ICAM1, IFNE, IL18BP, IL18R1, IL1R1, IL20RB, IL27RA, IL7R, IRF1, IRF4, LAG3, LOXL3, LRIF1, MAD2L2, MALT1, MICA, MICB, MR1, NFKBIZ, NR4A3, ORAI1, PIK3CG, PRDM1, PRKCZ, PTPRC, PYCARD, RAG1, RELB, RFTN1, RNF125, RNF8, SHLD1, SUSD4, TMEM98, TNFRSF13C, TRAF6, UNC13D, UNC93B1, ZP3]
GO:0006955	immune response	ImmuneSystemProcess	0.00	[ACKR3, ACP3, ADGRB1, ADGRE1, ADORA2B, AGER, AHSG, AIRE, AKR1C1, ALDOC, AMBP, AMPD3, ANGPT1, APOE, APOL1, AQP4, ARG2, ARHGAP45, ARSA, ASS1, ATP6V0A2, ATP7A, ATP8B4, BACH2, BAIAP2, BCL2, BCL6, BRAF, BST1, BTN3A1, BTN3A3, BTNL9, C1D, C2, C4A, C4BPA, C5, C7, CCL2, CCL26, CCL5, CCR1, CCR9, CD14, CD22, CD24, CD38, CD40, CD47, CD58, CD83, CDC42EP2, CDH17, CEACAM6, CFD, CFH, CHAMP1, CHRNB4, CIITA, CLEC2D, CNRIP1, COL17A1, COL1A1, CPPED1, CRAT, CRISPLD2, CSNK2B, CST9, CTSH, CTSW, CXCL1, CXCL2, CXCL3, CXCL5, CXCL8, CXCR4, CXCR5, CYBC1, CYP27B1, DEFB1, DGAT1, DLC1, DLL1, DNAJC3, DNASE1, DPP7, DSC3, DSN1, DSP, ECM1, EDA, EGR1, EHHADH, ELMO1, ELOF1, ENC1, ENDOU, EPHB6, ERAP1, ERAP2, EREG, F12, FABP5, FCGRT, FCRLB, FGA, FGB, FGL1, FGL2, FOS, FOXP1, FYB1, FZD5, GAB2, GALNS, GATA2, GATA3, GBP3, GBP4, GCH1, GLIPR1, GPLD1, GPR183, GPX1, GRAP2, GSDMD, HFE, HLA-DMB, HLA-DRA, HLA-F, HLX, HMOX1, HPSE, HSPA1A, ICAM1, ICAM3, IFI16, IFI27, IFIH1, IFIT2, IFITM2, IFITM3, IFNE, IKBKE, IL18BP, IL18R1, IL1R1, IL1RAP, IL20RB, IL27RA, IL32, IL36B, IL7, IL7R, IRF1, IRF4, IRF5, ISG20, ITGA4, ITGAM, ITGAX, ITGB7, ITM2A, KLRG1, KRT16, LAG3, LCP1, LFNG, LGALS3, LGALS9, LOXL3, LRIF1, LRP1, LTB4R, MAD2L2, MALT1, MAN2B1, MANBA, MAP2K4, MAP3K1, MAPK10, MATR3, MGAM, MICA, MICB, MID1, MID2, MIF, MME, MMP25, MMP28, MNX1, MR1, MUC1, MUC16, MUC20, MUC3A, MUC5AC, MUC5B, NAPRT, NCK1, NEU1, NFATC1, NFKBIZ, NHLRC3, NKX2-3, NLRC3, NME2, NOD2, NOS2, NPDC1, NPPA, NR1H4, NR4A3, OASL, ORAI1, OSCAR, PADI2, PCDHA4, PCDHA6, PDE12, PGAM4, PGC, PIK3CG, PILRB, PLA2G6, PLAC8, PLCL2, PLPPR2, POLR3G, POLR3K, PPP3R1, PQBP1, PRDM1, PRDX4, PRG4, PRKCH, PRKCZ, PRKD1, PRNP, PSMA1, PSMA2, PSMB10, PSMB8, PSMB9, PSMD12, PSMD9, PTPRC, PXDN, PYCARD, PYGL, QPCT, RAB24, RAB3A, RAB43, RAB4B, RAB9B, RAG1, RBM14, RELB, RFTN1, RNASEL, RNASET2, RNF125, RNF135, RNF166, RNF31, RNF8, S100P, SERPINA1, SH2B2, SH2D2A, SHLD1, SLC27A2, SLCO1B1, SNAP25, SNCA, SPNS2, SPON2, STAR, STXBP1, SUCNR1, SUSD4, TAB3, TAC4, TADA2A, TCF7, TENM1, TGFB3, THY1, TKFC, TLR3, TLR5, TMC6, TMEM98, TNFAIP6, TNFRSF13C, TP53, TRAF6, TRIM13, TRIM22, TRIM29, TRIM4, TRIM68, TRPM2, TTC4, TTLL12, TXNDC5, ULBP2, UNC13D, UNC93B1, USP18, UXT, VAMP2, VAMP7, VAMP8, VAV3, VSIR, VTN, WIPF1, WIPF3, ZDHHC11, ZP3]
GO:0006959	humoral immune response	ImmuneSystemProcess	0.00	[AIRE, AKR1C1, ATP7A, BCL2, BST1, C2, C4A, C4BPA, C5, C7, CCL2, CD83, CFD, CFH, CHAMP1, CST9, CXCL1, CXCL2, CXCL3, CXCL5, CXCL8, DEFB1, FGA, FGB, GATA3, GPR183, IFNE, IL7, MNX1, PGC, PLA2G6, PSMB10, PTPRC, SPNS2, SPON2, SUSD4, VTN, ZP3]
GO:0002431	Fc receptor mediated stimulatory signaling pathway	ImmuneSystemProcess	0.00	[BAIAP2, CD47, ELMO1, NCK1, NR4A3, PLA2G6, PTPRC, VAV3, WIPF1, WIPF3]
GO:0038094	Fc-gamma receptor signaling pathway	ImmuneSystemProcess	0.00	[BAIAP2, CD47, ELMO1, NCK1, OSCAR, PLA2G6, PTPRC, VAV3, WIPF1, WIPF3]
GO:0002686	negative regulation of leukocyte migration	ImmuneSystemProcess	0.00	[ADORA1, ADTRP, C5, CCL2, DDT, DDTL, ENC1, GREM1, HMOX1, IL27RA, MIA3, MIF, MMP28, PADI2, PLCB1, SLIT2, UXT]
GO:0002689	negative regulation of leukocyte chemotaxis	ImmuneSystemProcess	0.00	[C5, CCL2, DDT, DDTL, GREM1, MIF, MMP28, PADI2, SLIT2, UXT]
GO:0002757	immune response-activating signal transduction	ImmuneSystemProcess	0.00	[BAIAP2, BCL2, BRAF, BTN3A1, BTN3A3, BTNL9, CD22, CD38, CD47, ELMO1, ELOF1, FOXP1, FYB1, GATA3, GPLD1, GRAP2, HLA-DRA, ICAM3, LGALS3, MALT1, MICB, MUC1, MUC16, MUC20, MUC3A, MUC5AC, MUC5B, NCK1, NFKBIZ, NR4A3, PLA2G6, PLCL2, PRKCH, PRNP, PSMA1, PSMA2, PSMB10, PSMB8, PSMB9, PSMD12, PSMD9, PTPRC, RELB, RFTN1, RNF31, SH2B2, TAB3, THY1, TRAF6, UXT, VAV3, WIPF1, WIPF3]
GO:0002429	immune response-activating cell surface receptor signaling pathway	ImmuneSystemProcess	0.00	[BAIAP2, BCL2, BRAF, BTN3A1, BTN3A3, BTNL9, CD22, CD38, CD47, ELMO1, ELOF1, FOXP1, FYB1, GATA3, GPLD1, GRAP2, HLA-DRA, ICAM3, LGALS3, MALT1, MICB, MUC1, MUC16, MUC20, MUC3A, MUC5AC, MUC5B, NCK1, NFKBIZ, NR4A3, PLA2G6, PLCL2, PRKCH, PRNP, PSMA1, PSMA2, PSMB10, PSMB8, PSMB9, PSMD12, PSMD9, PTPRC, RELB, RFTN1, RNF31, SH2B2, TAB3, THY1, TRAF6, UXT, VAV3, WIPF1, WIPF3]
GO:0002377	immunoglobulin production	ImmuneSystemProcess	0.00	[BCL6, CD22, CD40, FGL2, IL27RA, IL7R, ITM2A, LRIF1, MAD2L2, PTPRC, RNF8, SHLD1, TRAF6]
GO:0006956	complement activation	ImmuneSystemProcess	0.00	[AKR1C1, C2, C4A, C4BPA, C5, C7, CFD, CFH, SUSD4, VTN]
GO:0019724	B cell mediated immunity	ImmuneSystemProcess	0.00	[AKR1C1, BCL6, C2, C4A, C4BPA, C5, C7, CD40, FGL2, IL27RA, LRIF1, MAD2L2, PTPRC, RNF8, SHLD1, SUSD4, ZP3]
GO:0016064	immunoglobulin mediated immune response	ImmuneSystemProcess	0.00	[AKR1C1, BCL6, C2, C4A, C4BPA, C5, C7, CD40, FGL2, IL27RA, LRIF1, MAD2L2, PTPRC, RNF8, SHLD1, SUSD4, ZP3]
