# Supplementary material for: Insight of a Metabolic Prognostic Model to Identify Tumor Environment and Drug Vulnerability for Lung Adenocarcinoma
Source: Front Immunol. 2022 Jun 23;13:872910. doi: 10.3389/fimmu.2022.872910 (PMC9262104; doi:10.3389/fimmu.2022.872910)
Supplement: Supplementary file 5 [file DataSheet_4.pdf]

Supplementary Table S4: The construction of protein-protein interaction (PPI)

node1	node2	coexpressio	experimentally_determined_interacti
RPL9	RPS10	0.997	0.989
RPL9	RPS26	0.992	0.996
FGA	FGB	0.991	0.986
PSMA1	PSMA2	0.991	0.997
RPL9	RPS28	0.989	0.995
RPS10	RPS28	0.988	0.965
RPS10	RPS26	0.985	0.988
RPS26	RPS28	0.978	0.99
EGR1	FOS	0.961	0.062
AHSG	AMBP	0.958	0.057
POLA1	PRIM2	0.958	0.993
ATP5D	NDUFS7	0.952	0.365
AHSG	APOH	0.94	0
AMBP	FGA	0.94	0
MATR3	RBM14	0.939	0.435
ALYREF	MATR3	0.938	0.128
ALYREF	RBM14	0.935	0
MATR3	SNRPF	0.934	0
PSMB8	PSMB9	0.931	0.979
BHLHE41	NR1D1	0.926	0.072
POMP	PSMA2	0.926	0.664
BOP1	DDX49	0.925	0.245
ATP5D	NDUFB8	0.924	0
DNAJB11	MANF	0.924	0
CHTF18	POLA1	0.923	0.369
UTP15	WDR43	0.923	0.952
NR1D1	PLCB2	0.922	0
BYSL	PNO1	0.921	0.994
DDX49	UTP15	0.92	0.093
DNAJB11	DNAJC3	0.918	0
AMBP	FGB	0.917	0
DDX49	WDR43	0.917	0.152
NDUFB8	NDUFS7	0.916	0.91
APOM	FGB	0.914	0
COL1A1	COL6A1	0.914	0
BYSL	DDX49	0.913	0.25
AMBP	APOH	0.911	0.056
IFITM2	IFITM3	0.892	0
LSM5	SNRPF	0.891	0.991
BYSL	WDR43	0.888	0.928
NDUFS7	NDUFV2	0.888	0.995
BOP1	BYSL	0.887	0.103
ATP5D	NDUFV2	0.886	0.904
BOP1	DHX37	0.882	0.06
BYSL	UTP15	0.879	0.933
CHTF18	PRIM2	0.878	0.142
NDUFA7	NDUFB8	0.873	0.901
BOP1	WDR43	0.872	0
POLA1	POLE2	0.87	0.369
COX8A	NDUFB8	0.869	0
MPHOSPH10	WDR43	0.869	0.937
PNO1	WDR43	0.867	0.559

IMP3	PNO1	0.866	0.559
PNO1	UTP15	0.866	0.581
BOP1	UTP15	0.865	0.057
NDUFA7	NDUFS7	0.865	0.973
MRPL27	MRPL54	0.862	0.904
ATP5D	NDUFA7	0.861	0.368
ATF3	FOS	0.854	0.271
RIOK2	UTP15	0.854	0
BYSL	DDX47	0.853	0.14
DDX49	PNO1	0.853	0.358
BOP1	DDX47	0.852	0.76
BOP1	MPHOSPH10	0.851	0
DDX47	DDX49	0.85	0
DDX49	IMP3	0.85	0.07
BYSL	POLR1C	0.849	0.272
DNAJC3	MANF	0.847	0
POLR1C	TWISTNB	0.846	0.985
PSMB10	PSMB9	0.846	0.963
BYSL	DHX37	0.845	0.824
DHCR7	LSS	0.845	0
AHSG	ITIH2	0.841	0
PNO1	RIOK2	0.84	0.984
BYSL	IMP3	0.837	0.937
ASS1	NAGS	0.836	0
BYSL	MPHOSPH10	0.836	0.938
DDX49	DHX37	0.836	0.161
DDX47	WDR43	0.835	0.152
POLE2	PRIM2	0.835	0.578
BYSL	PINX1	0.833	0.781
MRPL27	MRPL49	0.833	0.976
MPHOSPH10	PNO1	0.832	0.584
AHSG	FGA	0.83	0.086
DDX49	RRP15	0.83	0.16
DDX51	DHX37	0.83	0.161
MRPL27	MRPL57	0.83	0.818
BYSL	RIOK2	0.829	0.984
DHX37	MPHOSPH10	0.829	0.937
BOP1	RRP15	0.828	0
CHTF18	POLE2	0.828	0.788
DDX49	MPHOSPH10	0.828	0.165
DDX49	PWP2	0.828	0.167
BOP1	DDX51	0.827	0.245
HGD	HPD	0.826	0.244
BOP1	IMP3	0.825	0
BOP1	RBM34	0.825	0
DHX37	WDR43	0.825	0.585
MT-ND1	MT-ND3	0.825	0.944
DDX47	IMP3	0.824	0.07
MPHOSPH10	UTP15	0.823	0.975
MRPL14	MRPL27	0.823	0.822
PWP2	WDR43	0.823	0.787
FCF1	PNO1	0.822	0.559
MRPL1	MRPL27	0.821	0.921
IMP3	WDR43	0.82	0.937

MRPL14	MRPL49	0.82	0.838
DHX37	UTP15	0.819	0.58
BOP1	PWP2	0.818	0
NDUFB8	NDUFV2	0.817	0.901
RPL39L	RPS26	0.816	0.787
FCF1	IMP3	0.813	0.815
PWP2	UTP15	0.813	0.787
RPL39L	RPS28	0.813	0.787
AMBP	ITIH2	0.811	0
PSMA2	PSMB9	0.811	0.994
HLA-DMB	HLA-DRA	0.81	0.963
CXCL1	CXCL2	0.809	0
DHX37	PWP2	0.809	0.712
BYSL	PWP2	0.808	0.839
PSMA1	PSMB9	0.808	0.994
ATP5D	ATP5L2	0.807	0.787
RPL39L	RPL9	0.807	0.787
PSMA1	PSMB10	0.805	0.985
SNRPB	SNRPF	0.799	0.994
ASL	ASS1	0.798	0
IFIH1	IFIT2	0.793	0
IFIT2	OASL	0.793	0.16
PNO1	PWP2	0.79	0.556
COL1A1	COL5A1	0.779	0.192
OASL	USP18	0.779	0.172
DHX37	PNO1	0.773	0.577
MT-ND3	NDUFS7	0.756	0.943
MPHOSPH10	PWP2	0.755	0.929
RPL36A	RPS28	0.755	0.952
CXCL8	PTGS2	0.753	0
ORC4	POLA1	0.748	0.056
RPL36A	RPS26	0.748	0.952
IMP3	UTP15	0.736	0.815
RPL36A	RPL9	0.732	0.952
PPAN	RRP15	0.719	0.821
BOP1	UTP20	0.711	0
MRPL54	RPL23L	0.708	0.864
CXCL2	CXCL3	0.705	0
NDUFA1	NDUFB8	0.704	0.844
PSMA2	PSMB8	0.704	0.993
EIF1AX	RPS28	0.703	0.972
POMP	PSMA1	0.702	0.938
BOP1	PPAN	0.701	0.347

network.

combined_score
0.999
0.999
0.999
0.999
0.999
0.999
0.999
0.999
0.999
0.981
0.999
0.994
0.974
0.957
0.977
0.955
0.938
0.934
0.999
0.983
0.98
0.994
0.96
0.946
0.991
0.999
0.922
0.999
0.989
0.944
0.937
0.969
0.999
0.931
0.956
0.98
0.956
0.988
0.999
0.992
0.999
0.926
0.995
0.988
0.996
0.977
0.999
0.987
0.998
0.944
0.998
0.949

0.955
0.965
0.99
0.999
0.997
0.949
0.91
0.933
0.901
0.942
0.996
0.988
0.971
0.949
0.902
0.904
0.999
0.999
0.987
0.967
0.905
0.999
0.99
0.988
0.993
0.995
0.939
0.992
0.961
0.999
0.937
0.913
0.957
0.939
0.993
0.999
0.997
0.935
0.982
0.964
0.972
0.948
0.998
0.988
0.917
0.971
0.999
0.931
0.999
0.993
0.996
0.962
0.998
0.998

0.993
0.983
0.986
0.999
0.966
0.991
0.988
0.978
0.977
0.999
0.999
0.96
0.988
0.985
0.999
0.984
0.971
0.999
0.999
0.999
0.936
0.994
0.957
0.955
0.915
0.925
0.999
0.998
0.999
0.943
0.985
0.999
0.987
0.994
0.982
0.977
0.975
0.939
0.997
0.999
0.996
0.996
0.92
