# Supplementary material for: Insight of a Metabolic Prognostic Model to Identify Tumor Environment and Drug Vulnerability for Lung Adenocarcinoma
Source: Front Immunol. 2022 Jun 23;13:872910. doi: 10.3389/fimmu.2022.872910 (PMC9262104; doi:10.3389/fimmu.2022.872910)
Supplement: Supplementary file 6 [file DataSheet_5.pdf]

Supplementary Table S5: The expression of 192 common elements in 'MRGs' and 'DEGs'.

id	futime	fustat	ACADL	ACADS	ACER2	ACP2	ACPP	ACSL1	ACSS3	ACYP1	ADCY1	ADCY3	ADCY5	ADH1C	ADH6	ADSSL1	AGPAT4	AK7	AKR1C1	AKR1C2	AKR1C4	ALDH1A2	ALDH2	ALDH4A1	ALDH5A1	ALDOC	AMDHD1	AMPD3	AOC2	AOC3	AOX1	ARG2	ARSA	ASL	ASMT	ASNS	ASS1	BDH1	BDH2	BPNT1	BST1	CA8	CA9	CBS	CD38
TCGA-05-4244-01	0	0	3.7244	9.9814	5.5991	10.1548	6.0428	11.231	5.6198	6.1811	7.0509	10.1263	4.4558	3.5201	3.9366	7.2226	6.7664	7.1357	6.9729	6.3199	1.2608	5.8023	11.9535	8.0068	9.3104	9.8102	1.7839	8.7114	5.1521	9.0177	6.058	8.3373	9.6198	10.276	0.7639	9.5848	11.2748	7.953	8.4842	9.8819	6.8904	2.4693	4.1688	7.741	5.7091
TCGA-05-4249-01	1523	0	2.8951	9.931	6.4154	9.6976	6.2421	10.8782	5.8088	4.8902	7.1268	9.6218	9.7324	6.2236	2.6209	6.833	6.7448	5.5948	6.6873	4.9057	0	5.5056	12.4117	8.7734	9.6852	9.8131	2.3745	9.586	4.7244	9.8876	7.6552	6.9309	9.0887	9.7476	1.194	8.6993	12.1778	7.606	7.9407	10.0361	6.1272	6.5101	6.9784	5.9656	6.7141
TCGA-05-4250-01	121	1	4.5777	8.9859	4.9313	10.0927	6.4132	10.5746	7.1045	6.037	7.5749	10.0077	4.4522	3.9626	4.1358	6.3849	7.5239	5.7739	7.0442	4.834	0	6.2902	11.6087	7.935	9.5003	9.975	1.869	8.5095	3.656	10.0194	5.7114	7.8744	9.5948	10.9835	0.4132	10.4485	9.8216	8.4936	8.5419	9.5212	6.4897	3.4526	9.0431	9.802	7.1624
TCGA-05-4382-01	607	0	2.408	9.1736	6.4922	10.6925	7.1734	11.3301	6.1177	6.5596	6.2776	10.1519	3.4345	4.0699	1.9529	8.2477	7.6026	3.497	6.7986	6.6857	0.9688	9.2073	10.9278	8.9115	9.0618	9.6666	3.8953	9.7419	4.5977	11.1084	7.6463	7.5828	10.3008	9.9278	0	8.8166	10.3212	7.2838	9.158	9.2126	8.8854	2.6472	7.6246	7.9159	7.6255
TCGA-05-4384-01	426	0	6.8175	9.4433	6.039	10.2253	7.7469	12.4388	7.5015	4.8323	5.9562	10.2627	5.1126	5.5676	1.5021	9.3918	6.8484	6.3184	16.1024	16.078	1.1517	6.3726	12.6275	8.7186	10.348	9.2728	5.3032	8.5455	3.9691	11.2365	7.2756	7.3272	10.3138	9.6195	1.5021	8.1917	10.8245	9.5209	8.9176	9.9477	6.7454	6.4452	2.056	8.3913	7.6754
TCGA-05-4389-01	1369	0	3.5113	9.7489	7.4663	10.5861	7.7953	11.5787	11.5435	5.7006	6.5279	10.1344	3.5113	12.0388	9.6207	7.5795	6.559	5.4357	16.4159	16.452	6.4133	10.8792	12.6547	9.0078	9.1329	9.3818	2.7452	9.0775	2.4229	7.9657	6.3612	10.5139	10.3807	10.7536	1.8815	9.8197	11.5128	9.5345	8.2027	10.3187	6.2059	7.5358	4.2341	8.9745	6.8425
TCGA-05-4390-01	1126	0	0	8.1623	5.838	10.8568	6.6064	11.7918	7.7734	6.7503	4.955	9.9206	6.2548	1.544	0	8.7973	7.7692	4.8943	11.505	12.1165	1.8297	8.5444	10.4337	7.8312	8.3177	9.3023	0.7126	8.9671	2.0681	10.0092	10.2529	10.1946	10.8785	10.9037	0	9.4973	6.5681	8.5785	8.5144	9.5982	7.0154	2.0681	9.7995	9.2832	6.436
TCGA-05-4395-01	0	1	2.4046	9.6638	4.6985	10.28	7.1788	10.1575	2.6089	6.2662	4.0965	10.1705	1.7723	0.62	0	10.8194	7.1867	3.3401	11.9646	13.7949	0.343	3.2615	11.3541	8.0995	9.4396	9.3623	6.3822	9.7319	6.3445	8.4467	7.5199	7.9352	10.1061	11.2338	0.343	9.9586	13.4527	6.2459	7.4104	10.9268	5.5012	8.3263	10.7098	6.3493	5.9018
TCGA-05-4396-01	303	1	5.1026	9.7173	5.0375	10.283	8.447	12.4607	4.7015	3.4404	5.6519	9.325	3.336	10.1458	7.9639	9.1729	7.2539	5.3098	9.7684	10.2447	6.6153	5.4415	13.3307	8.0735	9.831	7.4164	4.2627	8.6809	3.1012	8.9632	10.9638	7.4737	10.6424	10.7262	0	8.5816	12.2494	9.8886	8.7713	9.8955	4.144	6.16	6.7333	8.6238	7.1879
TCGA-05-4397-01	731	1	5.6448	9.8939	4.8823	10.2946	8.0435	13.0933	7.4534	8.3493	6.6142	10.8016	7.4072	7.0893	2.1808	8.3454	6.995	5.7433	7.8513	6.6463	1.2174	8.8118	11.4872	9.1863	9.0695	9.4326	2.931	8.5488	2.6556	7.0562	5.1835	8.4632	8.9846	10.3734	0.2879	9.0151	13.2742	11.0482	8.3405	10.4334	8.402	3.0122	9.3766	9.9967	5.9988
TCGA-05-4398-01	1431	0	2.0161	9.2725	6.1055	10.3047	8.6737	11.5247	4.9649	5.5777	5.4107	10.0515	5.3809	1.4541	0.903	8.1605	7.4439	5.7155	4.9344	4.701	0	5.0899	10.4845	8.9027	8.5898	9.5924	1.7623	9.5462	4.9344	9.5761	6.0779	7.9983	10.6699	11.4717	0.7247	11.0403	13.4737	7.3237	7.9533	9.1003	6.7315	1.9363	5.2476	10.1278	7.6993
TCGA-05-4402-01	244	1	5.7698	9.2981	6.0156	10.7543	6.8946	11.6904	8.5891	5.4256	5.1789	9.5649	3.7598	5.8803	3.0839	9.1515	7.4978	7.4765	9.033	10.8583	1.427	7.4268	11.6384	8.6987	8.9705	8.6954	3.7339	9.2325	2.7113	10.2451	6.7403	7.7526	10.9669	10.6619	0	9.0697	11.7424	8.4185	9.0586	9.9561	6.6771	3.0423	9.3219	7.311	7.2755
TCGA-05-4403-01	578	0	6.0149	9.8032	9.9025	10.5219	7.0975	10.9631	6.5986	4.8199	4.0011	9.6892	4.2947	6.5308	5.2364	6.3558	7.5702	6.1844	10.454	10.7906	4.1441	6.7329	12.7422	8.5864	8.9739	8.6204	3.1799	8.8533	3.1799	10.5106	6.5566	7.5004	10.1174	11.5004	0.3539	7.3568	13.2282	9.2055	8.8884	9.9319	6.9267	6.4957	10.0046	7.5165	6.4868
TCGA-05-4405-01	610	0	4.0792	9.1024	6.2829	9.8652	6.9571	10.5462	6.2073	4.2396	6.3894	9.7289	5.4399	3.6924	1.8681	7.3576	6.6495	5.148	7.0247	6.043	0	7.8596	11.3186	8.7975	9.1282	8.4356	2.931	8.7316	4.1347	10.9731	6.6685	7.497	10.306	10.3255	0.7334	9.0311	12.6454	8.4245	8.6672	10.0325	5.9063	7.0752	2.7381	10.5834	6.412
TCGA-05-4410-01	0	0	3.1119	9.2821	6.1566	10.3357	8.4716	11.5922	5.5506	4.3301	8.4993	9.0418	5.6853	6.5112	4.4358	7.5974	6.0607	7.0331	7.6422	4.3301	0.8193	5.4021	12.9468	9.3747	10.2941	9.5615	1.3386	8.7141	2.9783	10.0907	6.6384	7.2456	10.8994	10.0413	0	8.3884	11.186	8.4497	7.7841	9.855	5.7483	5.922	9.4337	7.4475	8.395
TCGA-05-4415-01	91	1	4.01	8.4923	7.0697	10.496	3.7004	9.0652	3.7959	5.6534	6.8921	10.1357	1.4739	1.2224	1.8745	8.7977	7.5175	7.7074	6.8427	6.2416	0.5305	4.1964	11.7778	8.555	8.0786	6.3789	10.418	6.7799	3.0969	7.0407	7.4856	7.8766	8.6378	8.6586	6.7397	9.814	12.8914	6.0613	7.4963	10.047	4.9015	5.8102	6.0996	9.9634	11.5381
TCGA-05-4417-01	455	0	2.8785	9.0279	6.3633	10.8986	6.7257	10.9934	8.4452	5.5271	6.7343	9.2963	6.2832	2.4456	1.1832	8.6255	7.3601	6.0541	10.6412	9.7965	1.5391	6.8421	11.5471	7.7405	9.0332	6.6085	2.0624	8.3669	3.7084	11.0254	7.3545	8.6906	11.1087	8.8206	0.7096	9.0609	10.9662	7.3433	8.1517	9.782	6.8815	4.9478	3.7	8.9578	7.7951
TCGA-05-4418-01	274	1	3.9961	11.4078	4.7329	10.7328	5.4553	9.3342	4.8204	5.8785	4.0258	10.7776	2.1122	4.0835	7.275	9.522	6.8825	6.1251	12.4118	14.2254	3.1662	4.4554	12.695	8.577	9.7197	8.1008	5.3882	9.326	6.033	9.2158	4.6589	10.9947	8.8085	11.1567	0.9979	9.2595	13.6416	9.7373	7.7706	9.5478	6.63	7.0939	5.7529	6.6493	8.7101
TCGA-05-4420-01	912	0	0	9.7044	5.5389	10.2069	8.9592	10.8733	9.0812	6.6382	5.1151	10.8892	2.2269	1.6339	1.5062	8.5403	5.8854	4.5616	6.561	7.0323	0.3369	7.2867	11.8422	8.4214	10.1486	7.8072	3.6519	8.1143	6.9488	9.0579	6.9488	8.7925	11.2092	9.1185	3.4584	10.5751	11.2774	9.552	8.2274	9.5993	6.4118	2.7623	6.4887	10.6296	6.0547
TCGA-05-4422-01	365	0	7.7421	10.3593	6.5983	10.3816	7.677	11.2469	8.1884	6.0409	4.4192	8.999	3.5046	11.718	8.23	8.888	7.4193	7.1768	14.7489	14.3299	12.2513	2.8861	13.6437	7.7026	10.4122	7.2736	5.4963	9.5989	2.5535	7.1737	5.4668	7.7454	11.3945	10.8243	2.0169	9.7534	12.1182	9.1021	9.4091	9.6522	6.797	5.1456	6.3704	8.4631	9.1654
TCGA-05-4424-01	913	0	6.7325	8.148	6.8799	10.4304	7.729	11.8433	8.7577	4.6341	8.0911	10.017	4.9332	8.5965	2.5277	8.431	6.9088	6.8799	9.1197	9.8816	0.5623	10.1184	12.4863	9.6926	9.7247	10.3615	3.1087	9.096	5.342	11.6098	7.0556	9.4078	9.7263	9.6522	0.5623	8.8395	11.1365	9.3447	8.518	9.659	8.2821	7.7451	1.7584	8.8153	6.8916
TCGA-05-4425-01	669	0	0.6412	10.3165	7.7504	10.3222	6.8048	10.9268	5.4063	5.4623	4.1528	10.2851	2.7216	11.5202	3.1431	6.8957	6.4915	2.9477	7.693	5.3481	0	3.3152	13.2713	8.1448	9.1873	10.9193	4.4767	9.3056	4.0591	7.5014	4.2409	7.0926	9.7453	10.5358	0.6412	9.4089	11.5518	5.8738	9.642	10.035	6.8048	1.6953	9.8347	7.6773	5.8032
TCGA-05-4426-01	791	0	7.5296	8.9281	6.7229	10.4088	8.2363	11.1078	6.1904	6.3973	7.8351	8.8826	2.5217	3.9002	1.3115	9.0477	7.0702	6.1306	6.8093	4.6948	0	4.6098	11.1021	7.7758	9.6836	12.2421	4.9374	8.4148	5.157	8.7319	6.735	7.6491	10.4716	9.0436	0	8.7647	12.4377	7.0282	10.1448	9.66	6.4858	3.0204	12.5932	6.106	3.7173
TCGA-05-4427-01	791	0	3.4041	8.7993	6.1421	9.729	7.3213	10.3219	5.2415	5.7543	4.5671	9.9091	2.8185	1.5937	0.5893	5.671	6.734	3.099	5.1624	3.8701	0	5.057	10.9338	8.388	9.0965	8.8155	6.3438	7.9614	4.7939	9.1176	5.0126	9.6402	9.071	9.0017	0	9.6185	11.8276	9.0409	9.2574	9.3338	6.2703	2.1801	2.5543	9.898	6.4723
TCGA-05-4430-01	761	0	5.8995	8.6325	6.9519	10.1849	6.2831	10.3007	7.1102	5.26	7.8296	9.8371	5.5128	5.9072	4.8456	8.3975	7.3032	7.2767	8.9158	6.8479	0.3994	6.8359	11.6436	8.9291	9.2205	9.2442	2.0669	9.0872	3.7485	10.9927	8.4325	8.2488	9.9732	10.0009	1.543	9.1256	12.4868	9.2668	9.083	10.0838	7.3547	5.272	7.0797	9.1281	7.0935
TCGA-05-4432-01	761	0	4.657	8.3207	4.9674	11.0619	6.4753	11.8968	7.7288	6.0547	8.5355	10.358	5.6531	6.808	2.2478	8.5622	6.4985	5.1543	11.3337	11.5157	2.1574	5.8171	12.7213	9.1982	8.0128	9.1967	2.8191	8.6456	3.1821	9.792	6.4422	8.7718	10.6768	9.1939	2.5623	9.077	10.1343	8.4876	7.7714	9.8454	6.338	6.708	9.9619	9.109	8.082
TCGA-05-4433-01	730	0	6.3862	8.5867	10.0415	10.2605	7.9109	10.8192	7.2964	5.3459	4.9809	9.9369	4.3809	4.5431	3.0769	5.919	7.4407	6.7497	9.8914	9.5455	5.879	5.286	11.9019	8.7284	8.7863	9.2568	4.7344	8.533	3.6519	10.4925	6.4004	7.3407	9.6156	10.6684	0.869	8.6885	13.2256	8.6622	8.5005	9.9027	7.2105	5.0899	9.0823	8.0114	6.3574
TCGA-05-4434-01	457	1	3.5944	9.0056	6.9087	10.5667	6.6955	11.1974	5.6521	6.3684	7.0209	10.2951	5.9813	8.5366	3.6474	8.5798	8.375	6.5359	12.2976	11.5232	5.5233	6.0713	11.8549	7.9938	9.5664	8.9361	4.0133	9.5134	4.5334	10.5531	7.3577	8.2119	9.8395	10.3988	1.2366	9.4625	12.9029	9.1928	8.3877	9.5911	6.8923	6.449	8.4522	9.334	8.0771
TCGA-05-5420-01	457	0	4.9324	9.2347	5.3672	10.7425	6.164	11.3005	7.1898	5.6504	3.439	8.7282	2.1954	7.0603	7.3487	7.2247	6.0123	6.2682	9.7812	7.6287	7.555	5.2017	12.1885	8.395	9.8217	8.3989	6.381	9.4271	2.861	8.5408	8.1167	7.9811	9.8514	10.8051	5.3672	10.0714	12.4172	8.5339	9.8791	10.8059	8.7261	7.1079	2.3803	6.7362	9.7335
TCGA-05-5423-01	151	0	6.7641	10.2553	6.1858	9.8701	7.4421	11.5335	5.2889	6.1265	3.2915	9.5707	3.5836	5.7869	4.2159	6.6318	5.625	6.9636	8.1109	6.1113	0	5.5593	12.9136	7.6352	8.9591	10.2674	2.2213	8.8117	2.2213	9.4971	5.3678	9.3169	9.9619	10.7614	1.3017	9.3083	12.059	8.8962	9.3558	11.182	8.0079	3.4927	5.3678	7.4421	8.3724
TCGA-05-5425-01	882	0	6.3948	9.5913	4.9266	10.5956	7.2373	10.6611	6.9178	6.4236	4.0243	10.4318	7.3416	8.5676	7.8245	7.2588	6.4236	4.9792	9.6835	8.1772	2.7347	5.617	12.2092	9.2517	9.7757	7.9748	4.3385	8.6183	3.7488	9.3575	6.7384	8.77	10.3564	11.2706	1.7059	9.5796	13.3765	10.0066	8.7069	9.9166	7.1423	5.4956	5.4627	9.5007	8.871
TCGA-05-5428-01	670	0	1.8544	9.9625	6.5324	8.6855	7.6675	10.4652	5.3306	6.6869	4.2048	10.3821	6.0341	5.7831	0	7.6426	5.4215	5.2336	15.8931	16.0955	1.4563	4.8532	11.7447	7.9731	8.524	10.5313	4.809	8.2584	2.1661	7.9479	4.3962	8.0483	8.6547	11.639	0.9046	9.3361	12.8424	7.0518	10.2299	11.5292	7.2631	5.0559	4.9862	10.5491	5.7601
TCGA-05-5429-01	275	1	2.6812	10.2304	9.1076	9.7237	7.4953	10.5647	5.1928	7.3248	3.8225	8.4178	3.8993	9.3406	8.854	4.9255	5.785	7.9615	10.1889	7.8007	4.9255	9.6041	13.0172	6.9894	7.966	10.5543	1.7313	7.7236	1.3487	9.0708	4.4517	7.6724	9.8847	12.2562	0.8265	7.9969	13.9203	9.9162	10.0273	11.9902	7.1394	6.3475	12.7602	5.223	5.4442
TCGA-05-5715-01	62	0	4.303	9.4889	6.7228	10.8075	6.8057	12.4819	6.5168	5.1754	5.6267	9.1124	2.8572	7.716	5.4576	6.8157	7.0716	5.4317	5.3786	3.3743	0.8325	9.4936	12.7376	9.0359	8.8531	10.7498	2.5071	8.9497	3.0049	10.155	8.2661	7.2692	10.7576	9.7752	0	8.5537	12.2297	8.9011	8.2477	10.5426	8.7444	4.3589	4.5935	6.0906	4.3589
TCGA-35-3615-01	14	0	3.995	9.4468	5.6825	9.3467	6.8782	10.1174	4.7717	6.1729	6.4499	8.3241	3.6663	7.8023	3.0828	8.6887	5.8785	3.935	16.8452	16.172	1.2013	5.6549	12.0085	9.9766	10.4691	6.9058	5.8133	8.639	4.334	8.6773	4.855	8.1356	9.6349	10.8905	0.9816	8.8967	13.0647	8.751	9.1935	10.1802	6.4817	8.1347	1.1579	6.0859	9.2423
TCGA-35-4122-01	225	0	4.0512	9.7688	4.9139	10.1759	6.3381	10.7457	6.4165	4.7619	3.3989	9.7168	6.8016	2.0054	5.1568	6.499	7.4584	4.1768	8.9119	7.0072	2.4653	5.3108	11.7504	7.8242	8.0434	9.2191	3.0938	8.5675	3.3989	9.5071	6.3291	8.7128	9.4543	10.4748	0.5874	11.6867	10.9453	9.0268	9.0379	10.1946	8.6366	3.8647	8.8171	9.2949	8.513
TCGA-35-4123-01	182	0	1.8618	9.2569	5.1574	10.4812	5.3406	8.8642	5.0846	7.0713	2.8374	10.2272	1.4628	1.676	2.1741	8.8992	6.641	5.6864	5.1215	4.968	0	3.8251	10.8882	7.8496	7.9529	10.0953	3.8691	8.4624	3.0046	8.6108	4.6775	8.3124	9.9453	11.3934	2.1741	9.6817	11.9116	7.4575	8.7774	10.3309	7.3873	1.8618	5.5898	9.7757	7.4099
TCGA-35-5375-01	264	0	6.7637	8.6736	6.0788	10.5056	8.5075	8.9445	4.8453	8.7794	4.6033	9.4971	0	14.8363	0	6.7188	5.5393	5.1459	16.8936	15.762	1.0772	6.8495	12.4268	8.9861	7.6965	8.2106	2.4435	7.3093	3.7234	8.6135	7.7119	9.5965	9.31	9.7182	0	10.2417	11.0276	9.2	9.9608	10.0526	6.6082	4.4652	7.8491	8.5551	7.2154
TCGA-38-4625-01	2973	0	5.0489	8.2758	4.3327	10.4788	8.5807	12.1236	5.5688	6.0332	3.4774	10.4233	2.1307	3.0072	1.0884	6.0939	8.1412	4.4856	5.7905	4.852	0	4.1155	12.2456	8.4555	8.2875	9.1658	3.9146	8.8044	4.9862	9.4713	7.4139	8.0557	10.6762	11.4642	0	10.6203	10.4556	9.631	9.5994	10.6623	6.7475	2.461	12.6838	10.639	7.8505
TCGA-38-4626-01	3674	0	8.5055	9.2665	6.2065	11.3242	8.526	13.6137	9.5565	5.4663	5.9614	10.6708	6.3614	5.7532	2.2078	6.6995	9.3399	6.4831	9.0601	6.3614	0	8.6915	12.6591	9.3811	9.4015	8.4346	5.6334	8.9854	4.4471	11.6307	8.7033	7.984	9.6415	10.4691	0.7473	8.8541	11.5449	8.2763	9.8648	9.5818	8.5118	6.0027	2.5289	10.0443	7.7931
TCGA-38-4627-01	1147	1	4.6058	8.6271	6.0431	10.6876	6.6314	11.5533	8.0565	4.2661	7.1701	9.8004	6.2088	7.2621	4.0288	7.4812	8.1815	6.0108	11.5463	9.0696	0	9.4193	12.1387	8.3762	7.7121	10.1404	4.3198	9.3066	3.0525	12.3372	8.4742	9.4266	10.5638	9.5977	0	9.7685	10.5512	6.6681	9.8889	8.6535	8.7965	6.9536	8.2218	8.7174	6.2439
TCGA-38-4628-01	1492	1	0.7067	10.4024	6.4159	10.5459	7.6424	11.8456	5.5686	5.9976	4.7839	10.297	8.7793	3.7362	3.3001	6.6833	5.9468	5.5297	5.7908	6.1467	0	11.0996	12.9314	9.8639	9.2072	10.1234	1.6837	8.4831	2.2608	10.0936	7.6007	8.5618	8.955	10.9108	0	8.9309	12.2807	9.4469	8.4869	9.809	8.1539	1.179	4.1223	9.5605	3.253
TCGA-38-4629-01	864	1	0.3811	8.4716	3.8978	10.8708	6.2184	10.6344	6.1888	5.5058	8.2126	9.8265	2.2103	5.447	0	7.7443	7.5555	4.5278	6.3525	3.9552	0	5.0061	11.7221	8.4076	8.6153	10.9441	3.4563	8.8067	3.7435	10.1023	6.1888	8.5331	10.4866	11.1354	0	9.6068	9.4278	8.151	8.9009	9.1143	7.322	1.6397	10.0992	9.253	7.2721
TCGA-38-4630-01	1073	1	4.1023	8.4291	5.5708	10.2711	5.6301	10.5234	7.5766	6.6024	7.0487	9.7833	9.1963	5.05	1.22	7.6389	9.3831	7.2279	2.7092	2.3813	0.2888	3.0932	11.6002	8.9722	7.9864	9.3135	2.2531	7.272	6.8662	7.8379	4.1925	7.9018	9.2023	9.5034	0.2888	10.1106	10.478	9.1854	9.9888	9.6673	5.8288	6.229	9.2573	9.9502	5.4296
TCGA-38-4631-01	354	1	0.8358	10.5709	4.4198	9.7761	8.1828	10.4021	8.3792	7.0955	5.0858	9.8401	7.0015	2.5131	3.5834	6.0644	6.813	6.3124	15.0448	16.485	7.5589	7.2373	12.3148	8.8556	8.255	10.3379	6.823	7.0872	5.1187	8.8702	7.3317	7.9032	9.5196	11.5577	0.8358	12.4184	8.9774	9.518	8.564	10.1554	8.7438	9.6165	3.0812	8.7596	7.1993
TCGA-38-4632-01	1357	1	3.1121	9.9159	5.6856	9.4192	6.0443	10.7006	5.294	5.6198	3.8073	9.2414	4.2744	5.2071	4.7951	6.0774	5.2367	5.1146	7.8384	5.664	0	2.9786	11.9853	8.392	7.7591	6.8058	1.3387	8.7352	3.5491	8.3887	6.0105	8.8608	10.0056	10.4719	0.8194	11.1772	12.3246	9.1312	8.6278	11.073	7.131	2.6674	3.334	11.142	7.4158
TCGA-38-6178-01	448	0	4.741	8.5732	5.5066	10.2136	6.7321	11.5542	6.3459	5.6023	6.6448	9.4491	5.735	7.2136	3.6668	8.272	6.4781	4.7825	6.4781	4.9002	1.3562	6.3459	12.1779	9.5186	9.4332	9.3356	1.7399	9.2233	3.7528	10.5659	6.4654	9.3993	9.5845	9.9537	0	10.3638	12.7659	8.1306	8.8687	9.9795	6.2605	6.2605	9.946	10.8328	8.488
TCGA-38-7271-01	800	1	5.9304	8.8154	6.0175	10.4104	6.3076	11.369	7.6813	4.3745	6.0674	9.8321	6.4893	3.631	0	6.5134	7.175	4.4263	9.1789	7.8942	0	5.6099	12.3662	8.1547	9.5819	8.9095	3.4424	9.4409	4.3745	11.8605	9.9363	7.5452	9.9318	9.5472	0.815	8.5106	12.0917	7.7181	9.3598	9.6652	6.1774	4.7036	0	7.6157	8.0594
TCGA-38-A44F-01	133	0	7.2771	9.2527	5.2943	10.5105	5.7872	10.502	6.3584	7.5475	4.8845	9.4127	5.8313	6.9968	6.4174	7.4891	8.291	8.5347	9.507	7.5779	2.4767	7.1783	13.041	8.1903	9.1807	8.9287	3.2573	9.4715	4.504	10.9669	7.2982	6.2481	11.3163	10.4316	1.9463	9.3904	10.71	8.2618	10.2201	9.6316	7.0782	5.9291	10.4735	7.1262	7.7369
TCGA-44-2655-01	1324	0	6.7851	9.7542	5.6497	10.0166	8.0005	11.447	6.9929	4.5878	5.8765	9.0623	4.9917	5.8892	2.7983	8.828	7.1003	6.0835	15.6237	15.4285	0	5.7364	13.9851	9.4802	10.0086	8.2105	5.8508	8.7036	3.5057	10.4901	7.2378	7.9368	10.1196	10.2217	0	8.3022	12.406	8.9149	10.5005	10.602	7.5324	7.7318	1.9949	7.0645	9.1209
TCGA-44-2656-01	1429	0	5.3324	7.6054	6.2335	10.6062	8.5629	13.1888	6.2795	4.2596	4.9452	9.6312	3.6918	4.5869	0.8583	7.361	7.0926	4.7842	7.7577	6.1144	0	6.3192	12.7993	7.6892	10.8241	7.6154	2.6202	9.3881	3.0081	10.5284	5.6837	7.0863	8.5702	10.6075	0	9.5913	11.8177	8.0361	8.5242	10.0218	6.9979	2.7421	1.9471	9.8859	8.1939
TCGA-44-2657-01	1351	0	3.4682	8.8478	6.017	10.5504	5.6437	11.2723	6.742	6.0319	6.7953	9.6576	5.3193	8.1577	2.1229	7.4387	7.5565	6.2763	10.2476	8.0485	0	7.6607	12.2039	9.456	9.3458	9.1703	4.6141	9.2958	3.4682	11.1391	8.348	8.3077	10.8264	10.3829	0.7408	9.2328	11.4559	7.7353	9.5902	9.7359	7.0649	5.9868	5.7792	8.4148	9.384
TCGA-44-2659-01	1367	0	4.558	9.3258	6.4207	10.0999	6.1084	12.257	6.582	4.7973	7.3615	9.0222	5.9947	6.3002	2.8502	5.8053	6.6256	2.2003	9.2481	8.2136	0	6.6864	12.6877	8.0164	8.9155	9.2207	2.7833	8.0648	3.5592	11.0896	8.6862	8.5234	9.91	8.3884	0.986	10.5284	10.419	8.4903	9.4218	9.7445	6.3357	4.927	1.8462	10.1102	7.0771
TCGA-44-2661-01	1159	0	5.698	8.8817	5.8134	10.5266	6.9425	10.9424	7.3347	6.0409	6.5125	10.1106	4.1152	9.4919	4.0747	7.0871	7.9866	5.9982	9.3462	8.1321	0.5659	5.1724	12.4163	8.8263	9.8587	9.0559	4.4991	9.3387	3.0364	10.8327	8.0586	8.2273	10.4193	10.8959	1.9585	9.0559	12.2704	8.3664	10.2836	10.0836	8.4949	7.6041	6.6734	7.5273	8.0894
TCGA-44-2662-01	1280	0	1.0547	7.864	6.0831	10.5695	7.8905	13.0035	5.72	5.1918	5.2059	9.7172	2.8999	5.5862	0	8.745	8.4139	2.3075	7.6278	6.3413	0	9.9285	11.9617	8.9038	8.09	11.1467	0.4427	9.8204	3.2109	9.8037	7.3387	7.2929	10.0889	10.0498	0.4427	9.251	6.9181	9.2637	8.4994	10.3639	7.7817	0.7809	10.528	8.9703	6.8115
TCGA-44-2665-01	1301	0	2.8414	8.0868	5.155	9.9788	8.0066	11.6728	7.1961	6.1825	5.7069	8.9559	2.522	2.8414	0.9626	6.5024	8.8491	4.3871	5.6399	6.3597	0	5.8679	11.5528	7.1726	8.8828	10.1022	2.2616	9.5869	3.6848	10.413	6.98	9.0546	10.2729	9.3463	0.9626	9.5045	11.6869	8.1508	10.0997	10.9455	7.0693	3.8362	10.5052	9.2337	3.1802
TCGA-44-2666-01	97	1	6.2334	9.763	7.5144	9.4049	7.4227	12.0976	7.4574	6.134	7.7366	10.6278	4.3936	7.8189	4.8551	7.5275	6.9252	3.566	10.2107	8.18	0	5.1878	12.4724	8.0967	10.0728	9.9918	5.6619	9.5715	4.2743	10.4489	6.9252	7.6151	9.619	10.056	0.8751	8.9507	12.8017	8.5671	9.7187	10.0817	5.7651	6.3922	2.5278	9.7386	5.7871
TCGA-44-2668-01	761	1	1.0647	7.854	4.1191	10.8486	7.4136	12.4299	6.0789	6.102	6.2439	9.5192	3.323	1.2417	0.3481	8.2836	8.6675	4.073	5.5915	3.1563	0	5.7016	11.5421	6.9898	8.1651	7.3547	2.096	10.5552	4.0962	9.8235	6.6212	7.1364	10.0168	9.9996	0	8.7484	10.6242	7.5895	8.4437	9.9634	7.0944	7.0113	8.2474	9.8265	7.6476
TCGA-44-3396-01	1130	0	1.7914	8.1771	5.5506	10.8538	7.9162	12.2407	7.9202	5.7133	5.2192	10.0149	4.7038	2.0471	1.2281	6.5865	8.4524	2.2642	7.2798	8.0862	0	4.7162	11.0543	8.5049	9.0382	8.5305	4.5049	9.6504	5.1752	10.4045	7.2183	7.6699	10.1615	10.1465	0.741	9.3226	8.8234	9.3877	9.8046	9.3451	7.4684	2.0471	10.121	9.4262	8.1523
TCGA-44-3398-01	1163	0	7.3925	9.374	6.7443	10.1166	7.8219	11.069	7.2209	6.6428	5.9893	9.4818	3.1225	12.4104	2.146	6.8337	8.0581	4.7152	8.1349	6.6116	0	6.8337	11.9283	7.5623	9.3141	10.6696	4.9331	8.9791	3.9196	10.7816	7.7577	8.8947	10.3739	10.3284	1.4399	10.1513	11.8488	7.982	9.9536	10.1382	7.8301	5.1574	12.5583	7.2126	6.5861
TCGA-44-3918-01	1036	0	3.4484	8.4929	6.024	11.0279	9.2219	11.5128	5.8151	5.7139	6.9871	10.0225	3.109	1.7173	0.8179	6.2064	6.7521	6.3882	6.7001	5.2185	0	5.9898	11.0768	6.4587	9.2447	8.4096	3.3439	9.3747	3.6807	10.3703	6.4143	7.5591	9.5589	10.7532	1.1004	9.9083	11.3731	7.9879	9.5129	9.8457	8.0598	2.5748	6.4238	8.2917	8.8862
TCGA-44-3919-01	1026	1	4.2393	8.8214	7.0592	10.7701	6.7062	12.0669	6.3224	5.7724	6.2625	10.0379	7.2314	5.2872	1.7121	8.1193	7.665	6.8875	5.9574	5.2751	0	6.5039	11.5788	8.8723	9.2491	8.0467	4.4895	9.3373	4.7206	10.7515	6.9381	9.2451	8.0573	9.8843	1.3929	9.3065	11.5757	8.256	9.5042	9.4032	6.462	3.2411	10.5984	7.7194	7.4697
TCGA-44-4112-01	808	1	0.8506	9.3091	6.3808	9.9445	7.0911	11.5061	5.6321	5.7765	7.457	8.5445	3.9503	2.2062	1.3821	6.6234	7.3077	4.5291	6.2268	4.9221	0	6.5261	12.2504	7.6937	7.9978	10.8268	3.6141	8.1048	4.9598	10.2634	6.9715	7.4369	8.2298	10.7236	0.8506	9.8954	11.9147	8.3886	9.8307	10.8919	7.2631	4.3978	9.1287	7.4066	6.475
TCGA-44-5643-01	1013	0	2.6018	9.3555	6.3298	10.6478	9.4504	12.143	3.1517	5.9241	2.7298	10.985	3.2404	8.205	0	5.1443	8.6147	4.894	11.3514	10.1826	0.6447	3.0573	11.7832	9.0031	9.2393	10.5269	3.4029	8.9466	3.9679	7.2153	5.2334	8.1239	8.6373	8.354	1.4277	10.8859	13.7382	11.0113	7.2795	8.6795	4.3728	1.0887	3.8522	9.921	7.9495
TCGA-44-5644-01	863	0	1.2203	8.8346	5.098	10.7986	6.3481	9.7158	5.5305	7.1895	5.0409	7.9838	8.4213	1.2203	1.5826	6.7289	5.153	6.3002	5.8953	6.8993	3.541	5.4449	10.973	7.0225	6.9073	9.1936	4.821	7.6136	2.8043	8.1445	11.8462	10.8591	10.0248	8.3671	1.5826	11.5887	10.7599	8.3078	7.3973	8.7854	4.8545	8.3078	3.0195	9.7541	5.3304
TCGA-44-5645-01	852	0	6.3456	8.8669	6.9649	11.3276	7.2414	10.9065	5.9228	5.569	6.0779	9.025	6.4054	7.9888	1.7202	6.8332	8.6429	7.9424	9.1332	5.1698	0	5.4975	13.5462	9.0638	10.3002	8.0494	3.3476	9.8257	3.2639	11.2207	7.6403	8.1632	10.0983	10.4918	0.6542	8.1343	11.1496	7.382	9.4801	9.5424	6.1142	6.0532	7.1007	4.7139	6.9246
TCGA-44-6145-01	595	0	3.7007	8.9102	4.9979	10.6027	6.9341	10.4338	6.431	6.4204	5.4159	10.6333	3.389	6.1132	5.5581	9.6565	6.8339	4.7495	13.7192	13.2291	6.1393	4.407	12.7046	8.3558	10.0235	8.6125	2.8713	9.7119	4.0698	10.6836	7.8195	7.3522	10.3264	8.5137	0	8.3305	12.8888	9.4635	8.3585	9.7871	7.0139	3.6289	8.2892	6.2271	9.6711
TCGA-44-6146-01	728	0	6.1938	9.4549	9.2606	9.7485	8.821	10.5224	5.8878	4.7879	4.6649	8.3043	5.1779	7.4565	2.3395	5.6362	5.9628	5.3474	10.7271	9.5387	5.6877	4.5021	13.4133	8.2035	9.289	7.4189	2.5763	8.407	3.1163	10.7628	5.3313	7.0168	10.8472	10.7854	0	8.4147	12.3379	9.674	9.3817	10.0358	4.1817	8.1052	12.7688	5.8768	4.6904
TCGA-44-6147-01	845	0	5.8538	9.0527	7.0638	10.2148	6.4665	11.4655	7.2253	6.3933	7.6859	9.5996	8.0934	6.9726	6.3262	7.052	7.7678	9.3128	9.516	7.1933	0.6289	6.4486	12.5885	9.0542	9.5416	8.9702	2.0972	9.1321	4.6146	11.2538	8.8592	8.7651	9.9546	10.2854	1.0655	9.3901	11.819	9.0408	8.5831	9.5678	6.5446	5.7255	5.3255	9.3423	8.1528
TCGA-44-6148-01	704	0	7.4739	9.0241	7.5377	9.9927	6.4558	10.4032	8.9895	6.5983	5.1627	9.6959	6.5983	10.7549	7.5168	8.0991	8.6403	9.8642	10.3963	9.1881	0	3.6147	12.8026	8.9819	10.0615	9.2667	4.3423	9.9227	3.7356	10.9652	9.5055	7.9864	9.6937	10.1987	1.6521	8.1075	10.2112	8.279	10.0601	8.9337	5.9927	7.8269	9.6846	7.3703	7.9508
TCGA-44-6774-01	658	0	2.4974	7.9391	6.0658	10.5721	5.8269	11.3038	7.872	5.1067	6.5536	10.2999	7.143	0	0	5.1451	8.1893	3.8085	6.7532	5.0673	0.9482	10.9014	10.6308	8.3376	8.6922	8.1568	3.2272	8.6157	3.9012	11.8005	7.9282	8.8948	10.709	9.252	0	9.4624	10.1647	6.9064	8.7463	9.2028	8.8282	5.8269	4.8418	9.7648	7.872
TCGA-44-6775-01	705	0	6.1429	7.8114	5.7949	10.7836	6.5911	12.5654	8.5587	5.8839	5.8079	10.2729	4.9202	7.3887	2.9188	6.7685	8.5741	6.5986	8.3691	5.5833	1.5942	10.0798	12.3292	8.1609	8.7902	9.6888	4.1829	9.7185	3.0996	10.3514	7.3974	8.2169	10.1045	9.6967	1.3302	8.2899	11.475	8.5702	9.2085	10.239	7.9445	5.9561	9.5109	7.2711	7.9055
TCGA-44-6776-01	2616	0	8.1717	9.5471	4.7856	10.6066	6.5606	11.4706	5.3144	4.9951	5.0894	8.6278	4.0078	10.6811	0	8.6659	6.8573	4.4002	14.4988	14.1082	0	5.9047	13.0751	9.7521	10.9472	7.3391	3.0328	8.4984	3.2661	10.5878	6.6967	7.451	10.6747	10.0448	0	7.198	11.6387	8.5546	8.6812	10.0161	4.1891	8.0635	3.2324	6.6145	8.9728
TCGA-44-6777-01	987	1	2.9706	8.6315	5.5953	10.8277	7.5526	12.0909	6.9368	4.2392	7.6159	10.3794	5.7723	1.3663	2.5196	8.5689	7.9948	3.8755	7.0783	6.6499	0	8.6655	11.6462	7.8706	8.8112	11.1018	3.9263	9.622	3.3136	11.8605	7.8899	7.8523	10.5377	9.5305	0	9.7061	10.0512	7.4127	8.2603	9.0068	7.5925	2.0553	7.4054	9.6628	7.1226
TCGA-44-6778-01	1864	0	7.4021	8.2938	6.6976	10.8203	6.7997	11.6136	7.8812	6.7324	10.1182	9.9852	5.1911	5.7288	2.9963	7.0891	8.0217	6.4686	13.0529	11.8357	2.2652	6.5685	11.9054	8.1204	9.003	8.9958	3.6347	9.346	5.3788	12.0452	7.8578	8.3614	9.6356	9.0172	0.7088	9.7474	9.8698	8.4564	9.0658	9.7737	7.1803	7.3637	2.5226	7.7385	8.9204
TCGA-44-6779-01	500	1	0	7.3748	5.2948	11.0215	5.4343	11.1865	4.0128	5.2652	5.4865	9.8328	1.7617	1.7617	0	10.1866	7.6964	4.5917	13.4844	14.4999	0	6.1525	10.2294	6.9878	9.6856	8.251	3.2879	9.9455	4.3311	9.7983	9.3384	6.8853	9.6631	9.767	0	7.8882	11.9943	5.7006	6.9134	10.2023	6.4442	1.3751	8.9888	5.1404	8.4515
TCGA-44-7659-01	691	0	3.6958	10.2807	6.17	9.7726	7.0983	10.4848	3.9953	5.7959	7.2827	8.3359	3.926	6.243	5.7764	4.8778	6.9404	7.8436	8.5467	7.4646	0	10.0243	13.4993	9.0236	10.2789	8.7681	5.2619	7.5127	4.9493	9.6	4.6817	7.133	10.0055	10.1652	0.8052	9.4813	11.4577	8.2676	9.5332	9.6397	5.4949	6.5837	2.2821	9.5972	6.8592
TCGA-44-7660-01	592	0	1.1774	8.19	6.2292	10.5405	7.2676	11.7789	5.709	7.6127	5.9141	10.1378	2.6932	8.351	4.2858	8.4358	6.9071	5.326	14.9914	15.084	9.5048	3.4717	12.8185	8.4959	8.9019	10.3042	5.732	8.1691	7.1357	8.8035	4.0126	7.9879	9.554	9.2416	1.4235	10.4132	10.8247	9.4564	8.7608	9.5014	7.7486	7.114	12.0026	10.5368	5.4422

TCGA-44-7661-01	557	1	5.7984	7.9232	6.0971	10.7137	7.2134	10.6712	7.1889	5.854	8.0779	10.3742	3.9992	2.7936	1.6718	6.211	8.2535	3.3301	7.3495	5.599	0	7.3188	11.8892	7.5599	8.1621	7.6834	4.2086	9.0007	4.9963	10.7849	7.393	7.3959	10.0379	10.0856	0.3921	8.0578	9.8135	7.4275	9.0525	8.8768	7.2845	5.2891	6.7731	8.1093	7.0665
TCGA-44-7662-01	218	0	0	7.2389	5.8901	10.9878	5.6476	10.3694	6.0976	6.6332	6.1824	9.2326	2.3628	2.7276	0.9168	7.5573	7.8046	3.7153	4.2962	2.9091	0	4.4986	10.3806	7.5018	7.0553	7.466	3.8693	8.5659	4.4408	10.065	7.0163	8.3302	9.951	9.7673	0	9.1693	8.7792	8.3746	9.2827	9.9678	7.1485	2.5925	7.0504	10.8111	8.8841
TCGA-44-7667-01	1097	0	3.0574	7.7034	5.4357	9.8506	5.9756	9.262	8.7431	6.8396	3.9984	11.0327	6.1138	0	0.4146	7.5957	5.6325	7.6228	9.1906	9.7353	0.4146	3.6614	10.1497	7.9132	8.9112	10.2016	0	7.6422	2.873	8.5074	5.0858	7.9603	9.5439	8.6313	0	10.6616	14.3671	8.5669	7.966	10.0558	5.3559	4.2714	3.1067	11.5833	5.9832
TCGA-44-7669-01	574	1	6.9752	7.5434	6.4306	10.4937	5.8967	11.3583	9.2405	7.4604	10.2011	10.0198	7.5267	4.3878	2.8954	4.7086	7.9355	5.3311	13.3813	12.3071	0	9.3069	10.9473	6.7716	9.5238	9.6542	4.3665	9.268	4.5094	9.9572	5.962	8.5442	9.4358	8.6928	0.6901	11.2505	8.714	8.4102	9.2221	9.4811	6.0713	6.9822	6.7769	11.4167	7.5025
TCGA-44-7670-01	882	0	4.5961	9.8374	5.7595	9.6695	7.1177	10.649	6.7125	6.5315	6.4522	9.4764	8.9549	5.7346	6.6158	7.7518	6.6517	8.2652	12.9845	11.5598	2.572	7.8304	11.5461	8.8621	9.1091	9.9806	4.0406	7.7099	5.9228	8.7181	5.0644	7.7667	10.0952	8.7609	0.3886	9.5721	10.0198	8.8389	8.8477	9.1489	5.0375	7.7435	3.1268	9.6374	6.6824
TCGA-44-7671-01	889	0	6.765	9.0678	6.4062	9.9997	7.9525	10.9506	7.551	5.2428	6.4432	9.233	5.6208	11.6985	4.2687	8.5349	6.6429	7.1985	16.5237	15.1349	0.3952	5.5834	14.0695	9.7882	9.9651	8.2926	4.7796	7.7308	4.0111	10.7149	6.5146	7.6763	10.3769	10.0085	0	8.5152	11.3111	9.6837	9.7122	10.5558	5.7947	8.609	6.3371	9.9477	9.4207
TCGA-44-7672-01	719	0	3.5289	8.9363	5.5688	11.3473	5.7046	11.0817	6.908	5.5924	5.4579	9.7608	5.9246	5.4706	4.5636	6.4019	7.6107	6.1945	8.7302	7.271	3.6684	5.1119	12.4944	8.1719	8.7836	9.6922	2.7776	8.7899	3.2616	10.6565	6.6181	7.4532	10.6268	10.0391	0.4756	8.9154	10.0208	8.0833	9.2061	9.8291	7.5428	3.9124	8.5544	8.4157	9.0059
TCGA-44-8117-01	385	0	3.0403	9.0584	5.4522	10.3366	7.6027	10.7326	5.5622	6.8036	6.45	8.4966	4.2638	0.6582	1.1085	8.5774	6.3256	5.8128	6.3502	4.6434	0	4.7557	10.3122	8.9129	10.1739	9.8629	1.5962	8.0062	4.0539	10.2931	3.9498	6.5447	7.6942	10.5611	0.9009	8.0335	7.0279	7.9534	8.9033	10.2135	5.2588	1.1085	4.3761	10.3217	6.0325
TCGA-44-8119-01	285	0	2.9707	7.426	6.5523	10.4328	6.65	11.2401	8.9831	6.7661	5.7584	9.7031	9.015	5.0935	0.8392	9.6012	7.8836	6.4819	4.5335	6.1384	0	8.248	12.4319	8.3031	8.2292	10.2106	4.3364	9.1203	3.7401	9.7948	7.8511	7.372	10.4187	9.2089	1.039	9.7532	7.7786	6.4988	8.2666	9.4559	7.1969	4.4622	8.9645	8.4665	7.7786
TCGA-44-8120-01	260	0	5.0669	9.492	5.8557	10.1502	7.147	12.4245	6.8112	5.3106	5.7165	10.597	5.571	2.8415	2.596	7.3416	6.932	6.56	7.5737	6.4111	0	5.3508	11.3798	9.9196	10.3553	8.84	2.1257	8.4386	4.2647	11.1661	6.8219	7.7132	10.1222	9.9692	0.8805	9.043	10.1992	6.0875	10.2006	9.6719	6.0391	2.0299	0	8.0303	5.2901
TCGA-44-A479-01	486	0	0.6485	8.4206	6.0747	11.0986	8.1565	11.5122	7.0517	6.3899	6.1458	10.2351	9.6512	9.0512	4.4216	5.5537	8.7989	4.4961	8.0022	8.8741	5.3679	9.6088	12.1388	7.7326	7.9894	8.7311	3.9778	9.563	4.4594	9.712	9.4292	8.1422	10.5219	8.0275	0.6485	9.3846	8.2374	7.3959	9.1308	8.9588	8.232	3.7534	8.7994	6.6238	9.5909
TCGA-44-A47A-01	466	0	5.8143	10.1401	4.7872	10.5833	6.2715	11.2921	5.6178	5.6911	4.3173	9.1541	3.786	8.2331	4.2797	6.7475	7.1314	2.7289	6.517	5.7744	0	5.0098	12.8398	8.6315	9.0929	11.2144	5.8532	9.0697	3.786	8.1627	5.9988	8.9648	9.9093	10.1776	1.0169	10.3419	12.5212	8.4107	9.5284	9.6629	5.8659	0.5963	10.4123	10.9837	5.0098
TCGA-44-A47B-01	287	0	1.2287	8.9987	5.1122	10.4886	4.0595	11.2529	6.4126	6.9508	6.4574	8.848	7.5283	2.3307	0.9227	7.9423	6.9297	3.7591	8.4961	7.3616	0	5.2201	12.6234	8.1522	9.01	9.8815	3.3791	9.0568	5.4738	8.9616	4.8236	7.0227	9.058	9.0532	0	8.3532	11.2422	8.6277	7.9657	9.0665	5.8436	4.3081	11.4767	7.3014	5.7265
TCGA-44-A47G-01	351	0	6.7397	9.0414	5.3672	10.1529	5.4069	12.3488	6.3997	6.222	4.8095	9.4305	4.0968	4.5509	2.4862	7.9159	8.6008	6.3799	12.4354	12.6567	0	5.6741	12.6875	8.3286	8.8057	9.7061	5.8854	9.6138	4.4782	10.4372	6.3598	7.5315	10.4901	10	0.6557	8.5943	10.3407	8.5485	9.6992	9.6942	6.5311	4.0968	8.9091	7.7676	8.2999
TCGA-44-A4SS-01	415	0	2.8263	9.126	5.8728	10.3292	5.3726	10.9673	6.7159	5.5192	5.2309	9.75	9.0986	1.4122	1.685	7.2233	7.0593	3.4554	14.0957	13.9573	8.0265	3.892	12.5138	8.7637	9.5726	6.5209	2.1121	9.4012	6.5209	10.4426	6.8466	6.6061	8.9706	10.427	1.4122	8.8683	8.6629	8.8511	9.274	10.1283	7.1408	1.4122	5.2714	7.3802	7.2018
TCGA-44-A4SU-01	409	1	5.922	9.1983	5.0206	10.3744	7.2979	11.8447	5.1597	6.345	7.5716	8.9817	5.6792	0.5553	1.2682	8.6341	6.4495	6.3698	8.0543	5.9331	0	5.9108	11.3198	8.9996	9.6194	9.6891	3.0896	9.573	4.1995	10.0974	5.5406	8.555	11.0489	10.016	0.5553	9.9255	10.8364	9.1879	9.6701	9.779	8.1069	5.8057	7.95	8.9956	7.7708
TCGA-49-4486-01	2318	1	8.4379	10.8659	3.5816	9.8876	7.1596	11.7832	6.5612	6.0909	4.8209	9.2848	2.5819	10.117	0.7355	10.6626	5.966	7.8667	16.5744	15.9504	0.7355	3.6971	14.3822	9.6414	10.388	7.6378	4.9193	7.8585	2.9968	9.3568	8.0243	8.7552	10.6578	11.2095	0	7.5938	13.2308	10.0948	10.7034	10.6979	4.9034	8.4393	1.5825	9.0498	8.6049
TCGA-49-4487-01	855	1	7.0942	9.0199	4.088	10.4128	6.4831	10.3994	7.3979	6.8315	7.4335	9.5138	3.4737	3.2364	1.4249	7.7253	6.7771	4.9301	7.2855	5.045	0	10.1802	12.3076	8.1372	8.681	9.1877	4.5696	8.5735	5.7569	10.3132	6.8208	8.5905	9.4378	9.1751	1.4249	9.1518	11.8999	9.4482	9.0454	9.5458	6.5365	5.8441	5.6133	9.8588	8.3383
TCGA-49-4488-01	869	1	2.6175	9.7011	5.4928	9.8893	7.3041	11.7947	3.7522	5.0248	2.6175	9.8618	4.8522	3.0597	3.7522	8.0468	4.3794	5.5616	12.6875	12.0389	0	2.9268	11.6464	8.0667	8.9487	7.1635	2.2231	8.1056	2.2231	8.5517	3.6714	9.7512	8.362	10.583	0	9.8882	11.7991	9.0054	8.5203	10.7466	7.0562	3.5858	1.3031	10.3522	8.1285
TCGA-49-4490-01	385	1	4.4325	9.31	4.0801	10.3184	7.4902	11.1803	5.789	5.789	6.3321	9.0693	5.2291	8.6764	3.4978	8.9976	6.5921	5.5214	6.3488	3.6127	0	8.4048	12.3048	8.5379	8.9049	8.9077	3.4978	8.0136	4.6632	10.8502	7.254	8.0413	10.3886	9.9174	0.9532	9.8619	12.4241	9.238	8.7283	9.0495	7.389	6.0558	7.7738	8.3807	6.8827
TCGA-49-4494-01	1081	1	4.6998	10.0844	4.2395	10.7111	7.9176	10.2354	5.2872	4.261	2.9662	9.5207	2.3948	5.1896	2.0438	8.2572	7.3699	3.2931	10.8637	11.4741	0	3.8137	12.2754	8.7253	9.1692	7.0922	4.0044	7.8375	1.71	8.6579	4.8775	7.5013	9.6092	11.344	0	11.6359	10.2317	8.8081	9.789	9.4654	6.331	4.4225	11.6834	10.0392	7.4703
TCGA-49-4501-01	1421	1	4.2453	9.7904	6.4495	10.6188	7.129	10.9771	7.928	6.1229	6.7863	9.6977	7.6068	3.9323	2.1488	6.896	7.2516	5.4787	8.7572	5.8792	0.3382	7.1262	12.9849	10.5559	10.0192	10.5561	2.3112	8.6312	3.6582	11.1478	8.4578	7.7571	9.508	10.9224	0.3382	9.7392	12.8028	8.607	9.1005	9.312	7.1479	6.6601	8.4583	6.7724	5.9301
TCGA-49-4505-01	428	1	3.9101	9.6414	5.2709	10.5448	7.0079	11.5965	5.5294	4.8044	6.8624	9.2435	5.0194	6.1701	1.5678	7.5902	6.1474	5.8433	6.6122	4.4442	0	6.1644	12.1754	9.1091	9.4056	10.5421	2.2168	8.4895	3.5441	10.2335	5.4939	8.8126	10.1734	10.7667	0.3569	9.0981	11.8473	8.6361	9.1193	9.658	6.8933	2.8429	10.0182	9.2875	7.4833
TCGA-49-4506-01	999	1	2.7162	10.1056	4.7478	9.6997	5.7316	9.9828	3.8611	6.1344	8.84	9.7459	1.374	1.1335	1.1335	9.683	6.2445	2.0648	14.8311	14.1799	1.374	3.3439	10.94	7.4307	9.8265	8.5874	5.4457	7.3799	6.8432	8.3876	5.0888	8.7724	9.296	9.6725	0.4834	10.2644	14.7679	6.0328	7.2726	10.3202	6.1425	7.6834	6.4051	8.3773	7.6351
TCGA-49-4507-01	268	1	3.7498	8.8671	1.4486	10.4116	5.7803	9.6881	5.7984	6.2751	4.2204	11.4497	1.2531	4.8929	0	10.4072	6.9639	2.2645	14.9581	15.599	2.9851	2.6296	12.5502	7.545	9.1122	8.0921	6.9798	8.2692	5.7894	8.1139	9.1346	7.3832	10.4138	9.7258	2.4586	9.306	12.4631	8.8585	9.071	9.167	5.4214	2.6296	8.2946	10.5358	8.984
TCGA-49-4510-01	896	1	6.5967	8.9028	5.2372	9.7426	7.0187	10.7902	6.5434	5.7116	7.0745	9.2861	4.1377	4.1891	3.6058	9.4177	6.2546	6.4035	12.5973	11.9727	0	4.1377	11.4513	9.4157	9.92	7.9116	3.0044	7.3277	3.849	10.3112	6.4777	8.8391	10.7792	11.1528	0.3998	8.8268	11.7319	9.3028	10.3392	9.3649	5.8458	5.504	4.7772	9.3537	8.0794
TCGA-49-4512-01	905	1	2.4885	10.2271	5.023	10.3579	5.7352	10.7836	5.7559	4.6779	5.6929	9.2022	4.6779	5.023	3.2411	7.3298	7.4864	5.6929	5.855	3.9639	0	5.6494	14.0524	9.4169	9.8568	10.622	3.1188	8.4574	2.9851	10.1393	5.9296	8.404	10.3913	9.728	0	10.0553	13.2586	8.7297	8.2325	8.3962	7.0067	3.2411	4.132	6.823	5.6494
TCGA-49-4514-01	1700	0	3.6412	10.7021	2.2707	9.8608	7.2812	10.5462	6.4121	5.2664	4.4368	8.8459	2.8321	13.7224	10.435	8.5004	6.3727	6.0113	16.8114	15.777	9.7447	2.483	15.1531	8.9205	9.8925	7.7291	3.2351	8.5424	2.0216	7.5697	5.7898	10.3614	10.916	10.3377	1.3393	11.0014	12.6986	9.6802	9.7562	8.9273	5.5517	7.3707	2.0216	10.4294	8.0538
TCGA-49-6742-01	488	1	6.4513	10.1393	4.1215	10.4527	7.0793	10.1287	6.8183	5.6923	5.8867	8.445	4.8375	10.8366	10.2316	8.9804	7.3205	7.5954	13.4683	15.4703	7.687	4.9109	12.5269	9.1029	9.882	6.9633	7.6607	7.8357	4.4515	9.6566	6.7992	7.9999	10.5499	11.2003	4.592	8.0659	12.1419	8.8721	9.3504	10.1074	5.2866	7.9445	5.1153	7.9489	6.4513
TCGA-49-6743-01	1621	0	3.6934	7.466	6.2248	10.9583	7.6948	10.9728	6.2963	6.526	6.6165	10.1782	3.8644	3.8096	0	9.1905	6.838	5.8707	14.8128	13.1569	5.045	3.4994	11.5408	9.6226	9.0821	9.7517	5.455	8.5913	3.4994	10.6488	8.4795	7.1762	10.3071	9.4374	1.0602	9.4227	11.3536	6.8311	7.8723	10.3052	6.9749	2.6839	1.8756	11.1517	7.5053
TCGA-49-6744-01	1683	0	5.5492	8.3503	6.037	10.5029	6.7264	11.4451	6.9626	4.154	7.1028	9.6219	5.9565	4.1947	0.5938	6.6984	7.2549	5.2734	8.8206	8.0895	1.0131	8.0868	12.5531	9.4001	9.9119	9.3492	2.1904	9.1644	3.8317	11.4085	7.9239	7.9787	9.7188	9.9672	1.6025	8.4566	12.289	9.0247	8.8559	9.4577	7.2208	3.416	8.722	9.149	8.7993
TCGA-49-6745-01	522	0	5.7956	7.1451	7.5824	10.3592	8.2879	11.1707	6.9467	4.1057	7.13	10.2751	3.8347	7.0141	2.5641	6.6118	6.9177	5.0632	6.3931	4.5306	0	5.3679	12.0252	7.5026	9.6252	6.4265	0.9875	8.4118	4.1463	10.0214	7.3588	7.5544	9.6421	10.3849	0.5767	9.2696	12.3447	7.6404	8.6858	9.0704	6.9294	4.1057	6.6971	8.2558	6.5826
TCGA-49-6761-01	354	0	3.3968	8.5841	6.034	10.3068	6.9306	10.8106	6.3254	4.8512	4.3784	9.475	2.2223	1.9757	1.3025	7.2963	5.968	1.9757	5.538	1.678	0	4.4284	11.2771	7.071	7.976	9.0903	0.7935	7.7391	4.5687	8.6826	5.2067	7.6961	8.2917	9.623	1.3025	10.0546	11.3973	8.9261	8.841	9.2995	5.8072	2.2223	3.3509	10.2592	6.6437
TCGA-49-6767-01	677	0	0	8.327	5.7299	9.9018	5.9937	10.2658	2.6612	5.4464	6.284	9.4285	3.4672	1.1266	0	6.675	7.0025	3.6138	2.9086	1.985	0	4.0343	11.3815	7.3758	10.1541	8.9254	2.186	8.1155	4.9348	7.4263	3.747	9.2419	8.1093	11.009	1.1266	11.7566	11.5355	9.6814	8.9095	9.6616	5.7299	0.6706	2.6612	8.9481	5.2131
TCGA-49-AAQV-01	677	1	5.1474	8.4527	4.9776	10.8273	8.034	12.2741	5.0073	7.2058	6.0927	9.3625	7.4446	6.1471	1.8577	7.5032	6.8474	6.3288	7.4662	5.8435	0.7278	8.7462	11.5466	9.0466	9.1164	10.591	2.3963	9.1027	4.8021	8.838	9.6267	7.4267	10.1997	10.3494	1.5696	9.8967	11.3372	10.0258	9.0258	10.0863	6.6161	5.5319	9.8086	9.594	5.8435
TCGA-49-AAR0-01	4765	0	7.3542	9.3876	4.3103	10.7923	7.0806	11.3301	7.0806	7.3322	7.6353	9.6429	6.4474	9.9405	3.237	8.4328	8.2546	6.4391	15.9164	15.9654	2.4495	6.5115	12.3616	9.3563	9.1813	9.164	3.6892	8.3694	4.3807	10.7085	6.0324	7.6098	10.9159	11.0914	1.5768	10.5896	11.0549	9.1171	9.7976	9.2405	6.61	8.7477	5.0306	10.006	7.3143
TCGA-49-AAR2-01	2224	0	3.9863	9.929	3.9288	11.5708	8.3062	11.1269	9.5536	7.5863	4.8188	8.6424	2.8454	11.1408	6.0024	7.8096	7.8757	6.2916	14.4448	14.4795	11.0712	2.8454	13.0731	8.9139	9.7205	10.2605	4.8501	8.4987	3.5258	8.4433	2.9645	9.4731	11.4329	10.9287	1.1618	8.1037	10.582	10.0842	10.3991	8.7243	5.0519	6.8279	9.5846	4.7868	7.989
TCGA-49-AAR3-01	1893	0	1.4553	8.5762	2.9943	10.9889	5.5861	10.2325	5.1636	6.3268	5.4775	10.1875	2.9943	9.1477	0.9039	6.1957	8.9124	2.8273	12.9894	11.6542	0	4.1334	12.0405	8.1981	8.3406	8.6374	1.4553	8.8808	5.6871	9.7827	5.36	7.4404	10.2909	9.7769	1.4553	10.2115	8.5191	8.1099	9.0083	9.5627	6.5577	2.6384	3.3392	10.7347	8.5925
TCGA-49-AAR4-01	879	1	1.5319	8.8256	5.3699	10.5541	5.5162	10.8096	4.5667	7.0252	4.3158	10.1158	6.8628	7.08	3.2968	8.4394	8.8216	7.1	8.1662	5.9579	0	5.1313	11.2398	7.8838	8.5259	9.5997	4.1718	9.4255	5.8198	8.5259	6.2485	7.9811	11.1661	10.3336	1.8164	9.6972	11.2905	9.0343	9.6425	9.535	6.7147	3.3865	8.4466	9.6818	8.4103
TCGA-49-AAR9-01	260	1	0	8.8724	5.9336	10.2174	5.0093	8.7729	3.0605	6.6977	4.4667	9.4799	0.9395	2.4827	0	7.51	6.3041	3.5867	10.9522	13.0642	1.249	2.5965	10.2017	9.5656	9.3101	9.7491	1.249	9.2592	3.2111	7.5063	5.3711	6.2842	9.5847	9.8028	1.7201	6.9434	9.17	9.4558	8.6215	9.9499	9.0799	6.0972	4.5781	4.7137	6.4013
TCGA-49-AARE-01	1229	1	5.3595	8.7103	4.514	10.1882	6.2149	12.1477	5.2078	6.5937	5.2772	8.6865	2.7902	7.9338	2.9686	6.5241	7.0797	6.2924	16.9894	17.7095	4.2635	3.8733	13.4083	8.3386	8.1387	8.9469	3.4612	8.3223	4.3942	9.0446	4.9561	7.0087	10.15	10.0451	2.2145	11.1952	12.1808	6.4358	9.3823	9.9507	5.7975	6.4953	3.8902	11.7426	9.8688
TCGA-49-AARN-01	1135	1	4.1225	9.0276	5.4442	10.3187	7.2666	11.4347	5.0154	6.6475	7.7036	9.5349	7.4254	8.5843	5.0154	6.8755	7.0723	6.255	7.7191	6.8846	1.6955	5.3939	12.0521	8.4306	9.2892	9.5825	5.9759	8.3271	4.721	9.3432	5.3149	7.3175	9.9158	10.3619	1.6955	9.7088	11.5306	8.4523	9.8693	10.0096	5.8131	5.9587	10.5158	8.4399	5.9929
TCGA-49-AARO-01	3759	0	3.5081	8.8739	4.7824	10.5507	6.9248	11.2583	5.9022	6.6545	7.0176	10.5982	5.7713	6.7348	5.2219	7.8663	8.3495	7.8118	8.4094	7.9595	0	6.1911	11.5217	8.2655	8.9414	9.7317	1.7251	9.2833	4.5886	10.2538	4.0995	8.135	11.7477	10.1353	3.0866	9.4483	9.3788	8.5358	9.5986	9.5135	6.3525	4.4433	6.1985	9.5878	6.3827
TCGA-49-AARQ-01	6732	0	8.2656	8.0254	5.4119	10.9229	6.1543	12.9082	6.0724	8.8206	7.2807	9.316	3.5453	8.7971	0	8.4344	8.0378	4.8056	8.0592	9.6915	0	11.3203	12.672	9.4027	8.4645	6.0844	4.6203	8.3122	4.0644	6.7738	3.9112	9.7423	10.5468	10.4574	0.6432	10.6584	11.365	10.1827	9.5265	8.8384	4.2029	2.8439	12.7773	11.6231	7.1035
TCGA-49-AARR-01	4992	0	7.3099	8.9703	7.057	10.3072	6.5913	11.2421	8.0036	7.4804	5.8102	10.3565	8.2629	8.8637	8.1815	8.3557	8.9806	10.6573	11.6853	8.4086	1.8283	8.5781	12.6185	9.2867	9.1417	7.9892	3.8853	9.8383	5.314	12.3625	8.9674	9.2377	10.6504	10.3973	1.0147	8.3602	11.2247	8.6123	9.692	8.918	6.8683	6.948	4.5419	8.0179	8.6964
TCGA-4B-A93V-01	300	1	3.1983	8.3087	6.7904	10.5157	7.2022	11.8443	4.829	6.567	8.8973	10.5831	0.9725	6.4919	0.9725	9.0066	7.8575	6.2025	16.2936	14.6378	1.5481	2.1269	12.3132	8.0294	6.5888	8.9459	3.5341	8.1418	3.8551	7.6382	5.0122	8.8423	11.66	9.3936	0.5667	10.13	8.2755	9.4513	9.1649	10.3946	5.1938	2.5388	10.2676	11.2907	6.6931
TCGA-50-5044-01	624	1	7.0667	7.9463	4.0315	10.5153	4.9867	10.2708	5.8143	6.7475	7.3909	9.6259	2.3489	4.8397	3.0294	6.3907	8.9583	4.5557	8.86	6.6771	0	5.2421	12.0521	8.2861	7.6916	9.4301	4.6172	7.8213	4.8904	9.3622	7.2428	7.7529	11.0286	9.734	0	9.0193	9.7546	7.3997	8.5804	9.0752	5.3547	2.8362	13.6673	6.203	7.0106
TCGA-50-5045-01	2174	1	5.2242	9.3711	5.3036	10.4104	6.4824	10.4472	7.4147	5.9911	5.7629	9.6787	4.9159	5.5318	3.1067	7.1907	7.3148	6.0475	7.9957	6.1107	0.5089	6.9255	12.4169	8.3612	8.5391	10.7656	4.4415	8.9166	3.6347	10.5159	7.2074	8.268	10.1204	10.1088	1.1821	9.4621	11.5035	8.4873	9.8518	10.4243	7.7513	4.4131	5.9761	8.6856	8.1476
TCGA-50-5049-01	3094	0	2.1204	8.9638	5.856	10.9536	5.4213	10.6517	6.9963	5.8137	5.3832	9.9978	6.3061	6.0985	2.5903	6.7493	6.7935	5.3242	7.2288	4.611	0	8.9541	12.0418	8.3203	8.4271	9.64	5.1537	9.5158	3.7902	10.61	7.9747	7.4268	10.2583	9.8509	1.4191	9.3652	10.8462	7.2288	9.231	9.5824	8.0064	5.4024	7.6625	8.1578	9.5124
TCGA-50-5051-01	478	1	6.7842	9.4296	5.4967	10.2837	6.7326	11.0506	9.5448	5.9587	4.4538	9.5179	4.9639	7.5281	1.35	9.4133	6.947	5.4717	15.1733	14.659	2.0351	5.6381	13.3824	8.0839	8.3364	7.7205	5.0675	8.6153	5.1005	9.5963	5.4967	9.0599	8.3604	9.2412	0.8275	10.2167	8.8849	9.1119	9.1654	9.6487	6.2505	8.9153	9.4686	10.8617	6.112
TCGA-50-5055-01	1830	1	4.8698	8.9151	4.6958	10.2262	5.4383	10.6988	7.2096	5.3529	6.4898	9.8378	6.6415	7.8207	4.8283	6.4075	7.341	7.0102	9.2698	7.1433	1.8039	7.1262	12.2298	7.8728	8.7606	8.7961	3.3414	9.4841	2.7685	11.0682	7.7994	7.8328	10.2412	12.0757	1.412	8.6985	11.4909	8.2604	9.4941	9.2948	7.9715	5.2305	4.2516	6.4215	8.5402
TCGA-50-5066-01	1442	0	3.9826	8.9382	6.0132	10.0678	7.1331	10.9564	7.203	7.6284	4.8517	9.1668	4.9362	4.1334	1.8533	7.9815	6.9214	5.7815	14.1848	12.1841	0	5.1281	10.9514	7.1509	7.1859	10.1263	6.3577	8.7497	3.4035	8.7261	9.3384	8.6275	9.2042	10.8031	2.1649	11.2635	11.2345	8.3445	10.0702	10.9232	7.5501	5.8702	9.6688	9.6792	8.8861
TCGA-50-5068-01	1499	1	3.3793	9.238	5.5066	9.8023	6.2032	10.6676	5.9869	7.688	3.3793	9.1128	3.8759	5.7323	3.1203	6.6831	5.8222	5.9069	8.7201	7.7751	0	6.2687	11.855	7.9348	8.6012	9.985	3.8759	8.8647	1.834	8.5495	6.9755	8.7067	9.8133	10.867	0	9.7036	12.4893	7.9145	10.7299	11.4239	7.9348	8.6725	4.8259	8.598	7.4895
TCGA-50-5072-01	250	1	7.12	9.7949	6.5733	9.9171	8.3718	10.8529	7.362	6.1064	6.5872	9.6113	6.8922	3.5952	5.4731	9.4499	7.0614	7.27	13.274	14.9999	7.6411	6.995	12.1587	8.4747	9.4365	7.4599	9.8949	8.023	4.3174	10.511	7.0311	8.2072	9.7874	9.9539	4.9125	10.2267	11.395	9.6432	8.0833	9.7971	6.6615	8.5477	6.7571	10.0557	7.0002
TCGA-50-5930-01	282	1	3.3641	8.4955	4.4757	10.6676	8.3852	11.2197	6.1305	5.5284	6.0167	9.4819	3.9643	2.9336	2.9336	9.5276	6.9132	4.3869	11.0043	11.1539	1.8703	6.1576	13.302	9.1432	9.7148	9.6825	7.5868	8.7301	6.4701	11.1379	7.2517	7.9707	10.5383	9.7419	1.8703	9.6543	11.4481	10.0162	8.884	9.8372	7.0496	6.1576	3.3416	9.5172	8.1956
TCGA-50-5931-01	434	1	2.7402	8.2055	5.0802	9.65	7.5143	9.3373	6.9554	7.4375	7.1475	10.0255	6.3653	5.2333	2.2594	5.415	8.0285	4.0136	9.7602	9.9745	0.706	5.9151	11.2454	9.6646	9.426	11.0126	4.5296	7.027	2.5971	9.591	4.4896	6.9646	10.6508	8.9112	1.533	9.9341	9.4716	10.1933	8.9975	9.8279	6.8409	5.3041	11.3302	11.3766	5.053
TCGA-50-5932-01	1235	1	3.7954	8.1457	6.2124	10.0623	7.1058	11.1795	5.0626	5.621	6.8801	8.3622	3.9586	2.9355	0.8729	8.0763	5.3094	4.4991	11.2368	9.4445	0	4.6259	12.354	8.4721	10.7895	6.3657	2.1127	8.3976	2.7695	10.5601	4.989	9.3256	9.9975	9.2146	0	8.1179	10.8482	8.6348	9.561	10.7482	5.7602	5.3393	6.4809	5.4803	7.0927
TCGA-50-5933-01	2393	1	0	7.9026	5.238	10.1663	5.3505	10.2166	7.0178	5.0284	6.1269	9.658	3.6119	1.6046	3.1134	6.7839	7.4679	0.5948	5.3324	3.3483	0	4.911	11.0713	7.2483	9.2042	9.8966	1.6046	9.0151	4.1151	11.0658	6.7085	7.8118	9.2593	9.5771	0	9.863	10.4592	6.6511	8.4639	9.3813	6.8492	2.7253	5.5733	9.6857	5.3324
TCGA-50-5935-01	653	1	3.111	9.6031	5.881	10.1491	6.6392	12.3309	6.5636	5.3388	7.7086	8.5103	6.1886	5.2451	2.0646	7.1724	6.0706	5.7335	9.7133	7.6865	0	8.8355	12.726	6.7796	9.2638	9.6518	1.1848	9.0042	2.448	10.5369	8.4896	8.0243	9.6172	9.7447	0	8.7074	11.0458	8.1481	9.3309	10.0888	6.0006	4.7603	5.2927	7.4443	5.5883
TCGA-50-5936-01	257	1	5.083	9.3478	5.8765	10.3779	6.4948	10.3811	6.1971	5.2024	7.5998	8.9007	3.4898	3.8235	4.8831	9.3703	7.4082	4.8831	9.6517	11.3425	2.2188	8.2634	10.5461	8.8015	10.2409	8.1529	9.6266	9.4866	5.1734	10.2251	6.398	8.6943	9.8646	9.351	3.0548	7.8442	11.0475	8.7188	8.6678	9.707	6.5963	5.5336	8.1379	7.6106	7.0507
TCGA-50-5939-01	460	1	5.1094	8.2121	5.2457	10.5427	6.8051	10.4463	6.5126	5.4401	8.192	11.531	3.4934	3.7349	1.6099	10.4213	8.6184	5.9193	13.1515	12.8628	1.9016	5.8863	11.9254	8.7318	9.9234	10.7632	3.4934	9.077	3.8072	10.8368	10.0073	7.3605	9.3063	9.8622	0	9.04	12.3651	6.742	8.1228	9.4202	6.3406	4.9268	11.155	6.995	8.6457
TCGA-50-5941-01	1474	0	4.9301	8.5814	5.6904	11.1252	7.6372	11.3914	6.7121	3.7887	5.7941	9.6193	3.9167	5.8908	2.2769	7.0135	6.3421	5.617	7.2012	5.371	0.7146	5.0184	11.8689	9.2856	10.4182	10.5978	2.8895	9.2111	2.759	10.4175	6.3648	7.9774	9.8408	10.2231	0	8.4234	11.532	8.5197	8.4943	9.7968	6.7384	4.6275	7.1492	6.9551	8.5096
TCGA-50-5942-01	1847	0	7.0931	10.0659	6.5695	9.9231	8.098	10.1158	7.6655	5.8232	8.0005	9.1292	5.575	11.2344	3.9131	6.9661	6.8429	7.7364	10.1141	6.4051	0	6.0766	13.2092	8.493	10.5674	9.3384	4.7319	8.3449	3.4046	11.7043	7.3801	7.0728	9.7128	9.6973	0	8.0604	11.637	8.1798	10.2468	9.3722	5.9178	7.9341	4.1393	8.916	7.1527
TCGA-50-5944-01	1750	0	5.9958	8.7615	5.6726	10.1833	7.1044	10.2499	8.2628	4.8606	7.6068	9.4373	5.8509	5.8044	1.5005	6.5119	7.7648	6.3049	9.9823	9.1173	0	6.8986	13.2627	8.895	11.0245	7.9926	3.6539	8.1151	4.176	12.402	6.8536	8.1845	10.4065	9.4598	0.6869	9.4384	10.2926	8.9636	9.2832	9.5399	7.2788	6.4628	3.1285	8.2967	6.0632
TCGA-50-5946-01	1617	0	0	8.6258	6.4031	9.9911	8.5286	10.9389	7.5586	8.1001	4.6874	10.4428	6.6072	6.1971	1.1932	7.3034	4.723	6.3126	3.8265	4.8083	0	2.8939	10.0276	9.4551	9.3409	9.5948	3.07	8.5535	4.4103	7.6857	8.6731	8.6235	9.1878	10.0196	0	9.2247	10.3362	8.9912	9.0361	10.2087	6.0717	7.0504	7.068	9.9889	5.1978
TCGA-50-6590-01	1288	1	0.6262	7.9077	5.6413	10.6686	5.2463	10.895	3.9703	6.1629	9.3308	10.692	2.0912	0	0	7.7047	7.1583	4.067	6.4498	9.4821	0	5.0472	10.5168	8.322	9.4398	9.2988	6.4764	9.3704	3.0117	9.0582	5.873	8.076	10.1474	9.425	1.3983	9.7023	10.52	7.455	7.3769	9.4193	7.3387	2.0912	9.774	9.0988	8.9306
TCGA-50-6591-01	119	1	3.9941	7.0812	4.6027	10.3702	2.6727	9.8939	9.9288	7.0875	11.303	10.0962	8.3165	3.4003	2.8019	7.4149	8.4132	4.1478	2.5323	1.134	0	4.2419	10.34	8.6483	9.1616	10.1543	1.134	6.8982	2.1967	6.3929	3.3162	9.1415	8.0822	9.1079	0	9.6604	7.1556	7.0812	9.2463	9.2562	5.4401	8.4005	10.8191	9.529	2.5308
TCGA-50-6592-01	777	1	2.5134	8.3004	6.2301	10.7752	7.7883	9.8632	8.6654	6.3486	6.458	9.7783	2.4009	8.8237	7.3699	7.0951	7.2694	6.3024	16.1313	15.5763	6.6486	3.8352	12.6688	8.949	8.2094	9.1108	6.5728	9.1319	6.0273	9.8843	8.2157	7.3793	9.4058	9.8619	1.4397	9.7918	10.6155	7.8777	8.6455	9.6773	8.6624	4.9123	7.2833	10.0284	8.2607
TCGA-50-6593-01	336	1	3.7896	9.1152	5.6179	9.8796	6.6126	11.4956	6.5743	5.7782	6.8152	10.1656	5.3033	6.8951	4.869	7.0001	8.4243	7.6241	8.9733	6.0256	0.7149	6.2488	12.6502	8.74	9.6672	10.6549	2.7598	9.0186	3.855	10.8057	5.8279	7.7689	10.2727	9.8558	0.7149	8.1621	10.3513	8.9473	9.2136	10.2979	7.1895	3.6491	2.8903	8.5456	6.2366
TCGA-50-6594-01	370	1	0	9.3527	5.481	9.9824	7.7952	11.5356	3.6973	5.9927	5.4347	9.1608	0.7691	1.2681	0	6.8017	7.3609	6.0085	5.5035	5.75	0	3.7736	12.597	8.8057	8.1995	9.9271	4.162	7.0971	3.915	8.4811	5.5477	6.5235	11.0398	11.6551	0	9.5808	10.877	6.51	8.8034	10.0327	4.5993	1.2681	9.8455	10.9947	4.3728
TCGA-50-6595-01	189	1	1.8369	7.1415	6.0149	10.9069	5.7128	9.595	6.1517	6.2646	6.203	9.8337	3.0133	2.2805	3.3224	7.8411	7.8411	4.8406	7.0948	5.9262	0	6.1776	10.2942	7.6785	7.3147	9.2872	1.8369	8.9733	2.7629	10.2928	9.3224	8.1127	9.6364	9.1272	0	10.011	10.0872	7.4322	7.701	9.3821	6.9371	2.8935	10.6351	8.5788	7.5106
TCGA-50-6597-01	1268	1	7.0237	9.9955	3.2453	10.5431	7.7351	11.1404	8.782	5.0046	4.442	9.5356	3.7282	9.2253	7.6361	6.0244	6.0451	5.749	13.6585	10.7911	3.5069	3.7282	12.5519	7.7542	10.2354	6.4854	3.2453	9.0101	6.5302	9.9928	4.6727	9.3584	11.0316	8.0998	1.5286	9.8365	12.248	8.9034	8.9537	8.5221	5.0869	2.254	0	7.358	10.8335
TCGA-50-6673-01	22	1	4.7136	9.7645	5.3903	10.0955	7.6607	11.0263	7.0773	5.6541	6.9324	10.3142	9.1668	5.138	7.1011	7.3983	8.0689	7.5571	7.1244	5.9831	0	6.8436	12.4416	9.2882	10.0918	8.1337	2.7243	8.5789	3.6756	10.853	10.1219	8.3915	7.4911	9.4885	0	8.2893	10.863	9.1108	9.3979	9.3491	6.3234	7.8124	4.922	7.2198	6.5213
TCGA-50-7109-01	308	1	4.9777	7.9299	6.2833	10.1294	8.1719	11.3241	7.4504	5.926	7.9691	9.3338	2.5562	4.7676	0	8.2807	6.9783	7.7453	8.3389	8.3362	0.6872	6.2138	11.5956	7.4555	9.1338	8.6746	2.6986	9.045	3.2544	9.8223	5.8819	8.5965	10.2543	8.3059	0.6872	9.5145	9.3757	8.2111	8.4053	9.4543	5.7572	4.5962	8.8269	8.5008	7.8367
TCGA-50-8457-01	1125	0	4.4503	10.0329	6.5646	9.7891	5.9805	10.943	6.8011	5.2587	8.0062	9.3515	7.6555	4.921	2.5564	6.377	6.3693	5.6768	6.8794	5.4169	0	7.2258	13.8061	8.6943	9.7804	9.6972	4.0474	9.1311	4.717	12.0912	8.7459	6.8272	10.1366	9.5715	1.4727	8.6535	12.0337	7.9478	9.5916	9.9184	6.5024	6.1066	4.7993	7.4944	7.665
TCGA-50-8459-01	1119	0	4.3633	8.8761	5.5335	10.6494	5.2663	11.3765	7.6125	6.1598	6.7827	10.7201	6.7973	5.4437	5.2871	7.5749	9.3738	7.6532	9.6039	7.8339	0.6411	6.9874	12.8595	7.735	7.633	8.9416	2.5935	9.2063	3.5398	11.6035	6.1711	7.6948	10.7768	9.8256	0.6411	8.11	10.037	8.1158	9.5809	8.8518	7.9849	4.0096	9.1186	7.7501	7.3852
TCGA-50-8460-01	829	0	8.4933	9.7098	4.4637	10.2756	5.9184	12.1573	5.7624	5.1067	8.1692	9.77	5.6423	5.8269	2.4161	7.7672	7.1207	6.5441	7.7129	6.6984	0	5.1324	14.4753	9.8977	10.6384	10.2777	2.238	8.6054	3.8709	10.9687	6.3927	7.6153	11.5399	9.9699	0.6956	8.6836	12.7118	9.2469	10.8459	9.8217	6.6547	6.6368	10.3318	7.8929	7.0141
TCGA-53-7624-01	1043	1	4.6015	8.2667	9.8588	10.3709	3.3537	10.4788	7.5753	6.5495	5.3092	10.7911	2.1742	0	0.9094	6.4074	6.5083	5.6866	12.7221	8.1444	0.5253	7.5687	9.0178	9.2151	9.4046	8.6959	2.5437	8.2334	5.047	6.9228	7.6114	7.5545	9.7371	8.2015	2.7462	8.9919	11.423	7.8136	8.6682	9.3209	6.6412	7.7474	5.9352	11.2613	6.4659
TCGA-53-7626-01	929	1	6.0664	9.0605	5.5133	10.3255	7.1278	12.1934	7.1041	5.4769	5.6173	10.1712	6.0725	4.6048	2.4732	6.7394	6.6846	5.6421	6.3791	5.8343	0	6.7162	12.2666	8.7009	9.609	8.2709	3.4161	9.2877	3.6629	12.011	7.2835	7.8936	10.1147	9.824	0.8903	8.3722	12.0427	7.0831	9.1222	9.7803	6.5465	8.3024	1.0235	8.3346	7.6035
TCGA-53-7813-01	424	0	4.9924	6.3609	4.8961	10.3636	6.1682	10.9553	6.7115	6.683	7.6831	7.7394	1.0198	5.2083	1.6114	8.4906	6.9934	5.6236	9.6055	7.1372	0	9.2706	12.0063	7.268	8.5863	8.6356	1.0198	8.2873	2.6179	8.1054	5.3604	9.5614	9.2093	7.6686	1.6114	10.1256	10.1001	7.9341	9.9922	10.2647	6.6973	6.9817	10.217	6.4985	8.6651

TCGA-53-A4EZ-01	1071	0	0.8375	10.6427	3.329	9.9457	7.3285	11.3134	9.8585	8.0941	3.2113	9.1746	7.7484	6.0252	3.0147	9.4003	5.1862	6.1663	16.6959	15.5957	6.6057	8.7919	12.4799	7.4856	9.4327	9.569	1.9084	8.6801	3.5389	7.5385	7.4242	9.9396	9.9166	9.4859	0.8375	10.3823	11.1265	10.6266	8.7829	8.513	7.3737	5.603	3.1242	8.2098	4.55
TCGA-55-1592-01	701	1	6.8208	8.3162	6.0075	10.5924	7.2378	12.0511	7.8805	6.1407	5.6513	9.4645	3.8612	9.1546	3.9649	8.1734	6.7645	6.4169	7.4202	7.4199	1.9917	10.2733	12.0251	8.907	10.5249	9.1415	4.4667	8.9542	4.3942	10.4501	7.1798	7.761	10.9956	9.979	2.0867	8.8658	11.7346	8.4484	9.145	10.2507	7.504	7.2011	6.9253	9.0151	6.3845
TCGA-55-1594-01	1178	0	4.0295	9.0671	6.6333	10.9016	8.2008	12.5184	9.4032	6.3221	4.2835	11.1087	11.3991	6.6616	5.7141	6.0152	5.7671	5.3882	14.5482	14.7862	4.0973	7.7681	11.5824	8.5039	8.2332	10.0217	3.7209	8.3421	4.2241	9.3906	6.8552	9.7189	10.0658	10.1034	1.3631	9.9041	7.5032	9	9.0176	10.0636	7.2987	4.3124	4.1042	10.186	7.5584
TCGA-55-1595-01	1479	0	4.6849	8.9445	6.62	9.9059	5.6825	11.2271	6.166	4.6147	7.0536	9.3595	7.9067	1.7831	0.3843	6.1783	6.9577	3.9959	6.3446	4.1527	0	12.4199	11.4503	8.0128	9.6548	8.5479	5.1616	7.756	5.0195	10.9711	5.0728	7.4424	8.4239	9.6334	1.5014	8.6123	11.458	8.912	8.6986	10.4351	6.2837	4.5598	4.6737	7.7519	6.9036
TCGA-55-1596-01	2065	0	1.127	9.1168	6.2627	10.2653	8.8413	11.9098	8.7824	5.926	5.017	8.9484	3.9269	8.8616	3.0188	7.0297	5.809	5.7145	11.6605	13.7523	5.6135	4.0863	12.1593	9.9976	10.1445	8.3305	6.4106	7.9809	6.0728	8.5583	7.281	8.87	11.5216	10.742	3.5431	11.1899	11.0226	10.1839	8.5718	11.0986	8.8616	8.063	4.3452	11.1431	11.4112
TCGA-55-5899-01	930	0	0	8.5932	6.267	10.8042	4.8002	11.0704	11.2124	7.1914	6.4573	9.2077	4.4312	3.0755	8.3007	9.5654	6.5215	7.0922	15.3209	14.9503	10.2775	3.5679	11.9556	9.5235	9.5828	9.219	5.2318	8.9015	4.7085	7.9994	8.3968	11.5634	11.0662	9.7093	0.6521	10.9572	10.3582	9.2559	9.1554	9.6428	6.0226	10.3322	10.2106	9.8844	6.9461
TCGA-55-6543-01	435	0	4.7356	8.9687	7.3604	11.2208	7.5696	12.2772	5.2185	6.0246	5.2185	9.4596	2.1192	6.7626	0	8.4556	6.5981	7.0804	6.9628	7.4551	1.0805	4.3969	12.2139	8.078	9.3427	10.1028	2.589	9.4931	3.4638	10.1811	4.2355	7.8841	10.6561	10.1983	1.0805	10.0393	11.8183	7.3407	8.6483	9.0422	6.6146	6.7992	9.2791	8.0987	7.3055
TCGA-55-6642-01	2449	0	2.4821	8.447	6.7169	10.4853	6.1388	11.4632	6.5554	4.0065	7.1012	9.4469	7.0664	2.0964	1.5685	9.2904	7.8436	5.9375	6.0701	4.6201	0	6.7871	11.4582	7.7324	9.0968	9.0601	2.9171	8.0539	3.1479	11.4934	7.4155	8.3892	9.2097	9.0707	1.2083	9.9405	8.7815	7.1751	7.8922	9.1741	7.5876	3.6017	9.4558	7.0379	9.3938
TCGA-55-6712-01	171	1	2.4545	7.8142	5.5731	10.4034	4.3728	11.8844	4.6553	5.6264	4.1722	9.6407	3.1805	2.8627	2.8627	6.9517	6.5984	4.6553	5.8889	3.5552	0	6.4436	10.7743	8.0999	9.6511	8.1233	1.8832	9.1483	5.7028	9.7978	7.3898	7.906	8.7003	9.1415	0	9.5287	8.2044	4.309	7.8979	8.627	5.6264	2.1969	4.4207	10.261	7.7007
TCGA-55-6968-01	1293	1	1.9493	8.3056	6.9483	10.957	4.3947	10.8612	5.2119	8.4937	8.6082	10.4497	7.0639	0.653	0	8.9479	6.9007	6.8938	15.1999	14.7124	5.9196	7.0823	10.3313	5.0716	9.3797	8.9612	4.9959	9.0804	3.57	9.0804	4.8024	8.8981	9.0574	8.3966	1.1008	10.9902	9.9958	8.1192	7.556	9.9083	5.6829	10.4447	8.8029	9.9237	8.8258
TCGA-55-6969-01	1239	0	4.7105	8.2391	5.3212	10.8789	5.9066	11.0386	9.5291	7.5883	8.6166	9.7567	3.5896	1.6181	2.2614	8.294	7.265	5.7303	9.2814	6.4918	1.9106	6.6238	11.8826	8.0341	7.9461	10.5762	3.67	8.6529	4.8377	11.0388	6.6439	8.3875	9.9335	8.8075	1.0249	11.0933	10.6437	8.633	9.1403	10.1033	6.9519	5.0777	12.03	9.4402	8.9441
TCGA-55-6970-01	464	1	6.299	9.4696	6.0698	10.0244	7.2477	11.2327	7.4037	5.6665	7.4938	9.5143	3.6877	3.1749	3.2253	9.835	6.7241	4.8393	14.6144	16.0772	0.4018	4.756	12.2477	8.5343	10.5469	7.179	6.1633	8.543	4.0088	9.9159	6.8216	8.1432	9.5404	9.4413	0.7159	8.768	12.7011	8.9802	8.925	10.8989	6.0349	7.8437	3.9359	7.5468	8.4599
TCGA-55-6971-01	1400	0	7.1334	8.4531	5.8847	10.979	6.8116	10.9845	8.2525	5.8608	5.3466	9.8127	4.7098	1.965	0.9768	7.4691	7.2296	5.3119	9.3795	7.5308	0	8.8143	11.8092	7.4612	9.0799	8.8511	2.546	9.4571	4.9546	10.5475	8.0086	7.9103	9.6043	9.7247	1.965	9.1578	10.9948	8.0514	9.0274	9.0193	7.1134	6.5536	10.0766	8.5569	9.698
TCGA-55-6972-01	1632	1	6.1882	9.0702	5.0919	9.0392	7.0721	11.7636	3.4949	6.5464	6.8474	7.9877	3.8776	5.5791	0	7.6246	5.3152	5.5791	16.8048	17.027	3.4949	1.7151	13.1199	9.6107	10.4889	5.8021	5.3559	7.1438	3.1685	8.395	3.6341	7.8056	9.3174	10.639	0	9.5216	12.4621	8.6209	10.0596	11.1023	4.1798	8.8308	2.1456	7.9181	9.6233
TCGA-55-6975-01	118	1	2.376	7.7963	4.9624	10.274	5.1443	10.9464	6.2563	5.4786	7.5016	9.4968	4.0812	1.8134	1.4203	7.1221	7.8445	6.4206	14.069	13.6654	0	3.6707	11.3551	8.3827	9.1381	7.9752	4.0079	8.1599	4.5627	11.8146	5.0743	9.7	9.7541	9.5336	5.0743	10.5236	9.5531	7.8757	8.7204	9.5786	6.7588	7.2705	3.8921	10.8972	9.4804
TCGA-55-6978-01	176	1	2.5954	7.9254	5.6643	10.7758	4.8807	10.4684	6.7207	5.9089	5.9929	9.8378	4.1352	2.8327	2.5954	5.7246	8.1135	4.9663	6.1179	3.3229	0	3.8767	11.6072	7.5265	8.6457	8.3081	3.0365	9.3115	4.3287	10.9255	6.4446	7.3015	9.5437	10.0496	0.4439	10.4222	9.0485	7.9016	8.8932	9.2265	7.4303	2.5067	7.8373	9.8977	8.3048
TCGA-55-6979-01	237	1	4.6415	8.5515	6.0863	11.1624	7.3803	11.5873	6.4307	6.0561	7.6058	9.9619	5.4357	6.7183	1.2687	6.2819	7.3742	4.3737	5.2874	1.9331	0	8.0891	12.0636	8.2039	8.7273	6.9579	4.7588	9.8265	4.832	9.6262	4.5138	8.292	10.4437	9.6002	0.7695	9.5194	11.618	8.1619	9.0958	9.4382	7.652	4.6817	7.2023	10.2963	9.0638
TCGA-55-6980-01	2109	0	4.4738	8.2366	4.4152	10.5195	6.5923	11.1388	6.1823	5.2531	6.6314	9.44	6.3942	8.5233	3.423	8.687	7.5028	4.1537	8.4918	6.5107	0	6.1102	12.4699	8.4339	9.1736	9.7956	2.6565	9.3285	4.0802	11.9949	6.6185	8.0869	9.6023	10.1657	0	8.7446	12.1044	9.0635	8.6714	9.8027	6.8682	2.1816	6.0792	7.3233	6.2509
TCGA-55-6981-01	1379	1	2.836	8.147	5.4298	11.3331	7.8507	11.6798	5.0642	5.697	5.9837	10.1482	3.8235	3.5269	1.4617	8.8935	6.5677	4.2129	6.3829	4.143	0	4.8615	10.7969	9.3051	8.4445	10.4386	0.9086	9.446	3.0032	9.9223	5.7684	8.4577	11.6852	11.0009	0	10.0873	10.1598	8.8142	8.5257	9.8168	5.8141	1.8606	7.7299	9.6472	5.697
TCGA-55-6982-01	995	1	2.7073	8.494	5.5289	10.4277	6.2417	12.8047	6.8144	5.0954	7.7097	8.9858	3.9773	3.9773	5.5289	7.7287	7.0025	6.188	10.4993	6.5132	3.6987	6.0137	12.2443	7.7287	8.2094	7.8718	5.4398	8.8342	3.0661	11.2046	5.5289	7.3192	10.9814	11.2057	1.9129	8.4221	10.4122	7.4225	8.4414	9.8413	7.8887	4.7448	8.9813	8.1545	9.5936
TCGA-55-6983-01	2823	0	5.1883	8.4451	5.8691	10.4993	5.9206	11.0456	6.4748	6.3169	5.9867	10.4521	5.6824	7.2884	4.3825	9.1624	7.3303	7.6937	11.187	11.3262	2.488	7.0697	13.4156	8.1835	8.6677	8.6113	3.6261	8.7615	3.4025	10.4801	7.1184	8.4087	11.1652	9.7861	1.0453	9.6884	10.8942	8.7135	9.4386	9.7838	5.9786	7.1035	11.2679	8.0908	6.7656
TCGA-55-6984-01	760	1	3.8788	8.4482	7.363	10.0897	8.1792	10.6754	4.829	6.1575	4.3998	9.3264	3.7863	9.448	7.5149	7.2288	8.3943	7.1001	10.3852	8.7715	3.2058	3.8788	11.5224	8.819	8.1608	8.8902	1.9039	8.3865	3.7863	9.1233	4.5197	7.455	10.207	11.2547	0	8.7067	11.9562	9.8984	8.2502	10.3238	7.0312	6.3797	12.9985	6.1389	5.8268
TCGA-55-6985-01	1233	0	4.8385	8.5503	5.8877	10.7163	8.075	11.4151	7.557	4.8004	7.6511	9.6205	4.9817	6.255	6.2829	7.7394	7.5105	9.0803	8.1602	6.4887	0	6.8477	11.464	9.1118	9.7494	9.733	3.203	9.7593	4.2969	10.2452	6.5125	8.9178	9.9747	9.8797	0	9.1558	11.4727	8.8709	8.9161	9.5754	6.6149	5.5854	7.8896	10.3329	8.2047
TCGA-55-6986-01	3261	0	6.145	8.628	5.0434	9.7137	7.7013	11.9201	6.069	5.1056	8.0724	9.281	3.2811	5.4068	1.2948	7.9632	7.8081	3.3851	7.8766	4.2607	0	6.4864	11.5481	8.8913	10.1107	9.0716	5.3044	8.9023	3.7393	9.9818	7.3299	7.8101	9.8483	9.5145	0	9.83	12.0937	8.6333	9.6744	9.7409	5.6969	7.7701	9.6691	8.4505	5.8682
TCGA-55-6987-01	2137	0	6.2784	8.4257	5.6462	11.0002	6.9153	11.5555	7.3525	5.9397	5.6462	10.4498	4.7429	6.1905	1.0422	8.0135	7.7855	4.4944	11.8709	10.2517	0	6.0894	12.1721	8.2173	9.0372	9.2908	2.8789	9.2019	4.1658	10.0599	7.3742	7.6785	9.4221	9.4882	1.0422	10.2522	11.7695	8.8445	8.9159	9.9343	7.4606	5.0806	7.8862	9.6579	8.8892
TCGA-55-7227-01	952	1	4.6145	8.148	5.9975	10.312	6.1172	12.1647	5.9007	5.8497	6.9382	10.0783	5.26	3.2161	1.6198	7.0776	7.4525	4.9081	6.8196	4.6145	0	5.9337	11.9172	7.92	9.03	9.413	3.7484	9.0916	3.9554	10.9902	6.4147	7.1927	9.413	8.9232	0	8.9456	11.3278	8.723	9.1348	9.3116	6.4607	3.9554	6.1612	6.013	6.3305
TCGA-55-7281-01	872	0	1.438	9.5079	4.0343	10.3445	5.8575	10.8586	5.0652	4.6399	5.0152	9.57	6.8733	2.1437	0.6506	5.9401	6.7258	4.4271	6.7102	2.1437	0	10.0656	12.6252	8.6016	8.9497	8.2806	3.4927	8.892	2.1437	9.6343	4.9634	7.4759	10.1191	9.4609	0	8.4263	11.7844	8.3223	8.7655	9.3033	6.5082	2.8615	6.26	5.9798	6.4992
TCGA-55-7283-01	609	0	7.0315	9.2336	6.9044	9.9672	7.4793	11.4917	6.6402	3.5883	4.6182	10.326	4.2129	4.558	1.5874	5.9343	5.798	6.3959	7.091	4.7584	0	5.2889	11.9746	8.3119	10.4646	8.8999	1.3241	8.3435	2.7035	11.0602	7.3873	6.3758	9.1984	10.2855	0	9.2797	10.8717	7.1934	8.6707	10.0425	5.213	2.7035	4.769	7.4671	6.9402
TCGA-55-7284-01	243	1	8.0067	9.7062	8.571	9.7807	7.5546	10.9342	7.7962	5.1152	4.8911	9.2873	5.0488	6.7494	4.8911	6.7824	7.7573	6.8267	8.9585	8.7767	4.4309	4.9793	13.1462	7.782	9.7067	9.058	4.5699	8.8428	3.8166	11.6207	6.3853	6.9135	10.3238	11.1453	0.9523	8.375	11.5944	9.5093	9.4539	9.7904	6.915	7.1132	11.2318	7.4556	5.5099
TCGA-55-7570-01	824	0	2.1654	8.142	6.6613	9.5331	5.5998	10.4085	9.7954	7.7272	5.1107	8.2529	2.2991	3.8147	0.9042	6.9063	8.1308	5.4206	15.3589	15.851	3.5182	5.7707	11.0083	9.5271	8.6459	9.5491	6.7042	7.1382	5.1989	9.4856	4.9971	10.6128	9.5641	8.963	1.4557	10.4925	7.5904	9.1583	9.6409	9.822	4.9971	8.0737	5.4614	9.8556	6.8088
TCGA-55-7573-01	487	0	5.3303	9.2266	6.7693	10.1399	6.4638	10.4468	6.6594	5.7291	6.2067	8.8272	5.3587	6.4769	5.1138	7.1159	7.7995	7.8302	9.4626	6.7904	0	5.4407	13.5274	9.579	9.8255	9.8902	3.7877	8.7988	4.3934	11.1647	7.6464	8.4735	10.203	9.7403	1.3792	8.4449	11.3447	7.8948	9.2628	9.9059	5.2414	6.7799	4.5604	6.9853	7.6806
TCGA-55-7574-01	995	1	7.605	8.6101	5.8015	10.3395	6.2557	11.5843	7.853	6.2854	7.657	9.5909	5.6727	3.9344	2.713	6.8691	7.9581	4.0074	10.5724	11.781	0	5.4809	11.8586	7.9113	8.8299	8.3923	2.5268	9.1652	5.2596	11.2052	7.7504	8.1473	9.9846	9.6488	2.0618	8.8499	10.957	7.6683	9.0874	9.2802	6.357	4.0769	8.4851	8.6305	8.1954
TCGA-55-7576-01	670	0	4.669	8.941	6.2013	10.6742	5.8985	10.7435	6.7224	5.6683	8.5495	10.3922	4.6268	2.3659	1.6778	7.673	6.2896	6.3855	8.8561	9.3276	0	6.3981	11.8335	9.2932	9.3532	6.841	5.6958	8.5719	4.3611	9.9872	6.7124	8.317	9.6952	10.1007	0.5741	9.2699	12.8096	7.6238	8.8914	10.1183	6.2391	2.3659	0.3155	7.4492	5.7227
TCGA-55-7724-01	705	0	7.542	5.9801	6.9217	10.3259	8.5459	12.144	7.8732	5.8357	6.3248	9.7892	4.5877	8.4291	3.1725	8.5067	7.4623	6.8608	11.9336	12.7676	0	6.6467	12.1734	8.2026	9.2906	9.1475	7.0029	8.9773	3.4621	11.7973	8.127	7.8512	7.5651	7.5186	0	9.3626	10.6709	8.9483	9.0824	9.5459	7.6539	5.3247	8.1095	6.7308	8.0363
TCGA-55-7725-01	442	0	8.9163	7.7145	6.1785	9.5506	6.9407	11.6667	5.6843	5.9026	6.6552	8.5755	2.6443	8.8423	8.9747	5.8511	7.1473	4.9266	8.6368	3.9732	1.0359	4.9755	12.9639	6.1993	10.2232	8.9986	3.0623	8.5137	3.0623	10.0163	5.8246	6.6894	7.6779	7.4629	2.379	9.1423	12.7811	7.0928	10.413	10.1029	6.6552	7.8651	1.6326	4.7121	7.7917
TCGA-55-7726-01	652	0	0.9163	6.1517	5.3551	9.9466	6.2048	10.1619	6.6236	6.3053	5.5589	8.8122	2.8502	4.535	0	6.8402	7.6987	3.0177	9.6189	11.2048	0	4.4201	10.4497	8.394	8.0424	9.4931	1.4723	9.1247	4.6414	10.1038	7.8112	8.6001	6.9057	9.8501	0.9163	9.6221	11.3492	7.6987	8.2161	9.0077	6.4875	4.535	6.5251	7.5766	5.9594
TCGA-55-7727-01	119	0	6.1627	6.1062	6.8767	10.1022	7.9847	12.1527	7.4462	5.7108	6.9851	9.1818	2.8918	3.2105	2.2233	6.3849	6.7348	6.0272	9.1214	7.7404	0	4.1304	11.4877	6.5912	9.6473	10.3474	4.2742	9.3777	2.4821	9.3717	6.0272	8.7326	7.728	9.1728	0	9.3475	11.387	7.893	10.1355	10.4747	6.1992	5.4021	4.0488	9.3717	7.0664
TCGA-55-7728-01	704	0	6.6479	9.6339	6.1273	11.1023	6.6101	11.143	5.7241	6.0487	4.287	10.0844	3.4597	7.3865	3.1341	6.6847	8.9124	5.1477	9.0837	9.9808	4.0495	3.5985	12.9985	8.379	9.2839	9.1416	4.8471	9.8743	3.3591	8.988	4.0495	6.9352	11.1187	10.5359	2.234	8.4388	13.1452	8.803	9.9723	8.7982	7.6724	6.1798	9.6305	6.209	6.785
TCGA-55-7815-01	773	0	2.0022	5.5574	7.1322	9.6539	9.2099	11.7486	8.1577	5.0028	5.7842	8.1149	2.0022	0	3.4621	4.5263	6.8484	7.3866	13.8944	13.7507	0	7.4947	12.2714	5.6467	9.5206	6.823	5.8357	8.6718	4.0027	9.6806	13.3702	9.0429	6.5107	5.8102	0	9.8794	9.5784	7.1927	9.0956	10.3768	6.7033	5.8102	8.8874	5.7308	7.6964
TCGA-55-7816-01	468	1	4.0801	8.303	5.8524	11.0789	7.0743	11.4419	8.8439	6.0051	5.9954	10.6994	4.3165	8.3589	3.2634	6.0243	8.924	6.3244	8.2991	7.3463	0	12.6867	12.0629	8.1454	9.6135	6.872	3.9254	9.0628	4.1517	10.504	13.2365	7.2877	10.1228	8.3665	0	8.4469	11.6754	6.4855	10.6548	9.1319	9.4704	3.3266	9.8043	7.088	8.5415
TCGA-55-7903-01	567	0	5.2805	9.0584	7.0825	10.0441	6.7093	11.0256	4.487	5.4213	5.8899	9.8931	1.3189	2.6962	0	7.219	6.0825	4.4546	5.9139	4.0397	0	3.5803	12.5222	9.1511	9.4451	11.6565	3.083	8.2429	5.2239	8.7398	5.165	6.9004	9.3426	10.3481	0.9975	9.1113	13.0658	8.9257	8.6152	10.0359	5.853	2.5808	12.1875	4.5801	6.1749
TCGA-55-7907-01	343	1	1.7423	9.1612	6.0421	10.2123	6.3902	11.103	4.7015	6.0759	5.8982	8.95	7.6124	7.7445	7.4859	8.1137	7.5774	7.8113	6.9513	4.3049	0	5.8004	11.4748	8.9356	9.9491	9.7421	5.1135	8.9379	3.5785	9.9907	7.5171	7.6572	9.957	9.7114	0.8334	8.6709	10.1931	5.9898	8.8817	9.7836	6.443	2.859	1.3582	5.1135	7.4669
TCGA-55-7910-01	1040	0	1.9175	8.1655	4.6481	10.4408	7.7224	10.6915	7.3734	6.5766	7.3895	9.9738	7.3974	0.9455	0	8.7221	6.2654	4.4787	9.9845	6.2827	0	11.2449	13.3643	7.8378	8.4892	8.9846	7.5116	8.814	3.4835	9.4778	4.7004	10.3549	10.331	9.6373	0.9455	10.882	12.2843	7.861	7.9501	9.1653	5.7736	5.415	8.092	11.1744	6.9983
TCGA-55-7911-01	537	0	5.5543	6.8277	5.9616	10.4256	7.2547	11.7119	5.0551	6.1772	3.7747	10.2069	1.8652	1.3706	0	7.531	6.874	2.6522	7.3086	5.5214	0	3.4651	12.0305	8.2144	10.0087	6.8675	5.5047	7.9534	7.0044	8.8729	3.7747	8.2894	10.0589	9.9621	1.0407	8.6192	11.5149	9.027	9.2823	10.165	6.1125	5.1223	8.5842	9.5508	8.2895
TCGA-55-7913-01	561	1	2.926	9.1469	3.7376	10.7377	7.7215	11.3087	8.7269	5.8025	6.8871	9.6181	1.1029	3.8279	1.4446	9.0858	8.1407	5.7263	5.4902	4.4371	2.4072	2.926	10.345	7.6978	9.5584	8.4791	4.0441	9.2112	2.4832	5.9234	2.5554	9.4357	11.5348	9.4335	4.8782	9.818	12.8019	10.3889	8.0199	9.5904	4.9061	6.5847	7.2501	9.1447	6.4633
TCGA-55-7914-01	187	1	4.6717	9.1854	5.1692	9.7699	6.1182	8.9271	5.4911	5.5843	7.1692	9.6317	2.1694	9.5625	8.3324	5.2088	5.9418	6.7204	5.988	3.2473	0.9996	5.0217	12.9381	8.3894	10.096	9.9337	2.6998	8.4871	4.0868	9.2801	6.6717	7.7183	9.7365	10.2305	0	10.3053	10.7917	8.2614	10.7221	10.3819	5.149	4.5229	5.1013	10.9708	7.4831
TCGA-55-7994-01	603	0	1.5359	9.3955	6.978	10.4656	8.3154	11.889	3.7724	6.4396	4.3667	10.3458	2.6007	4.8824	1.6854	9.2012	7.5236	3.5947	4.7697	4.2515	0	3.9002	10.7969	9.2074	10.0356	10.0374	6.5461	9.3267	7.41	8.5009	4.8824	7.7191	10.2727	10.9597	1.3691	8.6275	10.1942	4.5726	9.4396	8.7662	6.2486	0.9633	7.5222	6.7761	7.5579
TCGA-55-7995-01	889	0	6.6923	8.2771	7.2673	11.3284	5.8116	12.3674	8.1693	6.8562	5.8261	11.3779	3.1108	15.0258	1.2717	9.9649	7.0256	7.7378	14.197	15.3003	0.8865	12.0117	12.964	8.4006	9.0109	11.3471	1.937	10.1053	4.6466	9.439	6.5117	6.8547	10.6816	9.8337	0.3594	9.2489	11.721	10.1817	8.0995	10.1262	6.6644	3.6225	11.5288	10.7517	7.9946
TCGA-55-8085-01	904	0	3.3286	9.6055	4.8671	10.5013	8.3971	10.547	6.9069	6.3831	5.9758	8.7803	3.6815	3.1135	5.4642	9.1404	7.5534	5.8422	15.8765	17.0124	2.7898	4.9343	13.4758	8.6417	10.2562	9.2005	4.1739	9.2458	4.9343	8.6666	5.2301	10.2036	10.5308	10.4808	0.7621	9.6728	11.4757	9.6636	10.1708	10.0888	6.3403	7.5292	6.8913	10.4161	9.0646
TCGA-55-8087-01	462	0	3.4754	8.7987	6.5141	10.319	5.7931	11.2736	5.5886	5.5886	8.0996	8.5924	4.4784	9.0407	0.7639	6.9607	8.574	4.9712	7.2292	5.0185	0	3.7243	13.4391	9.1502	10.1021	10.3347	2.2748	8.8212	3.1744	10.7723	6.2815	6.7052	9.5051	10.3984	0	8.728	12.3721	9.4722	7.5418	9.4443	5.8378	7.5579	6.1277	9.4392	2.5574
TCGA-55-8089-01	702	1	5.5076	8.5485	5.4122	10.991	6.2291	10.5976	6.725	6.1886	3.7522	9.8746	2.7701	3.796	0	7.9273	7.2825	4.7873	12.5112	11.0048	0	4.1738	11.7717	8.0815	8.9367	8.655	4.6743	9.765	4.301	9.8526	7.6807	7.5501	10.2965	9.2263	0.8732	8.5404	9.1492	6.5632	8.9207	9.519	7.1828	3.3432	5.0197	6.9886	9.5231
TCGA-55-8090-01	598	1	5.1944	8.499	5.2424	10.5778	7.8678	11.2072	6.6806	5.3115	4.4429	9.8169	3.7758	4.9416	3.7428	9.8004	6.9679	5.3002	9.7695	9.2494	0	4.5985	11.2635	7.2462	8.6853	8.5899	2.1397	9.3805	4.3799	10.0403	5.0125	8.4947	10.7702	9.7746	2.0345	9.3979	9.6489	8.5607	9.9043	8.7448	5.7371	3.022	9.1294	7.0745	6.0452
TCGA-55-8091-01	600	0	5.8491	8.5201	5.5003	10.6032	7.4205	12.5861	6.4782	6.8968	5.4762	9.2576	3.524	9.9738	4.3042	8.3465	7.9706	7.6931	11.0234	11.7391	0	5.4141	12.4888	7.2567	7.6667	9.1262	3.2099	9.2717	3.6152	9.6957	5.8207	8.8826	10.3858	9.4494	0.8076	9.1888	11.1788	7.302	10.0068	9.0019	6.4351	5.9576	10.3405	7.4793	7.6425
TCGA-55-8092-01	154	1	6.8158	8.6694	5.3273	10.6433	5.1397	9.1008	5.6029	6.6078	4.2322	9.4876	2.1887	7.7451	7.8738	10.71	6.0237	4.0505	10.6336	11.9739	8.3298	3.8426	12.7239	8.4739	10.0189	10.2515	4.7443	8.5184	3.5995	10.378	7.9354	8.4325	9.2952	9.7226	0.531	10.7685	11.1333	9.1893	9.5199	10.2976	5.9837	2.0405	8.7813	8.8666	8.9468
TCGA-55-8094-01	541	0	3.2241	9.2043	4.888	10.7818	7.8596	9.5523	9.0457	7.4711	5.7402	7.7675	1.4793	1.1323	3.8786	9.3849	4.888	9.3164	8.8265	7.1584	7.4516	4.5269	11.2032	8.9464	8.2125	10.2441	4.7023	7.752	4.735	7.4268	8.0917	8.4004	10.2377	10.3051	0	10.8863	11.5451	9.0028	7.6784	9.8862	3.9359	9.8135	4.3276	12.3462	3.552
TCGA-55-8096-01	719	1	2.5825	8.5964	6.0752	10.8352	5.5497	11.2196	6.7164	6.3144	7.524	9.8224	8.1023	6.8354	6.9814	8.0874	8.0725	6.3313	5.8204	6.0951	1.8052	10.4848	12.253	8.3853	8.2864	10.1781	2.6557	9.1168	5.1544	11.4377	6.8896	8.6091	11.0711	9.6694	1.3557	9.2674	11.5738	8.0249	9.2215	10.1388	7.0166	3.8489	9.435	7.6835	9.0187
TCGA-55-8097-01	476	0	6.4769	9.6316	5.6508	10.1405	5.6159	10.5953	5.9784	4.9176	7.185	8.8144	5.9413	5.8838	1.5857	6.2145	7.8604	3.7877	8.3186	7.2203	0	5.7614	13.4018	8.8523	10.6646	8.0748	2.2025	8.2348	3.869	11.2163	6.682	6.7317	9.5661	10.4624	0	8.791	11.0687	8.6842	8.5974	8.6385	5.9966	6.1346	4.9777	8.9779	6.0235
TCGA-55-8203-01	547	0	5.1113	8.3259	5.7969	10.4623	6.1975	10.9684	6.0379	6.9629	5.7434	9.2998	8.8095	4.4346	0	9.013	6.425	6.6853	9.4781	5.2859	4.3796	6.3483	12.3144	8.4378	9.7173	9.3099	2.3346	8.6386	2.214	9.8722	6.3555	8.9543	10.7702	9.445	0.855	10.1417	10.8586	8.2398	9.3017	10.1391	5.5453	7.1137	10.4642	7.3644	7.8923
TCGA-55-8204-01	515	0	3.9019	7.7593	6.2901	10.2107	6.2398	9.8713	5.5256	7.2858	6.3194	9.5432	4.3444	8.9343	4.931	7.3536	7.8085	9.3787	12.1737	12.7802	2.0769	6.2601	11.72	11.4766	8.899	10.1581	1.6535	10.6431	4.8794	9.564	6.5599	9.1492	9.8353	8.9232	1.0517	9.8106	12.1105	8.2662	8.2737	9.2524	4.9562	5.6694	10.0992	9.7435	8.2662
TCGA-55-8205-01	599	0	5.2247	8.4964	5.0565	11.0443	6.8485	10.9251	6.3522	6.0894	4.8817	10.5543	4.5527	3.0494	1.3687	6.3689	7.8283	3.8687	6.1866	5.0971	0	5.1623	11.8625	7.0787	10.3016	9.1452	3.1035	9.538	4.0993	9.7785	7.0888	7.5933	9.8255	10.1599	0	9.1298	9.0851	7.8581	8.1119	8.6384	7.5572	3.1035	6.6842	6.2894	8.6633
TCGA-55-8206-01	888	0	4.4957	9.164	5.3634	10.2367	7.2886	11.0868	6.6514	6.1496	6.685	9.8368	5.4439	6.3032	3.2125	6.0503	8.0338	4.398	9.7548	10.4479	0	5.9435	14.0694	9.2644	11.5848	8.0943	3.8369	8.8738	4.0577	11.1446	7.9467	9.4008	9.7011	10.6866	2.788	8.9931	10.7518	8.5605	10.1937	9.3101	8.9061	6.7298	4.1566	8.2702	6.9283
TCGA-55-8207-01	977	0	4.34	10.185	6.2026	9.8926	6.3055	11.4225	6.5711	5.6344	7.6416	9.6805	8.3068	5.9882	2.6054	7.58	7.7527	6.2446	6.5766	5.1202	0	7.5383	12.576	8.0835	9.4176	7.8716	5.9799	8.3117	4.7631	12.0831	8.4304	8.1579	9.5948	10.0332	0.447	9.2366	10.4533	8.0283	9.8742	9.7348	6.7127	5.5134	4.79	9.169	4.1757
TCGA-55-8208-01	674	0	4.9975	8.5424	6.2535	11.3879	7.4749	11.3892	6.7308	6.9208	6.292	9.9405	4.4689	9.6239	4.6868	5.7988	7.7826	6.3751	12.6353	12.0065	4.5021	8.2368	12.5132	8.4958	8.6258	8.4834	5.702	9.8382	4.7979	10.0986	7.0667	7.9665	10.4583	9.4519	0.6	9.2245	10.014	8.5243	8.956	8.8616	7.0887	5.453	5.5033	8.4999	9.1557
TCGA-55-8299-01	469	1	6.898	8.5204	5.5941	10.9422	6.2025	10.9096	7.766	5.95	6.5469	10.4487	4.7193	3.773	1.8214	7.3087	8.3756	3.531	9.4685	8.9689	0	5.0115	12.1942	8.4065	9.0343	8.9846	2.7893	9.1546	4.809	11.3759	8.2671	8.5932	9.6218	9.5666	1.8214	9.2488	11.4977	8.0972	9.6334	9.5464	7.3467	5.91	7.713	10.0714	10.3961
TCGA-55-8301-01	534	0	6.856	8.4593	5.3423	10.7896	5.0178	11.8259	7.088	5.7407	5.3726	9.6012	5.5552	2.8118	0.5161	6.856	7.1062	3.2634	6.6613	5.5286	0	9.2405	12.5636	8.357	8.924	9.0168	3.752	8.6976	3.752	10.5716	5.6448	8.0587	10.4182	9.951	1.1955	10.5806	12.0887	8.6583	8.8587	10.1731	6.2692	2.6232	12.3053	11.3189	9.3567
TCGA-55-8302-01	478	0	4.7265	8.8936	5.6521	10.4602	6.8114	10.7748	4.8143	6.1109	7.4411	9.5363	4.8424	5.699	1.0756	7.9573	6.3254	5.4658	10.3749	9.5287	0	3.837	12.0764	8.2237	7.8747	8.101	3.3017	9.2893	7.8262	9.8811	5.4476	7.967	10.9344	10.4927	1.6874	9.8099	10.2791	8.1068	9.066	9.6451	6.572	3.7206	9.0814	10.6377	6.572
TCGA-55-8505-01	440	0	3.6084	9.6931	5.7715	9.9311	7.166	10.7177	6.0912	5.4405	5.8174	8.6651	4.8428	8.5762	9.0261	8.5717	7.1541	5.2073	14.3116	15.4498	6.8495	5.7078	13.4233	9.0694	8.9352	9.5865	5.9051	8.4137	4.8428	10.7678	8.0941	8.0395	10.9541	11.4515	0	10.7019	11.4558	10.2262	8.6287	10.286	5.8765	5.318	8.895	10.6081	6.3106
TCGA-55-8506-01	11	0	2.1304	9.6799	3.8022	10.4903	7.1531	12.4394	3.6808	6.2023	4.4485	10.1675	2.3049	1.0882	0.6444	8.0035	6.2133	3.9142	4.4485	4.2493	0	3.8593	12.8764	9.4391	8.2582	6.5003	5.6106	9.3987	2.8467	8.8869	4.1151	8.3773	10.0607	9.8803	1.4272	8.4792	13.3563	7.8216	8.6588	10.4174	5.9629	0	8.0697	10.7406	6.6125
TCGA-55-8507-01	418	0	6.4073	9.8705	5.6508	10.535	7.4665	12.2944	7.8105	6.3317	6.8134	10.1345	5.9701	3.4959	1.3914	9.1011	7.0789	6.2105	7.4841	6.2724	0	8.3328	12.8444	7.8346	9.15	9.7423	3.1003	8.0384	4.601	10.6609	8.0994	7.9471	10.7218	9.9034	0.624	8.9372	10.282	7.6909	8.926	9.6462	6.4346	5.6034	7.8806	8.651	5.0647
TCGA-55-8508-01	617	0	5.149	8.3965	5.9126	10.8154	6.2675	11.0295	5.8027	6.1646	6.3164	10.3235	4.9976	4.3542	0	7.2747	6.9836	3.9209	7.7947	6.1286	0	6.4967	11.9983	7.8946	7.637	9.1219	2.1816	8.5976	2.8458	10.4252	7.3153	8.6536	11.218	9.0026	1.8689	9.1758	9.5908	7.4527	8.6557	10.0788	6.8899	2.1816	10.0003	6.9633	8.7886
TCGA-55-8510-01	539	0	5.8406	9.0542	5.4742	10.2528	6.7562	11.1946	6.9685	5.3058	7.2023	10.5454	5.3498	6.9685	1.7858	6.9825	7.8058	5.7614	12.3633	11.7248	0	5.1152	12.3337	8.6395	9.2365	9.4268	2.5597	9.5754	4.6713	11.1556	6.1071	6.956	10.6362	10.4481	0	9.7101	10.0192	8.354	9.6673	10.2864	6.8813	4.9536	3.9177	9.9179	7.2725
TCGA-55-8511-01	552	0	1.0607	8.6165	6.6255	10.1625	7.0546	12.2364	5.6711	6.3842	7.2525	11.051	3.6325	3.0105	2.8017	6.0359	7.0487	4.8961	6.4484	6.1725	0	2.8017	10.7983	8.9547	8.2834	8.4086	2.4178	8.7858	3.3548	10.3068	5.7605	6.7512	10.1876	10.4255	1.3945	7.8932	11.6284	7.8463	9.0787	9.571	6.0118	2.0901	0.6257	10.7455	7.422
TCGA-55-8512-01	607	1	6.9219	9.1608	6.7818	9.9147	7.4355	11.3414	7.7396	7.167	5.9894	9.4216	7.0784	9.3212	7.4532	8.365	8.22	10.2735	11.6319	12.3109	1.0489	6.3618	13.4439	9.6294	10.2735	7.6541	3.4088	9.5317	4.8476	10.7177	7.5714	8.6161	10.3056	10.2685	1.6498	7.7787	11.7774	9.7381	9.5789	9.2356	6.1716	6.4788	2.8458	8.0616	7.9287
TCGA-55-8513-01	791	0	8.5156	9.3313	8.5009	10.4267	7.7997	12.1215	7.9885	6.1291	5.8047	10.2888	5.8974	7.3528	5.8047	7.5824	8.8669	9.0456	9.6303	8.498	5.577	5.5544	12.6722	9.1302	9.5512	8.1852	5.5081	9.7495	4.4927	11.2311	7.6585	7.1909	10.7374	10.1943	0	8.7274	11.0517	8.5617	9.6451	9.534	6.9984	6.7123	11.2881	8.6362	7.7458
TCGA-55-8514-01	520	0	3.5011	9.2281	5.5286	10.0936	6.6397	10.066	3.9854	5.6995	3.5011	9.3129	6.0051	5.7695	0	6.305	5.9758	2.7669	5.3347	3.8628	0	4.5195	12.3231	9.0162	8.659	11.1427	2.8976	8.6999	3.9854	9.6056	6.4715	5.8607	11.3305	9.9783	1.5536	9.2312	12.0769	8.4164	8.1672	10.1675	6.6303	1.1955	10.5085	7.4843	5.9459
TCGA-55-8614-01	536	0	6.392	8.8213	5.492	10.5444	6.8108	9.735	6.7018	6.5061	5.7994	9.5239	3.7626	7.9977	2.735	7.4982	8.1808	6.2443	10.033	7.4209	0	5.512	12.1	10.6378	8.6513	8.9824	2.2545	8.2716	5.3875	9.7445	5.409	10.7399	10.4943	9.6641	0.7036	9.0558	11.1752	7.5763	9.7243	9.433	5.3875	7.9062	7.5019	9.952	6.9122
TCGA-55-8615-01	446	0	0	9.764	6.3625	10.3984	6.9227	10.7362	3.6741	6.0842	5.2842	11.1097	5.5377	1.0493	2.4	8.7096	6.9035	6.7033	8.8466	8.1217	0	2.6662	12.2513	8.0479	8.4073	8.984	3.7333	7.7475	3.2564	8.7885	8.737	9.6879	11.1811	8.8566	1.3809	9.2952	10.2178	10.0503	9.547	9.8016	7.7475	3.7902	5.6857	7.0735	4.0451
TCGA-55-8616-01	48	0	2.6443	9.932	5.8641	10.4481	4.0666	11.6038	5.7134	6.1993	4.2767	9.7296	5.4956	6.1468	4.1543	8.1395	6.2095	7.7466	8.2996	5.8111	0	4.3152	12.94	9.6661	9.7886	9.9242	1.0359	8.3972	4.46	10.8915	7.5544	8.1673	10.6101	10.3441	0.609	8.8439	12.6352	8.3352	8.7236	10.2925	5.3171	3.2332	1.7656	7.9925	5.9026
TCGA-55-8619-01	416	0	7.5362	9.3708	7.3324	10.5637	6.8547	11.2874	7.5453	5.5902	5.8158	10.1744	5.4816	6.4462	2.7706	6.5029	8.4289	6.2939	9.1607	7.4135	1.7353	6.2056	13.453	8.681	9.0398	9.5304	4.2521	8.9061	4.994	12.324	7.7386	6.8878	10.9385	10.1986	0.6621	8.5346	12.9324	8.15	8.9712	9.61	7.3272	7.0638	11.559	6.7878	7.8915
TCGA-55-8620-01	375	1	3.3118	8.913	6.3358	10.4846	3.1275	11.3258	4.2372	7.4645	9.9666	9.7953	6.6669	1.9915	3.9343	7.5123	7.6297	7.1263	15.5049	14.6817	4.191	5.7859	10.2101	9.8439	10.1303	11.354	2.6686	10.1748	6.6153	8.6541	6.1787	8.1888	10.2794	10.0804	0	10.0197	11.4602	10.0155	8.7228	8.9876	5.5679	10.7922	9.6078	9.369	7.8799

TCGA-55-8621-01	515	0	6.9935	9.2013	6.2479	10.914	6.9378	11.7716	7.9445	6.1749	5.1627	10.8069	5.9051	6.2274	3.6764	8.284	8.5317	8.5107	10.0966	8.1075	0	8.2666	13.3091	8.3776	9.3472	9.3389	4.587	8.7286	4.3799	12.4456	7.5828	7.836	10.6801	10.3336	0	9.7269	10.9543	8.0209	9.1491	9.2829	7.5252	5.77	8.6542	8.8806	8.0906
TCGA-55-A48X-01	689	0	5.9789	8.7355	5.945	10.5039	7.0009	11.7154	6.8501	6.1673	8.3402	9.662	6.5525	5.8746	4.0566	7.0602	6.6818	7.4552	7.936	7.172	0.5741	5.5377	11.9013	9.0198	9.9236	7.7189	3.5549	9.446	4.6124	10.5929	8.2937	7.1563	11.1154	10.1087	0.5741	9.2223	11.6407	8.5728	9.6246	10.0116	5.5972	5.6821	3.1247	8.5352	7.7939
TCGA-55-A48Y-01	630	0	5.3546	9.1423	5.3912	9.73	6.3647	10.5472	7.0983	5.135	5.6695	9.5505	3.5885	9.9459	6.3739	9.7228	7.042	6.1468	15.0348	14.1617	6.57	4.7121	13.0484	9.1436	9.9428	7.8487	4.8233	8.8587	2.7607	10.4454	6.1143	7.7427	10.1457	9.6283	1.0359	10.6549	12.4669	8.2103	8.7953	9.814	6.471	7.878	3.1773	10.3069	8.608
TCGA-55-A48Z-01	651	0	3.9007	8.763	5.5138	10.6314	5.6163	10.8481	6.4902	6.7634	8.5278	9.6532	8.5557	7.2331	4.0753	8.6294	7.3311	4.5889	7.3311	5.9323	0	8.8407	11.1826	7.5053	7.76	10.7031	1.7667	9.2849	3.8047	10.4481	10.0506	7.3896	10.3727	9.8875	1.9572	9.9272	11.5888	8.4878	9.479	9.5983	6.7246	3.9907	10.3864	10.1014	6.9488
TCGA-55-A490-01	99	1	0	9.0294	6.4848	10.2483	5.3696	9.6659	3.8852	4.8105	5.4851	9.7917	8.3841	4.4041	3.3713	6.6705	6.7518	5.6066	4.2589	5.5471	0	3.2272	11.4388	6.7452	7.3863	11.2546	0	8.4301	6.2439	9.5419	7.2943	7.638	7.3606	9.2587	0.9884	7.6315	11.5816	8.516	9.1009	8.6773	5.1869	0	3.2756	7.0376	5.3867
TCGA-55-A491-01	626	0	0.613	9.787	5.7532	11.2654	8.6096	10.2597	8.9495	5.1243	5.8491	9.4227	4.7797	10.1226	8.3512	7.0006	7.1207	4.5707	8.6615	5.7951	1.3719	8.11	11.7017	9.0262	8.242	9.8961	5.6042	9.9488	5.7813	9.4288	6.5244	7.7819	11.3231	10.1691	1.0418	10.0574	13.2274	8.7722	9.1266	10.2954	8.4615	8.4745	6.3758	11.3374	7.0301
TCGA-55-A492-01	596	0	6.0046	10.7181	1.6519	10.0743	7.8933	11.7794	5.589	6.3268	5.5233	9.1885	2.8929	8.7429	1.879	8.887	7.1153	3.6145	17.4798	16.338	2.2477	5.003	14.3404	9.0634	10.134	7.8267	4.5211	7.9286	3.7923	10.1278	5.3636	6.6076	12.2515	10.8203	0.6188	9.8423	10.67	9.8339	9.2232	10.6676	7.4953	8.3728	5.9522	10.2136	10.6776
TCGA-55-A493-01	28	0	1.5554	8.2661	4.8395	10.2997	7.1356	10.5152	6.2133	5.1854	8.4353	10.3745	4.1278	3.1303	0.5703	6.7759	8.1404	3.0481	6.1259	3.7659	0.5703	5.1064	11.5059	8.4373	7.8804	9.8758	1.7756	9.3097	4.2057	8.6919	4.8638	6.629	10.0501	10.61	1.2953	9.0834	10.9802	8.4613	8.6628	9.1576	5.789	0.5703	4.3709	9.2269	6.5257
TCGA-55-A494-01	481	0	0	9.7747	6.7532	10.6387	5.9993	10.3422	5.7248	5.4006	5.8957	10.6014	8.7872	0	0	7.3709	5.3228	5.6755	4.4491	0.916	0.916	4.6662	11.2727	8.9409	9.4519	10.2931	0.5296	9.5439	2.1851	5.4306	11.1998	6.1033	10.5689	11.1455	0	9.0834	11.5139	9.4871	5.7724	9.3865	6.9419	4.4781	7.6721	8.6443	0.916
TCGA-55-A4DF-01	614	1	7.0776	7.7578	7.9153	10.8803	7.8761	11.6568	5.4131	8.7866	7.0325	10.5464	3.0699	10.3729	1.9161	7.3139	7.612	5.9867	13.5921	13.0633	2.6054	11.3372	11.9178	8.5401	9.1702	7.7077	1.2552	9.391	3.8943	8.5807	5.4441	8.8911	9.1932	8.7201	0	9.3113	9.1644	8.8224	8.8356	9.7887	6.3313	5.7092	13.1652	7.2757	8.0037
TCGA-55-A4DG-01	608	0	6.389	8.8826	5.63	9.7713	7.3895	11.6103	6.416	6.5681	5.857	9.422	4.4309	5.3971	1.6368	7.8905	6.2459	5.415	8.563	5.4847	0	6.4425	12.2546	9.3355	10.5179	10.3184	2.6495	8.9129	3.4622	10.1538	7.6803	8.4639	9.7661	9.8476	1.8628	9.1213	10.8584	9.3623	10.2711	10.212	6.2257	7.1745	4.5228	8.1905	8.0075
TCGA-55-A57B-01	546	0	5.7984	9.1171	5.2218	10.1048	6.4377	11.3822	7.8177	5.5819	6.7528	9.4582	5.7234	4.5801	1.9339	7.7625	8.3081	5.6843	9.3658	6.8441	0	5.4367	12.8071	8.8612	9.7194	6.6901	3.295	8.0657	7.0269	11.4026	6.1745	7.7414	11.3921	10.8053	0	9.7901	11.7383	8.3382	9.9768	9.2102	5.6843	3.5766	6.8672	8.6031	4.3991
TCGA-62-8394-01	139	1	5.9378	9.7616	5.7387	10.951	6.9601	11.1592	4.8717	6.9504	7.2826	9.8673	8.4678	9.7971	5.2949	8.1208	5.8469	6.3444	8.8221	6.5312	1.8053	8.4796	12.5169	8.7276	8.973	10.1973	2.4788	8.0682	4.1739	9.3353	7.4736	9.415	10.2206	10.5177	1.8053	9.4683	12.0391	9.9787	9.549	9.3629	6.3737	4.4179	9.4409	8.5373	6.2608
TCGA-62-8395-01	1216	0	6.0232	10.2793	5.8908	10.2105	6.9158	11.7093	10.7631	4.5192	8.2274	10.1273	5.9432	7.3705	6.761	10.3288	7.3857	7.065	14.0991	14.654	0	5.4732	12.4636	10.32	10.299	6.6008	4.4914	8.8585	4.2129	10.6788	7.3705	9.5484	10.1862	10.2839	0.5247	7.8779	11.8242	8.2421	9.9119	9.8211	5.5428	3.2224	3.8698	9.0968	6.7956
TCGA-62-8397-01	1289	0	1.9707	10.5719	7.0326	9.4028	7.3999	10.8111	3.4879	5.1714	8.7875	8.9462	3.053	5.7031	5.2287	9.8534	6.2923	5.388	9.6948	8.594	0	4.5171	14.0105	8.9916	10.9151	10.6461	1.9707	7.6193	2.4275	9.9651	5.8563	6.9128	11.0444	10.3365	1.9707	8.4018	12.2457	9.3425	11.4525	9.6483	6.3189	3.2867	1.192	7.3747	5.4853
TCGA-62-8398-01	444	1	3.2679	10.0547	6.7343	10.2362	5.9408	10.2943	5.8404	5.7924	5.4149	9.4666	5.2396	3.2106	7.954	9.7345	6.6559	6.8561	12.8699	13.1558	6.4296	3.6594	13.1044	9.7326	9.355	9.7603	4.9379	9.9757	4.3849	9.7918	4.2204	9.2416	10.2856	10.1067	0.4597	8.428	9.4868	9.7628	8.6178	9.9757	5.9141	7.3665	8.2758	7.8166	7.1006
TCGA-62-8399-01	2696	0	6.1337	9.2817	5.1309	10.2077	7.1194	9.7788	6.6934	6.1414	5.8607	10.8207	5.4257	3.8694	1.709	6.9311	6.453	3.7928	7.6811	6.508	0	9.9066	11.3631	9.4918	10.4629	9.1515	0.8126	7.5299	4.1409	10.8593	8.058	9.035	8.9378	9.6503	0	6.5668	11.5983	7.491	9.3701	9.894	5.6916	2.4695	5.1817	8.8776	8.2867
TCGA-62-8402-01	1498	1	3.3028	9.0354	5.0237	11.1973	6.0875	11.9403	6.9619	7.0449	4.1092	10.2047	9.0313	1.984	0	4.5646	5.4221	2.7891	4.0674	2.3049	1.3092	7.5257	10.7252	8.8085	9.0039	6.4219	0	8.8796	3.5041	7.3582	7.5372	8.7405	11.1246	9.1636	1.3092	9.3603	7.7303	7.737	9.0899	9.2326	6.7927	8.4585	4.3384	10.8602	7.8274
TCGA-62-A46O-01	1454	1	6.2736	8.2778	8.4066	8.8342	6.5387	10.0037	5.3817	6.5645	4.7356	11.6651	4.5071	2.1192	1.6914	8.9097	5.1294	6.3045	9.4706	11.802	1.0805	4.2355	9.786	8.1017	9.4408	5.7832	8.8827	8.6523	4.4346	7.1613	4.6095	9.3601	9.8623	7.8461	4.8515	11.076	12.3122	9.0145	7.9309	10.1707	4.0538	8.249	2.2245	11.1458	6.7552
TCGA-62-A46P-01	594	1	6.2081	9.9097	4.69	10.3587	5.5504	10.9425	3.5269	6.4507	6.2657	7.5756	1.3241	4.7314	1.3241	10.4453	8.8724	4.8861	6.4507	3.4295	0	4.6034	13.0709	8.9135	9.3373	8.5495	3.7038	7.8522	3.2127	8.5025	6.1482	7.8542	11.2308	9.1872	0.8089	9.5079	11.8939	7.9563	9.344	10.1251	4.4128	4.1932	7.6716	10.5173	4.5112
TCGA-62-A46R-01	1725	1	1.0003	8.532	6.3669	9.7803	4.808	11.8987	4.21	6.3843	4.46	10.3912	5.63	6.8208	5.3405	7.8894	6.9893	5.7421	10.3135	9.4784	0	6.286	12.6777	7.9836	9.6544	5.045	3.0005	7.795	3.6444	8.4332	5.1299	7.6666	10.4211	10.3315	1.0003	9.7696	9.7918	7.258	9.5714	10.5959	6.7555	2.0005	1.0003	8.795	5.9548
TCGA-62-A46S-01	1653	1	6.0519	9.2376	6.5434	10.2364	6.2984	10.9633	6.2406	5.3638	5.3482	8.7356	3.8324	9.5332	3.2309	9.7676	6.7762	7.5459	11.7585	10.4093	2.7526	3.4796	12.6146	7.8656	9.3963	7.2139	3.787	8.8788	2.1801	9.5554	5.3638	7.6901	10.7856	10.8315	0.913	8.4594	11.4163	9.4419	10.2806	10.2016	6.2739	5.1827	3.5005	9.5341	7.3054
TCGA-62-A46U-01	2067	0	1.955	9.5686	5.8198	11.3228	6.1884	12.1417	5.7333	6.8333	9.0181	10.6183	2.1003	3.4415	6.3055	7.0093	7.2425	5.047	10.9363	9.7309	0.8655	1.7933	13.6043	9.3732	9.2655	6.9142	5.2785	10.1125	4.09	6.9385	4.0186	8.3954	10.0523	9.7434	0	10.0657	11.5203	8.8303	8.0334	9.0408	6.6086	1.159	6.0113	9.7735	8.9716
TCGA-62-A46V-01	2199	0	4.1993	10.9118	5.0556	9.6168	8.467	10.6181	5.7443	6.3381	8.3291	8.7662	2.8976	1.7997	6.9435	7.9694	7.2885	5.4157	6.2655	6.284	0	5.0119	14.2097	8.1889	10.176	6.8411	6.0979	8.2631	5.0119	9.8999	7.8441	7.503	10.2228	10.1932	0	9.6439	10.9853	7.8627	9.4007	10.809	6.1289	1.7997	12.6903	9.5938	8.8886
TCGA-62-A46Y-01	414	1	5.0696	8.6311	5.4793	10.5369	8.1959	12.0294	5.1734	6.1431	7.3119	9.5708	4.2129	7.339	4.6473	8.642	6.9519	7.5734	8.4773	5.1328	0	6.1634	12.6726	8.9343	10.638	10.5272	1.8099	9.3005	4.0034	9.1971	5.3254	7.7931	10.2324	10.5045	0.5862	9.8992	12.9323	8.9723	10.2809	10.0944	6.676	4.6182	11.2707	7.8647	6.8923
TCGA-62-A470-01	1194	1	7.1561	9.8392	5.4628	11.1197	7.131	11.3771	8.3975	7.1054	4.0883	8.7811	3.735	7.7082	6.2734	10.0633	7.3645	5.1983	15.875	14.9555	5.0453	5.6514	13.8314	9.4674	8.7385	8.7754	5.8797	8.4943	4.1891	9.7409	5.0981	9.9209	10.7658	10.2056	0	9.1886	8.6483	9.2158	10.0382	10.2789	5.9957	9.7758	8.4032	9.1007	5.3799
TCGA-62-A471-01	1246	0	3.1165	9.9306	4.3531	9.6972	7.7192	9.8625	3.4221	6.2612	3.6151	9.5104	3.5536	9.0607	6.1409	9.9899	7.3902	5.9149	15.7411	16.1991	4.8646	3.6151	13.2165	8.6327	8.3019	8.1774	8.1517	7.8238	6.7874	8.3019	3.5536	9.8473	11.0353	10.1103	2.3483	8.4569	13.7814	10.6201	9.171	10.2489	5.3358	6.712	6.4746	7.3725	7.9811
TCGA-62-A472-01	910	0	4.9048	8.5303	6.079	9.7402	7.915	12.4698	5.2722	6.6077	7.5566	10.2771	1.5511	8.35	4.3438	7.7306	6.9152	3.2761	8.9631	5.8567	0	4.6514	11.9504	6.8563	9.2597	6.3246	4.5361	8.9434	6.8321	9.0988	3.5383	7.1259	9.3606	9.7222	1.9619	10.4998	12.6514	9.1438	9.7023	9.8529	5.3078	2.5426	0	4.4748	5.4096
TCGA-64-1676-01	1728	0	2.9871	9.6647	2.1584	9.6056	6.3465	10.3266	3.8242	6.5628	5.008	9.2932	0	2.1584	0	7.0738	6.1633	1.9151	5.1556	3.5952	0.7595	4.3987	12.9108	7.6823	7.9959	11.4089	2.9871	8.217	3.2195	8.2665	5.2373	9.2223	9.8009	10.9796	2.3666	8.8357	12.9319	8.9269	10.5464	11.3997	7.7395	1.9151	3.543	9.7238	6.5307
TCGA-64-1677-01	628	1	6.4858	9.1983	3.8206	10.6104	8.1461	10.1726	5.6506	7.4865	3.2858	8.3418	3.2858	2.0984	2.0984	6.1502	5.8553	3.4392	7.4599	6.4681	1.0663	8.329	10.6155	8.2378	9.9142	9.5389	4.0283	7.5802	5.0799	7.8773	5.4843	9.2775	9.7626	9.9938	0	10.6365	11.8858	7.8502	9.4379	10.0603	7.4236	7.0712	7.9863	9.5089	8.06
TCGA-64-1678-01	1189	0	1.9296	7.6473	5.1175	8.7354	7.1635	11.5498	5.9293	8.613	4.3024	10.0801	8.4487	6.6478	0	6.4763	5.2298	3.9986	16.9519	16.4762	1.5226	7.3323	10.5502	7.1728	5.973	9.2719	0.9535	6.7265	4.4332	7.1635	4.9085	11.0082	8.1819	10.1828	1.5226	12.9264	11.4436	7.9879	9.7635	9.7836	5.3671	2.7267	4.4332	11.0146	6.6745
TCGA-64-1679-01	2488	0	0.3676	8.442	6.3049	10.0837	8.2311	10.8749	6.1485	4.8216	6.8277	9.8132	4.0339	5.7654	0	8.1075	7.9076	5.4482	7.1821	7.0142	0.6603	9.7617	12.0544	8.5307	7.9249	10.1618	3.9278	9.8991	4.9759	10.7	8.3203	8.4722	9.0927	8.8602	0.3676	8.2885	11.9279	7.9369	8.0784	9.8578	8.0969	1.9642	10.5674	7.9232	8.3111
TCGA-64-1680-01	1126	0	6.1243	8.7203	6.0118	9.4616	7.939	12.8631	5.1448	5.317	4.299	9.5093	2.6396	6.3916	3.6101	7.7615	5.995	7.2547	7.44	4.5951	0.8052	4.4546	12.4748	8.1662	10.4521	9.9422	3.9953	7.9301	1.697	9.5874	5.0826	7.4513	9.2809	10.5703	0	9.0824	13.4947	8.7075	10.1491	10.657	5.7764	7.1217	10.4044	4.6817	5.5875
TCGA-64-1681-01	1167	1	5.7094	9.6165	5.0199	10.6626	7.0505	11.4756	6.9696	5.768	5.7193	9.2029	5.6274	6.7106	5.9392	7.3058	6.3312	8.1698	10.7956	8.9388	0	5.739	13.9179	8.8803	9.932	10.4005	2.7619	8.5301	4.0792	11.0163	7.9618	9.1365	10.1167	9.8975	0	9.0581	10.6222	8.8216	9.4727	9.6763	6.6074	4.0792	8.6039	6.8208	8.3832
TCGA-64-5774-01	2676	0	0.953	8.9934	6.2544	9.9873	6.1345	9.2676	7.7752	7.1577	5.2467	9.7355	9.048	4.5231	0	9.0758	7.0189	7.3603	7.7314	6.3318	0	7.1145	11.0986	7.7752	8.6086	9.7038	1.9289	8.4125	2.7258	9.0956	7.1577	9.7014	10.6527	9.3783	0	11.1209	10.431	7.9546	9.2186	9.8137	5.6477	1.522	7.6955	9.0039	6.955
TCGA-64-5775-01	62	1	0.55	7.1269	4.6247	10.1547	6.0027	11.0439	6.8878	6.8821	4.357	10.5002	3.6015	3.7078	0	4.5408	9.1501	2.0871	6.1419	6.3856	0	6.4173	10.3381	7.5297	5.5241	9.5009	0	7.0732	4.779	9.0601	11.0892	8.5703	9.2656	8.7398	0	10.2763	7.5549	7.0329	8.6774	9.0374	5.7524	4.4209	12.0168	8.4378	4.6779
TCGA-64-5778-01	1305	0	4.724	9.3039	4.3729	10.5299	6.8014	10.3128	5.725	7.2627	4.1799	9.0973	3.1349	5.052	2.8535	8.2007	5.7391	5.1606	7.3662	5.8341	0	3.908	11.7902	8.5771	9.1384	10.1457	5.6819	8.9975	4.3363	8.7678	5.6673	7.9964	9.9552	9.8415	2.0404	9.0801	12.067	8.4819	10.115	10.1384	6.3009	5.8341	0.642	9.3027	8.3933
TCGA-64-5779-01	864	0	3.6388	7.6662	6.1901	9.9672	6.4143	11.4728	8.3769	6.2221	7.3777	10.4311	9.3185	3.8169	0	6.7899	7.0526	4.6283	5.4716	4.0486	3.4356	8.7198	11.0283	8.0868	8.0824	8.6772	2.0954	7.8868	4.2482	11.0734	5.332	9.3753	10.9576	7.9597	0.8626	9.4064	8.0693	7.9597	8.6392	9.0128	6.702	2.9158	8.3949	9.3959	8.8987
TCGA-64-5781-01	1559	0	0	8.8936	3.3224	10.02	6.1903	9.8862	5.2833	6.8832	4.9346	11.1714	1.193	1.5506	0	9.982	8.0895	3.4969	14.6103	14.0474	0	2.0757	11.6011	7.692	9.4579	9.1826	2.7629	7.701	4.6689	9.9028	9.04	8.406	10.2137	10.2942	1.8369	9.5561	13.4597	10.3104	8.9264	10.4615	6.1386	2.4599	7.332	6.6444	8.0089
TCGA-64-5815-01	866	0	3.9203	8.8546	4.708	10.6632	7.0029	11.1468	7.1378	4.1924	7.0887	9.585	3.1466	4.3673	1.3624	7.1297	7.9568	2.7002	7.901	7.4801	0	5.3598	12.1182	7.2537	8.9309	10.1648	4.129	8.5625	4.8326	11.423	6.2557	7.7784	10.1912	10.1499	0	8.476	11.6928	8.9675	9.023	9.0055	7.055	2.7002	6.527	9.0361	6.0992
TCGA-67-3770-01	610	0	5.1224	9.8807	5.305	9.856	6.5979	11.6393	8.6146	5.8662	4.7211	8.934	3.2416	4.7211	3.2416	7.8057	7.6252	7.562	11.1709	9.8735	0.9559	5.1224	12.8527	9.6006	9.7963	9.3008	5.9993	7.9552	3.7244	9.5124	5.372	8.992	10.4946	10.8234	0.9559	10.0217	11.5039	7.9277	10.4429	10.4134	7.1108	2.2508	3.8982	8.0907	8.933
TCGA-67-3771-01	610	0	2.0899	7.9536	8.0561	9.8772	8.9044	11.6803	5.7661	7.8475	5.416	9.2434	4.5329	11.1091	1.2035	8.3577	6.6348	5.7832	14.9305	15.8998	3.6698	12.0383	12.891	9.1996	7.4561	11.1435	5.253	8.6046	4.6119	10.0896	9.8731	10.2989	9.4884	9.5722	1.2035	10.0643	11.5253	9.1868	8.1484	10.3842	10.6978	8.2258	4.6824	9.1359	9.0692
TCGA-67-3772-01	573	0	7.5129	9.4817	5.6618	10.0274	6.7133	11.4652	6.9282	5.6882	4.8624	8.6884	4.04	10.4169	4.5529	6.6134	8.139	6.1061	6.6065	4.9525	0.554	7.6129	13.3251	9.8248	9.6955	10.663	1.5224	8.7803	2.0967	10.5731	8.4132	8.929	10.2867	10.9823	0.554	9.456	12.2049	7.1584	10.1448	10.1122	7.6604	7.0707	10.081	5.994	7.3361
TCGA-67-3773-01	427	0	9.421	10.9335	5.2083	9.7048	8.184	10.0825	4.8454	5.8201	5.4309	8.8731	2.3536	4.8454	3.2051	9.9893	5.3239	3.9438	8.334	6.0389	0	5.4309	12.4819	7.5231	9.3179	7.2091	5.1258	8.1942	3.4954	10.4371	5.0383	6.6335	9.9761	10.3144	1.0198	8.6578	9.0756	6.2089	10.9776	10.2106	6.5782	2.3536	3.9986	5.3961	7.1788
TCGA-67-3774-01	385	0	6.8495	8.6491	5.2736	10.2347	6.9348	11.2306	7.5259	5.4735	7.4853	9.6198	4.9844	7.9251	2.7545	9.0177	6.8811	7.4061	13.8764	12.8513	2.2726	5.0964	12.4941	8.4288	9.6678	9.2198	2.2726	9.1726	3.9717	10.8383	8.0796	9.2673	10.2627	9.3624	0	7.8312	11.1656	9.1289	9.8645	9.155	6.4571	7.7479	3.7195	8.2973	7.7692
TCGA-67-4679-01	448	0	6.4536	10.3213	5.3834	9.719	6.855	10.4279	6.2292	5.2374	7.055	9.1119	4.3173	6.262	3.0806	5.7023	6.7741	6.5714	9.1412	6.7589	0.6543	6.1614	13.3688	8.5622	10.8946	7.2953	4.5471	8.4995	3.4269	11.6634	6.3253	6.637	9.5214	10.7745	0	8.9295	13.2726	8.9055	9.955	9.6078	7.5462	7.7599	0	7.4875	5.6206
TCGA-67-6215-01	174	0	4.2667	8.7632	6.007	10.3685	8.1556	10.9605	4.6558	6.0803	4.1199	8.7398	2.2401	4.8913	1.5171	7.5854	7.3749	3.9214	8.5928	6.539	0	3.6056	13.8563	8.4844	10.1683	10.2151	3.0787	8.1462	4.0567	8.8566	3.8855	8.504	9.7934	9.7214	0	8.5518	12.6641	8.8566	8.4294	10.2142	6.0803	4.4251	9.35	8.7792	4.1505
TCGA-67-6216-01	141	0	7.3124	8.9916	6.2338	10.578	8.2335	12.0381	5.1843	6.2338	6.4823	9.0095	3.013	7.6496	0	8.9002	7.0491	3.1147	5.5537	3.2987	0	4.5841	13.0363	8.0044	9.9742	9.3335	6.0914	9.3597	3.4619	8.7063	4.2683	7.1275	10.8724	9.9793	0.6685	8.7345	12.4239	8.2671	10.6126	10.0572	5.8911	6.8497	11.8612	6.3215	7.7398
TCGA-67-6217-01	422	0	4.6695	8.7241	6.4593	10.1951	6.618	11.8021	5.7052	4.9604	6.952	8.6432	5.7966	4.2626	2.4721	6.4499	6.1133	6.4215	8.1447	5.4287	0	5.18	12.7888	9.9501	10.011	9.8717	4.0313	7.8954	2.3161	11.192	7.6479	8.5056	10.258	10.2145	0.6495	8.6258	11.5263	8.4112	9.3079	10.3825	5.8402	3.5611	5.1732	7.1613	7.5331
TCGA-69-7760-01	202	0	2.9765	7.668	4.3915	9.9708	7.296	12.2443	7.2315	4.5916	5.854	9.733	10.9697	3.6857	1.1694	5.3785	5.4212	2.7272	6.4462	4.2565	2.4255	4.159	11.4665	6.7099	8.6777	10.2647	2.2472	7.912	5.7406	9.7321	5.2423	8.5809	9.0984	8.693	0	10.0706	9.2408	7.1889	9.3662	10.9611	5.6357	5.4212	4.9364	8.0131	5.3785
TCGA-69-7761-01	186	0	3.8024	9.1142	7.5231	10.2768	6.3084	10.7307	6.2786	6.0272	4.9892	9.5974	3.9608	6.3084	7.6094	5.6625	7.2335	5.517	7.7499	5.8631	5.7946	4.4625	12.9656	7.6756	9.8515	8.2646	2.9023	8.9941	3.2682	10.2002	4.3901	7.0293	10.3937	10.7132	1.0561	9.0929	10.3134	9.0377	9.6404	10.385	7.5231	7.1537	10.7831	6.7311	9.5055
TCGA-69-7763-01	690	0	6.4083	7.8514	5.6285	9.9425	6.5333	10.1008	7.5755	5.4052	6.2493	9.7059	5.4052	4.1813	1.4716	9.4603	7.4445	5.1885	8.782	10.6997	0	5.6959	12.1655	8.285	10.5756	7.3998	2.6599	8.1624	3.8078	11.8125	7.1161	8.2565	9.4554	8.7105	0	8.263	10.5128	8.1713	8.9774	9.5403	6.3676	6.0705	3.0051	7.9374	7.8842
TCGA-69-7764-01	414	0	5.8587	9.5061	5.4628	10.1593	7.5081	10.6639	8.586	5.5265	5.4188	7.8615	3.5373	6.5521	6.3675	7.2371	5.7912	5.6072	8.7857	4.9468	0	5.2779	12.7179	7.7316	10.2248	7.3818	2.8008	9.2847	4.1357	10.2478	8.7032	8.102	9.7242	10.0499	0	8.3572	10.2151	8.7372	8.7461	10.3793	4.7827	5.0075	1.5799	5.6836	9.0413
TCGA-69-7765-01	165	0	3.4578	7.9444	6.887	10.088	7.5178	12.3861	6.2342	4.5529	6.2342	9.8234	5.8531	2.2465	0.6997	6.8088	8.2318	4.5529	8.2016	6.8484	0	8.5015	10.9044	7.483	8.8488	10.5159	2.5835	9.1008	3.7532	10.8931	7.7236	7.8627	10.1863	9.4656	0	9.3378	11.5398	7.1378	8.9794	10.1598	6.5643	4.3906	9.4205	9.1171	7.2426
TCGA-69-7973-01	230	0	5.8391	8.5894	6.4962	10.721	5.5364	10.8865	7.5067	6.6671	5.3931	9.7021	9.1779	5.1527	1.3494	8.4084	7.1589	7.7918	8.229	8.1875	0	11.9283	11.1525	7.908	8.751	8.001	0.6003	8.8584	3.2887	9.9527	7.6945	10.1815	10.5631	8.971	0.3311	12.0835	10.6442	8.298	7.7918	9.6725	5.9456	3.6566	3.5839	10.781	6.6524
TCGA-69-7974-01	184	0	6.2581	8.657	5.9573	10.5431	7.2952	10.7566	6.6698	5.6547	6.3071	9.8887	5.6714	4.3082	4.1737	8.2729	7.7261	6.4974	8.8132	6.7468	0	5.007	12.3625	8.54	10.1454	7.2148	4.2425	9.1452	5.4468	10.2008	6.6822	7.244	9.8534	10.6912	0	8.3581	10.1147	7.0096	8.1751	9.7415	6.3389	4.5263	2.2319	10.1315	7.8563
TCGA-69-7978-01	134	0	2.0094	8.7807	6.189	10.5315	7.8156	11.6502	6.1485	5.6658	7.9993	10.8136	4.1818	2.4252	3.5558	6.9233	7.2793	4.935	10.7464	9.847	0.742	6.2666	11.4607	7.8004	9.3133	10.0275	2.2323	9.8338	4.3096	10.4744	6.4609	7.9874	10.2232	10.2999	0	9.8977	9.9619	7.699	9.0642	8.7785	6.5043	2.332	4.0226	9.4346	7.6755
TCGA-69-7979-01	408	0	0.6598	9.0518	6.44	10.5342	5.9035	11.8821	5.2522	6.1114	6.7431	9.398	3.5157	4.7905	4.2461	8.8849	6.4591	6.44	13.4226	14.9361	0.3673	5.306	11.5441	9.7312	8.7611	8.9421	2.7653	8.7573	2.8834	9.3803	4.9066	8.5358	9.4644	10.5482	0.6598	11.1976	12.4316	8.4729	8.3045	9.9668	5.358	4.3727	8.074	11.2447	5.8823
TCGA-69-7980-01	362	0	4.0277	9.056	5.9311	10.3638	7.4905	11.0174	6.9589	5.0105	6.98	9.1197	8.5538	4.4273	5.1785	7.3701	5.746	4.3428	7.0911	6.7895	0.9401	9.7624	12.1106	8.8946	9.1217	6.5275	3.0616	9.7367	5.5046	10.3725	5.6525	8.2442	9.3868	9.2878	1.3397	7.9477	10.749	8.9501	9.1914	9.6486	6.6247	3.9441	8.2468	7.354	7.0417
TCGA-69-8253-01	426	0	5.1293	9.1681	5.0691	10.1324	6.6744	10.8882	5.7738	6.3996	7.7443	9.6302	3.5677	9.0909	1.0995	10.0755	6.7757	3.5325	15.0936	15.0875	0.3626	5.4826	13.5472	9.0369	8.9159	9.6574	2.974	8.2363	3.5325	9.9028	6.1025	9.0022	8.0498	9.9998	0.6521	8.4682	13.0173	8.5812	9.6071	10.3062	5.3575	6.8055	7.3367	9.5199	9.4521
TCGA-69-8254-01	409	0	6.6068	10.2384	8.7545	10.0274	7.5566	12.2517	7.4306	5.685	4.8848	8.9724	4.8728	12.0954	7.1917	6.6104	6.532	7.4223	8.5641	6.826	2.8871	9.9954	12.4014	8.3059	10.2594	8.8117	5.2539	8.9452	4.3877	10.2559	6.6985	7.3151	10.465	11.1767	0.5774	7.8228	12.3061	8.2947	9.1982	10.3892	6.3306	7.5357	8.4585	6.5088	8.7789
TCGA-69-8255-01	129	0	3.6291	8.6839	5.3846	10.7924	4.4094	9.5308	6.125	6.8456	5.8267	9.7286	6.7107	2.5833	7.2601	8.9179	6.4565	7.6004	11.4419	11.9602	11.3211	5.3059	12.0347	9.1873	10.5113	10.0839	10.8454	7.2444	6.1812	8.7926	8.957	8.6533	8.4737	10.8177	2.4432	11.4303	13.4267	9.1962	7.1319	10.419	5.2333	6.7996	6.5767	12.1622	8.7763
TCGA-69-8453-01	813	0	7.2159	9.5166	7.3295	10.6969	7.1531	12.074	7.4185	6.4552	4.135	10.4913	6.7249	6.4632	4.0944	6.894	8.2737	8.1154	10.0425	7.867	2.1412	7.873	13.1332	8.9798	9.0846	8.6355	3.8724	9.0624	4.7717	10.874	8.3359	7.6645	9.6626	10.4028	0.5727	8.3489	13.3363	8.7607	9.3456	9.3456	7.885	5.4716	4.7979	8.6408	7.451
TCGA-69-A59K-01	522	0	5.0435	8.6642	7.1355	9.6711	7.1355	11.4486	7.7442	6.5239	8.2891	10.16	4.8724	3.354	1.5084	8.2044	7.3895	4.643	14.4852	13.4181	1.157	4.1875	11.4423	8.0389	8.049	6.7714	4.8105	7.5916	9.9286	10.3401	4.8105	6.8884	11.4188	9.8795	0	10.7258	8.4576	8.8678	8.5716	9.9422	5.4202	3.067	3.9243	10.6439	8.2749
TCGA-71-6725-01	256	0	2.6564	7.9872	5.5312	11.7115	6.2339	12.1761	3.1879	5.8371	6.4083	9.7502	2.917	0.3918	0	5.2879	4.8927	5.217	6.9679	6.9462	0	3.7529	12.8886	9.9483	10.0516	7.907	0.6996	7.9783	3.3289	8.1145	3.4159	7.1425	9.635	10.5022	0	10.6591	12.5621	8.7598	6.076	10.5513	4.7326	0.9532	2.7233	11.0087	4.1828
TCGA-71-8520-01	210	1	0.696	9.5895	5.6433	10.0987	6.26	11.635	5.7301	5.2787	5.859	9.9842	2.9672	5.7301	5.4107	8.2922	7.2028	6.2716	4.8836	2.848	0	5.2555	11.5729	8.8775	8.275	10.3455	2.2388	7.8976	4.1985	9.9709	6.1127	6.9155	11.577	10.1445	0	9.4897	9.8597	6.9586	9.2226	10.0165	6.9441	2.0356	9.5444	8.6155	5.2319
TCGA-73-4658-01	1600	1	3.9434	8.5595	6.1768	10.4486	6.949	11.4669	6.5131	5.3056	6.5666	10.1136	6.5666	3.084	1.7231	6.7704	7.1318	2.7557	7.1318	5.6741	0	5.5731	12.1912	7.9125	8.4211	8.77	1.7231	8.5857	3.3513	11.3674	7.4821	8.3643	10.0105	10.1782	0	8.4664	10.8762	8.5792	8.6008	10.7338	7.4868	3.5055	10.634	8.6093	7.5006
TCGA-73-4659-01	711	1	6.8205	8.3492	6.4991	10.373	7.5196	12.0881	8.2296	6.5487	5.2529	10.4707	8.7945	6.2539	3.3915	9.4096	7.9285	5.8198	13.6039	13.9571	0	5.1366	11.8942	8.7026	8.7272	8.1179	3.8582	8.7077	3.8156	10.8549	7.974	8.3068	10.8267	9.9136	0.2762	10.2052	10.9102	8.8484	8.6782	9.858	7.0583	5.5016	9.2898	9.5831	7.1209
TCGA-73-4662-01	2515	0	3.5871	10.0577	7.1075	10.1599	6.5806	10.1729	5.3018	5.6025	7.0423	9.2923	7.1886	2.8737	2.017	6.1611	6.3759	6.322	6.9414	5.5744	0	6.4781	12.4833	8.4427	10.2801	9.3513	1.7417	8.4687	3.4075	10.7106	6.9794	7.2115	9.1636	10.246	0	9.3927	11.7285	8.1964	8.901	9.0565	6.8563	2.6223	4.9968	6.6224	7.1978
TCGA-73-4666-01	800	0	4.2562	7.9096	4.6949	10.6935	9.1849	12.0846	4.6809	6.8667	3.1777	9.8536	2.2549	3.593	0.5878	7.4743	6.003	2.329	13.8527	13.1904	0.5878	4.9273	12.1382	8.6661	8.7983	10.8818	2.9145	7.8145	4.532	8.3519	4.7633	7.2536	10.0291	11.3087	0	10.5573	11.0913	5.79	8.1961	9.9213	6.5974	3.3298	1.7886	8.6408	8.3881
TCGA-73-4668-01	467	0	3.024	7.7267	5.0859	9.7009	6.5277	12.0452	5.2158	5.9429	7.0021	10.3649	4.3354	1.308	0	9.153	7.0376	5.8659	9.3973	6.1115	0	5.896	11.3386	8.4403	9.9739	9.4418	4.2448	8.5352	4.148	10.0215	6.4729	10.091	10.1131	9.8223	0.5772	8.6359	11.206	9.2811	9.6518	9.9371	5.7585	1.6844	9.7124	9.662	7.0828
TCGA-73-4670-01	131	0	2.0024	9.0225	4.4445	10.3484	7.4745	10.5491	5.7629	7.548	4.6934	10.6935	2.9696	2.6307	1.2429	10.9104	6.6112	3.4383	13.4626	15.4941	6.9285	2.9696	10.974	7.8897	11.2173	9.2514	6.7822	8.3475	3.953	10.4276	5.0554	8.9284	10.1672	9.5653	1.7908	10.0086	12.0198	9.2865	7.0346	10.6233	6.3607	7.4087	8.8854	9.3465	7.1831
TCGA-73-4675-01	922	1	2.238	10.3913	5.013	9.4844	4.8208	10.3269	4.9565	6.2879	5.9476	10.155	4.2689	12.6671	4.0967	11.0046	7.7977	5.9035	13.5573	13.3468	0	3.6032	13.7251	9.6241	9.1636	8.287	2.4974	8.7115	6.2649	10.4038	4.7888	7.11	11.5849	9.9506	1.3499	9.2417	10.4022	5.6061	7.5145	9.5067	5.962	8.3771	7.028	6.4501	5.1577
TCGA-73-4676-01	281	1	4.9822	8.6206	3.9796	9.6523	7.3857	12.1356	7.4825	6.296	4.2435	10.4988	4.6292	11.0479	2.8743	6.2366	6.2666	2.5234	13.2539	14.3542	0	5.3237	12.3724	8.304	8.5734	10.2969	6.4256	9.3906	5.0754	10.1063	6.4606	7.3147	7.9723	10.7189	0	9.6617	10.6632	8.3927	8.7624	10.1889	8.4152	8.7179	0	9.6532	5.5997
TCGA-73-4677-01	38	1	6.9358	9.9186	6.5649	10.3829	7.9886	11.9085	6.3414	5.5801	5.3942	8.5323	3.8379	5.9941	3.3979	9.2469	6.7351	5.7906	15.1896	15.3584	1.5964	8.2589	13.5409	8.97	11.282	7.225	4.1276	8.7819	3.5834	10.543	6.986	8.0254	10.6272	10.3036	0.6583	7.5077	11.7762	9.3303	8.9287	10.4218	5.7132	9.5532	6.2167	10.7017	10.283
TCGA-73-7498-01	1189	0	6.1889	9.4593	6.7959	10.2106	7.7071	11.6465	6.5112	5.0032	7.5054	9.5706	5.9617	4.8884	1.398	8.9525	7.2556	5.3308	14.6242	14.6767	0	5.5141	13.0866	9.1488	11.1552	7.8297	4.2262	8.6923	4.5621	11.7761	7.0894	8.1472	10.6853	9.8554	0	8.197	11.0171	9.0981	8.8942	10.1188	6.848	8.3434	2.3717	10.3966	8.8759
TCGA-73-7499-01	1531	1	6.0325	9.2004	4.6842	10.5915	8.5598	11.5652	7.4749	7.9274	7.6238	8.2653	10.4885	6.1242	3.2073	8.1691	6.1168	7.2182	8.8271	6.8029	3.9377	4.3461	12.6958	8.568	9.0709	10.3528	4.4203	8.2962	3.3151	7.8422	5.7453	8.9611	9.9819	10.2218	1.2824	11.3075	12.8199	10.0811	10.7814	9.7106	7.6626	5.4585	9.1052	10.8369	8.0039
TCGA-73-A9RS-01	340	1	4.2177	8.2387	6.2777	11.0569	9.2384	12.5571	5.3122	6.5727	5.3122	8.5685	3.1574	7.1748	1.8643	6.9049	7.0268	7.8936	9.3477	11.464	0	5.1784	12.1292	8.4127	9.33	6.4904	8.2801	7.8055	4.2177	8.1476	7.6371	8.122	9.9274	9.6758	0	11.2689	10.8996	9.4292	8.5342	10.1923	5.5477	6.7834	0	11.5851	5.213
TCGA-75-5122-01	0	0	0	8.1479	4.7217	11.3497	7.2996	12.0221	6.6801	5.6703	5.1914	9.943	5.9914	2.7915	0	5.7405	7.5416	1.8232	8.1565	6.4199	0	5.4366	11.6906	7.6968	8.211	7.0554	0	9.9454	2.6034	7.4257	3.6833	8.4511	10.3272	9.1367	1.4289	10.1601	9.7251	7.984	9.0769	8.9787	7.8208	4.7672	3.6192	8.5925	10.0167
TCGA-75-5125-01	2027	1	4.0412	9.2	5.3282	10.4998	7.3429	13.5168	4.8237	5.6129	4.9733	10.2003	2.7377	3.7956	0.5998	8.1392	6.7917	4.5014	5.9259	3.8482	0	3.8482	11.0639	7.3244	9.3235	9.5337	3.9479	8.746	5.3096	9.1458	4.5339	7.5884	10.5443	10.2201	0	10.5423	10.6062	8.817	8.6382	9.2312	6.3743	2.7377	4.9747	9.1193	7.4615

TCGA-75-5126-01	0	0	6.0679	8.8139	5.5601	10.7198	7.7914	11.5331	8.2757	5.3787	5.106	10.4878	3.6941	5.5601	3.9384	6.2143	7.3664	4.7993	7.8573	6.183	0	6.4026	12.6896	8.5997	9.1854	10.5895	3.5047	9.1991	4.1473	10.4495	7.4607	7.7067	9.9441	10.5153	0	9.3458	10.787	7.6268	9.0768	9.2622	7.9108	5.4329	10.3511	8.0646	7.7705
TCGA-75-5146-01	2368	0	4.4298	10.1901	6.1674	10.2891	6.563	11.5536	7.4714	5.4437	4.8038	9.7261	4.0524	4.8038	3.7058	6.7264	5.1873	6.2636	8.2794	6.647	0	8.4616	13.4907	8.8683	9.4242	9.1279	5.4437	8.4054	3.6252	10.5897	8.6541	8.0822	10.3013	9.7868	0	9.9825	11.9967	8.1947	8.8661	10.2915	6.7167	6.2769	1.8398	8.0671	7.9685
TCGA-75-5147-01	1333	0	2.9603	9.312	5.8559	10.2577	6.9401	11.6992	7.7269	5.9689	6.8943	9.1982	6.7905	10.1042	5.8632	7.3268	7.3814	6.3593	8.4712	7.7249	3.9217	8.1352	12.002	8.3116	8.1989	9.9681	4.8141	9.4971	4.2471	10.005	7.7026	7.9878	9.6484	11.3591	1.1241	9.8536	12.6136	9.4673	9.8587	10.7688	8.3447	6.9156	9.7012	10.5707	7.7528
TCGA-75-6203-01	0	0	8.6185	9.1543	6.5939	11.0587	7.2638	11.8384	8.4271	5.088	6.0215	10.2524	5.578	7.2179	0.7551	7.8881	8.4808	4.7621	9.2404	7.4625	0	6.731	13.4104	8.8872	9.2888	8.864	4.072	8.6608	4.3885	12.2652	8.2494	8.2584	11.3162	9.8059	0	8.4328	11.1121	7.4904	9.7144	8.9667	7.8755	5.7165	6.6234	6.9474	6.6738
TCGA-75-6205-01	0	0	1.8247	8.1327	5.4666	10.7075	5.1597	11.45	5.5473	5.6724	6.2559	9.9342	4.5778	2.6052	0	5.5209	7.6511	1.4302	10.555	9.8436	0	4.7693	12.1463	8.5756	8.2296	8.9012	4.0227	9.7894	4.8135	10.1135	4.7238	7.4204	9.955	10.0317	0.8856	9.7558	10.6455	4.357	8.7561	9.2494	6.7519	3.7774	9.3138	7.189	7.5952
TCGA-75-6206-01	2590	0	7.9815	9.3355	6.9213	10.3862	8.4907	11.1567	6.3608	4.8738	6.4774	9.2443	3.4685	9.8133	2.0519	7.7095	7.3239	3.1987	14.6665	14.326	0	5.4106	13.0854	9.4645	10.7632	7.0085	2.7364	8.5621	2.4344	10.062	7.459	8.9575	10.5122	9.6488	0.7043	7.1997	11.5539	8.9786	9.8511	10.6737	6.5664	7.9303	4.1607	10.3984	9.0777
TCGA-75-6207-01	0	0	2.0275	7.7807	5.7243	9.5584	8.963	11.6209	5.4904	6.4955	4.7514	11.3586	2.5448	1.2133	0.5257	6.9551	5.7363	5.0861	10.2567	9.1375	0	2.9247	11.7342	6.6613	10.5532	9.5465	2.1752	6.7873	5.5593	8.8173	5.141	8.2198	9.8318	9.8443	0	11.3121	10.1974	8.1501	9.1981	9.9423	5.1589	0	2.0275	9.716	3.3548
TCGA-75-6211-01	0	0	1.5716	7.293	6.0606	9.583	5.9878	10.0009	5.2659	7.5524	6.4451	9.6032	8.111	9.9443	3.754	8.4188	6.247	7.0135	15.1322	12.7089	7.14	2.9212	12.6598	8.9156	9.1153	9.3673	1.2109	8.6275	3.9509	9.6268	8.1416	7.8422	11.0874	8.4625	0	10.7279	8.4326	7.1533	8.0728	10.4184	5.8952	5.5951	6.3264	10.0993	7.5524
TCGA-75-6212-01	1516	1	8.2926	9.9515	6.1528	10.4894	7.4625	12.6388	7.0775	5.1414	7.0775	9.0535	4.9726	4.4624	0.8314	8.908	7.0006	4.1817	9.3842	9.1168	0.8314	3.0027	14.4352	9.6053	10.9755	10.5034	3.4767	8.9721	3.1366	9.8195	5.5055	8.8687	10.6036	10.5752	0	8.0168	12.2901	9.0985	10.177	9.3992	5.755	4.4624	11.5574	7.9816	6.6437
TCGA-75-6214-01	1115	1	0	8.0543	7.8996	10.0028	6.9717	10.1638	4.7772	5.2031	5.1669	10.2743	4.1705	9.5359	6.1894	7.8339	9.313	4.0434	8.8163	10.7654	7.4082	8.9718	10.0382	9.0283	9.802	5.5596	5.413	9.664	3.2485	7.1975	6.7897	7.2083	7.5458	10.3435	0	7.5458	13.3306	9.4018	7.8107	9.7362	5.0395	5.2727	8.8226	10.8835	6.2654
TCGA-75-7025-01	3305	0	6.118	8.9401	5.8886	10.3191	5.9951	10.9616	7.1116	6.6452	5.925	9.2758	7.0552	7.8083	6.0121	8.0723	7.7231	8.7158	8.7928	6.6452	0.4628	7.5906	13.9479	9.0419	9.4873	9.2245	4.1409	9.0084	4.7192	11.359	9.14	8.9976	11.152	9.346	1.5316	8.6052	10.8531	8.5201	10.2215	9.6124	6.6722	5.5705	3.486	7.6159	7.4035
TCGA-75-7027-01	3059	0	3.3596	8.692	4.8184	9.8349	2.7006	9.7113	6.8608	6.7681	5.3862	8.7798	4.2219	2.2521	3.2321	9.2365	7.6843	4.5055	14.331	14.0837	8.0782	3.1403	12.8633	8.2838	8.493	8.1651	4.4105	8.873	3.5499	11.0948	5.136	8.904	9.9953	9.1435	1.5977	10.2287	8.8695	9.1822	9.125	9.9706	6.3226	8.6437	5.2192	9.1619	6.2143
TCGA-75-7030-01	0	0	7.0126	9.2437	7.8788	10.1843	5.415	10.656	7.3346	5.6724	6.1874	9.3232	5.8127	9.2362	6.1455	6.9892	8.1408	8.2535	10.3729	9.256	3.639	8.1031	12.6998	7.866	8.8103	8.4892	1.6245	8.8103	4.9149	11.5091	8.1194	7.7522	10.509	10.0854	0	8.0417	11.2895	8.2186	9.5386	9.095	6.8265	6.6129	9.0219	6.8913	7.5015
TCGA-75-7031-01	0	0	5.0556	8.6358	5.7173	11.0295	7.1263	11.6702	7.435	5.1892	6.9111	9.0846	6.2325	10.9842	3.4165	10.5465	8.1596	8.1704	16.0312	14.0295	9.4265	11.8211	12.6075	7.7977	8.6552	9.2183	2.4063	8.7591	3.2634	8.9656	6.3128	8.837	11.3691	9.3381	1.6556	10.262	14.0876	8.7519	8.9054	10.1517	6.3322	6.5129	2.3797	10.0544	7.8116
TCGA-78-7143-01	4961	1	4.8007	8.8111	6.1313	10.4355	7.2357	11.5145	5.1711	6.842	9.9122	10.1572	2.411	8.1083	4.7242	7.5703	8.3961	7.7643	5.4177	3.5893	0.5654	4.9198	11.4757	8.2193	9.4643	9.3212	2.6504	9.3649	2.9482	9.4134	5.7872	7.4863	9.5049	10.3335	0.9706	10.3346	11.155	8.8745	9.6939	9.9194	7.1358	4.1139	9.8484	9.5673	6.1703
TCGA-78-7145-01	826	1	3.5204	7.9628	5.5175	9.5584	8.2181	12.0006	5.1897	4.2944	5.6235	9.3046	4.5665	2.6409	1.4572	8.4967	6.6344	6.5109	6.7481	3.4453	0	5.4031	11.8152	8.5266	9.3771	10.6008	1.7345	7.6975	4.5665	9.7024	4.0117	8.9124	8.3319	9.7751	0.6617	9.3205	12.348	9.4158	8.6818	10.1435	6.4353	2.343	5.2321	8.7958	3.2823
TCGA-78-7146-01	173	1	1.8022	8.6185	4.1631	9.7593	8.7921	12.3643	5.3853	4.8259	3.6372	10.1343	1.3176	1.3176	0.9964	8.0235	8.0235	2.8012	11.6692	9.9801	0.5826	3.517	10.3	7.9324	8.6652	9.4481	1.5801	9.5164	5.3328	8.3808	3.9475	7.0311	9.6139	9.0358	0	11.147	11.1565	7.3327	8.2943	8.8194	5.3505	2.6943	2.1643	10.2794	4.7744
TCGA-78-7147-01	586	1	6.2796	9.1965	4.3891	10.9099	7.376	10.7312	7.8954	6.9796	4.9781	9.2104	2.407	8.4235	3.671	7.8643	4.7454	7.4029	7.4359	5.065	0.4763	6.5199	12.3896	9.428	10.3329	6.3154	2.6055	8.3143	3.3771	8.9665	4.5664	8.8054	11.2481	9.3779	0	10.3624	10.8637	8.4656	9.1781	9.7687	5.6183	4.1237	8.3743	9.9537	7.5152
TCGA-78-7148-01	626	1	6.2066	9.3461	5.1307	11.1404	6.876	12.0724	6.4542	3.8374	5.3345	9.3587	3.6049	9.8775	6.4629	10.5802	6.132	5.4791	14.4666	13.4951	4.4065	5.3905	13.0264	8.4852	9.3994	8.0658	6.8563	8.668	3.7258	9.6289	7.6468	7.9695	11.0496	9.7691	0.6152	8.6623	10.8462	8.5274	8.4766	9.4419	6.7101	8.034	6.3361	7.502	7.9894
TCGA-78-7149-01	3940	0	6.7546	10.0748	5.2667	10.3591	7.6835	10.3198	5.4598	6.4341	6.1278	8.9319	5.3665	9.5525	4.9438	10.2266	6.8542	7.1681	15.4466	15.1958	1.0723	5.7532	13.8506	10.1828	11.1958	8.4717	3.3749	7.9986	4.1328	10.4317	5.7532	7.7429	11.0416	9.4276	0.6336	8.3792	11.3167	9.0672	9.4218	10.2025	5.1815	6.3874	5.8148	9.0082	9.7109
TCGA-78-7150-01	666	1	8.0587	8.2584	5.6536	10.0709	6.6558	10.02	6.1955	6.0281	6.2068	9.519	2.9791	9.7499	8.3876	10.3776	8.6635	8.3219	14.0328	15.5686	3.2639	4.3978	12.4736	8.1548	7.9458	8.4194	8.1921	8.4552	5.569	10.4193	8.5466	8.6594	9.2493	10.1006	1.1026	10.7174	11.7978	9.5234	8.1519	10.2495	5.9499	7.7482	8.399	10.0724	8.2421
TCGA-78-7152-01	1215	1	1.9205	9.5041	5.9171	10.0605	6.2163	9.5513	4.4214	4.0691	6.0239	9.3434	3.8073	4.7043	0	8.277	6.5945	6.2342	11.4418	11.3056	0	4.9837	13.0354	9.134	10.2251	7.9213	4.2906	9.2725	4.0691	9.5744	5.3218	7.0121	9.0121	9.9279	0	9.6278	13.3135	9.648	9.2769	9.8444	6.1233	3.0752	9.7653	9.0302	7.9046
TCGA-78-7153-01	3635	0	2.2279	9.108	5.665	9.6569	6.9158	9.9747	8.751	6.7528	4.6409	7.7493	3.5159	10.3528	7.8496	7.8913	7.1396	5.7498	14.5268	13.3508	7.3333	4.2796	12.5983	9.4406	10.3172	7.8913	0.6907	7.9024	2.836	8.9841	5.5373	9.5963	9.6991	10.8537	0	9.3688	11.9695	10.5233	9.6689	10.342	4.8396	10.1219	5.9048	9.8792	6.3477
TCGA-78-7154-01	593	1	4.5621	7.778	7.7897	9.923	5.9477	10.4756	5.5125	6.3464	5.4549	9.322	7.647	8.573	3.5504	7.114	5.8617	6.4966	15.8534	14.8372	8.8455	13.3961	11.1581	9.14	8.3725	9.2156	2.5267	6.8933	6.1548	10.4947	9.0111	8.7361	9.2142	11.5483	0	14.2483	9.6056	9.3039	7.6079	9.6034	7.6035	5.4152	4.8331	9.6735	5.2891
TCGA-78-7155-01	1171	1	6.0018	8.6512	7.3845	9.8087	7.5628	9.1574	7.4141	7.4141	4.2868	9.8923	0.6587	0	2.6342	5.2272	4.4862	5.7144	3.1862	3.839	0	1.4521	9.8715	7.5716	10.1634	8.2709	5.8363	7.0919	5.4726	6.6681	3.0914	9.8833	6.7245	7.7412	0	9.2887	4.4485	8.732	7.9979	9.2927	4.7259	5.8363	1.155	12.454	5.1587
TCGA-78-7156-01	976	1	8.0138	9.9383	5.7677	9.5407	8.9434	11.9851	4.8259	5.3505	6.1803	8.6847	4.4689	6.9844	1.1659	8.9306	5.2598	6.7954	15.2952	15.0796	1.5189	4.8572	14.0066	9.0128	11.2706	5.8633	4.2026	7.4927	4.3419	11.0476	5.1878	7.269	9.8852	9.4396	0.6977	8.6107	9.2029	9.6238	10.436	10.9714	4.8879	7.6661	1.1659	9.905	9.6272
TCGA-78-7158-01	179	1	5.8122	9.7845	4.848	9.7585	5.9922	9.921	8.9711	6.3039	6.2499	8.4578	2.58	6.3992	5.7579	5.6064	7.8222	2.6768	6.6343	5.337	0	3.5076	12.2556	8.6608	10.8888	9.3313	4.848	8.4164	3.6092	8.5787	4.1373	7.1101	9.4978	9.4635	0	9.1461	12.5969	8.9959	10.3203	10.5158	5.569	3.6574	4.8334	8.2136	4.523
TCGA-78-7159-01	1974	0	3.1233	8.0643	6.768	10.2294	6.7236	10.5247	6.7534	7.0932	7.7054	9.1052	7.304	1.9087	0	8.6788	7.6431	7.3823	7.6391	6.2255	0	8.9211	11.8861	8.3851	9.4171	8.397	1.6798	8.4861	3.7138	9.9188	5.0664	9.8587	8.8209	8.7095	1.6798	9.9488	9.2272	7.5571	8.9079	9.704	6.5897	4.038	8.1287	8.789	8.0966
TCGA-78-7160-01	697	1	6.3754	8.8084	6.6537	10.2615	6.1758	11.3842	7.153	5.0793	6.7414	9.3077	4.8773	7.2278	3.4718	8.4791	7.3831	5.9588	15.8315	14.5353	5.8682	6.5113	12.9174	8.9159	10.3666	7.3994	4.4476	9.1178	4.1213	11.1906	7.2815	7.8817	8.9347	9.3807	0	8.5044	11.2142	8.8285	8.6153	10.4398	6.6171	7.1784	7.4885	5.8837	9.0644
TCGA-78-7161-01	291	1	4.6582	7.6686	5.0747	10.2757	5.8568	11.6684	8.2079	6.1233	7.6763	9.5846	4.5717	2.1066	0.4514	9.549	7.3061	6.9163	11.7601	12.616	2.2248	6.5197	12.2867	8.3033	9.5525	8.9604	2.4354	7.2654	4.4066	10.478	9.069	8.9594	11.3005	9.6066	1.072	8.9248	10.2472	8.6506	9.7593	8.9992	5.3067	2.703	11.8715	7.7197	7.2305
TCGA-78-7162-01	3169	1	7.9583	9.4434	6.255	10.2468	7.5087	12.0684	8.2753	4.8113	6.1623	9.5801	6.0068	10.4593	2.9234	8.4102	7.0119	6.9234	15.6241	15.6618	0.4499	7.8157	13.0551	9.3257	10.7577	8.4024	4.4975	7.9647	3.3942	12.0504	6.9996	7.4377	10.4916	10.103	0.4499	7.891	11.013	7.9261	9.4088	9.6605	5.9735	7.9131	2.8292	9.1286	7.7238
TCGA-78-7163-01	7248	0	7.1676	9.1755	6.6081	11.4781	7.7296	12.3475	8.2479	5.3716	2.0291	6.7974	0.4248	9.5344	0.7526	8.8502	5.234	4.7921	11.7825	13.4069	1.2446	11.9608	13.3102	9.0729	8.8714	10.0082	1.0194	10.7292	1.9027	6.7154	3.7361	8.5824	9.5184	10.2177	0.4248	9.4005	11.5997	9.1925	10.6607	9.0053	4.1238	5.9449	7.2303	7.3452	3.8433
TCGA-78-7166-01	258	1	3.6024	8.8861	3.1739	10.3043	7.2909	11.2061	3.7046	6.3443	5.3775	7.9606	3.2435	8.6081	7.3433	10.5541	6.4584	4.6223	15.3815	16.9408	4.7231	4.771	13.0065	8.6535	9.5239	8.7538	5.8373	8.4644	4.8174	8.6663	4.0917	7.3785	10.9121	9.7911	3.0238	8.4312	11.4357	8.5372	8.9757	8.8108	6.9127	6.902	7.6225	5.9352	8.0972
TCGA-78-7167-01	2681	1	5.2422	9.3411	5.1726	10.2371	7.8648	10.6536	5.1607	6.3493	5.9866	8.4987	4.4266	7.0417	2.7326	10.3923	5.6448	6.4557	14.5441	15.539	0	4.4659	13.2847	9.1804	10.6018	8.2272	3.9872	7.7177	2.97	10.1287	6.0134	8.7549	11.0249	8.8909	0.3754	7.9522	10.0468	9.5591	9.0171	10.1098	5.2868	9.9973	2.5924	9.2147	7.6848
TCGA-78-7220-01	807	1	6.9664	10.0677	5.2696	9.6352	6.565	10.1451	6.7489	7.2943	5.2107	9.1037	7.7317	4.4995	4.3618	8.7107	7.0246	6.4926	14.1333	15.5103	5.2696	4.8733	12.3368	8.3949	9.8991	7.4468	8.468	8.0474	4.7683	9.1359	9.2105	9.5654	9.0586	9.2092	1.0211	8.6602	12.6961	10.3525	8.189	8.9381	6.2212	7.4553	4.2299	7.9396	6.1911
TCGA-78-7535-01	949	1	1.047	8.8963	3.629	11.1431	5.9571	10.729	2.6624	7.147	4.3356	10.8333	1.9423	3.7859	0.4387	9.0001	5.9571	5.1486	7.2381	5.9153	0	3.5437	12.9412	9.7753	9.0493	10.1499	5.0437	8.7563	3.3052	8.6339	8.2128	8.136	10.3793	9.922	0	8.6326	13.5373	8.6157	9.4082	9.8048	7.0992	2.8159	10.3184	7.9228	7.1792
TCGA-78-7536-01	244	1	7.4301	8.6063	6.3687	10.3234	8.3362	9.75	3.388	6.4721	8.1795	9.8771	6.7949	9.101	0.5963	9.1635	5.0661	8.0983	6.4596	4.0761	0	11.4705	12.3268	7.0805	9.8466	9.3242	2.4196	9.2963	3.0295	8.5522	7.495	7.4305	8.8133	8.3109	0	9.2042	5.3904	8.4086	8.7658	10.2741	5.7054	6.3642	6.5686	6.1779	8.5463
TCGA-78-7537-01	1622	1	0.7768	9.8196	5.0641	10.6692	7.7741	10.7434	4.7181	4.7376	6.2391	8.6886	2.8202	5.4875	0	8.4909	7.0314	2.5833	8.6441	7.047	0	2.4947	14.338	9.0369	10.0894	7.093	4.6583	7.8317	3.202	7.5968	6.6095	7.7173	10.7182	10.859	0	9.641	13.1006	9.8656	9.9739	9.9851	5.5979	6.7831	0	10.2037	7.1375
TCGA-78-7539-01	791	0	4.5293	9.2833	5.3282	10.736	6.4911	10.5662	7.7746	4.7187	4.9967	8.5706	3.5365	3.0124	0.6682	6.1756	5.1954	5.5256	9.4353	7.9498	0	2.8451	12.3899	7.5639	10.5948	8.3259	0.9136	9.0297	2.7847	9.8147	5.049	7.685	10.9642	9.5372	0	8.7418	10.8998	8.0669	10.138	9.8657	6.1155	2.181	0.6682	6.5648	6.6349
TCGA-78-7540-01	1197	1	6.2955	9.2356	9.6313	10.6276	8.0029	9.9612	5.5464	6.3551	3.3144	8.6452	2.109	8.2709	6.4728	6.9105	6.4942	7.0613	9.1243	7.8517	5.2013	4.6738	12.7248	8.6198	8.835	8.0177	5.4943	8.5653	3.6552	8.9765	4.135	7.075	10.3746	11.4224	0.4129	8.188	11.3565	9.449	9.1831	10.2043	5.9382	5.7377	10.5686	6.2955	4.6358
TCGA-78-7542-01	321	1	4.4354	7.0237	6.4823	10.3942	5.153	10.3628	5.1187	6.9956	6.967	9.2929	0.6395	2.835	1.2597	8.4369	9.2188	5.1071	6.8393	7.4651	0	7.2859	9.4817	8.1961	5.988	9.5552	0.6395	9.1631	4.4539	6.4179	11.022	7.4219	9.5822	9.9701	0	9.2269	8.8386	8.3932	8.9164	9.3148	5.4759	4.0054	11.4345	10.3219	6.3054
TCGA-78-7633-01	1528	1	4.7807	9.6458	6.7094	10.0011	8.8792	10.5027	5.7771	6.0953	7.9594	9.3932	5.4274	6.7791	2.059	8.7816	6.2231	6.468	17.3005	15.192	0.365	5.6993	12.3175	9.1303	10.4999	7.7611	2.059	7.7025	4.3427	9.9942	4.1219	8.4744	10.8542	10.6823	0.365	8.7277	10.2943	9.5565	9.4655	10.5937	4.8106	8.5362	0.6561	8.3336	5.9816
TCGA-78-8640-01	7062	0	5.5929	8.387	6.6127	10.5081	6.6298	12.9419	8.3514	5.9053	4.7374	9.1176	2.6442	6.4392	1.1157	8.5531	6.7905	4.6053	13.966	13.1209	2.1703	11.0212	13.2955	8.8823	7.3609	11.0834	4.2981	7.7414	8.8859	9.9178	6.9213	6.9357	10.3227	10.2415	1.4597	11.7495	10.4339	7.0976	8.9972	11.1586	6.1968	6.4489	5.8243	11.6888	7.0602
TCGA-78-8648-01	1209	1	7.032	9.1318	5.939	11.1634	6.216	11.9183	8.407	6.2308	7.1138	10.793	5.939	4.215	0.8188	6.0092	8.8697	4.3828	8.2921	6.6484	0	7.6521	12.337	9.288	8.4327	7.9704	4.0911	9.4113	5.0481	12.3146	9.0131	7.6873	11.2013	9.7021	0.8188	9.1552	10.7025	7.9966	9.4684	8.9274	7.9438	4.2731	7.3621	9.1219	8.8907
TCGA-78-8655-01	2360	0	3.4768	9.3446	5.3086	10.7071	6.3698	11.0986	4.7865	4.0732	4.4939	9.1609	3.9603	2.263	0	6.7637	6.5067	3.1043	6.9818	3.9603	0	4.5728	12.3553	7.9797	10.1576	7.3101	3.5566	8.9233	3.9004	9.7897	4.8193	6.8779	9.8952	10.2781	0	8.1694	11.4471	8.1242	9.1134	9.5872	6.65	6.9152	4.1483	6.9962	5.8099
TCGA-78-8660-01	321	1	5.6771	9.0984	5.7571	10.9929	8.4955	12.3277	6.9686	6.2854	4.6048	10.7467	3.3692	4.2094	2.1699	5.7879	6.8998	4.4206	15.3305	15.4882	1.1154	6.4387	12.6956	8.5094	8.6647	8.1931	1.1154	9.1363	4.2542	10.2236	5.7414	7.3597	10.2431	11.0352	0	10.0165	9.9051	8.5186	8.7629	9.6197	5.8621	4.0149	5.9447	10.7696	8.1102
TCGA-78-8662-01	3361	1	3.0496	9.7089	6.8434	9.8979	5.6239	11.1436	8.1711	6.4102	4.0775	9.376	2.1074	1.5779	1.2164	8.6005	5.225	5.173	9.7538	11.4931	0	3.6148	11.3071	6.6761	10.1698	5.6239	1.5779	5.6993	4.1864	8.9696	2.7983	6.815	9.6267	9.7337	2.1096	9.4262	10.3319	9.8634	8.9923	10.6509	7.8823	4.3358	3.5347	11.5917	7.7511
TCGA-80-5607-01	0	0	5.685	9.754	6.2909	10.2487	7.4984	11.2866	6.182	6.6477	6.4737	9.3977	3.1917	6.2297	3.6313	7.3408	6.6477	3.8093	7.2434	6.2758	0	4.7997	11.9169	8.3436	10.3303	9.8914	3.723	8.7719	4.5216	9.7783	5.9357	8.6513	9.9012	10.0691	0	8.6805	12.3255	6.6938	8.9534	9.5022	5.4635	7.7317	3.6641	5.7077	9.0136
TCGA-80-5608-01	2832	0	5.9123	9.2151	6.0653	10.8894	6.9794	11.6524	6.4634	5.6925	5.0094	9.1571	6.5962	3.4502	1.0454	10.228	8.4319	7.3368	10.9414	10.0279	4.6707	5.8337	12.8468	8.4613	8.104	8.521	2.1847	8.9378	3.1948	9.1811	7.7519	8.4195	11.409	10.4567	0.4379	9.5791	11.0265	8.5376	8.9575	10.0488	6.6379	6.5369	10.3301	7.1295	6.4692
TCGA-80-5611-01	2595	0	7.1214	8.6701	5.5963	10.9552	7.4226	12.0827	7.9043	7.6756	4.3969	10.2201	4.3185	10.2975	1.418	7.5002	6.7175	6.7402	12.5682	13.6723	0.6391	6.0732	13.3876	8.6301	8.8793	11.0112	3.9007	8.9428	2.9428	5.6454	8.9558	8.418	9.888	10.6471	0	10.1825	11.4118	8.122	10.2508	9.924	8.2778	3.3101	7.5487	11.0651	6.8765
TCGA-83-5908-01	824	0	1.5249	7.3205	5.1052	10.5914	7.0815	10.2907	5.3507	7.0034	8.6254	10.2435	2.3592	4.5097	2.1308	4.7501	7.7956	5.4028	6.6512	5.8876	0.46	4.7501	11.2635	7.5604	7.9699	9.0825	2.7296	9.0118	4.3594	8.9028	5.1209	7.3939	9.244	10.0099	0.46	10.4354	9.6	8.4978	8.6268	9.3549	7.4807	3.1515	8.4558	9.4693	8.3058
TCGA-86-6562-01	376	1	5.3071	8.4793	7.171	10.7805	6.7788	12.4664	6.5586	4.5878	5.9314	9.9798	4.1388	4.0482	1.3829	8.6717	6.7998	6.0529	6.0294	4.8245	0	6.0412	11.4949	8.8732	9.2216	10.4288	2.542	9.7717	2.669	10.8294	7.3523	8.3052	10.65	10.9263	0	8.9058	10.8058	7.6582	8.9658	10.6447	6.9196	4.4531	6.3642	7.5133	5.8126
TCGA-86-6851-01	179	0	2.2335	9.7425	6.429	10.8409	6.8338	11.5735	5.302	5.5236	4.2436	10.1349	8.8566	5.8852	2.0472	7.6217	7.3392	3.9566	6.3854	4.5192	0	6.0431	12.3856	8.1226	9.798	9.0924	4.5012	10.3431	3.7276	9.6752	6.3455	8.2557	10.4454	9.1807	1.4377	10.4843	12.0289	8.336	8.3649	8.882	6.0981	3.5979	6.0597	9.8935	9.475
TCGA-86-7701-01	947	0	3.8776	7.6972	6.1405	10.2837	8.1142	11.2529	7.7687	6.6597	5.5975	9.4836	8.9732	4.98	3.9775	7.1302	6.6747	8.0865	9.0555	8.2452	5.1608	5.7465	11.7189	6.7186	7.6676	8.5016	1.6358	8.6414	3.2372	10.128	12.2379	8.9054	10.5902	9.1917	0	10.6763	9.865	6.8822	8.3972	9.7283	6.2643	3.8776	10.9808	9.0002	8.8134
TCGA-86-7711-01	1046	1	4.3728	7.5959	6.4612	10.2564	7.3396	11.3813	8.1413	5.6816	5.251	12.1628	1.9793	8.5452	0.3722	8.6921	6.559	0.913	14.8308	15.1156	1.3054	11.1874	13.055	8.8244	8.9222	9.1342	4.7175	8.9593	3.974	9.5815	8.1725	7.6502	9.6602	8.5084	0.3722	10.0774	8.3417	9.2079	8.1488	10.5335	7.4237	10.9308	3.4892	9.8294	7.5536
TCGA-86-7713-01	1157	0	3.6762	8.6738	6.9557	9.5643	5.2123	9.4871	6.9388	5.4766	4.9681	7.7762	2.8098	5.7264	0.9723	10.4289	5.7588	6.9074	8.3483	6.1247	0	7.2931	10.5608	9.1134	9.973	6.337	2.0448	8.3923	3.7809	8.0799	4.9116	8.217	11.3603	9.704	0.5666	7.9652	9.9041	7.3474	7.5516	9.6724	4.9231	10.7301	1.9074	10.6284	8.2605
TCGA-86-7714-01	625	1	6.5696	9.5742	6.7693	10.2336	7.8297	12.4475	5.5271	5.083	6.988	8.8613	4.6145	8.1006	4.7211	6.9556	6.1971	5.7195	9.014	5.3389	0	8.5328	13.1014	8.6352	10.8516	9.9148	5.7195	9.368	2.922	10.369	6.4034	7.4706	9.9274	9.8764	0	7.8297	11.7379	8.6146	10.2348	9.6887	5.0424	8.8025	6.4752	7.3786	4.237
TCGA-86-7953-01	997	0	4.7067	8.6412	6.0699	10.8465	7.5133	11.9701	8.7452	5.2226	5.4735	9.8738	4.4772	8.1796	0.9156	6.6562	6.702	5.5753	9.6908	5.7518	2.8489	11.923	11.9452	8.469	7.3583	7.2973	2.9626	9.5254	3.952	8.7462	3.6464	8.8977	9.8568	9.1511	0	8.7069	12.727	9.8696	9.4359	9.5097	7.8323	5.7198	3.3945	10.1306	7.0463
TCGA-86-7954-01	605	0	4.2628	8.6734	5.3718	11.1341	7.0632	12.4566	4.8225	5.5798	6.3496	9.8104	10.1315	6.0508	2.7267	5.0609	7.2922	5.4796	9.1001	8.3048	3.2575	7.2345	12.9913	8.7694	9.0767	7.9495	1.7691	9.6886	2.4723	8.9844	7.3736	8.707	10.003	9.3694	0.3421	9.2006	10.4007	8.8875	8.6931	9.872	6.7374	4.3971	6.4704	8.6175	9.3441
TCGA-86-7955-01	1072	0	5.1184	8.5005	4.6837	10.6075	7.3731	9.7714	3.4113	5.2612	3.6023	10.7298	1.1407	1.1407	0	8.8922	5.3168	5.9151	9.6313	9.8925	0	2.683	13.2276	9.533	8.9696	4.9317	0.6803	7.2153	6.2763	7.9837	9.6474	8.3438	9.801	10.5405	1.4892	9.9529	10.3899	8.784	10.5056	9.8998	9.3439	0	1.5661	11.7718	5.5008
TCGA-86-8054-01	1148	0	5.4472	8.145	6.6473	10.689	6.9683	10.9156	9.0701	7.3385	7.8935	11.4984	8.0522	1.999	0	6.2902	6.543	5.9912	6.7907	5.2211	0	7.2933	10.4982	8.0211	7.8337	9.2979	4.5635	8.2195	2.9988	9.1501	6.6134	10.9695	9.1802	8.805	0	10.6799	11.459	9.0512	8.9673	9.4347	5.3563	8.7198	4.6385	9.976	4.368
TCGA-86-8055-01	124	1	3.7006	7.9385	5.9002	10.2027	6.4806	11.4336	7.0785	5.7006	6.1117	9.644	4.6441	2.2803	0.8932	7.8039	7.8741	4.8511	8.9318	6.0098	0	7.5798	11.3811	7.6554	8.6065	10.0669	2.147	9.0911	3.7928	11.076	8.1273	7.5632	10.2282	9.4906	0.5146	9.7503	10.6545	7.4549	8.6716	9.2159	6.9742	5.3527	9.1388	9.325	6.8457
TCGA-86-8056-01	139	0	6.4242	8.3129	6.7463	9.5259	7.4361	11.8288	7.5658	4.3987	5.727	9.4314	4.5343	11.0059	5.727	5.1273	7.0055	6.1773	8.6641	8.4406	0.4291	4.9446	11.8802	8.1251	9.3379	7.1742	3.636	9.8678	2.5485	10.3247	7.4788	7.0319	9.1474	9.4734	0	8.8597	11.6183	8.6885	8.8682	9.6127	6.5035	4.0522	2.7529	5.4918	6.0167
TCGA-86-8073-01	740	0	5.365	7.7117	7.8514	9.8654	5.9821	12.7565	6.2001	5.816	7.8684	10.2141	5.2262	9.0739	1.768	7.1278	6.9059	6.8208	15.0735	14.7876	1.9067	6.4417	11.839	8.639	8.9915	8.6819	3.6652	8.9196	4.8671	11.1321	7.0699	7.4987	9.1218	9.4898	2.1496	10.2601	11.2637	8.4016	8.3341	9.832	7.9429	4.6863	4.5503	10.1931	7.596
TCGA-86-8074-01	24	0	1.8081	8.484	4.6199	10.5191	7.1767	11.253	6.8185	6.4569	7.3853	9.0475	4.6815	3.5065	0.4408	8.7248	7.5263	5.1125	5.4557	4.1536	0	6.9701	12.0776	7.5972	9.3966	10.4422	2.1935	10.0089	3.3125	9.4395	5.2678	9.2119	9.4432	10.8607	0.778	8.5638	12.3144	9.4798	9.5204	9.7939	6.9156	2.7481	9.6192	9.8136	7.9393
TCGA-86-8075-01	694	1	4.7393	7.7802	6.2396	10.6394	6.5826	11.0254	6.8345	5.7042	7.8989	9.7013	5.8894	7.5664	3.4962	7.7651	7.9229	5.1868	8.0182	5.8182	0	8.6677	11.5474	9.0202	8.3068	10.0909	3.8507	9.5231	3.8507	10.6696	7.2106	8.2292	10.5961	9.8401	0.652	9.001	10.622	8.4633	8.6646	9.8156	7.4007	7.0206	8.8505	8.5538	7.3409
TCGA-86-8076-01	993	0	5.8163	9.0055	6.1609	10.4145	7.1066	11.4682	6.9242	6.2669	6.8627	9.1321	5.5899	6.8334	5.1256	8.8594	8.0386	8.3071	12.038	9.8681	0.8316	5.9588	13.0837	9.2541	9.923	8.4016	4.272	9.2403	3.9469	9.923	8.8988	8.3946	10.1208	9.4574	0	8.7237	12.6617	9.0895	9.2421	10.1092	6.4772	8.8276	10.0351	10.0307	8.9187
TCGA-86-8278-01	944	0	7.4164	8.4231	6.2903	10.7576	7.8678	13.4381	6.6483	6.5399	6.4741	9.8401	4.0939	7.3854	0.8302	7.5661	7.489	3.44	4.372	5.3367	0	4.9817	11.9031	9.4614	10.0966	9.4024	3.721	9.1734	3.5399	9.7027	6.006	7.5495	10.6949	9.2393	1.1994	8.5622	12.2294	8.0817	9.7032	9.8639	5.6663	7.4932	11.0741	9.5985	8.1158
TCGA-86-8279-01	949	0	1.1278	8.3171	5.5523	10.4236	7.6382	10.8024	5.2152	4.8357	4.9369	9.2591	4.0363	0	0.3744	7.3494	6.8649	1.7529	6.4637	5.8552	0	4.2758	12.4262	8.5976	9.7296	9.5162	0.6714	8.1176	3.43	10.6011	4.5557	8.2266	9.2891	9.5045	0	9.304	11.7865	8.9751	8.7592	10.2896	6.3293	2.4448	7.605	11.6495	2.792
TCGA-86-8280-01	701	0	8.0394	9.2131	6.2526	10.6234	7.139	10.5895	7.9145	6.0299	6.5044	9.3324	6.0379	5.2509	3.7	8.3144	6.782	7.57	8.5298	5.7856	0	6.0617	12.4119	9.5553	9.8166	9.7098	2.9831	9.2411	3.853	12.0803	7.5645	8.2617	10.4722	10.7497	0.7883	8.5704	10.7467	9.2703	8.9094	9.8892	6.8743	5.4559	10.138	7.5422	7.8272
TCGA-86-8281-01	0	0	6.3226	9.8037	6.677	10.1022	7.3571	11.5112	5.0306	5.6365	7.0397	8.5625	4.4292	10.2139	1.0318	9.0721	6.4197	6.5962	15.9227	15.5053	1.6272	4.2024	14.3432	9.4573	11.0638	6.8654	3.7586	8.5732	3.2787	9.9577	6.0239	8.0067	10.677	10.7353	0.431	8.6439	11.1907	10.2844	7.9795	11.068	5.6264	8.5235	1.619	9.2884	8.3741
TCGA-86-8358-01	653	0	2.4962	7.8357	7.2497	10.0161	4.7114	10.4182	7.5237	7.7671	11.6459	10.0974	8.0674	1.5798	1.218	10.4704	8.3217	5.9697	14.7887	13.9933	5.2277	5.0658	9.3388	6.9504	11.1097	10.6304	3.4524	9.3967	5.4187	9.3535	5.7562	10.4336	9.4781	9.3359	0.7339	10.9607	11.0406	9.2754	8.0602	9.4875	4.9154	10.7374	7.0405	12.392	6.4353
TCGA-86-8359-01	444	1	2.551	8.8292	4.3099	10.8015	6.2311	10.7672	4.265	5.1302	4.9993	11.2046	2.9417	6.147	2.2157	9.2068	6.8329	4.8247	8.8597	6.77	1.7783	5.8147	12.8327	7.2734	9.8052	7.7594	2.2157	8.9861	3.6488	9.1677	9.9864	8.2635	10.2602	10.9977	2.2157	9.6104	13.0565	9.36	9.3263	10.3476	4.9146	9.042	6.8304	9.4605	9.376
TCGA-86-8585-01	353	0	5.7824	8.3453	4.5271	10.7345	6.0216	11.9396	7.6951	6.2686	4.4564	9.3626	2.7036	2.9292	1.0723	10.4172	8.2751	4.3036	8.2854	7.9986	1.0723	5.2247	12.4838	8.6407	9.1211	9.1125	1.6806	8.9674	3.7149	10.0659	6.1835	7.6367	11.088	8.9738	2.7036	8.5202	9.2124	8.5837	9.7409	9.8397	6.1835	2.7036	6.4721	7.664	7.6323
TCGA-86-8668-01	423	0	9.1906	8.5374	6.398	10.1885	5.5123	10.4926	8.3127	5.0763	7.9104	9.2743	5.2834	7.9543	4.0553	8.0826	9.0295	6.6646	7.1535	5.5586	0	5.1687	13.7608	9.4366	10.0495	8.5968	1.6925	9.0938	5.6682	11.0977	8.0137	7.5556	10.3076	10.4933	0	8.0627	10.2524	7.2901	9.9215	9.2797	6.2924	3.1992	7.4116	5.9887	7.4148
TCGA-86-8669-01	938	0	4.0663	9.7115	6.9004	10.4288	7.9542	12.2237	12.0778	4.9362	6.174	9.9165	8.8227	6.1096	3.6255	6.9673	7.0313	2.0533	10.0807	9.7191	6.5293	11.9563	13.3048	8.324	8.8247	9.1111	4.1196	8.3632	3.296	8.916	6.2113	10.5133	11.6368	10.2236	1.1765	9.1548	10.6605	9.5871	8.181	9.6035	6.3515	6.96	2.6894	8.4174	7.1319
TCGA-86-8671-01	839	0	5.8241	9.1484	5.2449	10.8578	5.4768	11.2448	8.2112	5.958	6.1154	10.3524	5.7522	4.9936	6.7609	7.0817	7.6941	4.5262	7.0817	5.9192	0	7.1392	12.5627	8.8473	9.4932	9.3348	3.8852	9.9996	4.5936	11.1883	7.9566	7.0553	10.9203	9.7548	0	8.5235	11.9884	8.0254	9.0736	9.0912	7.1504	4.5936	8.1758	7.3192	8.704
TCGA-86-8672-01	19	1	1.6225	9.3588	3.3232	10.3653	7.1108	10.7852	4.4456	6.6453	5.2639	8.8618	3.2197	0.7596	0.7596	9.0473	6.8058	2.1587	15.3582	15.2453	0.7596	5.0083	12.8172	9.0187	8.8006	10.3269	5.8008	8.5247	5.2376	10.5047	5.1558	7.2263	9.6991	9.4925	1.2546	9.6797	11.1114	7.9646	9.1617	9.6699	6.7878	4.3009	7.6349	11.7862	8.2306
TCGA-86-8673-01	862	0	3.6702	9.0768	3.857	9.9957	7.5821	11.9293	3.7973	6.0594	5.03	9.8081	1.9755	1.7424	0	8.7275	8.2406	5.4534	9.7941	7.3785	0.6658	6.0077	11.6342	8.2738	7.1172	9.6698	5.5468	7.6678	6.1336	9.4742	6.3252	7.9367	10.2152	10.3555	1.7424	10.6294	11.7471	9.3146	9.2986	9.5664	7.9051	2.6504	9.898	10.334	8.609
TCGA-86-8674-01	806	0	2.7271	9.4142	3.0544	10.3728	7.7111	10.3592	2.303	6.0971	5.689	7.2031	2.303	10.3946	2.9533	10.1503	5.689	4.2891	17.0691	15.8021	0	2.9533	14.6627	10.008	10.026	7.0745	1.6998	7.5646	2.8446	7.4158	6.0242	7.533	11.4775	10.6128	0	8.725	12.4707	10.2961	8.5127	11.3119	6.2959	9.5481	4.0878	9.7963	8.5391

TCGA-86-A456-01	896	0	6.7635	8.8711	5.7452	9.6397	6.6234	11.6045	7.9861	5.0926	5.8525	10.3463	3.7464	4.1821	2.1855	7.46	7.3282	7.1772	13.4481	12.5424	0	4.818	12.1222	8.6659	10.2208	9.6192	5.5767	8.2101	5.3232	10.7011	5.6634	8.6726	11.1939	9.8261	0	9.9233	10.7435	9.2048	9.1473	9.6922	6.3476	4.6916	2.2675	8.2995	8.5613
TCGA-86-A4D0-01	116	1	3.9901	8.0079	6.4824	9.6852	6.1362	11.9406	9.7412	6.9028	9.3641	9.9569	8.5278	5.964	5.0287	5.1087	7.2099	4.7826	15.9409	14.4431	7.9948	2.2396	13.2925	8.4474	8.1611	7.9682	3.5488	8.1913	4.0317	7.6248	8.4778	10.2469	9.5812	8.898	0	12.6203	12.6862	9.7867	8.0286	9.951	5.2565	9.6447	3.1449	12.3599	6.4673
TCGA-86-A4JF-01	737	1	5.0396	9.4593	5.3528	10.5477	7.0407	11.7221	5.3837	6.2785	6.0371	9.8126	4.0828	0.9153	0	5.9674	6.1937	2.318	6.8378	5.0003	0	4.8532	12.8582	8.4274	9.077	10.2909	4.5601	8.1611	4.9391	9.0793	4.6646	7.3071	9.8147	10.4781	0	9.3133	12.7566	8.4514	8.6004	10.7056	6.1037	3.5398	4.1788	9.2412	8.699
TCGA-86-A4P7-01	415	0	7.649	9.4201	6.1051	10.87	6.7703	11.2875	7.6249	5.9914	7.0227	10.2591	6.9691	4.6276	3.4544	6.3844	8.3401	5.9697	7.7189	6.8673	0	6.9077	12.8292	8.8327	9.4595	9.3918	2.9338	8.7977	6.1249	11.8672	8.1818	8.5434	10.6423	10.3841	1.2769	9.4731	11.7479	7.4481	9.0302	9.6799	7.3083	4.8808	2.6895	8.3776	7.8193
TCGA-86-A4P8-01	805	0	9.2771	9.4193	5.7392	10.5162	6.5543	11.7017	8.9084	5.8459	5.2448	10.2994	6.068	6.7441	1.901	6.5117	9.3332	6.3275	9.3117	7.0962	0.5418	5.7876	13.161	8.7309	9.6123	7.6582	4.7042	9.2009	5.118	12.8316	7.9676	6.7925	10.5565	9.4058	1.901	9.2209	10.3689	7.2935	9.9313	8.9206	7.3019	5.2448	3.5599	7.9729	6.3356
TCGA-91-6828-01	323	0	5.2809	8.0652	6.9132	10.3964	7.56	12.4478	6.8303	4.6188	5.8639	9.236	9.7261	1.8177	1.8177	6.003	6.5612	6.0587	10.3719	9.1634	1.8177	3.2353	11.83	8.228	9.2968	8.6069	1.8177	9.0693	2.785	10.0301	9.6899	8.3314	9.3368	9.3217	0.8811	9.1485	9.6631	7.1454	8.1098	9.2235	7.4463	8.752	3.7567	9.52	7.5919
TCGA-91-6829-01	1258	1	4.5678	8.2506	5.9402	10.6155	5.3304	10.2816	6.653	4.7994	8.3314	9.9192	3.5015	4.0878	2.847	6.6456	7.6309	5.4026	5.5209	3.901	0	9.1895	11.2756	9.7977	9.7526	8.7825	2.4973	9.1663	3.0405	11.3681	8.6102	8.9231	9.3533	9.0629	0	9.4004	10.258	8.1493	9.1831	10.1534	7.5812	7.2357	4.7264	10.9398	5.1534
TCGA-91-6830-01	60	0	6.4653	8.909	6.8668	9.8808	8.5087	11.2001	7.3729	5.6002	6.7586	10.4876	6.5145	6.5853	3.3498	8.5855	7.7103	7.4634	13.2117	13.6106	6.8949	7.1341	12.2073	8.4211	9.8994	8.7512	3.9597	9.2146	4.3332	12.2026	8.4146	6.7703	9.2907	9.7144	3.8869	9.8761	11.6649	8.4307	9.0984	9.2836	6.7381	4.387	1.9277	8.0053	6.4013
TCGA-91-6831-01	310	0	5.1275	8.9689	6.4374	10.4593	6.4114	11.0447	8.1858	7.3764	9.1425	10.5815	6.8864	1.3468	0	8.4789	6.779	5.8414	5.389	7.9268	0	8.525	12.2227	8.6186	9.6669	9.7233	2.6786	9.0739	3.3591	10.5475	6.9143	8.9847	9.8992	8.6327	0	9.7636	10.1123	8.2783	8.0155	9.0615	7.1442	2.8428	10.2379	10.7856	7.7152
TCGA-91-6835-01	79	0	4.5865	7.98	7.2882	10.7247	7.3534	11.6613	7.9381	5.6499	8.6922	10.6368	6.7366	6.7305	3.2597	7.1529	7.1205	7.22	7.1111	4.2524	0	8.8837	11.7175	8.6859	9.5935	8.0578	4.2862	9.8222	4.1088	10.8528	8.0504	7.5029	10.1013	9.9323	0.9282	8.9771	11.1025	7.9722	8.8474	9.0733	6.7965	5.5282	3.4951	7.9167	7.2715
TCGA-91-6836-01	417	0	1.467	8.7911	5.1815	9.6404	7.03	11.0616	7.8368	7.1558	9.1571	9.8087	1.6806	0	0.9124	6.8541	6.1791	3.3594	4.9945	2.0313	0	2.9292	11.5115	7.3121	9.0106	11.1316	2.4359	7.7973	3.9998	7.6047	3.9998	7.3739	8.5578	8.8977	0	10.4155	12.0715	8.5425	8.0772	9.1164	5.0532	4.2207	2.7535	8.4672	5.7293
TCGA-91-6840-01	372	0	4.2152	9.6877	5.9393	10.1952	8.1208	10.2959	5.0484	6.3439	6.0763	9.5966	9.2399	5.3749	0	7.5384	10.0551	7.7932	8.6652	7.1687	0	5.9393	10.6337	8.9094	9.2067	8.4707	1.3381	8.775	7.5205	9.6498	4.9448	8.0874	9.0387	9.1879	0.8189	8.1129	9.9586	8.3339	8.2203	9.0471	8.5985	6.2456	2.5338	7.9306	7.4655
TCGA-91-6847-01	842	0	3.539	7.4321	5.8552	10.2688	4.5407	10.2668	2.771	8.1518	7.6667	9.3295	1.0201	1.0201	1.0201	6.526	9.6705	4.0965	5.0685	4.9929	0	0.7531	7.3251	9.9844	7.7563	9.4849	0.4251	8.5061	3.4054	6.589	3.0941	8.7123	8.6897	9.9383	0	9.8398	9.2207	8.641	9.6205	9.8435	3.0941	3.6217	9.2373	10.909	2.1463
TCGA-91-6848-01	224	0	2.375	7.1467	4.8814	10.6586	4.8398	10.5067	5.9573	5.6069	8.3071	10.7044	4.3407	2.375	0.8777	4.3407	8.6274	3.9295	4.7528	3.9295	1.8125	8.5996	10.6315	7.8484	9.0392	8.8294	5.1085	8.6456	5.3046	10.3339	7.8898	8.2554	8.7836	8.9613	0	10.2332	8.0893	7.0127	8.927	9.3865	6.6045	4.9994	5.632	10.811	8.2407
TCGA-91-6849-01	35	0	4.1445	9.5585	5.1031	10.5242	7.2186	12.4285	4.3154	5.7653	5.6422	8.4402	3.4609	11.1287	2.1167	9.464	7.6842	5.8788	16.2885	15.4516	1.6892	6.7668	13.3725	10.1149	10.3134	8.3867	3.4609	9.2086	3.4609	9.7608	8.1699	9.6212	11.326	10.8426	0	7.6842	11.5507	8.4706	8.7419	10.1307	6.1055	8.328	2.6869	6.3611	9.6453
TCGA-91-7771-01	492	0	4.7701	8.903	6.4571	10.4848	6.9324	11.1627	7.104	5.5101	7.8247	9.117	5.5633	8.5319	8.9818	7.4769	8.25	5.2748	10.49	8.9554	5.4358	5.5633	12.4122	7.8101	10.2002	7.4489	2.1469	8.9235	3.7004	11.1248	8.2199	7.8083	10.1882	10.9064	2.478	8.7274	11.3944	8.053	9.7942	10.015	6.6664	6.1671	9.5544	7.5666	8.25
TCGA-91-8496-01	505	0	8.4744	10.1687	6.2648	9.8077	7.5033	11.2386	6.3766	6.9007	5.9219	9.3474	3.3636	8.5449	2.2845	7.8852	7.644	5.3397	8.2688	6.4423	0	5.9583	13.9021	8.9981	10.1153	9.4566	2.9945	7.0174	3.8243	10.9572	6.6344	8.1738	10.186	11.3438	0	7.8224	13.9255	7.9634	9.8865	10.5398	9.0198	7.5754	4.4535	6.4803	5.4201
TCGA-91-8497-01	434	1	6.7222	9.7997	6.0984	10.4128	6.81	11.7433	8.4146	5.2754	7.8205	9.9735	6.9935	5.9518	4.7801	7.0523	8.6251	5.503	8.5634	6.6288	5.1465	10.2616	12.9851	10.1534	9.4512	9.564	3.0498	8.8976	4.0201	11.9573	8.6467	6.7462	10.9624	9.6733	2.6543	9.5715	11.2614	8.5507	9.3998	9.3927	6.6856	6.9478	8.202	7.9689	7.0808
TCGA-91-8499-01	36	0	2.7882	8.6578	5.0139	10.4364	7.3318	11.2426	7.8689	9.5801	4.9043	9.9892	5.5388	9.6088	3.3857	6.4856	5.7905	5.6277	5.9506	5.6448	1.1253	4.3982	9.8869	7.5107	9.5129	10.706	3.2127	9.0746	5.3427	8.1375	3.8667	9.1474	9	9.286	0	10.5903	8.785	9.5768	10.4284	9.6088	6.1677	2.6592	8.7621	9.0571	6.7774
TCGA-91-A4BC-01	44	0	3.2457	8.3691	5.2056	10.8376	5.6613	10.236	6.8807	7.176	6.1466	10.4676	4.8934	4.0012	0.7248	6.1197	8.5458	4.8286	6.509	6.2737	1.2051	9.8312	11.8188	8.4413	8.3021	8.1278	0.7248	8.6075	3.5965	10.6932	7.2271	7.778	11.356	9.4338	2.9122	10.5206	10.7761	8.2286	9.0963	9.1083	7.0537	3.2457	6.944	11.3441	9.8384
TCGA-91-A4BD-01	603	0	5.4865	10.0582	5.429	10.6844	7.2542	11.791	4.2787	7.3269	5.6962	8.8411	2.8353	9.7976	5.2411	9.2861	7.7825	7.1243	9.8938	6.4986	0	5.1726	13.8405	8.4031	9.6305	10.6152	5.5952	8.7144	3.7305	8.6962	4.5725	8.1429	10.6814	10.3091	0.9082	9.2156	11.5813	9.5315	10.1401	9.4704	8.2783	6.4415	4.8493	9.1724	7.1777
TCGA-93-7347-01	683	0	5.0551	10.3069	6.0837	10.3747	6.6336	11.5151	7.2962	4.5566	5.6975	9.8526	5.8016	8.7295	2.3988	6.6504	7.3895	5.2591	12.0419	11.5885	0	6.7937	13.3236	8.1068	9.3456	8.8141	3.5222	9.1304	4.7356	10.7404	7.1696	7.2038	11.0071	9.6058	1.3528	9.0712	11.8458	8.5512	9.781	8.9552	7.2004	4.4323	1.3528	7.4833	8.6812
TCGA-93-7348-01	531	0	7.2751	9.0318	5.0019	10.793	6.9213	11.4674	5.964	5.7147	6.71	9.1529	4.3329	12.0269	5.2722	7.2627	8.1278	7.7437	7.4212	7.4487	0	8.0828	11.8266	8.8702	9.2849	9.483	2.3111	9.3909	3.357	9.6501	7.5889	9.1948	10.6669	9.9412	0	8.9705	11.1194	8.8035	9.268	9.8262	6.0812	7.2186	6.4597	6.7982	7.5231
TCGA-93-8067-01	186	0	4.8957	8.2016	3.9028	10.3151	6.6741	11.8479	6.7751	7.0353	7.6589	9.5429	3.7584	9.0857	5.7079	10.2682	8.0012	6.9381	17.0426	17.1114	11.4886	4.0087	12.966	8.3039	9.569	6.9515	3.7277	7.9322	4.5193	9.3029	4.2653	9.307	9.3888	9.848	0	9.443	11.528	9.9566	9.2445	10.2844	5.3929	4.4458	8.403	8.7783	6.6291
TCGA-93-A4JN-01	718	0	5.1326	8.6691	5.4255	10.0021	7.4213	10.2098	7.6771	7.6858	4.9114	10.2677	5.5069	6.0589	1.1679	6.8647	9.1261	5.8668	8.3713	5.5587	0	7.3123	11.0951	9.5232	10.0021	8.6288	4.8503	8.3059	4.206	10.0812	7.2388	9.1778	9.7505	9.7991	0	8.6794	12.1003	8.1287	9.7636	9.954	5.8668	6.2911	7.8827	9.7719	8.216
TCGA-93-A4JO-01	33	1	5.1505	8.2644	5.7502	10.4958	6.0752	10.2822	5.4728	6.0863	5.6334	10.113	5.0402	4.9207	3.8151	6.9653	7.2142	7.2642	8.4068	6.5399	1.0328	9.7566	11.911	8.6905	9.0449	8.9382	2.5128	10.219	5.5064	10.6878	6.6642	6.4617	10.856	10	0	9.0704	11.7448	8.4931	8.6739	9.8147	6.0974	7.9473	7.5342	9.5805	7.7085
TCGA-93-A4JP-01	578	0	8.59	8.6526	6.0044	10.3894	6.806	11.9252	8.7384	6.5176	6.9841	9.9195	8.678	8.8307	2.7995	6.7591	8.469	6.6434	10.7951	9.1009	0	7.1098	13.4391	8.6267	10.1001	8.4544	4.4375	9.2951	5.1848	13.199	8.3615	7.2448	9.8799	9.8094	0	8.3475	11.3252	8.9162	9.6868	9.8423	7.9532	6.2274	5.2122	9.5784	7.6534
TCGA-93-A4JQ-01	526	0	5.2826	8.7669	6.0241	10.5696	7.636	10.8353	7.893	6.0717	6.4481	9.7151	5.4064	5.5609	2.9143	5.9137	8.1359	5.2165	7.3617	6.5321	0	6.8698	12.143	7.6328	9.3072	9.0433	3.4629	9.532	6.3742	10.7035	5.7713	6.8562	9.5984	10.6316	1.2065	9.2649	11.4135	8.1886	9.5472	9.6964	5.9447	4.5107	4.6995	8.5658	7.4398
TCGA-95-7039-01	1272	0	4.5763	9.2449	7.0491	10.0839	5.5677	10.7775	4.6602	5.2261	6.1497	10.3073	4.5763	6.3525	1.2801	7.0875	5.9475	3.5502	7.3181	6.6582	0	4.9871	12.2639	8.3582	10.0128	6.3899	3.3626	6.1204	4.3923	10.3093	5.4311	7.5865	10.6652	9.9621	0.7776	8.9666	10.236	7.6664	8.1123	10.3012	5.1699	1.2801	8.9251	10.8613	4.8509
TCGA-95-7043-01	503	1	2.048	8.7421	5.1493	9.5694	8.1889	7.7162	3.6733	6.6961	6.739	10.1687	3.5818	15.5911	5.4109	8.4342	8.2459	3.8405	16.5216	16.1391	9.6062	9.707	14.7159	9.4851	8.6911	5.9575	1.3605	7.3301	4.5687	9.0734	2.8621	7.8257	10.8244	10.8503	0	11.9799	11.3083	10.0477	9.0156	9.2491	4.1261	2.2985	0	9.8264	4.4182
TCGA-95-7562-01	87	1	7.1445	7.7727	5.5339	10.9006	6.7881	12.3947	4.3749	6.2891	4.3111	9.6651	4.9986	3.6113	2.6749	8.3819	6.6073	6.6205	6.9116	4.9782	0	7.5033	11.4565	9.2411	8.4594	10.278	2.6749	8.9106	1.8848	7.8449	4.9152	7.5949	10.0477	10.5225	0	10.4066	12.7172	9.516	8.5064	10.6709	8.1279	6.9747	8.106	9.5681	7.5899
TCGA-95-7567-01	568	0	0.6439	8.9771	4.6867	10.3555	7.7618	10.5746	4.7332	4.3512	4.822	9.5749	3.9131	0.3576	0.6439	5.6902	5.5758	5.3255	7.1172	6.2066	0	2.9011	11.6548	9.0332	10.6387	9.7091	3.1035	7.7123	3.281	9.0355	5.1423	7.2569	9.7207	10.1081	0.8828	9.5542	12.8809	7.3539	8.5006	10.547	5.8035	0.8828	2.2866	9.2633	7.7914
TCGA-95-7944-01	377	0	2.7775	8.452	4.5886	11.3898	4.2706	9.1253	4.9204	5.4416	3.6682	11.7941	1.5009	3.444	0.45	9.1909	6.4249	2.3295	6.4064	6.3231	1.5009	4.3505	13.1132	7.7466	9.7378	7.3947	6.8109	9.4264	7.1857	8.8694	9.3377	10.8985	10.3218	9.8307	1.8328	9.3523	13.9437	7.8781	8.3998	9.0546	6.6414	0	5.8132	8.2724	9.6119
TCGA-95-7947-01	477	0	7.8	8.2702	8.1087	10.5577	7.5287	11.3421	5.2701	7.1701	5.1133	10.6933	3.9414	9.5519	3.5479	6.033	6.8906	6.6218	6.7045	5.8692	0.3847	9.9155	10.6912	8.693	8.2773	9.649	3.059	9.7941	4.4246	9.068	5.4723	7.8538	10.1869	9.921	0	9.0835	14.1232	9.8231	7.5335	10.3366	8.311	5.605	7.8407	9.7523	6.7254
TCGA-95-7948-01	476	0	1.5723	9.5387	7.3268	10.1251	7.5176	11.0894	4.806	5.0969	9.6773	10.9587	4.6255	6.0827	0.9904	6.4018	6.6907	4.5053	5.5153	4.3741	0	2.6469	11.6904	8.5445	11.2551	6.1377	0.9904	8.8636	3.5269	7.4702	5.6631	6.4654	10.0903	10.0093	0.4102	8.5343	12.2113	6.2227	8.9156	9.5907	5.3506	5.2166	10.3606	9.1442	6.1511
TCGA-95-8039-01	830	0	6.0638	8.7479	5.7901	10.425	7.911	11.8388	6.3149	5.3329	7.44	9.6282	5.9515	5.4896	2.3893	6.2507	6.9124	5.739	9.1591	8.8259	0.4703	5.2623	12.5183	8.2364	10.2801	7.5329	4.5922	9.1046	5.7285	10.4403	5.5969	6.7392	9.5672	9.9639	0	8.1153	12.434	8.5866	9.8396	10.0576	6.5407	3.8567	6.1326	7.7602	7.6399
TCGA-95-8494-01	84	0	0.7113	8.5947	3.4006	9.3254	7.6836	9.8386	3.215	6.5554	3.3108	10.0547	3.4851	5.1721	1.8276	8.7286	5.7001	4.3756	6.9014	4.8281	0	3.0019	11.8989	7.4193	9.8112	10.016	0	7.9222	4.2842	8.2737	3.4851	6.1214	10.8155	10.8262	0	10.9545	11.086	7.2843	8.9249	9.1455	5.5512	0	12.5936	2.8823	3.3108
TCGA-95-A4VK-01	651	0	3.4917	9.4409	4.9435	9.9331	6.7792	12.9155	5.3753	5.2514	5.8112	9.1104	4.393	5.7859	6.9597	9.9295	7.8107	7.4927	12.4874	10.9971	1.7825	4.5212	14.7414	8.7432	9.8743	6.4812	5.8605	9.0592	3.6118	9.901	6.7663	6.9709	11.0889	9.7587	0.9827	8.6036	11.0769	7.6761	9.6785	10.5655	5.4088	7.2507	2.3586	6.8602	8.9062
TCGA-95-A4VN-01	553	0	5.1594	8.3432	5.3517	10.3191	7.7512	10.7754	5.9682	5.7864	5.8104	10.2335	3.8042	3.5989	2.3698	6.5842	6.7424	6.1485	9.0472	7.8702	0	7.7135	12.299	7.3249	8.6398	7.095	2.3698	8.6647	6.0308	9.4192	5.8573	6.4919	9.8183	11.6737	0.9459	9.0217	11.4645	9.1282	9.1519	10.6392	6.4907	4.5945	8.7891	10.1815	7.4677
TCGA-95-A4VP-01	605	0	6.502	9.082	4.557	10.5624	6.3152	10.9948	7.4471	5.8998	7.5596	9.9497	8.8061	5.0202	3.5859	6.0213	7.8784	4.8471	9.9817	9.1435	1.8563	9.082	12.3599	8.4011	7.7752	8.0451	2.7583	7.9413	5.8483	11.2443	7.2624	8.7285	11.2191	9.3557	1.8563	9.9522	10.5956	8.5088	9.7917	9.5857	6.257	5.9618	5.7346	10.7943	7.2624
TCGA-97-7546-01	1285	0	7.2414	8.682	6.4621	9.5834	6.9131	11.2671	6.9379	5.5684	8.488	9.3349	4.3808	4.1877	1.3594	5.7045	8.1331	7.5292	6.3831	4.9668	0	7.8754	12.8195	8.6373	10.3673	8.6009	3.1422	8.7146	3.8649	11.9005	9.3026	8.7994	8.7271	9.5466	1.3594	8.1061	11.0696	7.9258	9.4005	9.2651	6.5769	6.6311	5.8875	6.2209	6.8361
TCGA-97-7547-01	1965	0	2.0228	8.8677	6.7404	9.8532	5.336	12.5429	5.8878	4.7217	5.8335	10.7035	4.8144	9.6801	3.0773	4.7217	7.5771	4.2767	6.0867	5.0423	0	12.4426	12.4095	7.7247	7.9276	9.998	2.5397	9.4367	1.8584	10.5898	8.4774	8.0705	9.8668	9.6308	0.5238	8.6254	12.7272	9.2656	8.3688	10.5487	8.334	5.7999	0	6.2027	6.2779
TCGA-97-7552-01	1932	0	6.5759	9.222	10.9067	10.1303	6.9461	11.0785	7.3181	5.7553	4.7066	9.435	6.6175	8.4035	5.1271	6.5331	7.0357	8.1765	9.3954	7.3758	4.3598	7.1999	12.7293	7.8874	9.971	8.5628	4.1107	9.0953	3.9464	11.3712	7.9991	7.1343	10.0389	9.9697	0.5509	8.0379	12.3141	8.8871	9.5759	9.8497	5.7799	7.1105	10.3215	5.9738	8.991
TCGA-97-7553-01	1870	0	7.217	8.8341	6.5662	10.9035	7.8953	12.2544	8.7839	5.8052	5.9372	10.1063	5.1265	5.6976	2.8581	6.4604	8.2144	7.23	9.6312	7.771	0	7.0714	13.2329	8.9559	10.1222	8.4069	3.8474	9.0729	4.3361	11.9103	8.4351	7.8091	10.09	9.5399	0.5326	8.2144	10.3774	8.2655	9.1622	9.6792	8.0418	6.2395	3.6465	9.0765	7.9141
TCGA-97-7554-01	775	0	4.5857	8.65	6.8587	10.3283	6.6006	10.0634	6.0119	5.978	6.2861	9.3232	4.7556	3.1706	2.0005	7.4353	6.9432	2.9075	6.7886	4.5553	0	8.153	11.4424	7.6518	8.1756	11.433	1.5854	8.6246	3.2486	11.2501	6.9665	7.8073	10.2907	10.2855	0.5853	10.1505	9.9173	7.3181	9.1223	10.0855	6.7821	2.5856	1.036	8.1403	8.2605
TCGA-97-7937-01	564	0	0	8.4318	5.8391	10.1344	6.0197	11.0471	4.3135	6.1397	4.3135	9.4298	2.5413	2.969	0.4873	7.9756	5.6787	2.8128	6.9441	7.1581	0	9.973	12.0655	8.8259	9.306	10.1313	2.4381	9.5575	3.4118	9.2773	5.1519	8.0958	8.6991	9.5894	0	10.0499	12.4794	7.1294	7.9825	10.2724	5.8492	0.4873	8.2439	8.7089	8.1591
TCGA-97-7938-01	18	1	5.4159	8.7339	5.7433	9.9122	8.7583	11.5651	7.3374	5.6767	5.6936	9.2392	5.807	6.985	2.9348	5.8529	7.3211	5.6422	9.697	6.9642	0	7.0783	12.8227	8.289	10.5345	7.6159	4.2575	8.6453	3.57	10.9294	8.4447	7.9766	9.1213	9.5301	0	8.6233	11.3435	8.2495	9.8903	10.4529	5.6936	5.9264	0	7.5708	6.0368
TCGA-97-7941-01	484	0	7.1402	9.875	7.8127	9.9176	6.4274	11.6504	6.5014	6.1732	7.0999	9.4977	5.8722	11.2337	9.3689	6.8025	8.046	7.4218	11.3888	10.6487	2.7703	6.0865	12.8977	8.7457	9.8027	8.6327	5.0024	9.2078	4.9729	11.4458	6.9607	6.5792	10.0246	10.8926	1.1977	7.9455	11.8746	9.1975	9.2688	9.852	6.4543	6.4436	6.695	7.2728	6.6575
TCGA-97-8171-01	568	0	7.8019	11.2245	5.3534	9.8621	8.5843	9.4502	8.8123	6.3456	6.7093	7.0128	3.0039	10.959	7.7652	9.0752	6.3506	6.4042	5.5216	2.7862	0	3.3601	13.369	9.7953	11.0869	10.9267	5.0329	7.9287	2.8991	7.8164	2.1277	6.7174	10.3267	11.8175	1.0864	10.3542	12.9612	9.5416	10.4696	10.4627	4.4999	5.0329	10.8088	7.8396	2.7862
TCGA-97-8172-01	545	0	4.9862	9.2455	7.0644	10.1081	4.7794	11.2252	7.1582	5.8016	8.2365	9.7756	6.8193	4.5541	2.2341	7.3079	7.7218	4.9619	8.2616	6.0523	0.3387	6.1683	12.9418	9.2661	9.9176	9.0265	2.713	9.1042	4.1648	12.4552	8.8492	6.7926	10.1483	9.7944	0.6128	8.9468	9.8389	8.1261	8.9115	9.2278	6.8789	4.9619	5.2497	8.3485	7.3055
TCGA-97-8174-01	164	1	8.8216	9.2562	6.8372	9.4982	8.1376	10.599	8.1818	5.6039	7.7579	9.6691	5.9931	5.3075	2.9716	8.8067	7.836	3.3657	11.6734	10.5443	0	6.112	13.1773	8.9723	10.3071	7.704	3.8218	8.3797	4.2531	11.5451	8.4959	7.9649	10.1664	9.9147	1.6736	7.8081	9.8477	8.8312	9.6598	9.6357	6.4914	8.7969	3.0821	7.785	9.3101
TCGA-97-8175-01	551	0	4.6031	7.9998	5.8932	10.5249	7.3247	10.203	5.2027	6.5738	2.5533	9.4774	1.6262	4.7779	2.0463	8.1557	8.2477	3.1699	11.3431	10.4626	0	4.5823	11.511	8.3966	9.0594	7.4361	3.1699	9.3696	4.3062	7.9939	4.6236	8.3122	11.0493	10.6407	0.4306	9.6979	9.4196	9.0753	10.0948	9.8312	5.4863	4.1167	10.5795	4.8318	5.104
TCGA-97-8176-01	468	1	5.671	9.5523	5.023	10.1597	7.8999	11.5379	7.6665	4.5604	5.602	9.563	3.3102	8.3759	8.4091	8.0369	6.7867	6.1041	14.2775	14.6435	6.0533	6.141	13.3103	8.3499	9.6061	6.631	4.9134	8.825	3.7535	9.0997	5.3933	7.5648	10.0295	11.0019	1.4771	10.2839	11.5307	9.3841	8.8951	10.5416	7.1184	8.1469	1.1304	10.9598	7.3144
TCGA-97-8177-01	499	0	7.2489	9.7731	5.7791	10.7221	6.4348	11.5054	7.3465	6.094	6.9654	9.5175	3.5417	11.7802	4.2105	7.9911	8.7444	5.277	8.0057	7.1856	0	9.4266	13.4755	9.1952	10.5061	11.1304	3.0968	9.379	3.0376	11.1269	6.5817	9.001	11.1106	11.1215	0.4259	9.0019	13.0102	8.7421	9.7165	9.9397	7.9396	7.3465	8.2157	9.4345	6.7614
TCGA-97-8179-01	435	0	5.4232	8.5688	5.3289	9.33	7.0796	11.0332	6.1624	5.3427	6.3661	9.5395	4.0463	5.0535	3.1936	8.9157	7.1523	7.3608	16.2004	13.688	0.8287	6.6981	13.0752	9.7035	11.2468	9.0219	1.5559	8.3581	4.8742	11.2891	5.3565	8.2005	9.612	10.3197	0	8.9134	8.6052	9.2605	10.0194	10.8342	6.4063	4.1125	0.8287	9.7426	8.8322
TCGA-97-8547-01	657	0	1.3403	9.2344	5.0519	10.5082	7.6002	11.0303	5.7714	5.7094	6.3475	9.3484	8.7961	0.8205	0	8.2783	7.9744	6.6412	7.18	8.0843	0.8205	5.9065	12.9676	8.2016	9.5153	10.5844	4.0286	8.6359	5.6446	9.0424	5.3244	7.093	11.0408	9.7126	2.2721	9.8709	10.719	8.6851	8.7378	9.1552	7.0359	1.3403	10.5403	8.0638	2.6695
TCGA-97-8552-01	626	0	7.6379	9.0915	5.925	10.1884	7.3232	11.1719	7.4034	6.8227	6.9562	9.2918	5.6375	7.5793	4.6311	7.9575	8.5639	8.9861	10.0156	6.5868	0.6869	5.9536	13.2746	9.969	10.0885	9.8415	2.9462	9.3288	4.3584	11.0939	7.593	8.0112	10.6843	10.7223	0	8.6142	11.3743	9.2984	9.4658	9.3123	7.0587	7.3665	6.9625	7.1106	7.5037
TCGA-97-A4LX-01	614	0	5.0301	9.5612	5.4486	10.7361	7.3122	12.2852	6.2851	6.0939	8.6327	10.5662	7.5877	3.8275	2.4327	8.3472	7.5373	4.147	7.055	4.438	0	4.9706	12.2424	8.071	10.0556	10.1763	3.9558	9.6523	3.6868	10.3767	5.895	7.0862	10.5339	10.074	0.526	9.0384	10.9372	8.1151	9.1171	9.8903	7.0261	5.1951	4.3159	9.3194	7.9088
TCGA-97-A4M0-01	652	0	4.0847	10.2831	4.4282	10.4026	7.1678	10.5305	4.123	5.4853	8.5528	9.8459	6.9402	6.5486	2.3521	7.3644	6.0791	3.053	7.8254	8.3049	0	4.1602	13.1378	7.1995	9.4273	9.21	2.4756	9.4158	5.4556	9.6452	6.127	6.8909	10.211	10.1063	0	8.8453	12.1213	8.5668	8.8381	9.6889	6.4006	5.7649	6.2625	9.5018	5.2804
TCGA-97-A4M1-01	601	0	8.0111	9.5674	3.6157	10.1727	5.8525	10.7129	8.0675	6.3791	6.1397	9.1223	4.141	7.3873	1.9771	7.1887	8.7912	5.2369	9.8459	8.8382	0	7.4493	13.6556	9.3998	9.8528	9.2102	4.6429	7.6277	5.3099	11.6611	7.2222	7.6518	10.6788	9.6715	1.3036	8.5987	11.9149	9.0247	10.4697	9.6973	7.7277	6.3184	2.863	9.0448	4.8284
TCGA-97-A4M2-01	624	0	8.0934	9.6985	6.0863	10.4218	5.8658	11.9674	7.6398	6.0343	7.2862	10.3214	5.8296	7.0497	3.5356	7.7243	8.9489	7.6152	10.5654	9.5263	0	8.8455	13.4638	9.4901	10.4538	7.6674	4.5333	9.0203	4.7294	12.0658	7.4659	6.8112	11.0872	10.816	0	9.0296	11.8878	9.1713	9.6655	9.7051	6.823	6.7213	5.054	9.8363	8.8635
TCGA-97-A4M3-01	540	0	4.3734	9.6802	4.4082	10.0563	7.1561	10.1245	4.923	5.5082	7.302	8.7846	4.3378	7.8045	0.5905	8.931	7.1251	3.6003	16.0154	16.2442	2.9214	4.2637	14.591	9.665	9.697	6.8239	2.1829	7.8684	4.4082	10.1872	5.6013	9.8715	10.8774	11.0885	0.5905	8.4387	12.5697	8.7715	8.9115	9.8839	5.0606	10.5005	0.5905	9.7632	6.0277
TCGA-97-A4M5-01	634	0	6.5795	9.3361	5.607	10.6116	5.9863	10.5693	6.718	6.1986	6.6654	9.9175	5.918	7.8032	4.7303	8.3995	8.096	7.7869	9.7023	9.4108	1.975	6.5732	13.2153	9.0632	10.2552	7.5004	3.7163	9.2837	4.2163	11.6897	6.4264	7.2175	10.9236	9.7848	1.174	8.488	10.8414	8.8081	9.451	9.92	7.0858	5.2423	3.5323	7.4494	7.0221
TCGA-97-A4M6-01	568	0	5.938	8.6759	4.8285	10.4053	5.6925	12.1064	5.791	6.4336	7.2501	9.7189	5.2482	8.4172	6.626	6.626	6.9153	4.3678	11.7493	10.7866	2.9733	5.4726	12.4423	8.5032	9.5877	10.0412	4.125	9.6726	3.4662	11.2256	6.4027	9.0005	11.2225	9.8672	1.7156	8.8403	9.8965	7.8928	9.5662	9.8917	7.3705	6.8252	7.5773	8.1809	6.2466
TCGA-97-A4M7-01	629	0	6.2021	9.7309	5.0357	10.6624	7.0452	10.4309	7.2545	6.1767	6.1331	9.458	6.5319	9.4084	7.1449	7.364	8.3463	7.9498	8.3018	6.1331	0.8949	6.7083	13.3633	8.9138	9.9912	9.4203	5.8075	9.6139	5.8404	10.1508	6.5453	9.0381	11.1061	10.8454	0	8.5202	9.8725	8.331	10.4363	9.6163	6.897	5.3863	5.8138	7.8213	8.0231
TCGA-99-7458-01	747	0	6.5499	8.4163	6.4927	10.1766	6.8481	12.2126	7.1125	4.9446	6.4767	9.739	5.2757	4.9132	3.9907	6.8646	7.5974	6.3181	6.7271	4.7093	0	6.893	12.5281	7.8768	9.3067	10.4002	3.5758	8.952	4.5141	11.1936	7.6813	7.6188	9.5918	9.8563	0.733	9.0472	11.4826	8.1717	8.3195	9.6121	6.7802	4.7978	11.9102	8.5459	6.745
TCGA-99-8025-01	1060	0	3.6241	8.6204	6.3453	9.9019	6.0728	10.0521	5.7983	6.5179	6.4146	10.0665	4.6044	7.0584	6.2911	9.0457	7.3003	8.7365	12.0483	11.0203	0	5.672	12.7062	8.0366	10.6319	9.8434	2.5021	8.486	4.9381	10.2439	4.7365	6.9849	9.8255	9.8298	0.7367	9.2949	10.9946	8.8229	8.7622	9.6524	5.3571	4.7721	5.1086	8.4438	7.7983
TCGA-99-8028-01	1118	0	4.3792	9.0377	4.7463	11.2431	6.1625	10.7588	6.981	6.8685	5.1302	9.6243	5.4474	4.0819	3.5028	7.0293	7.9453	6.3943	9.0126	6.5417	0	5.2816	12.4708	7.794	9.0412	8.9399	4.5744	9.427	5.1302	10.887	6.9612	8.3239	10.7534	9.1986	0.8963	9.4351	10.8414	8.5367	9.5111	8.9424	7.5994	4.8789	7.1539	9.1557	9.3472
TCGA-99-8032-01	44	0	5.0813	8.8966	5.3676	10.8696	4.7022	10.7934	7.5085	6.9104	8.3677	10.1611	8.9478	2.0458	1.7427	7.6861	8.5169	4.8267	10.0378	10.3806	2.7796	8.8522	11.467	7.953	7.7635	9.2846	3.7142	8.3712	4.1866	11.2543	7.4354	9.702	11.0507	8.8955	1.3587	10.1749	11.4429	8.4296	8.4345	9.2864	6.2936	6.5559	7.7628	10.8659	7.6585
TCGA-99-8033-01	656	1	5.1762	8.2833	6.0997	9.9588	6.2773	10.3771	6.3756	6.5541	5.0627	12.0035	2.1749	2.7781	1.3576	10.2798	6.3688	5.9159	14.5336	13.827	4.9024	4.3869	12.9754	7.9306	9.6187	9.4921	6.8216	9.2471	4.8049	9.4898	8.1705	8.4466	9.3908	9.4637	1.3576	9.218	11.2406	7.5098	8.9821	9.8974	6.2989	6.776	10.2721	9.1874	9.1262
TCGA-99-AA5R-01	658	0	8.8361	9.4223	5.5756	10.098	6.307	11.4853	8.2655	6.8591	6.3934	10.2194	6.8456	5.5254	0.6256	6.4661	8.4671	4.6685	8.4912	6.8524	0	6.9695	12.6597	8.6845	9.3665	9.1171	4.9728	9.09	4.9975	13.0684	7.7764	6.6347	10.1548	10.067	2.0899	8.9996	11.7695	7.6573	10.0307	9.2205	6.7614	5.0692	4.2681	8.9655	7.1947
TCGA-J2-8192-01	739	0	3.525	8.6642	6.7141	10.4082	6.3525	11.8655	7.1513	5.743	5.7631	9.6149	5.2962	8.5676	2.1305	7.5014	8.01	5.7124	6.8686	6.5153	0	8.9817	12.8912	8.0677	9.5273	9.0302	2.5559	9.3984	3.5713	10.8532	7.4651	8.9731	10.4078	9.4341	0	8.4487	10.7707	7.8665	9.6087	9.8158	7.5163	5.8688	8.6265	6.5271	6.319
TCGA-J2-8194-01	724	0	6.5259	8.6503	6.0816	10.216	7.0756	11.876	6.6248	4.668	6.1183	9.4078	3.2813	7.8259	2.4225	9.2335	6.2398	6.1092	16.2698	15.0128	2.3001	3.7705	12.7802	8.356	11.3555	7.5385	2.4225	9.2029	3.8599	9.7608	5.2339	8.3508	10.0002	9.5571	0	7.9982	10.7694	8.913	8.9234	10.2349	7.2049	6.2315	4.862	9.5844	9.0827
TCGA-J2-A4AD-01	550	1	7.29	7.2132	5.0653	9.8045	5.2765	11.5712	7.0326	6.4307	6.5728	9.0123	4.5441	8.1057	4.6412	8.2665	6.73	6.417	12.3497	14.136	0.8576	4.71	11.201	9.4465	9.101	6.5291	5.5129	7.7883	6.3611	10.6738	7.8606	6.6583	10.4517	9.8553	1.1493	9.5209	10.9006	7.1444	10.2135	10.9378	4.8178	6.9544	3.6465	9.7794	6.0524
TCGA-J2-A4AE-01	1079	0	8.7485	9.1003	6.5238	10.1865	7.8695	12.33	7.2452	7.1035	4.5152	9.4925	4.7232	9.0006	4.5879	7.5931	7.6662	7.284	8.9653	6.5331	0	4.5879	12.5427	8.7123	10.538	7.9681	4.905	8.774	5.2789	10.9016	7.0973	9.4077	10.3463	10.3219	1.9837	10.6329	11.3203	8.8773	10.1696	9.3217	5.5023	4.6572	7.2811	8.1946	8.8257
TCGA-J2-A4AG-01	988	0	4.4846	8.8106	5.0479	9.565	6.9507	11.2199	5.8819	5.5419	7.6537	9.8125	4.8926	3.9666	1.4268	4.1607	6.6847	3.056	6.6119	4.6554	0	5.2746	13.7035	9.2486	8.7942	7.6334	2.3045	8.8846	4.2054	10.5795	8.1126	7.8014	9.1549	9.4149	3.7422	8.5881	11.7748	8.4556	8.9664	9.9338	6.5867	4.0176	8.9714	7.1863	6.6284
TCGA-L4-A4E5-01	578	0	3.6295	9.8423	6.2667	9.9202	7.0843	10.5694	4.9975	5.9747	11.4958	10.2578	3.7805	1.7116	1.7116	7.287	6.0057	4.7758	5.2252	2.6917	0.5411	4.4248	12.2829	8.7115	8.8937	6.9632	4.1568	7.7413	3.7805	8.7713	6.9151	6.9415	9.4642	10.7953	0.5411	10.6427	12.0565	9.7779	8.6766	9.737	5.1532	2.7899	2.1551	10.8293	6.1788
TCGA-L4-A4E6-01	435	0	9.1061	9.8268	5.8373	12.0987	7.0713	12.4598	8.5605	5.9192	6.7371	10.2726	4.6709	5.4899	2.2908	6.3513	10.5155	4.1445	8.8151	5.9058	0	6.6277	13.1971	9.1134	9.0051	7.3175	6.2911	8.6905	4.9031	11.7868	8.663	6.8663	10.7319	10.4833	1.6892	8.6531	10.5188	7.6245	10.0596	9.0915	8.3155	5.8788	2.6907	8.0926	6.7961
TCGA-L9-A443-01	193	1	4.2456	11.2655	3.8557	9.7286	6.7433	11.8043	5.7492	6.8664	7.7888	8.8702	8.5268	5.5035	5.7033	7.3759	7.6383	7.1262	7.5064	6.7204	0	5.4489	12.2381	7.5971	9.1616	6.7583	5.333	8.4957	4.6842	9.9372	6.4601	6.9424	9.4835	9.3681	1.0862	9.1038	10.5984	8.1064	9.0905	9.1214	5.4113	4.2456	3.9106	7.9997	5.8226
TCGA-L9-A444-01	307	0	0	9.763	5.8184	10.7329	6.1622	11.3481	5.0396	5.5643	6.2221	9.4084	6.9003	2.6588	1.7128	6.7041	6.4013	2.8226	6.4647	4.0828	0	5.0396	12.7625	7.8186	9.7012	9.9023	2.9697	8.5462	3.7979	9.9149	5.1362	6.7791	10.8112	9.3928	0	9.5398	10.6009	9.0192	8.4864	9.1406	6.1925	1.3326	1.413	8.2763	9.1328

TCGA-L9-A50W-01	442	1	3.6152	10.377	4.5551	10.6219	7.199	10.2684	8.1337	5.4641	6.6552	9.3123	3.6587	8.4036	9.2752	6.9372	5.8014	6.0827	11.3954	7.084	1.8584	5.7216	11.8234	7.962	9.8937	10.1871	5.6905	9.5654	4.6004	9.2479	6.4414	7.0821	10.2015	10.4577	0	9.3063	11.9216	7.835	9.4816	9.067	5.0558	4.7487	4.3725	5.6263	5.593
TCGA-L9-A5IP-01	58	1	2.0241	8.1187	5.3838	9.8914	7.0205	11.2034	4.6992	5.8895	6.3038	11.954	2.0241	2.4282	0.908	9.0365	8.7902	5.1722	13.5952	12.6186	2.4282	2.3056	10.7446	8.1657	8.2491	7.8657	2.3056	9.3326	3.777	7.9903	6.5994	7.553	10.1125	10.1849	1.4609	9.2684	12.4297	9.2796	9.1115	11.0195	5.8572	7.4181	5.6514	7.2118	7.5854
TCGA-L9-A743-01	664	0	4.9859	9.1947	6.3695	10.2022	7.2798	11.94	6.9348	6.3192	5.9073	10.4593	5.5484	5.4583	3.6785	7.2621	8.8199	7.5324	8.63	6.5181	0	5.9847	12.0508	8.1608	9.1831	9.2837	3.3177	9.6419	5.0694	10.154	6.7884	8.1431	10.6363	10.2223	0.5579	9.0142	10.96	8.2773	9.4882	9.6152	6.2403	5.6468	6.0691	9.4032	8.6266
TCGA-L9-A7SV-01	565	0	1.7063	9.7063	6.3921	9.4013	5.8941	9.992	3.095	7.584	4.8724	11.2192	7.0637	3.3296	2.4664	6.2705	6.2203	6.9886	12.2746	8.6604	0.4617	3.5313	13.3342	9.1352	9.6044	10.3161	3.7892	7.6693	8.4115	9.3539	8.3986	7.2069	9.3481	9.291	0.4617	10.0946	11.7961	9.0749	9.083	9.5217	4.4175	1.3271	1.5682	10.1368	4.4427
TCGA-L9-A8F4-01	476	0	1.8113	8.1162	5.6299	10.3491	8.2351	11.912	5.3333	6.949	6.0498	10.5824	4.3686	5.7692	0.877	8.7915	6.9827	4.2782	7.0763	3.8039	0.877	4.9788	11.0332	9.3831	9.1858	9.4536	2.5895	8.743	3.928	10.4845	5.4483	7.3428	10.7033	9.4415	1.6282	9.9245	9.7064	8.8489	8.7682	8.8397	6.5715	6.1209	3.7843	11.4121	8.1633
TCGA-MN-A4N1-01	827	0	1.4125	9.3471	4.2692	10.2987	5.7708	11.8395	5.6205	6.4568	5.3389	9.8638	3.611	3.6592	0	7.2929	6.3506	5.08	5.8868	1.9666	0	5.6685	11.6476	8.9766	7.6737	6.9685	3.0839	7.635	4.2056	9.0519	5.7926	8.3738	9.7133	10.9904	1.1677	9.9852	11.0428	9.2618	9.4092	10.5711	5.6205	5.9752	3.2188	11.3019	3.4558
TCGA-MN-A4N4-01	1175	0	0.9715	8.2211	3.9462	10.3948	4.5887	10.8094	6.0623	5.6161	5.4829	10.2099	3.3404	4.0751	0	8.3371	7.1426	3.3404	6.1485	6.3861	0	6.3861	11.1015	8.7387	8.5738	10.098	1.5469	9.2657	5.0317	10.884	7.8735	7.1675	10.0033	9.5105	0	8.5592	11.1187	8.8329	8.7866	9.781	6.9598	1.2877	3.6019	8.8176	6.1721
TCGA-MN-A4N5-01	84	0	5.4566	8.2542	5.218	10.4174	8.2672	11.0112	6.7613	5.4822	9.3707	10.0249	3.2122	2.6131	0	8.1198	7.0626	4.6673	7.1331	4.0659	0	5.6612	10.8608	8.2672	9.567	9.1385	5.0046	9.1049	5.8699	8.9156	4.7749	7.3582	10.2784	10.2957	0.4789	8.4889	11.9765	9.3378	9.4188	9.6786	5.6724	4.2563	5.2247	11.0522	7.0455
TCGA-MP-A4SV-01	2620	1	3.4374	9.4273	5.2714	10.0481	7.5401	11.4362	5.6609	6.6342	6.776	10.2125	7.6331	6.9557	3.3188	6.8527	6.4769	5.7322	7.5032	5.0094	0	5.155	12.6683	8.5804	9.4895	10.0712	2.3993	8.5642	4.9706	9.2215	5.5599	7.4498	9.8558	9.9132	1.1907	9.6715	11.7371	9.5083	8.7675	10.0163	5.8113	1.6497	10.692	8.0991	7.697
TCGA-MP-A4SW-01	1778	1	6.943	9.6392	5.6621	10.6096	6.4516	11.3432	5.7255	6.398	5.4227	9.2449	3.9181	10.3794	1.9	6.7792	7.5312	6.3902	8.5616	7.0949	1.9	4.703	13.2566	8.9462	9.8807	10.1826	3.1278	9.0502	4.0429	9.5262	4.0822	6.9484	11.5652	11.1947	1.2425	10.0507	12.1131	7.9018	9.302	10.5403	7.2279	4.4257	4.848	10.0308	7.1281
TCGA-MP-A4SY-01	1501	1	2.011	9.187	5.9349	10.1669	6.3944	11.5159	6.024	5.9349	8.2086	9.8878	4.9482	5.1016	3.8061	7.7732	7.5313	6.3103	9.8066	11.0632	0.8999	6.5017	12.9356	8.355	9.791	10.0904	5.7957	8.4312	5.654	10.389	5.6908	7.5973	9.1621	9.8313	0	9.2642	10.1383	9.0069	9.6316	9.8871	6.0525	2.9057	8.7406	7.9828	5.9247
TCGA-MP-A4T4-01	2617	1	2.5443	8.4689	5.522	10.701	7.3089	11.8519	5.522	5.3952	5.8588	10.4846	4.2016	3.8613	3.5989	7.9597	7.0389	6.3863	8.0779	7.6621	0	5.5959	11.5128	8.5362	9.0597	9.6022	3.3481	8.6218	4.7857	9.5427	6.0393	7.3753	10.2887	10.8436	0.5688	10.5791	10.7263	8.6359	9.0226	9.3098	6.8998	2.9573	4.3093	10.2266	8.232
TCGA-MP-A4T6-01	1790	1	6.3029	10.1171	5.8234	11.1264	6.7234	12.3169	5.0533	6.6198	6.4907	9.2077	3.8979	3.1781	2.5254	8.2039	8.1229	6.2827	5.6659	3.1186	0	3.0565	11.3733	9.7856	9.6614	10.0435	1.0689	9.4565	3.668	8.8533	4.5437	7.01	10.2431	11.1333	1.3009	9.8917	10.302	7.1999	8.9747	10.3667	6.3359	7.4498	8.9347	6.7869	5.2876
TCGA-MP-A4T7-01	167	1	6.8037	9.9035	5.1279	10.402	6.8431	11.2607	6.7337	5.5508	7.4459	9.6357	4.1861	6.8431	8.1774	8.6382	7.7179	4.7325	13.2828	14.7958	3.2961	4.6076	13.6792	9.0568	10.0067	9.1516	4.6838	9.3372	4.9945	10.0482	5.7413	8.8635	10.0135	10.2165	0	9.1738	11.5333	9.6673	9.1815	9.926	6.1703	6.0978	5.2735	8.3743	8.1052
TCGA-MP-A4T8-01	161	1	1.1762	8.684	5.2933	10.324	4.9058	11.4137	5.9808	5.1975	5.6356	9.0828	7.595	2.3784	0.8796	9.4976	7.3384	5.5215	9.6441	8.2329	0	3.0249	11.5314	8.5514	9.4255	8.6161	4.1192	8.4407	5.3976	10.0304	5.6477	7.9863	9.9737	9.378	0.5058	10.2647	12.3834	9.0548	9.1781	10.0035	5.9517	2.5944	2.0175	10.8844	6.7391
TCGA-MP-A4T9-01	1265	1	7.601	8.7318	5.6434	9.9655	7.5031	11.7543	7.8126	6.025	9.3232	9.7862	7.897	4.115	2.0656	8.5704	7.8604	5.4984	7.7946	6.2149	0	5.6202	12.2444	8.8158	9.0243	9.3133	3.1643	8.8183	5.2033	11.2934	6.1993	7.7518	10.8905	10.4517	1.134	10.0147	10.8926	8.8094	9.3277	9.4187	6.7244	2.8019	8.9212	9.8894	5.2644
TCGA-MP-A4TA-01	950	1	3.0481	6.9936	5.1917	11.4072	7.0823	9.7846	6.8795	6.9844	6.4097	9.8211	3.7997	9.4417	4.6342	7.279	7.5043	6.8545	12.6253	12.4157	10.5301	6.1225	11.1456	9.455	10.325	8.1216	7.5235	8.5132	5.0581	9.2861	7.5107	7.8534	10.5344	11.6358	2.2127	10.2485	11.6361	9.7633	8.1444	9.6504	6.5218	5.3139	3.9689	11.3155	8.2284
TCGA-MP-A4TC-01	74	1	0	8.3236	5.9848	10.7431	5.7489	11.9234	4.8906	5.539	4.624	10.3162	3.0495	3.5191	0	6.7957	7.9755	4.3297	7.8895	6.8307	1.2418	5.9848	10.7994	7.7255	8.2566	9.6785	1.2418	9.4234	3.6292	10.6485	6.9363	6.9343	10.1971	10.246	1.2418	9.5479	11.253	7.7773	8.9739	10.2406	6.9576	0.541	7.3693	10.3497	8.0591
TCGA-MP-A4TD-01	307	1	8.1691	9.3769	5.0673	10.1734	5.4554	10.3913	8.5243	5.9338	6.5186	9.4695	6.534	9.9849	2.7896	8.5699	8.3993	5.8984	15.3892	14.0682	4.8868	5.8125	13.3139	8.7425	9.4661	8.2177	1.3095	8.5339	3.8875	11.1872	7.4752	8.4409	10.7602	10.2413	1.3095	10.0838	10.1284	9.5903	9.6534	10.1801	6.1191	8.363	7.9726	10.5106	7.3802
TCGA-MP-A4TE-01	896	1	3.0087	9.4784	4.6316	10.7533	7.3844	11.2455	8.9262	6.7673	7.3341	9.2673	9.889	4.5252	2.1777	9.7608	4.8457	6.3968	15.4503	15.0558	6.3663	2.5474	12.9389	8.3394	8.4884	8.124	3.4184	8.6804	3.4767	8.4177	7.2982	8.7978	11.4061	9.4668	1.6792	10.5325	8.5693	9.835	9.411	10.0397	4.8892	8.7588	9.2693	9.7797	6.8956
TCGA-MP-A4TF-01	336	1	4.885	7.892	4.3629	10.3182	5.6655	10.0214	3.7263	6.7638	8.6286	7.9737	2.3443	0.8605	1.3958	11.503	6.6859	5.467	8.47	6.7638	4.1138	2.7462	9.737	8.5205	9.9035	10.143	5.4127	8.9943	4.885	8.2028	5.777	8.7191	10.0986	9.4861	1.7853	9.4696	9.8356	8.7835	8.7511	9.0012	4.7603	5.6184	10.191	6.9749	7.0568
TCGA-MP-A4TH-01	741	0	5.2265	9.0054	6.2945	10.0816	7.5637	11.8693	5.8164	5.3782	6.5359	9.0686	6.3848	3.1443	1.2533	8.1224	7.5496	3.5934	8.495	6.6529	0.5473	6.1236	13.1198	8.6288	9.7922	7.5602	4.2471	9.1436	3.7986	10.9009	7.7545	6.7584	10.3816	9.4772	0.943	8.3249	10.1352	8.1946	8.6929	9.6879	5.9729	6.152	6.7571	6.599	7.4953
TCGA-MP-A4TI-01	429	1	4.7349	8.6522	4.649	11.3107	6.0239	11.0134	6.8683	6.3299	5.635	9.7882	5.3203	3.7335	0	6.2641	9.0746	3.492	6.6128	3.8913	0	5.7766	12.1174	8.1017	8.0524	9.0774	3.2018	9.4173	4.5889	10.3095	5.9421	6.864	10.2759	10.247	0.597	8.665	9.9419	6.4783	9.1791	9.3188	7.4492	2.7308	8.327	7.1954	8.5134
TCGA-MP-A4TJ-01	339	1	6.6644	8.66	6.2165	11.1529	7.1635	11.0527	7.1982	6.8286	4.8112	9.415	8.4882	6.7133	6.5615	7.7655	7.8278	8.6253	8.7299	7.1896	0	8.9291	12.1317	8.4309	9.3324	8.7709	2.6416	9.5379	4.9399	10.5437	8.0999	7.7974	10.4904	9.1381	0.9057	8.5733	10.112	8.3011	8.706	8.9497	6.883	5.6157	11.3318	6.8615	8.9799
TCGA-MP-A4TK-01	582	1	4.8937	8.8272	4.5415	10.9223	5.2905	12.172	6.2903	5.8321	6.8067	9.6592	4.9641	2.5736	2.311	6.6447	8.1812	3.5725	8.7824	7.5639	0	6.2154	11.5678	8.2695	8.0143	10.1909	2.5736	8.7677	5.4296	11.3505	6.9637	8.5632	10.2555	10.0598	1.3138	9.2319	10.5065	8.1311	9.4252	8.9377	6.4943	4.0746	6.7072	9.7897	6.7212
TCGA-MP-A5C7-01	2248	0	3.9174	10.1623	5.6309	9.7696	7.7821	10.7946	4.9092	5.9639	8.9337	7.7486	3.1513	9.9799	4.3578	9.0703	6.0111	6.6584	14.6061	12.6957	1.1667	4.5232	13.4519	9.4847	10.5176	6.6163	2.9335	8.0607	3.8772	8.9544	2.4763	9.7593	11.6856	9.7765	0.5008	9.5095	11.2481	9.7377	9.7621	10.8993	3.2172	3.5594	5.7458	8.4614	5.9051
TCGA-NJ-A4YF-01	2161	0	0.6848	9.5226	5.3177	10.9805	7.7058	12.4479	7.8647	6.3216	7.4442	10.7382	5.7672	2.0135	0	9.9611	6.9997	4.3099	8.6731	8.0381	0	2.2157	11.6102	8.9152	9.2127	7.2156	4.9716	8.6112	4.6261	8.9006	5.0792	8.6174	9.4593	9.1949	1.1474	9.7267	12.4486	11.4898	7.7993	8.5702	6.5998	5.361	5.4523	10.6477	4.7615
TCGA-NJ-A4YG-01	2261	0	6.3341	9.2611	5.3313	10.4009	5.9181	10.9434	5.5779	6.518	5.4504	8.7067	3.6493	4.1015	2.3339	6.9405	7.938	2.987	10.9544	12.2096	0	5.9854	13.1323	8.6121	9.3822	9.3709	2.6314	8.49	3.7763	9.6071	5.5426	7.2201	9.8303	9.5571	1.9586	9.7061	11.8885	8.962	9.7609	10.4911	6.4338	4.9282	8.0511	9.1482	5.0589
TCGA-NJ-A4YI-01	4	1	3.9791	9.9105	4.097	10.0021	7.8701	10.567	4.8678	5.8259	5.4362	9.0536	1.2282	7.1564	0	8.66	6.8217	4.2575	7.329	8.7926	3.9791	3.3784	14.1657	7.4668	9.7238	9.0092	2.9474	7.129	3.8507	10.1458	5.4802	6.2127	9.3743	10.1103	0	9.2978	12.1852	7.8067	8.4899	10.6958	7.6802	0.7411	7.773	8.7437	6.8387
TCGA-NJ-A4YP-01	50	0	6.811	9.4113	4.7228	9.9727	6.1522	9.45	5.2418	6.3222	7.517	8.9301	3.6361	3.8797	3.8797	7.2639	7.409	5.7439	10.9099	6.9088	3.4806	3.3786	13.2253	9.0246	10.5541	8.319	2.8273	8.2068	4.2315	8.9672	4.6948	7.0813	9.9691	10.5186	1.1829	8.4724	12.868	7.9803	9.0587	10.392	6.4281	6.0563	11.7142	6.4783	9.3972
TCGA-NJ-A4YQ-01	1432	0	8.0514	8.4146	5.3191	10.8018	7.6071	11.9299	7.2387	6.7257	7.133	9.1487	3.4652	2.5902	1.1734	9.7287	7.3759	3.0931	13.6963	15.4898	0	3.8261	12.1264	7.8305	9.2833	10.0387	3.8885	8.7889	4.3098	9.7169	5.4071	5.9328	10.4528	9.6143	1.1734	9.9618	9.5014	6.4533	8.6425	10.6601	8.6871	4.9891	3.7208	10.2756	8.2429
TCGA-NJ-A55A-01	15	0	6.6023	9.3474	6.2179	9.83	6.2514	10.0671	7.4437	7.1202	6.0183	8.9448	7.3846	10.4172	8.0307	8.0307	7.5417	9.9661	9.6673	7.2081	0.9063	6.852	12.4277	8.199	9.7041	8.0921	2.8318	9.5728	4.6208	11.5689	7.299	6.39	9.7792	9.9003	0.9063	8.5446	10.9425	8.0113	9.322	9.5494	5.8752	6.4229	4.5542	7.315	8.1196
TCGA-NJ-A55O-01	13	0	7.9899	9.3859	6.9889	10.9944	7.5113	11.5169	6.542	6.1261	6.5865	9.3769	3.5952	4.5701	3.5952	8.3225	7.8834	6.2964	9.3593	7.5523	5.0735	5.7881	11.9507	8.945	9.4475	7.4404	5.1226	9.2735	4.2097	10.327	6.4583	7.9632	10.9807	10.5551	1.1156	8.0291	13.321	9.5295	8.61	10.3728	6.7361	6.0263	11.85	7.5205	6.1851
TCGA-NJ-A55R-01	603	0	5.3105	9.7758	6.1628	10.6952	6.0205	10.5683	5.3018	7.7079	10.7287	8.9048	8.3911	8.3524	8.7202	8.8025	7.9374	7.3269	15.5371	14.192	8.9928	6.2197	13.2442	9.7666	9.7997	8.0978	6.8916	9.4074	7.0439	10.3133	7.5725	10.3934	11.3496	10.413	0.5662	9.0703	11.4543	10.5091	8.804	10.2771	5.293	8.5558	11.8257	7.9709	8.5503
TCGA-NJ-A7XG-01	617	0	5.6171	9.4125	5.0063	10.1448	7.5285	9.8268	3.135	6.7009	6.3094	9.2328	1.5017	5.4152	0.938	7.0884	8.2809	4.5223	8.0782	5.1984	0	2.3565	12.8687	8.2977	10.0925	9.1058	3.0578	8.8833	3.5838	7.825	2.9762	8.518	10.523	10.222	0	9.1976	11.181	9.3267	8.9793	9.8831	3.2083	6.1134	8.1604	9.5136	6.3585
TCGA-O1-A52J-01	1798	1	6.8012	9.5641	5.5377	10.1405	8.8762	11.8587	6.4608	5.6656	6.4529	9.8434	4.6115	2.5322	0.9686	8.4225	7.2633	7.8705	9.7687	7.7474	0	2.5322	13.287	9.2289	9.2358	10.5318	4.5242	9.2963	3.1136	9.7663	6.1073	9.4492	10.3898	10.6776	0.5641	8.3461	10.2517	9.5586	9.5206	9.7726	7.4991	1.9526	5.8966	8.1462	4.2617
TCGA-S2-AA1A-01	513	0	7.2007	9.4581	7.4696	10.3373	8.2651	10.6711	6.7782	6.0698	5.261	9.3714	6.585	13.1994	3.2506	6.3151	8.2558	5.7214	10.6089	7.1617	0	4.6578	11.8684	8.0536	8.5934	9.52	1.2081	9.1171	4.4572	11.5635	6.7782	7.0933	11.3806	10.6179	2.0963	9.6737	11.1023	7.5725	9.1755	9.2856	6.1651	7.0448	11.6123	6.3846	8.5737

id	futime	fustat	CDS1	CES1	CHST13	CKB	CP	CPS1	CPT1C	CYP1A1	CYP1B1	CYP26A1	DCK	DEGS2	DGAT1	DGAT2	DGKE	DGKG	DGKQ	DHODH	DHRS4	DPYD	DTYMK	EHHADH	FHIT	FPGT	GAL3ST1	GALT	GCAT	GCDH	GGCT	GGT5	GLS2	GLUL	GLYCTK	GMPPA	GPD1	GPX1	GPX3	GPX4	GSTM4	GSTZ1	GUCY1A2	GYS2	HAGHL
TCGA-05-4244-01	0	0	9.2896	9.4364	3.3328	11.1619	12.291	2.4693	6.0119	0	10.2389	0.7639	8.9592	6.3073	10.0178	7.3941	7.2293	6.3449	8.9368	7.7053	8.6695	10.573	9.128	8.9501	4.5662	9.2606	2.8646	8.7494	7.6285	8.4544	10.5056	6.9729	7.2526	14.3745	5.6701	9.6389	7.4001	12.8167	11.3452	14.0954	7.7339	8.1288	3.8344	0.7639	6.2489
TCGA-05-4249-01	1523	0	10.218	8.8357	3.1777	9.4988	11.943	3.7262	4.6149	0.4027	10.0277	0.7171	9.408	7.7977	10.7818	8.7466	7.2295	4.8424	8.7314	7.4041	7.9263	10.1203	8.5598	7.0377	4.9664	8.1725	7.3652	7.9062	5.7667	8.3338	9.528	9.63	7.3596	13.9576	5.8815	9.8183	5.4641	11.5475	10.6824	12.0665	8.4539	8.0376	4.5765	2.1832	5.8815
TCGA-05-4250-01	121	1	10.242	9.1149	3.1088	10.633	10.992	5.8833	5.5776	0.4132	9.9981	0.4132	10.3445	4.0227	9.5386	8.5186	7.5699	6.3145	8.6507	7.9173	8.7699	10.393	10.1682	8.8546	3.9315	8.3249	8.3308	8.4061	6.7429	8.2451	9.4587	9.3046	6.6121	13.2567	6.6121	9.6998	4.1893	12.8631	11.7022	11.6351	6.679	8.5762	5.0659	1.4103	6.5974
TCGA-05-4382-01	607	0	9.6235	10.0759	3.8953	10.6007	13.735	5.1285	6.3428	9.8363	11.3769	4.846	9.6683	6.4884	9.247	7.6043	7.7267	6.638	9.0682	6.9486	8.7708	9.828	9.8108	8.2969	4.6942	8.7477	7.1805	8.3935	6.5449	7.7748	9.5591	10.2746	5.3222	13.9188	6.1714	9.8614	3.8953	12.706	11.5816	11.3522	8.7135	8.5746	3.3692	0.7806	6.3597
TCGA-05-4384-01	426	0	10.271	13.2105	4.6687	11.3209	12.865	9.9891	5.2111	0	9.0449	0	8.674	4.3609	9.5821	9.4734	7.7387	7.4966	9.2757	7.472	9.1507	9.6262	8.3158	8.0122	5.2579	8.7306	5.6577	9.3342	7.2699	8.3487	11.0374	8.346	7.241	14.8209	6.4851	9.5004	7.9742	12.2283	13.1965	11.703	8.5455	8.5896	5.9562	0	6.9374
TCGA-05-4389-01	1369	0	9.5469	15.7508	4.6338	9.0595	12.87	8.0576	6.7909	0.4175	10.8274	0.7408	10.5902	8.6821	9.9922	10.7595	7.9869	8.1303	10.0481	7.6961	9.5311	9.389	10.0302	9.0087	5.9323	8.9264	7.8902	9.6237	8.2464	8.5462	10.4484	9.6457	8.8769	13.2412	6.6243	9.9997	6.2949	13.002	15.579	12.825	8.6108	9.4366	2.6711	2.593	5.0243
TCGA-05-4390-01	1126	0	8.665	8.4736	2.2726	13.4532	12.319	14.1699	6.6621	0	9.8405	2.8849	9.9489	10.5247	10.4845	8.5369	7.5799	6.0336	8.6763	7.0984	9.4461	9.2832	9.4213	7.2634	5.3433	7.5688	6.8415	8.9854	6.2668	9.0684	10.9037	9.0989	8.3771	14.5763	8.1429	9.8183	2.7545	11.7624	10.0358	11.7769	8.0898	7.6038	3.7153	0.7126	5.5344
TCGA-05-4395-01	0	1	9.6719	6.5813	1.3844	7.9711	16.974	1.8814	3.7955	0	8.955	1.2279	8.8397	2.1666	9.6968	9.8322	6.922	5.1765	10.1917	8.1549	7.8605	10.5468	10.4055	8.098	4.4729	8.9531	5.155	7.4987	7.2968	8.1093	10.689	9.3804	3.135	14.0106	6.0259	10.4544	4.8268	13.9233	13.0963	12.4627	6.532	8.7757	2.671	5.568	7.8369
TCGA-05-4396-01	303	1	10.927	8.0572	0	9.5318	13.157	16.1757	4.4743	0.8141	9.3873	0	8.9277	7.787	10.8589	10.1429	7.2249	6.2775	9.6024	6.6701	7.9365	9.7354	8.3125	9.1767	3.2234	8.5434	7.892	8.7688	7.3905	7.9329	9.6164	8.1214	6.4375	13.9596	8.1448	10.8493	2.8207	11.8622	10.0997	11.1272	7.5967	6.2052	4.7832	0.8141	5.0038
TCGA-05-4397-01	731	1	7.4625	13.7807	5.4102	11.5913	14.325	3.2651	6.0859	1.4683	9.3134	0.7334	10.7094	9.9043	9.5459	9.3214	8.2206	7.7017	8.7398	7.5687	10.2626	9.0365	10.2726	9.6972	4.5564	9.0512	7.623	8.6242	9.3885	9.0701	9.8604	8.1003	4.5152	14.0135	7.3082	10.8331	9.0552	12.7864	13.675	12.1654	8.7975	9.5983	3.1621	0.5278	7.1217
TCGA-05-4398-01	1431	0	9.5411	8.438	4.0769	11.1967	8.6442	4.5485	5.0336	0.2839	11.2561	1.4541	9.2881	5.5105	9.8982	7.0196	7.8939	6.0259	9.0945	6.8986	8.3802	9.9097	9.9499	9.0094	5.921	8.4235	5.9967	9.0708	7.9316	8.9338	10.6886	8.9027	4.9447	13.877	6.935	10.9226	3.7202	12.9179	12.8028	12.1489	7.6856	8.7938	4.4799	0.5211	8.3864
TCGA-05-4402-01	244	1	10.433	8.7029	4.8929	11.4418	13.862	4.5656	4.3941	0.568	9.9382	3.346	9.2203	8.7857	10.3841	8.4841	5.9719	7.0259	8.6861	6.7694	9.6953	10.4649	9.4966	9.1794	4.4901	8.9122	6.3545	8.1813	7.5188	8.2629	10.8711	10.3766	7.5874	14.302	5.5571	9.9367	4.9046	12.6528	11.0324	11.1415	7.9754	8.8986	3.346	1.1416	5.11
TCGA-05-4403-01	578	0	10.733	9.7422	4.7767	9.9012	11.001	5.6418	5.2886	0.6378	9.2621	0.6378	9.5523	9.9088	10.4172	8.009	7.3883	5.9319	9.4378	6.2856	9.4363	10.3942	8.8191	8.2375	5.1143	8.4877	8.6019	8.8714	8.1695	8.5811	8.9293	9.3557	5.9319	14.9868	8.7274	9.6368	7.4214	13.5336	12.4123	11.9866	8.7922	8.8602	4.5727	2.7137	6.1566
TCGA-05-4405-01	610	0	9.5089	10.6515	3.8332	11.1272	12.93	4.2894	6.2202	3.3613	12.506	6.1812	8.542	6.3548	10.2978	7.2736	7.5175	3.8332	8.5291	6.551	8.3183	10.7217	8.6411	8.5875	3.8987	8.5322	4.5152	7.9969	8.3147	8.9091	11.8605	9.5605	5.8243	14.2639	5.4833	10.0614	6.4007	12.4053	10.8747	12.2196	8.2565	6.6109	4.5965	0	5.7011
TCGA-05-4410-01	0	0	10.287	9.0479	4.026	10.8045	9.7942	4.5811	3.726	3.4514	12.0995	0.8193	9.1774	6.7034	9.0624	8.4591	8.1374	6.3716	9.1697	7.6087	8.8408	10.6735	8.942	9.6899	5.1458	8.1008	7.4025	9.1031	9.7729	8.4242	10.5844	9.6131	7.5744	13.7307	4.7949	9.9007	5.4021	12.1093	11.0019	11.7391	8.1646	7.3762	2.2698	0	6.9373
TCGA-05-4415-01	91	1	4.9645	5.0444	4.0875	10.5345	13.17	15.5335	4.2647	0.9175	7.8138	1.2224	9.3685	4.4811	8.6778	6.6196	6.6518	8.0691	7.9548	8.0762	10.017	8.4249	9.4624	9.1769	4.7901	8.8798	7.3741	8.2548	8.1091	7.5314	10.4226	8.1991	3.1699	11.4291	5.3576	9.7866	1.4739	11.2406	10.2993	10.4933	7.6188	7.9523	3.6503	6.127	5.8881
TCGA-05-4417-01	455	0	9.783	8.7001	2.8785	9.6329	12.472	13.7281	6.3521	1.8244	11.9223	0.7096	9.5967	4.6882	9.4995	4.9178	7.6792	6.3854	8.8307	6.8016	8.6012	10.0475	8.7981	7.8676	4.1309	8.0945	5.4867	8.4079	7.8237	8.2301	10.8982	9.8979	5.6417	13.8259	5.7648	10.2783	4.5773	11.9097	10.9149	11.4018	8.3303	7.8398	6.5991	1.1832	6.3854
TCGA-05-4418-01	274	1	9.8058	8.4235	3.8035	9.5115	13.574	15.0484	5.477	0	10.1599	2.3186	9.7706	8.0384	11.3269	5.1658	5.8372	5.0109	9.0859	7.3738	9.3812	8.7596	10.1349	7.7529	5.1923	9.3153	6.9731	8.8774	7.6397	8.275	9.847	9.5491	6.3709	13.9229	6.4443	10.5988	6.7192	13.4189	11.4749	12.0767	9.1337	9.3192	2.935	6.4498	6.4388
TCGA-05-4420-01	912	0	10.958	7.4605	4.5775	10.76	13.828	4.0909	9.2332	1.2109	8.3991	0.8391	10.6519	8.7731	10.6064	9.6563	7.7833	7.2498	9.0944	6.5233	8.8398	9.8965	10.4742	9.13	5.4198	8.9087	7.665	9.4076	7.9613	8.2966	10.9436	10.1097	7.0695	13.1533	5.942	10.0418	3.975	13.4354	10.4678	13.0646	8.8491	8.7792	4.317	1.2109	7.6499
TCGA-05-4422-01	365	0	10.057	11.7415	7.3456	10.6668	11.989	13.9376	3.58	0	9.1164	0.3834	9.3362	7.3156	9.9113	9.6084	6.9505	5.6176	9.7074	7.367	9.1004	8.9	8.8418	9.1337	7.4472	7.7733	8.2831	9.4174	7.102	8.8235	10.0096	7.4991	8.5229	13.4454	8.9055	9.7569	3.7199	12.3761	11.6873	11.2813	8.1884	8.4266	3.8166	2.1206	7.4796
TCGA-05-4424-01	913	0	9.6943	10.9101	4.3605	9.2709	13.503	5.342	6.3241	0	10.5645	0.9659	9.2798	6.8081	9.2643	8.3554	7.8321	7.0812	8.7059	7.3867	9.2207	10.149	8.9396	8.419	4.1443	8.3295	6.6798	8.3679	8.0081	8.681	10.0045	9.5586	5.827	14.5026	5.1996	9.7424	7.9702	11.9442	12.3823	11.0984	8.9027	9.1623	6.0613	1.5393	3.7938
TCGA-05-4425-01	669	0	10.459	7.3512	4.4023	10.3038	14.532	4.3238	5.9799	1.0835	12.9014	8.2756	9.7274	9.2876	10.8329	8.4027	5.5163	5.941	8.2008	7.5279	8.9259	10.9202	9.1236	8.9998	5.224	9.2033	4.0098	8.5435	8.7638	8.537	10.0662	10.9013	8.4334	13.9655	6.5356	10.0007	5.8875	12.5503	10.1517	11.7287	9.0448	8.5018	1.4216	0.6412	6.6686
TCGA-05-4426-01	791	0	10.023	7.615	4.6444	10.5452	15.992	3.9286	5.6999	0.6716	11.1377	0.3745	8.9568	5.5799	9.872	8.5948	5.2722	6.4174	8.3363	8.7044	9.3517	11.0213	9.0613	9.5185	5.9625	8.5404	4.5379	8.8744	8.2768	8.5747	9.0613	9.9792	8.5848	13.7552	6.1548	9.7402	4.2315	13.5008	13.319	12.5052	8.416	8.6692	4.6614	0	6.1548
TCGA-05-4427-01	791	0	10.216	8.3706	3.0114	9.4704	10.201	4.2218	6.3616	0.5893	10.3607	0.5893	9.6766	1.0065	8.9572	6.3704	6.8267	5.8708	8.9889	6.5521	8.1363	9.8819	8.6766	9.5857	4.8199	8.4642	2.9182	9.3236	5.3165	8.1031	10.2556	8.5168	6.5903	12.6067	5.7677	8.6166	5.2221	11.1065	10.9581	11.3442	6.6853	6.7272	4.2218	1.3298	4.0567
TCGA-05-4430-01	761	0	10.256	10.9445	4.4629	12.3795	10.649	5.5525	6.3353	0.3994	10.3915	4.1624	9.5665	7.3263	9.4946	9.9768	7.1268	6.032	8.7278	6.9105	8.9699	10.1379	9.5439	7.6602	3.8793	8.5199	7.5117	8.1671	8.8869	8.4418	10.2154	10.3572	6.8156	13.1243	6.4867	9.7594	6.3967	12.9735	11.9943	12.5483	8.5103	8.1066	5.5623	0	6.6376
TCGA-05-4432-01	761	0	10.39	9.5126	3.9999	10.7083	13.505	7.9299	10.4939	1.7257	10.122	1.5941	10.161	7.4387	9.9826	11.798	7.6789	6.7627	9.3346	6.7665	8.3546	8.9363	9.7374	7.7011	4.7215	8.6354	5.6858	9.593	6.7589	8.2279	9.4173	9.2088	6.8804	13.758	6.1976	9.948	5.8097	11.6829	11.3329	11.573	8.9132	7.4193	4.239	0.3656	5.9031
TCGA-05-4433-01	730	0	10.506	10.9309	4.1317	8.9004	11.59	4.062	4.3225	0.869	9.4852	0	9.3197	8.6971	10.0026	7.8294	7.8605	6.5731	10.3102	6.6102	8.82	9.8507	8.6475	9.0609	5.0899	8.0282	6.2041	8.3946	6.4421	7.1614	9.2723	8.6083	4.6419	13.9084	8.3875	9.4634	8.1563	12.1123	11.9229	10.6618	8.533	8.6534	4.2617	1.4076	6.4421
TCGA-05-4434-01	457	1	10.381	11.488	4.8256	10.6842	13.024	4.575	6.0518	0.2941	10.8092	0.9293	9.5183	8.0831	9.8492	8.844	7.6485	8.4205	9.1429	7.2863	9.1662	9.5991	9.3846	7.755	4.9469	8.4587	6.6829	8.9119	7.9456	8.2963	9.8796	9.6451	5.4728	14.0708	6.4676	9.8202	5.4431	12.6871	12.2248	11.1658	8.325	8.9467	3.511	0.9293	7.1157
TCGA-05-5420-01	457	0	8.3243	8.2541	4.432	6.9073	15.091	14.6833	4.3071	0	11.6574	1.4803	10.9719	5.9086	10.0315	8.7681	7.0111	6.3013	7.3328	7.5687	9.7533	9.8763	10.2913	8.2414	5.2017	10.0796	5.7255	9.5038	7.7476	9.0375	10.1564	9.0375	6.6359	14.457	8.3679	10.2227	4.7525	13.9896	11.6773	12.2984	8.8233	8.8403	4.3709	0.9222	7.4333
TCGA-05-5423-01	151	0	9.686	9.0754	3.4927	9.9002	13.643	3.5836	3.2915	0	10.0282	3.0576	10.5744	4.8499	10.3007	9.0031	7.2883	6.4624	9.114	7.5644	9.1784	9.9134	9.5102	8.2142	3.3956	10.1788	7.1685	9.3655	8.4888	8.8373	11.454	7.8243	9.6457	14.0849	6.3109	9.3149	8.189	14.1129	12.1597	12.4635	7.8289	8.8046	4.1578	0.7929	7.0853
TCGA-05-5425-01	882	0	9.2743	9.5527	6.5596	11.8516	12.844	15.5903	5.617	0.6467	10.2882	0.6467	9.8061	6.8344	10.2448	9.8483	6.9577	6.4141	8.753	7.3911	8.5766	10.1143	10.0177	8.1885	3.1568	8.5321	7.5649	8.0005	8.8499	9.139	10.3115	9.2261	7.8601	14.5156	6.6517	9.9392	8.9556	12.8972	12.1015	12.108	8.105	7.2155	4.2976	1.9366	7.306
TCGA-05-5428-01	670	0	9.9498	12.3844	3.8156	10.827	15.225	4.2048	4.2048	3.9841	12.6521	3.281	10.6188	3.8156	10.1527	8.486	8.4824	6.8915	8.2419	7.8585	8.8852	10.0096	11.7912	8.3576	6.1089	10.1083	3.4049	8.2082	9.2079	8.885	10.3074	7.6675	8.1825	12.4254	6.0341	10.2099	3.4049	14.7607	11.562	13.0003	9.022	7.9222	2.9957	2.8287	8.8743
TCGA-05-5429-01	275	1	10.977	9.1612	3.249	9.8964	10.03	3.6555	4.6864	0	8.1461	3.8993	10.2922	9.7051	10.1876	7.8205	6.8988	5.7017	9.2573	7.0241	9.3527	9.5839	9.6155	9.6944	4.8109	10.3368	9.5032	8.3885	7.7236	8.444	9.5426	7.9068	4.1711	12.2973	9.2592	9.8905	9.1495	13.0663	9.5737	12.0824	9.211	9.1357	4.9255	0	5.162
TCGA-05-5715-01	62	0	10.867	9.9887	1.7409	10.4745	11.85	4.6102	3.5767	0.8325	10.993	4.5149	9.1671	9.3387	10.4538	9.5014	5.7364	5.5566	9.0273	7.2889	9.2787	10.0259	8.9678	7.5395	5.2949	7.9748	7.1998	8.1036	9.1827	8.7985	10.509	9.103	5.7151	15.2624	5.7985	10.2423	7.215	12.7772	10.8075	11.762	7.7686	8.3651	5.0449	0	4.9016
TCGA-35-3615-01	14	0	9.9658	13.487	3.9653	11.6131	8.8742	3.3815	5.1604	0	9.6171	3.3815	9.1438	3.2886	10.7214	6.1132	5.8299	7.6933	8.9527	7.8086	9.3178	9.1254	9.5826	7.9037	5.7274	9.4458	5.7096	9.0846	9.3286	9.166	9.9417	8.4775	9.6431	13.4386	7.4144	10.3914	5.0656	13.1563	13.9538	13.2126	6.3557	9.7123	4.6832	0.4059	7.7683
TCGA-35-4122-01	225	0	9.9036	9.6015	4.5305	10.514	11.086	6.408	6.908	0.5874	10.8393	0	11.3167	5.007	9.609	8.7385	7.9643	7.1921	7.9526	6.425	8.4734	10.5894	11.0051	9.1442	5.4989	9.6839	6.0624	8.487	7.1721	8.7042	9.556	8.848	6.167	14.1216	6.2739	9.8346	7.8652	14.2109	12.5138	12.3997	7.8714	9.1416	4.2548	0	7.3691
TCGA-35-4123-01	182	0	9.7295	7.5175	3.7797	10.9167	7.7291	4.1445	6.5827	0	11.4012	4.968	10.5471	7.7022	9.1216	7.7763	8.101	5.0468	7.753	6.7743	9.6606	10.3726	10.2121	8.4334	4.7733	9.8224	5.6616	9.2957	6.6599	8.2013	10.1577	8.8602	6.9382	12.6302	6.1098	9.3922	4.6271	13.6103	12.4626	12.8025	7.7821	8.646	3.9533	0	8.8818
TCGA-35-5375-01	264	0	8.0446	16.398	3.7234	9.8066	15.202	3.8398	6.2569	0	9.3933	5.5045	10.2834	8.9282	10.1678	6.7783	7.6653	7.2474	6.2981	7.3093	11.1924	8.0202	11.4375	8.4307	5.6066	10.1146	2.9372	9.1445	9.1973	9.282	10.8324	9.1217	9.4379	13.8661	5.2756	10.1712	1.0772	12.9714	10.7733	12.2161	10.1892	9.9085	4.7885	0	5.1905
TCGA-38-4625-01	2973	0	9.4283	9.1827	2.5329	8.1977	6.9106	6.6975	3.8314	1.2679	10.8328	0.3579	9.6985	3.8874	10.5322	8.7657	6.9905	5.2002	7.8399	7.6925	8.6947	11.7951	9.6902	8.8403	5.0116	8.8573	7.4139	8.9061	8.7431	9.7761	11.0495	9.0067	6.0018	13.3251	6.5622	9.7295	5.2964	13.4188	11.6007	13.0858	8.0461	8.569	3.8874	0	6.6738
TCGA-38-4626-01	3674	0	9.8319	11.1454	6.7398	10.4885	11.957	6.0229	6.385	3.0425	12.2256	1.2372	8.7891	5.5242	9.6385	7.8295	6.9629	7.133	9.0765	7.5361	9.5972	11.1249	8.4412	8.4497	5.481	8.4494	5.5523	8.6335	7.9479	8.7126	9.5946	9.7341	6.9837	15.3235	7.1469	9.8048	5.3431	12.7766	13.517	11.306	9.0059	8.47	6.0279	1.2372	5.7472
TCGA-38-4627-01	1147	1	9.2602	11.1902	3.6639	9.8541	13.517	6.7825	7.7926	0.7905	11.9354	7.8756	9.3685	6.3574	9.0896	6.9493	6.2088	6.6525	8.3587	7.5917	9.5702	10.625	9.0126	8.0878	5.1414	8.7007	6.1283	7.8756	8.2608	8.7532	9.5373	10.92	7.4484	14.3706	6.3315	10.037	5.7522	13.1724	13.1224	11.8721	8.5107	9.3984	6.2159	0	5.819

TCGA-38-4628-01	1492	1	10.499	9.9242	4.7334	12.5705	9.7507	3.3898	4.5509	0.3963	10.2896	6.9087	10.2583	4.2963	10.1095	9.5892	7.6673	3.8031	9.2676	6.5582	9.715	9.6495	9.3552	9.0126
TCGA-38-4629-01	864	1	10.441	8.6319	4.7867	11.4908	9.1096	5.669	6.7831	0.6822	10.0799	1.1435	10.6686	3.0972	9.9446	8.301	4.9079	7.422	9.5066	8.0278	9.4408	10.3165	10.3237	7.4818
TCGA-38-4630-01	1073	1	9.0228	6.4608	3.3962	11.829	12.152	6.1044	7.9826	1.4713	7.8967	4.1749	9.7635	8.2777	9.474	8.5695	7.1557	6.0183	9.1627	7.5203	9.7786	7.189	9.8701	9.2928
TCGA-38-4631-01	354	1	10.039	11.896	5.9772	11.7104	15.178	8.3024	9.2283	0	8.1433	2.8636	11.2326	6.1625	10.0284	9.0041	9.0709	5.1187	8.9974	7.6519	8.4349	7.3596	10.3423	8.9729
TCGA-38-4632-01	1357	1	9.4698	7.8951	3.5491	10.4996	10.265	4.436	4.0924	1.7198	10.2883	0	10.4421	4.7548	10.5209	8.2324	7.4414	6.4871	7.702	7.2077	8.9534	10.091	11.032	8.1219
TCGA-38-6178-01	448	0	9.0831	9.3286	3.1376	11.2905	14.166	4.4634	6.1851	0	10.6869	2.6916	8.4817	6.4135	10.4406	8.8097	6.784	4.9002	9.7308	7.762	8.8548	11.0141	8.3044	8.3361
TCGA-38-7271-01	800	1	9.6639	10.8843	3.8745	10.6917	11.064	5.1362	5.2268	0.815	12.6319	1.3326	10.3026	6.0674	9.5949	8.4802	7.3597	6.2937	8.9254	6.9365	9.076	10.5506	8.6008	8.0219
TCGA-38-A44F-01	133	0	10.415	11.401	3.1685	10.5762	13.614	4.6423	5.9691	0.6515	9.9373	1.0987	9.1935	8.4758	10.2057	7.2718	7.5779	6.6068	9.461	6.9575	9.8855	10.279	8.623	8.0263
TCGA-44-2655-01	1324	0	10.493	9.3454	3.4724	11.4869	10.308	4.7632	4.8946	0.3323	10.6383	0	9.1787	5.5173	10.4849	9.2238	6.9513	6.8317	8.918	7.9117	10.1265	8.8427	9.0451	8.4104
TCGA-44-2656-01	1429	0	9.988	9.6745	3.147	9.9048	13.181	4.0864	5.0433	4.6191	11.1931	1.2356	9.4255	3.3523	10.0667	7.1349	7.3986	7.1011	8.7018	7.157	8.8865	10.4958	8.6037	7.8282
TCGA-44-2657-01	1351	0	9.8207	10.3944	5.8424	10.3814	11.319	4.4903	5.8082	0	10.7853	2.1229	10.1419	8.9374	10.4584	8.2174	7.6844	5.9244	9.3353	7.2919	9.2668	9.7729	9.1103	7.7578
TCGA-44-2659-01	1367	0	10.561	9.4817	2.4794	10.8032	10.203	4.9424	5.3534	8.0091	11.4303	1.3975	8.8878	3.6371	9.6005	6.537	6.6864	6.2075	9.6098	7.4478	9.1564	10.1029	8.3743	8.8164
TCGA-44-2661-01	1159	0	10.163	10.5605	4.8981	9.3095	12.648	5.4028	5.4028	0	10.795	3.8041	10.5673	7.3895	10.0566	9.303	7.5826	6.0514	8.3516	6.9255	9.3431	10.5152	8.9123	7.9195
TCGA-44-2662-01	1280	0	10.33	7.7934	4.0704	8.6294	14.958	5.8243	5.8781	0	11.6425	1.4831	9.7649	1.4831	9.1548	7.9143	7.9977	7.2496	9.4538	7.5162	8.0312	11.3019	9.5877	6.8613
TCGA-44-2665-01	1301	0	9.5554	10.2872	4.0984	7.8815	13.926	5.9587	6.5973	0	10.7084	8.9796	9.363	4.1761	9.743	6.6528	6.9471	6.4948	8.4341	7.8873	8.5756	10.3462	8.6908	7.6558
TCGA-44-2666-01	97	1	10.681	9.6228	4.1779	11.0023	12.577	3.2236	5.0493	2.4822	11.9548	7.8162	8.8745	8.237	10.1941	8.1738	8.9237	4.422	8.6916	8.0107	9.2232	10.7225	8.617	8.886
TCGA-44-2668-01	761	1	9.557	8.2658	3.0173	11.2103	13.322	4.7801	5.8726	1.7893	11.2139	0	9.5042	2.4957	9.9023	8.455	7.4113	7.2317	9.3968	7.3809	8.9118	11.0307	9.3933	9.0922
TCGA-44-3396-01	1130	0	9.9103	9.0227	4.7405	11.322	11.692	7.9121	5.8815	0.9223	11.6428	2.1232	9.6696	3.409	9.9974	8.7829	7.6173	6.9654	8.667	7.3762	9.0628	10.3487	9.658	8.365
TCGA-44-3398-01	1163	0	9.466	11.4911	4.5404	8.8498	13.565	5.899	6.1015	0.8926	11.1471	5.6875	10.6455	5.3962	9.2881	6.0925	7.109	5.0682	8.0488	6.7558	9.7573	10.8737	9.5729	7.7831
TCGA-44-3918-01	1036	0	9.9787	9.3555	2.9754	9.0745	13.032	6.4835	7.1431	5.127	11.9646	5.9369	10.0711	7.6026	10.1084	8.0802	8.6032	6.9296	8.003	6.9114	9.4058	10.5025	8.7368	8.3091
TCGA-44-3919-01	1026	1	10.408	9.7846	1.3929	10.3463	14.728	5.9724	5.067	0	10.4863	1.3929	9.7354	7.2623	9.6916	8.1804	7.5294	6.0093	8.843	6.3047	9.0044	10.7157	9.3851	8.7793
TCGA-44-4112-01	808	1	10.431	8.7613	2.8123	11.3772	11.785	3.8733	6.1796	13.48	13.4703	3.9503	10.5114	7.0214	10.4654	7.6768	7.7805	4.0233	9.203	6.1554	8.5623	10.2155	9.3514	8.1501
TCGA-44-5643-01	1013	0	9.4352	6.0884	2.3056	9.107	8.5645	5.6764	5.4158	1.0887	8.7186	7.8509	9.5866	7.6631	9.0546	9.3716	8.7378	5.508	8.2696	7.0596	9.7137	6.8288	9.6262	10.5141
TCGA-44-5644-01	863	0	9.654	6.3945	1.5826	10.3337	13.106	10.4745	6.173	0	8.0206	2.4995	9.3751	6.8081	9.5079	9.0971	7.9409	6.1863	8.1713	6.3715	8.9052	6.7822	9.7463	7.5536
TCGA-44-5645-01	852	0	10.553	9.0372	3.1751	8.7678	13.908	3.5017	5.4415	1.4442	10.589	1.1026	9.5268	6.2725	9.1455	8.1548	7.0672	6.8332	8.8827	7.9357	8.1178	10.2289	7.9221	8.301
TCGA-44-6145-01	595	0	9.7238	13.8603	3.9569	8.6241	15.377	7.4645	5.3726	0	10.8012	0.7064	9.1376	4.0145	9.0902	7.0827	5.9459	6.7	8.255	6.9997	8.2928	10.8792	8.5012	8.4263
TCGA-44-6146-01	728	0	10.893	9.4278	2.7796	8.8138	9.3774	4.3185	3.7693	0	7.9079	0	8.6338	8.9184	10.3911	9.0823	8.8095	6.0141	8.6916	6.7662	8.3968	9.2585	7.7566	8.7104
TCGA-44-6147-01	845	0	10.793	10.5236	3.8739	12.9259	12.312	6.666	6.1375	1.0655	10.6279	4.905	8.956	9.0937	10.6344	8.7572	7.5828	5.9715	8.4919	7.8958	9.187	10.121	7.9668	8.3101
TCGA-44-6148-01	704	0	11.084	10.5827	4.138	11.5955	11.264	6.3173	7.2177	2.5413	6.8668	2.6683	8.7194	9.1105	8.8809	7.5252	8.006	6.1204	8.3938	7.1968	9.4125	10.5578	7.9539	8.65
TCGA-44-6774-01	658	0	8.9086	9.6593	4.3587	9.6921	12.282	5.9184	6.5819	0.9482	12.6019	6.2532	8.9444	5.1451	9.5811	8.3125	7.3868	5.9404	8.3541	7.7086	8.5751	9.2209	8.7401	7.1142
TCGA-44-6775-01	705	0	10.594	10.3675	4.1829	9.9745	13.723	4.8206	6.2133	1.0069	10.6545	8.3602	9.9939	6.0904	9.3585	8.6522	7.6612	6.5214	8.9925	6.8145	8.8452	10.4247	8.8608	7.8748
TCGA-44-6776-01	2616	0	10.595	8.8252	3.0328	12.0287	9.2197	6.7458	4.4002	0.7811	9.1602	0	8.1717	8.0082	10.9197	7.6386	6.7554	5.5316	9.5908	6.8662	9.5742	8.606	8.5601	7.4966
TCGA-44-6777-01	987	1	9.1803	10.0512	4.852	8.8363	13.691	5.7723	6.9975	1.0371	12.4781	3.9754	9.5285	5.1586	9.68	7.5647	7.5846	7.6691	8.328	6.7868	8.8786	11.1196	8.0673	8.1119
TCGA-44-6778-01	1864	0	9.1029	11.3411	4.3695	12.0664	9.1987	9.9132	8.1039	1.1821	11.0698	1.1821	10.0999	4.8539	9.2917	8.0357	8.5226	6.2212	7.9752	7.3858	8.4217	10.562	8.8527	8.1529
TCGA-44-6779-01	500	1	10.014	4.493	3.0308	7.6684	15.494	3.2879	4.0823	0	10.4705	0	8.9906	2.7178	9.8812	7.4687	5.1726	7.587	7.8083	6.6528	8.9483	11.6874	9.3769	5.2948
TCGA-44-7659-01	691	0	11.357	9.8211	3.3174	11.1144	10.92	3.083	3.926	6.3526	12.3859	3.205	8.1358	6.7532	9.3685	7.593	6.2142	4.8406	10.1165	8.6115	9.2476	10.0881	8.3916	8.617
TCGA-44-7660-01	592	0	8.7095	15.1096	4.3762	11.022	14.207	3.5771	6.5342	1.4235	8.6891	2.1258	8.8116	9.2356	8.4485	8.937	7.9945	7.546	8.239	7.8256	9.7549	8.1921	10.3886	10.0641
TCGA-44-7661-01	557	1	10.479	9.6481	4.0271	10.3048	10.508	7.3984	6.9728	0.3921	10.8232	0	9.7118	2.9765	9.6325	5.2542	7.8932	6.7976	9.2092	6.9511	8.6871	10.5372	9.1058	8.6055
TCGA-44-7662-01	218	0	9.2827	9.6773	3.9544	12.6355	10.419	5.1549	7.7441	2.2773	10.2007	1.8733	8.976	3.7153	9.6266	8.381	7.0808	4.252	8.8668	7.6473	8.8	10.0123	9.5696	7.8663
TCGA-44-7667-01	1097	0	8.0095	7.0427	3.3677	11.9924	6.8999	8.9917	9.7742	0.9991	7.6565	0	8.7045	0.4146	10.0051	8.4686	6.8891	6.3142	8.9641	7.35	10.3927	8.3097	9.9339	8.0608
TCGA-44-7669-01	574	1	8.9272	11.038	2.3181	10.7048	11.489	14.7092	7.7404	1.7881	10.0824	1.3411	9.5982	3.3933	9.0791	5.2168	7.9535	6.8311	7.7153	7.2498	7.4284	9.2709	9.7223	8.1625
TCGA-44-7670-01	882	0	10.119	14.9746	4.5397	14.0174	12.251	4.9679	6.3897	3.2245	9.2187	0.6945	9.5873	6.721	9.638	9.7484	7.4693	7.1588	8.3193	7.7683	10.0445	7.7622	10.3952	9.0044
TCGA-44-7671-01	889	0	10.558	15.1825	2.5176	12.4749	11.814	13.7009	5.2428	0	9.4496	3.3858	8.238	5.905	11.2927	8.778	6.3516	6.4222	9.2617	7.9266	8.7418	8.9236	8.7975	8.5855
TCGA-44-7672-01	719	0	9.8273	10.2638	4.5872	10.9648	12.19	5.1756	6.3126	1.5619	11.5892	2.4047	9.4822	6.1155	9.7398	6.6065	7.249	6.3126	9.3802	7.2527	8.9172	9.7509	9.1267	7.2228
TCGA-44-8117-01	385	0	10.577	8.8893	0.9009	12.8109	8.5805	5.3018	5.8128	0	10.3842	2.4918	9.1242	9.3319	9.2969	8.9077	8.1988	3.4747	8.4567	7.188	8.6481	10.3663	8.2599	6.3359
TCGA-44-8119-01	285	0	9.9582	8.1645	3.1524	8.362	15.142	8.7062	9.3878	0.8392	11.1979	5.5556	9.8509	4.9657	10.4773	9.34	7.9526	4.771	8.6619	7.2774	7.7462	10.1647	8.6277	5.7444
TCGA-44-8120-01	260	0	10.009	10.2766	2.7839	11.1378	7.5616	5.8275	5.5963	4.3663	10.4589	1.8168	8.7796	2.8968	10.9416	7.9816	6.6106	7.0648	9.6857	7.6176	9.1833	9.2141	9.1162	9.2263
TCGA-44-A479-01	486	0	8.4792	9.1161	2.9654	5.5004	12.916	8.7823	9.2566	0	11.8297	2.47	9.6714	7.8508	9.2343	8.8741	8.2907	7.3911	8.7598	7.316	8.1541	10.1412	8.7692	8.5789
TCGA-44-A47A-01	466	0	9.6962	7.7715	4.2412	8.9775	13.226	4.8397	4.2797	1.6074	9.9539	1.0169	8.8425	8.2478	9.9033	9.7984	6.3641	5.9755	8.7077	8.1315	9.2628	9.3327	9.1196	7.2622
TCGA-44-A47B-01	287	0	10.198	6.7433	3.9799	11.4888	9.9884	8.5505	7.2849	0.5339	9.6053	0	9.5486	7.8723	10.4319	9.4724	8.2057	5.9096	8.2594	6.0032	8.5302	10.1167	8.3922	7.1952
TCGA-44-A47G-01	351	0	9.8371	9.7994	5.0032	10.4965	13.99	7.6934	5.3264	1.1048	11.4757	1.1048	9.5083	4.9233	10.3795	8.069	7.0464	6.7704	9.7569	6.6097	9.2107	9.9514	9.1852	9.3392
TCGA-44-A4SS-01	415	0	10.38	13.582	2.9347	10.5674	13.766	4.9499	5.5192	0	11.3221	2.4412	9.3845	2.286	9.827	9.1029	7.8782	7.6466	10.1096	7.1006	8.278	9.2026	9.0631	8.9187
TCGA-44-A4SU-01	409	1	9.7144	10.9418	2.73	10.4835	14.578	4.2714	5.2691	0.5553	9.7898	6.6584	8.5224	7.4721	10.3619	7.4759	7.7042	6.345	10.5743	7.8581	10.1177	10.2257	9.1926	8.0543
TCGA-49-4486-01	2318	1	10.391	15.0287	2.9354	12.7089	11.749	2.9354	2.7339	0.4141	10.3739	0	8.1205	7.0553	12.336	7.3829	5.3773	3.7692	8.4351	8.1596	9.7818	8.0334	9.2763	8.965
TCGA-49-4487-01	855	1	8.7728	9.2941	3.36	12.4965	13.01	14.0597	5.7112	0.8817	11.5419	0	9.5409	6.131	8.7336	7.8829	7.3163	5.2821	8.0297	6.6376	9.3898	8.9723	8.3754	7.9484
TCGA-49-4488-01	869	1	9.8702	8.0745	2.7804	11.5676	14.362	3.2936	3.6714	0.7939	10.2995	2.7804	9.1335	6.5119	10.9247	8.2517	6.7953	7.0321	8.4734	8.1658	10.3417	8.7417	9.7839	8.7904
TCGA-49-4490-01	385	1	9.6597	9.619	6.2097	11.9381	12.951	4.3686	5.5214	0	9.8991	4.3017	8.1765	8.9272	10.8043	7.6466	6.1539	4.2315	9.444	7.8709	9.5075	9.8057	9.229	8.2359
TCGA-49-4494-01	1081	1	10.444	7.8249	3.3744	11.2886	14.49	2.3148	4.2176	0	10.2523	0	9.6243	7.5402	9.6602	7.9577	7.2512	7.5836	8.7645	7.1964	9.5292	10.6674	10.4796	7.764
TCGA-49-4501-01	1421	1	9.9066	10.286	4.7059	11.7699	9.7546	5.0885	5.5778	0.3382	11.0816	3.07	10.1113	7.6377	10.5183	9.5426	7.1613	6.8604	9.2896	7.6454	9.4383	10.054	9.3811	8.914
TCGA-49-4505-01	428	1	9.8853	9.0081	5.239	9.1396	15.064	4.8189	5.3425	0.3569	10.8406	2.0311	9.0817	6.1302	10.349	8.0023	6.54	7.1487	9.317	8.425	9.5942	10.9079	9.7068	8.5393
TCGA-49-4506-01	999	1	8.7994	7.2614	3.5043	9.5862	13.087	14.9011	7.3164	0	10.6171	1.5801	9.4639	6.3816	11.7584	6.902	5.1385	7.32	8.2435	7.9293	9.4455	8.8488	10.6967	6.3326
TCGA-49-4507-01	268	1	9.2965	16.242	5.2084	8.421	15.799	14.2403	5.1674	0.4286	9.1583	0	9.4006	4.2986	11.01	7.4905	6.1259	8.421	9.0636	7.7208	9.7278	9.9242	11.5303	8.0663
TCGA-49-4510-01	896	1	10.377	10.9219	3.6432	12.2485	13.195	13.8517	9.7645	2.821	8.251	0.7125	9.0869	7.4592	11.1255	7.2206	6.8652	7.4086	8.621	6.9864	8.2721	8.0639	9.9626	8.4681
TCGA-49-4512-01	905	1	9.7069	10.7767	4.762	11.7904	8.4671	7.2001	5.3565	1.3433	10.2372	7.4678	8.2177	5.7963	11.2642	8.4125	7.0741	5.4096	9.2134	7.8018	9.7007	9.1028	9.1347	7.6164
TCGA-49-4514-01	1700	0	10.363	15.4234	6.8164	12.8619	9.918	14.0592	4.5821	0	9.774	0	9.2363	7.5285	11.3713	10.3563	5.5751	7.201	10.0298	8.5365	9.1197	7.4549	10.3755	8.4253
TCGA-49-6742-01	488	1	9.6553	10.0908	7.527	11.2431	13.919	13.736	5.7534	1.6947	9.1338	0	9.099	8.924	10.1947	8.6028	5.9402	5.2014	8.7894	7.5444	9.5369	8.4695	9.8945	7.2158
TCGA-49-6743-01	1621	0	9.3174	12.7138	3.6316	11.8201	15.476	5.4903	7.5561	1.0602	10.9325	4.5389	9.2611	7.2204	8.8778	8.3246	7.9312	3.6316	7.7827	7.1724	8.7797	10.3412	9.417	9.0763
TCGA-49-6744-01	1683	0	9.9753	10.3898	2.4809	10.2405	14.406	5.0698	6.4268	2.3429	11.2833	4.1947	9.1056	7.0649	9.356	8.5998	7.9026	6.1239	8.9103	7.7608	9.2817	10.5145	8.2404	8.4545
TCGA-49-6745-01	522	0	10.69	9.6261	2.8852	10.4293	9.229	4.3338	5.6337	0	10.6242	4.8087	9.4242	4.1463	8.7435	8.2488	7.4386	6.0306	8.2811	7.4222	9.0799	10.421	8.7936	9.5998
TCGA-49-6761-01	354	0	9.8243	7.5713	2.6166	11.2277	7.3656	3.2927	4.6966	0.7935	10.5548	0	8.971	6.4637	10.9191	7.0472	7.2061	7.867	9.1715	6.5224	8.5718	8.9626	9.5586	8.5896
TCGA-49-6767-01	677	0	10.511	7.2525	3.3039	11.5035	5.9686	3.018	4.9348	1.4725	8.8274	5.0423	9.7702	3.018	9.4077	8.2561	6.5069	6.7952	9.3113	7.0739	10.1067	8.0299	9.41	8.5954
TCGA-49-AAQV-01	677	1	9.84	8.2264	2.9798	10.3278	14.009	5.3924	5.2629	0	8.6797	1.9827	8.6493	7.7407	11.085	9.4814	7.1994	5.5113	10.2013	8.5161	9.2697	9.8061	9.5625	7.2531
TCGA-49-AAR0-01	4765	0	9.4472	15.0502	3.9428	13.2878	13.038	5.6745	5.31	2.1604	9.5877	1.3146	8.3388	7.2686	10.567	8.1376	7.5231	6.7294	9.7419	8.3607	9.8323	8.8917	10.4193	7.8735
TCGA-49-AAR2-01	2224	0	9.3402	8.5036	5.7604	9.9634	9.2785	15.6993	7.0258	1.514	8.213	1.1618	8.1918	10.7085	11.7772	9.174	7.2239	7.5051	10.2117	8.3911	9.9508	6.4933	10.5171	8.4096
TCGA-49-AAR3-01	1893	0	9.5068	10.8617	3.5177	11.2988	11.636	4.06	6.373	0	9.556	1.4553	8.7112	3.9008	9.8842	8.2278	7.9463	7.2778	9.566	8.0304	9.6135	10.0608	9.9213	7.5766
TCGA-49-AAR4-01	879	1	10.209	9.0098	2.7387	11.5594	12.312	5.0784	6.8151	1.177	9.5986	2.2579	8.5887	7.4618	10.4351	6.2244	7.6365	6.1366	10.09	7.298	9.8887	9.988	9.7936	8.5528
TCGA-49-AAR9-01	260	1	10.346	6.5641	5.0297	10.5167	9.3286	2.702	4.9246	0	8.2299	2.9789	9.4708	6.0177	10.4653	6.9326	7.2949	6.8199	9.3308	7.4431	8.6933	7.0964	9.8616	8.5331
TCGA-49-AARE-01	1229	1	9.9803	13.3715	3.0501	9.2613	13.278	3.4612	7.3849	1.2421	10.5048	5.1161	8.2114	9.6328	10.1868	7.4502	6.2237	5.2772	8.5195	7.3611	8.5753	7.1322	9.4459	8.0744
TCGA-49-AARN-01	1135	1	9.6697	8.5125	2.4535	11.3403	13.211	4.0593	6.3766	0	9.9843	4.9119	8.6176	8.3862	10.7751	8.5359	7.0961	5.6512	9.7418	8.0909	9.6602	10.8412	8.9361	8.4887
TCGA-49-AARO-01	3759	0	10.117	10.1077	3.9991	11.1926	13.732	7.323	6.4698	6.7733	10.1819	2.1568	8.7028	7.7043	9.9437	8.3797	7.588	6.1682	9.8373	7.394	9.9009	9.3913	8.7557	7.6432

TCGA-38-4628-01	4.88	9.1755	6.8546	8.7584	8.9467	9.0612	10.9162	9.1263	8.9614	14.8688	5.8473	9.8589	9.5033	13.7969	11.2837	12.7949	8.9309	8.8121	3.3001	1.819	7.2754
TCGA-38-4629-01	4.5088	8.2173	4.4895	9.4259	8.2185	8.5104	9.7864	9.3329	3.7435	14.1478	6.2645	9.958	3.4563	14.0553	11.9566	11.7745	8.1556	8.8432	4.3032	0.3811	7.5532
TCGA-38-4630-01	6.6667	8.8477	6.4716	8.9157	9.0514	9.5361	8.9594	7.4212	5.1703	13.1462	6.1411	10.0091	4.5196	11.9885	9.5839	11.236	8.5879	8.6967	4.2937	1.3511	5.001
TCGA-38-4631-01	2.699	9.0363	9.9314	8.2807	9.1266	8.7305	10.1314	8.1513	6.5605	12.4488	9.3599	10.3406	3.2679	12.447	9.7644	12.0486	8.8556	9.2929	2.2998	1.3615	3.6748
TCGA-38-4632-01	4.3303	9.1602	6.9012	8.9265	8.5651	8.6417	10.3317	9.073	5.789	12.646	7.0418	10.4141	5.1769	14.5279	12.2173	12.9425	9.1928	7.8432	4.1557	0	8.9036
TCGA-38-6178-01	3.834	8.8465	7.0951	8.0351	8.5128	8.4848	10.2547	10.4273	6.0726	14.5492	5.9686	9.5948	5.7971	11.2551	11.4463	11.2426	8.6411	8.1063	4.741	0	6.7635
TCGA-38-7271-01	4.5248	8.0027	5.9833	8.2869	7.6045	8.6643	9.4996	10.7225	6.2937	14.2321	6.2074	9.6933	5.7589	12.3434	12.5628	11.7589	8.7354	7.9484	5.1047	0	5.0059
TCGA-38-A44F-01	6.5726	8.5717	8.2043	8.8736	7.3547	8.4896	9.6866	10.6686	5.9426	13.96	7.8483	9.9306	7.7561	12.8224	12.2422	11.954	9.1878	8.8736	4.1798	0.6515	8.0168
TCGA-44-2655-01	5.5575	8.7869	6.7369	8.3464	8.4998	8.6101	9.4116	9.0042	9.3263	14.4585	7.0017	9.9161	7.1273	13.2595	14.2168	11.6582	8.609	9.9169	4.9799	0.3323	4.4235
TCGA-44-2656-01	4.6813	8.4224	3.5981	8.8267	7.361	8.134	10.5552	8.5199	6.8016	14.7066	6.2795	9.4452	8.1353	12.9383	11.5671	12.1363	7.176	8.6962	4.1317	0.8583	6.3387
TCGA-44-2657-01	5.2188	8.4923	4.9635	8.833	8.8728	8.9551	9.3897	10.4187	7.6702	13.7047	6.7865	10.1488	5.6819	12.485	12.7451	11.8853	8.484	8.5108	4.1521	0.7408	7.5041
TCGA-44-2659-01	4.1698	8.6602	5.432	8.4504	9.3155	8.8164	12.3103	8.6526	6.4587	13.537	5.745	9.6491	5.9798	12.5111	12.3934	11.7738	8.7435	7.3615	5.5271	0.7258	4.4326
TCGA-44-2661-01	6.7108	9.3818	5.1335	8.8655	8.855	9.0957	9.67	10.3095	8.7366	13.963	7.3936	9.8392	7.6871	13.63	12.7079	12.2924	9.0216	9.0375	5.1724	1.2874	7.4892
TCGA-44-2662-01	4.708	8.7127	2.4085	8.0179	6.8524	8.5137	10.4982	9.5426	3.2658	14.4846	6.6609	9.6979	3.2109	13.0979	13.7524	12.1168	7.9584	7.4962	4.2169	0.7809	7.0134
TCGA-44-2665-01	4.7343	9.0582	4.4511	8.528	8.1363	8.5985	9.2246	9.9022	5.0761	13.8617	6.3174	10.2801	6.0442	12.6885	12.5783	11.566	8.3443	7.804	4.655	0	5.8203
TCGA-44-2666-01	6.1846	9.0128	6.4346	8.936	8.6518	8.8382	10.4402	8.3937	8.9974	13.0102	6.5988	9.676	4.6307	11.9921	11.3545	11.5496	7.711	8.696	3.5143	0	7.6937
TCGA-44-2668-01	3.6397	7.4897	4.645	8.933	7.7861	8.6645	9.2284	10.0916	3.951	14.2635	6.0315	10.5033	3.6709	12.3449	12.1858	11.7248	7.7185	8.3149	4.7216	1.7893	5.4635
TCGA-44-3396-01	5.0443	7.9524	1.2281	8.4207	7.2652	8.9625	10.7687	9.8754	6.3654	13.9727	6.9154	9.9133	4.2404	12.5276	10.868	11.5084	8.4075	7.7832	5.8705	0.741	8.2145
TCGA-44-3398-01	5.9196	9.669	4.5136	8.9482	9.4963	8.0371	10.005	8.9913	7.1356	14.5122	6.5203	9.6964	8.8751	13.2364	15.0809	12.0126	8.8404	8.5102	5.6994	1.6514	6.3414
TCGA-44-3918-01	5.6275	8.5045	4.3541	9.26	6.2505	8.1955	9.4018	9.1177	7.4347	14.2145	6.5613	9.4172	3.109	13.1311	11.4264	12.2033	8.6709	8.742	4.4326	2.2672	7.3428
TCGA-44-3919-01	5.9649	8.6443	4.4034	8.5036	6.5346	8.5343	10.0309	10.069	7.6557	14.4361	6.3685	9.6149	4.4685	12.4979	11.9109	11.8356	8.1917	8.3525	4.6097	0.9823	6.2316
TCGA-44-4112-01	6.7418	9.1064	5.7552	8.8099	7.3825	8.7041	9.8364	8.9395	6.1142	14.3053	5.6085	9.6651	2.9685	12.4616	11.4095	12.3379	9.0298	9.9591	3.566	1.9302	8.1727
TCGA-44-5643-01	2.3056	7.4737	1.4277	8.6352	7.5975	9.7004	10.5878	7.4095	9.2593	14.995	6.1469	9.107	3.6168	12.1198	9.1144	10.9604	9.3229	8.4243	2.3056	0.6447	7.5199
TCGA-44-5644-01	2.319	7.6839	7.968	8.2332	5.3063	7.6136	11.012	8.178	8.9689	12.8158	8.4324	10.8854	2.4995	11.3622	9.9207	11.3691	5.8791	7.8892	3.7693	0.7355	5.4227
TCGA-44-5645-01	4.973	9.2434	3.8845	8.6978	6.9109	8.881	10.3029	11.4504	8.5466	14.7793	6.2832	9.0669	4.616	12.1086	11.1342	11.4362	8.6325	7.4826	5.1231	0.6542	6.8186
TCGA-44-6145-01	4.407	8.8381	5.6335	8.322	7.2597	7.6574	9.4096	9.5931	3.4734	14.6118	6.0039	10.1452	5.7225	12.3197	12.91	11.1185	7.6115	7.5251	4.7153	2.5978	6.2149
TCGA-44-6146-01	5.379	8.7549	7.3408	8.1677	8.4242	7.5942	9.2054	8.1472	5.8318	14.8628	8.7457	10.2081	7.3998	11.9124	12.6206	10.0713	8.7623	7.1338	5.5693	0	5.0657
TCGA-44-6147-01	5.031	8.6874	5.9204	9.085	8.0255	8.8126	10.3314	9.831	6.0569	14.4237	6.3457	9.442	5.8262	11.6237	11.585	11.6656	8.3469	8.6592	5.031	1.0655	6.6814
TCGA-44-6148-01	5.8659	8.617	6.0167	8.8049	7.9939	8.7121	8.5971	11.15	8.1813	14.2527	6.6376	9.3812	5.3638	11.9893	11.9528	11.3814	8.8855	8.4764	4.5213	1.0506	7.0175
TCGA-44-6774-01	3.8085	7.872	3.7095	8.5811	7.5169	7.8663	10.1447	10.7352	5.5689	13.4046	6.5102	9.8266	3.0764	11.8659	11.2016	11.2135	8.0696	7.4873	5.2545	0	5.3886
TCGA-44-6775-01	4.5367	9.0564	3.8201	8.4082	7.4188	8.4039	10.8643	10.1887	5.0577	15.0542	6.423	9.6684	7.7107	12.2045	10.5245	11.2676	8.421	7.5606	3.9671	0	5.9908
TCGA-44-6776-01	5.3656	8.5074	7.8238	8.8366	9.0858	8.6272	9.7254	8.8724	8.8207	14.2499	6.4464	9.9485	5.9389	11.3215	12.7209	12.2952	8.0635	8.0163	4.9286	0.7811	7.0017
TCGA-44-6777-01	4.6248	8.2229	4.7985	7.5647	8.1009	8.4502	10.2412	10.66	6.3011	14.9679	5.6416	9.7509	7.7629	12.8549	12.3023	12.2145	8.0673	7.5767	5.5474	0	5.8134
TCGA-44-6778-01	5.2644	8.9732	3.903	8.376	6.5094	8.3312	9.3017	8.6782	6.2455	14.8103	6.3502	9.2088	7.7082	11.7649	13.5184	10.7854	8.8723	7.6364	6.2575	1.1821	4.7892
TCGA-44-6779-01	1.3751	8.4995	1.3751	7.8587	5.9991	7.1824	10.4327	8.823	0	13.595	4.8123	9.6758	3.401	12.6084	10.0939	11.6996	6.8249	9.3313	3.6038	1.3751	5.4074
TCGA-44-7659-01	5.7162	8.6622	5.4708	8.9004	7.995	8.8638	10.0841	9.9268	8.053	13.2886	6.1088	9.327	6.9751	12.0658	10.3442	12.2998	8.8215	6.606	3.7767	2.2439	7.685
TCGA-44-7660-01	5.732	8.9577	4.2222	7.9238	8.1733	8.7268	11.7911	9.2831	8.298	14.8981	6.6107	9.5098	4.5928	12.2463	12.0843	11.5041	10.1311	8.2024	3.0267	7.348	7.2833
TCGA-44-7661-01	4.7507	8.3311	5.2891	8.5869	6.6348	8.4402	10.1309	10.0332	5.0786	13.4827	6.7523	9.7427	7.0965	12.207	13.3395	11.1086	7.2375	7.69	6.3445	0	6.7895
TCGA-44-7662-01	5.2365	7.853	2.5199	8.3182	8.9962	8.44	10.3774	10.889	5.2478	13.6463	6.4597	9.9484	3.2152	12.2255	10.6297	11.8519	7.3373	7.8681	5.2021	1.1269	7.1635
TCGA-44-7667-01	4.9212	7.0058	3.968	8.1598	8.251	8.9032	11.5102	8.6361	8.1138	13.4557	5.3791	9.5858	2.1142	10.7976	8.9525	11.1363	7.9641	7.9679	3.6989	0	5.3906
TCGA-44-7669-01	5.4061	8.4454	3.0636	7.941	6.6358	8.3866	10.3576	8.1961	7.6263	13.8373	6.5534	9.2851	4.928	11.0527	11.3269	10.0857	8.8039	7.7892	5.0263	2.6354	4.5094
TCGA-44-7670-01	3.2245	8.1342	7.4414	7.9123	9.0689	8.1678	9.7857	7.9668	8.4099	13.3636	6.3737	10.3124	5.5753	11.4191	11.5645	12.5096	8.4653	7.8556	4.3559	1.3481	5.2516
TCGA-44-7671-01	5.9789	8.3791	6.7139	7.8597	8.6469	8.8605	10.9349	8.9095	8.0328	14.3191	6.8304	9.9347	3.4702	12.367	13.5992	12.2994	9.2535	9.662	5.9424	1.1765	6.9565
TCGA-44-7672-01	3.9493	7.6496	5.7046	8.7733	8.077	8.7061	9.8041	10.9521	6.4413	13.8714	6.6521	10.19	7.832	12.6321	11.458	11.9748	8.6898	8.3891	4.5872	0.8326	6.5773
TCGA-44-8117-01	5.2805	8.7783	6.7465	8.4084	9.6727	7.9037	10.3653	8.9181	6.4163	11.9662	6.2344	9.1054	5.4522	12.0573	11.8008	13.4864	7.489	7.8809	4.4083	0	6.781
TCGA-44-8119-01	3.9511	7.3904	2.8704	8.4427	6.0778	7.7454	9.3552	10.1504	7.6542	14.0013	6.1166	9.3359	4.4794	10.9776	11.3141	11.3972	7.5648	7.342	4.8256	2.452	5.0711
TCGA-44-8120-01	4.9009	8.8384	7.5759	8.4777	7.3882	8.5333	10.4269	10.0535	6.922	11.8775	6.0756	10.4157	6.0391	12.0685	11.2341	11.8421	8.5593	7.513	5.4374	0.3565	7.2382
TCGA-44-A479-01	5.222	8.8669	7.3415	8.6861	6.9626	8.2854	10.513	9.4943	6.4567	14.0269	7.1644	10.2359	4.8766	11.5055	10.6482	10.4761	8.9473	8.2509	4.8766	2.3141	6.0625
TCGA-44-A47A-01	4.6761	8.6993	4.9632	8.8852	8.402	9.117	10.9389	9.2838	8.72	14.2916	6.6406	10.8417	7.022	12.6773	10.9386	12.2567	8.1984	8.4888	2.936	0	7.8408
TCGA-44-A47B-01	4.1713	8.3606	7.6872	8.7009	8.0265	8.3213	9.8503	8.4817	7.2973	13.6971	6.4648	8.8953	4.9751	12.3718	10.0652	11.921	8.3233	7.9185	4.2413	0	7.4269
TCGA-44-A47G-01	5.9936	8.1069	4.977	8.7605	7.5278	8.645	9.9547	10.0455	6.5131	14.6533	6.6097	9.8721	5.6741	12.7551	12.889	11.6601	8.6029	7.9021	4.1907	0.6557	7.1954
TCGA-44-A4SS-01	3.9958	8.7821	6.4404	7.8646	5.7296	8.2371	10.3249	9.802	3.3806	13.7736	7.2287	10.2984	7.4954	12.9943	11.2501	11.6725	9.8676	7.218	4.8145	1.0757	5.0007
TCGA-44-A4SU-01	6.6381	8.8788	6.0502	9.5907	8.1215	9.2404	10.8683	10.3161	5.2336	14.393	7.1096	10.1626	4.3399	12.0791	9.9059	11.2321	7.6343	9.8694	2.73	0.9554	6.8049
TCGA-49-4486-01	7.3123	8.5335	7.8059	9.2608	9.0865	9.9012	10.5307	7.6711	9.5919	14.599	6.4503	10.1944	4.112	13.2231	13.3081	13.1467	9.1512	10.9641	2.4121	0.4141	7.3772
TCGA-49-4487-01	3.7694	8.0799	6.6005	7.4475	8.2652	8.6481	10.6393	10.4393	5.2182	14.3134	6.5365	9.0683	4.4073	12.0107	11.4159	12.0959	8.6721	8.8327	4.4073	0.8817	5.6641
TCGA-49-4488-01	3.7522	8.4135	8.2552	8.7168	8.6125	8.5741	11.3991	8.2995	7.0562	14.4981	5.6902	9.5915	4.3794	13.1849	11.5012	12.765	8.5	8.7927	2.6175	0.7939	6.717
TCGA-49-4490-01	6.5204	8.3464	8.3228	8.1434	9.525	8.1577	10.6845	9.8749	6.8941	14.2411	5.4917	10.2074	4.4325	12.9156	12.0627	11.4322	8.5773	10.8236	3.9108	0.9532	7.6331
TCGA-49-4494-01	5.8527	8.542	7.7277	8.7767	9.0194	9.2134	11.9139	9.5261	7.8781	14.2231	7.421	10.1326	6.2633	14.3795	10.8738	12.9402	8.1746	11.3479	2.5428	0	6.6777
TCGA-49-4501-01	4.7632	9.0491	5.7787	8.6446	8.3912	8.8666	9.6744	9.2669	8.0638	14.0108	6.3779	9.9407	7.3921	13.1427	12.7707	12.3904	7.75	8.4137	3.9814	0	5.6403
TCGA-49-4505-01	4.8613	8.7889	6.5616	8.6462	8.5811	8.4996	10.9559	9.4521	7.011	14.8695	5.8781	10.3426	6.2252	13.1918	12.0047	12.5621	8.633	9.4807	5.0564	0.6427	5.5206
TCGA-49-4506-01	4.1789	8.3207	3.7798	7.8444	8.3028	8.0981	10.9934	9.178	3.4528	14.9141	7.2688	11.068	9.0817	14.5996	12.5728	13.2114	6.995	10.1773	3.9	4.0806	10.6215
TCGA-49-4507-01	5.0815	8.0862	6.1681	8.7479	8.6282	8.514	10.8934	9.5892	3.9243	12.7023	6.7854	10.7302	5.2872	13.8782	11.6243	13.0216	8.3721	10.6226	2.7081	2.1567	8.1247
TCGA-49-4510-01	6.2546	8.5352	5.9464	8.5367	6.9157	9.092	11.269	9.5782	7.8126	13.8099	7.4644	9.5932	4.3559	13.945	12.9026	12.5502	7.8065	8.4694	5.7719	0	8.3727
TCGA-49-4512-01	4.5417	7.7379	6.5188	9.33	8.5372	9.0886	11.351	10.3211	7.7968	13.0806	6.7933	10.1055	8.0892	13.2993	10.2851	12.756	8.4383	8.7192	2.8378	0	5.7142
TCGA-49-4514-01	4.0932	8.457	9.0941	9.624	11.163	9.1955	11.4038	9.3708	5.7697	13.4155	7.124	11.1656	10.1405	12.8508	12.5561	13.168	9.4753	9.0717	3.1129	0	6.2182
TCGA-49-6742-01	4.72	8.9464	8.5025	8.4573	8.417	7.979	10.5099	8.641	5.9048	14.7579	8.5348	10.7537	7.3272	11.7951	12.1466	11.5612	9.5458	9.5954	5.0473	2.4527	3.516
TCGA-49-6743-01	4.2816	8.7594	2.6839	7.4346	8.7507	8.0123	9.6599	8.651	5.8972	14.664	5.455	10.3346	3.0097	11.9341	11.1657	11.2264	8.1062	8.712	4.9967	0	5.1144
TCGA-49-6744-01	5.9446	8.2624	6.2157	8.6261	8.3458	8.6036	9.7162	10.64	6.6626	14.2528	5.9327	9.703	7.2787	12.4077	12.8068	11.7582	8.8685	7.8302	5.0698	0	4.9322
TCGA-49-6745-01	4.0639	8.0369	3.6208	8.0705	6.7434	7.8759	10.2523	9.2014	6.9059	13.8569	6.175	9.4002	7.5297	12.4117	11.9873	11.4502	7.74	8.6153	5.105	1.307	5.105
TCGA-49-6761-01	6.23	7.7823	4.3266	8.1904	8.7676	8.5675	12.8743	9.0649	7.1174	12.9527	5.951	10.4254	3.2927	12.3526	12.5442	12.8638	8.5813	9.9483	2.9259	0	7.4555
TCGA-49-6767-01	4.3172	8.1615	6.284	8.2477	8.4735	8.4492	10.2172	10.7723	5.4658	12.1284	6.1095	9.2155	4.6922	12.3272	9.7305	11.9423	7.634	9.185	2.3623	0	4.1349
TCGA-49-AAQV-01	6.1866	8.2367	4.1481	9.1749	9.3763	8.9891	11.6064	8.5251	5.8352	14.5736	6.8839	10.6686	4.9472	11.761	10.4397	11.7312	8.4116	9.6756	3.4384	0.4093	7.2531
TCGA-49-AAR0-01	6.4555	8.0188	6.3189	9.066	8.3737	9.4706	10.7939	10.3365	8.9318	13.8873	7.3759	10.7464	8.5501	12.9093	13.1087	12.6217	9.07	8.6865	4.4478	0.9939	7.7055
TCGA-49-AAR2-01	6.6615	8.1699	7.8681	8.9065	9.0831	9.4942	10.2857	8.3397	6.8512	12.6611	9.0929	11.3591	4.0948	12.3181	12.6147	12.8714	9.8538	9.9767	2.7155	0	7.3331
TCGA-49-AAR3-01	3.9008	7.972	4.06	8.4088	7.7904	8.8125	10.1761	10.7191	3.6234	13.6411	7.22	10.4866	2.1649	12.6177	11.143	11.9715	7.7904	8.7261	4.06	0	7.9102
TCGA-49-AAR4-01	6.5004	8.3936	5.737	8.8574	7.7078	8.7282	10.2487	9.3056	5.4344	13.8166	6.7741	10.4572	5.7198	12.0406	11.1578	12.0354	9.294	9.055	3.8945	1.5319	7.9293
TCGA-49-AAR9-01	1.249	8.9031	2.4827	7.5843	7.8881	7.3447	9.2494	9.3623	2.2239	13.2128	7.7286	9.6774	2.2239	11.8947	8.5473	12.4614	6.8081	7.9102	2.8924	0.9395	6.7539
TCGA-49-AARE-01	4.6246	8.0981	2.692	8.7656	7.6366	8.7087	11.206	9.1327	4.3626	14.7478	6.7174	10.9899	4.0423	12.0305	9.2783	11.9711	8.6297	8.8117	3.3368	0.9338	5.9001
TCGA-49-AARN-01	6.1222	8.8349	6.9644	9.3905	8.067	9.011	10.306	10.3212	5.3417	13.3385	7.2666	10.3495	7.4065	12.6596	10.7703	12.2763	9.1445	8.5787	3.8513	2.2422	7.7191
TCGA-49-AARO-01	4.9533	9.0454	5.2441	9.3511	7.471	8.4528	10.1069	10.2318	5.2658	13.2044	7.3282	9.8437	6.8901	12.6047	11.2738	12.228	8.8632	9.2358	4.3238	1.1064	7.5706

TCGA-49-AARQ-01	6732	0	9.7725	5.4308	1.6992	11.222	12.916	3.1482	8.8472	3.1482	8.432	2.458	7.4007	8.8647	9.498	8.1899	7.1893	5.9334	9.8404	8.6535	7.9177	10.58	10.095	6.8176	7.0555	8.5338	7.4617	9.4567	10.375	10.0905	12.9954	9.9312	6.0844	13.8921	7.5935	10.5785	8.7223	13.0429	12.1776	12.4264	9.3749	8.6595	2.458	0.6432	7.3117
TCGA-49-AARR-01	4992	0	11.563	12.1311	3.7821	11.8652	13.073	5.2184	6.3144	1.0147	8.8123	3.2742	8.3262	9.8156	9.4701	6.3688	7.5761	6.942	9.0737	7.3238	9.2089	9.9127	8.106	7.6838	6.1269	8.5539	4.3855	8.7589	7.6766	8.7193	9.0723	9.7632	5.9831	14.2926	7.6402	9.8123	8.8716	11.788	12.9653	11.6399	8.6659	8.509	5.7567	2.022	8.6981
TCGA-4B-A93V-01	300	1	9.1673	7.3499	0.9725	10.983	14.099	13.8385	7.6029	0	8.2346	2.4141	7.6206	7.2891	8.6866	8.7011	7.0163	6.9947	10.0112	8.5938	11.341	7.0741	10.5955	7.2348	6.0745	6.9443	4.647	8.0918	7.6277	9.5638	11.8339	8.5087	7.5108	13.8777	7.0844	10.9099	1.9585	12.1726	12.1999	12.9248	9.7151	8.6415	3.7035	0	8.9779
TCGA-50-5044-01	624	1	8.6445	8.2956	5.3547	9.7951	10.965	5.9638	6.9758	0	9.1473	1.6074	8.5452	4.4915	9.4373	7.9403	5.9156	4.9394	9.1576	7.8914	9.4918	8.8537	9.6647	7.6628	5.4251	6.9108	6.7057	8.6665	8.4471	8.5804	9.9299	10.591	5.5564	13.4419	6.2999	10.8124	1.6074	13.3403	12.987	11.7813	8.4847	8.8537	5.4922	2.0252	7.6773
TCGA-50-5045-01	2174	1	9.1643	10.5691	5.4363	11.2234	10.834	5.4363	6.1964	2.2652	11.0681	5.3189	10.563	6.5813	9.8413	8.0634	7.6727	6.3007	8.2673	7.3071	9.455	10.2169	9.8727	7.6179	3.7751	9.101	6.029	9.0166	9.2944	9.306	10.6381	9.5998	5.818	13.9384	7.0778	10.1957	7.9371	14.3309	12.4247	13.2854	8.7621	8.856	5.7172	0.5089	7.2877
TCGA-50-5049-01	3094	0	9.2641	9.5462	2.8356	10.8325	11.233	4.1029	8.2427	0	11.8103	4.9604	10.4937	8.2453	9.4337	8.4364	7.5282	6.0262	8.1494	6.7716	9.6342	10.6552	9.8535	8.24	5.036	9.165	4.5777	8.4978	8.0312	8.5674	10.6305	10.5098	6.3363	13.7749	6.3659	10.0659	4.6755	13.2052	11.5291	11.8636	9.1109	7.9226	5.1309	0.6397	6.6646
TCGA-50-5051-01	478	1	9.834	9.6939	2.0351	9.5237	13.374	14.2671	5.4717	0	8.5749	2.8473	8.7356	8.9897	10.4458	7.4725	7.2187	8.164	9.1924	7.5932	9.856	7.9366	9.493	6.6899	5.0338	7.3053	4.7727	9.1905	6.0288	8.2656	10.8399	9.2867	7.6162	13.7226	6.2939	9.8425	4.2339	10.8363	11.5024	11.5276	8.6903	8.782	4.1732	0.8275	6.2651
TCGA-50-5055-01	1830	1	9.8172	9.7097	2.3654	10.2589	11.642	3.2181	5.781	0	12.0266	5.0614	10.618	7.4884	9.8366	8.1382	7.0009	5.5702	8.7771	6.6887	9.3064	9.7381	9.2164	7.3703	4.8283	8.6195	6.0756	8.7634	8.3623	8.9421	10.6302	10.8297	6.5292	12.8276	7.1176	9.8872	6.7678	13.1611	11.8144	12.4272	9.2833	8.1167	5.1315	0.8723	7.5408
TCGA-50-5066-01	1442	0	8.5662	9.0889	1.8533	11.1836	11.347	4.7149	6.7912	0.9039	11.264	1.8533	10.9386	3.9826	9.0707	8.3013	7.5433	7.245	6.2627	6.8578	9.855	10.8467	11.1793	7.9309	5.36	10.0104	5.3903	8.8097	8.3864	8.8727	10.2252	9.0704	6.7912	13.3219	7.0784	10.0407	4.1334	14.7978	12.2402	13.261	8.735	9.1762	4.27	0	7.7325
TCGA-50-5068-01	1499	1	9.9455	8.6695	3.5988	9.787	14.936	3.3793	5.1376	0	10.8173	3.2556	11.3398	5.3339	9.5254	8.6905	8.3522	4.4277	7.5368	6.8739	9.0207	10.74	11.2716	7.6999	5.5066	10.5736	2.3993	8.5788	9.4219	9.5785	10.5864	8.6075	8.3858	13.8245	6.1347	9.7286	3.1203	14.569	10.9922	13.8261	8.5362	7.9648	4.178	2.3993	8.7024
TCGA-50-5072-01	250	1	9.8984	11.8194	6.757	11.1592	14.055	15.4001	6.2	2.8083	10.9494	3.9801	9.2565	6.5162	9.9195	7.6377	6.8696	7.475	8.3718	8.2567	9.6714	8.7454	10.1849	9.4133	4.618	8.8787	7.5083	7.9441	8.8816	8.0076	10.7456	9.5511	4.6449	13.705	6.3627	10.4156	7.5622	12.081	12.4413	12.0051	8.3227	8.883	4.9336	5.9748	4.9766
TCGA-50-5930-01	282	1	9.8744	10.9482	4.8525	11.7755	14.686	14.1262	6.4701	3.6952	11.1285	0	9.0523	8.3909	10.2417	6.9754	7.2391	4.7846	8.6224	7.3371	9.0439	9.0157	9.2943	8.1011	3.836	8.6658	5.3043	7.5667	7.7909	8.0222	9.4393	10.2622	5.0388	14.8185	5.6478	10.1554	2.6581	12.3851	11.356	11.7799	8.7569	7.4942	3.1648	0	5.5693
TCGA-50-5931-01	434	1	8.2659	8.8174	4.2711	10.4667	13.117	4.4061	7.4891	0	9.0619	2.9899	9.7851	5.945	9.7242	7.5536	8.3068	8.0074	8.7192	7.294	9.0966	7.1475	9.3302	10.744	4.9682	8.6025	4.0689	8.23	9.6635	8.4698	10.3397	8.8748	7.9788	13.9037	6.2858	8.8883	4.3176	10.3703	9.6097	10.9023	9.6086	8.0109	6.3542	1.533	6.8488
TCGA-50-5932-01	1235	1	10.91	9.7102	1.8048	10.6139	8.3975	3.2192	3.6597	0	8.4569	0	8.9758	6.6724	11.0132	9.8264	4.9114	6.4087	9.7322	7.5644	8.1653	8.094	8.3725	7.8166	4.8082	9.4001	6.3289	8.3743	8.4483	8.4153	9.7045	8.403	6.9927	14.2693	4.3889	9.4944	7.1862	11.3163	11.6034	11.6708	7.5193	5.6331	4.8913	0.5014	5.9272
TCGA-50-5933-01	2393	1	10.045	9.4108	3.5503	10.5096	6.9112	8.389	5.6581	0.5948	10.8471	4.1976	9.581	1.0147	10.0907	7.8934	7.8778	5.137	8.8057	6.5836	8.2388	10.3775	9.4639	8.843	3.6708	8.3477	5.2765	8.4129	7.6549	8.1916	10.4979	9.3031	5.3862	13.6677	5.238	10.0158	5.3505	12.3015	12.7574	11.5743	6.9178	6.3599	4.4542	0	5.7702
TCGA-50-5935-01	653	1	10.364	9.4032	2.269	11.5787	12.701	3.9674	4.8899	1.1848	10.8831	3.3093	9.0672	7.3961	9.7053	9.7307	5.8009	5.1448	8.8173	7.2827	9.2173	10.0206	8.1481	7.6273	4.6909	8.0609	6.5732	9.0273	7.5705	8.4428	10.8347	9.45	7.6909	14.4218	4.2826	9.8096	5.1186	11.0536	11.2997	11.9479	8.1053	8.0648	4.5801	1.1848	3.6392
TCGA-50-5936-01	257	1	9.5887	8.8684	5.0194	8.1492	14.935	14.713	6.1826	1.2998	10.5104	0	9.1261	5.3901	10.3271	6.5633	5.6219	7.9332	9.0345	7.2016	8.7532	9.285	9.1504	9.3956	4.6923	8.3098	4.8469	8.5177	7.1727	7.867	10.5447	10.1849	4.5644	14.1339	4.9184	9.9492	4.2687	11.9201	10.49	12.329	8.4408	8.5119	4.6923	0.7916	5.7645
TCGA-50-5939-01	460	1	9.5351	12.6185	4.0045	8.3086	15.957	11.8364	5.0511	0	10.4463	0.752	9.4043	4.8604	10.356	8.5981	6.3406	7.838	8.9669	6.9716	9.0757	10.6808	9.1131	8.7954	4.2317	8.7679	3.0917	7.085	7.1969	7.7444	9.0192	9.9234	3.7349	13.6991	5.8004	9.6972	7.6245	12.1731	13.0658	11.3116	8.6966	8.5169	4.6005	0	6.5126
TCGA-50-5941-01	1474	0	9.6059	9.4768	1.8335	11.3324	11.218	3.9767	4.7699	0	10.6586	0	9.2571	5.827	9.8305	7.8565	7.0135	6.3759	9.2944	7.3042	9.0073	11.1572	9.4199	8.8574	4.9301	8.2639	5.3485	8.2057	8.4447	8.4553	10.7003	9.7034	5.6904	13.9384	6.2234	10.0919	5.9063	12.881	12.3056	11.4355	8.1772	8.258	4.7356	0	5.415
TCGA-50-5942-01	1847	0	11.291	10.2377	4.0306	10.3863	13.48	5.4749	4.7662	1.1881	12.6483	2.0692	8.6688	7.7493	9.7374	7.7664	7.1066	4.9264	8.3162	7.9113	8.742	11.8165	7.6472	8.7545	5.5942	8.6647	7.3408	8.6688	7.497	8.6597	10.0624	11.5247	7.8686	13.6069	6.8104	9.1319	4.1907	11.5036	12.3448	11.5007	8.2722	8.7221	5.8394	0.7131	4.7319
TCGA-50-5944-01	1750	0	10.414	11.3871	4.2239	9.9979	11.181	5.2087	3.7873	0	9.5293	0.6869	8.6759	6.4326	9.9571	7.5656	7.2788	7.136	8.4655	7.012	8.8308	11.0755	8.3783	8.6759	5.1355	8.5984	6.6232	7.9073	8.7162	8.3967	10.1388	11.9244	9.1823	13.9875	5.6726	9.432	8.0268	12.4051	13.0185	11.6404	9.0223	8.6009	6.3049	0	3.5068
TCGA-50-5946-01	1617	0	9.187	4.5357	2.7633	11.9577	10.962	3.2269	8.5865	4.9044	9.1392	1.9615	9.709	10.0584	10.4069	8.5361	7.1321	5.7568	8.2372	7.2917	8.6225	9.737	9.1886	8.9048	5.2229	9.2042	6.0508	8.8424	8.791	8.2279	11.1162	8.3495	7.5186	13.565	6.4882	9.3373	3.3228	11.5721	10.4825	12.221	7.5512	7.9785	3.1765	1.1932	5.7482
TCGA-50-6590-01	1288	1	10.034	7.7344	4.1131	11.7891	10.685	8.4687	6.1072	0	10.2945	2.5586	9.4875	5.4393	9.2881	8.3122	7.1693	6.6426	8.3391	7.4946	8.9928	9.7952	9.0844	8.3097	3.7549	7.561	4.1577	8.0594	6.971	8.5208	10.1335	9.6766	2.2643	13.0813	6.152	9.4777	1.3953	12.4354	11.0712	11.6896	8.5732	7.8062	3.9194	1.0614	6.8334
TCGA-50-6591-01	119	1	8.2099	2.5308	1.134	11.2765	9.4856	4.2868	8.9054	0.6757	6.7298	5.2718	9.6784	6.4234	10.7172	7.4149	7.3371	4.3725	8.8873	6.7298	8.4738	7.626	9.5692	6.8837	3.9389	6.8493	5.3158	8.7072	8.6906	8.4208	10.3453	5.8518	3.7597	12.7919	6.5204	9.026	1.4814	10.5964	10.6036	12.5455	9.07	7.8739	5.9245	0.6757	2.6727
TCGA-50-6592-01	777	1	9.6141	15.3582	3.6991	6.9824	16.796	4.8495	6.1628	0.8924	10.8727	7.0398	9.4769	9.4149	8.5715	9.448	8.083	8.7525	7.9494	6.7668	8.9707	10.2596	9.4058	9.6975	3.3812	8.3753	7.4984	7.9037	6.7496	7.512	9.9488	9.566	5.1396	13.2209	9.0245	9.6062	3.9595	12.15	12.7861	12.4779	7.7855	7.4881	4.3102	4.0739	4.6175
TCGA-50-6593-01	336	1	10.336	10.7744	4.7365	11.3304	13.357	4.2929	6.261	0.7149	11.8451	5.3033	8.9227	5.876	11.2343	9.6385	7.0428	6.0113	9.4568	7.1639	8.8143	10.6973	9.6976	8.9435	5.4587	8.5692	5.7782	8.361	8.8014	8.5799	10.5031	9.7725	7.1246	14.4891	5.7613	10.3082	9.2746	12.7371	11.2929	12.8532	8.915	7.6474	5.0475	1.1909	7.4392
TCGA-50-6594-01	370	1	10.777	7.5077	4.5567	5.944	5.6612	2.5679	8.7943	0.7691	9.4475	4.0438	9.657	2.1767	9.8032	8.8079	8.1574	6.406	9.0302	8.7874	10.013	9.2244	9.2613	9.7579	4.5129	8.7735	1.2681	9.1549	7.8224	7.9996	12.835	7.3793	5.0308	12.8248	5.1784	9.8466	5.9766	11.7188	17.7368	11.4672	9.0321	7.5571	3.6168	0.7691	8.1104
TCGA-50-6595-01	189	1	9.6913	8.9733	3.8585	11.2743	10.507	6.228	7.0181	1.193	8.8581	2.0757	9.04	3.793	8.5279	7.0465	6.7772	7.1015	8.3102	7.1611	8.7684	9.5008	9.6924	8.1786	1.8369	7.442	2.7629	7.7663	7.2121	8.1031	11.1174	11.0103	2.7629	12.9919	6.4244	9.8313	1.8369	12.3741	9.8138	10.9981	7.5207	8.2413	6.5285	1.193	6.112
TCGA-50-6597-01	1268	1	10.555	11.2012	4.6727	12.3781	11.863	6.9376	3.2453	0	10.102	0	7.9261	8.7349	10.2943	9.281	5.5014	6.5879	8.5801	7.5075	10.065	8.4305	8.6529	8.6796	5.9603	8.158	8.158	8.5766	8.8631	8.3578	11.5321	10.8079	8.8035	13.2565	5.5311	9.6878	6.8342	13.7365	11.9111	13.5822	8.8602	9.2963	3.382	0	7.8516
TCGA-50-6673-01	22	1	10.465	10.214	4.9414	10.3738	10.935	8.3426	4.8033	4.774	11.1904	1.0852	9.0753	7.5267	10.4829	8.9919	7.4776	7.1474	9.1864	7.7423	8.6989	9.2449	7.8758	9.627	5.0183	8.6024	7.0773	7.7796	9.0917	9.1738	11.4403	9.5674	6.421	13.8294	6.7862	9.8616	7.3935	11.8131	11.9429	11.65	8.9102	7.9165	5.7772	0	6.07
TCGA-50-7109-01	308	1	10.253	8.7603	1.7827	9.7858	15.146	16.2624	5.6734	0	11.0909	1.7827	9.9603	8.717	8.9523	5.3883	8.0038	5.6207	8.1811	7.251	9.1223	8.9819	9.0416	6.943	3.428	8.3191	4.7349	7.9007	6.7763	7.919	10.3136	9.3556	5.566	13.1862	6.8545	9.9399	2.6986	11.7916	11.0628	11.212	7.7494	9.0078	4.7995	0	6.2019
TCGA-50-8457-01	1125	0	9.9421	9.8514	4.0474	10.7174	14.005	4.7882	5.1901	0.53	10.4114	0.9166	8.8959	5.5864	10.2995	8.877	7.2597	5.8197	8.982	7.6071	9.6714	10.7882	8.1529	8.0979	5.7619	8.4642	6.0785	8.904	7.6169	8.6407	9.5157	10.6243	8.2799	13.6982	6.4594	9.3052	5.4757	12.1029	12.2047	11.8261	8.4079	8.4939	4.5898	1.2213	6.6307
TCGA-50-8459-01	1119	0	9.2938	11.0432	4.5813	9.228	12.22	6.4081	6.929	5.803	10.4509	3.315	9.2861	6.6841	9.9264	6.3098	6.9746	6.005	9.1801	6.7753	9.384	9.6518	8.2005	7.4922	5.2451	7.6353	7.0858	8.5281	7.3706	8.412	8.9852	11.2765	4.2827	14.7596	7.3159	9.7031	8.6596	12.9078	12.1156	11.7819	9.209	7.9396	6.3199	1.0834	7.3706
TCGA-50-8460-01	829	0	9.5293	9.1129	3.9308	9.2454	10.377	4.1976	4.3803	0	12.835	0.6956	8.8861	6.6004	10.3212	8.938	6.15	5.4715	9.3519	8.838	9.3831	11.4873	8.513	8.4221	6.822	8.2101	6.0724	8.6327	8.8203	9.2051	10.8819	11.3461	8.7264	13.9105	6.2707	10.3266	6.515	13.4986	13.255	12.2322	9.1835	9.1589	5.0806	0	7.2671
TCGA-53-7624-01	1043	1	11.006	11.2631	3.8693	8.2015	7.8129	6.7687	7.7562	2.7462	8.2522	3.0048	9.6811	6.6788	7.9481	7.5073	7.176	6.7452	9.2321	7.5281	8.3293	7.2277	9.4979	8.277	1.862	8.5812	4.4061	8.6029	7.3876	7.9454	10.9448	7.4933	5.9955	12.7133	4.2147	9.8046	2.4307	10.8611	8.1264	9.7177	9.1652	7.5553	1.6762	0.5253	5.066
TCGA-53-7626-01	929	1	9.9505	10.5749	3.491	10.9608	13.543	4.7338	4.5357	0.3612	10.9804	0.3612	9.62	5.0998	9.2218	7.9155	7.8369	6.3791	8.499	7.5585	8.9588	10.9173	8.6811	8.2841	4.1528	8.4834	3.8165	8.5036	8.0204	8.5666	10.2278	10.9234	6.2822	14.4205	6.2502	9.2578	7.0831	12.0055	12.2255	11.5439	8.5171	7.3876	5.7298	1.2766	5.7605
TCGA-53-7813-01	424	0	9.8676	7.555	1.0198	9.7554	14.422	6.0389	6.3789	2.3536	9.2658	0	9.3748	6.1474	9.8543	7.6903	7.8141	6.482	7.9522	6.8465	8.6112	8.4698	7.5231	7.9158	6.1264	7.9578	5.2479	8.5863	6.0389	7.654	10.9732	7.5069	6.1887	15.1411	5.8968	9.7399	3.8441	10.0224	9.397	10.6384	7.7328	7.5707	3.8441	3.9438	4.037
TCGA-53-A4EZ-01	1071	0	9.5456	14.6593	3.2113	8.2514	13.221	2.4135	4.8934	0	8.3116	0	9.2255	8.1044	11.4954	3.0147	6.9188	8.5177	9.634	7.3941	10.2372	6.6572	10.2874	7.9292	6.8016	7.3526	8.0837	7.8722	8.206	8.8071	10.8768	7.7589	7.6387	13.6574	7.5169	10.3492	3.2113	11.3962	10.7993	11.9708	9.2657	9.4946	2.4135	1.7487	4.8349
TCGA-55-1592-01	701	1	9.8052	11.3266	4.9049	9.0743	13.441	4.1301	4.8653	1.2346	10.336	2.7406	10.4142	10.85	9.9553	7.655	7.9767	5.0056	8.0566	7.4736	9.0043	10.3417	9.2948	8.7849	5.9327	9.3417	5.2713	9.175	8.9264	8.26	10.2635	10.0258	6.8549	14.4088	6.4169	10.3287	7.2837	11.7519	11.7063	11.5937	10.864	8.3786	3.5965	2.906	6.2728
TCGA-55-1594-01	1178	0	9.0089	7.3308	2.3018	10.0119	12.475	3.9213	9.6363	3.3832	9.7546	0.4783	11.348	4.1301	9.4284	7.3131	7.7602	7.497	9.1503	7.3202	8.2334	8.1638	9.8657	9.2204	3.6772	8.327	4.4995	8.0138	9.1067	8.7035	10.1816	7.8854	6.8699	13.0697	8.6409	10.0925	5.5052	11.6806	12.0633	11.191	8.2668	8.9956	4.2541	1.3631	3.7209
TCGA-55-1595-01	1479	0	9.3324	8.9605	3.208	10.5923	11.957	5.2683	5.8214	0.3843	9.71	5.6653	9.5513	5.0597	10.3121	6.1904	7.7942	6.5323	9.1942	7.3493	8.3854	10.1236	9.4211	7.9853	5.0987	8.4259	5.3992	8.9292	8.786	8.7445	10.4897	9.5264	6.8281	13.9751	6.6155	9.4295	4.7019	12.0841	10.9931	11.6791	7.806	7.5831	5.0464	0.3843	4.0764
TCGA-55-1596-01	2065	0	9.1741	6.9282	6.2516	6.0728	15.816	3.81	7.6938	1.127	9.3546	2.1866	10.8916	9.242	10.0397	10.544	6.6759	9.2546	9.1667	7.8389	9.5106	6.8854	9.707	8.2345	3.0188	9.3398	8.6504	8.011	9.1291	8.3225	10.804	8.2058	4.3602	12.9054	10.053	9.9248	4.1835	11.5386	11.1359	11.9243	9.0619	8.7026	3.3047	0.6709	3.3047
TCGA-55-5899-01	930	0	8.8506	10.1056	5.4736	11.7906	8.8938	5.997	7.0178	2.3221	10.0898	5.3578	10.1197	5.2535	9.6593	9.301	8.3059	8.9917	9.457	8.2145	10.391	9.2163	10.1969	7.2304	7.263	7.4436	7.0862	8.3369	7.8889	9.1409	11.2258	10.2514	8.5652	13.6468	7.005	10.6137	4.1815	11.7154	12.6339	11.5237	8.6373	10.2164	3.6358	0	5.997
TCGA-55-6543-01	435	0	10.031	8.8129	4.4713	8.0929	14.38	3.7887	4.2776	0.6391	8.2674	0.6391	8.4738	7.8356	9.6621	6.1657	6.4678	6.1769	8.3312	7.8635	9.4957	10.6294	8.4624	7.9077	5.6129	8.2848	2.7168	8.4805	8.1814	8.7803	10.284	12.0323	7.8461	14.0769	6.374	10.1152	7.1557	12.5841	10.8274	11.6967	8.1647	9.5097	4.7356	0.6391	4.7356
TCGA-55-6642-01	2449	0	9.1225	9.843	1.8565	9.8776	11.201	13.5601	6.2423	0.7271	10.9726	6.1654	8.8343	9.2144	9.6318	7.2761	7.3003	5.4021	7.9772	6.7871	8.3007	9.3291	7.2453	7.5578	4.5411	8.2748	4.6201	8.5514	7.1285	7.9659	9.5317	9.8223	5.3566	14.4072	6.056	9.6293	3.0371	10.3147	10.0391	10.7978	8.5514	6.1785	5.7036	0	3.7496
TCGA-55-6712-01	171	1	10.39	8.691	3.5552	9.4551	11.469	4.8016	4.309	0.9232	10.84	0.9232	9.7001	4.6553	9.919	8.992	5.5177	6.3984	8.3855	5.7755	9.1019	11.5255	8.776	8.5219	4.0212	8.1543	4.2422	8.2175	7.1917	7.7131	9.9082	10.1908	5.8889	15.6295	5.5177	9.5409	5.4601	13.1261	12.5459	12.3389	7.0428	9.6104	3.5552	0	6.3674
TCGA-55-6968-01	1293	1	8.2819	10.4902	3.4237	12.9617	10.166	2.7493	9.5336	0.653	10.4069	4.3549	9.8342	8.1916	9.4978	8.8689	9.432	7.2812	7.9256	6.9349	9.0885	8.9496	9.9787	7.9789	3.9364	8.2833	3.7648	8.2028	6.3218	7.7216	10.089	9.1136	9.2241	13.8932	5.8353	9.0896	3.57	11.6252	10.0799	11.533	8.5074	7.5427	2.4801	0.653	5.2119
TCGA-55-6969-01	1239	0	8.5702	9.9583	3.0433	9.934	12.003	9.0399	9.7665	4.1896	10.6378	4.0159	9.7596	6.7904	10.0005	9.932	8.1888	4.9385	7.9072	7.5622	9.0871	9.0521	9.1838	8.33	5.5295	9.0195	6.6587	8.2684	6.9398	8.316	9.8009	10.4428	6.9993	13.764	6.9398	9.692	3.1025	11.8159	10.7446	11.3396	8.784	7.5004	3.3665	0.4275	5.1355
TCGA-55-6970-01	464	1	9.1635	8.7595	5.4714	11.165	11.656	16.1246	4.146	0.7159	10.4794	0	9.3765	7.7195	11.4906	8.387	6.8175	4.4516	9.6061	7.4227	9.04	10.0656	9.354	8.7216	5.5024	8.6852	5.755	8.1067	8.6192	7.8677	9.7471	8.1498	5.4397	14.8579	5.2579	10.3203	5.196	11.9933	13.0491	11.6022	8.768	8.1234	5.9475	1.1921	7.2386
TCGA-55-6971-01	1400	0	8.8019	10.3847	4.1257	8.839	13.185	5.1244	7.093	1.5539	11.0479	0.9768	9.1455	7.2572	9.2459	7.4533	6.9422	7.5157	8.3627	6.735	9.0538	10.5762	8.4963	8.0775	5.2398	8.12	5.2398	8.1869	5.5088	7.9812	9.7014	9.9147	8.8899	14.3331	6.4772	9.4807	6.1436	12.2094	11.4797	10.4669	8.2752	8.5935	5.1244	0.9768	6.1237
TCGA-55-6972-01	1632	1	10.336	12.8638	3.761	12.4264	6.5227	4.504	2.9726	0	7.3796	0	9.1448	4.3509	10.8112	9.5081	6.6152	4.2679	9.0486	7.6978	9.755	6.1188	9.7701	7.8267	7.0721	8.3774	7.3992	9.4123	9.0885	9.1793	9.9722	4.7685	8.7297	14.341	5.0428	9.879	3.1685	12.1761	12.9159	12.716	7.0349	9.0702	2.4767	0	4.8277
TCGA-55-6975-01	118	1	10.485	12.2255	3.4671	9.2114	12.047	14.1782	6.2876	0.8783	10.7392	1.4203	9.9648	6.7476	9.3614	7.0509	6.3333	6.8767	8.5339	7.5858	9.4198	7.9213	10.2213	7.8757	3.9307	8.8569	4.4565	7.3318	8.0037	7.8339	10.459	10.5593	3.5725	13.7713	5.8991	10.3501	1.4203	11.8035	10.5204	11.7895	7.5476	9.2316	5.8156	0	4.5106
TCGA-55-6978-01	176	1	9.99	9.6504	4.5883	10.2016	10.984	7.4893	6.4973	0	11.1127	3.8767	10.1445	3.1579	8.5536	5.3186	7.8797	6.9227	8.8756	6.9141	8.9633	10.2541	9.2186	8.538	3.9792	7.6528	6.0091	8.3276	5.8649	8.1284	10.1879	10.3218	4.4992	13.7633	6.8344	8.9719	2.8327	12.3548	10.2752	11.0555	7.5544	7.7887	5.1087	0.4439	5.856
TCGA-55-6979-01	237	1	9.1306	9.244	4.1629	10.0539	13.098	4.4686	5.1221	7.2394	10.9756	4.2186	9.6743	4.6415	9.1542	7.787	8.0474	6.6592	8.565	6.6491	9.3829	10.7732	9.3799	7.8676	5.8774	8.5051	4.6817	8.9347	7.5198	8.4793	11.5909	10.378	4.5577	14.4743	6.9903	9.8857	3.6982	13.0756	10.7042	11.453	8.0397	8.4359	6.3206	1.2687	6.6692
TCGA-55-6980-01	2109	0	9.4001	10.3353	4.6869	10.2901	14.578	4.7356	4.8725	0	9.3045	1.469	8.7682	4.9976	10.1638	8.2023	6.9218	6.9836	9.3868	8.0325	9.5778	10.933	7.8061	7.862	5.1846	8.895	6.5107	8.4191	8.3426	8.047	11.0543	10.1921	4.6869	14.4897	5.441	9.9887	6.8126	12.4748	11.7561	11.8047	7.8999	8.7914	4.8725	1.469	5.1846
TCGA-55-6981-01	1379	1	10.275	9.5272	3.2886	10.1195	14.984	3.4127	4.9865	0	9.896	2.4292	9.1622	3.5269	9.7137	8.2832	7.7833	7.397	8.9636	8.0015	8.8926	10.7819	9.0616	8.0356	4.5192	8.5453	5.6219	8.7627	9.3887	8.6818	11.91	9.3716	5.1734	14.362	6.5942	9.8637	6.321	12.1256	10.8979	12.069	7.9916	9.4296	4.6759	0	5.596
TCGA-55-6982-01	995	1	10.461	10.3895	4.9737	12.827	13.551	4.9307	5.4998	0.9425	11.021	7.6776	9.4696	6.2592	10.3907	9.0044	7.7658	6.2935	8.9354	6.8025	8.8935	10.0898	8.8544	9.5763	3.7977	8.3939	7.2321	8.2139	8.6806	7.5486	10.3776	10.4798	7.3096	13.8553	5.7427	10.1716	6.2061	12.3246	11.7661	12.2018	8.0379	8.6511	3.2167	0	6.4986
TCGA-55-6983-01	2823	0	10.02	9.7396	3.7067	11.1738	11.947	4.2544	7.1619	0	11.2138	6.7274	9.53	9.8028	9.8994	8.4913	6.1029	5.8951	9.6722	7.6228	9.4205	8.8506	9.2574	7.9239	5.409	8.401	7.2785	8.2319	7.515	8.6411	10.1368	10.4365	6.9994	14.5535	6.5422	10.1894	4.7864	11.9472	10.6235	11.3657	8.6113	8.3882	5.0407	1.0453	6.4633
TCGA-55-6984-01	760	1	10.345	9.1018	2.8872	9.7593	13.383	3.4666	4.8746	0.9367	8.4406	0.9367	9.509	7.4635	10.6562	7.1567	7.6783	5.5455	9.6535	7.355	8.9984	9.1583	8.6716	9.0254	4.1991	8.8954	7.3789	7.9583	8.7564	8.7318	9.3259	9.571	3.9658	13.1219	7.1192	10.1361	7.1192	12.4329	9.9272	11.4308	7.6783	8.2802	4.461	0	6.5859
TCGA-55-6985-01	1233	0	10.867	11.2831	5.4442	12.3394	12.923	4.8004	6.2408	8.2759	10.8965	5.6077	9.0235	8.88	9.1899	8.2408	7.0481	6.1066	8.6688	7.4191	8.967	11.0847	9.3382	8.9579	4.1225	8.3591	5.9759	8.1678	8.3102	8.5095	10.7941	9.6776	6.0751	14.2188	5.8694	9.7864	4.9119	12.2029	11.011	12.0169	8.2549	8.126	4.5009	1.6955	6.4523
TCGA-55-6986-01	3261	0	10.652	8.7475	4.6842	8.8668	13.485	5.3565	3.7393	0.788	8.7764	0.788	8.9326	5.2504	9.7592	6.4981	6.5438	4.2607	8.5293	7.8943	9.605	10.9459	8.4743	8.2343	7.2835	8.0309	4.9783	8.0917	9.6783	9.2055	10.1936	10.4179	5.4068	13.4384	6.9178	9.4767	5.7756	12.8084	11.4375	11.9675	7.3299	9.1082	4.4161	1.2948	5.1357
TCGA-55-6987-01	2137	0	9.1063	10.3765	5.1823	10.1252	14.588	14.6535	5.3541	0.7708	10.7272	0.4363	9.4457	4.9383	9.2184	8.7001	7.6036	6.3363	8.5327	6.6266	8.616	10.4203	8.9778	7.9614	5.5297	8.7075	3.397	8.4272	8.0017	8.4078	10.8433	9.2868	6.3613	14.9975	6.8983	9.8335	5.0191	12.5224	11.7625	11.4507	7.8197	7.8129	4.3767	0	6.119
TCGA-55-7227-01	952	1	9.8139	9.0732	1.2523	10.534	12.353	4.5732	4.4418	0	10.7675	0	9.1854	3.3196	10.0301	6.318	6.7191	6.4379	8.6603	7.0326	9.0379	10.8829	8.3722	8.5688	5.3845	8.4522	4.8049	8.6229	8.0685	8.354	10.2054	10.2795	6.355	14.0517	5.8497	9.0602	7.3277	12.4704	11.0441	12.0651	8.6102	8.1861	5.1797	0.758	6.3792
TCGA-55-7281-01	872	0	10.02	8.5455	5.1135	10.4558	12.393	5.7547	4.1313	0	10.9408	5.6765	9.1453	5.4318	10.072	7.3968	6.6704	6.718	9.0539	6.718	9.05	10.8871	9.1868	8.224	5.4318	8.8105	4.4648	8.312	7.9224	8.5009	10.1505	9.7845	6.4624	13.9207	6.3858	10.4154	7.264	13.5944	10.7902	12.7264	8.9874	8.6495	4.2222	0.6506	5.2919
TCGA-55-7283-01	609	0	10.049	9.61	3.6472	9.8142	12.537	3.8107	2.4624	0.5862	10.0453	0	8.9402	8.2131	10.8229	8.3028	6.3523	4.8861	8.9737	7.8459	8.8169	9.952	8.5524	8.4066	5.4793	8.2946	5.9224	8.7332	7.1885	8.8174	10.2774	8.012	8.1121	14.3504	5.8109	9.7298	4.0478	12.2733	12.3781	12.3724	7.8923	7.6293	3.9576	0.5862	6.535
TCGA-55-7284-01	243	1	10.088	10.4918	4.0785	10.3872	11.968	7.1229	5.6509	0.6989	9.4391	0.6989	9.1344	8.2208	9.8288	7.6936	8.0548	7.0088	9.889	6.6897	9.0369	10.2183	7.7258	9.3133	5.5868	8.6597	8.0649	8.3407	6.4013	7.7552	9.3378	9.5578	5.7792	13.6076	8.6481	9.4176	8.8231	12.0789	12.3745	10.9739	9.0161	8.0766	5.7627	1.1678	5.5774
TCGA-55-7570-01	824	0	9.6543	14.0467	2.1654	8.7518	9.9226	4.6164	5.7591	4.0606	8.7749	2.8279	10.2626	7.5473	9.5915	7.6258	5.5998	9.8468	8.6708	9.3135	9.6669	7.2498	10.1993	8.5367	6.0138	8.8099	5.6254	7.1865	7.5871	8.1286	10.7249	8.9643	6.2634	12.8938	6.4403	9.6958	5.0738	11.0985	11.0418	10.7771	9.8705	9.7961	3.0717	0	5.6381
TCGA-55-7573-01	487	0	10.319	11.368	3.0366	10.8355	13.113	4.2791	5.5434	0	10.5742	3.512	8.9014	7.3392	9.9312	10.2235	7.3744	4.598	9.3036	7.4688	8.6322	11.3597	8.5066	8.4449	5.3587	9.3226	6.3692	8.2614	8.0897	8.6911	9.7881	10.8992	8.3943	13.7989	5.7929	9.944	6.4769	11.5866	11.9546	11.5982	9.4643	7.6522	4.0885	1.3792	5.1468
TCGA-55-7574-01	995	1	9.402	9.7978	3.2827	11.5866	10.613	5.6271	6.7885	0.843	11.8391	0.843	9.5891	5.9754	9.9926	8.1233	8.1477	6.029	9.1831	7.3269	8.8477	10.1337	8.7524	8.0816	5.3745	8.3714	6.0807	8.8769	7.1846	8.5349	10.1059	10.7518	5.2596	14.0398	7.1284	9.6359	3.16	11.9719	11.5958	12.0916	8.1677	7.5572	5.4018	1.3716	6.9821
TCGA-55-7576-01	670	0	10.693	8.6764	3.9002	11.4821	11.854	7.8966	6.7587	4.7498	11.2696	0.7934	9.3962	5.6614	9.0156	8.5075	7.8907	4.641	8.7544	8.009	9.0291	10.9467	9.7011	9.7351	4.5833	9.1415	6.3513	8.2881	7.3726	8.233	10.6858	9.6994	6.5222	13.8085	5.1179	10.42	7.3895	12.1078	11.2307	11.7315	8.1621	7.9288	4.5232	0.7934	5.788
TCGA-55-7724-01	705	0	10.124	11.1456	3.0025	8.5225	12.86	5.6175	5.7308	1.0014	10.8763	7.0581	9.6611	5.8855	8.1588	8.2883	8.0582	7.4375	5.957	5.1727	9.7912	10.8725	7.2508	9.2906	3.9096	9.0659	2.0022	7.542	4.2507	6.7577	10.3549	7.1009	7.0581	15.1348	5.0903	8.7033	7.724	10.4806	13.9795	10.3725	9.5708	8.542	6.5727	0	2.0022
TCGA-55-7725-01	442	0	11.076	10.9374	2.0536	8.065	13.486	2.8684	1.6326	0	12.1167	0	9.3837	4.5915	8.9353	7.1685	6.9032	5.5288	6.0006	7.3279	9.0458	10.4119	7.0246	7.6631	5.5613	9.6689	3.5241	8.2655	6.2795	7.0817	11.3934	8.9163	7.7917	13.3823	6.0699	9.1689	7.6098	10.8233	11.3242	10.8596	8.4414	8.0594	4.8233	3.5241	4.237
TCGA-55-7726-01	652	0	9.5776	9.4071	2.4427	10.4947	8.2966	5.8304	5.8963	0.9163	11.2156	4.6414	9.1542	0.9163	8.2716	5.154	4.9204	5.4163	7.2037	6.0588	8.3305	10.1439	9.2686	8.4537	2.4427	8.2743	0.9163	6.0198	7.4432	7.3907	10.5623	10.1314	4.2285	13.7567	4.4787	9.9557	2.6608	11.1291	10.1084	11.023	6.6365	7.8506	5.8077	0	5.258
TCGA-55-7727-01	119	0	10.342	9.4014	2.4821	8.2641	13.732	3.4714	2.8918	1.5033	10.3576	0	9.8459	3.7912	9.0843	9.2596	8.1522	5.0492	6.9641	6.0869	8.4608	10.6702	8.0659	8.737	5.6853	8.4709	3.3469	8.4421	6.8425	7.3682	10.5357	7.1526	7.8246	14.9297	5.3704	9.4924	5.338	10.7976	10.7774	10.8422	9.0358	7.4763	3.6923	1.5033	3.3469
TCGA-55-7728-01	704	0	9.6861	8.8255	4.7312	9.6101	12.646	3.4597	4.467	0	8.1347	1.3114	7.8074	7.6353	10.674	7.0858	7.9845	6.6155	10.0085	6.5824	9.5373	9.5415	8.4677	7.3477	6.8467	7.5793	9.5264	9.3228	7.3145	8.4946	9.4897	9.8061	6.1119	13.8116	9.3704	9.8403	6.9279	13.734	11.3408	12.5555	9.3808	8.7543	3.4597	0.7998	7.8772
TCGA-55-7815-01	773	0	9.3931	8.6647	0	6.3427	15.026	7.1526	3.0025	0	11.8992	5.0472	9.9425	4.6466	6.6041	8.4291	6.5727	7.542	5.9801	5.8102	8.5521	10.5107	6.9686	8.2929	3.4621	8.8605	3.4621	7.6539	4.5263	6.7443	10.6158	6.771	8.408	14.4022	3.3245	9.4123	4.5263	9.9262	10.7477	10.136	7.9277	7.9512	5.4623	1.0014	1.0014
TCGA-55-7816-01	468	1	8.2388	6.6916	3.7052	8.5332	8.4755	6.5922	6.8289	0	10.4764	0.5161	9.2497	2.8118	9.3655	8.1907	7.7837	5.6323	8.1476	7.9308	9.0812	11.6283	8.0051	7.9	5.146	9.1359	5.2638	9.226	6.3925	8.0315	9.3605	9.6363	6.2692	14.0252	6.6551	9.6907	7.847	11.7009	11.098	10.7443	8.856	7.6511	3.752	0.8955	5.0925
TCGA-55-7903-01	567	0	9.7886	7.9608	2.4553	11.1774	13.87	5.1036	5.5186	0.5833	9.6911	1.3189	9.4149	4.3528	9.9875	7.7297	8.4928	6.7161	8.9004	8.0448	8.6447	10.0897	9.4616	8.9168	4.8531	8.895	5.8654	9.1139	7.1191	8.8231	10.96	7.9109	5.9139	13.9714	6.3526	9.3733	4.6099	13.5925	11.0637	12.9411	8.6681	8.1371	2.9956	0	6.5875
TCGA-55-7907-01	343	1	10.183	9.1905	2.5087	11.3853	10.583	3.9139	6.2929	9.5761	12.4128	2.6944	9.2001	9.3037	10.6148	7.1863	7.9816	7.1707	8.7385	6.6704	8.7429	10.0872	9.5357	9.8548	4.2466	8.5512	7.7286	8.1539	7.5891	7.4606	9.8873	9.4815	8.4851	13.4427	6.1729	10.2018	3.1405	12.2988	11.2088	11.632	6.9694	8.9517	3.3761	0	6.249
TCGA-55-7910-01	1040	0	9.1906	7.282	1.9175	9.2598	14.823	12.8566	6.4901	0	10.1287	1.9175	8.7158	5.506	10.2242	8.4553	7.4053	5.7239	8.781	7.2026	7.1886	7.6038	9.4437	7.0191	4.5938	8.2333	5.0215	8.6035	7.3572	8.1514	10.2889	8.0792	4.6481	13.8263	4.5374	9.9605	1.9175	11.3105	9.8947	10.7478	9.4341	6.7603	5.1773	2.2338	5.351

TCGA-55-7911-01	537	0	9.6348	8.0856	3.0705	9.9319	11.433	8.8761	4.5687	0	12.3286	0	9.7384	4.721	9.6539	7.6835	8.1833	5.9738	8.6114	7.8146	8.9922	10.758	9.7815	8.8216	5.268	8.3667	2.6522	9.3458	6.7519	8.9365	11.0399	10.321	6.773	13.4712	6.5056	10.149	3.2415	12.3914	9.5262	13.0002	7.6345	9.3528	4.5687	1.639	7.2696
TCGA-55-7913-01	561	1	9.607	4.8782	2.242	9.3698	11.882	16.0978	5.3536	0.6543	7.0014	0	9.1144	6.1325	10.5783	4.9334	5.1236	7.0584	9.9473	6.6061	9.18	7.5859	9.7325	7.8562	5.9965	8.2443	6.224	9.3767	7.1747	7.9909	12.1316	6.4586	3.2206	13.363	7.8828	9.8905	6.8941	11.9099	9.704	11.3215	9.1499	8.7834	3.7376	0.6543	7.3924
TCGA-55-7914-01	187	1	11.186	8.2305	2.9063	12.1294	14.915	3.8067	4.0868	1.5845	11.112	2.3214	9.6637	4.8819	9.2673	9.7483	5.3916	6.2567	8.5767	7.851	9.1265	8.5384	8.7691	9.0999	4.9993	8.5311	3.3917	8.7608	9.1247	8.4505	10.7135	9.1792	7.3167	11.5268	5.8697	9.5169	6.8002	11.1107	10.1642	11.2979	9.3201	8.1767	3.8573	0	7.0049
TCGA-55-7994-01	603	0	10.611	6.699	3.3479	11.252	12.316	4.4733	3.7724	0.3968	9.8186	0.3968	9.6274	4.1522	10.3246	6.2546	5.8178	7.8026	9.2043	7.426	8.7888	12.3876	9.0222	9.2613	4.8028	9.4422	4.7529	8.2282	7.5011	8.1059	9.6469	8.6919	5.0978	12.7578	6.3751	10.2683	3.517	12.1111	10.1267	11.3541	6.7508	9.3031	2.1644	0.9633	7.1194
TCGA-55-7995-01	889	0	10.261	15.444	3.2023	7.2883	15.955	3.4834	7.3396	0.6469	10.1916	4.9536	9.6366	10.716	8.9979	6.6318	8.2098	8.8155	8.3552	7.2883	8.7529	9.9377	9.5672	8.7431	5.1388	8.3252	4.1904	8.8308	7.1995	8.4458	9.9133	8.846	6.8349	14.647	5.7897	9.4105	5.487	11.9011	11.2081	11.2285	7.9898	8.3873	3.246	1.8262	6.3856
TCGA-55-8085-01	904	0	9.0198	12.3014	6.1127	12.9025	11.397	9.7829	4.6839	0.7621	9.9757	1.7806	9.1368	5.6556	10.7758	8.1806	6.3153	6.6562	9.9657	8.276	9.9985	8.8989	9.2549	9.5013	5.9353	8.7467	8.3065	9.1034	8.761	8.7987	10.3243	9.5523	7.639	14.2291	7.6738	10.0276	3.8301	12.4156	12.6556	12.8749	8.6354	9.0333	3.0543	0.4307	7.654
TCGA-55-8087-01	462	0	10.825	10.0336	2.3753	9.4209	13.072	3.7243	2.932	0	7.8884	0.4319	8.6374	8.5352	10.0356	8.1051	7.0123	4.7457	9.5787	7.7172	8.7455	9.7863	8.2577	7.7827	5.8201	8.2863	7.9386	9.2346	8.7469	9.1947	9.2152	11.194	7.5391	14.3153	6.4173	9.3777	5.5461	11.3528	10.8491	11.8264	8.6323	8.8747	4.1782	0.7639	4.1214
TCGA-55-8089-01	702	1	10.052	8.3291	3.796	8.5112	12.302	5.0269	5.31	1.6225	11.6337	0	10.25	3.9592	9.4271	7.2435	7.8462	7.9273	8.8651	7.4769	8.7736	11.3452	8.768	8.1861	3.1539	9.2692	2.6794	8.0433	6.1553	8.0018	10.2514	9.9142	4.1058	14.4926	6.9455	9.3962	5.5337	12.9089	11.3966	11.1982	8.316	7.6865	4.1402	0.8732	5.693
TCGA-55-8090-01	598	1	10.282	9.5087	3.2737	10.9946	13.824	4.7556	5.1192	0.9479	9.0888	0.6955	8.4754	4.58	10.4725	5.6329	7.0812	7.5091	8.9727	7.1298	9.569	10.5557	8.794	7.9074	6.0983	7.7355	3.1782	8.7185	7.4694	8.6885	10.5608	10.2751	7.0205	13.8609	6.0852	10.1439	6.4445	12.6119	10.7791	12.0951	7.5879	9.6202	4.6879	0.3892	7.449
TCGA-55-8091-01	600	0	10.199	12.4952	3.7818	9.302	14.955	5.524	6.4414	0	10.1157	4.2766	9.1388	6.2836	8.9777	6.4414	6.7079	6.0666	9.2523	7.1578	9.9101	10.487	8.5836	7.4853	5.1654	8.6003	4.1903	8.0026	7.6773	8.4599	10.8644	11.9571	7.2099	14.3477	6.7233	9.4117	4.5779	12.7744	11.846	11.8019	8.0026	8.8282	5.1194	1.3222	5.9044
TCGA-55-8092-01	154	1	8.6882	8.8388	1.689	8.7728	12.878	14.1899	5.642	0	10.4929	0	8.1243	6.4626	10.4817	8.2624	6.7812	3.9709	9.0424	7.9089	8.7169	9.8386	9.0713	8.6804	5.6925	8.8423	6.4626	8.1718	7.3203	8.5025	10.5123	9.8774	4.5661	12.9493	6.1099	10.1365	5.7532	12.2246	10.9026	10.8584	9.3325	6.6971	3.8426	0.9182	8.3807
TCGA-55-8094-01	541	0	9.9938	6.0306	1.4793	11.2061	14.416	16.1441	4.1926	6.3375	8.2497	0	8.9129	3.3133	10.5535	9.7875	8.2888	5.8486	9.0699	7.5694	8.7268	7.2232	10.129	9.0795	3.8189	8.261	6.2723	9.274	8.4743	7.5512	10.899	6.7507	8.3533	13.4959	8.129	11.2565	3.3133	12.0327	12.3556	11.9736	6.7427	9.0145	3.0272	3.0272	7.1463
TCGA-55-8096-01	719	1	9.3352	10.1483	3.612	9.9406	15.187	6.1594	6.4285	2.3374	10.7244	5.2402	8.7969	8.0874	11.1327	9.2055	7.2911	5.6783	8.2792	7.2174	8.9753	10.279	8.7349	8.2156	5.8362	9.0357	7.7266	8.9809	8.761	8.5164	11.2352	10.0116	6.2801	14.6022	7.0475	10.703	8.4853	12.4276	10.5616	11.6681	8.133	8.5152	5.2162	1.3557	7.5458
TCGA-55-8097-01	476	0	10.767	10.5889	2.9645	11.148	8.8442	4.3934	4.6217	1.1382	8.9002	0	8.5098	8.194	10.863	9.5858	7.7277	5.8239	9.3644	7.8972	8.7893	9.7783	8.4232	7.5263	5.9032	7.9848	7.6919	9.0646	7.459	8.6629	11.0829	10.6241	7.4978	13.0252	7.2698	9.4287	5.7399	12.0164	11.2287	12.2186	7.3428	8.1821	4.4736	2.2025	4.8188
TCGA-55-8203-01	547	0	9.4805	8.1127	3.5752	9.6441	11.957	7.7984	6.0817	4.7701	10.5923	3.8425	9.2403	8.4175	9.8989	9.0152	7.5841	5.6064	8.9787	7.4735	9.3304	9.1321	8.5464	7.2806	5.0943	8.4137	5.8785	8.7566	6.9768	8.4562	10.4313	9.5335	7.8063	14.2913	7.7203	9.9882	4.2629	11.7823	9.9427	11.1659	7.9524	7.4603	4.4346	0.855	5.9272
TCGA-55-8204-01	515	0	10.611	11.4207	2.2496	10.4436	13.125	6.0069	5.3658	0.6196	9.8286	2.0769	8.5608	6.808	9.103	9.0068	6.0307	5.5588	7.4717	7.6246	8.9665	9.4469	9.5235	8.9856	3.6166	8.4333	0.6196	7.489	9.429	8.4344	10.3956	10.0166	6.3857	14.5576	5.6995	10.2944	4.931	12.0815	9.6874	11.7211	7.4586	8.5401	5.3845	2.2496	5.0758
TCGA-55-8205-01	599	0	10.219	8.3488	1.18	8.0163	10.915	7.0686	4.2508	0	10.5999	0	9.7847	3.1557	9.0227	9.2036	7.1986	7.6556	9.4402	7.2384	9.3324	10.7346	9.6999	8.6153	4.0724	8.3046	2.4411	8.6611	6.4124	8.3696	9.9326	8.6801	5.2727	13.7469	6.7374	9.8129	2.81	12.6258	10.6356	11.4655	7.3321	7.698	4.9718	0.3967	7.0376
TCGA-55-8206-01	888	0	10.727	12.251	3.0675	9.5855	12.228	4.2491	4.7062	0.3733	8.9034	1.1252	9.1026	8.3817	10.291	10.2867	7.5922	6.7258	9.4018	8.3257	9.5364	10.7424	7.7628	7.9082	6.0503	8.487	7.6163	8.8379	8.5018	9.223	9.3898	11.0089	8.961	13.9064	6.8784	9.4616	6.9832	12.1249	11.8145	11.7063	8.6278	7.9622	5.6446	0.3733	4.4957
TCGA-55-8207-01	977	0	9.8329	12.2976	1.4937	12.4847	13.147	4.8922	5.3939	1.2945	11.418	1.8248	8.9566	5.7362	9.4685	8.8852	6.5984	7.0849	8.5358	7.3423	9.0692	10.7309	8.2817	8.7866	6.1146	8.8112	5.1202	8.855	7.7551	8.7759	10.1064	10.5871	6.781	14.555	5.8587	9.5598	7.6728	12.2907	12.591	12.1833	8.4167	7.8192	6.1445	1.0633	6.4504
TCGA-55-8208-01	674	0	8.8227	12.0564	3.685	10.3823	11.89	5.6877	6.292	2.3578	11.8342	1.3489	9.4455	5.3835	9.1061	7.9363	7.2143	7.4581	8.5701	6.5678	9.5659	9.8909	9.0628	8.7894	5.0657	8.2837	6.1526	8.3911	8.3113	8.3665	10.0305	10.067	5.1309	14.4433	7.3708	9.8972	4.5974	12.5231	12.4046	11.2635	9.2175	8.1133	4.6868	0.6	6.0993
TCGA-55-8299-01	469	1	9.3009	10.387	2.7893	9.5529	14.326	12.6098	6.7359	1.4273	12.2475	2.1306	8.9244	5.4759	9.6233	6.7012	6.9823	5.189	9.1759	7.8996	8.9839	10.5323	9.0957	8.4834	5.7034	8.3769	4.0917	8.6775	8.2391	8.4332	10.0793	11.4328	5.332	14.5924	7.2338	9.8168	3.1048	12.2995	11.1613	11.0729	8.7613	8.3271	6.0908	1.4273	5.9598
TCGA-55-8301-01	534	0	8.7533	8.8111	4.3165	11.1773	14.078	3.5011	4.9985	4.2528	10.035	1.6556	8.8385	5.6694	10.624	9.2809	7.1062	6.0147	9.0938	7.8817	10.0376	10.247	9.3367	7.3154	6.5331	8.2632	5.4456	9.5228	8.2971	9.3577	10.6098	9.557	6.872	14.0481	6.4645	10.1812	3.5549	12.4214	11.1252	11.5851	7.7893	7.918	2.8976	1.6556	6.5398
TCGA-55-8302-01	478	0	9.9912	8.9736	3.0355	12.721	10.497	3.1298	4.4623	0	8.9036	0.6358	8.6146	5.2932	10.2545	4.6649	7.6544	6.1565	11.0625	7.6974	9.3499	9.3736	9.7218	9.0113	5.3334	7.9392	6.9599	8.9957	7.3461	8.3515	9.6893	8.9317	5.9778	13.3865	6.8325	10.5767	4.7856	13.0687	12.267	12.4693	6.5551	9.1681	2.9346	1.0756	7.6183
TCGA-55-8505-01	440	0	9.3672	11.8713	1.1234	11.3526	14.821	11.93	5.5892	1.1234	9.4774	3.8037	8.8512	8.4433	10.6999	6.538	6.7969	6.8197	9.3775	7.5976	8.8786	7.4835	9.3672	8.431	3.9756	8.7833	8.1034	8.0272	7.5348	8.1669	10.2184	10.377	5.1841	13.4977	8.0042	10.8096	7.166	12.4369	11.8738	11.693	8.978	8.4408	3.7414	3.7414	6.6857
TCGA-55-8506-01	11	0	9.9745	8.2449	3.616	12.5906	12.926	3.616	4.656	1.9318	9.521	1.7015	8.9292	6.8351	10.6139	12.4427	7.3895	2.7291	9.1823	6.9447	9.3466	11.3182	9.5143	8.5739	2.3049	9.0304	2.9555	8.3205	10.237	9.1851	9.8267	8.7709	7.2036	13.2542	6.4547	11.044	5.6914	12.4771	10.8586	12.962	8.6385	9.989	3.2397	0	8.3078
TCGA-55-8507-01	418	0	9.4965	10.4211	2.5534	12.0633	13.232	3.9644	8.6314	4.8914	10.1841	0	8.2248	6.946	10.4376	8.6987	7.6291	5.6967	9.0222	7.3325	9.4657	8.7266	9.5876	8.1108	5.6508	8.4735	6.2724	8.7211	7.581	8.9671	9.8788	8.8263	7.7208	13.6542	6.9957	10.0862	7.7059	11.895	10.8689	11.8472	8.4713	7.6719	3.2717	3.1885	5.133
TCGA-55-8508-01	617	0	9.167	9.0848	1.469	9.3811	13.45	11.969	7.5375	0.914	9.9749	4.5301	8.591	7.5848	9.7166	8.8835	6.9836	5.4985	9.4318	7.6874	8.864	8.2905	9.5039	6.5247	4.7356	7.9787	6.3636	8.6683	6.5923	8.614	10.4619	10.0622	6.6185	14.3673	7.873	10.3023	2.6565	11.4918	9.731	11.0668	8.3965	7.8946	3.0132	1.469	5.441
TCGA-55-8510-01	539	0	9.796	10.4959	4.9536	11.9542	12.356	4.4867	5.4133	8.5458	11.6333	2.7022	9.0935	5.0895	10.129	8.7625	7.1963	6.7885	9.545	7.4597	9.7932	10.2535	9.264	8.5123	5.6249	8.5362	6.592	9.0189	7.6343	8.8339	10.0192	9.6333	7.3503	13.6815	6.6721	10.3848	8.0486	13.0785	12.5491	12.0173	8.7887	8.5075	4.5634	1.5039	7.7205
TCGA-55-8511-01	552	0	10.397	9.4938	4.2417	10.7132	12.511	3.3548	5.5592	0	10.5659	1.6653	9.3702	6.1284	11.138	8.4314	7.5361	4.1118	10.1522	7.733	9.3086	10.6531	8.8902	6.3936	4.815	8.01	4.8961	8.4893	7.8429	8.4292	9.6315	10.1117	3.5679	14.2019	6.6491	10.3117	3.1929	13.1069	10.0846	11.9401	6.8861	7.4491	3.1929	0.6257	8.5842
TCGA-55-8512-01	607	1	11.439	12.4636	3.2557	11.7739	11.061	4.765	4.765	8.7666	11.178	9.3733	8.1619	9.3652	9.6942	7.854	7.81	5.9401	9.664	7.9997	9.4488	9.1114	7.8473	7.8473	6.1609	8.2769	4.5839	8.8708	7.8902	8.9586	9.0603	9.9556	7.2248	14.0546	7.3292	9.5076	7.6919	11.8513	12.3958	11.8311	8.3697	8.3556	3.6734	2.2453	8.4424
TCGA-55-8513-01	791	0	10.831	11.4911	5.5544	10.9654	12.353	5.5081	5.4108	0	10.3784	0	9.017	8.587	9.9392	6.7426	7.5196	5.7461	9.179	6.8204	9.4678	10.489	8.1962	8.825	5.7659	8.6474	6.7123	8.8036	7.9414	8.735	8.9297	10.1077	5.7854	14.085	7.2927	9.7656	6.8578	12.7109	13.4911	11.5697	7.7207	7.7258	4.5395	0	7.7997
TCGA-55-8514-01	520	0	9.1	9.2754	2.0792	9.7946	14.094	2.2842	4.7094	1.8402	8.8904	1.1955	8.035	9.0795	11.8915	8.9099	7.4895	3.7288	8.7655	6.9418	9.0253	9.9607	9.0898	6.3167	6.3739	8.5621	5.6995	9.841	10.963	8.8746	10.9487	12.0361	7.5652	14.0368	5.8032	10.8382	6.1031	13.0401	10.9955	13.5239	9.8918	9.1235	2.0792	1.8402	9.2512
TCGA-55-8614-01	536	0	9.4106	9.9045	2.5919	11.8848	12.056	8.3263	6.8348	0	10.9328	3.1973	8.379	6.4027	10.0176	6.7531	6.6575	6.7531	8.4193	7.9472	9.6632	8.5436	9.4487	8.9878	4.6374	8.1284	4.4003	8.5436	9.4616	8.7889	9.0812	10.7668	6.5743	13.7039	5.8786	9.8874	4.7762	12.3258	10.8099	11.6532	8.8092	9.0196	5.0193	0.7036	6.5648
TCGA-55-8615-01	446	0	10.057	6.9665	2.5392	12.4629	13.449	12.0854	5.4522	1.0493	9.1398	2.6662	9.0982	7.4583	10.6866	8.3611	7.4801	5.6496	9.0231	7.2911	8.6154	6.6582	9.3822	7.8142	4.4499	7.4273	7.4627	8.7954	7.9638	9.1763	10.6968	8.9532	8.1519	13.6951	7.3864	10.2191	3.9976	12.1285	11.1953	12.6179	7.3155	6.9479	4.1789	1.3809	7.1352
TCGA-55-8616-01	48	0	10.082	10.4808	1.6326	13.653	12.974	4.5272	3.7661	1.3649	10.1228	6.2598	8.5199	8.0365	11.8246	9.2117	7.4799	4.9513	8.883	7.5942	9.0299	10.2251	9.0133	7.2848	4.3528	8.4686	6.1889	9.1369	9.3574	8.8089	10.9026	9.7826	7.7431	12.7652	5.8899	10.0287	4.2767	12.535	11.7469	12.7596	7.941	8.1206	2.5178	1.8584	7.342
TCGA-55-8619-01	416	0	9.8327	12.3082	4.4185	10.685	11.169	4.766	5.1909	0.6621	9.8682	0	9.0382	7.6784	10.3194	8.2361	7.8264	6.2723	10.3232	6.7648	9.0274	10.0567	8.3227	8.2719	5.1441	8.4425	6.8547	8.9114	8.0678	8.4068	8.8288	9.7629	5.071	14.406	7.252	9.9664	10.3601	12.5626	13.2703	11.8148	9.7336	7.8375	5.3227	1.458	7.2241
TCGA-55-8620-01	375	1	8.6434	13.9339	3.1275	9.9657	8.3319	5.9616	8.1845	1.4782	8.9654	1.4782	10.3802	3.7551	9.4203	7.1385	8.0222	8.9183	9.2875	6.95	8.6229	7.6254	10.407	8.4358	3.9343	8.3702	8.1211	8.2788	8.0156	10.4653	10.5036	8.9342	7.6934	13.2359	8.0962	10.2718	5.9196	11.6106	10.0055	12.6146	8.9289	8.8	3.1275	0.6738	6.0157
TCGA-55-8621-01	515	0	10.246	12.4833	4.0935	10.8693	11.393	6.3556	5.6209	1.6521	9.7432	2.893	8.839	7.9414	9.9645	6.9252	7.9817	7.4298	9.1654	7.3562	9.7232	10.2637	8.587	8.2387	4.929	8.3912	5.1841	8.9242	7.1043	8.5128	9.7867	9.3673	7.3083	14.5499	6.9504	9.6965	10.3312	12.3452	13.6877	11.6938	8.9572	7.6802	5.9558	1.0506	6.3366
TCGA-55-A48X-01	689	0	10.174	10.1214	3.9235	11.6932	13.413	4.3264	5.5678	0.9837	10.9767	1.5632	9.2847	7.1323	10.0454	8.3957	7.6539	5.7751	9.6815	7.1769	8.5369	10.851	9.0823	8.4082	5.216	8.6997	6.9332	8.6555	7.9925	8.4869	9.4081	10.5792	5.8381	14.0652	6.7423	9.8987	4.8012	11.7306	11.3065	11.4621	8.1297	8.3596	3.1408	0.9837	6.8501
TCGA-55-A48Y-01	630	0	9.4051	10.5871	3.1503	11.093	11.96	16.4425	6.2299	0	12.0987	1.0359	9.1596	4.6226	10.5134	6.3085	6.1252	2.7607	8.7782	7.5544	9.1493	8.6215	9.1583	9.1583	4.7121	8.5998	6.8517	7.6252	8.223	8.5786	10.6564	10.257	3.9242	14.7249	6.5859	10.3678	5.1564	12.3766	11.87	11.14	8.0679	8.5526	4.5915	1.0359	7.1473
TCGA-55-A48Z-01	651	0	9.3373	8.8691	3.4714	9.9776	13.161	6.1135	5.9549	1.2878	9.7685	5.4676	8.7899	8.0541	10.6429	7.5392	7.9791	5.4035	8.9244	6.5799	9.2773	9.5876	9.5947	7.7536	5.6987	7.7489	5.5289	8.8795	7.2423	8.664	11.609	9.8497	5.0106	14.1123	6.6298	9.8504	3.1968	12.1021	10.421	11.9155	8.4064	7.8972	3.9464	0.9717	6.378
TCGA-55-A490-01	99	1	8.8379	8.4177	3.7334	11.2786	13.549	3.4383	5.5622	1.308	9.368	0.5772	7.6669	5.5164	10.1949	9.4383	5.3172	7.7451	7.5353	6.4033	9.089	8.6207	9.4104	8.608	4.4373	6.42	8.7793	8.0048	6.6635	8.051	9.9936	9.3144	6.9599	13.8349	4.9083	9.5608	7.4607	12.3064	11.8264	13.6717	7.5656	10.5979	2.3034	0	3.679
TCGA-55-A491-01	626	0	9.4831	7.9524	4.5707	10.5412	13.072	2.6539	6.1256	1.0418	10.3809	1.0418	8.793	6.3851	10.5757	7.5165	7.5858	5.5235	8.5395	5.7673	8.4056	9.5106	9.2597	8.1455	5.4023	8.0176	8.0363	8.8863	8.6839	9.147	11.8547	9.6137	5.4728	14.3069	6.4123	10.6186	4.5382	12.8569	11.4617	11.9456	8.5559	8.0304	4.5049	1.0418	6.2711
TCGA-55-A492-01	596	0	9.9556	15.8162	3.7923	11.8571	10.026	14.9226	5.3636	0	7.4824	0.6188	7.2019	4.1811	10.8895	8.5584	6.6374	7.2123	10.1312	7.9475	10.6501	7.5499	9.5809	7.491	6.2976	7.822	7.6875	8.6028	9.648	10.0786	10.4318	8.7838	9.2963	13.9732	7.9997	11.1811	5.9556	13.1958	13.4063	12.7678	9.3159	10.3863	3.3371	1.879	7.2433
TCGA-55-A493-01	28	0	10.096	7.2731	2.1353	10.0508	10.033	4.0872	6.9041	0	9.4412	0.9778	9.7368	4.5719	9.6935	8.1625	7.4515	7.3522	10.1673	7.2503	9.0064	9.818	10.2824	8.5975	4.2795	7.5249	6.7759	8.6902	6.4235	8.421	10.36	9.1758	5.3827	12.6132	6.7631	10.4911	4.1673	12.2887	13.8767	11.2554	6.4235	7.3307	3.7136	0.5703	7.7565
TCGA-55-A494-01	481	0	10.311	5.1889	2.0371	7.2832	12.881	2.0371	4.6408	11.893	11.2953	2.6603	9.0391	8.1762	11.3726	7.3977	8.7916	6.6423	9.2618	7.0031	8.4112	8.5922	9.4121	7.1454	2.7581	7.8285	8.793	9.0775	8.1403	8.0683	11.022	7.1272	6.2553	13.7672	7.9696	9.7249	3.094	12.0084	7.9594	11.4925	7.6479	9.7556	3.9251	0.916	5.4006
TCGA-55-A4DF-01	614	1	9.3029	13.7882	2.4914	10.4781	15.261	4.724	7.7732	0	9.5027	0.9446	9.7294	9.1051	8.1729	7.0171	8.5526	8.1281	7.8241	7.7943	9.2199	9.8347	9.7168	8.271	4.868	7.79	2.9016	9.1551	5.316	7.7883	10.145	8.5383	6.6295	14.3883	6.0282	8.9261	4.4156	10.736	11.0488	10.5888	8.2642	8.6015	2.711	0.5482	5.2821
TCGA-55-A4DG-01	608	0	9.7547	9.7843	3.317	10.8053	9.7068	4.6284	4.7746	6.8312	11.7182	3.2386	8.708	9.3296	11.4401	10.1724	8.905	6.0416	9.6907	8.1616	8.9381	11.0348	8.0626	7.5483	6.5274	9.0012	6.9406	8.8986	9.353	9.1815	10.5663	9.1227	7.2006	13.8786	5.6754	9.6487	5.1623	11.5651	11.1461	11.8149	8.3483	6.7558	3.0676	1.8628	7.8416
TCGA-55-A57B-01	546	0	10.219	9.6432	3.8828	10.0574	11.514	8.0233	5.9126	2.0613	9.4216	1.2693	8.7662	6.42	10.2344	7.8064	7.3474	5.6134	9.6685	7.2737	9.38	10.6819	8.5498	8.0733	5.1803	8.8078	4.9364	8.5252	5.7425	8.6696	10.6997	10.8112	7.4758	13.8127	6.5342	10.0759	6.0874	12.2786	12.0111	11.9011	8.0389	9.8738	5.5387	0	7.4758
TCGA-62-8394-01	139	1	9.5562	8.699	3.88	11.6149	12.986	2.6794	4.7432	7.3613	10.4369	2.3669	8.9008	10.0708	10.8777	9.3936	5.8364	4.8511	7.7465	7.3392	9.7598	9.311	9.8481	9.7767	5.027	8.8666	8.2025	8.8626	8.5421	9.1744	10.7673	9.4148	6.0047	13.4262	6.984	10.1213	4.8717	11.9855	12.0367	11.2553	8.3047	9.5846	4.0706	0.5016	5.1334
TCGA-62-8395-01	1216	0	10.358	11.5219	3.471	12.4978	9.9986	4.651	5.5428	3.0801	9.3735	1.4618	8.7228	6.1174	10.2859	8.3585	7.2551	4.651	9.586	7.4231	8.2138	8.8802	8.498	8.4152	4.7947	8.2484	7.9333	8.3623	7.6257	9.1254	10.4382	9.8126	7.2919	13.9018	6.846	10.2359	4.9254	11.9689	12.9334	11.99	8.4536	9.2707	5.242	0.5247	7.5365
TCGA-62-8397-01	1289	0	10.501	12.6179	3.9633	10.1691	5.9482	4.2109	3.2867	0	9.004	2.4275	8.2919	6.8438	10.9907	8.6786	6.4691	5.4375	9.7447	7.2695	9.7442	12.4025	7.8283	9.103	5.9949	7.9088	7.8739	9.7249	9.3063	8.6889	11.0661	11.8872	7.0486	13.1147	7.0801	9.5279	7.3361	13.9769	12.7153	12.6624	7.961	8.8708	3.2867	0	6.2093
TCGA-62-8398-01	444	1	9.7434	11.1443	0	8.8361	13.614	14.4181	6.018	0	10.3764	0.8079	8.8585	4.3587	10.9523	6.0264	7.7275	7.7678	9.7912	8.2204	8.6611	9.1347	9.527	7.627	4.0346	7.95	6.4911	8.1497	8.4328	8.3237	10.4186	9.9164	5.6807	13.6097	7.4525	10.2817	5.9141	12.4744	11.3961	10.545	9.324	8.7047	5.477	6.4233	6.9771
TCGA-62-8399-01	2696	0	8.9445	9.4888	2.5648	12.3881	8.616	11.7549	6.0538	4.8384	12.8607	2.4695	8.4469	7.5591	9.3368	9.1913	7.6811	5.2788	9.2913	7.8132	8.1305	9.0399	8.2369	8.1435	3.7529	8.5658	8.4024	8.4578	9.2641	8.0416	10.3233	9.3095	6.4716	13.844	6.2952	9.865	5.1769	11.3963	12.5641	11.503	7.8059	7.1543	5.3203	0.8126	6.0782
TCGA-62-8402-01	1498	1	9.2373	8.0039	1.3092	8.2291	14.592	2.1534	4.9336	0.5779	8.705	0	9.7502	5.0878	10.0519	7.0503	8.0094	6.8492	8.778	7.747	8.8421	8.905	9.9344	6.4302	4.1092	8.3146	5.4549	8.5256	8.4766	8.5953	10.308	8.7403	6.9845	13.5754	9.2183	10.3726	3.151	12.2599	10.5328	11.3683	8.1678	8.7748	3.6808	2.3049	3.7352
TCGA-62-A46O-01	1454	1	10.808	11.9968	5.493	10.5016	13.244	16.0675	3.7293	1.0805	10.2402	0	9.8335	6.7402	10.3269	8.084	7.86	1.0805	8.0204	6.7175	8.9459	5.7537	10.2782	9.1352	2.8342	9.0828	5.1062	8.2088	8.0142	7.5738	10.6105	6.4859	5.5283	14.236	4.3582	10.5576	2.8342	10.3045	12.1155	10.6511	7.6678	7.5611	2.4487	3.9007	4.1922
TCGA-62-A46P-01	594	1	9.8962	6.9044	4.0034	12.2466	12.107	4.1327	6.0857	1.3241	8.5379	2.8104	8.3479	9.1457	12.126	9.0811	7.176	3.5269	9.1816	7.6016	8.779	8.5895	9.3272	6.949	6.0204	8.1173	8.4002	9.1854	9.258	7.4914	10.698	8.7264	7.3523	14.202	8.0616	10.7219	5.596	12.9004	10.4304	12.8801	8.8373	7.4236	2.9573	2.2508	9.0239
TCGA-62-A46R-01	1725	1	10.512	7.5124	3.5241	11.0888	9.9699	2.701	3.5241	1.5854	9.9128	1.3223	9.8015	4.3582	10.2331	7.2485	8.3516	6.0986	9.9351	7.6153	9.7094	10.0498	9.0409	8.532	4.8832	8.7828	5.8954	9.5124	8.1091	8.1091	11.3694	8.3067	6.7688	13.44	5.6153	10.137	6.7011	12.0796	11.1618	11.9453	8.4865	8.0477	2.701	2.1704	6.5931
TCGA-62-A46S-01	1653	1	10.37	8.143	4.6088	11.6277	14.118	13.3088	5.4243	2.0323	8.4412	0	9.0508	6.636	12.7452	6.2573	7.3645	6.9094	9.8739	7.0264	8.5852	8.1698	8.2452	8.8095	4.9957	8.1207	7.5994	8.8152	8.1157	7.9986	9.8603	8.7736	6.3304	14.3087	7.1071	9.8881	2.1801	12.1632	11.0926	11.3089	8.6299	6.636	5.3005	0.913	5.644
TCGA-62-A46U-01	2067	0	9.3651	10.9522	5.1674	10.7363	14.557	2.465	7.3518	0.8655	11.5948	0.8655	9.7558	5.3674	9.5035	8.1878	6.6564	7.8112	9.144	6.5592	9.0929	10.6111	9.0698	9.2713	5.1508	8.5591	5.4779	8.6006	8.2475	8.0603	10.4711	9.6608	6.6327	14.1995	6.3857	9.7482	6.6026	12.6449	11.6409	11.8119	9.7162	8.5269	2.465	0.4967	7.0093
TCGA-62-A46V-01	2199	0	8.8856	9.1889	4.3473	10.7154	9.0139	4.5133	6.9141	0.5814	13.333	5.9083	7.955	8.9006	10.9702	9.4941	5.5894	3.9441	8.184	8.1717	9.5519	10.569	8.7673	9.465	4.7176	9.4841	7.4322	9.2793	8.9507	8.8284	9.8931	8.4363	8.4446	13.664	5.8349	10.3523	8.1365	13.2919	12.5496	12.9104	8.0634	9.328	3.2383	1.7997	6.424
TCGA-62-A46Y-01	414	1	10.328	10.3732	4.2888	9.7668	13.947	4.1327	5.0259	1.3241	10.6542	0.5862	9.019	6.8862	10.0719	8.3981	7.383	6.213	9.138	8.0589	9.6068	11.3042	8.9225	8.5848	6.4214	8.9143	4.558	9.0036	8.3345	9.1251	10.6302	9.7349	6.2704	14.1016	6.535	10.0301	7.1016	12.7684	11.306	12.221	7.5582	8.949	3.4627	1.3241	7.9343
TCGA-62-A470-01	1194	1	10.372	12.0283	5.149	10.0962	12.402	14.5915	7.3645	0	8.4288	0	9.0175	8.1758	10.2796	8.1697	8.0203	6.5448	9.7554	6.8521	9.364	8.8154	9.257	7.6651	5.3366	7.8531	6.4666	8.6483	8.2301	7.9996	10.672	7.7251	5.7204	13.9358	7.4884	9.7533	6.5448	11.8429	12.3785	11.5601	7.8765	9.1072	4.0883	0	7.2525
TCGA-62-A471-01	1246	0	9.5524	12.6417	2.0247	8.7776	15.183	14.7347	4.6462	0	9.0648	0	9.1439	6.5794	11.0577	5.6904	6.6324	5.0318	9.5306	7.875	8.8723	7.488	10.4856	8.6252	5.2218	8.1689	8.0551	8.1281	9.7927	8.9971	10.0559	8.7105	4.8646	13.2537	6.2708	11.0888	7.412	12.5129	12.2909	12.4041	9.5216	8.796	2.9354	6.8843	4.9386
TCGA-62-A472-01	910	0	9.8117	7.7566	3.1247	10.4409	12.326	2.5426	4.1996	0.9747	10.2539	0.9747	8.7403	5.038	10.9768	7.6771	6.6637	7.1972	8.2112	7.8561	9.2984	10.6161	9.2041	9.3648	5.1203	8.3488	5.8079	8.6251	5.3764	8.0629	12.2796	9.0179	8.3111	13.8099	5.6779	9.3627	6.4731	12.5231	11.4848	12.2553	7.5931	8.7977	3.9522	0.9747	7.9034
TCGA-64-1676-01	1728	0	10.382	7.356	4.082	11.2295	14.862	2.9871	5.609	4.7353	10.8734	4.249	10.9895	2.7099	10.441	8.1898	7.1602	7.2809	7.9841	8.8248	10.2002	11.58	10.5498	8.0115	4.1955	9.6394	2.9871	8.8831	7.8433	8.6665	10.7418	9.9549	4.1955	14.3104	6.1064	9.8731	3.7518	14.4443	12.8483	13.3567	7.4562	9.5693	3.2195	0	7.6577
TCGA-64-1677-01	628	1	9.1574	5.6188	2.0984	11.6479	14.006	3.1141	4.5161	0	9.4672	5.0325	9.7108	8.1572	9.5412	7.7364	7.3187	6.4319	7.773	6.8009	9.8192	9.9208	10.1268	7.4953	6.194	8.491	5.9088	9.0483	9.6334	9.7258	11.0283	9.7089	7.3187	13.4124	7.1841	9.8108	3.8206	13.1054	10.3835	12.9539	8.19	9.4694	3.5778	0	5.8553
TCGA-64-1678-01	1189	0	10.225	10.3629	0	8.1725	8.1206	12.1625	8.2545	0.9535	9.3629	1.5226	12.1189	6.3495	8.8334	8.8743	7.5854	6.9722	7.2983	7.1915	9.6814	6.5646	11.7136	6.2642	3.9986	7.8366	4.9085	8.2937	9.3506	9.2565	10.6291	8.3193	6.4914	12.6505	6.0966	9.5833	1.9296	12.4376	10.2093	11.5906	8.0757	10.4505	3.6134	0.9535	6.1735
TCGA-64-1679-01	2488	0	9.4291	9.8171	2.8844	11.792	14.669	5.486	6.5209	1.5999	10.365	6.2232	8.773	9.2969	9.4207	9.6986	6.4412	4.5087	8.934	5.9255	8.8004	10.9352	8.9046	9.7574	4.6654	9.1066	5.4767	7.1412	8.0938	8.2871	10.2647	9.9299	6.1543	14.4985	4.8067	10.3754	3.0452	12.4277	11.2061	12.1253	8.3805	9.244	4.527	0.6603	6.6722
TCGA-64-1680-01	1126	0	10.563	10.3843	2.6396	9.5171	13.913	2.803	2.803	0	9.8483	0.8052	9.4228	6.606	10.4856	7.5986	7.3959	5.2897	8.8981	8.097	9.1472	11.7042	9.4291	8.2746	5.542	9.1576	4.2432	8.8239	8.3722	9.2226	10.1387	11.7301	8.7975	14.5769	6.17	9.7096	4.639	13.2306	12.06	12.3309	8.7482	8.8707	3.083	1.697	6.5611
TCGA-64-1681-01	1167	1	10.548	10.9635	2.908	12.1938	14.016	3.5213	4.5265	0.4451	9.8412	1.6638	9.1212	7.6605	9.928	8.4323	7.0147	6.9188	9.1445	7.8822	9.4187	9.732	8.4915	8.4443	4.3588	8.8842	7.548	8.4725	7.4289	8.5357	11.0133	10.23	8.6989	14.4989	5.7584	9.6991	5.9307	12.5627	12.0118	12.1955	8.9879	8.3356	4.9709	1.0596	4.9372
TCGA-64-5774-01	2676	0	9.3444	7.1577	2.8244	9.5211	12.792	13.3024	8.3865	0.5538	10.0725	1.7397	9.9255	9.3619	9.1961	7.6053	8.0621	6.4133	9.3022	7.6664	9.5506	7.8648	9.7741	7.6191	6.1818	8.5302	5.5358	8.9814	5.7006	8.1947	9.6702	9.0441	7.1765	14.0585	6.2893	10.0549	2.246	11.5081	9.2698	10.5025	8.3804	8.6925	3.7188	0	5.3824
TCGA-64-5775-01	62	1	5.2875	5.0656	3.3621	6.2248	3.7053	8.9802	3.7582	0.55	8.9842	0.55	9.8735	0.9473	9.4051	8.7906	6.3776	5.8716	6.7943	7.1833	9.3922	10.1582	9.6936	6.9213	4.1836	8.9385	4.3893	8.7398	7.038	8.5002	8.7554	8.5811	1.2584	12.9685	5.9603	9.319	6.2248	12.7218	11.7923	11.5348	4.8269	6.1794	2.0871	2.61	6.3117
TCGA-64-5778-01	1305	0	9.0952	8.579	4.0948	10.6521	13.918	5.0293	3.1349	0	10.8513	2.0404	9.3401	6.2818	9.9199	6.7605	7.7469	5.5761	8.355	7.1031	9.796	9.937	10.5304	9.2908	7.1928	9.0525	7.1193	9.5634	8.9347	9.1828	11.5507	8.6004	6.3568	13.9912	6.1822	10.216	4.9345	13.6898	11.8393	12.779	8.3319	8.8721	2.5035	0.603	8.0393
TCGA-64-5779-01	864	0	9.8455	9.2406	3.6388	8.9329	9.2289	9.281	8.1168	0.8626	11.5508	1.3987	9.5217	3.5408	9.9046	7.8818	8.304	6.4004	7.955	7.1135	8.5755	8.7475	8.8611	7.1802	4.5295	7.447	6.3721	8.2035	6.0367	8.384	10.3602	9.7852	7.5825	12.8213	7.7672	8.999	2.5632	10.4097	10.4591	10.6903	8.5124	6.702	6.1407	1.3987	5.4173
TCGA-64-5781-01	1559	0	9.5387	10.0983	2.2805	10.0932	14.295	11.8742	5.7819	0	9.4164	3.4969	9.1338	3.4969	10.5817	9.4245	6.1647	7.3606	8.7197	7.9501	8.7652	9.8292	10.0474	8.4938	4.2964	9.0409	3.921	8.1658	8.1625	8.3189	10.8361	10.1424	3.793	13.5578	5.5241	10.6678	6.0849	13.5526	11.7467	12.5688	8.9472	7.9688	2.4599	1.193	8.5715
TCGA-64-5815-01	866	0	9.5932	10.1278	5.3319	10.7153	12.161	6.5739	6.2408	0.8363	11.812	4.9835	9.5195	4.129	9.6352	6.1952	5.1523	7.2611	8.1205	6.4103	9.5051	10.2104	9.1015	8.6097	4.129	7.8298	5.9786	8.5595	7.6589	8.4042	10.2417	11.1988	6.5007	13.9084	5.9786	9.9083	8.8098	12.3385	10.9414	12.1975	7.6922	8.5383	5.3319	0	5.0872
TCGA-67-3770-01	610	0	10.441	11.5701	2.922	12.4166	10.921	0	2.731	0.9559	9.1005	0	10.1597	8.3865	11.071	8.778	6.3378	6.6662	8.8176	6.321	9.9809	9.6041	10.3029	8.0857	5.2703	8.895	6.2336	8.8116	9.3924	9.2076	10.3125	8.8266	10.3221	13.8149	5.8892	9.9007	7.9877	14.5985	14.2746	13.1383	8.0246	9.4614	4.4993	0	6.3873
TCGA-67-3771-01	610	0	9.4673	14.0335	2.9097	10.1144	14.813	3.8104	10.5564	4.165	10.5376	2.7789	9.9146	10.2253	9.7102	7.9307	7.5035	8.7206	8.558	6.557	9.1501	9.1359	9.0194	8.8545	3.9985	8.8021	6.4854	7.9799	8.6262	8.6333	9.8221	8.7816	7.4126	14.4152	6.0617	10.8114	3.4292	11.3005	11.8825	11.8692	9.1996	8.673	5.1771	0.7238	7.1535
TCGA-67-3772-01	573	0	10.53	10.0287	1.7402	10.518	12.694	4.1582	5.4464	0	11.826	6.988	10.3193	8.9235	10.4228	7.5386	6.9557	6.2986	8.8522	7.4134	9.3703	9.9213	9.6433	7.5709	4.6636	8.9039	3.9983	8.5671	8.302	9.1662	9.5978	10.4213	8.8092	14.1812	6.255	10.4545	7.9167	13.1432	12.5348	12.2884	7.8885	9.322	3.8185	0.554	6.453

TCGA-67-3773-01	427	0	10.873	9.1479	3.9438	11.186	14.746	4.037	5.3961	0	12.8157	4.43	9.8771	3.3575	9.9436	5.1258	6.4985	7.0945	7.8075	7.7117	10.0683	11.3415	9.2537	9.8692	4.2854	9.8865	1.6114	8.5192	7.8075	8.3015	10.7674	8.8338	7.2966	13.0374	5.7112	9.1993	7.1685	13.9201	11.958	12.5376	7.8659	8.7775	4.2854	0	6.0389
TCGA-67-3774-01	385	0	10.693	10.3938	1.8297	11.1158	11.507	11.6089	6.8811	0.7126	10.0798	0	9.4506	6.3707	10.5205	7.6866	7.7564	6.809	9.4806	6.7331	8.7268	9.5886	8.4474	7.8984	4.138	7.9733	6.2906	8.2067	8.0796	8.2587	8.4631	9.0266	7.4061	14.0986	5.9316	9.1123	6.0751	12.1206	12.9914	11.6884	7.8352	7.345	5.5928	0	7.5847
TCGA-67-4679-01	448	0	11.413	10.2284	1.1027	10.9787	10.225	4.4745	4.5113	0	9.877	2.1515	8.8312	6.656	10.2928	8.2091	7.2633	5.2153	8.9779	7.9493	9.2191	10.7033	8.3169	7.7637	5.3834	8.4741	5.2592	9.1393	8.4625	9.1096	10.7679	9.7022	7.9593	13.3404	6.8261	9.3881	6.4153	12.1739	12.6023	12.0917	9.2858	6.7966	5.8532	0	6.0781
TCGA-67-6215-01	174	0	10.114	8.9691	3.6491	10.5515	9.3983	3.313	2.7988	1.316	8.8887	1.0822	8.7332	5.4415	10.1358	9.7435	6.859	5.5939	8.42	8.4541	8.9124	12.0934	8.6203	7.4195	4.7783	8.442	6.2021	8.7167	9.11	8.8391	9.8608	7.9363	8.4618	14.3827	5.2849	9.5944	7.7316	12.3726	10.8133	11.7322	8.2601	8.0782	4.5451	0	6.9312
TCGA-67-6216-01	141	0	9.7944	9.8799	4.5482	9.1133	14.841	2.7853	3.6086	0	10.5836	1.7473	8.7103	5.4011	10.2229	5.0106	5.4983	4.4736	8.7758	8.0339	9.5572	11.7659	8.7144	8.696	6.6355	8.3411	5.3182	9.2466	9.0066	8.554	10.4182	10.8536	6.0535	14.7167	6.353	9.7848	4.6191	13.1575	13.2313	11.6899	7.7983	8.256	3.6086	2.3577	5.3808
TCGA-67-6217-01	422	0	10.307	10.0062	3.4898	11.248	9.5281	4.3047	5.2024	0.6495	9.7272	1.0957	8.783	6.2596	9.5241	10.026	7.4835	5.4663	9.6291	7.2556	8.5388	11.4468	8.2749	7.9855	4.8791	9.1768	6.0025	8.7202	8.8088	8.7222	10.8237	10.0086	7.1088	13.513	5.8544	10.011	5.4477	11.8281	11.8622	11.6486	8.6299	7.598	4.3047	0	6.898
TCGA-69-7760-01	202	0	10.531	8.9998	3.0867	7.8969	14.359	4.3915	6.3828	0	7.9195	2.2472	9.1326	3.6857	9.1359	7.7829	7.9956	8.6353	7.0932	6.8016	8.8425	11.684	8.3589	7.9195	5.5803	8.5701	6.35	7.8352	8.2476	8.2177	13.1841	8.2972	5.3345	13.3568	5.0652	9.3594	4.5148	12.6159	10.9053	11.7308	8.0912	8.0611	2.8572	0.7001	2.0437
TCGA-69-7761-01	186	0	10.545	8.6017	3.6245	6.9101	12.511	4.4974	4.3138	0	9.8422	3.4214	10.0916	6.5519	9.6239	7.8778	8.213	5.8764	9.0078	6.7015	8.2756	10.8553	8.353	8.7591	5.6778	8.972	7.4269	7.9362	5.1068	7.5893	9.2172	9.0123	4.1035	13.0748	7.6292	8.9941	8.6892	11.5024	10.0562	10.2087	8.6334	6.9036	4.7784	0.6226	5.8896
TCGA-69-7763-01	690	0	9.8568	10.4946	2.5182	12.9914	9.8461	10.1772	6.7228	0.67	10.237	2.5182	8.6545	6.6312	11.1731	6.8975	6.786	5.6793	8.9672	7.0469	9.2148	9.2936	8.5239	7.7987	5.4252	8.4957	6.0188	8.2264	8.0349	7.9164	9.0042	9.3845	5.257	14.2959	5.9788	9.4322	7.6368	11.7065	12.7468	12.147	7.9339	8.3201	6.7937	0.67	7.1704
TCGA-69-7764-01	414	0	9.9624	8.7943	2.6565	12.003	9.0316	13.9222	4.1357	0	9.3389	0	8.6636	6.8295	9.7222	8.4116	7.5597	4.7114	7.7803	7.3587	8.9376	9.0467	8.6939	7.9677	5.6072	8.4235	6.7781	8.5041	7.1182	8.2292	10.7789	8.5354	7.0549	13.8237	5.3024	9.7819	3.4525	10.9708	10.9949	11.2887	6.8295	7.7976	5.3501	2.6565	3.5373
TCGA-69-7765-01	165	0	9.6166	10.2769	2.2465	8.718	14.852	5.929	5.3111	1.8061	12.4345	5.5605	9.1057	4.3021	9.6831	8.6856	7.9947	5.5021	7.7107	7.0923	8.1341	9.6684	8.4484	8.2016	3.3734	8.778	5.7227	8.3851	8.3302	7.782	10.4741	8.7991	6.083	14.1619	5.091	9.7965	4.7992	11.7609	11.0346	11.7158	7.5897	7.0521	4.0536	1.1689	5.5218
TCGA-69-7973-01	230	0	9.1408	8.9576	1.7321	8.2128	12.036	11.3134	6.4373	0.3311	10.4915	2.4293	9.1553	9.5914	9.4706	6.9818	7.5629	7.349	7.8151	7.3694	9.4449	8.6133	9.1229	7.6582	4.5671	7.69	6.2976	8.9673	6.8843	8.1355	10.0761	9.2371	8.3996	14.0368	5.6812	10.021	4.4528	11.2884	10.7398	10.4546	6.833	8.5719	5.8649	1.0229	5.4107
TCGA-69-7974-01	184	0	10.094	9.3758	3.9991	10.5678	10.543	4.7476	4.8094	1.4665	12.7978	1.4665	9.4279	5.991	9.7899	7.0357	8.4312	5.9298	8.598	7.2486	8.8598	10.9811	9.0323	7.5963	4.2866	8.3865	2.6532	8.5007	7.4069	8.2757	9.6065	8.1998	7.5221	13.8445	6.2963	9.7873	4.7476	13.0933	11.5718	12.2477	8.4937	7.3715	5.4273	0.667	6.9002
TCGA-69-7978-01	134	0	9.8803	8.9599	5.2474	11.29	14.602	8.9373	5.9272	2.332	11.2525	1.4229	9.4346	5.1954	9.0473	8.075	8.1158	6.5149	9.365	7.304	8.6063	10.8829	8.8338	9.0116	4.427	9.0504	6.0048	8.7596	6.5149	8.2397	8.9144	9.6352	4.0707	13.7378	6.1485	10.1728	3.5965	12.2769	12.2324	11.5333	8.0696	7.5439	5.1277	0.742	6.1279
TCGA-69-7979-01	408	0	9.4229	8.9276	3.8706	13.6978	14.604	3.5157	5.2631	6.9296	9.2923	4.9066	9.0037	9.0861	10.3473	8.3552	7.2945	3.094	9.1308	7.5547	9.4723	10.6716	9.7366	8.3868	5.138	8.0306	6.3608	9.3721	9.7753	9.172	10.9981	11.5952	6.0684	13.38	6.774	9.7479	4.058	11.5327	13.2412	12.2948	9.32	8.5564	1.9633	0.3673	7.6985
TCGA-69-7980-01	362	0	11.101	9.075	5.051	10.9455	12.651	4.8359	4.5265	0	9.1989	0	9.2864	6.1829	9.1574	7.4148	7.2476	7.4379	9.9598	7.3212	8.8564	9.7383	8.325	8.4363	3.8245	7.7601	8.3482	8.542	7.23	8.6657	9.9744	9.3467	5.1906	13.848	6.6601	10.0451	4.8512	12.2169	11.0182	12.1375	7.6151	5.9311	3.7277	2.3163	5.7705
TCGA-69-8253-01	426	0	9.8394	10.6705	0.6521	11.3729	12.442	5.3972	4.9408	2.1468	9.0416	0.8931	8.2472	5.3575	10.9373	8.6089	6.6825	3.987	9.8218	7.8852	8.8692	8.7762	8.7245	6.1085	4.7395	8.5221	4.858	8.9858	8.3303	9.2738	9.5075	8.9421	4.8	14.115	6.6126	10.0469	5.7587	12.3898	12.8659	11.975	9.3138	8.0591	3.9069	0	7.2654
TCGA-69-8254-01	409	0	11.254	10.6757	3.0673	9.3408	13.868	5.0968	5.1174	0.5774	9.9179	1.4449	8.6889	9.6224	10.4276	8.2394	6.6068	4.454	9.5342	6.4773	8.8791	10.7682	8.2947	8.3447	5.4207	8.4117	7.5623	9.111	8.2287	8.5892	9.2649	9.1836	8.0766	14.4381	7.1195	9.9083	5.5091	11.4668	11.2905	11.7917	8.4025	8.1422	4.3706	3.6229	5.6641
TCGA-69-8255-01	129	0	7.8925	11.334	9.2739	9.0686	17.326	16.4847	3.753	7.8687	8.5515	2.8283	9.7465	3.4207	10.2575	7.1657	7.7949	5.5897	8.6722	8.2156	9.3211	10.7426	10.0631	7.6923	5.4593	8.7852	7.9258	8.8639	9.1248	9.0216	11.1242	7.2885	7.174	12.8036	10.639	11.0046	4.831	10.6879	10.9854	12.349	8.5956	8.9012	3.4936	6.856	5.9156
TCGA-69-8453-01	813	0	10.07	11.5712	2.8749	10.6243	11.104	5.4716	5.2858	0	10.2403	1.2998	9.082	9.2169	9.9947	6.9801	7.4789	6.815	9.3217	7.2532	9.8728	10.3787	8.6842	8.2508	5.4233	8.3549	6.7182	8.8834	7.5668	8.691	9.7146	10.0597	6.2208	14.6592	8.069	9.7904	10.3856	13.2229	13.0244	12.072	8.5722	8.7229	4.7192	1.5604	7.1727
TCGA-69-A59K-01	522	0	10.892	9.1262	2.7082	10.039	10.148	6.2945	4.1379	4.0866	9.8061	1.157	9.6503	6.8883	9.3178	7.071	8.4652	8.3691	9.4158	8.1617	8.5236	8.3957	9.2095	8.7938	5.9515	9.1598	6.0078	9.4991	7.8403	8.143	11.011	8.8583	6.5899	13.9412	5.5008	9.5101	3.5934	12.3418	12.4531	11.4537	8.529	7.3573	4.607	0	5.4408
TCGA-71-6725-01	256	0	10.448	5.6879	3.4978	12.8509	11.723	2.5831	3.0315	0.6996	8.1671	0	8.4194	8.8435	9.8815	8.527	7.5963	2.6564	8.5832	8.5147	8.8044	11.1797	8.8764	5.5312	4.7326	8.2103	6.2218	8.4506	8.3465	8.8697	11.4019	12.3254	7.4725	13.5605	6.3265	9.9542	4.1324	12.4021	9.7524	12.2377	8.9002	8.3753	3.9695	0	8.4544
TCGA-71-8520-01	210	1	10.244	8.3781	2.9672	11.2999	14.151	4.3377	8.9821	0	10.8633	7.1408	9.4209	4.9136	9.6024	8.2807	7.0626	6.5828	9.4897	7.4116	9.0462	9.9247	9.6102	8.4967	3.3645	9.0342	8.4437	8.0267	8.0772	8.4591	10.9701	9.7172	8.5597	13.955	6.564	10.4153	4.0444	12.4611	10.3262	11.6168	8.2488	8.8851	3.9317	0	6.3722
TCGA-73-4658-01	1600	1	9.8069	10.6219	3.084	10.793	13.431	5.1271	5.8134	0	10.9727	1.9549	9.8686	4.362	10.587	8.3942	6.1183	4.8389	8.2353	6.5926	8.7173	10.1145	8.787	8.0535	4.5509	8.4868	5.054	8.6114	8.4016	8.227	9.9935	10.1854	5.2191	14.242	6.4385	9.4957	4.686	13.1993	11.7024	12.4173	8.326	9.5762	6.3188	0	7.3712
TCGA-73-4659-01	711	1	9.3484	11.1102	5.4537	12.3931	12.337	14.5329	6.9017	0.2762	10.442	0.5078	8.911	8.7443	10.0208	9.1802	7.8528	6.8525	9.517	7.6235	9.4237	10.5774	9.5796	7.2762	4.7967	7.8701	5.2042	8.9398	7.6556	8.3018	9.597	9.8573	6.4616	15.3954	6.1761	10.0433	4.9224	12.8161	12.3241	11.6703	8.0051	8.5807	6.2698	0.7074	8.0869
TCGA-73-4662-01	2515	0	9.773	9.1741	3.3086	9.0546	9.7677	3.8676	5.3103	0	10.8089	1.4011	9.3002	4.1023	9.6334	9.0107	8.0342	5.7792	9.4007	6.5735	8.6315	9.543	8.5386	7.3774	3.8906	9.1433	6.38	8.4924	8.0148	8.8022	10.8792	9.3937	7.3298	13.0159	5.8157	9.4112	7.0007	12.0329	11.6471	12.4719	8.0687	7.0828	4.6113	0.7683	5.4942
TCGA-73-4666-01	800	0	10.417	7.6776	3.2558	9.1413	12.273	3.2558	4.4007	0.3237	9.3833	2.2549	9.7303	2.7079	8.6455	5.4843	7.4966	7.8177	8.5479	7.8337	8.616	10.6497	10.1121	8.7934	4.2751	7.8546	2.0942	8.7256	6.6525	8.2767	9.6553	9.0809	6.6705	14.3847	6.7468	10.0477	3.4342	12.829	12.9916	11.9293	7.8558	9.7544	3.6228	0.8111	7.4475
TCGA-73-4668-01	467	0	10.112	8.8191	1.7908	11.9626	14.018	7.239	6.6912	0.3173	9.9196	4.7325	8.6772	3.4707	9.1532	9.3686	7.7183	5.6567	8.3745	8.0456	8.6797	10.7977	9.2908	8.9626	4.3702	8.6532	5.2347	7.7906	9.0212	8.0212	9.6992	10.2176	6.9311	12.959	5.4367	10.2842	4.3178	11.9989	11.1803	12.2163	8.3594	7.7664	3.9782	0.5772	6.6208
TCGA-73-4670-01	131	0	9.6687	10.1524	2.9696	8.2806	14.929	13.858	5.2019	0.3486	10.0792	2.0976	9.538	4.3702	11.1231	8.4903	5.1249	6.8035	8.1984	7.7093	9.2625	8.6062	10.2306	7.3325	2.1869	8.4692	4.1435	7.9627	8.5878	7.942	11.0648	9.6774	3.1133	13.6085	6.807	10.6155	4.3891	13.2883	11.2252	12.1841	7.5522	9.5262	3.9782	0	7.5501
TCGA-73-4675-01	922	1	10.289	9.2059	5.0937	11.4539	11.443	3.9598	5.2069	0	9.9556	4.2455	8.8203	6.9932	11.3943	5.5404	5.2892	3.3196	9.0884	8.0154	9.6129	7.4571	9.3053	7.2467	3.7095	8.878	7.9391	8.3971	7.8509	8.9588	10.7453	9.3594	9.9735	14.4203	6.2236	10.6796	8.4208	11.8458	12.5104	11.4737	8.9289	8.9855	4.3145	0	9.2601
TCGA-73-4676-01	281	1	10.426	10.312	0.6114	10.9959	8.4945	8.149	4.2027	1.6373	12.6062	0	9.4998	3.0683	10.2304	8.9495	8.5017	4.3217	7.4697	7.7356	8.9011	9.4818	9.6342	9.7332	5.6612	9.2127	2.7665	8.4785	8.3112	8.5411	11.0739	7.7604	5.1841	13.8185	5.8838	9.7668	7.2371	12.6321	13.1322	11.4232	8.4285	8.5267	5.0061	0.6114	4.5005
TCGA-73-4677-01	38	1	10.743	13.6283	2.9889	12.5018	11.147	7.788	3.8951	1.1087	10.0503	1.2902	8.6143	8.1834	11.0958	9.2366	5.462	5.2591	9.0295	7.4228	9.8776	9.2469	8.4271	8.4618	6.2566	8.3019	5.8056	8.6532	9.4322	8.3977	9.3114	9.5537	6.3919	15.4131	6.3618	9.9263	5.3843	12.7415	13.2755	12.1451	9.1701	8.4499	5.6811	0	7.2984
TCGA-73-7498-01	1189	0	10.388	11.7653	1.7879	11.7581	10.907	4.9659	4.9533	0.3478	8.7233	0	8.3793	6.1781	10.983	9.5119	7.0275	6.1943	9.521	7.8297	9.4711	9.7773	8.6449	7.6456	5.8297	8.0828	7.3087	9.2601	8.2457	8.6596	11.1	11.5418	7.925	14.2023	6.5411	10.0537	5.3883	11.7155	12.4471	11.7256	8.4303	8.7335	6.7196	0.3478	6.4496
TCGA-73-7499-01	1531	1	9.9644	9.7272	4.185	10.9512	14.878	2.4996	4.8899	2.5882	9.8637	4.3461	10.2266	8.5083	10.2881	8.7422	6.6206	7.1652	8.7673	7.7706	9.2634	10.524	10.1122	8.4355	5.593	8.9927	7.6316	8.5896	8.6436	8.6149	11.417	9.1265	7.147	14.2555	7.5513	9.6229	5.6143	13.1241	12.0382	12.4465	8.2747	9.7781	2.3042	0.4416	6.168
TCGA-73-A9RS-01	340	1	10.166	4.3481	2.8404	8.0016	15.297	15.7039	4.2844	1.465	8.558	1.465	9.6789	7.7117	9.0383	8.0693	6.4474	2.6512	9.1018	6.4619	10.2276	8.2345	9.4637	8.0788	5.5202	8.1512	2.6512	8.394	8.5613	8.8553	10.5868	6.5044	3.1574	15.1528	5.5477	10.5175	3.0076	12.1253	9.7906	11.9737	8.662	6.7246	2.6512	0.911	7.6749
TCGA-75-5122-01	0	0	8.0863	7.1005	5.0512	8.583	12.763	5.4366	4.4134	0	8.6665	0.8846	10.4778	4.0207	9.3964	8.6635	6.7611	6.9408	9.1323	6.859	9.1414	11.2314	9.3082	7.3379	4.8542	8.4757	2.3871	8.6269	8.1306	8.5245	9.6343	10.1135	7.3825	14.9683	7.2604	9.396	4.0207	13.5557	11.5681	12.0088	7.9249	8.1945	4.4696	0	7.574
TCGA-75-5125-01	2027	1	9.6922	7.579	2.8451	7.1832	11.038	8.5114	5.2716	0.5998	10.5282	2.8451	9.6109	3.6253	8.7289	8.1126	7.7919	5.798	9.5917	6.3921	8.9098	10.1674	9.408	9.5297	3.7956	7.6089	3.8482	8.7836	7.5945	7.6399	12.5417	8.9908	6.3472	13.6345	6.7646	9.5457	3.5637	12.0466	11.529	10.4059	6.3833	8.5496	3.7956	0.5998	6.1413
TCGA-75-5126-01	0	0	10.193	10.0057	4.8513	9.4644	14.153	8.5485	4.8906	4.4397	10.8249	0.8449	9.4854	6.3472	9.2903	7.8523	6.3752	6.3472	8.0254	7.1079	9.2809	11.1835	9.1454	8.1398	3.0296	8.3374	4.6372	8.8641	7.7386	8.6859	9.5557	8.489	4.7694	14.6922	6.5323	9.5542	7.031	13.0826	12.9981	11.5793	8.2458	8.0774	5.6769	0	5.3224
TCGA-75-5146-01	2368	0	9.9237	9.6961	3.3524	9.3541	12.946	4.6494	4.28	0	9.3681	1.6444	9.6812	7.2808	10.4456	9.8041	8.3902	5.0397	8.496	7.1573	9.1712	9.1622	9.8536	7.6551	4.5655	9.5319	8.2017	9.3257	9.4063	8.2461	10.3325	9.7108	7.3133	13.4914	5.9364	10.0952	4.4298	12.3737	12.2287	12.1861	9.089	7.234	5.0397	1.2735	5.3212
TCGA-75-5147-01	1333	0	9.5872	9.0379	4.8587	10.9608	13.59	4.6027	6.2794	0.6688	10.2476	2.5882	10.1977	6.9709	9.8887	9.2036	7.0275	7.1994	9.3762	7.2252	9.8812	9.4912	10.0333	8.7788	4.5492	9.8049	7.0405	9.4394	8.1015	9.4078	11.1248	9.0355	8.3616	14.1014	7.0405	9.9144	5.651	12.3893	12.2454	12.0472	8.7073	8.2723	4.0036	1.8695	4.9579
TCGA-75-6203-01	0	0	9.5441	12.4249	5.5143	10.9032	12.77	4.3404	4.3404	1.2482	8.3409	0	9.3077	5.8255	9.1634	6.9231	7.0483	5.9906	8.5056	7.6322	9.703	10.4218	8.3592	8.5111	5.4702	8.2678	6.3359	8.6159	8.2623	8.646	10.6495	10.2948	7.3993	14.4888	6.8215	9.7144	10.6654	12.9484	13.8315	11.8308	8.2363	9.5704	6.2732	0.7551	5.3043
TCGA-75-6205-01	0	0	9.9806	7.5173	5.8095	8.5134	9.038	5.5209	5.3812	0	11.0651	0	9.8181	2.6052	9.9919	8.2538	6.0314	7.0757	8.8702	7.1203	8.8214	11.4482	8.9905	8.7159	4.8563	8.6572	2.1342	8.51	7.5697	8.3876	10.4583	10.3742	3.8639	13.476	6.2719	9.5912	3.3677	12.9298	11.4213	12.6492	8.184	7.2465	4.5257	0.8856	6.3032
TCGA-75-6206-01	2590	0	10.314	12.3462	3.7641	12.0673	11.468	12.1025	4.5252	0	12.3566	1.5301	8.5452	2.986	10.8502	8.6929	7.3239	3.7641	8.7905	7.652	8.3578	8.4076	8.3051	8.1507	3.8919	8.2983	6.9943	8.3559	7.2242	8.2584	10.0543	9.5113	7.7051	14.1889	6.1972	9.5781	3.8294	11.7544	12.7468	11.5363	7.731	6.9362	5.7171	1.5301	6.6042
TCGA-75-6207-01	0	0	10.731	8.1094	2.4318	9.526	13.302	5.2943	4.7977	0.5257	9.0894	0	8.6741	3.9132	9.5185	8.9819	7.7068	6.332	8.6865	7.5224	9.1063	10.2824	8.3245	8.6987	4.3771	8.2757	3.1558	8.4476	8.0909	8.7349	11.0016	8.0979	8.4567	12.5783	5.4763	8.8046	5.8506	11.7569	10.1362	11.5662	7.4299	7.385	3.7342	0.91	7.8863
TCGA-75-6211-01	0	0	9.6589	15.4697	2.3059	9.5042	12.868	7.8849	9.5068	0.729	10.5665	0.729	9.4645	4.9034	7.4217	7.7981	8.3124	8.2792	8.8076	8.1549	9.034	7.8647	10.0175	7.8849	2.486	8.1382	5.137	7.8401	6.4451	7.8524	9.6577	9.8783	9.2	14.2373	6.0888	9.8813	3.754	11.58	10.4246	11.2118	8.9215	7.311	4.0686	0	6.0173
TCGA-75-6212-01	1516	1	9.984	8.2452	3.833	10.179	14.953	4.1817	3.9098	0.8314	10.7764	0	9.2241	5.008	9.7715	8.9404	6.4643	5.6692	8.6966	8.7961	10.0915	11.5404	8.5362	9.209	7.1185	7.9268	6.4124	8.9404	9.2975	8.3557	10.1741	11.9782	7.7081	14.4371	6.502	9.8503	8.6637	13.3292	14.0791	11.5666	8.521	10.2656	5.6013	0	5.5301
TCGA-75-6214-01	1115	1	10.23	6.8479	2.9973	10.5886	11.02	3.904	4.3096	5.3283	8.6989	0.6847	9.5109	8.7136	11.6152	9.6285	8.1729	4.416	9.4389	7.2645	8.5569	10.7952	10.1337	8.3791	2.5507	8.3981	4.3953	9.1742	7.7731	7.8737	11.0456	8.0108	6.2767	13.2746	6.5442	9.9673	5.5502	12.7064	11.3075	11.1504	8.0142	7.1612	2.4739	0	7.7651
TCGA-75-7025-01	3305	0	10.724	10.8872	3.4374	10.9409	14.595	4.2015	5.8513	1.094	9.9386	3.6696	8.7415	8.633	9.4015	6.7452	7.6297	6.4342	9.5714	8.0144	9.2994	10.6548	7.7075	8.7402	6.1644	8.4551	6.6934	8.648	8.4656	8.4008	9.785	11.3853	7.3905	13.7296	6.6121	10.0955	5.7836	13.0444	13.3704	11.8202	8.128	8.4248	4.2596	2.1388	6.6342
TCGA-75-7027-01	3059	0	9.6928	11.0514	3.0921	8.5948	9.7239	15.8813	5.815	0.3668	10.6682	0	9.3522	2.9907	10.2519	7.303	6.7795	7.2242	10.7132	8.1072	8.2508	8.1387	10.0991	7.744	4.7733	7.9162	7.0649	7.6843	6.7371	7.984	10.948	10.1906	5.112	13.1564	7.2158	10.4109	2.6349	11.6942	12.2354	10.9412	8.7981	7.3293	4.3707	1.7295	5.1712
TCGA-75-7030-01	0	0	10.528	10.8654	2.858	10.8734	12.593	5.7857	5.2668	1.6245	9.8254	4.143	9.1436	8.1982	9.5668	6.8786	7.4073	5.7016	9.3162	6.5818	9.0171	10.0631	7.8135	8.1085	5.2668	8.0254	7.2777	8.2923	7.268	8.2386	9.0756	10.8925	5.3053	14.0734	7.3253	9.4468	4.9149	11.9312	12.1267	11.2257	8.8136	8.2386	6.1666	3.5131	6.228
TCGA-75-7031-01	0	0	9.5537	11.8934	3.092	10.5763	14.414	8.2439	3.2634	0	8.7118	1.0533	8.501	8.0987	10.3614	8.357	7.0227	5.0556	9.076	7.4791	10.7106	9.8033	9.1073	7.7335	5.7751	7.4608	7.7408	8.4838	9.096	8.0527	10.6764	8.4487	5.8577	14.1292	7.7262	9.3066	5.2311	11.9634	11.6736	10.6592	7.9371	8.7733	3.092	1.0533	4.5595
TCGA-78-7143-01	4961	1	10.895	8.6167	4.9649	10.0551	13.339	3.1173	5.4965	4.4668	10.2581	0	10.0041	7.6891	10.4909	8.083	7.6027	6.0184	7.9743	6.9298	9.1011	9.8663	8.9252	8.9379	5.5857	8.6962	6.6277	8.5088	7.6063	8.2829	11.2113	8.6464	7.5884	14.0999	6.4649	9.1478	6.1894	12.417	10.6618	11.3672	8.3224	7.8357	3.4056	0	5.8958
TCGA-78-7145-01	826	1	9.9534	11.8972	3.5917	10.3563	14.1	8.6981	4.3775	0.6617	9.5717	4.2509	9.3414	5.9833	9.2917	7.1656	7.112	7.4512	8.873	8.0445	9.4437	11.27	9.2993	9.5969	4.6691	8.2523	6.6174	7.553	7.0436	7.9728	11.1257	8.0729	4.9115	13.855	4.2509	9.6943	7.1773	12.5928	10.7216	12.363	7.1301	8.3654	4.6691	0	4.5665
TCGA-78-7146-01	173	1	10.33	8.7986	3.3858	8.6652	9.5708	3.3858	4.3855	0.5826	9.6121	0	9.3393	4.9238	9.9596	7.4233	7.2829	7.3504	8.6723	6.7677	9.2065	10.1646	9.4011	10.0981	4.5477	8.5487	6.2597	8.6741	6.8508	7.5397	10.0522	6.6864	2.579	12.8335	4.3151	10.1375	2.4535	11.6874	12.7108	11.4497	6.7544	8.1974	2.9007	0.5826	4.3151
TCGA-78-7147-01	586	1	9.9098	10.9204	4.9046	10.1242	14.707	2.407	3.8382	1.5637	9.8879	0	9.2401	7.7324	10.9925	9.3711	5.6636	8.2802	8.2359	7.9603	9.7036	10.2894	9.7777	9.394	6.3981	9.2231	7.1322	9.0251	9.1456	8.4799	10.8481	9.7399	8.9136	14.22	5.8316	10.0007	6.868	12.396	11.9915	11.7194	8.1351	8.6665	3.2642	1.359	5.9181
TCGA-78-7148-01	626	1	10.017	12.327	3.1662	9.6915	13.636	15.5615	4.7293	0	10.1796	0	9.6172	7.0424	10.476	8.4307	6.5957	6.8228	9.4219	7.6002	9.3603	9.5296	9.4241	8.1654	4.8932	8.3837	6.7319	8.3483	7.1863	8.2023	9.9599	8.956	7.181	13.913	6.3357	10.317	2.9842	12.0593	12.1565	11.7239	8.668	8.985	5.6416	0.6152	4.6087
TCGA-78-7149-01	3940	0	9.7251	14.5032	5.1815	12.7491	11.52	5.8936	5.5644	0	9.9644	0	8.372	8.288	11.2472	8.0684	6.9855	4.4564	9.5568	7.8719	10.2425	7.8023	9.2443	8.474	6.861	8.4691	7.6951	8.2519	9.4183	8.7183	9.7521	9.4043	8.5713	14.0761	6.4341	10.534	4.0867	12.8828	12.9549	12.7712	8.6546	10.1025	4.5612	1.0723	8.7648
TCGA-78-7150-01	666	1	9.5122	13.829	5.4791	10.4703	14.175	13.1842	5.8386	0.6542	9.5623	0.6542	9.6202	8.1343	9.1156	4.7451	7.0486	4.8917	8.8562	6.8186	8.707	7.721	9.9779	10.2501	4.616	9.329	8.3727	9.2466	9.7247	7.6152	10.5292	9.6107	3.8845	13.8567	5.1698	10.8404	7.7824	11.4014	13.1604	11.3337	10.151	7.6812	3.6409	4.2315	4.9464
TCGA-78-7152-01	1215	1	10.064	7.7822	4.5413	11.9861	11.587	7.7761	4.4214	0	11.2418	0	9.1051	7.486	11.1159	6.6884	6.8597	3.0752	9.7339	6.5523	9.4182	9.8224	9.1002	8.97	5.9821	8.2478	6.2342	8.7006	7.8821	9.3402	10.5628	8.9044	7.3693	14.0677	5.51	9.794	5.5956	12.3331	10.3769	12.5784	8.8239	8.9321	3.3625	0	8.9349
TCGA-78-7153-01	3635	0	9.7728	7.7461	6.7446	11.5582	12.28	10.2891	3.1672	0	8.2112	3.065	9.0706	8.905	10.7535	8.1657	5.242	6.5877	8.5244	8.3066	9.5598	8.04	8.936	9.3197	7.283	7.9516	8.3961	9.0049	9.1616	9.4784	11.2032	8.2727	8.2698	13.6209	8.4248	9.8098	2.2279	12.16	10.635	11.6478	7.8913	8.0501	3.065	0	7.8419
TCGA-78-7154-01	593	1	9.7302	16.0665	3.6219	8.6196	13.788	3.9894	4.2372	0.6738	10.4277	2.5267	11.2142	8.5341	7.8091	9.504	8.7085	8.8115	7.7262	8.741	8.7909	8.5387	10.0597	8.1118	4.191	8.4673	7.031	7.1567	9.2214	9.0111	12.1226	7.8799	6.9005	12.9437	6.5617	9.8634	5.4352	11.2275	11.8521	11.3096	8.0516	7.916	4.9981	0.6738	4.9151
TCGA-78-7155-01	1171	1	10.076	5.4913	2.3365	11.8686	3.7243	3.2751	10.0475	1.7289	7.198	3.5131	9.8536	6.831	8.2187	9.153	8.2627	2.161	8.1467	6.7245	5.5637	6.8814	9.7537	7.5891	3.0914	5.1352	5.5459	9.2753	9.056	8.8793	10.6365	6.9773	9.3805	12.5384	5.8508	9.013	1.4521	10.6079	10.1561	11.2701	7.7529	9.6618	5.4345	2.6342	5.0369
TCGA-78-7156-01	976	1	10.457	15.0256	1.1659	12.6407	12.675	10.0567	3.8759	0	8.7693	0	8.5232	2.2422	10.3695	7.4264	7.1001	3.9933	8.557	7.575	10.1353	8.7754	8.0816	9.5016	6.5106	8.9044	6.9117	8.3416	6.9844	8.2264	9.7374	8.3164	9.785	14.6048	5.0858	9.7655	3.4529	12.0323	13.0922	11.3121	7.7162	9.3352	5.6113	2.039	7.3238
TCGA-78-7158-01	179	1	10.457	8.1768	2.7675	9.7306	7.1968	2.9334	3.0822	0	12.8518	1.1666	9.1524	7.7066	10.5003	9.0386	4.5742	6.7333	8.3629	8.1994	9.6218	9.951	8.765	8.4545	6.2887	8.5869	7.2404	8.8491	8.6279	8.9555	10.7524	7.8274	9.0192	13.5967	6.2499	9.3144	6.9618	12.1866	9.7157	11.4961	8.5244	7.6718	4.1029	0	6.7501
TCGA-78-7159-01	1974	0	9.2908	8.0252	2.8198	9.2548	14.815	12.9273	6.6543	0.6332	10.4607	4.6259	9.7674	8.0937	10.3274	7.8741	6.1152	6.2041	9.117	6.746	9.146	9.0376	9.1242	8.3923	5.4586	8.4445	4.7785	8.5533	7.0815	8.0221	9.8325	9.1113	6.7236	14.8445	5.919	10.0728	2.8198	11.4262	9.8881	11.5267	8.082	7.7541	5.5964	0.6332	4.0856
TCGA-78-7160-01	697	1	10.048	10.0278	2.2587	9.9736	12.888	16.0231	5.0244	0	9.9201	0.7057	9.7047	4.2703	9.5026	8.1023	7.2873	7.6511	9.3997	7.4261	8.7231	9.7986	8.9531	8.3287	4.7478	8.5268	6.4292	7.9815	7.153	7.5623	10.5496	9.1422	5.5563	14.6185	6.2732	9.5137	5.9441	12.2593	13.1742	10.9993	8.5119	8.714	5.3033	0.7057	5.9878
TCGA-78-7161-01	291	1	10.41	8.5338	1.6801	8.1211	14.816	14.4565	5.6716	0	8.3299	1.5044	8.6689	7.8495	10.3237	9.9717	7.838	5.7126	9.2018	8.6767	9.2104	7.362	7.9103	7.7172	5.772	8.7139	6.7587	8.7876	7.378	8.4224	10.4847	8.5664	5.9795	13.2192	6.8672	9.747	1.978	11.0753	10.4419	11.6108	7.422	8.3785	5.447	0.7949	4.4314
TCGA-78-7162-01	3169	1	10.058	13.9788	4.4975	11.5598	9.9593	12.2638	4.8484	1.8324	11.3052	0.4499	8.7768	6.0068	10.3491	7.9898	7.2281	6.7281	8.5463	7.5794	9.1705	9.7685	8.8473	8.4071	5.5689	8.0001	7.1335	8.2564	8.1656	8.374	9.7435	9.1201	8.499	13.8991	6.4366	10.0537	4.2701	12.0873	13.9354	11.4028	8.3222	8.845	6.7722	0	3.3942
TCGA-78-7163-01	7248	0	10.655	9.8955	1.6108	6.8754	16.089	2.1453	2.1453	0.4248	6.0153	0.7526	8.652	5.8017	10.02	6.5618	6.6965	7.7866	9.4725	8.6322	9.207	8.2673	8.2217	7.4619	6.1041	7.6138	2.8405	9.1908	8.3604	9.4208	10.5065	9.2983	7.4647	14.5891	6.4756	10.9447	4.0952	12.516	14.1645	12.0126	7.1642	9.487	4.0058	0	4.6419
TCGA-78-7166-01	258	1	9.6493	9.0321	4.0142	9.7795	14.382	12.462	5.5658	0.5319	8.8983	0	8.7463	7.1395	11.7679	7.5149	6.7003	5.826	9.681	7.9671	9.8918	7.9013	10.1812	6.2712	6.3123	8.6556	8.2123	9.1432	9.2994	7.7924	10.5386	9.484	4.2006	13.9569	6.9496	11.2045	3.0238	13.3245	10.5407	12.5732	9.1702	9.7714	3.2435	2.5617	7.2193
TCGA-78-7167-01	2681	1	10.204	14.3467	3.5844	11.835	11.92	13.1446	4.0915	0	8.9614	0.3754	8.1792	6.9089	10.6636	8.2157	6.2057	6.3546	9.795	7.4597	9.5433	8.4135	8.9861	8.3779	5.2535	8.9195	6.8982	8.3254	8.191	8.6989	9.8166	9.6547	8.0622	14.4112	5.0614	9.8862	3.3097	11.5485	11.5149	12.2836	7.8555	7.8313	4.6134	0.673	6.2848
TCGA-78-7220-01	807	1	9.6012	13.4083	3.2072	10.7968	13.644	12.919	5.17	0	9.5347	2.9433	9.8998	5.9481	8.4386	7.6637	7.8231	8.0834	8.9887	7.3591	7.895	7.6637	10.1935	9.5829	2.4938	8.7402	6.8097	8.0724	7.7899	7.6747	9.8484	7.9335	5.4672	13.9872	7.6747	10.4098	7.4637	11.7255	14.607	11.9514	9.8676	7.6855	4.9231	3.1245	4.5637
TCGA-78-7535-01	949	1	9.38	7.5891	3.9933	12.3297	14.77	3.5437	4.31	0	8.4945	1.9423	7.9015	7.1828	11.3266	10.5839	6.716	2.7412	9.2327	8.2637	9.5042	11.0278	10.1789	7.5918	5.3878	9.0416	3.587	8.7973	8.6896	8.211	11.437	10.231	7.3524	14.7122	7.3174	10.9532	3.5437	13.5179	10.9194	11.8136	8.6896	8.5198	3.4054	0.7747	8.5337
TCGA-78-7536-01	244	1	9.5538	5.8012	0	6.3325	15.123	10.7925	5.934	0.3287	8.7148	2.0253	9.2308	9.1349	9.9661	8.7096	7.4991	3.9622	9.095	7.2979	9.2413	10.3136	9.682	8.2957	5.0988	8.8165	1.9316	7.9479	6.9038	8.5414	10.5557	7.4555	7.1883	14.6622	5.1723	9.2648	2.8363	11.6372	9.2672	10.593	8.3499	7.59	3.8126	0.8219	4.0088
TCGA-78-7537-01	1622	1	10.229	7.0195	2.5833	11.9112	12.882	4.4386	7.1664	1.2789	9.5145	2.0737	8.1232	5.4875	11.1446	6.9463	5.7004	3.5037	8.581	7.8452	9.5835	8.581	8.5249	7.9736	5.1822	8.0188	7.2899	8.5675	9.149	9.9907	9.7115	9.0408	6.2046	14.1112	6.6304	10.3615	4.2622	12.3603	12.9064	11.9288	9.2454	8.0416	4.6785	0	7.0509
TCGA-78-7539-01	791	0	9.3023	9.8693	4.6527	9.9643	13.299	2.9031	4.0541	1.3061	9.0499	0.9136	10.1675	6.6687	10.3171	4.1289	5.4499	4.8124	9.9962	7.3823	9.0194	9.5855	8.5713	7.6948	6.928	8.7155	6.9139	9.1256	8.0952	8.4257	11.2573	9.0264	7.5884	13.6283	7.2628	9.6921	8.5735	12.3569	11.4003	12.4994	8.3338	8.8645	3.7725	0.3725	5.8394
TCGA-78-7540-01	1197	1	11.262	9.072	2.8672	10.3237	10.238	3.5366	4.9305	0.4129	7.2428	0.7336	9.0269	7.6522	10.924	7.2954	6.9766	6.9728	9.7855	7.1278	9.2362	9.4346	8.7774	9.7297	5.1211	8.5331	8.7914	7.9224	7.2301	8.3075	9.602	9.5093	4.9919	14.3528	9.2954	10.055	7.5539	12.8838	11.1252	11.9435	7.6546	8.3797	3.8335	1.5794	5.3137
TCGA-78-7542-01	321	1	11.099	6.6875	1.081	11.2946	11.688	5.5636	6.2108	0.3549	7.1537	0.3549	9.7335	3.2698	9.204	7.3266	8.0952	3.6362	9.7873	8.073	7.8547	10.4815	9.858	7.1452	2.835	7.7478	2.1199	8.3259	6.7486	7.9947	11.133	8.6975	3.8743	11.8583	6.1211	10.4588	2.294	12.455	10.9258	10.9437	6.3003	8.5772	2.5213	0	7.3039
TCGA-78-7633-01	1528	1	10.036	16.0593	6.1074	11.8787	9.1639	11.9902	5.1864	0	9.3777	0	8.707	4.0232	10.3464	8.8641	7.4925	5.8071	9.1199	7.6985	9.2033	8.7787	9.0074	8.8326	6.5005	8.5433	8.2043	8.0732	8.6008	8.2569	10.4892	7.7496	8.8783	14.7721	5.9212	9.7077	3.2249	11.4489	12.798	11.5761	8.6294	8.7654	6.3199	0.6561	3.5778
TCGA-78-8640-01	7062	0	9.7829	10.4217	4.4599	8.3306	14.299	3.1019	6.8574	1.1157	8.9176	6.9143	8.9366	8.0861	9.4872	8.6527	7.5525	7.6893	8.8035	9.0569	10.1101	8.4194	9.8968	8.5328	5.3254	9.8278	3.8501	7.9327	8.6485	9.78	10.9136	9.0004	5.0985	13.3476	5.1228	10.4026	7.3455	13.33	10.601	13.0199	8.1138	7.7414	3.2858	1.1157	4.8584

TCGA-78-8648-01	1209	1	8.9829	11.4874	5.5959	9.8892	10.513	6.3163	6.7542	3.3459	12.1134	0.8188	9.2342	5.7675	9.5838	6.498	7.4899	6.043	8.9251	7.015	9.1741	10.5314	8.7671	8.384	4.4347	7.9714	5.1133	8.2956	7.5203	8.9894	9.421	11.0727	5.5258	14.1266	7.1453	10.1836	4.7121	13.2173	12.3884	11.7438	9.1952	7.8937	5.9026	1.7189	7.3279
TCGA-78-8655-01	2360	0	10.147	9.7489	0.7078	10.4162	14.026	7.5138	5.1375	0	10.1187	1.821	8.9252	3.7041	9.8418	6.0768	6.0494	4.9726	8.6998	7.6565	9.3749	11.4137	9.3047	8.2039	4.2275	8.7666	3.2068	8.6799	7.4156	8.834	11.2042	11.2466	6.4762	14.939	6.3249	9.7969	7.0997	12.9073	10.9477	12.5631	9.0699	9.0056	4.3669	1.536	5.6553
TCGA-78-8660-01	321	1	10.065	9.2786	5.1699	9.3253	12.431	4.0149	4.4971	0	9.7995	0	9.2813	4.2542	9.9202	5.2605	7.6005	5.8328	9.1228	8.5048	8.5385	9.3641	10.3405	8.0949	5.8765	7.9692	4.8579	8.4529	7.4784	9.2595	9.9432	9.3461	6.8857	13.4771	7.2037	10.9742	3.2854	13.0378	12.4371	12.8345	7.9696	8.4696	3.3692	1.737	8.0638
TCGA-78-8662-01	3361	1	9.8134	9.0884	3.8315	13.4358	10.591	3.5347	7.2216	7.0946	12.0214	1.5779	8.3889	8.7231	12.1269	7.9495	4.8806	1.2164	7.5261	7.7904	9.2794	6.1528	9.7516	5.8885	5.225	7.4295	7.6164	8.6795	10.35	9.3609	11.1449	7.9261	8.3775	10.9036	6.112	10.2018	6.1925	12.6134	12.2842	12.8212	8.453	9.5838	3.6148	2.6541	7.2406
TCGA-80-5607-01	0	0	10.856	8.7311	4.2404	11.2198	12.812	4.5216	3.6313	1.3936	9.8627	0	9.1542	8.438	10.5054	6.3922	5.7735	4.8412	8.6428	7.348	9.0096	11.7218	8.6574	9.2633	6.6477	8.0988	6.7818	8.6486	7.8432	8.2503	9.8334	9.0562	6.2909	13.5644	6.9992	9.4989	5.4635	12.4442	11.2325	11.7663	8.4346	8.5828	4.7997	0	7.348
TCGA-80-5608-01	2832	0	10.362	8.49	2.8133	11.3208	14.147	11.7611	5.2024	5.4565	8.402	0.4379	9.1345	10.0851	11.0628	8.4171	5.3602	6.839	9.8859	7.4464	9.3079	9.0121	9.6755	7.5207	4.5666	7.3358	6.6785	9.4737	7.1727	8.8171	10.5573	8.8441	6.8524	14.8395	7.3742	10.6369	3.9905	11.9975	11.2402	10.9682	8.4857	8.1818	4.2545	0.4379	4.5228
TCGA-80-5611-01	2595	0	10.261	12.2761	4.7947	8.8655	15.25	2.9428	3.0438	2.1192	8.9574	1.0805	10.5592	9.1622	9.7528	7.8669	7.1725	5.3426	8.6422	8.6642	9.4729	10.5176	9.7603	7.6236	6.8969	9.0908	0.6391	8.5029	6.8278	8.409	10.8493	9.6997	6.8419	14.3333	6.3247	10.4571	1.0805	12.1687	10.3867	12.4291	9.7924	9.4909	1.0805	0	6.1086
TCGA-83-5908-01	824	0	10.017	8.3841	2.8091	11.849	11.737	8.9864	4.9028	0	9.7346	0.8082	9.7986	3.4281	9.2911	8.8545	6.3792	7.6998	8.5637	7.6946	8.6205	10.6506	9.7642	8.304	4.3328	8.3136	0.46	8.5023	7.3835	8.4491	9.9019	9.593	5.6494	14.1529	6.6458	9.226	3.2112	12.2687	10.9256	11.1671	8.06	7.7205	4.8846	1.8594	5.0894
TCGA-86-6562-01	376	1	10.129	9.8921	3.1763	10.9744	14.304	5.0278	5.4729	0	11.1395	2.4027	9.219	9.0174	9.1955	8.9042	7.3523	4.8515	9.4881	7.071	8.9383	10.3102	8.8056	7.7828	4.7689	8.0094	7.071	8.2318	8.5838	8.3407	10.7061	9.3563	6.5	15.5428	5.7988	9.3456	5.9812	12.225	11.0176	11.5424	7.964	8.4987	4.5553	1.3829	4.6812
TCGA-86-6851-01	179	0	9.5957	7.9015	4.6559	8.5061	14.189	4.6395	5.2272	0	10.7094	1.0971	9.1499	6.1966	9.0396	8.9148	8.0422	6.4098	9.4853	6.4897	8.5228	10.5741	8.8279	9.0954	5.0648	8.2815	6.2022	9.3893	6.6979	8.5605	9.2025	9.4876	6.1453	14.1466	6.174	9.7357	4.8103	12.113	11.0699	11.1061	7.9135	7.588	3.8749	5.3531	6.2188
TCGA-86-7701-01	947	0	9.6068	8.1467	1.6358	10.0527	11.855	12.2712	6.0744	0	11.6113	1.6358	10.4275	8.3279	8.9082	9.2047	8.1031	5.1177	8.5791	7.3324	8.7834	8.9702	9.9365	7.6751	4.7731	8.312	7.0522	8.1251	5.1177	7.6374	9.7567	8.9671	5.0273	13.5967	6.733	9.7011	2.3827	11.2322	10.1429	10.3387	8.2148	8.1087	5.7745	0	3.7703
TCGA-86-7711-01	1046	1	10.65	8.6525	0.6678	5.7143	14.473	5.6225	7.1731	0	15.8367	1.4676	9.4173	1.8673	9.4645	9.2143	9.0063	3.5354	9.8493	6.8218	9.5549	10.0997	9.6722	8.2471	3.7399	8.9138	0.913	7.2166	8.5107	8.1109	9.8087	9.331	2.18	15.4099	4.7806	9.9385	2.5857	12.6348	11.2788	11.8866	9.1774	7.1192	5.2731	1.9793	5.7776
TCGA-86-7713-01	1157	0	8.8808	6.2302	1.5479	12.7649	12.848	14.3645	3.9249	1.2886	8.5799	1.6619	8.438	6.9834	10.5682	5.9449	7.2686	6.7094	8.7451	8.1945	7.7118	9.7726	9.3164	7.75	4.8408	7.6351	5.6456	8.037	8.2227	8.3333	10.795	8.1154	7.5516	13.9127	6.1396	9.9926	4.0766	11.7155	9.5352	10.9075	8.4853	6.8149	4.9001	1.7676	7.6326
TCGA-86-7714-01	625	1	11.022	10.4084	2.922	8.1006	14.442	4.1633	4.4993	6.7442	11.9539	0.9559	9.469	7.0507	9.2946	7.562	5.6935	4.9577	8.1682	7.7813	8.7435	11.7985	7.7874	9.2572	5.2347	9.2648	6.3039	8.6756	8.2506	8.5832	10.267	11.8257	7.1495	15.0878	6.1971	8.8176	3.2416	12.4489	10.7576	11.59	7.5183	8.2551	3.2416	0	3.9163
TCGA-86-7953-01	997	0	10.743	6.706	2.7885	9.3333	13.557	4.133	6.0572	0.3734	10.0581	3.7765	9.8331	4.0061	8.995	8.6315	7.4342	8.9794	9.0365	6.7343	8.5399	10.5131	10.7109	8.5599	5.2453	8.5699	1.4713	8.5497	6.2032	8.1796	10.6008	8.5868	4.3781	15.1703	6.1137	9.6929	4.0324	11.9232	9.2468	10.3972	8.0633	7.566	4.9876	0.6698	7.0268
TCGA-86-7954-01	605	0	9.6304	9.4227	4.0462	7.7142	14.593	3.2165	5.4086	0	10.4404	2.7267	9.3506	5.0493	9.9158	8.8139	6.5969	7.7912	9.4725	7.2774	8.5855	9.9305	8.2539	7.9788	4.2425	8.7151	7.7487	8.7052	7.3502	8.5907	9.9094	9.8977	5.8973	15.092	6.8115	9.9711	6.1682	12.0246	12.2656	11.9097	7.2498	8.0978	4.7668	1.8781	4.5367
TCGA-86-7955-01	1072	0	11.116	8.8277	1.6363	9.9717	16.61	15.8393	4.1835	1.3254	11.6837	0.3798	10.1786	8.6312	10.0859	8.1793	7.9348	5.8638	10.2531	8.2016	9.5337	6.9354	9.4657	8.781	6.7007	8.6894	4.0586	8.2178	8.5267	8.7701	10.1039	6.9635	6.758	12.4129	5.6554	10.6331	2.5409	11.538	8.0928	12.2157	8.857	7.4961	3.2379	0	4.5602
TCGA-86-8054-01	1148	0	9.4102	8.1228	0.7365	11.1455	10.401	15.574	8.0861	1.8735	9.9245	2.414	9.6455	4.6617	10.0525	6.2403	6.8442	4.2467	8.3504	6.949	8.2139	8.6569	9.3199	7.5144	5.1417	7.5698	2.9375	8.9274	7.2623	8.4471	11.1092	8.9343	7.78	13.3514	6.1211	9.1794	3.1688	10.948	10.5517	10.618	8.6232	6.1619	4.7888	1.2216	4.4361
TCGA-86-8055-01	124	1	9.4532	9.7646	3.0508	10.0943	13.527	5.7359	6.4241	1.1928	11.271	6.8776	9.082	5.3827	8.998	7.5733	6.4168	7.9233	9.3056	6.757	9.0934	10.5156	9.0532	8.747	4.4875	8.7806	6.0755	7.9335	7.6554	8.1095	11.365	10.2887	7.0508	13.9017	5.5371	9.5991	5.7242	12.2375	10.8864	10.8291	7.8039	7.7955	5.3066	1.1928	5.2097
TCGA-86-8056-01	139	0	11.346	9.9932	3.323	11.5824	9.8175	6.6097	3.7885	0	11.9387	0.7595	9.2828	7.2484	10.2527	8.3983	7.2777	6.1065	9.1099	6.6045	7.7004	11.391	7.9979	8.6024	4.3987	8.6868	6.6649	8.7089	7.5997	8.1143	9.6843	8.0038	8.1847	12.899	6.5734	9.5177	7.4158	11.6845	10.8646	11.6175	8.0554	6.5681	5.9855	0.4291	6.47
TCGA-86-8073-01	740	0	10.89	13.1878	3.7037	11.2473	12.467	4.1005	6.8976	0	10.1174	2.539	9.7041	5.5135	10.4208	7.5675	8.5212	6.6437	8.7059	6.2852	8.9835	9.7213	8.5399	9.5826	4.7243	8.5946	6.4129	7.6733	9.2065	7.5438	10.2625	8.4563	8.9389	12.9434	5.6081	9.0318	5.3161	11.543	12.1229	11.5437	8.5558	7.8727	3.4997	0.4262	5.8248
TCGA-86-8074-01	24	0	9.2695	7.7439	3.3635	10.8715	14.196	3.9684	6.0839	1.0512	10.8587	7.2119	9.2382	3.6365	9.5347	10.0887	6.3723	7.0186	8.4869	6.9241	8.2258	11.3967	8.9354	8.5693	5.1565	9.1574	7.2119	7.6699	7.9414	8.2432	10.8868	8.8795	4.9385	13.8767	5.6115	9.6305	3.6774	11.7382	10.1862	11.563	7.9498	8.5097	4.3933	0.4408	5.1565
TCGA-86-8075-01	694	1	9.5909	10.1029	4.0873	10.7341	13.191	4.8859	5.8754	2.6843	10.6832	5.5367	9.0831	5.2314	9.1607	7.2464	7.5577	7.3736	8.8662	6.2504	8.3491	10.6647	9.1027	8.6276	4.6437	8.8796	5.5805	7.9007	7.7556	7.7499	10.9468	10.3164	7.2918	13.6118	6.3191	9.4454	3.0751	12.102	11.0756	10.612	7.9581	8.4991	5.3166	0	5.7277
TCGA-86-8076-01	993	0	10.186	10.24	2.7754	11.9119	14.221	10.8963	5.7962	10.072	11.2254	0.8316	9.0624	7.3774	10.078	7.9228	6.8961	5.2783	8.9012	7.7945	9.019	10.2889	8.2174	8.3628	6.2225	8.5049	5.846	8.6302	8.0018	8.8775	10.2437	9.6355	5.6131	14.6449	5.7759	9.8687	6.0465	11.945	11.1021	12.0315	8.7708	8.4148	4.8612	1.3557	7.6647
TCGA-86-8278-01	944	0	9.9214	10.9966	1.3537	8.6981	15.194	3.9561	5.5026	0	9.5687	7.5015	8.5613	4.8321	9.3428	7.707	7.4317	5.0845	9.122	8.6157	8.822	11.5789	8.3845	8.4275	5.5907	8.2765	2.7451	8.8886	9.2061	9.0818	9.581	11.5996	8.0886	14.4858	5.9405	10.2608	6.9133	11.9601	13.0914	11.0622	8.0482	8.0283	3.44	0.3326	4.9698
TCGA-86-8279-01	949	0	9.1553	9.012	2.7289	9.8185	15.001	4.1847	7.2091	9.8461	10.4779	6.9011	8.0723	6.0992	9.9546	8.5309	7.8345	4.8506	10.1734	8.582	8.9546	10.0307	8.6718	7.6188	5.3365	8.6604	5.6912	8.5076	9.2705	8.6915	10.9515	9.9672	6.8126	14.0816	5.8772	9.9787	4.0363	12.2364	9.6306	12.5842	8.9911	7.5634	2.5938	0	7.3309
TCGA-86-8280-01	701	0	9.7633	10.6294	2.5174	10.5311	12.738	4.9107	5.3049	0	10.2789	4.3666	9.0799	7.2908	9.5721	7.8455	7.2431	7.0821	9.2947	7.5166	9.6868	10.8181	8.81	7.6993	5.9974	8.7869	7.208	9.1191	9.3775	8.5841	10.6689	10.7915	8.6971	14.0327	6.7483	9.7777	6.1603	12.4363	12.7034	11.5456	9.1471	8.7889	4.8932	0.4473	6.1455
TCGA-86-8281-01	0	0	10.331	11.7694	3.226	12.0696	9.3798	6.9971	5.2836	0	9.7953	4.2294	8.7481	5.4306	10.5371	7.2589	6.9572	5.8862	9.4027	7.8018	8.4779	7.665	8.2738	8.3891	4.3076	8.9477	7.1464	8.8744	8.5071	8.4144	10.5687	8.7907	7.7208	14.8156	6.3901	9.8804	4.5195	12.1572	11.9193	11.9051	8.0227	8.0532	5.2707	0	6.0769
TCGA-86-8358-01	653	0	8.4531	12.3817	4.8505	11.5006	11.204	3.8997	9.971	5.6072	9.8539	3.0521	10.2819	5.7562	9.2923	8.2915	9.0965	7.9446	8.9724	7.8565	9.349	8.0956	10.0262	8.1267	5.6649	8.1958	6.5823	7.6997	7.8851	9.209	10.9287	8.9221	9.1961	12.7206	5.9849	8.766	4.0802	10.614	10.9099	10.028	7.8647	8.1233	6.5315	0.7339	4.7475
TCGA-86-8359-01	444	1	9.6524	7.8227	1.497	9.5544	14.723	13.7269	4.8553	1.1474	8.8388	1.497	8.4872	4.5901	11.6779	9.8082	7.3235	6.8252	9.1723	7.0535	8.5241	9.4692	8.8766	7.9628	2.0135	8.3619	7.8381	8.386	8.3217	8.7722	10.5581	9.1416	3.1535	15.3597	6.4574	10.8521	3.4223	12.6321	10.4069	12.58	6.9295	7.0535	5.7013	0	7.3877
TCGA-86-8585-01	353	0	8.5713	9.874	3.8312	13.1281	12.926	14.3773	4.5271	5.7233	10.1551	1.0723	8.8924	11.0873	11.4453	8.5115	6.6789	4.1328	8.4537	7.8955	9.8413	8.4445	9.1853	7.608	4.8911	9.1462	3.1243	8.5879	6.9344	8.5288	10.6617	9.6528	4.3036	14.4941	6.2267	9.8328	4.9945	13.4849	11.0902	12.8351	9.4402	7.5583	2.7036	0	7.3882
TCGA-86-8668-01	423	0	11.107	9.4226	1.9914	10.8746	14.298	8.3916	6.0385	8.3161	10.791	2.45	8.9836	8.2992	10.2131	9.1306	7.6143	4.8344	9.1152	6.7568	9.0568	10.2655	8.3527	8.9449	5.1386	8.6334	6.1333	8.1484	7.7401	8.7113	9.4733	11.3537	9.3505	13.7111	6.3061	9.5852	5.9887	12.4956	11.7259	11.7141	7.9148	7.7699	5.3375	1.315	7.9673
TCGA-86-8669-01	938	0	9.1127	12.6188	1.8158	8.5615	14.535	3.296	3.8936	0	9.0912	1.1765	8.9714	6.3177	10.6856	9.2019	7.8674	7.879	9.246	6.4587	9.3874	9.1691	8.4875	7.3867	4.9656	8.161	6.7138	9.0728	6.6153	9.1817	10.7723	8.71	8.2393	13.4771	6.0964	10.2448	3.8936	11.4134	12.6103	11.6706	8.4747	8.5299	4.315	2.4358	4.487
TCGA-86-8671-01	839	0	8.8965	10.5685	4.4555	9.4832	12.077	4.5262	6.5137	0	11.2614	2.1064	9.4619	7.5231	9.6813	8.1481	7.934	7.052	9.4998	7.2582	9.5752	10.2968	8.9278	7.7395	5.2449	8.4341	7.04	9.0332	7.6941	8.9553	10.1288	11.1149	6.8324	13.8758	7.4613	9.9247	7.3825	12.8582	12.3895	12.1805	8.6477	8.2483	3.989	0.6332	7.339
TCGA-86-8672-01	19	1	9.5735	11.9088	4.4456	12.9519	11.514	9.4067	5.7645	0	10.1916	1.6225	8.3209	4.1957	10.5776	6.4533	5.5465	6.8236	10.8397	6.6048	8.4732	9.4185	10.1517	8.7504	5.3884	8.1072	6.2317	7.9198	8.5962	8.0349	10.1958	10.4013	3.6759	12.9662	6.9502	10.857	3.1082	13.4443	12.4373	12.4091	8.9189	8.3731	1.9153	1.6225	8.3021
TCGA-86-8673-01	862	0	9.0893	8.2095	4.2169	11.4396	14.678	4.7126	5.7492	2.3521	8.8861	1.7424	8.1952	5.2007	10.513	8.1362	7.2785	4.2617	9.6361	7.9366	9.3181	8.6496	10.0252	9.5394	5.7333	7.8534	6.1693	8.824	8.5288	9.0737	11.227	9.4587	5.1775	14.383	5.3534	11.0658	4.1706	12.3908	13.1809	12.248	8.508	8.4583	3.4556	0	7.9296
TCGA-86-8674-01	806	0	9.3436	14.1043	3.912	11.4371	11.993	3.1489	4.9179	0	8.9184	5.3142	8.3705	8.6523	11.2694	5.8523	4.8632	4.6211	8.9351	8.6197	9.4727	7.3822	9.3018	7.6559	5.6082	8.5273	8.4068	9.0634	9.3927	8.8725	10.3703	9.0589	6.1888	15.0597	7.6234	11.2952	3.546	11.9722	11.0992	12.4941	9.777	9.0374	3.1489	0	6.3762
TCGA-86-A456-01	896	0	10.434	10.9164	5.5405	11.4532	11.309	5.0926	4.5531	0.6704	9.7538	0.6704	9.1352	3.6132	10.8653	8.3811	8.5926	7.1231	9.8769	7.5629	9.1543	10.6366	8.3049	8.3527	5.3444	8.4393	7.8596	8.8097	8.0325	8.6511	10.8856	9.9683	7.2959	13.6098	6.1089	10.4467	7.0217	12.9015	11.7349	12.0553	8.8154	8.0615	4.8772	1.7509	7.9658
TCGA-86-A4D0-01	116	1	9.7707	13.8663	0.9491	8.1327	12.433	14.3455	5.805	0	7.8334	2.2396	9.7714	4.4246	10.4411	8.5423	7.2099	8.4064	9.1934	8.3124	9.87	7.2189	10.2025	6.5627	6.0476	7.187	5.7056	8.4627	7.1402	8.8273	11.0292	7.9108	7.4047	13.6329	8.2027	10.1132	3.2988	11.2149	11.2906	11.0914	8.5743	8.0988	3.6592	1.5167	4.1116
TCGA-86-A4JF-01	737	1	9.8456	8.2686	3.965	11.5089	12.006	5.8504	5.0003	2.318	9.2338	0	9.019	5.9674	9.7465	7.8812	7.0212	4.0828	8.5156	6.8764	9.1508	10.0505	9.831	8.6474	5.6987	8.5736	5.8281	8.5704	8.0713	8.6394	11.4242	8.6975	5.6739	14.2591	6.1584	10.2893	4.8532	11.9978	10.6197	12.4677	8.6537	9.9649	4.5601	0.9153	7.2692
TCGA-86-A4P7-01	415	0	9.4121	11.968	3.3238	11.5315	11.277	4.3201	5.8323	1.2769	10.0294	2.1113	8.9355	7.3675	10.5989	7.2865	7.9959	6.1919	9.405	7.5355	9.0954	9.5636	8.6073	7.6456	4.482	8.3237	7.1341	8.6351	8.4282	8.7885	9.7332	9.2314	7.054	14.2397	7.0996	9.8579	7.2146	12.1697	12.8501	12.1625	8.3102	8.1652	6.1346	1.9435	7.028
TCGA-86-A4P8-01	805	0	9.2525	13.1837	5.977	11.2022	7.333	6.0778	6.1162	4.0834	9.7439	0.9348	9.1647	6.1444	9.9547	6.5117	8.9287	6.9119	9.5531	7.1982	9.6511	10.4067	8.0217	8.3201	5.7144	8.5122	6.1901	9.2009	6.6293	8.5392	8.7631	8.8863	6.311	14.1934	7.4965	9.1098	9.0516	12.5113	13.9069	11.8828	9.024	7.6999	6.4071	1.4973	7.0671
TCGA-91-6828-01	323	0	9.7588	8.421	2.5971	7.9521	14.219	4.5164	3.7683	0	10.4223	0	9.3902	7.5792	9.9067	8.5465	6.7078	5.5114	8.6438	6.4545	8.9429	10.2733	8.2797	8.3106	4.2868	7.7461	7.9223	8.48	6.5221	8.507	9.2031	8.5036	7.4252	14.3841	7.8078	9.3311	5.6629	11.748	10.8764	11.2293	7.6291	8.4351	5.9839	0	4.5685
TCGA-91-6829-01	1258	1	9.1494	10.6622	4.1729	10.0112	12.878	5.4881	7.6934	11.721	12.2024	4.0878	8.7448	5.0448	9.0321	9.1054	7.2158	6.653	8.3429	7.1439	8.0751	10.413	8.5399	9.258	4.659	8.7031	6.2054	8.3521	7.8009	8.1148	10.4698	10.5819	7.1751	13.5532	5.5844	9.1054	2.7396	11.5569	11.0744	10.5712	8.4014	6.885	4.659	3.211	4.131
TCGA-91-6830-01	60	0	10.227	13.2629	4.9842	9.9382	12.85	6.3615	4.7577	2.4848	11.2734	0.8207	8.8951	6.8762	9.7132	9.3647	8.1563	7.9792	7.7824	7.0942	8.6679	9.556	7.2402	9.4173	3.6432	8.4219	7.0448	8.1983	6.348	7.6944	10.1612	9.0068	5.9789	13.3788	5.6002	9.173	8.0435	11.6791	13.9148	10.8759	8.2609	8.4211	5.7099	0	5.3521
TCGA-91-6831-01	310	0	9.1985	8.1193	3.1239	7.8371	13.086	15.8057	6.7274	0	10.3068	3.5613	9.0531	6.7168	9.3551	9.7533	8.3137	5.5402	8.9645	8.3277	9.5903	9.8139	9.4357	8.253	4.1049	8.649	2.2806	8.9734	7.0962	8.2639	11.1732	9.8922	6.2886	14.1111	5.7199	9.2778	2.2806	11.56	9.7286	10.9054	9.544	6.7378	4.2288	0.8252	5.1275
TCGA-91-6835-01	79	0	9.6169	9.6586	5.7741	10.507	11.897	4.8798	5.348	0.9282	11.5501	0.9282	9.7777	6.7847	9.4669	9.2834	8.4806	6.6995	9.0254	6.4542	8.774	11.0239	8.5656	7.9354	5.3156	8.6428	6.6933	8.339	7.4937	8.341	9.3988	9.4868	7.1846	13.6699	6.2808	9.2506	5.1047	11.8898	11.7982	11.3122	8.3043	8.1863	5.0856	0.9282	5.1785
TCGA-91-6836-01	417	0	9.3922	6.3835	2.0313	6.8431	9.9437	4.4992	7.4532	0	7.7829	1.6806	9.6228	2.8431	8.9863	8.5322	8.4939	6.8319	8.5944	7.621	8.6584	9.0143	9.7044	8.1752	4.2544	8.3812	5.5909	8.135	7.6904	8.246	10.0726	7.4349	3.7858	12.4779	5.9095	9.6452	1.8666	11.8012	11.7738	11.7351	7.4164	7.1954	3.9595	2.7516	4.1507
TCGA-91-6840-01	372	0	9.7783	11.4487	7.9078	15.0557	6.6199	4.5803	6.6812	2.4814	8.0011	2.9776	8.9634	8.1208	9.9527	10.2036	7.382	4.6696	8.0601	7.3074	9.0908	7.9396	8.8886	8.8048	2.4814	8.7744	8.0684	9.311	8.1129	8.371	8.9276	8.3974	6.6153	13.4323	6.486	9.8167	4.1546	11.1033	11.4039	10.1248	8.1405	9.681	7.1299	1.3381	4.3831
TCGA-91-6847-01	842	0	9.8697	4.1251	0	10.9553	9.8859	4.6823	6.4935	0	7.4632	0.4251	9.2735	5.0537	9.7377	7.5128	7.0694	4.6629	8.7182	7.095	9.4718	9.2775	9.4207	8.1326	5.9542	8.3859	3.2055	7.466	8.9185	8.7712	11.563	9.2256	6.2358	12.1585	6.8122	9.1042	3.2055	11.1772	10.5096	11.0623	8.6348	8.1396	2.8417	0.7531	2.8417
TCGA-91-6848-01	224	0	5.2422	7.7508	2.121	8.861	10.253	5.3046	6.6533	9.1624	11.1892	2.121	10.768	2.375	8.7896	8.1754	7.8005	8.8374	8.4896	7.8692	8.1454	9.1603	9.9168	8.2487	5.5042	8.538	0.8777	7.3529	7.6095	8.4556	10.0053	10.0017	5.1431	13.5747	7.022	9.1666	2.7787	11.6379	10.0549	10.0617	7.4734	6.8857	6.4193	0	5.1085
TCGA-91-6849-01	35	0	10.954	14.0788	3.4609	9.2801	13.167	5.0556	3.5997	0	9.5306	0	8.7382	6.8104	10.6723	5.2368	6.3215	5.7355	8.8292	7.8533	9.5431	9.2113	8.3577	7.9011	6.2175	9.1275	6.6277	7.9211	8.8606	8.6044	9.8205	10.9059	8.338	15.1469	7.0831	10.1717	5.4353	12.6698	13.1091	11.4009	8.7935	9.5797	4.7916	0	6.3215
TCGA-91-7771-01	492	0	10.096	10.3211	6.6985	8.8575	12.466	10.0926	5.187	2.478	11.6579	1.0996	9.7585	7.5269	11.8097	8.2254	7.3613	5.0192	8.2281	6.65	9.1422	9.661	7.7953	9.0825	5.8473	8.6561	6.65	8.762	7.3713	8.2688	9.808	9.7022	7.3155	13.6996	9.1739	9.3164	4.6439	11.8183	12.1303	11.5023	8.3544	7.2192	6.256	1.0996	4.9674
TCGA-91-8496-01	505	0	10.188	11.7101	3.6572	11.1151	12.655	3.1283	3.2507	2.0348	8.931	0	9.7388	9.2091	10.333	9.167	7.2332	5.7035	8.1401	7.8852	9.7035	10.4917	9.2016	7.3474	3.1283	9.4945	7.2258	8.9447	8.3803	8.6626	9.8263	9.5988	8.8892	14.4276	6.7737	9.447	10.5928	13.8119	12.4619	12.8326	8.1519	9.0475	4.5989	0	6.9557
TCGA-91-8497-01	434	1	9.8951	11.4053	2.494	11.1502	11.508	6.6856	6.0269	0	9.9111	2.1076	9.0639	7.5822	10.0334	7.7949	7.402	6.4547	9.7125	7.3385	9.3118	9.5321	8.2329	7.2282	6.1122	8.0292	7.1564	8.8529	7.4628	8.9254	9.3842	11.2963	8.4034	13.5812	7.7469	9.916	8.392	11.8768	12.688	11.4632	8.7831	7.5107	5.1193	0.7328	8.6704
TCGA-91-8499-01	36	0	9.1546	7.707	2.1841	12.2892	9.558	7.3051	6.2926	4.5146	9.0587	1.4711	11.084	6.8233	10.0712	8.9663	8.5137	5.7905	7.2779	7.9743	9.5293	7.8689	10.1888	9.1441	4.6224	10.0162	3.7448	8.5997	8.8156	9.6915	10.0874	8.3405	8.8911	14.1465	7.543	9.1944	3.1177	12.4133	11.0161	12.931	7.9155	9.5577	5.9087	7.3888	6.031
TCGA-91-A4BC-01	44	0	8.1075	9.4767	4.1676	8.311	12.041	14.4688	8.5658	0	10.6269	3.4318	8.9111	7.176	9.6665	9.5639	7.5671	4.8286	9.7217	7.0032	9.4591	10.2669	9.3867	7.9865	6.1332	7.3696	7.1161	9.7515	7.4696	8.8266	10.3172	10.7583	5.3278	13.7099	7.9828	9.7967	2.2976	12.2689	10.8882	10.9908	8.2961	7.3066	5.4187	1.2051	6.738
TCGA-91-A4BD-01	603	0	10.731	11.372	5.1726	10.2748	13.103	1.4612	4.4621	0	9.1503	0.9082	8.3576	7.3031	10.2539	6.3042	6.9096	5.8356	8.7294	8.6932	10.3691	10.7201	9.2283	9.2198	5.1726	8.6526	5.429	9.2982	9.2006	9.5315	11.995	11.8617	7.3961	14.0983	7.1777	10.1667	7.4111	13.2407	12.4117	13.402	7.455	9.4614	2.6462	1.4612	6.7775
TCGA-93-7347-01	683	0	10.305	11.6317	4.3523	10.7388	12.208	4.2675	5.184	0.4735	11.3589	3.6616	9.3274	5.3305	10.6653	7.7382	7.2998	7.2269	9.1204	7.3034	9.1686	10.0211	8.9975	8.4792	5.6198	8.2803	5.8703	8.465	7.0393	8.8655	11.2128	10.4061	8.5347	13.3798	6.576	9.9072	6.6671	13.2289	11.8087	12.7915	7.9088	7.632	4.3246	1.1147	6.9194
TCGA-93-7348-01	531	0	10.571	10.053	2.4914	9.8491	15.063	3.8943	5.621	0	10.7394	5.9942	8.8864	8.2518	9.904	8.3888	7.3055	5.8196	9.3608	6.396	9.2755	9.6249	8.5777	8.1514	4.9411	7.9422	7.1532	8.1846	8.485	8.7246	10.4131	9.9295	8.0033	14.7173	6.7982	10.0084	8.5802	11.7398	10.2411	11.5255	7.8466	9.4561	3.8943	1.8647	7.1862
TCGA-93-8067-01	186	0	10.242	8.7034	3.4924	12.7962	10.808	17.2085	5.333	0.3615	8.1648	0.891	9.6743	6.8939	10.7226	8.3143	6.8048	8.4754	9.7155	6.9843	8.5766	8.0808	10.4259	9.5968	5.4123	8.7877	6.7099	8.1591	8.3	9.0621	9.7823	8.4649	5.2382	13.7681	7.6176	10.3005	5.3021	12.471	12.1342	12.2318	6.666	7.9406	4.9095	1.4378	6.2023
TCGA-93-A4JN-01	718	0	9.4852	9.4097	4.6014	12.6984	12.785	5.1664	8.216	0.8729	10.2958	3.0843	9.3253	8.0585	9.6205	7.5612	7.4037	4.5253	9.0204	7.605	8.9587	9.2216	9.6987	7.9093	5.5964	8.8393	7.7089	8.5414	7.9168	8.5621	10.1868	10.9319	6.7963	13.1415	6.6367	9.8954	4.1396	11.5875	10.8949	11.393	8.054	8.0266	4.1396	2.2451	6.1546
TCGA-93-A4JO-01	33	1	9.6175	10.3234	0.6069	10.8997	14.144	4.6168	5.6783	0.6069	10.4854	0.6069	8.9336	7.0812	10.4094	9.069	9.1615	5.9093	8.9836	7.2739	8.6439	10.5557	8.9836	7.6719	5.7075	8.0894	1.6286	8.9505	9.2335	8.994	11.3323	9.4355	5.884	13.4232	6.9036	10.6564	7.001	12.8959	11.8532	12.9549	7.6382	7.5644	6.2036	0	7.9503
TCGA-93-A4JP-01	578	0	10.115	12.9452	3.2245	10.4684	11.272	5.5333	5.8035	1.7591	8.2955	1.1325	9.2134	7.0331	10.1117	7.267	7.9965	6.1965	9.2581	7.4715	9.0966	8.9304	8.3702	8.2226	5.3486	8.5718	4.8491	8.7477	7.8501	8.8117	10.9802	8.8218	4.9453	14.3607	7.1508	9.2711	8.6282	11.8362	13.0441	11.1832	8.6033	8.5183	5.7618	1.1325	6.6319
TCGA-93-A4JQ-01	526	0	9.621	9.3618	4.5912	9.0217	12.999	5.3612	6.3968	1.4561	10.9276	3.344	9.4535	4.2382	11.1573	8.4644	7.6423	6.4116	9.7551	7.3386	9.2173	9.4417	8.7755	7.8957	5.7126	8.3008	6.7287	9.1005	7.2709	8.4944	10.3754	10.0917	6.2392	13.4379	7.3994	9.7463	4.17	12.6012	11.4946	11.4148	8.2845	7.9877	4.7161	2.0184	7.8663
TCGA-95-7039-01	1272	0	10.007	8.5736	1.2801	12.7928	11.562	3.6356	4.3923	2.8931	12.3507	2.1926	8.7079	3.6356	10.0081	8.9142	7.5935	3.9341	9.9297	7.9674	8.579	9.25	9.0032	8.5546	3.9341	8.0891	5.6521	9.0564	8.3392	7.8826	9.4039	9.0757	8.2841	13.6923	5.141	10.1027	5.3825	13.2105	11.2961	12.7337	7.3181	7.2587	4.8861	0	7.4998
TCGA-95-7043-01	503	1	8.6828	16.6882	0.835	8.8805	7.4613	2.8621	5.742	1.3605	9.3668	3.9903	9.0221	7.9026	9.775	8.7047	8.8243	7.7845	7.7162	8.7838	8.2898	8.0464	10.1949	4.7893	3.2664	8.4336	6.4596	8.2754	9.7218	9.2038	9.3599	8.5713	8.4144	14.7833	5.2117	10.0583	3.4842	10.598	8.6746	11.5432	8.3431	8.8612	4.4701	0	3.4842
TCGA-95-7562-01	87	1	10.157	7.1762	2.7729	12.1994	15.354	3.1094	3.9829	0.5349	8.2918	3.1825	9.6453	8.7753	9.4155	8.0346	6.7525	4.3434	7.9715	6.9644	9.1647	10.7916	9.1563	7.9163	5.4475	9.1151	0.9243	8.806	7.6715	9.1788	12.5412	8.3974	5.6926	15.5162	6.4309	9.3641	2.4564	11.9684	12.4145	11.4142	7.2588	7.345	3.0323	0	4.9782
TCGA-95-7567-01	568	0	9.5181	7.7859	3.7417	12.121	7.5272	4.822	6.1845	0	9.6703	1.0876	8.7358	7.9719	9.5501	9.3291	6.723	4.0419	8.625	7.4218	8.7192	10.1249	9.6196	8.7443	5.4233	8.0148	7.5179	8.8404	9.3139	8.4211	10.0064	10.213	6.9202	13.0555	6.6602	10.3493	3.439	12.2855	10.3147	13.0554	7.7877	7.8453	2.9011	1.2669	7.6163
TCGA-95-7944-01	377	0	7.1378	6.8156	3.2905	10.5896	12.287	10.3325	5.7273	0	8.9018	0.45	8.2638	3.444	10.0916	6.6572	7.5208	6.1701	9.366	8.0007	8.9422	9.308	8.8024	7.8804	5.6868	7.6651	4.3505	7.896	6.8062	8.8012	10.8088	9.341	6.1847	13.6398	7.0804	9.6971	5.131	13.2011	9.6293	10.5779	9.362	8.0711	2.5257	0.7925	8.5426
TCGA-95-7947-01	477	0	11.028	8.6085	2.9492	10.7139	13.546	3.6568	4.104	0	9.4895	1.7844	9.6016	10.5746	9.7373	7.5071	8.6994	3.9124	8.558	6.9771	8.5599	9.7762	9.1618	9.1664	4.3181	9.0005	2.6311	8.4872	8.8076	8.5128	10.866	8.2179	3.2096	14.4677	5.7349	9.7625	6.5198	11.5399	10.3954	11.5823	6.7296	8.5708	4.2268	0.3847	4.2732
TCGA-95-7948-01	476	0	10.143	10.8536	3.3977	11.1222	10.758	2.3995	4.9815	0	8.4253	4.8395	8.9048	5.2419	10.976	7.8939	4.9815	2.9833	9.2367	8.7546	9.3538	8.3223	8.7134	6.943	6.171	7.7251	3.1529	8.6115	9.4887	9.1136	13.4883	10.0951	8.9409	13.109	6.3269	9.6391	4.856	13.0046	11.6802	12.5515	7.5305	7.6883	2.791	0	8.6475
TCGA-95-8039-01	830	0	9.9777	9.8225	4.9022	10.2512	13.088	5.8778	4.497	0	10.5787	1.3456	9.0032	5.1096	9.3575	9.7041	6.7566	6.2938	9.4232	7.0234	9.287	10.2943	9.0236	8.6979	6.0045	8.1725	5.6642	8.3934	8.9491	8.8429	10.9545	10.7865	7.9941	13.8615	6.5761	9.6212	6.6388	12.3421	12.6272	12.6546	6.191	9.221	4.8645	0.8243	5.4896
TCGA-95-8494-01	84	0	10.388	8.0902	4.2362	12.0841	8.4661	4.6565	4.1865	0.7113	8.821	0	9.0533	2.6085	10.3604	9.0058	6.6408	7.0887	9.6106	7.7102	9.4293	10.2507	10.145	8.534	4.1865	8.155	3.7126	8.4757	7.8363	8.7963	10.4785	8.4043	5.3404	13.3864	6.2152	10.4746	4.1865	12.8021	12.5491	13.3251	6.6772	8.827	3.0019	7.7363	8.2855
TCGA-95-A4VK-01	651	0	10.604	8.3337	2.7775	12.1492	11.308	5.1948	4.6389	0	10.1586	0.9827	8.6777	6.4412	9.4216	7.7466	6.9484	4.6669	9.565	7.4927	9.6158	8.2894	9.3194	7.5044	5.68	8.4787	7.58	8.3402	7.1201	7.9736	9.6725	11.0308	6.4733	14.0706	6.4331	10.2313	4.5212	12.541	10.8682	11.9744	8.3682	9.3402	4.0964	2.1428	8.0205
TCGA-95-A4VN-01	553	0	10.101	8.8785	0.9459	11.0755	10.779	4.7516	5.834	0.5491	11.2636	0.5491	9.146	5.2847	10.9201	6.5202	7.1336	4.2874	9.4785	7.0654	9.2434	9.8064	9.4776	8.6042	5.3186	7.8944	5.1215	9.2561	7.1618	8.5921	11.0025	9.1401	5.6599	13.7194	7.4601	10.1359	2.9039	12.1681	12.7083	12.082	8.2298	8.3738	5.7118	0.5491	7.0146
TCGA-95-A4VP-01	605	0	8.5954	9.8011	2.3767	9.7369	10.368	13.9552	6.6066	0	9.9476	1.0345	8.9197	5.6961	10.1962	7.7648	7.1283	5.7392	9.6577	8.1633	10.376	7.9956	10.6479	6.9441	5.066	7.2317	7.3941	9.1594	8.1068	8.7753	10.462	9.5975	5.9978	13.6954	8.0509	11.3187	5.2758	12.5024	13.1189	11.9669	9.0467	8.5171	6.2767	1.3632	5.4589
TCGA-97-7546-01	1285	0	10.797	10.9109	3.6418	10.4067	11.298	5.1262	4.5186	0.6059	10.7192	0	9.1103	6.545	8.7676	9.8432	7.4709	5.1262	8.0785	6.4535	8.4756	10.8769	7.5086	8.01	5.0824	8.9092	5.4355	8.3448	7.8232	8.4127	9.4776	10.2411	8.6028	13.4727	5.8682	8.6783	7.5574	11.5746	12.8666	11.3092	8.4082	6.3921	6.061	1.0313	4.7319
TCGA-97-7547-01	1965	0	11.043	9.0867	3.0005	7.9457	13.826	4.1401	5.023	0.9072	10.1182	6.9743	8.6757	9.7315	11.6724	6.8969	7.8829	5.0423	9.531	6.9232	8.6247	11.0111	8.1595	7.9559	4.5436	8.3986	5.8112	7.8748	7.3751	8.3223	9.6901	8.4415	7.3751	15.4712	5.3669	10.264	4.0285	11.7382	10.8518	11.6763	7.9482	6.1503	4.5704	1.6728	4.673
TCGA-97-7552-01	1932	0	10.978	10.7264	4.2929	9.7881	13.668	4.4849	4.0309	0.9487	10.5559	0.5509	9.9081	8.4522	9.1591	8.4035	8.0954	6.9019	8.9096	6.6107	8.8632	10.7084	7.6773	9.0879	5.4675	8.846	6.6107	8.5287	6.5689	8.4881	8.7062	9.4824	5.6523	14.1055	7.507	9.1328	5.5558	11.8681	11.7764	10.7581	8.3714	7.5719	5.8279	3.1547	5.6787

TCGA-97-7553-01	1870	0	9.8269	11.7527	4.9708	10.5171	11.518	4.7006	5.1448	9.6799	11.9321	1.4781	9.5698	6.2649	9.1003	7.2722	7.5169	6.0189	8.6035	7.3493	9.0873	10.9937	8.4755	8.8936	4.1672	8.7442	4.3039	8.4863	7.622	8.1681	9.7301	9.8169	5.7936	14.7851	6.7747	8.9726	9.284	12.2671	12.8058	10.9682	8.1568	7.8569	5.7222	1.6926	4.2372
TCGA-97-7554-01	775	0	10.72	9.4704	3.8587	10.6789	10.604	5.6154	5.5243	0	11.1868	2.5856	9.6108	7.7656	9.6116	5.1706	7.3887	4.2486	8.5282	6.0341	8.1901	9.7405	8.3627	8.0641	3.4601	7.7541	3.5242	8.7489	8.3383	8.1606	10.3925	9.2757	6.5399	12.5715	5.8336	9.1106	4.5553	12.085	10.6094	11.6863	8.0424	7.393	4.7011	6.7623	5.9196
TCGA-97-7937-01	564	0	10.078	8.0142	1.141	11.9289	13.681	4.0591	7.7247	0	9.7808	4.3984	8.6144	6.1802	9.6075	7.5408	7.7732	3.1756	8.5006	7.3621	8.2466	10.3851	8.9541	7.4869	3.9508	9.104	4.7395	8.0714	7.9216	8.7089	11.4277	9.5567	7.7518	11.4834	5.2916	10.0792	3.2383	11.6973	9.1647	11.2406	8.6364	7.7758	2.5413	1.141	5.8791
TCGA-97-7938-01	18	1	11.194	10.8362	2.6866	10.6084	11.934	5.4362	4.2112	3.7098	11.1561	1.1431	8.9851	6.6365	10.0411	8.1113	7.129	5.4159	7.9085	7.7105	8.2446	11.4916	7.9301	9.2137	5.1717	8.6506	6.5738	8.3056	7.1476	8.2147	10.2184	10.3043	7.7756	13.6786	5.2879	9.267	5.7913	11.4001	11.9908	11.7571	7.5799	6.0631	5.5522	1.492	2.0076
TCGA-97-7941-01	484	0	10.354	11.0586	4.2319	10.7369	12.327	4.1814	6.2549	0	9.5074	1.1977	8.2907	9.8953	10.3322	7.9032	7.329	4.9277	9.262	7.4053	9.4444	9.6226	8.2681	8.5062	6.3088	7.9646	8.4499	8.6971	8.5011	8.492	8.8716	10.3069	7.0585	13.1953	8.3143	9.8656	4.3961	12.5486	12.1852	11.6912	9.1767	8.5776	5.4495	2.8371	6.422
TCGA-97-8171-01	568	0	9.9755	7.1237	2.3022	12.6978	8.2372	3.148	4.1351	0	8.5184	0.6431	8.4659	10.0319	11.2499	9.3174	4.6527	6.1137	9.006	7.836	11.5354	9.2134	8.6613	8.2172	7.7855	8.6327	9.1399	9.2794	9.7806	8.7421	10.7915	7.2489	8.1287	13.6222	8.9333	9.3155	6.4924	12.5502	9.6893	11.4061	8.3535	10.172	3.1015	0	7.4337
TCGA-97-8172-01	545	0	10.038	10.7406	3.8575	10.3883	10.381	5.8487	5.3746	0.3387	10.5048	0.6128	9.1486	7.1093	10.0058	8.5806	7.7055	6.4604	9.1364	7.2388	9.0416	10.0692	8.1096	7.2909	6.1087	7.6852	7.8365	9.0868	7.2933	8.663	9.8043	9.1526	7.493	13.781	6.7572	9.1832	6.0349	11.5751	12.3725	11.6947	8.7674	7.9916	6.4253	2.0619	6.0864
TCGA-97-8174-01	164	1	9.8862	10.214	2.9716	12.4147	9.3099	14.6547	6.6175	0.865	9.7643	0.865	8.8789	4.9468	9.9736	7.2824	7.546	5.1606	8.9063	7.8617	8.9323	10.5262	7.976	7.9728	6.0478	8.1537	6.1234	8.8697	8.3773	8.523	9.2497	10.6223	8.388	15.0501	6.6255	9.1908	3.2459	11.8683	13.0057	11.6619	7.9916	7.479	7.9394	0.6299	6.1403
TCGA-97-8175-01	551	0	10.655	7.582	2.3713	11.7094	14.682	5.9266	4.5398	0	9.5518	0.4306	9.9881	2.7893	9.907	7.159	7.0759	5.9673	9.1628	6.9022	8.9157	10.8062	9.4932	8.2641	6.3522	8.5016	5.8241	8.6387	8.368	9.102	11.1169	8.6412	6.6706	12.9942	6.881	10.2174	4.722	13.5764	11.0226	12.8022	8.676	8.7171	3.9961	1.9194	6.5092
TCGA-97-8176-01	468	1	9.9623	10.7472	5.6195	12.9539	12.115	14.1815	5.3307	0.6732	9.7265	0	8.5161	4.6314	10.6846	8.066	6.0272	7.7526	10.0047	8.0369	8.7668	8.9342	9.9089	9.6548	5.3727	8.4717	8.4996	8.1975	7.6917	8.6266	10.8264	8.9568	6.8251	14.3907	8.6135	10.4059	4.7636	12.372	13.2999	11.3853	9.106	8.4465	4.699	1.7564	6.2237
TCGA-97-8177-01	499	0	9.6241	10.7706	5.5014	10.3356	13.282	5.8059	5.9571	0.7543	10.113	5.5864	9.3213	7.5346	10.4569	9.4768	7.1087	5.0566	9.3857	7.9234	9.3701	10.6257	9.1884	7.7081	5.7518	8.925	6.3644	8.9607	10.509	9.4063	10.6316	10.2713	8.4451	14.105	6.715	10.4692	7.0045	13.3109	11.6237	12.5956	8.2773	8.7058	4.6059	0.7543	6.6572
TCGA-97-8179-01	435	0	9.1914	12.2004	2.9254	12.4414	10.317	15.6389	4.323	2.6854	8.6699	0.473	8.3868	3.5207	9.5964	9.2832	6.9453	7.6189	9.7781	8.6863	8.9716	8.4791	9.5588	8.4649	6.6873	8.1875	5.1824	8.8566	8.6009	9.4235	11.3824	8.4232	7.7259	14.3725	6.1624	10.1873	7.7753	11.9766	12.3269	11.8046	9.1237	7.9607	5.2865	1.5559	5.7384
TCGA-97-8547-01	657	0	8.6248	9.7958	4.7158	11.2973	11.222	3.8864	5.2096	0	9.835	8.4011	8.7837	5.5768	10.6377	8.8011	5.5056	7.3455	8.8305	7.7463	9.4895	9.2104	8.677	7.6992	4.4385	7.6389	6.7376	9.7988	8.9448	9.1179	11.2622	9.2778	9.1039	14.1368	6.8472	10.1723	7.3657	13.061	13.1313	11.9064	8.6107	9.2104	3.454	0	7.0359
TCGA-97-8552-01	626	0	11.016	12.2199	5.2321	11.2028	12.833	3.582	4.8904	1.1502	8.6627	0.6869	8.654	9.2091	10.4411	10.103	7.6641	5.5082	9.4009	8.3379	9.3898	9.675	8.587	7.4891	6.1768	8.1513	7.3011	8.8459	9.0423	9.4267	9.4658	11.3924	9.5794	14.2086	7.25	9.7299	8.7894	12.7197	12.7363	12.4139	7.989	8.8111	4.2236	0	8.1586
TCGA-97-A4LX-01	614	0	8.8938	9.346	5.4043	11.8718	13.876	3.7821	5.6261	0	11.0345	2.7482	9.582	5.7727	9.9539	7.0787	8.099	6.8109	8.9883	8.0008	9.6693	10.6491	8.8366	9.3797	4.4084	8.3123	7.1157	9.2821	7.1112	8.3452	10.0225	10.248	6.341	13.8631	6.7711	9.5536	8.3276	12.4877	12.5652	11.4406	8.1265	8.5403	4.4084	0.526	6.7535
TCGA-97-A4M0-01	652	0	10.048	8.9124	4.803	11.2596	12.06	2.0681	4.4585	0	9.4991	2.3521	8.6722	7.4978	11.1305	7.6233	6.385	6.0888	9.2963	6.8346	8.863	10.8801	8.8947	8.3132	5.0415	8.8207	6.9615	10.0214	7.7263	9.1242	11.1375	8.368	7.5123	14.1408	5.3139	10.1199	6.9456	12.1274	14.1296	12.2147	8.5029	7.7991	3.2035	0	7.2393
TCGA-97-A4M1-01	601	0	9.6159	12.7567	3.7789	12.0558	9.3373	7.0517	5.4778	1.9771	8.0595	0.5748	8.3809	8.0029	10.8184	8.754	7.5879	5.1199	9.2125	8.3048	9.9879	10.4722	8.8007	8.0382	6.1889	8.8126	8.1038	9.6276	9.2686	9.442	10.2507	10.9986	8.0436	13.6841	7.2222	10.1273	8.7719	12.1821	13.7401	12.2208	8.6276	8.6453	3.8783	0	6.1792
TCGA-97-A4M2-01	624	0	9.6943	11.69	5.2872	11.4093	11.317	5.5618	4.7029	12.098	12.7839	1.5492	8.7979	6.918	10.8903	7.5275	8.2059	5.9242	9.7101	7.6293	9.7953	9.4004	8.438	8.2905	5.7672	8.2636	7.0017	9.3113	7.8135	8.7193	9.4911	9.1568	7.1411	14.5527	7.7791	9.5226	10.2146	12.9431	13.5886	11.9863	8.3661	8.4988	4.5029	0.9733	8.031
TCGA-97-A4M3-01	540	0	9.8408	12.7113	3.7703	11.5626	11.001	11.4424	5.0823	2.3353	8.8256	2.0125	8.577	5.0162	10.7574	9.0597	6.6386	3.4746	9.9716	8.4658	9.1766	7.3829	9.3182	7.1714	5.6602	8.5758	7.226	8.8352	7.689	8.5085	9.7488	9.2634	8.0692	14.4738	6.9881	10.2334	4.849	12.1569	12.4162	12.6263	9.0223	8.3742	5.2061	1.0083	5.0606
TCGA-97-A4M5-01	634	0	10.086	11.3757	4.6118	12.0895	10.149	13.3883	6.5017	3.1629	9.0243	2.591	9.0845	8.0212	10.7819	7.2771	7.5464	5.0731	10.6166	7.7897	9.0368	9.1529	8.7349	7.83	4.5869	7.73	7.1425	8.756	8.5295	9.045	9.2632	9.7139	6.1226	14.2152	7.8589	10.0781	8.3014	12.5214	12.6181	11.6886	8.3541	8.1796	5.3792	0.8777	7.6003
TCGA-97-A4M6-01	568	0	10.096	10.5821	0.5429	9.1762	13.385	3.8328	5.1402	0.5429	10.2618	3.2751	9.05	8.104	10.3346	9.3519	7.9392	5.502	9.8693	7.5359	9.3791	9.8037	8.5195	6.7228	5.1023	8.4834	7.0001	8.6499	8.711	8.3581	9.7155	10.1908	9.1807	14.0756	6.995	9.8764	7.4214	12.0585	12.0079	11.5313	8.9376	8.4831	2.6966	0.9364	4.5192
TCGA-97-A4M7-01	629	0	10.041	10.3885	4.8546	10.8908	12.949	5.2463	6.1507	0	10.3549	1.6548	9.1372	8.7231	9.5512	8.292	7.6123	7.0592	9.3545	7.7937	9.1059	10.443	8.4545	8.131	5.4156	8.6935	8.2106	8.7186	8.1767	9.2113	10.4269	9.7199	9.2403	13.6028	7.4338	10.1137	7.8935	11.9985	11.9968	12.0015	9.1677	9.0857	5.6803	2.003	6.6964
TCGA-99-7458-01	747	0	10.302	10.1774	3.0502	9.4638	14.39	5.1468	5.3122	0.995	12.1646	5.921	9.4705	5.6437	9.9511	8.312	6.9864	6.3061	8.4873	7.8522	8.7461	10.7168	7.8039	8.5738	4.763	8.675	5.0053	8.5637	6.9598	8.2412	10.7304	9.2832	6.2318	14.4411	5.7976	9.0678	6.8061	12.0332	12.7966	11.7743	7.9769	8.1038	6.0052	1.2167	5.7716
TCGA-99-8025-01	1060	0	10.678	10.8026	2.8069	12.6065	12.116	4.8903	5.8154	9.1357	10.8989	3.3215	8.3541	7.2849	10.3065	7.5694	8.5668	3.874	8.2647	7.7918	8.5389	9.2686	10.007	8.1778	4.8408	8.2176	5.156	9.3199	8.506	8.3497	11.0451	10.2451	7.0219	12.2295	6.1011	10.3872	4.0293	12.9728	10.8502	13.0176	8.3245	8.0311	4.4146	2.9996	8.6494
TCGA-99-8028-01	1118	0	9.1848	10.6184	5.2975	10.6129	12.51	5.8755	7.9403	1.1966	11.5574	0.8963	9.0967	6.4734	9.3819	7.367	7.925	6.3185	8.839	7.117	9.1141	10.3244	8.7808	9.7903	5.8434	8.8578	6.5215	8.8498	8.5249	8.6784	10.8942	11.7752	5.7426	14.212	7.0005	9.9478	4.3491	13.4908	12.5595	12.7687	8.931	8.1005	5.4041	1.8416	7.5136
TCGA-99-8032-01	44	0	9.4599	9.6939	3.3767	10.079	14.399	14.5606	8.3609	2.0458	10.4792	3.43	9.4283	5.76	10.3431	7.1237	6.7201	5.8989	9.2873	7.1398	9.387	8.6366	9.1766	6.5195	5.9816	7.8445	4.5419	8.3931	6.4437	8.3847	10.4729	10.6037	7.4835	13.3565	7.6668	9.9775	3.9146	11.6	11.258	10.663	9.0535	7.4866	4.866	1.9022	4.9414
TCGA-99-8033-01	656	1	8.9403	11.1116	4.3597	11.7035	14.678	16.507	5.1447	0	9.6756	1.3576	8.2261	6.9133	10.296	6.474	7.8202	6.3202	8.4902	8.2299	8.2592	10.3437	9.4273	8.408	4.1849	8.4484	6.6186	7.985	8.6573	8.1207	10.764	8.8157	5.4325	13.6287	6.1951	10.2016	5.9887	12.4256	12.3706	12.2015	8.0438	7.7772	2.1749	2.9337	8.6753
TCGA-99-AA5R-01	658	0	8.7258	12.2393	2.8015	11.1938	11.233	5.3445	6.7971	3.6942	10.3305	2.0899	9.0359	5.6862	10.2912	7.1947	8.865	7.3941	9.2257	7.8117	9.3187	10.1284	8.3898	8.049	5.817	8.3971	5.803	9.2504	7.4354	8.8084	9.4382	9.4189	5.9111	14.2493	7.665	9.4223	8.9434	12.4239	14.0512	12.1143	9.2086	7.9543	7.0065	0.6256	7.4974
TCGA-J2-8192-01	739	0	10.024	11.0333	2.9556	9.4033	14.435	6.0917	5.56	0	10.2821	7.9055	9.4773	5.5484	8.8961	7.3133	7.1926	6.4548	8.8353	7.3733	8.8032	10.8834	7.8502	8.1558	5.3503	9.0795	3.0238	8.3575	7.6382	8.2847	10.9129	10.5896	6.986	15.387	5.8964	9.3378	8.4778	12.0687	10.7205	10.8899	7.9931	7.9781	5.2962	1.3229	5.1513
TCGA-J2-8194-01	724	0	9.8555	10.0069	3.3446	9.3833	13.184	15.8892	5.5347	0.5222	10.7421	2.0189	9.3050	4.1706	10.4849	9.0319	7.1215	7.5916	9.9571	7.7951	9.1713	10.2386	8.2359	7.9933	5.3618	8.4027	5.4934	8.9143	7.6046	8.1254	11.9802	9.5966	5.872	14.8581	6.2646	9.4939	3.3446	12.127	11.9149	11.0208	8.5548	7.2134	5.8933	1.4565	6.2728
TCGA-J2-A4AD-01	550	1	10.87	9.464	4.9563	10.7882	14.098	4.4401	4.4668	4.1413	11.0075	4.8788	9.4125	8.1517	10.1148	7.4955	8.0338	6.5035	9.5494	8.0691	8.5943	9.0631	9.4789	9.747	5.716	9.1965	3.7203	8.328	7.9053	7.9907	10.2448	8.4948	6.6271	13.826	5.5	10.354	5.0477	13.232	11.6064	11.8884	8.162	8.8165	3.479	1.3918	7.489
TCGA-J2-A4AE-01	1079	0	10.475	11.0819	3.3863	10.6804	11.525	4.0327	3.7454	0.6699	9.4171	0	9.1656	7.6323	10.5621	8.0921	7.4147	5.4642	8.4742	7.3271	9.4016	9.7318	8.6047	8.0637	6.5145	8.471	5.9513	8.7021	8.0509	8.5557	10.8298	10.8944	7.5663	14.066	7.1702	9.4479	6.4958	11.8215	12.1021	11.7225	9.1096	8.3332	4.3156	2.5181	7.0911
TCGA-J2-A4AG-01	988	0	10.192	9.2433	3.8588	10.3611	10.717	3.9666	5.3953	0.6441	10.475	1.0879	9.4617	3.8588	10.19	9.5746	7.781	4.9462	8.9938	6.6608	8.4156	10.9467	8.1038	8.6696	5.4887	8.681	6.3182	8.3772	7.2885	7.8138	9.517	9.9036	6.3682	13.375	6.9308	9.3775	6.1906	12.2635	11.1332	11.5476	8.6428	8.2943	5.1198	2.3045	8.5453
TCGA-L4-A4E5-01	578	0	9.4304	8.2569	3.0498	12.0531	12.858	4.1568	6.6533	8.8388	11.5146	2.6917	8.3703	5.3431	10.3844	10.0566	6.9367	6.1234	8.9359	6.7046	9.639	9.7749	9.1624	8.7379	3.5755	8.485	7.1915	8.7878	8.6043	7.3885	11.703	8.8909	6.2837	12.7571	7.0549	9.7215	3.9171	11.871	10.6756	11.792	7.2444	9.4285	3.4	1.7116	6.1139
TCGA-L4-A4E6-01	435	0	8.6571	12.0408	6.9468	11.2133	7.7697	5.6582	6.6912	0.638	10.7577	0.638	8.9751	4.702	9.8384	7.287	8.2299	5.2788	9.3346	7.124	9.7835	10.2822	8.2538	8.1865	5.8232	8.5691	5.8788	8.938	7.5322	8.4661	9.1923	9.2195	6.3415	15.5215	7.8429	9.5533	7.3621	13.3674	13.3879	11.718	9.0692	8.6024	6.1738	0	6.7888
TCGA-L9-A443-01	193	1	10.281	9.3925	1.0862	10.6431	10.653	2.5203	6.8734	3.0532	11.539	4.2023	8.7873	6.2842	10.0205	8.5312	8.2911	4.6523	8.8968	7.097	8.9555	10.5931	9.0514	7.5929	5.9976	8.5266	7.8844	8.8554	7.7289	7.8178	11.3464	9.5105	8.6017	14.0915	6.4134	9.8371	4.3684	13.0175	11.3551	12.0534	7.8106	9.7866	5.02	2.3019	7.9049
TCGA-L9-A444-01	307	0	9.857	8.2938	4.0828	11.0613	10.057	4.3207	5.167	1.3326	12.0259	3.2253	9.2418	6.5134	10.5925	7.8896	7.1521	6.5134	9.417	6.9365	8.7911	9.4377	8.2369	7.7793	5.4686	8.1319	7.1901	9.4472	8.2692	8.6616	11.3111	9.7893	6.2221	13.5834	7.0971	9.2508	3.9474	13.0279	11.185	12.0684	8.0635	7.0317	3.631	0	6.8442
TCGA-L9-A50W-01	442	1	10.119	7.8748	2.8078	8.8578	15.706	3.8953	5.8111	0.4595	9.5424	1.8584	8.421	8.3264	10.626	9.6297	5.7114	7.4014	9.1503	7.461	8.9868	9.23	8.8992	8.5187	4.4099	8.2094	9.0558	8.6646	7.9357	8.3956	10.3428	11.4335	7.7868	13.9009	7.302	10.1284	4.0339	12.057	10.3814	11.6438	7.648	9.0969	4.2485	1.5239	5.5704
TCGA-L9-A5IP-01	58	1	10.051	15.545	3.6315	7.2538	14.348	12.8369	6.0791	1.2108	8.4286	2.0241	7.9268	4.0301	10.8384	9.1035	7.0058	7.1419	10.4374	7.7849	9.1789	9.6849	9.1443	8.121	4.2454	7.9946	3.7301	8.5089	7.1508	8.5381	10.7065	8.7065	3.4697	14.2048	5.6462	9.9704	3.2875	12.8773	12.4168	11.9352	8.0101	7.2579	3.9506	2.0241	8.412
TCGA-L9-A743-01	664	0	9.7424	10.4546	4.4446	11.2299	13.333	5.8844	5.9298	2.3921	11.7313	0.9593	8.9903	6.673	10.1152	8.5757	8.2304	5.8376	9.1947	7.2216	8.9982	10.0639	9.2739	7.9675	5.519	7.9429	6.426	8.7265	7.8425	8.6386	10.626	10.2085	6.776	13.7692	7.0054	9.8092	5.2232	12.3104	11.6341	12.2955	8.9175	7.9891	5.6738	0.5579	8.0183
TCGA-L9-A7SV-01	565	0	9.2124	6.4114	1.0919	13.6392	6.2094	6.8957	5.2609	0.4617	10.9396	0.4617	8.6591	5.3302	10.4074	10.7348	8.4509	3.2744	8.7002	7.6879	8.6644	8.1909	9.6478	9.0769	5.3302	8.2614	8.1815	8.6537	9.77	8.0746	11.0272	8.6865	8.7351	11.5842	5.7191	9.4363	6.5890	10.9556	10.2675	12.3446	9.2913	10.0393	3.3296	0	5.2323
TCGA-L9-A8F4-01	476	0	9.5421	9.1848	3.76	12.8596	10.544	4.0423	8.1118	5.3775	10.4824	2.4857	8.9054	9.0558	10.1631	7.6475	7.4683	6.9875	9.9064	7.6861	9.2262	8.4121	8.8914	8.5245	5.2406	8.6034	5.2565	9.0828	7.0204	7.7331	9.6926	10.5452	5.3482	13.0004	6.8373	9.5717	4.0784	12.151	10.8218	12.3351	7.4615	7.5284	2.3737	0	6.997
TCGA-MN-A4N1-01	827	0	10.006	8.1175	2.8545	12.499	12.702	3.5094	5.4527	3.5611	8.0512	2.5816	8.579	10.106	10.627	7.7228	7.5544	2.1124	9.9517	7.3343	9.0935	8.9884	10.7439	7.8373	5.6083	8.2091	6.1458	8.3847	9.6424	8.7751	11.6472	8.2883	7.0156	12.0002	7.5123	11.1498	4.5507	13.1326	11.2324	12.3564	8.7165	8.6964	2.8545	0.5013	7.9699
TCGA-MN-A4N4-01	1175	0	9.7025	10.3819	4.6452	12.8644	13.26	4.8752	6.435	5.8383	10.8935	4.9667	8.8465	10.2673	10.0021	9.2935	7.6293	2.9498	9.8237	7.7788	8.907	8.8145	8.8037	8.3773	4.3376	7.9148	3.6475	8.5068	9.0248	8.6892	10.4817	10.3662	4.6726	13.0349	6.2831	10.2712	3.4712	12.8732	14.0295	11.9704	8.4425	7.4387	3.3404	0.9715	7.9399
TCGA-MN-A4N5-01	84	0	9.3174	8.168	1.3645	11.8468	10.779	4.9133	7.0239	1.3645	10.1458	0.8379	8.3583	6.3029	10.3784	6.9009	7.6595	2.053	10.5668	6.9384	8.7072	10.0456	9.5436	8.947	5.0046	8.1527	4.9688	8.7748	8.3108	8.1817	11.1469	8.8978	6.4851	13.2141	6.7821	10.3132	5.2924	12.1861	13.2272	11.8408	8.0007	8.4794	4.754	0	8.6087
TCGA-MP-A4SV-01	2620	1	9.8848	8.8244	3.744	11.5969	11.299	4.0348	6.1521	1.4383	11.2007	2.8044	9.3753	6.47	10.0941	8.9507	6.4489	4.9706	9.084	7.6669	9.1822	10.4773	9.2542	8.4258	5.6113	8.5483	6.3465	8.6634	7.9449	8.2198	10.8736	8.9681	8.3465	12.9938	7.0703	9.4246	4.6405	12.0507	11.1243	12.4596	7.8965	9.0942	3.4374	1.1907	7.2553
TCGA-MP-A4SW-01	1778	1	10.158	8.802	4.7526	11.3097	12.968	3.3374	4.9567	0.5414	9.8368	3.4618	8.8836	8.8555	11.5075	7.036	7.4695	6.3505	10.6633	7.843	8.8304	9.6483	9.5527	8.0896	4.703	7.7666	6.4665	9.0011	7.8963	9.1333	10.6029	9.3083	6.6012	13.8106	6.444	10.7398	5.7863	13.0059	11.5146	12.6451	7.3254	8.8079	3.2011	0.5414	8.1277
TCGA-MP-A4SY-01	1501	1	10.401	10.6928	3.6655	12.1709	14.222	5.8723	6.2542	0	11.0256	6.5086	9.1133	5.1376	10.5322	8.1852	8.488	5.0648	9.1827	7.3403	9.1869	9.997	8.5642	8.5855	6.024	8.4598	6.8273	8.6799	7.9529	8.0875	9.2773	9.3258	7.8591	12.944	6.8273	9.5925	8.1193	12.5606	12.3087	11.4895	7.8429	8.7352	3.5097	1.847	5.1197
TCGA-MP-A4T4-01	2617	1	10.731	8.6568	4.7857	10.2002	10.847	4.3458	6.5736	1.9635	10.0105	5.5668	9.24	6.0913	9.9625	7.2275	8.3219	6.8644	9.3273	7.3984	9.5789	9.8315	9.5038	8.2999	5.04	7.9007	5.04	8.9241	8.1086	8.455	9.3563	10.0299	5.1619	13.6462	6.3092	10.0279	5.0186	12.4197	12.3126	11.6059	8.0011	8.5134	3.762	0.5688	7.6095
TCGA-MP-A4T6-01	1790	1	10.154	7.9177	2.9916	9.524	13.057	3.5824	5.8783	0.45	8.6593	1.3009	8.5718	9.6069	10.6791	9.2237	7.6312	5.5354	10.6238	9.0293	9.4316	8.5704	8.6774	7.3818	5.2465	8.354	5.3537	8.3241	7.6988	9.0533	10.2445	8.5466	9.3872	15.1253	6.6517	10.2309	6.0555	12.1235	10.8065	11.6478	7.8009	9.5566	2.6144	0.7924	7.1375
TCGA-MP-A4T7-01	167	1	9.5975	10.1614	4.7325	9.9723	12.407	14.4136	5.5234	0.5272	8.9044	0	8.7023	6.1071	11.8599	6.5759	5.9615	5.4525	9.609	7.4855	8.8396	8.8884	9.4579	7.7269	6.3052	8.1408	7.944	8.4399	9.4714	8.6462	10.118	9.4954	6.2726	14.1437	7.4201	10.3152	6.5076	13.0064	12.3415	11.8974	8.9463	7.9232	4.0773	2.549	8.5062
TCGA-MP-A4T8-01	161	1	10.367	8.767	3.0249	11.1869	10.764	13.6608	6.2108	0.5058	9.7323	5.0407	9.0445	4.3145	9.6927	9.3147	5.3239	6.4788	9.6089	7.7094	9.3768	8.2687	9.5933	6.7893	6.6333	7.5255	5.3087	8.5021	8.2126	8.4072	10.878	10.0878	6.3436	13.2648	6.4201	10.3297	2.1242	12.109	11.3331	11.7529	7.1508	7.8785	4.5133	1.6321	8.47
TCGA-MP-A4T9-01	1265	1	9.1879	11.5035	3.3448	12.2725	9.7882	4.5424	6.4695	0	10.9001	0	8.6865	4.5909	9.3623	8.208	7.4317	6.5142	9.7395	7.6128	9.5046	9.9923	9.1601	8.4847	4.5909	7.8617	6.708	8.8535	8.0801	8.4831	10.4837	9.4543	5.8267	13.8978	6.7298	10.0007	9.3366	12.1399	13.3298	11.3938	7.8228	9.2402	6.1677	0.4836	6.5757
TCGA-MP-A4TA-01	950	1	9.6344	11.5189	5.8057	10.8532	14.05	14.712	6.3104	1.7756	9.8423	1.1452	9.5904	7.9648	10.7925	9.4189	8.0464	7.0386	9.5506	8.6859	8.7947	9.0765	10.2427	7.7684	4.0312	9.156	6.0004	8.9966	8.7386	9.9083	10.3273	9.1945	5.9447	13.0531	9.3338	10.9049	4.2614	12.0721	12.0126	12.539	8.2187	8.8789	2.9758	1.3873	5.8468
TCGA-MP-A4TC-01	74	1	9.9388	10.0939	4.9125	11.8433	14.032	6.4352	7.3684	0.541	9.2017	6.0556	9.1644	6.3084	10.1984	8.2414	7.9304	4.362	9.4032	6.8593	9.0343	10.9028	9.9725	8.1311	4.0012	8.3574	4.7992	9.1961	8.1427	8.3541	10.2215	11.5453	5.496	14.2289	6.3967	10.2584	2.6914	12.7899	9.8276	11.8974	7.7348	7.2354	3.7316	2.0651	7.0196
TCGA-MP-A4TD-01	307	1	8.697	10.4377	3.9805	10.568	10.463	14.0336	6.6729	0	10.894	6.8923	8.3866	5.7211	10.7804	8.7623	6.8559	7.6608	9.6321	7.3845	9.789	8.0155	9.2402	7.7208	6.1191	7.0093	7.2368	8.8801	8.1617	9.0507	10.7287	10.6027	7.1443	13.4564	7.9218	9.8891	5.3017	11.8068	12.6156	12.6265	9.2081	8.8922	5.372	1.3095	7.4871
TCGA-MP-A4TE-01	896	1	10.223	6.8902	2.1777	11.8335	12.101	16.0088	7.9447	0	8.6664	6.3685	9.0727	8.4796	10.4879	9.1485	4.2189	8.0127	9.7161	7.5664	8.922	5.2812	9.3596	8.2102	6.4916	6.7378	7.0232	8.812	6.1684	9.1563	10.2922	8.4009	7.8293	12.8491	8.9712	9.9234	6.8188	12.4607	11.2512	12.0514	9.1496	8.4884	3.5327	2.0299	5.5891
TCGA-MP-A4TF-01	336	1	9.2782	6.4904	1.7853	8.9592	13.637	10.5031	4.9243	0.8605	7.7073	0.8605	9.0553	4.6707	11.09	6.4639	5.5446	6.0499	10.3751	6.2331	9.0294	9.3376	9.2043	7.8053	3.894	9.116	4.7603	8.5924	7.1258	7.6674	9.9602	9.4326	5.2978	13.065	6.3532	10.2978	5.1061	11.2986	9.6088	10.9747	7.644	8.1948	2.3443	0	5.3274
TCGA-MP-A4TH-01	741	0	10.185	9.9165	3.5372	11.1266	12.098	5.8281	5.9079	0	10.4822	0	9.0234	3.2874	10.7561	6.3768	7.6786	5.2959	9.4326	7.0939	9.0413	8.8583	8.2891	7.4312	4.7704	7.4847	6.6262	9.1704	6.7113	8.287	9.4403	10.9958	6.3848	13.8101	6.5142	9.7771	4.9533	11.5349	10.8111	11.18	7.7202	7.0189	5.5154	0	7.0393
TCGA-MP-A4TI-01	429	1	9.4419	8.8039	3.8913	10.4108	12.475	5.164	6.2447	0	10.8563	0.597	9.6319	7.3335	9.7807	7.3754	6.8997	6.3299	9.6984	7.1648	9.2873	10.1663	9.7789	7.7669	5.3568	7.9917	3.1191	8.8952	6.59	8.5864	9.724	11.088	4.5578	13.9104	7.2004	10.0022	3.6178	12.9648	11.443	11.9413	8.0243	7.9213	4.4578	1.3435	7.2929
TCGA-MP-A4TJ-01	339	1	10.205	11.4277	3.283	9.775	13.315	5.4812	7.2977	4.6698	12.0837	2.4241	9.2827	7.7713	9.2562	7.7539	7.6129	5.7622	9.0577	7.0057	8.6494	9.883	8.844	7.2734	5.0581	8.239	5.9973	9.041	6.9457	8.3521	9.9225	10.445	6.6768	14.2928	7.5403	9.7764	6.2827	11.904	10.9327	11.2456	7.9753	8.0953	4.0635	2.4241	6.1471
TCGA-MP-A4TK-01	582	1	9.3351	10.75	4.1966	10.8933	14.353	6.3959	6.6086	1.3138	10.4827	7.6303	9.3072	3.7423	10.0695	8.6392	7.3307	6.4044	9.1787	7.5639	9.3856	9.252	9.576	8.4642	4.6016	8.0461	6.031	8.454	7.7177	8.5789	10.3995	10.2146	5.616	14.1442	6.5411	10.1854	8.657	12.615	11.8385	12.2147	8.2925	7.4252	3.6879	0.9932	7.4418
TCGA-MP-A5C7-01	2248	0	10.294	11.568	3.1513	10.9263	12.53	3.9174	3.2172	0	8.2417	0.5008	9.0445	9.0814	10.5757	8.4917	7.484	6.3488	10.357	7.9021	9.2168	6.8779	8.5678	8.5897	5.4781	8.4893	8.4884	9.0891	8.1975	8.5164	9.5902	8.1832	9.2995	14.0764	8.1934	9.9082	6.1692	11.3699	12.0805	10.6952	7.0413	8.5049	4.3867	1.1667	3.9565
TCGA-NJ-A4YF-01	2161	0	9.8324	7.479	1.1474	11.4262	12.741	16.3591	9.8082	1.497	8.4847	2.6933	7.8533	6.3859	10.6771	9.1934	7.7795	4.265	8.8039	6.9928	8.8677	7.0535	9.4679	8.1748	4.3534	7.273	7.3825	9.7483	9.0585	8.4823	10.871	8.308	4.3534	12.8543	6.147	10.609	3.4223	12.4168	11.115	12.8594	10.2284	7.8647	3.5018	0	8.754
TCGA-NJ-A4YG-01	2261	0	9.8016	9.5359	3.7142	10.3041	12.997	3.8931	5.1319	5.0589	11.8275	4.1015	8.2648	5.9181	12.1443	8.1551	6.8567	4.7844	9.1802	7.4917	9.5785	8.8973	8.8563	7.6285	6.4338	8.0217	5.8183	8.962	8.7539	9.1171	10.6968	9.5747	7.4824	14.1465	7.0514	9.9739	8.5972	12.2471	12.6828	12.41	8.7384	8.2068	3.435	1.9586	8.2402
TCGA-NJ-A4YI-01	4	1	9.8831	10.3354	4.402	11.9849	6.9988	8.6137	6.2388	0	9.0789	2.1233	7.7493	5.7723	10.4374	8.3981	6.9987	6.6198	10.3074	7.2485	9.6861	11.2763	8.8917	7.8907	5.2193	7.7382	7.2612	8.5634	6.7151	8.548	9.8435	9.8826	6.8886	13.1135	6.6198	10.1938	9.4385	13.24	12.0476	12.5646	7.5363	8.6719	3.1789	2.9474	5.7737
TCGA-NJ-A4YP-01	50	0	9.4985	7.3337	4.6222	11.7505	9.2574	13.0903	6.4742	1.1829	10.1708	5.8809	8.7053	8.2592	11.2328	7.3585	6.8207	3.1906	9.1532	7.3428	8.9334	9.3144	9.4688	8.1653	6.1471	8.4278	7.0784	8.0033	7.5565	8.6977	10.0957	10.1944	5.2418	14.0502	9.1932	10.2897	4.7503	12.5924	9.5096	12.4606	7.5774	8.8982	4.8554	1.0117	9.6292
TCGA-NJ-A4YQ-01	1432	0	9.8036	15.1657	3.6923	7.8966	16.168	6.2302	6.7761	0.7028	10.6082	1.5277	9.9744	7.0049	9.7323	8.4305	8.355	5.5492	9.5981	7.8225	10.0093	9.7914	9.3924	6.009	3.8261	8.7616	3.0931	8.615	7.326	8.3015	10.1781	9.2978	4.5217	14.8176	6.5912	10.6723	4.6354	11.3477	13.0801	12.3767	8.6311	8.7683	3.1955	0.7028	6.6374
TCGA-NJ-A55A-01	15	0	11.491	12.727	3.5224	11.9965	11.431	4.1383	4.8567	0	11.0343	6.1661	9.1418	10.066	9.477	6.9874	7.5349	6.4805	8.3025	7.4795	9.4735	9.5811	8.4176	8.4213	5.7636	8.3032	4.8996	8.6922	7.2748	8.4576	9.7251	9.4229	7.6773	13.7115	6.7502	9.177	6.5494	11.6641	12.0116	11.862	7.9916	7.9514	4.1383	2.1689	7.8621
TCGA-NJ-A55O-01	13	0	9.9672	9.5454	5.0735	11.826	9.7055	13.0089	4.0152	1.1156	9.2762	0	8.9312	6.2639	10.2593	7.4644	7.4597	4.799	10.7509	6.9141	9.1466	9.3347	9.144	8.3173	4.1157	8.4119	6.2307	8.5883	8.4068	8.4981	9.7092	8.7338	5.7881	14.2203	7.26	10.3741	6.15	12.6855	12.1364	11.5512	8.4817	7.8727	5.1226	0.663	8.972
TCGA-NJ-A55R-01	603	0	9.7426	11.6712	4.5599	12.4973	7.4182	7.9598	6.4865	0.9717	9.4786	3.2342	11.0116	9.5849	10.2341	8.8453	8.3234	7.5833	10.7826	7.8072	9.3637	6.9849	9.0449	7.5743	5.3365	8.3614	8.2906	8.6469	7.2286	8.4976	9.1475	9.7401	9.1938	13.974	8.9415	10.1943	5.4199	11.393	13.3948	11.3573	9.6069	8.8056	4.2495	1.9573	7.427
TCGA-NJ-A7XG-01	617	0	9.936	7.4513	2.2214	9.8845	14.417	1.7179	3.135	0.938	8.5317	2.2214	7.642	9.7691	10.1786	6.406	7.2876	4.4025	9.932	9.0521	9.0406	10.6427	9.0001	7.3375	6.3423	8.039	6.5398	8.7205	8.3853	9.1908	10.7301	9.5607	8.5878	14.2455	5.7084	9.8782	2.3565	12.111	9.4954	12.6739	8.3554	7.8104	2.3565	0	7.2447
TCGA-O1-A52J-01	1798	1	10.184	14.5048	4.6394	10.1945	11.805	4.3989	3.335	0	9.4384	0.5641	8.1703	9.1618	9.7266	10.4805	4.6394	6.5146	8.7695	8.7622	10.0483	10.5406	8.2721	8.6108	5.1082	7.8189	7.0297	9.2427	8.1116	8.931	9.7155	10.3727	10.5741	14.0359	5.8324	9.5346	7.5479	12.5183	13.3339	11.7682	8.575	9.771	4.0695	0.9686	6.5371
TCGA-S2-AA1A-01	513	0	9.5505	9.771	3.6015	7.9234	13.569	5.0201	6.671	7.5525	10.7139	1.2081	8.8257	7.1617	10.6007	6.7632	7.8016	6.2545	9.0545	7.2325	9.3084	9.1804	8.3134	6.7076	5.5301	8.3338	7.522	9.1458	7.7803	8.777	9.6445	10.313	5.6666	13.0596	8.003	10.2484	9.2046	11.6948	11.3527	11.4895	8.9927	8.2742	5.6479	0	7.5424

id	futime	fustat	HAL	HEMK1	HGD	HK2	HMGCL	HMOX1	HPD	HYI	IL4I1	IMPA1	INPP1	INPP5E	IPMK	ITPKA	ITPKB	JMJD7-PL	KHK	KMO	LAP3	LDHAL6B	LDHD	LIPC	LPCAT4	LRAT	LYPLA2	MBOAT1	METTL2	MGAM	MGLL	MIF	NAGS	NAT1	NEU1	NME2	NME3	NME4	NMNAT3	NNT	NOS2	NPL	NUDT2	OPLAH	OXCT1
TCGA-05-4244-01	0	0	7.6809	9.7151	5.9804	11.2493	9.9933	8.9571	2.0502	8.3638	8.7718	9.6546	7.9034	7.997	6.0351	9.037	10.5259	9.0971	7.3863	6.244	10.8333	2.1669	5.775	2.6406	8.3958	5.4913	10.3752	10.6849	8.1162	2.7937	13.3041	11.7581	8.851	7.2394	11.042	13.0094	9.5523	11.0354	6.9031	10.7182	3.2292	8.1216	7.1535	9.2447	9.9046
TCGA-05-4249-01	1523	0	5.3087	7.9062	3.4986	10.7054	9.4026	10.6197	0	7.7418	8.1831	9.0206	8.2477	8.22	6.5943	3.3241	10.6894	8.4723	5.5873	5.7552	11.4938	1.194	6.6275	2.6945	8.2973	7.855	10.3565	10.1005	7.7183	4.9958	12.3388	11.5286	6.7577	7.4659	11.126	12.8336	9.2719	10.5408	6.0168	10.5443	4.1256	8.1333	6.939	10.1098	7.2013
TCGA-05-4250-01	121	1	2.3159	8.8442	8.8639	10.8502	9.6429	9.9369	0.9962	7.7432	7.6241	9.5899	9.2428	7.3383	6.6219	11.2575	9.1431	7.8873	7.7453	6.1283	11.8226	0.734	6.7385	2.2165	10.0554	6.1355	10.3275	8.8535	9.7149	1.7317	13.4039	12.0569	8.3353	7.9369	11.212	13.9697	9.0639	10.9635	5.0942	10.2709	5.2279	8.0332	7.8127	8.1303	8.9232
TCGA-05-4382-01	607	0	6.8292	8.4859	5.9547	12.1288	9.8027	11.1083	0.9688	8.4675	11.2052	8.4108	8.6978	8.1208	6.2953	5.0885	9.3406	8.1803	7.4321	7.4979	11.7735	1.2844	5.6997	4.1882	9.0695	6.0411	10.4142	9.4269	8.3012	7.0142	12.9026	12.7837	7.7071	8.2914	11.919	12.6764	8.6015	10.7639	4.9501	10.7621	5.933	9.9363	7.8986	8.2825	8.1046

TCGA-05-4384-01	426	0	8.6168	9.3545	10.2105	9.699	10.077	11.3336	0.6879	8.9858	7.0435	8.5217	8.0919	8.6719	5.9982	7.9279	10.2978	9.315	6.8669	7.3288	10.8458	1.7839	9.0365	2.2219	8.903	7.5256	10.6457	8.9853	8.1887	3.509	11.7608	11.6035	7.2981	7.5865	10.488	11.8006	9.3983	10.3331	5.6401	10.1215	6.0916	9.2728	7.8912	9.2295	9.2842
TCGA-05-4389-01	1369	0	7.6678	8.5001	9.1871	7.9983	10.137	10.2105	0.4175	8.4083	9.4159	9.1059	8.004	7.587	6.3315	6.3375	10.5181	8.1862	7.5236	6.9863	11.6685	0.4175	8.2304	2.5105	8.8116	7.5554	10.5188	8.4275	8.6414	2.1229	11.7986	13.1209	7.6654	6.76	12.374	13.766	9.3263	10.7179	7.7238	10.9282	9.1355	9.0311	7.5436	9.0882	9.2496
TCGA-05-4390-01	1126	0	6.9958	8.7848	7.0436	12.6882	9.8899	10.1043	0.7126	8.7415	8.2665	9.8565	7.5507	7.6226	6.5187	7.4006	9.4819	7.2208	8.2626	6.0487	10.5007	0	7.0366	2.0681	8.4181	5.6672	10.5739	9.7695	8.7229	2.0681	12.0393	13.5758	7.5408	6.7157	11.284	13.1493	10.0401	11.305	3.115	10.9271	4.138	7.5058	7.7734	9.179	7.9809
TCGA-05-4395-01	0	1	11.88	8.8124	8.982	10.8282	10.602	8.4731	4.1628	8.2636	8.3961	9.1373	8.5892	7.3404	7.3115	7.2312	8.6902	7.168	5.0717	7.1054	11.5553	0	7.4262	2.1666	10.105	7.6059	11.4205	7.5859	8.5832	1.9829	8.719	13.2631	9.3361	6.9252	10.476	14.4267	10.3425	11.7534	5.0063	6.8432	3.9029	7.8437	8.6475	8.5234	5.6241
TCGA-05-4396-01	303	1	9.3136	9.659	10.4122	9.3841	9.5897	8.801	0.8141	8.5897	6.583	8.7813	7.5332	8.4249	6.6484	7.7969	10.0418	9.2214	5.9461	5.4004	10.721	3.336	8.7887	4.3186	8.9344	5.6519	10.1621	7.9942	8.2917	1.7113	12.9676	11.1483	6.3328	7.3032	11.194	11.0557	8.8821	10.6628	6.9697	11.5501	4.5227	8.0071	7.7003	10.973	6.9072
TCGA-05-4397-01	731	1	6.5645	8.155	8.8708	10.6255	10.703	10.922	1.4683	9.5588	8.6013	9.2841	8.7925	8.2088	5.1116	6.3931	10.0912	7.5296	7.6106	8.0298	10.9004	1.682	8.6742	6.5442	8.4161	4.8384	9.9958	10.0955	8.1165	2.1808	9.2235	13.0894	5.3951	7.4072	12.756	12.7336	9.3239	10.6832	7.6955	11.9919	2.8886	9.8414	9.0919	8.773	11.882
TCGA-05-4398-01	1431	0	4.2185	9.2996	5.0991	10.8827	9.6529	11.3002	1.3348	8.3471	9.9319	9.3248	8.2763	8.4497	6.6115	7.31	9.3145	8.2692	6.5441	7.4337	11.6476	2.2971	4.7368	4.1317	8.7695	1.6667	10.2614	9.7039	9.9259	3.6718	12.4763	13.222	6.9579	6.9903	10.901	13.6942	9.5703	10.5211	4.1137	9.6078	6.5357	8.4737	7.9457	10.3768	7.0765
TCGA-05-4402-01	244	1	6.4169	8.2401	5.1789	10.7462	10.925	10.1539	3.5677	8.6162	8.9196	8.7195	7.981	7.2437	5.6292	5.2169	9.7658	8.3646	7.293	6.5788	11.5699	0.568	7.3563	2.8626	8.6545	6.1143	11.6384	9.5492	8.2412	6.2255	11.9347	12.317	7.7691	7.3349	11.892	11.9835	9.2611	10.619	6.32	9.9406	3.3114	8.2742	7.4268	8.4655	9.9663
TCGA-05-4403-01	578	0	1.9184	8.4959	7.6955	11.4642	10.376	10.4583	1.257	8.0469	8.2214	9.1946	10.6115	7.2343	5.1373	10.1569	9.3557	8.9069	6.4989	6.5324	11.3624	1.4158	7.9647	2.3702	13.3937	1.9184	11.1494	9.9624	8.1708	5.2886	13.1363	12.292	7.2047	8.6497	11.318	12.9417	9.4619	8.7858	5.9054	10.0674	4.315	9.3241	8.5988	8.3314	9.8974
TCGA-05-4405-01	610	0	7.0823	8.6096	0.7334	9.5478	9.2177	10.6081	0.7334	7.8157	6.9137	8.6218	7.8889	7.8472	6.3193	7.0681	10.8333	8.0981	6.8782	5.4144	10.8186	1.5791	6.6109	3.0512	8.6625	4.8823	11.0409	9.5529	8.6291	5.9063	12.0469	11.8054	7.7748	8.0946	11.087	12.3094	9.3139	10.2654	6.0285	11.3286	4.384	7.8513	6.4991	8.8951	9.2882
TCGA-05-4410-01	0	0	6.5352	8.487	6.7856	10.8726	10.242	10.0714	1.7197	7.9748	9.2676	8.5054	7.3221	8.2322	6.8823	8.8082	10.0424	8.4306	5.7869	6.3343	11.5095	2.0208	6.2894	5.6418	8.7324	5.574	10.8959	8.5937	8.7808	2.6671	13.0481	11.8525	6.4744	6.141	11.538	12.5281	9.1388	10.4146	4.5811	11.3317	3.731	8.5054	6.3447	8.8895	8.9103
TCGA-05-4415-01	91	1	9.0202	8.1137	2.0395	12.7024	9.1111	9.5368	5.7279	7.2841	7.517	9.6018	8.2921	7.8614	6.7385	4.3299	9.1065	7.2987	6.5313	5.7533	11.5405	2.663	5.3731	4.9015	9.3141	7.0407	10.3508	6.5183	8.8148	1.4739	10.9569	12.8439	6.8813	5.506	9.919	13.4065	8.6427	11.5648	6.7915	10.151	2.7608	7.0792	8.4023	7.8587	8.0056
TCGA-05-4417-01	455	0	8.6021	7.9844	8.7001	9.8718	9.4641	10.0927	1.8244	8.5537	8.847	8.8625	8.9223	7.8075	6.0262	8.8347	11.4851	7.9662	7.1745	6.569	11.2562	0	7.5676	3.9048	8.559	6.2231	10.1414	8.3724	7.4362	3.211	12.0658	11.8679	6.2353	7.2072	11.586	12.9384	8.8743	10.5185	5.7307	11.1637	6.1732	9.1263	7.1759	9.5619	8.6667
TCGA-05-4418-01	274	1	10.401	9.2965	10.0416	9.7897	10.034	10.037	5.9345	8.0749	9.735	9.636	8.7485	7.9107	5.8785	8.381	8.8916	8.3563	7.5161	6.8659	11.7375	2.3186	9.0375	3.0552	9.9802	5.6397	11.1257	6.4498	8.6264	1.4124	12.0713	13.2791	7.5682	8.0565	11.067	13.7707	8.7574	11.6849	6.4052	9.8192	3.3181	8.8486	8.9864	8.8805	7.3853
TCGA-05-4420-01	912	0	9.4943	8.2236	4.2785	10.7541	9.589	9.6259	0.3369	8.4595	6.8711	8.4947	8.5795	7.7919	6.4686	4.6847	8.4639	8.5187	7.8283	5.0856	9.3076	0	6.3103	2.9702	9.1354	0.8391	10.7832	8.6444	9.2937	3.0179	11.3127	14.2313	7.598	6.783	13.18	13.6018	10.0533	10.4399	4.1129	10.6785	8.4216	7.4278	7.759	9.7044	7.773
TCGA-05-4422-01	365	0	6.3941	10.246	11.9022	8.0594	10.197	10.4582	1.1491	8.0417	9.5766	9.5059	7.5771	8.4266	6.8888	3.0003	9.5475	9.349	7.6229	5.7952	11.4375	2.4767	9.4555	10.0151	8.839	5.1944	9.8297	8.3275	8.2971	1.9029	12.2537	13.006	7.6856	6.855	11.017	12.9453	9.2857	11.4104	6.3571	11.2195	2.5535	9.3177	7.6727	8.8128	8.9237
TCGA-05-4424-01	913	0	4.4886	8.1528	5.1227	9.9672	9.8838	10.0734	1.2809	7.288	8.0734	8.2143	8.1333	6.3497	6.4475	2.6423	10.206	7.2534	7.021	7.5717	11.5599	1.2809	9.3874	2.7484	8.4665	4.8875	10.5986	10.7706	8.2621	3.7434	11.2706	11.6841	6.2894	8.081	11.588	12.8287	8.8882	11.3454	4.7149	11.5679	7.9731	8.941	7.0083	8.3469	10.4319
TCGA-05-4425-01	669	0	2.2978	8.7128	9.4762	9.5864	9.272	11.0857	0.6412	8.1815	10.4956	9.7004	6.0788	7.4422	6.3402	4.6149	9.5716	8.4264	8.2849	6.4218	11.7556	1.4216	8.2035	6.5615	10.1735	7.1154	9.3946	8.6153	8.5778	2.9477	12.5807	12.7181	5.941	6.2688	10.867	13.2687	9.3409	10.5595	7.0191	10.7436	4.0098	8.762	7.6292	9.466	7.3951
TCGA-05-4426-01	791	0	6.3084	9.2501	5.0833	11.7721	10.345	11.3357	1.4743	8.9823	6.6031	8.2906	9.0977	7.6171	6.596	5.0319	8.9395	9.4233	6.6268	5.7423	10.8561	0.6716	8.0325	1.6205	9.4013	5.2042	10.7303	8.8835	8.3349	3.2171	12.2744	13.3348	7.872	7.2607	11.466	12.6927	10.0431	12.1176	6.9577	10.9527	3.9331	7.7542	8.8253	9.7537	10.6047
TCGA-05-4427-01	791	0	2.0098	8.9526	1.5937	9.8718	10.174	9.3684	0	7.6043	7.6501	8.71	8.5483	8.8972	6.3527	10.9596	10.1294	8.1267	6.0924	6.218	11.2219	2.1801	5.833	2.5958	9.3111	2.0098	11.1701	10.2035	9.249	2.8185	11.998	11.3591	8.0073	6.3966	10.826	12.4106	7.7341	8.4181	3.099	10.5905	3.9664	7.9903	7.2561	7.9289	8.5424
TCGA-05-4430-01	761	0	5.0813	8.0487	3.3576	8.8008	9.7108	10.7119	2.9446	8.5822	8.338	8.4497	8.8986	7.6442	6.4555	9.5377	9.4464	7.8133	7.1066	6.5837	11.4855	1.3757	6.3691	3.1658	9.2235	2.6097	10.9318	9.1638	8.1183	3.8154	11.5024	12.7962	6.2532	8.5526	11.378	13.4849	10.0246	11.5398	5.248	11.5172	5.719	8.5241	8.2526	7.8929	9.9837
TCGA-05-4432-01	761	0	7.429	7.7714	7.4553	8.5884	9.6813	10.2896	0.6571	8.6033	6.9902	8.1348	8.0128	7.715	6.3068	5.4587	9.2172	7.8402	8.4049	6.6862	11.4326	0.3656	7.5975	3.6482	8.5819	3.5089	10.6211	8.7565	8.2126	2.2478	9.9534	12.1559	8.4899	7.5693	11.89	13.0569	9.1352	10.377	5.0197	10.4326	4.6734	8.8288	8.1885	9.4557	8.9624
TCGA-05-4433-01	730	0	5.0899	8.6445	8.3875	12.1088	10.279	10.4411	0	7.8549	8.232	9.2012	9.9601	7.5077	5.8586	8.9663	9.9027	8.4122	5.549	6.575	11.7394	2.7624	8.4157	0	12.408	2.3596	10.9825	9.9151	7.9974	5.8169	12.4421	11.0568	6.4283	6.8461	10.707	12.258	8.4705	9.6064	5.919	10.5073	5.0899	9.7532	7.3918	8.8753	9.8481
TCGA-05-4434-01	457	1	4.2545	8.8211	9.0345	11.6572	10.246	11.0631	2.523	8.6056	9.9955	9.6402	8.585	8.004	6.017	6.1331	9.6569	8.7719	6.6656	7.5258	12.443	2.3419	7.5334	3.3282	9.9584	4.9256	10.6468	9.1934	8.2994	3.1189	11.8559	11.7701	7.9745	7.5988	10.723	12.9095	9.3679	10.0261	5.4654	9.7065	4.4905	9.8133	8.5028	8.1638	9.1331
TCGA-05-5420-01	457	0	11.414	9.1531	9.8088	9.8303	10.401	11.251	1.8817	7.2343	9.6524	10.1336	8.3323	6.9393	7.2672	3.1787	7.7049	7.9174	7.6194	9.7276	12.6352	1.4803	6.9073	3.9374	8.7502	3.0286	10.0773	8.0055	9.2581	2.4529	9.7228	13.0971	7.1172	8.5199	12.349	13.5696	9.4732	11.452	5.82	9.7679	2.1954	10.8673	10.4185	8.0646	8.2583
TCGA-05-5423-01	151	0	3.3956	9.1775	6.653	10.1108	10.709	10.0272	1.9747	8.4954	8.6617	9.6536	7.6029	7.4781	6.2145	8.1412	9.5318	8.3977	6.6193	8.8615	12.0774	0	8.3369	2.9247	8.207	6.9031	10.4953	9.0377	9.0968	4.5675	11.7472	12.6247	7.6615	8.4681	12.217	12.5524	10.762	11.4166	6.3241	10.6696	1.3728	9.7202	9.3909	8.3105	9.0377
TCGA-05-5425-01	882	0	9.2835	8.5952	10.1151	9.92	9.7692	11.0535	1.0917	8.4849	8.247	8.939	8.7088	7.3315	7.0468	5.7743	9.2703	7.6688	6.8732	8.0173	11.6662	1.0917	6.4331	7.3488	9.9907	3.622	10.6671	9.0586	8.6852	2.9611	11.8509	11.8426	6.141	6.2197	13.058	13.4397	9.5913	11.0665	5.6498	11.7389	3.483	10.1392	8.5868	9.0104	9.5774
TCGA-05-5428-01	670	0	6.5593	9.6387	6.9435	10.1098	10.216	10.4978	2.1661	8.7681	8.3987	9.6544	9.4804	7.3582	7.0423	3.9841	9.0147	8.4647	9.2098	7.4503	11.0415	1.8544	4.7635	2.9957	9.1157	2.6397	10.686	8.581	11.154	2.6397	10.3094	14.6661	8.3691	9.5576	12.041	14.3602	10.1831	10.8747	3.9023	10.4419	3.4049	8.1997	9.656	9.2015	9.0649
TCGA-05-5429-01	275	1	2.283	8.8826	10.4182	11.0837	9.6113	9.8882	1.3487	7.9505	5.4743	9.378	10.8371	7.6616	6.4145	5.8637	8.3885	7.916	5.6146	3.9377	10.8432	2.0334	8.7874	2.9929	12.5654	3.4665	10.0046	10.3472	8.6301	4.597	12.7274	13.6298	6.3747	8.6799	11.229	13.414	9.1534	9.9549	5.6581	9.6931	0	6.8988	8.7721	8.6103	8.4279
TCGA-05-5715-01	62	0	3.5767	7.0205	7.5395	11.775	10.25	10.4745	1.357	8.4244	8.9176	8.4416	8.9879	6.5533	4.4128	5.2062	9.7918	9.1173	6.9552	7.1723	11.2305	2.0438	7.2376	3.2613	8.5326	8.1158	11.0636	10.0238	8.4545	3.9121	12.0108	12.9948	8.0788	8.3719	12.601	13.1374	9.8984	10.4997	7.5364	10.6923	3.0049	9.3318	6.8356	8.8869	8.3753
TCGA-35-3615-01	14	0	6.6072	9.9858	12.0106	7.9017	10.297	10.724	0.4059	9.1465	6.1911	8.729	7.652	8.0646	5.237	7.3922	8.925	9.0828	7.2447	6.244	10.9872	0.9816	7.5229	3.995	8.3453	6.1729	10.6369	8.4853	8.6331	1.5604	12.7006	12.0676	6.5455	7.8559	11.317	13.8419	9.4996	12.0695	6.8213	8.66	3.904	8.4536	8.5985	9.366	6.4922
TCGA-35-4122-01	225	0	3.7071	8.9973	7.4081	10.4794	10.649	10.8413	0.5874	9.1279	9.3521	10.1834	9.2215	6.236	6.8775	9.7837	7.6034	7.2069	7.8946	8.6608	12.075	1.5898	4.9612	6.408	9.1326	3.0063	10.632	9.5425	10.025	0.5874	12.0613	13.1594	7.4666	8.5637	12.733	14.5341	8.7094	10.6876	5.7075	9.7704	3.961	10.0578	8.9672	7.4584	7.0072
TCGA-35-4123-01	182	0	2.4306	8.3697	5.8709	10.9877	10.86	10.0259	0.9094	8.8548	9.0373	10.0994	8.7003	7.9401	6.1098	8.964	7.8496	7.9114	8.3355	7.4126	12.0964	2.0264	4.8631	4.2482	9.1727	3.8251	11.4849	9.6638	9.3951	2.5435	12.1737	13.4531	8.448	7.2275	11.416	14.1816	10.6084	11.2141	3.8691	9.9581	3.8691	8.7079	8.9792	7.5415	9.2234
TCGA-35-5375-01	264	0	1.0772	7.9311	12.149	9.5033	10.021	9.65	1.0772	8.1424	8.6212	10.0175	7.7644	6.1023	5.5393	4.5359	7.6965	7.8432	8.7693	6.5924	10.6331	0	6.8633	2.4435	8.5594	2.4435	9.8402	9.2054	9.7501	5.6066	9.178	14.385	6.3183	9.658	12.326	14.4521	9.4216	12.2693	7.5504	10.4846	10.1878	8.7794	9.9381	3.8398	12.2543
TCGA-38-4625-01	2973	0	3.0072	8.8332	6.8355	10.6635	9.347	10.3993	2.461	8.2156	8.6062	9.1293	8.2732	7.5845	6.3741	5.5254	8.9487	7.2999	7.077	7.6524	11.9641	1.0884	5.8621	2.5329	10.3166	1.8215	10.2013	8.9388	9.2963	2.3053	11.8736	13.1216	6.592	6.7995	12.281	13.4983	10.0104	10.1396	3.7125	8.7897	3.1964	8.6702	9.1044	7.8434	8.8924
TCGA-38-4626-01	3674	0	5.3749	8.6716	5.2603	11.8503	9.9255	14.0362	2.7828	8.3985	8.3323	9.1141	8.7958	7.9077	6.3211	3.8427	10.2809	9.3604	6.5836	8.0744	12.1147	2.0591	7.3842	3.1567	8.2731	4.2382	10.6883	8.8541	7.9998	4.7436	13.052	11.3433	7.2997	6.9707	11.202	12.248	8.8964	10.0452	5.875	10.6058	3.8653	11.4901	6.9123	8.6907	9.1982
TCGA-38-4627-01	1147	1	6.5438	8.3425	6.2918	9.6673	9.7631	11.6252	2.3257	8.492	9.1519	8.8993	8.7798	7.0118	6.0351	7.8756	9.9326	7.2825	6.6994	7.6786	11.4298	0.4487	7.2791	6.1056	9.0299	5.5759	10.2577	8.6742	7.4333	1.9703	11.9181	12.9758	7.0521	7.2097	11.586	13.1908	10.1088	10.8283	6.3954	10.3765	5.6829	9.2281	8.1742	7.7146	9.0617
TCGA-38-4628-01	1492	1	1.9428	8.5923	2.2608	8.8959	10.979	10.0231	0.9621	9.1686	7.6023	9.7112	9.2602	6.9425	5.3059	5.9905	8.9243	7.577	7.5317	7.2762	10.6873	0.3963	8.5564	3.8031	7.4367	4.4079	12.1561	10.0226	8.8343	4.7334	12.027	13.2603	7.8444	9.0916	12.772	13.7202	10.8628	11.0551	6.9087	10.7836	3.1019	8.7091	7.1621	9.3593	9.4058
TCGA-38-4629-01	864	1	0.9312	8.8404	4.9925	12.1203	10.324	11.1303	0.6822	9.2588	10.3742	9.4347	8.3486	7.6946	6.2184	9.1127	9.02	8.1541	7.0879	9.6982	12.2794	1.4925	6.2416	3.7757	9.4665	4.7708	10.4054	8.8911	8.6494	1.8956	11.5686	13.7197	8.8241	7.3912	11.274	14.2643	9.6622	11.549	4.8024	9.2693	5.3327	9.3517	9.4061	8.151	9.3551
TCGA-38-4630-01	1073	1	3.3021	9.4659	6.5488	11.872	10.056	8.9536	4.227	7.4118	9.6614	8.3972	7.0939	8.2123	6.4498	3.0552	9.6594	8.5432	8.3987	4.899	11.4258	1.22	4.614	3.4262	8.951	2.7573	10.4194	8.7332	7.9416	1.3511	8.1507	12.9721	6.0034	6.8826	11.891	13.3754	6.3357	10.1244	7.8518	9.603	4.0258	7.2966	7.3087	8.9999	7.8039
TCGA-38-4631-01	354	1	5.3857	8.1944	12.8239	10.0558	10.089	9.9395	3.5834	8.1377	9.0251	10.0038	9.4677	5.6109	5.9222	6.7927	7.0103	7.4594	7.5247	5.9306	12.3804	0	6.4612	1.746	9.4868	5.1825	11.2079	8.4095	10.198	3.8421	9.4272	14.3772	8.6707	7.51	13.787	13.6729	9.7889	11.7877	7.2298	10.1829	5.6109	7.8708	8.8134	5.8651	8.0819
TCGA-38-4632-01	1357	1	3.4516	9.17	1.7198	10.5431	10.014	11.7101	2.2701	8.411	7.8656	10.511	8.94	7.5572	6.6051	11.2596	9.6468	9.6555	8.1388	7.4307	11.5113	0.8194	6.061	5.6421	8.7558	2.2701	10.7366	9.0482	9.4991	2.021	12.2977	13.7948	8.118	7.7178	11.855	14.307	10.2705	11.3878	4.5814	10.959	3.884	8.828	8.2722	9.4729	8.7091
TCGA-38-6178-01	448	0	4.9002	7.4248	5.404	11.5125	9.6476	8.991	0	8.1303	7.8265	8.6536	8.094	8.0606	6.8635	5.3498	9.8887	9.7738	5.7215	5.6439	11.4935	1.3562	7.3646	2.2928	8.1104	6.7107	12.1667	9.8635	8.4942	3.9108	12.2601	11.7003	6.9016	7.6655	11.502	11.9666	9.5201	10.8805	6.2895	10.0776	4.0532	7.3714	7.2362	9.9706	8.5849
TCGA-38-7271-01	800	1	2.262	7.9396	4.0828	10.2388	9.5071	10.7277	2.6588	8.5971	9.1344	8.4461	8.4555	7.5875	5.7589	5.8184	10.8491	8.0594	7.1032	7.0975	11.7671	1.7128	6.4396	3.5398	8.7584	6.3076	10.7514	8.9477	7.6866	4.0828	11.605	11.6855	5.8755	7.0971	10.869	12.4815	9.0107	9.793	6.6722	10.4802	2.6588	9.1072	7.553	7.6705	9.4361
TCGA-38-A44F-01	133	0	5.6949	9.2296	8.572	10.641	10.113	10.2069	3.2573	8.906	8.5927	9.2755	9.8947	7.8811	3.6989	6.8185	9.5317	9.7338	7.2277	7.2532	12.1711	1.7151	7.7825	3.0738	11.5106	5.9426	11.1838	9.6022	7.3344	5.7263	12.6773	12.5484	6.6728	8.1503	10.55	12.6348	9.6995	10.0284	6.0949	9.6624	5.3357	9.1736	8.4919	8.0881	8.7432
TCGA-44-2655-01	1324	0	7.2793	9.428	8.0265	8.5405	10.826	10.1528	7.3725	9.0965	6.6972	8.9546	8.2807	7.3497	5.1992	11.2739	10.4152	9.2043	6.6294	6.5216	11.4506	0.8294	9.5481	2.7436	9.282	7.0921	11.2962	8.5782	8.4366	2.1265	11.4617	11.9547	7.1139	8.6101	11.243	12.7511	9.5265	10.4823	6.5581	9.7568	3.5382	9.1768	7.578	8.584	8.4322
TCGA-44-2656-01	1429	0	3.3135	8.6609	10.098	13.3719	10.625	10.5809	1.993	8.3769	9.6797	9.0774	8.1298	7.745	6.4591	10.6303	9.8709	8.2282	5.9364	7.77	12.0177	0.6247	7.3586	2.4156	8.2425	2.4156	10.7821	8.8783	8.6829	2.6824	13.4658	11.3881	7.7174	8.0376	11.138	13.1059	8.8624	10.6762	4.5203	10.4532	3.0559	9.5491	8.3313	8.9646	7.9068
TCGA-44-2657-01	1351	0	2.947	8.8974	6.118	10.4377	10.264	10.3528	2.3296	9.0885	9.1729	9.0218	8.7522	7.7712	5.3433	3.7093	9.995	9.0568	7.4832	7.6996	11.7515	0.7408	7.9423	3.378	8.7477	7.6746	11.2542	9.5048	8.2654	3.1785	11.5525	12.0758	7.0503	7.3642	11.653	11.7352	9.6637	10.1282	5.8921	10.5709	2.1229	9.3532	8.2717	8.6547	8.9055
TCGA-44-2659-01	1367	0	2.8502	8.7932	1.2064	8.5393	9.9752	9.4087	0.986	7.7696	8.0796	8.5682	7.8567	7.3955	5.2332	8.4246	10.4889	8.629	6.2569	6.4262	10.9152	0.986	8.3571	3.2479	8.1013	6.3121	11.0543	9.4544	8.7402	2.4794	13.3299	10.677	6.6587	7.9701	12.395	12.1693	8.4692	10.0536	5.7883	10.985	4.089	8.1798	7.2819	7.4095	9.0813
TCGA-44-2661-01	1159	0	6.7627	9.2089	7.3644	10.1564	10.302	11.5675	1.9567	9.4446	8.6155	9.1033	9.0663	7.6147	6.5674	3.2699	9.343	8.7398	7.4522	8.3286	11.7185	1.9567	7.5862	3.647	8.717	5.2289	10.52	9.2136	8.1246	3.1962	12.5502	12.159	7.9109	7.9394	12.095	13.2186	9.7852	11.1664	6.6773	10.7498	1.9567	10.2234	8.8355	7.9023	9.074
TCGA-44-2662-01	1280	0	7.4526	8.4275	1.4831	12.649	9.9104	11.1995	0.7809	7.4943	8.4456	8.4048	8.5528	7.6173	6.5269	9.7809	8.3181	8.1945	5.9495	7.6682	11.4835	1.4831	3.6837	1.0547	8.8787	0.4427	10.6348	9.5074	9.2905	4.4242	12.1703	11.3154	6.2759	6.0677	12.045	12.7506	9.8284	11.1191	3.419	10.305	3.0325	9.1379	7.8394	7.6356	10.2743
TCGA-44-2665-01	1301	0	1.7536	9.0651	7.8162	9.8762	9.363	11.3797	0.5601	8.1971	7.9813	9.3939	8.4282	7.9797	6.115	9.2326	9.4468	8.4479	5.2237	7.6528	10.9726	2.1113	7.0068	4.9039	9.4151	5.3353	9.8363	8.9453	8.528	3.1027	10.6919	12.2756	7.8581	7.9688	10.945	12.709	9.9171	11.6717	6.2826	10.3974	2.3976	8.6056	9.2774	7.8162	9.3004
TCGA-44-2666-01	97	1	4.0013	9.0391	3.8426	11.4408	9.3377	8.9137	1.1707	8.4295	5.3081	8.6741	9.0629	8.1164	5.9122	6.9301	9.7775	9.511	7.7819	4.5019	11.4631	0.8751	6.4689	6.5482	10.6824	7.9919	9.8858	8.7032	8.8421	2.1165	13.3455	11.6212	7.7701	6.8065	10.747	13.1512	9.2346	10.7518	7.0575	11.0175	3.1577	7.5144	6.2334	9.3208	9.3274
TCGA-44-2668-01	761	1	4.4058	7.5751	6.1472	11.0852	9.0937	10.3554	1.5413	7.6148	9.6321	9.1384	8.3789	7.0944	6.5176	7.2705	9.5156	7.3157	5.6332	7.4585	12.4819	1.6707	5.6554	3.4723	9.4518	3.6709	10.3354	9.4234	8.0833	5.1891	12.4266	12.1585	8.1977	7.3809	10.529	12.9242	8.8509	10.5829	6.5158	10.6891	6.0789	8.2478	7.8248	8.8855	9.7726
TCGA-44-3396-01	1130	0	6.8966	8.9118	5.6885	10.1542	9.8982	10.1442	0.9223	7.6431	9.4352	9.4769	8.3699	8.0068	5.5158	7.6784	9.1751	8.1329	5.7719	7.9137	11.6335	2.0471	5.2364	3.9583	9.4149	2.2642	11.5903	9.0722	9.3165	5.9353	10.3696	12.7591	6.5141	8.0464	11.254	13.0711	9.0431	10.6597	4.7881	10.1522	6.182	9.0942	7.6482	7.4352	6.9528
TCGA-44-3398-01	1163	0	8.4899	8.7356	9.2965	10.9271	10.156	11.6451	1.1921	7.9887	8.1609	9.9672	9.2857	7.6418	5.4396	6.7785	8.7657	8.9293	7.2034	7.9045	11.0484	2.8065	6.9076	3.9196	8.9102	5.9093	9.4008	9.075	9.199	1.9992	11.1861	13.2319	7.7831	8.3808	11.364	13.6806	9.4511	10.8542	6.4156	10.4858	3.2578	9.2985	8.7486	6.349	8.7515
TCGA-44-3918-01	1036	0	4.9244	8.5918	9.6189	9.4732	9.8112	10.8868	2.0183	7.8876	10.2224	9.7915	9.7313	8.4321	5.9636	5.9005	9.4871	8.238	7.0689	8.0189	11.7762	1.3366	4.6888	4.3807	8.8449	4.9778	10.3595	9.4356	8.9991	2.1481	10.5587	13.2891	7.363	8.1709	11.894	13.282	9.232	10.8512	4.8501	9.9672	4.4579	9.9026	8.795	8.4776	8.0327
TCGA-44-3919-01	1026	1	5.0947	8.5631	7.475	10.5333	9.9997	10.3065	0.7228	8.0257	9.5724	9.1828	8.7984	8.0869	5.5501	6.2502	9.9431	8.6878	7.433	6.3306	11.7732	1.3929	4.8564	2.7068	8.6298	6.9944	10.8944	9.089	8.256	2.1945	11.8242	12.6099	7.7107	6.9111	11.469	12.7327	8.6867	9.7605	6.5397	11.1172	3.6676	8.7632	7.0271	8.0324	10.0738
TCGA-44-4112-01	808	1	5.3203	8.3747	5.349	9.9851	9.6377	10.2596	1.1406	8.4302	6.9785	8.9324	9.0562	7.9978	5.9921	7.4236	9.2953	8.6661	7.0228	5.8594	11.2792	1.3821	4.5038	1.9302	8.6831	7.061	10.4918	10.0262	8.8706	2.8123	12.132	12.2781	7.5588	8.6732	11.825	13.3802	10.1348	11.5037	6.5511	10.7857	5.1029	7.9018	7.6366	8.5779	8.718
TCGA-44-5643-01	1013	0	1.9324	7.8857	0	11.0601	8.8371	9.0981	0	7.791	7.8452	9.1217	10.5962	8.6495	6.6782	6.1583	9.7246	10.6468	6.8387	4.96	10.9815	0.6447	7.0897	2.3056	10.145	8.9499	9.8541	9.0437	8.5467	3.4029	8.0388	12.0528	4.921	4.4115	10.5	13.6067	7.4143	9.0817	8.18	10.8996	6.2466	9.7014	9.1859	8.92	10.2845
TCGA-44-5644-01	863	0	10.636	8.3787	9.8043	10.0394	8.8943	8.9879	1.8718	11.379	7.5857	9.6847	8.6172	6.6641	5.5511	8.7457	9.7706	9.7989	7.2569	7.7373	9.8203	0.7355	4.0264	8.4015	9.8075	3.9663	10.4195	7.533	7.9945	2.319	12.6857	12.2751	6.616	5.9113	11.575	12.1616	8.9936	9.2933	1.8718	10.5697	2.8043	5.8293	7.431	9.3923	8.936
TCGA-44-5645-01	852	0	5.7021	9.0134	2.3266	9.599	10.136	8.8544	0	9.1949	9.0352	8.3322	7.8268	8.1045	6.0656	1.1026	10.5668	9.6086	6.1251	6.6945	11.4427	0.6542	9.0776	4.0436	8.6635	7.5147	10.8721	9.0959	8.1977	3.6358	12.3871	10.5941	6.0153	7.2026	10.314	11.373	9.1667	9.695	6.2725	10.9817	1.7202	8.4993	7.0297	7.5101	8.8949
TCGA-44-6145-01	595	0	8.7606	8.1752	10.1989	9.5328	9.6944	10.6265	2.9907	7.9111	9.2304	8.3048	7.7192	7.7951	6.523	7.5545	9.6486	7.7619	6.4439	7.0232	11.2196	2.741	5.7734	3.389	9.1537	5.7899	10.5275	9.179	8.7158	3.5532	11.8764	11.568	5.2818	6.4623	10.671	12.6387	9.6083	10.8012	6.018	9.1632	3.9569	9.0266	8.0706	8.413	7.4799
TCGA-44-6146-01	728	0	5.085	8.5803	10.1193	10.5504	10.589	8.2188	1.8905	7.7674	4.7751	9.3481	10.4714	7.1431	5.3536	8.9595	9.4596	9.0197	5.4583	1.402	10.0942	3.3891	9.4494	3.0392	12.2329	2.3395	11.011	10.1508	8.4542	7.6982	12.8012	10.8514	5.8545	7.7107	9.2032	12.4034	8.5494	9.3371	4.1817	10.5915	4.7154	6.2115	6.9978	9.1538	10.9577
TCGA-44-6147-01	845	0	3.7622	8.6747	4.708	9.979	9.7773	8.8524	4.1205	8.1519	7.7602	9.1237	8.1389	8.6237	6.6891	4.0268	10.2405	8.6805	6.5068	4.838	11.3281	1.6717	8.7681	4.0268	8.7554	4.4797	10.751	9.7826	8.0877	4.7671	12.3293	11.8075	6.5446	8.2072	11.174	12.1474	9.4943	9.9806	6.3553	10.4572	4.4797	7.3705	7.6936	10.0779	9.0616
TCGA-44-6148-01	704	0	2.9935	9.0808	6.4292	9.497	10.002	8.648	3.2587	8.468	6.4954	8.3636	8.2741	8.2232	6.217	4.8238	10.1795	9.4269	6.406	5.0183	10.6017	0.6189	7.8672	2.0752	9.4358	6.7567	10.9196	8.5317	9.7383	3.8999	11.8216	11.6802	7.6726	7.1968	10.629	12.2334	9.3353	9.2953	7.0587	10.3556	2.5413	7.546	7.6459	7.9096	8.7592
TCGA-44-6774-01	658	0	1.9215	8.0993	4.2217	10.1232	9.4851	9.6527	0.9482	7.6344	8.4754	8.4871	8.5985	7.4341	6.0456	4.7561	10.4032	7.734	6.8437	5.9969	11.4821	0	7.8777	3.7095	8.2434	2.908	10.7839	9.104	8.0944	3.9012	9.9429	12.3264	6.1625	7.1712	11.4	12.8662	9.9846	9.8874	5.3231	10.5747	5.6512	8.1283	7.2787	7.663	9.0341
TCGA-44-6775-01	705	0	6.3355	8.6594	3.2603	10.3061	9.9599	10.0159	0.5896	8.0816	9.1443	8.0389	8.6043	8.0333	6.223	7.0137	9.6809	8.4039	7.1064	7.3061	11.5599	2.333	6.473	4.8959	8.6504	7.0744	10.9727	9.1597	7.9967	4.9202	12.7565	11.2812	6.5759	7.4975	11.234	12.003	8.6266	9.3402	6.0359	11.768	3.5361	9.054	6.7484	8.8211	10.8604
TCGA-44-6776-01	2616	0	6.2287	8.044	11.78	10.3505	10.241	9.2118	0	9.0105	4.9367	8.5324	7.7439	8.0866	4.9622	6.0681	10.0151	9.482	7.1219	5.3714	10.7657	1.285	8.279	1.285	9.6995	6.3732	10.9845	7.5639	8.0163	1.6577	12.3433	11.9018	6.0525	7.9082	11.634	12.5197	9.3907	10.9019	5.7399	10.8257	4.4002	7.45	6.5715	10.3671	7.0804
TCGA-44-6777-01	987	1	4.5937	8.0037	7.8738	13.2682	9.8104	11.1346	1.3663	8.1483	11.0348	8.9264	8.2176	7.1602	7.0034	8.4888	10.3487	7.0895	7.2308	7.3985	11.7825	1.3663	6.5236	3.7682	8.5139	6.2621	10.0237	8.906	8.1763	3.8228	12.1739	12.5554	7.7131	6.4474	11.064	12.9729	8.7718	10.7152	5.7157	12.4424	5.7861	9.9037	7.9948	8.1337	9.9634
TCGA-44-6778-01	1864	0	5.0601	8.3227	4.5366	11.1237	9.2134	11.534	1.823	8.3226	9.649	9.5156	8.3025	8.3649	6.7238	1.823	10.2458	8.5978	7.0326	8.4084	12.1827	2.444	5.8606	2.6031	8.5201	3.3948	8.8825	8.3284	8.1942	3.2093	11.4211	10.9856	5.0872	6.5971	10.219	11.9313	8.3897	9.1358	6.3161	10.639	5.1139	10.6173	7.7556	7.556	10.1386
TCGA-44-6779-01	500	1	8.0574	7.2744	5.9442	10.1148	9.3223	10.037	0	6.8889	9.0118	7.8438	7.0585	6.8845	8.0831	6.9787	9.1569	7.1341	5.8651	7.0076	12.6153	1.3751	5.981	0.8456	9.5043	7.9026	10.0854	9.617	7.5568	2.0662	11.2012	11.901	5.5119	8.0873	10.609	12.1905	9.0676	9.8466	3.401	8.2697	3.0308	9.1789	6.9039	8.1655	9.6966
TCGA-44-7659-01	691	0	4.914	9.5548	5.6745	8.4727	10.152	10.7786	1.697	8.5148	6.8792	8.9227	7.1674	8.5496	4.9493	7.2965	10.7114	9.9769	7.1738	6.038	11.4275	1.3189	8.1848	2.2439	8.6997	6.3916	10.7362	8.2535	7.9737	0	14.0288	11.0027	6.2358	7.3765	10.467	12.521	9.327	11.1027	6.2286	10.9557	2.4553	9.6625	6.628	8.5639	6.5949
TCGA-44-7660-01	592	0	8.0799	8.1627	6.3752	10.8984	8.5142	11.228	1.4235	9.0575	8.989	8.4217	7.4135	7.6494	6.7298	4.2858	9.432	8.9724	6.6866	6.5364	9.7827	2.8696	6.9421	2.4922	8.9419	9.5443	10.4146	9.5677	8.4146	5.4282	9.0328	13.592	5.5881	7.0559	10.487	13.5356	8.5584	10.5515	7.8729	10.5232	4.6663	8.7037	7.4417	7.7458	10.4975
TCGA-44-7661-01	557	1	2.0437	8.5845	0.9537	11.9205	9.3676	10.5708	2.0437	7.7657	9.2826	8.9587	8.4824	8.2327	6.0435	9.969	9.9482	8.0696	7.2195	7.1975	11.9973	1.8067	5.6448	3.3301	10.0107	4.9821	10.2189	9.0803	8.9183	2.8572	11.9828	12.8192	7.703	6.9475	10.238	12.9916	8.4376	10.2079	4.3915	10.1603	5.7573	8.8604	8.4324	6.9511	9.6221
TCGA-44-7662-01	218	0	4.1354	8.6691	1.7518	10.914	9.3614	9.7848	0	8.3076	8.7607	8.3784	8.3061	8.1792	5.5232	8.8558	8.8604	8.1404	6.7549	6.9987	11.7428	1.4729	5.6218	3.9266	9.1597	2.7906	11.0763	9.4774	7.994	3.1685	11.0274	12.8336	7.5459	8.4089	11.471	12.5623	9.9143	11.0018	4.5542	10.3372	4.5723	7.795	8.2398	8.0173	10.1016
TCGA-44-7667-01	1097	0	3.4579	8.0946	0	10.4652	9.8875	9.3142	0.7363	7.4825	8.5816	9.6007	8.2898	8.0409	6.1682	4.5427	12.2672	6.5219	10.0389	4.8847	10.8774	0.7363	6.3021	2.1142	8.8301	2.221	11.8524	7.3906	9.0436	0.7363	9.7932	12.8095	8.5387	6.1749	10.869	13.8747	8.4968	9.8987	3.8729	11.2341	4.4134	8.0698	6.9251	9.7211	9.5876
TCGA-44-7669-01	574	1	5.4872	8.9326	7.9496	11.5019	9.4705	10.4995	3.3506	7.7083	8.7611	9.463	8.0994	8.0164	6.7011	5.7316	9.9032	7.9119	7.9883	7.3248	11.4047	1.7881	4.4898	1.7881	8.6636	2.9537	10.4903	10.0689	9.0357	2.7713	10.6292	12.8259	4.838	6.8116	10.509	12.2213	8.1485	10.2101	6.0177	11.8988	2.9537	8.9488	7.4902	6.2907	10.8236
TCGA-44-7670-01	882	0	0.6945	7.9596	9.0953	11.2684	9.9444	9.5135	1.9193	7.3515	7.0201	8.8515	8.3875	7.7435	6.0561	7.2437	9.2254	7.5572	7.5006	5.1132	10.2584	0.3886	4.289	2.2356	8.3565	6.5072	11.3242	10.0804	8.5014	3.3169	11.1065	12.2555	7.4794	6.8997	14.068	12.9064	8.1802	10.597	7.3404	10.8336	6.2964	9.101	7.9215	8.1491	9.5532
TCGA-44-7671-01	889	0	5.2784	8.4149	10.6198	9.2105	10.199	9.6832	0	7.7564	6.0971	8.4761	7.7436	7.842	6.1804	7.2109	10.3697	8.084	7.4164	4.8276	11.0429	0.705	8.5616	2.3494	8.9868	6.5195	11.6911	8.57	8.4736	2.159	11.4053	11.5163	6.5683	7.5534	11.425	12.7502	9.3141	10.687	5.5834	9.9952	4.446	7.9959	7.0589	8.9895	7.6584

TCGA-44-7672-01	719	0	2.2942	8.4771	7.5061	10.2238	9.497	10.1813	0.8326	8.6758	9.8472	8.5778	8.0895	8.2199	5.3095	10.6258	10.1127	7.9044	7.4605	7.3481	12.3038	0.4756	6.1555	3.712	9.0168	4.0204	10.8702	9.2558	7.962	2.693	11.7105	12.6382	7.9114	7.5092	11.718	12.8027	9.091	9.7437	5.1441	10.3379	5.1441	8.6469	7.4937	9.4086	9.2156
TCGA-44-8117-01	385	0	3.7159	8.4603	4.8162	10.5772	9.6985	10.9615	0.3664	9.1282	7.6078	8.3949	6.5153	8.0743	6.8073	9.9654	7.8649	8.6518	6.3743	6.0081	10.5444	0.6582	8.0541	2.1599	9.0748	1.7279	11.2965	9.2411	9.1376	3.0403	11.2355	13.3227	6.9625	6.9988	11.134	12.8592	9.6742	11.4287	3.2738	10.8458	5.974	8.1019	7.1708	8.8088	8.4048
TCGA-44-8119-01	285	0	10.525	7.8755	1.0371	11.0158	9.2394	9.4097	1.0371	8.3617	9.9438	9.3968	7.4992	8.5438	7.3017	4.4963	9.4515	8.1605	7.3611	5.767	10.9586	1.2111	6.1597	2.3061	8.8708	7.1172	10.1139	9.5032	7.9859	2.9214	8.9435	12.5487	6.7972	6.0088	10.79	12.1861	8.3492	9.3116	4.3175	10.4089	7.6617	8.4899	8.1619	9.669	7.733
TCGA-44-8120-01	260	0	2.3001	8.7915	6.9187	8.4912	10.625	9.6842	1.5668	8.5132	5.8477	8.7319	7.5285	7.9508	6.1343	6.7105	9.625	8.7479	7.2265	5.8833	10.8641	2.0299	4.8173	1.5668	8.9215	5.4559	11.8101	9.0033	8.1333	2.4556	12.8295	11.9365	6.1343	7.3088	11.372	12.7511	10.2938	11.3722	4.2002	9.086	4.3465	7.6583	6.7784	9.5049	7.2057
TCGA-44-A479-01	486	0	6.3403	8.1933	7.6737	8.2482	9.0435	10.1383	1.4343	8.1936	9.3111	8.5874	8.2509	7.9634	6.0378	4.667	9.9754	8.4396	7.2693	7.991	11.7873	1.9402	6.4379	3.9778	8.442	4.9042	9.8568	9.8309	7.833	1.7093	11.2548	11.4528	6.0378	7.1239	11.226	12.1571	8.7465	9.6834	5.6218	10.4121	9.0788	9.7237	7.253	8.8153	9.4563
TCGA-44-A47A-01	466	0	6.0217	9.0969	5.2615	10.8747	9.3588	11.1934	1.6074	9.4299	9.2017	8.2695	7.9433	8.5862	4.2016	8.2502	10.4749	9.5698	7.563	6.9746	11.6991	0	7.9099	4.4243	8.8116	7.7338	10.7465	9.3689	7.7916	3.6158	12.3037	12.8061	8.005	6.8081	11.182	13.0284	9.7305	10.4291	6.5489	10.4614	3.1998	9.6989	7.1208	9.3977	9.0503
TCGA-44-A47B-01	287	0	7.2849	9.0287	4.2413	10.2275	9.2021	9.8185	1.2287	9.0432	9.0858	8.8512	8.8343	8.6079	5.055	12.2352	9.4559	8.9757	6.3455	5.1406	10.8831	1.6956	5.8095	1.2287	8.3729	6.0816	10.1687	9.8206	7.5178	2.8619	11.7618	11.9226	8.5608	7.5178	10.982	12.2834	9.9213	10.5153	6.3507	10.3808	4.8685	8.3393	5.8548	9.4605	8.4283
TCGA-44-A47G-01	351	0	5.4831	9.1046	7.5546	11.3605	10.062	10.399	1.1048	8.2534	9.1645	8.8442	8.9537	8.6304	5.2412	10.3935	9.7111	9.2162	6.997	7.3718	11.6981	2.3299	7.0082	4.4782	9.0586	5.4831	11.4477	9.638	8.2242	3.4305	12.495	11.8878	8.2814	7.0147	10.944	12.856	9.5354	10.7181	5.7531	10.4155	4.1907	9.1994	7.2179	9.6891	9.1994
TCGA-44-A4SS-01	415	0	6.7159	8.9154	9.7473	10.4247	9.9976	10.2101	1.4122	8.0237	9.4928	9.0207	8.6707	8.1214	4.9755	4.4625	9.8435	8.107	7.1782	6.9041	11.8883	1.685	5.7145	4.8702	8.5416	4.6332	11.1833	9.2334	8.5437	2.286	10.8146	11.9493	5.7886	7.868	11.1	12.3556	8.9594	9.9014	5.4839	10.372	2.1121	9.6037	7.6506	8.5156	9.2268
TCGA-44-A4SU-01	409	1	4.0844	10.1346	4.0023	10.8237	10.618	8.6426	1.9325	9.2673	8.3983	8.3711	7.8841	8.4351	4.3061	6.6651	10.1478	9.881	7.1625	6.6054	10.9331	1.7432	8.6375	3.2406	9.1949	6.2592	11.7422	9.3188	7.9293	4.4982	12.0868	11.9549	7.1388	7.3403	11.636	11.4959	10.2806	10.7327	6.6176	10.6895	3.5021	6.8816	7.7139	9.5641	9.5028
TCGA-49-4486-01	2318	1	5.0409	10.2131	10.404	9.2553	11.834	10.1775	2.2195	9.8787	4.5188	8.2879	7.9641	8.2042	3.2697	6.173	9.759	9.3779	7.337	5.2036	10.9384	0	9.7607	1.5825	10.2686	7.6257	11.1558	8.542	8.222	0.7355	11.4959	13.294	7.9012	8.41	10.829	14.3642	10.4184	12.0386	7.0553	10.1932	3.2191	8.3332	8.8739	10.2518	8.9907
TCGA-49-4487-01	855	1	7.1972	8.6751	7.0582	10.7279	9.5827	10.5991	0	7.5637	9.1193	8.4433	8.1198	8.6174	5.4583	4.2243	11.1424	7.9385	5.9889	7.0979	11.3169	0.8817	4.6685	3.6774	8.5115	6.0959	11.0231	9.6658	7.5993	1.8186	11.5955	12.4637	5.8858	6.8736	11.119	12.9422	9.0637	10.6865	4.088	10.5905	4.0147	8.8978	7.3979	6.6618	9.115
TCGA-49-4488-01	869	1	3.0597	8.8941	1.9764	9.4609	10.23	9.7289	0.7939	9.91	8.9664	9.7609	8.2655	7.8725	5.6486	8.2307	9.3964	7.5156	7.7358	5.1121	11.4038	0	5.7106	2.4337	9.3206	1.6787	10.9535	8.7417	8.7927	1.3031	11.6506	13.7973	8.2201	7.6587	12.078	12.8813	9.4995	10.0326	3.9705	10.835	3.1814	8.6419	7.9771	9.6441	8.2758
TCGA-49-4490-01	385	1	2.506	8.9021	5.0368	11.2334	10.738	10.2005	0	8.6734	8.2873	7.4051	8.6088	7.6667	3.7191	7.8938	9.6647	8.3228	5.6855	6.6583	10.9061	1.9291	5.9722	2.506	8.987	7.3728	12.4932	9.7282	8.6157	4.5525	12.6527	13.3129	7.6865	6.6061	11.777	12.9897	11.0321	11.4583	6.3488	9.8444	4.8149	7.3647	7.3399	8.1718	8.6895
TCGA-49-4494-01	1081	1	4.0546	8.8759	8.9614	10.2266	9.9945	11.4173	0.8889	9.1711	7.9585	8.5506	8.7474	7.5577	4.15	7.9626	9.2057	7.5555	8.0744	7.4499	11.4714	0.8889	5.3488	2.4707	8.4915	4.8914	11.2797	8.4251	9.1599	1.4349	11.6587	14.7258	5.8169	8.5615	12.007	14.0855	9.9824	10.9107	3.207	10.1043	3.9525	8.4892	7.0119	8.6761	8.0409
TCGA-49-4501-01	1421	1	2.6517	8.9984	8.4867	9.1783	11.433	11.2656	3.8018	9.576	7.9824	9.3748	8.655	7.7429	5.7148	6.7584	9.3976	9.1933	7.6222	6.1512	11.2512	0.612	8.4739	2.525	8.2656	7.0987	12.1741	9.6337	8.0481	1.7561	12.5462	12.237	8.1576	6.9829	12.389	13.2131	10.1813	10.4574	6.8103	10.8245	3.158	8.5703	6.9678	8.8925	9.537
TCGA-49-4505-01	428	1	3.6119	8.3988	4.014	10.2602	10.747	10.178	1.5678	9.141	9.1649	9.1069	8.8434	7.2345	5.239	8.0686	10.0282	8.5174	7.9032	7.0385	11.4525	0.3569	8.1578	3.1006	9.2514	5.9256	11.5846	9.5286	8.4838	2.7253	12.4727	13.4111	8.4599	8.007	11.725	13.3018	10.7998	11.898	6.553	10.3285	4.7752	8.3611	8.631	8.2683	8.7172
TCGA-49-4506-01	999	1	9.15	9.0398	10.0346	9.0255	8.6052	10.5879	4.3005	8.4684	8.3754	9.4969	6.1262	7.5731	6.0151	10.6518	9.8658	9.4177	8.4833	8.5249	11.368	2.427	7.0991	2.427	9.7612	4.9838	11.1151	6.429	8.2244	1.7603	11.1816	14.9471	8.9636	7.3799	11.038	15.7306	11.0309	12.3978	5.0197	8.6444	2.7162	8.4215	9.7392	8.84	6.7871
TCGA-49-4507-01	268	1	8.4879	8.4064	8.4948	10.3064	9.6452	10.0986	4.8589	9.5471	9.3634	8.5424	8.354	8.7222	3.0468	9.6717	9.1817	8.4753	5.9424	7.9252	10.4733	0.4286	7.7628	2.0402	9.4334	5.1949	10.8931	7.7605	7.3446	1.4486	10.209	14.5182	6.4508	7.6555	10.53	14.9608	11.1309	12.649	5.6171	9.6803	2.2645	8.4225	9.253	8.4527	8.5624
TCGA-49-4510-01	896	1	8.9668	9.4177	5.1875	7.6035	11.052	11.4756	0.9693	10.094	6.8166	8.8094	8.7411	8.2017	4.9248	7.8649	10.5128	9.3195	7.0527	5.8926	10.147	0.7125	8.2464	1.8296	8.7561	5.3317	11.3436	7.613	7.7282	1.5438	11.8283	13.376	7.7217	6.9681	11.517	13.7675	10.6305	11.9578	5.8052	10.0965	6.0473	8.4798	7.0948	9.4378	7.3476
TCGA-49-4512-01	905	1	5.0896	8.8761	2.8378	9.0784	10.374	9.6446	2.6737	9.6809	8.2539	8.7166	7.8745	8.8134	3.4584	6.5188	9.5157	9.9979	6.1307	6.0507	10.4392	2.0267	7.6333	4.8797	8.6546	7.5529	11.6013	8.5312	8.0353	3.1188	12.5585	13.242	7.8264	7.0823	11.051	12.9298	10.8215	11.617	6.4444	9.502	4.5417	8.2761	7.5351	9.672	7.4297
TCGA-49-4514-01	1700	0	6.7359	8.2623	10.4673	10.6196	10.504	9.6816	2.0216	8.0468	7.3859	7.238	8.3187	6.9827	3.727	9.5843	9.4861	8.4633	7.0354	5.5941	10.8099	1.7204	9.3456	4.0932	8.8191	4.6275	11.9994	8.9609	6.6395	2.0216	12.4487	14.0506	4.027	7.6598	11.484	14.1045	9.0003	10.978	6.0283	10.5298	2.483	8.5976	6.6615	9.0594	6.8164
TCGA-49-6742-01	488	1	7.1939	7.979	10.3142	12.335	10.771	10.0303	4.4999	8.6681	6.2567	8.1592	8.4876	7.5035	5.1417	7.7383	9.5856	8.4995	6.2587	5.5512	10.7267	4.182	8.6028	1.317	10.0033	5.8499	11.3708	9.9907	7.8262	6.1211	11.1599	12.8041	6.7992	9.469	11.263	12.717	8.8858	11.1842	6.5232	9.0224	6.5464	8.7273	8.0898	8.75	6.9974
TCGA-49-6743-01	1621	0	9.2611	7.4615	9.3609	10.8619	9.9	10.9094	2.0894	7.4512	8.6596	8.0334	8.5376	6.2037	8.0717	5.7155	8.6932	7.1114	6.6382	6.5222	10.8488	2.0894	5.8707	1.6647	8.7452	6.9687	10.5161	9.4576	8.8422	8.6875	11.2888	12.4529	6.6713	8.7615	10.927	12.4144	8.8405	10.4148	5.9987	11.2274	5.3437	8.5626	7.302	7.0418	10.7298
TCGA-49-6744-01	1683	0	5.6695	8.4897	4.7531	9.9473	10.051	10.1327	1.6025	8.0271	8.9055	8.4376	8.9701	7.1959	5.7536	4.9322	10.0085	8.0457	6.6996	7.2243	11.7794	2.3429	7.9715	3.9314	8.6628	6.9744	11.3145	8.8415	7.7369	3.609	12.6227	12.1807	7.611	7.2403	10.789	12.9259	8.927	10.5115	6.3568	10.6501	3.8317	8.7438	7.5378	8.1003	8.3435
TCGA-49-6745-01	522	0	2.1505	7.9029	4.3338	11.2607	9.5998	10.3544	1.5683	7.7783	7.3266	8.8079	9.3738	7.5181	6.3328	8.9736	9.4272	7.0993	6.6875	6.8167	11.9656	1.7895	6.3151	4.4679	8.56	3.7317	10.7956	9.3544	8.975	2.6793	11.8927	11.5224	7.4097	7.7921	9.9072	13.042	7.4869	10.1728	3.7841	10.4096	5.385	8.5239	6.9695	5.5605	9.0411
TCGA-49-6761-01	354	0	1.678	8.4842	7.125	10.9742	9.4032	10.5548	1.678	7.7822	9.7549	9.5086	8.5813	8.0537	4.0986	10.9609	9.9853	6.8595	6.4191	6.6129	11.4485	0.7935	6.3122	2.2223	8.5619	4.3266	11.2108	9.5317	8.6355	1.3025	10.7332	13.1853	7.8115	6.034	11.011	12.8588	9.7427	10.4834	3.2927	9.9246	4.9908	8.1312	6.1428	9.9537	8.7283
TCGA-49-6767-01	677	0	1.7514	8.1787	2.3623	11.2879	9.778	9.698	1.985	8.0089	9.3689	8.7221	8.1459	8.2783	4.9066	7.4113	9.3757	7.2966	5.7974	6.235	11.2298	1.4725	7.3394	3.2147	9.6013	1.1266	11.0065	10.0117	9.119	2.3623	11.4636	12.6785	7.3861	6.0592	12.198	13.0589	7.6805	10.2776	5.3659	10.3565	2.5195	6.9203	7.6468	6.9132	8.8534
TCGA-49-AAQV-01	677	1	6.7005	7.9713	7.767	9.9969	10.288	11.3284	1.7208	9.307	8.8111	8.8555	8.2169	7.7735	5.0508	6.4145	9.7821	9.6906	7.5409	8.1652	11.5165	0.7278	6.9695	3.2523	9.1692	6.2748	11.728	9.4198	8.4847	4.4804	11.6834	13.7764	7.8349	7.5941	11.152	12.8332	10.4394	11.3472	6.1996	10.1772	4.008	8.5096	8.2792	9.8903	9.7659
TCGA-49-AAR0-01	4765	0	5.7159	9.5153	8.3585	9.7518	10.547	11.2876	4.8951	9.9048	9.6576	7.9651	8.8396	8.8643	3.6326	2.1604	9.8199	9.312	7.7733	7.1447	10.9029	0.5809	8.816	2.4495	8.7444	5.9653	11.5882	9.1042	7.6208	2.4495	11.7763	13.466	6.363	6.9537	10.878	12.8492	9.9955	10.6769	6.8081	9.8112	5.3975	8.6917	7.1427	9.6865	8.7642
TCGA-49-AAR2-01	2224	0	7.1314	8.7453	10.4549	7.4753	10.67	9.7152	2.7155	10.352	9.2979	8.7052	6.4933	9.6578	2.9645	5.5093	10.9028	10.5214	7.3422	5.342	10.6034	2.2364	9.8777	2.8454	9.7184	3.6013	11.6232	8.2399	7.4294	3.0746	10.7328	14.3016	8.5085	6.9628	11.81	13.6171	12.0867	12.4649	5.9456	9.2424	3.8066	8.6793	7.2824	11.0093	6.9044
TCGA-49-AAR3-01	1893	0	3.2796	8.04	6.9214	9.8359	9.2545	10.4583	1.4553	8.7876	9.4506	8.5259	8.073	8.4809	3.8142	8.4088	9.6085	8.77	7.4604	6.8592	12.0095	0.9039	6.3881	4.06	9.3992	2.421	11.3127	9.4771	7.9206	2.421	10.9328	12.51	7.8296	7.6844	10.735	13.1827	10.2929	11.2767	4.7619	9.283	5.8702	8.6717	8.1325	9.3805	7.8241
TCGA-49-AAR4-01	879	1	6.5594	9.2969	6.1495	9.6796	9.8607	10.0673	0	9.3777	8.7135	7.8447	9.0686	9.4055	3.832	9.1429	10.0076	10.0783	7.9895	7.4381	11.0457	1.8164	5.7198	2.7387	10.1624	3.471	11.3082	9.7233	8.0713	6.3635	12.1708	12.9651	7.3435	8.1014	11.102	12.8161	9.6994	9.936	6.0705	10.5923	4.0119	7.9878	7.4305	9.5701	9.3619
TCGA-49-AAR9-01	260	1	2.2239	7.3848	9.086	12.6612	10.169	10.0681	1.7201	8.3245	6.9002	8.6498	7.9294	8.7048	5.387	5.2545	10.2163	8.2626	6.7577	6.7893	10.7468	0.9395	6.7416	0	10.4693	8.3865	11.6083	9.103	8.2561	3.4111	8.7095	12.6588	4.7388	6.6391	11.921	12.6409	10.9957	12.3081	6.7167	10.513	3.2111	7.4469	8.1223	9.0565	9.0212
TCGA-49-AARE-01	1229	1	4.514	8.2974	9.9757	11.2083	9.7396	11.9015	1.8996	8.6793	9.3009	7.865	8.4252	8.5893	4.1571	4.6769	8.8802	9.1877	7.4998	5.1222	10.7128	0	9.3695	3.1273	9.5148	3.1273	10.9249	10.129	8.4517	6.814	11.07	14.3987	6.1237	9.2857	12.034	13.2279	9.0081	10.9639	6.5593	8.9772	3.6298	9.1197	7.7839	10.2558	5.975
TCGA-49-AARN-01	1135	1	5.6296	8.8937	7.7036	10.3379	9.5627	10.2443	0.8043	8.5523	6.4878	8.1864	9.233	8.843	4.5477	8.2047	10.4141	9.5684	7.4041	4.8595	11.2666	0	7.3548	1.6955	10.6121	7.5046	11.406	10.0075	7.574	2.2422	12.6929	11.7283	6.4275	6.4646	11.028	12.6749	10.0649	10.6312	7.1802	10.0549	2.9479	7.0563	8.1337	8.88	8.1413
TCGA-49-AARO-01	3759	0	3.8911	9.5572	4.5537	11.0038	10.059	9.9417	1.7251	8.5198	9.0519	8.2104	7.9291	8.7246	4.5537	8.803	9.8273	9.3357	7.3465	6.9011	10.8667	1.4487	8.402	2.8762	9.8821	5.4673	11.1387	10.4849	7.6432	3.5794	12.5329	12.1949	7.0493	7.8411	10.565	12.5958	10.2848	11.6406	5.644	9.5921	5.1767	8.0533	7.9892	8.6786	8.2816
TCGA-49-AARQ-01	6732	0	2.1279	9.9001	6.9415	10.6684	10.308	12.3339	1.4251	11.189	8.5793	7.1725	9.179	9.7706	2.5984	7.415	9.883	10.3056	5.9436	7.3642	9.4193	2.1279	3.3993	2.8439	9.2842	7.5379	11.8307	9.8597	7.7333	2.5984	12.0059	14.2807	7.0916	7.4339	12.804	12.8216	10.2165	10.9543	6.7287	10.5278	1.7141	7.9971	8.2201	8.7395	10.3621
TCGA-49-AARR-01	4992	0	3.7821	9.5381	6.3418	10.4754	9.7504	10.8857	5.137	9.0447	6.7962	8.8139	8.9644	9.4618	6.6437	3.5504	10.4867	9.8286	6.5914	7.0728	11.4273	2.022	7.4215	3.8346	9.4238	4.2372	10.6763	9.2796	7.8398	3.5504	12.1967	11.3565	6.3418	8.9951	10.31	11.72	9.4753	9.6946	5.8868	9.5401	5.849	9.0599	8.389	8.0599	9.0873
TCGA-4B-A93V-01	300	1	7.6996	9.5304	9.4293	9.9634	10.358	11.3389	0.9725	8.8345	9.8267	7.4245	6.3288	7.8988	4.0769	2.5388	10.4438	9.409	7.8227	7.1587	11.0824	0.9725	9.2608	2.2776	10.0628	1.7679	11.3365	9.7027	8.1925	0	7.9162	13.734	6.095	7.0792	10.699	13.5806	11.1065	12.4479	6.0005	10.2653	3.1206	6.9392	7.4123	10.1194	6.9449
TCGA-50-5044-01	624	1	1.0169	8.9877	3.3522	10.4376	10.036	11.0258	1.6074	11.463	7.6212	8.6917	9.2307	8.8409	2.3489	11.5404	9.9024	8.0669	5.7825	5.3576	10.9993	2.0252	6.0775	3.1998	9.9921	5.7878	12.101	7.468	6.964	2.3489	12.5554	14.2883	9.8165	7.1523	10.304	14.0343	10.6971	11.3039	4.3538	9.626	6.0554	8.4471	8.5173	6.4082	9.0279
TCGA-50-5045-01	2174	1	4.4131	9	3.6347	9.4768	10.427	11.3569	2.2652	8.8538	9.477	9.1611	8.4389	7.7766	4.8749	8.9354	9.7169	8.376	7.2931	8.4247	11.8834	0.8844	6.9749	3.4793	9.0504	4.6981	11.0968	8.856	8.0611	3.1759	11.2751	13.3436	7.3186	7.7082	11.909	13.9115	10.5743	11.3161	6.3161	10.2796	4.3842	9.6217	8.9204	7.8525	9.9938
TCGA-50-5049-01	3094	0	3.0452	8.2664	1.0814	9.7931	9.7674	11.2308	0	8.0916	10.7838	9.6945	8.4911	8.0404	6.7191	4.5436	10.0286	7.588	8.0858	7.1351	12.2638	0.6397	5.4584	4.767	8.8979	4.9343	10.6351	8.7107	8.5438	3.0452	11.0539	12.9146	6.0013	7.2017	11.264	13.0037	9.3455	9.9206	6.2752	10.1441	5.4946	10.0569	8.3502	8.8463	8.8411
TCGA-50-5051-01	478	1	9.1078	9.4345	10.3925	7.3478	9.9672	9.7326	2.6831	8.7491	6.3919	8.7171	7.4016	9.2777	4.1732	5.1005	10.7454	8.9451	8.7284	5.4458	9.7469	1.7328	10.008	2.8473	9.6584	3.1286	10.7616	8.481	8.0421	0.8275	11.088	12.9866	6.5538	8.9631	10.742	12.8774	9.722	11.4146	5.6154	10.3799	1.35	6.7007	7.1177	10.1893	7.196
TCGA-50-5055-01	1830	1	3.2181	8.6306	7.7049	9.5527	10.067	10.1555	2.581	8.3424	9.3638	9.1504	9.018	8.1211	5.0614	6.3051	10.0441	8.553	6.9699	7.6871	12.0081	1.8039	6.2112	4.55	8.9101	6.2589	11.1406	8.3066	7.722	4.4439	11.0148	12.6544	7.1933	7.5408	12.198	13.1943	9.6881	11.0418	6.3351	10.1161	4.6488	8.4676	8.7327	7.6463	8.9101
TCGA-50-5066-01	1442	0	6.4324	9.8011	2.6384	9.8523	10.598	10.833	3.2796	8.6271	9.2772	10.3747	9.299	7.0502	6.8359	6.6229	7.8185	7.8624	7.9137	8.7556	11.7689	0.9039	5.4199	4.2033	9.295	1.8533	10.652	8.3367	9.2627	0	9.9161	14.5968	7.2284	7.9102	12.422	15.1379	10.2899	11.1047	4.2033	9.8925	2.1649	9.7155	10.4503	6.6101	10.2094
TCGA-50-5068-01	1499	1	3.7893	9.4038	7.0141	9.4326	10.449	10.3545	0	9.1887	8.7741	10.3308	8.8434	7.0703	6.2199	4.5902	8.051	9.2441	8.2318	7.8517	12.3565	0	5.7323	1.834	9.186	0.8915	10.4271	9.0343	8.862	2.3993	10.9214	14.1391	8.8162	8.8831	11.693	13.9362	10.7138	10.6523	5.2061	10.325	1.4384	9.3097	10.4851	7.5032	9.6677
TCGA-50-5072-01	250	1	9.3442	7.8228	11.716	10.3359	10.08	10.3183	9.6907	8.1677	7.0394	8.3858	8.5135	7.7031	6.1818	7.3741	9.7107	7.8973	6.3718	6.9056	11.2042	2.2312	7.7749	3.539	9.5723	5.3801	10.8875	9.4005	7.8519	2.987	10.6477	12.7897	7.402	6.0572	10.179	12.9351	9.1964	11.7595	5.9535	9.2941	4.7723	7.9972	8.3698	8.972	8.0535
TCGA-50-5930-01	282	1	8.2086	8.593	9.6802	10.828	10.174	9.8392	0	7.6592	7.9277	8.4079	7.7559	7.9388	7.451	5.1509	8.9397	8.3327	6.648	7.1065	10.7841	2.6581	7.5868	4.191	9.0523	7.3011	10.6629	8.3679	8.4741	6.5335	12.2719	11.9462	5.7223	7.0351	11.559	13.16	9.6108	11.3936	6.5942	10.9656	4.0821	8.6417	7.3952	9.1019	9.4988
TCGA-50-5931-01	434	1	6.9481	8.4621	3.7683	11.0496	9.4945	8.2451	1.178	8.3496	5.3181	9.0755	7.8584	8.581	6.4195	2.5971	10.2172	8.2923	7.1848	2.2479	10.3425	1.8177	8.48	2.4381	8.5905	7.9643	10.8351	10.931	7.9009	2.0554	9.2871	13.9112	6.5804	6.8169	10.139	13.1824	8.6731	10.971	8.4826	11.0781	3.9561	7.8346	6.8724	10.3941	10.4935
TCGA-50-5932-01	1235	1	6.1961	7.6415	6.4504	8.5219	8.6867	8.4586	0.8729	9.2526	5.7284	8.9734	7.7761	7.5988	6.2601	9.0703	10.5684	8.4586	5.7598	6.0933	10.8905	1.4128	6.935	2.2451	9.6525	4.1396	9.8204	9.2356	8.5558	1.8048	12.2515	11.2384	4.9312	6.5433	11.568	12.5771	9.2085	11.1426	5.6923	11.0561	4.5253	7.278	7.2107	9.7045	8.8502
TCGA-50-5933-01	2393	1	1.0147	8.428	6.0289	11.8537	9.6486	10.1663	0	7.7826	9.5474	9.3273	8.4064	7.4679	7.1219	8.6875	9.2338	7.3006	6.4574	6.6823	11.592	2.3454	5.7152	3.9816	8.9878	0.5948	10.3245	9.1879	8.714	3.1134	12.4452	10.9233	6.2187	6.3236	10.974	13.0311	8.0404	10.094	3.4189	10.8869	2.9323	8.5972	7.0515	8.9286	8.3123
TCGA-50-5935-01	653	1	2.0646	8.2661	5.2207	11.4586	9.5538	9.4927	0.7107	8.4989	8.7683	8.5859	8.0088	7.8387	5.2451	7.5656	10.5717	8.1801	6.4896	5.8394	11.4966	0.7107	8.7464	3.0005	8.5545	7.3906	10.4164	9.1439	8.0784	3.2135	12.3254	12.1788	8.3558	7.7433	11.531	12.1033	9.2382	10.818	6.4939	10.8527	4.6909	7.8546	5.8654	8.3073	7.6909
TCGA-50-5936-01	257	1	10.939	7.9673	9.5142	10.957	9.3001	12.2235	4.4241	7.5095	8.6588	8.6652	7.5998	7.9418	6.8088	5.5107	9.7019	7.7197	5.5546	5.6675	11.0244	2.2188	6.8999	2.7757	9.5694	5.9636	10.4461	7.6267	7.6636	2.4292	11.0152	12.6037	5.9804	7.6636	10.67	13.1605	7.1357	11.7897	6.7215	10.3851	3.7471	8.6417	8.2252	8.9346	7.533
TCGA-50-5939-01	460	1	9.394	7.6044	6.4908	12.0884	9.7196	10.4199	3.2031	8.224	8.7369	8.9086	8.7248	8.2187	6.2266	6.6067	9.5257	7.958	6.448	6.7298	11.156	0.752	6.6368	3.4934	10.361	5.1655	11.3074	8.3455	8.0502	2.8394	11.6192	12.1046	7.1628	5.9831	10.741	12.9482	9.0211	11.6475	6.3527	9.1131	5.3703	8.5115	8.5115	8.7272	8.1509
TCGA-50-5941-01	1474	0	4.5896	7.9604	5.8107	10.6677	9.8276	10.9456	0.7146	7.4713	10.1786	9.2251	7.609	7.9642	5.9667	9.0494	10.1029	8.282	6.1315	7.039	12.195	0.7146	7.6279	3.0092	8.5223	2.8895	10.9119	9.0459	8.45	2.759	11.7937	12.2295	6.173	6.8628	11.676	12.9279	9.0529	10.702	5.1544	9.943	4.6644	9.2541	7.7954	9.3995	8.6567
TCGA-50-5942-01	1847	0	3.4892	8.6778	4.086	9.4079	9.8349	8.373	1.1881	9.4035	5.9953	8.1246	8.057	7.8686	6.7603	4.086	10.1356	9.0525	7.1608	3.9524	10.7097	1.8307	8.4981	3.2189	9.1612	8.5678	9.9569	8.0912	7.8843	2.4529	13.62	11.3804	8.6204	6.8022	10.571	12.5114	9.1385	9.7614	6.8747	10.7097	3.1163	6.3497	7.5716	7.9228	8.891
TCGA-50-5944-01	1750	0	4.176	8.134	3.7221	9.9219	10.267	9.0868	1.7822	9.6046	6.4616	8.228	8.3941	6.7593	5.9958	3.7873	10.003	8.3217	6.8221	6.8309	11.0049	0.6869	8.7636	2.3975	8.5525	9.1141	10.6824	9.1173	8.1527	3.2537	12.0807	12.2517	6.2011	7.167	11.333	12.6178	10.4284	11.7903	6.7098	11.2111	2.0176	7.9434	5.9396	6.6843	9.1517
TCGA-50-5946-01	1617	0	5.093	9.1507	3.8265	9.0316	9.9066	8.5584	1.1932	8.6516	7.4224	8.6923	8.0763	8.4122	6.7606	6.6994	10.5516	9.5954	7.7202	6.8929	10.6782	2.076	6.8757	0.7166	9.0958	6.001	11.365	10.8021	8.7452	5.3421	10.7637	12.311	5.8958	7.3582	11.419	12.1603	9.3636	10.6224	6.2034	12.022	5.1851	6.9224	7.5212	9.7788	10.7909
TCGA-50-6590-01	1288	1	0	7.6704	1.3953	9.9113	8.6158	10.681	0	9.1886	10.1733	7.8646	8.202	8.7012	6.971	4.7882	9.1808	7.4594	7.5107	7.2585	12.0564	0.6262	5.5771	2.6859	9.1031	1.3953	9.8249	7.7563	8.9561	1.8943	10.8826	12.5039	6.3379	6.5536	10.789	13.3832	7.9271	9.9672	4.8163	10.2143	2.5586	8.9827	7.7527	8.8899	8.4598
TCGA-50-6591-01	119	1	1.134	7.2264	2.6727	10.8772	9.0603	9.3424	0	8.4597	6.1427	8.8653	8.4991	8.9391	5.1557	5.5361	10.637	7.81	5.9341	4.5842	10.215	0	3.227	0	7.6983	4.53	11.4079	8.7052	7.7632	1.9953	9.4022	12.3122	6.0599	4.8911	11.176	12.4165	7.42	10.987	5.0295	11.1958	4.3303	6.1354	7.3583	7.8739	10.3097
TCGA-50-6592-01	777	1	7.0906	7.5255	10.1622	12.1412	9.8632	10.9374	1.8355	6.6231	10.1162	9.5635	8.3276	7.3205	7.5322	6.3024	9.07	7.6901	7.4646	6.8296	12.034	1.8355	6.341	2.8061	9.7876	7.3511	10.4116	9.2512	8.5564	2.5134	11.2661	11.9128	7.8856	7.3699	9.062	12.6849	7.7995	10.3227	2.715	9.8888	8.3043	8.4927	8.083	8.0461	8.7177
TCGA-50-6593-01	336	1	6.3879	8.2709	6.2119	10.1672	9.8727	10.2394	1.8342	8.6213	7.7335	8.5678	8.7049	7.8293	5.9823	10.1664	10.1518	8.8281	7.2772	7.308	10.9817	0.7149	6.3879	3.6491	9.2182	1.5481	11.1348	9.1644	8.824	3.5734	12.7003	12.6389	7.3226	7.1831	11.67	13.4111	9.6535	10.6382	4.6653	11.4076	4.7365	8.2709	7.7796	10.2964	10.1445
TCGA-50-6594-01	370	1	6.6782	8.0232	2.3855	10.0785	10.649	9.9187	1.6382	9.4737	8.6524	8.9564	9.3896	6.1583	6.5754	2.5679	8.4379	8.267	6.4839	4.267	11.7252	1.6382	9.3789	1.6382	9.238	5.8236	11.4669	8.5932	8.4172	1.2681	12.1006	13.3416	8.6576	7.0131	10.9	13.8462	9.7754	12.4171	5.2334	12.0531	3.9808	8.2703	7.3233	7.8314	6.2004
TCGA-50-6595-01	189	1	3.1238	7.8	0	11.6619	9.2648	10.5036	1.193	8.432	9.1555	8.6818	8.78	7.881	5.9564	5.3977	9.3625	6.6163	6.3208	5.897	12.6019	1.193	3.6526	4.1473	9.3064	6.2155	11.0036	8.9264	8.5812	2.8935	10.8652	12.0625	7.1868	6.8271	10.904	12.8836	8.3562	10.7881	3.8585	10.2446	5.9413	7.9908	8.5493	6.6351	9.0748
TCGA-50-6597-01	1268	1	3.7282	8.2547	7.4849	10.6569	10.42	9.622	2.7344	9.7504	8.9051	8.2855	9.4005	7.3329	0.9579	7.9093	9.8201	8.0848	4.6833	5.1072	10.281	0	8.4384	2.7344	7.5589	7.8158	12.1146	7.7159	6.1446	1.9364	13.1906	12.7807	7.2901	6.4391	12.79	13.9354	10.9111	12.2475	6.3913	10.058	2.5141	8.3411	7.1819	6.846	5.9603
TCGA-50-6673-01	22	1	6.8577	7.8895	8.8949	10.7757	9.7287	10.6629	0.6424	7.9436	7.9544	8.7294	8.525	7.4685	5.9703	9.147	9.6743	7.7309	5.8449	6.22	10.9461	0.6424	7.8584	3.9089	8.5746	6.8716	11.1681	9.6782	7.4866	1.9276	12.7159	12.355	6.5036	6.402	11.582	11.9492	8.4598	10.1154	6.8224	11.0425	5.9042	8.4621	5.5714	9.4601	8.2285
TCGA-50-7109-01	308	1	7.3243	8.1	4.5215	10.3779	9.6857	9.5769	0	7.1204	8.1959	9.6478	6.6417	7.9655	6.4933	1.1508	10.3039	7.9935	5.7181	6.0277	10.5285	1.1508	7.852	1.7827	8.1931	4.1768	10.1682	8.2693	8.3659	1.7827	10.1826	12.0811	5.5845	7.63	11.759	12.9655	8.9785	11.3483	5.6561	11.8426	10.3212	8.2891	6.1778	8.9923	9.5085
TCGA-50-8457-01	1125	0	5.0031	8.8688	3.9263	8.7399	9.7118	9.6605	1.8731	8.4538	8.2985	8.4396	7.8429	8.4211	6.1524	6.0106	11.1269	9.2207	7.076	5.4317	11.3273	0.53	7.7626	2.6613	8.8674	7.2216	10.6909	9.3714	7.7596	3.0182	13.3081	11.7681	8.1686	6.6877	10.577	12.6826	9.9277	10.3499	6.969	10.7623	3.6055	8.3444	7.2429	8.7593	7.505
TCGA-50-8459-01	1119	0	4.0588	8.6834	7.4091	10.9795	9.5543	9.707	1.925	8.7602	8.6451	8.5105	9.1591	8.3366	5.4983	9.4816	10.0704	8.5149	6.2306	7.4062	11.024	1.4214	6.9223	4.8567	10.5169	5.7885	10.8163	7.903	7.4091	3.7343	12.0602	12.1527	7.5579	7.2591	10.663	12.6367	9.2507	10.7805	5.6506	10.181	4.3633	8.7221	7.8017	8.0864	8.6971
TCGA-50-8460-01	829	0	2.9663	9.2557	5.9904	10.7228	9.9574	11.1718	1.163	9.6959	7.1817	8.8419	9.2321	8.1187	5.0267	6.1374	10.7531	8.7453	6.6368	5.2135	10.9481	2.0349	9.2557	3.0764	8.6483	8.7782	11.0392	8.6987	7.3296	3.5277	12.2308	13.2886	7.991	7.2199	11.139	13.0229	10.6395	12.0028	6.6547	10.5695	5.7459	10.494	7.0278	8.6439	8.4581
TCGA-53-7624-01	1043	1	1.6762	8.0181	5.7933	10.7236	8.7381	9.2705	6.8858	9.3645	9.3703	8.5682	7.4359	8.5396	6.9071	3.0817	8.2502	7.8108	7.6225	7.9779	12.3265	4.5751	7.6655	5.1398	9.0587	3.5286	10.4613	8.0009	7.9299	1.6762	10.5332	12.4415	6.6284	5.3563	10.814	11.658	8.5121	9.832	4.7023	9.6499	2.1742	7.9557	7.0041	8.6821	9.7342
TCGA-53-7626-01	929	1	5.3518	7.7931	2.3973	10.1606	9.6873	10.6031	2.4732	7.6814	8.7589	8.1138	8.4773	8.2304	6.2769	1.8322	10.6501	8.1775	6.9485	6.7996	11.3206	0.8903	7.457	3.491	8.3346	3.2534	10.9568	9.4408	8.3409	3.9286	12.6285	11.4766	7.0117	7.0617	10.507	11.7604	8.5569	10.1407	6.7044	10.7025	5.4012	9.1022	7.4095	8.7824	9.1208
TCGA-53-7813-01	424	0	7.1477	8.4488	7.6903	10.1519	9.6338	8.824	1.6114	7.8866	5.6995	9.4274	7.8207	8.4906	3.0346	1.0198	9.5273	8.0783	6.2321	4.8578	11.6371	1.0198	6.3244	2.3536	8.3015	5.3239	9.7708	9.9418	8.1942	0	11.3027	11.0289	6.3428	7.5471	11.422	13.6384	7.5784	11.0825	5.6236	10.4699	2.6179	7.8659	7.7604	8.0672	7.0836
TCGA-53-A4EZ-01	1071	0	7.3907	8.7906	10.4251	7.1321	10.856	9.5547	1.5691	9.1944	7.4974	9.7825	8.7816	9.4129	3.2113	4.2553	8.8606	9.9095	8.8379	6.7467	9.8609	1.5691	9.2131	3.3844	9.2293	1.7487	11.272	10.1036	8.7867	1.5691	11.7553	12.3399	8.1808	6.3584	12.665	13.1256	8.0455	9.9651	7.7271	11.6184	3.2113	7.7431	5.5189	11.6222	9.0883
TCGA-55-1592-01	701	1	4.9562	8.7804	6.885	11.1288	10.987	12.718	1.5333	8.129	6.7146	8.5695	8.1239	7.2685	5.9134	4.4667	9.9653	9.4084	7.8272	7.3037	10.9765	1.6623	9.2672	2.6188	9.0873	5.4516	10.8315	10.5695	8.9961	5.8873	11.8163	11.4529	7.3922	8.2965	12.556	11.6292	9.2861	11.1122	6.2109	10.4188	7.5446	9.6814	8.0741	8.9597	10.6456
TCGA-55-1594-01	1178	0	6.6044	8.2219	8.5711	9.8988	9.8505	10.1743	1.3631	7.5702	8.7262	8.88	9.5766	7.4875	6.1249	6.2643	11.6411	8.4531	6.6257	7.822	11.5018	0.8369	7.0346	3.1476	9.2204	6.1885	10.3069	11.62	8.4187	2.3018	9.9212	11.3579	5.8481	7.2057	13.635	12.6005	8.6122	10.8921	5.1212	11.7395	4.5488	8.5039	6.024	8.2408	10.8689
TCGA-55-1595-01	1479	0	7.702	10.1145	3.3443	8.3133	9.248	10.1575	1.1511	7.0808	7.7154	9.3166	8.2854	8.8379	5.6911	9.7179	10.1888	8.9971	6.6082	5.1483	11.0993	1.5014	6.8625	3.3443	8.6269	7.131	10.4107	10.352	8.1353	1.1511	11.3608	11.2938	7.6656	5.7899	10.755	13.5357	8.9763	10.6081	6.3608	10.9892	5.9763	8.0077	7.8429	9.4679	8.2366
TCGA-55-1596-01	2065	0	8.5628	8.0662	12.1677	10.2611	10.818	10.0542	1.127	7.8861	7.8883	9.3269	7.392	6.6421	6.6074	3.6827	8.9086	8.0662	8.2215	8.0179	12.2063	1.752	9.0998	3.4679	9.4196	2.9093	10.6483	9.6433	8.6778	7.7142	8.5673	12.8383	9.7009	7.9876	12.932	12.6989	9.9309	11.9759	6.6842	9.7111	3.1205	8.6312	8.8616	9.705	5.6649
TCGA-55-5899-01	930	0	0.6521	7.875	9.7813	10.9547	10.345	10.0072	2.6191	8.2797	8.7424	10.2178	7.6676	8.1978	5.8475	5.3974	8.7041	7.8466	8.8279	7.6192	11.4089	0.6521	7.2194	7.9368	8.4707	12.9032	10.5146	7.3715	8.209	1.4407	10.7173	13.0566	6.6502	7.128	11.944	13.0286	8.7546	10.5275	7.536	11.6725	3.1701	8.4707	8.6081	7.2413	8.7237

TCGA-55-6543-01	435	0	5.475	9.1107	3.3101	11.108	9.6938	10.7906	1.0805	8.545	9.3861	8.6179	7.6717	7.5046	5.8544	3.0438	11.0949	9.0051	7.096	7.6243	11.0663	0.6391	7.3157	2.4487	8.7091	3.4638	10.6704	7.8976	8.5095	5.5627	11.8628	12.2863	4.5468	7.4179	10.983	12.5415	8.9655	10.097	5.6613	11.0516	2.7168	8.8861	7.4366	6.9036	10.0852
TCGA-55-6642-01	2449	0	7.0943	8.2927	5.757	9.7396	9.0458	9.6612	1.2083	8.0205	8.0925	8.7533	7.4538	7.4752	7.9921	2.6421	9.8726	8.0856	7.8252	5.4855	11.3827	1.2083	7.3988	4.2742	8.6442	2.3021	9.8337	8.1502	8.2311	1.8565	11.8722	11.6266	4.7452	6.7524	11.008	12.6628	8.7201	10.458	5.8745	11.0594	4.4999	7.7145	7.3876	8.4388	8.5937
TCGA-55-6712-01	171	1	2.8627	8.6106	1.4816	11.5738	10.558	9.582	0.9232	7.9656	8.5072	9.5904	8.8674	6.9827	6.3674	6.544	8.6972	7.7436	6.9294	5.7461	11.8289	0.9232	6.2533	3.1805	8.6433	5.0165	10.9752	9.2727	8.811	3.3165	11.7074	12.2647	7.5223	9.8756	10.594	13.4838	9.3078	10.6357	3.5552	10.3128	2.8627	8.1554	7.033	7.9872	6.6117
TCGA-55-6968-01	1293	1	6.9281	7.8667	5.4758	10.434	9.6917	10.2152	1.1008	8.0337	10.4891	9.4686	8.5718	8.802	6.2905	4.0898	9.0758	8.4496	7.7897	7.2257	11.3675	1.9493	4.8317	3.7648	8.9777	5.4758	10.3699	8.6209	8.3212	2.8672	10.1865	12.2262	5.4758	5.6667	10.094	12.3196	7.9592	9.8953	8.1369	11.2658	0.9041	9.4176	7.0701	8.1369	9.0543
TCGA-55-6969-01	1239	0	2.4553	8.0567	8.7919	10.5585	10.27	10.0276	1.0249	8.1362	10.5807	9.7722	7.5222	7.8349	5.7581	3.0433	9.8422	8.0918	8.4062	6.8115	11.6102	1.0249	5.6826	3.7461	8.5794	1.6181	11.0627	8.9532	8.2603	1.7717	9.3501	12.7555	7.2683	6.1291	11.215	12.5395	9.1605	9.6418	6.4239	11.1361	0.9053	9.1769	7.5276	8.1473	10.0938
TCGA-55-6970-01	464	1	9.2298	8.2011	9.8725	10.8865	9.0923	11.0532	0.7159	7.593	8.3443	8.9636	8.1676	7.704	7.7945	6.7459	10.3979	7.4809	7.4051	6.7275	11.8578	2.1807	7.5444	1.8359	8.4185	6.0064	10.4862	8.7933	8.5129	3.0121	11.6666	12.291	5.6574	5.8302	10.81	12.4678	8.5039	10.3631	5.0215	12.0008	5.0498	9.8273	8.486	10.3152	10.0159
TCGA-55-6971-01	1400	0	8.0032	9.0774	5.598	12.0068	9.1131	11.2841	1.5539	8.0008	9.8642	8.5382	7.8051	8.6846	5.8608	5.2763	10.0604	8.6981	7.5243	7.0961	11.8582	0	5.9081	3.4169	8.9812	4.5401	9.8884	7.9977	8.5863	3.5421	11.5178	11.7569	7.1823	6.9759	10.597	12.6955	8.805	10.7345	5.6584	10.5587	3.5421	9.848	7.062	7.6116	9.2638
TCGA-55-6972-01	1632	1	5.7107	11.1075	9.9507	5.1854	11.602	9.0486	1.7151	10.1	3.7052	8.7755	8.2182	8.4144	5.2732	0	9.6665	8.121	6.3086	7.011	10.8783	0	9.7182	0	8.6499	6.7126	11.3112	9.3148	8.9785	1.0987	10.4855	12.5817	7.212	8.335	11.226	13.6996	9.8454	11.1371	6.6152	10.3033	3.8776	7.2231	8.6376	8.5828	7.3395
TCGA-55-6975-01	118	1	5.6569	7.2547	7.4192	9.8251	9.2296	9.7846	3.5725	8.413	7.1467	8.2219	7.4192	7.4814	6.3776	5.3658	10.4443	7.1564	7.3821	7.1328	11.0658	0.8783	7.1394	3.0949	8.4326	4.5106	10.7364	7.9411	8.6194	3.2299	9.8758	12.5978	4.8826	7.3015	10.996	12.3494	8.4291	11.3225	3.7627	9.6299	6.7919	7.3834	8.1683	7.9655	8.1299
TCGA-55-6978-01	176	1	2.3112	8.2631	4.276	10.6768	9.0534	10.5414	0.7828	8.0541	9.3892	9.0267	8.7877	8.2167	6.1402	10.2043	9.4428	7.579	6.9503	7.1215	12.6567	1.4861	5.6847	4.0117	9.0247	2.3112	10.3239	10.1011	8.9213	3.3229	11.2769	11.68	6.8659	8.9213	10.515	13.0329	8.1322	10.1297	4.1053	10.3112	6.001	9.4869	7.498	8.0105	8.7399
TCGA-55-6979-01	237	1	3.5324	8.9797	2.1774	10.633	9.5724	11.2413	1.2687	8.1294	10.6853	8.4062	8.6835	8.4735	5.6123	4.9353	9.5387	7.902	6.3883	8.0531	12.4283	0	5.7882	5.0625	8.169	2.8762	10.855	9.2288	7.7778	3.6177	11.6031	11.6827	5.8424	7.8053	11.129	12.7322	8.8818	9.8553	5.2071	10.5466	5.6533	10.1193	7.8719	8.43	10.2174
TCGA-55-6980-01	2109	0	6.234	8.7146	3.7418	10.7044	10.523	10.6867	1.469	10.096	8.5865	8.6901	8.1937	8.113	4.5843	8.1313	10.2686	8.4988	5.7527	6.1322	11.0567	1.469	6.3942	4.7827	8.4153	5.9126	11.7591	9.43	7.873	2.8458	12.2654	12.3538	7.2158	6.579	11.358	12.1191	9.559	10.5513	5.8697	10.5749	4.3542	8.1038	7.6241	7.9779	8.7799
TCGA-55-6981-01	1379	1	6.7898	8.1062	3.7313	10.917	10.172	9.3426	0	7.4127	7.6119	8.4039	8.1062	7.3743	4.946	7.6192	9.5777	8.2669	6.293	6.0917	10.4341	0.9086	6.6456	1.8606	8.817	4.5192	11.3718	8.7539	7.9045	4.5192	11.8629	12.9956	5.5696	7.4844	11.894	13.0227	9.8528	10.9972	3.0032	10.5133	2.836	7.2045	7.4193	8.8309	11.1839
TCGA-55-6982-01	995	1	3.7977	7.5767	8.2839	11.7044	10.01	10.1979	0.9425	7.875	9.8713	9.4161	8.4104	7.4379	5.1338	8.7716	9.198	7.7224	6.4361	6.6769	12.0055	0	6.8494	3.2167	8.9381	7.5557	10.8068	9.5194	7.7597	2.8978	12.2742	13.4019	8.6108	7.3911	13.019	13.0395	8.9861	11.2231	5.8619	13.3433	2.7073	8.7716	6.2061	9.1284	7.7097
TCGA-55-6983-01	2823	0	7.2145	8.1496	8.1729	9.0413	9.4282	11.0497	1.6451	9.3114	8.079	8.4034	8.2044	8.0889	5.409	7.4196	11.1137	8.448	8.5204	6.0367	11.1181	0.7733	5.6522	3.8904	9.1244	5.9947	10.4104	8.5661	8.4899	2.3938	12.07	12.9086	7.5806	8.2935	10.438	12.7578	9.2929	9.9789	5.8778	10.654	4.1997	9.0089	8.0373	9.1933	8.716
TCGA-55-6984-01	760	1	2.4778	8.5361	10.1172	12.3367	9.7409	9.9527	0.9367	7.6782	6.1678	9.2757	10.0164	8.1562	4.9189	5.8033	8.9984	8.6748	5.4276	5.27	10.4601	2.8872	7.1474	1.9039	11.8609	6.7904	11.3792	8.2371	8.0155	4.3998	13.34	13.152	6.5996	7.3141	9.8027	13.3756	9.2215	10.5692	6.1575	9.5831	3.0553	7.1287	8.5074	9.097	8.1837
TCGA-55-6985-01	1233	0	5.8321	7.7544	1.9946	10.8988	9.5895	9.7606	1.6955	7.892	9.5373	8.7182	8.5955	8.0468	6.1222	6.9117	10.0809	7.8601	7.5764	6.3899	11.4712	2.2422	6.3766	2.6378	8.8892	5.6296	10.8755	9.9191	8.269	5.7344	11.3075	12.1265	7.0563	8.8336	11.251	12.4942	9.2977	10.7905	6.1827	10.5794	5.0154	8.4827	7.3679	8.4149	9.7216
TCGA-55-6986-01	3261	0	5.4313	8.6723	7.8174	11.3733	9.5776	10.104	1.2948	8.3984	7.3708	8.3416	8.7426	7.2004	5.2776	8.3188	9.6948	8.5035	6.1738	5.1452	11.0301	0.788	6.6624	2.4224	9.4394	8.6826	10.4331	8.1108	8.2062	2.4224	12.8021	13.5379	6.3127	6.8191	10.818	13.5678	9.1696	10.8077	6.5771	10.2988	3.8157	8.3893	6.791	8.924	8.5795
TCGA-55-6987-01	2137	0	6.4456	8.4018	9.2487	11.5561	9.1696	11.2098	3.9521	7.9894	9.8365	8.8131	7.9262	8.2087	6.392	2.2881	10.192	8.4951	7.1509	8.184	12.3044	0.4363	6.59	3.5352	9.6066	2.4286	10.5381	8.5092	8.6766	3.3478	10.851	12.5844	5.6863	6.9487	11.078	13.5335	9.1536	10.0411	4.624	10.4005	4.8703	10.2273	7.6089	7.9957	9.4369
TCGA-55-7227-01	952	1	4.0183	8.574	6.241	11.6082	9.9268	10.36	1.9124	8.135	8.7191	9.2782	7.8132	7.654	5.0352	9.9988	10.0401	9.3334	7.1944	6.5764	11.4766	3.5067	6.2009	2.3636	9.007	8.774	10.7404	8.7016	8.0001	1.9124	13.0711	12.0194	7.6786	7.2707	10.321	13.0167	9.2192	10.2105	5.4992	10.6502	3.7484	8.7254	7.2114	8.0685	9.5188
TCGA-55-7281-01	872	0	2.9704	8.4757	0.6506	9.8158	10.36	11.5551	1.0973	8.732	8.7736	8.1096	8.7617	8.0364	3.7587	6.3858	9.7938	8.4571	6.8786	6.822	11.06	1.7133	7.1413	3.8182	8.7898	7.0939	12.0814	9.043	7.6305	3.9832	13.3827	12.3308	7.9592	7.1644	11.194	13.3255	10.0915	10.9404	6.8663	9.9884	5.4507	8.4963	7.5406	8.3107	8.6658
TCGA-55-7283-01	609	0	3.0032	8.5886	2.1727	10.8053	9.3819	9.7858	2.7035	9.4873	6.7513	8.9135	8.3851	7.4548	5.5427	4.7584	10.7226	8.0176	7.7847	6.5567	11.4965	1.0018	6.463	0.5862	8.9505	6.7383	11.3595	9.4569	8.7092	3.9103	12.0806	12.4492	7.8238	7.2844	11.235	13.7341	9.2527	11.2149	6.8616	10.597	4.3254	8.1685	7.091	10.129	6.5114
TCGA-55-7284-01	243	1	3.879	8.3324	9.344	10.4852	9.7699	10.0486	2.0415	8.2536	7.0982	8.8977	9.9087	7.8023	5.8666	9.213	9.8185	8.6901	5.8746	7.2596	11.319	3.3274	8.6659	4.8605	12.2725	5.1152	10.2507	8.5828	7.6517	4.7478	13.5117	11.1579	6.4536	7.5546	10.862	11.9043	9.0914	9.1653	6.5479	10.3724	5.7792	9.473	8.529	8.98	8.6593
TCGA-55-7570-01	824	0	2.9138	7.6034	6.6488	12.0041	9.5913	9.5757	10.9852	7.3483	6.7025	9.4751	6.4618	7.2662	5.7119	3.7225	9.4582	8.5401	7.8525	4.3129	10.1089	1.4557	6.4403	1.6684	8.8312	1.8538	11.0177	7.7449	8.5076	1.8538	8.7297	12.7561	4.7626	6.1616	10.433	13.0784	8.1531	12.1699	8.0596	10.3296	3.3435	8.6088	8.4674	9.5741	5.6755
TCGA-55-7573-01	487	0	6.3122	8.8143	5.0101	9.5188	9.8127	9.4025	2.0713	8.9821	7.8774	8.6048	7.7786	7.8752	6.1427	3.6099	10.826	10.0621	6.681	5.8539	11.627	2.0713	6.7693	2.7234	8.7962	7.3321	11.2107	9.4462	8.2538	2.0713	12.6703	11.369	7.914	7.189	11.178	11.4132	9.944	10.2109	6.5649	10.7455	4.2791	7.9044	6.8616	7.4291	8.5626
TCGA-55-7574-01	995	1	7.4499	9.2728	4.3816	9.7639	9.1391	10.0755	2.5268	8.1835	8.641	8.4879	8.4326	8.6276	5.1985	11.3648	10.8728	9.4875	7.1136	6.3075	11.2877	2.313	6.6124	3.5985	8.8867	2.713	10.028	9.3058	7.9764	3.2827	12.0908	11.9312	7.0869	6.5156	10.389	12.6011	9.241	10.4402	5.5063	10.0776	5.7169	9.1552	7.3897	9.2007	8.8499
TCGA-55-7576-01	670	0	7.4104	7.6851	4.4282	9.2117	9.6612	9.7795	1.883	7.3078	7.3443	8.0575	8.9876	8.382	6.0338	7.435	9.9313	8.2836	8.1699	6.5678	11.2054	1.4387	7.1523	2.8785	9.4943	6.7291	11.2229	9.1913	8.7046	5.6261	11.898	11.5224	7.816	6.9787	11.1	12.8575	9.3946	10.627	6.2298	10.0715	4.3439	8.6944	7.4432	8.6112	9.0401
TCGA-55-7724-01	705	0	3.81	7.5027	2.0022	11.3259	9.7973	10.2532	2.0022	7.7249	5.4013	9.1218	8.9483	6.1526	6.5574	1.5869	10.8794	7.8038	5.5488	7.7377	11.635	3.4621	4.5877	1.0014	7.7033	4.0027	8.279	8.6359	8.3604	4.6466	11.5027	8.9891	4.8608	7.6682	10.416	12.2101	4.8101	9.2413	7.0797	10.9232	5.0028	10.7376	8.0956	5.2882	10.0071
TCGA-55-7725-01	442	0	2.8684	8.9194	1.6326	11.1016	9.899	9.2231	0	8.3881	5.3186	9.386	8.7361	7.602	5.3171	0	9.8291	7.9224	4.5027	7.2649	11.8947	2.379	5.7976	3.0623	8.2802	9.4546	9.118	9.9551	8.3139	2.0536	12.8455	9.6557	6.3369	7.1473	10.54	12.4122	6.4795	10.137	5.5613	11.4323	0	8.1897	6.2795	4.7121	9.1288
TCGA-55-7726-01	652	0	6.4144	7.9156	0	10.5547	9.2351	8.413	0	7.4867	6.4069	8.7704	8.9877	7.4578	5.386	4.4201	8.785	7.7048	4.3528	5.1242	10.7494	0.9163	3.4277	3.7466	9.0446	7.0087	9.7225	8.511	9.0226	3.0177	11.7894	11.1258	7.3686	6.3053	10.103	12.6447	7.2464	9.2093	4.4787	10.4515	3.8389	7.3363	8.0031	5.2242	10.508
TCGA-55-7727-01	119	0	4.6877	8.7525	5.9861	10.6448	9.7691	9.9725	0	7.5623	5.8415	8.9148	8.9474	7.3682	5.633	4.7869	9.6864	7.4763	5.2504	6.1853	11.8303	0.9392	4.1304	1.9078	7.2515	6.8075	9.5106	10.1449	9.1023	4.1304	10.8849	9.7602	6.7348	8.3917	11.119	12.3048	6.3529	8.9093	6.5634	11.6999	3.0599	9.1309	7.5628	6.3034	10.3885
TCGA-55-7728-01	704	0	5.3708	8.9985	8.5962	11.2971	10.013	11.4831	1.5121	8.8169	11.38	8.4495	9.7662	9.1369	4.6904	9.4313	9.8275	10.071	5.8844	8.2842	10.6897	3.4597	8.8696	1.9868	12.156	3.0069	11.2943	9.838	7.0173	4.7109	12.9064	13.1087	5.2911	7.5551	10.71	12.6305	9.8789	10.4924	6.2447	9.411	2.7129	10.9517	7.5206	8.9366	8.4264
TCGA-55-7815-01	773	0	9.1976	7.3158	9.0057	11.1803	9.6286	10.3316	2.3242	7.6493	5.1807	9.3866	8.4207	6.4623	6.8484	4.0902	9.9188	5.7577	3.6079	9.8547	11.3736	2.5874	4.5263	3.81	6.5727	8.1627	7.9098	10.0797	7.6894	2.5874	10.4176	7.6805	5.4291	8.4827	12.374	12.262	3.7031	8.7875	6.6894	11.4038	4.1096	9.2123	6.6467	4.1727	9.4271
TCGA-55-7816-01	468	1	2.8976	8.5745	2.0039	9.8671	9.7459	10.4946	3.1279	8.1041	9.0255	9.0144	8.9405	8.01	5.9459	4.7445	10.247	8.6288	6.718	7.289	12.4434	2.4063	5.8631	3.9254	9.4484	5.4738	9.841	8.7662	8.1585	1.1955	11.4352	11.5977	6.0051	7.4169	10.376	12.1178	8.7155	9.8724	7.6676	10.9978	2.4063	9.4152	7.5387	8.7605	9.5677
TCGA-55-7903-01	567	0	3.1654	9.7161	5.2045	10.457	9.628	9.759	0	8.0235	9.0514	9.0784	7.617	8.4565	5.2239	10.3618	9.7381	9.3415	6.9593	4.9268	11.0861	1.9962	6.9019	2.3179	8.593	6.8216	10.9079	9.5827	8.4296	3.083	12.3305	13.1052	9.2547	6.4213	11.13	13.698	9.4493	10.6919	6.7093	10.3743	1.9962	7.5912	7.538	8.0284	8.1191
TCGA-55-7907-01	343	1	4.5652	7.6466	9.585	10.3629	10.044	10.2246	1.3582	8.1387	9.1053	9.7182	9.3019	7.6744	5.3805	8.1015	8.749	8.6653	6.9266	6.1679	11.4116	2.5087	7.0819	2.0452	9.6566	3.3761	10.6196	9.9608	8.5162	3.3761	13.2759	12.4386	7.5171	8.2197	11.102	13.2524	9.2712	10.8226	3.9869	10.2179	3.4808	8.8081	6.2191	8.8306	7.3123
TCGA-55-7910-01	1040	0	10.772	8.6706	4.5938	9.436	9.7362	9.6868	0.9455	8.4691	7.2443	8.4246	7.7536	8.5581	5.7489	6.2479	11.4805	8.3114	6.4373	6.9842	9.9511	2.2338	8.6739	0	8.7062	1.9175	10.1535	9.2576	8.8521	2.7127	9.5686	12.0722	5.3179	7.7963	10.46	12.4455	9.33	11.7739	5.845	11.6224	2.7127	7.3408	6.5049	9.9646	7.0894
TCGA-55-7911-01	537	0	4.9846	9.6145	8.3374	9.1729	9.7446	10.8799	0.6122	8.0106	9.4991	8.7003	8.5739	8.0429	4.5029	9.8183	10.1053	9.235	7.9704	6.7572	12.057	2.5254	6.0791	2.3865	9.4991	6.9125	10.8641	9.4465	8.784	1.639	11.7371	12.2208	6.2187	7.5515	11.107	13.9916	8.5558	11.271	5.8991	11.2146	5.7369	8.4154	7.7129	7.7907	9.3551
TCGA-55-7913-01	561	1	9.5802	8.4415	8.5225	11.3005	9.6038	9.8948	0	7.8347	7.7999	9.1308	7.809	8.6993	6.31	6.6685	11.6278	8.6721	8.0273	7.3887	9.9377	2.6898	6.6398	0.364	9.3477	5.2267	10.37	7.1833	8.6014	2.4072	11.1437	12.6834	6.4958	6.1087	11.711	13.1893	8.8251	10.8847	7.4551	11.2515	4.6985	8.2248	7.1043	10.154	7.522
TCGA-55-7914-01	187	1	4.2472	7.9476	3.2473	11.0884	9.0721	11.0063	0.9996	7.7745	8.237	9.3302	5.988	7.2281	5.6287	6.7542	10.8743	8.8002	7.5423	4.6861	11.642	1.3215	5.614	3.0868	9.3268	5.9418	10.2075	9.0532	8.9751	1.5845	13.7394	12.4153	6.9997	6.4256	11.797	13.1505	8.2353	10.4715	6.7806	10.7637	3.4588	7.37	7.2472	9.476	8.8018
TCGA-55-7994-01	603	0	4.7011	8.6651	9.5239	13.4303	10.094	10.3245	1.1804	7.9996	9.7513	9.1762	8.4084	7.272	6.6769	9.3096	8.2865	8.2358	7.6101	7.0231	11.9122	0	6.6589	1.9447	10.3023	3.3023	10.2668	8.596	8.9371	2.6007	13.15	12.1893	6.8841	7.0208	11.238	12.8352	8.6031	10.5161	2.6741	10.5308	2.0587	9.1499	5.964	9.1353	8.8566
TCGA-55-7995-01	889	0	5.4779	7.8901	11.8188	11.6672	9.4589	11.0374	1.2717	9.4087	12.0055	8.0786	8.2811	8.9144	6.341	5.5051	10.0436	9.0827	7.5552	7.4856	11.3395	1.0919	9.4635	2.607	9.6432	5.0054	7.9154	10.6714	8.2029	4.3187	9.895	12.8055	5.634	7.2297	11.213	11.7452	9.3266	11.2375	7.7959	10.14	2.7951	9.4745	6.9648	7.816	10.5317
TCGA-55-8085-01	904	0	7.3954	9.4627	9.6918	10.3212	10.118	11.071	2.1633	8.7115	10.0111	9.1125	7.0727	8.2937	5.7796	5.2697	10.4652	9.1885	6.8071	7.3191	11.7243	1.2581	8.9135	5.8682	9.1721	6.9803	11.3586	8.6935	8.5157	2.2712	10.9715	13.1271	7.9865	7.0727	12.13	13.3019	10.1546	11.8894	6.0229	10.0175	4.2285	8.8798	7.0764	8.9826	8.1927
TCGA-55-8087-01	462	0	7.5391	9.4047	0.7639	9.7329	10.065	8.5661	1.4569	9.0041	7.513	7.985	7.971	8.6475	5.6993	1.2607	10.5505	10.2488	4.6203	5.8529	10.6419	1.6296	9.1564	1.0336	8.4557	5.5886	10.5292	8.7574	7.8343	3.646	13.24	11.3259	7.5579	6.6903	11.722	11.2407	9.5326	9.6113	6.1529	10.51	1.2607	7.4754	7.1176	8.9263	8.8111
TCGA-55-8089-01	702	1	9.5329	8.409	5.6458	11.5153	9.5174	10.911	0.8732	9.0539	9.6505	8.6097	8.7486	7.4359	6.4023	3.796	9.2804	7.7575	6.4543	7.8984	13.0151	1.9675	7.3685	2.5825	10.4912	5.3837	10.544	7.6923	7.9396	1.9675	11.2798	11.3981	7.1992	6.916	10.529	12.6457	8.5242	9.4218	4.0705	9.0832	4.0344	10.0358	8.0524	7.6748	8.2564
TCGA-55-8090-01	598	1	2.5741	8.71	2.2377	11.7255	9.6276	10.1635	0.3892	8.9542	8.3031	8.6557	8.8347	8.1118	5.0125	8.1069	9.7566	8.7879	8.1655	5.9255	10.8963	1.9211	6.7734	2.7833	9.8034	5.0125	10.7064	8.4691	8.1469	5.0933	11.9539	13.6008	8.0592	7.4719	10.179	12.6142	8.7396	11.5744	5.3775	9.9356	4.5421	8.2779	7.284	9.307	8.5162
TCGA-55-8091-01	600	0	3.8584	9.0639	9.0669	9.6145	9.7094	10.9563	1.8583	8.4691	9.1675	8.4933	8.9272	8.3695	6.3448	7.7009	10.2183	8.7933	7.1482	6.7153	11.9607	2.2483	6.1375	3.5703	9.0124	7.6587	10.4355	8.2178	8.3613	3.0228	11.8776	13.0664	6.8127	7.0959	10.464	12.1897	9.6614	10.2714	5.9663	10.2727	4.2196	8.7525	7.3359	8.1044	9.0187
TCGA-55-8092-01	154	1	8.5649	9.8167	8.3338	9.8067	10.148	9.0166	0.9182	7.9504	8.9239	9.2757	8.1403	7.8765	5.5353	3.0211	8.087	8.644	6.3684	5.9897	12.3352	2.323	8.843	3.2407	9.7844	5.3112	10.4203	8.4187	8.2413	2.6641	10.6829	11.6801	5.6925	6.0817	10.711	11.8634	9.8017	10.1331	5.9837	10.2573	2.5591	8.3298	7.8461	9.4628	8.4484
TCGA-55-8094-01	541	0	9.1125	8.63	10.991	10.602	11.474	6.986	3.6235	8.8578	6.5782	12.5447	9.0294	7.4218	4.2389	6.677	6.0823	7.9939	5.3031	5.9387	11.4912	3.129	8.3024	9.2389	8.5218	2.3708	11.6481	7.5234	8.1862	0.6745	10.2074	12.6992	6.2155	6.5078	13.538	13.3501	9.5535	9.6497	3.0272	10.869	2.3708	6.1565	9.5795	10.075	10.0329
TCGA-55-8096-01	719	1	3.5364	9.1987	5.713	11.7678	10.206	10.4331	0.9527	8.9174	9.9299	8.3907	8.7296	7.7561	6.1016	5.597	9.8946	9.0204	5.7727	7.3582	11.104	2.7254	7.7435	3.8798	9.163	7.1619	11.5627	8.6844	7.5289	3.5747	12.0235	12.8884	7.0645	6.2801	11.477	11.719	10.6787	11.0772	5.2402	11.1558	5.0222	9.1716	7.399	9.7183	10.3215
TCGA-55-8097-01	476	0	5.9876	9.6497	5.96	8.9836	9.9793	10.667	0.4857	8.9018	6.2794	7.9425	8.3736	8.0619	4.7981	2.2025	10.2307	10.5167	5.5666	6.121	10.4555	1.9269	8.5748	1.3792	8.8856	5.1791	11.4313	8.9111	7.5137	1.9269	12.8286	11.422	7.3814	5.9413	10.989	11.9491	9.9185	10.4787	6.1263	9.9586	3.6099	8.349	6.8416	9.989	7.885
TCGA-55-8203-01	547	0	7.7092	8.8238	5.5453	10.3111	9.393	10.2748	1.1461	8.8522	8.7961	8.4842	7.9641	8.1106	5.7543	5.9557	9.107	8.5291	8.4371	6.1503	10.9282	0.855	7.3466	3.2468	8.91	4.4346	10.2936	9.1548	8.2378	1.1461	10.3121	12.243	6.4914	7.4995	10.973	13.0709	9.5444	10.8514	4.3224	10.4156	3.2468	8.3095	7.1346	9.2886	7.9734
TCGA-55-8204-01	515	0	2.6701	7.1883	5.439	10.4341	9.5087	9.2537	0	7.1896	7.1587	9.1782	10.3665	7.0838	6.2191	3.8492	9.4121	8.4654	6.4098	4.6319	11.1239	1.8806	6.0658	1.6535	10.1099	3.7945	10.8089	9.1769	8.1208	4.1833	10.7418	12.116	6.1554	7.7268	10.429	12.8418	7.9149	9.483	5.814	11.2867	5.3658	8.2408	8.0081	8.7631	11.0463
TCGA-55-8205-01	599	0	6.5008	8.8852	6.2777	11.0898	9.2328	10.3496	0.7074	7.4198	10.5412	9.0144	8.7088	8.2352	7.3822	7.3574	9.1419	8.1102	8.4204	7.6289	12.2945	2.1639	6.248	2.2623	9.0502	2.3545	10.117	9.0346	9.7136	2.2623	11.8093	11.6857	5.8171	8.6441	10.577	13.1828	8.6395	9.192	4.4931	10.4943	4.9424	9.6536	7.3795	8.1685	9.2456
TCGA-55-8206-01	888	0	8.8729	8.5896	6.2256	10.3785	9.7174	10.3442	2.659	9.4703	7.1974	8.5435	7.8922	8.0818	5.8506	4.3148	10.3963	10.081	6.9973	7.5672	10.7147	2.2748	9.0147	3.6459	8.2367	8.0532	10.8044	9.0641	8.1923	4.9323	11.7811	11.1889	7.1664	6.8117	11.097	11.1831	9.6394	9.7499	6.6472	10.579	4.2052	8.9211	7.35	8.6202	9.0114
TCGA-55-8207-01	977	0	5.4668	9.4974	1.2945	9.8703	9.8568	9.4291	1.4937	8.4454	8.4042	8.3117	8.2092	7.427	5.5019	7.5127	9.8358	9.1934	7.2127	6.4381	10.6877	1.8248	7.3488	1.8248	8.462	6.9173	10.496	9.6987	8.0324	4.9271	13.9466	11.8622	7.4391	6.5091	11.118	12.623	9.8455	10.9842	6.6722	10.4097	4.0229	8.6061	7.0577	8.6537	8.9267
TCGA-55-8208-01	674	0	3.7963	8.5779	6.8772	10.7073	9.4487	11.594	1.6149	7.6823	10.5503	8.7241	8.3207	7.8615	5.7163	4.3278	9.8838	8.4561	7.4949	8.0889	12.2865	1.3489	6.044	4.0419	9.0931	5.7303	10.6263	9.4109	8.2564	3.7417	11.5257	11.9151	5.8383	7.6955	11.694	12.6177	8.603	9.4803	7.5781	10.1141	7.1839	10.3934	7.7826	7.5483	8.0445
TCGA-55-8299-01	469	1	6.6536	8.5948	3.5827	11.017	9.6425	12.0278	1.4273	7.9683	8.7463	8.7069	8.3719	8.0816	5.0115	3.1739	10.0265	8.8747	7.0484	7.0381	12.9033	2.2634	6.5005	4.228	9.4451	3.9803	10.7149	8.0905	8.4766	2.6984	11.865	10.5583	6.6776	7.039	10.724	12.9177	8.5402	10.4619	5.1206	10.0177	4.3224	9.8363	8.4783	7.6555	8.655
TCGA-55-8301-01	534	0	3.2634	9.0994	6.1779	11.3264	10.07	10.5193	1.8402	8.048	10.4488	9.466	9.2869	8.2872	5.5286	6.5728	9.7738	8.6598	8.1054	7.0831	13.0914	0.5161	6.6114	3.0552	9.352	4.407	11.0026	8.9531	8.3148	2.8118	11.2328	11.8839	7.7724	6.3851	10.6	12.8032	9.4368	10.6894	6.0619	10.1377	5.2798	10.0975	7.4421	9.797	8.4072
TCGA-55-8302-01	478	0	3.3805	9.2635	7.0531	11.3635	10.349	10.1599	1.9141	8.5772	9.2728	8.6725	8.3949	8.6725	4.9497	11.3698	9.1499	9.2726	7.1282	5.9046	12.3426	2.2858	5.699	1.4122	10.1015	5.3531	11.7208	9.9179	8.4979	3.837	12.548	12.7724	8.3043	7.1005	10.733	13.2326	10.1	11.4208	4.633	10.0758	4.1386	8.1213	7.7651	9.0764	7.7051
TCGA-55-8505-01	440	0	7.4882	8.7639	11.6128	11.8517	9.69	11.2902	3.4618	8.5232	8.09	8.3604	8.9881	8.1932	5.2526	7.9154	9.2067	8.8821	6.6827	6.1345	10.3942	1.9805	9.4988	3.2094	9.7613	4.177	10.6824	8.4579	8.3961	5.6915	12.3189	13.1793	7.6536	6.6774	11.072	12.7764	9.3201	11.098	5.5715	10.4457	4.8721	7.7396	7.8432	10.7828	6.0533
TCGA-55-8506-01	11	0	9.6459	7.7926	7.7019	10.3127	9.5043	11.4702	1.9318	7.748	8.9052	7.6421	9.511	7.9255	5.296	7.2363	9.656	8.6567	7.7129	5.3554	11.1072	1.0882	6.7546	3.3232	8.4609	5.0485	10.9224	7.4277	8.1509	2.3049	11.4722	13.1574	7.4418	6.6534	11.395	13.3248	10.7744	11.3147	2.3049	11.4234	3.4022	7.4039	6.5873	9.331	9.6878
TCGA-55-8507-01	418	0	6.8944	8.8194	4.7535	9.8688	9.656	10.6311	1.3914	9.3815	7.9546	8.4243	8.1023	8.3663	4.8378	4.6944	10.6966	8.9356	7.6112	6.3644	10.5455	0	9.0207	3.0062	8.6949	5.1106	10.9047	9.8461	8.6195	3.4959	11.8291	12.7981	7.5646	7.2932	11.742	12.9609	9.7054	10.0065	6.505	10.0826	3.425	8.5975	6.8272	11.0118	8.1051
TCGA-55-8508-01	617	0	7.8946	8.5677	5.6836	10.3451	9.0801	10.1823	0	9.9443	8.6629	7.8892	7.4454	8.5439	5.3184	4.2904	9.9441	8.4631	7.5295	5.4164	10.8669	0.914	6.6443	2.1816	8.6962	4.0802	10.6403	8.8835	8.0325	2.4385	12.0092	13.0598	5.4985	7.0621	11.018	12.8737	9.2447	10.7599	5.3184	10.5125	3.2989	7.9626	6.8012	9.5522	7.748
TCGA-55-8510-01	539	0	4.4468	8.8608	6.7232	9.9584	9.7801	11.198	2.7022	8.8119	9.3466	7.9873	8.0352	8.6615	5.1897	7.064	10.3378	9.3314	7.7136	7.7197	11.3481	0.6889	6.9895	3.6585	8.8871	2.9508	10.0274	9.0887	8.4505	2.5597	12.2663	12.3649	6.3752	8.0816	11.429	13.0383	10.0962	11.2601	5.973	10.9771	5.3924	9.8207	7.1842	9.3477	7.7699
TCGA-55-8511-01	552	0	6.9317	9.1003	4.729	10.17	9.3972	11.1588	1.0607	8.0257	9.072	8.6363	7.3373	8.1436	7.0546	8.1601	8.748	9.2659	7.4769	7.588	11.1609	1.3945	6.3936	3.6325	9.6886	5.438	11.3362	9.1116	8.2192	2.9099	12.0718	13.4945	6.0713	6.7027	11.556	12.3787	9.9604	11.1168	5.2449	8.0997	4.8425	8.2478	7.3708	9.9861	9.0888
TCGA-55-8512-01	607	1	7.2196	9.7245	7.8473	10.4141	10.279	12.1631	2.2453	8.7863	5.3883	8.5389	8.4291	9.2214	6.6951	3.6116	10.2408	10.2286	5.4449	5.7359	10.9761	2.6656	9.1239	2.3994	10.0342	5.4158	10.0401	9.4032	7.8706	6.5045	11.8349	11.305	5.7526	8.5368	10.976	11.4107	9.7808	10.413	6.2344	10.2903	5.0933	8.3367	7.9413	8.7524	8.5938
TCGA-55-8513-01	791	0	3.6859	9.3867	10.0594	10.9568	10.011	11.32	1.3125	8.4185	7.7945	8.8438	8.7045	8.806	5.861	6.9298	10.3848	9.7717	6.1038	8.2844	11.7784	1.3125	8.2983	2.6306	10.5904	5.685	10.4733	9.6303	7.5254	5.861	12.9294	11.1165	6.2744	8.1998	10.646	11.9206	9.1529	9.3146	6.0825	10.3343	4.9036	11.0349	7.7156	8.7475	8.777
TCGA-55-8514-01	520	0	5.8524	9.2094	3.9254	10.5038	9.8267	10.2129	2.0792	8.7213	8.5525	8.4855	7.5203	8.3523	3.9854	4.3008	10.7162	10.4295	8.063	5.7307	10.5548	0.7183	6.2931	1.5536	9.918	0.7183	12.2764	9.1352	8.1509	2.7669	12.3133	13.8848	7.3764	7.7581	12.154	13.1106	11.2533	10.9149	4.3008	9.3386	2.2842	7.8005	6.2812	10.0884	8.2942
TCGA-55-8614-01	536	0	3.7626	8.4739	2.4331	9.6819	9.9675	9.6267	0.7036	8.7125	7.6901	8.793	9.0575	8.6445	4.9029	2.9846	9.9899	9.8257	6.7719	4.5809	10.4905	1.8133	7.6413	3.2929	8.8764	7.4626	11.3674	8.7641	7.9361	3.5468	11.6806	12.9197	6.4134	6.3369	10.773	12.2361	9.8845	10.7389	5.5317	10.7116	2.5979	7.5763	6.8506	9.5825	10.4657
TCGA-55-8615-01	446	0	9.094	8.7041	8.347	10.9099	9.6629	8.6966	1.3809	9.305	6.9978	8.0566	8.9201	9.4544	5.6341	10.5574	12.5205	9.5252	7.3284	6.3454	9.3811	0	9.029	1.0493	9.44	1.8773	10.5455	10.0291	7.8108	2.0733	11.1095	13.5206	7.2256	8.2336	11.593	13.7081	10.0749	11.3835	5.3039	11.5102	5.2028	8.6034	7.1784	10.7515	9.051
TCGA-55-8616-01	48	0	5.6241	8.1608	2.6443	10.4914	10.196	10.1221	3.0623	8.7032	6.6046	9.1248	8.0958	9.068	5.6241	7.9715	10.0331	9.2949	6.7061	4.9661	10.0709	1.6326	9.3586	1.6326	8.8862	4.0207	11.938	9.7928	8.1422	4.0207	12.6389	13.0981	8.4039	7.4148	11.31	13.0207	10.1201	10.6488	6.2989	10.9386	4.1134	6.0699	6.2697	11.613	9.9474
TCGA-55-8619-01	416	0	4.0639	8.6416	6.8691	11.5831	9.8565	11.6326	1.7353	8.5092	9.0587	8.8544	9.1797	8.5902	5.6076	5.8306	10.3716	9.5017	6.7158	7.5448	11.2431	1.9679	7.6026	3.2834	11.3197	3.7258	10.9021	8.8598	7.5498	4.4573	12.9368	12.3777	6.8401	7.3633	10.821	12.1618	9.2925	9.1175	5.9305	10.0864	5.3016	9.7473	7.9368	9.0777	8.8325
TCGA-55-8620-01	375	1	7.2779	8.2815	9.3174	11.5155	9.8708	9.993	4.9151	8.5702	8.9728	9.185	8.3035	9.2957	6.388	4.7965	9.0905	8.4822	8.4042	6.0519	10.7059	0	5.2217	1.9915	9.6661	1.4782	10.5166	8.3384	8.8473	5.267	11.221	13.8808	4.598	6.5434	11.174	12.7334	9.3281	10.6956	7.1015	11.1581	2.5267	7.869	8.4334	6.1548	10.6652
TCGA-55-8621-01	515	0	3.3372	8.7681	5.0965	11.2489	9.8733	11.5973	2.893	8.8181	9.5273	8.6384	8.2766	8.734	5.7122	7.0116	11.0078	9.4927	7.5475	7.3805	12.0026	1.3824	7.7026	5.7981	9.3928	4.8773	10.4987	9.13	8.1187	4.264	12.301	11.4989	6.8865	7.3468	10.778	11.9976	9.0564	10.1342	5.6052	10.0774	5.9558	9.235	7.5787	9.0107	9.196
TCGA-55-A48X-01	689	0	6.9843	8.898	5.2534	10.208	9.9819	10.2492	2.2958	8.6803	8.1601	8.5238	8.4771	8.5616	5.3942	4.4281	10.2317	9.3966	6.5735	6.0976	11.0958	0	6.2805	2.4326	8.9559	5.9789	11.4767	10.2744	7.9244	3.5549	13.0142	12.08	6.7157	8.6021	11.503	11.767	9.6128	10.2976	5.4274	10.1328	3.3426	7.9785	7.402	8.5912	8.8096
TCGA-55-A48Y-01	630	0	5.8511	8.8652	10.8927	7.9224	9.925	10.7946	0	8.1174	7.1831	8.7885	7.7536	7.4059	5.0912	6.9773	10.2925	7.7431	6.4913	4.8308	11.2415	2.0536	7.2848	3.5885	8.829	4.494	11.04	8.9702	8.0791	2.6443	11.0204	12.1041	4.1112	7.1311	10.711	13.4854	9.1794	11.2688	5.6241	10.7835	5.4616	8.7074	7.2049	10.6962	7.3184
TCGA-55-A48Z-01	651	0	3.2705	8.9057	3.5912	11.0919	9.6072	10.1704	0.9717	8.6412	10.4303	8.7405	8.4898	9.0474	5.248	6.9876	9.7461	9.3722	6.0848	6.3866	10.6161	1.7667	4.6454	2.8574	8.9872	7.256	10.8489	9.657	7.6225	3.1968	12.3828	13.0236	7.274	6.7698	11.075	12.8448	9.6639	10.4073	5.7382	10.4916	4.1937	8.3838	7.4744	8.7946	9.4771
TCGA-55-A490-01	99	1	8.1839	7.0802	5.6497	8.8487	9.33	8.9061	0	7.4097	8.2091	9.1679	8.1716	7.6773	7.6171	11.7781	10.4223	8.4505	5.378	4.6518	10.6111	0	5.2811	3.1493	8.6633	0.5772	10.3055	8.912	8.5082	2.5656	13.2518	14.4131	7.9311	5.1869	11.77	13.6185	8.7842	11.491	3.2272	9.4424	3.4383	6.308	6.2345	8.7842	8.31
TCGA-55-A491-01	626	0	7.8065	8.0069	9.4746	10.1784	9.8447	10.5792	1.3719	8.308	10.3713	8.6969	8.8896	7.2567	4.6336	5.3471	9.5085	8.6252	7.7544	6.0832	10.732	0.613	4.6025	3.884	9.1321	5.2299	11.418	10.3153	7.7685	3.2434	12.8294	14.1982	7.3673	7.325	11.956	13.3543	9.1934	10.4639	6.1473	10.4316	11.7124	8.6728	7.9022	9.3883	8.0009

TCGA-55-A492-01	596	0	7.7281	10.0599	11.1694	10.0098	11.193	8.4999	1.6519	10.347	6.4681	8.1623	7.6762	8.474	1.0505	5.8117	10.1436	10.2847	8.0337	6.2267	11.3622	0	10.5986	1.3823	8.6498	6.6139	11.3495	8.2461	7.9349	2.9933	10.8703	13.4183	6.6374	8.2487	11.209	14.02	11.2928	12.1729	5.8117	9.5534	5.589	7.7245	7.5166	9.4633	6.6451
TCGA-55-A493-01	28	0	2.2863	8.483	5.0228	12.4163	9.7684	9.797	0.9778	7.1138	9.3546	9.3467	8.9227	8.5253	5.789	9.9505	9.4612	8.7153	6.0251	6.672	11.917	2.7692	5.1064	4.7119	9.619	2.5478	10.9258	8.4067	9.3638	2.6628	12.5456	12.0477	7.9471	6.3135	10.492	12.9951	9.5167	10.7835	3.6593	10.0949	4.6296	7.548	6.67	9.2796	9.6278
TCGA-55-A494-01	481	0	8.1267	9.2544	4.5344	10.4758	9.374	7.6542	0.5296	9.3275	5.701	9.1102	9.2649	8.4603	5.5851	8.2868	10.4618	9.5961	6.8206	2.883	10.0486	2.0371	8.8159	0.5296	9.2957	4.7399	9.9336	9.323	8.7916	0	12.4759	13.0286	6.8889	7.8909	12.221	13.6524	9.518	10.5016	4.9614	10.7728	2.7581	3.5953	6.3287	11.314	7.4535
TCGA-55-A4DF-01	614	1	3.5402	8.1353	6.1174	11.5857	10.124	10.0324	1.2552	8.2229	10.1907	8.6962	7.6981	8.4187	5.9972	4.9129	9.666	9.5789	7.0195	7.0912	12.2444	2.0828	8.2988	2.6054	8.8928	6.8652	10.1087	10.4692	8.3791	4.9349	9.7065	11.44	6.6694	7.8902	11.085	12.2462	7.4838	9.0978	6.6889	11.2647	10.0589	9.4234	7.6524	7.3994	11.53
TCGA-55-A4DG-01	608	0	3.6557	9.7325	7.394	11.8477	10.64	8.7894	1.3687	8.8021	7.9065	9.0253	8.3987	7.9624	4.9814	5.857	10.5215	10.0158	6.513	6.0695	10.255	1.3687	9.9741	2.3839	8.3987	8.3389	11.0586	9.9396	7.9746	3.5296	11.9146	11.1408	7.1318	6.0983	10.97	12.5848	10.0952	10.1035	4.3952	11.926	2.0582	8.7135	6.3799	10.4704	11.6795
TCGA-55-A57B-01	546	0	4.192	9.6945	6.7242	9.1921	10.202	9.4897	1.9339	9.381	7.5687	8.2903	8.2441	8.4945	5.2488	6.512	10.3525	9.9591	7.739	6.3209	11.1454	1.7942	7.7222	2.2865	9.4149	8.4355	10.685	9.0107	7.4145	3.8479	12.2792	11.0797	5.6237	7.0578	10.757	12.1075	9.3954	10.0309	6.0105	10.0725	6.5613	7.5236	6.5774	7.9736	8.9918
TCGA-62-8394-01	139	1	1.8053	8.1923	7.1704	10.5501	9.9353	9.8739	1.4133	8.7364	8.8394	8.032	8.4073	8.9404	4.8301	8.3984	9.5924	8.4125	6.3047	5.3498	11.2546	1.4133	6.2841	2.9361	8.344	5.918	10.9281	9.4968	8.1779	1.6226	12.143	13.1961	6.7862	6.7587	12.173	12.1168	9.3864	10.3365	5.4675	11.4845	3.9593	7.6542	7.5298	9.0965	10.8303
TCGA-62-8395-01	1216	0	5.6348	9.011	9.7219	9.0746	10.584	10.3518	1.675	9.3242	6.8727	8.5462	8.104	8.5394	6.0329	6.7196	10.8108	9.2207	7.4602	4.531	11.358	0.5247	9.226	3.0032	9.1766	6.5272	11.2045	9.4288	8.1845	2.3067	13.2426	12.4274	8.5292	6.7015	10.799	12.1754	9.5761	10.6648	6.8786	11.1681	5.6348	8.3332	6.5942	9.5255	9.2501
TCGA-62-8397-01	1289	0	3.4879	8.699	8.8251	10.2124	10.59	10.2901	1.9707	10.055	6.0708	8.5497	9.0603	8.3317	5.5764	8.6316	9.9902	9.5193	6.2093	5.4355	10.5538	1.9707	8.3185	1.2986	8.839	8.8482	11.68	7.2557	7.7126	2.7739	13.5429	13.9888	7.1559	4.9844	12.387	11.7718	10.2212	11.6775	6.6686	10.8099	2.7739	6.8254	7.2419	8.7192	7.8739
TCGA-62-8398-01	444	1	10.952	8.6995	8.9021	11.6927	9.8516	10.2691	4.0012	7.567	9.3603	8.4881	9.5704	7.3665	6.2774	3.8592	9.5248	8.5801	6.37	5.4868	11.3259	0.4597	8.7047	2.6449	11.4917	7.0847	10.9047	9.2707	8.0933	2.4605	10.348	13.4751	6.9111	6.7189	10.53	13.3027	8.6274	11.4925	3.7825	10.233	4.5558	8.4233	8.817	8.6465	7.4586
TCGA-62-8399-01	2696	0	4.2015	8.5407	1.094	10.2333	9.7153	9.8223	0.8126	8.0662	7.4683	7.9496	8.1703	9.5928	6.9221	6.6287	8.1531	8.8566	6.3756	5.756	10.4598	2.4695	5.374	4.2596	8.8148	2.3675	12.208	9.4735	8.6882	3.3862	11.3916	12.1225	6.4777	4.6772	10.996	11.4991	9.3121	10.9041	3.333	11.2006	5.6045	8.1778	5.916	9.6745	9.6772
TCGA-62-8402-01	1498	1	4.7608	8.9914	8.7084	12.059	10.07	9.4625	0.5779	7.895	10.0442	9.1449	9.2549	7.8857	5.2272	7.222	9.8788	9.6235	6.6863	9.3811	12.3027	0.5779	5.3012	1.984	9.6008	6.8918	11.1521	10.573	7.8148	3.3028	8.7403	12.8244	6.3627	7.0821	12.307	12.7726	8.8398	10.082	3.7352	11.9661	10.7469	8.3297	7.2503	9.2825	11.0272
TCGA-62-A46O-01	1454	1	9.8657	8.1786	11.0882	10.5725	9.796	7.7671	1.418	7.7037	4.456	9.5543	8.3187	7.3506	7.911	2.7168	9.6624	8.6995	5.9749	7.4867	11.6121	1.6914	3.9007	4.8234	8.4441	5.1062	10.4769	7.5266	8.6721	0	11.039	14.1734	5.0344	5.261	11.283	12.65	8.7619	10.449	6.9103	10.3496	1.9211	7.1669	7.7335	8.3486	9.118
TCGA-62-A46P-01	594	1	8.3808	9.3819	3.7847	9.0731	10.599	10.3816	0.8089	10.01	8.3574	8.3808	7.4236	8.7135	2.0027	5.5504	9.7911	9.0363	7.4913	4.9847	10.64	1.3241	8.2155	2.2508	9.5198	5.4044	11.6368	7.176	6.8954	0	12.4423	13.3676	5.213	7.3851	12.587	13.7876	11.0229	12.4573	4.4128	9.9909	6.2081	8.463	7.3589	10.7264	6.2797
TCGA-62-A46R-01	1725	1	6.5856	9.2767	2.701	9.915	9.9707	8.8539	1.3223	7.9501	6.8739	9.5514	7.4269	8.0505	4.5242	10.2592	10.2222	9.4004	6.0319	7.1478	11.7902	0.5852	6.7555	3.6444	8.3427	2.46	10.8559	8.5686	8.6208	2.0005	12.6531	11.6641	5.9195	8.178	11.51	13.0765	9.3315	10.1408	3.9548	10.4616	2.9074	7.8679	7.1805	9.0613	6.5705
TCGA-62-A46S-01	1653	1	9.9647	9.5037	10.5892	10.9585	9.9981	10.4121	1.4678	8.8184	8.2028	9.8868	8.2598	8.3189	5.0734	4.8487	9.47	8.9932	5.5881	6.168	10.914	0.913	7.3214	0	9.12	7.166	10.9415	7.6383	7.9936	2.437	12.5993	12.4278	7.7039	7.6637	11.304	12.852	10.1837	11.1246	3.6414	10.1409	5.2174	8.5489	7.3723	10.8748	6.9304
TCGA-62-A46U-01	2067	0	3.6448	9.1544	9.508	11.4979	9.5234	11.6959	1.159	8.0473	11.2515	8.7249	9.4616	8.2495	6.5841	10.8354	9.2866	8.7221	6.8034	9.148	12.712	2.7558	7.71	2.9215	9.1843	6.1218	10.1221	9.6215	8.0823	0.8655	12.7808	11.7452	8.8852	7.0635	11.942	12.8436	8.8915	10.2038	6.5903	10.9632	0	10.1146	7.4636	8.6619	10.3262
TCGA-62-A46V-01	2199	0	5.4157	9.4599	8.5704	9.388	10.234	11.3645	4.2377	10.259	8.4166	8.5438	6.0118	8.1717	4.5133	9.5128	9.9485	10.4139	5.941	5.147	11.293	2.1615	8.7739	4.4816	9.0899	7.2325	10.7578	9.1815	8.2133	2.1615	12.8991	13.0843	7.9376	7.4446	11.728	13.0936	9.4197	10.1735	6.3381	9.6696	3.3826	7.7104	7.8348	8.615	8.1339
TCGA-62-A46Y-01	414	1	3.9103	9.1082	5.2323	11.3094	10.225	11.176	1.0018	8.907	8.5873	8.5311	8.4045	8.1303	4.7848	8.8878	9.4682	9.3568	7.5269	7.1988	11.4186	1.5874	7.5193	3.0032	9.0977	3.1731	11.2372	9.071	8.676	3.2512	11.8924	12.2541	5.9577	8.1406	11.194	13.3091	9.563	11.0001	5.8862	10.8931	4.0909	9.0964	7.2372	8.5961	9.4834
TCGA-62-A470-01	1194	1	8.0806	8.8505	10.4372	9.0277	10.183	10.3703	4.534	8.9369	6.9655	9.0277	7.193	7.4381	3.4399	4.6088	10.1566	9.6443	6.3824	6.913	10.6434	0	9.0345	4.1891	8.8233	3.595	10.63	8.7301	8.0541	1.7921	11.3704	11.9219	6.5062	8.2419	12.061	13.3967	9.8963	10.7096	3.9799	10.56	3.4399	9.7819	7.1561	9.4163	9.0446
TCGA-62-A471-01	1246	0	9.3366	8.4357	11.9128	11.1492	10.206	10.0015	7.1874	8.3018	6.7452	8.6838	8.9276	8.3547	4.6462	8.5719	9.4013	8.9738	6.4848	6.9624	11.1449	2.3483	9.5256	2.4864	9.791	4.813	11.6578	9.1964	8.2901	3.0288	11.1921	13.9955	7.4378	5.9149	10.507	13.6314	8.5504	11.1351	5.602	10.8873	4.8646	6.9395	8.3748	10.2502	7.077
TCGA-62-A472-01	910	0	4.6514	9.6443	8.262	10.7547	9.7882	10.1981	0.9747	8.5945	8.3835	9.9003	8.865	8.8945	5.5647	8.9714	10.0391	9.1755	7.4344	5.9915	11.3674	1.5511	6.5041	1.9619	9.0016	6.5934	10.4268	8.8164	8.7403	1.5511	12.9144	11.9297	7.6217	7.2067	9.4998	13.14	8.618	10.89	6.1591	9.9921	3.5383	9.7756	6.4573	8.6599	8.6251
TCGA-64-1676-01	1728	0	3.2195	8.6215	2.7099	9.7772	10.984	10.537	0	8.7702	8.1294	9.2803	7.6125	8.6316	4.0217	8.9473	9.1788	8.2035	5.8208	6.9741	11.5409	1.9151	8.827	4.4453	8.9392	4.1399	11.2882	9.0184	8.508	3.2195	10.5296	13.6805	8.4831	8.061	11.843	14.4805	11.2245	11.9386	3.5952	10.131	2.3666	8.8445	9.517	8.6316	9.1081
TCGA-64-1677-01	628	1	5.5531	9.1629	4.2928	10.43	9.6194	9.2406	0	7.612	8.1615	9.2639	7.8365	8.5888	4.7095	4.88	8.2114	8.3581	7.6815	6.1386	11.067	0	5.5863	1.6727	8.1846	6.9488	10.7897	10.1781	9.209	4.7686	10.9641	13.3138	6.194	7.106	12.819	14.4534	9.7949	10.8003	4.4454	10.4949	2.9193	7.7873	8.1236	8.1572	9.9305
TCGA-64-1678-01	1189	0	8.9851	8.6672	4.4945	8.6199	10.277	7.1253	0	9.7882	4.8069	8.6199	7.7196	7.2983	5.0781	3.8188	10.0842	8.2764	9.0166	4.9706	10.9451	0.9535	5.6621	1.9296	8.6061	4.1585	10.0867	10.4212	9.7882	0.9535	9.04	14.3261	8.6233	8.9609	12.61	14.3231	8.1152	10.3639	2.9176	10.8303	3.2371	6.1735	8.4253	7.9282	9.068
TCGA-64-1679-01	2488	0	6.7443	7.1677	2.7035	12.3911	9.5778	10.3681	1.2934	7.1026	9.6685	7.6296	8.1435	6.9814	6.8891	6.7246	9.2962	7.1052	6.8494	6.0849	10.7594	0	4.9491	5.2534	9.1313	1.8527	10.4752	9.7742	8.7537	3.7212	12.441	13.1914	6.3671	7.1821	11.285	12.3424	9.6305	10.7843	5.4289	11.3528	6.4651	7.6339	7.7532	7.9318	9.7756
TCGA-64-1680-01	1126	0	4.299	9.6559	5.995	10.8239	10.237	10.1463	1.3189	8.6006	5.1714	9.6837	9.0584	7.8247	5.7566	7.637	9.4737	8.9004	7.3689	3.2003	10.765	0	6.9665	0.8052	9.1693	6.6603	10.3859	9.2244	9.1897	1.3189	12.4666	12.3048	8.7381	8.3393	11.366	13.8655	9.5288	11.6735	6.9751	10.1577	3.519	7.9865	8.3192	7.5185	7.9608
TCGA-64-1681-01	1167	1	4.9028	8.3564	6.5694	9.428	10.875	9.8743	2.5993	10.043	8.9603	8.2301	8.2712	7.494	4.549	5.5846	9.9621	8.2542	6.2336	6.1294	12.202	0.4451	8.0411	3.0407	8.5772	6.5804	11.9109	9.4989	8.1238	3.9502	12.6943	12.223	8.0071	7.3157	11.439	12.1127	10.1975	11.0475	6.8161	9.6985	4.7942	8.4622	6.5804	8.3388	8.2474
TCGA-64-5774-01	2676	0	9.363	8.1692	5.9933	11.6876	8.9599	8.8574	0.5538	7.8728	8.361	8.9004	8.3621	8.5859	5.8368	3.9106	11.7515	7.8792	8.455	5.7085	11.5081	0	6.83	1.522	9.1478	6.8652	9.9129	8.7564	8.8559	0.953	11.0643	12.8117	7.7566	7.3521	11.226	13.1635	8.9074	10.8842	6.3065	11.5751	5.6744	6.0956	6.4054	9.2577	8.4516
TCGA-64-5775-01	62	1	0.55	8.6791	0.9473	11.004	9.2839	9.8293	0.55	7.3705	7.0779	9.9288	7.621	7.8817	5.9055	11.1026	9.2525	7.1554	6.5568	5.4725	10.8852	1.7314	3.7582	0.55	10.2817	3.8539	9.9357	7.2769	8.0901	0.55	10.4054	12.8952	10.1142	6.064	10.402	13.3694	9.3511	11.1585	2.0871	9.3965	3.6557	8.0452	9.0539	7.1694	9.8856
TCGA-64-5778-01	1305	0	3.1349	9.6802	1.8459	9.3459	10.592	10.4862	2.0404	7.903	9.4793	8.8365	9.2335	8.5006	2.746	9.7392	8.7117	8.692	6.3882	6.8688	11.8543	1.0271	6.6456	3.6342	9.4714	1.0271	11.5622	9.1815	8.1436	0.603	12.0402	12.9831	6.9112	7.6346	12.125	13.783	10.6428	10.9605	6.4793	10.7467	2.5074	9.1893	7.6383	8.1409	9.3227
TCGA-64-5779-01	864	0	5.8446	8.5091	3.8984	8.0427	9.4133	7.5948	0.8626	7.9228	8.0594	9.0105	8.3698	8.2966	5.6931	4.0486	11.3917	8.717	7.2343	5.9674	10.9258	2.3481	6.6323	3.8169	8.8406	4.3674	10.4997	10.3611	8.2852	1.7887	9.3166	12.0692	6.8829	8.7585	11.42	12.3514	7.7941	9.2753	5.2721	10.3109	5.623	7.2439	7.2594	8.4797	9.1142
TCGA-64-5781-01	1559	0	7.7405	7.9725	7.2613	10.0657	9.9838	8.8897	6.2155	8.9377	7.8523	8.9033	6.9522	8.4835	4.7049	7.7917	9.1729	8.3703	6.3142	6.2134	11.0291	1.8369	8.3759	2.6193	9.9239	4.9039	11.2657	6.9892	8.4989	1.193	10.9242	13.4473	5.9413	6.8106	11.637	14.1555	10.6666	11.5568	4.9646	9.4286	2.7629	8.5015	8.3189	8.7863	8.1131
TCGA-64-5815-01	866	0	3.1466	8.0665	7.7876	12.0564	9.3733	10.8541	0.8363	8.2872	9.7507	8.5261	8.5444	7.8231	5.766	10.1941	10.0614	7.3815	6.5648	6.6412	11.1433	0	6.9671	4.6186	9.6093	7.499	10.196	9.1522	8.1487	3.0126	12.9269	13.4549	7.5361	6.1319	11.368	12.8782	8.7132	10.7234	5.6579	10.094	5.7867	8.4471	7.7721	7.8867	8.2264
TCGA-67-3770-01	610	0	2.731	9.6952	8.9551	10.4955	12.04	9.4125	4.0855	10.953	7.3879	9.2549	9.4005	6.3378	4.6145	6.4034	8.7531	9.5599	8.0487	8.1106	11.353	0.9559	7.7053	1.5259	8.4806	7.3119	11.2075	8.8355	8.9551	2.2508	12.3023	12.713	9.125	7.159	12.96	13.439	10.7411	11.2763	6.5408	10.992	3.7397	9.3115	7.8939	8.3454	9.1821
TCGA-67-3771-01	610	0	7.4181	8.263	5.9292	10.3247	9.7355	10.503	3.6698	8.0553	9.7768	8.8341	8.5852	7.9113	6.8041	3.3392	9.7907	8.3889	7.6543	7.0367	11.1966	2.2954	7.8718	3.7418	8.5405	4.6868	10.3708	9.9407	8.3945	4.3606	10.6489	12.693	8.0131	7.5987	11.845	12.4587	8.091	9.996	6.0896	10.1553	10.6103	9.0918	6.9173	7.8798	7.3848
TCGA-67-3772-01	573	0	4.7159	8.3709	6.5925	9.6065	11.067	9.2617	1.9294	10.053	7.9964	9.4317	9.0727	6.5134	6.1731	5.8724	9.3357	8.9428	7.2694	6.6596	11.6822	0	8.3972	4.1955	8.0754	6.7197	11.5224	10.2428	8.3811	2.2465	11.8305	12.3163	6.9227	7.3651	12.369	13.2529	10.4263	11.2265	5.9618	9.8121	4.5867	7.958	9.0007	7.7635	7.6803
TCGA-67-3773-01	427	0	4.3595	9.2218	2.3536	9.0366	10.628	11.6084	1.6114	8.897	8.1901	9.5172	8.2967	7.5784	4.8454	5.2864	8.8912	9.1427	5.2083	6.9246	11.6602	1.0198	6.4653	3.2051	8.9994	7.8337	10.4824	9.5883	8.4274	1.0198	13.9045	12.5518	8.3792	7.7672	12.687	13.6258	9.2043	11.205	5.7667	11.4673	3.9438	10.5931	7.8848	6.4985	8.3294
TCGA-67-3774-01	385	0	8.1847	8.9689	8.0346	9.0871	9.9102	10.1052	2.611	8.5118	7.388	9.1742	8.7053	8.0486	5.8856	8.4865	9.314	9.0424	7.1356	7.0105	11.2621	1.8297	6.7841	2.2726	9.1485	6.7841	10.435	8.6024	7.7692	1.8297	11.9153	11.6198	7.8632	7.599	11.339	11.6885	9.4546	11.1327	5.9164	10.0636	5.175	8.3463	7.4115	9.3596	8.6582
TCGA-67-4679-01	448	0	5.8674	9.5799	1.952	11.1968	9.9757	9.5928	0.6543	9.2195	7.017	8.3273	8.2396	8.8055	5.6037	3.9397	10.4158	9.9023	6.4717	6.5195	11.1954	1.1027	8.4001	2.8702	8.5731	7.7173	10.7299	8.973	7.6488	3.9397	13.4323	11.9745	8.8018	6.7665	11.305	12.1981	10.1743	10.8229	6.4346	11.1167	6.366	8.2203	7.5329	8.4672	9.1066
TCGA-67-6215-01	174	0	7.0083	8.7938	4.5451	13.2389	9.3949	10.9159	1.316	8.2868	7.859	8.3883	8.238	8.0822	6.0236	9.4839	10.2908	9.283	6.3732	6.4323	10.7196	2.1216	7.6048	0.8031	9.1245	5.7216	10.3079	8.8473	8.0802	2.2401	13.3698	12.35	8.9562	6.8636	11.179	13.6831	9.3973	11.1088	5.4901	10.7563	4.2385	10.348	6.2452	9.001	8.9344
TCGA-67-6216-01	141	0	5.4794	9.1133	6.1762	10.7523	9.8144	11.4319	0.6685	8.858	7.9913	8.2615	8.9249	8.0274	5.9195	7.4979	10.0379	9.2975	6.7565	5.875	11.0916	1.4689	7.4156	4.08	10.6831	6.7186	10.5979	9.6537	8.0241	2.7853	12.5013	12.8101	7.3803	7.0871	10.548	12.7054	9.8907	10.9349	6.0014	10.6821	0	8.0307	7.2013	8.5334	8.7365
TCGA-67-6217-01	422	0	5.8544	8.8854	3.5611	7.8534	10.535	8.6216	1.436	9.2718	7.6436	8.2346	8.6382	7.7041	5.7363	3.3359	9.8726	9.7579	5.6541	5.8833	11.33	1.436	7.9855	3.2522	8.0364	7.3986	11.6021	8.8468	7.9227	3.3359	12.4182	11.6406	7.0294	7.0231	11.805	12.188	9.7683	11.1565	5.7363	11.254	2.8588	8.2775	6.3925	8.5943	8.1534
TCGA-69-7760-01	202	0	3.6139	9.2243	6.7854	9.9591	9.0363	8.9551	1.8067	8.1034	6.0567	9.3553	7.5479	6.8648	5.3785	9.1326	9.5178	7.417	5.1688	5.9694	10.4513	2.7272	7.9343	3.189	9.6106	6.211	10.4724	9.9672	8.6667	3.8818	11.2274	12.4067	7.7159	6.2351	11.217	13.5239	9.1612	10.8931	5.2891	10.988	2.8572	7.3074	5.9878	6.7854	6.1865
TCGA-69-7761-01	186	0	3.7455	8.0075	9.2533	10.8256	9.4796	9.8337	1.0561	7.3465	8.7503	9.2836	9.4948	8.0899	6.9358	8.5277	9.6453	8.3998	6.0084	6.2456	12.4289	3.0028	7.2074	3.8571	11.1456	5.3742	9.9492	9.8649	8.1866	4.9137	12.3445	11.2325	6.4217	7.5061	10.512	12.124	9.334	9.1337	5.7946	10.571	1.0561	8.8896	7.3574	8.7841	9.513
TCGA-69-7763-01	690	0	8.7703	8.2093	4.5882	9.5426	9.5608	9.6737	1.7503	8.1002	6.992	8.1323	8.3308	8.0316	5.6959	10.4886	10.3955	8.4256	6.9617	6.141	11.0491	2.9073	6.6051	3.0167	10.4043	4.2723	10.7848	8.5695	8.2741	1.4716	12.2999	11.9552	5.8218	6.7066	10.272	12.9235	8.7186	10.5319	5.3224	10.2363	5.9237	8.1262	7.4784	8.9362	7.3005
TCGA-69-7764-01	414	0	5.2779	9.1879	7.5237	8.6058	10.114	8.3007	0	9.7435	5.9069	7.9867	7.8358	8.1026	5.6459	6.7868	10.0372	9.0965	6.2324	4.4425	11.0232	1.218	7.3875	0	8.6683	6.7066	10.298	10.7453	8.6636	2.6565	12.2362	11.1037	5.3264	5.9697	11.686	12.8364	8.8902	11.1856	6.2084	11.1604	5.1758	6.9033	6.0728	8.0458	9.2536
TCGA-69-7765-01	165	0	5.265	8.3218	2.9757	10.5257	9.664	10.6225	1.5224	8.2492	8.0064	8.7456	8.5695	7.5276	7.0923	6.4245	10.6053	7.2485	6.6474	6.1778	11.3207	1.5224	5.8991	2.4248	8.3414	6.7845	10.4067	9.196	8.8468	4.1582	11.6665	12.234	6.6917	8.5671	11.598	12.7324	8.8546	10.6775	6.2461	11.7421	4.1068	7.9553	7.508	8.0936	9.2796
TCGA-69-7973-01	230	0	8.8243	8.3863	5.8839	12.062	8.9219	9.7683	1.7321	8.1016	6.3018	8.6693	8.1355	7.8234	6.742	5.7658	10.037	7.7509	8.7085	5.2337	10.1939	1.0229	8.3548	2.6822	8.2052	5.7861	9.6163	10.081	8.0283	1.1954	11.2076	12.4836	7.2201	7.6284	10.649	13.1353	9.0809	11.2502	5.4623	11.8689	6.4287	8.0312	7.4246	9.6775	9.3612
TCGA-69-7974-01	184	0	7.8563	8.5239	3.7696	13.1006	9.5295	12.6298	0.912	7.8673	9.3069	8.9203	8.5547	8.106	6.3124	10.4219	8.7904	8.2947	7.1612	7.2325	11.6489	2.2693	6.2909	3.4585	9.3898	4.9256	10.7436	8.0749	8.3228	2.9004	12.4101	12.8758	8.5054	7.9672	10.611	13.2155	8.865	11.4823	5.612	10.4713	5.7988	9.828	6.8349	8.5216	7.6668
TCGA-69-7978-01	134	0	4.675	8.5798	8.9124	11.2958	9.2676	10.7722	1.0063	7.8085	10.0404	8.6719	8.5348	8.4177	6.6515	8.8612	9.8134	8.0696	7.0921	6.9611	11.5029	2.4252	6.3582	3.853	9.4332	4.675	10.0107	9.5335	9.1123	3.853	12.286	12.1232	7.3612	7.1588	11.183	12.4645	9.3876	9.9114	4.427	9.4555	6.0568	9.2793	7.6223	8.7818	8.7618
TCGA-69-7979-01	408	0	5.0897	8.2088	8.009	11.3915	9.7877	8.9129	2.4954	8.2468	7.6414	8.425	8.5991	8.9514	4.5438	6.0112	9.8493	8.6645	7.8566	4.9361	9.9913	1.731	7.0418	1.1109	8.2886	6.9227	11.2164	9.2633	9.0518	3.4407	11.4773	13.6078	7.2998	5.7719	12.09	12.394	9.1537	11.0549	5.274	8.7832	3.7821	6.7701	7.6925	10.8045	7.0763
TCGA-69-7980-01	362	0	4.2296	8.3536	8.0513	10.2056	10.124	10.1833	0.6892	8.5068	8.4501	7.983	8.6991	7.9004	4.7065	6.0492	9.0494	8.0529	6.8795	7.1906	11.2139	2.0222	3.8245	2.4024	8.307	7.173	11.5088	10.8549	7.4148	4.2296	12.295	12.2622	5.9455	7.5693	10.718	11.9957	9.8926	11.0448	6.2997	10.7587	4.1572	8.5205	7.5411	8.234	10.0087
TCGA-69-8253-01	426	0	9.5938	8.6729	11.0173	10.5178	9.7627	9.9767	1.585	8.5877	6.4091	8.5463	8.4076	9.0564	5.0814	10.2521	10.5821	9.081	6.8912	5.9117	11.2803	1.4406	6.1555	0.8931	9.5494	9.6007	11.5281	8.1285	8.1792	1.9475	12.4992	11.9322	5.7201	7.7672	10.988	13.2099	9.5069	11.181	4.8437	10.0403	4.8437	8.4482	6.7376	9.7311	7.2438
TCGA-69-8254-01	409	0	6.2858	8.5404	8.6435	11.745	10.191	8.9836	1.4449	8.6126	7.3552	8.4989	10.0386	8.5222	5.2446	7.7685	9.9419	9.212	5.8158	5.8407	11.2887	1.3083	5.9433	2.07	12.4536	7.7895	10.5931	9.052	7.9373	6.3039	13.0003	11.277	6.1673	7.4692	11.258	12.3149	9.149	9.8247	5.4123	11.1761	3.3014	8.4601	7.4712	8.8046	7.2558
TCGA-69-8255-01	129	0	11.791	8.8016	10.3636	10.6065	10.358	10.0105	9.7135	8.159	9.7725	10.0894	8.1815	7.4322	5.4502	7.2987	8.2813	9.4089	8.5805	8.3882	11.5042	3.0857	7.4934	10.262	10.1015	2.5149	10.8579	7.8806	8.7242	2.5833	10.4353	12.6402	5.2752	5.7838	11.991	13.7565	9.3153	11.9223	7.8039	10.1579	2.8836	8.7013	8.5674	12.4372	7.2311
TCGA-69-8453-01	813	0	4.4241	8.8503	7.0347	11.7227	10.062	11.0217	2.9676	8.6219	9.0716	8.4807	9.3872	8.4081	5.7581	8.7975	9.6969	9.2333	6.9725	8.6623	11.895	2.1412	8.1628	3.2148	11.887	3.1371	11.0705	9.383	7.7247	4.6923	12.3399	12.1303	6.8459	7.3512	10.867	12.7999	9.2973	9.7872	6.072	10.0774	5.5336	9.7998	7.3853	8.364	9.3304
TCGA-69-A59K-01	522	0	2.0268	9.2868	2.7082	10.0246	10.041	9.5767	2.4074	8.5754	7.635	9.558	8.8487	8.2518	6.339	6.2368	9.9449	8.5145	7.2869	6.0036	10.7635	1.5084	7.5448	0	8.8176	6.2717	10.9749	7.6007	9.8471	2.957	11.1243	10.9156	8.7696	8.1893	10.02	12.0805	8.2806	9.5485	5.8164	10.1772	3.7334	7.9909	6.7134	8.6039	7.8557
TCGA-71-6725-01	256	0	2.7261	9.0419	0.3918	8.6065	10.082	10.8655	1.1687	8.2431	5.735	7.9385	6.8044	8.2537	6.4501	7.0985	9.725	9.0444	5.4306	6.0903	10.0537	1.5222	6.3707	0.9532	9.0255	4.3902	11.7648	9.1321	8.4912	5.4917	12.1931	11.5478	6.6694	8.4621	12.025	13.1206	10.5982	12.4094	3.3289	10.2798	3.3731	6.7594	8.0309	8.971	8.9556
TCGA-71-8520-01	210	1	3.6758	8.6626	5.368	10.1919	10.476	9.3583	1.1635	9.1371	8.1549	7.9236	9.8315	8.2009	5.607	8.4742	9.6718	8.231	7.6588	5.6835	11.4631	0	7.9492	3.8718	8.6381	8.4283	11.5723	9.4039	8.4412	4.0976	12.7903	12.8736	8.1887	6.6288	11.801	12.1596	9.9484	10.2642	6.033	10.0946	3.3645	7.2854	6.7249	8.5994	7.9707
TCGA-73-4658-01	1600	1	4.7796	7.9499	6.0321	9.0366	9.4478	11.5284	2.3299	8.1408	8.0562	8.826	8.9753	7.5278	6.2331	9.7317	9.7327	7.4634	6.6086	6.9538	11.3805	0.6557	6.222	4.362	9.4442	5.6577	10.6324	9.4044	8.5217	4.4405	12.3296	12.3979	7.6193	8.4687	11.417	13.5576	10.0257	11.4628	4.8676	11.1439	6.6182	9.0319	7.6853	8.5149	8.6156
TCGA-73-4659-01	711	1	10.317	8.7055	6.016	11.9688	9.4533	12.2026	3.2705	8.2986	7.0428	8.514	8.0801	7.8646	6.6092	3.8582	9.2834	8.5444	8.13	7.2445	10.6998	1.18	7.4534	2.3839	9.1251	2.6972	10.3887	8.8055	8.7157	2.2623	11.9498	12.828	6.4088	6.2579	11.05	13.5507	10.2876	11.3293	5.6177	10.8552	5.7816	10.4738	7.7562	9.2327	8.933
TCGA-73-4662-01	2515	0	4.0213	9.0308	4.1411	10.1883	9.65	8.8165	1.8394	8.2396	7.9432	9.1997	7.9547	8.9429	5.6301	10.6544	10.3759	8.4023	6.1162	7.0897	12.1938	1.4011	7.5206	3.2024	8.3566	7.3235	11.1569	9.9878	7.9574	2.3177	11.1146	11.3976	8.6234	7.2184	12.09	12.3091	9.4672	9.7668	6.8298	10.7641	3.982	8.0394	6.8417	9.1881	8.7885
TCGA-73-4666-01	800	0	1.3272	8.3903	9.425	11.0311	9.6271	10.6772	2.5923	7.6968	9.4652	7.6741	8.1924	8.0559	6.353	7.4784	8.3641	8.1081	6.6724	7.1655	11.8464	1.0044	8.1406	3.3298	9.7632	3.8908	10.0912	9.4646	9.589	2.0066	9.3503	12.0927	7.4763	8.0158	11.405	13.5278	9.3097	11.6151	2.7624	10.1611	3.8908	9.2761	8.0856	7.9156	8.2872
TCGA-73-4668-01	467	0	9.1151	7.8518	1.308	11.3831	9.9171	12.3464	1.157	8.1016	7.085	7.8777	8.9936	6.9253	5.7119	9.2412	10.591	7.8192	7.0612	5.2604	10.3149	1.5695	7.8595	2.8868	9.0682	0.3173	10.7815	10.3266	8.5814	2.5656	11.7983	11.7849	7.1936	7.6721	12.257	13.1143	8.8293	11.4829	2.8868	10.6063	5.6211	5.9138	6.7083	8.5295	10.4751
TCGA-73-4670-01	131	0	10.768	8.4638	10.2736	11.6772	9.4751	10.8496	2.6307	7.3383	8.3581	10.2168	6.1767	7.2259	6.7532	8.2602	9.6079	8.7115	7.5118	7.6792	10.4381	2.271	8.6011	2.7528	9.7529	3.5093	10.6223	6.6071	8.8808	2.1869	9.9017	13.9027	6.0634	6.7678	11.32	13.8709	7.6575	11.4177	3.4015	10.4363	2.9184	7.7149	8.1409	10.0519	5.9846
TCGA-73-4675-01	922	1	4.5232	8.6781	12.2653	8.3582	10.351	10.0785	1.5154	9.427	6.981	8.8282	7.6696	7.7916	5.5404	8.3362	9.8257	9.3436	8.0552	6.0019	10.6123	1.3499	8.0308	3.1284	9.5943	8.4846	10.6872	7.1835	8.6716	2.3298	13.445	13.6307	9.2703	7.8891	9.8028	13.3644	10.134	12.5114	5.9035	7.3996	4.7888	7.3707	7.2408	9.3207	6.515
TCGA-73-4676-01	281	1	1.3691	8.3699	8.7179	10.8289	9.672	10.6101	1.8633	8.1131	6.7911	9.6062	8.1703	7.002	5.922	9.531	9.1717	7.2117	8.2083	6.838	10.7568	2.0587	6.9598	1.6373	8.7781	6.3622	9.9146	9.2786	9.504	2.2308	11.7062	12.8593	8.0831	9.0084	10.544	13.3555	9.348	10.4402	6.5608	10.4741	4.5981	8.2298	6.4863	9.4818	10.3451
TCGA-73-4677-01	38	1	8.2629	8.8737	11.5656	9.7554	9.9056	9.2829	0.6583	8.2212	6.7219	8.5118	7.7899	7.4611	6.7699	8.3473	10.5377	8.6396	6.2267	6.9412	10.8745	1.1087	9.0658	2.822	8.9923	6.4456	10.6736	8.7406	8.1338	2.1601	11.7835	12.0576	5.9134	8.2845	11.61	13.0135	9.5905	10.915	5.1808	10.1491	4.7866	9.3133	7.0186	10.8028	7.0629
TCGA-73-7498-01	1189	0	7.3186	9.5757	6.8029	8.2548	10.364	9.035	2.3472	9.0167	6.6715	7.8689	7.3953	8.8454	5.1433	5.3308	10.9592	10.5664	7.7957	6.6036	10.3919	1.2405	9.863	2.4225	9.361	6.8616	10.795	9.2799	7.9617	2.2678	12.2284	11.9207	6.7564	6.5283	10.6	12.267	9.788	10.5729	5.5055	10.095	5.8503	7.7515	7.9898	10.3343	7.4458
TCGA-73-7499-01	1531	1	4.127	8.9287	9.4334	8.3206	10.505	10.5113	1.8101	8.9042	9.2679	9.6294	8.437	8.1637	5.4228	4.6015	8.8569	8.7154	6.5127	7.5802	11.6512	0.7792	7.3664	4.127	8.8254	6.9013	11.3453	9.2265	8.0936	4.0355	11.4804	13.4085	8.246	8.6175	12.404	13.1821	10.1261	10.62	6.35	11.3785	3.7585	9.5579	8.5111	7.6	10.861
TCGA-73-A9RS-01	340	1	10.001	8.2132	10.855	11.7822	9.3496	11.8751	1.8643	8.1911	5.6315	7.5174	7.3249	8.2218	6.9364	6.0854	7.3563	8.375	7.0493	7.9654	11.1987	0	6.418	1.465	9.1018	2.1767	11.0152	8.8164	8.6174	4.1477	7.9866	13.7407	4.8222	6.4904	11.034	12.4807	9.7381	12.0954	6.1934	10.2581	0.9311	7.4464	7.1302	10.2053	4.6304
TCGA-75-5122-01	0	0	2.3871	8.9592	0.8846	11.1195	9.4856	12.5841	0.8846	7.777	9.7745	8.8965	8.7847	8.211	6.2855	8.9345	9.2352	7.7935	6.8757	8.4308	13.0464	2.1325	6.2855	4.1638	8.8012	2.7915	10.4076	8.9269	7.9299	3.8619	11.2196	11.7359	6.7384	6.4199	11.53	12.8962	9.7062	10.5858	4.0207	10.8083	2.6034	10.9729	7.8528	8.3891	9.6647
TCGA-75-5125-01	2027	1	5.5348	9.0635	3.8989	13.0058	10.064	9.8689	1.3485	6.7435	8.8035	9.8665	9.4858	9.0731	6.243	10.1854	9.3293	7.9264	5.9608	5.756	11.8833	1.0221	6.4097	2.8451	9.1113	2.8451	10.4113	8.3129	9.3635	2.945	11.3034	11.7801	7.5712	6.8117	10.45	13.0387	9.0759	10.5782	4.3271	10.3849	3.1263	8.9311	6.9861	7.3656	8.7425
TCGA-75-5126-01	0	0	3.3997	8.0077	3.2866	11.6482	9.9003	11.628	1.7606	8.2857	8.8244	9.0938	9.3961	7.6726	6.5569	8.1439	9.1933	7.4008	7.3361	8.1435	11.878	1.3743	7.394	0.8449	8.9221	6.4689	10.318	9.1208	9.0337	4.3297	12.7932	12.2728	8.4697	7.273	11.016	13.2245	9.7669	10.1673	5.8261	10.5616	3.8615	10.342	8.0077	7.3803	9.743
TCGA-75-5146-01	2368	0	9.0039	8.3656	1.9392	9.5165	10.775	9.3557	2.1838	9.0292	6.5411	9.5873	8.8034	7.4829	6.5302	2.8833	9.1747	8.771	9.3444	5.554	11.6877	0.7729	8.3469	2.7376	9.1764	7.1857	10.6373	9.1352	8.0167	2.3929	11.4649	12.8936	7.4066	7.5662	11.887	12.8914	9.9754	9.8257	5.9857	10.6086	3.9236	8.0402	8.0518	8.3248	9.8668
TCGA-75-5147-01	1333	0	2.5882	9.0903	8.4819	10.5819	10.14	9.7805	1.1241	8.0341	8.9785	9.5285	8.5974	8.0236	5.6678	5.9483	8.9434	9.0958	7.6933	7.7933	11.7314	0.6688	6.9227	5.6927	9.1774	6.5799	10.6318	10.1268	9.2072	3.501	12.4344	11.7329	7.9307	7.2224	12.095	12.4313	9.2007	9.7586	6.3011	10.4105	4.8878	8.7622	8.4724	8.5863	10.8664
TCGA-75-6203-01	0	0	2.358	8.5775	7.2894	10.6488	10.135	11.2926	2.9778	9.2483	8.1812	8.4443	8.4836	7.4904	5.3292	1.9073	10.5156	8.5801	7.0403	7.6929	11.5045	0	8.2164	0.7551	8.5697	6.786	10.3807	8.4357	7.7749	3.5003	12.7111	11.7658	5.4924	7.0926	11.384	12.3783	8.9981	9.9178	6.5202	10.6476	5.3081	9.1208	8.3256	7.3515	9.2664
TCGA-75-6205-01	0	0	2.1342	8.3912	6.422	9.7963	9.418	10.6425	0	7.6393	9.2408	8.3431	8.4726	7.8811	6.3186	8.5978	9.6639	8.0242	7.046	7.2021	12.0559	1.8247	5.8312	3.6853	9.1236	0.8856	10.7336	8.1671	8.3088	3.4815	11.3524	11.7497	7.2862	5.9146	10.345	13.1581	8.9638	10.0979	5.8525	10.5679	4.1658	9.1081	8.0521	8.7785	9.4215
TCGA-75-6206-01	2590	0	7.9414	8.9104	6.1722	9.5518	10.434	9.5554	2.4344	8.9501	7.8678	7.9153	7.7855	8.1666	5.9985	3.6957	10.552	8.9254	6.2759	6.2942	11.0774	1.5301	8.6619	2.4344	9.0523	6.5664	11.1391	9.2985	8.4627	2.0519	12.4571	11.545	6.8043	6.712	10.7	12.4954	9.4015	10.6428	6.4569	9.8173	3.8919	9.2664	6.9362	10.0311	6.9943

TCGA-75-6207-01	0	0	3.2915	8.2741	4.4369	11.952	8.9476	9.0766	1.8629	7.345	7.4605	8.1254	8.7673	8.0722	7.1552	8.6074	9.7952	7.3031	9.0107	5.2503	10.9675	0.91	7.0102	0.5257	9.4106	4.7747	10.325	9.6504	10.396	3.3548	12.7114	12.398	7.2582	7.1008	10.449	13.4655	9.5183	11.6593	7.1192	9.8696	2.6495	7.5799	7.4662	8.27	8.0627
TCGA-75-6211-01	0	0	8.4462	7.5824	8.3034	7.7011	9.0683	9.6565	3.6061	8.8588	6.8034	9.455	7.064	7.5675	6.3782	2.7902	9.9804	8.1449	8.3271	4.7935	10.5188	2.1001	7.3866	2.1001	8.9696	2.9212	11.3062	9.4125	8.7158	0.729	9.5718	12.8401	5.9422	6.9988	10.967	13.2291	9.2303	10.802	1.5716	10.6632	2.1001	6.9688	7.6839	6.3668	7.8318
TCGA-75-6212-01	1516	1	3.4767	9.0053	6.3028	11.143	10.443	11.019	7.1741	9.4837	9.0197	8.1924	9.1189	7.1585	5.3488	5.8359	10.1838	8.5392	6.1685	6.6497	11.5415	0.8314	8.7935	4.4624	8.1652	7.1819	10.8981	8.328	8.2228	3.5744	13.1889	13.9089	8.0553	6.0205	10.679	13.2023	9.9053	10.7262	6.688	11.3285	6.1841	6.9908	7.0353	8.3033	9.0674
TCGA-75-6214-01	1115	1	10.355	7.8549	6.1773	11.7363	9.5732	8.9067	1.3326	7.6452	7.0747	8.5099	9.7274	8.1607	5.4334	6.3212	8.7908	9.6011	5.1289	4.3102	10.9472	1.6443	3.8443	1.4968	11.1697	5.2499	11.5692	9.854	7.9989	6.2991	10.3596	12.271	6.3537	5.9267	10.678	12.7976	9.3959	9.4964	6.5253	10.5305	1.778	7.718	8.4758	9.7687	9.045
TCGA-75-7025-01	3305	0	4.2308	9.7607	5.1617	8.7529	10.113	9.2217	2.8179	9.6978	7.3196	8.6122	9.0179	8.3582	5.9605	5.2647	10.3049	9.7062	6.1447	6.0201	11.1393	2.4695	9.4804	3.2778	8.7466	7.7837	11.3431	9.7776	7.5504	3.2778	12.4131	11.5784	7.6434	7.7308	11.002	11.5937	10.7034	10.4045	6.3763	10.3514	3.7928	8.0478	6.5259	8.9676	8.5553
TCGA-75-7027-01	3059	0	8.2985	7.6029	10.2263	10.6532	9.0855	9.0474	5.9291	8.1441	7.2997	9.6471	8.5665	7.4199	5.963	5.5013	9.9437	8.5543	6.969	5.9094	10.9804	2.4176	6.6357	2.566	8.7792	6.5176	10.3056	8.1839	7.9438	1.9617	9.1332	12.4816	6.4379	7.1409	10.288	12.9713	8.9186	11.0491	4.9859	10.7862	3.4768	8.0246	8.0166	10.0416	8.4046
TCGA-75-7030-01	0	0	4.0553	8.1672	7.7025	10.5285	9.8435	9.8171	1.0298	8.6082	7.3328	9.0188	9.2948	7.8334	5.5812	8.2236	9.9657	8.5345	7.5521	4.8371	10.971	2.6342	7.6208	4.5157	10.7817	4.5157	10.7471	8.5784	7.853	3.639	12.5181	11.9997	7.688	6.8265	10.49	12.2235	9.0812	9.6362	6.1666	10.0518	3.7549	7.0009	8.2135	8.0244	8.9414
TCGA-75-7031-01	0	0	6.3128	8.3187	10.0704	10.0733	10.624	10.0235	2.4063	7.5629	9.232	9.7805	7.7335	7.748	4.269	1.6556	8.6241	7.8658	6.4882	8.1567	11.7651	2.0792	7.2229	6.924	8.5963	5.3114	10.9618	9.047	7.993	1.0533	10.3286	14.404	7.9869	7.2839	11.206	13.31	9.1434	9.8777	3.4165	11.1808	4.0984	8.4263	7.7262	6.1476	10.0145
TCGA-78-7143-01	4961	1	2.124	9.1827	8.1793	10.5294	10.167	9.2672	1.2866	8.6547	8.5149	8.8401	8.7123	8.305	5.5566	9.0649	10.2296	8.7705	6.5323	6.7591	10.5953	0.9706	6.7741	1.5457	8.5069	8.4105	11.0796	9.8891	8.1356	4.8968	12.5667	11.2817	6.6691	7.977	10.807	11.8838	9.3659	10.5984	5.6533	11.1686	1.7652	7.9577	6.2452	9.94	10.1243
TCGA-78-7145-01	826	1	2.8877	7.8868	5.3215	11.848	10.939	10.4914	0.6617	7.028	7.3306	11.5088	8.5692	7.4702	5.9014	9.078	9.4145	7.1656	6.2335	5.5076	11.7412	0.6617	7.2003	2.343	7.8797	7.4702	11.5152	8.6592	8.0915	2.8877	12.7919	12.0856	7.6356	7.7608	11.06	12.9171	8.1279	9.7913	6.5915	9.6532	2.6409	7.2672	6.7559	6.8822	8.0976
TCGA-78-7146-01	173	1	1.9946	8.5608	8.3504	11.1768	9.4191	10.0829	1.5801	7.1096	8.3372	9.5036	7.4107	7.5281	8.0776	4.6078	9.0882	6.2217	6.6568	5.5532	11.0882	0.9964	6.7208	0.5826	8.7627	3.2415	9.8492	9.1	8.6967	1.8022	12.1422	12.256	6.9759	7.8383	10.864	12.0522	7.9787	10.133	6.0482	9.443	5.2784	6.9117	7.0373	9.5105	8.9324
TCGA-78-7147-01	586	1	5.298	8.7184	6.3083	10.5067	10.823	9.3737	0.8339	9.0403	8.5701	7.0831	8.737	7.815	3.988	5.6747	9.3626	8.931	7.0656	5.0439	11.1429	1.1202	7.6561	2.5097	8.157	7.5121	12.0154	9.7038	7.7973	1.7431	12.6667	12.5153	6.9751	6.4442	11.792	12.8351	9.3004	10.7536	7.4649	10.5933	3.5795	7.8125	7.5028	10.1368	10.1715
TCGA-78-7148-01	626	1	10.568	7.9151	10.1243	11.5455	9.7814	9.7735	6.5391	8.3488	7.9421	8.8684	7.9277	7.2072	6.0875	7.181	9.738	8.1302	6.6639	5.3338	11.0582	1.6448	6.5878	4.9686	8.9941	6.7534	10.5696	8.3901	7.8467	1.8714	11.9673	13.0057	6.4973	7.7178	10.519	13.0083	9.2649	11.8187	6.3357	10.7966	5.0168	7.8731	8.3294	9.2385	8.0398
TCGA-78-7149-01	3940	0	3.653	8.8824	9.4516	8.4123	11.171	9.2165	6.0579	9.7683	6.6819	8.4829	8.196	7.7104	3.8861	1.6615	10.3142	9.515	6.8496	5.9595	10.0161	0.6336	8.7574	2.8208	8.7428	8.0832	11.691	8.135	7.4487	1.9096	11.861	13.5037	7.1174	7.5834	11.694	14.3565	10.6365	12.1028	6.216	10.0858	6.4883	8.6427	6.7097	9.8046	8.2519
TCGA-78-7150-01	666	1	8.0742	7.5592	11.3745	10.2996	9.5424	10.3256	7.7787	7.4741	7.4808	8.7293	8.7254	7.4356	7.0423	6.5537	9.3014	6.9178	7.1049	4.6817	11.5446	2.8701	6.4534	5.0748	8.8163	6.4999	10.3385	7.7862	7.9424	1.7202	12.0662	13.2542	6.5625	7.3721	10.053	12.373	9.2601	11.0802	4.8636	10.9803	2.4828	7.2577	7.6972	8.4925	6.8548
TCGA-78-7152-01	1215	1	3.3625	8.0583	6.8128	10.0989	10.1	10.5813	0	8.4556	7.4574	8.9833	8.4479	7.6214	5.2879	6.5088	9.8818	8.4402	7.5883	4.5384	11.2357	0.9475	5.623	1.5149	9.5638	7.2335	10.8319	8.7697	8.2727	0.9475	12.0265	12.7664	7.6282	7.1975	10.938	13.4309	10.6082	10.6463	5.5956	10.4351	2.4963	9.083	6.6489	9.8761	8.2465
TCGA-78-7153-01	3635	0	2.9551	9.2931	10.5513	8.8271	10.521	10.8528	0	8.1918	6.9027	10.1195	7.0287	8.2698	5.3972	0	10.0783	6.9663	7.0907	4.3517	10.8021	0.6907	8.9086	7.3925	9.3834	6.8249	10.9518	6.8171	8.1992	0	12.152	13.0346	8.5971	6.512	10.977	13.7172	9.7044	10.8566	5.7165	10.5845	3.5913	9.492	6.7528	10.2203	9.8507
TCGA-78-7154-01	593	1	5.7059	7.6384	10.8086	12.7254	10.057	11.7049	2.1928	6.9674	6.3075	8.4284	9.7221	7.1077	5.4744	6.055	8.9842	7.2723	7.54	5.0138	11.2632	1.4782	6.6669	1.7576	8.8286	10.1326	9.976	9.2113	8.3905	7.3897	10.7903	12.3656	5.6894	6.8861	10.775	13.8501	9.3729	10.8314	3.1275	11.0485	4.0436	8.4209	8.5909	8.1273	6.4581
TCGA-78-7155-01	1171	1	3.0914	7.4904	0.6587	10.6951	8.9637	8.4367	2.6342	7.6662	6.8932	8.3472	8.0742	7.3994	6.7086	5.9755	8.6197	7.5451	5.7716	3.4998	11.0117	0	2.7628	1.1093	8.5141	10.7387	11.8331	7.1866	9.3138	3.0914	7.7529	12.6084	4.9314	2.8808	11.121	12.1274	7.9715	10.3087	0.6587	11.94	1.7289	7.7986	6.4562	7.7873	10.4758
TCGA-78-7156-01	976	1	7.8524	10.0183	9.5503	6.6421	10.904	8.0069	1.1659	9.3724	4.9831	8.1208	7.8485	8.2023	6.3327	3.1834	10.3819	8.8316	5.2513	5.9549	10.5099	0.6977	9.7521	1.1659	9.3	9.3184	10.4699	7.8446	7.9572	0	11.3473	12.0096	4.2969	7.4574	9.7961	13.1525	10.2348	10.7821	5.9384	10.3417	6.0097	6.5301	6.0373	9.7098	6.5965
TCGA-78-7158-01	179	1	4.1029	8.9591	0.5007	9.5467	9.9037	8.7732	1.4113	8.8008	6.9999	8.977	6.4685	8.1768	5.905	5.2609	10.7635	8.6294	7.5426	5.1238	11.2607	1.6204	7.9316	4.8059	8.4304	7.4013	10.7216	9.2725	8.2573	1.1666	13.0723	12.0346	7.8406	5.1131	11.16	13.442	9.3378	11.093	7.291	11.0804	3.217	7.7461	6.5345	9.9321	8.8043
TCGA-78-7159-01	1974	0	8.8727	7.9142	7.309	11.748	9.2946	9.5439	0	8.2523	7.301	9.4584	8.016	8.1563	5.9957	1.4076	9.7564	8.7925	7.3201	5.1069	10.544	1.6798	7.1669	2.28	9.0526	4.3025	10.3795	9.0837	8.5426	2.7026	11.8	12.2351	7.0458	6.5979	11.345	13.1809	8.7544	10.1788	5.0664	10.7017	5.5463	7.4521	7.4384	9.9704	8.1339
TCGA-78-7160-01	697	1	9.5273	8.0135	11.596	10.1885	9.2478	10.9533	1.8171	7.0851	8.3988	8.1703	8.2321	7.76	6.8558	7.9743	10.2063	7.4208	6.5913	6.6823	11.4733	0.7057	7.7431	3.3873	8.6475	6.816	10.2194	8.8758	8.4817	5.4562	11.9871	11.9435	5.2564	7.6097	10.714	12.2795	8.1221	10.0571	5.9734	11.1044	3.2019	9.4885	8.2169	8.5943	8.0586
TCGA-78-7161-01	291	1	9.3309	8.8313	8.4499	10.6465	8.8138	8.0942	0.4514	8.7198	5.8565	9.031	7.9212	8.5324	5.64	4.6996	10.6947	9.5426	7.4595	5.6189	10.4025	1.6801	8.2043	5.9106	9.191	4.303	9.8408	9.8677	7.6502	1.8367	10.831	12.7649	5.4711	6.7734	11.06	12.975	9.5073	10.5473	5.6552	9.6288	3.3003	6.1003	7.1911	11.0015	8.7462
TCGA-78-7162-01	3169	1	4.3239	8.7562	9.5814	10.7042	10.235	10.3732	2.3291	9.1181	6.6357	8.6655	8.4515	7.4067	5.3663	4.3239	10.5701	8.6551	6.8876	5.2266	11.2414	0	8.1415	1.9735	8.5826	6.8747	10.6379	8.8518	8.1245	0.7923	12.1081	10.2926	6.8926	6.9789	11.346	12.4551	8.3321	10.171	5.7367	10.6524	7.0838	9.2566	7.7873	8.6366	8.7464
TCGA-78-7163-01	7248	0	3.8433	8.8577	6.5928	9.4606	10.604	8.0534	1.4393	10.077	5.3482	6.9925	8.1435	7.4786	4.0362	4.0362	10.7778	9.4884	6.4355	5.8819	10.5281	1.2446	8.5725	2.5344	8.1365	6.3111	11.9325	8.0974	8.1678	1.0194	11.7127	10.8654	6.7248	7.7889	11.678	11.5494	10.2152	11.0554	6.2013	10.7277	0	6.1605	8.3362	8.5608	11.1078
TCGA-78-7166-01	258	1	10.186	9.5838	11.0033	10.0239	10.346	10.1533	2.0429	9.3924	8.5245	8.0854	7.8548	8.6081	2.6668	7.363	8.2101	9.5143	6.6433	5.9245	10.0803	2.6668	8.6898	2.5617	9.4504	5.7444	11.5452	8.525	7.9437	3.6544	10.9575	15.0082	5.8816	7.5836	10.356	14.247	10.7043	12.8777	6.1943	9.3196	3.8455	8.2487	8.6423	9.1075	6.1222
TCGA-78-7167-01	2681	1	9.494	9.2075	8.2099	6.9511	10.434	7.5029	0.9195	9.1866	5.833	8.4858	7.2249	8.5467	5.0614	6.5909	10.7809	7.9974	7.3421	5.0568	10.2736	0.9195	9.6606	1.7559	9.4407	6.5085	10.8933	9.6769	7.9932	0.9195	11.9933	11.6195	6.1704	6.3702	10.45	12.9438	9.5878	11.0998	5.5656	9.7008	4.648	6.1525	5.2535	10.0504	8.7102
TCGA-78-7220-01	807	1	10.591	7.2265	11.2592	8.8572	9.3983	9.8009	9.9153	7.4329	6.0256	9.6547	8.881	7.707	6.6708	7.909	8.6398	8.053	5.566	5.2101	11.6245	3.6234	5.8225	5.8093	8.7125	5.5646	10.38	8.8763	8.9091	0.599	10.7471	11.6176	6.9242	7.1077	11.321	12.2898	8.1345	10.3174	4.1269	11.8245	3.0367	8.053	7.9754	8.4212	8.5195
TCGA-78-7535-01	949	1	6.3828	9.2655	4.591	9.1583	11.043	11.8674	2.2955	9.6895	6.8378	8.4005	8.3432	8.1795	2.2955	8.9262	10.3696	9.4269	6.7223	5.4027	9.4067	0.7747	5.1772	3.6699	9.5994	5.4478	12.397	9.0318	6.9161	4.7707	11.2362	13.0272	7.0264	8.6013	10.837	12.7082	11.1814	11.8284	4.844	9.2862	3.5437	7.3492	7.5944	9.3551	9.1072
TCGA-78-7536-01	244	1	7.4513	7.5452	7.2257	10.0494	9.5557	10.6447	0.5963	7.6076	10.0191	9.2321	7.7716	8.853	6.8115	2.6722	9.6331	7.279	7.2783	7.5853	10.8677	0.5963	4.2607	1.6075	7.7495	3.6159	10.0191	9.7577	9.1922	2.6427	10.0529	12.4653	3.8386	7.2502	10.108	12.2107	8.4899	9.6303	2.487	11.3816	0.6066	8.6326	7.8198	10.0134	10.9902
TCGA-78-7537-01	1622	1	3.2569	9.0251	9.3124	9.8749	10.331	10.2374	1.946	9.5024	7.7026	7.9108	7.7215	8.1452	4.5744	1.4507	10.0482	9.901	6.8323	5.2309	10.4502	1.0496	8.5533	3.0237	10.0296	5.9947	10.7167	8.0474	7.9838	2.4004	12.9674	12.7	7.4128	6.769	11.452	13.4291	10.0559	10.5745	6.4001	10.1603	2.4004	9.743	6.829	9.4335	8.7327
TCGA-78-7539-01	791	0	4.0541	9.1026	2.4378	9.1801	9.9171	10.1384	0.3725	9.189	8.2923	8.4538	8.4814	8.6379	4.5656	2.7217	9.9407	8.8266	6.9376	7.1952	11.4656	0.3725	6.9104	2.2718	8.1305	9.2661	11.2398	9.3745	7.5526	1.6144	12.5735	10.9555	8.479	6.4577	13.469	13.2091	9.5141	10.4973	7.0127	10.7504	2.7847	8.4367	7.2902	8.5431	9.315
TCGA-78-7540-01	1197	1	6.4291	8.4263	9.8192	12.0408	10.51	9.7757	1.4098	8.7277	6.0449	8.9803	10.2332	7.8538	3.0515	10.0573	9.4935	8.6173	5.5185	6.2314	10.7007	4.6924	8.4728	2.2158	12.5326	6.2585	11.6873	9.8428	8.4714	6.1347	14.2161	10.7013	5.9766	8.2427	11.159	12.4299	8.9115	9.1888	4.9615	9.5275	3.2149	8.6921	8.0103	8.6886	9.0305
TCGA-78-7542-01	321	1	3.3509	8.1315	1.5618	13.0025	10.718	8.4134	1.081	7.4065	7.7984	8.7291	6.9045	8.0135	5.43	4.2576	8.1387	8.573	6.1551	4.8054	12.4843	1.081	4.7059	2.2096	9.3528	9.4307	11.5385	9.2363	8.496	0.6395	11.7386	11.9324	8.3069	6.2797	10.539	12.8339	9.1283	8.0413	2.835	10.224	3.8466	6.3554	8.7291	6.8944	10.1313
TCGA-78-7633-01	1528	1	5.7465	9.1407	11.1769	8.2637	10.142	8.007	1.7239	8.7109	4.7947	8.6849	7.9693	8.0102	5.8724	4.3221	10.4884	8.4415	7.814	4.8177	10.2314	0.8982	9.5876	1.5923	8.9623	7.424	10.5344	8.6656	8.246	1.8445	12.2574	12.2043	7.6524	7.0158	11.004	12.7895	9.4394	12.082	5.5654	10.7873	6.0076	7.0286	6.4396	9.7846	7.0569
TCGA-78-8640-01	7062	0	5.7576	8.1791	5.4843	11.9108	9.6008	11.1939	1.1157	7.8423	9.4154	8.1849	8.1791	6.942	6.5867	3.0004	7.9118	8.8912	7.7016	7.8357	10.872	1.4597	9.007	1.1157	9.7044	5.8626	11.0244	10.6363	9.2682	8.0098	10.1739	13.2429	7.14	7.2657	11.741	13.3096	9.9666	12.125	6.0394	11.3855	3.2858	9.0265	8.1674	8.2582	8.3565
TCGA-78-8648-01	1209	1	3.2332	8.7493	4.6254	11.0269	9.6035	11.6326	3.5478	8.4164	8.7664	8.4798	8.2311	8.2921	5.7876	5.1133	10.9376	8.8531	7.5768	7.9716	11.3164	1.7189	8.6142	4.215	8.9763	4.5332	10.4975	8.6649	7.778	2.4812	12.2407	12.1006	6.909	6.2598	11.23	12.4964	9.6357	10.1703	5.4772	10.286	4.6254	10.4351	8.5753	8.4134	9.2703
TCGA-78-8655-01	2360	0	3.2068	8.6185	2.0589	10.5964	9.0648	10.3315	0.7078	9.264	8.3326	8.3538	8.1757	7.8086	4.6107	7.6105	10.131	9.3738	4.6635	6.7929	10.9642	0	7.5038	2.6009	9.6431	5.398	10.6382	9.6026	8.3538	1.821	11.7025	13.0154	7.0454	7.5287	11.575	13.2527	10.0201	11.6047	6.6132	10.0583	5.0846	8.844	8.3426	8.9345	8.4871
TCGA-78-8660-01	321	1	3.79	8.8836	9.2191	10.9671	9.5851	11.0352	1.4594	8.3789	10.1148	8.5682	8.8548	8.2957	5.9734	10.5162	9.9432	9.0195	7.5718	6.5152	11.224	1.1154	6.7747	1.737	9.6998	5.1699	11.2873	8.5571	8.7706	1.1154	12.0034	12.7353	6.8713	6.7824	11.891	13.631	9.7573	10.8697	4.9425	10.73	5.098	9.2401	6.5326	10.2876	8.53
TCGA-78-8662-01	3361	1	4.3358	8.3426	2.3135	9.8386	9.6023	10.1665	2.3135	7.3121	6.2954	8.0645	7.6261	7.7015	5.0913	7.2903	8.8128	9.5751	8.2914	4.6833	9.8302	0	8.0174	2.1074	9.3507	1.2164	11.3596	9.7393	8.1744	6.012	10.9579	14.1777	5.0342	7.0592	12.067	13.6601	11.1052	12.2186	2.1074	10.3035	3.4499	6.3762	6.8097	9.5498	5.46
TCGA-80-5607-01	0	0	7.4919	8.2541	8.5234	11.3219	10.091	10.0213	0.8589	8.5462	8.1641	9.5199	9.1124	7.9228	5.685	10.3421	9.6247	8.8375	6.6525	5.6443	11.5959	1.7828	6.7274	3.3148	10.1805	7.6638	10.6073	9.1251	8.1792	2.5563	12.8246	13.2763	7.8984	7.1138	11.514	13.1772	9.6292	10.5474	4.4061	10.6051	4.7997	7.9982	7.0708	9.4744	8.0915
TCGA-80-5608-01	2832	0	8.9242	8.2871	11.0314	12.2173	9.8155	9.6083	1.2742	8.6642	7.0392	8.48	7.8747	8.1498	5.1884	8.0471	9.8728	8.9749	7.8064	5.812	10.7025	1.2742	8.2574	2.1847	9.196	4.0535	10.7526	8.9951	7.8528	1.6452	12.1607	13.1938	7.144	7.0075	11.267	13.1696	9.1391	11.5282	3.8906	10.9816	5.4686	7.9387	8.5863	10.6136	7.4697
TCGA-80-5611-01	2595	0	4.7051	8.8324	6.7552	11.6064	10.265	10.9729	1.418	9.2882	10.5381	7.4778	8.1104	7.7297	4.5762	5.1522	8.7376	7.4958	6.6529	8.6259	11.5956	0.6391	7.901	2.2933	8.9362	7.5738	10.66	9.8264	7.884	4.6095	8.5785	13.089	6.8207	7.7106	12.42	13.3405	9.4151	9.9648	7.1272	11.1936	1.9211	9.5808	8.7876	9.2159	10.2698
TCGA-83-5908-01	824	0	2.1308	8.212	2.0015	10.3274	9.0378	9.3908	1.0885	7.9668	8.7966	8.8858	8.5851	8.526	5.1364	8.0901	9.5453	8.5465	7.28	6.3437	12.4506	2.7296	3.2112	3.1515	10.063	2.6455	10.8309	9.6669	8.7788	2.2495	10.9372	10.9477	6.3126	7.5078	10.54	12.0362	8.0209	10.4522	3.3237	11.5182	4.8093	8.5231	8.4177	8.4767	10.7705
TCGA-86-6562-01	376	1	6.9759	7.6772	2.2485	12.1318	9.9987	11.1219	0.6192	8.5172	7.9835	8.0771	8.1767	7.5175	6.3181	5.6832	10.4322	7.8175	6.9002	6.609	10.9504	1.8797	7.868	2.4027	8.6962	2.9942	10.8909	9.937	9.0293	7.0882	11.0486	12.3981	6.7928	8.5818	11.035	12.3507	8.7457	10.4506	5.6373	11.2028	3.5541	8.6868	6.8807	9.0455	10.8555
TCGA-86-6851-01	179	0	4.8956	8.7268	3.4923	10.8828	9.3286	10.1038	0.6504	7.9171	9.9844	8.146	8.4115	8.2616	6.086	5.205	10.0041	9.1347	7.6663	7.0625	11.9322	1.0971	5.2381	3.1194	8.9325	1.0971	10.7199	10.1283	8.5151	2.1433	12.6953	11.5437	7.0099	6.7293	11.418	12.0925	9.0309	9.6519	6.3354	11.3118	2.9166	9.1535	6.8588	8.644	10.8992
TCGA-86-7701-01	947	0	7.0407	8.0294	7.9017	10.7611	9.3066	9.837	1.0382	7.3022	8.0859	9.0612	7.5097	7.6526	6.5983	7.0174	11.4451	7.8301	6.5232	6.7009	11.1682	1.0382	5.9322	4.4643	8.958	4.5316	9.9883	9.0091	8.8369	2.057	11.0937	12.57	5.5333	7.7825	10.393	12.3341	8.3327	9.6585	6.1188	10.4785	3.3901	8.5551	7.7045	8	7.9144
TCGA-86-7711-01	1046	1	9.2943	7.1819	8.3391	11.9882	9.269	10.383	2.844	6.725	9.3697	8.369	7.8745	6.5545	8.4281	6.8293	9.0055	7.4506	6.666	8.977	11.252	2.4368	3.063	2.7206	9.939	2.4368	10.649	9.299	8.7504	5.468	8.019	13.0794	4.7651	8.1017	12.578	13.2382	9.3885	11.4879	5.1349	9.7858	4.6682	8.0799	6.5858	8.1488	11.7839
TCGA-86-7713-01	1157	0	8.4087	9.3798	4.6876	11.5065	9.4803	9.1891	2.3472	8.1726	7.7659	8.0978	6.9218	8.621	7.0916	2.7595	10.7805	9.9737	5.6782	4.7719	10.5985	1.7676	7.9666	0	9.6266	3.4409	10.9071	8.7936	8.9665	2.1266	11.8631	11.7366	6.6588	5.1354	10.615	12.8227	9.2427	10.2012	1.2886	10.5505	3.3418	7.2595	6.8363	10.5161	5.6594
TCGA-86-7714-01	625	1	5.3389	8.6822	6.5262	10.8618	9.9468	10.4955	0.9559	8.2537	6.0149	8.5937	8.21	7.5476	5.9563	6.1406	9.8821	8.7903	5.2626	5.3786	10.8061	0.9559	8.0807	4.558	9.1128	9.0166	9.9954	9.29	8.0907	3.9163	12.7458	11.5247	5.4044	6.8538	10.814	12.3756	8.5474	9.9343	6.3039	11.2937	3.0907	7.4267	6.6118	8.2858	8.6352
TCGA-86-7953-01	997	0	7.3531	7.9123	3.3021	10.5414	10.234	10.0192	2.8489	8.2169	9.8865	8.7191	8.8412	8.5667	6.7463	3.1179	10.4176	8.6998	5.9185	8.768	11.3713	1.6173	5.7279	2.5904	8.2749	6.622	11.3056	8.7059	8.8197	5.4444	12.5231	12.1551	6.8047	6.8534	11.366	12.9984	8.7272	10.345	5.6451	12.4248	10.2393	8.9937	7.7175	8.4213	9.7909
TCGA-86-7954-01	605	0	6.5929	8.4084	7.9635	10.9547	9.6196	11.3497	1.3816	7.5612	10.4135	8.6067	8.3821	7.5713	6.0739	5.1614	10.2004	8.4141	5.238	7.0833	12.4143	1.5227	7.5467	3.4814	8.5019	5.5798	10.811	10.998	8.109	2.1631	11.1682	11.2398	4.5532	6.4545	11.633	11.8036	8.5957	10.8355	4.9653	10.5872	4.3619	9.6937	6.6363	9.4395	8.8948
TCGA-86-7955-01	1072	0	7.9295	9.3506	9.533	12.6468	10.564	7.8805	1.4892	7.6111	5.2201	9.6577	6.967	7.5435	6.7256	4.1348	7.6555	8.9452	7.3426	7.2587	11.5947	1.7697	8.5489	0	9.7504	6.2875	10.9405	8.3661	9.0749	5.4912	9.9695	13.3168	8.118	8.4203	11.388	13.7105	10.0824	11.2771	6.8626	10.5086	1.771	9.5977	7.8106	9.4093	9.3285
TCGA-86-8054-01	1148	0	7.7491	8.0136	3.4582	11.1856	9.0861	8.5571	0.7365	7.8929	6.9162	10.3085	9.4825	8.534	6.9836	2.5839	13.5166	8.6329	7.1505	5.0114	11.1163	0.4147	3.9684	0.7365	9.8426	3.1688	10.2694	9.2049	8.8242	1.8735	10.3281	13.1427	6.9568	8.3356	10.638	12.449	7.7357	9.4492	5.2339	11.8151	2.414	8.1602	7.8711	9.8618	10.3741
TCGA-86-8055-01	124	1	3.3221	8.369	3.5504	12.2052	9.7431	9.4664	0	7.6029	8.9947	8.4259	8.4078	7.6399	6.1557	7.379	9.3873	7.0877	6.6925	7.4292	10.8828	1.8367	5.6149	3.6523	8.3118	4.7853	10.5046	8.302	8.1139	2.9742	12.3326	12.0999	7.275	6.3196	10.929	12.1537	9.0321	9.9844	5.469	10.4819	4.0383	8.5776	7.4549	7.4265	9.5749
TCGA-86-8056-01	139	0	4.3258	9.2593	6.498	10.6092	9.7812	9.8974	0.4291	7.6686	7.4638	8.4719	8.7594	7.9862	6.4066	2.1585	9.5494	9.2796	6.013	6.8395	10.9365	2.6315	7.5525	2.71	8.4705	7.1033	10.756	9.6121	8.1305	8.1933	13.5784	10.9939	6.3465	7.0477	11.917	12.133	8.6604	9.5739	6.191	12.262	5.2374	8.1727	7.1033	8.2649	9.2296
TCGA-86-8073-01	740	0	3.9482	8.0975	7.5006	9.7318	10.195	10.5981	1.768	7.4494	7.883	9.136	7.9509	8.8906	6.5568	3.4551	8.8298	8.422	7.2054	6.0826	11.0279	1.4428	6.3776	1.4428	8.5781	6.8295	10.4123	10.2702	9.5736	6.159	12.6742	10.7714	5.7622	8.5198	11.703	12.2717	7.9688	9.3441	6.2915	12.1003	3.4551	8.5076	8.1024	9.5286	8.959
TCGA-86-8074-01	24	0	2.4029	8.295	5.1993	10.8791	10.094	10.1238	0.4408	8.6825	9.1687	8.3323	8.7076	7.7631	6.2353	7.2119	9.7022	7.4575	5.929	6.1039	11.2732	1.2807	6.0686	3.9684	8.1982	4.0328	11.0916	9.1745	7.8235	2.9617	12.2512	11.93	6.8854	6.5534	11.647	11.9752	9.0662	9.8181	4.9552	11.6734	2.9617	8.0582	6.5257	10.0957	11.0754
TCGA-86-8075-01	694	1	3.5323	8.5439	3.2148	11.1231	9.4222	10.2959	1.28	8.2607	9.8811	8.4968	8.5593	7.5901	6.132	8.3159	9.4071	8.1195	6.56	6.7958	11.047	1.7161	4.6103	3.5323	8.4338	6.1896	10.9053	9.8567	8.6213	6.6983	11.6943	11.3827	6.0348	6.1495	12.222	11.9256	9.0531	10.2787	5.6061	10.4172	4.4116	8.3804	6.7793	7.7287	9.3844
TCGA-86-8076-01	993	0	6.8383	8.6246	7.3466	10.552	9.8837	11.0708	1.1173	8.2644	8.9424	8.7682	7.9135	8.1907	6.8578	4.5127	10.313	8.5545	7.705	6.6788	11.8009	1.3557	7.4338	3.1994	9.295	6.9242	10.8043	8.1576	7.9089	4.2126	12.1739	12.5574	6.8675	7.5664	10.76	13.2247	9.3658	10.8156	6.6607	10.3006	3.7932	9.3835	8.0972	9.6327	8.5379
TCGA-86-8278-01	944	0	4.1789	8.448	6.4991	10.9833	9.5815	11.7423	0.6027	8.1497	8.0014	7.8947	8.8933	8.3676	5.6589	6.2903	9.9669	9.3193	7.1308	5.5508	11.6177	1.6203	6.5115	5.7659	9.4335	5.9344	10.4864	9.0067	7.9366	2.8526	12.5206	11.287	7.8206	7.2593	11.193	12.4513	9.5534	10.5673	5.7169	10.5288	1.9456	8.192	7.1129	9.2048	9.014
TCGA-86-8279-01	949	0	7.1411	9.026	1.1278	10.5355	9.697	9.6095	1.9866	8.7671	8.1539	9.3727	8.0959	8.145	5.6321	2.5938	11.2104	9.2677	6.7883	5.1616	10.9358	0.9175	8.5505	2.663	8.4575	4.0363	11.579	8.7222	8.1314	2.5938	10.7964	12.1171	7.96	6.2528	11.589	12.3462	10.0486	10.5621	4.8207	10.4286	1.7529	7.2796	6.264	10.0813	8.9046
TCGA-86-8280-01	701	0	2.9152	9.2411	5.2089	10.5484	10.579	9.8236	3.2816	8.9572	9.3707	8.433	7.7874	8.3292	5.8231	5.9302	10.1282	9.3584	6.911	6.4514	11.7235	1.2951	8.5579	3.4349	9.121	7.5506	11.2779	8.6285	8.6031	5.1362	11.8529	12.0443	7.4402	7.139	11.93	12.0552	10.2563	11.0308	5.8415	10.3176	2.7689	8.8054	7.6942	8.7234	10.4558
TCGA-86-8281-01	0	0	7.1952	9.2168	12.5737	8.8572	10.365	9.8233	0.7625	7.4992	5.419	8.5492	7.2223	7.9934	5.8344	5.4306	11.2478	9.1739	6.7499	4.865	10.8801	0.7625	9.9837	2.3725	9.2916	6.0392	11.2881	8.6527	8.0739	1.9205	11.6804	10.3768	5.8862	7.9039	11.291	11.9539	9.2649	11.6895	5.6959	10.0175	5.7151	8.9009	7.0088	9.1704	6.5433
TCGA-86-8358-01	653	0	3.6174	7.4325	8.0745	10.8053	8.6446	9.2187	1.5798	8.5676	7.7279	9.4276	8.2669	8.5224	7.6241	4.5162	8.7616	8.3336	9.0361	4.762	10.3885	0.7339	5.3962	5.3023	9.5666	7.3292	9.7742	8.5353	8.635	1.5798	9.4373	11.9627	5.9073	6.8952	11.65	12.4314	8.0566	9.8997	7.8231	11.4556	2.6716	8.0276	6.9032	5.9388	10.6389
TCGA-86-8359-01	444	1	8.1595	8.0078	9.2607	10.5863	9.9866	11.5524	1.1474	8.1678	7.5648	8.7861	8.6596	7.9127	6.0577	7.5602	10.2829	7.739	4.4735	7.2112	9.9672	1.497	8.0447	4.1213	9.372	1.7783	11.0643	8.4576	8.0678	3.3381	12.3254	12.9227	5.9766	7.0336	10.808	13.4067	10.5288	11.9394	3.1535	10.3821	2.0135	7.1858	6.6789	10.7003	8.1472
TCGA-86-8585-01	353	0	7.4164	8.8823	3.1243	11.8944	10.105	10.2613	1.6806	8.0096	10.8964	8.8551	7.7916	8.3981	4.1328	2.9292	9.4741	9.2284	6.9023	8.1029	10.7558	2.1072	7.9924	3.5883	9.8832	4.7797	11.0153	8.6407	7.8888	3.1243	9.7957	13.9224	5.839	7.8685	10.965	12.7881	10.3711	12.4289	6.4794	10.5768	4.0391	9.8638	8.0832	10.4619	8.2175
TCGA-86-8668-01	423	0	5.1687	10.0209	5.1983	10.0373	9.581	9.3733	5.1687	8.8039	9.1322	8.2889	8.4418	7.7699	6.3852	2.7975	10.1746	8.8079	6.8941	5.0425	10.9383	0	7.4211	2.45	8.4724	8.3916	10.9383	8.2682	7.5866	1.9914	13.0897	11.4234	8.6297	5.8835	11.33	11.7973	10.8956	10.497	6.4481	10.8031	3.0773	8.2577	6.8341	8.8574	8.4724
TCGA-86-8669-01	938	0	7.3482	8.525	10.8981	8.8856	9.9618	9.8495	1.1765	6.7158	8.3545	9.4981	8.0602	8.3269	5.369	3.2003	10.4042	8.4385	7.4496	5.6349	10.8057	0.705	10.5052	3.9535	7.7285	3.8936	10.2963	8.3184	8.1429	2.0533	11.1042	12.0683	8.3212	5.8189	12.629	13.081	9.0036	8.4799	6.5966	10.6119	1.8158	8.4976	6.1227	9.5894	8.3632
TCGA-86-8671-01	839	0	2.9284	9.3384	5.8519	10.1611	10.538	11.5174	2.1064	9.1232	10.7929	8.2742	8.4321	8.9681	5.1133	5.692	10.801	9.4698	6.8588	8.0145	12.5327	1.68	7.9662	4.4555	9.1568	5.5465	11.4238	8.8981	7.6233	2.2802	11.8819	11.9969	7.2941	6.9005	11.415	11.7893	9.8098	10.3343	6.2257	10.0582	5.3468	9.7857	7.4201	8.5343	9.1761
TCGA-86-8672-01	19	1	6.0323	8.6766	10.0796	10.888	9.4602	10.792	1.9153	8.3189	9.7908	8.8317	8.0538	7.7445	5.3644	10.6776	8.458	8.6569	6.4044	6.0697	10.9037	1.2546	5.9376	2.8554	10.3034	1.9153	11.3334	8.7128	8.8599	2.3668	9.9029	13.3467	7.1951	7.6922	11.601	13.7175	10.1787	11.2058	3.3232	7.416	4.4456	8.1236	7.6971	8.9394	6.3094
TCGA-86-8673-01	862	0	6.9898	8.9996	8.6155	9.0467	10.257	11.452	0.6658	9.1026	8.3308	7.776	8.7243	8.6538	4.2169	7.9227	10.5705	9.1869	8.1446	5.7051	10.7259	0.6658	6.0208	2.7793	9.5664	4.8943	12.0776	7.6594	9.1001	3.2034	11.6249	13.6185	6.7117	7.2893	10.964	13.0977	10.0817	11.87	6.5129	10.4297	3.3763	10.1042	7.1592	8.5871	9.0924
TCGA-86-8674-01	806	0	7.1218	9.2561	12.4369	5.6247	10.182	11.7399	2.303	9.0154	5.8261	8.1541	6.4889	8.4469	3.4	5.7354	11.0629	9.4564	6.7812	5.076	10.7272	1.0869	10.291	1.0869	9.1111	5.5401	11.67	7.5343	7.6679	0	11.7611	13.5804	6.782	7.1956	10.568	12.9106	10.0906	12.707	6.1888	9.08	3.4748	8.7869	7.2867	10.689	5.1639
TCGA-86-A456-01	896	0	4.3166	10.0876	5.0674	8.7495	10.79	11.9839	1.9845	8.5485	8.1725	9.0732	8.5636	7.733	4.589	9.478	10.1354	9.1741	6.4647	7.9235	12.1346	1.1262	8.0357	1.1262	8.9595	6.6576	11.514	8.9248	7.8148	2.3618	12.2655	12.2516	8.4014	7.9658	11.624	13.273	10.1817	10.6627	6.1455	10.0837	4.3588	9.6407	7.1772	8.9388	8.1087
TCGA-86-A4D0-01	116	1	6.9084	8.2739	11.4135	10.5798	10.435	8.9559	0	7.4106	7.3772	11.0036	6.4521	8.2782	6.2552	2.2396	11.3566	7.7548	8.4201	7.4344	11.3819	1.5167	6.0876	8.049	8.6364	5.2565	10.6349	7.7042	8.3764	2.4991	10.4288	12.3159	6.6589	6.3814	10.552	13.5392	8.1446	9.8521	5.8753	11.331	1.923	8.4143	7.8654	11.06	8.049
TCGA-86-A4JF-01	737	1	4.0054	7.8538	3.2351	11.5358	9.3382	11.3654	0.9153	8.4544	10.2126	7.9628	8.3355	7.6366	5.7824	8.1341	9.7479	8.5086	6.9036	6.2792	11.9306	1.8709	6.3586	2.318	9.349	3.3014	10.7359	10.1557	8.5278	5.2558	12.7533	13.2726	6.8039	7.7772	11.266	12.9364	10.5078	12.0609	5.3528	9.5828	3.8808	8.6004	6.8764	9.041	8.6394
TCGA-86-A4P7-01	415	0	5.2836	8.7977	4.0984	10.2943	9.5045	11.8991	3.6305	8.7881	8.2448	8.5897	8.2259	8.7776	5.248	4.1761	10.4035	9.9672	7.0766	6.9804	11.2584	1.2769	7.4559	1.2769	8.9341	2.6826	11.0023	10.0904	8.1338	5.8203	12.2992	11.91	6.4328	6.576	11.568	12.968	10.0249	10.4404	5.0761	9.7316	5.9268	9.5572	6.8848	9.0892	8.0968
TCGA-86-A4P8-01	805	0	4.0037	9.3353	4.1217	10.6181	9.8993	11.5222	2.4745	8.722	7.9407	8.8107	8.3587	8.9843	5.3614	4.0441	11.0264	10.5292	7.3777	7.873	11.2896	0	8.4423	3.734	9.054	3.6838	10.424	9.4721	7.5038	3.3385	12.2337	11.0327	7.1153	6.0582	10.473	11.5186	9.7034	9.3635	6.6556	10.3569	6.6159	10.1919	7.8726	8.507	8.4329
TCGA-91-6828-01	323	0	6.7648	8.7771	8.9467	10.5289	9.5133	10.4267	1.4241	7.6858	9.2518	9.3499	8.2035	7.9521	6.1123	1.8177	9.0716	7.196	5.9617	7.1504	11.4589	2.381	7.3453	2.785	7.9812	3.6763	9.7261	9.2195	8.236	4.6674	12.5376	11.4515	7.5009	6.3689	11.583	12.166	8.5562	9.2133	5.5377	10.8982	3.5781	9.1527	6.8929	10.4684	9.4313

TCGA-91-6829-01	1258	1	1.8405	7.7113	3.6864	9.2988	9.9605	9.9425	1.6159	7.0995	6.6461	8.3814	8.0318	6.9406	6.9345	3.95	10.4881	6.9765	7.1248	5.6239	10.9345	1.3498	4.659	3.5015	8.0684	7.1439	11.2369	9.536	8.029	1.8405	11.8735	10.9724	7.0847	7.1491	10.999	11.8228	8.7465	10.3843	5.8783	12.0614	4.7168	8.4931	7.493	7.9797	10.7444
TCGA-91-6830-01	60	0	6.5145	8.395	9.2495	10.7148	8.9386	10.4404	3.1148	8.4282	7.9182	8.5768	8.3381	7.1805	6.4272	2.834	9.5706	8.0725	6.5492	7.1181	11.3731	2.0233	7.7824	4.798	8.2095	4.2191	9.7737	8.9609	8.4243	4.0291	12.6812	11.3871	5.0524	6.9762	10.706	12.5045	8.9742	10.2673	7.0448	10.2538	6.7066	8.9917	7.8795	8.1524	8.7331
TCGA-91-6831-01	310	0	8.2961	7.9084	0.8252	10.1343	9.2561	10.0178	0.8252	9.0212	10.198	8.5519	7.5646	7.9629	6.7585	5.6987	9.4421	7.5168	7.6016	6.045	11.3538	1.7291	8.402	2.8428	8.2048	5.0953	10.5356	8.764	8.0155	2.8428	9.8506	11.9539	6.1544	7.265	10.667	12.7237	8.6214	9.0594	5.3622	10.9319	4.3968	8.0828	7.6613	10.834	9.6224
TCGA-91-6835-01	79	0	6.7427	8.1863	5.0068	10.8591	9.4218	10.4555	0.5375	7.6912	9.3371	8.7734	8.6409	7.9902	6.6023	4.8575	10.4222	8.3043	7.033	7.5342	11.9304	2.0567	5.7741	3.9065	8.7674	7.0337	10.4693	9.3696	8.5673	6.2974	11.6215	11.3243	5.9944	7.5982	11.447	12.3497	8.3329	10.0966	6.9571	10.782	4.6133	9.737	7.22	8.2063	9.1453
TCGA-91-6836-01	417	0	5.283	7.9388	2.8431	11.8149	8.4939	9.3893	0.9124	7.6471	7.1988	9.4249	7.3201	8.4233	6.8596	9.581	9.9174	7.6306	7.1697	4.2333	11.3822	0.9124	5.3153	1.467	8.2207	7.9206	9.9135	9.5768	8.8238	2.3132	8.9527	12.1902	6.9395	6.2309	10.307	13.1961	9.5584	11.8643	5.8334	10.4469	5.5909	6.6851	6.5692	9.0663	9.8031
TCGA-91-6840-01	372	0	3.5481	8.014	2.2692	7.6079	9.4002	9.442	0.8189	7.8819	8.0857	8.6974	8.2017	8.7775	6.0094	5.206	9.3953	8.994	4.8975	5.5361	11.7454	1.7191	7.1299	1.3381	8.6921	4.485	10.8086	9.2777	7.6135	2.9776	10.0204	11.3375	5.7268	8.7925	11.973	11.5381	8.9897	9.1063	6.3439	10.644	3.3461	7.7009	6.8047	9.9564	9.0759
TCGA-91-6847-01	842	0	2.9091	8.246	1.2454	10.7766	9.7605	8.8014	0.7531	7.8153	5.286	9.3391	7.808	7.7495	4.7014	6.9018	10.1679	8.5919	7.2072	4.954	10.2835	1.2454	3.5809	0.4251	9.0825	6.1687	11.4534	9.2767	8.3901	0.7531	10.0201	12.8161	6.216	5.2613	10.695	13.3369	8.3945	10.3938	5.9622	10.8604	2.0301	5.0832	7.7969	8.4675	10.0067
TCGA-91-6848-01	224	0	2.375	7.6994	2.121	11.6261	9.175	10.0674	0	8.333	9.8277	9.1895	8.1458	7.9739	6.5668	6.2067	10.1431	7.1208	6.9679	6.922	11.7435	1.4195	3.5713	2.5909	10.0651	4.7528	11.4162	8.133	9.0597	3.0938	11.0285	12.1911	6.1736	6.554	10.559	12.756	7.6218	10.768	4.8398	10.2452	8.035	8.971	7.9545	8.2918	10.1323
TCGA-91-6849-01	35	0	7.1525	9.5564	11.8207	8.4105	10.428	10.9585	2.7141	9.1329	7.338	8.4246	7.692	8.318	5.5424	4.9557	11.0687	9.1812	7.4926	7.6179	11.0567	2.4461	9.4248	1.0788	8.6003	5.1031	11.0544	7.9731	7.9922	3.3073	11.9126	12.2227	5.1936	6.7219	10.864	12.4765	10.2316	11.4033	6.9725	8.7307	0	8.8043	6.1286	10.1855	6.6277
TCGA-91-7771-01	492	0	6.9052	9.2435	10.3749	10.219	9.1436	11.3296	2.478	7.9041	8.2219	9.8666	7.8101	7.3563	6.6744	3.4964	10.1539	7.9666	6.9119	6.4544	12.3304	3.2587	7.8101	6.0844	8.5072	6.5033	9.2917	8.593	7.7729	2.6189	12.3018	11.593	5.8615	7.4106	10.812	12.1545	8.7734	10.015	6.6084	10.5929	5.4166	9.8693	7.092	10.6859	8.4659
TCGA-91-8496-01	505	0	3.5658	9.5609	7.1492	10.1609	11.161	10.3336	3.2507	10.829	8.1024	8.8986	8.2761	7.5695	6.2054	2.9945	9.3493	9.633	6.5535	8.0512	11.1945	1.3498	8.2688	1.3498	8.6543	7.026	11.1724	8.6899	8.7943	2.8471	12.1233	12.6152	8.6007	7.2332	11.596	12.9349	11.1197	11.6626	6.7004	9.9204	3.4682	8.8631	7.5458	7.5812	8.632
TCGA-91-8497-01	434	1	3.3599	9.2526	5.6433	10.0785	10.084	9.9444	0.7328	9.5847	8.622	8.5067	8.0825	8.8876	5.8887	3.8973	10.3395	9.9845	7.5188	7.506	10.9591	1.2165	9.1216	4.2879	9.1386	6.9785	10.8133	8.7765	7.6069	2.7985	12.5765	12.0829	7.7063	6.8269	11.643	12.1726	9.4836	10.7168	6.4547	10.8302	2.7985	8.3487	7.3561	9.3799	8.7895
TCGA-91-8499-01	36	0	2.1841	8.7258	5.836	10.5195	9.6926	10.0574	1.9832	8.226	9.0714	10.7438	7.1275	6.5866	5.2337	3.3018	10.011	8.4371	7.4835	5.1452	11.0671	0.6698	5.0139	2.3604	9.0083	8.4783	10.2184	9.8806	9.1561	4.8163	8.3299	13.7675	5.3636	6.0569	11.131	13.3511	8.5753	10.2718	7.9911	12.2883	3.9267	8.8288	9.5383	7.1457	10.7932
TCGA-91-A4BC-01	44	0	6.7642	9.1896	8.1346	9.5184	9.3811	10.4777	2.4775	8.5432	9.1032	8.7716	7.8746	8.4919	4.8286	3.813	9.8609	9.2167	7.7974	6.3205	11.0948	1.5647	6.9279	4.8286	9.3482	2.2976	10.9963	8.1312	8.4167	2.0921	10.0352	12.5274	5.786	6.9201	10.849	12.4305	9.9077	10.5561	4.1142	10.6657	5.3511	9.0755	7.7401	9.3235	9.0185
TCGA-91-A4BD-01	603	0	5.2073	9.6807	8.7501	10.2459	10.684	10.2553	0.9082	9.8666	7.4622	8.9242	8.6498	8.2201	0.9082	7.6502	9.2492	10.122	7.2287	6.8898	11.7914	0	8.518	1.86	8.9164	8.6017	11.0117	9.2698	7.7651	3.5261	12.9952	12.8643	7.935	7.2707	11.205	13.5883	10.323	11.0522	6.3972	10.6046	2.6522	9.2113	8.1506	8.4327	8.9059
TCGA-93-7347-01	683	0	2.7714	9.0343	8.1955	9.4534	10.343	11.0907	2.2884	8.5761	8.9822	8.954	9.0278	8.2201	5.8117	7.5773	10.3358	8.8643	7.4454	6.4695	11.6267	1.557	7.1966	4.8948	8.7149	5.4761	11.065	8.7557	7.9704	2.8512	13.8985	11.7974	8.2164	6.9424	11.141	12.8134	8.9876	10.25	6.4598	10.9648	3.7476	9.0795	6.2912	8.8571	8.9359
TCGA-93-7348-01	531	0	5.0884	8.2796	7.7303	11.0452	10.448	9.1029	1.8647	8.8681	8.0244	8.4528	8.6718	7.8257	5.2474	4.2351	10.722	8.8056	6.8031	5.6446	10.9224	1.8647	7.7392	3.688	8.138	7.0846	11.0528	9.0427	8.6025	3.957	12.8379	11.7217	8.258	7.5023	12.01	12.2227	10.3941	10.8886	5.8694	11.8738	4.1302	9.0208	6.4838	8.493	10.1093
TCGA-93-8067-01	186	0	8.7699	8.3513	11.1656	10.5212	9.957	9.39	0.891	8.3287	7.7069	8.5992	7.9473	8.6024	5.6271	10.4723	9.8237	8.0625	6.789	6.0407	11.4091	1.582	7.0632	0.3615	9.2068	6.79	11.8466	9.0357	7.3544	0.891	11.8429	11.2704	6.5827	6.8011	10.723	13.459	9.5422	12.9599	7.1264	9.6828	4.4458	7.573	8.1461	9.6048	7.5944
TCGA-93-A4JN-01	718	0	4.7647	8.9335	2.4782	9.9951	9.8369	9.698	2.2451	9.3943	7.3943	8.0081	8.796	8.7198	5.0077	6.9832	10.5169	9.5634	7.6757	5.879	11.0888	1.9669	5.0445	2.2451	9.1548	5.7379	11.5234	11.1911	7.6945	3.3425	12.3436	11.637	6.4504	6.4708	11.233	11.2554	9.7477	10.051	6.7908	9.859	3.2822	7.8482	6.4435	8.9806	9.5297
TCGA-93-A4JO-01	33	1	7.0474	9.4742	1.6286	10.7042	9.2888	10.5125	1.6286	8.0677	9.8113	8.4506	8.1991	8.6868	4.9936	4.1055	9.8256	9.5557	7.5226	6.2481	11.3916	0.6069	5.6635	3.1449	9.3351	3.3061	11.7151	9.3433	8.2346	4.015	10.4142	12.1736	6.1301	8.3408	11.089	13.6151	10.3293	10.9267	5.3487	9.9985	4.6168	8.7283	7.1834	9.5212	9.3339
TCGA-93-A4JP-01	578	0	2.9555	8.8494	3.8193	10.7148	9.8094	10.7083	3.0276	8.6729	7.7271	8.6498	7.8225	8.1993	5.3902	3.3422	10.5797	9.2478	6.6194	7.8488	11.2265	0.8439	7.9322	3.692	8.3789	6.0742	10.403	9.9906	7.3986	4.0788	12.3241	10.5752	6.1808	6.5968	11.702	11.8177	9.2543	10.201	5.9682	10.9685	6.3181	8.6151	7.731	9.0967	9.9661
TCGA-93-A4JQ-01	526	0	3.4629	9.4854	7.0227	10.1903	9.1655	9.7092	2.0184	7.8827	9.2002	8.9973	8.9408	8.9561	5.3146	6.5252	9.9135	9.5564	6.2459	7.2858	11.4334	2.2996	6.5115	4.3657	9.2339	4.4829	10.6959	9.2852	8.2248	2.5348	11.6704	12.2404	6.9122	6.6987	10.279	12.9279	9.6447	10.595	5.6387	10.6296	4.7632	8.2395	7.9424	9.8931	8.9497
TCGA-95-7039-01	1272	0	5.8965	8.4612	5.478	9.4996	10.533	9.6686	2.7472	9.6124	8.4587	7.8247	8.7729	7.7162	4.8147	9.4838	9.7061	8.5157	8.2433	4.3923	10.8909	0.7776	7.726	1.2801	9.1447	4.6602	11.4084	9.7877	8.7054	1.2801	13.6445	13.0814	7.9838	7.3626	11.954	13.6659	10.4426	11.9078	4.3923	10.3279	4.7395	7.8957	7.5392	8.7395	9.3978
TCGA-95-7043-01	503	1	3.0098	7.9167	12.0653	9.6547	9.4709	9.9888	2.2985	8.972	6.9854	7.7162	8.6007	7.9986	5.7629	1.7448	8.7864	8.8117	8.23	6.5414	9.9686	0	8.4504	0	8.7709	5.538	10.1987	7.4959	9.1922	4.0598	12.1408	12.4023	6.7496	6.5589	12.262	13.9494	9.1942	10.8036	1.3605	7.7	3.9174	8.6066	6.2378	8.4375	5.4372
TCGA-95-7562-01	87	1	1.6979	7.87	6.5532	12.2416	10.536	10.1065	1.2305	8.4997	9.1445	9.1709	6.9169	7.8561	6.2556	4.3111	11.3764	7.6523	6.1184	7.2302	11.6027	1.2305	3.3186	1.483	7.9243	8.2456	11.2966	10.0311	8.6045	3.8545	10.6436	12.2594	5.8911	7.0694	11.593	12.27	9.2895	10.5487	5.7418	10.615	2.0596	8.2605	7.6523	6.3774	9.5901
TCGA-95-7567-01	568	0	4.5021	8.3717	5.6094	8.4723	9.1614	9.8782	0.8828	7.9914	9.1279	8.3345	7.5201	8.7424	5.7136	8.1752	10.4003	7.7277	7.7611	5.6803	11.2378	0.8828	6.7269	2.9011	9.4969	3.7417	10.8926	10.1955	8.8297	0.8828	11.154	13.1121	6.4855	6.6195	11.207	13.4454	8.8466	10.996	4.7484	9.5379	4.5021	7.9426	6.5987	9.2427	6.5257
TCGA-95-7944-01	377	0	9.4431	8.7259	1.3011	10.4326	8.9623	9.7817	2.5257	10.382	9.8506	8.4922	9.0967	8.6238	5.547	6.6624	9.5307	8.3489	7.551	6.7353	12.0547	2.4309	6.582	3.444	10.2512	0.45	11.1748	8.1307	9.9363	0.7925	13.1211	13.0515	5.4537	6.2347	10.119	14.123	9.4302	10.8665	5.3795	10.0093	2.6146	8.7953	8.2413	7.4888	8.106
TCGA-95-7947-01	477	0	2.4813	8.2602	7.2905	12.0054	10.048	9.9386	0.9387	7.6436	8.3893	8.4423	8.05	9.0389	7.0388	4.923	9.8948	8.7068	4.6315	9.2128	11.4163	2.02	8.986	1.907	8.1246	2.6311	10.3908	10.3242	9.0548	6.41	10.952	11.4586	4.2268	7.8371	10.826	11.6631	9.7498	11.2686	6.9091	11.7705	4.2031	9.0229	8.5685	9.1525	11.3647
TCGA-95-7948-01	476	0	1.9857	8.9332	7.2878	7.7157	10.599	10.5451	0.7294	10.11	5.8962	8.356	6.561	7.8758	5.2544	7.197	9.1425	10.0542	7.908	4.3668	11.108	1.4031	11.077	1.5723	9.3782	5.1107	11.2981	8.5722	9.2249	1.7236	11.0968	13.6915	6.736	7.8534	12.087	13.4555	11.3463	11.7489	6.6676	10.6459	3.0421	6.4074	6.3269	9.3163	6.5958
TCGA-95-8039-01	830	0	3.6059	8.2529	8.939	10.6753	9.7668	10.9348	1.1084	7.9744	7.6697	8.5078	9.0751	7.9041	5.291	9.492	10.3058	9.0194	6.0431	7.2266	11.4307	1.5493	6.0302	3.3021	9.3157	2.5873	11.0859	9.2414	7.7856	3.8178	12.078	12.0933	7.2412	6.9262	11.403	12.9727	9.4417	10.4258	5.2767	10.4844	4.1664	8.6817	6.3009	7.5239	8.6363
TCGA-95-8494-01	84	0	3.909	9.0722	8.2068	11.5086	10.258	9.2758	0.7113	9.2518	9.3087	8.4124	7.9069	8.3934	5.5899	10.8735	8.4835	9.351	7.3381	5.4359	10.9748	1.1857	5.6276	0	9.887	1.8276	11.161	9.3552	9.351	2.0658	13.1142	14.5191	8.6392	5.6276	10.341	13.7518	10.798	12.0514	3.565	10.4564	3.7126	6.3898	7.3018	8.8768	8.4706
TCGA-95-A4VK-01	651	0	10.248	9.9566	8.535	9.3553	10.465	9.6198	2.5559	8.8631	7.3047	7.6055	8.0447	7.982	5.68	7.5763	10.5345	9.6742	6.3736	6.7839	10.85	2.671	5.7206	2.671	9.1648	5.8726	11.0866	10.13	8.1868	4.0546	12.6134	12.6935	6.5875	7.336	10.477	12.8125	10.424	11.0308	5.3582	9.8791	5.2143	7.4611	6.9137	8.975	8.0447
TCGA-95-A4VN-01	553	0	3.7555	9.0846	8.0699	8.8729	9.5336	11.5816	0.5491	7.9577	9.8713	8.6972	8.5535	8.8058	5.8573	10.376	10.783	8.5938	6.2719	6.4579	11.572	1.2567	6.0102	3.4841	9.0809	2.6076	11.0955	9.2496	8.8191	3.3595	11.2384	13.2255	7.274	7.4939	10.332	13.1029	9.0165	10.7566	4.1436	9.6662	4.6488	8.9025	7.3039	9.7043	8.3918
TCGA-95-A4VP-01	605	0	7.2072	9.3407	8.6148	9.512	10.136	11.0328	3.6475	9.6215	8.2291	8.3898	8.5578	8.701	3.6475	4.4573	12.5623	9.8778	6.9762	7.9676	10.6699	0.6081	8.1313	4.8987	8.5598	3.9706	11.3393	9.978	7.0955	3.1477	11.6391	13.58	6.7886	7.0788	11.155	12.5523	9.9291	11.0607	4.2343	9.6217	4.4224	8.946	7.4428	8.926	8.0679
TCGA-97-7546-01	1285	0	3.5158	8.3448	3.3778	10.8255	9.5696	9.4509	2.2183	8.6148	7.2293	8.4149	8.711	7.5003	6.1378	3.0543	10.0066	8.2391	6.4343	6.6264	11.1691	2.0467	6.8229	3.0543	7.3789	7.9534	10.8812	9.4194	8.1358	3.5158	12.0539	10.3276	7.1221	6.8751	10.654	11.0316	8.9551	9.6985	6.4621	10.693	2.8606	7.7305	6.3558	7.9258	9.0448
TCGA-97-7547-01	1965	0	6.3021	8.3262	0.5238	10.9991	9.7345	9.1414	0.9072	7.8942	6.5993	8.9619	9.2398	7.1535	5.8445	1.4598	10.7138	9.4771	7.2248	6.1129	10.8839	1.4598	7.5903	2.0228	9.2166	8.001	11.1468	8.5783	8.0613	2.4267	13.4028	12.1569	7.3675	7.8422	10.722	11.9252	9.7352	10.9127	4.46	11.9088	0.9072	7.9761	6.9387	9.4789	9.4275
TCGA-97-7552-01	1932	0	1.5161	8.2423	8.2859	11.5407	9.7527	10.037	0.5509	7.6548	8.4316	9.14	9.5794	7.9043	6.6712	7.4734	10.105	8.1765	6.3451	5.6451	12.0677	1.2601	8.3755	1.9223	11.7954	3.5479	10.3191	9.7888	7.8266	4.8761	12.3386	10.5728	6.1822	7.9043	10.53	11.7275	8.4153	8.4541	6.0159	10.9185	4.9647	9.2343	7.6575	7.9484	9.9213
TCGA-97-7553-01	1870	0	5.6599	8.4881	3.9342	11.4828	9.8832	11.969	1.6926	8.123	8.8574	8.9841	7.9946	8.0046	6.7207	3.4359	10.4156	8.715	6.5784	8.5076	11.916	1.4781	7.4632	4.4874	8.935	3.6043	10.6364	9.3237	8.4444	8.1681	12.0428	11.0103	6.1426	8.513	11.242	11.7207	8.3587	9.2054	5.8836	11.0324	3.1026	10.0959	6.7146	8.066	9.848
TCGA-97-7554-01	775	0	1.3224	7.4966	1.8079	11.6868	9.0355	9.6643	0.5853	7.2135	9.0205	9.0021	7.4353	7.7821	6.9951	7.5969	10.3855	6.9196	5.7228	5.8039	11.3272	1.0004	6.4016	2.7011	8.8925	2.46	10.3806	9.1593	8.6337	3.808	12.5765	11.9806	6.5164	7.2953	11.959	12.1661	8.8849	10.2919	4.2486	11.2147	4.4601	8.0829	7.7656	7.8081	9.6117
TCGA-97-7937-01	564	0	5.3353	8.3037	0.8508	7.9216	9.3005	9.3599	0.8508	7.9421	7.2212	8.2871	8.207	8.209	5.3209	5.937	9.8478	8.9424	5.6787	3.6123	10.8664	1.3824	7.7465	1.3824	8.9174	2.6376	10.8164	9.0687	8.7884	3.5168	11.5774	11.7683	6.3173	8.5291	10.683	13.7592	9.3014	10.749	3.8337	11.5259	2.2066	7.0397	5.7342	9.5505	9.7244
TCGA-97-7938-01	18	1	5.0188	8.5203	6.4496	9.5697	9.4968	11.5031	1.492	7.6592	5.3456	9.0424	8.1363	7.5336	6.1143	5.807	10.1654	8.9236	6.823	7.3581	10.8561	1.492	7.2312	3.1465	8.1517	7.1414	10.8836	10.0851	7.8643	3.1465	12.9992	10.9544	7.2881	6.9145	10.936	11.9961	8.5391	9.7737	7.2078	10.5728	1.492	7.7105	7.5477	8.8386	9.7189
TCGA-97-7941-01	484	0	6.8995	9.3128	9.8691	11.158	10.281	9.8214	1.8431	8.4998	7.6133	8.39	9.7527	8.222	5.4279	5.8318	9.9892	9.5615	6.1846	5.7826	11.1355	1.7067	8.1679	4.5434	11.5895	5.395	11.3072	9.7249	8.0407	5.2057	12.8409	12.2392	7.0759	6.9266	10.805	12.7208	9.779	10.0604	6.4111	10.4082	5.2308	8.2126	7.6198	9.3561	7.3691
TCGA-97-8171-01	568	0	2.0319	9.7889	3.9896	10.1891	11.023	10.1285	0	10.011	7.0956	8.5522	4.4264	7.8985	5.688	2.5297	9.3904	9.5142	5.8544	4.5456	11.2576	0	9.0418	3.2366	9.8259	6.3103	11.8143	9.7755	8.9141	0.3572	12.832	12.5095	10.2087	4.7463	12.44	13.6094	10.0795	11.7214	7.2857	10.4086	1.9293	7.5398	6.2636	10.1984	9.5192
TCGA-97-8172-01	545	0	6.6203	9.3268	1.2161	9.6532	9.8913	9.6785	3.0259	8.9207	7.7224	8.8459	7.754	8.9834	6.2204	3.072	10.7599	10.0667	6.4332	6.8355	11.1551	1.2161	8.1585	3.3592	8.9706	6.4386	10.0966	8.6573	7.9224	2.5269	12.0062	10.7799	6.8023	6.964	10.943	11.7569	8.9652	9.084	5.6951	10.8818	2.9782	8.426	6.8659	10.0038	9.2655
TCGA-97-8174-01	164	1	6.157	9.1683	8.7898	8.8552	9.9338	9.5518	1.067	9.3295	6.8509	8.5422	8.3797	8.1722	6.0715	2.0041	10.2791	9.3403	6.0348	5.7452	11.0102	1.402	7.99	3.579	9.2748	5.9297	10.2586	7.7922	7.785	3.5456	11.9175	11.5692	7.7469	7.2176	10.662	12.0736	9.0309	10.6493	5.6201	10.5998	7.4476	8.862	7.3001	8.8346	8.0949
TCGA-97-8175-01	551	0	5.189	9.0357	6.1829	12.8172	10.048	11.2238	1.031	8.2163	8.8813	8.913	8.3574	8.4319	5.3818	9.1461	8.676	8.8557	6.4103	7.9505	11.0534	1.7802	4.6638	2.6363	9.6525	7.1971	11.3472	9.2965	8.4535	4.356	12.7523	12.9143	8.5571	7.4789	12.812	13.6	9.6178	11.2398	5.6044	10.6306	4.9173	7.6684	7.5205	8.025	9.2077
TCGA-97-8176-01	468	1	7.8599	9.4988	10.6777	9.8855	9.2326	11.3327	6.0661	8.4933	7.6764	7.9563	7.6999	7.8488	6.0915	8.9603	9.5176	8.3446	6.1588	5.6798	10.4738	1.4771	5.4336	2.5253	9.6644	4.5236	10.8687	9.2182	8.4465	5.2653	11.6929	13.6055	5.22	8.1008	10.599	13.7712	9.5995	11.6415	5.6877	9.642	4.8254	8.9893	7.7082	9.0077	6.715
TCGA-97-8177-01	499	0	5.761	9.1152	7.4801	12.3613	10.816	11.8417	3.454	9.0704	9.8828	9.1206	9.1383	7.7152	5.5966	4.7963	9.4099	9.1687	7.4027	7.0354	11.0704	1.6137	9.6951	2.976	8.4106	7.6745	11.5782	9.694	7.6721	3.4081	11.9293	13.1461	7.9814	7.3556	12.295	13.0683	10.7592	11.5913	6.3401	9.9133	4.1279	9.6077	7.4493	8.4062	8.0428
TCGA-97-8179-01	435	0	7.2802	9.5082	7.469	8.2334	10.269	9.4989	1.5559	8.4351	5.7109	8.6958	7.6583	8.1011	4.8355	8.754	10.9854	8.9826	7.2539	7.7737	11.1343	0.473	9.1904	3.2536	9.2615	7.6721	11.0628	9.293	8.6224	2.0372	11.5791	10.3752	5.3835	7.4563	11.829	12.4772	9.2076	11.3887	5.4618	10.0935	6.1467	7.6666	6.8514	8.6365	7.9001
TCGA-97-8547-01	657	0	4.0948	9.586	5.5768	9.7945	9.5123	10.0135	0.8205	9.0791	8.5813	7.7769	8.2748	7.7515	4.9121	10.7071	9.4567	9.3268	7.4089	6.4423	10.0629	0.8205	6.8376	4.537	9.3337	4.6732	10.5757	8.7248	8.1751	4.0948	12.913	12.9512	7.9023	6.0468	10.855	13.0965	10.2138	11.0914	5.8113	10.8558	2.8581	7.8068	7.4183	8.806	9.7348
TCGA-97-8552-01	626	0	7.4644	10.2094	4.9767	9.8803	10.562	10.0426	2.5553	9.9909	7.9402	8.4198	8.013	8.8517	5.6724	3.4271	10.0122	10.5452	6.3249	7.3129	10.9792	0.6869	8.7754	2.0175	9.3261	7.9361	11.6566	9.3693	8.1307	3.8495	12.2696	12.2161	7.7111	7.9855	11.15	12.1089	10.8374	11.1627	6.2595	10.3043	3.7219	9.1896	7.4291	8.2395	7.975
TCGA-97-A4LX-01	614	0	3.8716	8.9905	9.1045	11.3224	10.255	10.9965	1.2139	8.2839	10.0288	9.7617	7.6171	8.3137	5.6516	8.9189	10.5728	9.3499	6.7534	7.3755	12.2805	0.526	7.9401	4.147	8.8213	4.5506	10.7597	8.511	8.0278	2.0284	13.1499	12.1486	7.5811	7.8076	11.03	12.2441	9.6851	10.3609	5.5195	10.545	5.8843	9.4711	7.5167	8.7331	8.5889
TCGA-97-A4M0-01	652	0	4.2667	9.0799	8.2036	12.2827	9.7927	10.024	1.244	8.7384	8.9525	8.7088	7.7662	8.7839	4.803	8.1946	10.8891	9.8909	6.6857	6.6044	10.9936	0.5422	7.083	1.902	8.4493	6.0693	10.6964	9.3099	7.6949	2.4756	13.6354	11.8689	8.085	7.6432	10.495	12.5749	9.956	10.4935	5.7889	10.3637	2.885	8.1232	7.2609	9.4706	8.0256
TCGA-97-A4M1-01	601	0	4.2928	9.8875	7.1445	10.7759	10.39	9.2397	3.1427	10.031	6.7138	8.5896	8.5144	8.909	3.6157	3.2205	10.3417	10.4369	7.7969	7.7255	10.8982	2.1461	9.3258	2.6745	8.5221	7.104	11.5246	9.0674	6.9525	3.7789	11.8105	11.4593	7.9807	7.057	11.514	11.8342	10.3919	10.2071	6.5395	9.7959	5.292	8.1418	6.6907	8.9294	9.0301
TCGA-97-A4M2-01	624	0	5.4067	10.0565	7.0757	11.1291	10.617	11.6189	2.9529	8.8578	7.8767	8.8165	7.9769	9.0869	5.3736	3.4104	10.7546	10.3443	7.2269	8.8846	11.0782	1.2898	9.4814	3.6507	9.2893	5.0769	11.2102	9.3502	7.6776	4.0367	12.0735	11.8069	6.8107	6.9688	10.751	12.339	10.0565	10.3931	5.8658	9.985	4.8547	10.2418	7.5829	9.9559	8.2339
TCGA-97-A4M3-01	540	0	7.9385	9.4781	11.8422	8.5185	9.5539	9.2539	2.0125	9.1883	5.8933	8.0025	8.7066	8.6979	4.4082	5.6013	10.7141	8.8336	7.4565	6.7945	10.3307	1.5961	10.2509	1.332	9.2729	7.343	11.0258	8.3181	8.0194	0.5905	12.4449	11.53	4.3734	4.849	10.172	12.8385	9.5732	11.4382	6.6015	9.784	4.923	7.088	6.2356	10.3222	7.8558
TCGA-97-A4M5-01	634	0	6.5083	9.2876	9.8233	10.4706	9.9181	10.7166	2.3751	8.6569	7.6779	8.6621	8.3186	8.7091	5.2423	9.1044	10.3891	9.6423	7.0367	6.8035	11.4671	2.591	7.9176	2.2536	9.3351	6.263	10.9644	10.1899	7.3603	4.797	11.8748	12.3579	5.8037	7.6612	10.781	12.7876	9.3583	10.5804	5.3936	10.5384	6.0964	8.9866	7.4734	10.1591	8.1395
TCGA-97-A4M6-01	568	0	4.9824	8.6824	8.241	9.9368	10.31	10.5669	1.9035	9.3929	9.5795	8.7282	8.4514	8.1064	4.1986	5.559	10.9109	9.987	6.1441	6.8389	12.1482	2.3539	8.684	3.6348	8.4959	6.9742	11.2382	9.4894	7.5252	4.0069	11.556	11.7016	7.9525	7.6584	11.658	11.5	10.1992	10.3916	5.8029	9.6645	2.8868	8.5515	6.7352	9.131	8.5462
TCGA-97-A4M7-01	629	0	8.4563	8.9926	7.3752	9.7345	9.911	10.2483	1.4431	8.4073	9.1801	8.0769	8.6478	8.3802	5.3863	4.2516	10.1447	9.3345	6.7691	6.7296	11.2579	0.5157	8.5202	3.1961	8.7735	7.5173	10.9032	9.793	7.6374	4.5183	12.7362	11.6762	6.042	6.8057	11.775	11.7679	9.4418	10.5523	6.5585	10.4677	6.0514	8.876	6.6844	8.0279	8.968
TCGA-99-7458-01	747	0	2.314	9.5618	6.8605	10.1211	9.7692	9.5937	1.4088	8.1897	8.5268	9.9282	7.8314	7.6696	6.595	8.9208	10.7569	8.5813	6.2041	6.6435	11.719	2.1078	6.7361	3.8976	8.7089	7.9424	10.277	9.0189	8.511	2.5767	12.7054	11.3996	7.9731	6.8103	10.548	12.5553	9.3395	9.9678	6.4331	9.9868	4.5141	9.455	7.4658	8.4658	8.0705
TCGA-99-8025-01	1060	0	4.8903	9.0173	6.2663	8.9204	9.5047	11.2065	0.7367	8.0409	7.3005	8.6965	8.8544	8.8261	5.4033	9.9214	10.1124	9.115	8.3943	5.6125	10.6374	2.1151	5.7896	3.874	10.2183	3.3688	11.2415	9.5901	8.5592	2.874	12.5775	13.066	7.4726	8.7566	10.76	14.0334	10.2949	11.4226	6.2538	10.2772	5.4914	7.4117	7.2601	9.2371	8.0403
TCGA-99-8028-01	1118	0	2.7222	9.1858	4.5744	10.3777	9.9453	11.0982	0	8.6842	11.0185	8.7188	8.4559	8.0286	3.9674	10.301	9.9654	9.0114	7.1834	8.0161	12.0308	1.8416	7.4547	3.5566	9.436	3.265	10.8495	9.1944	7.3094	2.5204	11.8058	13.1713	7.567	6.7173	11.289	13.3598	10.0184	10.9437	5.1653	10.191	6.6069	10.2458	7.8596	7.4042	9.6249
TCGA-99-8032-01	44	0	9.2637	8.3694	8.1645	10.2625	9.3943	8.9994	0.8337	8.5475	7.6737	9.2284	8.0499	8.7286	6.085	4.6804	11.6297	8.6295	7.7577	5.5079	10.7632	0.8337	7.4992	7.4992	9.017	3.6256	10.4178	8.9592	7.9416	1.9022	10.493	12.8814	6.7361	6.5317	11.155	13.455	8.886	10.4839	5.408	10.2431	4.9229	7.8319	7.4058	9.4152	7.6073
TCGA-99-8033-01	656	1	9.39	8.5058	9.0432	10.8023	9.6927	10.4308	1.9009	8.0527	9.0639	9.0443	8.4521	7.8449	5.4584	7.7976	9.2489	8.7892	7.4632	6.9802	11.8817	2.9337	4.5878	1.5622	9.0271	7.919	11.0481	8.9311	8.896	2.1749	12.0344	12.4278	6.4929	6.5053	10.732	13.5057	10.471	11.3535	2.858	8.3261	4.7004	7.8978	7.469	8.5166	6.3619
TCGA-99-AA5R-01	658	0	3.9688	9.3133	3.1044	10.697	9.9774	10.8187	3.5678	9.4919	8.5039	8.429	8.7605	8.7731	5.0218	5.0924	10.8095	10.0516	7.441	7.4419	11.4479	1.0606	8.1871	1.3943	8.9434	3.5678	10.688	9.1886	7.3895	4.4349	11.948	12.0862	6.6332	6.1504	10.673	12.123	10.1165	10.1575	6.3934	9.9672	6.7971	8.8361	7.9126	8.5468	8.5819
TCGA-J2-8192-01	739	0	3.8595	9.1712	4.8089	11.3309	10.083	10.126	1.0883	8.3079	8.0761	8.6126	8.692	7.431	6.7192	6.3122	9.7342	8.2189	5.73	7.7329	11.1389	1.7015	7.0114	4.7294	8.4284	5.9144	10.2379	9.2701	8.4268	4.9204	11.6185	11.0505	6.2495	7.136	10.735	11.6007	9.3952	10.0252	6.0676	11.1518	2.6452	9.004	6.8979	7.6544	9.7399
TCGA-J2-8194-01	724	0	7.837	9.2869	9.0711	9.0379	9.6264	10.2112	1.8547	8.0319	7.6761	9.3557	8.2992	8.1254	6.6872	8.0796	10.1594	8.5895	7.8921	5.9526	11.0093	2.4225	7.9173	2.4225	8.5813	6.3748	11.0862	8.2484	8.3807	3.4052	11.3379	11.8627	6.2061	6.1092	11.09	12.7366	8.5747	9.8831	5.5748	10.4669	6.2398	8.7847	7.0662	10.0697	8.4775
TCGA-J2-A4AD-01	550	1	4.975	9.627	7.2092	10.106	10.473	9.6373	1.5993	8.1034	5.9491	8.7541	8.1078	8.1247	5.487	5.2459	9.2324	9.1523	7.2297	6.3537	10.2772	1.5993	7.5116	1.1493	9.9436	6.5291	11.1517	10.2848	7.5897	5.4064	11.2342	12.5026	7.4825	9.2246	11.746	12.6129	10.2239	11.6028	5.612	10.2442	3.7641	7.2328	7.7723	9.478	10.0184
TCGA-J2-A4AE-01	1079	0	6.1445	9.8722	8.5625	11.0343	9.6787	9.0401	0.6699	8.7307	7.3315	8.7759	9.105	8.7701	4.4387	6.1445	9.8965	10.4985	6.4761	6.6157	11.1253	1.7502	7.2225	2.361	8.5959	7.6451	10.7179	10.0802	7.8512	3.1182	12.6624	11.7577	7.787	6.5873	11.058	11.5425	8.9805	9.4769	6.7702	11.0217	2.361	8.0763	7.0848	9.0768	10.362
TCGA-J2-A4AG-01	988	0	5.84	8.4533	3.056	9.8693	9.5999	9.5915	0.6441	8.1278	8.6451	8.9169	9.1026	8.086	4.749	8.807	10.027	9.3516	5.6098	7.0324	11.4558	0.6441	6.8627	2.6006	8.2576	6.9107	11.8298	10.4	7.6856	2.4601	12.3416	11.424	7.0397	7.3985	10.622	12.9196	10.5768	10.7267	6.2452	9.9868	3.8017	7.8808	6.4633	8.4038	7.7013
TCGA-L4-A4E5-01	578	0	6.0949	8.3959	7.9009	9.4991	9.9357	9.2588	0.5411	7.6495	7.331	9.0078	7.3447	8.8868	5.4964	9.0305	9.7965	9.3421	6.7784	5.4901	10.1415	1.8993	7.7227	2.0654	8.5102	2.0654	11.914	9.9059	8.6572	1.7116	12.2147	12.2698	8.5475	7.4796	11.437	12.5409	9.7051	10.1839	3.7319	11.6562	4.8229	7.1779	7.2093	10.2694	10.4227
TCGA-L4-A4E6-01	435	0	4.0016	9.1032	3.7264	11.851	10.194	12.8176	1.9188	9.1159	8.7814	8.6229	8.6729	8.6491	4.8485	4.9814	10.8674	9.9479	6.4008	9.0029	11.5155	2.1168	7.7151	0.638	8.8589	4.4683	10.9601	9.1278	7.6764	2.7141	13.2905	10.8187	7.2767	6.7296	11.105	12.1577	9.3246	9.8626	6.1055	10.2006	6.2601	11.968	8.5152	8.0357	8.6551
TCGA-L9-A443-01	193	1	6.8384	9.6622	9.3534	9.8893	10.189	8.9681	1.4248	9.0059	7.4364	8.6669	8.2031	8.8342	1.929	9.9626	9.8867	9.1616	6.9168	6.4722	10.5672	0	6.8664	2.4575	8.5615	7.2591	10.8817	9.2901	7.438	1.6988	13.9916	12.6009	8.4385	7.6941	10.736	14.154	10.1489	11.4891	7.0427	9.9779	5.4673	8.6887	8.1356	8.6183	8.3837
TCGA-L9-A444-01	307	0	3.9474	9.6625	1.7128	8.9254	9.8379	10.2168	0.815	8.4373	9.9299	8.5952	7.5646	8.9411	4.4263	10.0563	10.4679	9.5762	6.4266	6.2902	11.5647	1.7128	7.1976	3.7169	9.0932	4.0167	11.0629	9.4202	7.6705	2.6588	12.6361	12.815	9.0276	7.04	12.213	12.5174	10.0072	10.0839	5.6099	10.9016	5.5873	8.7147	8.3146	10.5731	8.8491
TCGA-L9-A50W-01	442	1	5.9224	8.5766	8.6831	8.7487	10.458	10.3163	2.8078	9.813	9.1299	8.6185	8.2906	9.4378	5.1503	7.3359	10.4375	10.3348	7.9423	3.7322	11.9444	1.0877	8.5595	5.6153	9.3894	6.6923	10.8487	8.9667	8.0986	4.7285	13.6821	13.2863	8.2252	5.8678	11.405	12.263	10.7256	11.1415	6.639	10.441	4.2485	7.2425	6.034	9.9582	6.3843
TCGA-L9-A5IP-01	58	1	8.1657	8.4341	9.4293	10.1586	9.2425	11.0698	7.4725	9.038	6.9955	8.956	7.5821	8.3782	7.233	7.9032	10.2574	8.6191	7.4839	9.9878	10.9747	2.9209	6.4117	1.4609	10.5228	4.5179	10.9455	7.1641	8.6335	2.6458	9.6389	13.2702	6.1521	6.4117	10.171	13.3113	10.6284	11.2258	3.9091	10.4831	4.3421	9.0158	8.7438	9.7532	9.0698
TCGA-L9-A743-01	664	0	4.3475	8.6923	7.271	10.2217	9.2892	10.8242	1.2729	8.3989	9.2626	8.7723	8.794	8.6807	5.963	10.1582	10.4217	9.0659	7.6007	7.318	12.034	2.2561	6.9569	4.1313	8.9673	3.7811	10.5453	9.3437	8.4074	3.7811	12.4708	12.5244	8.2007	7.3772	10.768	12.822	9.5854	10.3992	5.9739	10.7811	5.0283	8.8804	7.4838	10.0785	9.3952
TCGA-L9-A7SV-01	565	0	5.634	9.0236	1.7063	6.8061	10.536	8.295	1.7063	8.214	7.2036	7.8142	8.3627	8.8528	6.9183	7.1884	9.6707	7.9458	8.854	4.0315	9.7219	1.0919	7.8429	2.0064	8.2204	3.7493	11.3038	9.5904	8.5902	1.8641	12.3098	11.9825	7.1274	8.3105	11.763	13.4335	9.9409	9.8039	7.3932	12.1898	6.8967	6.7963	5.5552	10.0367	10.1574
TCGA-L9-A8F4-01	476	0	7.2636	9.3492	4.6824	10.6424	9.8082	10.0573	0.877	8.9293	10.5212	8.5278	7.3985	8.9657	5.3031	2.4857	9.1502	9.6383	6.9958	7.2374	11.0715	1.6282	7.4683	4.1137	8.9389	2.6865	11.0326	8.8876	8.1378	4.9202	11.4026	13.9036	6.832	7.5703	11.075	12.4603	9.6277	10.9813	6.9827	9.5247	3.8464	9.0352	7.8389	9.9503	7.8546
TCGA-MN-A4N1-01	827	0	4.8077	9.208	7.3119	8.6464	10.666	9.2608	0.5013	9.6674	7.1435	7.8241	9.6801	9.0025	3.4001	9.8663	9.6647	9.0326	7.3793	5.4314	10.1709	1.4125	7.8963	2.9351	8.6299	5.324	12.6979	9.7973	8.3666	8.9083	11.0762	13.4255	7.2581	6.978	10.676	13.0811	10.5202	11.2226	3.611	11.5506	4.2056	6.4905	6.6058	10.4396	10.6256

TCGA-MN-A4N4-01	1175	0	7.0773	8.4285	2.2761	10.0267	8.9636	11.8971	1.9571	8.2345	8.007	8.4021	8.0434	7.9899	5.0735	5.4357	8.9733	8.8359	7.1691	5.9187	10.2146	1.2877	5.5584	2.9498	9.4092	4.1551	11.1973	10.489	7.7143	5.6161	11.7487	12.9166	5.5136	7.4974	11.785	12.4413	10.3343	11.2946	4.0334	10.3149	4.726	8.3521	7.6741	10.057	9.7747
TCGA-MN-A4N5-01	84	0	3.5878	9.6598	5.927	9.8385	9.9093	11.0336	1.3645	8.3535	8.8396	9.0662	8.9377	9.0893	4.9688	10.7345	10.3199	9.8625	7.0999	5.3009	11.907	0.8379	8.75	2.053	9.437	5.9639	11.7853	9.2977	8.1054	1.3645	10.4318	11.8201	7.7884	6.4724	11.508	12.4936	9.3325	10.9387	5.7693	10.6045	3.9235	10.1468	6.535	9.4063	9.6945
TCGA-MP-A4SV-01	2620	1	3.8759	9.0737	6.9457	10.4009	9.5032	9.9145	1.1907	7.7673	9.4229	8.7774	8.0945	8.8107	6.0901	9.8471	10.0446	9.3992	7.3031	6.1132	11.237	0	6.7704	3.1203	9.1539	4.7591	10.7809	9.7645	9.0165	1.834	12.02	12.3343	6.7647	6.0901	11.087	13.0676	9.4353	10.4208	4.8897	10.0968	3.5988	8.3159	6.7361	10.1324	8.5948
TCGA-MP-A4SW-01	1778	1	5.0784	9.0328	9.8012	11.5755	10.92	11.799	2.4734	9.2396	8.8409	10.2283	8.9084	8.9395	5.0588	9.7364	9.7555	9.5992	7.3198	6.8671	11.3583	1.7123	8.3453	2.5871	8.8181	3.2011	11.516	9.7279	7.9742	5.039	12.6391	12.3967	7.8487	7.8487	11.566	12.352	10.5292	10.706	4.331	11.0232	3.6304	9.7356	6.797	10.0201	7.6602
TCGA-MP-A4SY-01	1501	1	6.5086	8.9232	8.0194	10.2528	9.8812	11.1086	1.2009	7.8883	8.0107	9.3699	8.2746	7.9778	5.1376	7.648	9.9152	8.4075	6.8745	6.7649	11.0136	2.011	7.3091	3.2718	9.5656	6.8052	10.9155	9.7386	8.7322	2.2917	12.3031	12.0711	8.8025	8.029	10.658	13.2066	9.6941	11.1338	4.7536	11.2811	3.3957	7.8831	7.1729	9.9834	10.241
TCGA-MP-A4T4-01	2617	1	2.4195	9.3142	3.5989	10.3951	10.472	10.3251	0.9758	8.556	9.609	8.2481	8.3478	8.9427	5.7848	9.7856	9.4023	9.1703	7.2747	6.8278	11.7281	1.7726	7.3522	3.6553	10.0616	3.415	10.9838	9.1173	9.2156	3.2042	11.8431	12.2542	7.5734	6.7131	10.553	12.8549	9.2156	10.8606	5.5067	9.8892	4.1238	8.9666	7.2088	9.5342	8.6654
TCGA-MP-A4T6-01	1790	1	3.4438	9.6994	5.9822	9.3832	11.371	9.1652	2.1022	10.635	8.9255	7.4559	8.4608	8.8544	4.0004	4.2428	10.5772	11.052	7.0506	6.3943	10.8284	0	8.9663	2.6144	8.9332	7.4224	11.8451	10.5727	7.9437	2.9916	10.3062	12.0008	8.8498	7.3368	11.968	12.0335	10.9198	10.15	5.905	9.2024	2.6981	8.387	6.1989	9.5796	7.5462
TCGA-MP-A4T7-01	167	1	10.822	9.3874	7.9998	10.7847	9.7338	10.8586	3.9595	8.4741	8.6489	7.7299	8.7053	8.7548	5.1279	8.7997	10.2372	9.2108	6.7003	6.4467	10.6625	2.4359	7.1469	1.467	10.1786	5.091	10.9817	8.4196	7.5582	4.1507	12.2519	13.4991	6.1525	6.4722	10.504	13.3868	9.6936	11.1995	5.3153	10.152	3.9998	8.643	7.5136	10.2711	7.2756
TCGA-MP-A4T8-01	161	1	7.0764	8.2843	5.5215	9.3925	10.117	9.1106	0.5058	8.4825	7.2202	9.3587	6.9885	8.5818	5.8712	8.2785	10.7015	8.7739	7.687	6.3581	10.8657	0.5058	8.5786	2.7823	8.9472	3.6734	11.7487	8.6737	8.4337	2.5944	9.9222	13.147	7.2174	7.1928	10.924	13.0157	9.5438	11.0355	4.6157	9.8247	4.5654	7.0215	7.9242	8.4337	9.0582
TCGA-MP-A4T9-01	1265	1	3.227	8.4408	0.8452	11.5177	9.8313	11.1524	1.5807	8.7587	7.3645	8.7861	8.5658	8.0928	5.2341	9.2259	10.2635	9.2721	7.2551	7.487	10.8713	0.4836	6.2149	3.0987	8.938	5.8468	11.2469	9.1641	8.3433	3.0987	12.3171	11.8555	8.0587	6.0939	11.241	12.2264	9.2656	10.4364	5.7217	10.5237	4.5175	9.3375	7.9583	8.4174	9.15
TCGA-MP-A4TA-01	950	1	9.8423	8.9549	11.059	10.7878	10.464	10.4114	3.1825	7.7605	8.9013	9.2673	8.9537	7.9342	6.1308	6.2038	9.4846	8.6929	6.9391	7.1528	11.5069	3.5239	8.0107	2.7345	9.592	4.9492	11.8344	9.7438	8.3655	3.9951	10.8291	12.6721	6.3104	6.6949	10.945	12.5198	9.4026	10.67	6.1556	9.5668	4.1344	8.65	7.9365	10.4255	9.8934
TCGA-MP-A4TC-01	74	1	6.7839	8.575	4.8456	11.4806	9.5557	12.2366	1.7114	8.4889	9.4376	8.9778	8.5681	8.7559	5.6213	8.9869	9.4032	8.5454	7.8955	6.2717	11.5169	1.2418	5.5944	3.1267	9.7686	0	11.1356	9.029	8.7574	4.6763	12.0082	12.7568	7.8445	7.7951	10.676	12.9168	9.1621	10.1136	4.4245	10.5359	3.2	8.145	7.1547	9.6535	10.8419
TCGA-MP-A4TD-01	307	1	6.6659	9.1716	10.387	7.3587	9.6508	9.2681	3.3033	8.1324	7.3413	8.0982	7.5605	9.0707	5.3373	7.8589	10.7524	9.2796	7.6414	5.8926	10.1226	0.5781	7.6763	5.1893	9.1124	4.5037	10.7003	9.3918	7.968	2.1538	10.9922	12.9508	7.1342	6.6659	11.41	13.3285	9.9115	10.6446	6.2274	10.1734	4.7349	8.35	7.3972	10.3395	8.0017
TCGA-MP-A4TE-01	896	1	9.9773	8.711	11.7457	11.4499	9.6739	9.5093	1.6792	8.295	5.8283	8.9065	8.0468	8.0053	5.7746	5.939	11.2727	8.5423	8.2517	6.4902	10.0263	0	8.6475	6.9531	9.248	4.7307	10.5222	8.7892	8.0706	3.5865	11.3145	13.8088	5.376	7.5427	11.596	13.8571	9.924	11.0035	4.4104	10.2547	2.6522	5.9698	8.3197	10.7522	7.1848
TCGA-MP-A4TF-01	336	1	9.8599	8.0864	8.2843	11.3768	10.708	9.0442	2.0917	8.7361	7.4172	9.994	7.3801	8.9209	4.885	8.4279	9.0553	9.4496	7.3108	5.424	11.9816	2.5592	4.8031	0	9.7092	6.7746	11.578	8.038	8.3863	2.3443	10.4426	11.5236	7.2927	6.1193	11.051	12.1767	8.6551	10.3024	5.9771	9.4646	2.0917	8.1541	7.2624	8.5491	7.8519
TCGA-MP-A4TH-01	741	0	7.9582	9.4911	7.3148	8.7681	9.6142	9.7672	2.3649	9.0078	8.3673	8.5386	7.7514	8.7294	5.2616	6.8789	10.4897	10.1505	7.2614	6.9146	10.8833	2.0802	7.2287	1.2533	10.0303	3.2176	10.5863	8.4153	7.9009	1.2533	12.7754	11.4613	7.1973	6.9344	10.563	12.1524	9.175	9.8517	5.2616	10.3483	3.067	8.5314	7.5637	8.9793	7.9475
TCGA-MP-A4TI-01	429	1	3.6178	8.9411	4.3559	10.3372	9.9252	10.7632	2.1978	8.6248	11.1075	9.2169	8.2353	8.8382	4.9888	5.3568	10.0733	9.1112	6.4082	7.6391	12.9056	1.018	6.4017	4.163	9.5256	4.2818	11.2101	9.6328	7.8526	3.6178	11.023	12.5099	6.7634	7.628	10.257	12.6692	9.3372	9.8285	4.1212	9.5788	6.0687	9.4248	7.9898	8.0014	8.9826
TCGA-MP-A4TJ-01	339	1	7.9703	8.715	7.1547	10.7325	9.3499	10.445	2.9976	7.7062	10.2319	8.9624	7.8929	8.4125	5.9162	2.6416	10.1646	9.2644	7.1895	7.9043	12.2207	1.856	6.6891	3.1474	8.4199	4.3983	10.3086	9.1359	8.0674	3.283	11.3598	11.9611	5.8079	7.6321	10.833	12.2694	8.7823	9.447	5.5365	10.2457	5.394	9.7605	7.6511	7.8553	9.1625
TCGA-MP-A4TK-01	582	1	5.2535	8.9906	4.0746	10.6716	10.003	10.7291	0.5804	8.6557	8.6809	9.1897	8.2993	8.6158	4.8695	10.1425	9.5798	9.456	7.2619	7.3209	10.9219	0.5804	7.5863	4.4788	9.3128	4.8197	10.9478	9.3505	8.3083	3.3097	11.1979	12.8503	6.863	5.9408	11.149	12.7795	9.641	10.9193	5.2535	9.9008	5.8446	8.4106	7.3484	9.453	9.9149
TCGA-MP-A5C7-01	2248	0	6.9904	9.7662	9.5136	6.2812	10.669	8.2476	2.111	9.3203	6.0621	8.1286	6.4135	8.5662	5.5308	3.7043	11.6262	9.5769	7.4764	4.0784	10.1628	0.5008	10.8296	0.8718	9.2861	6.8625	10.4567	8.8519	7.9269	0	11.5839	12.3135	7.8615	6.7446	11.722	12.5418	10.251	9.984	3.9945	10.0628	4.1711	7.7435	7.1716	10.2505	6.5411
TCGA-NJ-A4YF-01	2161	0	8.8117	8.9617	2.6933	9.1723	10.136	8.4749	1.1474	8.839	8.5038	7.5878	7.7953	8.2458	5.5599	1.1474	11.0712	8.6704	6.4614	6.5201	10.6881	0.6848	10.3279	0.6848	8.7438	6.2543	11.4439	6.9651	8.2996	2.2157	9.3667	13.4285	8.7682	6.2194	12.366	13.1332	9.2434	10.4407	5.7181	11.5978	4.5901	6.3859	7.7472	11.393	5.4236
TCGA-NJ-A4YG-01	2261	0	4.9005	9.0589	7.2004	9.5626	10.007	10.8643	3.8931	8.3643	7.1022	8.5535	8.8918	8.1464	3.51	8.3101	10.3489	9.1672	7.3515	7.0693	11.1522	0	6.835	3.435	9.1987	7.8612	11.3176	10.2249	7.8684	2.3339	13.184	11.4282	7.5147	7.4059	11.154	13.6386	9.6626	11.3911	5.6626	10.7078	3.6493	8.4923	7.1718	9.6647	8.6206
TCGA-NJ-A4YI-01	4	1	3.7098	9.0334	4.6536	9.9364	9.5653	9.7555	5.2704	9.1084	6.882	8.3924	8.3275	8.3924	2.816	7.8866	11.1815	9.676	6.8456	7.1458	10.2608	1.5917	7.329	5.1114	9.1334	6.9447	11.589	8.566	7.915	1.5917	13.0666	11.5753	8.1883	7.0063	10.253	12.2663	10.6881	10.3732	5.7011	9.6995	2.5109	7.8825	7.2228	8.1917	7.7805
TCGA-NJ-A4YP-01	50	0	5.7233	9.11	10.4204	8.9036	9.6194	11.1673	1.0117	8.0247	8.1422	8.5517	8.2806	7.9875	5.4321	10.2605	10.1796	9.0126	7.7684	5.6065	11.3366	2.4112	5.5306	4.6948	9.8241	7.4025	10.9436	8.9331	7.9904	2.2663	12.2522	12.7294	4.7773	6.7339	10.764	13.6381	10.1812	11.393	6.72	9.7923	4.8038	8.9294	7.3807	9.8042	8.213
TCGA-NJ-A4YQ-01	1432	0	8.0203	8.9237	8.8916	12.0013	9.4813	10.1385	2.0492	7.3886	10.1594	7.9304	6.9978	8.4093	6.091	4.2154	9.387	9.6721	7.8713	8.8825	11.5784	0	7.6118	2.9828	9.6754	4.3098	10.722	9.5804	8.355	8.2637	10.5574	12.3327	7.1837	7.0119	11.276	12.8663	10.0021	12.4972	4.9891	9.4428	3.5449	9.122	8.3906	8.6447	8.6739
TCGA-NJ-A55A-01	15	0	3.7267	9.1595	5.8316	9.6312	9.7206	9.4192	3.1486	8.2552	7.8774	8.3223	8.2245	8.9763	5.8316	4.6712	10.3588	9.484	6.3977	5.6378	11.2594	1.857	6.8737	1.4586	8.7164	5.1331	10.4098	9.9043	8.0546	3.819	12.0978	11.0135	6.3628	8.8481	10.69	11.5263	9.1215	9.8244	6.1836	9.9148	3.6281	7.3921	7.9309	7.9361	9.2658
TCGA-NJ-A55O-01	13	0	7.6308	8.9381	10.5773	11.6009	9.9426	10.1089	1.9698	8.6921	7.9491	8.8387	8.9016	8.1317	4.799	9.2856	10.3498	9.3137	7.3513	7.1596	10.8901	1.9698	7.085	1.1156	10.4392	5.4068	10.95	9.1779	8.0226	1.7371	12.5148	12.3562	6.7122	7.2872	11.145	12.7547	9.4832	10.1627	5.4459	10.8532	4.7053	9.9469	7.7372	9.0535	7.9046
TCGA-NJ-A55R-01	603	0	8.3213	9.5539	10.9138	10.0802	10.3	9.0696	6.7982	8.3917	6.6788	8.4065	7.693	9.2855	5.5139	6.7825	10.6452	8.9216	8.7691	5.7755	10.3344	0.9717	9.4898	4.1554	8.9514	6.2336	11.1411	8.4146	8.0646	3.3407	10.919	12.474	5.921	6.9931	11.375	11.9748	10.1228	10.7977	6.1772	10.7318	5.2662	9.1753	8.2238	9.7023	9.8051
TCGA-NJ-A7XG-01	617	0	3.0578	9.2155	8.7	11.2805	9.9252	9.7537	0	9.1884	6.066	8.2872	7.9822	8.3144	5.0468	7.0034	10.2859	9.7046	6.6419	4.1022	10.4535	0	8.8291	0	9.232	7.0787	10.8953	9.3782	7.4959	3.5838	12.3101	12.9649	8.7531	7.6017	11.086	12.8634	10.5408	12.0403	5.7942	10.3761	4.7603	4.9645	6.9774	8.6728	9.0792
TCGA-O1-A52J-01	1798	1	6.9923	9.4011	6.0143	11.4128	9.7899	10.0857	1.543	9.5802	8.2289	7.5662	8.6764	7.3505	5.7321	4.3319	10.2753	9.4164	6.1364	6.5615	10.8015	1.543	9.7821	3.2649	9.1497	7.1069	10.8673	8.7774	8.2313	4.1878	12.6578	11.7366	8.566	5.4137	12.428	11.1341	9.9138	9.7914	6.507	11.1021	2.8549	8.86	6.077	7.6268	9.6361
TCGA-S2-AA1A-01	513	0	2.9169	9.6254	8.9909	9.6967	9.6772	9.1239	4.7659	8.3612	9.0073	8.1698	8.7399	7.9956	5.3331	3.4367	10.8487	9.8602	7.1675	6.2514	11.1522	1.8563	8.0957	3.0369	9.852	1.2081	10.5955	9.0294	8.4521	3.0369	12.9687	11.7882	6.3503	6.3032	11.903	12.291	10.1693	10.3358	7.1813	9.9092	2.6419	7.7673	8.2955	7.9428	8.9008

id	futime	fustat	OXCT2	P4HA3	PAFAH2	PC	PCK2	PDE1A	PDE1C	PDE3A	PDE5A	PDE6D	PDE9A	PIK3CG	PIPOX	PLA2G15	PLA2G6	PLCB1	PLCB2	PLCB4	PLCD4	PLCE1	PNPLA4	POLA1	POLE2	POLR1C	POLR2H	POLR2J	POLR3G	POLR3K	PPAT	PPOX	PRIM2	PRPS2	PTGS1	PTGS2	PYGL	QPRT	RFK	RRM2	SGPP1	SGPP2	SMPD1	SMPD2	SMPD3
TCGA-05-4244-01	0	0	2.9966	6.4865	9.4899	11.0325	9.6849	5.3988	3.1176	4.8716	7.6456	8.8845	8.344	6.9405	4.3609	9.334	7.762	8.1449	8.8651	8.4587	5.9883	7.3077	8.2887	8.6133	6.5523	8.8576	9.9088	9.6658	6.6256	8.946	9.098	8.6688	7.9263	10.0985	8.5299	9.2743	10.4854	8.9962	9.1957	10.5198	9.6211	10.026	9.3588	7.9097	6.5307
TCGA-05-4249-01	1523	0	3.1777	6.3139	8.6768	9.5708	9.109	6.437	3.0149	4.793	7.5348	8.6915	8.9235	8.4298	4.6337	9.8085	7.0448	6.7361	9.2749	5.0668	4.9958	9.0715	8.1448	8.6688	5.4214	9.3699	8.8876	9.3358	5.0389	9.2809	8.3609	8.1316	8.3003	9.8568	9.8157	11.3192	8.6389	7.5124	9.3902	7.9178	9.0912	10.2384	9.8043	7.6155	7.7467
TCGA-05-4250-01	121	1	3.656	6.5317	9.5336	7.7998	9.5793	6.202	2.3159	5.0659	8.4409	8.871	7.5598	7.0764	4.6366	9.8036	7	5.8085	9.7451	6.867	5.215	6.0729	8.3747	8.8782	7.1624	9.0711	9.7896	10.188	7.3294	8.5355	9.7056	7.9447	8.2886	10.7562	8.5611	9.8842	10.4281	8.4897	9.2317	11.4828	9.2901	10.6456	9.2592	7.3733	3.9626
TCGA-05-4382-01	607	0	3.5569	8.0548	9.8062	8.4498	9.706	7.2088	2.5326	6.0671	8.4687	8.9504	8.3738	7.8045	4.8698	10.2544	8.117	9.1144	9.6426	3.2653	6.0516	8.6262	6.9981	8.1767	5.5893	8.6911	9.8562	10.1348	6.304	8.4229	8.078	8.3893	7.8396	10.2052	9.864	6.9791	10.5191	10.8318	9.2824	9.1979	10.4366	8.7508	9.7789	7.859	7.6787
TCGA-05-4384-01	426	0	3.7896	7.447	9.4672	11.0252	9.6625	5.8834	6.1915	7.9812	8.7214	7.8838	8.3131	6.8936	4.1783	9.7075	8.5549	7.7137	10.2624	5.9704	4.8323	7.8612	8.6954	8.0326	2.5575	8.4704	9.4471	9.6937	5.3899	8.0088	7.5727	8.6912	7.0745	9.1401	8.5942	7.953	10.4295	7.2925	9.6284	7.8838	9.5471	7.8986	9.8641	8.536	6.0786
TCGA-05-4389-01	1369	0	1.8815	6.5539	9.3969	9.5933	10.345	5.8082	2.593	3.9159	6.7498	8.9512	6.9403	7.6702	6.104	9.8234	6.8169	9.4048	8.8181	6.9754	5.7554	8.2011	8.817	8.4019	7.7216	9.8341	10.2838	10.2981	6.2447	9.0864	9.0153	8.0002	9.7095	10.3176	9.4436	7.0208	9.7466	10.9629	10.398	10.2552	9.7511	9.4366	10.0829	8.3716	10.2288
TCGA-05-4390-01	1126	0	2.7545	7.4276	8.8837	8.4813	10.254	6.0614	9.8977	5.4314	6.9348	8.6965	9.4559	6.6438	5.4099	9.0052	7.345	8.5737	8.0965	2.7545	4.138	7.0436	7.9099	8.41	8.024	9.171	10.2355	10.0968	5.6672	9.0684	8.7337	8.2191	7.5209	9.78	8.2067	15.0796	12.2315	9.3426	8.9578	10.7849	10.9959	9.0615	9.1371	8.3349	5.2972
TCGA-05-4395-01	0	1	0.343	6.194	10.0366	10.072	8.2251	5.2996	2.671	9.9481	6.8937	8.5741	7.2918	6.2911	4.5411	8.9805	6.3682	7.8721	6.9967	4.9572	4.7426	5.3855	8.7481	7.4127	6.4895	8.781	10.7203	12.2844	7.3523	8.9511	8.516	8.0824	7.924	9.1102	11.062	9.1578	12.0698	9.8181	9.7122	11.0646	9.0501	9.6398	8.4752	8.4979	5.8557
TCGA-05-4396-01	303	1	2.4722	3.5378	9.3676	9.4974	8.7307	8.9299	4.5227	10.06	8.4106	8.7021	8.5528	6.3991	3.8725	8.3397	7.4228	10.8445	7.2322	4.6588	6.8228	7.9194	8.2456	8.4781	5.0704	8.4626	10.2617	9.7883	4.3186	7.2249	8.0937	7.4973	6.7228	10.2555	7.8594	8.183	8.0613	9.5303	11.7267	8.801	9.7659	9.3493	10.4533	8.0735	10.6836
TCGA-05-4397-01	731	1	2.6556	7.4769	8.6072	11.5769	10.651	4.6482	1.778	6.6558	6.2114	9.6472	7.2275	5.8631	6.0088	9.8215	8.1154	8.1958	7.1601	7.375	7.4277	6.9207	7.8759	10.0406	8.6064	8.5377	11.1593	10.1106	7.0728	9.4873	8.5411	9.1145	8.6672	11.2416	7.8623	7.5139	9.7735	12.0996	10.229	11.955	9.025	7.2297	11.5609	8.0423	7.7275
TCGA-05-4398-01	1431	0	2.5855	6.5084	9.19	9.932	10.314	5.6545	7.171	5.2633	7.8761	8.973	8.9668	8.0225	7.661	9.4254	7.797	7.8387	9.7196	6.0211	5.205	5.8302	8.5206	8.2652	7.4742	9.2557	9.609	10.7972	7.4331	8.3685	8.7695	8.4834	7.9213	10.1171	9.6784	6.6274	12.0149	11.0727	9.2644	10.6109	9.7574	9.2891	9.027	8.6426	7.2097
TCGA-05-4402-01	244	1	3.4129	6.7338	9.7322	10.1001	9.8095	8.2921	4.5055	4.6232	8.2959	8.7029	7.827	7.3022	6.7141	9.8902	7.3626	9.1668	8.8084	6.6427	5.3423	8.6674	9.5238	7.9017	5.9608	8.9857	9.598	9.4684	5.8138	8.8825	8.4655	7.5783	7.8007	9.647	9.4041	9.7585	11.1125	10.2324	9.6665	9.6613	9.8493	9.6128	10.3823	8.0083	5.9774
TCGA-05-4403-01	578	0	1.5588	5.9187	10.3716	8.6019	10.075	6.4506	2.991	4.5895	9.1483	8.3462	6.446	7.6542	4.6861	10.2681	6.9167	6.6947	8.0429	9.05	4.6386	8.5169	8.6184	7.6799	4.7169	8.7265	9.698	9.9225	4.1888	8.6316	7.6362	7.2528	6.9134	9.7219	9.4198	9.3078	9.2641	7.1549	10.7099	9.4516	9.0733	10.0782	9.825	8.2481	11.8708
TCGA-05-4405-01	610	0	0.7334	8.6649	9.0145	9.2897	9.4294	7.533	3.3613	7.448	8.4799	8.7561	9.3392	7.4964	5.5045	9.7764	6.878	7.8555	9.0743	5.9063	6.0717	8.8167	8.052	7.9781	3.7645	8.7406	9.3079	9.7299	5.7374	8.3207	8.6023	7.8841	7.3576	9.2557	9.5905	12.6341	10.8583	6.7238	9.162	8.6363	8.585	9.0743	10.1423	7.7922	8.3503
TCGA-05-4410-01	0	0	2.6671	5.5028	9.6952	10.5678	9.8115	6.0934	2.9783	5.0155	7.6964	8.8801	8.7036	7.9715	9.0583	9.123	7.557	9.6791	9.2407	6.141	5.1143	7.1308	7.8477	8.4622	4.216	8.7783	9.363	9.8692	5.922	8.4993	9.2426	7.7538	7.0076	10.0836	8.5413	7.0746	9.652	10.2684	9.4076	8.5937	9.8739	9.9338	8.908	8.0318	7.2148
TCGA-05-4415-01	91	1	3.5443	5.9619	8.7846	10.4632	8.555	8.4267	8.5257	9.6765	6.5392	8.478	5.5749	6.1986	2.558	8.1969	5.9096	4.6439	6.2159	5.9925	5.7872	5.8552	7.5858	9.3061	7.9471	9.159	9.706	9.4459	8.8034	8.7627	10.0894	7.3741	7.7792	10.0894	8.6427	12.6931	13.1678	10.9033	9.7521	11.2095	9.6851	8.7744	8.2839	7.3702	4.618
TCGA-05-4417-01	455	0	2.6048	8.2957	9.3109	9.6851	9.8805	7.988	4.0776	7.9366	9.3799	8.8898	6.7343	8.2023	7.3148	9.3451	7.3376	9.455	9.6375	5.5663	5.193	8.3557	7.9403	8.3217	6.0949	8.2692	8.689	9.2788	4.8871	8.1091	8.3971	7.2379	7.0977	9.8941	9.0049	12.0295	9.8321	9.8026	9.821	9.3507	10.2303	9.7578	9.8639	7.8636	8.3585
TCGA-05-4418-01	274	1	0.7353	5.8372	10.3106	10.1724	9.2602	7.391	5.0403	5.0403	8.821	9.1815	9.5883	7.436	3.8035	8.8538	6.5808	8.6117	7.7215	3.9961	5.7508	5.0834	8.8117	7.9383	6.9423	9.6611	10.5085	10.3166	7.2595	9.2789	8.6019	7.7395	7.5063	9.7277	9.6	10.3698	11.4991	8.7956	10.4749	10.6855	10.1056	9.86	9.3981	8.7783	5.5808
TCGA-05-4420-01	912	0	1.7513	6.5315	9.3868	9.4922	9.9898	6.7481	6.6758	9.7252	7.3904	7.9628	8.6605	5.9906	8.0456	8.95	7.1167	8.7826	8.4585	4.7425	4.609	6.3481	9.5178	8.5042	6.9426	9.96	10.533	10.6462	6.6229	8.3499	9.7021	7.2016	8.0033	10.0241	6.7124	12.7422	10.0433	11.4957	12.9115	11.9311	9.8463	8.0311	10.7543	7.6039	6.5437
TCGA-05-4422-01	365	0	3.2969	4.2216	8.8011	9.9707	9.7406	7.8583	3.3409	8.6176	7.2628	7.8827	8.1657	7.9781	9.5209	9.4006	9.3551	8.8177	10.4725	3.8776	5.4367	6.8962	8.4619	8.5145	6.2969	9.3954	9.6034	9.7513	5.5996	8.7253	9.1829	9.14	7.2536	11.1049	8.8796	4.8431	9.3591	10.1418	10.6568	8.5606	10.8173	7.4991	9.1251	9.0476	9.8122
TCGA-05-4424-01	913	0	3.8897	7.2668	8.9673	8.5105	9.9541	7.2534	3.3968	6.5092	9.189	8.5067	7.9256	8.2733	8.3427	10.1196	7.3323	8.7353	9.1671	6.1119	5.1227	9.2952	7.7673	9.192	5.9094	8.5029	9.543	9.7303	4.7914	8.878	8.2212	7.5313	8.696	11.3193	9.5857	9.1308	10.6127	9.8372	8.9563	10.0258	9.096	7.8078	9.5758	7.5716	7.7225
TCGA-05-4425-01	669	0	2.7216	5.8738	8.7012	9.3934	9.6829	4.9899	0.6412	6.3988	7.7421	8.5693	8.866	7.7653	5.3872	9.7953	7.0741	5.7138	10.107	3.9059	5.2454	9.0263	8.5883	8.9019	7.0315	8.3738	9.8444	9.6461	6.7903	8.995	9.2107	7.7429	7.9032	9.8842	9.5154	9.9088	9.0463	9.294	9.7197	9.2979	10.1531	10.0938	10.2304	7.0315	9.017
TCGA-05-4426-01	791	0	3.6163	5.9761	9.4252	10.7182	9.7382	6.0809	3.7173	4.5743	7.2652	8.7607	8.3283	6.8727	5.0449	9.6669	6.7628	6.6481	9.3287	5.7485	6.202	8.387	8.5521	8.8835	5.8259	8.9883	10.068	9.7561	6.4715	8.1212	8.9499	8.1011	7.2992	9.9	9.093	9.1347	11.7581	4.8511	9.4795	10.0826	9.6478	10.3566	9.9919	8.1471	5.5619
TCGA-05-4427-01	791	0	2.3324	6.5443	8.9917	10.3957	9.5415	5.1212	2.3324	5.0126	8.7708	7.8236	8.598	7.5287	4.3343	9.2406	6.4475	9.8155	8.7578	3.099	5.4885	6.2513	6.9555	8.8171	4.4719	8.4287	9.8718	9.7065	4.8455	7.5599	9.4622	7.5444	7.7544	9.1854	8.8171	8.2102	11.364	9.4032	10.2478	10.2322	9.9267	9.9803	9.2213	7.0898	5.0787
TCGA-05-4430-01	761	0	3.5269	8.3947	9.2158	8.7494	9.4976	7.8536	4.4836	7.1689	8.9103	8.6394	7.3292	7.7676	4.8615	9.9455	6.1073	8.1298	9.2042	5.9599	4.7801	7.3434	9.1678	8.0505	5.9072	10.2177	10.0058	10.0624	4.504	9.0063	8.5191	8.2691	7.6647	10.4924	9.4944	12.7992	9.7888	8.4218	9.8202	9.3036	9.9654	9.0855	9.9167	8.026	7.4099
TCGA-05-4432-01	761	0	5.0068	10.4829	8.5721	9.1932	9.8139	6.6134	3.9197	7.0343	8.4849	8.3541	6.7199	7.3394	6.6303	9.5092	7.8237	8.8712	8.895	5.7335	5.3404	8.8906	7.545	9.3449	6.3225	8.7185	10.1672	9.9821	6.3736	8.4393	9.133	8.7936	7.5889	10.521	8.6549	11.0822	10.1343	9.5268	9.7374	10.3019	10.3045	8.0881	9.7286	8.6383	7.8813
TCGA-05-4433-01	730	0	2.3596	3.0769	10.1003	8.5168	10.262	4.8629	3.0769	5.0545	8.6378	7.7318	5.9769	8.0922	4.8629	10.1111	6.8667	7.2423	9.9225	8.3478	4.9033	8.9079	7.7541	8.4157	4.8213	8.4604	9.0109	9.5988	4.062	8.1353	8.2842	7.1105	6.6102	10.1441	8.9734	9.122	8.8979	6.7717	9.8597	10.1325	9.1601	9.523	9.4241	8.2016	10.3167
TCGA-05-4434-01	457	1	3.0016	6.781	9.1173	9.425	10.095	6.2872	4.0133	5.6968	8.4592	8.5798	8.3897	7.904	6.9302	9.7243	8.3179	5.9657	9.5438	6.9802	5.7524	7.5068	9.0768	8.6127	6.283	9.1788	10.1239	9.3655	6.5911	8.8044	8.9427	8.3552	7.7966	10.1952	10.048	8.1304	12.1677	10.8348	9.9515	10.6646	9.7104	7.6222	9.0834	7.5561	8.1931
TCGA-05-5420-01	457	0	2.1954	6.3337	9.7929	8.2155	10.707	8.477	7.3248	4.3071	7.1866	9.3119	5.9299	9.0643	6.2343	8.9225	6.2849	9.2347	8.5546	4.8453	4.1704	5.3356	9.2088	8.0597	7.7596	9.9771	10.2674	10.5518	7.7832	9.6508	8.3679	8.6315	8.1625	10.595	8.1625	10.4947	9.2707	10.2537	11.739	10.5841	9.8486	7.6609	8.7412	8.7169	6.3964
TCGA-05-5423-01	151	0	3.75	6.6944	9.7634	9.448	10.105	7.8008	2.2213	4.1578	7.6314	8.9634	7.6771	8.1223	5.7869	9.7671	6.9971	5.2338	8.5689	4.2159	3.9683	7.772	9.8311	8.0761	5.7481	9.5614	10.1355	10.6793	6.2975	10.3628	8.5745	9.3639	8.1109	10.3787	8.9006	9.7426	9.2965	10.7693	10.7187	8.7832	9.4912	8.0761	9.8553	8.5408	6.9031
TCGA-05-5425-01	882	0	4.2976	6.6679	9.6493	9.9276	9.7494	7.4852	5.2604	7.0713	8.0215	8.8727	8.7166	8.0005	6.4045	9.6534	6.3555	10.2652	8.7492	3.9732	4.5963	7.4988	8.4276	8.4347	7.4529	9.7541	10.1297	10.758	4.9792	8.7811	9.0402	8.7511	8.7339	10.0687	7.8913	11.0918	9.8528	10.6676	10.0906	10.9264	10.3458	8.9374	9.513	8.2134	8.2674
TCGA-05-5428-01	670	0	2.8287	6.902	9.5425	6.3745	9.2986	8.6358	2.1661	5.0176	7.4281	9.2906	9.61	6.8375	4.3352	8.7512	4.6173	5.1998	6.8264	3.281	3.4049	3.8156	9.2058	7.1436	7.6299	10.5142	11.2029	11.3889	8.4504	9.5492	9.3802	8.7972	8.4576	10.1018	8.6006	8.9087	10.6315	9.6853	10.4256	10.8517	9.7111	9.1735	7.8907	8.7158	4.511
TCGA-05-5429-01	275	1	2.4958	5.4696	9.4955	7.9881	9.6423	7.3994	1.7313	4.8501	9.2213	8.8346	6.3475	6.0266	4.8109	8.4725	6.5516	5.1928	10.1134	8.4472	4.0416	8.7899	9.1297	8.2303	6.4275	9.5897	10.7963	10.1889	6.0935	8.7974	8.2266	7.8595	8.0012	10.5528	7.4449	12.0451	7.058	8.0314	11.0806	10.8474	8.7175	9.1534	9.0934	8.1382	10.1063
TCGA-05-5715-01	62	0	2.8572	6.9764	10.0249	9.1414	9.6106	6.6349	3.1388	5.8772	7.808	8.8261	9.3917	6.7649	4.4648	10.2726	5.3512	5.8963	10.1863	8.5746	4.7423	8.2177	8.3164	8.505	6.0906	8.9175	8.624	10.161	3.8353	8.8916	8.4705	7.7478	7.3864	9.7255	8.9968	10.1068	9.663	7.153	8.7418	9.5563	9.1885	9.9909	10.8268	8.4768	9.6363
TCGA-35-3615-01	14	0	1.8476	5.4233	9.8528	10.3279	9.0872	7.573	3.1894	10.911	7.7358	8.7586	7.7897	6.3442	4.0526	8.8406	7.4443	6.1859	7.4893	5.4123	4.6274	7.0076	9.653	7.4335	5.5081	9.9578	10.6233	10.0767	6.6638	8.9317	7.8879	8.9007	7.6636	9.8744	7.9327	8.3	10.3474	5.7965	10.936	7.868	10.7337	8.7854	9.39	7.7113	6.0859
TCGA-35-4122-01	225	0	2.591	6.5149	9.5666	7.2926	10.404	8.7402	1.5898	4.0943	8.3227	8.957	7.6366	8.0867	7.7554	9.9246	4.0512	7.0406	8.5386	6.3995	3.1764	4.3992	9.0475	6.8527	8.0894	10.1255	11.0362	10.6686	8.1468	9.5209	9.248	7.6108	8.4145	10.0058	9.5894	9.5386	10.2995	10.5254	11.1164	11.5809	10.5738	9.1416	9.4187	8.3336	4.6794
TCGA-35-4123-01	182	0	2.0264	6.7031	8.9294	9.897	10.358	7.2401	0.9094	3.8251	8.7071	9.2128	8.0612	7.5449	6.933	9.5254	5.4748	8.1397	9.1122	8.2849	4.5749	5.1751	7.6144	7.0476	8.0301	9.7325	10.6847	10.8032	8.3289	9.043	9.3694	8.1171	8.2269	9.9337	8.8465	8.9281	12.6412	8.5512	10.1092	11.1717	10.3176	9.6426	8.8992	7.9656	4.7499
TCGA-35-5375-01	264	0	1.0772	8.7721	7.7421	7.2368	10.594	7.9953	2.1142	4.3908	7.7496	10.1837	6.3773	6.1023	4.5359	9.1641	5.356	7.5925	6.7637	3.458	4.5359	9.5596	9.7631	8.9149	9.6421	10.7493	11.4415	11.3743	7.9827	9.5553	7.9635	8.5031	9.4766	11.9036	7.5758	7.5758	10.5001	8.9116	9.93	10.8394	8.9574	7.0318	8.3545	8.2426	7.8979
TCGA-38-4625-01	2973	0	1.5711	6.5835	8.1627	8.8125	9.8217	6.6416	2.5329	5.4896	8.2648	8.5986	4.4301	6.4128	4.4301	8.9437	7.2501	6.5665	8.1048	3.8874	3.8026	8.6662	9.1394	9.0663	8.321	9.7381	10.2906	10.3169	8.6163	10.0304	8.9593	8.7134	8.7861	10.8346	7.9326	12.818	11.77	10.6797	9.7338	11.1994	9.1799	6.2462	9.1022	8.4966	4.6883
TCGA-38-4626-01	3674	0	2.4056	7.9663	9.2109	9.1786	10.445	7.3763	5.03	7.0809	8.9638	8.3501	7.9399	7.3881	4.5621	10.0115	7.6379	7.3182	9.4373	6.8401	5.8413	7.9465	8.9515	7.8871	4.9792	8.4956	9.1258	9.2273	6.4235	8.084	7.6181	7.3203	6.9418	9.2468	8.682	12.0713	10.3516	9.2164	9.6333	8.666	9.2366	8.5357	10.2488	7.9883	6.761
TCGA-38-4627-01	1147	1	4.3198	8.6097	9.4475	8.6832	9.9407	9.2755	5.1112	8.5562	10.932	8.8658	7.199	7.8644	5.4611	10.4082	5.762	8.9048	8.247	8.426	5.049	8.2963	8.1317	7.9478	5.8467	8.4757	9.6018	9.8444	5.2282	8.5893	7.8779	7.8979	7.8068	10.3836	10.138	10.4648	10.2282	8.6258	9.7728	9.3305	9.8966	9.6471	10.9832	8.109	7.3742
TCGA-38-4628-01	1492	1	1.5342	8.2166	10.2255	8.8432	8.7211	7.8951	7.0288	7.9216	7.2095	9.6748	8.8571	5.9468	5.9976	9.9799	5.3174	4.88	8.4046	3.2044	3.8353	8.6637	9.0144	8.6192	5.7575	9.275	10.4301	10.8604	6.6519	10.0218	8.7741	8.5982	8.771	11.5895	7.8163	8.2469	8.2439	11.2555	9.715	10.1672	10.3195	8.5576	10.4982	9.038	4.5702
TCGA-38-4629-01	864	1	1.6397	7.3329	8.9669	9.0849	10.243	7.3463	3.0453	4.818	9.012	8.92	5.5715	7.8893	5.2775	10.5365	5.1482	7.0531	8.379	5.9557	4.1883	5.6775	10.1134	8.745	7.4818	8.5941	9.7031	10.503	7.1758	8.8067	9.1229	7.6606	7.8369	11.0546	10.018	9.8685	11.0514	9.5995	9.4873	10.1648	9.9918	7.9381	9.7052	8.3698	5.0197
TCGA-38-4630-01	1073	1	2.7573	5.2054	8.5268	9.2094	10.251	4.6526	2.2531	6.9436	7.689	8.5346	9.6286	5.2648	6.2543	9.4045	10.8985	8.4783	7.41	8.7518	5.7889	9.07	8.4961	9.0387	7.6276	9.5541	11.2628	9.2249	6.4462	7.8954	9.5898	8.4512	8.3183	10.1086	10.326	6.4716	11.0804	12.0358	7.9272	11.6221	7.4043	8.5995	9.3009	7.7411	5.7538
TCGA-38-4631-01	354	1	1.3615	6.1305	9.4354	8.4935	10.107	6.833	4.3659	4.5219	7.5649	9.6507	8.6082	6.3683	4.7909	8.8184	6.596	7.0364	5.1509	7.0535	4.8312	6.0473	7.909	8.175	7.6119	9.7019	11.2762	11.8128	7.6349	9.7328	9.8564	8.5029	8.4841	11.1901	6.2543	11.2688	10.3844	11.5874	10.4795	12.1284	8.558	8.5154	9.0432	10.0863	4.6626
TCGA-38-4632-01	1357	1	3.5491	5.9404	9.6204	9.5544	10.29	6.2467	1.3387	4.1557	8.2431	9.2134	8.828	7.659	4.2744	9.5806	5.7069	8.6053	8.297	2.9786	4.3303	4.7951	9.8789	8.0194	7.9135	9.0953	10.4189	10.9418	6.8254	9.655	9.2573	8.6053	8.0654	9.8801	8.6389	8.5297	9.1852	10.1668	9.6302	11.0362	9.3599	9.5632	8.7533	8.3588	5.4023
TCGA-38-6178-01	448	0	3.5754	7.7464	9.9411	8.9437	9.8744	6.7214	7.1829	7.3578	8.2753	8.2972	8.8197	7.3089	5.235	9.7764	6.7426	9.1037	7.996	7.3231	5.579	9.1774	7.3371	8.2011	4.9374	9.1598	9.358	9.4397	5.6023	8.3874	9.3092	7.8427	7.5137	9.872	8.8735	12.7183	10.1257	11.0119	8.9161	9.7188	8.1063	9.8379	10.2089	7.574	5.4816

TCGA-38-7271-01	800	1	3.9474	8.0007	8.7251	8.3215	9.6946	7.4249	2.262	7.3992	9.2678	8.626	8.2149	9.0297	4.9361	9.2272	6.6722	7.7337	9.2381	7.1826	5.312	7.7941	8.4079	8.2869	5.0396	8.1738	10.0852	9.8676	5.2268	8.1275	8.1078	7.6813	7.6759	9.8223	9.288	13.1893	9.3431	7.6972	9.3261	8.9231	9.5501	8.6643	10.2378	7.3597	7.0648
TCGA-38-A44F-01	133	0	2.9726	6.1539	9.3379	8.0451	9.4026	6.1882	2.4767	3.6989	9.5162	8.4096	8.6561	6.6319	4.5748	9.7654	9.1607	6.7047	9.923	8.8254	5.7722	7.3998	8.0881	7.5993	3.9326	8.6458	10.1067	9.7094	5.5442	8.3015	7.3138	9.0028	6.6484	9.6128	9.4219	10.6082	9.1184	7.4936	9.5059	8.251	9.2527	7.4519	9.9876	8.7849	9.5406
TCGA-44-2655-01	1324	0	2.3628	8.1315	10.1452	10.5336	9.4094	7.0017	4.5405	4.8564	8.7503	8.9001	8.1447	6.6353	3.8546	8.8597	7.4037	8.3104	9.4564	9.0323	4.6782	7.1112	10.0434	7.512	4.8172	9.0869	10.0983	10.0473	6.0273	9.106	7.858	9.0042	7.6204	10.1176	7.9164	9.7303	9.4538	9.6719	10.4948	7.8138	10.054	9.1922	9.7994	8.2915	6.1897
TCGA-44-2656-01	1429	0	1.993	6.7589	9.4531	10.6757	10.076	7.043	3.2737	3.5516	8.7386	8.3032	6.5825	8.7288	5.9908	9.6019	7.5143	7.6352	9.6987	6.1479	4.8535	6.8086	8.5825	8.4657	5.4171	9.2727	9.5228	9.8933	7.0161	8.5629	9.094	7.6313	7.7667	10.3814	9.4436	7.7951	10.9561	10.0094	8.8965	10.0029	9.5344	9.214	9.8337	7.6034	6.6924
TCGA-44-2657-01	1351	0	5.6629	6.2125	9.4684	9.2328	9.7415	7.8779	4.3068	6.6486	9.3409	8.9764	8.6785	8.9115	5.1659	9.8681	7.717	6.5995	9.8024	6.8963	5.3902	7.6021	8.6182	8.5757	4.7994	8.9974	9.2819	10.213	5.5225	8.8415	7.8899	8.2935	7.5094	10.0101	9.2166	8.8557	8.9609	9.6481	8.9115	10.0385	9.4547	8.7158	10.4543	8.2935	7.6984
TCGA-44-2659-01	1367	0	3.145	7.415	9.5737	10.0883	10.941	7.9832	8.0182	5.8221	8.4257	8.3178	6.4641	7.8383	5.0025	9.4238	7.132	7.5086	8.7911	8.394	6.501	8.4745	7.294	7.738	4.4106	9.2712	9.3484	9.5793	4.7278	8.6596	8.8505	7.4878	7.6655	9.0892	9.1927	7.4039	9.4975	9.8392	8.7346	7.7247	9.3317	8.8678	10.4978	7.5034	6.6256
TCGA-44-2661-01	1159	0	3.647	6.4103	9.7003	8.6564	9.5751	9.3084	1.9567	5.4669	9.1701	9.0015	8.3622	8.8202	6.0926	9.9617	7.5311	5.6436	10.298	5.4352	6.1907	6.6705	9.8647	7.8936	6.1228	9.1841	9.7575	9.8247	6.1907	9.2864	7.7593	8.4057	10.6982	10.2135	10.009	9.1983	9.5851	7.0086	10.0874	9.2853	9.9108	8.8339	9.7194	8.5679	7.1567
TCGA-44-2662-01	1280	0	1.9534	5.67	9.9012	10.309	9.2314	6.1648	1.2848	5.7588	8.4314	8.3613	7.0412	8.2415	6.5928	10.1965	6.4578	8.3597	9.9044	3.2658	4.7663	6.2214	7.7411	8.96	6.1356	8.5693	9.9423	10.5821	7.6093	9.1733	8.96	7.5498	7.6173	9.8711	10.246	9.2123	10.4609	10.1255	9.3028	11.0674	8.834	9.8633	9.1598	8.0377	5.7002
TCGA-44-2665-01	1301	0	2.8414	8.0716	9.3241	9.3421	9.5269	7.8433	3.3238	6.7577	8.369	8.9369	7.2733	7.676	5.3689	9.669	7.5863	8.3735	8.146	6.256	6.4872	7.1773	8.7007	8.1117	5.8679	8.7349	9.6808	9.6168	5.5551	9.433	8.3714	8.6299	7.2822	9.8704	9.5518	12.1981	10.5426	8.3694	9.3294	8.5664	8.9782	9.171	9.7969	7.7731	6.0851
TCGA-44-2666-01	97	1	4.1779	5.754	8.7147	8.4618	10.367	5.5249	2.8592	5.6971	8.5739	8.0813	8.2802	6.7903	4.1098	8.7887	7.5788	9.4382	8.0407	9.5644	5.7087	8.3574	8.6652	8.9274	6.1763	9.3843	10.3647	9.4022	5.7201	8.2898	9.1321	7.9633	7.7728	10.4372	8.7276	12.7557	10.6744	6.3337	9.2875	9.5027	9.5101	9.4417	8.9434	7.6404	6.8541
TCGA-44-2668-01	761	1	2.5639	7.1314	8.0011	7.7371	9.769	5.5247	2.4957	5.9058	7.9075	9.2635	6.9711	7.9238	4.5806	9.137	6.6172	7.4769	8.3977	6.3049	5.3426	6.3198	6.8938	8.2004	6.8737	8.4861	10.0658	9.8968	6.4243	8.1856	8.911	7.1564	7.8877	10.116	9.8113	11.1851	10.6759	9.1453	8.8821	10.563	9.495	11.2253	9.3481	7.0973	6.2177
TCGA-44-3396-01	1130	0	2.2642	7.2922	8.6045	8.2318	9.758	7.68	3.282	6.0759	8.6081	9.1338	8.3787	8.9351	4.6789	9.74	7.5239	8.5844	9.0382	4.8786	5.5087	6.6488	8.969	8.3173	6.872	8.8495	10.114	10.4195	6.3576	8.3738	8.5173	8.1409	7.984	10.1803	9.0437	10.5325	10.2857	7.5274	9.239	10.6856	9.1054	10.2559	9.989	7.9635	5.5016
TCGA-44-3398-01	1163	0	2.8922	8.0925	9.4207	8.7385	9.804	8.8829	5.2897	5.723	8.9571	9.5099	8.0947	8.0014	5.7347	9.6153	7.2496	8.6444	8.7952	8.0371	5.6263	6.6974	9.8185	8.0811	6.75	9.6223	11.3707	10.1414	7.5124	9.0989	8.2591	7.7462	7.7859	10.1835	9.4988	13.733	10.8512	7.4537	10.9085	10.0471	10.6787	9.8301	9.936	9.3524	8.5355
TCGA-44-3918-01	1036	0	2.6643	7.4155	8.6559	7.6984	11.249	7.7195	2.9754	5.3045	9.2969	8.8966	8.3332	8.3246	5.2332	9.5339	7.1156	8.9465	8.8675	7.9214	4.7717	6.3482	8.3195	7.5941	7.4155	9.7696	10.7003	10.8095	6.9159	8.9499	9.0247	7.8056	7.7351	9.5849	9.277	9.2734	11.3406	6.7367	9.9495	9.8991	10.2723	7.9639	9.6738	7.9372	6.2213
TCGA-44-3919-01	1026	1	3.1383	7.1902	9.1908	9.7104	9.6602	6.6372	2.9077	4.9197	8.6412	8.8843	8.5792	8.1788	7.4538	9.5062	6.4405	6.1547	8.8843	4.5307	5.6185	7.8349	9.0686	9.2818	6.8716	8.8328	9.6333	9.8327	6.6698	8.6486	8.6799	7.4048	9.8286	10.8755	9.6567	9.5431	10.0616	6.1613	9.9345	10.1669	9.7572	7.7664	9.9531	8.4253	6.4405
TCGA-44-4112-01	808	1	3.1751	8.132	9.012	8.7439	8.9228	7.6482	3.1094	6.2498	8.79	9.2494	7.9888	6.9341	4.9966	8.6124	6.9341	8.8854	7.4635	6.2268	5.2306	5.8079	8.4143	8.134	6.6525	9.675	10.6159	9.9885	5.8487	8.6038	9.2926	7.6072	7.5088	10.3427	9.0375	12.8784	10.7771	7.074	9.9297	10.0521	9.5538	9.6449	9.8863	8.6862	4.8636
TCGA-44-5643-01	1013	0	2.8474	3.324	7.6988	8.5832	9.8488	4.3728	0	3.915	6.9256	7.6671	5.2549	6.3298	4.6568	8.5467	8.1373	9.0314	7.826	6.7173	5.276	5.5947	9.0772	9.0907	7.6555	9.328	11.2144	10.2694	5.1211	8.6393	8.9759	6.6782	7.8614	10.6589	9.1202	8.7661	7.683	7.5462	9.3292	11.0227	8.18	10.0183	9.6149	8.9662	6.2678
TCGA-44-5644-01	863	0	5.257	6.2632	9.0652	9.5593	9.8476	4.8873	7.8482	7.877	9.2559	8.9123	7.6475	5.4883	7.5484	8.4157	9.09	9.8917	9.9808	2.1127	6.0768	6.5864	6.2124	7.4366	6.9622	8.8027	10.2111	10.541	6.8585	8.0856	8.5926	7.8482	7.1563	9.1058	6.4613	10.1742	9.2372	10.0321	10.4718	9.6031	9.5452	7.8852	9.4359	8.0169	7.3744
TCGA-44-5645-01	852	0	2.3266	3.8274	9.4789	9.7604	9.0898	5.6204	9.2736	5.0248	9.5209	8.3975	8.1015	8.6918	4.2749	9.8393	8.146	6.3353	10.3869	5.8094	5.1466	8.6938	9.0715	9.2534	4.616	8.1892	8.8071	9.705	5.4604	8.9071	8.0017	8.5062	7.1742	10.4355	9.511	9.4149	8.4218	7.9221	9.1581	7.4873	10.0166	9.2829	9.679	8.5265	9.1967
TCGA-44-6145-01	595	0	1.8184	8.0118	9.3011	10.1476	8.7027	6.7088	1.5336	8.0672	8.7621	9.0266	7.7106	7.8903	3.7692	9.3112	6.7517	6.0732	10.0696	7.3795	5.3944	7.1802	8.249	8.8502	6.2026	8.0398	9.6823	10.4344	4.3184	8.5087	8.2002	7.6254	7.8154	10.133	9.7271	10.1035	10.4969	9.0663	11.9418	10.1167	9.5608	8.798	10.4005	8.2609	7.3849
TCGA-44-6146-01	728	0	0.5373	2.4627	10.5928	8.8453	8.9622	3.8617	3.9059	4.6127	9.0643	7.0625	7.8328	6.2203	3.8617	8.8523	7.4749	6.8306	6.3694	8.8034	4.9012	9.1966	8.3119	8.5922	3.1163	8.3622	9.3601	9.5945	3.0392	8.3622	7.6982	6.7114	6.7114	9.78	7.1839	7.897	6.6672	5.8657	10.6559	8.3682	8.0424	9.6303	9.4334	8.2491	10.759
TCGA-44-6147-01	845	0	3.8191	7.8958	8.9465	8.8161	9.6652	7.2617	6.0087	7.5951	8.9039	8.2152	8.7315	8.1333	4.4073	9.3337	7.72	8.6415	8.7608	9.0616	5.6647	9.6128	8.5624	8.3493	5.7404	8.8507	9.3676	9.3877	4.582	8.5893	8.2724	9.1433	8.6728	9.6633	8.505	10.0996	9.3877	8.8231	9.1475	7.7605	8.9417	7.9155	9.9562	8.4699	9.1558
TCGA-44-6148-01	704	0	2.2479	2.5413	9.2655	8.6169	8.8823	5.6824	6.2377	7.4117	10.5711	7.9939	8.2258	8.006	5.2052	9.13	8.5359	8.5971	9.6258	8.9002	5.7558	9.0936	8.784	8.6208	2.785	8.5276	9.0808	9.7095	5.6366	7.8967	7.3889	8.9865	6.8734	10.0108	10.005	9.9858	8.6935	8.0764	9.1272	5.7269	8.7158	8.3135	9.9099	8.4872	9.0403
TCGA-44-6774-01	658	0	5.6512	9.6241	9.0238	10.1412	9.3682	7.5022	5.4512	9.1257	10.203	7.9875	8.4299	7.759	5.0673	10.1412	6.6368	9.0186	10.0493	7.9875	5.0267	8.8104	8.35	8.3744	4.852	8.7772	8.8104	9.7817	6.4501	8.1331	8.6428	7.0448	6.9286	9.9223	10.088	12.0435	10.14	8.5203	8.8747	10.1051	9.4062	8.3458	10.5026	7.8196	5.5404
TCGA-44-6775-01	705	0	2.7121	7.991	9.3049	9.1495	9.7527	6.7347	4.2225	6.2897	8.7564	8.3424	7.9795	8.5626	5.4729	9.8834	7.0249	5.7949	9.7072	5.0133	6.1221	6.8879	8.8371	9.6071	6.0022	7.9326	8.9311	9.4284	5.8209	8.2875	8.904	7.7141	7.7107	10.8219	9.8283	10.0939	10.2534	11.0809	10.0024	10.2287	9.4305	10.1282	10.2876	7.469	6.643
TCGA-44-6776-01	2616	0	1.9537	6.1579	10.1166	10.4539	8.952	5.2341	4.2985	8.4279	7.6259	8.4984	8.7598	5.5539	2.1991	8.4006	7.2602	8.8922	6.9524	9.7629	5.3403	7.7146	9.2378	8.3851	4.0708	8.5765	9.0215	10.158	4.2449	8.2212	7.3582	8.635	6.7066	8.7882	7.0881	9.0839	8.538	5.9887	9.7913	7.9591	9.922	9.3098	10.5301	7.7439	7.7726
TCGA-44-6777-01	987	1	3.2352	8.4372	9.4525	10.1686	9.7052	7.909	4.0228	7.2675	9.6708	8.0531	8.4437	9.6113	6.0263	10.8745	6.4122	8.0184	9.7465	5.6109	4.9036	7.7906	8.4696	8.476	4.0228	7.8345	9.0461	9.8602	6.3484	8.0616	8.3468	6.4474	7.202	10.1856	11.37	8.9404	11.2387	7.4737	9.0801	9.3527	10.1052	8.8917	10.0497	7.1812	5.2611
TCGA-44-6778-01	1864	0	4.5755	4.2301	8.976	8.6759	9.5119	5.9223	5.676	6.5194	9.2776	8.4975	7.242	9.2783	5.3113	9.9782	7.8928	9.4066	9.9809	10.1564	5.8918	8.0252	7.4395	9.0743	7.6664	8.2066	8.7649	9.5106	6.918	8.2189	9.4465	7.7038	7.7169	9.4582	8.8507	10.4448	9.3627	7.5115	9.7019	10.0228	9.6482	8.6951	9.5005	7.0482	8.5594
TCGA-44-6779-01	500	1	2.3176	6.7628	9.1326	11.1419	8.8827	4.9674	1.7617	7.8978	8.3552	8.0047	4.7708	7.9217	3.6038	9.9467	7.1665	6.2002	8.3138	6.5705	4.441	9.5963	8.5952	8.0703	6.4573	7.3886	9.22	8.8792	4.684	7.9638	8.0444	7.3748	6.9968	9.0676	10.935	7.4816	10.7734	4.0823	11.5785	11.5008	9.7282	6.7521	10.3965	7.7614	6.4175
TCGA-44-7659-01	691	0	2.6396	4.4046	8.9161	9.6083	9.5534	4.7633	1.697	4.4546	7.1371	8.1699	8.6388	6.812	3.6958	8.4636	8.7975	5.8899	8.2141	6.8868	5.4463	7.5244	8.3624	8.3225	5.8152	8.6576	9.7565	9.8187	6.2711	8.561	7.8389	8.6198	7.1749	9.3749	8.1165	9.3354	10.285	5.317	9.6289	7.4888	8.9708	9.6559	9.0604	7.7861	7.3503
TCGA-44-7660-01	592	0	6.6535	6.9858	7.5493	8.3529	10.128	4.4335	0	2.7841	9.192	8.1521	7.6464	6.9222	6.8131	8.9195	5.7434	9.417	8.8763	9.2773	6.7969	7.6002	7.8256	9.5868	8.011	9.5181	10.9351	11.3755	6.4184	8.2188	8.4925	8.1306	8.7225	11.2272	8.5142	7.6693	9.7056	7.5939	7.9485	9.9509	8.2106	6.7184	8.6773	7.8935	7.8202
TCGA-44-7661-01	557	1	2.2472	7.3714	9.0346	9.2258	9.2593	6.6484	4.5537	6.6618	9.2188	8.7848	8.1319	8.1855	4.184	9.5885	6.918	7.843	9.4033	7.7454	5.599	8.0356	8.3451	8.7868	6.5794	8.8221	9.6726	9.6902	8.0044	7.7829	9.2761	7.2134	7.8509	10.0592	9.1245	9.7284	11.5569	6.7854	9.1659	11.0126	9.7505	9.6335	9.4369	7.3216	7.1193
TCGA-44-7662-01	218	0	3.347	7.4732	9.0382	9.9037	9.4212	5.801	3.6143	6.2343	8.8646	8.4202	5.6896	6.9102	4.4986	9.5222	8.1644	7.8478	9.728	2.4435	5.1549	7.5321	6.993	8.0156	7.0195	8.7576	9.892	9.9771	6.0208	8.7357	8.8632	8.1836	8.1792	9.6679	9.5577	10.7718	10.6086	10.2109	8.5072	10.4494	8.9417	7.906	9.7356	7.2474	5.5598
TCGA-44-7667-01	1097	0	5.5114	6.6081	7.933	10.2596	10.568	4.5219	6.8972	10.357	7.7988	8.8635	3.2209	4.8891	8.6167	8.9775	7.7554	9.69	7.3112	8.5114	5.5426	5.6895	8.002	9.1823	6.8801	9.9246	10.8539	10.2576	7.1138	8.2142	9.5021	7.5087	8.6906	9.6457	7.6373	10.8067	12.1539	9.1016	9.5795	12.4257	8.7057	6.6081	9.0207	6.8057	4.6032
TCGA-44-7669-01	574	1	4.0298	6.6579	8.6864	8.8362	9.432	5.3744	4.0298	8.6971	8.6252	8.5395	5.3851	7.9553	5.7807	8.7266	8.8545	9.1452	8.992	8.1656	5.7482	7.0304	0	10.384	7.0836	8.4089	10.1251	10.036	7.4629	9.5123	10.1545	8.1687	8.187	10.2271	7.7751	9.7508	9.8205	5.9115	9.7118	12.0112	8.3106	9.5406	9.8416	7.5481	8.6372
TCGA-44-7670-01	882	0	4.1947	5.3642	9.0319	8.0124	10.02	4.8948	3.0198	6.4161	7.7043	8.7984	7.7477	5.2748	5.5847	9.5674	8.16	8.7412	7.3997	7.0983	5.9447	8.0516	9.3427	8.7329	7.7926	10.7174	10.6651	10.0395	5.6123	8.4715	8.8726	7.6198	8.6653	9.7736	8.6269	12.18	9.7898	11.335	8.8943	11.5896	8.5486	8.7234	9.6097	7.7987	7.8087
TCGA-44-7671-01	889	0	2.6682	7.4941	9.6926	10.9584	8.5139	7.8459	6.7053	7.0069	8.4961	7.8945	7.834	6.0215	2.9877	8.9199	7.0623	8.1575	7.5067	8.5748	5.1305	8.5152	8.4439	7.8983	5.5254	9.4988	9.5753	9.8081	5.9933	8.4015	8.0603	8.5065	7.0555	9.3611	8.0823	10.1887	10.4227	10.0511	9.7737	8.4697	9.5188	9.4792	10.1481	7.7915	6.5046
TCGA-44-7672-01	719	0	5.5206	7.1533	9.0469	10.4383	9.8136	7.0508	5.0955	5.768	8.508	8.3516	8.1222	8.9915	5.5206	10.0803	6.7016	9.9016	9.1984	5.6938	5.2065	7.1807	8.4124	8.542	7.7109	8.6342	9.0827	10.0165	5.0622	8.3533	8.1202	8.0833	6.9945	10.0295	9.2931	7.7109	10.8011	9.8211	9.1946	10.1962	9.2741	8.8534	10.102	7.7945	7.5904
TCGA-44-8117-01	385	0	4.8455	7.8835	9.3576	10.6112	9.2133	5.92	3.6175	6.107	8.5415	7.8083	9.6317	6.3411	3.3163	9.1792	8.7471	5.7675	7.7553	3.0403	4.7086	6.8036	7.0657	7.9651	6.5334	8.6683	9.5066	10.3804	7.5855	8.3686	9.0332	8.4255	8.6033	9.8652	7.9517	9.5112	12.3312	9.2999	8.7354	9.0576	9.4724	8.7413	9.65	7.6533	5.5083
TCGA-44-8119-01	285	0	6.2221	8.2654	8.2752	8.9891	8.2542	6.4519	5.3476	8.2752	8.5091	7.6895	4.6998	7.495	8.568	8.764	9.0259	10.9496	9.3747	8.6113	4.8521	6.7129	7.8379	9.5434	6.8409	8.9202	9.4842	9.6944	7.7132	7.7294	8.7917	7.1576	7.1576	9.5711	10.347	12.7264	9.3499	7.3629	9.7283	10.4932	9.4858	6.1544	10.0441	7.9557	6.7661
TCGA-44-8120-01	260	0	2.3799	8.7146	9.6733	10.5505	9.6862	6.6713	3.4715	8.7685	7.7378	8.8364	8.0939	6.7105	4.634	10.2951	7.2057	9.0468	9.7867	7.9879	5.4091	7.1413	8.3619	8.0549	4.0124	8.5932	9.9216	9.9216	6.1741	8.357	8.3875	8.0396	7.5263	10.1823	7.9426	13.0868	9.5787	11.4908	8.7328	7.9606	9.1206	9.5056	9.6817	7.1556	5.6696
TCGA-44-A479-01	486	0	4.8485	8.6681	8.6762	8.6376	9.2159	7.5171	5.3679	6.8746	8.4504	8.259	7.6206	8.9222	7.7892	9.3574	9.847	10.3538	10.4623	8.377	6.2888	7.721	8.1187	8.463	7.2637	8.3794	9.2539	9.8555	7.0014	8.0647	8.6273	7.6975	7.5481	9.9266	8.5332	7.5568	9.2296	10.1111	9.3512	10.1237	11.1671	7.9167	9.5368	7.3515	7.9099
TCGA-44-A47A-01	466	0	2.8362	5.4251	8.8711	9.9561	10.981	8.8376	1.3422	3.9856	7.2765	8.5746	9.0847	7.3048	4.8653	9.2307	9.2599	7.233	9.9479	5.4922	5.9988	7.7782	7.8504	7.9963	6.7127	9.9957	9.7563	9.3521	7.3643	8.3844	8.1602	8.4128	8.7041	9.7572	9.1959	7.7983	9.9921	6.6843	8.6353	9.8656	7.9523	10.0676	10.2533	8.6592	7.6221
TCGA-44-A47B-01	287	0	5.931	7.9735	8.1083	10.3539	8.4835	6.3897	2.1962	5.4295	8.3838	8.3253	5.888	5.9415	4.4916	8.7543	7.7783	9.2686	8.7347	3.806	4.5479	7.3298	8.004	8.3413	5.8436	8.3631	10.0938	9.1197	4.8006	8.0265	8.3213	7.6	10.3026	9.8199	9.8168	7.3258	9.9471	5.149	9.822	9.2559	9.8856	9.9399	8.8871	7.7695	8.5068
TCGA-44-A47G-01	351	0	2.7557	7.1611	9.2343	9.7131	9.3186	6.3598	2.6272	4.5859	8.1639	8.9786	8.5573	7.9961	5.0788	9.5806	7.7978	5.857	10.4645	5.3264	6.3188	6.7397	7.6193	8.0905	5.1271	8.4734	9.8013	9.2963	5.6741	7.7172	8.9687	8.1443	6.9219	10.1789	9.1737	12.0535	10.6516	7.8052	10.0337	10.3452	9.2937	10.333	9.4537	8.2103	6.4192
TCGA-44-A4SS-01	415	0	1.9143	6.9729	10.2196	8.4279	9.1388	6.4496	2.5813	5.2094	8.4228	9.1087	7.7652	8.0018	5.7296	9.8286	8.1243	9.5546	9.5332	8.8819	5.7145	7.1577	7.1633	8.3394	5.2933	8.5587	9.1231	9.8599	4.6962	7.4367	8.266	7.9861	7.2655	10.1802	8.46	10.1726	10.5214	6.7006	9.7749	10.3936	9.0795	9.0616	9.5071	8.2763	6.04
TCGA-44-A4SU-01	409	1	4.5854	6.7866	9.6981	9.6834	10.174	5.2866	4.8431	4.3399	8.4483	8.1904	8.8839	6.8816	6.1297	9.321	9.6142	9.3811	9.432	6.829	6.1394	8.2517	9.751	8.7169	5.555	9.2006	10.0912	9.9038	5.8766	8.5057	8.744	8.9863	6.811	10.5369	8.2976	12.0981	9.0643	8.9374	8.3751	9.018	9.1091	8.6255	10.0579	7.4057	8.5978
TCGA-49-4486-01	2318	1	6.1529	6.0696	9.6251	9.9578	10.279	8.6676	1.5825	2.4121	6.4278	9.0334	8.4989	7.3736	2.4995	8.1729	8.0553	4.4559	7.5094	3.9662	4.8209	6.1928	10.009	7.4086	4.8872	9.7662	10.8258	11.0125	5.8709	8.8657	7.3801	9.2403	7.5015	10.4768	5.6594	5.3773	10.8896	4.2445	9.7485	7.2787	9.7623	5.0697	9.6257	9.2482	7.6184
TCGA-49-4487-01	855	1	5.5389	8.2533	8.3118	8.3644	10.457	6.1652	0.8817	7.8464	8.2292	8.1713	9.6718	7.6118	4.088	8.4846	7.7816	6.7771	8.7074	4.2243	5.3433	6.964	7.3087	7.7981	6.4418	9.0222	10.6247	10.3189	5.9658	8.8591	8.5606	8.2453	7.9434	10.5194	8.1154	5.5393	10.5086	9.4183	8.9181	10.6224	9.9599	6.3992	9.9877	7.4405	6.7771
TCGA-49-4488-01	869	1	2.9268	5.6696	9.0299	8.4645	10.377	6.0827	8.1133	3.6714	7.6215	9.2511	8.0902	6.6967	2.4337	9.5498	4.3276	6.1439	6.923	3.2936	4.2181	4.2181	9.696	7.4565	5.7893	9.6011	10.6933	10.3302	5.827	9.2854	8.7193	9.0817	9.309	10.1731	8.1056	6.717	10.8946	8.8672	9.7851	10.0367	9.5694	9.6664	10.093	7.7101	5.2078
TCGA-49-4490-01	385	1	4.0801	7.4598	9.8778	10.2863	9.5541	6.076	2.506	4.7661	8.8478	8.7566	9.3227	4.4937	7.0853	9.755	8.6531	8.7906	8.9077	6.3152	6.0354	8.6698	8.7123	7.706	3.9108	9.0393	10.3621	11.2606	5.7644	8.6863	8.0188	8.1338	7.389	10.4774	8.1904	10.8266	9.9354	11.3451	9.029	9.1021	7.812	9.047	10.5789	7.9711	6.6471
TCGA-49-4494-01	1081	1	1.4349	6.5523	9.0312	7.745	10.38	5.1669	0.8889	2.7997	7.1815	9.1376	8.7026	4.7765	4.2395	8.8672	5.0605	9.3627	8.6199	2.4707	3.8425	6.4853	9.1513	7.3723	7.8012	9.2488	10.699	11.6832	6.574	8.0331	8.504	7.9142	7.4726	10.6049	7.8464	12.8171	10.99	7.2646	10.4704	10.9894	9.5277	9.9199	9.5421	8.1574	5.9354
TCGA-49-4501-01	1421	1	4.8582	7.4101	10.6395	9.6533	10.022	8.016	4.3978	6.3266	8.9927	8.6729	8.3143	7.0903	5.0545	9.6337	5.6326	8.0766	7.9997	7.5364	4.5356	8.219	9.8651	8.1522	5.5937	9.2476	9.6879	10.0676	6.05	9.3458	8.1562	8.6569	7.6736	10.5143	8.5162	7.8883	9.3615	10.932	10.0619	9.1979	9.8938	9.0824	10.2511	8.4534	6.3733
TCGA-49-4505-01	428	1	3.7981	7.5147	9.5766	9.3864	9.7874	7.7991	4.3873	5.4202	7.9091	9.2434	8.9779	7.0993	5.1276	10.4013	5.9189	6.2306	8.0776	5.0925	5.1276	6.8448	8.3636	8.2226	4.8044	9.0817	10.2112	10.0729	5.5029	9.3515	9.2706	8.1873	7.2717	10.1857	9.7034	11.5405	9.5547	10.0942	4.4092	7.7991	9.6091	9.6215	9.9345	7.541	6.0469
TCGA-49-4506-01	999	1	2.0648	5.8358	9.7162	8.0491	10.388	8.0812	7.2002	8.2919	6.4562	8.4981	8.1127	5.9045	2.9571	7.5941	6.3038	7.434	7.7962	5.6652	6.2819	4.7263	8.9378	7.0084	7.2196	9.8719	10.5842	10.2366	8.7131	9.9754	9.5151	8.6344	8.0939	9.7311	8.4265	9.7182	12.0464	6.7503	10.2174	11.7588	10.0504	9.7829	8.2128	9.0343	6.1013
TCGA-49-4507-01	268	1	4.7884	9.3302	8.7007	10.7352	9.9426	4.5536	2.9851	5.3497	7.7559	9.4006	7.6204	5.0667	3.9568	9.5291	8.0719	8.4297	8.2948	5.6171	5.7621	3.7122	8.0939	7.6255	7.378	9.7127	10.9505	11.4349	5.7435	10.1571	9.2407	8.5781	8.2063	7.7398	10.137	5.8769	9.8515	9.7068	10.6818	10.722	9.5644	9.4678	9.0055	8.721	6.2159
TCGA-49-4510-01	896	1	3.6058	5.9313	8.965	8.6245	9.9718	6.8005	3.4876	6.7198	8.4882	8.1114	8.0777	5.8378	3.5675	9.583	8.4392	7.8106	9.785	8.407	5.0552	6.5033	8.9924	7.6083	4.5453	9.3317	10.6349	10.5914	5.2125	9.2874	7.48	8.7139	7.0948	9.4358	7.6774	10.2508	9.8063	9.4706	10.3684	6.6109	10.903	8.9105	9.9008	8.4392	7.3812
TCGA-49-4512-01	905	1	3.8142	7.6555	9.1562	10.1886	10.297	6.4057	2.8378	4.0994	8.1294	8.9203	9.0515	5.6713	5.6045	9.8081	8.0097	5.5582	9.2862	5.1534	5.8161	7.4864	9.6842	8.3763	4.9169	9.2448	9.9539	11.3348	5.7142	9.164	7.72	8.7683	10.0044	10.8085	9.0004	7.1773	8.8085	9.7475	8.801	8.9018	8.0153	8.6893	10.1877	7.8456	6.101
TCGA-49-4514-01	1700	0	3.727	4.1564	9.8113	10.805	10.595	4.4368	3.9576	2.2707	7.2981	9.0921	9.9281	5.2948	4.6275	8.5424	7.7081	8.1385	7.928	6.1882	5.8484	8.1848	9.7803	8.1188	6.9652	9.4815	10.6356	11.3345	4.027	8.8433	8.7769	7.9726	7.7081	10.5877	6.5123	7.1707	8.678	9.9606	10.3557	10.3912	8.0455	8.2943	9.5226	8.8356	7.2954
TCGA-49-6742-01	488	1	3.6071	6.0417	9.8392	9.2515	10.161	5.3407	3.0802	6.6678	9.394	8.8583	8.6028	3.8503	4.6359	8.5435	8.121	8.8835	7.0307	4.4999	5.7534	8.0293	8.2574	8.6703	6.268	9.5559	9.7103	9.1548	5.8684	8.2325	7.1567	7.666	7.9002	10.3218	6.2956	10.8195	10.26	11.0577	10.584	11.1814	10.4201	8.0169	8.9394	8.4075	8.6913
TCGA-49-6743-01	1621	0	2.2625	7.6681	10.115	9.5587	9.4698	5.2847	2.8008	7.1778	8.8437	8.5852	4.7573	7.6132	3.6316	9.0156	7.0359	8.9741	7.4836	4.3213	5.0916	9.7159	8.1147	9.1177	7.7173	8.4395	9.5793	9.5293	5.7739	8.3844	8.6587	7.398	7.9909	10.4256	8.7231	12.9073	11.3365	10.446	9.4895	11.308	9.6858	8.9056	9.4509	7.0886	6.7679
TCGA-49-6744-01	1683	0	2.6068	8.2233	9.1721	8.8157	9.5836	6.9626	3.609	6.648	9.033	8.52	7.3924	8.2621	6.9086	9.9376	6.1448	7.7943	8.9716	7.5139	4.9796	8.3138	8.4998	9.0205	3.416	8.3704	9.5682	9.2788	5.2153	8.596	8.6036	7.388	7.1135	10.117	9.518	9.4345	9.6736	10.0815	10.1471	9.1911	9.2023	8.8124	10.0395	7.1501	8.2859
TCGA-49-6745-01	522	0	2.3019	6.75	9.1043	9.5934	10.159	5.7828	6.0522	6.2041	9.7822	8.145	8.8789	7.9524	4.9065	9.3695	6.451	7.2088	9.7299	8.1324	5.5754	8.1069	7.9863	8.7114	6.3503	8.4789	9.5334	9.9679	7.5065	7.6008	9.3326	6.5144	7.315	10.5185	8.968	8.3674	11.919	5.4018	9.6702	11.0063	9.7249	9.199	9.4344	7.2698	5.7568
TCGA-49-6761-01	354	0	4.6126	6.9393	8.8333	10.9546	9.6963	4.0355	4.776	3.5849	7.4494	8.9412	8.6141	6.1871	3.0588	8.3865	6.7458	9.6744	8.1161	4.0986	5.6892	5.6047	8.7132	8.0256	5.8628	9.9441	10.0939	10.2002	7.7733	7.8162	9.5331	7.4434	7.6989	9.729	7.942	11.444	9.755	5.3945	9.3096	9.7538	9.2877	8.9434	9.1678	6.5339	5.6892
TCGA-49-6767-01	677	0	2.9086	6.3794	9.0205	8.9166	9.7574	2.186	0.6706	2.7902	8.0266	8.6602	8.4221	5.5951	5.2131	9.7876	6.4297	7.6805	9.2538	2.186	5.485	5.911	8.1787	8.3995	7.148	9.2883	10.202	12.5005	7.0223	7.9354	8.4419	6.9688	7.4113	10.0669	7.0865	3.2147	12.574	10.2589	8.6707	10.6527	9.2326	6.2052	8.989	8.0002	4.0343
TCGA-49-AAQV-01	677	1	4.393	6.1538	9.7065	9.6464	9.946	4.3472	4.4804	3.6417	7.4883	8.9126	8.6762	5.592	5.3467	8.8483	8.9398	8.1684	9.1062	7.6088	6.206	7.4688	9.0349	8.3821	6.8839	9.6339	10.0185	10.3515	6.3809	9.2288	8.8452	9.2185	7.3079	10.5096	8.0177	9.996	9.7842	10.482	8.8788	10.4667	8.9694	8.7251	9.6569	7.5669	7.1929
TCGA-49-AAR0-01	4765	0	4.5119	7.1924	8.8857	9.2778	9.955	6.6953	3.9886	4.3807	8.2546	9.0753	8.2709	5.588	5.7824	9.9398	9.0579	8.5914	10.4559	7.5728	5.8082	7.7593	8.3165	8.6226	7.6702	9.1838	10.4761	11.6693	6.086	9.714	7.4636	8.9376	7.4308	9.438	8.3098	7.4797	10.526	9.5529	9.095	9.5914	8.2142	6.7823	9.8924	8.1677	10.1344
TCGA-49-AAR2-01	2224	0	2.9645	6.2687	9.0978	9.9217	9.9732	4.1461	1.1618	3.5258	7.0444	7.8333	8.7009	6.5799	5.6041	9.3982	9.6367	7.6676	11.7203	8.213	6.03	5.6223	7.632	7.9713	8.204	9.8577	11.181	11.2916	5.7271	9.3756	7.5487	9.2997	6.977	10.3819	7.8566	7.3441	9.6192	11.3587	10.5024	9.7163	9.2969	6.6258	9.8246	9.3715	9.8872
TCGA-49-AAR3-01	1893	0	3.2796	6.8686	8.7468	8.4809	9.283	5.8485	0.9039	4.6662	8.3013	9.0515	6.4469	6.5172	6.3423	9.5493	7.5634	7.4673	10.1151	5.7113	6.6975	4.6612	7.1509	8.1503	6.9723	9.1254	9.6181	11.1053	7.2697	8.7468	8.7728	8.1053	8.4088	10.1487	8.2772	7.8891	9.6944	10.2929	8.3826	10.745	8.2609	8.1503	9.4486	8.3976	6.2296
TCGA-49-AAR4-01	879	1	4.4878	7.0041	9.1218	9.3771	10.008	3.5024	2.2579	2.7387	8.0281	8.8826	9.3184	7.6547	5.2555	9.582	8.9939	8.1913	10.4357	6.7234	6.1749	6.9463	8.2037	7.8208	6.3524	8.2671	10.2739	10.1311	5.649	7.6181	8.0336	8.8846	7.7165	9.7565	9.8089	10.5505	8.442	8.7066	10.1576	9.0413	8.3996	9.9378	8.678	7.3209
TCGA-49-AAR9-01	260	1	2.2239	7.8153	9.3543	9.7975	7.0964	2.5965	0.5449	3.5305	7.1718	8.4022	7.462	3.3475	2.2239	9.257	5.5653	8.9746	6.0679	9.0676	5.6731	9.5347	8.8976	9.1126	7.072	8.0959	10.9354	11.1121	5.5083	8.6727	8.7593	7.4278	7.3689	10.5266	8.5826	11.6477	11.5792	10.5212	9.2018	11.7279	9.0187	5.8326	8.7531	7.4354	5.5653
TCGA-49-AARE-01	1229	1	1.7118	7.3571	9.1067	7.7137	10.581	4.3942	2.3495	2.692	6.5312	9.5642	9.5917	4.5977	3.5758	9.2878	8.9105	3.8278	8.5722	5.1899	6.2325	9.1031	8.5033	8.6214	6.6005	8.9023	10.5899	10.89	5.5814	7.4198	7.8046	9.1844	7.5482	10.629	6.488	6.3738	10.7797	10.9645	10.1723	10.8599	8.0045	7.3651	9.1466	8.4924	11.3701
TCGA-49-AARN-01	1135	1	4.4025	7.0152	9.3776	10.5546	9.9715	5.7743	1.3176	4.6369	7.3614	8.5983	9.5937	5.8509	4.5009	9.6893	8.19	7.6295	9.3046	7.6076	5.2876	8.2047	7.8929	8.1565	5.2876	8.7832	10.4484	9.8716	5.2598	8.3237	8.1221	8.7156	7.6185	9.8751	8.1144	12.869	9.6604	6.8571	9.4285	9.7139	9.1502	9.0723	10.7807	7.7394	8.5503
TCGA-49-AARO-01	3759	0	3.0866	7.8911	8.9782	9.3176	9.8373	5.8163	3.2702	5.5044	9.2909	8.4646	8.1354	6.9785	5.3084	10.0894	9.36	7.3741	10.3164	7.0111	5.6606	7.4282	9.0584	7.6009	5.8455	8.5994	9.971	10.0366	4.9262	8.3946	7.7475	8.9124	7.7667	10.0126	9.9618	7.575	10.5987	9.9551	8.2132	9.689	8.8543	7.5441	10.2373	8.5624	7.8875
TCGA-49-AARQ-01	6732	0	4.369	8.4437	8.6932	9.6004	10.381	5.5218	2.7264	2.5984	6.1038	9.25	8.2497	4.4454	6.4699	10.0158	9.1999	6.0724	9.5265	7.6947	7.0371	7.6018	7.4197	8.8366	7.5159	9.0022	11.0936	11.8038	5.2077	7.48	7.8327	8.817	7.4007	10.4888	7.2963	6.8176	10.5726	9.5254	8.523	11.1793	8.6044	8.808	8.9075	8.8295	6.6502
TCGA-49-AARR-01	4992	0	2.022	7.4258	8.9241	8.3216	9.3158	6.9949	6.1787	8.1662	10.6398	8.27	7.7841	6.8039	4.1151	9.4076	9.3135	7.6981	9.8245	9.632	6.1479	8.8155	8.0516	7.7774	3.4861	8.7349	9.7219	9.3602	4.9353	8.2676	7.057	9.9324	6.5289	9.6067	8.5668	9.2653	9.3444	7.5644	9.8318	7.3145	8.3489	7.804	9.985	9.6964	10.3176
TCGA-4B-A93V-01	300	1	4.4388	9.2253	7.9446	9.4749	11.783	5.632	5.632	8.3582	6.1643	7.9162	4.3047	6.6454	1.9585	8.8649	7.8665	9.6664	10.2351	2.2776	6.4289	5.6459	8.7094	8.2067	6.8336	9.302	10.8419	10.8233	6.596	9.7348	8.7243	8.9459	6.5375	11.7618	7.3456	7.1784	11.6685	8.7615	10.6353	8.6536	9.5397	8.3174	8.0994	8.368	7.6417
TCGA-50-5044-01	624	1	4.4243	8.2861	8.7512	7.8213	9.72	5.7053	1.0169	4.2016	7.9161	9.0834	6.2999	5.3181	3.3522	9.312	6.4926	6.0992	7.8789	5.7878	5.8143	8.3281	8.8149	7.67	6.1828	8.976	10.1117	10.6989	8.0778	8.0669	8.1984	8.1472	7.8789	9.7648	8.0833	9.2283	11.018	7.1208	10.125	11.4135	9.0222	6.9159	8.8977	8.3418	5.7053
TCGA-50-5045-01	2174	0	3.4235	7.7682	8.9904	8.6511	10.225	7.9495	5.4916	6.1542	9.6669	9.1738	8.077	8.2189	5.8606	9.8587	7.1655	7.1655	9.6069	5.9815	3.903	7.0551	8.5076	7.2521	7.2561	9.4412	10.3145	10.5614	5.7288	8.8743	7.863	8.5259	7.5315	10.6075	9.0811	10.1335	11.0976	9.2257	10.18	9.9107	9.8928	7.6607	9.8264	8.5242	6.4617
TCGA-50-5049-01	3094	0	1.0814	7.7834	8.4035	7.9193	9.8404	7.8195	4.1937	6.7716	8.8941	9.186	7.5754	9.0948	5.5979	9.8742	6.1561	8.0767	9.2771	4.6436	4.5436	6.99	8.1265	8.4686	5.8279	8.9012	9.6258	10.0348	5.9887	8.3203	8.5395	7.8616	8.2846	10.2304	9.4108	9.0254	9.9519	8.2131	9.9511	9.5761	9.9115	7.9843	9.7319	8.0189	6.2115

TCGA-50-5051-01	478	1	2.4976	8.1125	8.6323	9.7326	10.096	5.1642	1.7328	7.0349	7.7996	8.3399	8.7011	5.4717	5.1642	8.9013	9.5642	9.1359	9.4915	7.3883	6.3221	7.599	8.663	7.9991	5.9406	8.8047	10.6478	10.8393	6.5538	8.8896	8.0251	8.4334	6.2208	10.3983	7.9183	13.4558	10.2298	6.8535	11.54	9.2705	9.5463	7.672	9.7521	7.8029	10.3313
TCGA-50-5055-01	1830	1	3.0832	7.0738	9.1292	8.5043	9.6136	8.2604	4.4439	6.0756	9.3758	9.2304	8.1424	8.6487	5.6911	9.3728	6.8428	8.1842	9.6028	6.982	5.2621	7.5149	8.8594	7.7163	6.4075	9.2043	9.9431	10.3879	5.1653	9.1504	7.5473	8.3365	7.4131	10.6392	9.2891	8.8307	8.9225	9.7534	9.921	9.5171	9.4347	8.095	10.3154	8.99	7.2096
TCGA-50-5066-01	1442	0	4.06	8.6119	9.1344	7.3642	9.9546	9.0228	6.9109	4.9362	8.7897	9.5273	7.7147	8.1635	5.5597	9.3955	5.1636	7.9765	8.1981	5.449	3.9826	6.4894	8.4914	6.9317	8.5893	10.2062	11.3555	11.48	8.0208	9.2233	8.9662	8.5259	8.7022	10.4276	8.2319	11.0068	11.3873	10.0129	10.6867	11.3684	9.7884	6.9214	9.3519	8.325	4.3337
TCGA-50-5068-01	1499	1	0.8915	6.0062	9.24	7.4335	9.7227	8.2424	2.616	4.3083	8.8954	10.4747	6.8527	7.0518	5.0285	8.8567	5.8439	6.0062	8.1757	5.9869	3.5988	7.1155	9.9703	7.55	6.7984	10.0282	10.8604	11.3601	7.2912	9.8697	8.8514	9.3405	10.7219	10.8417	8.7375	12.0354	9.4362	5.7323	11.0614	10.0341	9.6555	8.4542	9.2359	9.0105	5.7779
TCGA-50-5072-01	250	1	3.4195	7.0614	10.2065	10.3715	10.431	7.6344	3.9372	8.7045	8.3735	8.3372	9.1758	5.5174	4.3174	8.7454	8.5583	7.781	7.4332	5.1176	5.7457	7.802	8.3938	8.6607	7.741	9.5161	10.465	9.9185	6.4868	9.1349	8.9151	7.2614	8.4154	10.2003	7.5657	11.1533	12.3455	10.4819	10.2211	11.6023	10.3554	8.2654	9.0608	8.0179	7.4637
TCGA-50-5930-01	282	1	2.9336	9.0666	9.5014	9.9461	9.525	7.0923	4.0821	7.1874	8.5218	8.272	8.2782	8.5679	3.6952	9.2943	5.3981	9.1019	9.3003	7.9849	4.7133	7.0496	7.9924	8.0586	5.9251	8.3679	9.5457	10.5227	5.9251	8.1825	8.4955	8.0941	7.2642	10.2466	9.2446	10.0217	10.75	10.4506	10.6711	9.5811	10.2575	8.527	9.8987	7.7822	10.1453
TCGA-50-5931-01	434	1	4.3625	6.9258	8.4646	8.7909	8.5522	5.2333	2.7402	5.5954	8.2351	8.0866	8.4465	6.6087	5.9301	8.7192	9.5815	9.3898	7.6789	8.0731	5.0253	5.7042	9.6156	9.8309	5.8535	8.8513	10.6998	9.8148	6.1513	8.8133	9.43	9.2502	8.3154	10.813	6.7338	10.7014	8.8194	8.9036	8.5078	10.8588	7.384	5.5572	8.8113	7.6565	5.8216
TCGA-50-5932-01	1235	1	1.4128	5.8251	9.4811	9.3448	8.9187	5.533	3.7515	5.3831	8.1284	8.218	7.2467	5.9565	3.2192	9.0353	7.2546	9.3337	10.4901	4.65	4.989	6.2043	8.4397	8.8161	5.0077	9.0625	9.2534	9.4944	5.0445	8.796	7.9316	8.2983	6.6001	10.6606	7.039	5.937	8.5333	10.4963	10.0628	8.6677	10.2229	10.424	10.2761	7.4143	5.7379
TCGA-50-5933-01	2393	1	2.7253	8.0926	9.3444	9.4471	9.8008	6.2957	2.022	6.3327	8.6268	8.4722	8.5665	7.8934	5.9115	9.8614	6.5128	7.1376	8.8637	7.599	5.1159	7.1789	7.2189	8.3757	5.9236	9.2141	8.9119	9.4429	7.148	8.0681	9.6522	7.7016	7.9805	9.7167	10.079	7.0291	11.0388	8.8996	8.8024	9.8172	10.0778	9.0818	8.9511	7.0347	3.9816
TCGA-50-5935-01	653	1	4.185	7.4231	8.9934	9.0927	9.8776	5.9862	3.8449	5.0647	8.0265	8.4586	8.8535	6.9965	4.2346	9.2397	7.673	9.653	8.1186	8.8693	5.5496	9.838	8.4108	8.5348	4.98	8.4664	9.3992	10.1612	5.6627	8.5099	8.5715	8.8455	7.032	10.4821	8.03	10.0162	9.7468	10.7465	9.4261	7.3234	9.463	9.1226	10.1361	7.6549	6.9821
TCGA-50-5936-01	257	1	2.2188	6.8182	9.6931	9.5314	8.8639	5.9294	5.8401	6.2396	8.9722	8.7704	8.7263	7.1727	3.5807	8.7583	6.3855	7.8939	7.3768	4.4241	5.3901	6.7215	8.7944	8.7288	6.1826	8.9958	9.9683	9.351	6.9262	8.7011	8.1603	7.244	7.764	10.5795	9.1852	13.2355	10.7274	9.3639	10.0855	10.1639	9.6078	8.9303	9.1743	8.2669	5.3647
TCGA-50-5939-01	460	1	1.9016	7.7166	9.3599	9.2179	8.9269	6.9638	3.3065	8.3725	8.2577	8.2611	8.5061	8.0239	4.6406	9.614	7.5316	7.5369	8.2992	4.894	5.1094	7.5369	8.0576	8.5516	5.7462	8.0427	10.0006	9.4558	7.9855	8.0239	8.7753	7.3193	6.6467	10.0875	12.381	9.9938	11.2937	10.3685	9.5377	10.4618	9.5297	8.7617	9.397	8.5516	4.826
TCGA-50-5941-01	1474	0	2.759	5.7941	9.2374	9.8133	9.7481	6.4198	3.9767	5.4366	8.4386	7.9604	8.1052	8.7194	4.8681	9.7503	6.4306	9.0372	9.0771	7.5658	5.2303	6.8387	7.9266	8.2879	6.26	9.4253	9.7727	9.4833	6.0666	8.3572	8.7194	7.4004	8.4943	9.5253	9.69	7.8881	10.1775	10.8027	8.7901	10.0145	10.0314	9.7968	9.991	7.59	6.6211
TCGA-50-5942-01	1847	0	2.4529	4.5069	8.95	9.5352	10.175	7.2649	4.0306	7.0025	9.5944	7.7277	7.5861	8.4724	4.1393	8.5433	7.8081	7.7321	7.6609	7.4661	5.0429	9.667	9.3284	8.7694	2.6122	8.6912	9.1773	9.2832	5.0429	7.8163	7.9714	7.9677	8.8375	10.0035	9.5672	9.3341	9.0525	6.2441	8.8948	6.7171	10.0581	10.2664	9.7095	7.5909	7.624
TCGA-50-5944-01	1750	0	3.6539	7.1608	9.1424	9.4585	9.777	7.5375	4.8906	6.7674	10.3465	8.003	8.6256	7.221	4.7007	9.5715	7.0916	6.587	9.5316	6.743	5.2323	8.8385	9.3933	8.8035	5.0046	8.5895	8.8595	10.1481	5.1603	9.2063	7.9788	7.7929	7.5469	10.0039	9.1656	8.9811	10.2757	9.2387	8.8633	9.1686	9.7061	8.9024	10.2571	6.4224	5.6377
TCGA-50-5946-01	1617	0	4.6324	8.0848	9.581	9.7195	9.7414	5.0233	0.7166	5.3421	7.4187	7.9637	9.5222	5.376	5.494	9.275	8.052	10.0821	9.6281	4.7053	5.6592	10.5528	6.9713	8.5744	7.3667	9.3615	10.4138	10.074	6.1125	8.4284	9.077	8.4525	7.4828	9.59	7.2281	13.2539	9.4121	10.354	8.5071	11.7055	9.2417	8.3693	9.3495	6.4831	6.0225
TCGA-50-6590-01	1288	1	2.9111	5.873	8.9749	8.3487	9.5253	5.3841	1.6663	5.287	8.9444	7.868	5.0472	7.8443	4.8439	9.8353	6.0491	6.5947	8.8799	3.0117	4.9743	5.7762	8.6337	9.1257	7.3722	9.3881	9.2181	10.4734	7.228	8.1311	11.3708	7.2944	8.5483	10.4517	9.1411	4.5071	11.7863	7.9809	9.5816	10.4399	9.9867	6.6347	8.8948	7.8062	4.8163
TCGA-50-6591-01	119	1	3.6946	8.6047	7.7311	8.9993	8.2273	7.5406	9.2605	8.6179	6.9749	8.5597	8.6267	5.0295	3.4797	8.6505	6.6718	7.2773	5.4401	6.9126	6.8392	9.0457	8.1137	9.8785	7.928	8.9374	9.7301	9.9101	5.7433	7.8885	9.6636	7.8442	8.8164	9.8526	7.7829	10.2244	9.7321	10.508	8.9566	11.6227	9.0667	6.9618	8.979	8.2099	3.9389
TCGA-50-6592-01	777	1	2.5134	6.8968	8.9994	8.3276	9.7197	5.4249	1.4397	8.8155	8.1442	8.039	7.2573	7.672	3.4951	9.3098	7.0445	8.7265	8.3711	8.0225	5.4533	8.5482	7.5521	8.6775	6.703	9.5709	10.8135	8.5857	8.9149	8.0716	9.7736	7.6227	7.7967	10.6112	9.4203	11.1734	11.2616	9.7167	10.315	11.2656	9.333	10.2536	8.7265	7.8242	6.7381
TCGA-50-6593-01	336	1	3.7211	7.3848	9.3474	9.9298	9.7998	6.4737	3.5734	6.1084	9.4865	8.6735	7.8374	7.2934	5.0193	9.9364	7.1895	7.9798	9.8786	7.0775	5.9527	7.118	7.788	7.8733	6.2967	8.9416	9.8815	9.781	5.4375	8.4952	9.078	8.0408	7.3681	9.6512	9.1466	9.41	10.5915	10.7347	9.0556	9.5671	9.5086	9.6976	9.5634	7.9276	6.9855
TCGA-50-6594-01	370	1	1.2681	6.0398	9.4431	9.8847	9.957	4.7198	5.3625	4.0438	6.7261	7.8623	9.1423	6.1865	5.7873	9.345	6.3196	8.8916	7.4738	6.6881	3.4408	6.144	8.9605	8.3124	7.2384	8.8787	10.2668	10.8464	5.5258	8.8959	9.9794	7.1624	7.901	9.8521	7.7297	6.1003	10.8275	11.0896	7.744	11.3985	8.0075	4.2177	9.3037	6.9239	4.0438
TCGA-50-6595-01	189	1	3.1238	7.5655	8.586	8.3362	9.061	6.4564	2.6193	6.7856	8.6854	8.9187	6.8271	6.7343	3.793	9.5853	5.8478	7.5655	6.9892	8.2566	5.5833	8.6265	8.5956	8.8897	6.4458	8.7393	10.2114	9.9259	7.0811	8.4678	8.848	6.8988	8.5812	10.0631	9.6991	10.588	10.0369	7.3941	9.5548	10.9711	9.7931	7.9576	10.011	7.8612	5.4411
TCGA-50-6597-01	1268	1	2.5141	5.4084	10.1954	8.0493	10.207	5.2387	0	0.9579	7.7604	8.6152	8.7634	4.6184	8.866	9.3904	6.6569	5.0046	8.8977	3.5069	6.6836	7.0649	8.7974	7.6765	3.2453	8.9995	9.8424	11.2132	5.749	9.2024	7.2098	8.6325	6.0857	10.5746	7.8218	4.6727	8.9119	10.4489	8.8276	8.3619	10.6383	8.5369	11.3382	8.9063	7.255
TCGA-50-6673-01	22	1	2.7243	6.3434	10.3025	9.9737	9.3428	7.2939	2.9505	6.3334	8.5828	8.3149	9.4761	7.2306	4.3269	9.5791	5.9958	9.8703	10.0118	6.4675	5.8492	8.883	8.7983	8.1366	5.6216	9.0662	8.8966	8.8518	5.4095	8.1902	8.0809	7.7118	7.787	9.4726	8.6144	8.9285	10.4653	10.8158	9.6402	10.0459	10.7425	9.9345	10.0094	8.7179	9.5738
TCGA-50-7109-01	308	1	4.9495	6.6505	9.0681	8.5486	9.8416	6.9572	4.0228	8.2051	8.1521	8.6767	7.99	6.8994	5.2095	9.6674	7.3352	10.0749	8.9106	8.1811	5.0592	7.7657	7.0531	7.8016	6.6505	9.4939	9.6412	10.3792	6.3603	8.4131	8.9069	7.9443	7.2452	11.0554	8.1254	7.0465	11.0001	7.8328	10.1574	10.1136	11.5824	7.6652	9.0197	8.3031	6.9501
TCGA-50-8457-01	1125	0	3.8395	7.5671	8.6066	9.9666	9.677	6.7305	3.6987	6.7896	9.576	8.3064	8.2402	8.7943	5.2248	9.3253	8.7399	8.06	9.5026	7.4438	5.34	8.2016	9.1046	8.7885	3.7941	8.1164	9.1526	9.7898	5.0031	8.9946	7.9242	8.6407	7.7418	9.9742	9.3763	7.8176	9.6097	8.9871	9.0999	6.6242	9.35	9.3935	9.5681	7.8732	7.321
TCGA-50-8459-01	1119	0	3.5398	8.8289	8.6047	9.26	9.9393	7.0917	2.7214	7.161	10.0054	8.5411	8.8448	8.6476	6.1022	10.3264	8.4308	7.4877	10.2901	6.8045	5.4061	7.9592	7.2325	7.9494	4.9641	8.2623	9.3218	9.9302	4.9641	7.8409	7.1325	8.0953	6.4176	9.5671	10.361	8.2884	10.2875	8.136	9.2022	8.4355	9.4794	7.681	10.4047	8.3012	7.4832
TCGA-50-8460-01	829	0	1.5154	5.1067	8.677	10.4709	10.355	7.1712	3.448	4.942	8.0538	8.4733	8.5729	7.2555	4.6173	9.6297	8.7115	6.0456	9.5545	6.3274	6.5345	7.8586	8.236	7.6827	4.5037	8.9561	9.9556	10.1498	5.795	9.0502	7.6652	8.9561	6.8763	9.8893	9.5628	9.8617	10.184	9.4239	8.3878	8.9899	8.703	9.1129	9.6081	8.3054	7.6564
TCGA-53-7624-01	1043	1	7.4792	5.1927	8.4002	8.7455	8.7991	3.0048	0.5253	3.0048	6.5895	8.0662	7.4863	5.047	1.862	9.5291	6.6726	8.335	9.301	4.0331	5.475	5.5847	7.8498	8.9956	6.8088	8.8048	10.2652	9.4074	5.9856	8.182	9.9038	7.9221	8.6094	9.6515	7.1804	6.3386	10.9512	10.7206	9.9307	11.4396	12.0098	5.4169	8.7991	7.5919	6.3307
TCGA-53-7626-01	929	1	4.0326	6.8252	8.6177	9.1089	9.4272	6.0967	5.9113	7.5476	9.6851	8.4376	8.6072	8.9074	5.1235	9.3799	6.9198	6.8889	9.477	6.9198	5.311	9.3688	8.0204	8.292	5.7064	8.0914	8.8256	9.8655	5.7298	8.2208	7.5993	7.4071	7.0053	8.9578	9.6027	12.8489	8.9303	6.973	9.1521	8.9603	9.4218	9.4759	9.8673	7.6614	8.9921
TCGA-53-7813-01	424	0	3.3575	6.6973	8.8974	8.2341	9.1082	5.2864	1.6114	5.4309	7.9158	8.4144	8.7536	6.5782	6.1264	7.5069	7.3702	10.5333	8.3431	7.423	6.3244	7.343	7.9281	8.436	7.1477	9.6923	10.892	10.2574	7.0504	8.2967	9.5998	7.8848	8.5901	10.1256	6.3058	10.8216	9.3155	7.7188	10.3937	9.3109	9.9598	8.1215	7.8075	7.005	6.3058
TCGA-53-A4EZ-01	1071	0	2.9427	6.465	8.6662	9.7604	10.185	4.9678	1.3638	3.2714	6.9235	8.8046	8.6648	4.3968	9.2217	9.0428	7.9818	10.1327	10.3789	3.7645	7.7615	7.9751	7.9997	8.3573	7.8986	9.6044	11.1071	10.9601	5.9072	7.9841	8.6759	8.3026	8.0732	9.8917	7.7797	5.7261	11.7187	9.4555	10.8457	10.6334	10.3747	8.2098	8.8122	8.1147	5.8395
TCGA-55-1592-01	701	1	3.6597	8.7617	9.9006	8.6822	9.5931	6.885	3.4254	5.35	8.2154	8.7427	8.3585	7.2352	5.5213	9.817	9.1122	8.9208	8.7617	6.8311	5.369	10.0938	8.0448	8.0269	5.6278	8.8972	9.7133	9.9762	6.1735	8.9184	9.3957	8.0239	8.1211	10.0152	9.1532	9.3371	9.4158	11.1964	9.6452	10.2078	8.5011	8.4859	10.4914	7.7793	9.283
TCGA-55-1594-01	1178	0	6.3221	7.7205	10.0573	9.6645	9.4607	6.2569	4.3956	4.4223	7.4901	7.6516	7.9045	7.0604	9.4041	9.7819	10.2395	8.3715	9.3864	3.8834	5.2158	7.073	8.2538	9.2051	9.632	9.0187	10.0847	9.4965	6.7798	9.3503	9.7454	7.8066	7.8242	10.1909	7.8588	8.8197	10.2893	11.0685	9.1642	11.5674	8.2238	8.4054	11.5122	8.4352	7.1942
TCGA-55-1595-01	1479	0	3.5834	7.2858	9.0438	11.0952	8.9174	7.0336	3.5834	6.3228	8.6098	8.0348	7.368	7.4152	3.7563	9.266	8.7659	8.7939	8.8146	4.5027	6.1225	7.6568	9.1178	8.3875	5.8445	9.1445	11.1408	9.5548	6.382	8.8465	8.7039	8.1891	7.8275	9.6652	8.3889	11.2968	9.0857	8.6512	9.5843	10.2787	9.0312	8.781	9.3385	7.156	6.0312
TCGA-55-1596-01	2065	0	1.9856	6.5898	9.7846	10.2	10.386	5.8976	3.0188	4.8787	7.0865	8.5262	8.0726	6.1827	4.4411	9.8388	8.3171	7.883	6.8189	3.1205	4.2297	8.0977	9.2727	8.6007	7.8974	9.7364	10.9609	10.447	6.2516	9.4961	8.8799	8.362	8.5075	11.023	7.1727	11.2384	8.5583	10.8712	10.5639	11.0373	8.3091	6.0472	9.5643	8.3198	8.5239
TCGA-55-5899-01	930	0	3.6358	6.0352	9.5055	9.6734	10.616	4.9139	2.9742	7.0368	8.1594	9.0238	7.7272	6.557	5.2318	9.4535	8.155	11.7479	7.9097	6.7145	5.3777	6.7834	7.7731	8.3621	8.0808	8.9966	9.8202	10.0707	7.1102	8.7296	9.5457	7.8322	8.6394	9.5402	8.7412	7.1572	9.184	10.5845	9.9417	11.8539	9.1481	3.2589	9.3448	8.4846	10.7168
TCGA-55-6543-01	435	0	1.418	6.0491	9.3757	9.5119	10.261	6.4123	2.2933	5.0097	7.9601	8.2984	7.8356	7.3207	4.0538	9.8335	6.8136	8.5401	8.6543	9.3938	5.6771	8.4019	9.0315	8.3731	5.2185	8.8447	9.4546	10.1811	7.0804	7.9144	7.9699	7.2851	6.8489	9.5425	9.4931	10.5252	9.1858	5.9223	9.5174	8.1506	9.3818	9.0114	10.7677	7.2218	8.5763
TCGA-55-6642-01	2449	0	3.6776	8.4279	8.7621	9.5457	9.8482	7.0734	4.0065	8.8951	9.3399	7.8312	7.9884	9.102	4.0065	8.9874	6.4833	9.5163	8.3917	7.8061	5.4461	8.4085	7.5171	8.5888	6.2914	8.729	8.6952	9.2413	5.9681	8.2468	8.7313	7.6781	7.2638	9.8471	9.1725	14.685	9.3768	8.4388	9.8665	10.0798	9.8013	8.5615	9.6797	7.5119	6.3505
TCGA-55-6712-01	171	1	1.8832	5.8446	9.3716	8.6593	9.059	5.8219	1.8832	4.6553	8.7758	7.9301	6.0921	7.9194	4.6553	9.0444	7.9821	8.114	8.0809	7.8925	5.5456	9.324	5.0165	8.7965	7.4051	8.497	8.7819	9.3057	7.5081	7.7793	8.6784	6.8767	7.6945	10.0948	8.7432	12.7797	9.4932	7.4722	8.8253	9.5478	9.4551	9.9868	9.9893	7.4575	5.9529
TCGA-55-6968-01	1293	1	3.4237	6.3218	8.5299	7.3936	10.551	5.7913	2.3239	5.3996	8.5586	8.9496	7.637	7.7791	6.1464	9.0956	7.3436	8.9988	8.6804	7.3936	7.039	7.8416	7.5516	9.5546	8.2196	8.4305	9.76	9.9573	7.4747	8.7796	9.4404	8.0461	8.1573	10.772	8.7435	5.892	9.376	9.0292	10.5197	11.6473	9.2662	7.637	8.8671	7.0762	11.1698
TCGA-55-6969-01	1239	0	3.8533	8.3269	8.6615	9.7058	9.8356	6.7308	2.9817	7.1294	8.6883	8.3098	7.9898	7.8884	6.5461	9.5742	8.036	10.0286	8.9391	9.8329	5.7945	6.6238	6.9679	8.8706	6.5881	8.7381	10.075	10.4978	7.3002	8.7795	9.3417	8.1768	7.9995	10.9346	8.9052	11.498	10.281	8.8175	11.0106	10.3321	9.3371	6.178	9.7925	8.2506	8.5781
TCGA-55-6970-01	464	1	3.95	5.3853	9.5984	9.3189	8.7531	5.94	4.8065	8.4493	8.4636	8.3703	7.6996	8.2718	3.95	8.6098	7.0968	7.3648	8.5217	6.854	5.5625	7.9203	8.7975	8.5846	7.0625	8.8981	9.5121	9.5936	6.5862	7.7671	8.6837	8.2926	8.2627	9.897	8.2597	9.5058	10.9486	7.2538	10.6606	10.6457	10.8885	9.8196	9.5978	7.4548	7.2075
TCGA-55-6971-01	1400	0	2.9591	6.7865	8.4451	8.4808	10.033	5.9763	5.2763	10.605	8.4444	8.0878	7.5973	9.0825	4.4148	9.2106	7.3543	8.4127	9.502	5.4778	5.5688	6.9759	8.6641	8.3542	6.4615	7.836	9.3962	9.4431	5.7089	8.0723	8.3152	7.4848	7.1032	9.9811	8.7542	11.9279	10.1844	7.5681	9.8282	9.664	10.8049	9.8374	9.4003	7.5233	7.2014
TCGA-55-6972-01	1632	1	2.7458	5.1854	9.893	8.2564	8.9151	7.0223	2.4767	2.1456	6.2106	8.7219	6.4366	2.9726	3.9855	7.8766	8.9818	9.5372	7.9181	5.4718	4.4295	9.213	9.2514	7.9316	5.6131	9.2648	10.0581	10.4832	5.7107	8.8945	7.9976	10.0902	6.6152	10.5692	4.0859	4.2679	9.3631	9.7851	10.0158	7.8267	10.0379	3.1685	8.891	8.3653	6.8897
TCGA-55-6975-01	118	1	3.2299	8.5534	9.2556	9.4004	10.832	7.2547	4.2174	7.6949	10.0157	8.8413	5.997	7.4541	3.4671	9.1742	7.1649	9.195	7.0326	5.4233	5.794	7.4949	7.5016	8.23	6.9761	8.8726	9.9947	10.1043	6.7018	8.0817	8.8039	7.3015	7.7465	10.204	8.6648	8.5789	12.1593	10.7257	10.3021	11.473	10.1326	3.9307	10.384	7.7799	4.841
TCGA-55-6978-01	176	1	3.5169	7.6193	8.7995	8.3956	10.102	6.774	2.5954	6.774	9.5625	8.2999	7.1224	8.2816	5.1237	9.5333	6.7158	7.704	9.6748	7.1447	5.407	7.8143	7.375	8.9116	6.7644	8.4411	9.4134	10.3579	6.5593	8.2149	9.2557	7.0923	7.4453	10.7032	8.9202	9.1231	10.1288	6.9608	9.1716	11.3899	9.9112	8.9479	9.4949	7.2678	5.5908
TCGA-55-6979-01	237	1	3.2413	7.6214	8.7929	8.4621	10.147	6.891	3.0084	5.5045	8.5873	8.9285	8.708	8.0965	8.8292	9.9186	7.2987	8.3038	10.2318	5.2071	5.633	8.3229	8.258	8.7791	6.4885	8.3636	9.4886	9.9953	5.9449	7.9062	8.4032	8.0965	7.7824	10.0823	9.7612	10.4327	10.2743	9.5844	9.3349	9.9508	9.7087	9.3127	9.6977	7.9725	6.5975
TCGA-55-6980-01	2109	0	5.4985	7.5715	10.1091	10.3924	9.167	5.8254	3.6432	5.6836	9.6507	8.4953	8.8368	7.2911	6.1467	10.3971	7.0525	7.3233	9.6618	5.5265	5.441	8.3465	9.0297	8.1761	3.6432	8.3965	9.1035	10.2159	6.2675	8.6366	7.6177	8.4003	6.579	10.2696	9.2171	9.9786	10.3838	10.3372	9.3657	7.5915	9.3753	9.1058	10.5857	7.9779	6.7547
TCGA-55-6981-01	1379	1	3.2886	6.6582	9.9144	9.9609	9.6846	3.7313	3.7313	4.8173	8.1462	8.3074	9.1556	6.6582	6.3829	9.996	7.4702	7.1521	9.3868	4.4043	5.2081	8.9534	8.8475	8.0784	6.3523	8.5697	9.7136	10.7558	5.4873	8.8801	9.0377	7.8118	7.4045	10.3493	8.5663	11.0813	11.0895	10.3847	8.4372	10.4441	9.3011	9.6007	10.816	7.8341	6.0231
TCGA-55-6982-01	995	1	3.3531	7.1596	9.4122	9.152	9.0652	6.7663	4.4115	5.8845	8.0463	8.1638	7.6247	7.3591	5.5854	9.8165	6.4538	8.0277	8.2273	5.0154	5.4398	7.9546	8.4829	8.908	5.0954	8.2537	9.025	9.4901	4.4727	8.1592	9.5901	6.8836	7.3911	10.4956	8.949	7.4682	10.8127	11.2468	8.8801	10.1623	9.9676	9.5266	10.1797	7.9705	6.3918
TCGA-55-6983-01	2823	0	2.8132	7.766	9.2978	9.3645	9.5615	7.6045	5.8336	9.8836	8.205	8.7461	7.5913	7.6383	4.7102	9.1335	7.1902	8.844	9.2312	8.6614	5.9623	7.3398	8.5347	8.6602	5.76	8.1957	9.4649	9.6199	5.9623	8.4713	8.4199	8.1764	6.6932	10.838	9.0645	11.0586	9.7994	6.8567	11.7379	9.4547	9.9733	9.0452	10.3399	7.5094	8.4436
TCGA-55-6984-01	760	1	0.9367	5.0441	9.7207	8.9367	8.6356	4.336	2.697	4.829	8.9082	8.1188	8.9041	6.1575	1.9039	8.3626	7.1287	5.3329	7.289	7.3469	5.2312	6.653	9.1444	7.7475	4.829	8.8336	10.2203	9.7983	6.9375	8.5918	8.5001	7.7898	6.4568	9.5394	8.8565	10.637	7.4257	5.4582	10.8413	10.6386	9.2629	9.7066	9.2082	7.953	10.1137
TCGA-55-6985-01	1233	0	5.0483	6.7709	9.1913	8.8336	9.9811	5.4928	5.3149	7.1578	8.4272	8.2479	7.9236	7.4065	4.5477	9.3857	6.5589	7.9412	9.5612	8.1375	5.5399	8.1678	9.044	7.9801	5.8509	9.0623	9.574	10.0488	5.4928	8.4706	8.5095	8.0829	7.9758	9.6893	8.6846	14.9964	10.3363	9.6789	9.2312	9.997	9.249	9.1765	9.964	8.4766	9.1765
TCGA-55-6986-01	3261	0	3.2811	4.9102	8.9791	9.9182	9.487	5.5024	2.7685	3.573	8.0161	7.845	7.7312	6.9856	4.0865	9.0619	7.1046	6.791	9.0775	8.0005	5.4791	6.652	8.8935	8.5179	3.6585	9.6208	10.3624	9.6034	5.6559	8.4355	9.8021	7.4606	6.9689	10.2541	8.5462	9.7604	9.7808	5.8501	9.9942	9.2371	9.4212	10.3648	9.8044	8.7716	6.5661
TCGA-55-6987-01	2137	0	3.5785	6.6622	8.6766	8.2644	9.5908	5.7913	2.7333	5.8891	7.8987	8.1914	6.2385	8.5064	6.7966	9.3198	7.2458	7.9428	9.7191	5.6564	6.036	7.1196	7.4606	8.2545	6.036	8.8676	10.3754	10.5622	7.9817	8.5814	9.0245	8.2275	7.4076	10.8775	9.7904	11.039	11.3559	6.855	10.6681	10.0079	9.7239	7.9614	9.6605	8.2561	7.0712
TCGA-55-7227-01	952	1	2.3636	6.8716	8.7511	9.4955	9.8348	6.7379	2.7068	4.8745	9.1794	7.9117	7.8221	7.7451	4.6145	9.7494	6.9136	7.583	8.9355	5.152	5.6054	7.8439	8.0906	8.2065	5.4082	8.723	9.6058	9.379	5.0352	8.1232	8.357	7.0627	6.318	10.1893	9.3998	10.2071	8.8211	6.0882	9.4968	9.2718	9.5642	9.9041	9.3225	7.4121	6.8108
TCGA-55-7281-01	872	0	3.3387	7.1701	9.2529	9.3456	8.5367	6.0559	1.9445	4.5016	7.6346	9.1011	8.5009	7.1119	6.5787	9.4736	6.5614	7.0387	9.5248	4.8822	5.8432	7.2533	8.9923	7.7306	4.1774	8.5278	9.3046	10.4709	4.4271	8.6819	7.8777	7.8985	7.3319	9.7789	9.8058	12.7235	9.6302	8.7768	9.3989	8.948	8.7936	9.739	9.887	7.6794	5.7239
TCGA-55-7283-01	609	0	1.5874	5.4465	8.9014	8.1785	9.4558	3.9103	3.9103	4.9577	8.6433	8.1457	6.9519	7.0148	4.0909	9.1572	5.5885	8.2008	9.3776	5.3786	5.3073	8.0008	8.4361	9.0008	5.112	9.7759	10.12	10.176	5.3958	8.7213	9.4651	8.4814	8.1934	10.4758	8.4403	12.3545	9.6393	9.1885	9.3568	9.6954	9.432	9.1316	9.7101	7.4382	5.8364
TCGA-55-7284-01	243	1	1.5211	5.3427	9.673	8.9782	9.8235	6.3022	5.4495	5.2513	9.3895	8.198	6.5285	7.5426	5.1281	9.9213	7.6427	8.7117	9.9539	8.5435	4.731	9.1376	8.6346	8.7654	4.6257	8.3268	9.5518	9.7212	5.1786	8.3586	7.2873	7.4556	6.9001	9.9539	8.7838	9.1408	8.6244	6.9076	10.7389	8.2956	9.4818	9.1886	9.6452	7.9053	10.5943
TCGA-55-7570-01	824	0	3.4041	6.7221	9.2977	9.3688	8.5025	9.4635	1.8538	4.4538	10.1315	9.252	3.4041	5.3606	2.4215	8.9768	8.4296	8.492	7.2744	7.8052	6.4689	6.1964	8.3662	9.4293	8.0945	9.5022	10.3964	10.1918	8.4833	9.4865	9.9952	7.7768	8.3814	10.2647	6.504	13.4748	11.4698	10.0561	10.7771	11.1947	9.1428	4.6418	7.9697	7.8052	8.1707
TCGA-55-7573-01	487	0	2.8885	6.191	9.8865	9.5985	8.9813	6.1097	6.4898	5.6158	8.6492	8.5286	7.6579	7.9978	4.5495	9.3181	8.4249	6.4105	9.1227	7.501	6.3969	8.7514	8.1438	8.0423	4.8984	8.6428	9.1123	9.0351	5.2414	8.6195	7.8098	8.5473	6.7904	9.7061	9.2111	9.7972	9.9155	7.0737	8.9396	7.4946	9.9463	10.0326	9.9393	8.16	8.2309
TCGA-55-7574-01	995	1	4.2674	8.6732	8.3224	10.1111	9.0683	7.9535	2.8778	7.4235	9.1707	8.3786	7.5693	8.4058	4.2066	9.185	8.2148	8.5132	10.1296	6.5648	5.6727	7.4033	8.1436	8.2825	5.9571	8.2031	9.4486	9.691	5.4809	7.7017	8.2862	7.8969	7.4033	9.8508	8.5442	9.7744	10.4035	8.1436	9.3094	9.133	9.76	9.6083	9.7143	7.9065	6.6471
TCGA-55-7576-01	670	0	2.9257	7.7812	9.9273	9.1621	9.4415	6.64	3.6702	6.64	8.1229	8.8222	8.22	7.5895	5.5225	10.0041	7.1045	7.8892	8.5644	4.3782	5.9621	7.7764	7.8082	8.402	6.4229	8.7347	9.7251	9.483	6.2623	8.5075	9.9327	7.4452	8.6094	9.8794	8.4431	10.7412	11.3355	9.0647	10.01	10.5681	9.3977	9.8407	9.5291	7.2529	5.5225
TCGA-55-7724-01	705	0	0	6.3604	8.983	6.8608	9.2626	7.4123	2.5874	6.0903	9.5887	8.4705	7.2413	8.2743	5.5264	8.7342	5.5878	7.4291	8.1878	8.5841	6.4947	10.127	9.7274	8.9571	6.3427	8.3293	9.3692	9.3293	6.3427	8.1878	8.2365	6.2508	7.5727	10.3538	9.4623	10.2975	10.7628	6.0903	9.2341	10.0784	8.96	8.6176	9.2221	7.3779	5.6753
TCGA-55-7725-01	442	0	2.0536	2.8684	9.213	7.5218	9.52	3.386	3.386	4.3894	8.6177	8.1529	9.0045	7.6853	4.237	8.1422	7.0011	8.8685	8.4501	7.4104	6.2197	9.3211	9.1794	8.6632	4.7121	9.8815	9.0765	9.4696	6.0923	8.4327	9.2508	8.0537	7.0477	10.2346	8.5343	9.4196	8.522	4.7687	9.1288	8.5666	9.8258	9.2358	8.2506	7.2897	6.6702
TCGA-55-7726-01	652	0	0.9163	8.413	9.0102	7.6801	7.9156	6.4441	1.8726	7.5149	8.8163	8.8307	5.2911	6.6365	4.359	8.3633	5.4163	7.1415	7.7708	5.1176	6.5297	6.5841	7.1325	8.0617	6.4875	8.6513	9.5692	9.8897	6.3373	8.7438	8.45	5.6887	7.8561	9.5826	10.856	11.6703	10.4679	6.0967	9.4388	10.3687	8.7438	6.7832	8.8279	7.3599	5.6122
TCGA-55-7727-01	119	0	2.8918	7.5487	9.4495	7.611	8.5054	7.0762	4.5247	3.4714	8.036	8.7586	8.3355	7.6642	3.8838	8.2641	5.127	7.8705	7.5837	4.0528	6.0673	8.3639	8.1522	8.8927	5.338	9.0829	9.9419	9.2895	6.697	7.8649	9.0332	7.1619	11.3919	10.4673	8.1288	11.4479	9.7947	8.3558	9.5376	10.3706	9.4033	10.0255	8.6801	7.4763	5.493
TCGA-55-7728-01	704	0	2.8674	3.5985	9.2055	9.8115	10.75	3.5985	1.3114	1.8452	8.4106	8.4662	8.2052	7.2065	6.8788	10.7891	11.3661	7.3769	11.5032	8.2339	5.5639	8.5211	8.6289	7.4024	5.1018	8.5399	10.0985	10.6855	4.2594	7.9308	6.4664	8.1431	6.6155	9.7674	9.7668	9.2675	9.8198	6.8467	9.0448	8.6544	8.9985	7.5148	9.3361	8.9256	10.161
TCGA-55-7815-01	773	0	0	7.7644	10.4725	6.0903	8.1627	7.2697	3.1725	5.5264	8.3427	8.7274	6.5107	7.9859	3.7031	7.5727	4.9097	9.8962	7.6894	3.7031	5.3247	7.6894	7.7711	7.6894	7.1424	8.7578	8.8038	8.7777	7.5727	7.8855	8.741	6.2123	7.2123	10.097	8.7875	10.3293	11.8286	6.2317	10.7957	9.9571	10.4827	10.5294	9.1653	5.2507	5.2123
TCGA-55-7816-01	468	1	0	6.8878	8.7832	7.847	9.4564	8.7972	4.407	6.4855	9.909	8.0196	6.6177	8.4091	5.2475	9.8777	7.604	9.7328	9.9882	8.1167	6.4715	9.7508	9.2302	8.5581	4.5463	8.2872	8.675	9.3548	5.6323	7.8281	8.2243	8.0409	6.8452	9.8987	12.604	7.2958	11.1357	9.2062	9.3271	7.9857	9.3904	8.5044	9.6426	7.4242	12.0566
TCGA-55-7903-01	567	0	5.1448	5.7365	9.2827	8.5986	9.3056	3.7502	1.5816	4.487	7.3602	8.0448	8.6478	6.1549	4.639	9.3337	7.2988	5.0612	8.5705	3.4548	6.0394	6.1346	7.5799	8.4022	5.4869	9.1885	10.5841	9.9808	6.2334	8.2453	9.5215	8.7415	10.2178	10.3492	8.5067	15.0194	10.5949	8.2117	9.027	11.3248	9.3248	8.955	9.038	7.1447	6.5342
TCGA-55-7907-01	343	1	2.859	7.6239	9.3751	7.9682	9.0075	6.2784	1.3582	4.3608	8.2607	9.3232	8.8551	6.8277	6.6255	9.4399	7.9771	5.8205	10.493	4.7442	5.6513	10.0246	7.9084	7.7911	6.43	9.1494	9.8115	10.3229	5.9538	8.5406	8.7852	7.5715	7.6239	10.8865	8.5254	11.0948	9.3631	9.9616	9.3751	9.4415	9.2893	9.4815	9.2211	7.5109	6.494
TCGA-55-7910-01	1040	0	1.9175	8.7189	8.4323	10.1825	10.273	4.7004	2.2338	9.3154	7.467	8.8463	8.6374	6.6974	4.2868	8.9418	9.8692	9.459	8.892	9.021	5.845	7.3491	8.3931	7.8261	6.9342	8.4515	9.7502	10.5356	5.284	9.3217	8.6103	8.1135	5.9781	9.8094	7.7287	12.7431	9.5721	9.7236	10.6688	10.7044	10.4253	7.8667	9.0666	8.2158	8.1977
TCGA-55-7911-01	537	0	3.5326	5.1002	9.481	10.9762	9.4021	5.0551	2.5254	3.597	7.8415	8.4464	8.9177	7.379	4.3617	9.0401	7.7201	8.4616	9.4322	3.7747	6.2591	6.3642	8.9975	8.5063	6.9061	8.8598	9.9115	9.9123	5.5543	8.4332	9.3126	8.1353	7.5269	10.5211	8.178	7.8315	9.7553	5.8602	9.369	11.6293	9.877	9.5498	9.4595	6.9744	7.0044
TCGA-55-7913-01	561	1	1.1029	5.4703	9.4572	8.8797	10.206	3.4272	6.0599	11.045	5.7783	8.377	8.1826	5.3936	6.9982	9.0171	10.3287	10.2799	9.6509	9.3394	4.1644	5.0996	9.3928	8.9807	7.588	8.4838	10.4366	9.7576	4.7912	8.8095	8.3556	8.724	7.7235	10.3335	6.9319	12.5676	8.5158	9.3903	10.8528	10.171	10.0141	9.0314	10.0981	7.7904	8.0119
TCGA-55-7914-01	187	1	3.7542	7.5539	8.4464	9.514	9.5992	6.0327	2.5844	5.2472	7.1833	8.2637	10.9658	7.0049	5.1891	9.2184	7.5384	9.2448	8.1104	8.4065	5.5992	10.1311	7.2377	8.2329	6.5767	9.6655	10.062	9.6954	7.9908	8.3167	9.829	8.3894	6.6359	10.452	8.0814	12.0089	7.6823	6.4911	8.4065	9.4433	10.6043	9.2484	9.635	7.3031	9.2742
TCGA-55-7994-01	603	0	1.3691	4.913	9.7967	9.9026	9.893	5.9567	2.1644	4.0177	9.126	8.4165	8.9884	8.0993	5.5023	9.8757	7.6518	8.2114	10.0097	8.0046	5.743	4.9724	7.6358	8.3396	7.0278	9.2687	10.3359	9.6768	7.9147	7.3497	9.0036	7.0828	8.6629	9.8887	8.4125	8.5071	11.5942	9.4779	8.4869	11.5489	9.7556	10.2197	9.5613	6.865	5.4717
TCGA-55-7995-01	889	0	3.1108	9.1359	8.3652	8.6167	9.3802	6.4383	1.5755	3.8659	7.2426	8.7497	8.6198	8.1363	6.0704	9.5635	8.0906	9.4791	10.1248	9.9138	5.8189	8.2084	7.8286	9.6345	7.4925	8.4702	10.5089	9.8413	5.634	9.2536	8.6177	7.6713	7.9721	10.7326	9.7878	6.153	9.7236	10.8423	9.4225	10.6464	8.6986	9.9163	9.5357	8.1862	10.3308
TCGA-55-8085-01	904	0	2.9281	5.5524	9.2713	9.6036	9.6778	7.2871	3.1704	9.1501	7.7491	7.7269	8.9187	7.0875	6.204	9.0637	8.5375	8.1133	9.9926	3.9647	5.2955	7.0387	7.7528	8.2921	7.6415	10.2065	10.9201	9.8884	6.0907	9.2508	8.9321	8.7772	8.0522	10.3255	8.5348	7.5694	10.7162	10.8732	9.4179	10.4513	12.9802	8.6887	9.0476	7.3592	7.9134

TCGA-55-8087-01	462	0	4.7457	5.0794	9.2312	9.645	9.3701	6.4407	2.0501	4.2327	8.6844	8.2918	8.7125	7.0622	3.5632	9.4629	8.9191	9.0166	8.8055	4.9712	5.0942	9.6412	8.8639	8.3392	4.0624	9.1288	9.7162	9.5278	5.3378	8.4714	7.8053	8.4742	7.4469	9.51	8.5147	5.6298	8.2886	6.1387	8.9891	6.3135	8.3685	9.9531	9.8101	8.0204	7.4612
TCGA-55-8089-01	702	1	2.7701	6.2291	9.4139	8.416	9.6592	5.7045	2.1132	4.1402	8.3254	8.4829	8.1799	8.3122	5.5076	9.8533	8.1443	6.3145	9.4572	4.7654	6.0415	8.5707	9.398	8.6917	7.2553	8.3066	9.413	9.42	7.5745	7.9347	8.897	7.4497	7.8332	10.2445	8.5676	8.5692	10.9323	6.6131	8.9765	11.1797	9.9721	8.5911	9.0777	7.7823	7.6778
TCGA-55-8090-01	598	1	2.9076	5.2186	9.3432	9.0328	8.9762	5.4811	2.6473	3.8705	8.6396	8.2965	9.1717	6.6453	5.0125	9.2054	7.7081	9.8198	8.806	5.1697	5.7784	7.2285	8.4449	8.0541	6.1177	8.2576	9.8674	9.7669	4.7222	8.5089	7.8092	7.6604	7.117	9.8306	8.6026	13.3914	10.7431	6.6498	9.6671	10.2596	9.1608	10.4543	9.7195	7.7188	6.9355
TCGA-55-8091-01	600	0	3.2099	7.0598	8.9093	9.5604	9.276	6.7487	4.846	6.3043	8.6904	8.1604	8.5708	7.9663	4.4099	9.2873	7.6013	8.7712	9.0527	8.9126	6.0421	7.2813	8.8542	8.4873	5.7318	7.9089	8.7233	9.983	5.1654	7.9792	8.4858	7.772	6.5551	10.0549	9.7382	12.1153	10.9804	5.9044	8.7945	9.2118	10.0437	9.8919	10.221	7.6506	6.8877
TCGA-55-8092-01	154	1	3.2407	6.5542	9.2736	9.9564	9.497	7.6933	2.1887	7.7834	8.2084	8.2456	9.4737	7.6492	3.7502	8.9866	8.9649	8.3456	9.2715	3.5456	5.6675	5.3898	8.4131	8.4299	5.7651	8.7551	9.6105	9.9124	5.2785	8.8938	9.1206	8.9219	7.4653	10.3199	10.347	10.1786	8.6295	8.0965	10.9851	8.961	8.4055	8.4558	8.8925	7.352	8.0219
TCGA-55-8094-01	541	0	4.9168	4.8587	9.964	10.0321	9.5985	3.4767	5.8336	9.2919	7.863	8.6664	9.3694	3.4767	9.7002	9.1659	8.8731	8.2666	7.6444	2.9177	5.7561	7.3232	7.5829	7.6444	6.6257	9.7669	9.93	10.6748	6.0437	8.5006	9.6508	8.8768	7.4466	8.8713	5.6911	9.6238	11.3733	11.1636	9.8116	11.5882	9.0795	6.9236	9.2101	8.261	7.8335
TCGA-55-8096-01	719	1	3.5747	7.8527	10.302	9.9456	9.6085	7.114	5.3546	7.0026	8.9072	8.7061	8.0641	7.6481	6.8971	9.9383	9.0589	9.6477	10.6488	8.8708	7.789	9.4516	8.6057	8.6138	5.6246	8.5102	9.4535	10.2966	4.4525	9.3525	8.5065	8.404	7.0061	10.38	9.4573	11.8793	9.4694	12.2979	8.4702	9.3198	9.7183	9.6297	9.5753	7.5142	7.1075
TCGA-55-8097-01	476	0	4.9921	5.0971	8.7962	9.8191	9.0196	5.1468	3.869	5.0629	9.3277	7.9704	9.4203	6.9343	3.9829	9.4395	8.9675	8.3943	9.56	6.4898	5.531	8.6644	9.1402	8.3383	4.7772	8.9478	9.6475	9.7584	5.3727	8.8194	7.885	8.0683	6.896	9.7631	8.5129	10.7039	7.3031	6.6708	9.0881	6.6129	8.7621	8.6313	10.067	8.2726	7.0346
TCGA-55-8203-01	547	0	3.8013	7.0447	9.1363	9.0264	9.1311	6.3555	2.8209	4.893	8.449	8.4328	7.4234	7.5028	3.9967	8.8506	7.6319	8.9833	9.2208	4.6819	5.3155	6.9008	8.1665	8.5275	7.0839	9.1832	9.6019	9.842	6.561	8.6632	8.9245	8.656	6.9105	10.2108	7.86	9.7903	8.3058	9.6506	10.4746	10.153	9.655	8.2164	9.8797	7.4866	8.2006
TCGA-55-8204-01	515	0	1.6535	6.9711	8.6032	9.3984	9.9548	6.1445	2.7869	6.0773	8.5297	8.6424	7.544	7.6324	3.8492	8.9748	6.4927	8.0785	7.6633	7.5969	6.0773	7.9746	8.675	8.8778	7.5808	9.4948	9.4109	9.8166	5.9579	8.3516	8.2662	7.9021	8.186	12.3165	8.6957	11.984	11.8813	6.6551	9.5465	10.5818	9.4797	8.7596	8.5587	8.5065	8.0319
TCGA-55-8205-01	599	0	2.8737	5.9412	8.9355	9.4546	9.7591	5.7252	3.3473	5.8171	8.4269	8.8609	4.6649	8.6835	5.8959	9.8789	6.8799	7.0821	10.1407	6.9664	5.0971	7.0025	8.8291	9.2434	7.8601	9.0842	9.9322	10.1679	7.5054	8.7822	9.0091	7.3629	9.0562	10.3563	9.5642	9.199	11.1044	7.286	9.4376	11.57	10.0583	10.0829	9.2524	7.8854	6.4547
TCGA-55-8206-01	888	0	2.7249	3.5029	9.2208	9.4062	9.8521	6.2142	5.2999	6.4759	9.693	7.9639	8.3137	8.9342	5.5108	9.5777	8.4033	6.2594	11.4717	6.1315	4.9462	9.3248	7.9808	8.8927	4.9736	8.7829	8.7276	9.8736	5.7112	8.9105	7.9118	8.5617	7.2721	10.0766	10.176	8.2268	9.4933	6.6084	9.4268	7.4138	9.5104	8.6331	9.522	8.0676	8.4781
TCGA-55-8207-01	977	0	3.2806	7.5882	8.5015	10.4574	9.8461	7.4086	3.7775	7.4148	9.8087	8.742	8.6718	7.7158	5.236	9.3494	7.833	7.3679	9.4367	7.427	5.7558	7.3774	8.4289	9.307	4.5998	8.2817	9.5132	9.6771	5.7558	8.2145	8.0483	8.3427	9.5591	10.228	9.8675	10.8436	10.0039	6.3892	8.9879	7.1711	9.9312	9.9594	9.8472	7.1003	5.4786
TCGA-55-8208-01	674	0	1.8394	6.9927	8.5622	8.2019	10.104	6.1836	3.2097	4.0864	8.4682	8.5484	7.9574	8.5142	5.2134	10.0446	7.4496	8.0669	9.8791	7.4998	5.8121	7.7449	8.0189	8.6794	6.0327	8.637	10.268	9.578	5.6437	8.4218	8.4347	8.1917	7.6883	10.1848	9.7135	8.1452	9.4109	8.8727	9.222	10.4088	9.4958	7.8094	10.3851	8.1712	7.3252
TCGA-55-8299-01	469	1	0.5083	9.0912	8.9182	8.6104	9.339	7.4267	4.0184	8.5789	9.2253	8.1171	7.0575	7.7234	4.9924	9.4538	7.5938	8.2551	9.1916	6.283	5.7607	8.0335	8.2391	8.0543	6.2108	8.7669	9.657	9.7176	8.6089	8.5303	8.4576	7.7992	7.5906	9.6742	8.9042	11.4166	10.8498	7.7799	9.3437	10.4212	9.109	8.7229	9.7219	7.8317	6.6353
TCGA-55-8301-01	534	0	3.752	6.5398	8.6766	9.2948	11.106	6.6976	2.7206	5.8842	8.5328	8.3683	7.8416	7.9535	4.5985	9.592	7.9832	8.0827	9.7473	6.7619	5.542	7.951	5.7976	8.5614	7.1461	9.2978	10.1833	10.3684	6.7154	8.3999	8.5955	8.2531	7.552	10.6063	8.9189	8.3513	11.3336	8.3361	8.669	9.4608	9.2659	7.8308	9.7372	8.3341	7.0275
TCGA-55-8302-01	478	0	3.594	6.5294	9.9196	8.2865	9.8929	5.1875	2.7088	2.9346	7.6741	8.7432	7.5389	5.4658	3.9957	9.3083	8.4935	8.4553	8.7837	8.9202	6.6928	6.8042	8.5627	7.6544	7.0104	8.8492	9.9113	10.1937	4.696	8.3972	8.732	8.0048	7.2178	9.9046	7.8191	7.2016	11.1523	5.9778	8.221	10.5299	8.6784	10.2636	9.5069	7.3753	6.2221
TCGA-55-8505-01	440	0	3.0128	7.5976	9.7145	10.2026	10.051	6.8046	4.177	7.9808	8.4998	8.3526	9.1554	5.9608	3.2985	9.0805	6.9697	7.6662	8.6542	4.177	6.4238	7.6828	8.556	8.0689	6.289	9.1207	9.772	9.9065	6.7021	8.2613	7.5576	7.4882	7.0616	9.6588	8.8345	11.4384	10.9199	10.4972	9.7807	10.7702	8.983	9.2338	10.3525	8.4187	8.0721
TCGA-55-8506-01	11	0	5.2109	6.9248	8.3332	9.3498	9.5731	4.1612	1.4272	4.521	5.9013	8.8557	8.7313	6.1688	4.4108	8.8592	8.7893	6.8563	8.0746	1.4272	4.7795	5.0238	8.7465	7.7963	7.6134	7.6941	10.7749	10.4274	6.4731	8.9225	8.3532	7.7889	7.9288	9.2665	8.7274	13.3568	11.1755	9.4669	8.9309	9.0071	9.7748	9.1682	10.0385	7.6502	7.1184
TCGA-55-8507-01	418	0	6.322	7.8312	7.987	9.7277	10.348	5.6967	3.4959	9.7757	7.8543	8.0707	8.8787	5.8934	9.2276	9.4714	8.1797	8.4446	9.4758	8.6255	5.571	8.802	8.0114	8.5854	6.946	9.4142	9.6246	10.4068	5.0878	8.1332	8.2274	7.8516	7.5769	10.3601	8.4129	10.4616	10.4297	9.8846	9.0445	10.0417	9.5856	9.0282	9.5211	8.5915	9.0132
TCGA-55-8508-01	617	0	4.2237	7.7598	8.5976	9.1424	9.1035	6.1286	4.1537	9.7548	7.8957	8.4003	6.1102	7.4887	4.3542	8.9952	8.2023	9.6883	8.7446	9.617	6.5656	6.3942	7.8892	8.6075	5.4113	9.0754	9.5808	10.061	6.5521	8.3965	7.9728	9.215	7.0135	10.8232	9.402	10.4261	9.985	9.6316	9.9519	9.417	9.0125	8.3386	9.8394	7.9472	7.8344
TCGA-55-8510-01	539	0	2.8319	6.9472	9.2712	8.3567	10.354	5.6069	5.0633	4.6362	9.5231	8.8066	8.4505	7.4241	5.745	10.4354	8.3046	8.1639	10.8267	5.3058	5.5326	8.2934	8.1546	8.1202	6.1574	8.6351	9.9124	10.5779	5.4133	8.008	8.2849	8.7887	7.0836	9.8846	9.235	7.8098	10.2807	11.4523	8.2678	9.4686	9.3409	8.5968	10.0644	8.233	7.733
TCGA-55-8511-01	552	0	3.6325	6.6646	9.4675	8.9212	9.5239	6.3653	2.0901	5.022	8.223	7.8119	9.2428	7.4581	3.8653	9.6413	8.0283	7.6458	9.2312	4.4709	4.8425	5.8583	8.576	8.2349	6.3558	9.1995	9.8778	10.0562	5.6864	8.576	8.8635	8.2033	7.0664	10.4637	8.328	6.9058	8.2139	8.8852	8.0403	10.0961	7.6844	9.2075	9.4373	8.0785	6.8458
TCGA-55-8512-01	607	1	2.2453	3.8969	9.0632	9.3675	9.4448	6.7026	3.9477	5.1595	8.8602	7.8473	7.0841	5.2835	4.3768	9.3368	9.5442	6.603	8.971	9.6343	6.0015	8.8358	8.5138	7.6618	3.3343	9.0546	9.6707	9.4326	6.0489	8.4775	7.2093	10.015	5.965	9.1661	7.5673	9.5606	9.344	9.3152	8.8068	6.7173	8.8374	8.4065	9.4776	9.2772	10.0172
TCGA-55-8513-01	791	0	2.2355	5.0071	9.5584	8.6655	9.9489	6.1137	4.3944	5.7854	8.643	8.1741	7.4901	7.9969	4.1749	10.5595	9.1378	6.9472	10.7678	8.5925	5.4844	8.3809	8.8321	7.5254	3.412	8.2569	9.3079	9.5122	5.577	7.8516	7.111	8.7425	6.8852	9.5355	9.0293	10.3952	9.7631	7.3788	9.5369	7.8882	8.8896	8.2464	9.9726	8.8577	10.6348
TCGA-55-8514-01	520	0	4.9084	5.7173	8.4803	8.9687	9.0569	8.9481	3.8628	2.8976	6.3952	8.4619	9.0778	5.5879	4.4783	9.3755	8.6976	7.044	10.8453	4.3923	5.8684	6.0619	8.9779	7.9734	4.3473	9.5321	9.8501	10.3744	6.7389	9.5333	8.1574	8.9871	7.1592	8.9926	8.4433	10.831	6.9189	10.5317	8.6475	6.7902	9.2141	9.4951	10.58	8.5834	7.8817
TCGA-55-8614-01	536	0	4.7085	7.4156	8.4866	9.8681	9.6529	6.8506	1.529	9.0961	9.2133	8.6625	8.3817	6.7277	6.3257	9.9064	9.3493	8.4482	8.0154	8.7066	5.783	9.8177	7.8522	8.9051	7.3167	9.2795	10.3412	9.8826	5.7497	7.686	8.3291	8.5605	7.9618	9.2184	8.2891	14.1368	9.5765	7.5952	8.422	9.2366	9.4821	7.6092	9.6686	7.6683	6.6119
TCGA-55-8615-01	446	0	5.2236	7.9853	9.4356	9.9703	9.6995	5.4522	7.0328	6.6195	8.0241	7.6814	9.7244	4.9517	4.5521	9.1274	10.2599	9.2952	10.0232	2.8909	4.9266	8.0853	8.6948	8.0566	6.3904	9.316	9.8526	10.2339	5.9155	8.213	7.9607	8.5643	6.5797	10.0898	6.6429	10.6922	10.012	8.5684	8.441	9.3589	10.3748	9.1829	10.5579	8.279	7.6082
TCGA-55-8616-01	48	0	5.6394	6.136	8.9443	9.9589	9.0133	6.3274	1.6326	4.5915	7.3318	8.6614	8.6425	5.4443	2.0536	9.006	8.2281	5.0229	8.7146	6.4538	4.9513	8.4173	8.1014	8.0249	5.9401	9.2607	9.2508	9.9994	5.8771	8.497	8.5137	8.9762	7.2702	10.0503	7.5544	10.5562	9.5717	9.7156	9.0366	7.9594	9.2693	8.9611	9.965	8.4566	7.007
TCGA-55-8619-01	416	0	2.6419	5.3227	9.2628	8.8973	9.7792	5.3844	4.8265	5.1677	9.3975	8.2051	7.518	8.3818	4.7965	10.346	8.8471	6.7177	10.5503	7.7108	5.7234	8.5255	8.6912	7.8375	4.0639	8.2165	9.3695	9.5861	4.9403	8.033	7.666	8.3148	6.5395	9.2309	9.6925	8.9645	9.5349	7.5226	9.2858	8.2583	8.9994	9.0885	10.19	8.4043	8.892
TCGA-55-8620-01	375	1	4.9709	5.7385	9.1339	7.9124	9.7363	5.0508	1.4782	4.4487	9.8374	9.2257	3.69	6.9077	7.2272	8.9394	9.6034	8.0156	8.6348	6.9638	6.749	5.2445	8.0419	9.7622	8.2137	9.1108	10.3666	10.4774	7.4201	9.7533	9.2723	8.0931	8.2424	10.7023	9.044	5.4352	8.3725	10.0749	10.2345	10.7339	8.7309	6.6153	9.5252	8.6239	11.1449
TCGA-55-8621-01	515	0	3.4118	7.1211	9.1189	8.9865	10.856	5.4895	5.8391	7.2332	9.8731	8.0878	8.1103	8.1998	5.6209	10.5449	8.439	8.2988	10.1092	8.0239	5.7841	8.7286	8.4829	8.2666	4.7681	8.3659	9.1244	9.619	6.258	8.2489	7.9288	8.579	7.4163	9.6635	9.3859	8.6672	10.5706	8.7979	8.9432	8.8953	9.2829	8.3256	10.0514	8.2988	8.0849
TCGA-55-A48X-01	689	0	3.1408	7.1866	9.3477	9.6246	9.1484	5.9102	5.3942	6.654	8.6514	8.3445	8.9127	8.1472	3.7769	9.2175	9.1828	8.2129	9.4859	8.2317	6.0338	7.6678	8.1043	8.3314	5.3603	8.7938	9.5634	9.7087	6.0657	7.7322	7.816	8.8936	6.6954	9.7502	8.7338	11.8767	9.5342	10.4819	8.5912	9.2767	8.4349	9.6002	10.502	8.0144	7.7021
TCGA-55-A48Y-01	630	0	1.8584	8.4217	9.6091	9.7451	10.183	7.736	3.8207	11.173	8.9874	8.3399	9.0652	6.6094	2.8684	8.7936	6.2598	7.913	7.2702	5.6989	4.3894	7.5942	8.2753	8.3994	5.9646	9.0294	9.8604	9.3271	5.2188	8.711	9.0177	8.1096	7.4586	11.1238	8.3994	12.7836	10.9585	9.9258	10.6564	9.5375	10.1597	9.0001	8.9867	6.5621	6.6094
TCGA-55-A48Z-01	651	0	3.8535	7.8378	8.2828	9.5381	9.7717	5.3535	4.2676	4.4048	7.7749	8.4267	9.4732	6.2922	4.8273	9.3533	8.6961	9.7891	8.9666	3.037	6.2833	8.5557	8.827	9.0964	7.4783	8.4704	9.6152	10.5376	5.8744	7.3397	8.3005	8.2783	7.2005	10.74	8.8024	9.2557	10.0989	9.2849	9.258	9.5921	9.3394	8.8194	9.961	7.7853	7.3525
TCGA-55-A490-01	99	1	3.2272	10.5664	9.0047	11.3983	8.7267	5.086	3.679	5.7716	6.5084	9.3968	9.0456	6.1768	5.8101	9.8548	8.541	9.2806	8.7858	1.308	5.9544	6.4608	8.558	7.2988	7.8503	8.7761	10.3851	11.693	4.9548	9.586	7.7217	5.3867	6.6281	10.6215	9.0866	12.5516	9.1215	8.8942	8.2105	9.8371	11.0388	9.4935	9.6225	6.8473	5.872
TCGA-55-A491-01	626	0	5.3091	6.5813	8.6174	9.4343	10.041	6.1473	1.3719	4.664	7.2919	8.1562	9.4938	6.863	4.9623	10.3746	8.7999	7.3673	10.097	7.6594	4.9866	9.0115	8.7115	8.1669	6.2002	8.852	10.3872	10.0935	4.1651	8.1509	7.733	7.4394	7.6365	9.9473	9.5519	10.0738	9.8034	10.828	8.3085	10.6157	8.7687	9.912	10.6827	7.4526	6.9272
TCGA-55-A492-01	596	0	1.879	7.3654	9.1932	11.4229	10.19	6.3074	2.5412	1.6519	7.4737	8.5357	8.9571	3.0872	2.9933	9.4211	9.7918	6.1959	7.3178	7.0173	5.2865	7.4781	8.8771	7.186	4.7679	9.2335	10.67	11.312	5.7698	8.9288	6.5494	9.6139	6.2878	9.4222	7.1042	6.1747	10.4813	10.4058	8.9398	6.9376	8.8321	6.8126	10.7579	9.4785	6.6528
TCGA-55-A493-01	28	0	3.7136	5.9334	9.1551	8.9082	9.6375	6.0955	2.8683	5.4482	7.3984	8.9227	9.1166	7.3263	7.6698	9.7859	8.3606	8.3457	9.6296	7.8594	5.9103	5.5562	7.4909	8.6559	7.7369	8.6119	10.4577	10.4945	7.4354	8.9916	9.6207	7.1054	7.8135	10.1178	9.1241	6.6972	10.597	10.2586	8.2593	11.4432	9.1563	9.863	9.0336	7.8951	7.964
TCGA-55-A494-01	481	0	5.8297	4.5885	9.3855	9.4491	9.4969	1.8721	2.5553	4.4195	7.0991	7.7285	8.5569	3.7929	9.6043	9.068	9.4564	9.3418	10.3637	8.5671	5.5582	7.3197	8.3232	8.1381	6.1867	9.0391	10.045	10.8146	4.1934	7.5072	9.3932	8.1082	7.1409	9.782	5.7606	13.9653	7.4277	8.7129	8.7175	10.8032	9.1218	9.3119	9.9125	7.651	6.9262
TCGA-55-A4DF-01	614	1	4.3517	8.9799	7.8152	7.6656	9.9626	3.5402	0	3.6505	8.0614	7.6819	5.4131	7.1309	7.1069	9.5542	7.3755	10.4744	9.9785	9.6936	5.8661	9.2057	6.7018	9.7505	9.1795	8.6388	10.7875	9.4282	9.1795	8.4872	9.9253	7.612	8.1911	11.5698	8.4399	8.6833	10.7132	9.6839	9.38	10.8764	9.7659	8.1911	9.5328	7.7298	10.2264
TCGA-55-A4DG-01	608	0	5.599	8.3365	9.2062	10.1248	10.134	5.9947	2.2302	4.8557	9.0188	8.2163	7.6427	7.6541	3.7717	9.3635	9.1644	8.7651	9.3989	5.6754	5.9337	6.1634	7.4164	7.286	5.2845	8.7297	9.4378	9.0783	6.2459	8.0338	8.6637	9.4649	7.0708	9.3542	8.308	11.4888	9.0527	10.4274	9.0076	6.811	9.3367	7.4341	9.6722	7.9986	8.2517
TCGA-55-A57B-01	546	0	2.2865	7.4323	9.7907	9.3336	9.6559	6.4667	7.5725	6.0571	8.9757	8.5029	8.1683	7.2235	5.0482	9.4014	9.4953	6.7097	11.1354	6.3839	6.0184	9.4356	8.848	7.5591	5.1084	8.5566	9.6349	8.884	5.2081	8.1576	7.8773	7.9512	7.0842	9.3431	9.2051	9.4253	10.3703	6.7854	8.2474	8.7151	9.4029	9.4451	10.0191	8.0976	6.9175
TCGA-62-8394-01	139	1	4.2703	7.497	8.6933	9.406	9.8065	4.1058	4.3312	4.2068	7.771	8.5896	9.0943	5.5467	6.6008	9.1618	8.1013	7.9901	7.91	3.9973	4.9319	9.0998	8.43	10.2485	7.8696	8.6311	10.7472	10.4485	4.6744	8.5943	8.1486	8.1882	8.1882	11.755	7.7154	9.674	10.6874	10.8577	8.9778	10.9966	9.5901	8.6889	10.1132	8.4387	7.7851
TCGA-62-8395-01	1216	0	3.7313	7.3781	10.1098	10.0418	9.1766	7.6193	3.471	8.8268	7.9343	8.104	9.6528	7.0931	3.471	9.2981	9.0735	7.59	8.3965	7.0555	7.2879	9.857	9.5934	7.9991	3.4127	8.6741	8.975	9.6893	3.9921	8.4152	7.9916	7.9359	6.1971	9.3485	8.6043	8.2274	10.6745	6.2312	9.8583	7.4844	10.7203	7.8992	10.5382	7.4487	10.0334
TCGA-62-8397-01	1289	0	2.7739	4.4221	9.5866	10.7128	8.9581	5.2287	1.2986	3.8216	6.8254	7.8002	9.5562	6.9668	4.0924	9.21	8.9195	6.9835	10.3575	4.0924	5.1714	6.6686	8.839	7.5868	1.9707	9.2963	9.4991	10.4492	4.8448	8.9064	7.5647	7.6615	5.2838	9.174	9.3294	6.2093	9.7545	10.7585	9.0081	8.1284	9.4667	9.6298	10.9707	8.3511	5.1714
TCGA-62-8398-01	444	1	1.8589	6.3522	10.1691	10.5098	9.3096	7.2715	6.3588	6.4668	8.2823	7.5825	8.9576	7.4463	3.0886	8.8828	9.1202	6.893	10.3592	7.4555	6.2843	6.9201	8.8585	8.6053	6.479	8.9445	9.5123	10.0719	5.831	9.237	8.3455	7.8118	7.3828	10.0434	9.922	9.5343	10.415	9.98	9.6049	10.9817	9.0867	9.5351	8.4881	7.7826	8.2633
TCGA-62-8399-01	2696	0	4.045	7.6462	9.3955	10.2553	8.3797	6.2672	4.2596	9.5671	8.3908	8.4233	8.8209	6.52	3.333	8.6205	8.758	9.7153	8.3365	5.4	5.1769	7.6075	7.351	8.679	5.681	9.8022	9.1941	9.6852	7.2033	7.8252	8.1435	8.7655	7.6489	10.4473	7.8467	12.9244	10.1128	10.8081	9.6256	9.8102	8.7402	6.9881	9.1969	7.6131	8.0228
TCGA-62-8402-01	1498	1	2.6824	2.7891	9.1336	8.9315	10.186	3.4401	5.3012	3.151	8.5136	8.2962	8.4866	6.9901	6.8181	9.6591	9.9913	9.4534	10.0108	8.1858	5.8617	6.9214	9.4008	9.1894	6.3798	8.5656	9.6476	9.9242	6.3798	8.2734	8.869	7.7067	6.3883	9.3924	7.332	8.6334	7.7437	9.9667	8.5879	11.4761	8.4029	9.8351	9.9524	7.1387	9.2027
TCGA-62-A46O-01	1454	1	2.9428	6.3545	10.3668	10.1014	9.5564	2.4487	3.1381	6.5814	8.1042	9.566	10.017	4.3969	1.6914	7.6913	6.9693	8.4487	5.0827	3.9007	6.6944	5.3226	7.6991	8.6897	7.7671	8.9719	9.786	10.2441	6.3247	8.5806	9.7805	7.9043	7.9375	10.6258	6.2943	11.6571	11.2643	10.0193	10.3001	12.1299	11.0424	7.7999	10.4899	7.6874	5.6928
TCGA-62-A46P-01	594	1	3.9341	4.1327	9.4717	8.9135	9.0322	6.0204	2.2508	6.9578	6.877	8.5754	10.1377	4.0695	3.4295	9.4748	9.2474	7.78	9.4641	7.1069	5.6618	6.1173	8.012	7.2491	4.7717	9.1419	11.3352	9.7001	5.7849	8.2586	7.6564	8.5114	5.9693	9.9221	7.075	7.2844	8.5582	5.8044	10.5582	7.6293	9.6348	6.9044	9.6279	8.2082	10.3529
TCGA-62-A46R-01	1725	1	4.045	4.808	9.4425	9.1453	9.3865	5.3405	1.8078	3.5855	7.7353	8.5818	9.6915	6.7622	5.6005	9.5291	8.2651	8.4885	9.3203	5.4925	6.4764	8.5968	8.673	8.1755	6.1091	8.0091	9.594	9.8056	5.8335	7.9921	9.5724	8.4373	10.1668	10.6061	9.0894	6.7488	10.1189	10.5025	9.0105	9.6535	9.9736	9.6061	9.5514	8.2294	6.3493
TCGA-62-A46S-01	1653	1	3.4213	4.7808	9.2397	10.7271	9.1291	6.1891	6.1083	9.3066	8.3775	7.9505	8.7924	6.557	8.1764	9.1684	8.945	10.0362	10.2226	8.0257	4.7808	7.7638	7.9218	7.5114	4.9136	8.945	10.262	9.939	5.5655	8.5455	8.3149	8.1983	6.6676	10.6716	8.5974	9.429	10.5167	9.5341	11.3876	8.0643	10.2316	8.5658	8.8652	7.4544	7.6978
TCGA-62-A46U-01	2067	0	2.5686	7.0411	9.0598	9.8698	10.788	1.7933	4.1244	3.5468	5.9309	8.7094	8.3105	9.1471	7.8032	9.6267	7.1661	7.2697	10.7842	4.5357	5.788	5.7333	7.8451	9.3083	6.4549	8.7009	9.5442	9.671	5.688	8.2573	8.2974	7.4902	7.9106	10.723	9.3139	7.2148	10.5668	4.8554	9.843	10.8509	10.1635	9.692	9.2255	7.6374	6.172
TCGA-62-A46V-01	2199	0	3.7449	7.0117	9.0687	9.9507	9.3933	6.1693	2.3133	4.7176	7.9018	8.3965	8.9126	5.544	5.3818	9.5108	9.319	7.1742	8.6076	2.9906	7.6003	7.2931	8.6914	7.9694	6.5362	9.4415	10.4097	9.7383	5.1393	8.1339	7.8472	8.465	7.6077	9.7804	9.1004	7.1692	11.6903	5.2374	8.4548	9.8284	10.2712	8.54	9.9288	7.2036	5.3645
TCGA-62-A46Y-01	414	1	1.0018	4.1327	9.4722	10.2258	9.6411	5.3958	3.2512	4.6473	8.2844	8.6183	9.7127	7.0425	5.2889	9.7923	7.8553	5.091	10.2486	6.7585	5.9104	7.339	9.8206	8.5543	5.9461	9.2432	9.8404	10.4753	6.1835	8.2774	8.7964	8.5389	7.4172	10.2008	9.563	10.0232	10.4738	5.3786	9.3289	10.1495	9.3401	9.099	9.9015	8.5658	7.5153
TCGA-62-A470-01	1194	1	1.7921	8.6483	8.9544	9.3821	9.1641	6.2267	1.7921	6.2028	8.0474	7.4685	4.6088	5.0981	6.9983	9.3498	9.5668	7.8225	10.4022	6.1537	5.5412	9.7839	7.3859	7.7082	6.0503	9.4241	9.6287	10.1674	6.7403	8.617	8.7995	8.2241	7.4884	9.7819	7.2171	10.4487	10.055	9.8295	10.6861	9.0514	10.2959	7.7251	9.5644	7.4483	8.5475
TCGA-62-A471-01	1246	0	3.8886	5.7326	9.5156	11.735	8.1334	7.6251	1.8309	7.9426	9.561	8.6289	8.4272	4.0753	2.4864	7.8687	8.6963	8.0689	7.8433	5.098	5.9865	5.6321	8.5326	8.5105	6.625	9.6503	10.5965	10.4358	6.327	8.4778	9.0454	7.7227	7.4077	9.917	7.9366	11.3317	11.348	10.2736	10.1977	10.9288	8.4694	7.8529	8.1723	8.9108	6.3085
TCGA-62-A472-01	910	0	1.9619	4.6514	8.9378	10.8649	10.014	4.1996	3.1247	3.4131	7.7041	8.1923	8.862	6.2716	3.9522	9.0365	7.6703	6.9152	9.3044	3.1247	6.7696	6.235	8.35	8.2934	7.0579	8.3198	10.4348	9.8423	7.2161	8.3962	9.1215	7.7566	6.6909	10.6143	8.7239	6.9828	12.4874	4.6514	9.8225	10.173	9.9504	9.0445	8.6804	7.333	6.4573
TCGA-64-1676-01	1728	0	0.7595	6.5199	9.6424	10.1239	10.183	6.1493	3.5952	1.6223	7.439	9.612	6.3708	6.2965	5.3147	10.1248	6.6249	4.5768	8.4406	2.8552	4.0217	3.323	7.9562	6.7037	6.6045	9.6323	11.6815	11.4577	6.1352	9.4642	9.357	9.3887	7.992	11.1582	8.9892	13.0776	9.832	10.3658	9.9478	9.9295	9.6133	6.2965	8.9289	8.2567	5.5028
TCGA-64-1677-01	628	1	2.6939	6.4135	8.2942	8.5087	10.533	5.1704	1.0663	1.0663	7.5384	9.2143	8.2007	5.7121	6.2979	8.6763	7.4327	9.5992	7.5553	3.8206	4.4454	5.5863	8.8159	8.1405	8.1349	10.7753	11.7722	11.3146	7.106	9.006	9.101	8.2273	10.8249	10.6384	6.3949	9.5154	10.8162	7.1622	9.5808	11.0508	9.4402	8.6608	8.9998	8.1461	6.194
TCGA-64-1678-01	1189	0	4.2322	7.86	8.5093	7.066	11.521	6.872	2.9176	7.1445	6.9506	9.2207	8.2677	5.3001	4.4332	7.9226	9.6654	9.5367	4.9958	4.4332	4.2322	6.0361	7.5854	8.1394	8.7226	10.461	11.3122	11.4329	8.5167	10.585	9.6737	8.4253	8.5781	10.3983	5.907	12.0172	9.74	9.1003	11.6389	11.831	10.3276	6.8487	9.4735	8.0038	5.3994
TCGA-64-1679-01	2488	0	4.7141	7.7705	9.0404	9.2076	9.0827	6.0696	2.4203	6.4218	8.2207	8.5091	8.4214	8.9037	6.2996	10.039	5.55	8.8755	8.832	3.4028	5.3592	8.3031	8.3755	8.2991	6.6474	8.4214	9.4248	9.9406	5.1745	8.8755	8.5917	6.0634	7.8177	9.8049	10.096	11.1853	10.4078	10.9482	9.3517	9.9958	9.7011	10.2736	9.6691	7.3195	6.2943
TCGA-64-1680-01	1126	0	0	3.7767	9.3536	9.6652	8.5724	6.8499	2.4553	3.3174	7.824	8.773	9.6193	5.4463	3.519	8.7829	7.2405	7.0745	5.8899	5.114	5.175	6.3124	9.5818	8.6709	2.9498	9.327	10.5617	10.6429	5.7764	9.0563	9.1061	8.6971	7.383	10.4628	7.3895	12.4812	8.6496	4.7231	9.7032	10.06	8.6307	10.6945	9.3733	8.6709	6.5611
TCGA-64-1681-01	1167	1	3.0407	6.8299	9.5754	9.232	10.202	5.4716	4.549	5.9477	8.7006	8.4563	8.342	6.6955	5.9975	9.5498	7.182	7.5283	9.0881	6.8161	4.2805	9.1296	9.8349	8.3372	5.5064	8.9536	9.3422	10.5201	5.0517	9.2031	7.4678	8.5827	7.1928	10.8026	8.3832	10.5437	9.1664	9.6395	9.1052	8.8227	9.4066	8.0705	9.5401	8.4458	8.7555
TCGA-64-5774-01	2676	0	3.0035	7.4786	8.8603	9.4408	10.525	5.9503	7.5085	7.9655	8.4098	9.0607	8.1692	6.2366	5.739	8.3965	9.5761	10.2648	8.5841	8.297	4.5231	6.1054	8.4224	8.9421	7.5879	9.0607	9.8463	10.2191	6.7318	8.1069	9.5109	7.0032	7.8532	10.551	8.2356	12.1598	10.0052	8.136	10.202	11.7262	10.1255	8.6974	9.8953	7.8849	6.8419
TCGA-64-5775-01	62	1	2.496	8.3907	7.5584	9.4635	9.5464	4.3893	1.9202	2.61	7.1269	9.2514	4.6516	5.9277	4.6247	9.8898	5.9277	8.9629	7.0632	7.8559	5.689	9.2807	7.7291	8.661	7.1554	8.9467	9.2425	9.5065	8.9548	8.6173	9.9452	6.8242	8.8397	10.3272	8.7161	8.9921	13.177	8.3767	8.9123	11.0119	9.8417	3.8539	9.9828	6.2159	2.9064
TCGA-64-5778-01	1305	0	2.8535	6.5845	9.1102	10.2292	9.512	5.3002	1.621	2.5035	7.6496	9.7661	9.2159	6.3476	4.4435	9.0408	7.1193	6.5529	8.6158	4.6364	4.9345	6.0301	9.5859	8.448	5.7531	9.7713	11.097	11.2441	5.5917	9.325	8.6737	9.5504	8.6719	11.2716	6.8345	7.1566	8.9408	8.7865	10.4919	10.289	9.263	8.1141	9.305	8.5573	6.2818
TCGA-64-5779-01	864	0	7.4127	8.4764	8.4797	9.0105	9.0036	7.8412	3.6388	10.782	9.6577	7.9262	8.412	9.5055	8.5316	8.8015	9.3387	9.2308	9.5345	5.2721	5.4979	8.2546	7.836	9.0444	6.8729	9.0555	9.8659	9.9601	5.9245	7.6069	8.9874	7.5886	7.8463	11.0865	9.3405	10.0419	11.2127	8.4597	9.466	9.9801	9.4168	7.7344	9.7798	7.2899	5.6701
TCGA-64-5781-01	1559	0	2.7629	9.3035	9.3945	9.2813	8.6194	6.3693	3.4123	8.1818	7.3258	9.5548	8.6218	5.5441	4.0386	8.8113	6.1517	7.7492	7.2491	3.4123	5.3301	5.9262	8.6475	7.5207	6.5285	9.1072	11.1557	11.308	6.4026	9.0593	8.8154	8.3925	7.5655	10.4228	10.558	11.0681	10.5771	6.4351	10.6202	10.6482	9.1794	8.2505	9.3093	8.062	4.5551
TCGA-64-5815-01	866	0	1.747	8.6298	8.8546	10.5354	10.101	7.4544	3.2692	6.6091	8.5863	8.2452	8.5291	7.2831	4.708	10.2162	6.4367	8.5833	8.8716	4.91	4.8718	7.4284	7.8915	7.8577	5.2745	7.943	9.8469	9.9763	4.7923	7.943	7.7773	7.4673	7.2313	10.071	9.4237	9.8941	11.857	8.0236	9.4819	9.8773	9.7775	9.913	10.1519	7.319	5.0534
TCGA-67-3770-01	610	0	5.0007	5.305	10.641	9.9591	10.51	9.2281	5.8892	4.0034	7.9344	9.368	8.0036	6.6393	6.1596	9.3221	7.7564	8.4186	8.6077	2.922	4.6145	7.2948	10.8264	6.4193	5.2703	9.2943	9.8145	10.4955	8.2639	9.3243	8.7499	8.5618	8.1869	10.3782	8.0757	6.7315	10.5517	11.3705	9.9288	8.933	9.8993	9.8039	10.5997	8.5107	5.2347
TCGA-67-3771-01	610	0	3.8104	7.6035	7.9499	8.3691	10.04	7.3097	4.6119	5.3015	8.3164	8.1217	4.7229	7.7849	5.5624	9.2155	8.5303	10.1211	8.6568	7.6511	5.416	8.1149	7.9074	7.9574	7.0295	9.1541	10.5306	9.97	6.1304	8.3022	8.2538	8.1351	7.6464	9.7907	8.9181	8.8687	10.2633	9.9483	9.9559	9.3454	9.6457	9.4021	9.4779	7.3791	9.8782
TCGA-67-3772-01	573	0	5.2474	7.0038	10.5802	8.4012	9.8988	9.3626	4.5814	6.4607	8.9802	9.4288	7.8626	7.8713	6.0358	9.575	6.1256	8.014	8.6468	7.9499	4.2319	8.2998	9.582	8.1075	6.5785	9.0072	9.3575	10.4731	5.1365	10.1667	8.5988	8.479	7.8124	9.6382	8.4171	11.0221	8.9714	10.403	10.2506	8.8493	9.0474	10.5162	10.3351	8.6619	8.2228
TCGA-67-3773-01	427	0	1.0198	5.9215	8.979	9.3478	10.947	7.7117	3.2051	4.6818	8.2712	8.9312	8.8974	7.2191	4.9451	9.3591	8.1531	5.3239	8.3248	4.2072	5.2479	5.7392	9.1635	7.3968	4.7929	9.6797	9.8771	10.5679	5.2864	8.9907	7.229	7.3339	7.6613	9.3681	9.2754	9.0338	9.3086	6.767	10.021	8.5978	9.261	9.6131	9.6923	7.3339	5.8717
TCGA-67-3774-01	385	0	5.2251	5.1493	9.1322	10.1786	9.2105	6.7244	3.5677	12.096	8.566	7.4698	8.2222	7.5799	4.0293	9.3832	7.9699	8.0205	10.4236	7.1382	5.6118	7.9663	8.8239	8.2375	5.3433	9.0159	9.6088	9.2382	5.4735	8.8381	8.0659	8.2466	6.698	10.757	8.6189	10.173	8.8158	9.428	9.573	8.789	9.9638	10.4034	10.0145	8.2436	9.8851
TCGA-67-4679-01	448	0	2.8702	4.7141	9.0264	10.0719	9.9871	6.7512	4.5821	6.2292	8.8015	8.4578	8.6452	7.5551	4.398	8.968	9.6912	7.1744	9.0824	6.8478	5.5692	8.6657	7.8234	8.1227	4.4745	8.851	9.8094	9.2738	5.0502	8.6327	7.6238	8.3273	6.8973	9.5592	10.507	10.3323	9.2372	6.463	9.1841	7.7976	8.8724	9.7346	9.8296	7.4453	9.3645
TCGA-67-6215-01	174	0	1.8508	3.9214	9.2312	11.1298	9.1968	2.9455	2.7988	3.5608	6.8193	8.3931	8.9873	6.7282	4.4983	9.5158	7.6021	4.7386	9.0963	4.0241	5.6377	8.4449	8.7682	8.7483	5.9817	9.1303	9.8416	9.769	5.6697	9.0686	8.3673	7.9964	7.5312	10.1092	8.5774	6.7232	9.7976	5.1702	8.8438	9.9074	9.0625	10.7042	9.5888	8.5914	7.862
TCGA-67-6216-01	141	0	2.3577	3.9207	8.9748	10.1364	9.9032	5.9054	1.1235	3.8038	7.6575	8.3528	7.7279	7.3231	4.813	9.248	8.1097	5.6415	10.2524	6.2225	5.3394	6.774	9.104	8.6691	5.421	8.9866	10.4832	9.859	5.0876	8.376	8.2021	7.8979	7.6066	9.7906	9.4978	10.5957	10.1985	5.607	9.5656	9.7388	9.1301	10.112	9.9818	8.2364	6.0789
TCGA-67-6217-01	422	0	3.8724	7.5152	10.2419	10.4793	8.5943	6.0894	4.7326	5.7363	8.3568	8.6006	6.6096	8.5068	4.3854	9.2539	7.3691	9.3513	10.2378	5.7516	5.18	8.1875	8.4136	9.0539	5.3097	9.0227	9.2144	9.1084	5.91	8.768	7.9528	8.9811	8.0704	9.1723	8.4422	7.9125	9.4211	10.9593	8.8378	6.9912	8.9827	8.868	10.1285	7.979	8.3597

TCGA-69-7760-01	202	0	4.5148	6.7689	8.9146	7.8391	9.8115	4.7673	3.9417	3.9992	7.9814	7.8113	5.9735	4.3029	4.2565	9.9636	6.8954	9.6759	5.8225	10.4345	5.4212	8.0476	9.1358	9.3933	5.4	9.4562	10.459	10.4312	6.3278	9.3484	9.0068	8.2506	7.7073	11.1726	7.0117	16.1719	10.2344	10.5229	9.4879	9.6459	8.8594	8.6353	9.2906	7.2903	5.5803
TCGA-69-7761-01	186	0	1.8869	5.7805	9.5383	8.7788	9.0743	6.6088	2.4107	5.3742	10.6027	7.7996	5.8224	9.5806	6.0857	9.1787	7.0341	7.8612	9.9374	7.0518	4.9892	9.6038	7.9234	8.8207	5.7662	8.2847	8.8016	9.2302	5.3928	8.257	8.7519	7.3382	7.0635	10.385	8.8764	9.7736	8.0375	7.6094	9.4926	10.0018	10.1093	9.8096	9.1557	8.2078	8.6759
TCGA-69-7763-01	690	0	3.2134	6.8831	8.6629	9.3832	9.0256	7.0404	4.5154	9.1688	9.2785	8.0478	8.7347	7.0142	4.1335	9.0386	7.0142	7.2732	7.8806	6.6225	5.5578	7.9751	7.8514	8.0284	4.7234	9.1894	9.4235	9.0675	4.5154	8.0251	7.3743	8.0414	7.2842	9.2183	7.4396	9.4938	9.5114	7.5755	9.7618	8.2713	10.6465	9.6367	9.4615	7.2732	5.7444
TCGA-69-7764-01	414	0	1.5799	5.9849	9.7014	10.0317	9.8162	5.9389	4.5574	5.2779	8.4085	8.1639	9.4442	6.8295	3.2661	8.5431	8.3484	8.8136	7.9524	6.5002	5.4188	9.3911	8.1505	8.2576	6.2962	8.9801	9.7578	10.2414	4.4738	8.1061	7.9753	8.3514	7.6577	9.6259	6.9886	7.8972	8.5735	10.9164	9.131	9.5257	10.7827	8.9162	10.2579	7.6718	6.5212
TCGA-69-7765-01	165	0	2.8564	8.4891	8.7726	9.1492	8.8251	7.079	4.1582	7.3403	8.7301	8.3525	8.0433	8.0569	4.4329	9.3068	6.4245	8.9669	8.2819	5.4618	5.9141	7.8023	8.1002	8.3302	3.8185	8.9794	9.2824	9.5545	4.8312	8.1002	9.6551	7.4679	7.0038	10.0392	9.1268	14.6678	11.0614	6.2813	9.265	9.4242	9.4845	9.8924	9.6936	7.7573	4.5529
TCGA-69-7973-01	230	0	4.1722	7.5864	8.7793	8.8751	11.938	7.0224	6.8133	8.7596	8.3484	8.511	8.4611	7.177	5.5364	8.4291	8.7432	9.3308	9.7814	5.2731	5.0099	7.4268	8.7767	9.3606	8.1591	8.8169	9.8321	9.4453	7.7578	9.0018	8.7345	8.2645	7.8734	10.1917	7.1511	12.7056	7.9701	8.5292	9.1054	10.7372	10.8569	8.1047	9.9535	8.0326	8.285
TCGA-69-7974-01	184	0	2.7191	6.3903	9.0452	8.6706	9.3139	5.169	3.9991	6.3124	8.754	7.8924	8.8668	8.3555	5.4661	9.852	7.2898	6.5884	9.6027	5.7988	5.5226	7.712	8.1633	8.6581	6.9144	7.9101	9.1697	9.5551	6.5436	7.6937	8.9133	7.3994	6.8715	9.7959	9.8598	9.0847	11.7188	6.5161	8.8037	10.9523	9.8149	10.9695	9.163	7.3087	7.3033
TCGA-69-7978-01	134	0	3.4708	7.7851	8.6358	8.3612	9.436	6.399	3.5965	5.9112	9.0527	9.0822	8.4205	8.8188	6.1209	9.9922	7.0677	10.177	9.5184	7.5439	6.2666	7.2572	7.9802	8.7325	5.6562	7.7562	9.3018	10.0252	7.1857	7.3344	9.0885	7.2604	7.729	10.358	9.7988	8.4458	11.7036	9.128	9.9993	9.1461	9.8987	10.0791	9.6808	7.5671	6.7036
TCGA-69-7979-01	408	0	3.2339	6.8878	9.1218	9.465	9.527	4.058	1.1109	5.8096	7.6311	7.9745	8.585	5.6605	5.0774	8.7917	9.0328	7.4863	7.477	4.1074	5.1499	7.131	6.4206	7.5435	7.8458	9.1807	10.9604	10.5696	5.1732	8.5055	10.8487	8.8608	8.3906	8.4951	8.0987	12.3512	11.0637	6.6954	9.0207	9.2372	8.6088	6.8843	9.958	6.9193	5.4276
TCGA-69-7980-01	362	0	3.5124	7.488	9.5282	10.3206	8.8474	6.146	3.2591	4.4678	8.6336	7.6061	7.9637	6.8444	4.9546	10.2319	8.0446	9.1281	9.0872	4.4678	5.6259	7.045	8.6581	8.7149	6.1584	8.743	9.5198	9.5495	4.4477	8.3857	8.0955	8.3112	8.1354	9.826	8.4465	11.6518	9.051	10.984	9.3862	10.2182	9.239	9.3742	10.3008	7.5459	5.8645
TCGA-69-8253-01	426	0	1.4406	6.7182	9.2098	10.4885	8.5419	4.9935	4.431	5.648	7.6714	8.3226	9.7774	6.1785	2.9206	8.5095	7.5557	9.4574	7.6157	9.9573	5.3474	6.8419	8.3594	7.5988	4.4118	9.0092	10.0316	9.8963	6.0413	8.4867	8.5015	8.8115	6.1145	9.0741	6.8983	11.1351	9.4533	5.3676	10.0371	7.336	8.9654	9.9689	9.2943	8.1577	6.0096
TCGA-69-8254-01	409	0	1.8902	4.4045	9.6345	7.3415	9.5776	5.3441	2.7878	4.5928	8.2869	7.6568	8.8321	7.9913	4.3533	8.7561	7.9431	6.9858	8.592	9.7878	5.3087	9.2311	8.1091	8.1207	5.6215	8.6102	9.5771	9.694	3.6514	8.415	7.6568	8.1053	6.6177	10.1055	8.5174	13.6299	9.8818	6.7976	9.9376	8.4701	9.3287	9.9659	10.2399	7.6462	11.0401
TCGA-69-8255-01	129	0	3.6924	3.6924	9.0415	11.1998	10.764	4.0714	2.1139	5.9022	8.6391	7.9917	8.2196	7.0222	12.2069	8.3356	8.6405	6.6322	9.0988	3.0378	6.0485	7.0375	8.2007	9.0116	7.9159	10.3618	10.4416	10.2217	8.176	9.2118	9.7585	7.7859	8.2102	9.9357	6.6078	6.0241	11.8429	10.9705	9.0767	11.5661	9.4218	4.4828	8.3068	8.3207	6.0902
TCGA-69-8453-01	813	0	2.5542	4.5192	9.562	8.9201	10.505	5.8943	3.6663	6.698	8.8609	8.2508	7.6601	8.2415	5.7581	10.9943	8.3767	6.8459	10.4331	7.8549	5.5931	8.3935	8.0638	8.1554	5.6219	8.7428	9.4787	10.0558	4.4882	8.4449	7.402	8.0768	7.0076	9.9281	9.4438	10.6001	9.7872	7.6391	8.7526	9.6582	9.2333	8.4122	9.7162	8.8303	9.1713
TCGA-69-A59K-01	522	0	3.5179	7.3245	9.3274	8.8332	9.0243	5.7683	6.7049	4.4535	7.6175	8.8215	9.5258	8.5855	4.5323	9.6624	8.5901	5.6318	8.8176	5.6672	6.8657	7.6099	8.8037	7.797	4.8724	7.9698	10.7331	10.3103	5.5008	7.3627	9.1914	7.5353	6.9896	9.0613	7.805	9.4568	11.7428	9.5065	8.6685	10.5109	7.9978	7.4866	8.6794	6.8958	6.3169
TCGA-71-6725-01	256	0	3.8497	6.2218	9.5299	9.8502	10.969	4.6089	1.6709	4.9951	6.7624	8.0378	5.9936	5.1038	4.1065	9.6981	6.2809	6.6381	8.5536	9.0134	5.064	8.9502	7.81	7.5442	5.9433	9.1646	9.7961	10.3587	6.0558	9.3241	7.8709	8.2299	6.9352	9.3547	7.1116	11.7593	7.4495	10.7617	9.6162	8.5049	6.3707	8.7742	10.2338	8.5024	7.6376
TCGA-71-8520-01	210	1	4.2464	7.4063	10.074	10.2591	9.3759	5.607	3.604	6.6106	7.5745	9.1057	7.9088	6.1635	5.6252	10.0294	8.5041	8.9856	8.8094	3.3645	5.8743	9.559	8.7994	8.2399	6.0054	8.0301	9.4223	10.0732	4.757	7.8557	8.8292	8.3149	6.5062	9.8836	8.0437	13.5705	10.8045	10.4483	7.9088	10.7083	9.3349	10.6399	10.9576	7.6923	7.5032
TCGA-73-4658-01	1600	1	3.2676	7.82	9.295	9.9217	9.3871	8.1905	3.9964	7.1019	9.5771	8.4163	7.867	8.3743	5.1031	9.6348	5.5904	6.9355	9.2804	5.7836	4.1907	7.278	8.577	8.1705	5.5198	9.0271	9.8543	10.2529	4.62	8.7983	8.6818	7.3408	8.8057	10.1918	10.209	9.7002	10.9785	6.969	10.0392	9.4667	10.0797	9.5587	9.7854	6.6012	4.5509
TCGA-73-4659-01	711	1	2.6495	8.1863	9.0864	9.7781	10.329	7.5245	8.1926	7.4689	8.3386	8.1299	6.9615	7.1404	4.4658	9.7582	7.3712	9.0758	9.7812	5.4257	5.1959	7.9655	8.3304	8.2477	7.7118	8.8635	9.7822	10.0154	6.6308	9.1218	8.2745	7.7964	7.1099	9.6585	8.3769	13.8998	9.6806	7.9384	10.135	11.0557	10.7263	8.5615	9.66	8.5436	8.0098
TCGA-73-4662-01	2515	0	4.1602	6.7154	9.9108	11.4562	9.0341	6.3388	6.6189	5.5956	7.8626	8.7793	7.1075	8.1964	4.692	9.187	7.9202	8.3943	8.5674	5.2405	5.5385	8.1519	9.1559	8.9504	4.8292	9.1144	9.2649	9.5016	5.3188	9.0527	8.7847	8.8188	11.838	10.7701	8.432	7.7562	7.8369	6.2877	10.1183	9.0539	9.4494	9.7618	10.044	7.7795	5.425
TCGA-73-4666-01	800	0	0.8111	4.4007	8.7494	9.7519	9.3497	5.3214	2.2549	3.593	8.202	8.7375	4.0957	7.2872	5.1684	9.7464	7.6344	8.1133	8.6335	8.3114	4.7499	5.803	6.9974	8.8464	8.0694	9.4303	10.8381	10.683	7.7264	9.1483	9.6598	8.0789	8.9461	9.6763	8.3159	7.4743	12.3668	8.589	8.5096	11.7194	8.6499	8.5096	9.6495	7.808	6.2987
TCGA-73-4668-01	467	0	5.1573	7.4526	8.4475	10.7965	9.4866	6.6063	2.934	6.6981	8.1706	8.4905	8.7809	6.9713	3.2646	9.9894	6.4848	7.7116	8.618	3.4383	4.6359	6.2438	6.8838	7.6703	6.5843	9.4083	10.198	9.6707	6.2391	8.9324	9.7	7.8822	8.6824	9.9644	8.5343	14.0661	11.2366	8.0047	9.3021	10.3803	9.6029	10.2825	9.9876	7.4073	3.7858
TCGA-73-4670-01	131	0	1.4005	7.6634	9.4448	9.7873	9.8726	5.8746	5.2019	5.6575	7.5782	8.7459	9.8582	5.8543	3.067	8.563	6.6627	6.682	8.1971	3.4015	4.1875	5.2648	9.2127	9.1236	6.9285	9.66	10.1493	10.8663	6.2818	10.0948	8.8623	7.8646	8.6342	10.2375	8.9637	11.0528	11.4183	8.7956	10.2537	11.7652	9.9323	9.0827	9.0776	8.5817	5.2545
TCGA-73-4675-01	922	1	3.0224	7.3015	9.7066	10.6069	9.5879	8.4569	1.7984	4.9127	7.9973	8.5007	8.6349	5.9257	4.173	9.6342	8.472	4.6884	8.005	4.6709	6.15	10.2899	9.383	8.0696	4.5426	8.1536	10.0216	10.5472	5.695	8.7051	7.8929	7.8235	6.6458	10.1929	8.0102	11.6292	9.8788	6.1625	9.9619	9.2925	10.0884	9.7152	9.3388	8.2711	7.2139
TCGA-73-4676-01	281	1	1.0394	6.7495	8.7088	9.673	9.1111	6.7353	6.4778	4.7753	8.4328	8.3768	4.8299	6.0073	4.4665	9.2993	6.1747	8.8342	7.839	8.6245	5.0978	6.2467	9.1329	9.3642	6.9475	8.7919	10.2706	10.9627	6.9475	8.0053	9.6062	9.2249	8.4107	11.0155	8.5247	10.2981	11.6772	8.2967	10.1303	12.13	9.4374	8.5164	9.4527	8.1436	4.5005
TCGA-73-4677-01	38	1	2.2505	5.0734	9.4791	10.382	9.6648	6.1897	4.2857	9.5823	7.6188	8.2507	7.9503	6.9048	3.3166	8.8648	6.6421	6.4503	9.3775	8.8175	4.2857	6.9761	8.5634	7.5771	3.6841	9.2875	9.9752	9.8279	5.1221	8.4137	7.3142	8.7081	7.6803	9.737	7.413	7.9704	8.9448	8.3163	10.0064	7.0504	10.3227	9.9246	10.2316	8.4499	6.4214
TCGA-73-7498-01	1189	0	6.7344	5.4615	8.9057	10.8358	9.7688	6.1396	4.7637	6.3662	9.4565	8.3054	8.1781	7.2912	3.6996	9.1571	9.7099	8.3482	8.8274	7.0981	5.2608	8.4314	9.2152	8.0769	3.8435	9.0093	9.6881	9.6258	4.643	8.1521	7.2582	8.8386	7.4615	9.9136	8.4674	6.7528	9.3847	9.2887	9.2943	6.6031	9.3735	9.1619	10.2891	7.5502	6.848
TCGA-73-7499-01	1531	1	2.8253	6.7318	9.284	8.283	10.267	6.1536	2.0782	3.7198	7.4983	9.1531	9.4108	6.9013	9.2334	9.247	7.8376	8.0219	9.07	6.3751	4.396	6.2449	9.9101	9.2648	8.3931	9.8414	11.3947	11.2797	5.8742	8.8998	8.5182	9.2056	8.7978	11.4876	7.4865	6.3998	9.4535	10.6855	9.5524	11.0571	9.3979	7.6316	9.2922	8.0018	6.168
TCGA-73-A9RS-01	340	1	0	5.7734	8.4202	9.2961	10.579	4.2177	9.609	4.2177	6.5979	8.4015	5.601	5.4051	1.8643	8.3788	8.031	9.5288	7.1392	3.6373	5.1784	7.8775	7.6624	8.3084	7.7056	9.3122	9.5357	10.6826	7.402	8.7293	9.6943	8.6076	7.4536	10.4021	6.7484	10.797	11.6713	11.8228	10.5295	12.5684	9.1018	2.4333	9.9949	8.8023	5.4051
TCGA-75-5122-01	0	0	2.9578	4.4134	8.6331	9.3274	10.244	5.1914	2.6034	5.5188	8.3177	8.8417	8.8497	9.3815	6.8485	10.4649	6.6439	8.1349	10.5216	5.8291	5.4919	7.17	8.2193	8.6423	5.9525	8.8257	9.3387	9.7048	5.1576	8.4321	8.9093	8.1607	8.0031	10.1376	10.315	9.2751	10.6449	5.5711	9.5715	10.8814	10.311	9.0145	10.0562	8.5573	6.9208
TCGA-75-5125-01	2027	1	1.8389	6.7578	8.5674	11.0659	9.4775	5.3827	2.3572	4.6271	7.3611	7.6767	8.6949	7.9325	6.9802	9.4069	7.8251	6.838	9.2521	3.1263	5.2522	6.2528	7.6621	7.6584	6.1726	8.7529	10.3368	10.6805	7.3566	8.6022	9.0951	6.9078	8.0182	9.8375	9.2175	7.5906	10.2679	9.7243	9.0039	11.0711	9.8162	9.3681	9.4211	7.2628	6.4864
TCGA-75-5126-01	0	0	3.1638	5.7428	9.2262	8.376	9.8489	6.0156	2.8815	5.2336	8.2295	8.8567	7.1079	8.0774	5.8057	10.4923	5.866	6.4026	9.4644	5.9796	4.3297	6.6514	8.6662	7.9807	5.9613	8.9664	9.1952	10.1492	5.8856	8.9502	8.6027	7.5183	8.0603	10.0146	9.2882	10.2086	9.7878	9.2243	9.5295	10.948	9.5993	9.5709	9.7813	8.0603	7.3096
TCGA-75-5146-01	2368	0	3.1368	5.6204	9.7752	9.1058	8.9615	6.9246	4.1127	4.5655	8.484	9.0946	8.4988	7.6753	5.1589	9.2804	7.0988	7.3197	8.4903	6.2232	4.6494	8.9512	9.2027	8.1982	5.7777	8.998	9.6019	10.8731	6.427	8.6155	7.4482	8.5542	7.4006	10.7142	8.4293	7.2542	8.7186	7.6803	10.2543	8.1912	9.3789	8.8216	10.7154	8.4931	6.2232
TCGA-75-5147-01	1333	0	3.5381	7.9202	9.6527	8.5708	10.569	7.3556	3.8645	5.1736	8.7755	9.0675	9.2568	7.8013	10.4768	9.3864	7.2023	9.1752	8.275	7.6903	4.5309	8.7923	9.7483	9.0142	8.9382	9.7776	10.7961	10.4347	5.5087	9.1205	9.0588	8.6007	8.495	11.0926	8.8986	10.2388	10.4884	10.9282	10.4524	10.2303	8.447	8.7933	9.9887	7.7646	7.5292
TCGA-75-6203-01	0	0	0.7551	6.0517	9.5898	10.087	10.451	5.7901	6.7496	5.5358	9.6118	8.233	8.1481	8.6754	6.0215	10.9833	7.2573	7.1773	9.8147	6.795	5.578	9.0097	9.532	8.5566	4.072	8.2395	8.6899	9.7528	6.5732	8.1127	6.9149	7.2245	6.4763	9.981	9.8452	9.26	10.405	7.5967	9.331	8.3317	9.7482	8.3652	10.752	8.0009	7.1636
TCGA-75-6205-01	0	0	0.8856	7.5306	9.079	8.5267	9.7838	6.8506	3.1089	6.0864	9.3368	8.5788	5.2588	9.2231	6.1043	10.1929	6.0314	8.4129	10.3566	5.6962	5.9146	8.7013	7.9715	8.3729	6.1043	8.1798	9.7301	9.4036	7.1465	8.0474	8.8412	7.34	7.3625	9.6086	11.123	10.3742	11.8115	7.3919	9.1081	10.3576	9.814	9.6303	10.0058	7.7452	6.7856
TCGA-75-6206-01	2590	0	2.5933	6.6227	10.0163	11.2691	9.2709	8.5859	3.6239	7.0225	7.9067	7.7855	8.8741	6.4569	3.1987	9.4963	7.5147	7.4795	8.5403	4.9926	5.0757	7.6154	7.6014	7.6788	3.1987	8.9696	8.673	9.2471	5.4527	8.3475	8.0481	7.8887	6.8204	8.673	8.1792	10.7772	9.6999	9.6306	9.4267	6.8124	9.949	9.6352	10.0696	7.3408	6.7292
TCGA-75-6207-01	0	0	2.7472	6.5026	9.3043	9.3477	10.382	4.9491	2.3092	4.1097	7.9871	7.8177	6.7287	6.2343	3.5298	9.1228	7.1773	8.2823	7.6542	3.4738	5.7001	6.3554	7.6573	8.7889	5.5991	9.5949	10.8227	10.2941	8.1501	8.241	9.6449	8.046	8.5425	10.8763	7.7278	7.8317	11.8149	9.477	9.3192	9.9532	9.0141	8.5408	10.3808	7.0443	4.6286
TCGA-75-6211-01	0	0	4.1774	7.8889	10.0811	9.3658	9.0033	6.3668	2.3059	8.2484	7.4668	7.099	5.3612	6.5296	9.3915	9.286	8.9254	9.8416	9.2128	4.5044	4.9655	7.963	7.4422	7.9167	7.0426	8.4435	9.3887	10.3626	7.5775	9.2255	9.4097	7.9668	6.9988	9.3353	10.164	12.2049	7.8401	10.1825	11.5556	10.7556	9.2936	7.9284	9.5042	7.8647	8.22
TCGA-75-6212-01	1516	1	3.0027	4.8992	8.8186	11.5267	11.014	5.5779	3.0027	4.561	7.1388	8.1924	8.1808	8.289	5.5779	9.5236	8.1093	5.2038	9.424	5.1414	5.6692	7.3704	9.9355	8.7393	3.7518	8.6441	9.6038	10.0639	7.0775	8.3385	8.5543	7.3771	7.2125	9.9286	10.37	8.0553	11.9398	7.7557	8.7074	8.8555	9.6549	10.3421	10.4689	7.8562	5.7127
TCGA-75-6214-01	1115	1	2.693	5.4027	9.1335	9.5025	8.4194	3.5397	2.3928	3.4623	7.7237	8.7598	5.8452	4.3531	6.704	8.8308	6.0174	7.3232	7.2299	5.0918	5.2031	8.021	7.7407	8.684	6.2424	9.0012	9.5582	9.8059	6.0899	8.2909	8.6978	7.1114	8.3896	9.9011	7.0299	8.5882	10.9115	9.2212	8.9311	11.3837	8.6733	8.6872	9.6151	7.718	7.7284
TCGA-75-7025-01	3305	0	3.9423	6.4152	9.7212	9.602	10.12	6.8001	5.2647	6.6775	10.0394	8.1947	8.4931	7.384	5.7125	9.7462	9.6577	8.7389	10.3382	9.229	5.374	8.6302	8.0102	8.3075	4.6117	8.5968	9.965	9.3824	6.3228	8.768	7.2578	9.0074	6.8577	9.8545	9.0948	10.0038	9.6799	8.3195	9.4604	7.521	9.0388	8.9881	10.2954	8.3665	10.4089
TCGA-75-7027-01	3059	0	2.9371	7.7938	9.3387	9.854	8.9443	6.1686	5.6501	8.0485	9.0224	8.8721	6.7134	6.58	3.7802	8.9451	8.0981	9.9381	8.4132	5.4734	6.1333	6.7292	8.3169	8.5521	6.3434	8.459	9.2503	9.8857	5.3862	8.8424	8.8872	8.0996	7.79	10.1683	8.5894	11.6014	8.7	8.1564	10.0495	11.5555	8.4626	8.4942	9.1582	7.3803	6.022
TCGA-75-7030-01	0	0	2.858	6.9288	9.0672	8.053	8.9506	6.6129	2.858	5.8652	9.7963	8.0755	7.5664	8.3255	3.9619	9.3807	7.5098	7.8334	8.898	8.403	5.0571	8.6698	8.0417	8.0012	3.2224	8.0012	9.0418	9.5325	5.1447	7.6733	7.3064	7.9535	6.6129	9.7731	9.206	11.1053	9.4893	7.8595	9.7224	7.8068	9.2536	8.8597	10.3303	8.3066	9.3603
TCGA-75-7031-01	0	0	4.8558	5.35	8.6513	9.4174	10.345	6.4958	2.4063	9.2819	7.5471	7.1914	7.2333	6.6272	8.9278	8.8956	10.8419	10.0819	9.4665	7.8389	4.9084	6.0573	8.7081	8.3089	6.8317	9.3501	9.9184	10.4422	5.2718	9.0204	9.0703	7.2125	7.1151	10.7539	9.0703	7.7766	11.2994	11.6169	11.4835	8.866	10.4164	7.6891	10.2833	8.0701	7.17
TCGA-78-7143-01	4961	1	3.8987	6.3337	9.0662	9.3586	10.081	5.7997	5.5712	3.1949	8.4752	8.503	8.5665	6.8177	6.4089	8.928	7.2218	7.0126	8.6533	3.3387	5.6283	8.8964	8.265	9.5764	6.86	8.5883	10.2197	9.8862	5.8958	8.8685	9.0095	8.3941	7.679	11.0936	8.4935	9.0713	9.9251	8.5883	9.1875	10.422	8.9576	8.9906	9.191	8.265	6.7614
TCGA-78-7145-01	826	1	4.4938	6.5201	9.8643	10.0082	9.9844	5.2125	2.343	8.2265	8.1625	7.8689	8.5692	7.05	6.9243	9.7703	5.6572	5.4228	8.3755	3.3661	4.4938	6.5562	9.7866	8.3267	5.738	9.6739	10.3174	9.9406	6.4059	8.4325	9.6626	5.7996	7.489	10.7791	8.98	10.864	11.3724	10.4098	9.1087	11.6006	10.3048	9.564	9.3581	6.8389	5.9014
TCGA-78-7146-01	173	1	2.3162	6.8446	8.9324	8.2874	9.1079	4.0376	2.9007	3.8007	7.3148	8.357	7.9586	5.5321	4.9238	9.2526	7.6439	7.8907	8.741	8.1273	6.1427	5.7209	7.6002	8.6599	7.2643	8.8399	10.5498	11.0555	6.2967	7.0697	10.1571	8.741	7.947	10.8281	8.0535	7.3193	12.3952	10.1362	9.3226	11.1256	9.4533	8.1552	9.532	7.4399	6.714
TCGA-78-7147-01	586	1	2.6954	6.5322	9.5669	10.4063	10.134	4.9959	3.0762	1.5637	7.0734	8.6568	9.2522	4.8856	5.6747	9.3445	8.5327	7.6728	8.351	4.1557	5.991	8.0289	9.4361	9.6613	6.3846	9.2175	10.7434	10.4269	5.9367	8.637	8.5494	8.8539	8.282	10.8494	7.7691	9.8765	9.9115	9.6848	8.8428	10.273	8.464	8.3902	9.662	8.6762	7.9282
TCGA-78-7148-01	626	1	1.3759	6.889	9.5896	11.0247	9.5916	6.7027	6.0417	8.2858	7.9447	8.3878	8.5378	6.0762	3.0781	8.4874	6.7885	9.1735	9.8636	6.4542	4.7293	8.1137	8.7682	8.4615	6.921	8.4373	9.3006	9.9098	5.9823	8.7558	8.3436	7.0307	7.2781	10.7132	7.6884	9.1881	10.2341	9.3295	11.3521	10.6568	10.7113	9.7117	9.9522	7.8027	7.5062
TCGA-78-7149-01	3940	0	5.2033	6.8194	9.9355	9.8943	10.054	7.8306	4.8084	5.5644	8.7247	8.7757	8.4192	5.1815	4.0867	8.8941	8.2519	7.4847	9.6763	6.6555	5.4777	8.4423	8.3157	7.0885	5.3665	10.3469	10.0953	10.7466	5.9969	8.3107	7.6243	9.1703	7.041	9.269	9.2003	7.5198	10.2594	10.2378	9.2818	7.5284	10.2742	5.4598	10.2742	8.3107	7.4533
TCGA-78-7150-01	666	1	2.3266	7.546	9.4002	10.0303	9.6244	6.4054	4.358	9.2948	8.974	9.2709	7.5101	4.358	4.1406	8.1284	6.4628	9.4221	5.1466	4.8917	6.2179	7.6731	8.2201	8.6009	6.2832	8.6777	9.9943	9.7402	6.7356	8.2692	9.6065	7.3004	7.6403	10.2488	7.9789	11.6265	11.423	10.5492	11.5172	11.6312	10.5787	10.8944	8.6614	7.8268	7.8875
TCGA-78-7152-01	1215	1	3.6019	6.0031	9.385	9.3567	9.9306	6.8008	1.9205	7.4634	7.655	8.5972	8.7006	6.8938	3.8999	8.3971	6.402	8.7006	10.2217	7.6146	5.4803	6.8711	8.3771	8.188	4.8036	9.1576	10.1969	9.6447	5.3873	9.0928	8.6811	7.7514	7.1225	10.1946	8.0583	7.4634	10.1552	5.4803	10.1715	9.0404	9.765	9.8618	9.1002	7.7943	9.6039
TCGA-78-7153-01	3635	0	4.9583	7.2368	9.7606	10.8595	9.949	6.1373	2.7063	9.2603	7.7788	7.9569	7.9817	5.7165	9.9062	8.9468	8.3534	9.5091	9.4596	4.4103	5.068	7.5568	9.716	8.3803	6.0056	9.0169	10.5747	10.0849	6.0464	8.998	9.2516	8.4878	6.8013	11.412	7.4341	4.1358	11.3308	6.4219	11.2585	7.8687	11.074	7.9098	8.6901	7.4943	8.8446
TCGA-78-7154-01	593	1	3.6219	6.0024	9.5391	9.3467	10.581	5.5312	3.6219	4.9709	8.8104	8.2338	7.3322	5.2445	4.633	8.5953	7.2041	8.3489	7.5857	6.2816	5.9889	10.3968	8.7688	8.962	6.5976	8.6042	9.6351	10.5514	7.8166	9.0161	9.22	6.9148	7.8726	10.0279	7.2099	9.2943	10.2898	10.1911	9.4387	10.6647	8.7268	9.4934	8.6326	7.9088	9.8616
TCGA-78-7155-01	1171	1	4.1986	5.4537	8.3343	8.3876	6.5661	4.0042	1.4521	5.8363	10.4633	8.0711	8.4318	5.7302	3.2751	8.2016	5.2711	9.9605	5.9487	10.761	5.0869	7.1101	6.7557	9.3792	8.4074	9.537	9.8804	9.3256	7.9242	8.3751	11.0361	7.0357	9.85	9.1308	5.3551	8.7573	10.928	10.2331	8.4934	11.6721	11.2926	4.0042	8.763	7.0857	3.6524
TCGA-78-7156-01	976	1	1.8022	4.3855	9.5016	9.7109	10.277	6.8663	3.9933	5.1878	8.0086	8.0173	6.7791	5.8003	3.0811	7.6528	9.2857	8.8551	6.8352	6.4192	4.9472	8.6515	9.3226	8.1014	4.6225	8.7876	10.1794	9.9582	5.5742	8.1273	7.4777	8.5806	6.7377	10.2452	7.6213	5.8003	10.5935	9.6043	8.9506	7.5125	9.6089	6.7544	9.5249	7.4471	7.3125
TCGA-78-7158-01	179	1	2.4762	5.0957	9.1323	8.4731	10.244	7.7265	2.2432	3.8357	6.4342	8.008	8.4714	6.1182	3.877	8.3592	8.8348	8.7301	7.5746	3.3404	5.2765	6.0202	8.7088	8.5582	5.3663	10.5401	10.2552	9.565	7.188	8.2376	9.3734	8.8787	8.0127	10.1475	7.8792	8.88	8.9935	4.4149	8.885	8.9722	8.9663	9.4929	9.3734	7.1589	6.5977
TCGA-78-7159-01	1974	0	3.885	6.2466	8.8315	8.7246	9.8763	6.746	4.7194	8.0995	8.551	8.7057	8.4683	7.304	5.7222	8.6652	7.794	8.7209	9.1704	4.2196	5.6449	7.6629	8.211	8.7727	7.0697	8.9868	10.4073	9.8325	6.8041	8.274	9.1524	7.9755	7.8047	10.5683	7.6471	11.2602	8.322	8.6962	9.9294	9.9359	10.5413	8.8608	9.1552	7.6901	8.9615
TCGA-78-7160-01	697	1	2.0547	6.3971	9.8379	9.3093	8.7291	6.4397	5.4141	7.7973	9.6866	8.2351	7.7044	7.8297	4.1213	9.45	6.8558	8.9876	9.3889	7.7891	6.308	8.3145	8.3924	8.3484	5.5563	8.7879	8.9159	9.6794	5.106	7.9152	8.9458	7.9851	6.9764	9.6671	8.6811	10.8121	9.9012	9.2627	11.1615	10.924	9.8439	9.2862	9.9563	7.6511	6.589
TCGA-78-7161-01	291	1	4.7398	9.033	8.4695	9.7171	9.6342	6.5023	4.5264	7.6815	8.4864	7.5377	9.4831	5.1058	3.0615	8.8031	8.7037	10.0736	10.3927	9.9461	6.5764	8.7828	8.1862	8.0205	7.0342	9.2251	9.3834	10.0887	4.9082	8.2572	8.1844	8.2999	7.0819	10.4604	6.3416	12.2451	9.127	10.2705	9.8609	7.8948	10.3425	7.6528	9.5826	7.9537	7.3128
TCGA-78-7162-01	3169	1	3.3942	4.7341	9.51	8.7995	9.946	8.1582	5.591	6.14	9.5071	8.2719	7.811	7.2421	4.0328	8.7995	7.1669	8.9755	8.0765	7.4191	5.591	9.878	8.471	8.358	4.9199	9.1904	9.0541	9.7728	5.379	8.1893	7.8412	7.696	7.3784	9.7268	9.122	9.6631	10.1451	9.2627	9.0035	8.0882	10.0417	7.4435	10.0968	7.0282	7.6389
TCGA-78-7163-01	7248	0	0.4248	7.7595	10.0423	10.8029	9.4592	6.567	0.7526	1.7641	4.8273	8.9171	9.2504	3.8433	5.2855	9.8465	6.6332	9.5602	7.6159	10.1947	5.007	7.1258	9.2035	8.7945	3.8433	8.3724	9.4725	10.7781	5.6913	9.7716	8.5061	8.1365	6.5354	10.6846	6.1326	12.458	7.4307	8.6667	9.9182	7.8692	8.7027	9.7332	11.576	7.9514	8.4535
TCGA-78-7166-01	258	1	3.6024	6.5016	9.583	9.8481	8.7267	5.7079	3.3098	3.9323	7.4924	8.5337	8.6867	3.8	2.7646	8.0759	8.5663	7.4829	8.5932	6.7309	5.6831	5.9868	8.8108	7.8986	6.8359	9.5195	9.9576	10.8845	6.611	8.4589	8.0925	9.2261	7.2193	9.8467	6.1943	11.3866	10.2756	9.5821	10.7513	10.7073	9.549	8.4237	9.5204	8.7978	6.9496
TCGA-78-7167-01	2681	1	6.3174	7.5923	9.1468	10.4879	9.3083	8.0923	2.5974	4.8985	8.1203	8.3092	7.1664	5.3301	2.7957	8.2874	9.0071	10.0637	7.744	5.4329	4.8695	7.1301	7.6446	7.4815	5.1844	9.0699	9.7369	10.34	4.9823	8.1141	8.0379	9.0129	7.5053	9.3251	7.2017	6.0067	10.2052	9.5454	10.6013	5.6787	9.6972	9.873	10.404	8.4507	5.7521
TCGA-78-7220-01	807	1	3.7392	6.1498	9.6098	10.754	8.6097	5.9238	3.8463	6.9303	8.2802	8.419	8.7054	5.626	2.736	7.773	7.0639	10.0794	7.9426	3.6234	6.6189	8.1051	9.1226	8.5396	7.5375	9.6602	10.3168	9.9207	5.8356	8.5296	8.7036	6.8933	8.5376	9.846	7.1131	11.0829	10.5257	10.1827	11.5227	11.76	11.223	9.1119	8.5868	8.3015	5.769
TCGA-78-7535-01	949	1	3.9608	8.3713	9.5544	10.5704	9.8248	4.2027	1.047	4.7327	6.962	9.3082	4.9638	5.5166	5.2053	10.0605	8.1455	7.0842	9.8304	3.9608	5.9153	7.1252	7.8712	8.5475	6.4894	8.9636	9.9034	11.1627	6.4837	9.1366	8.3775	8.34	6.8195	10.3677	9.273	10.8854	10.09	10.4968	9.199	9.6204	9.1266	7.7808	9.444	8.405	8.5746
TCGA-78-7536-01	244	1	4.0316	7.1601	8.0108	7.5092	9.7658	4.7739	2.4196	10.128	6.3525	7.402	2.0253	6.7579	3.6457	8.971	8.7122	10.8562	8.9606	1.3422	4.6322	9.5389	8.7245	10.0149	8.4899	9.4302	10.1141	10.0609	6.505	9.0016	9.1316	7.8067	6.913	11.2402	8.7087	6.844	11.8094	5.3182	10.7879	10.8297	9.0581	7.053	9.0525	7.8377	8.018
TCGA-78-7537-01	1622	1	3.753	5.6398	8.7411	10.5156	10.23	7.6309	2.5833	6.9505	7.6453	8.2853	8.7144	5.7775	3.753	9.1927	8.1158	6.8245	10.4435	6.1976	6.4302	7.5781	8.6273	7.6592	4.7376	9.2023	10.1468	10.3603	4.4386	8.3498	7.4248	8.1415	6.4001	9.9507	7.6257	6.8997	9.0447	7.3225	9.2171	7.3854	10.1354	8.8092	10.1372	7.2123	8.0396
TCGA-78-7539-01	791	0	5.049	5.0618	9.3493	11.3805	9.561	5.6323	5.9047	3.3407	6.7811	8.5038	6.871	7.3145	5.0995	9.7923	7.189	9.217	10.1293	3.676	4.9967	5.5531	8.8471	8.4826	5.7236	8.8925	9.0427	10.4151	3.3407	8.6964	8.0874	9.317	7.2738	10.0803	8.402	6.097	7.67	10.5876	9.5861	7.3617	9.4192	9.3056	10.8162	8.7761	6.1989
TCGA-78-7540-01	1197	1	1.4098	4.5363	10.6711	9.4116	8.9632	4.6358	2.578	2.4956	8.3159	8.0702	7.1071	7.0506	3.5773	9.976	7.2553	8.9593	10.55	8.5034	5.0216	8.0738	8.6781	7.6064	3.8667	8.4221	10.1653	10.7167	4.5769	8.309	6.6451	7.121	6.5966	9.3768	7.2491	8.6581	7.7354	5.2772	10.9005	8.9497	8.7653	9.3367	10.4349	7.6711	11.2793
TCGA-78-7542-01	321	1	0.6395	3.9544	9.4951	10.8783	9.2777	4.1022	0.877	2.2096	7.7641	8.5312	4.6904	5.3826	3.0925	9.3142	7.0268	4.3978	7.2573	2.7775	5.5721	9.6398	7.3637	8.8377	7.1817	8.7739	9.3274	9.6909	8.1401	8.6411	9.9095	7.5188	8.6451	9.1936	7.0543	10.7225	9.7326	5.3235	9.0029	11.0983	8.7958	5.813	9.9141	6.7746	5.9232
TCGA-78-7633-01	1528	1	5.5209	4.4606	9.786	10.1272	9.0844	9.395	4.9244	7.4191	8.3783	8.8188	8.3387	5.6255	2.1554	9.2835	7.7025	8.6252	6.4586	4.9912	5.4177	9.2934	8.0483	8.1542	5.209	8.4002	9.6401	9.6112	5.4749	8.301	7.3056	8.5057	7.3134	10.175	8.1351	7.7763	9.6265	10.7049	8.2623	8.3245	10.7434	8.1987	10.4703	7.1994	5.8724
TCGA-78-8640-01	7062	0	1.97	5.9604	8.9706	8.2746	11.439	3.3696	1.4597	2.8912	7.1513	8.9263	7.4063	6.0265	3.0004	9.97	7.2156	9.5433	10.0705	3.1968	5.9332	6.9143	7.6934	8.1138	6.5422	9.5646	10.4156	11.1213	7.3403	8.5994	8.9332	8.9071	8.1586	10.3888	7.7609	10.5797	11.4501	11.7136	9.4787	10.5034	7.134	5.8333	8.4462	8.0923	9.3401
TCGA-78-8648-01	1209	1	3.5478	9.674	8.7468	9.4715	9.7604	8.1673	5.2643	8.555	9.3947	8.2921	7.3002	8.7235	5.4772	10.4518	8.1402	6.9806	10.5172	6.7337	5.5729	8.4102	8.4134	8.3026	4.7535	8.0391	9.429	9.6981	5.1448	7.5559	7.9483	8.0349	7.3949	9.6329	10.323	12.7063	10.1968	9.161	9.1725	9.6385	9.182	7.5559	10.7577	7.788	7.3416
TCGA-78-8655-01	2360	0	0.7078	4.3219	8.351	9.9617	9.6224	5.4616	2.8744	5.1113	8.1774	8.4795	8.019	7.0104	4.7531	10.1187	7.3885	5.4195	9.0902	4.3219	5.9788	6.4451	8.5903	8.0945	5.0015	9.494	10.2588	10.4433	4.4939	8.1177	7.7229	8.5445	7.1838	9.8632	8.8696	12.9165	9.2829	7.4629	9.2215	9.2352	9.1217	8.5174	10.6388	6.9152	7.4102
TCGA-78-8660-01	321	1	3.5949	5.2823	9.6197	8.7648	10.053	4.8579	2.6438	2.8907	7.0454	8.0856	8.436	6.7976	3.1964	10.2431	7.5961	8.5025	10.3643	6.7747	5.7256	5.9464	6.6958	7.3706	8.6955	9.2205	9.9686	10.1727	8.1102	8.7879	8.9983	7.6348	7.1215	9.5383	8.3865	9.013	10.5548	9.8099	8.2769	11.266	9.2554	9.5992	10.3617	8.7216	6.2854
TCGA-78-8662-01	3361	1	3.5347	5.5846	7.6453	8.4746	10.569	4.7447	1.5779	3.4499	7.4074	8.488	10.3706	3.2636	4.5547	9.1127	7.6164	6.784	7.1085	7.1957	4.8143	7.4297	7.3732	7.2841	6.731	11.4138	10.9507	10.407	7.1892	9.0165	9.4317	9.2476	9.0311	9.9354	5.936	10.1936	10.5009	11.4545	8.2231	9.9138	8.9388	6.0981	8.9504	8.2516	5.3934
TCGA-80-5607-01	0	0	3.4281	4.7128	9.0362	8.1213	9.3176	5.9921	3.8093	3.9677	7.7956	8.2503	9.2147	6.7711	4.9209	8.4481	8.1381	8.227	8.4548	9.065	5.4093	9.0022	8.4312	8.8272	5.9921	8.5009	10.0348	9.9519	5.0681	8.0567	8.0742	7.807	8.0872	10.376	8.1792	11.6741	10.3698	6.0641	9.5624	10.7271	8.7475	10.2331	9.0953	6.9714	7.8935
TCGA-80-5608-01	2832	0	4.3827	5.7219	9.2177	9.4358	10.466	4.8231	7.6563	9.26	7.162	8.7366	9.26	5.8516	4.2811	9.1088	7.8305	8.9991	9.435	6.8656	5.0716	6.6884	8.0758	8.4305	7.3304	8.8912	9.9937	10.7236	5.8604	8.6218	8.0019	7.8305	7.1584	10.8991	6.8256	8.9971	10.8715	9.5878	9.054	9.5239	9.8536	9.2202	8.8126	7.9156	9.8403

TCGA-80-5611-01	2595	0	2.9428	6.4403	8.6897	8.7072	10.799	4.1014	0.6391	2.1192	7.7701	8.9735	2.1192	6.6227	5.2399	9.2923	6.9821	7.4868	9.1281	2.1192	4.4713	7.6113	7.8179	9.4751	8.0449	8.5292	9.5152	10.1253	6.431	8.7949	8.7913	9.0709	7.8976	11.6208	9.4116	7.4132	11.5963	10.4871	9.3648	10.7426	9.616	6.1545	9.2585	7.9891	5.5107
TCGA-83-5908-01	824	0	5.2546	6.3057	8.3905	9.1082	9.8762	5.2259	2.9559	4.4113	8.3292	8.2641	3.9326	8.0416	7.5747	9.2538	7.1517	10.1337	9.4282	8.4963	5.1052	6.568	7.5518	9.6063	8.0681	8.8604	9.9355	9.8388	6.624	8.304	9.7968	7.8077	7.6841	10.5124	8.7273	7.8514	9.9182	5.3639	9.3323	11.8103	9.2758	9.2493	9.2013	7.5747	7.2224
TCGA-86-6562-01	376	1	3.3379	7.5996	9.791	9.2486	9.9527	6.5338	4.4879	6.0175	8.8051	8.3761	9.1015	6.9512	3.6771	9.8109	6.11	5.8126	8.0007	4.9041	4.8781	9.4433	8.3069	8.3644	5.7708	8.3761	8.8381	10.4676	5.0513	8.2547	8.1713	8.0097	7.0995	10.2442	8.8632	12.3672	10.6432	9.8633	9.5158	10.7149	8.9918	9.6624	10.4316	7.6847	7.7544
TCGA-86-6851-01	179	0	2.6807	6.7058	8.817	9.4967	9.8766	5.6059	2.5464	4.1072	6.9105	8.5539	8.9526	9.0635	5.4122	9.8537	7.985	8.1761	10.5044	4.537	5.676	5.85	7.8755	8.9401	5.6103	8.4613	9.7193	9.966	4.8678	7.9605	8.6212	8.127	7.6238	10.0732	9.0214	7.6588	8.2509	10.1392	9.1376	9.6041	10.9099	9.4328	10.663	8.6097	7.8825
TCGA-86-7701-01	947	0	3.0663	9.2824	9.543	7.7963	10.037	7.1518	2.6482	10.244	8.8825	8.5551	8.5712	8.6106	3.8776	8.8633	7.6751	10.4527	9.1361	5.6887	5.9812	7.0057	7.4674	8.7583	7.6676	8.1838	9.0612	10.3827	6.6446	7.9761	9.0498	7.2349	6.9697	10.782	8.1521	11.0311	11.45	8.2552	10.3747	10.2188	10.7003	8.2847	10.1536	7.6065	6.7891
TCGA-86-7711-01	1046	1	1.746	8.9914	9.9753	10.2822	9.4959	6.1625	2.2708	7.0375	7.8128	8.9326	4.1277	7.5377	3.5354	9.22	5.3584	9.4559	7.9014	7.6519	7.3185	8.1262	7.1374	8.6704	5.8382	8.6859	9.7408	10.1043	7.1222	9.1144	10.1124	7.7616	7.5716	10.0213	9.8995	13.0719	10.7096	10.7038	9.3376	11.9989	9.2496	6.0323	9.4161	8.1769	4.6514
TCGA-86-7713-01	1157	0	5.1935	5.5226	9.5211	9.7817	9.7426	5.3965	8.7904	8.7369	7.4294	6.8454	9.1597	6.1346	3.7555	8.728	10.1678	10.8447	9.1097	2.597	6.2209	8.425	8.265	9.4876	6.3456	8.9885	9.9253	9.6315	5.2674	8.3684	8.8321	8.4589	8.0554	9.5491	6.4244	10.0714	10.6134	8.7818	10.3409	9.8446	9.1493	8.545	6.8544	7.7532	6.5333
TCGA-86-7714-01	625	1	1.9334	6.1596	9.2791	9.3348	9.3556	6.1596	2.731	4.3741	8.2321	7.7183	8.5689	8.0504	3.7244	8.8116	6.842	5.7701	8.0757	8.4421	5.1609	7.3457	8.473	8.6386	4.1633	8.6723	9.2527	9.3158	4.6688	8.4653	7.9877	7.3204	6.9446	10.1478	9.8907	10.143	9.1514	6.0817	9.3243	8.1396	9.471	10.3853	9.816	7.6591	7.3622
TCGA-86-7953-01	997	0	2.5904	6.1137	10.0787	10.1099	8.815	6.462	3.386	6.0636	8.0435	9.3718	8.2639	8.1439	6.8826	9.2228	7.7376	8.7882	10.0858	3.9242	5.483	7.3738	8.2583	8.9547	6.652	8.5989	10.2638	9.811	5.9783	8.9564	9.0341	7.3321	7.2809	10.1256	9.6815	10.834	9.4023	10.1363	10.6044	10.7973	8.2288	11.6099	9.2719	7.1032	6.5189
TCGA-86-7954-01	605	0	1.6511	6.6746	8.7098	10.6731	9.325	5.7109	2.9923	5.6811	7.5803	8.3844	7.7964	7.9353	8.4609	9.8259	8.1312	8.7992	10.0511	4.5858	4.915	9.6872	7.8442	9.0492	5.973	8.7561	9.3512	9.6961	5.6584	8.5408	8.3774	7.9986	8.03	10.5588	9.4654	8.8511	8.8603	11.3082	8.5168	10.1725	8.8009	8.3751	10.7487	8.7949	7.2345
TCGA-86-7955-01	1072	0	2.3833	5.9577	10.4095	10.7078	10.228	3.6722	0.9288	8.7671	8.15	8.8171	7.6684	3.5289	0.6803	9.151	9.3278	7.5152	5.3598	6.754	4.9599	8.3097	8.7611	8.4267	8.8476	9.0191	10.3502	10.406	7.422	8.2308	9.728	7.963	7.6272	13.2496	6.4653	11.002	10.6837	11.0916	8.8479	11.1992	7.8413	5.2725	8.5776	7.8657	6.0392
TCGA-86-8054-01	1148	0	2.5839	7.1107	8.5314	9.1116	9.4298	5.4472	9.0853	11.094	9.8165	8.3723	6.5887	5.5635	3.5837	8.5025	9.5462	9.7198	6.9721	7.5275	4.4799	7.0826	6.7491	8.606	7.5065	9.1669	10.0038	9.9061	7.128	8.5533	10.1807	8.0719	7.5685	9.5144	9.6676	11.7927	11.887	6.517	11.2676	12.3518	8.7175	7.3533	9.3504	7.2872	5.3679
TCGA-86-8055-01	124	1	4.3728	7.2055	9.5069	9.0979	9.7431	6.7339	6.173	6.6065	9.2088	8.5482	8.7237	7.7272	5.3974	9.5187	6.9544	9.8522	8.7193	7.6275	5.3827	8.9356	7.6797	8.1687	6.6316	8.2863	9.6895	9.4363	6.0663	8.1858	8.2235	7.4156	7.4794	9.7553	9.3066	13.4057	11.2904	9.3046	9.1801	10.5349	9.4646	8.9701	9.999	7.5603	8.3615
TCGA-86-8056-01	139	0	5.1556	4.5768	9.5333	8.5245	8.5053	6.5942	4.0218	5.578	8.4168	7.9703	8.1761	8.0629	4.5557	9.4226	7.4619	8.0925	9.088	9.1874	5.1834	9.3984	8.538	8.519	4.4222	8.6467	9.0612	9.063	4.0522	7.7765	8.0441	8.9269	6.7966	9.45	9.1126	9.4284	8.4521	8.8226	9.9942	8.0365	9.1008	9.8511	10.0598	7.1707	7.6328
TCGA-86-8073-01	740	0	3.0387	7.7211	9.607	7.4702	9.1605	7.3927	5.3408	6.1024	9.7683	8.4773	8.7306	7.0625	5.3036	9.7089	7.7628	9.4926	9.4293	6.864	5.7622	11.5914	7.9982	8.0793	6.4866	8.8385	9.3706	9.6228	6.2334	8.0571	9.2633	7.6113	7.2566	9.5605	9.713	9.1271	12.2067	8.8023	10.4539	8.8938	8.6747	7.7421	10.4543	7.4952	5.9014
TCGA-86-8074-01	24	0	5.3204	7.8661	9.0507	10.0706	9.655	5.408	6.6335	6.1866	8.4837	8.6395	7.8393	7.2014	5.6009	9.5246	7.0809	7.5094	8.616	4.238	4.5988	8.6523	8.9676	9.4689	6.881	8.9563	9.9386	9.2535	4.7786	8.5363	8.4147	8.0602	8.3259	10.6798	9.0063	12.7574	10.5407	10.3136	9.798	10.4119	9.482	9.1975	10.44	8.6879	5.3332
TCGA-86-8075-01	694	1	3.4592	7.6529	9.05	9.5629	9.005	6.9917	7.0046	7.0332	9.0086	8.1903	8.2058	7.5772	5.1173	9.3175	6.5826	7.0978	9.3702	8.9946	5.4068	8.9791	7.323	9.2351	6.3243	8.8439	9.9891	9.8316	5.5824	8.8484	8.2605	7.4297	8.534	9.9777	9.2562	12.5839	10.0825	10.8278	10.1296	10.4725	9.0854	9.3208	10.2136	7.4951	6.1023
TCGA-86-8076-01	993	0	2.5053	6.9876	8.725	9.8884	9.2121	6.5866	2.6909	6.6607	8.09	8.3747	8.0321	8.2343	4.8804	8.9577	7.4723	6.992	9.2706	7.1188	5.6804	7.2903	8.1176	8.1116	4.4393	8.7785	9.9108	9.7704	6.0033	8.6146	8.8965	9.0571	7.1107	9.6376	8.3491	9.4717	10.5282	7.1626	10.4612	8.1337	9.2495	9.0454	9.5405	8.6846	8.1536
TCGA-86-8278-01	944	0	3.6631	6.3784	9.5994	11.1788	9.8265	6.1393	5.2897	5.6736	7.7136	7.9533	7.6744	6.7595	5.4693	9.496	8.1346	6.5479	8.8566	5.9766	6.6257	7.2222	8.3204	9.1466	4.871	8.6023	10.032	9.1972	4.526	9.1159	8.7727	7.8403	7.1423	10.0266	9.7856	6.8914	9.9796	5.5026	9.6248	10.196	8.9367	11.2438	10.4617	8.2073	7.2071
TCGA-86-8279-01	949	0	6.3968	6.9469	9.0901	9.7772	9.193	6.5043	3.5443	5.3045	7.7059	8.1068	8.6075	5.3679	5.2605	10.3489	8.4403	8.4143	7.9427	3.5072	5.3471	8.4756	8.444	8.6249	5.3783	9.9522	9.8288	10.0356	5.0573	9.1265	8.7981	8.822	9.9647	10.0429	7.8457	11.2622	8.4292	9.9659	8.2708	9.0317	8.113	7.3702	9.5952	8.1526	8.801
TCGA-86-8280-01	701	0	4.2054	6.6262	9.4377	10.3307	9.7204	7.2603	6.0379	6.1455	9.6757	8.1835	8.2624	7.934	5.7175	9.4286	8.5055	9.1135	10.1833	7.0626	5.0439	8.5565	8.7782	8.7431	5.9726	9.622	9.4094	10.547	6.2663	8.5509	8.0553	8.7431	7.0389	9.8697	9.0084	8.4023	10.1454	10.0747	8.6377	9.079	9.4991	8.6338	10.2477	7.951	7.2327
TCGA-86-8281-01	0	0	4.7423	7.857	10.0838	11.4388	9.2812	6.3037	4.4057	7.486	7.7664	7.5274	8.1885	6.5162	3.0552	8.8098	8.0887	8.6602	8.9072	9.4328	5.076	8.264	8.236	7.9615	3.721	9.1369	10.0596	9.6855	5.2707	8.5824	7.3136	8.2575	7.1986	9.0324	7.8635	9.0768	8.9393	9.644	10.9391	7.3073	10.3897	8.5719	10.0643	7.5571	8.8042
TCGA-86-8358-01	653	0	4.1891	5.9232	8.4059	8.9819	10.935	5.4187	3.6933	7.0036	9.3185	8.9122	9.6735	6.5107	6.9961	8.8472	8.0566	8.79	8.8942	9.9087	6.2716	8.3217	5.6649	9.2536	8.2976	9.5295	9.7555	10.3928	8.8678	8.8922	9.8268	8.0566	8.5146	9.75	8.3803	7.0904	8.9591	9.28	9.7818	12.5681	8.7191	5.0075	9.4674	7.0404	11.152
TCGA-86-8359-01	444	1	3.5018	9.2201	9.0894	11.3507	9.1118	4.9434	3.0514	9.0067	8.3334	8.8445	8.696	6.9721	4.7288	8.7024	7.3877	8.6704	8.9475	2.9417	5.0264	7.8457	6.5539	7.0731	5.7013	8.8969	9.9628	10.2167	4.8247	9.2333	8.679	7.5602	6.6088	9.5992	7.0925	10.0929	11.1003	10.6146	10.0985	8.9315	9.5626	9.9584	9.3956	8.47	10.4663
TCGA-86-8585-01	353	0	3.1243	6.8542	8.6644	8.9124	9.5397	4.1328	1.6806	1.6806	7.4071	9.2442	3.5883	6.4062	3.7149	9.1409	8.4215	7.935	9.5461	8.1237	5.6301	5.347	8.3157	7.9735	7.1736	8.9386	10.1509	10.9645	6.4433	8.8924	7.6403	8.7183	7.1288	10.938	10.8905	6.9937	10.6841	9.9676	6.6743	9.4356	8.2596	6.0458	8.5671	10.9968	7.6874
TCGA-86-8668-01	423	0	3.7707	6.6433	9.2814	9.3668	9.5768	7.5003	5.9371	6.3196	8.9211	8.2222	7.9104	8.3527	5.2834	9.2465	7.8879	5.9371	9.1055	4.9776	5.7098	7.9885	9.4021	8.2365	4.6553	8.1747	9.5223	9.8198	6.118	8.4963	8.0465	8.0302	6.7667	9.6654	9.3798	9.243	11.6534	6.6856	8.1101	8.0667	9.5003	9.5369	10.0934	8.0137	6.4603
TCGA-86-8669-01	938	0	4.527	8.366	8.6547	9.2096	10.311	5.9275	2.9876	5.9124	7.9615	8.4411	8.0465	7.5653	9.4259	9.2564	8.1842	8.8005	10.0195	5.0226	4.0663	7.4505	8.4671	8.396	3.9535	8.7737	10.0128	9.9406	6.8302	8.2662	8.1716	7.466	6.938	10.6217	8.1684	7.7624	9.5608	10.2788	10.9537	8.4542	10.0493	8.377	9.4547	8.0972	10.6395
TCGA-86-8671-01	839	0	2.7027	6.3282	9.4033	8.7583	10.105	7.5907	4.9428	5.81	8.2674	8.3925	8.0162	8.4113	5.4406	10.1288	8.5952	7.9078	11.0346	6.9528	5.9708	7.5274	8.4819	8.0615	5.4035	8.3122	9.1386	9.6763	3.5655	8.2112	7.9662	8.0822	6.8042	9.6744	9.527	6.8601	9.3199	9.9634	8.8106	9.2088	9.5673	9.8489	10.3237	7.6313	8.3589
TCGA-86-8672-01	19	1	2.1587	7.9525	8.224	9.2013	9.0032	5.8008	1.6225	7.2086	8.0601	8.6643	4.2493	5.746	4.6976	9.2579	7.6127	6.1212	9.0623	5.0986	5.9212	5.1275	6.9175	7.4393	6.5202	9.2431	10.4313	10.8624	6.2581	8.6887	8.6692	8.0873	7.3624	9.9257	8.5936	9.4659	10.2608	10.5574	9.2628	9.4687	8.8028	9.0187	9.2693	7.653	5.7828
TCGA-86-8673-01	862	0	2.7793	8.2656	8.3894	9.7067	9.841	6.2269	3.0069	8.1272	7.0988	8.9813	7.8335	4.8362	5.6175	9.8271	8.4267	6.654	8.3969	3.2034	6.1093	5.8406	7.4285	7.7952	6.3461	8.9644	11.2312	11.5495	6.4754	8.3275	9.3266	9.0611	7.0031	9.6801	8.3868	8.004	10.6683	5.7333	8.879	9.9899	9.029	8.6003	9.6583	8.3665	6.9965
TCGA-86-8674-01	806	0	3.2375	7.2971	9.7319	10.6761	9.1387	5.8938	2.1285	4.3697	6.5507	8.6442	8.1711	2.8446	1.0869	8.1254	9.0172	7.4944	9.1067	9.3398	4.6855	7.5603	8.0386	7.162	5.8382	9.7146	10.638	10.4227	5.8801	9.0359	7.9063	9.4633	7.5033	9.6535	5.5226	10.4548	9.8666	10.3831	10.1582	9.1008	10.34	7.6639	9.6545	7.8048	9.4225
TCGA-86-A456-01	896	0	4.3588	6.2723	9.9092	10.0726	9.63	5.1657	2.908	3.9806	8.0806	8.2527	8.3605	6.3265	4.3166	9.8308	8.8657	7.1231	9.6798	6.7556	6.0965	8.3448	9.1667	7.7046	4.2483	9.3281	9.7947	9.4441	5.6801	8.6763	8.823	8.4115	7.2005	10.1071	8.4872	10.4936	10.4351	11.0313	9.4206	8.5408	9.3439	7.2406	8.9629	7.2236	6.9196
TCGA-86-A4D0-01	116	1	4.4858	6.1923	9.3621	9.5873	12.143	4.5154	2.2396	6.4521	7.1685	8.4947	8.3417	5.6796	8.7807	8.6108	12.0171	10.4268	8.3479	6.9251	5.7562	6.3405	7.6517	9.2931	7.5693	9.2899	10.2941	9.9971	7.4968	9.1645	9.723	8.3642	8.0286	10.3883	7.1402	10.5626	11.418	8.972	10.6281	10.7931	8.7436	7.2099	9.3016	8.7683	9.031
TCGA-86-A4JF-01	737	1	4.0446	5.9047	8.2583	9.7686	9.5786	4.7382	3.0156	6.0175	7.4863	9.2317	8.1158	5.9154	3.965	9.2135	8.2309	8.9727	8.5637	8.62	5.4583	7.306	7.662	8.6313	7.5403	9.1304	10.3162	10.145	6.5616	9.5241	9.0116	7.2143	7.7917	10.7685	7.6808	14.217	10.605	10.1049	9.4602	11.3695	9.4818	8.8631	9.4066	7.7055	5.9775
TCGA-86-A4P7-01	415	0	5.7069	6.7514	8.9313	9.7203	10.087	6.2197	5.2659	6.3001	9.6888	8.0459	8.5555	7.355	4.2135	10.1921	8.623	7.666	10.7267	7.9384	6.2914	9.2337	7.9188	8.0741	6.576	9.0689	9.5176	9.8799	5.6261	8.7252	8.1982	8.442	7.5244	9.2484	8.5055	12.8908	8.8297	9.7992	8.877	9.0791	9.1591	8.1241	10.5134	7.7124	8.7916
TCGA-86-A4P8-01	805	0	1.7132	4.3322	9.0278	7.6808	10.283	5.891	6.526	6.3027	10.437	8.3567	7.536	7.7405	5.484	10.3842	9.6273	7.7859	11.7299	8.316	6.0182	8.5947	8.4686	8.0641	3.463	7.8443	9.1346	9.444	5.8229	7.4559	7.1341	8.3405	6.5889	9.0919	9.3209	11.0965	10.0924	7.7497	8.7216	6.4826	9.5311	6.5105	9.9984	8.0493	8.7555
TCGA-91-6828-01	323	0	3.1003	5.184	9.2497	8.4595	9.2929	6.6486	3.7683	5.9839	8.6061	8.0922	8.7548	8.5913	9.2377	9.3884	7.986	10.0093	9.3311	8.4571	6.2302	8.0561	7.4393	8.5881	5.2493	8.4766	9.5925	9.3665	5.2809	7.5728	8.7771	7.2765	7.0012	10.015	9.1377	8.8288	9.2968	8.8812	9.5183	8.9565	9.9015	10.559	9.1697	7.0106	8.101
TCGA-91-6829-01	1258	1	3.901	9.2543	9.6882	9.2543	9.3703	7.824	4.0878	8.282	9.5333	8.4973	8.294	7.17	6.5214	10.017	5.8656	10.359	7.9649	8.0031	5.5046	9.2494	8.6467	9.3246	7.3451	8.4618	9.8567	9.328	5.8656	8.1856	9.247	8.0002	8.4512	10.5264	10.148	12.145	10.9769	10.4315	8.7552	9.5969	8.3384	7.5616	10.4724	7.399	4.7994
TCGA-91-6830-01	60	0	4.5843	6.3749	9.1124	8.925	9.0821	6.8856	6.0305	7.7519	10.629	8.293	8.4903	7.3527	6.1127	9.5764	7.3795	8.113	9.0281	7.9748	5.2396	9.4125	8.9318	8.8348	4.3332	8.72	9.636	9.8761	5.21	8.217	8.0266	7.5426	7.4188	10.0777	9.1084	9.6703	10.9136	10.3399	9.9661	9.4651	9.6905	8.0181	10.0394	7.8606	7.5187
TCGA-91-6831-01	310	0	5.4153	7.4029	8.9533	10.1154	9.718	5.0287	3.2463	10.234	8.403	8.2639	7.8224	7.7926	5.307	9.0944	7.0881	8.8969	8.3619	3.2463	5.22	8.7433	8.8168	9.4706	6.8864	9.2814	9.799	9.8409	5.9354	8.5608	8.5578	7.5349	8.0326	10.4983	9.6696	11.7299	11.5732	8.9261	9.9166	11.7562	9.853	6.3168	10.0507	7.885	6.7168
TCGA-91-6835-01	79	0	3.19	6.7058	8.9835	9.004	9.8578	6.6995	5.6369	7.8306	8.951	8.4192	7.6672	9.3597	5.214	10.0146	7.605	7.3171	9.9323	8.0131	5.6237	8.4115	7.8867	9.3021	5.9635	8.67	8.6409	9.6746	5.5282	8.1035	8.7808	8.453	8.5396	10.3265	9.6505	9.64	10.6172	9.8466	9.6843	10.0482	9.8114	7.8104	10.2414	7.7811	7.5745
TCGA-91-6836-01	417	0	4.5544	6.0697	8.3196	9.7195	8.7651	2.6538	2.549	4.7563	7.015	9.1527	8.6651	6.4285	2.0313	8.6604	6.6851	10.1007	6.6601	8.2121	5.1815	6.9395	7.2591	9.4063	6.8921	8.2606	9.9887	10.5921	5.5095	8.8433	10.5519	8.4159	7.6274	10.606	7.548	13.9865	11.0248	9.5482	6.6547	12.5657	8.7205	9.4259	7.5817	8.1417	4.5544
TCGA-91-6840-01	372	0	2.2692	4.9802	8.607	7.6469	9.7453	5.5732	4.0252	6.4982	8.7825	9.1281	8.5698	6.4231	6.4359	10.4583	9.0922	9.7466	8.3843	5.6845	6.0763	10.5843	8.3509	9.0069	6.9808	10.3644	10.3289	9.3402	4.7537	7.9575	8.2604	7.6247	8.1483	10.8135	9.0091	8.3543	11.2486	10.1415	8.2568	9.7298	9.8228	5.9212	9.6276	8.1866	7.6022
TCGA-91-6847-01	842	0	3.035	5.5094	8.4076	8.2574	10.366	2.9734	1.4402	3.6998	7.7481	8.7005	9.0076	3.2055	0.7531	8.5996	6.7447	7.724	5.1681	9.8175	5.6926	8.1691	7.6932	9.7041	8.0398	9.2847	9.5014	9.8875	7.602	8.0842	9.2743	8.2148	8.6047	11.1263	5.7116	10.9822	10.551	4.1807	9.0779	10.5971	8.3603	5.8973	9.0352	7.2845	5.1121
TCGA-91-6848-01	224	0	2.7787	8.0165	7.6936	8.0714	9.4741	6.6889	5.9573	6.9939	8.4516	8.5681	6.7463	8.8321	3.7615	9.9143	5.6794	7.1467	8.9145	4.9218	5.7708	8.6754	6.554	8.8268	6.3617	9.0665	10.1737	10.0321	9.2202	8.9996	9.6986	7.3894	8.7047	9.9635	8.9516	9.0977	10.3123	8.6486	10.0665	11.2371	8.7305	5.3936	10.0764	7.8112	6.5315
TCGA-91-6849-01	35	0	2.1167	5.7653	9.781	10.6761	9.254	8.1587	2.7141	9.3056	7.9078	8.4011	9.387	8.3626	3.9505	8.6491	7.8182	7.0713	9.9656	8.8257	4.7916	6.4917	9.5543	7.3225	3.9505	8.815	10.1464	10.2489	5.674	8.2192	7.5278	8.6885	6.5442	9.757	8.0172	12.408	9.4388	7.0949	10.0678	7.41	10.3895	9.0929	10.0021	7.7749	5.7355
TCGA-91-7771-01	492	0	4.1813	6.6664	8.7487	9.2837	9.8749	6.4094	5.0444	11.579	10.351	8.0561	7.9299	8.6036	8.7771	9.0946	7.5967	9.0642	9.5532	6.5655	4.8	8.8069	7.11	8.4588	6.035	8.686	8.9252	9.2745	5.4166	8.0279	8.696	7.7804	7.11	10.1755	8.6719	8.6163	11.6057	9.9515	10.7423	8.877	10.4605	8.6638	8.8593	7.8174	9.0015
TCGA-91-8496-01	505	0	1.3498	3.3636	10.4564	9.2016	11.792	8.029	7.2258	4.8127	9.0683	8.9605	7.99	7.9856	5.2544	10.4785	7.1173	5.5688	10.5863	5.2834	3.8243	6.8435	9.5726	7.9723	4.8519	9.7851	9.8471	10.651	5.0672	9.4787	7.7464	8.756	7.1803	9.1844	8.9627	8.6121	10.317	7.1492	10.3863	7.7202	9.3493	9.0932	9.474	8.9172	6.4032
TCGA-91-8497-01	434	1	4.3825	6.0984	8.9622	9.0939	10.369	6.7222	4.2879	6.7842	9.1597	8.1276	8.2008	8.5558	4.9442	9.8389	8.9812	9.3049	10.4461	8.4936	6.3297	8.3162	8.8088	8.2169	3.96	8.4722	8.8467	9.7694	5.4162	8.8024	7.6408	9.0459	6.6949	9.4097	9.4097	7.2782	9.3229	10.1947	9.4782	7.163	9.6509	8.8632	10.274	8.6562	8.2643
TCGA-91-8499-01	36	0	2.5175	7.707	8.1968	8.1705	9.4325	6.5232	3.2127	7.6487	8.7028	8.9646	9.0116	8.382	8.9833	8.7734	8.7869	9.6781	8.8875	9.4239	4.5873	4.69	9.2667	9.7038	9.711	9.7746	11.532	10.6667	7.8725	10.0772	9.8797	9.1798	9.9295	14.5944	11.213	9.4667	10.0725	8.6994	10.3953	10.5798	8.8118	5.821	9.0198	7.9365	5.9228
TCGA-91-A4BC-01	44	0	3.7444	7.5166	8.0972	9.6308	10.014	7.1161	5.932	8.5204	8.782	8.6075	8.6338	8.0483	7.4373	9.4688	8.8431	7.7357	10.1639	4.7607	5.9924	7.8047	7.3244	8.6479	8.0903	9.391	10.0343	10.8757	6.8966	8.4195	8.3748	8.8695	8.0069	9.7882	8.2473	7.3819	10.0785	9.2215	8.9306	9.8394	10.5642	6.9045	9.9126	8.0972	7.4319
TCGA-91-A4BD-01	603	0	2.4285	3.4119	8.9779	11.1831	9.8561	3.7305	1.86	2.8353	6.8991	8.518	9.3162	4.8164	4.6751	9.6112	8.2243	5.0634	9.6193	6.5263	5.3381	5.8792	8.7174	7.6502	4.8606	9.3241	10.4676	10.3782	5.6211	9.3437	7.6752	8.5821	6.456	9.4122	9.3379	7.5792	10.247	6.0981	9.871	8.0776	9.5434	9.8138	10.2037	8.4901	7.1601
TCGA-93-7347-01	683	0	2.1688	5.6312	9.3369	9.532	10.173	6.9055	2.5013	5.6084	8.4783	8.4232	8.8952	8.5103	5.8216	9.8187	7.2269	6.4406	9.8163	6.5975	5.7712	7.8923	8.7699	8.0424	4.5082	8.2591	9.2299	9.9113	5.5735	8.5691	8.137	7.477	6.9378	9.8482	9.8038	8.8054	9.1414	10.3552	9.6998	8.8278	9.2631	10.0916	10.1875	7.4989	7.0265
TCGA-93-7348-01	531	0	3.957	7.4811	9.7955	9.2941	9.3345	7.2934	2.4914	5.9331	8.5719	8.5166	8.4555	7.0491	5.4131	8.7646	8.3069	8.6219	9.1804	9.24	6.0091	9.187	8.2642	8.2518	5.0884	8.7088	9.7486	10.0762	4.4682	8.8803	7.7613	8.3279	6.5565	9.8939	8.1581	13.233	9.9118	9.4629	8.8099	9.1184	9.0642	9.8848	10.8587	7.8257	7.9427
TCGA-93-8067-01	186	0	1.582	5.9398	9.6703	10.4993	9.2977	5.7236	4.5547	10.734	7.3864	8.6317	8.6594	5.5326	3.2972	9.1537	7.3818	10.2699	7.6343	7.8149	5.6104	8.6034	8.0547	8.5779	6.666	8.8995	10.3533	9.502	4.2653	9.1208	8.8208	7.5689	7.5336	10.1535	7.5987	10.575	10.4833	10.8127	10.2972	10.3157	9.7012	10.3514	9.6407	7.3965	6.1741
TCGA-93-A4JN-01	718	0	3.9195	7.1274	8.8385	8.8306	9.657	6.8177	5.0263	6.9785	9.1887	8.218	8.0081	7.3966	4.4991	9.7518	8.4889	6.1119	8.8214	6.0408	5.7267	9.3253	7.1945	8.9262	7.7652	9.3676	9.476	9.2095	5.7491	8.1158	8.4083	8.7339	8.4789	10.0634	8.2499	12.5741	10.3638	9.0307	8.8798	10.5222	8.7269	8.8372	9.9216	7.3496	7.5864
TCGA-93-A4JO-01	33	1	1.854	6.2929	8.4112	8.885	9.0844	5.0171	3.4512	4.2313	8.2997	8.3245	8.2766	6.9286	5.2129	9.8038	8.994	5.9218	10.8368	6.6188	6.5318	8.1008	7.0757	7.683	5.292	9.2385	10.143	10.4869	6.7225	8.0787	8.0675	9.0634	7.5684	9.236	9.284	13.5916	8.8945	9.1023	8.6232	7.8944	8.9212	7.9775	10.1222	8.4354	8.739
TCGA-93-A4JP-01	578	0	2.7147	5.47	8.9351	7.9716	9.8992	5.6406	5.9865	5.57	10.5658	7.8649	7.9014	6.9841	5.087	9.8504	8.5246	8.279	9.5535	9.4815	5.9027	9.5164	7.9251	8.9595	6.4987	8.8899	8.879	9.027	5.1204	8.523	8.2511	8.4163	6.8008	10.373	9.1502	9.9894	9.4572	10.163	9.2996	8.8231	9.6458	6.3165	10.3881	7.5097	7.6647
TCGA-93-A4JQ-01	526	0	2.9143	7.7039	8.5791	8.3801	9.4005	5.6761	5.1113	5.1113	8.5984	8.1908	8.4192	7.5713	6.1446	9.3474	8.5117	8.9611	10.5027	8.6051	5.9549	8.3914	7.2	7.7514	6.9122	8.2519	10.0148	10.055	5.3612	8.6837	8.8332	8.1972	7.3463	9.0385	9.0373	9.4417	10.7727	9.1412	8.8686	9.8691	9.5269	8.2906	10.0767	7.6296	7.7367
TCGA-95-7039-01	1272	0	4	6.7083	9.0271	9.3096	10.087	5.4547	2.4021	6.2479	8.3392	8.5185	4.9206	4.7395	4.4873	9.6699	6.0444	8.0475	7.6147	5.0506	5.1983	7.6356	8.6955	7.7357	6.8931	9.3481	9.3465	10.6885	6.1926	8.9269	8.9769	8.016	7.6094	10.2136	7.7785	11.5778	10.8019	11.4819	9.5682	10.624	8.4166	9.6144	8.9417	8.2874	5.141
TCGA-95-7043-01	503	1	1.3605	6.5101	8.2971	9.963	10.311	6.2674	2.6976	5.0504	7.7406	8.7074	7.825	4.8688	7.0772	8.998	7.8099	8.2533	8.8576	3.9903	5.3841	8.0205	7.4449	8.2347	6.955	8.8539	10.7243	11.0593	7.3088	9.0963	8.4504	8.5291	8.4472	9.3685	6.1921	5.7629	8.4537	9.9309	8.5075	10.6884	8.7605	6.4723	11.1179	7.8931	5.1808
TCGA-95-7562-01	87	1	2.0503	7.1261	9.2781	9.0697	11.491	6.6401	2.0503	4.6575	7.1536	8.7485	7.7245	6.7283	7.6652	8.6423	5.7418	5.1522	7.3959	4.1381	4.6316	6.7645	8.2689	9.2781	9.0515	9.1528	9.947	10.4156	6.1038	9.5239	9.601	7.2246	8.1757	11.4051	8.378	10.0131	12.2234	5.2405	10.1719	11.0498	8.5537	9.8633	10.3342	7.7122	6.9798
TCGA-95-7567-01	568	0	2.7881	5.9154	8.5128	9.5319	9.6497	5.1987	2.6655	4.2266	7.2133	8.706	8.8492	6.723	3.8859	9.3603	6.841	6.6602	8.5327	3.0058	5.1876	6.2392	7.9092	9.7968	7.0016	9.1428	10.5012	10.0844	6.8656	8.427	9.476	8.7214	8.3104	9.3473	7.4194	5.4881	10.7487	10.4993	9.5269	9.6603	9.7455	9.2067	9.3088	7.3111	5.7742
TCGA-95-7944-01	377	0	3.3946	6.9625	8.4745	9.252	8.8717	8.0829	1.3011	9.972	6.5184	7.7367	10.0226	6.8976	3.6682	9.5077	6.8249	5.547	8.8979	2.5257	5.8875	4.8305	8.2024	8.286	5.6868	9.6453	9.4217	10.9108	5.6132	8.9367	8.2431	7.7915	7.6394	9.8741	12.915	7.363	10.7697	5.2044	10.4083	10.0958	8.9833	8.5398	8.9474	8.0007	6.8887
TCGA-95-7947-01	477	0	2.7006	6.2685	9.5845	8.0534	9.8168	6.7946	2.8909	4.5984	7.871	8.8076	8.3221	8.1697	6.6911	8.4321	6.5531	9.1085	9.2506	9.2892	6.9806	10.6459	8.4398	8.8917	7.1043	8.3303	9.759	9.7827	4.179	9.171	8.7997	8.2487	7.6145	10.9852	9.0622	9.1061	10.2859	5.1005	10.1509	9.6224	8.8184	9.5913	9.8382	7.8118	7.4974
TCGA-95-7948-01	476	0	3.1529	5.8057	9.1374	8.8868	9.6302	7.9582	3.8235	3.7896	6.7021	7.6248	8.2725	5.3737	4.4842	8.603	9.1196	4.6255	6.8839	5.6347	6.446	9.651	9.6917	8.6534	4.526	9.9346	10.2117	10.5805	5.8392	8.7032	9.3868	8.6917	7.3989	9.7347	7.3705	12.1394	9.3013	8.0033	8.0162	8.6332	6.814	8.6917	9.3118	9.4321	6.6631
TCGA-95-8039-01	830	0	3.5595	7.2558	9.5613	10.21	9.6063	5.8395	3.6944	4.1664	8.3857	8.9547	9.4529	7.7679	5.0605	9.3382	7.5179	8.8526	9.3788	5.562	5.7285	8.3885	7.911	8.3532	5.8001	9.0771	9.9689	9.3165	5.4772	8.9592	8.447	7.6288	7.0943	10.1593	9.2284	8.8271	10.8646	6.236	9.4505	9.5112	9.9783	9.4912	10.5859	7.6757	8.1819
TCGA-95-8494-01	84	0	0	5.1974	9.2382	9.6477	9.1292	3.9689	4.6925	3.4851	7.3867	8.8331	8.2253	6.0022	4.8281	9.394	8.4391	6.9909	8.827	3.781	5.3629	5.4284	8.8471	8.4285	7.7351	8.014	10.5409	11.6461	6.6591	8.863	9.2773	8.1529	6.6223	9.9746	8.8451	7.4031	11.6801	7.4564	10.0456	10.9834	7.9335	8.9677	8.6461	8.2527	6.2758
TCGA-95-A4VK-01	651	0	2.7775	5.9429	9.6215	10.4684	9.3205	5.3753	2.5559	4.4902	7.8706	9.7368	9.4459	6.3142	4.3244	8.5426	9.5043	5.836	10.5894	7.9652	4.8243	7.8631	8.5576	8.3073	5.5657	8.3956	8.8554	9.809	4.0964	8.4123	7.6377	8.4185	7.0475	9.3819	6.8296	8.058	10.1419	12.4009	10.9298	9.4039	9.6215	9.6198	9.7789	8.0205	7.6019
TCGA-95-A4VN-01	553	0	1.5123	6.3339	8.8265	10.4371	9.5736	5.5067	2.3698	5.5643	8.324	8.7401	8.1961	7.552	4.7762	9.0685	7.2697	7.7789	9.7454	4.6752	5.6731	7.406	8.3288	8.4522	6.8792	9.2441	10.1624	9.93	7.0351	8.611	8.9136	8.0524	7.6911	9.9712	9.0535	9.7609	11.7441	9.087	9.3055	11.4022	9.0932	8.937	9.0535	7.3249	6.8506
TCGA-95-A4VP-01	605	0	4.557	8.5067	9.1028	8.9567	11.156	6.6524	4.9485	6.2669	8.679	8.8061	8.3671	4.7093	5.6059	9.0764	11.0793	9.8053	9.8263	6.9067	6.7041	7.8294	7.5676	7.5231	6.1546	8.5067	10.787	10.5423	6.2669	8.0537	7.0732	8.513	6.5186	9.5768	7.1391	9.5936	9.4636	9.9606	8.9445	9.2591	8.5212	7.1283	10.488	8.8312	7.9717
TCGA-97-7546-01	1285	0	4.3066	6.5531	8.743	9.299	9.0855	7.1167	5.3271	6.7824	9.6407	7.9134	7.68	8.2809	4.4853	8.5715	6.676	7.5003	8.1518	7.0671	5.5363	8.4984	8.9866	9.4039	5.5524	8.8707	9.7863	9.2725	6.1903	8.5735	8.1518	8.165	7.2807	10.459	8.7182	10.3691	8.9398	7.4624	9.7021	7.7481	9.2492	9.2342	9.4444	8.2038	6.6081
TCGA-97-7547-01	1965	0	2.6444	4.8802	9.9332	9.6115	9.3338	6.1234	2.4267	5.5129	9.8087	8.5617	8.657	7.499	4.7454	9.359	6.8374	9.5173	10.5919	10.164	5.2052	9.3667	8.3163	8.3669	1.4598	7.8172	8.9453	9.6954	4.8366	9.4275	8.2432	7.2969	6.1052	9.8532	8.1827	8.7713	8.3746	9.2951	9.1246	7.1972	8.0351	11.1507	9.4691	7.0714	8.7885
TCGA-97-7552-01	1932	0	1.9223	3.7608	9.913	8.6555	9.5385	5.3073	4.3598	6.0568	6.9944	8.1484	7.1297	6.9446	4.6274	9.3794	6.9241	9.8957	9.3884	9.2096	4.8761	9.2619	8.0455	8.6116	3.9892	7.8788	8.8085	9.3333	4.8297	7.9619	8.0506	7.6374	7.0306	10.0847	9.1821	8.4674	8.2577	7.1488	10.1092	8.8729	9.4739	9.8635	9.4824	7.9965	11.1416
TCGA-97-7553-01	1870	0	1.8792	7.1686	9.4589	8.8496	10.135	5.3162	5.5947	6.9825	9.6119	8.1568	8.8313	8.9701	5.7583	10.4724	7.0666	7.3174	9.9029	7.2513	5.608	8.0071	8.4462	9.1933	6.0963	8.5615	8.9714	9.5681	5.6342	7.826	7.826	7.7046	8.5077	10.0722	9.6108	12.6345	9.9062	8.6604	9.5216	9.7271	9.9512	7.6447	9.6458	7.6608	7.5275
TCGA-97-7554-01	775	0	3.0881	8.0961	9.2498	9.7464	9.5715	6.9837	3.9549	6.4764	8.2337	8.6301	7.5437	7.9607	4.393	9.4447	5.5399	9.4259	8.1631	5.3045	5.393	7.8241	7.2628	8.5572	6.6589	8.6925	9.3316	9.7447	5.4436	8.1505	9.3215	7.9255	8.8955	10.3692	8.8145	8.7489	8.5105	6.6228	9.2367	9.393	8.6319	9.7829	10.6287	7.7145	6.4186
TCGA-97-7937-01	564	0	3.1756	6.8618	9.0567	8.8884	9.4727	4.9604	1.9307	4.9789	6.5141	8.7227	2.6376	5.5852	5.7011	9.2002	7.6942	10.234	8.5213	3.7925	5.8889	9.0697	7.5988	7.8201	7.683	9.1007	9.4841	10.3924	6.726	9.3133	9.6029	8.0164	7.7329	9.5505	8.4501	12.4928	9.6324	9.2894	9.5153	8.5709	9.9765	8.6667	9.3269	7.2787	7.0309
TCGA-97-7938-01	18	1	2.0076	6.235	8.8762	9.2628	8.7216	6.9217	6.6965	6.8096	9.5521	8.3517	8.6278	6.8927	3.4947	8.7257	6.6452	9.5766	7.6683	6.2805	5.7269	8.6647	8.6078	8.3808	5.7269	8.5274	9.1795	9.3562	5.8828	7.8455	8.238	7.698	7.7836	9.6037	8.0567	12.8898	9.7333	9.9293	9.6554	7.664	10.6	9.7697	8.8293	7.0783	5.9407
TCGA-97-7941-01	484	0	2.8371	5.4707	9.7922	7.5959	9.6089	6.297	3.1837	5.0595	8.6905	8.1048	8.967	6.9793	4.6213	9.371	8.5946	6.8639	10.5237	9.4783	5.9115	9.0415	8.2453	8.222	4.4533	8.9819	9.879	9.6874	3.9291	7.6316	7.1101	8.2777	6.6665	9.9311	8.8434	7.8354	9.9543	6.4596	8.7833	7.9587	8.3245	9.6869	9.7985	8.7822	11.0024
TCGA-97-8171-01	568	0	2.2176	4.1805	9.6868	9.162	10.97	6.3506	2.2176	4.3087	6.6085	7.9036	8.5715	5.0329	3.4739	7.6407	9.0865	7.5463	11.4118	2.7862	4.246	7.9567	8.6543	8.4939	6.8529	11.1537	10.5129	10.4095	6.5669	8.4493	9.1627	9.3256	7.78	9.4862	7.2328	3.8279	9.9511	11.2913	9.147	9.4108	8.9981	7.9354	8.5853	7.9906	7.3038

TCGA-97-8172-01	545	0	5.9755	5.9198	8.9729	9.0959	9.7715	6.8092	3.4317	6.7712	10.1807	7.9082	8.0845	8.7248	4.5703	9.4997	9.1104	7.0701	10.2099	7.4456	5.7024	9.0155	8.3332	8.5361	5.1671	8.9257	9.0505	9.9449	4.5863	7.8348	8.2391	8.2566	6.9142	9.4167	9.5393	7.5015	8.3729	7.2958	8.9721	7.1609	9.0945	9.014	9.7163	7.8348	9.0952
TCGA-97-8174-01	164	1	4.3913	6.0358	9.1244	10.9441	9.1915	7.0429	4.7843	7.3566	9.5435	7.8239	6.8372	7.9791	4.3913	8.6929	9.8313	8.2765	10.2127	7.0157	6.1006	8.8688	8.4716	8.6621	4.2323	8.7719	9.118	9.4426	4.6333	8.0283	7.0065	8.2599	7.0003	9.5053	8.988	7.2517	8.5932	7.9822	8.6145	7.158	10.1231	9.0451	9.5471	7.9069	7.2875
TCGA-97-8175-01	551	0	2.9921	5.9017	9.162	8.342	9.242	5.5519	2.3713	3.5154	8.2348	8.9327	8.9099	7.4009	4.6638	9.6649	6.9557	7.1834	9.2429	3.5154	5.0137	6.6507	8.796	7.8856	6.27	8.9088	10.1988	10.5731	6.7705	7.7499	8.0384	7.6586	7.5529	10.1812	9.0929	9.8262	11.603	7.6385	9.8858	9.9592	9.332	10.6989	9.4472	8.1522	5.3204
TCGA-97-8176-01	468	1	1.9903	6.8401	8.8933	10.3564	9.3789	4.8553	4.1893	6.631	7.895	8.6113	6.6223	6.2909	3.2211	8.8678	7.6324	8.0499	10.2004	7.8891	5.8747	7.2982	8.7392	8.537	5.9736	9.4964	10.3485	10.3629	5.0492	8.9041	8.3964	8.1916	7.5974	10.0311	7.6999	9.3737	9.566	11.5594	10.6244	10.8016	8.5802	9.6739	9.9597	7.7763	8.0596
TCGA-97-8177-01	499	0	4.2105	8.1196	9.4012	9.8199	9.5853	7.1924	4.0099	5.3024	8.6112	9.0363	9.2195	7.6024	5.6763	10.1157	8.1976	8.5781	9.7748	5.3396	5.2117	7.3404	9.258	8.0689	5.933	9.5434	9.9781	10.3387	5.5864	9.4865	7.9755	8.4437	7.5049	10.2645	9.7877	10.6285	10.0278	9.5152	9.3648	9.2677	9.2572	8.8842	10.3085	7.7269	8.4565
TCGA-97-8179-01	435	0	2.3974	7.3945	9.8909	10.5767	9.5424	5.81	6.9588	6.0401	9.0716	8.1112	6.3593	5.4101	3.8661	9.5838	8.1957	6.586	9.2975	5.7279	5.9065	8.4901	7.8907	8.0492	6.5387	9.5588	9.8701	10.1159	5.7384	9.0229	8.1092	9.4716	8.3323	9.6825	7.7651	7.3263	10.4875	11.2034	9.1297	8.8758	9.4684	8.5893	9.4605	7.7625	5.9969
TCGA-97-8547-01	657	0	5.1794	6.9669	9.0796	9.885	9.6712	3.9593	4.9121	4.8368	8.2631	8.1751	8.1865	6.7272	5.2096	9.8044	8.3747	5.7915	10.4118	3.454	7.0774	6.8663	8.8402	8.1674	5.888	8.7837	9.8253	10.1462	6.7581	8.378	8.4744	7.9611	6.7581	9.9941	8.9016	9.5288	11.0197	10.1636	8.31	9.5511	9.3825	9.6341	10.2194	7.2102	6.6523
TCGA-97-8552-01	626	0	2.5553	4.8904	9.7947	9.1911	9.5573	5.7884	4.27	4.7985	9.0181	8.1861	8.3939	7.5839	4.7666	9.9968	9.6985	7.907	10.9109	7.968	5.6896	7.9855	9.1669	8.1463	3.6537	8.6821	9.5002	10.4089	5.3661	9.3465	7.1232	9.4683	6.3593	9.7778	8.9186	7.6423	9.0834	9.9424	9.0288	7.2672	8.9403	8.2937	9.7185	9.4645	8.9528
TCGA-97-A4LX-01	614	0	3.5847	6.8168	9.6928	10.0108	10.218	7.0261	3.1568	5.3274	8.4132	8.1059	9.5637	8.7301	5.9474	10.3429	8.5856	8.2146	10.4227	6.1124	5.2627	7.1019	8.5179	8.1668	5.5469	8.5266	9.9016	9.8183	5.9979	7.8928	9.2657	8.149	7.4199	10.4502	9.8585	7.251	10.5382	10.4794	8.6407	9.7246	10.0927	8.3704	10.0869	7.8902	7.3438
TCGA-97-A4M0-01	652	0	3.4033	5.2634	8.6962	10.3222	9.6427	4.8494	2.4756	3.7838	7.042	8.4531	9.6402	7.3115	5.2287	9.4042	9.3294	8.6706	10.1711	5.4556	6.227	6.6038	8.7674	8.5102	4.5456	8.364	10.0521	9.9946	4.68	8.4285	7.9477	8.5598	7.1815	10.339	9.2419	8.7167	10.1909	5.9678	9.3212	7.8683	9.2527	9.6656	9.8075	8.4902	7.7418
TCGA-97-A4M1-01	601	0	5.8646	4.4302	9.4592	9.1217	9.699	5.9123	5.6281	4.0586	9.3544	8.0056	8.8102	6.6975	5.0995	9.9665	9.3014	6.2458	9.8351	6.9639	6.3534	8.5335	9.1292	8.2617	5.16	8.9509	9.4339	9.1051	5.7381	8.6042	7.0085	9.339	7.2174	9.5383	8.9495	8.0138	9.3422	9.5767	9.8164	6.9752	9.0727	6.5621	10.0144	8.3809	8.8612
TCGA-97-A4M2-01	624	0	2.8602	4.2707	9.607	10.1028	10.557	6.0965	4.8547	6.3813	9.8078	8.0652	8.0652	8.0521	6.1851	10.5886	10.3421	7.18	11.4511	7.6674	5.9692	8.1532	8.1311	7.3642	3.5943	8.7743	9.965	9.725	6.2865	8.1261	7.7728	8.8545	6.8352	9.4309	9.2567	8.4969	9.5773	9.3211	9.1665	7.0549	9.3994	7.0286	9.9104	7.9824	8.7077
TCGA-97-A4M3-01	540	0	5.5709	8.4429	9.2139	10.7573	9.0011	6.1354	3.9225	6.4259	8.5085	9.0082	8.8029	5.3738	3.337	8.1356	7.8304	8.184	7.7343	4.8741	5.4757	9.4175	8.3136	7.8527	4.145	8.9781	9.4377	9.8901	5.3561	8.5165	8.4513	8.5304	6.2926	8.9444	7.1251	9.7171	10.6469	5.4257	9.9213	8.1014	11.1414	8.7171	9.361	7.4964	7.0222
TCGA-97-A4M5-01	634	0	3.0213	6.1904	9.6423	9.9775	9.5422	6.4404	4.6604	9.4927	9.5094	7.9907	8.3186	6.8908	4.6604	9.9775	8.8778	8.5981	10.0539	8.286	5.6437	8.5278	7.7676	7.9448	6.0875	8.6335	9.0347	9.2203	5.055	7.9643	8.0025	8.8041	6.689	9.5615	8.8454	9.4063	9.6248	10.7312	9.3785	8.2587	10.6628	8.4418	9.7744	7.7311	8.3242
TCGA-97-A4M6-01	568	0	4.2687	6.6785	9.5148	9.9555	9.586	4.9614	4.6821	4.2341	8.8331	8.2605	8.2012	6.4105	5.4577	9.8365	8.9878	9.5748	10.9521	7.3383	6.0616	7.8071	8.1206	7.9038	4.3355	8.6856	9.2177	9.3151	5.2654	8.8331	7.3423	9.011	6.7537	9.9772	8.8588	8.348	9.0375	11.1978	8.9456	8.9443	9.1037	9.183	10.2204	8.5741	8.1922
TCGA-97-A4M7-01	629	0	3.5	5.7161	9.1806	10.4545	9.0298	6.4911	5.4156	6.0883	9.1377	8.286	8.626	8.3038	5.1448	10.1008	8.8099	7.7073	10.9656	6.8707	5.3102	9.2916	7.577	8.1087	6.4349	8.0952	9.5916	8.6369	6.0607	8.3077	7.6773	8.7778	7.2867	9.9347	9.7958	9.4674	10.2022	11.4965	8.7792	8.7749	9.7199	9.4266	11.1889	8.0279	11.2792
TCGA-99-7458-01	747	0	3.1611	6.7715	9.3838	10.206	9.249	6.913	4.7274	6.2254	9.0107	7.8272	8.59	8.5344	5.0778	9.4645	6.905	7.6291	8.9423	6.6531	5.8729	8.537	9.0354	8.4413	4.493	8.3314	9.0517	9.2109	6.1534	7.9712	9.2732	8.0032	7.2939	10.3273	8.9645	9.386	10.2189	8.6696	9.6453	8.7146	9.5247	9.963	9.8496	6.9327	6.7135
TCGA-99-8025-01	1060	0	3.5021	7.423	9.0896	10.2561	8.8994	6.4146	3.2726	7.1219	8.2275	8.3861	8.4967	5.7365	4.1695	9.0834	7.7677	7.4535	7.9958	7.1115	6.5126	7.1594	8.5932	8.2363	6.2411	8.8554	9.9455	10.943	6.7365	9.7331	8.7466	9.0584	6.9144	10.4362	7.4806	7.0834	10.4449	6.7852	10.1159	9.2957	9.2888	8.9322	8.3018	8.0457	7.4258
TCGA-99-8028-01	1118	0	4.723	8.295	8.5714	8.6164	9.8927	6.5746	3.4469	3.4469	8.5491	8.7837	7.3931	7.6436	5.6466	9.9937	7.6865	8.2199	10.2483	6.5283	5.5702	6.8738	7.9802	7.7044	5.0943	8.4488	9.7926	10.2686	5.5304	8.4146	8.0685	8.1319	7.3173	10.2951	9.3755	11.5367	10.1034	9.0945	9.474	9.1502	9.6413	7.9678	9.6138	8.2032	7.4511
TCGA-99-8032-01	44	0	3.3767	8.699	8.5654	8.8436	10.958	7.2624	6.7201	9.2161	8.8191	8.0689	8.0563	7.5722	6.1814	9.0736	9.8688	10.2342	9.1341	8.4779	5.8112	6.952	6.6656	8.4247	5.4981	8.9049	9.8278	10.111	6.5973	8.3677	8.6408	8.2485	6.7881	9.6788	8.618	12.4655	9.685	8.481	9.8518	9.4185	9.1678	7.7478	9.9612	8.0563	6.8772
TCGA-99-8033-01	656	1	2.6935	6.8754	9.3748	9.8778	8.3049	5.8876	1.119	9.7818	8.3986	7.9489	9.0184	6.4091	3.4798	8.6753	8.1065	7.3737	8.3173	6.4289	6.164	6.2027	7.6398	8.3401	6.7396	9.1894	10.0273	9.989	6.6357	9.3203	8.7053	7.6055	8.1626	9.9633	8.7635	10.4647	9.8838	10.5305	9.6852	10.4682	9.2719	10.5825	8.4278	7.5554	6.1718
TCGA-99-AA5R-01	658	0	3.5002	5.803	8.9096	8.5468	9.881	5.6554	6.2256	6.3364	10.0183	8.3922	8.0549	7.1513	4.573	9.6597	9.0814	7.5189	10.6358	8.3229	6.1504	9.4662	8.1013	8.0666	4.8423	8.0281	9.5459	9.6382	5.4196	8.3781	7.0778	8.5901	7.1291	9.0314	9.1325	10.5264	9.4979	9.3877	8.4267	8.179	8.9593	6.79	9.989	7.9223	7.9855
TCGA-J2-8192-01	739	0	1.5246	7.3099	9.7046	10.3147	9.0831	7.089	6.9987	6.8933	9.2621	8.5917	8.0514	8.3755	5.7531	9.77	7.9499	9.4215	9.4743	8.2442	5.2823	8.8739	9.1009	9.0446	5.0404	7.7631	8.7931	9.1418	5.0239	8.7205	8.2864	7.8194	6.8471	10.4193	9.8827	11.7516	10.2877	9.4597	9.2055	8.6841	9.9004	10.054	10.3583	7.0364	7.4278
TCGA-J2-8194-01	724	0	4.7167	6.5529	9.5844	10.216	8.6361	7.1747	4.8094	7.5851	8.2232	8.2254	8.2852	6.9891	3.2151	8.644	7.6492	10.0306	9.4559	8.0395	5.521	7.627	8.7206	8.1072	4.304	9.388	9.3393	8.9273	5.7834	8.0226	8.6612	8.3092	7.4186	9.639	8.3789	11.6518	10.2817	10.109	9.9367	7.5486	9.6532	10.3279	9.6986	7.6863	6.3049
TCGA-J2-A4AD-01	550	1	2.8258	6.7628	9.8292	7.9271	8.7863	5.4338	2.0868	4.8384	9.1635	8.8243	6.1791	4.9373	3.6752	9.9484	9.597	9.3546	7.9486	8.4685	6.6795	8.0293	9.144	7.6493	5.8233	8.7982	10.1454	10.2356	7.29	8.1803	9.073	8.7267	7.8707	10.218	7.4923	10.0195	11.2778	10.4609	9.4498	10.1459	10.2327	8.0864	9.6758	8.0929	6.73
TCGA-J2-A4AE-01	1079	0	3.4656	4.2721	8.7895	9.4158	10.021	4.623	5.6624	2.9071	8.4414	8.1107	8.922	6.4958	4.6906	8.6877	9.0847	6.2265	9.0156	6.9739	6.648	9.1234	8.9429	9.0737	6.7145	9.053	9.5424	9.3217	7.1523	8.2234	8.2291	8.3226	7.7715	10.489	8.2033	10.8057	9.8694	7.5113	9.5974	8.9856	9.0816	8.3723	9.6138	8.526	7.9197
TCGA-J2-A4AG-01	988	0	4.865	7.9612	9.5789	10.79	8.0373	6.4816	2.9549	4.998	8.4186	8.4763	7.781	7.1974	5.0962	8.6481	7.5961	9.1817	9.6734	5.9492	5.6429	7.2729	9.0253	8.2813	2.7286	8.586	9.6503	9.6135	5.8956	10.0707	8.9219	7.9644	11.1274	9.6614	8.7777	12.0987	9.0743	6.2127	9.5237	8.3401	8.997	9.6155	9.4823	8.1243	7.0274
TCGA-L4-A4E5-01	578	0	5.3751	8.1638	9.2197	9.4256	10.121	4.702	4.1195	4.7995	7.1892	7.9441	9.9073	5.7734	3.8275	8.921	8.9061	8.1454	8.1752	3.4609	5.0774	8.863	8.2418	8.5941	7.5791	9.1135	10.3912	10.8586	5.6481	7.8305	8.8431	8.1006	7.9888	10.1134	7.0939	11.9094	11.4257	11.1645	9.4131	10.9383	9.6741	5.7972	9.0992	7.4347	6.5659
TCGA-L4-A4E6-01	435	0	1.9188	5.6582	9.2262	8.7382	10.589	7.0351	4.6709	6.1849	9.6743	8.2695	7.8076	7.6082	6.1849	11.6319	8.6963	7.3326	11.1811	7.3572	6.1626	8.6431	7.7189	7.1915	3.8428	8.3626	9.3183	9.5628	6.082	7.8775	6.9789	8.1304	7.2294	9.0345	9.4377	10.3913	10.2403	7.2611	9.1909	7.5451	9.5022	6.046	10.5028	8.011	7.7379
TCGA-L9-A443-01	193	1	1.929	8.4455	8.9086	10.0675	9.1979	6.4965	6.9276	3.9106	8.8335	8.2937	9.24	4.6523	4.6523	8.6808	9.0095	8.9485	10.2321	8.5764	6.7732	6.9007	7.5459	7.9645	5.2923	8.5224	9.6471	10.2992	5.7033	8.4125	8.2113	8.6448	6.7806	9.8626	8.0586	12.7987	10.5921	7.8602	9.1315	8.9953	10.4871	8.1612	9.1243	8.0737	6.8939
TCGA-L9-A444-01	307	0	3.2253	6.477	8.7785	11.0983	10.022	6.2796	3.8745	4.0828	7.8878	8.2075	8.0959	8.1393	5.5172	9.2863	8.5106	6.7957	9.8355	4.4263	5.2557	7.749	7.9034	7.1051	4.9714	9.2775	10.0949	10.0314	5.5172	8.0429	8.3419	9.7843	11.5295	9.5041	8.7354	5.0725	7.7337	10.2851	8.4555	9.4456	10.1128	7.6324	9.7427	7.7743	6.8442
TCGA-L9-A50W-01	442	1	1.8584	6.3647	9.0639	8.4476	9.7658	4.7884	4.846	4.9194	7.0312	8.3565	10.0802	6.1298	6.5083	9.2399	8.9104	7.9685	10.8892	4.0339	5.7319	8.9372	8.6088	7.9335	5.0395	8.8763	10.2582	11.1132	4.846	8.3365	7.5874	8.1699	6.8175	9.6083	8.8763	9.8719	7.0188	9.7739	9.0405	7.9291	10.0626	9.7917	10.9777	7.3426	8.2836
TCGA-L9-A5IP-01	58	1	4.3421	4.9853	9.1499	10.7331	8.4432	4.5179	5.8016	10.098	7.7496	7.9449	4.816	5.8895	1.8597	8.3061	7.3768	7.4291	9.5345	5.3686	5.1367	5.5948	8.1073	8.0344	5.7902	8.7585	9.688	10.7413	6.7302	8.8269	8.682	8.6111	7.3146	9.1818	9.0048	8.4559	11.3036	6.9039	8.8504	11.0654	8.7541	6.7597	9.394	8.2901	5.868
TCGA-L9-A743-01	664	0	2.3921	7.1563	8.3667	9.8107	9.5127	6.3106	3.6785	5.8135	8.55	8.6317	9.1558	8.545	4.8508	9.2517	7.7725	6.3446	9.937	5.8612	6.0784	7.6319	7.8215	8.3993	5.739	8.8108	9.7487	9.8577	5.2769	8.5062	8.7504	8.3522	7.1515	9.974	8.9795	11.0154	10.2661	9.7069	8.8833	9.8415	9.2595	9.6648	9.5756	8.5614	7.5759
TCGA-L9-A7SV-01	565	0	6.3725	8.1512	8.8946	9.4712	9.3917	3.0298	3.3827	3.3827	8.6436	7.9258	8.9681	5.2891	4.4919	8.7988	10.3482	9.2805	8.1589	4.5159	6.528	8.6577	7.5379	9.2415	7.2682	9.8942	10.1927	10.3652	6.9093	7.8524	8.44	9.6438	9.2234	9.6807	7.2032	4.4175	10.8811	10.9763	8.9374	9.5291	9.7328	6.7716	8.9843	7.5755	6.0905
TCGA-L9-A8F4-01	476	0	4.7287	6.949	8.5294	10.8188	9.5788	6.0407	2.3737	4.9001	7.9511	8.0055	7.3586	7.6595	4.817	9.6031	9.0535	8.6972	9.8506	3.7148	5.392	6.8942	8.8251	8.5261	6.8583	9.3212	10.7716	9.8149	6.8478	8.219	8.4401	8.3131	7.8856	10.387	8.4191	7.6171	9.7013	9.7042	10.0223	10.1674	9.165	4.3686	9.0109	8.6454	7.8907
TCGA-MN-A4N1-01	827	0	5.7148	10.0785	9.6903	9.0743	10.159	4.0333	6.6719	3.2818	7.2656	8.8498	9.0743	3.4001	4.1047	9.468	9.1917	7.1227	7.9579	7.7866	5.6447	7.7509	7.9747	7.4925	6.4431	9.6551	10.5352	10.666	6.1792	7.9089	8.1495	8.399	6.4905	10.1483	7.2463	10.1557	9.169	10.5368	8.0169	10.9637	7.8028	8.6819	9.965	7.9039	7.7729
TCGA-MN-A4N4-01	1175	0	5.4983	8.5125	9.0924	9.0393	9.3144	5.9878	3.0369	4.8752	8.4524	8.7075	9.1569	5.319	3.754	10.2023	7.6501	7.782	9.5536	3.3404	5.8139	5.8504	7.3858	7.5685	6.5281	9.2187	9.7066	10.0426	6.2194	8.8022	8.0797	8.5756	7.7565	9.4254	8.3856	11.6947	10.3413	10.5954	8.7452	7.9114	9.3751	7.6081	9.3252	8.2048	8.9815
TCGA-MN-A4N5-01	84	0	5.3904	3.9964	8.514	9.2704	9.5298	5.4566	3.3853	6.4401	8.7545	8.1798	9.4861	5.9639	4.1642	9.35	8.1013	7.5179	9.0427	4.1956	6.2062	6.697	7.1168	7.4961	6.1751	9.2584	10.1662	10.0531	5.9909	7.6022	9.3861	8.2782	7.3644	9.3483	7.6511	8.9493	11.7294	8.2727	8.767	10.5809	9.1871	9.6242	9.9841	7.6425	6.9384
TCGA-MP-A4SV-01	2620	1	4.4839	6.0534	8.3876	10.4529	9.9145	5.2391	1.834	4.6891	7.2855	8.0991	8.6573	7.1892	7.5234	9.0117	8.8739	9.1441	10.0516	7.1021	5.2227	6.7186	7.8913	8.4769	8.0649	9.4609	10.1937	9.9505	7.4861	8.6248	9.7103	8.1103	8.8765	10.1863	8.0968	8.0276	11.2028	10.0109	9.5284	10.0446	9.9989	9.239	9.1528	7.3901	7.43
TCGA-MP-A4SW-01	1778	1	4.4856	6.5669	9.6168	10.4914	10.118	5.3278	1.4965	3.8284	7.5771	8.4985	9.1403	6.6478	6.9536	9.6894	9.4276	7.9715	9.9313	4.4257	5.3113	8.918	7.9101	7.4988	6.1431	8.5951	10.0086	10.2408	6.6802	9.1918	7.843	7.9425	6.8886	9.1691	8.9817	8.0775	9.9327	12.3279	9.2152	9.6227	8.7296	9.2229	10.19	8.023	11.2399
TCGA-MP-A4SY-01	1501	1	4.7536	6.8052	9.0274	8.8302	9.0547	5.4405	2.2917	5.6786	8.5013	7.9351	6.8383	6.326	4.3563	9.9294	8.2107	7.3708	9.2642	7.6727	5.5512	6.4597	7.7959	8.9321	5.6908	9.224	9.7882	9.7854	8.6707	7.8182	10.0468	8.0337	7.1987	10.2007	8.7337	10.0887	11.9853	5.1726	9.7776	9.9542	9.8155	9.4614	9.5016	7.8778	5.904
TCGA-MP-A4T4-01	2617	1	3.5402	6.8402	8.9112	9.4825	10.597	5.9742	2.8646	4.7342	8.1887	8.3521	8.2618	7.5734	5.1423	9.7997	8.2039	8.3542	9.9976	8.6808	5.3446	6.631	7.566	7.7457	5.8588	8.6876	10.2809	10.2342	7.0121	7.8216	8.3371	8.0281	7.386	9.444	9.1135	7.3776	10.5663	9.1098	8.463	10.3326	8.8292	8.6022	9.4932	7.7554	7.4905
TCGA-MP-A4T6-01	1790	1	7.3368	6.8247	10.0883	9.0543	10.517	6.753	1.8326	2.8523	6.3462	8.5828	8.3405	5.727	6.4966	10.4566	9.8064	7.2318	9.461	6.7725	5.9569	9.5885	7.889	8.0884	6.1176	9.3617	9.6197	10.4745	4.4742	8.9935	7.8957	9.4973	9.9822	9.2977	7.5061	10.5606	8.944	9.127	6.4247	7.6365	6.2622	8.9841	10.6195	8.2411	9.0222
TCGA-MP-A4T7-01	167	1	2.4359	4.4418	9.0032	9.922	9.0793	7.1469	3.0104	7.3121	7.9939	7.7596	10.2783	5.8222	3.7858	9.1583	9.1142	8.0048	10.0325	6.5006	5.6555	7.0826	8.8308	7.3081	5.5777	8.7023	10.122	9.9736	5.3779	8.2249	7.7944	8.5961	6.8921	9.0922	7.5916	9.9029	9.571	8.8026	11.1073	10.0258	9.1649	9.5121	9.4147	8.5288	6.7096
TCGA-MP-A4T8-01	161	1	4.0106	7.9366	9.0491	10.458	10.444	5.3087	4.7113	7.2889	7.9291	8.0018	7.5824	4.5133	2.8677	8.9812	8.5611	7.9684	9.4842	6.0736	5.586	6.8271	7.5472	8.1393	6.2512	9.3374	9.6962	10.4116	7.4361	8.6722	9.5308	8.5005	7.0538	9.8681	7.2174	10.0385	11.7705	9.1018	10.5774	9.8018	9.0353	8.4389	9.605	8.4231	5.586
TCGA-MP-A4T9-01	1265	1	3.2871	6.7189	9.26	8.6047	10.012	5.0029	3.8219	5.0898	8.8634	8.4056	9.4136	6.9221	4.3303	9.9534	8.3309	7.6675	9.4633	7.2993	5.6887	8.1219	8.3696	8.2837	6.2455	8.7358	9.4584	10.1289	5.8862	7.5498	8.3451	8.078	7.0183	9.8059	8.1137	12.501	11	10.2654	8.8323	10.3776	9.4795	9.4001	9.8785	7.5375	6.4166
TCGA-MP-A4TA-01	950	1	4.2614	6.5091	10.2538	10.4469	10.232	5.7088	2.8998	5.5437	7.5694	8.7617	8.3406	6.0714	8.0877	9.2842	8.3004	7.8072	10.2263	5.9912	5.2541	7.4184	8.4939	8.8565	7.5235	9.3037	10.392	10.2765	6.596	8.3987	9.1116	8.4596	8.476	10.0318	8.7856	12.8737	11.0154	10.9812	9.9002	10.55	9.474	9.0102	10.2812	7.765	8.3406
TCGA-MP-A4TC-01	74	1	2.214	7.6589	8.78	9.5636	9.1837	4.7992	1.4956	4.7267	8.1994	8.2085	8.7143	5.8086	4.3936	9.7813	7.3034	7.6782	8.8457	6.9038	6.3411	7.9884	7.8615	8.6634	6.1875	8.5887	9.7739	10.0903	6.4503	7.833	9.1587	7.9885	7.0595	9.9932	8.8641	11.2838	10.8873	6.7839	9.4499	10.0981	9.2882	9.524	10.1858	8.1497	7.0297
TCGA-MP-A4TD-01	307	1	4.6805	8.532	8.6105	10.1105	10.003	8.308	3.6813	7.4098	8.6373	8.4305	8.8527	6.1987	6.3014	9.5338	9.5097	8.6832	10.1233	8.1492	6.4224	8.6693	7.3887	7.594	6.0019	9.0127	10.1066	10.2466	6.6161	8.6123	7.8154	8.9821	6.7738	10.0923	8.3012	8.5529	10.3796	10.9522	9.6113	8.5048	8.8122	8.395	10.4565	7.8248	7.0183
TCGA-MP-A4TE-01	896	1	3.3577	8.4724	9.0727	8.812	10.891	5.3606	1.8652	5.0704	7.4412	8.2399	8.6363	5.5353	7.5563	9.038	8.769	9.276	9.7775	2.3117	5.4212	6.7731	6.7789	7.9028	6.9986	9.2533	10.2871	10.7096	5.8755	9.0715	8.399	8.6379	6.8956	9.7797	5.1796	12.3651	11.6459	9.5907	9.8097	9.4119	10.0547	9.3362	10.264	8.2123	8.2976
TCGA-MP-A4TF-01	336	1	2.5592	5.7111	8.9734	9.6252	8.7511	2.9117	5.2676	5.9771	6.8782	8.6954	7.9407	5.6421	3.7263	8.1948	7.8721	8.8462	7.5277	2.7462	5.6421	6.2487	8.3399	8.0513	7.1755	8.884	10.0649	9.6562	7.3587	7.449	9.5844	8.5141	8.4142	9.2083	7.8467	10.1606	9.1411	6.032	9.5875	10.7709	9.23	7.3871	9.1053	7.9925	5.7554
TCGA-MP-A4TH-01	741	0	3.8456	5.5299	9.0183	10.1881	9.2718	6.7489	3.067	8.535	9.2357	7.8585	7.9978	8.1763	3.9354	9.6453	8.8654	7.3519	10.2889	7.1418	5.5154	7.3064	8.3311	8.1809	4.3486	8.3311	10.0493	9.5285	4.6161	7.9582	7.2198	8.3165	6.2427	9.7549	8.9007	8.4134	7.6851	7.9952	9.6117	7.4579	10.0816	8.9871	9.5937	7.8178	7.2242
TCGA-MP-A4TI-01	429	1	1.6089	7.4062	8.8686	8.3326	9.8662	5.8165	2.1978	4.4936	8.5913	8.7551	6.3662	7.7116	4.5578	10.1265	8.6393	6.4866	10.9542	6.235	5.9894	7.5154	6.6204	7.4785	6.9543	8.9971	9.693	9.8072	7.5113	7.8905	8.402	7.8905	7.9213	9.5797	9.9943	8.8859	11.5654	7.0689	8.5748	10.3554	9.2955	7.9545	9.6623	7.5394	7.6649
TCGA-MP-A4TJ-01	339	1	2.9976	8.039	8.6093	8.8219	9.2582	6.7371	3.283	7.7597	8.0687	8.313	7.9803	8.5667	5.4527	9.3538	8.4562	8.1976	10.5233	8.4345	6.0742	7.7655	7.6943	8.3405	6.1111	8.4418	8.924	9.5862	6.7371	7.8278	8.1317	8.516	7.1098	9.5581	8.3249	11.2136	9.2417	8.768	9.2144	9.7964	9.4104	8.7937	9.7836	8.1496	9.7027
TCGA-MP-A4TK-01	582	1	5.0742	9.2532	9.0725	10.15	9.3527	6.4864	4.941	5.857	8.729	8.5732	8.7513	7.2008	5.5412	10.2343	7.8873	9.4021	9.5381	4.9868	5.8692	8.4642	8.1861	7.5751	6.0846	8.2508	9.9927	10.1387	5.116	7.7938	9.6029	7.9231	7.5487	9.8751	9.1387	10.5304	10.289	8.0417	9.6781	10.4601	9.2284	8.8177	10.2758	7.3263	6.7076
TCGA-MP-A5C7-01	2248	0	2.3645	7.4706	9.5104	9.3296	10.341	7.0413	3.0823	6.7558	7.3063	7.5105	9.0858	5.5817	4.6478	9.0007	10.0328	6.0567	10.5098	3.7495	5.3371	10.0538	9.0296	8.0918	5.6065	9.3099	10.3837	9.6979	4.5743	8.2117	8.1561	9.2629	7.4191	10.2267	7.5558	7.912	8.619	10.4732	9.8773	7.1465	11.1155	7.504	9.9347	7.2641	10.3552
TCGA-NJ-A4YF-01	2161	0	4.3099	6.7376	9.415	9.458	9.8815	4.5155	1.1474	7.5832	5.6673	7.0601	7.2505	5.2034	4.8853	8.717	7.8188	6.6088	7.0402	2.2157	5.2034	5.0531	8.3939	7.9091	4.7935	9.3574	10.9748	10.497	4.4766	8.7045	10.1262	8.9315	6.4373	9.1616	8.1001	6.3963	9.581	11.2918	8.7941	9.3126	8.3886	7.3506	8.9097	7.513	6.3216
TCGA-NJ-A4YG-01	2261	0	4.1954	7.234	9.0353	10.2795	9.4575	4.9005	2.6314	3.435	7.6843	8.254	9.6209	5.7111	5.4504	9.5985	8.807	6.798	10.577	4.3258	6.0115	5.3313	7.6822	7.6741	4.1954	9.5648	10.2339	9.5866	6.518	9.217	8.1196	8.8033	7.1602	8.4737	8.7089	9.8833	9.5201	9.321	8.9999	8.2457	8.8527	8.957	9.4094	8.0678	8.2096
TCGA-NJ-A4YI-01	4	1	2.6715	6.5799	9.287	9.2429	9.9205	5.8596	0	4.402	6.4419	8.786	8.8938	6.0904	5.6049	9.9993	8.8121	8.4599	10.6615	3.1789	5.9719	5.0248	7.6272	7.7312	5.6246	8.6013	10.1813	9.9394	6.408	9.2839	8.0195	8.5376	9.5919	9.1505	8.0082	10.4513	10.2537	9.6168	8.1548	8.3093	8.8712	9.1975	9.5919	7.5621	9.4468
TCGA-NJ-A4YP-01	50	0	3.6653	7.4219	8.7864	8.9043	8.1173	5.474	2.9745	9.5745	8.1225	7.8062	8.3586	5.8238	5.3536	8.9905	7.9028	7.8094	8.2699	5.3446	5.9177	6.5268	8.3155	8.1981	6.0002	9.7426	10.1938	9.5201	7.396	8.9436	9.0211	8.4989	7.6598	9.7452	7.8176	10.1077	9.092	5.1622	9.6657	9.4631	10.031	9.7478	9.1793	7.0315	8.1369
TCGA-NJ-A4YQ-01	1432	0	2.0492	7.4029	8.966	8.1912	10.171	5.4282	1.8119	3.3808	9.3829	8.5121	4.7743	7.8423	4.0059	9.8504	8.0751	9.7873	9.8872	4.9891	5.6061	6.0506	7.8185	7.9957	4.4407	8.3015	10.2068	10.4154	6.2423	9.1668	8.4744	8.6493	6.4533	9.8719	8.6402	7.3428	10.6916	11.1395	10.219	10.4566	8.3523	7.5599	9.0228	7.6884	6.8168
TCGA-NJ-A55A-01	15	0	1.857	5.5105	8.8177	9.04	9.1901	6.3161	5.5912	6.7266	9.9164	8.1554	7.9867	7.9565	4.7198	9.0352	9.1418	7.0553	9.5827	9.1617	5.9987	8.5614	8.9279	7.9666	4.6712	8.9888	9.4875	9.5893	5.425	7.9309	7.7727	9.4805	7.8567	9.4963	9.1215	12.647	8.548	7.1293	8.9045	8.0875	8.9937	8.1598	9.7662	9.1124	8.9562
TCGA-NJ-A55O-01	13	0	1.9698	3.5952	9.8572	9.7836	9.7951	6.2194	5.1702	9.3834	8.1305	8.5189	8.9569	6.5865	3.6632	10.0136	8.3356	8.1377	9.5543	7.3811	5.5574	7.7568	8.6691	8.2799	5.8183	8.7437	9.5499	9.9211	5.2162	8.5004	7.2709	7.8473	6.7827	9.7683	7.9665	9.1981	9.3334	6.3176	10.362	9.6622	9.4487	8.9986	10.0426	8.5166	7.9564
TCGA-NJ-A55R-01	603	0	4.1938	8.4498	10.0725	10.0955	10.005	6.8168	3.5326	6.407	8.2962	8.6216	7.3483	6.6781	5.9605	9.2387	9.4322	9.391	8.6136	10.8762	8.4348	7.7948	7.9709	8.1266	5.665	8.9051	9.8942	9.8952	5.7121	8.7463	8.346	9.0901	7.1006	8.7284	8.1549	11.6558	9.5718	9.8543	9.9266	9.3544	9.031	8.266	9.9404	7.89	9.6451
TCGA-NJ-A7XG-01	617	0	4.0505	2.8897	9.1094	10.8412	9.5738	3.9684	1.906	1.2472	7.3082	8.512	9.5773	3.8359	3.0578	9.0804	9.3812	6.0248	8.7637	7.929	5.4152	8.6048	7.1594	7.1502	4.3709	9.3969	10.0065	9.7274	5.3519	8.6499	8.1357	8.8012	6.6293	8.6334	7.4131	11.1957	9.1249	10.1725	8.9462	7.2227	7.9181	8.5826	9.0962	8.663	7.5069
TCGA-O1-A52J-01	1798	1	2.1208	5.6382	9.2185	10.3949	10.346	5.3474	3.4018	5.1082	7.5944	7.7083	9.2324	6.9814	6.9814	9.945	9.1485	8.2244	11.1809	3.0316	5.596	8.7727	10.1165	9.124	4.7967	8.931	9.673	9.7821	5.4298	9.1963	7.8041	8.8614	7.9784	10.0346	8.97	7.0918	10.8867	12.5648	7.9112	8.4719	9	9.4344	11.0891	8.5605	9.5017
TCGA-S2-AA1A-01	513	0	3.1477	5.4886	9.1673	7.522	8.9571	6.9864	4.6947	5.6479	9.776	8.3074	8.8809	8.4055	4.4135	9.0844	10.3911	6.483	11.2088	9.9042	5.6289	8.6812	8.3457	8.0818	4.1727	8.4682	10.166	9.9876	4.4135	8.1829	7.1878	8.6995	6.7345	9.617	9.6206	9.1755	8.1466	9.2977	9.573	7.8143	8.9263	8.6927	11.1363	8.6812	8.8215

id	futime	fustat	SMS	SPHK1	SRR	SULT1A1	TDO2	TK2	TRDMT1	TRMT11	TXNRD2	UAP1	UGT1A1	UGT1A6	UGT1A9	WARS2	XDH	NT5C3A	TKFC	ECI1	NMRK1	BCO1
TCGA-05-4244-01	0	0	10.77	7.3339	7.5556	9.6781	5.1662	10.3653	6.9888	7.7125	7.7268	10.6732	1.5851	2.6583	0.025	8.7506	11.5472	8.6463	10.3484	10.1553	8.7738	5.194
TCGA-05-4249-01	1523	0	10.066	6.5908	7.2456	9.1627	6.6783	9.8219	6.9006	7.2479	8.5287	11.159	0.9681	4.4792	0	8.4631	9.9234	8.2027	9.1594	9.7082	7.9557	6.0452
TCGA-05-4250-01	121	1	11.732	8.3724	6.9372	8.6542	7.3442	8.8893	6.7113	7.7021	8.0369	10.1991	0	3.1823	2.2809	8.871	10.8971	9.2894	9.7004	9.8126	7.5213	6.4244
TCGA-05-4382-01	607	0	10.626	9.1497	6.1505	9.3872	7.9287	9.3491	6.6064	7.3594	8.6565	10.0426	0	0.8965	0	8.3132	6.0147	8.5098	9.0331	9.5224	8.014	6.624
TCGA-05-4384-01	426	0	9.3572	6.7618	8.3105	10.2552	5.2111	9.4382	7.477	7.6182	10.2305	10.0003	2.452	0.8433	1.5955	8.6719	5.4913	8.0919	10.2157	9.1228	8.8938	3.0584
TCGA-05-4389-01	1369	0	10.336	7.1203	6.349	9.927	7.0282	9.362	7.267	8.1059	9.5933	10.467	6.7082	13.9576	9.8473	8.0301	5.9164	9.4684	9.6655	9.6432	8.1644	6.4076
TCGA-05-4390-01	1126	0	9.9748	8.5405	6.4787	7.0713	5.2003	9.0837	7.3952	6.8967	9.2518	10.4196	0	1.544	0	8.9222	5.838	9.179	8.5567	10.0208	6.3928	5.4314
TCGA-05-4395-01	0	1	10.578	9.375	6.9882	9.7833	5.7373	9.4295	6.4895	7.3452	8.8405	11.5397	3.8209	11.3612	7.0069	8.5669	11.2582	10.2995	10.7719	10.9266	9.4716	4.9065
TCGA-05-4396-01	303	1	10.234	5.0021	6.5982	7.9463	5.1951	10.3347	6.57	7.4543	9.1129	11.055	4.107	7.5845	0	10.1054	7.4228	8.6321	10.0937	9.0064	8.5233	6.8228
TCGA-05-4397-01	731	1	12.073	8.0336	8.2875	8.2523	7.3556	9.5435	6.8396	7.8262	9.4233	10.6848	1.1019	11.3536	8.2732	7.8079	6.3702	8.8516	10.0193	10.5846	8.9541	6.8506

TCGA-05-4398-01	1431	0	10.604	9.3172	6.7148	7.8277	6.4558	8.9705	6.7462	7.6795	9.27	11.1386	0	2.477	0	8.6196	8.9768	9.656	9.9077	10.1032	8.6667	5.1353
TCGA-05-4402-01	244	1	10.104	7.8443	7.9303	9.8141	6.7107	9.3907	7.2933	7.4268	9.4576	10.3544	0	7.9001	4.872	9.0336	8.1729	9.4046	10.0547	9.5772	8.2629	5.3846
TCGA-05-4403-01	578	0	10.653	7.6053	7.4096	9.4822	5.1599	10.9263	6.4275	7.2711	8.8102	10.7813	6.3475	8.4927	7.2714	8.5843	8.5419	9.6388	9.9708	10.4128	8.2335	8.1998
TCGA-05-4405-01	610	0	9.9623	8.112	7.6241	9.4404	5.2009	9.4971	6.9181	7.1975	8.5825	10.7217	0	3.3293	0	7.7573	4.9764	9.4568	8.8647	9.167	8.1257	4.5965
TCGA-05-4410-01	0	0	9.7628	7.7524	5.8339	7.6312	5.8281	9.8926	7.107	7.557	8.5174	11.0686	0	4.3839	0	8.6983	7.2369	9.3978	10.1511	9.1963	9.0707	6.8251
TCGA-05-4415-01	91	1	10.969	8.2495	7.956	7.3779	4.0492	8.6053	6.2916	8.3851	8.3124	11.0098	0	0.5305	0	8.5047	7.8995	9.8588	9.9743	9.8373	8.3463	2.663
TCGA-05-4417-01	455	0	10.315	8.7806	7.4574	7.9808	8.336	9.7126	6.8815	7.5334	9.3366	10.4441	0	2.4904	0	7.5579	9.3758	8.6115	9.3647	9.3771	9.0138	3.6365
TCGA-05-4418-01	274	1	10.894	9.3749	7.5391	7.6682	7.0619	9.4675	6.1044	7.5324	8.501	10.633	4.0404	6.2532	0.8959	8.654	8.4038	9.7651	9.8334	11.1759	9.0219	6.6155
TCGA-05-4420-01	912	0	11.552	8.793	6.5851	9.1994	7.8407	8.9485	8.1115	7.5112	8.6938	10.3959	0	0.113	0	8.1494	7.5563	9.4176	8.9953	9.6226	7.7467	0.3369
TCGA-05-4422-01	365	0	10.751	5.7611	7.1021	9.6169	6.3025	9.1376	7.7685	8.0983	10.7662	10.8663	9.6669	13.1701	8.485	8.7832	5.9934	8.5347	10.0709	10.0429	8.9228	9.5016
TCGA-05-4424-01	913	0	11.473	6.5809	7.2151	8.7844	5.3756	9.2564	7.2352	6.1218	8.7207	10.3986	0	2.3435	1.1874	8.6743	8.1161	8.1576	9.6254	9.2732	7.3618	3.1087
TCGA-05-4425-01	669	0	10.366	8.4739	7.4931	8.3193	2.8391	10.2974	5.4439	7.4879	8.4474	10.2524	0	0	1.0835	8.3193	9.7871	8.8274	10.3159	9.9205	8.2387	6.8889
TCGA-05-4426-01	791	0	9.9151	7.586	7.4572	9.8743	5.3158	10.9578	6.6048	7.3288	8.7696	10.1417	0	2.5217	0	8.4628	8.8826	9.1833	9.5553	9.7236	8.9069	4.9092
TCGA-05-4427-01	791	0	10.978	7.0551	6.7691	8.1979	6.6568	9.5532	6.8771	8.5267	7.624	10.1779	0	0	0	7.7238	5.6852	8.5089	8.796	8.1551	8.4518	2.1801
TCGA-05-4430-01	761	0	11	8.7856	7.6872	9.1231	7.3547	9.7185	7.0799	6.8559	8.6371	10.6519	1.1371	5.6627	1.8402	8.392	7.3826	8.5501	9.1742	10.008	8.2313	0.9686
TCGA-05-4432-01	761	0	11.077	7.6248	7.1183	9.1045	5.6365	8.5038	7.6082	7.0022	8.9167	10.9202	0	3.8604	0	8.0712	5.0324	9.1789	9.268	10.0618	7.7959	5.1188
TCGA-05-4433-01	730	0	10.738	6.5439	6.2697	9.2607	5.1913	10.7979	6.1879	7.8241	9.0361	10.6633	6.3978	9.4138	5.667	8.4972	9.6722	9.563	9.6531	9.4752	8.7535	6.8252
TCGA-05-4434-01	457	1	11.456	9.2912	7.4795	8.944	7.8185	9.7193	6.7174	8.6293	8.429	10.3925	5.3336	12.4611	8.3484	7.7413	9.0031	9.1641	9.7759	9.5638	9.05	6.7721
TCGA-05-5420-01	457	0	10.386	8.6634	7.2875	8.7291	9.6683	9.2581	7.5273	8.2155	8.6477	11.7796	4.9396	5.5586	2.5354	8.3443	5.1661	9.9617	9.972	11.0237	10.4947	2.861
TCGA-05-5423-01	151	0	10.891	5.9729	7.3763	10.6631	7.3608	10.2639	8.0038	8.5323	9.0377	10.4117	0	3.75	0	8.7169	7.0853	9.0851	9.4615	10.9366	9.4794	6.9465
TCGA-05-5425-01	882	0	10.844	8.1202	6.1693	8.2567	7.5692	8.902	6.5157	7.4942	9.2913	11.3071	3.0065	7.2515	3.8654	8.6531	7.9016	9.3919	10.216	10.729	7.7657	7.3715
TCGA-05-5428-01	670	0	10.345	7.9941	7.9236	9.8292	7.7863	8.2707	7.9117	8.6671	9.0766	11.266	7.3344	13.7299	10.5387	9.1669	1.4563	9.3224	8.4396	10.3427	9.6684	3.9023
TCGA-05-5429-01	275	1	10.845	7.9501	6.6812	9.6212	5.992	10.1134	6.6549	8.0858	8.3885	10.9698	6.8387	8.6854	8.7665	9.6877	9.4095	9.9277	10.2051	10.7169	8.8897	7.6253
TCGA-05-5715-01	62	0	10.504	7.8885	6.9607	8.6096	5.5566	9.4684	5.4576	7.366	9.2856	11.3187	2.3258	8.7265	9.0122	7.4522	10.0548	9.1153	9.6685	9.7855	7.7634	8.083
TCGA-35-3615-01	14	0	10.508	6.1702	8.3973	10.4635	6.3327	9.3582	7.0472	6.9097	9.4415	10.9087	1.1	4.531	0.9431	9.5549	5.9332	7.9781	9.9668	10.9453	8.9375	5.3901
TCGA-35-4122-01	225	0	11.126	8.7105	7.4159	9.6556	9.1983	9.1909	7.084	7.7007	7.8806	10.8872	0	7.1891	3.0516	9.4293	9.0475	9.7537	8.9005	9.886	8.9186	6.425
TCGA-35-4123-01	182	0	10.713	10.5961	8.0545	7.8523	9.0949	9.0514	7.5175	7.6305	8.5188	11.4054	0	0	0	9.258	3.7328	10.19	8.6269	10.2563	9.5877	4.3449
TCGA-35-5375-01	264	0	10.942	7.7917	8.2785	8.6775	7.4714	7.9442	6.5062	8.5466	8.4809	10.5703	6.2156	14.2002	11.0201	8.9607	4.3124	11.129	7.6888	10.7343	8.5551	1.0772
TCGA-38-4625-01	2973	0	12.635	7.6685	7.2952	8.3386	7.3229	9.1736	7.9091	9.3706	9.0426	11.679	0	1.024	0	8.5818	7.7967	9.6774	8.4909	10.2394	7.7596	1.7017
TCGA-38-4626-01	3674	0	10.516	8.0651	7.9012	10.7067	7.7483	9.9055	7.31	7.7961	9.6366	10.0557	0	3.7324	1.6725	8.3249	6.5227	8.534	9.3273	8.847	8.6611	2.7828
TCGA-38-4627-01	1147	1	10.709	9.2354	8.1476	8.6231	7.148	10.7378	6.8062	7.578	8.7261	10.0756	2.5181	11.7859	6.3435	8.5492	8.9713	8.775	9.1889	9.4527	8.7459	4.9158
TCGA-38-4628-01	1492	1	11.197	8.0483	9.2143	10.118	5.749	9.2475	7.6355	7.7288	8.6296	11.2376	0	2.8084	0	8.1824	6.6151	8.5258	9.8131	11.1009	8.063	6.2219
TCGA-38-4629-01	864	1	12.509	10.9736	8.3663	7.6904	8.1889	10.3728	7.4422	7.9541	8.514	10.7987	0	2.3874	0	8.2038	9.1864	9.0937	10.4201	10.0775	8.7287	3.4955
TCGA-38-4630-01	1073	1	10.994	8.449	6.6648	7.2321	8.6126	7.8125	6.5212	7.2884	9.1991	10.4072	0	2.9578	1.6295	8.9861	7.9927	7.4268	10.1048	8.7362	6.6285	2.608
TCGA-38-4631-01	354	1	11.45	9.6517	7.1123	8.1272	4.2517	8.0985	6.5485	8.0309	10.2799	9.5425	0	3.7609	0	8.171	5.6109	10.3449	9.332	10.5101	8.5369	6.8624
TCGA-38-4632-01	1357	1	10.522	9.1519	7.143	7.5862	7.0079	9.7114	7.2946	7.5096	8.2325	10.884	0	7.9294	4.095	9.1449	4.9459	9.496	8.766	10.9579	8.3688	5.904
TCGA-38-6178-01	448	0	10.086	7.7311	7.1921	8.4595	5.3498	9.5323	6.1057	7.7146	8.8615	10.0993	0	8.2463	0	8.4107	7.9512	10.0755	9.0622	9.3163	7.6148	7.251
TCGA-38-7271-01	800	1	9.8977	6.8042	7.6033	8.1851	7.8379	9.5589	6.427	7.2198	8.8323	10.9249	0	3.2253	0	7.8896	5.2841	8.905	9.4042	9.1883	8.5403	3.631
TCGA-38-A44F-01	133	0	10.503	7.8078	8.0201	9.6054	6.5983	10.8668	6.7047	7.7097	8.7886	10.0181	3.4839	9.8027	5.7585	8.042	9.8808	9.4807	9.6856	9.4551	8.783	7.5297
TCGA-44-2655-01	1324	0	10.237	5.9714	7.4645	10.2966	7.7971	10.21	7.1166	7.8756	10.0969	10.2538	2.8649	12.8397	8.5357	9.0627	7.2053	8.6928	10.6742	9.6474	9.7868	3.6012
TCGA-44-2656-01	1429	0	11.304	7.7644	8.0662	9.5698	6.6772	9.9402	6.7261	7.4992	8.6772	10.3667	0	2.1008	0	8.0699	10.8731	9.3071	9.6024	9.4464	9.1146	6.3531
TCGA-44-2657-01	1351	0	10.179	7.1522	7.2318	8.8913	6.4744	9.424	6.6678	8.0077	9.15	9.934	1.8088	10.0656	6.7706	7.2607	7.9024	9.0292	9.4268	9.8078	8.1779	4.7994
TCGA-44-2659-01	1367	0	9.2191	6.3246	6.6688	6.4096	5.9947	10.2391	7.5965	6.8343	8.7055	9.7814	0	2.0295	2.3751	8.4246	6.0312	9.6049	9.0787	9.4368	8.1268	6.1687
TCGA-44-2661-01	1159	0	10.462	7.7272	7.5021	11.3054	6.4345	9.5814	7.579	8.011	8.7366	10.4429	0	5.7102	0.8186	9.0842	7.8582	8.97	9.2321	9.9858	9.0042	4.4041
TCGA-44-2662-01	1280	0	11.919	8.5398	6.334	7.6512	7.7435	10.5388	6.7929	8.4807	8.3165	10.3004	0	1.3163	0.9078	7.9917	12.892	8.5556	9.367	9.3256	7.7459	4.3242
TCGA-44-2665-01	1301	0	10.004	8.5544	6.6224	8.2929	7.4481	11.2339	7.5648	8.2929	8.2819	11.0605	0	0.9626	0	8.0968	9.6674	9.074	8.6641	9.5026	9.1922	5.3853
TCGA-44-2666-01	97	1	10.303	6.2931	8.0333	9.0021	2.3703	10.1421	6.8901	7.7811	9.1619	10.2552	0	1.8738	0	8.8276	9.2952	8.7571	10.2738	9.4623	8.8591	5.7428
TCGA-44-2668-01	761	1	10.864	9.6189	7.3246	6.4335	8.0804	9.6797	6.5046	7.5183	8.9028	11.6537	0	1.7927	1.2366	6.8466	10.6805	9.0994	8.5133	8.597	8.7926	2.5639
TCGA-44-3396-01	1130	0	10.235	9.3437	7.342	8.6558	9.1384	10.1351	7.6863	7.8851	8.5886	9.8733	0	2.2642	0	8.366	9.7721	9.1639	10.5321	9.0757	8.3394	3.5534
TCGA-44-3398-01	1163	0	10.559	8.6534	8.2367	9.2322	9.083	11.1282	7.9373	8.0925	7.9771	10.5237	0	4.3417	0	8.9705	9.7859	9.3439	8.3845	9.3382	9.5525	6.214
TCGA-44-3918-01	1036	0	9.9705	9.7139	8.0007	8.5126	8.822	9.1616	7.147	7.2004	8.7624	10.1642	0	0.7959	0	8.1785	6.3347	9.1674	8.1651	9.3838	8.9521	3.804
TCGA-44-3919-01	1026	1	10.275	7.7176	7.5532	9.0296	6.6038	9.2864	7.0199	7.8163	8.8481	10.0864	0	2.5344	0	8.463	9.3991	9.1836	9.3503	9.6591	8.5706	4.9042
TCGA-44-4112-01	808	1	11.253	8.4749	7.0286	7.3895	6.3597	9.32	6.8612	7.5183	8.1525	10.542	0	1.9302	0	8.8125	6.5877	9.4375	8.6789	10.1317	9.1682	3.0407
TCGA-44-5643-01	1013	0	10.997	7.2448	6.6238	8.048	4.2067	8.4846	7.1653	7.9916	7.2208	9.1733	3.0247	10.1315	6.3259	8.3239	5.7983	8.4571	10.2341	9.3166	8.1659	4.162
TCGA-44-5644-01	863	0	8.9936	6.6841	6.3244	5.7778	1.8718	8.3288	4.5201	7.7266	10.0403	10.4355	0	0	0	7.3686	8.1102	11.295	8.9242	9.5323	7.0803	5.354
TCGA-44-5645-01	852	0	10.742	5.953	7.7605	8.2826	5.3832	10.5442	7.6236	7.9789	9.0622	9.6709	0	0.6542	0	7.7635	8.146	9.7576	9.7412	9.2356	8.5751	3.6409
TCGA-44-6145-01	595	0	10.97	7.5157	7.1401	9.3743	7.3122	9.58	6.2271	7.8514	8.4184	11.1661	4.2805	12.8467	7.6529	7.2477	8.5187	8.4422	9.1584	9.4661	9.4375	6.0596
TCGA-44-6146-01	728	0	10.527	6.3463	6.7433	8.3994	2.2048	10.612	5.4845	7.8271	9.1038	11.2984	4.6049	7.6338	5.609	8.4449	8.6173	9.3788	9.6531	9.1932	9.2032	7.1568
TCGA-44-6147-01	845	0	10.01	7.2093	7.6044	7.807	5.3343	9.6778	7.4357	7.4035	9.2878	10.508	2.35	6.6397	4.1164	8.0405	8.2099	8.9812	9.671	9.6218	8.3542	5.984
TCGA-44-6148-01	704	0	9.7011	6.1227	8.1385	7.7855	8.8221	10.1036	7.3034	7.8804	8.9604	9.5872	1.9468	6.5599	4.2824	8.8083	6.9124	8.6861	10.0594	9.3185	8.6729	3.4826
TCGA-44-6774-01	658	0	10.063	9.2229	7.98	7.6696	8.1041	9.8631	7.0347	7.3625	8.7833	10.3487	0	2.0916	0	7.643	6.4804	8.6327	8.567	8.6661	7.7465	1.5154
TCGA-44-6775-01	705	0	10.812	8.798	7.0834	9.0222	7.1936	10.0939	6.4482	7.9356	8.2968	10.2474	0	3.8248	1.994	8.4421	9.8017	9.5683	9.3289	9.2073	8.6358	5.7
TCGA-44-6776-01	2616	0	10.039	5.017	7.9122	8.8252	3.2661	9.2999	5.9557	6.6145	10.0546	10.5905	1.6245	11.0347	5.7133	8.8022	7.226	8.3789	9.9326	10.2132	8.7975	5.0586
TCGA-44-6777-01	987	1	10.238	8.4567	7.2231	9.0547	7.6236	10.439	6.7164	6.8275	8.3257	10.3092	0	2.4421	0	8.1945	9.2874	8.3398	9.1432	8.9679	8.3699	3.3136
TCGA-44-6778-01	1864	0	10.293	6.8257	7.3556	9.247	6.7238	9.624	7.8177	7.3013	8.4194	10.0114	0	2.8567	2.4691	8.2821	5.1139	9.0083	9.1901	8.3198	8.0462	1.823
TCGA-44-6779-01	500	1	10.19	8.2496	6.8875	7.1259	6.0693	9.9101	5.0391	6.7414	8.9116	11.1636	0	0.8456	0	8.1774	9.1202	10.8706	10.2709	9.979	8.8407	6.5705
TCGA-44-7659-01	691	0	9.8483	6.3985	7.0397	9.1485	2.6396	10.0107	7.1523	7.6207	9.3733	10.3583	0	4.6817	0	8.8071	6.2571	8.4075	9.8898	9.9378	8.9687	3.3174
TCGA-44-7660-01	592	0	11.405	8.552	5.6793	7.3992	9.1367	7.8494	6.8399	7.0468	8.9797	10.6389	5.4874	12.1112	9.1768	8.1606	6.4741	9.2225	10.8805	9.0918	7.3179	0.8806
TCGA-44-7661-01	557	1	11.298	9.12	7.648	8.2609	8.2282	9.9354	6.9871	7.6878	8.5246	10.0037	0	1.3587	0.9136	8.0829	11.1338	9.7289	9.4179	8.5477	7.9269	2.0437
TCGA-44-7662-01	218	0	11.094	10.2672	6.8167	7.0902	8.8768	9.7702	6.8887	8.0547	9.6204	9.7315	0	0	0	8.0833	6.9418	9.3051	9.4556	9.3058	7.9822	1.1269
TCGA-44-7667-01	1097	0	10.257	7.6561	7.2989	6.8185	7.2929	8.7576	7.2335	7.6964	8.9271	10.1395	0.4146	1.5838	0	8.0225	5.4795	9.0095	9.5605	9.5548	7.05	0.7363
TCGA-44-7669-01	574	1	11.515	6.9238	7.882	7.9155	5.3956	8.405	6.7471	8.013	9.2745	10.1806	1.1268	6.1555	1.2454	8.8381	2.8347	10.3804	9.7487	9.7742	7.7548	1.7881
TCGA-44-7670-01	882	0	10.784	8.1377	6.642	9.2373	5.9228	8.6229	6.3575	6.783	9.2029	9.8524	6.0428	13.7288	10.4441	8.4385	5.6834	8.3795	10.1102	9.1884	7.1897	5.2748
TCGA-44-7671-01	889	0	10.067	6.9797	8.2884	10.0282	5.7782	9.6603	6.592	7.8926	10.1786	10.6117	0.8782	10.6698	5.6366	9.3879	6.438	8.5226	10.1584	9.6659	8.3383	6.9674
TCGA-44-7672-01	719	0	10.998	9.3859	6.7844	7.4557	7.8759	10.3102	7.0033	7.1213	8.88	10.3846	0	5.3219	1.7343	8.3167	6.6465	9.1467	9.9745	9.2108	7.9977	4.215
TCGA-44-8117-01	385	0	9.7055	9.8093	6.4629	6.2232	7.0311	9.2161	7.4585	7.7102	9.4271	10.6158	0	0.6582	0	9.3306	4.0788	8.7548	9.6063	9.5771	7.2023	1.7279
TCGA-44-8119-01	285	0	11.665	9.5516	6.4019	7.9122	5.6866	9.0872	7.3675	8.0532	9.276	10.1063	0	0.6098	0	7.7542	2.3809	9.1155	9.8595	8.5025	7.0586	0
TCGA-44-8120-01	260	0	10.346	9.2108	7.8615	9.3677	3.9352	10.152	7.1725	7.8701	9.7206	9.516	0	5.8124	1.8643	9.4242	6.4897	8.9779	9.8568	9.8317	8.428	7.0709
TCGA-44-A479-01	486	0	10.239	7.6265	7.2158	8.1479	8.0586	9.6573	7.2637	7.9065	8.7465	10.063	2.2011	4.9135	0	8.3695	8.1039	9.4364	9.4771	8.7804	8.5959	8.0647
TCGA-44-A47A-01	466	0	10.164	6.6895	7.2035	9.3419	5.8143	9.6926	6.6257	7.7058	10.1217	10.8206	0	8.0249	0	8.1156	11.2299	9.2009	10.5497	9.9314	8.4471	6.1416

TCGA-44-A47B-01	287	0	10.428	8.0748	6.1597	8.5139	5.5026	9.1587	6.4574	8.1747	8.6679	10.0211	0	2.1962	0	8.8966	8.2252	7.8075	8.6759	9.2914	7.7365	0
TCGA-44-A47G-01	351	0	10.879	8.9943	7.0392	9.4347	8.0752	10.5238	6.8731	8.3025	8.6283	10.2434	5.5765	13.3559	9.2004	8.1384	9.5231	9.2737	9.3055	9.2092	9.2629	5.5377
TCGA-44-A4SS-01	415	0	11.055	9.0109	7.3937	9.8565	6.7159	9.9073	6.4857	8.1328	8.5003	10.3618	7.4576	12.8322	10.2512	8.1887	9.0284	9.347	9.2174	8.5178	7.6976	7.8122
TCGA-44-A4SU-01	409	1	11.339	7.5423	8.2169	9.2189	5.0818	10.1237	7.3319	7.5716	9.6382	10.1029	0	6.23	0	8.367	8.7345	9.0401	10.0521	9.6842	8.026	4.0023
TCGA-49-4486-01	2318	1	10.254	4.2122	8.3784	10.8319	7.1049	9.2506	7.1427	7.7843	11.1594	10.764	0	2.2481	0	9.1845	6.1862	8.4962	10.8064	10.8216	9.4191	5.1119
TCGA-49-4487-01	855	1	10.71	8.9943	7.2646	8.0389	8.4293	8.885	7.1293	7.3834	9.4741	11.1054	0	1.4249	0	8.8166	4.4635	8.7672	8.9891	9.8483	8.4293	1.4249
TCGA-49-4488-01	869	1	10.637	8.8905	7.6107	9.342	6.7567	10.2919	7.0641	6.5909	9.7214	10.3095	1.5724	10.9944	7.13	8.5174	1.6787	9.6897	9.8565	9.7075	8.8717	3.2936
TCGA-49-4490-01	385	1	10.248	7.9996	6.7856	9.1575	3.6127	9.9816	6.7882	7.6125	9.0571	9.5121	0	8.8231	5.8309	8.4824	8.8088	8.8356	10.6886	10.2479	8.8562	6.8597
TCGA-49-4494-01	1081	1	10.903	9.1939	9.0401	8.2451	6.1351	9.9694	6.6038	6.4006	9.0947	10.3823	0	3.162	0	7.921	9.899	10.1729	10.2934	10.7747	9.1339	4.585
TCGA-49-4501-01	1421	1	11.008	6.9557	6.9656	9.7529	6.4668	10.0912	7.3342	7.757	8.6408	10.5239	0	4.0694	0	7.9953	8.7043	9.1564	10.0465	10.48	8.7791	8.1442
TCGA-49-4505-01	428	1	10.609	8.1426	7.8402	8.8049	7.1111	10.5047	7.5235	6.7237	8.8739	10.3329	0	3.9237	0	8.3174	7.8718	9.3322	9.946	10.1278	8.7268	6.1813
TCGA-49-4506-01	999	1	10.984	11.5719	7.4259	9.0288	8.548	8.4997	5.0197	7.9434	8.6052	12.1185	7.5865	12.3702	7.6602	8.9671	7.6973	11.5449	10.066	11.7779	9.79	1.9206
TCGA-49-4507-01	268	1	10.103	8.9521	6.5299	10.9139	7.5974	8.7019	5.4784	7.7489	9.3449	11.665	3.1432	12.5177	8.584	8.3262	7.5897	9.699	10.445	12.4586	10.2212	4.1091
TCGA-49-4510-01	896	1	9.7324	7.6631	7.4026	11.2564	3.2174	8.9238	7.3075	7.0364	10.0165	9.5836	0	2.821	0	8.5454	5.6395	8.727	8.9295	11.6729	8.6739	2.6843
TCGA-49-4512-01	905	1	10.899	8.5357	8.0143	7.7868	6.101	10.6402	6.5781	7.1925	9.6801	10.0066	0	5.065	1.9569	7.966	5.4858	9.0133	9.7578	10.5893	8.7504	6.0847
TCGA-49-4514-01	1700	0	10.848	7.7472	7.1054	11.2045	6.7767	8.9851	5.5751	7.5043	8.9609	11.3591	3.5935	12.6338	9.3126	8.7744	6.9474	9.6599	10.5869	11.0886	8.457	7.4101
TCGA-49-6742-01	488	1	11.098	7.1949	7.7064	9.8143	6.8467	9.1642	5.713	7.1029	9.2085	10.5542	5.2738	3.5403	4.6659	9.1087	7.4916	10.4936	11.0368	9.7013	9.1396	8.3327
TCGA-49-6743-01	1621	0	10.413	8.1331	6.7998	10.1859	6.9371	8.6055	6.2661	7.8039	8.8285	11.1048	0	3.6392	2.5406	7.6448	11.3841	8.4485	9.0125	9.7698	8.4948	3.567
TCGA-49-6744-01	1683	0	11.126	8.0621	7.081	8.8826	7.2787	10.3265	7.0704	7.0484	9.1438	10.347	0	6.0362	0	8.7575	7.5883	9.3011	9.5605	9.3571	8.6591	4.9561
TCGA-49-6745-01	522	0	11.434	7.8482	7.6223	9.4096	7.2231	9.7481	7.1249	7.467	8.4201	9.5083	0	3.7317	0	8.3544	10.0605	9.8469	9.4436	8.4568	8.0836	5.4513
TCGA-49-6761-01	354	0	10.619	10.8815	7.4448	6.9393	8.0092	9.6809	6.6647	7.0311	8.2012	10.689	0	1.678	0	8.1199	8.1976	10.133	9.1641	9.8382	8.1648	0
TCGA-49-6767-01	677	0	11.582	8.561	6.3235	6.1818	4.4403	9.2992	6.0846	7.9901	10.7099	9.9664	0	1.1266	0	7.4164	8.5597	8.8571	9.2227	8.5184	8.4996	5.5773
TCGA-49-AAQV-01	677	1	10.514	8.0862	7.7849	10.8255	6.5419	9.8828	7.1569	8.1896	9.6523	10.3753	0	4.9728	0.1986	8.3301	9.8467	9.3933	10.4271	9.6928	8.8151	5.3697
TCGA-49-AAR0-01	4765	0	10.713	7.9619	7.687	9.7568	5.2169	9.4276	6.9304	7.5347	10.3069	9.8999	3.7388	12.4131	7.3477	8.2824	6.7627	8.7903	9.9376	10.6747	8.8977	3.7436
TCGA-49-AAR2-01	2224	0	10.403	7.7876	6.8384	11.0889	6.0163	9.8947	6.4633	8.6552	10.7464	9.1724	3.9664	13.0066	10.6341	8.884	6.7799	9.6209	10.9906	11.3625	9.0565	6.7553
TCGA-49-AAR3-01	1893	0	11.765	10.843	7.5824	8.2568	7.5897	9.4981	6.3423	7.9615	8.5156	9.8591	4.3349	10.5449	7.5788	8.0495	8.2024	9.287	9.9174	9.3936	7.6536	5.2652
TCGA-49-AAR4-01	879	1	11.131	9.4457	8.0326	8.0091	7.2086	9.7678	6.5498	7.4146	9.2379	9.6583	0	5.9681	2.1534	8.3064	9.8878	8.8613	10.2472	9.1186	8.7911	4.9071
TCGA-49-AAR9-01	260	1	12.275	10.071	6.8669	6.7539	4.1683	9.6879	5.2545	7.5172	8.0138	10.0205	0	3.337	0	8.1668	9.0676	8.6727	9.5266	9.8292	8.7984	4.013
TCGA-49-AARE-01	1229	1	11.142	9.0635	7.6293	8.7581	7.2229	9.7738	5.9958	7.9418	10.1564	10.5997	0	3.6052	1.9783	6.9635	9.3715	9.1338	10.4249	10.0538	9.5424	4.3303
TCGA-49-AARN-01	1135	1	10.178	7.747	7.2749	10.0033	4.9119	10.2914	6.2967	7.4316	8.9836	10.5095	2.3476	5.7913	3.8757	7.8967	9.4882	9.1464	10.0889	9.7913	8.8002	6.8937
TCGA-49-AARO-01	3759	0	10.791	9.1883	7.8709	9.112	6.9851	10.7136	6.8474	7.6348	9.3137	9.6971	2.8418	6.5952	0	8.0626	8.5447	8.5799	10.3557	9.4755	8.6968	5.2219
TCGA-49-AARQ-01	6732	0	10.534	7.6573	7.4722	9.6895	5.4861	10.6259	6.0724	8.4507	9.1088	10.1395	2.1555	13.347	10.4653	7.6947	9.4098	9.9394	8.8768	10.6579	8.2037	5.7346
TCGA-49-AARR-01	4992	0	9.7478	8.3045	8.2255	9.7649	6.0176	10.0201	7.8106	7.4258	8.9362	10.5425	3.0868	7.3322	2.8693	8.4021	5.9596	8.7623	9.7632	9.2533	8.9119	2.9323
TCGA-4B-A93V-01	300	1	10.709	8.8177	6.9573	8.4404	5.0122	10.1454	7.9017	7.6962	10.8555	10.8829	0	0	0	8.9371	2.7598	11.445	10.4192	11.2211	9.7813	0.5667
TCGA-50-5044-01	624	1	12.295	10.5502	7.4423	8.1209	6.7475	9.7699	5.3903	7.6845	8.7817	11.3021	0	3.6954	2.6882	7.3997	6.4926	9.3622	9.5939	9.6611	9.5939	3.1998
TCGA-50-5045-01	2174	1	10.139	9.2945	8.2814	8.8638	8.7579	9.692	7.0459	8.0194	9.1123	10.3514	0	4.8772	3.1514	8.1519	8.6886	9.0709	9.4324	10.7961	9.7541	3.1067
TCGA-50-5049-01	3094	0	10.442	8.8926	7.4771	7.9424	8.8393	9.4144	7.1907	7.7943	8.2374	10.2818	0	3.1396	0	8.1995	1.0814	9.2323	9.1736	9.909	9.168	1.4191
TCGA-50-5051-01	478	1	10.438	6.8758	7.863	10.8172	3.7435	9.2468	7.4016	7.5699	9.9728	10.0664	4.6606	12.4801	8.6935	8.6068	4.9276	8.6822	10.0177	10.0943	9.5192	3.3639
TCGA-50-5055-01	1830	1	10.241	8.7941	8.0892	10.25	9.1462	9.7367	7.0915	7.7777	8.9947	10.1732	2.3012	7.6016	4.2541	8.6723	6.5549	9.7714	9.6691	9.8885	9.0134	4.4979
TCGA-50-5066-01	1442	0	10.948	9.0809	8.3542	9.4122	8.9662	9.4647	8.2194	8.5053	9.1004	11.0636	2.4327	9.888	5.6675	8.3901	3.9008	9.556	8.4235	10.745	9.7567	3.4035
TCGA-50-5068-01	1499	1	10.862	7.9983	8.5943	9.6214	8.4717	8.6815	7.4757	8.3968	8.5593	11.1263	0	4.9165	3.4767	8.2505	4.9508	9.4415	8.8217	10.8551	9.5426	2.971
TCGA-50-5072-01	250	1	10.858	8.6463	7.4427	9.7896	6.6215	8.8588	6.2	7.597	9.3308	10.2689	2.2896	8.8426	4.0147	8.7792	7.5586	9.8305	10.139	10.2189	8.7235	6.5307
TCGA-50-5930-01	282	1	10.304	8.5327	6.8716	7.6358	6.2611	9.6399	6.1029	7.6831	8.4578	11.3957	1.2756	3.5275	0	8.3327	8.4359	8.6702	8.9971	9.5811	9.1296	2.6581
TCGA-50-5931-01	434	1	11.377	7.8101	5.7808	9.4087	6.2974	9.5417	6.0171	7.8817	9.3843	9.9535	3.2214	9.1242	5.9609	7.9161	5.498	8.0832	10.423	8.8787	7.9311	1.533
TCGA-50-5932-01	1235	1	10.201	5.9272	7.3236	8.9348	3.7954	9.5885	7.2228	7.7513	8.8094	10.0555	0	0.5014	0	8.4083	7.4108	9.0422	9.9357	9.5362	7.9823	2.582
TCGA-50-5933-01	2393	1	10.794	8.7706	6.5323	7.5761	6.4636	9.5915	6.8619	7.6254	8.1941	11.927	0.6238	2.3367	0	8.3646	9.1352	9.9165	8.2289	9.0179	8.4108	3.5503
TCGA-50-5935-01	653	1	10.295	6.7697	7.1814	9.2488	5.2691	9.584	7.185	7.618	9.0638	10.9483	0	0	1.1848	7.9617	9.121	8.7927	9.5154	9.4721	8.9157	7.1467
TCGA-50-5936-01	257	1	11.119	8.5509	7.4969	7.8304	6.0457	9.5555	7.0104	8.1039	8.3851	10.8026	2.8667	4.537	2.2634	7.6106	8.8156	9.3184	10.1077	10.0952	7.8894	2.922
TCGA-50-5939-01	460	1	11.16	9.303	6.9259	9.453	6.4119	9.1962	6.5017	8.1193	8.6234	11.0819	3.1739	11.1614	6.849	8.3364	8.7365	8.4423	9.7463	9.5364	8.5724	1.6099
TCGA-50-5941-01	1474	0	9.8982	7.9068	7.2126	8.0677	7.6232	9.5465	6.5439	6.8468	8.9943	9.9654	0	4.143	0	8.3201	7.6138	8.9906	9.4781	9.1667	8.5096	4.0896
TCGA-50-5942-01	1847	0	9.705	4.3375	7.3297	9.7793	2.6122	9.8339	7.3179	7.2709	9.1336	9.842	0	3.6447	0	8.6413	9.85	8.5848	9.8708	9.1171	8.7567	5.2017
TCGA-50-5944-01	1750	0	10.564	5.055	7.2194	9.9055	5.0318	9.5587	6.9422	7.256	8.9933	9.8475	0	3.8319	0	8.7473	9.1884	8.6608	9.0002	9.4218	8.3677	6.4017
TCGA-50-5946-01	1617	0	10.269	7.8877	5.6399	7.7496	5.0655	9.0093	8.0642	6.8678	9.6327	11.0349	0	5.3413	0	7.7732	8.0415	9.3559	10.435	9.621	7.3064	1.8372
TCGA-50-6590-01	1288	1	11.182	10.1083	7.5826	6.5619	9.0552	9.2338	6.0372	7.3816	9.3892	10.5564	0	8.3854	4.1404	7.6275	7.7381	9.5877	8.9996	8.2388	8.508	1.3953
TCGA-50-6591-01	119	1	11.472	7.203	7.9085	6.463	3.8219	8.063	7.1312	8.4847	7.8812	10.7424	0	0	0	8.1043	6.6202	9.5452	8.0886	8.5552	6.6886	0.6757
TCGA-50-6592-01	777	1	10.002	9.353	6.8507	7.5652	7.6598	8.9836	5.0305	7.6258	8.4702	10.2229	8.9171	12.8986	8.8752	8.2945	9.557	9.6621	8.5808	8.7482	8.7338	7.4067
TCGA-50-6593-01	336	1	10.529	7.6506	7.4498	10.2394	5.6914	10.1239	6.4315	7.7626	9.1773	9.8962	0	5.9599	1.3876	8.3239	8.344	9.2656	10.327	9.5362	8.8302	5.3941
TCGA-50-6594-01	370	1	12.481	9.0508	7.1762	10.0112	7.0897	8.196	5.9927	6.4876	10.087	9.9824	0	0	0	8.7565	13.62	8.4023	9.4091	10.0122	8.6347	2.5679
TCGA-50-6595-01	189	1	11.452	9.7427	7.8046	6.8753	8.6475	9.6811	6.6163	7.8289	8.2686	10.2987	1.4005	5.703	0	7.7535	9.5573	10.8305	9.3397	9.0714	7.9872	2.2805
TCGA-50-6597-01	1268	1	11.474	6.5593	7.7145	10.2207	3.7282	10.2116	5.0046	7.2727	7.9648	9.6306	0	5.5559	3.6173	8.1956	4.9173	9.696	10.4528	11.3908	8.2186	9.3113
TCGA-50-6673-01	22	1	9.7985	6.9723	7.2123	8.0199	7.1474	10.3336	7.6607	7.379	8.9532	9.7037	5.231	9.9368	8.1128	8.9	9.7124	9.0677	9.4941	9.0403	7.9979	6.7109
TCGA-50-7109-01	308	1	10.615	6.3576	7.1578	6.7106	6.9783	9.2131	8.6107	7.7037	9.6212	10.7609	0	2.0117	0	8.9574	3.6546	9.7748	9.0349	9.1197	8.178	0.6872
TCGA-50-8457-01	1125	0	9.8576	6.0924	7.7455	9.1345	5.2248	9.7216	6.8296	8.1325	9.1356	10.039	0	4.7613	0	8.7081	7.333	8.3502	9.2991	9.9989	9.1816	5.1546
TCGA-50-8459-01	1119	0	10.411	10.5876	7.5294	9.4427	6.929	10.6812	6.768	7.7908	8.8999	9.5446	2.9018	7.3792	5.0171	8.0594	7.8894	8.6294	9.398	9.0763	8.588	4.4764
TCGA-50-8460-01	829	0	9.1652	6.4004	7.5665	9.8048	6.3604	11.3124	6.2822	7.7795	9.3749	10.2024	0	5.1879	0	9.0619	9.1822	8.5032	10.3893	10.1756	8.9289	7.5071
TCGA-53-7624-01	1043	1	11.337	8.078	6.3053	9.2879	6.199	8.7321	6.0729	7.7938	8.859	9.6172	7.2743	13.313	9.106	7.6964	5.9347	9.5135	9.8737	9.563	7.4396	6.9792
TCGA-53-7626-01	929	1	9.6413	6.9826	7.4767	8.0914	6.2339	9.4194	7.0053	7.2352	9.134	10.1128	0	2.465	0	8.1295	6.8645	8.4517	9.2335	8.7889	8.0236	6.0846
TCGA-53-7813-01	424	0	10.755	5.4094	7.6075	6.5469	6.3966	8.6243	7.6015	7.423	8.6504	10.746	0	0	0	8.0393	6.2869	9.2464	8.4614	8.3015	9.2658	3.6212
TCGA-53-A4EZ-01	1071	0	10.728	6.6117	8.2576	9.1763	4.7104	8.0151	6.3375	8.359	9.7139	10.2233	5.7769	14.0042	9.9904	8.4233	5.9988	10.3448	9.7017	9.7367	9.0768	7.332
TCGA-55-1592-01	701	1	9.9249	6.669	7.3995	10.3806	5.4869	9.293	7.2091	7.2378	8.9095	10.62	0	5.8092	0.7502	8.3903	7.4514	8.3668	9.391	9.3593	8.4946	4.7683
TCGA-55-1594-01	1178	0	10.49	7.3883	6.3236	9.3547	6.0152	9.0033	7.9396	8.3103	9.0766	9.9875	1.591	8.3403	3.3558	8.0116	7.004	9.2109	10.2563	9.6983	7.4163	5.2902
TCGA-55-1595-01	1479	0	9.248	8.2752	6.1116	8.7465	6.1288	9.2091	7.0302	7.1529	9.0686	10.3886	0	0	0	7.8677	4.4021	8.6258	9.515	9.7958	8.8945	3.0035
TCGA-55-1596-01	2065	0	10.817	7.5679	7.8365	7.8537	6.8562	8.9999	6.8854	6.8113	9.4938	10.1482	0	3.3439	2.733	8.2542	7.476	10.5134	9.2264	10.5342	7.9397	8.9915
TCGA-55-5899-01	930	0	9.9163	7.4043	7.4449	7.0922	11.589	9.77	6.4759	8.2366	9.437	9.7972	7.7037	14.2925	10.7824	7.3413	5.5635	11.2392	11.3952	9.184	8.4066	0.6521
TCGA-55-6543-01	435	0	9.7851	7.2403	8.0636	8.4161	2.4487	10.5788	7.2432	6.9303	8.8912	10.1707	0	1.6914	0	8.4441	9.4729	8.5183	10.2962	9.1281	8.2804	0.6391
TCGA-55-6642-01	2449	0	9.7285	8.0027	6.4156	6.5853	5.7216	9.3711	6.056	6.6145	8.5912	10.3383	0.7609	2.2906	0	8.3372	6.7871	8.4551	8.5764	9.2752	8.3747	1.8565
TCGA-55-6712-01	171	1	12.074	7.6597	7.5464	7.1005	6.2702	9.6655	6.7382	7.4203	9.3949	9.8811	0	3.3486	0	8.3303	11.3015	9.0172	10.2139	9.4159	8.8507	3.4408
TCGA-55-6968-01	1293	1	11.581	8.2622	7.5888	7.7904	5.6171	9.1562	6.2258	8.0922	8.2686	10.4844	2.4577	8.292	4.8325	7.992	4.0898	9.2716	9.6977	10.3761	8.0304	1.7179
TCGA-55-6969-01	1239	0	11.092	9.0243	6.8704	7.3283	6.4807	9.1786	8.0284	7.4184	9.4171	11.0713	0	2.6263	0	8.6455	4.1896	9.8786	9.3516	9.593	8.5637	2.4553
TCGA-55-6970-01	464	1	10.108	6.6372	7.1583	9.2687	6.1826	9.0191	7.1369	7.6794	9.3189	10.904	2.6968	2.5366	0	8.2582	6.8092	8.6758	9.2398	9.5724	8.9135	3.3668

TCGA-55-6971-01	1400	0	9.6657	8.3206	7.1749	8.6043	7.0724	9.4846	7.062	7.3797	8.8019	9.9089	1.741	6.4459	3.5635	7.4372	6.8724	9.1529	9.0193	8.7574	8.6812	3.1284
TCGA-55-6972-01	1632	1	10.182	3.9948	6.0534	9.4291	5.4718	8.9752	8.079	7.5562	10.352	10.6937	0	3.4949	0	7.4472	6.9172	8.6499	9.9323	10.6646	9.8964	4.7068
TCGA-55-6975-01	118	1	10.865	9.8884	7.8906	7.1308	6.4623	9.3251	5.9192	7.8017	8.6619	9.9924	0	3.0949	0	7.8757	7.7521	9.2575	9.7129	9.2693	8.2697	3.5725
TCGA-55-6978-01	176	1	11.499	9.2826	8.0165	8.1451	7.6609	9.6151	7.4893	8.0305	8.3469	9.6665	0	5.4004	0	8.0927	7.8073	9.6517	8.5126	8.7375	8.0561	4.6098
TCGA-55-6979-01	237	1	10.429	8.6986	7.8039	7.9273	8.3102	10.1976	6.3206	7.8977	8.7297	10.3818	0	1.2687	0	8.2778	10.7772	9.8553	9.2722	9.2689	8.0085	3.345
TCGA-55-6980-01	2109	0	11.468	7.1445	6.975	9.4001	5.441	11.3367	6.8012	7.2911	9.0467	9.725	0	6.2659	0	8.3582	8.5844	8.8171	9.4826	9.251	8.4631	8.687
TCGA-55-6981-01	1379	1	11.086	7.8954	7.5397	7.4914	5.0259	10.6168	6.6456	7.0697	9.1355	10.1742	0	1.4198	0	8.7273	9.1885	9.131	10.2332	9.1906	7.4844	2.4292
TCGA-55-6982-01	995	1	11.411	8.4255	6.5742	9.3258	6.6387	9.4393	6.5132	7.697	8.3748	10.3503	2.7808	5.5182	0	7.658	9.7345	9.887	9.894	9.4602	7.784	6.6651
TCGA-55-6983-01	2823	0	10.636	6.929	7.8686	8.4942	6.8829	9.4423	6.6014	6.5748	9.1462	11.0093	1.0403	6.9507	0	8.7413	7.0311	9.091	9.8689	9.6577	8.8932	6.0107
TCGA-55-6984-01	760	1	11.52	7.7014	6.1486	8.3546	5.4582	10.7577	6.4417	8.2502	8.3904	11.3563	0	6.7731	3.3957	7.9636	10.303	9.7759	9.8464	9.5252	8.7349	2.4778
TCGA-55-6985-01	1233	0	9.9715	8.8011	7.5501	6.9206	6.7105	9.6776	7.1196	7.3482	8.53	10.4407	0	5.7624	1.4653	8.0829	6.5928	9.1079	9.4112	9.4746	7.4746	4.5009
TCGA-55-6986-01	3261	0	10.72	5.8516	7.4215	8.4445	4.1469	10.0126	6.4747	7.4959	9.2161	9.929	0	3.3851	0	9.2544	9.5748	8.6953	10.2167	9.0658	8.2343	1.2948
TCGA-55-6987-01	2137	0	10.15	8.21	6.6701	7.7552	6.9237	9.0372	7.3649	7.6373	8.8075	11.6729	4.9169	11.7081	7.7974	7.4343	5.8454	9.5335	8.8903	9.2868	8.9533	4.1658
TCGA-55-7227-01	952	1	10.108	7.636	7.6612	8.1689	6.9703	10.34	7.2315	7.4862	8.6102	9.6696	0	3.6221	0	9.007	8.3631	9.108	9.5309	9.2958	8.8211	3.5067
TCGA-55-7281-01	872	0	10.235	7.5279	8.0683	9.6405	5.8288	9.76	6.0804	7.2479	8.6879	10.4148	0	1.9445	0	8.6556	9.5281	8.1935	9.8012	9.9171	8.8319	4.0343
TCGA-55-7283-01	609	0	10.111	5.1093	7.1467	9.3873	3.1731	9.4288	6.463	7.3255	8.9823	10.5979	0	2.91	0	9.2925	8.2797	9.5495	10.2959	9.8946	8.268	5.7717
TCGA-55-7284-01	243	1	10.88	7.4739	7.0083	10.0016	3.4963	10.5565	6.2735	7.7635	8.9348	10.6677	5.0977	7.5435	5.7461	7.9295	7.8596	9.5185	9.3005	9.5142	8.8729	7.583
TCGA-55-7570-01	824	0	11.032	7.6108	8.2751	9.9641	4.7855	8.6174	6.9779	8.7963	8.3643	9.8143	2.744	8.6967	3.6607	8.6553	4.0224	9.5896	10.9803	9.4091	7.6911	1.2061
TCGA-55-7573-01	487	0	9.4379	6.5804	7.5618	9.24	3.869	9.6029	7.281	7.8201	8.5473	10.7032	0	5.4293	2.2025	8.8893	7.768	8.2838	9.3679	9.3361	8.2038	4.7345
TCGA-55-7574-01	995	1	10.22	8.6523	7.0595	8.9083	7.828	9.5049	6.6471	7.8077	8.6704	10.1828	0	3.8575	0	8.5835	5.5558	8.5594	8.8223	8.7174	8.4589	2.313
TCGA-55-7576-01	670	0	10.39	8.1861	7.9649	9.0699	6.0176	9.6956	6.6293	7.1147	8.936	11.0705	1.527	3.0505	0.775	8.9918	4.9795	9.3083	9.1846	9.2573	8.438	5.216
TCGA-55-7724-01	705	0	11.006	4.443	7.9862	8.8326	6.2696	9.7842	6.8977	6.9571	6.9916	10.2305	5.0318	10.5105	4.9612	8.2074	5.8608	9.2579	8.2697	6.9336	9.0877	3.1725
TCGA-55-7725-01	442	0	10.496	3.8734	8.5494	10.7504	5.1133	9.5097	7.6556	8.1475	7.602	10.7635	0	3.6501	0	9.0481	9.7868	9.5627	8.1845	7.28	9.5344	6.3555
TCGA-55-7726-01	652	0	10.355	9.1443	6.5547	6.5841	8.1812	9.4626	6.8623	7.5832	7.8889	10.4368	3.1461	9.5626	5.4664	7.3754	10.286	9.6701	8.159	7.5899	9.0275	0.9163
TCGA-55-7727-01	119	0	11.093	6.5646	7.547	8.319	5.1644	9.8545	7.1984	8.5054	8.036	11.1159	0	0.9392	0	8.9501	5.4633	9.3435	8.051	6.9534	9.3915	3.5861
TCGA-55-7728-01	704	0	10.418	7.8334	7.6603	9.6964	4.3141	10.6048	6.4296	7.6881	9.4212	9.8398	5.2486	7.4319	5.5983	7.3801	9.0609	9.0718	9.9098	10.062	8.5484	7.5322
TCGA-55-7815-01	773	0	9.9188	5.4287	8.5553	8.1778	7.1927	10.1865	7.0141	7.8916	6.2883	11.0349	0	3.7064	0	8.8038	10.5841	9.8405	7.3158	6.2317	8.6395	6.0252
TCGA-55-7816-01	468	1	9.8631	7.578	8.051	9.6831	6.1432	10.2657	7.1636	7.9709	8.3187	10.4155	2.4659	0.9734	0	8.613	6.0432	9.1776	9.0558	8.5614	8.5415	4.9187
TCGA-55-7903-01	567	0	11.825	7.2764	6.995	8.3613	3.8533	9.5097	7.5986	7.383	8.7991	10.6572	0	2.07	0	8.7975	7.5185	9.1511	9.9205	9.0146	7.8837	6.2142
TCGA-55-7907-01	343	1	10.854	8.6059	7.1534	7.8799	7.194	9.3144	6.5188	7.415	8.903	9.9039	0	5.2679	0	7.2909	7.3123	8.8576	9.2857	9.6454	8.0724	7.3883
TCGA-55-7910-01	1040	0	9.4418	7.7863	5.5212	10.131	4.9369	8.9977	7.1471	8.5862	8.7749	10.8932	0	0	0	8.5259	7.576	10.3648	9.1276	10.7392	8.5186	0
TCGA-55-7911-01	537	0	10.206	8.3248	7.2136	10.562	6.8075	9.2601	6.7377	7.9097	8.4221	10.3868	0	0	1.0407	8.0048	9.4167	10.0146	10.2451	9.3667	8.4724	6.2391
TCGA-55-7913-01	561	1	10.547	6.8022	5.7845	8.3819	6.7554	9.2042	6.5542	7.1133	9.5894	11.2602	0	1.4954	1.935	8.3391	8.4271	9.6291	9.6239	9.5883	8.4534	1.4446
TCGA-55-7914-01	187	1	10.83	7.2966	6.0978	7.3212	5.6575	9.7032	6.6646	7.588	8.9979	10.6167	0	0	0	9.0586	9.9054	9.1247	10.1792	9.1311	8.614	7.5189
TCGA-55-7994-01	603	0	9.8974	7.489	6.8601	8.7588	5.0709	9.9937	6.4131	7.6173	8.7138	9.6164	0	3.2066	0	8.2509	9.0362	9.5152	9.7936	8.9098	8.4044	5.5023
TCGA-55-7995-01	889	0	11.101	8.0746	6.8985	10.8381	7.4719	8.5529	5.5494	7.6532	9.3505	10.9816	6.8351	12.9705	8.6661	6.9115	7.372	9.0133	10.0926	10.4943	8.6414	4.9136
TCGA-55-8085-01	904	0	10.916	7.751	6.65	9.4354	6.9961	9.7099	6.5743	7.4934	10.1333	10.4452	6.6318	13.6211	10.698	8.7471	5.2566	8.9444	9.8514	9.7033	7.7079	7.4366
TCGA-55-8087-01	462	0	10.312	3.9486	7.9605	8.9065	1.0336	9.3401	7.0394	7.8121	9.9087	11.1611	0	2.4692	0	8.0087	7.3789	7.9569	9.626	9.2093	8.9294	5.2211
TCGA-55-8089-01	702	1	11.094	9.0144	7.4048	7.7324	8.2779	10.4234	6.2608	7.415	8.3198	10.4031	4.1601	11.2733	6.9909	7.6057	8.8741	10.558	10.088	8.5485	7.8924	5.9571
TCGA-55-8090-01	598	1	10.174	9.3569	8.0477	10.4524	6.9997	10.6004	6.7569	7.4336	9.2167	9.2741	0	2.9573	0	7.5948	11.0456	9.1035	10.2723	9.3965	8.6875	2.5741
TCGA-55-8091-01	600	0	9.7325	9.0087	6.8213	8.6416	7.5299	10.5808	6.617	7.6152	9.073	10.2399	0.9711	8.6498	4.5286	7.7293	11.1491	9.0353	9.2795	9.0456	8.3077	4.7284
TCGA-55-8092-01	154	1	10.508	7.4327	7.6234	8.7006	6.9412	10.0325	6.8881	8.2645	10.0325	11.0099	0	6.4992	0	7.96	4.3312	9.505	9.8943	9.749	9.2143	4.2322
TCGA-55-8094-01	541	0	9.8816	8.2024	6.7725	9.5315	1.1323	9.514	5.4953	7.9528	9.5117	11.5871	0	1.9556	0	8.7265	3.2241	10.1699	10.659	9.8725	8.6278	4.888
TCGA-55-8096-01	719	1	10.891	8.4456	6.4853	9.587	7.0611	10.0073	6.8666	7.6163	9.5905	10.1175	0.9151	7.4949	0	8.5988	10.2442	8.9584	9.7135	10.044	8.1616	6.0685
TCGA-55-8097-01	476	0	10.452	5.5601	6.8657	8.5944	4.249	9.8557	7.2661	7.6863	9.422	9.5353	0	3.7357	0	8.1499	6.0146	8.3508	9.6411	9.4478	8.1191	5.3866
TCGA-55-8203-01	547	0	10.087	7.3113	7.045	7.4669	6.6739	9.8164	7.0403	7.3067	8.7525	11.0137	0	1.5955	0	8.1907	9.1248	9.267	9.697	9.2585	8.3095	3.7588
TCGA-55-8204-01	515	0	10.656	8.7761	8.0297	8.0171	6.9587	9.7426	7.055	7.7158	8.4807	10.8192	5.1864	10.6211	8.1231	8.3867	8.1208	9.566	9.4301	8.9794	8.3821	2.0769
TCGA-55-8205-01	599	0	11.4	7.6303	6.5724	7.773	7.5353	9.3996	6.8991	8.0234	8.1052	9.3549	0	1.8204	0	8.6012	9.3948	9.2871	9.833	8.8461	7.9698	2.4411
TCGA-55-8206-01	888	0	10.292	5.5464	7.0171	9.3745	3.6459	9.9955	7.2859	8.0943	9.5249	9.6768	0	1.8884	0.881	8.2578	10.8958	8.2438	9.0768	9.0237	8.339	6.5641
TCGA-55-8207-01	977	0	9.6316	6.902	7.0166	9.3437	5.2635	9.672	6.8553	7.7478	8.5131	10.0131	1.0783	3.4407	4.2317	7.9243	7.7008	8.3363	9.2693	9.8865	8.2057	5.0429
TCGA-55-8208-01	674	0	10.534	9.392	6.9061	9.1026	9.1147	9.4423	6.7992	7.9423	8.8869	10.6649	4.9697	11.2276	8.0891	7.5836	7.6219	8.9886	8.6794	8.6812	8.6648	5.702
TCGA-55-8299-01	469	1	10.011	9.5084	7.5983	7.1927	7.766	9.6854	6.3592	7.8634	8.8669	10.5232	0	2.5229	0	8.5789	7.8945	9.1493	10.0306	9.0343	8.8485	3.3635
TCGA-55-8301-01	534	0	10.751	8.1087	7.3719	8.0503	6.7958	9.2466	6.9938	7.7638	9.8922	10.4416	0	0	0	7.9759	5.3576	9.927	9.7538	9.3895	8.6366	4.9187
TCGA-55-8302-01	478	0	11.979	9.1165	7.4697	8.6784	5.62	9.9311	6.1565	7.1236	9.7378	9.1625	0	2.0745	0	8.1327	9.3963	9.1778	10.0322	9.6168	8.2103	4.533
TCGA-55-8505-01	440	0	10.835	9.5604	7.5099	7.8283	5.0104	9.5687	6.2998	8.0847	9.0551	10.0401	7.3576	13.16	7.7382	8.4261	9.3188	9.945	9.8815	9.9732	8.7795	6.7969
TCGA-55-8506-01	11	0	10.429	9.3969	7.3827	9.3246	5.5598	8.4068	5.1435	7.465	9.657	12.0883	0	3.6808	0	7.7889	7.0709	9.2559	9.3234	10.4037	7.9487	0
TCGA-55-8507-01	418	0	10.279	7.5692	6.643	8.108	4.6639	8.9388	7.4532	7.6093	9.7514	9.5169	0	2.5992	0	7.3469	6.4346	8.2037	9.4358	9.8091	7.807	2.6806
TCGA-55-8508-01	617	0	10.64	8.2431	6.9422	7.081	4.2904	9.1736	7.1811	7.2992	8.8115	11.0674	0	3.6544	2.4124	7.46	7.4887	8.6839	9.8615	9.7651	8.4228	3.423
TCGA-55-8510-01	539	0	11.301	8.3174	6.8525	10.4544	8.0114	9.9087	6.1574	7.2378	9.7342	9.5661	4.5148	12.7039	8.8516	8.0352	8.4455	9.1537	9.6717	9.6243	8.5387	5.6069
TCGA-55-8511-01	552	0	11.146	10.9559	6.3309	7.467	8.6942	9.9075	5.2655	7.5612	10.4653	11.8091	0	3.1929	0	6.9634	9.943	8.5364	9.6676	9.6423	8.3783	2.6847
TCGA-55-8512-01	607	1	10.164	7.1829	8.0054	10.2247	3.3343	10.1024	7.2351	7.551	9.5359	10.1862	5.4574	9.6467	5.6375	8.1753	7.0321	8.5757	10.1629	9.8172	8.4731	2.0727
TCGA-55-8513-01	791	0	10.673	6.6252	7.6223	9.3462	5.7854	10.1629	6.4438	7.1264	9.272	10.294	5.6798	8.3341	4.3882	8.3185	8.2812	9.1359	9.614	9.2082	9.0577	4.1144
TCGA-55-8514-01	520	0	12.119	7.6515	5.3989	8.8221	3.1279	9.8621	5.8032	8.141	9.5644	11.5506	0	1.5536	0	8.0975	8.9798	9.184	9.8205	10.1593	8.529	0
TCGA-55-8614-01	536	0	9.9629	8.6774	6.4613	8.1932	5.5894	9.3837	6.9346	7.4471	9.7519	9.6641	5.6614	12.1666	8.1218	8.3263	6.7531	7.9324	9.896	9.3437	7.9654	2.0507
TCGA-55-8615-01	446	0	10.329	6.9666	5.9909	9.2804	3.6741	9.7253	7.4273	7.5143	10.2805	10.8261	0	1.3809	0	8.7406	7.8581	9.0481	9.8273	10.4511	9.2478	3.7333
TCGA-55-8616-01	48	0	10.703	6.63	7.4259	8.8974	3.8734	9.5717	7.2253	7.0704	9.4328	10.5871	0	4.8348	3.3192	8.7625	8.126	9.0694	10.2053	10.1833	8.4991	5.373
TCGA-55-8619-01	416	0	10.303	6.9343	7.4067	9.476	6.062	10.0208	6.5029	7.6577	9.2804	10.3153	4.7498	6.679	2.762	7.9161	8.5945	8.7607	9.5806	9.4665	8.6666	3.4464
TCGA-55-8620-01	375	1	11.369	7.2639	6.9783	7.2272	5.9753	8.6108	6.9077	8.0581	8.1845	9.1476	1.9455	6.238	3.5837	7.685	5.0247	10.5576	9.9221	10.2223	8.1242	3.3958
TCGA-55-8621-01	515	0	10.142	8.4226	7.4117	9.9011	5.7558	9.8617	6.7424	7.4163	9.5298	9.6937	1.7075	5.7349	1.7028	7.9414	7.642	8.7752	9.7522	9.3112	9.1654	3.8999
TCGA-55-A48X-01	689	0	9.672	7.128	7.92	8.441	6.3339	9.1791	6.4989	7.5857	9.2744	9.9105	0	5.1969	0	8.0007	7.7779	8.7584	9.7478	9.1017	8.6485	4.9225
TCGA-55-A48Y-01	630	0	10.95	8.3373	6.2179	7.2603	8.0594	9.3306	6.488	7.397	9.2383	10.9343	5.389	12.8695	8.7331	8.4501	5.9153	9.0467	9.7971	10.1637	8.68	2.379
TCGA-55-A48Z-01	651	0	10.466	8.7819	6.0908	8.5849	6.4028	9.4226	6.9262	8.2581	8.7977	9.8452	0	1.7667	0	7.7243	8.8751	9.3168	9.1836	10.3585	8.9158	4.8273
TCGA-55-A490-01	99	1	11.418	8.0771	6.9161	7.838	3.9325	8.1138	5.6777	7.2437	9.3894	10.2436	0	0	0	7.6946	5.4851	7.9513	9.0945	9.8002	7.0591	5.6497
TCGA-55-A491-01	626	0	10.785	8.5718	6.7777	6.3293	6.7398	9.8204	7.529	7.6404	9.7939	9.9997	0	3.6607	0	7.3811	8.7511	9.175	9.5386	9.442	6.9886	5.1675
TCGA-55-A492-01	596	0	10.174	5.7556	8.3045	11.089	7.6026	9.0045	6.9058	6.8798	11.7281	10.2018	0	9.499	5.7258	8.0058	5.589	8.5564	10.5673	10.6345	9.9122	4.8236
TCGA-55-A493-01	28	0	10.965	9.069	7.0168	9.6065	7.1797	9.0349	6.3135	7.7693	8.707	10.0181	0	1.7571	0.844	7.8504	10.3326	9.932	9.4261	9.7756	8.3478	3.208
TCGA-55-A494-01	481	0	10.384	7.7925	6.0804	7.8527	3.0171	8.5121	6.4137	7.2416	9.6117	10.0175	0	0	0	9.2397	9.2866	9.2481	10.8947	9.3682	7.1678	7.1899
TCGA-55-A4DF-01	614	1	12.507	8.2182	7.7989	8.5667	6.8594	8.7911	7.5133	8.8061	8.5998	10.7973	5.7287	11.9917	8.7253	7.7392	6.1364	9.9706	9.4263	9.2281	8.1162	5.1568
TCGA-55-A4DG-01	608	0	9.4168	6.405	7.0569	10.3314	4.8291	10.3178	6.8178	8.4562	9.1789	10.8575	0	2.2302	0	8.7457	8.6618	9.3165	9.3565	9.2152	8.6674	6.6385
TCGA-55-A57B-01	546	0	9.347	7.6434	7.8192	9.107	6.5827	10.507	6.8835	7.5374	9.5308	9.2442	0	3.4375	1.99	8.4429	9.4179	8.8054	10.793	9.479	8.4118	6.1674

TCGA-62-8394-01	139	1	11.215	7.5624	6.8305	8.4947	5.716	9.2591	6.3591	8.4213	10.1999	10.5493	2.685	7.6105	6.45	8.7994	8.9033	9.3702	9.9242	10.6054	8.1717	7.546
TCGA-62-8395-01	1216	0	9.3793	6.3655	8.3907	10.1682	5.4874	9.3953	6.9	7.161	10.6637	9.5544	3.9053	6.1984	3.0344	8.6026	7.6823	8.0971	10.2823	9.9736	8.8029	6.7956
TCGA-62-8397-01	1289	0	9.9049	6.5714	5.2794	9.9005	2.7739	11.1239	6.6686	8.1508	10.1377	8.9282	2.4078	8.2922	0	7.9862	7.8097	8.0027	8.8932	9.4152	9.0444	6.7094
TCGA-62-8398-01	444	1	11.335	7.8499	7.361	8.8942	7.5071	9.9933	5.8499	7.6912	9.8904	10.2982	11.6522	12.7065	7.8507	8.4576	8.1706	8.9963	10.4114	9.5768	8.3288	8.1421
TCGA-62-8399-01	2696	0	10.085	6.9475	6.6304	8.1103	5.7229	9.0105	7.2999	7.491	8.8124	10.4352	1.0233	3.3068	0	8.8707	7.8034	8.2387	10.216	9.4558	7.384	3.9773
TCGA-62-8402-01	1498	1	10.353	8.6609	7.2888	7.6263	7.7169	9.0394	6.2174	7.5218	8.9955	9.8013	0	0	0	8.5824	6.2269	10.036	10.2888	9.5072	7.6999	6.4465
TCGA-62-A46O-01	1454	1	11.714	5.9466	6.3739	7.0863	1.0805	8.2197	5.3623	8.8377	9.7924	11.1301	0	2.8863	1.0652	8.5139	7.1891	9.5141	8.7767	9.7054	8.881	3.9535
TCGA-62-A46P-01	594	1	9.7362	5.2706	7.03	9.5432	5.5734	9.3221	5.9693	6.6146	10.8302	10.9907	0	2.2508	0	8.9708	6.2657	9.502	9.726	10.3261	8.7964	4.849
TCGA-62-A46R-01	1725	1	11.185	7.2147	8.6203	8.8524	5.9314	10.204	6.9722	8.2721	9.3371	10.1762	1.4677	6.221	1.8763	7.6445	7.1091	10.0437	10.0306	8.2698	9.3293	5.673
TCGA-62-A46S-01	1653	1	10.096	5.5448	6.8656	9.8129	4.5824	10.1043	6.5771	7.3013	9.4029	10.7795	1.3652	3.0451	0	8.5775	7.5994	9.0351	9.5503	9.5238	8.9215	5.4243
TCGA-62-A46U-01	2067	0	10.241	8.6318	7.5258	9.6891	7.2735	9.8774	6.7531	8.1212	8.4595	10.524	3.9523	11.2359	7.243	8.0581	9.2491	8.9574	9.5209	9.4111	8.7179	6.2598
TCGA-62-A46V-01	2199	0	10.195	7.5872	8.1384	8.6385	4.7176	10.8237	6.1593	7.4851	9.5039	10.7861	0	1.9162	0	8.1056	10.1127	8.2157	10.2659	9.1185	8.5438	5.9781
TCGA-62-A46Y-01	414	1	10.568	6.5066	7.9131	8.5252	5.5427	10.622	7.048	7.5193	8.4914	10.3462	0	5.4273	1.8161	8.6796	10.9447	9.5716	10.101	9.4894	8.6293	3.1731
TCGA-62-A470-01	1194	1	11.392	6.1795	6.1817	10.5717	5.5789	8.509	7.9501	7.7988	10.3184	10.4701	5.1575	13.3491	9.6142	8.7259	6.2028	9.7052	10.0946	9.8547	8.4794	5.0981
TCGA-62-A471-01	1246	0	12.005	8.746	6.6707	8.9031	6.4754	8.3501	5.1404	8.8174	10.1209	10.3491	3.1625	9.8716	5.575	8.6892	8.4315	9.9834	9.2263	10.1679	9.9464	7.2853
TCGA-62-A472-01	910	0	10.585	8.3058	8.0564	8.8071	5.7048	9.9755	7.3243	7.1877	8.3921	9.5378	0	2.2698	0	7.9936	6.9267	9.8514	10.0285	8.9463	9.4349	7.4331
TCGA-64-1676-01	1728	0	10.923	8.7143	7.504	9.9719	8.999	9.9917	7.4216	8.0535	8.2001	10.9112	0	0.7712	1.6157	8.7732	10.3204	8.4203	9.2162	10.7277	9.7344	4.3505
TCGA-64-1677-01	628	1	10.771	7.7565	7.4824	7.6837	7.6368	8.8124	8.3092	8.1008	8.8368	10.4334	0	2.6939	0	8.8976	6.4858	9.4582	9.6956	9.8283	8.1179	3.2858
TCGA-64-1678-01	1189	0	10.941	7.2091	9.0937	9.8244	5.1175	7.6473	8.9418	8.0299	9.0905	10.5547	2.1557	11.6879	8.7416	9.2763	4.7163	9.4094	9.0655	11.3015	9.3732	0
TCGA-64-1679-01	2488	0	11.151	8.84	6.2676	8.0151	6.082	9.5969	7.4305	7.5222	9.3803	10.9286	4.6344	11.426	6.3421	8.6079	9.2412	8.4777	8.5125	9.7636	8.1801	3.2788
TCGA-64-1680-01	1126	0	10.122	4.1557	8.1932	9.0563	2.803	9.9715	6.9922	7.7263	9.0482	10.2884	0	3.4885	1.4899	9.6977	10.459	9.2208	10.0107	9.6665	9.3667	3.6958
TCGA-64-1681-01	1167	1	11.112	6.5192	7.5269	9.9973	5.5178	10.3399	7.364	7.7383	9.0746	10.0444	1.6238	4.9798	0	9.1978	6.9985	9.455	10.2498	10.1077	8.64	7.5227
TCGA-64-5774-01	2676	0	10.557	7.5071	5.8223	7.6328	7.0549	8.5788	5.8718	7.8619	9.0454	10.4639	0	0	0	8.4302	9.5146	9.0758	9.6196	9.4839	8.1408	1.7397
TCGA-64-5775-01	62	1	12.802	10.3325	7.6984	5.3214	4.7039	9.5092	6.0027	7.5297	8.6156	11.422	0	1.5166	0	8.0975	8.5546	8.3947	8.7951	8.8191	7.7939	0
TCGA-64-5778-01	1305	0	11.205	7.3412	8.4005	11.1174	6.4539	9.2159	7.8282	8.0978	8.5946	10.3935	0	3.1349	0	8.5613	7.467	9.1634	8.9285	10.9085	10.409	5.4787
TCGA-64-5779-01	864	0	12.608	8.4184	7.6926	6.6203	6.1574	8.9085	7.8412	7.6428	8.7475	9.9672	2.0954	0	0	8.384	1.7887	9.0399	8.9757	8.8303	8.2966	5.7158
TCGA-64-5781-01	1559	0	10.463	9.4263	7.0113	10.0236	7.088	8.9584	5.7986	7.4535	8.3276	11.5385	4.8796	11.5576	8.0919	7.6648	6.4458	10.6125	9.7322	11.1906	9.9297	3.5768
TCGA-64-5815-01	866	0	10.69	9.1115	7.1776	9.8382	8.3252	9.8493	5.8071	7.0803	8.3772	10.4448	0	4.0627	0	8.5322	11.2684	8.7505	9.3835	9.3352	8.8373	3.5847
TCGA-67-3770-01	610	0	10.179	7.7265	7.3196	10.7505	8.5725	10.023	6.7057	8.1962	9.7639	9.4556	0	5.7691	0	8.3905	8.3743	9.0502	10.093	11.3756	8.9247	8.1105
TCGA-67-3771-01	610	0	9.5979	8.9036	6.1507	10.8208	6.2587	8.8787	6.7787	7.1535	8.8985	10.5639	2.9345	11.099	7.7681	8.0596	6.9404	8.563	9.3044	9.256	8.2289	6.7085
TCGA-67-3772-01	573	0	10.625	7.1613	7.1547	8.9179	7.2095	9.6931	7.5709	8.1246	8.5329	11.1303	0	5.6662	0.5632	8.302	7.7447	9.5891	9.8173	11.014	9.1814	6.4911
TCGA-67-3773-01	427	0	9.6375	6.7811	6.768	9.7588	5.8968	9.9995	7.5471	7.8402	8.453	9.3086	0	2.6179	0	9.1738	2.0297	8.6016	9.8224	10.233	9.7502	3.8441
TCGA-67-3774-01	385	0	10.159	6.3812	7.2272	10.101	6.1932	9.597	7.2931	7.9022	9.4387	10.7922	1.4391	8.4424	4.2627	8.3854	4.955	7.9099	9.535	9.54	8.1099	2.611
TCGA-67-4679-01	448	0	9.7597	5.6047	7.4272	10.1402	4.1408	9.6485	7.1513	7.3521	8.86	10.7975	0	2.7523	0	7.9392	6.656	7.956	9.6434	9.633	8.5904	2.1515
TCGA-67-6215-01	174	0	10.287	6.2968	7.2451	9.2899	3.0787	10.5475	6.2238	7.7467	8.7218	11.2509	1.0146	6.7909	3.6126	8.3227	10.2491	8.638	9.6931	10.2259	8.5561	5.1851
TCGA-67-6216-01	141	0	10.064	6.5174	7.7613	9.1881	4.4348	10.485	6.2673	7.6368	9.0441	9.6747	0	0	0	8.1462	9.499	9.3056	10.1348	9.5338	8.8697	4.9292
TCGA-67-6217-01	422	0	10.307	6.329	7.7284	9.1363	5.2024	9.7038	7.2285	7.9627	8.8889	10.5061	0	1.7212	2.7176	8.8324	8.1301	9.482	9.7234	9.7077	8.6443	6.0152
TCGA-69-7760-01	202	0	11.002	6.0244	6.5608	10.4494	6.497	11.1808	7.5671	7.9634	8.5344	9.9196	0	2.7135	0	8.5441	5.6175	9.9735	9.6289	9.2541	8.1809	6.5934
TCGA-69-7761-01	186	0	10.635	7.2696	6.9968	8.1623	6.4487	10.4218	6.864	9.1747	8.3813	10.3425	4.1699	8.2294	4.4063	7.9923	9.9934	10.0252	9.301	8.5693	7.841	7.3431
TCGA-69-7763-01	690	0	9.9021	6.6936	7.3092	9.4735	7.4688	9.5335	6.8686	7.0339	9.1036	10.0413	0	2.7815	1.3631	9.0628	5.9788	8.2602	8.8012	9.1221	8.8611	2.1848
TCGA-69-7764-01	414	0	9.6852	4.5427	7.2815	8.2099	3.3623	9.6199	7.8399	7.2809	9.1581	10.156	0	1.218	0	8.3514	4.1357	7.8441	8.7726	9.6783	8.9533	7.1855
TCGA-69-7765-01	165	0	10.017	7.9685	7.631	8.4039	5.4412	9.9381	7.1631	8.5015	8.1769	11.3854	0	3.8852	0	8.7051	7.8577	9.2503	9.0077	8.7767	8.9212	4.474
TCGA-69-7973-01	230	0	10.507	6.2861	7.9369	7.9295	4.9509	8.9568	6.3803	7.0109	9.0398	10.3099	0	0	0	8.1871	8.6829	8.4664	9.5131	9.6925	7.3895	2.8973
TCGA-69-7974-01	184	0	11.794	9.2017	6.8349	8.9727	6.5928	10.083	6.6361	7.3612	8.9212	10.6004	2.4831	9.5853	4.1763	7.5128	10.323	8.6696	9.9997	9.8694	8.2411	6.4054
TCGA-69-7978-01	134	0	10.514	8.4628	6.9604	8.1315	7.3071	9.3701	6.8704	7.5722	8.1707	10.3345	0	3.9814	0	8.5025	10.9805	8.5709	8.9134	8.7895	8.7774	4.7309
TCGA-69-7979-01	408	0	9.6528	9.027	8.4715	9.5506	1.731	8.3616	6.1879	7.1924	9.9896	10.9433	4.5011	12.2518	8.5785	8.0537	2.3389	8.3398	9.4744	10.1261	7.8294	0.9029
TCGA-69-7980-01	362	0	10.598	6.7488	7.0375	9.5028	5.3399	9.6781	6.8093	7.1973	9.2907	9.9424	0	4.4416	0.8669	8.0244	8.5748	8.7666	9.0418	10.1081	8.0261	6.9446
TCGA-69-8253-01	426	0	10.213	6.5996	7.7327	9.8336	3.8791	9.9263	6.5347	7.7916	9.875	10.9455	0	6.9829	0	9.2936	9.1282	8.2913	9.9519	9.8802	9.3975	1.4406
TCGA-69-8254-01	409	0	10.182	5.6996	7.5963	9.557	5.1478	9.7254	6.2111	7.2157	8.7878	11.3303	0	5.7378	2.9404	8.001	9.2	9.1371	10.1933	9.3988	8.9772	7.7288
TCGA-69-8255-01	129	0	10.926	8.5227	6.3854	7.5089	5.7016	9.1511	5.4774	8.1167	9.2392	10.5693	8.6928	2.8982	5.6583	8.5769	6.6876	10.1579	10.078	10.23	8.401	3.6924
TCGA-69-8453-01	813	0	10.633	7.4398	7.2953	9.3903	7.8149	10.193	6.7642	7.5556	9.4039	9.8163	5.8559	9.0763	6.1299	8.0898	7.607	8.9489	9.4569	9.5676	8.5386	6.0188
TCGA-69-A59K-01	522	0	10.037	6.8034	6.8434	10.004	4.8417	9.8471	6.6529	7.3245	7.4916	10.0246	6.7618	11.7909	7.8192	7.952	4.1379	7.4617	9.8701	8.8697	8.4398	2.0268
TCGA-71-6725-01	256	0	10.849	7.3624	7.9628	8.7198	4.4115	10.7526	6.8942	8.1063	9.2298	10.9094	0	0	0	8.3271	9.6231	9.3765	10.136	10.3859	8.6306	0
TCGA-71-8520-01	210	1	9.875	8.7664	7.1861	8.9767	5.9193	10.2319	6.1383	7.8519	9.3069	10.6895	0	5.3241	0	8.3914	8.3994	9.7246	9.9726	9.8798	7.9419	7.1534
TCGA-73-4658-01	1600	1	11.569	8.4935	7.4259	9.7929	6.4289	9.638	6.5753	6.9423	8.952	10.1904	0	3.567	1.9846	8.0155	11.6635	8.7567	8.6615	9.7511	7.9125	1.9549
TCGA-73-4659-01	711	1	10.675	7.5943	7.2065	9.8375	6.324	9.5632	6.6702	7.541	9.2613	10.5716	3.4882	11.6704	7.0936	8.5534	6.4372	8.5123	9.6705	9.7805	7.9618	2.8315
TCGA-73-4662-01	2515	0	10.175	6.7157	7.7319	10.2441	6.5664	9.7524	7.6219	7.916	7.8762	10.2438	0	1.6368	0	8.226	6.2571	8.5692	9.8284	9.741	8.3842	3.6423
TCGA-73-4666-01	800	0	11.451	7.7154	7.9736	9.3062	5.816	9.6549	5.9686	7.6289	8.8837	9.8507	0.9302	9.6376	5.1747	8.1367	10.1531	8.3374	10.4566	9.7682	8.2035	1.1748
TCGA-73-4668-01	467	0	11.004	9.1428	7.4524	7.2272	3.8113	10.0715	6.8162	8.3551	8.5381	11.3145	0	2.934	0	8.4536	8.3156	8.9859	9.2766	9.2488	7.7183	0.9884
TCGA-73-4670-01	131	0	11.491	9.3715	6.8128	8.8991	6.7893	8.9629	6.4401	8.5494	9.5642	10.5914	0.8661	3.573	1.0864	8.6092	9.3519	9.5252	10.6919	10.6019	7.7702	2.5656
TCGA-73-4675-01	922	1	10.394	8.6481	8.5599	10.8642	5.2069	9.6098	5.7122	6.8839	9.8491	10.4487	3.8792	13.5981	9.1086	8.3431	8.3541	9.5645	10.2167	10.4323	9.7535	4.2217
TCGA-73-4676-01	281	1	12.018	7.5065	7.4758	9.0875	6.6161	8.7936	6.4606	7.5327	8.4174	11.4468	0	5.3115	1.2589	9.2108	7.0314	9.1796	10.05	9.5835	8.508	6.8715
TCGA-73-4677-01	38	1	9.9117	5.2488	7.45	9.0021	5.6482	9.6284	7.4846	7.8345	9.7803	11.2668	0	6.1682	0	8.5344	6.8873	8.2397	10.1572	9.8325	8.8923	2.2505
TCGA-73-7498-01	1189	0	9.3988	6.4133	8.3488	9.666	4.5284	9.2767	7.5895	7.0215	10.3146	9.7506	0	2.0944	0	7.4153	7.5834	8.0622	10.3214	9.6981	9.0001	2.494
TCGA-73-7499-01	1531	1	10.324	7.7436	8.2197	9.8198	7.8667	9.002	7.0607	7.7371	9.4557	10.1303	0	3.7268	1.9262	8.5653	7.0837	9.4357	9.562	10.5102	9.1666	7.9125
TCGA-73-A9RS-01	340	1	9.9621	6.8103	5.6023	8.0503	3.1574	9.2716	4.524	7.8989	9.7835	11.5105	0.911	0	0	8.0443	6.3101	9.1827	11.0782	9.9506	8.0551	0
TCGA-75-5122-01	0	0	10.662	7.9761	7.9864	9.4494	6.2378	9.9553	6.8485	8.211	8.5279	9.3723	0	0	0	7.9249	8.0545	8.9714	9.0051	9.6632	8.6114	5.3192
TCGA-75-5125-01	2027	1	10.724	7.7005	7.6923	9.0635	7.066	9.4806	7.6876	6.9323	8.6437	10.4009	0	2.6217	0	8.2581	6.5273	9.1313	9.2618	9.4383	8.3792	3.5637
TCGA-75-5126-01	0	0	11.176	7.2916	7.4562	8.6606	7.0912	9.8326	6.6514	7.4672	8.5607	9.8699	0	4.7358	0	8.011	11.7414	9.0619	9.1042	9.5373	8.4435	5.4329
TCGA-75-5146-01	2368	0	10.647	5.1567	8.3979	9.2738	6.1386	8.6642	8.4528	7.6602	10.0341	11.0837	0	2.5756	0	8.3248	7.9685	8.2258	9.8711	10.2948	8.6667	5.3959
TCGA-75-5147-01	1333	0	10.928	7.8442	7.1405	9.122	8.5067	9.5158	7.6033	7.8865	8.4519	11.111	0	7.2929	5.0795	8.7335	8.3936	9.2864	9.6415	10.1211	9.0841	7.2167
TCGA-75-6203-01	0	0	10.729	6.418	7.708	9.8603	2.358	10.7439	7.0853	7.1842	9.219	9.5599	0	0	0	7.6171	10.6389	8.6778	9.633	9.3464	9.1297	5.772
TCGA-75-6205-01	0	0	10.594	8.9998	7.5541	9.2494	7.932	10.3669	6.7856	7.1973	8.0974	9.5396	4.7168	12.7315	9.157	8.2933	11.6798	10.0141	9.2271	9.1731	8.0885	4.357
TCGA-75-6206-01	2590	0	9.5805	5.7066	6.988	9.1158	3.4685	9.701	7.4435	7.4642	9.8133	10.17	0	1.7989	0	8.3251	6.3159	8.3051	9.2738	9.5746	8.5133	6.3608
TCGA-75-6207-01	0	0	11.382	6.95	7.1011	7.8783	5.1939	9.3824	6.1653	8.1898	8.9781	10.1095	0	2.5448	0	8.2721	7.0539	9.3785	10.3592	9.6855	7.9872	3.2252
TCGA-75-6211-01	0	0	10.668	7.2603	6.793	11.1137	5.83	9.1805	6.899	6.6288	8.9097	11.2312	6.8722	13.3945	8.8637	8.1549	3.8226	9.6902	9.971	9.1624	8.178	5.6528
TCGA-75-6212-01	1516	1	10.115	5.6681	7.7233	8.5984	4.3006	11.3506	6.1208	7.8659	10.1906	9.5113	0	4.0088	0	9.0334	9.7097	8.2599	9.8183	9.3858	8.8186	5.2925
TCGA-75-6214-01	1115	1	10.964	9.3634	6.0812	7.2153	5.493	8.4966	6.9994	7.2094	8.0642	10.1933	4.1437	11.0242	8.818	8.3431	9.1436	9.343	9.9061	9.8426	9.8766	5.1046
TCGA-75-7025-01	3305	0	10.093	7.3016	7.1401	9.8022	5.0176	10.4359	6.7401	8.3348	9.2453	9.5864	0	6.1512	3.396	7.5906	8.3263	9.366	9.7308	9.6159	8.9752	6.6668
TCGA-75-7027-01	3059	0	11.514	9.1087	6.828	9.5518	7.5324	9.2455	6.6771	8.4327	8.5654	10.3069	5.8353	12.5634	7.6768	7.4027	6.9033	9.9275	10.0629	9.3919	8.1608	5.5907

TCGA-75-7030-01	0	0	9.879	9.6929	7.2844	8.1514	6.3061	10.655	6.7863	7.7865	8.3941	10.2275	4.6011	8.293	4.8657	8.3851	8.4975	8.7276	9.4618	9.2287	8.6845	6.1024
TCGA-75-7031-01	0	0	9.9406	7.1965	6.8064	9.7841	4.8558	9.4265	7.3328	7.9497	9.8342	10.3153	0	11.3906	6.6628	8.7447	6.4072	10.113	9.777	9.9295	9.4709	7.1808
TCGA-78-7143-01	4961	1	10.859	6.8889	7.3223	8.3775	4.6154	9.5011	7.2897	8.3941	8.392	10.0885	0	2.6504	0	7.8418	6.9745	10.5691	9.133	10.0968	8.6008	5.4811
TCGA-78-7145-01	826	1	10.978	6.7216	7.4627	8.408	6.0482	10.1859	7.427	7.3259	8.8477	10.6935	0	6.1414	6.3149	7.9895	7.6313	9.0904	8.9649	8.8694	8.1781	6.1814
TCGA-78-7146-01	173	1	10.942	6.6251	6.0034	9.5636	6.9985	8.805	7.2265	7.784	8.3765	12.4235	5.4513	11.4642	7.3113	8.4907	8.6635	9.1476	9.1627	8.736	7.7937	4.0376
TCGA-78-7147-01	586	1	10.962	6.1291	7.4067	10.2106	3.2042	9.5743	6.9706	7.9049	9.0845	10.5355	3.0961	11.0437	6.4521	9.0164	9.4426	9.241	10.4875	10.3499	8.6399	6.4312
TCGA-78-7148-01	626	1	10.334	6.4565	7.9307	8.2332	6.7462	9.626	6.2475	6.8955	9.1276	10.4068	6.3309	10.9623	6.8163	8.4484	7.9712	9.1208	10.0173	10.0372	8.5501	3.9409
TCGA-78-7149-01	3940	0	9.6666	6.4902	8.2119	10.2889	5.9069	9.2443	6.6316	7.4668	10.644	10.096	2.7692	14.1065	8.9723	7.7735	8.0172	8.4169	10.9241	10.2509	8.0355	3.9899
TCGA-78-7150-01	666	1	10.444	8.7856	6.6527	9.1464	7.036	8.2611	6.0532	7.8699	8.6716	10.887	4.6481	9.59	4.1064	8.9462	7.8875	9.7822	9.1811	10.4138	8.6136	6.6962
TCGA-78-7152-01	1215	1	10.636	6.5037	7.322	9.797	4.652	9.134	5.5391	7.1225	9.2218	10.4958	0.9953	2.8942	0	9.1035	7.7638	9.1646	9.5424	10.4818	8.5443	3.7083
TCGA-78-7153-01	3635	0	10.74	5.2198	7.4457	7.6742	1.7892	9.6959	6.7196	7.7908	10.7078	10.626	2.4597	2.9274	0	8.3882	5.6995	9.4071	9.8858	10.1417	8.6444	6.3477
TCGA-78-7154-01	593	1	10.706	6.6973	7.7252	9.1894	7.0112	9.8448	6.5342	7.9921	8.2788	10.5036	6.7315	14.3431	9.9812	8.3802	4.4088	10.3436	9.8289	10.2423	8.0022	8.3542
TCGA-78-7155-01	1171	1	10.941	5.9984	7.572	7.5451	4.3289	7.3388	8.8196	8.6491	9.1906	10.9934	0	1.7289	0	8.0835	4.4097	9.8787	8.1672	9.5758	5.7918	0
TCGA-78-7156-01	976	1	10.158	3.7097	7.9413	11.2372	7.7767	9.3476	7.2072	7.5655	10.7106	9.83	0	0	0	9.3338	4.2026	8.545	10.6228	10.2737	9.1937	5.9237
TCGA-78-7158-01	179	1	9.9756	5.7554	7.4767	9.6032	3.217	9.1639	6.722	7.5525	9.954	10.8219	0	2.2432	0	8.8374	8.8137	8.6475	10.3965	10.0235	8.3016	3.0822
TCGA-78-7159-01	1974	0	10.629	7.4947	6.9869	9.1041	5.6763	8.8174	6.9004	7.7541	8.3466	10.9521	0	0.6332	0	8.6691	5.4943	10.1511	8.8332	8.9096	7.2423	2.7026
TCGA-78-7160-01	697	1	10.381	6.9446	8.1808	8.1415	7.6374	9.4552	6.7156	7.5671	9.1047	10.8191	4.4928	11.2784	6.0674	8.7291	7.2756	9.3821	9.5012	9.0576	9.4526	6.2494
TCGA-78-7161-01	291	1	10.073	6.8899	8.2499	8.4347	4.162	9.4705	7.4616	7.2893	9.519	10.5001	0	0.8251	0	8.1134	7.362	8.9476	9.5461	9.5645	8.3315	4.3557
TCGA-78-7162-01	3169	1	9.925	6.0022	7.2796	10.2262	6.4366	9.5786	7.0992	7.0323	10.1092	10.1287	2.0067	11.7527	7.5187	8.7971	4.9372	8.2956	10.114	9.2463	8.6139	5.2182
TCGA-78-7163-01	7248	0	10.794	4.2303	8.0042	9.9718	2.2528	12.5192	7.1116	8.1848	8.133	10.2468	0	1.842	2.2987	6.6081	11.0017	9.5634	11.3416	10.0893	10.0507	4.9442
TCGA-78-7166-01	258	1	10.896	7.8048	8.4016	8.2571	7.0837	9.0934	5.8596	7.6555	10.0509	11.2129	0	6.2067	1.4837	8.8021	9.2346	9.3807	10.318	11.2124	9.9679	5.3303
TCGA-78-7167-01	2681	1	9.0465	5.5647	6.3715	9.7424	4.1887	8.5974	8.4894	7.3615	9.6478	10.2973	5.7285	11.9787	7.5885	7.9348	5.7281	8.421	10.333	9.8803	9.0433	4.6134
TCGA-78-7220-01	807	1	10.154	8.1401	6.7896	8.7748	5.7276	8.3086	6.7068	8.579	9.2537	10.6353	7.9455	10.8547	6.8963	8.4652	6.8681	8.8297	8.8951	10.017	7.7832	6.2702
TCGA-78-7535-01	949	1	10.634	8.0403	7.8584	8.0693	3.1405	9.8191	5.9067	7.3772	9.3614	10.3264	0	2.1769	3.1171	9.0586	5.0437	9.5206	10.6801	11.4622	8.8974	4.5478
TCGA-78-7536-01	244	1	11.536	7.0714	6.0957	5.7473	4.5714	8.3004	7.0778	6.2906	9.7254	11.0174	0	0.5963	0	9.3595	6.8726	10.0163	9.395	9.5679	6.2859	0
TCGA-78-7537-01	1622	1	10.23	7.0612	7.8386	9.2521	7.1154	9.6991	6.2594	6.9629	10.419	11.3216	0	1.4767	0	8.7673	7.8762	8.3702	9.6313	10.3766	8.9312	3.202
TCGA-78-7539-01	791	0	9.9693	6.9959	6.1624	9.8802	5.7788	9.3103	6.823	7.9394	9.273	10.4917	0	1.8683	0	7.9168	6.4382	8.7268	9.2027	9.9285	9.0995	8.2173
TCGA-78-7540-01	1197	1	10.689	7.4457	7.1019	8.9497	1.8684	10.9431	5.1211	6.7947	8.6343	11.0056	9.4127	10.6342	7.6533	8.0269	10.667	10.1324	10.2399	9.8245	9.2252	8.2553
TCGA-78-7542-01	321	1	12.287	10.4491	8.5203	6.983	5.5028	9.954	7.371	7.5662	8.3483	10.8757	0	4.8804	2.5192	8.0166	7.9351	10.1789	10.3701	9.2154	9.1051	1.9217
TCGA-78-7633-01	1528	1	9.6396	5.2453	6.8432	11.1389	4.4028	8.7149	7.6267	6.8384	9.8441	10.4586	0	3.6457	0	8.6936	3.3111	7.8375	10.3104	9.5117	8.0005	6.86
TCGA-78-8640-01	7062	0	9.6676	7.7769	5.6214	8.6274	5.1228	9.7666	4.7055	7.6353	9.6973	11.108	0	0	0	8.7997	7.4211	8.463	8.8894	10.4082	8.4748	2.8912
TCGA-78-8648-01	1209	1	10.36	9.6139	7.1642	8.4705	7.8751	10.1855	6.6593	7.5082	8.6486	9.8845	4.209	8.6843	4.7914	7.7321	4.3828	8.018	9.2415	9.2213	8.7544	2.8302
TCGA-78-8655-01	2360	0	9.4877	8.0159	7.615	8.844	6.4659	9.9009	6.3808	6.8133	9.5633	10.0922	0	4.6477	0	8.576	8.6976	9.2799	10.2294	9.8203	8.414	1.536
TCGA-78-8660-01	321	1	10.053	9.5688	6.6595	8.3663	6.9208	9.3015	5.5391	8.164	9.35	10.3559	0	1.737	0	8.1072	8.9013	8.9223	10.0566	10.2001	8.1254	3.8496
TCGA-78-8662-01	3361	1	10.869	9.0523	7.0804	8.1677	3.9598	8.1941	7.0376	8.8022	10.4916	9.9548	0	0.9311	0	8.1477	5.173	9.1058	9.6072	11.7827	8.4773	1.5779
TCGA-80-5607-01	0	0	10.552	7.1014	8.2659	8.72	6.588	9.739	6.182	6.7163	9.0759	10.376	0	2.9087	0	9.0272	9.4628	9.4924	9.879	9.5931	8.3363	5.2017
TCGA-80-5608-01	2832	0	10.788	5.8293	7.2044	7.7232	2.8843	9.2245	7.0925	7.3399	9.7337	10.7196	0	5.1457	0	7.6384	6.278	9.1749	9.7427	9.6174	8.2355	5.2024
TCGA-80-5611-01	2595	0	11.083	8.3168	6.3953	8.5615	6.8628	9.1194	6.3545	7.4958	8.7931	11.0077	1.3628	9.8829	6.4956	7.9763	6.9499	9.0498	10.0583	10.1368	8.7168	2.8342
TCGA-83-5908-01	824	0	11.895	8.3868	6.905	6.3463	8.06	9.7604	6.3661	8.06	8.7106	10.0488	0	3.3637	1.4906	8.0292	9.3095	9.5431	9.3466	8.5246	7.2224	4.9561
TCGA-86-6562-01	376	1	10.463	8.7225	7.1988	8.3667	6.4654	9.8872	6.7213	7.1976	9.3034	11.2369	0	1.051	0	9.0009	9.0916	9.1403	9.897	9.1621	8.5199	3.1763
TCGA-86-6851-01	179	0	9.3028	7.1963	6.651	8.8985	6.8799	9.1962	6.958	7.9169	8.6825	10.7865	2.6084	9.6129	5.6324	7.2743	7.5577	8.3835	9.503	8.9165	8.9683	5.469
TCGA-86-7701-01	947	0	10.872	7.2631	6.9155	7.1411	7.5345	8.9114	7.3605	7.6972	8.5099	10.6726	0	1.8783	0	8.4804	5.0732	9.7461	9.7965	9.0669	9.0149	5.283
TCGA-86-7711-01	1046	1	11.625	8.9401	5.9326	9.051	9.6252	8.473	4.433	7.4162	8.8036	11.8691	6.3896	12.3238	7.6168	7.8266	8.0417	7.9571	9.3362	9.2136	8.3651	3.1612
TCGA-86-7713-01	1157	0	10.164	5.565	5.7028	9.1034	4.9116	8.5366	7.7548	7.3622	9.734	10.3829	0.6012	0.5736	0.726	8.6077	7.5236	9.0734	9.7947	9.5551	6.5667	2.1266
TCGA-86-7714-01	625	1	9.7435	4.7222	8.2504	9.2965	3.9163	10.3984	7.5403	7.4656	8.3074	9.6952	1.8035	2.5827	0	9.4956	9.1177	8.7116	9.5087	8.9441	8.3946	5.4361
TCGA-86-7953-01	997	0	10.698	8.2213	7.2767	7.8191	6.8008	9.3569	7.0908	7.4707	8.8959	10.0282	0	1.4212	2.1617	8.2373	11.459	8.6812	9.4579	9.6189	9.5793	3.1179
TCGA-86-7954-01	605	0	10.492	7.4616	6.6974	10.7507	7.2523	9.4143	7.2947	7.4726	8.9403	10.8905	0	6.4911	3.1106	7.5943	9.3776	9.308	9.8408	8.8875	8.1118	7.0917
TCGA-86-7955-01	1072	0	11.481	6.8526	7.061	7.0382	2.683	9.7382	6.8209	7.5712	10.5971	10.1321	0	0.7072	1.5719	8.5443	6.0783	9.3796	10.7634	10.2485	8.1688	2.8124
TCGA-86-8054-01	1148	0	9.8639	6.2289	6.3381	7.1518	4.4361	8.5469	7.5711	8.5065	9.2833	10.8138	0	0.7365	0	8.4207	5.2339	9.726	9.1063	9.2987	8.0485	0.7365
TCGA-86-8055-01	124	1	10.17	8.8062	7.3214	8.6285	7.5035	10.6701	7.0415	7.3221	8.6716	10.0176	2.6666	11.3705	7.3697	8.5614	9.0671	9.5903	9.5212	9.0202	7.5991	5.5103
TCGA-86-8056-01	139	0	10.042	5.6848	6.2889	9.715	5.8876	10.2036	7.4816	8.1622	8.5998	12.1028	5.7698	12.6156	8.0313	8.9544	7.8606	8.5447	8.7914	8.6763	8.6873	7.8058
TCGA-86-8073-01	740	0	10.662	6.5683	7.3454	8.2632	6.5031	9.4704	7.0919	8.0664	8.962	10.5943	4.4153	6.8289	6.3727	7.6562	5.1018	8.7878	9.6459	8.1297	8.8854	5.9097
TCGA-86-8074-01	24	0	11.033	8.7411	8.8192	7.3605	6.7673	10.606	7.0423	8.4753	8.9511	10.6316	2.5046	10.9305	6.2207	9.2678	9.0321	9.039	8.9645	8.8077	8.6803	4.5773
TCGA-86-8075-01	694	1	10.534	9.0253	6.2139	7.0142	7.2865	9.2947	6.9423	7.4674	8.2872	10.3644	0.9389	2.2184	0	8.1946	7.3661	9.2629	9.0162	9.6542	8.0528	5.3674
TCGA-86-8076-01	993	0	10.09	6.9921	7.4393	8.7785	7.735	9.4922	7.1547	7.6508	9.3075	10.9202	0	8.0796	3.021	9.2337	8.8026	9.4267	9.4044	9.7113	8.8763	4.272
TCGA-86-8278-01	944	0	10.114	7.469	7.1811	8.9729	5.2509	10.4646	5.5984	7.9964	8.9706	10.5852	0	2.1625	0	7.3144	11.1854	9.0797	10.0648	9.3269	9.1159	3.176
TCGA-86-8279-01	949	0	10.497	7.3022	6.0453	7.7317	4.6439	10.6654	6.3968	7.7257	9.4419	11.1372	0	0.7924	0	8.7413	3.6839	8.2329	10.3639	9.7649	8.2013	1.6202
TCGA-86-8280-01	701	0	10.528	7.4206	7.8655	8.7758	5.9892	10.0252	7.0026	7.8202	10.3303	10.1725	0	4.8932	0	8.4254	7.25	8.8617	9.4632	9.5822	8.8841	6.5499
TCGA-86-8281-01	0	0	9.8155	5.9557	6.4239	9.5133	5.359	9.4701	6.9037	6.4772	10.2889	10.6803	0	4.6674	0	8.8593	5.2707	7.7837	9.4393	9.5009	8.8946	3.3295
TCGA-86-8358-01	653	0	10.191	7.0662	7.2673	7.877	7.0904	8.5984	7.3113	7.9715	9.1412	10.421	3.135	7.2964	0	7.8273	4.5574	10.6867	8.6253	9.5916	7.3173	3.7654
TCGA-86-8359-01	444	1	9.525	7.2354	6.1745	8.7149	5.227	9.2664	5.5966	7.7431	8.8896	11.0826	0	2.8228	0	8.4896	8.7722	9.2721	8.7003	10.0825	8.6575	4.3534
TCGA-86-8585-01	353	0	11.07	9.1838	7.3863	8.3106	5.9461	9.6782	6.2053	8.5071	9.7147	10.0374	0	2.9292	0	7.9924	8.3696	8.737	10.2417	10.6178	9.1577	2.7036
TCGA-86-8668-01	423	0	9.3138	6.8003	7.2881	9.2917	4.5436	10.0435	6.7468	8.2077	8.3625	9.3814	0	2.6342	0	8.4011	10.154	9.3926	9.8242	10.1062	7.9843	2.2388
TCGA-86-8669-01	938	0	10.614	6.3027	7.6803	8.993	5.5927	9.5764	7.337	8.2212	9.6058	10.1551	0	4.3191	0	8.3012	8.5051	9.1177	8.9513	9.6209	8.8623	7.9652
TCGA-86-8671-01	839	0	9.8178	8.883	7.7343	8.6516	8.4952	10.1464	6.7682	7.2839	9.0898	9.358	0	3.8304	0	7.732	8.6237	8.8351	9.6168	9.4358	8.4952	6.3769
TCGA-86-8672-01	19	1	10.614	9.6184	6.2384	10.0722	6.1496	9.0901	6.3951	8.3731	9.2725	9.9782	0	1.304	0	8.9697	7.0212	9.1011	9.8594	10.3277	8.2439	3.5103
TCGA-86-8673-01	862	0	10.495	8.5732	7.2215	9.7097	7.0485	9.277	5.374	9.9095	9.4647	12.0946	0	0	0	8.2901	7.7169	9.6914	9.3279	9.7817	8.961	5.2682
TCGA-86-8674-01	806	0	10.035	7.2426	7.5347	9.8261	5.9074	8.7777	6.4148	6.6266	10.991	11.2306	0	4.3697	0	9.232	9.6106	8.2182	10.3617	11.0625	9.1559	5.4502
TCGA-86-A456-01	896	0	10.936	7.4621	7.7881	9.8859	7.2796	10.0406	6.9476	7.8887	9.2447	9.8778	0	3.2763	0	8.3396	8.1513	10.4655	9.6525	9.6588	9.0034	7.2005
TCGA-86-A4D0-01	116	1	11.122	6.433	7.6466	8.8405	6.0272	8.5673	7.5443	7.6945	9.5367	10.5497	2.6628	12.2532	7.8485	7.6584	7.0918	10.901	9.839	9.738	8.7436	4.8306
TCGA-86-A4JF-01	737	1	10.561	7.9309	6.612	8.9522	5.9364	9.1417	6.7809	8.641	9.1429	11.2213	0	2.8482	0	8.6632	9.1327	10.1922	9.8456	10.6334	8.9222	4.3568
TCGA-86-A4P7-01	415	0	10.037	7.6286	7.486	9.038	6.6044	9.7162	6.6663	7.6145	9.2039	10.9266	0	2.2414	1.3159	7.9606	7.2238	8.92	9.5408	9.6916	8.9271	6.8074
TCGA-86-A4P8-01	805	0	10.02	8.112	6.5074	10.8587	5.7392	10.0545	7.622	8.0981	9.8098	8.9382	2.2524	1.218	2.5695	7.2893	5.6239	8.6514	9.1578	9.3281	9.4544	2.8838
TCGA-91-6828-01	323	0	9.8365	6.312	7.7763	8.2239	7.0293	10.1059	6.5993	7.8662	9.2968	10.1451	0	4.0869	0	7.5009	8.4032	8.2481	9.0257	8.7604	7.9764	6.5221
TCGA-91-6829-01	1258	1	10.609	8.8197	7.617	6.8913	6.0341	9.982	7.2007	8.2408	8.3198	10.6512	0	4.4078	2.6016	8.0822	5.5687	8.5852	9.4587	9.6451	7.6826	7.1751
TCGA-91-6830-01	60	0	10.939	7.214	7.0316	10.8341	5.4815	9.5589	6.9936	8.0224	8.2058	9.9561	4.8799	11.6029	6.5681	8.3415	8.0266	9.2567	9.7469	8.9204	8.7816	7.8748
TCGA-91-6831-01	310	0	11.258	8.762	7.1908	7.0466	5.0953	8.9997	7.4029	6.1853	8.2383	10.7211	0	0.8252	0	7.9718	6.4879	9.3699	9.5574	9.3027	6.6069	0
TCGA-91-6835-01	79	0	10.368	7.3764	7.074	8.1386	7.9167	9.1715	7.0386	7.5469	8.8698	10.3143	0	5.0581	2.3232	7.7738	7.3089	8.7494	8.8057	8.2982	8.2519	4.5865
TCGA-91-6836-01	417	0	11.349	6.7316	6.1337	7.8284	5.25	9.4443	6.9905	8.8308	8.4009	10.383	2.9491	6.8801	6.6446	7.3121	7.0779	8.8516	8.5288	9.1836	8.1052	3.1603
TCGA-91-6840-01	372	0	11.113	5.6789	7.6009	7.7167	7.3282	8.433	8.0393	7.7271	8.8509	9.3672	2.2108	4.2993	2.9649	8.8979	5.9571	8.9071	9.3838	9.8529	6.6704	5.9571

TCGA-91-6847-01	842	0	11.069	9.1914	7.7371	3.9118	2.9734	8.5556	7.9367	8.2114	9.0852	10.6092	0	4.6452	2.6891	8.4322	9.6904	9.188	9.0352	9.2711	7.3587	1.0201
TCGA-91-6848-01	224	0	10.433	9.1652	7.8847	7.0405	8.4487	9.2895	8.1501	8.4997	8.8241	12.1702	1.833	2.7699	1.809	7.3821	7.6936	9.32	9.1196	8.3033	7.5845	3.0938
TCGA-91-6849-01	35	0	9.8329	6.9275	8.1916	9.4295	3.5997	9.7608	6.5782	6.9597	9.8848	10.6099	2.6232	9.0956	5.0762	8.9862	5.9058	8.2511	10.4732	9.8573	9.3457	6.9597
TCGA-91-7771-01	492	0	10.401	6.0347	6.5493	9.8145	6.9052	8.9769	7.5447	8.2688	9.366	10.7648	2.7848	5.9579	2.5628	8.8397	7.4489	9.2021	9.0595	9.3809	8.6699	7.104
TCGA-91-8496-01	505	0	10.252	6.0421	7.4338	10.2369	4.3477	9.5726	7.4847	8.2284	9.5475	10.2843	0	0	0	8.0031	6.5887	8.6543	9.0496	11.537	9.4645	4.1096
TCGA-91-8497-01	434	1	9.5256	6.1545	7.332	9.7922	4.8808	9.7551	7.2218	7.8289	10.4261	10.019	2.8458	4.2007	3.1196	7.5571	7.5418	8.7634	9.3318	10.3852	8.6586	7.0084
TCGA-91-8499-01	36	0	11.636	7.4194	7.5828	8.3896	6.6043	8.7359	7.6012	9.0888	8.523	10.6631	1.5947	4.2394	0	8.2594	4.1805	9.5577	10.2821	10.0168	7.5746	0.6698
TCGA-91-A4BC-01	44	0	10.24	9.0506	7.1765	8.71	8.4167	9.6867	6.3679	8.6804	9.2542	9.7818	0	0.7248	0	7.6063	2.9122	9.2878	9.1976	9.2511	8.6099	5.7515
TCGA-91-A4BD-01	603	0	10.257	6.4488	8.4438	10.7975	4.6247	10.3543	6.9409	7.2036	9.7587	9.8724	0	2.8156	4.5783	8.8049	9.5073	8.9929	9.9765	10.1766	10.01	5.6716
TCGA-93-7347-01	683	0	9.8305	7.7387	7.6035	9.8902	6.9378	10.5998	6.8038	7.4061	8.648	9.8409	2.0788	8.1435	3.7642	7.9088	8.9953	8.4964	10.0372	9.5597	9.0712	6.2549
TCGA-93-7348-01	531	0	10.316	8.3919	7.5185	10.3444	7.4044	9.5608	6.2659	7.2186	9.6583	11.2449	0	6.7153	2.9256	7.564	11.0178	9.0713	9.491	9.6067	8.058	4.4245
TCGA-93-8067-01	186	0	11.315	7.9869	7.0098	8.8379	5.814	9.3747	6.8011	6.7332	8.8908	10.2689	8.9484	14.6212	9.548	8.9527	5.9598	9.8187	9.1435	9.5461	8.2374	5.7236
TCGA-93-A4JN-01	718	0	10.007	8.5817	7.5619	8.9841	6.6783	10.0878	6.7469	7.7624	9.3337	9.7937	0	5.6331	0	7.9118	7.9487	9.1283	9.0781	9.5948	8.358	4.36
TCGA-93-A4JO-01	33	1	9.8214	8.1578	6.7516	9.1363	7.9685	9.4837	6.1083	7.907	8.8961	10.3958	0	6.5338	0.9376	8.0304	5.884	9.0492	9.9017	9.1941	8.4506	1.6286
TCGA-93-A4JP-01	578	0	10.609	6.7296	7.411	10.4097	3.7781	9.9809	6.6886	7.7364	9.4807	10.2574	0.5471	3.4427	0	7.8747	8.1817	9.6015	9.1959	9.7232	8.8911	4.0093
TCGA-93-A4JQ-01	526	0	9.4606	9.4889	5.9856	8.8054	7.3071	9.1187	6.8372	8.3573	8.7698	10.0136	5.2888	8.3465	9.0183	7.9853	7.8473	9.8731	9.0551	9.573	8.5725	5.3303
TCGA-95-7039-01	1272	0	10.613	8.1372	6.1936	8.1706	4.6189	9.6762	6.5078	8.1454	8.9056	10.0463	0.7776	1.9475	0	8.0935	7.1249	9.0467	10.4683	10.3829	8.5044	6.8401
TCGA-95-7043-01	503	1	11.13	6.4341	8.8475	9.8802	4.5687	8.2384	6.5468	7.0519	8.75	10.9417	4.6659	13.7654	9.7591	8.3043	8.6494	8.6152	10.8349	10.0423	7.2727	5.7836
TCGA-95-7562-01	87	1	10.16	8.0407	8.4005	6.7283	7.2715	8.793	7.3608	7.8865	8.819	11.4521	0	5.9602	0	9.0044	5.1339	10.0471	8.6743	10.4199	8.7635	1.483
TCGA-95-7567-01	568	0	9.9701	8.892	5.949	9.386	5.9154	8.8693	7.0267	7.466	9.5946	10.5218	0	0.6439	0	9.3067	5.479	9.1964	10.4744	9.2473	8.295	4.1826
TCGA-95-7944-01	377	0	10.143	8.3477	6.4234	7.6709	5.9824	9.7372	5.2743	7.1601	9.1376	11.7463	4.8947	5.3019	3.7245	8.605	8.5298	10.0886	9.63	9.5895	8.6438	5.1158
TCGA-95-7947-01	477	0	10.955	6.0705	6.573	8.7728	5.5496	8.9037	6.257	8.4077	8.8645	11.4134	0	2.7669	0	7.5192	8.4103	9.0663	10.3372	10.3017	9.0263	4.34
TCGA-95-7948-01	476	0	11.114	7.5508	8.7549	10.5501	3.6454	10.0051	12.3505	6.981	9.8549	10.0577	2.1282	6.1556	0	9.0125	8.0272	8.7066	10.3174	10.8813	7.6929	2.2074
TCGA-95-8039-01	830	0	10.553	7.649	6.4632	10.4979	7.1344	10.4169	6.4672	7.9984	9.1097	9.981	1.0718	4.8906	1.503	8.2602	7.7806	9.0065	8.8111	9.1601	8.6124	5.9152
TCGA-95-8494-01	84	0	11.644	7.7409	7.4371	10.1458	4.7276	8.3824	6.311	7.6055	9.8315	9.6512	0	4.5728	4.1805	8.1366	8.8351	9.3254	10.2887	10.3701	8.1399	5.0935
TCGA-95-A4VK-01	651	0	9.9889	7.0151	6.6927	8.0553	5.2698	9.6939	5.4577	8.05	9.3987	10.1593	0	0	0	8.6708	9.4215	8.7949	10.4286	10.6507	9.4081	6.7336
TCGA-95-A4VN-01	553	0	11.241	9.3523	6.503	7.8985	7.2078	8.8729	7.1144	7.3538	8.7877	10.7285	1.5675	4.3132	0	7.185	12.0713	9.7989	9.3338	8.8757	9.1936	0
TCGA-95-A4VP-01	605	0	10.034	8.5651	7.0114	11.0062	6.6373	9.2216	6.3802	7.6147	10.0765	10.1152	0.6081	2.0514	0	7.9165	5.4589	8.9322	10.073	10.3968	8.8493	5.1747
TCGA-97-7546-01	1285	0	10.21	7.122	7.527	9.4573	5.3823	9.1638	7.7305	8.3993	8.2316	10.8728	0	0	0	9.1211	7.4322	8.4859	8.5129	9.512	8.5636	4.0122
TCGA-97-7547-01	1965	0	10.552	5.5044	7.3043	10.093	5.6445	10.2186	6.649	6.9022	8.495	11.8596	0	0	0	8.0083	10.7462	8.5227	9.4744	9.7961	8.7406	7.8748
TCGA-97-7552-01	1932	0	10.2	5.7823	7.1998	8.3795	6.0159	10.471	6.9406	8.0582	8.0905	10.6403	8.1113	7.5734	6.527	7.7507	9.8948	9.6446	9.4664	8.8204	8.7889	8.2378
TCGA-97-7553-01	1870	0	10.508	7.1555	7.6272	9.3905	7.4885	9.9289	7.0857	7.3093	9.1177	9.8612	0	6.7515	2.5536	7.9562	4.7006	8.8313	9.4516	8.6856	8.5598	5.3162
TCGA-97-7554-01	775	0	9.9986	7.7625	7.1473	8.1706	7.0063	9.7755	6.4437	7.3539	7.8803	10.7706	0	2.3624	1.2873	7.641	8.2004	9.4217	8.9491	9.3045	8.3583	1.3224
TCGA-97-7937-01	564	0	10.936	7.3512	6.6987	8.384	2.4381	9.7244	7.7437	7.815	8.6738	11.2133	0	1.1926	0	8.4518	3.11	9.6506	9.2623	9.4882	7.8878	1.5892
TCGA-97-7938-01	18	1	9.5868	5.1819	7.16	8.7278	3.7749	9.663	7.2019	7.6511	8.7339	10.6316	0	3.2417	0	8.5345	8.9379	8.4397	8.7401	8.6945	8.6256	7.3955
TCGA-97-7941-01	484	0	10.64	6.2279	6.6419	9.4202	4.3737	9.9162	6.186	7.6757	9.4418	9.9143	3.803	10.427	6.3011	8.2033	5.9797	9.0075	10.3223	9.7581	9.2133	8.7533
TCGA-97-8171-01	568	0	10.132	5.4342	7.0601	10.3188	1.425	8.2253	6.6296	9.4754	11.6738	10.2838	0	5.1522	0	9.0076	7.9103	8.6452	10.3542	10.1839	8.2401	5.0204
TCGA-97-8172-01	545	0	9.7832	6.3691	8.0558	8.5716	5.9694	9.5018	7.1285	7.8922	10.1331	9.9809	0	1.3599	0	8.1275	7.015	8.3867	9.1982	9.2568	8.4003	2.5916
TCGA-97-8174-01	164	1	9.8795	5.3437	7.6755	10.4813	5.9992	9.4197	7.1989	7.4834	9.8296	9.6854	0	1.2442	0	7.5877	2.6326	7.9775	10.628	9.4993	8.8527	4.4284
TCGA-97-8175-01	551	0	11.27	8.2477	7.0612	8.8793	6.4981	10.852	7.1449	8.1128	8.4805	9.9526	0	4.4268	2.3751	8.1749	11.025	9.2797	9.3658	9.0883	7.9998	2.4651
TCGA-97-8176-01	468	1	11.273	7.6697	6.5044	9.9709	7.7366	8.9377	6.2464	7.2082	9.2926	10.3623	2.5951	5.6265	4.0141	9.0342	7.9354	8.8861	10.472	10.4285	9.1137	7.615
TCGA-97-8177-01	499	0	10.801	7.9663	7.2242	9.5723	5.1433	9.685	7.3495	7.5319	9.4494	10.595	0	5.5415	1.4656	8.0182	9.3535	9.3558	9.6735	10.5908	8.822	5.5123
TCGA-97-8179-01	435	0	9.8891	5.539	7.5852	9.0758	6.7614	9.956	8.2665	7.6161	9.9689	10.2511	2.0973	10.3475	4.7375	8.3918	1.7348	8.8936	10.0312	9.2104	8.859	7.0504
TCGA-97-8547-01	657	0	10.428	9.47	7.1208	8.4141	6.6301	11.1298	6.958	7.2102	8.7936	9.399	2.5234	8.163	6.5747	7.5945	10.0305	8.833	10.1809	9.8447	7.7515	7.8313
TCGA-97-8552-01	626	0	10.682	6.2004	7.5348	10.2931	4.3584	10.416	7.25	8.0366	9.8868	9.8812	0	6.8847	4.2759	7.8661	8.6864	8.5801	9.7423	9.9786	8.9004	5.6197
TCGA-97-A4LX-01	614	0	9.9338	8.8178	7.4734	9.7551	8.0591	9.6124	6.8168	7.1066	9.1731	9.8169	0	3.3559	0	6.9304	6.7178	8.9475	9.5645	8.9202	9.0789	5.16
TCGA-97-A4M0-01	652	0	9.956	6.4837	6.0677	11.4166	4.8264	9.5967	7.4154	7.5689	8.882	9.8141	0	1.4981	0	8.5994	8.0432	9.8199	9.1088	10.0864	9.2	5.4405
TCGA-97-A4M1-01	601	0	10.019	6.5878	6.6347	10.2119	3.8294	9.7259	6.8338	8.0111	10.2697	9.5841	0	2.7041	2.3784	8.8351	8.5506	8.5987	9.7167	10.0967	8.0436	6.1093
TCGA-97-A4M2-01	624	0	9.9482	7.1256	6.9663	9.8492	6.0343	9.8701	7.4058	7.664	10.4593	9.5534	2.8244	5.1054	0	7.4017	7.1895	8.5027	10.1488	9.5763	9.0149	5.517
TCGA-97-A4M3-01	540	0	9.94	6.3041	6.2011	9.9168	4.9469	9.1199	6.4343	7.6239	9.5941	10.0501	0	5.492	0	8.9039	4.7165	7.9205	9.965	10.2182	8.6766	3.6592
TCGA-97-A4M5-01	634	0	10.505	7.1598	7.5183	9.3629	6.3094	9.8226	6.7798	7.6158	10.0579	10.2402	3.0653	6.4743	1.4749	7.9424	5.7145	9.6071	10.1313	9.8916	9.0967	5.0731
TCGA-97-A4M6-01	568	0	10.288	7.7853	7.1346	10.9577	5.9487	9.9239	6.8135	8.0798	9.8264	10.8601	1.9673	9.8586	5.8578	7.3095	7.9419	9.4541	9.687	9.3779	8.7589	7.0996
TCGA-97-A4M7-01	629	0	9.5174	7.6282	7.8516	9.6781	7.9991	10.7981	6.9779	7.7655	9.3993	9.9654	2.3443	6.3095	6.0696	8.4598	9.0405	9.2727	10.0467	9.3422	8.27	6.8494
TCGA-99-7458-01	747	0	9.5906	6.9987	7.668	8.3895	6.2126	10.589	7.6554	7.2693	7.9807	10.512	3.7637	8.6177	7.2572	9.0899	8.5612	9.4899	9.8214	9.2028	9.3395	5.2127
TCGA-99-8025-01	1060	0	10.815	8.2017	7.2002	8.9663	6.5845	9.6796	6.7046	7.5994	8.741	10.6822	0	7.3839	4.3601	8.1408	7.4753	9.2235	9.6482	9.9929	9.1382	2.3216
TCGA-99-8028-01	1118	0	10.474	9.1314	7.7321	9.2081	8.9662	10.4009	7.108	8.0404	9.2039	10.2026	0	5.7507	1.8927	8.5249	5.0003	9.1181	9.0842	9.4819	9.0494	2.7222
TCGA-99-8032-01	44	0	10.652	8.5006	7.594	7.3094	5.1142	9.1619	7.5023	7.8369	9.8658	10.2943	0.8494	1.9395	0	8.7911	4.3887	10.5502	9.6629	9.9886	8.5185	2.1764
TCGA-99-8033-01	656	1	10.467	9.805	6.8146	8.5441	6.9683	8.8307	4.8834	8.0331	9.1443	11.8829	6.3589	12.9648	8.5775	8.6636	8.1666	9.9422	10.0767	10.2454	8.5304	4.3038
TCGA-99-AA5R-01	658	0	9.8768	8.5047	7.6478	11.1968	6.7249	10.0285	7.0307	7.8082	9.4882	9.5109	0.6998	0	0.7704	8.2373	4.8694	8.5257	9.4189	9.6224	9.2073	4.322
TCGA-J2-8192-01	739	0	10.589	7.8953	7.8487	9.1843	7.1398	10.6969	6.9293	7.9389	8.8388	10.0707	1.2382	5.8608	0	7.8525	10.2167	9.6797	9.6764	8.9774	8.6209	6.3591
TCGA-J2-8194-01	724	0	10.166	6.9652	7.2008	9.0522	8.0007	9.7069	7.4938	7.5418	9.4287	10.4304	5.6116	12.5567	7.4352	9.057	7.4295	9.0223	9.5198	9.0711	8.5731	2.4225
TCGA-J2-A4AD-01	550	1	11.027	8.1615	7.7573	9.5902	5.5756	10.2969	6.8415	8.6266	8.9622	10.5003	5.2584	11.9503	8.7238	8.8575	6.7842	8.7568	10.0991	9.6707	8.1078	4.1741
TCGA-J2-A4AE-01	1079	0	10.149	7.0337	7.3061	10.2509	4.2721	9.9371	7.5797	8.9772	9.4637	9.7879	0	3.8672	0	9.0123	9.0673	9.2948	9.5447	9.3753	9.365	7.0659
TCGA-J2-A4AG-01	988	0	10.508	7.3444	7.4453	9.008	7.1178	9.5237	6.2769	8.4276	8.4695	11.2134	0	1.4268	0	9.05	7.5052	9.1144	8.5388	9.8054	8.1126	2.9549
TCGA-L4-A4E5-01	578	0	9.9431	8.9834	7.0607	8.1523	4.7995	9.416	7.3885	7.7716	8.9651	9.8401	0	2.7899	0	8.6293	7.6913	9.381	9.927	9.7884	9.4342	4.4248
TCGA-L4-A4E6-01	435	0	10.759	8.0749	7.4974	9.9324	6.2911	10.0649	7.1804	7.5791	9.6662	9.2619	1.7285	0	0	8.1217	6.3611	8.2617	9.0602	9.1249	9.3531	3.3862
TCGA-L9-A443-01	193	1	9.9289	7.9657	7.1233	9.5303	5.2714	9.2784	7.5285	7.7924	8.4844	10.0136	0	4.7651	2.3972	8.5933	6.0103	8.9485	9.17	10.0553	8.9761	6.705
TCGA-L9-A444-01	307	0	9.4042	8.9422	7.9948	8.7173	6.2367	9.8943	7.7941	7.7388	9.1032	9.302	0	0	0	7.5588	4.7853	8.8251	10.2612	9.653	8.8958	6.1622
TCGA-L9-A50W-01	442	1	10.119	6.2695	7.8507	9.3443	6.5609	10.1547	6.6923	6.7588	10.0931	10.1303	0	5.3303	1.4387	7.2603	8.639	9.22	10.5537	10.1792	9.3788	7.3259
TCGA-L9-A5IP-01	58	1	10.804	8.7175	7.4006	9.8043	7.4618	9.0556	4.6497	8.1435	8.8016	10.7985	3.1346	10.54	5.537	7.2906	9.5673	9.8071	10.2251	10.6932	9.6702	5.8016
TCGA-L9-A743-01	664	0	10.423	9.0777	7.5309	9.0519	8.3605	9.6563	7.2443	8.377	8.7692	10.3262	0	5.4765	2.9995	8.4014	8.9287	8.9782	9.2098	9.039	8.3056	6.1663
TCGA-L9-A7SV-01	565	0	9.5452	5.5957	7.0446	9.4324	2.4664	8.4509	7.7089	7.7321	9.5532	11.3902	0	0.4617	0	8.429	6.5802	9.0699	10.157	9.9067	9.1762	4.9449
TCGA-L9-A8F4-01	476	0	10.583	8.9725	6.6906	8.0743	5.0893	10.0925	6.9684	7.852	10.9708	9.5677	0	1.4911	1.506	8.3499	7.7852	8.5146	9.2044	9.3297	8.5776	3.5181
TCGA-MN-A4N1-01	827	0	10.153	9.6894	7.8987	7.6107	4.601	10.0266	7.0657	7.144	9.3462	10.7585	0	3.2818	0	8.0692	5.5959	10.5723	10.2109	11.4692	8.4025	4.601
TCGA-MN-A4N4-01	1175	0	10.461	9.4972	6.5899	9.1188	7.3004	10.014	6.4588	7.4347	8.8161	11.3503	0.9394	0	2.2457	7.5012	8.4465	9.1905	9.5783	9.9608	8.7771	4.2674
TCGA-MN-A4N5-01	84	0	9.1919	9.4195	7.5515	9.5081	6.183	9.5995	6.2885	7.5697	8.9447	10.5052	1.1686	1.0801	0	8.1601	9.3421	8.8555	9.5058	9.7417	9.4573	4.9133
TCGA-MP-A4SV-01	2620	1	10.788	8.8961	6.85	7.8357	6.5043	9.2673	7.0189	8.5916	8.9457	10.6766	1.2095	4.8611	3.2511	8.4612	7.6639	9.0389	9.5074	9.6316	8.4751	4.2768
TCGA-MP-A4SW-01	1778	1	10.417	8.4524	7.443	12.655	6.3505	9.4054	6.398	7.7846	9.7789	10.1815	1.7635	3.0387	2.7067	7.7905	9.3711	9.3145	10.3789	10.8525	9.28	7.3737
TCGA-MP-A4SY-01	1501	1	11.162	8.8745	6.3596	8.1504	5.5906	9.8825	6.3795	7.851	8.905	9.8029	2.0431	6.6401	0	8.4276	7.7931	8.7526	9.4282	8.7085	7.5973	4.5553
TCGA-MP-A4T4-01	2617	1	10.77	9.2461	6.8649	7.9681	6.0498	9.7447	7.2733	7.566	9.4776	10.131	0	4.9754	3.2765	7.577	7.9399	8.8107	9.7358	9.2742	8.7809	3.1265

TCGA-MP-A4T6-01	1790	1	10.072	5.9326	7.8931	10.3967	4.9883	9.9874	6.4907	7.5575	9.6763	11.2618	0	1.0765	1.2396	7.3104	8.0689	9.2843	10.5913	10.3261	8.0189	6.0475
TCGA-MP-A4T7-01	167	1	10.083	7.1848	6.5758	9.4091	5.5909	10.134	6.6664	8.0366	10.1042	10.8729	2.3107	2.2056	2.3158	8.5097	8.3354	8.5236	10.4816	10.0856	8.9044	3.5344
TCGA-MP-A4T8-01	161	1	10.545	9.4642	6.7164	8.0206	3.8932	8.6766	7.4978	7.8208	10.4738	10.376	0	1.978	0	9.0038	5.5605	9.3383	9.0307	9.773	8.6542	3.7654
TCGA-MP-A4T9-01	1265	1	10.849	9.6895	8.0875	10.0541	6.8139	10.0563	6.3757	7.4482	9.7968	9.5954	0	1.9213	0	7.9767	10.3279	8.9036	9.5479	9.0614	8.1322	3.4003
TCGA-MP-A4TA-01	950	1	10.914	8.8836	7.1733	8.6252	6.0452	9.6656	6.9613	7.8432	9.4534	10.2116	3.1812	9.5618	5.9908	8.2323	8.6773	10.1601	10.8654	10.0156	8.7603	6.9659
TCGA-MP-A4TC-01	74	1	11.139	10.1775	7.941	7.4153	6.1965	9.4961	6.4503	8.1027	9.2205	10.5491	0	2.0651	0	8.7112	8.2884	9.2986	10.3233	9.2466	8.9998	5.2072
TCGA-MP-A4TD-01	307	1	10.133	8.0419	6.5062	8.0017	6.1792	9.7224	7.0668	7.4551	10.0065	10.2141	2.7926	11.4034	6.7809	8.2249	7.1392	8.7623	9.74	9.7785	9.2141	6.9794
TCGA-MP-A4TE-01	896	1	10.721	6.6416	5.7659	9.0489	4.3802	9.1214	7.0712	7.5152	9.4341	10.5648	5.1591	12.2871	8.2841	8.9649	8.1194	9.4829	9.1861	10.3972	7.8652	5.8646
TCGA-MP-A4TF-01	336	1	10.668	9.9982	7.5952	7.0479	3.894	9.8775	6.5914	8.0063	8.6725	10.9052	0	0.8605	0	7.5716	9.3156	9.0619	10.0158	8.9474	8.2577	3.5365
TCGA-MP-A4TH-01	741	0	9.8929	6.4185	8.3553	9.3906	6.8846	9.4365	7.4196	7.9768	10.1114	10.0297	0	0.943	0	7.0743	9.0957	8.4727	9.191	9.4678	9.2567	2.9853
TCGA-MP-A4TI-01	429	1	10.501	9.8173	7.0917	8.0828	8.1752	9.5306	7.2054	7.4364	9.4097	10.3344	1.018	3.2018	0	7.9335	8.2378	9.4205	9.724	9.1559	9.0977	2.0269
TCGA-MP-A4TJ-01	339	1	9.7072	8.5686	7.2402	8.5799	8.9083	9.4613	7.0057	8.0533	8.7795	10.4028	2.586	6.0602	4.2987	8.5898	8.317	8.7823	9.4415	9.0996	8.5125	5.7388
TCGA-MP-A4TK-01	582	1	10.537	9.2105	7.905	7.4662	6.2249	10.6125	7.2153	7.6869	8.8366	10.0538	0	1.3138	0	8.297	8.8428	9.0498	9.9934	9.4105	8.9274	2.4483
TCGA-MP-A5C7-01	2248	0	9.7586	4.0309	7.1369	9.4729	6.7164	9.1858	6.4753	7.4155	11.3304	10.7192	4.5574	10.9435	6.4304	7.0992	7.1549	8.4305	10.2386	9.6217	9.099	5.6309
TCGA-NJ-A4YF-01	2161	0	10.055	8.8111	6.9139	10.5587	4.8553	10.2056	7.6148	7.318	10.6162	10.7551	0	0	0	8.3676	6.1346	8.584	9.8954	10.0605	7.4442	0.6848
TCGA-NJ-A4YG-01	2261	0	9.9879	7.8327	6.8984	9.8174	5.3105	9.6492	6.8992	7.7457	8.9553	10.3761	3.9567	6.9663	0	8.2012	8.8883	8.7578	9.5866	9.8797	8.5221	4.1492
TCGA-NJ-A4YI-01	4	1	9.3222	6.2303	6.3759	8.679	4.7646	9.0985	6.0761	7.2737	9.1454	10.7586	3.8694	5.3049	2.0689	7.5672	6.4194	8.2309	9.4454	10.6917	8.3981	5.2451
TCGA-NJ-A4YP-01	50	0	10.564	9.1479	7.5715	8.2496	5.5306	8.9738	5.8173	7.6652	9.4563	11.4804	0.9217	0.0957	0	9.1602	8.637	9.3609	9.3316	10.4377	8.2472	6.0618
TCGA-NJ-A4YQ-01	1432	0	10.923	7.5938	6.6374	7.8619	7.6483	9.3978	6.9102	7.8813	8.8412	10.5825	0	0	0	8.0445	9.733	8.6694	10.5658	9.536	9.1269	1.8119
TCGA-NJ-A55A-01	15	0	9.9134	6.8418	7.4656	8.6457	7.0362	9.7056	7.2829	7.8181	8.8316	10.5264	0	7.9774	4.4501	8.0546	7.0553	9.1056	9.895	9.3474	8.8536	4.3386
TCGA-NJ-A55O-01	13	0	11.001	6.9315	7.1273	10.145	4.1635	9.551	6.6549	7.8473	10.214	10.8051	5.5869	2.9779	2.9575	7.9832	3.0002	9.0987	9.4949	10.2111	9.0941	5.1226
TCGA-NJ-A55R-01	603	0	9.5786	7.4154	7.1937	7.8257	7.6778	9.8494	5.9437	7.7046	9.6095	10.6019	4.9895	10.5746	5.9567	8.3148	7.6348	9.786	9.9944	10.2682	9.1172	6.5544
TCGA-NJ-A7XG-01	617	0	9.0408	6.3995	7.228	10.2198	3.8814	11.0067	6.5955	7.642	9.5208	9.7475	0	4.0055	0	8.479	9.2912	9.0962	10.0332	10.1389	8.6466	3.8359
TCGA-O1-A52J-01	1798	1	10.95	6.5423	6.3954	11.3805	5.9819	11.2349	6.3123	8.2058	9.4474	9.4164	0	2.7531	0	7.8194	8.2405	7.728	10.1892	9.7031	7.8822	8.2788
TCGA-S2-AA1A-01	513	0	9.548	7.5594	7.5025	8.6344	6.6986	10.4544	6.671	7.8475	9.1273	10.4884	3.0722	10.3171	5.5136	8.1095	6.8207	8.6697	10.0522	9.3156	9.278	4.6198
