# Supplementary material for: Insight of a Metabolic Prognostic Model to Identify Tumor Environment and Drug Vulnerability for Lung Adenocarcinoma
Source: Front Immunol. 2022 Jun 23;13:872910. doi: 10.3389/fimmu.2022.872910 (PMC9262104; doi:10.3389/fimmu.2022.872910)
Supplement: Supplementary file 7 [file DataSheet_6.pdf]

SupplementaryTable S6: The results 192 common elements with univariate Cox regression model.

id	HR	HR.95L	HR.95H	pvalue
GCDH	0.576560335	0.433332089	0.767129478	0.000157208
HEMK1	0.686332892	0.544175602	0.86562653	0.001480058
GALT	0.687891138	0.520877096	0.908456566	0.008375129
JMJD7-PLA2G4B	0.711034966	0.598681405	0.844473735	0.000101781
TRDMT1	0.714948872	0.588471582	0.868609301	0.000729912
NNT	0.735328164	0.601720205	0.898602879	0.00265613
NEU1	0.740786113	0.596261889	0.920340668	0.006736032
PPOX	0.741254316	0.602393572	0.912124542	0.004668774
BDH2	0.74471462	0.614239235	0.902905308	0.002706347
PLA2G15	0.756181089	0.595523668	0.960179871	0.021823862
GSTM4	0.767941733	0.624091041	0.944949481	0.012594901
ITPKB	0.776918362	0.649127853	0.929866339	0.005904722
TK2	0.789405529	0.641587981	0.971279245	0.025388371
FHIT	0.793597092	0.688575052	0.914637182	0.001413125
PCK2	0.794071849	0.634095322	0.994409008	0.044557393
ALDH2	0.794510383	0.692259376	0.911864497	0.001065683
GLS2	0.79753647	0.727666838	0.874114893	1.32E-06
MBOAT1	0.797597911	0.671616465	0.947210887	0.009929916
HYI	0.811709655	0.676026143	0.974625864	0.025393264
ACSL1	0.8297462	0.699286658	0.984544391	0.032481513
ALDH4A1	0.831723902	0.695303451	0.994910422	0.043822226
ARSA	0.838374923	0.705381765	0.996442702	0.045456935
SULT1A1	0.840786747	0.741816398	0.952961348	0.006647581
PLCB2	0.843175592	0.74831576	0.950060279	0.005090375
GPX3	0.844666174	0.746255497	0.956054527	0.00756203
ACSS3	0.84531276	0.765075312	0.933965129	0.000958172
ARG2	0.846269892	0.71984953	0.994892266	0.043176709
PIK3CG	0.858732087	0.775812301	0.950514443	0.003287046
ACPP	0.860851183	0.753426239	0.98359298	0.027579157
ACER2	0.86985388	0.763377564	0.99118157	0.036356252
LDHD	0.870942478	0.793627979	0.955788884	0.003575951
GGT5	0.87112251	0.762463334	0.995266781	0.042380814
PIPOX	0.89844272	0.81287603	0.993016513	0.035975678
ADH1C	0.951212369	0.904945759	0.999844424	0.0492894
CPS1	1.055373094	1.019979952	1.091994371	0.00195721
ITPKA	1.068198374	1.007824994	1.132188398	0.026246112
AMDHD1	1.099596521	1.004925456	1.203186267	0.038740881
GYS2	1.146876212	1.014267448	1.296822695	0.028820467
HK2	1.172810573	1.036944942	1.326477989	0.011165636
POLE2	1.178894169	1.058525323	1.312950602	0.00274421
SPHK1	1.201947404	1.076608037	1.341878857	0.001061601
PYGL	1.211842119	1.051716457	1.396347193	0.007876398
MIF	1.225814861	1.050643536	1.430192088	0.009656618
NME4	1.241620192	1.030352864	1.496206548	0.022955399
POLR3G	1.279094673	1.113157535	1.469767872	0.000516472
RRM2	1.283039048	1.144218286	1.438702055	1.99E-05
TKFC	1.291618908	1.02067571	1.634485261	0.033144072
PPAT	1.309034402	1.086693694	1.576866669	0.004577714
UAP1	1.323177882	1.031450869	1.697414545	0.027549472
DTYMK	1.359011441	1.130904528	1.633128219	0.001067131
WARS2	1.386390557	1.045021746	1.839271561	0.023493365
NT5C3A	1.41503166	1.137318865	1.760556922	0.001843868
SMS	1.585456479	1.277342002	1.967892893	2.91E-05
