# Supplementary material for: Insight of a Metabolic Prognostic Model to Identify Tumor Environment and Drug Vulnerability for Lung Adenocarcinoma
Source: Front Immunol. 2022 Jun 23;13:872910. doi: 10.3389/fimmu.2022.872910 (PMC9262104; doi:10.3389/fimmu.2022.872910)
Supplement: Supplementary file 8 [file DataSheet_7.pdf]

Supplementary Table S7: The construction of metabolic prognostic model based on lasso regression.

id	futime	fustat	DTYMK	GCDH	HEMK1	JMJD7.PLA2G4B	NEU1	NNT	NT5C3A	POLR3G	PPOX	SMS	TKFC	TRDMT1	UAP1	WARS2	riskScore	risk group
TCGA-NJ-A4YI-01	0.010958904	1	8.8917	8.548	9.0334	9.676	10.2529	9.6995	8.2309	6.408	8.5376	9.3222	9.4454	6.0761	10.7586	7.5672	0.818176612	low
TCGA-55-8506-01	0.030136986	0	9.5143	9.1851	7.7926	8.6567	11.395	11.4234	9.2559	6.4731	7.7889	10.4292	9.3234	5.1435	12.0883	7.7889	1.005730538	low
TCGA-NJ-A55O-01	0.035616438	0	9.144	8.4981	8.9381	9.3137	11.1451	10.8532	9.0987	5.2162	7.8473	11.0014	9.4949	6.6549	10.8051	7.9832	1.039942903	low
TCGA-35-3615-01	0.038356164	0	9.5826	9.166	9.9858	9.0828	11.3167	8.66	7.9781	6.6638	8.9007	10.508	9.9668	7.0472	10.9087	9.5549	1.885552439	high
TCGA-NJ-A55A-01	0.04109589	0	8.4176	8.4576	9.1595	9.484	10.6903	9.9148	9.1056	5.425	9.4805	9.9134	9.895	7.2829	10.5264	8.0546	0.85622436	low
TCGA-97-7938-01	0.049315068	1	7.9301	8.2147	8.5203	8.9236	10.9361	10.5728	8.4397	5.8828	7.698	9.5868	8.7401	7.2019	10.6316	8.5345	0.709478814	low
TCGA-86-8672-01	0.052054795	1	10.1517	8.0349	8.6766	8.6569	11.601	7.416	9.1011	6.2581	8.0873	10.614	9.8594	6.3951	9.9782	8.9697	3.880104086	high
TCGA-50-6673-01	0.060273973	1	7.8758	9.1738	7.8895	7.7309	11.5819	11.0425	9.0677	5.4095	7.7118	9.7985	9.4941	7.6607	9.7037	8.9	0.738678495	low
TCGA-86-8074-01	0.065753425	0	8.9354	8.2432	8.295	7.4575	11.6466	11.6734	9.039	4.7786	8.0602	11.033	8.9645	7.0423	10.6316	9.2678	1.548047628	high
TCGA-55-A493-01	0.076712329	0	10.2824	8.421	8.483	8.7153	10.4916	10.0949	9.932	7.4354	7.1054	10.9649	9.4261	6.3135	10.0181	7.8504	2.263477866	high
TCGA-93-A4JO-01	0.090410959	1	8.9836	8.994	9.4742	9.5557	11.0886	9.9985	9.0492	6.7225	9.0634	9.8214	9.9017	6.1083	10.3958	8.0304	0.886708425	low
TCGA-91-6849-01	0.095890411	0	8.3577	8.6044	9.5564	9.1812	10.8635	8.7307	8.2511	5.674	8.6885	9.8329	10.4732	6.5782	10.6099	8.9862	1.801206678	high
TCGA-91-8499-01	0.098630137	0	10.1888	9.6915	8.7258	8.4371	11.1308	12.2883	9.5577	7.8725	9.1798	11.6364	10.2821	7.6012	10.6631	8.2594	1.275801984	high
TCGA-73-4677-01	0.104109589	1	8.4271	8.3977	8.8737	8.6396	11.61	10.1491	8.2397	5.1221	8.7081	9.9117	10.1572	7.4846	11.2668	8.5344	1.110528414	low
TCGA-91-A4BC-01	0.120547945	0	9.3867	8.8266	9.1896	9.2167	10.8494	10.6657	9.2878	6.8966	8.8695	10.2399	9.1976	6.3679	9.7818	7.6063	0.764627646	low
TCGA-99-8032-01	0.120547945	0	9.1766	8.3847	8.3694	8.6295	11.1549	10.2431	10.5502	6.5973	8.2485	10.6519	9.6629	7.5023	10.2943	8.7911	1.738173043	high
TCGA-55-8616-01	0.131506849	0	9.0133	8.8089	8.1608	9.2949	11.3096	10.9386	9.0694	5.8771	8.9762	10.7026	10.2053	7.2253	10.5871	8.7625	1.197491037	low
TCGA-NJ-A4YP-01	0.136986301	0	9.4688	8.6877	9.11	9.0126	10.7635	9.7923	9.3609	7.396	8.4989	10.5644	9.3316	5.8173	11.4804	9.1602	2.165937979	high
TCGA-L9-A5IP-01	0.15890411	1	9.1443	8.5381	8.4341	8.6191	10.1713	10.4831	9.8071	6.7302	8.6111	10.8042	10.2251	4.6497	10.7985	7.2906	2.292889473	high
TCGA-91-6830-01	0.164383562	0	7.2402	7.6944	8.395	8.0725	10.7056	10.2538	9.2567	5.21	7.5426	10.9386	9.7469	6.9936	9.9561	8.3415	1.912816612	high
TCGA-05-5715-01	0.169863014	0	8.9678	8.7985	7.0205	9.1173	12.6009	10.6923	9.1153	3.8353	7.7478	10.5035	9.6685	5.4576	11.3187	7.4522	0.692051482	low
TCGA-64-5775-01	0.169863014	1	9.6936	8.5002	8.6791	7.1554	10.4019	9.3965	8.3947	8.9548	6.8242	12.8018	8.7951	6.0027	11.422	8.0975	4.982189339	high
TCGA-MP-A4TC-01	0.202739726	1	9.9725	8.3541	8.575	8.5454	10.6761	10.5359	9.2986	6.4503	7.9885	11.1385	10.3233	6.4503	10.5491	8.7112	3.331661962	high
TCGA-91-6835-01	0.216438356	0	8.5656	8.341	8.1863	8.3043	11.4473	10.782	8.7494	5.5282	8.453	10.368	8.8057	7.0386	10.3143	7.7738	0.716522861	low
TCGA-95-8494-01	0.230136986	0	10.145	8.7963	9.0722	9.351	10.3407	10.4564	9.3254	6.6591	8.1529	11.6441	10.2887	6.311	9.6512	8.1366	2.347151007	high
TCGA-MN-A4N5-01	0.230136986	0	9.5436	8.1817	9.6598	9.8625	11.5082	10.6045	8.8555	5.9909	8.2782	9.1919	9.5058	6.2885	10.5052	8.1601	0.792444954	low
TCGA-95-7562-01	0.238356164	1	9.1563	9.1788	7.87	7.6523	11.5925	10.615	10.0471	6.1038	7.2246	10.1595	8.6743	7.3608	11.4521	9.0044	1.01533589	low
TCGA-05-4415-01	0.249315068	1	9.4624	7.5314	8.1137	7.2987	9.919	10.151	9.8588	8.8034	7.3741	10.9687	9.9743	6.2916	11.0098	8.5047	7.315548522	high
TCGA-44-2666-01	0.265753425	1	8.617	8.8382	9.0391	9.511	10.7469	11.0175	8.7571	5.7201	7.9633	10.3027	10.2738	6.8901	10.2552	8.8276	1.171584796	low
TCGA-55-A490-01	0.271232877	1	9.4104	8.051	7.0802	8.4505	11.7696	9.4424	7.9513	4.9548	5.3867	11.4176	9.0945	5.6777	10.2436	7.6946	1.947181505	high
TCGA-86-A4D0-01	0.317808219	1	10.2025	8.8273	8.2739	7.7548	10.5519	11.331	10.901	7.4968	8.3642	11.1219	9.839	7.5443	10.5497	7.6584	1.855622362	high
TCGA-55-6975-01	0.323287671	1	10.2213	7.8339	7.2547	7.1564	10.9958	9.6299	9.2575	6.7018	7.3015	10.8645	9.7129	5.9192	9.9924	7.8757	4.329442771	high
TCGA-50-6591-01	0.326027397	1	9.5692	8.4208	7.2264	7.81	11.1762	11.1958	9.5452	5.7433	7.8442	11.4721	8.0886	7.1312	10.7424	8.1043	1.094873563	low
TCGA-55-7727-01	0.326027397	0	8.0659	7.3682	8.7525	7.4763	11.1189	11.6999	9.3435	6.697	7.1619	11.0925	8.051	7.1984	11.1159	8.9501	1.668172546	high

TCGA-05-4250-01	0.331506849	1	10.1682	8.2451	8.8442	7.8873	11.2124	10.2709	9.2894	7.3294	7.9447	11.7315	9.7004	6.7113	10.1991	8.871	3.822172228	high
TCGA-86-8055-01	0.339726027	1	9.0532	8.1095	8.369	7.0877	10.9286	10.4819	9.5903	6.0663	7.4156	10.1703	9.5212	7.0415	10.0176	8.5614	2.284075637	high
TCGA-69-8255-01	0.353424658	0	10.0631	9.0216	8.8016	9.4089	11.991	10.1579	10.1579	8.176	7.7859	10.9259	10.078	5.4774	10.5693	8.5769	2.011557931	high
TCGA-73-4670-01	0.35890411	0	10.2306	7.942	8.4638	8.7115	11.3202	10.4363	9.5252	6.2818	7.8646	11.4905	10.6919	6.4401	10.5914	8.6092	4.283382523	high
TCGA-38-A44F-01	0.364383562	0	8.623	8.4896	9.2296	9.7338	10.5501	9.6624	9.4807	5.5442	9.0028	10.5032	9.6856	6.7047	10.0181	8.042	1.036541376	low
TCGA-69-7978-01	0.367123288	0	8.8338	8.2397	8.5798	8.0696	11.1833	9.4555	8.5709	7.1857	7.2604	10.5144	8.9134	6.8704	10.3345	8.5025	1.707285593	high
TCGA-62-8394-01	0.380821918	1	9.8481	9.1744	8.1923	8.4125	12.1732	11.4845	9.3702	4.6744	8.1882	11.2145	9.9242	6.3591	10.5493	8.7994	1.30457973	high
TCGA-86-8056-01	0.380821918	0	7.9979	8.1143	9.2593	9.2796	11.9171	12.262	8.5447	4.0522	8.9269	10.0424	8.7914	7.4816	12.1028	8.9544	0.46728579	low
TCGA-67-6216-01	0.38630137	0	8.7144	8.554	9.1133	9.2975	10.5477	10.6821	9.3056	5.0876	7.8979	10.0643	10.1348	6.2673	9.6747	8.1462	1.175297838	low
TCGA-05-5423-01	0.41369863	0	9.5102	8.8373	9.1775	8.3977	12.2174	10.6696	9.0851	6.2975	9.3639	10.8912	9.4615	8.0038	10.4117	8.7169	0.963559479	low
TCGA-55-8092-01	0.421917808	1	9.0713	8.5025	9.8167	8.644	10.7111	10.2573	9.505	5.2785	8.9219	10.5075	9.8943	6.8881	11.0099	7.96	1.329106775	high
TCGA-MP-A4T8-01	0.44109589	1	9.5933	8.4072	8.2843	8.7739	10.9236	9.8247	9.3383	7.4361	8.5005	10.5445	9.0307	7.4978	10.376	9.0038	1.644260098	high
TCGA-97-8174-01	0.449315068	1	7.976	8.523	9.1683	9.3403	10.6623	10.5998	7.9775	4.6333	8.2599	9.8795	10.628	7.1989	9.6854	7.5877	0.794081634	low
TCGA-69-7765-01	0.452054795	0	8.4484	7.782	8.3218	7.2485	11.5979	11.7421	9.2503	4.8312	7.4679	10.0167	9.0077	7.1631	11.3854	8.7051	1.307270535	high
TCGA-MP-A4T7-01	0.457534247	1	9.4579	8.6462	9.3874	9.2108	10.5037	10.152	8.5236	5.3779	8.5961	10.0826	10.4816	6.6664	10.8729	8.5097	1.674103281	high
TCGA-55-6712-01	0.468493151	1	8.776	7.7131	8.6106	7.7436	10.5936	10.3128	9.0172	7.5081	6.8767	12.0738	10.2139	6.7382	9.8811	8.3303	5.102625989	high
TCGA-78-7146-01	0.473972603	1	9.4011	7.5397	8.5608	6.2217	10.8636	9.443	9.1476	6.2967	8.741	10.9423	9.1627	7.2265	12.4235	8.4907	4.354357241	high
TCGA-67-6215-01	0.476712329	0	8.6203	8.8391	8.7938	9.283	11.179	10.7563	8.638	5.6697	7.9964	10.2866	9.6931	6.2238	11.2509	8.3227	0.940980598	low
TCGA-55-6978-01	0.482191781	1	9.2186	8.1284	8.2631	7.579	10.515	10.3112	9.6517	6.5593	7.0923	11.4988	8.5126	7.4893	9.6665	8.0927	1.828827655	high
TCGA-78-7158-01	0.490410959	1	8.765	8.9555	8.9591	8.6294	11.16	11.0804	8.6475	7.188	8.8787	9.9756	10.3965	6.722	10.8219	8.8374	1.394304666	high
TCGA-86-6851-01	0.490410959	0	8.8279	8.5605	8.7268	9.1347	11.4178	11.3118	8.3835	4.8678	8.127	9.3028	9.503	6.958	10.7865	7.2743	0.447972336	low
TCGA-35-4123-01	0.498630137	0	10.2121	8.2013	8.3697	7.9114	11.4159	9.9581	10.19	8.3289	8.1171	10.7132	8.6269	7.5175	11.4054	9.258	2.442337466	high
TCGA-69-7974-01	0.504109589	0	9.0323	8.2757	8.5239	8.2947	10.6108	10.4713	8.6696	6.5436	7.3994	11.7942	9.9997	6.6361	10.6004	7.5128	2.314622346	high
TCGA-69-7761-01	0.509589041	0	8.353	7.5893	8.0075	8.3998	10.512	10.571	10.0252	5.3928	7.3382	10.6351	9.301	6.864	10.3425	7.9923	1.716645479	high
TCGA-93-8067-01	0.509589041	0	10.4259	9.0621	8.3513	8.0625	10.7229	9.6828	9.8187	4.2653	7.6589	11.315	9.1435	6.8011	10.2689	8.9527	2.072454386	high
TCGA-55-7914-01	0.512328767	1	8.7691	8.4505	7.9476	8.8002	11.7967	10.7637	9.1247	7.9908	8.3894	10.8298	10.1792	6.6646	10.6167	9.0586	2.06532757	high
TCGA-50-6595-01	0.517808219	1	9.6924	8.1031	7.8	6.6163	10.904	10.2446	10.8305	7.0811	6.8988	11.4521	9.3397	6.6163	10.2987	7.7535	3.66545671	high
TCGA-L9-A443-01	0.528767123	1	9.0514	7.8178	9.6622	9.1616	10.7363	9.9779	8.9485	5.7033	8.6448	9.9289	9.17	7.5285	10.0136	8.5933	1.171200566	low
TCGA-69-7760-01	0.553424658	0	8.3589	8.2177	9.2243	7.417	11.2165	10.988	9.9735	6.3278	8.2506	11.0017	9.6289	7.5671	9.9196	8.5441	1.775257784	high
TCGA-71-8520-01	0.575342466	1	9.6102	8.4591	8.6626	8.231	11.8006	10.0946	9.7246	4.757	8.3149	9.875	9.9726	6.1383	10.6895	8.3914	1.573496239	high
TCGA-44-7662-01	0.597260274	0	9.5696	8.44	8.6691	8.1404	11.471	10.3372	9.3051	6.0208	8.1836	11.094	9.4556	6.8887	9.7315	8.0833	1.550082368	high
TCGA-91-6848-01	0.61369863	0	9.9168	8.4556	7.6994	7.1208	10.5589	10.2452	9.32	9.2202	7.3894	10.4325	9.1196	8.1501	12.1702	7.3821	1.896982089	high
TCGA-35-4122-01	0.616438356	0	11.0051	8.7042	8.9973	7.2069	12.7325	9.7704	9.7537	8.1468	7.6108	11.1262	8.9005	7.084	10.8872	9.4293	2.873221231	high
TCGA-69-7973-01	0.630136986	0	9.1229	8.1355	8.3863	7.7509	10.6493	11.8689	8.4664	7.7578	8.2645	10.5072	9.5131	6.3803	10.3099	8.1871	1.871404068	high
TCGA-55-6979-01	0.649315068	1	9.3799	8.4793	8.9797	7.902	11.1292	10.5466	9.8553	5.9449	8.0965	10.4291	9.2722	6.3206	10.3818	8.2778	1.558158104	high
TCGA-55-7284-01	0.665753425	1	7.7258	7.7552	8.3324	8.6901	10.8621	10.3724	9.5185	5.1786	7.4556	10.8796	9.3005	6.2735	10.6677	7.9295	1.431804767	high

TCGA-05-4402-01	0.668493151	1	9.4966	8.2629	8.2401	8.3646	11.8916	9.9406	9.4046	5.8138	7.5783	10.1039	10.0547	7.2933	10.3544	9.0336	2.000969417	high
TCGA-78-7536-01	0.668493151	1	9.682	8.5414	7.5452	7.279	10.1075	11.3816	10.0163	6.505	7.8067	11.5362	9.395	7.0778	11.0174	9.3595	3.661062338	high
TCGA-50-5072-01	0.684931507	1	10.1849	8.0076	7.8228	7.8973	10.1786	9.2941	9.8305	6.4868	7.2614	10.858	10.139	6.2	10.2689	8.7792	6.003839502	high
TCGA-71-6725-01	0.701369863	0	8.8764	8.8697	9.0419	9.0444	12.0245	10.2798	9.3765	6.0558	8.2299	10.8487	10.136	6.8942	10.9094	8.3271	1.162627335	low
TCGA-50-5936-01	0.704109589	1	9.1504	7.867	7.9673	7.7197	10.6697	10.3851	9.3184	6.9262	7.244	11.1189	10.1077	7.0104	10.8026	7.6106	2.995759072	high
TCGA-78-7166-01	0.706849315	1	10.1812	7.7924	9.5838	9.5143	10.3563	9.3196	9.3807	6.611	9.2261	10.896	10.318	5.8596	11.2129	8.8021	4.296117034	high
TCGA-44-8120-01	0.712328767	0	9.1162	8.5333	8.7915	8.7479	11.3718	9.086	8.9779	6.1741	8.0396	10.3463	9.8568	7.1725	9.516	9.4242	2.031629321	high
TCGA-49-AAR9-01	0.712328767	1	9.8616	7.3447	7.3848	8.2626	11.9211	10.513	8.6727	5.5083	7.4278	12.2746	9.5266	5.2545	10.0205	8.1668	3.888140294	high
TCGA-35-5375-01	0.723287671	0	11.4375	9.282	7.9311	7.8432	12.3259	10.4846	11.129	7.9827	8.5031	10.9415	7.6888	6.5062	10.5703	8.9607	1.192289977	low
TCGA-49-4507-01	0.734246575	1	11.5303	8.514	8.4064	8.4753	10.5301	9.6803	9.699	5.7435	8.5781	10.1032	10.445	5.4784	11.665	8.3262	3.916110156	high
TCGA-05-4418-01	0.750684932	1	10.1349	8.275	9.2965	8.3563	11.067	9.8192	9.7651	7.2595	7.7395	10.8939	9.8334	6.1044	10.633	8.654	3.383371427	high
TCGA-05-5429-01	0.753424658	1	9.6155	8.444	8.8826	7.916	11.2289	9.6931	9.9277	6.0935	7.8595	10.845	10.2051	6.6549	10.9698	9.6877	4.230303045	high
TCGA-73-4676-01	0.769863014	1	9.6342	8.5411	8.3699	7.2117	10.5438	10.4741	9.1796	6.9475	9.2249	12.0181	10.05	6.4606	11.4468	9.2108	5.285918972	high
TCGA-50-5930-01	0.77260274	1	9.2943	8.0222	8.593	8.3327	11.5589	10.9656	8.6702	5.9251	8.0941	10.3038	8.9971	6.1029	11.3957	8.3327	1.33920875	high
TCGA-44-8119-01	0.780821918	0	8.6277	7.7454	7.8755	8.1605	10.7903	10.4089	9.1155	7.7132	7.1576	11.6652	9.8595	7.3675	10.1063	7.7542	2.681938329	high
TCGA-44-A47B-01	0.78630137	0	8.3922	8.3213	9.0287	8.9757	10.9816	10.3808	7.8075	4.8006	7.6	10.4284	8.6759	6.4574	10.0211	8.8966	0.950282576	low
TCGA-78-7161-01	0.797260274	1	7.9103	8.4224	8.8313	9.5426	11.0595	9.6288	8.9476	4.9082	8.2999	10.0726	9.5461	7.4616	10.5001	8.1134	0.72035694	low
TCGA-4B-A93V-01	0.821917808	1	10.5955	9.5638	9.5304	9.409	10.6985	10.2653	11.445	6.596	8.9459	10.7093	10.4192	7.9017	10.8829	8.9371	1.593937907	high
TCGA-05-4396-01	0.830136986	1	8.3125	7.9329	9.659	9.2214	11.1936	11.5501	8.6321	4.3186	7.4973	10.2338	10.0937	6.57	11.055	10.1054	1.93069884	high
TCGA-L9-A444-01	0.84109589	0	8.2369	8.6616	9.6625	9.5762	12.2125	10.9016	8.8251	5.5172	9.7843	9.4042	10.2612	7.7941	9.302	7.5588	0.374923982	low
TCGA-MP-A4TD-01	0.84109589	1	9.2402	9.0507	9.1716	9.2796	11.4102	10.1734	8.7623	6.6161	8.9821	10.1328	9.74	7.0668	10.2141	8.2249	0.814593314	low
TCGA-50-7109-01	0.843835616	1	9.0416	7.919	8.1	7.9935	11.7587	11.8426	9.7748	6.3603	7.9443	10.6147	9.0349	8.6107	10.7609	8.9574	1.192229565	low
TCGA-91-6831-01	0.849315068	0	9.4357	8.2639	7.9084	7.5168	10.6674	10.9319	9.3699	5.9354	7.5349	11.258	9.5574	7.4029	10.7211	7.9718	2.11150434	high
TCGA-78-7542-01	0.879452055	1	9.858	7.9947	8.1315	8.573	10.5394	10.224	10.1789	8.1401	7.5188	12.2868	10.3701	7.371	10.8757	8.0166	4.608152604	high
TCGA-78-8660-01	0.879452055	1	10.3405	9.2595	8.8836	9.0195	11.8907	10.73	8.9223	8.1102	7.6348	10.0534	10.0566	5.5391	10.3559	8.1072	1.324476888	high
TCGA-91-6828-01	0.884931507	0	8.2797	8.507	8.7771	7.196	11.5828	10.8982	8.2481	5.2809	7.2765	9.8365	9.0257	6.5993	10.1451	7.5009	0.773166421	low
TCGA-50-6593-01	0.920547945	1	9.6976	8.5799	8.2709	8.8281	11.6702	11.4076	9.2656	5.4375	8.0408	10.529	10.327	6.4315	9.8962	8.3239	1.42766694	high
TCGA-MP-A4TF-01	0.920547945	1	9.2043	7.6674	8.0864	9.4496	11.0505	9.4646	9.0619	7.3587	8.5141	10.6677	10.0158	6.5914	10.9052	7.5716	2.005509984	high
TCGA-MP-A4TJ-01	0.928767123	1	8.844	8.3521	8.715	9.2644	10.8327	10.2457	8.7823	6.7371	8.516	9.7072	9.4415	7.0057	10.4028	8.5898	1.0736944	low
TCGA-73-A9RS-01	0.931506849	1	9.4637	8.8553	8.2132	8.375	11.0335	10.2581	9.1827	7.402	8.6076	9.9621	11.0782	4.524	11.5105	8.0443	3.030729317	high
TCGA-55-7907-01	0.939726027	1	9.5357	7.4606	7.6466	8.6653	11.102	10.2179	8.8576	5.9538	7.5715	10.854	9.2857	6.5188	9.9039	7.2909	1.691528421	high
TCGA-44-A47G-01	0.961643836	0	9.1852	8.645	9.1046	9.2162	10.9439	10.4155	9.2737	5.6741	8.1443	10.8789	9.3055	6.8731	10.2434	8.1384	1.025904208	low
TCGA-86-8585-01	0.967123288	0	9.1853	8.5288	8.8823	9.2284	10.9653	10.5768	8.737	6.4433	8.7183	11.0696	10.2417	6.2053	10.0374	7.9924	1.613855348	high
TCGA-38-4631-01	0.969863014	1	10.3423	8.7305	8.1944	7.4594	13.7869	10.1829	10.3449	7.6349	8.5029	11.4501	9.332	6.5485	9.5425	8.171	1.587539724	high
TCGA-49-6761-01	0.969863014	0	9.5586	8.5675	8.4842	6.8595	11.011	9.9246	10.133	7.7733	7.4434	10.6194	9.1641	6.6647	10.689	8.1199	2.47974124	high
TCGA-69-7980-01	0.991780822	0	8.325	8.6657	8.3536	8.0529	10.7176	10.7587	8.7666	4.4477	8.3112	10.5979	9.0418	6.8093	9.9424	8.0244	0.863515134	low

TCGA-05-4422-01	1	0	8.8418	8.8235	10.246	9.349	11.0172	11.2195	8.5347	5.5996	9.14	10.7505	10.0709	7.7685	10.8663	8.7832	0.956147237	low
TCGA-50-6594-01	1.01369863	1	9.2613	7.9996	8.0232	8.267	10.8995	12.0531	8.4023	5.5258	7.1624	12.4814	9.4091	5.9927	9.9824	8.7565	2.80841214	high
TCGA-91-6840-01	1.019178082	0	8.8886	8.371	8.014	8.994	11.9727	10.644	8.9071	4.7537	7.6247	11.1133	9.3838	8.0393	9.3672	8.8979	0.993257935	low
TCGA-55-8620-01	1.02739726	1	10.407	10.4653	8.2815	8.4822	11.1735	11.1581	10.5576	7.4201	8.0931	11.369	9.9221	6.9077	9.1476	7.685	0.887820706	low
TCGA-86-6562-01	1.030136986	1	8.8056	8.3407	7.6772	7.8175	11.0354	11.2028	9.1403	5.0513	8.0097	10.4632	9.897	6.7213	11.2369	9.0009	2.031544227	high
TCGA-95-7944-01	1.032876712	0	8.8024	8.8012	8.7259	8.3489	10.1188	10.0093	10.0886	5.6132	7.8915	10.1433	9.63	5.2743	11.7463	8.605	2.104814471	high
TCGA-44-8117-01	1.054794521	0	8.2599	7.9037	8.4603	8.6518	11.1336	10.8458	8.7548	7.5855	8.4255	9.7055	9.6063	7.4585	10.6158	9.3306	1.570062058	high
TCGA-49-4490-01	1.054794521	1	9.229	8.1577	8.9021	8.3228	11.7774	9.8444	8.8356	5.7644	8.1338	10.2479	10.6886	6.7882	9.5121	8.4824	2.24690131	high
TCGA-67-3774-01	1.054794521	0	8.4474	8.2587	8.9689	9.0424	11.3385	10.0636	7.9099	5.4735	8.2466	10.1593	9.535	7.2931	10.7922	8.3854	0.967001427	low
TCGA-69-7979-01	1.117808219	0	9.7366	9.172	8.2088	8.6645	12.0903	8.7832	8.3398	5.1732	8.8608	9.6528	9.4744	6.1879	10.9433	8.0537	0.865011696	low
TCGA-44-A4SU-01	1.120547945	1	9.1926	9.2404	10.1346	9.881	11.6356	10.6895	9.0401	5.8766	8.9863	11.3388	10.0521	7.3319	10.1029	8.367	0.796621377	low
TCGA-69-8254-01	1.120547945	0	8.2947	8.5892	8.5404	9.212	11.2584	11.1761	9.1371	3.6514	8.1053	10.1821	10.1933	6.2111	11.3303	8.001	0.8712116	low
TCGA-62-A46Y-01	1.134246575	1	8.9225	9.1251	9.1082	9.3568	11.1941	10.8931	9.5716	6.1835	8.5389	10.5678	10.101	7.048	10.3462	8.6796	1.027603342	low
TCGA-69-7764-01	1.134246575	0	8.6939	8.2292	9.1879	9.0965	11.686	11.1604	7.8441	4.4738	8.3514	9.6852	8.7726	7.8399	10.156	8.3514	0.450580046	low
TCGA-44-A4SS-01	1.136986301	0	9.0631	8.2371	8.9154	8.107	11.0999	10.372	9.347	4.6962	7.9861	11.0547	9.2174	6.4857	10.3618	8.1887	1.547369015	high
TCGA-86-A4P7-01	1.136986301	0	8.6073	8.7885	8.7977	9.9672	11.568	9.7316	8.92	5.6261	8.442	10.0366	9.5408	6.6663	10.9266	7.9606	0.639722261	low
TCGA-55-8619-01	1.139726027	0	8.3227	8.4068	8.6416	9.5017	10.8208	10.0864	8.7607	4.9403	8.3148	10.3027	9.5806	6.5029	10.3153	7.9161	0.879288706	low
TCGA-91-6836-01	1.142465753	0	9.7044	8.246	7.9388	7.6306	10.3067	10.4469	8.8516	5.5095	8.4159	11.3485	8.5288	6.9905	10.383	7.3121	1.354348718	high
TCGA-55-8507-01	1.145205479	0	9.5876	8.9671	8.8194	8.9356	11.742	10.0826	8.2037	5.0878	7.8516	10.2788	9.4358	7.4532	9.5169	7.3469	0.573374342	low
TCGA-67-6217-01	1.156164384	0	8.2749	8.7222	8.8854	9.7579	11.8049	11.254	9.482	5.91	8.9811	10.3074	9.7234	7.2285	10.5061	8.8324	0.675600565	low
TCGA-86-8668-01	1.15890411	0	8.3527	8.7113	10.0209	8.8079	11.3297	10.8031	9.3926	6.118	8.0302	9.3138	9.8242	6.7468	9.3814	8.4011	0.791645396	low
TCGA-53-7813-01	1.161643836	0	7.5231	7.654	8.4488	8.0783	11.4221	10.4699	9.2464	7.0504	7.8848	10.7546	8.4614	7.6015	10.746	8.0393	1.005482687	low
TCGA-05-4384-01	1.167123288	0	8.3158	8.3487	9.3545	9.315	10.4884	10.1215	8.0919	5.3899	8.6912	9.3572	10.2157	7.477	10.0003	8.6719	1.052003682	low
TCGA-69-8253-01	1.167123288	0	8.7245	9.2738	8.6729	9.081	10.9878	10.0403	8.2913	6.0413	8.8115	10.2127	9.9519	6.5347	10.9455	9.2936	1.27575063	high
TCGA-67-3773-01	1.169863014	0	9.2537	8.3015	9.2218	9.1427	12.6874	11.4673	8.6016	5.2864	7.3339	9.6375	9.8224	7.5471	9.3086	9.1738	0.81775047	low
TCGA-49-4505-01	1.17260274	1	9.7068	8.4996	8.3988	8.5174	11.7252	10.3285	9.3322	5.5029	8.1873	10.609	9.946	7.5235	10.3329	8.3174	1.398329536	high
TCGA-MP-A4TI-01	1.175342466	1	9.7789	8.5864	8.9411	9.1112	10.2573	9.5788	9.4205	7.5113	7.8905	10.5013	9.724	7.2054	10.3344	7.9335	1.702143511	high
TCGA-50-5931-01	1.189041096	1	9.3302	8.4698	8.4621	8.2923	10.1394	11.0781	8.0832	6.1513	9.2502	11.3766	10.423	6.0171	9.9535	7.9161	2.44733327	high
TCGA-91-8497-01	1.189041096	1	8.2329	8.9254	9.2526	9.9845	11.643	10.8302	8.7634	5.4162	9.0459	9.5256	9.3318	7.2218	10.019	7.5571	0.293832841	low
TCGA-55-6543-01	1.191780822	0	8.4624	8.7803	9.1107	9.0051	10.9825	11.0516	8.5183	7.0804	7.2851	9.7851	10.2962	7.2432	10.1707	8.4441	1.089932945	low
TCGA-97-8179-01	1.191780822	0	9.5588	9.4235	9.5082	8.9826	11.829	10.0935	8.8936	5.7384	9.4716	9.8891	10.0312	8.2665	10.2511	8.3918	0.626753761	low
TCGA-L4-A4E6-01	1.191780822	0	8.2538	8.4661	9.1032	9.9479	11.1049	10.2006	8.2617	6.082	8.1304	10.759	9.0602	7.1804	9.2619	8.1217	0.649636996	low
TCGA-55-8505-01	1.205479452	0	9.3672	8.1669	8.7639	8.8821	11.0722	10.4457	9.945	6.7021	7.4882	10.8354	9.8815	6.2998	10.0401	8.4261	2.228057878	high
TCGA-55-7725-01	1.210958904	0	7.0246	7.0817	8.9194	7.9224	10.5395	11.4323	9.5627	6.0923	8.0537	10.4961	8.1845	7.6556	10.7635	9.0481	1.300636451	high
TCGA-L9-A50W-01	1.210958904	1	8.8992	8.3956	8.5766	10.3348	11.4048	10.441	9.22	4.846	8.1699	10.1187	10.5537	6.6923	10.1303	7.2603	0.751676264	low
TCGA-62-8398-01	1.216438356	1	9.527	8.3237	8.6995	8.5801	10.5297	10.233	8.9963	5.831	7.8118	11.3346	10.4114	5.8499	10.2982	8.4576	3.450293519	high

TCGA-86-8359-01	1.216438356	1	8.8766	8.7722	8.0078	7.739	10.8083	10.3821	9.2721	4.8247	7.5602	9.525	8.7003	5.5966	11.0826	8.4896	1.108109713	low
TCGA-55-8615-01	1.221917808	0	9.3822	9.1763	8.7041	9.5252	11.5925	11.1502	9.0481	5.9155	8.5643	10.3293	9.8273	7.4273	10.8261	8.7406	0.761054691	low
TCGA-38-6178-01	1.22739726	0	8.3044	8.4848	7.4248	7.9738	11.5024	10.0776	10.0755	5.6023	7.8427	10.0859	9.0622	6.1057	10.0993	8.4107	1.206931688	low
TCGA-67-4679-01	1.22739726	0	8.3169	9.1096	9.5799	9.9023	11.3045	11.1167	7.956	5.0502	8.3273	9.7597	9.6434	7.1513	10.7975	7.9392	0.388755108	low
TCGA-05-4417-01	1.246575342	0	8.7981	8.2301	7.9844	7.9662	11.5855	11.1637	8.6115	4.8871	7.2379	10.3148	9.3647	6.8815	10.4441	7.5579	0.931738402	low
TCGA-05-4434-01	1.252054795	1	9.3846	8.2963	8.8211	8.7719	10.7231	9.7065	9.1641	6.5911	8.3552	11.4555	9.7759	6.7174	10.3925	7.7413	2.03176943	high
TCGA-05-5420-01	1.252054795	0	10.2913	9.0375	9.1531	7.9174	12.3488	9.7679	9.9617	7.7832	8.6315	10.386	9.972	7.5273	11.7796	8.3443	1.640301564	high
TCGA-50-5939-01	1.260273973	1	9.1131	7.7444	7.6044	7.958	10.7414	9.1131	8.4423	7.9855	7.3193	11.1599	9.7463	6.5017	11.0819	8.3364	4.385692999	high
TCGA-55-8087-01	1.265753425	0	8.2577	9.1947	9.4047	10.2488	11.7219	10.51	7.9569	5.3378	8.4742	10.3119	9.626	7.0394	11.1611	8.0087	0.416361053	low
TCGA-55-6970-01	1.271232877	1	9.354	7.8677	8.2011	7.4809	10.8098	12.0008	8.6758	6.5862	8.2926	10.1078	9.2398	7.1369	10.904	8.2582	1.568767094	high
TCGA-44-A47A-01	1.276712329	0	9.1196	9.117	9.0969	9.5698	11.1823	10.4614	9.2009	7.3643	8.4128	10.1641	10.5497	6.6257	10.8206	8.1156	1.152837062	low
TCGA-73-4668-01	1.279452055	0	9.2908	8.0212	7.8518	7.8192	12.2568	10.6063	8.9859	6.2391	7.8822	11.0041	9.2766	6.8162	11.3145	8.4536	1.797648307	high
TCGA-55-7816-01	1.282191781	1	8.0051	8.0315	8.5745	8.6288	10.3763	10.9978	9.1776	5.6323	8.0409	9.8631	9.0558	7.1636	10.4155	8.613	1.03459385	low
TCGA-97-8176-01	1.282191781	1	9.9089	8.6266	9.4988	8.3446	10.5991	9.642	8.8861	5.0492	8.1916	11.273	10.472	6.2464	10.3623	9.0342	3.777082193	high
TCGA-55-8299-01	1.284931507	1	9.0957	8.4332	8.5948	8.8747	10.7236	10.0177	9.1493	8.6089	7.7992	10.0112	10.0306	6.3592	10.5232	8.5789	2.224878194	high
TCGA-55-8097-01	1.304109589	0	8.4232	8.6629	9.6497	10.5167	10.9889	9.9586	8.3508	5.3727	8.0683	10.4518	9.6411	7.2661	9.5353	8.1499	0.623909754	low
TCGA-95-7948-01	1.304109589	0	8.7134	9.1136	8.9332	10.0542	12.0867	10.6459	8.7066	5.8392	8.6917	11.1136	10.3174	12.3505	10.0577	9.0125	0.427290035	low
TCGA-L9-A8F4-01	1.304109589	0	8.8914	8.7331	9.3492	9.6383	11.0752	9.5247	8.5146	6.8478	8.3131	10.583	9.2044	6.9684	9.5677	8.3499	0.916114502	low
TCGA-95-7947-01	1.306849315	0	9.1618	8.5128	8.2602	8.7068	10.8258	11.7705	9.0663	4.179	8.2487	10.9549	10.3372	6.257	11.4134	7.5192	1.294331385	high
TCGA-50-5051-01	1.309589041	1	9.493	8.2656	9.4345	8.9451	10.7415	10.3799	8.6822	6.5538	8.4334	10.4383	10.0177	7.4016	10.0664	8.6068	1.755346569	high
TCGA-55-8302-01	1.309589041	0	9.7218	8.3515	9.2635	9.2726	10.7332	10.0758	9.1778	4.696	8.0048	11.9785	10.0322	6.1565	9.1625	8.1327	2.232892133	high
TCGA-55-A494-01	1.317808219	0	9.4121	8.0683	9.2544	9.5961	12.2208	10.7728	9.2481	4.1934	8.1082	10.384	10.8947	6.4137	10.0175	9.2397	1.918814013	high
TCGA-97-7941-01	1.326027397	0	8.2681	8.492	9.3128	9.5615	10.8053	10.4082	9.0075	3.9291	8.2772	10.6399	10.3223	6.186	9.9143	8.2033	1.167957614	low
TCGA-44-A479-01	1.331506849	0	8.7692	8.2854	8.1933	8.4396	11.2261	10.4121	9.4364	7.0014	7.6975	10.2393	9.4771	7.2637	10.063	8.3695	1.359197645	high
TCGA-55-7573-01	1.334246575	0	8.5066	8.6911	8.8143	10.0621	11.1777	10.7455	8.2838	5.2414	8.5473	9.4379	9.3679	7.281	10.7032	8.8893	0.548709021	low
TCGA-49-6742-01	1.336986301	1	9.8945	7.979	7.979	8.4995	11.2629	9.0224	10.4936	5.8684	7.666	11.098	11.0368	5.713	10.5542	9.1087	7.119846837	high
TCGA-91-7771-01	1.347945205	0	7.7953	8.2688	9.2435	7.9666	10.8123	10.5929	9.2021	5.4166	7.7804	10.4013	9.0595	7.5447	10.7648	8.8397	1.193445265	low
TCGA-97-8177-01	1.367123288	0	9.1884	9.4063	9.1152	9.1687	12.2951	9.9133	9.3558	5.5864	8.4437	10.8006	9.6735	7.3495	10.595	8.0182	0.647637784	low
TCGA-44-6779-01	1.369863014	1	9.3769	7.1824	7.2744	7.1341	10.6087	8.2697	10.8706	4.684	7.3748	10.1895	10.2709	5.0391	11.1636	8.1774	7.914491481	high
TCGA-95-7043-01	1.378082192	1	10.1949	9.2038	7.9167	8.8117	12.262	7.7	8.6152	7.3088	8.5291	11.1301	10.8349	6.5468	10.9417	8.3043	2.831648188	high
TCGA-91-8496-01	1.383561644	0	9.2016	8.6626	9.5609	9.633	11.5963	9.9204	8.6543	5.0672	8.756	10.2517	9.0496	7.4847	10.2843	8.0031	0.548537556	low
TCGA-S2-AA1A-01	1.405479452	0	8.3134	8.777	9.6254	9.8602	11.9034	9.9092	8.6697	4.4135	8.6995	9.548	10.0522	6.671	10.4884	8.1095	0.561354988	low
TCGA-55-8204-01	1.410958904	0	9.5235	8.4344	7.1883	8.4654	10.4285	11.2867	9.566	5.9579	7.9021	10.6561	9.4301	7.055	10.8192	8.3867	1.579315074	high
TCGA-55-8621-01	1.410958904	0	8.587	8.5128	8.7681	9.4927	10.778	10.0774	8.7752	6.258	8.579	10.1417	9.7522	6.7424	9.6937	7.9414	0.939745314	low
TCGA-55-8514-01	1.424657534	0	9.0898	8.8746	9.2094	10.4295	12.1539	9.3386	9.184	6.7389	8.9871	12.1192	9.8205	5.8032	11.5506	8.0975	1.276774419	high
TCGA-49-6745-01	1.430136986	0	8.7936	7.8759	7.9029	7.0993	9.9072	10.4096	9.8469	7.5065	6.5144	11.4342	9.4436	7.1249	9.5083	8.3544	4.104876467	high

TCGA-69-A59K-01	1.430136986	0	9.2095	8.143	9.2868	8.5145	10.0195	10.1772	7.4617	5.5008	7.5353	10.0373	9.8701	6.6529	10.0246	7.952	1.713699623	high
TCGA-93-A4JQ-01	1.44109589	0	8.7755	8.4944	9.4854	9.5564	10.279	10.6296	9.8731	5.3612	8.1972	9.4606	9.0551	6.8372	10.0136	7.9853	0.642881522	low
TCGA-93-7348-01	1.454794521	0	8.5777	8.7246	8.2796	8.8056	12.0097	11.8738	9.0713	4.4682	8.3279	10.3163	9.491	6.2659	11.2449	7.564	0.549892172	low
TCGA-55-8301-01	1.463013699	0	9.3367	9.3577	9.0994	8.6598	10.5995	10.1377	9.927	6.7154	8.2531	10.7508	9.7538	6.9938	10.4416	7.9759	1.188273512	low
TCGA-55-8614-01	1.468493151	0	9.4487	8.7889	8.4739	9.8257	10.773	10.7116	7.9324	5.7497	8.5605	9.9629	9.896	6.9346	9.6641	8.3263	0.846984773	low
TCGA-55-7911-01	1.471232877	0	9.7815	8.9365	9.6145	9.235	11.1068	10.2146	10.0146	5.5543	8.1353	10.2056	10.2451	6.7377	10.3868	8.0048	1.200239187	low
TCGA-55-8510-01	1.476712329	0	9.264	8.8339	8.8608	9.3314	11.4288	10.9771	9.1537	5.4133	8.7887	11.3009	9.6717	6.1574	9.5661	8.0352	0.98862852	low
TCGA-97-A4M3-01	1.479452055	0	9.3182	8.5085	9.4781	8.8336	10.1715	9.784	7.9205	5.3561	8.5304	9.94	9.965	6.4343	10.0501	8.9039	1.879483274	high
TCGA-55-8094-01	1.482191781	0	10.129	7.5512	8.63	7.9939	13.5383	10.869	10.1699	6.0437	8.8768	9.8816	10.659	5.4953	11.5871	8.7265	2.936423211	high
TCGA-97-8172-01	1.493150685	0	8.1096	8.663	9.3268	10.0667	10.9427	10.8818	8.3867	4.5863	8.2566	9.7832	9.1982	7.1285	9.9809	8.1275	0.415004868	low
TCGA-55-A57B-01	1.495890411	0	8.5498	8.6696	9.6945	9.9591	10.7567	10.0725	8.8054	5.2081	7.9512	9.347	10.793	6.8835	9.2442	8.4429	1.015570091	low
TCGA-55-8203-01	1.498630137	0	8.5464	8.4562	8.8238	8.5291	10.9725	10.4156	9.267	6.561	8.656	10.087	9.697	7.0403	11.0137	8.1907	1.196137401	low
TCGA-J2-A4AD-01	1.506849315	1	9.4789	7.9907	9.627	9.1523	11.7461	10.2442	8.7568	7.29	8.7267	11.0267	10.0991	6.8415	10.5003	8.8575	2.282424225	high
TCGA-97-8175-01	1.509589041	0	9.4932	9.102	9.0357	8.8557	12.8121	10.6306	9.2797	6.7705	7.6586	11.2698	9.3658	7.1449	9.9526	8.1749	0.814089459	low
TCGA-55-8511-01	1.512328767	0	8.8902	8.4292	9.1003	9.2659	11.5559	8.0997	8.5364	5.6864	8.2033	11.1457	9.6676	5.2655	11.8091	6.9634	1.463519872	high
TCGA-95-A4VN-01	1.515068493	0	9.4776	8.5921	9.0846	8.5938	10.3321	9.6662	9.7989	7.0351	8.0524	11.2406	9.3338	7.1144	10.7285	7.185	1.457177627	high
TCGA-44-7661-01	1.526027397	1	9.1058	8.4402	8.5845	8.0696	10.2381	10.1603	9.7289	8.0044	7.2134	11.2978	9.4179	6.9871	10.0037	8.0829	2.409083212	high
TCGA-55-7913-01	1.536986301	1	9.7325	7.9909	8.4415	8.6721	11.7111	11.2515	9.6291	4.7912	8.724	10.547	9.6239	6.5542	11.2602	8.3391	1.419496109	high
TCGA-97-7937-01	1.545205479	0	8.9541	8.7089	8.3037	8.9424	10.6828	11.5259	9.6506	6.726	8.0164	10.9355	9.2623	7.7437	11.2133	8.4518	1.029395076	low
TCGA-L9-A7SV-01	1.547945205	0	9.6478	8.0746	9.0236	7.9458	11.7632	12.1898	9.0699	6.9093	9.6438	9.5452	10.157	7.7089	11.3902	8.429	1.2624201	high
TCGA-55-7903-01	1.553424658	0	9.4616	8.8231	9.7161	9.3415	11.13	10.3743	9.1511	6.2334	8.7415	11.8253	9.9205	7.5986	10.6572	8.7975	1.595237721	high
TCGA-95-7567-01	1.556164384	0	9.6196	8.4211	8.3717	7.7277	11.2066	9.5379	9.1964	6.8656	8.7214	9.9701	10.4744	7.0267	10.5218	9.3067	3.42186807	high
TCGA-97-8171-01	1.556164384	0	8.6613	8.7421	9.7889	9.5142	12.4404	10.4086	8.6452	6.5669	9.3256	10.1321	10.3542	6.6296	10.2838	9.0076	1.026684626	low
TCGA-97-A4M6-01	1.556164384	0	8.5195	8.3581	8.6824	9.987	11.6576	9.6645	9.4541	5.2654	9.011	10.2878	9.687	6.8135	10.8601	7.3095	0.637426662	low
TCGA-67-3772-01	1.569863014	0	9.6433	9.1662	8.3709	8.9428	12.3688	9.8121	9.5891	5.1365	8.479	10.6245	9.8173	7.5709	11.1303	8.302	0.867184064	low
TCGA-44-7669-01	1.57260274	1	9.7223	8.3866	8.9326	7.9119	10.5089	11.8988	10.3804	7.4629	8.1687	11.5149	9.7487	6.7471	10.1806	8.8381	2.973263261	high
TCGA-05-4403-01	1.583561644	0	8.8191	8.5811	8.4959	8.9069	11.3182	10.0674	9.6388	4.1888	7.2528	10.6532	9.9708	6.4275	10.7813	8.5843	1.527415506	high
TCGA-93-A4JP-01	1.583561644	0	8.3702	8.8117	8.8494	9.2478	11.7015	10.9685	9.6015	5.1204	8.4163	10.6094	9.1959	6.6886	10.2574	7.8747	0.557512182	low
TCGA-L4-A4E5-01	1.583561644	0	9.1624	7.3885	8.3959	9.3421	11.4371	11.6562	9.381	5.6481	8.1006	9.9431	9.927	7.3885	9.8401	8.6293	1.359093228	high
TCGA-MP-A4TK-01	1.594520548	1	9.576	8.5789	8.9906	9.456	11.1491	9.9008	9.0498	5.116	7.9231	10.5366	9.9934	7.2153	10.0538	8.297	1.24678786	low
TCGA-78-7147-01	1.605479452	1	9.7777	8.4799	8.7184	8.931	11.7915	10.5933	9.241	5.9367	8.8539	10.962	10.4875	6.9706	10.5355	9.0164	2.132832545	high
TCGA-44-7660-01	1.621917808	0	10.3886	8.7268	8.1627	8.9724	10.4874	10.5232	9.2225	6.4184	8.1306	11.4049	10.8805	6.8399	10.6389	8.1606	2.994750843	high
TCGA-78-7154-01	1.624657534	1	10.0597	9.0111	7.6384	7.2723	10.7747	11.0485	10.3436	7.8166	6.9148	10.7059	9.8289	6.5342	10.5036	8.3802	2.736472386	high
TCGA-62-A46P-01	1.62739726	1	9.3272	7.4914	9.3819	9.0363	12.5869	9.9909	9.502	5.7849	8.5114	9.7362	9.726	5.9693	10.9907	8.9708	1.815895652	high
TCGA-44-6145-01	1.630136986	0	8.5012	7.6574	8.1752	7.7619	10.6708	9.1632	8.4422	4.3184	7.6254	10.9697	9.1584	6.2271	11.1661	7.2477	1.885357312	high
TCGA-55-A492-01	1.632876712	0	9.5809	10.0786	10.0599	10.2847	11.2087	9.5534	8.5564	5.7698	9.6139	10.174	10.5673	6.9058	10.2018	8.0058	0.589456572	low

TCGA-55-8090-01	1.638356164	1	8.794	8.6885	8.71	8.7879	10.1786	9.9356	9.1035	4.7222	7.6604	10.174	10.2723	6.7569	9.2741	7.5948	1.258256006	low
TCGA-55-8205-01	1.64109589	0	9.6999	8.3696	8.8852	8.1102	10.5771	10.4943	9.2871	7.5054	7.3629	11.3998	9.833	6.8991	9.3549	8.6012	3.098079255	high
TCGA-55-8091-01	1.643835616	0	8.5836	8.4599	9.0639	8.7933	10.4641	10.2727	9.0353	5.1654	7.772	9.7325	9.2795	6.617	10.2399	7.7293	0.851264267	low
TCGA-97-A4M1-01	1.646575342	0	8.8007	9.442	9.8875	10.4369	11.5143	9.7959	8.5987	5.7381	9.339	10.0192	9.7167	6.8338	9.5841	8.8351	0.538091131	low
TCGA-55-7994-01	1.652054795	0	9.0222	8.1059	8.6651	8.2358	11.2375	8.5308	9.5152	7.9147	7.0828	9.8974	9.7936	6.4131	9.6164	8.2509	2.566286828	high
TCGA-91-A4BD-01	1.652054795	0	9.2283	9.5315	9.6807	10.122	11.2047	10.6046	8.9929	5.6211	8.5821	10.2573	9.9765	6.9409	9.8724	8.8049	0.682473627	low
TCGA-NJ-A55R-01	1.652054795	0	9.0449	8.4976	9.5539	8.9216	11.3752	10.7318	9.786	5.7121	9.0901	9.5786	9.9944	5.9437	10.6019	8.3148	1.129549198	low
TCGA-86-7954-01	1.657534247	0	8.2539	8.5907	8.4084	8.4141	11.6334	10.5872	9.308	5.6584	7.9986	10.4923	9.8408	7.2947	10.8905	7.5943	0.879352339	low
TCGA-95-A4VP-01	1.657534247	0	10.6479	8.7753	9.3407	9.8778	11.1549	9.6217	8.9322	6.2669	8.513	10.0338	10.073	6.3802	10.1152	7.9165	1.245879113	low
TCGA-05-4382-01	1.663013699	0	9.8108	7.7748	8.4859	8.1803	11.9192	10.7621	8.5098	6.304	8.3893	10.6256	9.0331	6.6064	10.0426	8.3132	1.543823647	high
TCGA-55-8512-01	1.663013699	1	7.8473	8.9586	9.7245	10.2286	10.9761	10.2903	8.5757	6.0489	10.015	10.1635	10.1629	7.2351	10.1862	8.1753	0.571996161	low
TCGA-55-A4DG-01	1.665753425	0	8.0626	9.1815	9.7325	10.0158	10.9703	11.926	9.3165	6.2459	9.4649	9.4168	9.3565	6.8178	10.8575	8.7457	0.396874565	low
TCGA-55-7283-01	1.668493151	0	8.5524	8.8174	8.5886	8.0176	11.2351	10.597	9.5495	5.3958	8.4814	10.1108	10.2959	6.463	10.5979	9.2925	1.900483237	high
TCGA-05-4405-01	1.671232877	0	8.6411	8.9091	8.6096	8.0981	11.0869	11.3286	9.4568	5.7374	7.8841	9.9623	8.8647	6.9181	10.7217	7.7573	0.616744398	low
TCGA-67-3770-01	1.671232877	0	10.3029	9.2076	9.6952	9.5599	12.96	10.992	9.0502	8.2639	8.5618	10.1785	10.093	6.7057	9.4556	8.3905	0.817386699	low
TCGA-67-3771-01	1.671232877	0	9.0194	8.6333	8.263	8.3889	11.8452	10.1553	8.563	6.1304	8.1351	9.5979	9.3044	6.7787	10.5639	8.0596	0.832073914	low
TCGA-55-A4DF-01	1.682191781	1	9.7168	7.7883	8.1353	9.5789	11.0852	11.2647	9.9706	9.1795	7.612	12.5072	9.4263	7.5133	10.7973	7.7392	2.151265905	high
TCGA-97-A4LX-01	1.682191781	0	8.8366	8.3452	8.9905	9.3499	11.03	10.545	8.9475	5.9979	8.149	9.9338	9.5645	6.8168	9.8169	6.9304	0.641152754	low
TCGA-55-8508-01	1.690410959	0	9.5039	8.614	8.5677	8.4631	11.0179	10.5125	8.6839	6.5521	9.215	10.6395	9.8615	7.1811	11.0674	7.46	1.177202528	low
TCGA-NJ-A7XG-01	1.690410959	0	9.0001	9.1908	9.2155	9.7046	11.0864	10.3761	9.0962	5.3519	8.8012	9.0408	10.0332	6.5955	9.7475	8.479	0.635502939	low
TCGA-50-5044-01	1.709589041	1	9.6647	8.5804	8.9877	8.0669	10.3038	9.626	9.3622	8.0778	8.1472	12.2954	9.5939	5.3903	11.3021	7.3997	3.578138766	high
TCGA-97-A4M2-01	1.709589041	0	8.438	8.7193	10.0565	10.3443	10.7514	9.985	8.5027	6.2865	8.8545	9.9482	10.1488	7.4058	9.5534	7.4017	0.569009866	low
TCGA-86-7714-01	1.712328767	1	7.7874	8.5832	8.6822	8.7903	10.8144	11.2937	8.7116	4.6688	7.3204	9.7435	9.5087	7.5403	9.6952	9.4956	0.897696605	low
TCGA-55-A491-01	1.715068493	0	9.2597	9.147	8.0069	8.6252	11.9562	10.4316	9.175	4.1651	7.4394	10.7845	9.5386	7.529	9.9997	7.3811	0.594602429	low
TCGA-78-7148-01	1.715068493	1	9.4241	8.2023	7.9151	8.1302	10.5193	10.7966	9.1208	5.9823	7.0307	10.3344	10.0173	6.2475	10.4068	8.4484	2.594473221	high
TCGA-97-8552-01	1.715068493	0	8.587	9.4267	10.2094	10.5452	11.1496	10.3043	8.5801	5.3661	9.4683	10.6816	9.7423	7.25	9.8812	7.8661	0.406448335	low
TCGA-64-1677-01	1.720547945	1	10.1268	9.7258	9.1629	8.3581	12.8193	10.4949	9.4582	7.106	8.2273	10.7708	9.6956	8.3092	10.4334	8.8976	0.857657006	low
TCGA-97-A4M7-01	1.723287671	0	8.4545	9.2113	8.9926	9.3345	11.7745	10.4677	9.2727	6.0607	8.7778	9.5174	10.0467	6.9779	9.9654	8.4598	0.621502186	low
TCGA-55-A48Y-01	1.726027397	0	9.1583	8.5786	8.8652	7.7431	10.7107	10.7835	9.0467	5.2188	8.1096	10.9501	9.7971	6.488	10.9343	8.4501	2.057955627	high
TCGA-97-A4M5-01	1.736986301	0	8.7349	9.045	9.2876	9.6423	10.7812	10.5384	9.6071	5.055	8.8041	10.5047	10.1313	6.7798	10.2402	7.9424	0.818223364	low
TCGA-55-A48Z-01	1.783561644	0	9.5947	8.664	8.9057	9.3722	11.0746	10.4916	9.3168	5.8744	8.2783	10.4658	9.1836	6.9262	9.8452	7.7243	0.773236339	low
TCGA-95-A4VK-01	1.783561644	0	9.3194	7.9736	9.9566	9.6742	10.4769	9.8791	8.7949	4.0964	8.4185	9.9889	10.4286	5.4577	10.1593	8.6708	2.147399998	high
TCGA-55-7726-01	1.78630137	0	9.2686	7.3907	7.9156	7.7048	10.1026	10.4515	9.6701	6.3373	5.6887	10.3551	8.159	6.8623	10.4368	7.3754	1.695455914	high
TCGA-97-A4M0-01	1.78630137	0	8.9947	9.1242	9.0799	9.8909	10.4945	10.3637	9.8199	4.68	8.5598	9.956	9.1088	7.4154	9.8141	8.5994	0.558212908	low
TCGA-50-5935-01	1.789041096	1	8.1481	8.4428	8.2661	8.1801	11.5311	10.8527	8.7927	5.6627	8.8455	10.2951	9.5154	7.185	10.9483	7.9617	0.858492179	low
TCGA-86-8358-01	1.789041096	0	10.0262	9.209	7.4325	8.3336	11.6495	11.4556	10.6867	8.8678	8.0566	10.1907	8.6253	7.3113	10.421	7.8273	0.702384826	low

TCGA-99-8033-01	1.797260274	1	9.4273	8.1207	8.5058	8.7892	10.7315	8.3261	9.9422	6.6357	7.6055	10.4674	10.0767	4.8834	11.8829	8.6636	4.668588695	high
TCGA-97-8547-01	1.8	0	8.677	9.1179	9.586	9.3268	10.8553	10.8558	8.833	6.7581	7.9611	10.4279	10.1809	6.958	9.399	7.5945	0.790287394	low
TCGA-44-6774-01	1.802739726	0	8.7401	7.8663	8.0993	7.734	11.3997	10.5747	8.6327	6.4501	7.0448	10.0631	8.567	7.0347	10.3487	7.643	1.015854628	low
TCGA-99-AA5R-01	1.802739726	0	8.3898	8.8084	9.3133	10.0516	10.6728	9.9672	8.5257	5.4196	8.5901	9.8768	9.4189	7.0307	9.5109	8.2373	0.5870845	low
TCGA-L9-A743-01	1.819178082	0	9.2739	8.6386	8.6923	9.0659	10.7675	10.7811	8.9782	5.2769	8.3522	10.4227	9.2098	7.2443	10.3262	8.4014	0.907222132	low
TCGA-78-7150-01	1.824657534	1	9.9779	7.6152	7.5592	6.9178	10.0531	9.8303	9.7822	6.7356	7.3004	10.4444	9.1811	6.0532	10.887	8.9462	5.856780989	high
TCGA-05-4425-01	1.832876712	0	9.1236	8.537	8.7128	8.4264	10.8667	10.7436	8.8274	6.7903	7.7429	10.3663	10.3159	5.4439	10.2524	8.3193	2.289693168	high
TCGA-05-5428-01	1.835616438	0	11.7912	8.885	9.6387	8.4647	12.0406	10.4419	9.3224	8.4504	8.7972	10.3447	8.4396	7.9117	11.266	9.1669	1.263676725	high
TCGA-55-7576-01	1.835616438	0	9.7011	8.233	7.6851	8.2836	11.1001	10.0715	9.3083	6.2623	7.4452	10.3896	9.1846	6.6293	11.0705	8.9918	2.208103349	high
TCGA-55-8208-01	1.846575342	0	9.0628	8.3665	8.5779	8.4561	11.6939	10.1141	8.9886	5.6437	8.1917	10.5335	8.6794	6.7992	10.6649	7.5836	0.785449847	low
TCGA-49-6767-01	1.854794521	0	9.41	8.4492	8.1787	7.2966	12.198	10.3565	8.8571	7.0223	6.9688	11.5819	9.2227	6.0846	9.9664	7.4164	1.777206544	high
TCGA-49-AAQV-01	1.854794521	1	9.5625	8.9891	7.9713	9.6906	11.1524	10.1772	9.3933	6.3809	9.2185	10.5138	10.4271	7.1569	10.3753	8.3301	1.208817328	low
TCGA-93-7347-01	1.871232877	0	8.9975	8.8655	9.0343	8.8643	11.1409	10.9648	8.4964	5.5735	7.477	9.8305	10.0372	6.8038	9.8409	7.9088	0.85611221	low
TCGA-55-A48X-01	1.887671233	0	9.0823	8.4869	8.898	9.3966	11.5027	10.1328	8.7584	6.0657	8.8936	9.672	9.7478	6.4989	9.9105	8.0007	0.842449588	low
TCGA-69-7763-01	1.890410959	0	8.5239	7.9164	8.2093	8.4256	10.2719	10.2363	8.2602	4.5154	8.0414	9.9021	8.8012	6.8686	10.0413	9.0628	1.362669152	high
TCGA-44-7659-01	1.893150685	0	8.3916	8.8638	9.5548	9.9769	10.4666	10.9557	8.4075	6.2711	8.6198	9.8483	9.8898	7.1523	10.3583	8.8071	0.784495626	low
TCGA-86-8075-01	1.901369863	1	9.1027	7.7499	8.5439	8.1195	12.2218	10.4172	9.2629	5.8824	7.4297	10.5341	9.0162	6.9423	10.3644	8.1946	1.340187961	high
TCGA-78-7160-01	1.909589041	1	8.9531	7.5623	8.0135	7.4208	10.7141	11.1044	9.3821	5.106	7.9851	10.381	9.5012	6.7156	10.8191	8.7291	2.602101124	high
TCGA-55-1592-01	1.920547945	1	9.2948	8.26	8.7804	9.4084	12.5563	10.4188	8.3668	6.1735	8.0239	9.9249	9.391	7.2091	10.62	8.3903	0.763754712	low
TCGA-86-8280-01	1.920547945	0	8.81	8.5841	9.2411	9.3584	11.9296	10.3176	8.8617	6.2663	8.7431	10.5276	9.4632	7.0026	10.1725	8.4254	0.83734788	low
TCGA-55-8089-01	1.923287671	1	8.768	8.0018	8.409	7.7575	10.529	9.0832	10.558	7.5745	7.4497	11.0939	10.088	6.2608	10.4031	7.6057	3.804920283	high
TCGA-44-6148-01	1.928767123	0	7.9539	8.7121	9.0808	9.4269	10.6288	10.3556	8.6861	5.6366	8.9865	9.7011	10.0594	7.3034	9.5872	8.8083	0.87145809	low
TCGA-55-7728-01	1.928767123	0	8.4677	8.4946	8.9985	10.071	10.7103	9.411	9.0718	4.2594	8.1431	10.4178	9.9098	6.4296	9.8398	7.3801	0.806172878	low
TCGA-44-6775-01	1.931506849	0	8.8608	8.4039	8.6594	8.4039	11.2338	11.768	9.5683	5.8209	7.7141	10.8118	9.3289	6.4482	10.2474	8.4421	1.245512288	low
TCGA-55-7724-01	1.931506849	0	7.2508	6.7577	7.5027	7.8038	10.4155	10.9232	9.2579	6.3427	6.2508	11.0064	8.2697	6.8977	10.2305	8.2074	1.985431157	high
TCGA-73-4659-01	1.947945205	1	9.5796	8.3018	8.7055	8.5444	11.0504	10.8552	8.5123	6.6308	7.7964	10.6745	9.6705	6.6702	10.5716	8.5534	1.869365902	high
TCGA-93-A4JN-01	1.967123288	0	9.6987	8.5621	8.9335	9.5634	11.2326	9.859	9.1283	5.7491	8.7339	10.0068	9.0781	6.7469	9.7937	7.9118	0.747972423	low
TCGA-44-7672-01	1.969863014	0	9.1267	8.7061	8.4771	7.9044	11.7175	10.3379	9.1467	5.0622	8.0833	10.9982	9.9745	7.0033	10.3846	8.3167	1.568253459	high
TCGA-55-8096-01	1.969863014	1	8.7349	8.5164	9.1987	9.0204	11.4772	11.1558	8.9584	4.4525	8.404	10.8907	9.7135	6.8666	10.1175	8.5988	1.012389222	low
TCGA-J2-8194-01	1.983561644	0	8.2359	8.1254	9.2869	8.5895	11.0897	10.4669	9.0223	5.7834	8.3092	10.166	9.5198	7.4938	10.4304	9.057	1.327243488	high
TCGA-44-6146-01	1.994520548	0	7.7566	7.5942	8.5803	9.0197	9.2032	10.5915	9.3788	3.0392	6.7114	10.5272	9.6531	5.4845	11.2984	8.4449	2.303683623	high
TCGA-05-4433-01	2	0	8.6475	7.1614	8.6445	8.4122	10.7074	10.5073	9.563	4.062	7.1105	10.7378	9.6531	6.1879	10.6633	8.4972	2.782442585	high
TCGA-05-4397-01	2.002739726	1	10.2726	9.0701	8.155	7.5296	12.7564	11.9919	8.8516	7.0728	9.1145	12.0734	10.0193	6.8396	10.6848	7.8079	1.463048297	high
TCGA-86-A4JF-01	2.019178082	1	9.831	8.6394	7.8538	8.5086	11.2658	9.5828	10.1922	6.5616	7.2143	10.5608	9.8456	6.7809	11.2213	8.6632	2.377925241	high
TCGA-J2-8192-01	2.024657534	0	7.8502	8.2847	9.1712	8.2189	10.7346	11.1518	9.6797	5.0239	7.8194	10.5892	9.6764	6.9293	10.0707	7.8525	1.083889971	low
TCGA-86-8073-01	2.02739726	0	8.5399	7.5438	8.0975	8.422	11.7025	12.1003	8.7878	6.2334	7.6113	10.6622	9.6459	7.0919	10.5943	7.6562	1.171312422	low

TCGA-MP-A4TH-01	2.030136986	0	8.2891	8.287	9.4911	10.1505	10.5627	10.3483	8.4727	4.6161	8.3165	9.8929	9.191	7.4196	10.0297	7.0743	0.402765429	low
TCGA-99-7458-01	2.046575342	0	7.8039	8.2412	9.5618	8.5813	10.5475	9.9868	9.4899	6.1534	8.0032	9.5906	9.8214	7.6554	10.512	9.0899	1.437241067	high
TCGA-55-6984-01	2.082191781	1	8.6716	8.7318	8.5361	8.6748	9.8027	9.5831	9.7759	6.9375	7.7898	11.5197	9.8464	6.4417	11.3563	7.9636	2.458420221	high
TCGA-05-4430-01	2.084931507	0	9.5439	8.4418	8.0487	7.8133	11.3781	11.5172	8.5501	4.504	8.2691	10.9997	9.1742	7.0799	10.6519	8.392	1.224521994	low
TCGA-05-4432-01	2.084931507	0	9.7374	8.2279	7.7714	7.8402	11.8898	10.4326	9.1789	6.3736	8.7936	11.0768	9.268	7.6082	10.9202	8.0712	1.483791482	high
TCGA-44-2668-01	2.084931507	1	9.3933	8.6645	7.5751	7.3157	10.5285	10.6891	9.0994	6.4243	7.1564	10.8639	8.5133	6.5046	11.6537	6.8466	1.124002314	low
TCGA-55-7815-01	2.117808219	0	6.9686	6.7443	7.3158	5.7577	12.3738	11.4038	9.8405	7.5727	6.2123	9.9188	7.3158	7.0141	11.0349	8.8038	1.533659629	high
TCGA-97-7554-01	2.123287671	0	8.3627	8.1606	7.4966	6.9196	11.9585	11.2147	9.4217	5.4436	7.9255	9.9986	8.9491	6.4437	10.7706	7.641	0.979096952	low
TCGA-50-6592-01	2.128767123	1	9.4058	7.512	7.5255	7.6901	9.062	9.8888	9.6621	8.9149	7.6227	10.0016	8.5808	5.0305	10.2229	8.2945	4.307778405	high
TCGA-05-4426-01	2.167123288	0	9.0613	8.5747	9.2501	9.4233	11.4664	10.9527	9.1833	6.4715	8.1011	9.9151	9.5553	6.6048	10.1417	8.4628	0.881778653	low
TCGA-05-4427-01	2.167123288	0	8.6766	8.1031	8.9526	8.1267	10.826	10.5905	8.5089	4.8455	7.5444	10.9777	8.796	6.8771	10.1779	7.7238	1.082018148	low
TCGA-55-8513-01	2.167123288	0	8.1962	8.735	9.3867	9.7717	10.6456	10.3343	9.1359	5.577	8.7425	10.6734	9.614	6.4438	10.294	8.3185	0.900405506	low
TCGA-78-7539-01	2.167123288	0	8.5713	8.4257	9.1026	8.8266	13.4688	10.7504	8.7268	3.3407	9.317	9.9693	9.2027	6.823	10.4917	7.9168	0.386354372	low
TCGA-38-7271-01	2.191780822	1	8.6008	8.6643	7.9396	8.0594	10.8691	10.4802	8.905	5.2268	7.6813	9.8977	9.4042	6.427	10.9249	7.8896	1.044866161	low
TCGA-73-4666-01	2.191780822	0	10.1121	8.2767	8.3903	8.1081	11.4047	10.1611	8.3374	7.7264	8.0789	11.4508	10.4566	5.9686	9.8507	8.1367	3.804353588	high
TCGA-86-A4P8-01	2.205479452	0	8.0217	8.5392	9.3353	10.5292	10.4729	10.3569	8.6514	5.8229	8.3405	10.0195	9.1578	7.622	8.9382	7.2893	0.360876662	low
TCGA-86-8674-01	2.208219178	0	9.3018	8.8725	9.2561	9.4564	10.5676	9.08	8.2182	5.8801	9.4633	10.0353	10.3617	6.4148	11.2306	9.232	1.951567566	high
TCGA-78-7220-01	2.210958904	1	10.1935	7.6747	7.2265	8.053	11.3206	11.8245	8.8297	5.8356	6.8933	10.1543	8.8951	6.7068	10.6353	8.4652	1.6667249	high
TCGA-44-4112-01	2.21369863	1	9.3514	8.7041	8.3747	8.6661	11.8252	10.7857	9.4375	5.8487	7.6072	11.2526	8.6789	6.8612	10.542	8.8125	1.085514875	low
TCGA-69-8453-01	2.22739726	0	8.6842	8.691	8.8503	9.2333	10.8669	10.0774	8.9489	4.4882	8.0768	10.6332	9.4569	6.7642	9.8163	8.0898	0.888399697	low
TCGA-55-7570-01	2.257534247	0	10.1993	8.1286	7.6034	8.5401	10.4328	10.3296	9.5896	8.4833	7.7768	11.0322	10.9803	6.9779	9.8143	8.6553	5.409757661	high
TCGA-83-5908-01	2.257534247	0	9.7642	8.4491	8.212	8.5465	10.5403	11.5182	9.5431	6.624	7.8077	11.8952	9.3466	6.3661	10.0488	8.0292	1.94894298	high
TCGA-78-7145-01	2.263013699	1	9.2993	7.9728	7.8868	7.1656	11.06	9.6532	9.0904	6.4059	5.7996	10.9782	8.9649	7.427	10.6935	7.9895	2.434571094	high
TCGA-MN-A4N1-01	2.265753425	0	10.7439	8.7751	9.208	9.0326	10.6759	11.5506	10.5723	6.1792	8.399	10.1526	10.2109	7.0657	10.7585	8.0692	1.423290527	high
TCGA-50-8460-01	2.271232877	0	8.513	9.2051	9.2557	8.7453	11.1385	10.5695	8.5032	5.795	8.9561	9.1652	10.3893	6.2822	10.2024	9.0619	1.049713686	low
TCGA-95-8039-01	2.273972603	0	9.0236	8.8429	8.2529	9.0194	11.4027	10.4844	9.0065	5.4772	7.6288	10.5534	8.8111	6.4672	9.981	8.2602	0.767363238	low
TCGA-86-8671-01	2.298630137	0	8.9278	8.9553	9.3384	9.4698	11.4154	10.0582	8.8351	5.3655	8.0822	9.8178	9.6168	6.7682	9.358	7.732	0.586861312	low
TCGA-91-6847-01	2.306849315	0	9.4207	8.7712	8.246	8.5919	10.6946	10.8604	9.188	7.602	8.2148	11.0686	9.0352	7.9367	10.6092	8.4322	1.202532616	low
TCGA-44-6147-01	2.315068493	0	7.9668	8.8126	8.6747	8.6805	11.1735	10.4572	8.9812	4.582	9.1433	10.0099	9.671	7.4357	10.508	8.0405	0.62973591	low
TCGA-44-5645-01	2.334246575	0	7.9221	8.881	9.0134	9.6086	10.314	10.9817	9.7576	5.4604	8.5062	10.7418	9.7412	7.6236	9.6709	7.7635	0.629360642	low
TCGA-49-4487-01	2.342465753	1	8.3754	8.6481	8.6751	7.9385	11.1191	10.5905	8.7672	5.9658	8.2453	10.7099	8.9891	7.1293	11.1054	8.8166	1.210356611	low
TCGA-86-8673-01	2.361643836	0	10.0252	9.0737	8.9996	9.1869	10.9644	10.4297	9.6914	6.4754	9.0611	10.4945	9.3279	5.374	12.0946	8.2901	1.306213189	high
TCGA-44-5644-01	2.364383562	0	9.7463	7.6136	8.3787	9.7989	11.5752	10.5697	11.295	6.8585	7.8482	8.9936	8.9242	4.5201	10.4355	7.3686	1.001736445	low
TCGA-38-4629-01	2.367123288	1	10.3237	8.5104	8.8404	8.1541	11.2743	9.2693	9.0937	7.1758	7.6606	12.509	10.4201	7.4422	10.7987	8.2038	4.394383853	high
TCGA-64-5779-01	2.367123288	0	8.8611	8.384	8.5091	8.717	11.4195	10.3109	9.0399	5.9245	7.5886	12.6081	8.9757	7.8412	9.9672	8.384	1.445561909	high
TCGA-64-5815-01	2.37260274	0	9.1015	8.4042	8.0665	7.3815	11.3683	10.094	8.7505	4.7923	7.4673	10.6902	9.3835	5.8071	10.4448	8.5322	2.135594705	high

TCGA-49-4488-01	2.380821918	1	9.7839	8.5741	8.8941	7.5156	12.0779	10.835	9.6897	5.827	9.0817	10.6365	9.8565	7.0641	10.3095	8.5174	1.63749656	high
TCGA-55-7281-01	2.389041096	0	9.1868	8.5009	8.4757	8.4571	11.1944	9.9884	8.1935	4.4271	7.8985	10.2348	9.8012	6.0804	10.4148	8.6556	1.627599249	high
TCGA-49-AAR4-01	2.408219178	1	9.7936	8.7282	9.2969	10.0783	11.1018	10.5923	8.8613	5.649	8.8846	11.1313	10.2472	6.5498	9.6583	8.3064	1.268964627	high
TCGA-05-5425-01	2.416438356	0	10.0177	9.139	8.5952	7.6688	13.0577	11.7389	9.3919	4.9792	8.7511	10.844	10.216	6.5157	11.3071	8.6531	1.287603418	high
TCGA-44-7670-01	2.416438356	0	10.3952	8.1678	7.9596	7.5572	14.0683	10.8336	8.3795	5.6123	7.6198	10.7844	10.1102	6.3575	9.8524	8.4385	1.851657123	high
TCGA-55-8206-01	2.432876712	0	7.7628	9.223	8.5896	10.081	11.0965	10.579	8.2438	5.7112	8.5617	10.2924	9.0768	7.2859	9.6768	8.2578	0.369985887	low
TCGA-44-7671-01	2.435616438	0	8.7975	8.8605	8.4149	8.084	11.4254	9.9952	8.5226	5.9933	8.5065	10.0669	10.1584	6.592	10.6117	9.3879	1.934928826	high
TCGA-55-7995-01	2.435616438	0	9.5672	8.4458	7.8901	9.0827	11.2133	10.14	9.0133	5.634	7.6713	11.1014	10.0926	5.5494	10.9816	6.9115	1.52737692	high
TCGA-49-4510-01	2.454794521	1	9.9626	9.092	9.4177	9.3195	11.5167	10.0965	8.727	5.2125	8.7139	9.7324	8.9295	7.3075	9.5836	8.5454	0.568618376	low
TCGA-86-A456-01	2.454794521	0	8.3049	8.6511	10.0876	9.1741	11.6241	10.0837	10.4655	5.6801	8.4115	10.9361	9.6525	6.9476	9.8778	8.3396	0.996046043	low
TCGA-MP-A4TE-01	2.454794521	1	9.3596	9.1563	8.711	8.5423	11.5956	10.2547	9.4829	5.8755	8.6379	10.7209	9.1861	7.0712	10.5648	8.9649	1.067298428	low
TCGA-55-8085-01	2.476712329	0	9.2549	8.7987	9.4627	9.1885	12.13	10.0175	8.9444	6.0907	8.7772	10.916	9.8514	6.5743	10.4452	8.7471	1.264549038	high
TCGA-49-4512-01	2.479452055	1	9.1347	9.0886	8.8761	9.9979	11.051	9.502	9.0133	5.7142	8.7683	10.899	9.7578	6.5781	10.0066	7.966	0.886694718	low
TCGA-62-A472-01	2.493150685	0	9.2041	8.0629	9.6443	9.1755	9.4998	9.9921	9.8514	7.2161	7.7566	10.5847	10.0285	7.3243	9.5378	7.9936	2.217286161	high
TCGA-05-4420-01	2.498630137	0	10.4742	8.2966	8.2236	8.5187	13.1796	10.6785	9.4176	6.6229	7.2016	11.5517	8.9953	8.1115	10.3959	8.1494	1.113104752	low
TCGA-05-4424-01	2.501369863	0	8.9396	8.681	8.1528	7.2534	11.5877	11.5679	8.1576	4.7914	7.5313	11.4731	9.6254	7.2352	10.3986	8.6743	1.602870958	high
TCGA-73-4675-01	2.526027397	1	9.3053	8.9588	8.6781	9.3436	9.8028	7.3996	9.5645	5.695	7.8235	10.3939	10.2167	5.7122	10.4487	8.3431	2.789197219	high
TCGA-53-7626-01	2.545205479	1	8.6811	8.5666	7.7931	8.1775	10.5068	10.7025	8.4517	5.7298	7.4071	9.6413	9.2335	7.0053	10.1128	8.1295	0.978817937	low
TCGA-55-5899-01	2.547945205	0	10.1969	9.1409	7.875	7.8466	11.9439	11.6725	11.2392	7.1102	7.8322	9.9163	11.3952	6.4759	9.7972	7.3413	1.689619641	high
TCGA-86-8669-01	2.569863014	0	8.4875	9.1817	8.525	8.4385	12.6294	10.6119	9.1177	6.8302	7.466	10.6136	8.9513	7.337	10.1551	8.3012	0.593252125	low
TCGA-86-8278-01	2.58630137	0	8.3845	9.0818	8.448	9.3193	11.1929	10.5288	9.0797	4.526	7.8403	10.1143	10.0648	5.5984	10.5852	7.3144	0.69880487	low
TCGA-86-7701-01	2.594520548	0	9.9365	7.6374	8.0294	7.8301	10.3929	10.4785	9.7461	6.6446	7.2349	10.8723	9.7965	7.3605	10.6726	8.4804	3.850390581	high
TCGA-78-7535-01	2.6	1	10.1789	8.211	9.2655	9.4269	10.8369	9.2862	9.5206	6.4837	8.34	10.6338	10.6801	5.9067	10.3264	9.0586	4.0383967	high
TCGA-86-8279-01	2.6	0	8.6718	8.6915	9.026	9.2677	11.5888	10.4286	8.2329	5.0573	8.822	10.4967	10.3639	6.3968	11.1372	8.7413	1.28435347	high
TCGA-MP-A4TA-01	2.602739726	1	10.2427	8.9083	8.9549	8.6929	10.9445	9.5668	10.1601	6.596	8.4596	10.914	10.8654	6.9613	10.2116	8.2323	2.729547734	high
TCGA-55-7227-01	2.608219178	1	8.3722	8.354	8.574	9.3334	10.3206	10.6502	9.108	5.0352	7.0627	10.1076	9.5309	7.2315	9.6696	9.007	1.192157991	low
TCGA-78-7156-01	2.673972603	1	8.0816	8.2264	10.0183	8.8316	9.7961	10.3417	8.545	5.5742	8.5806	10.1583	10.6228	7.2072	9.83	9.3338	2.33752114	high
TCGA-55-8207-01	2.676712329	0	8.2817	8.7759	9.4974	9.1934	11.118	10.4097	8.3363	5.7558	8.3427	9.6316	9.2693	6.8553	10.0131	7.9243	0.547204	low
TCGA-44-6777-01	2.704109589	1	8.0673	8.4502	8.0037	7.0895	11.064	12.4424	8.3398	6.3484	6.4474	10.2381	9.1432	6.7164	10.3092	8.1945	1.110142452	low
TCGA-J2-A4AG-01	2.706849315	0	8.1038	7.8138	8.4533	9.3516	10.6224	9.9868	9.1144	5.8956	7.9644	10.5083	8.5388	6.2769	11.2134	9.05	1.394301628	high
TCGA-86-8076-01	2.720547945	0	8.2174	8.8775	8.6246	8.5545	10.7596	10.3006	9.4267	6.0033	9.0571	10.0897	9.4044	7.1547	10.9202	9.2337	1.144734399	low
TCGA-55-6982-01	2.726027397	1	8.8544	7.5486	7.5767	7.7224	13.0188	13.3433	9.887	4.4727	6.8836	11.4111	9.894	6.5132	10.3503	7.658	1.291281487	high
TCGA-55-7574-01	2.726027397	1	8.7524	8.5349	9.2728	9.4875	10.389	10.0776	8.5594	5.4809	7.8969	10.2198	8.8223	6.6471	10.1828	8.5835	0.901182436	low
TCGA-86-7953-01	2.731506849	0	10.7109	8.1796	7.9123	8.6998	11.3664	12.4248	8.6812	5.9783	7.3321	10.6977	9.4579	7.0908	10.0282	8.2373	1.338559509	high
TCGA-49-4506-01	2.736986301	1	10.6967	8.0981	9.0398	9.4177	11.038	8.6444	11.5449	8.7131	8.6344	10.984	10.066	5.0197	12.1185	8.9671	6.213258422	high
TCGA-44-5643-01	2.775342466	0	9.6262	9.7004	7.8857	10.6468	10.5002	10.8996	8.4571	5.1211	6.6782	10.9974	10.2341	7.1653	9.1733	8.3239	0.76395858	low

TCGA-44-3919-01	2.810958904	1	9.3851	8.5343	8.5631	8.6878	11.4685	11.1172	9.1836	6.6698	7.4048	10.275	9.3503	7.0199	10.0864	8.463	1.113721345	low
TCGA-44-3918-01	2.838356164	0	8.7368	8.1955	8.5918	8.238	11.8944	9.9672	9.1674	6.9159	7.8056	9.9705	8.1651	7.147	10.1642	8.1785	0.761321577	low
TCGA-55-7910-01	2.849315068	0	9.4437	8.1514	8.6706	8.3114	10.4598	11.6224	10.3648	5.284	8.1135	9.4418	9.1276	7.1471	10.8932	8.5259	1.130479683	low
TCGA-53-7624-01	2.857534247	1	9.4979	7.9454	8.0181	7.8108	10.814	9.6499	9.5135	5.9856	7.9221	11.337	9.8737	6.0729	9.6172	7.6964	3.183718859	high
TCGA-86-7711-01	2.865753425	1	9.6722	8.1109	7.1819	7.4506	12.5778	9.7858	7.9571	7.1222	7.7616	11.6246	9.3362	4.433	11.8691	7.8266	3.180550294	high
TCGA-99-8025-01	2.904109589	0	10.007	8.3497	9.0173	9.115	10.76	10.2772	9.2235	6.7365	9.0584	10.8152	9.6482	6.7046	10.6822	8.1408	1.688040258	high
TCGA-53-A4EZ-01	2.934246575	0	10.2874	8.8071	8.7906	9.9095	12.665	11.6184	10.3448	5.9072	8.3026	10.7282	9.7017	6.3375	10.2233	8.4233	0.828506705	low
TCGA-86-7955-01	2.936986301	0	9.4657	8.7701	9.3506	8.9452	11.3884	10.5086	9.3796	7.422	7.963	11.4807	10.7634	6.8209	10.1321	8.5443	2.500721518	high
TCGA-38-4630-01	2.939726027	1	9.8701	9.5361	9.4659	8.5432	11.8911	9.603	7.4268	6.4462	8.4512	10.9937	10.1048	6.5212	10.4072	8.9861	1.614352391	high
TCGA-J2-A4AE-01	2.956164384	0	8.6047	8.5557	9.8722	10.4985	11.0581	11.0217	9.2948	7.1523	8.3226	10.1487	9.5447	7.5797	9.7879	9.0123	0.739467257	low
TCGA-49-4494-01	2.961643836	1	10.4796	9.2134	8.8759	7.5555	12.0068	10.1043	10.1729	6.574	7.9142	10.9028	10.2934	6.6038	10.3823	7.921	2.047446373	high
TCGA-44-7667-01	3.005479452	0	9.9339	8.9032	8.0946	6.5219	10.8692	11.2341	9.0095	7.1138	7.5087	10.2572	9.5605	7.2335	10.1395	8.0225	1.825544047	high
TCGA-75-6214-01	3.054794521	1	10.1337	7.8737	7.8549	9.6011	10.6779	10.5305	9.343	6.0899	7.1114	10.9635	9.9061	6.9994	10.1933	8.3431	2.26263759	high
TCGA-99-8028-01	3.063013699	0	8.7808	8.6784	9.1858	9.0114	11.2885	10.191	9.1181	5.5304	8.1319	10.4735	9.0842	7.108	10.2026	8.5249	0.866063969	low
TCGA-50-8459-01	3.065753425	0	8.2005	8.412	8.6834	8.5149	10.6634	10.181	8.6294	4.9641	8.0953	10.4106	9.398	6.768	9.5446	8.0594	1.044517226	low
TCGA-50-8457-01	3.082191781	0	8.1529	8.6407	8.8688	9.2207	10.5773	10.7623	8.3502	5.0031	8.6407	9.8576	9.2991	6.8296	10.039	8.7081	0.745859787	low
TCGA-05-4390-01	3.084931507	0	9.4213	9.0684	8.7848	7.2208	11.2841	10.9271	9.179	5.6672	8.2191	9.9748	8.5567	7.3952	10.4196	8.9222	0.926323301	low
TCGA-64-1680-01	3.084931507	0	9.4291	9.2226	9.6559	8.9004	11.3659	10.1577	9.2208	5.7764	8.6971	10.1223	10.0107	6.9922	10.2884	9.6977	1.436947948	high
TCGA-44-3396-01	3.095890411	0	9.658	8.9625	8.9118	8.1329	11.2537	10.1522	9.1639	6.3576	8.1409	10.2346	10.5321	7.6863	9.8733	8.366	1.648012297	high
TCGA-49-AARN-01	3.109589041	1	8.9361	9.011	8.8937	9.5684	11.0276	10.0549	9.1464	5.2598	8.7156	10.178	10.0889	6.2967	10.5095	7.8967	0.885169154	low
TCGA-38-4627-01	3.142465753	1	9.0126	8.7532	8.3425	7.2825	11.5864	10.3765	8.775	5.2282	7.8979	10.7089	9.1889	6.8062	10.0756	8.5492	1.392060804	high
TCGA-86-8054-01	3.145205479	0	9.3199	8.4471	8.0136	8.6329	10.6377	11.8151	9.726	7.128	8.0719	9.8639	9.1063	7.5711	10.8138	8.4207	0.971256356	low
TCGA-86-7713-01	3.169863014	0	9.3164	8.3333	9.3798	9.9737	10.6152	10.5505	9.0734	5.2674	8.4589	10.1635	9.7947	7.7548	10.3829	8.6077	0.996172159	low
TCGA-44-2661-01	3.175342466	0	8.9123	9.0957	9.2089	8.7398	12.0953	10.7498	8.97	6.1907	8.4057	10.4622	9.2321	7.579	10.4429	9.0842	0.76635256	low
TCGA-44-3398-01	3.18630137	0	9.5729	8.0371	8.7356	8.9293	11.364	10.4858	9.3439	7.5124	7.7462	10.5589	8.3845	7.9373	10.5237	8.9705	1.18747188	low
TCGA-64-1681-01	3.197260274	1	8.4915	8.5357	8.3564	8.2542	11.4394	9.6985	9.455	5.0517	8.5827	11.112	10.2498	7.364	10.0444	9.1978	2.168230869	high
TCGA-78-7155-01	3.208219178	1	9.7537	8.8793	7.4904	7.5451	11.1211	11.94	9.8787	7.9242	7.0357	10.9413	8.1672	8.8196	10.9934	8.0835	0.808639941	low
TCGA-MN-A4N4-01	3.219178082	0	8.8037	8.6892	8.4285	8.8359	11.7847	10.3149	9.1905	6.2194	8.5756	10.4612	9.5783	6.4588	11.3503	7.5012	0.855176649	low
TCGA-55-1594-01	3.22739726	0	9.8657	8.7035	8.2219	8.4531	13.6345	11.7395	9.2109	6.7798	7.8066	10.49	10.2563	7.9396	9.9875	8.0116	0.809124092	low
TCGA-64-1678-01	3.257534247	0	11.7136	9.2565	8.6672	8.2764	12.6104	10.8303	9.4094	8.5167	8.4253	10.941	9.0655	8.9418	10.5547	9.2763	1.254294529	low
TCGA-73-7498-01	3.257534247	0	8.6449	8.6596	9.5757	10.5664	10.6	10.095	8.0622	4.643	8.8386	9.3988	10.3214	7.5895	9.7506	7.4153	0.47152296	low
TCGA-62-A470-01	3.271232877	1	9.257	7.9996	8.8505	9.6443	12.0607	10.56	9.7052	6.7403	8.2241	11.3918	10.0946	7.9501	10.4701	8.7259	1.619364631	high
TCGA-78-7540-01	3.279452055	1	8.7774	8.3075	8.4263	8.6173	11.1594	9.5275	10.1324	4.5769	7.121	10.6889	10.2399	5.1211	11.0056	8.0269	2.450986159	high
TCGA-78-8648-01	3.312328767	1	8.7671	8.9894	8.7493	8.8531	11.2296	10.286	8.018	5.1448	8.0349	10.36	9.2415	6.6593	9.8845	7.7321	0.648964975	low
TCGA-78-7152-01	3.328767123	1	9.1002	9.3402	8.0583	8.4402	10.9381	10.4351	9.1646	5.3873	7.7514	10.6362	9.5424	5.5391	10.4958	9.1035	1.589675081	high
TCGA-62-8395-01	3.331506849	0	8.498	9.1254	9.011	9.2207	10.7991	11.1681	8.0971	3.9921	7.9359	9.3793	10.2823	6.9	9.5544	8.6026	0.683494129	low

TCGA-49-AARE-01	3.367123288	1	9.4459	8.7087	8.2974	9.1877	12.0336	8.9772	9.1338	5.5814	9.1844	11.1424	10.4249	5.9958	10.5997	6.9635	1.293505078	high
TCGA-55-6985-01	3.378082192	0	9.3382	8.5095	7.7544	7.8601	11.2508	10.5794	9.1079	5.4928	8.0829	9.9715	9.4112	7.1196	10.4407	8.0829	1.160208742	low
TCGA-50-5932-01	3.383561644	1	8.3725	8.4153	7.6415	8.4586	11.5675	11.0561	9.0422	5.0445	8.2983	10.2007	9.9357	7.2228	10.0555	8.4083	1.025218381	low
TCGA-55-6969-01	3.394520548	0	9.1838	8.316	8.0567	8.0918	11.2145	11.1361	9.8786	7.3002	8.1768	11.0915	9.3516	8.0284	11.0713	8.6455	1.656511555	high
TCGA-62-A471-01	3.41369863	0	10.4856	8.9971	8.4357	8.9738	10.5065	10.8873	9.9834	6.327	7.7227	12.0054	9.2263	5.1404	10.3491	8.6892	2.514488481	high
TCGA-91-6829-01	3.446575342	1	8.5399	8.1148	7.7113	6.9765	10.9993	12.0614	8.5852	5.8656	8.0002	10.6089	9.4587	7.2007	10.6512	8.0822	1.481783313	high
TCGA-MP-A4T9-01	3.465753425	1	9.1601	8.4831	8.4408	9.2721	11.241	10.5237	8.9036	5.8862	8.078	10.8493	9.5479	6.3757	9.5954	7.9767	1.111876314	low
TCGA-50-6597-01	3.473972603	1	8.6529	8.3578	8.2547	8.0848	12.7901	10.058	9.696	5.749	8.6325	11.474	10.4528	5.0046	9.6306	8.1956	2.377556972	high
TCGA-95-7039-01	3.484931507	0	9.0032	7.8826	8.4612	8.5157	11.9535	10.3279	9.0467	6.1926	8.016	10.6129	10.4683	6.5078	10.0463	8.0935	2.092528996	high
TCGA-44-2662-01	3.506849315	0	9.5877	8.5137	8.4275	8.1945	12.0445	10.305	8.5556	7.6093	7.5498	11.9192	9.367	6.7929	10.3004	7.9917	1.814947979	high
TCGA-97-7546-01	3.520547945	0	7.5086	8.4127	8.3448	8.2391	10.6539	10.693	8.4859	6.1903	8.165	10.2097	8.5129	7.7305	10.8728	9.1211	0.877639349	low
TCGA-50-6590-01	3.528767123	1	9.0844	8.5208	7.6704	7.4594	10.7891	10.2143	9.5877	7.228	7.2944	11.1816	8.9996	6.0372	10.5564	7.6275	1.978202068	high
TCGA-62-8397-01	3.531506849	0	7.8283	8.6889	8.699	9.5193	12.3865	10.8099	8.0027	4.8448	7.6615	9.9049	8.8932	6.6686	8.9282	7.9862	0.334459437	low
TCGA-55-6968-01	3.542465753	1	9.9787	7.7216	7.8667	8.4496	10.0935	11.2658	9.2716	7.4747	8.0461	11.5812	9.6977	6.2258	10.4844	7.992	3.508082371	high
TCGA-44-2665-01	3.564383562	0	8.6908	8.5985	9.0651	8.4479	10.9451	10.3974	9.074	5.5551	8.6299	10.0037	8.6641	7.5648	11.0605	8.0968	0.665884838	low
TCGA-64-5778-01	3.575342466	0	10.5304	9.1828	9.6802	8.692	12.1246	10.7467	9.1634	5.5917	9.5504	11.205	8.9285	7.8282	10.3935	8.5613	0.749476416	low
TCGA-44-2655-01	3.62739726	0	9.0451	8.6101	9.428	9.2043	11.2431	9.7568	8.6928	6.0273	9.0042	10.2369	10.6742	7.1166	10.2538	9.0627	1.810766015	high
TCGA-75-5147-01	3.652054795	0	10.0333	9.4078	9.0903	9.0958	12.0946	10.4105	9.2864	5.5087	8.6007	10.9282	9.6415	7.6033	11.111	8.7335	0.881844552	low
TCGA-44-2657-01	3.701369863	0	9.1103	8.9551	8.8974	9.0568	11.6528	10.5709	9.0292	5.5225	8.2935	10.1791	9.4268	6.6678	9.934	7.2607	0.550161615	low
TCGA-38-4632-01	3.717808219	1	11.032	8.6417	9.17	6.9555	11.8553	10.959	9.496	6.8254	8.6053	10.5215	8.766	7.2946	10.884	9.1449	1.942870351	high
TCGA-44-2659-01	3.745205479	0	8.3743	8.8164	8.7932	8.629	12.3948	10.985	9.6049	4.7278	7.4878	9.2191	9.0787	7.5965	9.7814	8.4246	0.414642053	low
TCGA-05-4389-01	3.750684932	0	10.0302	8.5462	8.5001	8.1862	12.3736	10.9282	9.4684	6.2447	8.0002	10.3361	9.6655	7.267	10.467	8.0301	1.115736626	low
TCGA-55-6981-01	3.778082192	1	9.0616	8.6818	8.1062	8.2669	11.8942	10.5133	9.131	5.4873	7.8118	11.086	10.2332	6.6456	10.1742	8.7273	1.895927559	high
TCGA-55-6971-01	3.835616438	0	8.4963	7.9812	9.0774	8.6981	10.597	10.5587	9.1529	5.7089	7.4848	9.6657	9.0193	7.062	9.9089	7.4372	0.786563262	low
TCGA-49-4501-01	3.893150685	1	9.3811	8.8666	8.9984	9.1933	12.3892	10.8245	9.1564	6.05	8.6569	11.0082	10.0465	7.3342	10.5239	7.9953	0.85804482	low
TCGA-44-2656-01	3.915068493	0	8.6037	8.134	8.6609	8.2282	11.1375	10.4532	9.3071	7.0161	7.6313	11.3043	9.6024	6.7261	10.3667	8.0699	1.970795892	high
TCGA-05-4398-01	3.920547945	0	9.9499	8.9338	9.2996	8.2692	10.9006	9.6078	9.656	7.4331	8.4834	10.6039	9.9077	6.7462	11.1386	8.6196	2.340809891	high
TCGA-NJ-A4YQ-01	3.923287671	0	9.3924	8.3015	8.9237	9.6721	11.2757	9.4428	8.6694	6.2423	8.6493	10.9233	10.5658	6.9102	10.5825	8.0445	1.825071539	high
TCGA-50-5066-01	3.950684932	0	11.1793	8.8727	9.8011	7.8624	12.422	9.8925	9.556	8.0208	8.5259	10.9475	8.4235	8.2194	11.0636	8.3901	1.140930953	low
TCGA-62-A46O-01	3.983561644	1	10.2782	7.5738	8.1786	8.6995	11.2825	10.3496	9.5141	6.3247	7.9043	11.714	8.7767	5.3623	11.1301	8.5139	3.136703948	high
TCGA-50-5941-01	4.038356164	0	9.4199	8.4553	7.9604	8.282	11.676	9.943	8.9906	6.0666	7.4004	9.8982	9.4781	6.5439	9.9654	8.3201	1.372915976	high
TCGA-55-1595-01	4.052054795	0	9.4211	8.7445	10.1145	8.9971	10.755	10.9892	8.6258	6.382	8.1891	9.248	9.515	7.0302	10.3886	7.8677	0.703773721	low
TCGA-38-4628-01	4.087671233	1	9.3552	9.0612	8.5923	7.577	12.7718	10.7836	8.5258	6.6519	8.5982	11.1974	9.8131	7.6355	11.2376	8.1824	1.124907726	low
TCGA-62-8402-01	4.104109589	1	9.9344	8.5953	8.9914	9.6235	12.3072	11.9661	10.036	6.3798	7.7067	10.3532	10.2888	6.2174	9.8013	8.5824	1.169492045	low
TCGA-50-5068-01	4.106849315	1	11.2716	9.5785	9.4038	9.2441	11.6934	10.325	9.4415	7.2912	9.3405	10.8617	8.8217	7.4757	11.1263	8.2505	0.745072043	low
TCGA-MP-A4SY-01	4.112328767	1	8.5642	8.0875	8.9232	8.4075	10.6577	11.2811	8.7526	8.6707	8.0337	11.1622	9.4282	6.3795	9.8029	8.4276	2.077888976	high

TCGA-75-6212-01	4.153424658	1	8.5362	8.3557	9.0053	8.5392	10.679	11.3285	8.2599	7.0775	7.3771	10.1145	9.8183	6.1208	9.5113	9.0334	1.813084207	high
TCGA-05-4249-01	4.17260274	0	8.5598	8.3338	7.9062	8.4723	11.1261	10.5443	8.2027	5.0389	8.1316	10.0659	9.1594	6.9006	11.159	8.4631	1.004494489	low
TCGA-78-7633-01	4.18630137	1	9.0074	8.2569	9.1407	8.4415	11.0037	10.7873	7.8375	5.4749	8.5057	9.6396	10.3104	7.6267	10.4586	8.6936	1.38982139	high
TCGA-73-7499-01	4.194520548	1	10.1122	8.6149	8.9287	8.7154	12.4037	11.3785	9.4357	5.8742	9.2056	10.3241	9.562	7.0607	10.1303	8.5653	0.927499917	low
TCGA-64-5781-01	4.271232877	0	10.0474	8.3189	7.9725	8.3703	11.6365	9.4286	10.6125	6.4026	8.3925	10.4628	9.7322	5.7986	11.5385	7.6648	2.101352327	high
TCGA-73-4658-01	4.383561644	1	8.787	8.227	7.9499	7.4634	11.4174	11.1439	8.7567	4.62	7.3408	11.5694	8.6615	6.5753	10.1904	8.0155	1.296242026	high
TCGA-50-5946-01	4.430136986	0	9.1886	8.2279	9.1507	9.5954	11.4194	12.022	9.3559	6.1125	8.4525	10.2694	10.435	8.0642	11.0349	7.7732	0.833308225	low
TCGA-49-6743-01	4.44109589	0	9.417	8.0123	7.4615	7.1114	10.9268	11.2274	8.4485	5.7739	7.398	10.4125	9.0125	6.2661	11.1048	7.6448	1.687584406	high
TCGA-78-7537-01	4.443835616	1	8.5249	9.9907	9.0251	9.901	11.4522	10.1603	8.3702	4.4386	8.1415	10.2301	9.6313	6.2594	11.3216	8.7673	0.516169482	low
TCGA-55-6972-01	4.471232877	1	9.7701	9.1793	11.1075	8.121	11.2264	10.3033	8.6499	5.7107	10.0902	10.1823	9.9323	8.079	10.6937	7.4472	0.714087468	low
TCGA-62-A46S-01	4.528767123	1	8.2452	7.9986	9.5037	8.9932	11.3039	10.1409	9.0351	5.5655	8.1983	10.0962	9.5503	6.5771	10.7795	8.5775	1.264402047	high
TCGA-49-6744-01	4.610958904	0	8.2404	8.6036	8.4897	8.0457	10.7894	10.6501	9.3011	5.2153	7.388	11.1259	9.5605	7.0704	10.347	8.7575	1.640749864	high
TCGA-49-4514-01	4.657534247	0	10.3755	9.1955	8.2623	8.4633	11.4838	10.5298	9.6599	4.027	7.9726	10.8475	10.5869	5.5751	11.3591	8.7744	2.360874042	high
TCGA-62-A46R-01	4.726027397	1	9.0409	8.1091	9.2767	9.4004	11.5102	10.4616	10.0437	5.8335	8.4373	11.1846	10.0306	6.9722	10.1762	7.6445	1.278189258	high
TCGA-64-1676-01	4.734246575	0	10.5498	8.6665	8.6215	8.2035	11.8431	10.131	8.4203	6.1352	9.3887	10.9231	9.2162	7.4216	10.9112	8.7732	1.491768447	high
TCGA-50-5944-01	4.794520548	0	8.3783	8.3967	8.134	8.3217	11.3328	11.2111	8.6608	5.1603	7.7929	10.5636	9.0002	6.9422	9.8475	8.7473	0.979749398	low
TCGA-MP-A4SW-01	4.871232877	1	9.5527	9.1333	9.0328	9.5992	11.5663	11.0232	9.3145	6.6802	7.9425	10.4174	10.3789	6.398	10.1815	7.7905	0.943219119	low
TCGA-MP-A4T6-01	4.904109589	1	8.6774	9.0533	9.6994	11.052	11.9676	9.2024	9.2843	4.4742	9.4973	10.0717	10.5913	6.4907	11.2618	7.3104	0.488618474	low
TCGA-O1-A52J-01	4.926027397	1	8.2721	8.931	9.4011	9.4164	12.4284	11.1021	7.728	5.4298	8.8614	10.9502	10.1892	6.3123	9.4164	7.8194	0.622967252	low
TCGA-50-5055-01	5.01369863	1	9.2164	8.9421	8.6306	8.553	12.1978	10.1161	9.7714	5.1653	8.3365	10.2412	9.6691	7.0915	10.1732	8.6723	0.976016831	low
TCGA-50-5942-01	5.060273973	0	7.6472	8.6597	8.6778	9.0525	10.5705	10.7097	8.5848	5.0429	7.9677	9.705	9.8708	7.3179	9.842	8.6413	0.816396064	low
TCGA-44-6778-01	5.106849315	0	8.8527	8.3312	8.3227	8.5978	10.2192	10.639	9.0083	6.918	7.7038	10.2932	9.1901	7.8177	10.0114	8.2821	1.208680427	low
TCGA-97-7553-01	5.123287671	0	8.4755	8.1681	8.4881	8.715	11.2416	11.0324	8.8313	5.6342	7.7046	10.5078	9.4516	7.0857	9.8612	7.9562	0.969852465	low
TCGA-49-AAR3-01	5.18630137	0	9.9213	8.8125	8.04	8.77	10.7347	9.283	9.287	7.2697	8.1053	11.7649	9.9174	6.3423	9.8591	8.0495	2.671644588	high
TCGA-97-7552-01	5.293150685	0	7.6773	8.4881	8.2423	8.1765	10.5298	10.9185	9.6446	4.8297	7.6374	10.1998	9.4664	6.9406	10.6403	7.7507	0.898683489	low
TCGA-97-7547-01	5.383561644	0	8.1595	8.3223	8.3262	9.4771	10.7221	11.9088	8.5227	4.8366	7.2969	10.5517	9.4744	6.649	11.8596	8.0083	0.79835846	low
TCGA-78-7159-01	5.408219178	0	9.1242	8.0221	7.9142	8.7925	11.3449	10.7017	10.1511	6.8041	7.9755	10.6288	8.8332	6.9004	10.9521	8.6691	1.415097872	high
TCGA-75-5125-01	5.553424658	1	9.408	7.6399	9.0635	7.9264	10.4501	10.3849	9.1313	7.3566	6.9078	10.7238	9.2618	7.6876	10.4009	8.2581	2.518437667	high
TCGA-55-1596-01	5.657534247	0	9.707	8.3225	8.0662	8.0662	12.9317	9.7111	10.5134	6.2516	8.362	10.8169	9.2264	6.8854	10.1482	8.2542	1.390089797	high
TCGA-62-A46U-01	5.663013699	0	9.0698	8.0603	9.1544	8.7221	11.9419	10.9632	8.9574	5.688	7.4902	10.241	9.5209	6.7531	10.524	8.0581	1.066526654	low
TCGA-55-6980-01	5.778082192	0	7.8061	8.047	8.7146	8.4988	11.3577	10.5749	8.8171	6.2675	8.4003	11.4681	9.4826	6.8012	9.725	8.3582	1.472880859	high
TCGA-55-6987-01	5.854794521	0	8.9778	8.4078	8.4018	8.4951	11.0784	10.4005	9.5335	7.9817	8.2275	10.1496	8.8903	7.3649	11.6729	7.4343	0.88921016	low
TCGA-NJ-A4YF-01	5.920547945	0	9.4679	8.4823	8.9617	8.6704	12.366	11.5978	8.584	4.4766	8.9315	10.0547	9.8954	7.6148	10.7551	8.3676	0.730966538	low
TCGA-50-5045-01	5.956164384	1	9.8727	9.306	9	8.376	11.9094	10.2796	9.0709	5.7288	8.5259	10.1389	9.4324	7.0459	10.3514	8.1519	0.785650025	low
TCGA-62-A46V-01	6.024657534	0	8.7673	8.8284	9.4599	10.4139	11.7277	9.6696	8.2157	5.1393	8.465	10.195	10.2659	6.1593	10.7861	8.1056	0.795799713	low
TCGA-49-AAR2-01	6.093150685	0	10.5171	9.4942	8.7453	10.5214	11.8098	9.2424	9.6209	5.7271	9.2997	10.4031	10.9906	6.4633	9.1724	8.884	1.348169936	high

TCGA-MP-A5C7-01	6.15890411	0	8.5678	8.5164	9.7662	9.5769	11.7224	10.0628	8.4305	4.5743	9.2629	9.7586	10.2386	6.4753	10.7192	7.0992	0.585726768	low
TCGA-NJ-A4YG-01	6.194520548	0	8.8563	9.1171	9.0589	9.1672	11.154	10.7078	8.7578	6.518	8.8033	9.9879	9.5866	6.8992	10.3761	8.2012	0.702109648	low
TCGA-49-4486-01	6.350684932	1	9.2763	9.9012	10.2131	9.3779	10.8292	10.1932	8.4962	5.8709	9.2403	10.2541	10.8064	7.1427	10.764	9.1845	1.16041438	low
TCGA-78-8655-01	6.465753425	0	9.3047	8.834	8.6185	9.3738	11.5751	10.0583	9.2799	4.4939	8.5445	9.4877	10.2294	6.3808	10.0922	8.576	0.985431292	low
TCGA-75-5146-01	6.487671233	0	9.8536	8.2461	8.3656	8.771	11.8868	10.6086	8.2258	6.427	8.5542	10.6469	9.8711	8.4528	11.0837	8.3248	1.234511034	low
TCGA-50-5933-01	6.556164384	1	9.4639	8.1916	8.428	7.3006	10.9744	10.8869	9.9165	7.148	7.7016	10.7938	8.2289	6.8619	11.927	8.3646	1.691477798	high
TCGA-55-6642-01	6.709589041	0	7.2453	7.9659	8.2927	8.0856	11.008	11.0594	8.4551	5.9681	7.6781	9.7285	8.5764	6.056	10.3383	8.3372	0.859695436	low
TCGA-64-1679-01	6.816438356	0	8.9046	8.2871	7.1677	7.1052	11.2849	11.3528	8.4777	5.1745	6.0634	11.1514	8.5125	7.4305	10.9286	8.6079	1.471962642	high
TCGA-73-4662-01	6.890410959	0	8.5386	8.8022	9.0308	8.4023	12.0904	10.7641	8.5692	5.3188	8.8188	10.1749	9.8284	7.6219	10.2438	8.226	0.70754073	low
TCGA-75-6206-01	7.095890411	0	8.3051	8.2584	8.9104	8.9254	10.6996	9.8173	8.3051	5.4527	7.8887	9.5805	9.2738	7.4435	10.17	8.3251	0.878850611	low
TCGA-80-5611-01	7.109589041	0	9.7603	8.409	8.8324	7.4958	12.4201	11.1936	9.0498	6.431	9.0709	11.0834	10.0583	6.3545	11.0077	7.9763	1.84880242	high
TCGA-44-6776-01	7.167123288	0	8.5601	8.6272	8.044	9.482	11.6344	10.8257	8.3789	4.2449	8.635	10.0389	9.9326	5.9557	10.5905	8.8022	0.925131801	low
TCGA-MP-A4T4-01	7.169863014	1	9.5038	8.455	9.3142	9.1703	10.5529	9.8892	8.8107	7.0121	8.0281	10.7697	9.7358	7.2733	10.131	7.577	1.344789183	high
TCGA-MP-A4SV-01	7.178082192	1	9.2542	8.2198	9.0737	9.3992	11.0868	10.0968	9.0389	7.4861	8.1103	10.7879	9.5074	7.0189	10.6766	8.4612	1.567920784	high
TCGA-64-5774-01	7.331506849	0	9.7741	8.1947	8.1692	7.8792	11.2261	11.5751	9.0758	6.7318	7.0032	10.5573	9.6196	5.8718	10.4639	8.4302	2.321820556	high
TCGA-78-7167-01	7.345205479	1	8.9861	8.6989	9.2075	9.7974	10.4498	9.7008	8.421	4.9823	9.0129	9.0465	10.333	8.4894	10.2973	7.9348	0.639698267	low
TCGA-62-8399-01	7.38630137	0	8.2369	8.0416	8.5407	8.8566	10.9962	11.2006	8.2387	7.2033	8.7655	10.0849	10.216	7.2999	10.4352	8.8707	1.548698001	high
TCGA-55-6983-01	7.734246575	0	9.2574	8.6411	8.1496	8.448	10.4379	10.654	9.091	5.9623	8.1764	10.6355	9.8689	6.6014	11.0093	8.7413	2.026190464	high
TCGA-80-5608-01	7.75890411	0	9.6755	8.8171	8.2871	8.9749	11.2672	10.9816	9.1749	5.8604	7.8305	10.7875	9.7427	7.0925	10.7196	7.6384	0.976088245	low
TCGA-38-4625-01	8.145205479	0	9.6902	9.7761	8.8332	7.2999	12.2812	8.7897	9.6774	8.6163	8.7134	12.6354	8.4909	7.9091	11.679	8.5818	1.552205748	high
TCGA-75-7027-01	8.380821918	0	10.0991	7.984	7.6029	8.5543	10.288	10.7862	9.9275	5.3862	8.0996	11.514	10.0629	6.6771	10.3069	7.4027	2.508710258	high
TCGA-50-5049-01	8.476712329	0	9.8535	8.5674	8.2664	7.588	11.2636	10.1441	9.2323	5.9887	7.8616	10.442	9.1736	7.1907	10.2818	8.1995	1.51370855	high
TCGA-78-7162-01	8.682191781	1	8.8473	8.374	8.7562	8.6551	11.3455	10.6524	8.2956	5.379	7.696	9.925	10.114	7.0992	10.1287	8.7971	1.375879149	high
TCGA-55-6986-01	8.934246575	0	8.4743	9.2055	8.6723	8.5035	10.8182	10.2988	8.6953	5.6559	7.4606	10.7197	10.2167	6.4747	9.929	9.2544	1.822475769	high
TCGA-75-7025-01	9.054794521	0	7.7075	8.4008	9.7607	9.7062	11.0015	10.3514	9.366	6.3228	9.0074	10.093	9.7308	6.7401	9.5864	7.5906	0.64740512	low
TCGA-78-8662-01	9.208219178	1	9.7516	9.3609	8.3426	9.5751	12.0666	10.3035	9.1058	7.1892	9.2476	10.8688	9.6072	7.0376	9.9548	8.1477	0.733744207	low
TCGA-78-7153-01	9.95890411	0	8.936	9.4784	9.2931	6.9663	10.9771	10.5845	9.4071	6.0464	8.4878	10.7397	9.8858	6.7196	10.626	8.3882	1.586934339	high
TCGA-38-4626-01	10.06575342	0	8.4412	8.7126	8.6716	9.3604	11.2018	10.6058	8.534	6.4235	7.3203	10.516	9.3273	7.31	10.0557	8.3249	0.795415761	low
TCGA-49-AARO-01	10.29863014	0	8.7557	8.4528	9.5572	9.3357	10.5651	9.5921	8.5799	4.9262	8.9124	10.7913	10.3557	6.8474	9.6971	8.0626	1.456841957	high
TCGA-78-7149-01	10.79452055	0	9.2443	8.7183	8.8824	9.515	11.6936	10.0858	8.4169	5.9969	9.1703	9.6666	10.9241	6.6316	10.096	7.7735	1.01843221	low
TCGA-49-AAR0-01	13.05479452	0	10.4193	9.4706	9.5153	9.312	10.8784	9.8112	8.7903	6.086	8.9376	10.7127	9.9376	6.9304	9.8999	8.2824	1.15350818	low
TCGA-78-7143-01	13.59178082	1	8.9252	8.2829	9.1827	8.7705	10.8068	11.1686	10.5691	5.8958	8.3941	10.8588	9.133	7.2897	10.0885	7.8418	0.973989985	low
TCGA-49-AARR-01	13.67671233	0	8.106	8.7193	9.5381	9.8286	10.3095	9.5401	8.7623	4.9353	9.9324	9.7478	9.7632	7.8106	10.5425	8.4021	0.663132049	low
TCGA-49-AARQ-01	18.44383562	0	10.095	10.0905	9.9001	10.3056	12.8036	10.5278	9.9394	5.2077	8.817	10.5338	8.8768	6.0724	10.1395	7.6947	0.265302333	low
TCGA-78-8640-01	19.34794521	0	9.8968	9.78	8.1791	8.8912	11.7414	11.3855	8.463	7.3403	8.9071	9.6676	8.8894	4.7055	11.108	8.7997	0.725622862	low
TCGA-78-7163-01	19.85753425	0	8.2217	9.4208	8.8577	9.4884	11.6781	10.7277	9.5634	5.6913	8.1365	10.7935	11.3416	7.1116	10.2468	6.6081	0.652649113	low
