# Supplementary material for: Insight of a Metabolic Prognostic Model to Identify Tumor Environment and Drug Vulnerability for Lung Adenocarcinoma
Source: Front Immunol. 2022 Jun 23;13:872910. doi: 10.3389/fimmu.2022.872910 (PMC9262104; doi:10.3389/fimmu.2022.872910)
Supplement: Supplementary file 10 [file DataSheet_9.pdf]

Supplementary Table S9: The estimated distribution of immune cell subsets using computational approach (CIBERSORT) in LUAD.

Mixture	B cells naive	B cells memory	Plasma cells	T cells CD8	T cells CD4 memory resting	T cells CD4 memory activated	T cells follicular helper	T cells regulatory (Tregs)	T cells gamma delta	NK cells resting	NK cells activated	Monocytes	Macrophages M0	Macrophages M1	Macrophages M2	Dendritic cells resting	Dendritic cells activated	Mast cells resting	Mast cells activated	Eosinophils
TCGA-05-4249-01	0	0.102147886	0.066949443	0.029334641	0.111139195	0	0.092493971	0.027889998	0	0	0.022697154	0.046635542	0	0.035342958	0.127522337	0.162465759	0.045597735	0.104058922	0	0
TCGA-05-4250-01	0.019003045	0	0.10995728	0.114285105	0.129276071	0.02910559	0.110677261	0.014200953	0	0	0.040285462	0.045566784	0.053517913	0.049927771	0.179499202	0	0.047917325	0.037593398	0	0
TCGA-05-4382-01	0.064761689	0	0.180152786	0.030750115	0.179757484	0.02675914	0.065919022	0	0	0.009459055	0.005542665	0.004419542	0.160867295	0.050010601	0.182344193	0	0	0.002792484	0	0
TCGA-05-4384-01	0.045942754	0	0.086064365	0.035425565	0.12435722	0	0.074407955	0.003593377	0	0.063545947	0.011652035	0.047258566	0.102964015	0.031496472	0.222524386	0.05998665	0.022933099	0.032964577	0	0
TCGA-05-4389-01	0.075939188	0	0.024270165	0.125331162	0.101401556	0.029194692	0.094299595	0.023943591	0	0	0.045990388	0.00733983	0.018953179	0.043294053	0.158078405	0.098391769	0.045777099	0.107795328	0	0
TCGA-05-4390-01	0.064435554	0	0.155690333	0.027115493	0.159569194	0.059587975	0.059809126	0.002265013	0	0.012108756	0	0.012934224	0.165966682	0.034050992	0.161600456	0	0.033390031	0.001987788	0.004670386	0
TCGA-05-4396-01	0.12813769	0	0.096337712	0.036899742	0.225611442	0	0.08105945	0.019743064	0	0.039038797	0.012566956	0.040986128	0.017745578	0.051259883	0.162010769	0.014893158	0.040113132	0.006909084	0	0
TCGA-05-4397-01	0.011298135	0	0.081862335	0.018093018	0.102190508	0	0.100183486	0.037073173	0	0.075032397	0.002823214	0.059203847	0.151120259	0.081979344	0.201360859	0	0.063079226	0	0	0
TCGA-05-4398-01	0.069705186	0.031738135	0.096713324	0.12993735	0.078980279	0.019531128	0.151626942	0	0	0.004552829	0.018369691	0.092183061	0.028759296	0.035397299	0.089934331	0.050819802	0.06203982	0	0.014378698	0.000436394
TCGA-05-4402-01	0.032213961	0.033871863	0.103417707	0.028629363	0.160226239	0	0.075762841	0.022454986	0	0	0.040156578	0.019142944	0.091268553	0.037199694	0.154445946	0.017889517	0.065467817	0.070175096	0	0
TCGA-05-4403-01	0	0.023591615	0.104069844	0.051943388	0.143132881	0	0.106797635	0.031352304	0	0.028921123	0.004101263	0.077422007	0.036393943	0.053620779	0.152152575	0.076552548	0.034640609	0.048928426	0	0
TCGA-05-4405-01	0.010649955	0.064756162	0.121915057	0.079268536	0.15618835	0	0.074648788	0.016220207	0	0.001705701	0.033263837	0.062627568	0.045336412	0.023253709	0.126599674	0.061411235	0.033831356	0.088323455	0	0
TCGA-05-4415-01	0.003331266	0	0.145955983	0.094058405	0.126475277	0.034375046	0.081256712	0.017140749	0	0.011748942	0.046715421	0.017465037	0.073275445	0.032717206	0.150119103	0	0.06823222	0.042540841	0	0
TCGA-05-4417-01	0.077527376	0	0.120357571	0.108493908	0.113013337	0.023388711	0.120003555	0	0	0	0.022101076	0.018806392	0.051342239	0.035971691	0.136060991	0.064829775	0.056748379	0.027324032	0	0
TCGA-05-4418-01	0.072887317	0	0.192541048	0.087684749	0.064762198	0.016488721	0.125279952	0.028327434	0	0.022741642	0.014205333	0.043094171	0.060821431	0.03888668	0.157333036	0	0.057250904	0.012767082	0	0
TCGA-05-4420-01	0.083651138	0	0.134661114	0.037821195	0.209919922	0	0.08003908	0.011741898	0	0.04436429	0	0.001576249	0.074331188	0.009311917	0.142061485	0.004329004	0.045304195	0	0.024045687	0
TCGA-05-4422-01	0.007282896	0.05058663	0.180949582	0.164770065	0.146090011	0	0.103145995	0.016746496	0	0	0.041618817	0.026826805	0.040511478	0.047668299	0.143453247	0.004697166	0.015144859	0	0	0
TCGA-05-4424-01	0.096700437	0	0.029770104	0.094288769	0.148316727	0.000274495	0.079835613	0.015583832	0	0.012765721	0.004651838	0.030077154	0.137234529	0.044000539	0.187405922	0	0.052549037	0.038087799	0	0.003027201
TCGA-05-4425-01	0	0.085220802	0.031708024	0	0.170822617	0.010981668	0.087920371	0	0	0.045149328	0.023472547	0.067520678	0.035430598	0.055991518	0.177732727	0.105456236	0.040524168	0.059416526	0	0
TCGA-05-4426-01	0	0.059814868	0.024437993	0	0.261043302	0	0.119201515	0.014705164	0	0	0.049630946	0.098713233	0.019404809	0.031679988	0.182453096	0	0.043747673	0	0.026691892	0
TCGA-05-4427-01	0.018358148	0	0.09037076	0.095624325	0.131967173	0.019733059	0.098234754	0	0	0.000588612	0.021309703	0.058744915	0.101521622	0.047125218	0.192100533	0.018386851	0.060741068	0.033263027	0	0
TCGA-05-4430-01	0.027650026	0	0.052339484	0.077743647	0.149839523	0.024080974	0.070985018	0.016570684	0	0	0.025806453	0.045929148	0.036357612	0.043776306	0.137756103	0.104783489	0.056725172	0.100421579	0	0
TCGA-05-4432-01	0.047720791	0	0.145248571	0.093980387	0.132270041	0.016173567	0.074469642	0.029802287	0	0.022868156	0.022125484	0.009615946	0.123826389	0.042115392	0.143309491	0	0.050121423	0	0	0
TCGA-05-4433-01	0.042408404	0	0	0.107503742	0.219263136	0	0.067335389	0.008269248	0	0.022499967	0.010851672	0.036107466	0.147625007	0.046154124	0.15509586	0.022824366	0.048557796	0.035737199	0	0
TCGA-05-4434-01	0.04562154	0.003817211	0.042656987	0.064481079	0.176573535	0.035681676	0.07547347	0.022497036	0	0.038088409	0	0.037603164	0.060612829	0.072189115	0.182723556	0.024646945	0.038096723	0.050158993	0	0
TCGA-05-5420-01	0.037199329	0	0.172163621	0.09026148	0.09411006	0.046085162	0.07376494	0	0	0.003020884	0.031923828	0.030829468	0.087185019	0.065038954	0.157969373	0.009294022	0.013394547	0.049391895	0	0.010543287
TCGA-05-5423-01	0.036406594	0.027273739	0.056268168	0.024020013	0.166954748	0.008842014	0.08055318	0.007725508	0	0.000242324	0.004227128	0.047063959	0.049255883	0.021484261	0.170909877	0.101916688	0.079168421	0.064083224	0	0.029474467
TCGA-05-5425-01	0.061160752	0	0.110824024	0.140105517	0.074414379	0.03287557	0.115281935	0.007681009	0	0	0.018914485	0.018504893	0.089744368	0.048011093	0.155416801	0	0.056679599	0.033786848	0	0.002374789
TCGA-05-5428-01	0.044576953	0	0	0.088148679	0.227421139	0	0.0754033	0	0	0.013376303	0.006823042	0.027194177	0.023086684	0.040718796	0.193843617	0.102365431	0.012703626	0.085942237	0	0
TCGA-05-5429-01	0.065119478	0	0.170551924	0.024547546	0.292093742	0	0.047727817	0	0	0	0.015947359	0.018459045	0.003083389	0.038721765	0.132662176	0.091381101	0.019695001	0.025912925	0.021378816	0
TCGA-05-5715-01	0.066768611	0	0.066266478	0.032999426	0.165720614	0	0.078167385	0	0	0	0.030244203	0.031073652	0.147292106	0.026135964	0.180956957	0.012096854	0.046178845	0.094674726	0	0
TCGA-35-3615-01	0.082235175	0	0.093299926	0.075976791	0.111836644	0.003237428	0.105534678	0	0	0	0.029466888	0.018611956	0.081420507	0.040349927	0.171630489	0.043450875	0.055530447	0.071836746	0	0
TCGA-35-4122-01	0.039925866	0	0.141044811	0.0678137	0.15726411	0.012391974	0.114048729	0	0	0.010262433	0.021725309	0.051713678	0.048096931	0.043525628	0.14973216	0.04863518	0.055495469	0.020239006	0	0
TCGA-35-4123-01	0.018979602	0	0.097351585	0.153909624	0.141717458	0.009941093	0.143104231	0.010802215	0	0	0.027640833	0.023405617	0.010526396	0.037579912	0.192354896	0.013037671	0.070878579	0.037491188	0	0
TCGA-35-5375-01	0.012862677	0.040716469	0.151369967	0.110204165	0.110465918	0	0.153243846	0.004033348	0	0.005695675	0.010469747	0.013341844	0.014985379	0.051205584	0.135916142	0.063933996	0.057510913	0.032299466	0.003902086	0
TCGA-38-4625-01	0.040835786	0.044125069	0.088603013	0.075024862	0.101291479	0	0.130834883	0	0	0.019864021	0.003551323	0.072378116	0.025385523	0.068011062	0.171822386	0	0.03987414	0.09577268	0	0.006038961
TCGA-38-4626-01	0.076696321	0	0.041366795	0.043388728	0.178905248	0.005330366	0.001002734	0.008743067	0	0.027902588	0.007894863	0.048503387	0.138728835	0.017803683	0.232046673	0	0.051489704	0.067969188	0	0
TCGA-38-4627-01	0.005810022	0	0.135576041	0.053229303	0.221079144	0	0.067791626	0.002161228	0	0	0.028125173	0.077346333	0.018342481	0.050005443	0.19840413	0.010486093	0.030134615	0.086005362	0	0
TCGA-38-4628-01	0.057668642	0	0.015214363	0.042359402	0.126899961	0	0.077510826	0.026129471	0	0	0.054368532	0.053893284	0.156700518	0.009803385	0.18126592	0.041192654	0.048135565	0.085662493	0	0
TCGA-38-4629-01	0.035385553	0	0.123863163	0.136710727	0.071241441	0.035948139	0.098802901	0.001826678	0	0	0.028593751	0.058229631	0.099087408	0.046086951	0.19023301	0	0.04591451	0.017041353	0	0
TCGA-38-4630-01	0	0	0.044420541	0.16279604	0.11380026	0	0.148992412	0	0	0.019789802	0.015893735	0.073247302	0.044010121	0.047415745	0.177994718	0.106165391	0.045473933	0	0	0
TCGA-38-4631-01	0.08695673	0	0.207966602	0.069067178	0.126248043	0	0.089454098	0.011573712	0	0.004060008	0.04510121	0.051550362	0.046652315	0.043419828	0.137178145	0	0.032277067	0	0	0
TCGA-38-4632-01	0.082626616	0	0.094315532	0.111285092	0.074387177	0.031455449	0.11629475	0.011075947	0	0	0.007930082	0.039078441	0.016256838	0.027976378	0.171594275	0.075874967	0.064002433	0.036685814	0	0.011452895
TCGA-38-6178-01	0.082251034	0	0.125128643	0	0.200402554	0	0.047443682	0.030827256	0	0	0.028039766	0.006334906	0.056511931	0.040299005	0.156273591	0	0.073971802	0.126877017	0	0
TCGA-38-7271-01	0.031424625	0.132349598	0.104860132	0.101546236	0.088162551	0.013570903	0.102130388	0.027062195	0.001337866	0	0.024445397	0.004608669	0.026660241	0.036483673	0.145713543	0.025229102	0.052865108	0.076823301	0	0
TCGA-38-A44F-01	0.017854455	0.092954102	0.091398534	0.058849877	0.115739732	0	0.120934988	0.017889014	0	0.029633326	0	0.037886903	0.062466602	0.059366323	0.135717581	0.069664113	0.031738959	0.050127465	0.007778024	0
TCGA-44-2655-01	0.075960205	0.049398817	0.108781135	0	0.196453819	0	0.144282463	0	0	0.000818737	0.011472533	0.027697911	0.011233254	0.040473944	0.145621357	0.085233137	0.050933431	0.040292917	0	0
TCGA-44-2656-01	0.021571152	0.012305136	0.133164308	0.163549789	0.069128244	0.021833117	0.066474847	0.015264202	0	0.015352606	0.022657122	0.037651229	0.055235588	0.060538921	0.140823184	0.043356674	0.035138227	0.07607456	0	0
TCGA-44-2657-01	0.159991939	0	0.107850011	0.074856937	0.09545757	0	0.127444997	0	0	0	0.042946001	0.034017143	0.039867547	0.03869117	0.094759495	0.034530667	0.049796463	0.099790061	0	0
TCGA-44-2659-01	0	0.081225593	0.090027981	0.043631291	0.189937521	0.000356441	0.126361413	0	0	0	0.031491472	0.059360302	0.016624352	0.036692078	0.160584244	0.010518533	0.065194365	0.087994415	0	0
TCGA-44-2661-01	0.032065078	0.003878668	0.090437519	0.074522884	0.116210354	0	0.112249973	0.010180237	0	0.009916279	0.016867703	0.018435651	0	0.032734148	0.202310713	0.093882626	0.064323878	0.096387873	0	0.011305045
TCGA-44-2662-01	0.002508405	0	0.099775517	0	0.202558266	0.018100959	0.042419635	0.006988784	0	0.031758844	0	0.083155162	0.19915639	0.044247082	0.172933883	0	0.019465662	0.02893014	0	0

TCGA-44-2665-01	0	0.025430123	0.021751599	0	0.283145432	0	0.052445014	0.020105729	0	0.024783162	0.006274578	0.044964579	0.033401093	0.010784752	0.189851336	0.108815214	0.102176439	0.05439289	0	0
TCGA-44-2666-01	0	0.038868565	0.181982055	0.072556436	0.151872744	0	0.117009229	0.008467172	0	0.053124113	0.001105896	0	0.008664453	0.010726872	0.057415909	0.030729442	0.060854433	0.097189437	0	0.078090554
TCGA-44-2668-01	0.033228469	0.028955443	0.086144808	0.034427605	0.186845627	0.015440184	0.070033271	0.009542865	0	0.043051626	0	0.087680095	0.033643838	0.054692255	0.10571847	0.040202878	0.068372337	0	0.04148294	0
TCGA-44-3396-01	0.057144193	0	0.149209507	0.072804511	0.141777014	0.02961375	0.095642047	0	0	0.025383079	0	0.020656493	0.107868216	0.044975457	0.142197598	0	0.03814869	0.011654521	0.011651578	0
TCGA-44-3398-01	0	0.048017404	0.073676914	0.047654974	0.159997646	0.00427451	0.071492773	0	0	0.019243777	0.020420165	0.03955069	0.015485776	0.022958592	0.139645389	0.132869828	0.063020529	0.095389575	0	0.019224519
TCGA-44-3918-01	0.02331689	0	0.176094781	0.057717853	0.158321192	0.001309761	0.112465116	0.015267013	0	0.022905025	0.021056328	0.031869439	0.007809448	0.048064808	0.170511281	0.078688464	0.038376069	0.026936982	0	0
TCGA-44-3919-01	0.035687892	0.006618952	0.131302179	0.068681792	0.130548876	0	0.110107532	0.001643426	0	0	0.046501606	0.033807318	0.011178389	0.069313451	0.135314804	0.094375421	0.037695268	0.079698124	0	0
TCGA-44-4112-01	0.005425981	0	0.18436526	0.048176196	0.181018203	0	0.097210405	0	0	0	0.021817713	0.023944302	0	0.042522931	0.164251312	0.066013022	0.034128716	0.097457802	0	0
TCGA-44-5643-01	0.108158263	0	0.105822271	0.064466265	0.047179694	0.034541294	0.156344943	0.027582561	0	0.029552815	0	0.040054035	0.043872671	0.049579886	0.116026597	0.083756883	0.052451546	0.040610278	0	0
TCGA-44-5644-01	0.157625645	0	0.173147971	0.081127132	0.014911947	0.009464107	0.12644842	0.005419357	0	0	0.040223871	0	0.140866134	0.007188343	0.100002822	0	0.079079599	0.016545911	0	0
TCGA-44-5645-01	0.043295613	0.026413777	0.064778001	0.007485798	0.196103459	0	0.132103222	0.028324697	0	0	0.007907878	0.020223851	0.017502557	0.033370024	0.16352152	0.081733756	0.079062793	0.087659535	0	0
TCGA-44-6145-01	0.019226963	0.048735951	0.12116279	0.146636486	0.084094612	0.030842177	0.091545687	0.014629081	0	0	0.034146677	0	0.082971647	0.04496364	0.126815277	0	0.043728646	0.06796771	0	0
TCGA-44-6146-01	0.050218325	0	0.093541815	0.075210713	0.337643155	0	0.032757526	0	0	0.065284786	0	0.050825654	0.054833223	0.037620195	0.124515986	0	0.028431315	0	0.037972578	0
TCGA-44-6147-01	0.078279741	0.011302342	0.136137082	0.000739016	0.164076661	0	0.118375694	0.024464557	0	0.048782919	0	0.050313436	0.031966785	0.028545528	0.155684025	0	0.069013859	0.046663681	0	0
TCGA-44-6148-01	0.015387054	0.063859985	0.155547667	0.017317098	0.161180646	0	0.11747365	0	0	0	0.024384037	0.061070951	0.039549806	0.015832103	0.165080417	0	0.033743951	0.124072588	0	0
TCGA-44-6774-01	0.077113094	0	0.107217142	0.053138361	0.188515464	0	0.099216578	0.020935281	0	0.006537078	0.025767785	0.065454386	0.089349541	0.031208902	0.130552329	0.021418286	0.037338392	0.015690464	0	0
TCGA-44-6775-01	0.029612092	0	0.078693027	0.011209944	0.184186139	0	0.061618013	0.04621914	0	0.01632276	0.009335222	0.067620456	0.078566332	0.035334621	0.158252999	0.080978947	0.063276085	0.058736259	0	0
TCGA-44-6776-01	0.124852057	0	0.147720604	0.07564027	0.097499691	0	0.095264745	0	0	0	0.061526429	0.033691348	0.039703761	0.023208177	0.112110651	0.04489333	0.041440481	0.099970011	0	0
TCGA-44-6777-01	0.040723919	0.006845733	0.114762683	0.030571556	0.18702321	0	0.050998042	0.026470922	0	0.001323529	0.015715819	0.058454764	0.039415825	0.034157987	0.170504058	0.075593555	0.05209918	0.070345138	0	0
TCGA-44-6778-01	0.048149771	0.016347317	0.111785072	0.105978206	0.118971591	0.023740926	0.127961889	0	0	0	0.031890143	0.015806994	0.085169076	0.052277542	0.171111169	0.000506258	0.043482812	0.027907585	0	0
TCGA-44-6779-01	0	0.013458864	0.028750304	0.151125742	0.109051714	0	0.108333968	0.044414969	0	0	0.057243232	0.021092841	0.085579566	0.074051615	0.150747055	0	0.017571837	0.108492979	0	0
TCGA-44-7659-01	0.03844029	0.065737135	0.11795658	0.05259902	0.173805485	0	0.083172577	0.005952993	0	0.014212046	0.021281859	0.033233808	0.045836431	0.040881831	0.130139021	0.042741218	0.052675156	0.078340259	0	0
TCGA-44-7660-01	0.112056283	0	0.0741485	0.124162898	0.02519614	0.009903525	0.131259906	0.031793644	0	0.004799914	0.032040897	0	0.128279884	0.052953445	0.187724976	0	0.041716328	0.022668978	0	0
TCGA-44-7661-01	0.033112507	0.005617632	0	0.025242943	0.193401404	0.076198021	0.103336372	0.016778875	0	0.018233301	0	0.083207952	0.046899593	0.046190632	0.202600772	0.026361161	0.084643427	0.038175408	0	0
TCGA-44-7662-01	0.079953019	0	0.1377267	0.078901266	0.128020884	0.010772699	0.063081066	0.019624769	0	0.032904642	0.011043627	0.034581287	0.118112673	0.045247251	0.141176117	0	0.033224891	0.042368901	0	0
TCGA-44-7667-01	0	0.059490417	0.139014276	0.104794677	0.095499454	0.014573711	0.090858748	0	0	0	0.076451237	0.076422075	0.102391486	0.037110646	0.147277457	0	0.048034367	0	0	0
TCGA-44-7669-01	0.050589549	0.002244428	0.075382339	0.065523911	0.125216519	0.012090742	0.080457947	0	0	0.012290278	0.027941825	0.01903238	0.131657821	0.049965883	0.210902303	0	0.039770263	0.074808751	0	0
TCGA-44-7670-01	0.07464146	0	0.115396696	0.077976268	0.076424279	0.007736442	0.152023753	0	0	0	0.053019953	0.028161553	0.190000215	0.054979815	0.133146413	0	0.033026669	0	0	0
TCGA-44-7671-01	0.043525742	0	0.140508435	0.012147099	0.150608187	0	0.121217433	0	0	0.014469965	0.017149755	0.007763298	0.070969655	0.026725096	0.207117782	6.10E-05	0.085999491	0.073026481	0	0
TCGA-44-7672-01	0.036596086	0.034237976	0.136880896	0.078442761	0.117453771	0.02698982	0.082521277	0.024907658	0	0.013682056	0.027850716	0.048856879	0.055196939	0.041157408	0.137010174	0.034625043	0.052716909	0.0455973	0	0
TCGA-44-8117-01	0.077116558	0	0.051609427	0.085695063	0.160882909	0.010143468	0.111156662	0.005110708	0	0.01500742	0.011327963	0.026840729	0.136912633	0.053329754	0.159280138	0	0.028986821	0.056316294	0	0
TCGA-44-8119-01	0.075089577	0	0.149599959	0.06348088	0.158743143	0.022032872	0.078936498	0.003115635	0	0.051380193	0.004316694	0.033801333	0.130463767	0.046690454	0.130571549	0	0.023575055	0.003156507	0	0
TCGA-44-8120-01	0.054479002	0.01313271	0.061697825	0.048293296	0.172123163	0.004600724	0.107422062	0	0	0	0.028065887	0.038292594	0.043817737	0.023148769	0.172021802	0.065224977	0.037756456	0.102681616	0	0
TCGA-44-A479-01	0.073882792	0	0.130538028	0.039361159	0.21488188	0	0.147886275	0	0	0	0.042759763	0.055141523	0.035869106	0.065703269	0.1300032	0.017972578	0.044948369	0.001052059	0	0
TCGA-44-A47A-01	0	0.053766897	0	0.049705671	0.109040598	0	0.132363692	0.058362365	0	0	0.019439271	0.052872824	0.013139334	0.03585312	0.158634414	0.134611073	0.082977701	0.09545164	0	0
TCGA-44-A47B-01	0	0.0116242	0.055517178	0.158112179	0.040754858	0.011577372	0.106846079	0.069298385	0	0	0.049736256	0.026912204	0.059063992	0.056886524	0.154159303	0.076928923	0.027647386	0.09493516	0	0
TCGA-44-A47G-01	0.000375083	0.086222833	0.120696207	0.122883544	0.06398021	0.028375186	0.087311991	0.023272421	0	0	0.027491065	0.044023748	0.04953052	0.056179831	0.124014857	0.060374982	0.034291185	0.070976338	0	0
TCGA-44-A4SS-01	0.077188863	0	0.03264166	0.123628333	0.117929012	0.018217487	0.142375205	0	0	0.005685565	0.019409525	0.048844176	0.062375607	0.074194337	0.174839426	0.002868354	0.044196363	0.046216521	0	0
TCGA-44-A4SU-01	0.040523326	0.09261325	0.169094641	0.022982103	0.117222254	0	0.162391257	0.026231379	0	0	0	0.02498751	0.050376431	0.048724369	0.079717298	0.065028751	0.048078832	0.052028597	0	0
TCGA-49-4486-01	0.062653701	0	0.206918139	0.039267368	0.183745082	0	0.149829173	0	0	0.005642103	0.025093965	0.045277738	0	0.030113671	0.149962098	0.032976634	0.068520329	0	0	0
TCGA-49-4487-01	0.050338098	0.095713115	0.106264404	0.150701557	0.06142789	0.057166464	0.115049051	0.026339472	0	0	0.007489372	0.016850739	0.066445428	0.06041043	0.136837941	0.000534213	0.044164969	0.004266858	0	0
TCGA-49-4488-01	0.032922253	0.016297154	0.106505409	0.095997547	0.151465659	0	0.096012793	0.036423119	0	0.004610625	0.020222109	0.062505413	0.025521492	0.050129616	0.119622606	0.10612112	0.05479392	0.020849165	0	0
TCGA-49-4490-01	0.126589736	0	0.070794114	0.055631966	0.095615726	0	0.107074405	0.077226306	0	0	0.030649071	0.079316121	0.090442975	0.03760251	0.11444004	0	0.04776222	0.066854809	0	0
TCGA-49-4494-01	0.062704967	0	0.109355402	0.04122537	0.143325251	0.009810803	0.126423503	0.059248708	0	0.001201677	0.002989536	0.068279751	0.015192348	0.051773463	0.172302675	0.041423651	0.070850903	0.01952547	0	0
TCGA-49-4501-01	0.072168062	0.01014302	0.124506052	0.052135274	0.169500434	0	0.099008076	0	0	0	0.044877607	0.039407338	0.024264997	0.036838537	0.125446791	0.047155418	0.058507754	0.095240913	0	0
TCGA-49-4505-01	0.029062263	0.007722011	0.115245063	0.086555225	0.107536413	0.000447957	0.092838343	0.024345965	0	0.018238951	0.014534626	0.060051647	0	0.041878261	0.127184393	0.083708656	0.070054979	0.093345434	0	0
TCGA-49-4506-01	0.051116715	0.00213887	0.128463624	0.185914567	0.048772495	0.035895828	0.111774204	0.009437219	0	0.010761129	0.018545836	0.039827322	0.053315444	0.053646774	0.13686324	0.034369222	0.045182493	0	0	0
TCGA-49-4507-01	0.044901944	0	0.128041064	0.185844392	0.021641655	0.004897696	0.104289123	0.054843265	0	0.039304787	0.053968229	0	0.108883801	0.051539483	0.134391282	0	0.01351939	0.047044352	0	0.006889536
TCGA-49-4510-01	0.027695106	0	0.202137899	0.090643731	0.074996838	0	0.145294757	0	0	0.044466307	0.027447662	0.041384797	0	0.021603199	0.152048126	0.066664534	0.047728958	0.042459994	0	0
TCGA-49-4512-01	0	0.050146932	0.080133564	0.0760779	0.063777949	0	0.091858779	0.079028934	0	0	0.021023927	0.092145285	0.063564575	0.03629693	0.146408487	0.048967075	0.063789054	0.086780606	0	0
TCGA-49-4514-01	0.099230091	0	0.130476563	0.140131496	0.006310995	0.040934067	0.132169435	0.019818623	0	0.006469387	0.033925279	0.020059848	0.098100555	0.042255041	0.144282021	0	0.058488188	0.027348412	0	0
TCGA-49-6742-01	0.126537546	0	0.112353928	0.042472227	0.060835919	0	0.048619593	0.018919022	0	0	0.082826751	0.019545744	0.151682148	0.00358414	0.189174523	0	0.063180219	0.065671231	0	0
TCGA-49-6743-01	0.059713061	0	0.13439163	0.06936734	0.142240847	0.010789143	0.085390786	0	0	0.051155792	0	0.016033527	0.159279139	0.040901227	0.161154027	0	0.044466331	0	0	0
TCGA-49-6744-01	0.027521884	0.006762403	0.133275031	0.074436886	0.137921786	0.00988246	0.097672885	0.011581991	0	0.003171911	0.012472919	0.06801128	0.050932508	0.043722426	0.105825662	0.052034357	0.061046905	0.087800319	0	0

TCGA-49-6745-01	0	0.050043458	0.021446369	0.141493747	0.131941441	0.021055591	0.088397662	0.01850797	0	0.01252568	0.009006221	0.06710342	0.049416035	0.047850099	0.156835639	0.049718566	0.059808323	0.066831328	0	0
TCGA-49-6761-01	0.068372053	0	0.075950126	0.081799374	0.170401585	0.02647578	0.140763453	0	0	0.0079405	0.045304212	0.048647536	0.033578714	0.050471993	0.130003333	0.036615566	0.062818593	0.006465889	0	0
TCGA-49-6767-01	0.026484856	0	0.044737789	0.07982122	0.148152255	0	0.149347981	0.017559851	0	0	0.112426595	0.067504596	0.06101638	0.055153115	0.121548428	0.055342398	0.060904534	0	0	0
TCGA-49-AAQV-01	0.047281126	0.07989862	0.085676139	0.066029274	0.081597399	0	0.146817769	0.05483445	0	0.007368005	0.009752551	0.036026714	0.091420405	0.059848893	0.104169642	0.04502592	0.047332988	0.036920106	0	0
TCGA-49-AAR0-01	0.101199536	0	0.096569163	0.124256522	0	0	0.143486628	0.045955638	0	0.009055969	0.012981499	0.026064398	0.144578306	0.048725674	0.179960572	0	0.025662096	0.041503999	0	0
TCGA-49-AAR2-01	0.066455973	0	0.242872701	0.132848395	0.002150837	0.023906707	0.125603248	0	0	0.01178749	0.026448978	0.015842568	0.090422593	0.042094407	0.148820224	0	0.032334294	0.023414069	0	0
TCGA-49-AAR3-01	0.070074437	0	0.082392022	0.16139336	0.017799101	0.033649711	0.12649079	0.05170999	0	0.003099563	0.031014931	0	0.118678283	0.073170795	0.185499298	0	0.030484578	0.004506665	0	0
TCGA-49-AAR4-01	0.065525616	0	0.153047176	0.212309609	0	0.029669469	0.138100348	0.028431873	0	0.009799747	0.011418379	0.037759032	0.07419776	0.039077	0.104662645	0	0.046698144	0.049303203	0	0
TCGA-49-AAR9-01	0	0.102127227	0.120350355	0.032959675	0	0	0.219951588	0.091921156	0	0	0.054315491	0	0.094899133	0.040236946	0.106184787	0	0.117586395	0	0.019467247	0
TCGA-49-AARE-01	0.096285018	0	0.137133184	0.080767196	0.028449355	0.041162343	0.156797117	0.035433398	0	0.032127006	0	0.031319717	0.12176533	0.06480627	0.128825725	0	0.043276971	0.001851369	0	0
TCGA-49-AARN-01	0	0.1492999	0.121498665	0.061601639	0.132905524	0	0.148496703	0.015265417	0	0.014268001	0.013118641	0	0.004866244	0.047041123	0.123549259	0.068093328	0.052053977	0.03665571	0	0
TCGA-49-AARO-01	0.048420181	0.052153967	0.060216307	0.059069215	0.133192388	0	0.124513431	0.020192613	0	0.073362008	0	0.049639138	0.020898483	0.037288568	0.14570619	0.081696348	0.068264151	0.012096375	0.001843767	0
TCGA-49-AARQ-01	0.027825241	0	0.07005589	0	0.095919434	0	0.227421214	0	0	0	0.126391025	0.06195346	0.047647032	0.08523938	0.155693323	0.00203788	0.033418202	0.06639792	0	0
TCGA-49-AARR-01	0.106645145	0	0.091858139	0.139845844	0.026174333	0	0.113975117	0.015510338	0	0.023480434	0.019561739	0.018833218	0.10438641	0.028575032	0.195175273	0	0.029905415	0.085236719	0	0
TCGA-4B-A93V-01	0.054982772	0	0.205242878	0.116242359	0.058373376	0.005395646	0.114367605	0.01296248	0	0.002248058	0.051123068	0.042869597	0.166571155	0.036938799	0.110730236	0	0.02195197	0	0	0
TCGA-50-5044-01	0	0.014149245	0.146553642	0.090137262	0.003410456	0.001419665	0.154966113	0.057632806	0	0.00149769	0.059840854	0.042617845	0.019405897	0.045912247	0.165150743	0.042265921	0.065781257	0.08925836	0	0
TCGA-50-5045-01	0.099746694	0	0.126883306	0.127605276	0.041034102	0.002226989	0.131319346	0.017254339	0	0	0.014198638	0.050373614	0.059385692	0.033584339	0.147142681	0.067241393	0.050204761	0.026826355	0	0
TCGA-50-5049-01	0.007865209	0.049446449	0.11991953	0.085875067	0.075486219	0	0.130171905	0.029932104	0	0.02578827	0.024723699	0.086737839	0.031907605	0.071536087	0.169548747	0.048706403	0.042354868	0	0	0
TCGA-50-5051-01	0.096798003	0.000299831	0.182802537	0.102847331	0.056274709	0	0.149183988	0.008385816	0	0.034165076	0.008051065	0.057808266	0.04643141	0.004265812	0.078879045	0.036014806	0.081983768	0.036340103	0	0
TCGA-50-5055-01	0.079529499	0.075131231	0.107253045	0.076520225	0.100287155	0	0.154420646	0.004754861	0	0	0	0.053550504	0.050599787	0.048289571	0.108215658	0.050451096	0.043310655	0.047686067	0	0
TCGA-50-5066-01	0.027595178	0.005196457	0.207110316	0.129995112	0.076140676	0.03644677	0.071776945	0.00338628	0	0	0.021667378	0.043779467	0.082973579	0.040373189	0.1658593	0	0.032965229	0.046148389	0	0
TCGA-50-5068-01	0.049861044	0.03442893	0.130701954	0.04790919	0.117535859	0	0.134510472	0.016794883	0	0.044098692	0	0.04712916	0.005021009	0.066789921	0.135716445	0.117358481	0.028640167	0	0.023503791	0
TCGA-50-5072-01	0.110548128	0	0.124854908	0	0.143573419	0	0.086523157	0.01077584	0	0.011844553	0.022353695	0.032557472	0.150793298	0.031501356	0.149254666	0	0.052338297	0.059187604	0	0
TCGA-50-5930-01	0.036675156	0.036354618	0.197263511	0.110625641	0.062165747	0.006321271	0.121602877	0	0	0.036287471	0.0133185	0.053240424	0.132348313	0.030272934	0.105694046	0	0.041388461	0.015896331	0	0
TCGA-50-5931-01	0.05488741	0	0.148846968	0.147105708	0	0	0.228829788	0	0	0.008212675	0.046174337	0.019780181	0.084854812	0.035239904	0.110492435	0	0.064197829	0.030795673	0	0.020582279
TCGA-50-5932-01	0.046971324	0	0.103525486	0.03119133	0.208562114	0	0.059879174	0	0	0.000988945	0.04369793	0.050017811	0.141080801	0.024867178	0.178543316	0	0.03380779	0.073231504	0	0
TCGA-50-5933-01	0	0.043677608	0.043926234	0.05986444	0.222045424	0	0.06367559	0	0	0	0.04516205	0.053599217	0.100243398	0.060140609	0.176554122	0.00241272	0.041729311	0.058263013	0	0
TCGA-50-5935-01	0	0.06618428	0.116551982	0.037811206	0.140373064	0	0.06784259	0.063516357	0	0.014643519	0	0.080493749	0.044600774	0.047070568	0.097391828	0.100273096	0.070463135	0.052783853	0	0
TCGA-50-5936-01	0.034866786	0.004227379	0.037654895	0.018030653	0.179603021	0.01463494	0.086933127	0.043328144	0	0.024593699	0	0.043441825	0.185763212	0.025812675	0.161457234	0	0.102469367	0	0.009874146	0
TCGA-50-5939-01	0.002247168	0.081592182	0.15293009	0	0.176508695	0	0.100593555	0	0	0.013499924	0.020023046	0.010751197	0.119807836	0.029971624	0.153065687	0	0.069648446	0.034371252	0	0
TCGA-50-5941-01	0.047852371	0.009460497	0.102283927	0.157466037	0.078640512	0.019662939	0.137926219	0	0	0.00828112	0.017764852	0.046292428	0.053267556	0.066066999	0.122550406	0.044837241	0.027061737	0.043642922	0	0
TCGA-50-5942-01	0.023329877	0	0.137724473	0.038655652	0.20794986	0	0.106609009	0	0	0	0.054740123	0.069231634	0	0.011850258	0.125423618	0.045198949	0.049615782	0.113873222	0	0
TCGA-50-5944-01	0.056297807	0	0.073981866	0.127668511	0.147699077	0	0.025151569	0.040183212	0	0	0.034056843	0.065455892	0.062474864	0.029914621	0.168041724	0	0.046267436	0.120194524	0	0
TCGA-50-5946-01	0.086490322	0	0.121897844	0.118955883	0.064802458	0	0.121688451	0	0	0	0.055722426	0.031902303	0.110701813	0.038532025	0.139517219	0	0.035366624	0.071864241	0	0
TCGA-50-6590-01	0.029963009	0.005919406	0.12369458	0.028065726	0.212661568	0	0.099464886	0.026767056	0	0	0.064198356	0.060391813	0.063709989	0.070596912	0.089616215	0.071936189	0.053014295	0	0	0
TCGA-50-6591-01	0.025443764	0	0.01951534	0.157639871	0.165764064	0	0.102505278	0.046747294	0	0	0.021310551	0.142590631	0.047949183	0.034041125	0.149346135	0	0.086950241	0	0.000196525	0
TCGA-50-6592-01	0.050337969	0.004936227	0.129352674	0.058949377	0.114769549	0.009677838	0.1528598	0	0	0.03322362	0.023579867	0.083893423	0.037635647	0.052674096	0.159142973	0	0.060712023	0	0.002736483	0
TCGA-50-6593-01	0.014388585	0.041326651	0.013058848	0.0318525	0.171839482	0	0.075444497	0.040426068	0	0.007376055	0.013709797	0.07180666	0.090861066	0.03571596	0.149656198	0.083022544	0.055696034	0.072163473	0	0
TCGA-50-6594-01	0	0.001579427	0.202904507	0.009861046	0.237079361	0	0.039273588	0.037979329	0	0.062843561	0.01663438	0.077828517	0.100513287	0.014605498	0.108644942	0.039055973	0.051196584	0	0	0
TCGA-50-6595-01	0	0.006420988	0.044539542	0.087336746	0.134502083	0.000830608	0.086859903	0.019166595	0	0.057398015	0.002324973	0.039938334	0.118933473	0.068635556	0.200473183	0	0.097755849	0.016273413	0	0
TCGA-50-6597-01	0.056251733	0.007071546	0.173740294	0.055366037	0.094301011	0	0.141803343	0.055677752	0.002732534	0	0.032439178	0.044762424	0.022203692	0.05460891	0.119433649	0.053489364	0.049400417	0.036718116	0	0
TCGA-50-6673-01	0.110944001	0	0.160472722	0	0.158021269	0	0.122786582	0.000353334	0	0	0.032118426	0.065086661	0.05772373	0.03489452	0.123209251	0.040775058	0.053058026	0.04055642	0	0
TCGA-50-7109-01	0.099453565	0	0.201076711	0.086469528	0.12889798	0.011685058	0.10057051	0	0	0	0.040342534	0.055293995	0.041240994	0.055006284	0.126618009	0	0.032922869	0.004599585	0	0
TCGA-50-8457-01	0	0.105391047	0.152366568	0.094130288	0.097214135	0	0.112016078	0.008538507	0	0	0.048418702	0.061418007	0.015186057	0.039967886	0.101365318	0.051241135	0.013012727	0.099733546	0	0
TCGA-50-8459-01	0.025233008	0.065330367	0.070106602	0.039758296	0.142403146	0	0.042501651	0.064821108	0	0.003856797	0.019795893	0.078005687	0.054203073	0.039247338	0.137974582	0.099582645	0.034183326	0.067417949	0	0
TCGA-50-8460-01	0.028446488	0.02421329	0.119097146	0.053166407	0.155386383	0	0.07069657	0.041080204	0	0.005211238	0.02910153	0.083637933	0.019157348	0.024650172	0.079731401	0.073210506	0.066279338	0.126934045	0	0
TCGA-53-7624-01	0.132706098	0	0.108231849	0.081812066	0.04472775	0.020306407	0.098094494	0.03001766	0	0.007952052	0.034086111	0.004707797	0.137050515	0.068986444	0.15159745	0	0.067053787	0	0	0.009037819
TCGA-53-7626-01	0.006741515	0.055172122	0.138998677	0.082542201	0.136663518	0	0.147963088	0	0	0.020984577	0	0.067010978	0.002629428	0.045299548	0.120403638	0.040071577	0.042516513	0.019839281	0.034895196	0
TCGA-53-7813-01	0.095481804	0	0.262439956	0.11130874	0.10085823	0.052883682	0.099319461	0	0	0	0.020682249	0.028571461	0.084726541	0.026447257	0.061728579	0	0.05531261	0.000239427	0	0
TCGA-53-A4EZ-01	0.105234918	0	0.139712859	0.094592082	0.04147071	0.007823959	0.173363383	0	0	0.004826487	0.027334036	0.041281521	0.058386627	0.041152738	0.094019988	0.091550033	0.038420893	0.040829766	0	0
TCGA-55-1592-01	0.097254352	0	0.117442445	0.039093811	0.154687281	0	0.065679756	0.017232571	0	0.010374685	0.001029565	0.059895902	0.164469348	0.035051831	0.177975925	0	0.013793241	0.010138241	0.022295401	0
TCGA-55-1594-01	0.131292484	0	0.129878204	0.091432837	0.118051915	0	0.083494461	0.005552339	0	0	0.061224045	0	0.106576508	0.051898897	0.150620326	0	0.001049545	0.03285179	0	0
TCGA-55-1595-01	0.034658859	0.023884958	0.162874116	0.103889118	0.073750073	0	0.131346631	0	0	0	0.047910113	0.022443563	0.066152792	0.05012523	0.190618262	0	0.050419999	0.041926286	0	0
TCGA-55-1596-01	0.065216168	0	0.160658974	0.043587648	0.14896141	0	0.108040216	0.028385484	0	0	0.029105746	0.083826656	0.046522589	0.060819012	0.125112646	0.009704993	0.066145699	0.023912758	0	0

TCGA-55-5899-01	0.006995298	0	0.128637031	0.054963486	0.14983711	0.001391367	0.145177992	0	0	0	0.047176082	0.054006762	0.085506719	0.072936178	0.184773124	0	0.015029519	0.053569332	0	0
TCGA-55-6543-01	0.070314928	0	0.125695706	0.014155027	0.242780424	0	0.002578767	0.002650346	0	0.014165194	0.002488979	0.018481337	0.128944434	0.027062652	0.19207958	0	0.059593984	0.0374882	0.009908615	0
TCGA-55-6642-01	0	0.019518733	0.273235867	0.102118771	0.127163544	0.034827003	0.001016324	0.049843563	0	0.014490239	0.02214271	0	0.092396499	0.031924832	0.127208965	0	0.025367386	0.05288584	0	0
TCGA-55-6712-01	0.056023654	0.035126172	0.07961417	0.099618855	0.140220102	0.006461032	0.086056325	0	0	0.001339945	0.007610815	0.048856938	0.053690072	0.055576832	0.144728612	0.036963699	0.067239343	0.080873434	0	0
TCGA-55-6968-01	0.062250342	0	0.14715647	0.098855652	0.065805616	0.011641449	0.093475862	0.045802967	0	0	0.055002973	0.027300351	0.162768873	0.071606097	0.134548884	0	0	0.017092389	0	0
TCGA-55-6969-01	0.045833148	0.024779284	0.217795067	0.058427935	0.146508443	0.023168406	0.093858646	0.004052101	0	0.028051176	0	0.010542359	0.144522474	0.038366307	0.140543706	0	0.023550946	0	0	0
TCGA-55-6970-01	0.029484169	0.018063758	0.097783961	0.067522226	0.151110583	0.011481464	0.100692564	0	0	0	0.050227516	0.03473707	0.078028306	0.039758829	0.182973855	0	0.072989165	0.048329142	0	0
TCGA-55-6971-01	0	0.103147096	0.112687331	0.086094509	0.100751489	0.019467355	0.110382728	0.012027693	0	0.004453138	0.029387789	0.032279283	0.109587816	0.036823359	0.134456759	0	0.036447762	0.048330402	0	0
TCGA-55-6972-01	0.041264125	0	0.274920507	0.125801662	0.031033991	0	0.085087902	0.054262898	0	0	0.073173749	0.034289337	0.021741844	0.025771228	0.141267703	0	0.068995726	0	0	0.022389329
TCGA-55-6975-01	0.083341454	0	0.146643988	0.07387973	0.161570306	0.025677273	0.096633478	0	0	0	0.011872969	0.01882128	0.089594498	0.018399676	0.140143641	0	0.08798617	0.019231969	0	0
TCGA-55-6978-01	0	0.000178467	0.084672288	0.131861304	0.115349965	0.043450223	0.131355246	0	0	0.003440743	0.038268957	0.023410632	0.059270351	0.069764815	0.181880084	0.009442582	0.062156229	0.043391258	0	0
TCGA-55-6979-01	0.028929235	0.013041311	0.136451294	0.087711467	0.133052711	0.005304106	0.134709664	0.000289125	0	0	0.033128077	0.052772323	0.051001275	0.068092424	0.154609212	0.036966122	0.030441701	0.026205426	0	0
TCGA-55-6980-01	0.073067253	0	0.066376862	0.050851061	0.205737362	0	0.061926877	0.061005794	0	0	0.021003361	0.109486529	0.025217506	0.034721589	0.086284631	0.104649838	0.065837103	0.033834235	0	0
TCGA-55-6981-01	0.113244819	0	0.088488326	0	0.132471563	0	0.133185498	0	0	0	0.03111248	0.063028206	0.055403186	0.00599042	0.092264258	0.104710181	0.073058092	0.107042973	0	0
TCGA-55-6982-01	0.027139672	0.051761723	0.139054876	0.017868979	0.106275684	0	0.127479267	0	0	0.017516183	0.022369891	0.030001115	0.100781916	0.058094527	0.153890584	0.039687986	0.062189053	0.027776952	0	0
TCGA-55-6983-01	0.063668759	0.090515639	0.065836219	0.095831619	0.143214493	0.005929008	0.1501253	0	0	0	0.033907596	0.006507714	0.045547605	0.029554838	0.141136712	0.029178747	0.041970278	0.051186676	0	0
TCGA-55-6984-01	0.072849731	0.04584245	0.093289414	0.020548987	0.215514573	0	0.102525209	0.069592277	0	0.036549832	0	0	0.051907514	0.009588722	0.099623469	0.007486389	0.060416109	0	0.061838379	0
TCGA-55-6985-01	0.002570018	0.028371764	0.141208125	0.125711519	0.122345793	0.002839496	0.120646112	0.003515222	0	0.032831117	0.021826539	0.067336528	0.04046693	0.057993911	0.106986671	0.023311493	0.045482408	0.029483472	0.003077305	0
TCGA-55-6986-01	0.057622884	0.034170298	0.0573014	0.061027087	0.180477227	0	0.11942881	0	0	0.00580527	0.018449767	0.033532545	0.05473262	0.026977346	0.173297563	0.074303232	0.035456932	0.057468037	0	0
TCGA-55-6987-01	0.071681652	0.001771019	0.153207314	0.120168747	0.069653986	0.010933381	0.125548101	0	0	0.038679957	0.018196983	0.030814211	0.108327181	0.057825189	0.120367167	0.000802365	0.027814171	0.028146475	0	0
TCGA-55-7227-01	0.024145033	0.003718575	0.021271474	0.075209928	0.203832992	0	0.102871463	0.02106516	0	0.013540151	0.012752387	0.064341986	0.02795491	0.057932024	0.147416805	0.102417554	0.056223279	0.058804303	0	0
TCGA-55-7281-01	0	0.086786129	0.036335611	0.047389209	0.106142171	0	0.106807409	0.061039042	0	0.011271526	0.033943055	0.092836432	0.018552098	0.046849335	0.129687304	0.10743714	0.0700703	0.038028073	0	0.006825166
TCGA-55-7283-01	0	0.059817261	0.060302444	0.13416478	0.106286664	0	0.120151015	0	0	0.001541956	0.026466043	0.074996543	0	0.038350436	0.148667507	0.031378599	0.066203791	0.099226366	0	0
TCGA-55-7284-01	0.000417098	0	0.040316918	0.03881992	0.180535601	0	0.095979287	0.077527431	0	0.065094521	0	0.025800403	0.067982684	0.042358971	0.193068396	0.056063977	0.050066268	0.047030809	0	0
TCGA-55-7570-01	0.135134017	0	0.183441302	0.114179807	0.050905319	0.01858057	0.136751789	0.000190054	0	0.003993565	0.01802139	0.010420769	0.135963771	0.031259419	0.105075123	0	0.00959388	0.046489225	0	0
TCGA-55-7573-01	0.030242027	0.089635463	0.068901852	0.061567142	0.153465201	0	0.120820283	0.004563264	0	0	0.029429498	0.032886275	0.008361448	0.046819039	0.110797811	0.102114225	0.048685022	0.09171145	0	0
TCGA-55-7574-01	0.133708498	0	0.110460092	0.037469387	0.136682754	0	0.125332976	0.010809515	0	0.027821914	0.016538664	0.051763523	0.034732001	0.02029374	0.137032114	0	0.077543659	0.069591945	0	0
TCGA-55-7576-01	0.048277273	0	0.021295457	0.089525961	0.13657144	0.025272355	0.095323973	0.037669418	0	0	0.013820292	0.048178745	0.102222408	0.043651092	0.157566722	0.047936847	0.041784272	0.085472019	0	0
TCGA-55-7724-01	0.047383926	0.019971503	0.113841598	0	0.184074476	0.009520624	0.089682547	0	0.02543665	0.00957086	0.001499376	0	0.05158083	0.041373851	0.177646338	0.016508915	0.082301208	0.066678005	0	0
TCGA-55-7725-01	0.062680535	0.039084762	0.063790978	0.051004597	0.099660592	0.013013693	0.149469625	0	0.032968906	0	0.01410852	0.013226534	0.041484883	0.05433199	0.125236981	0.060400786	0.053172839	0.115172779	0	0
TCGA-55-7726-01	0.050264886	0	0.182265997	0.012285355	0.238310243	0	0.054401864	0.015477559	0	0	0.027923227	0.037532848	0.017695538	0.0331417	0.107432357	0.095018334	0.068784568	0.023075581	0.004584132	0.007417574
TCGA-55-7727-01	0.028665863	0.108750082	0.120651702	0.018773333	0.124564882	0.054532801	0.08061211	0	0.017141149	0	0.00124897	0	0.048157749	0.055611363	0.179296851	0.016706324	0.046890565	0.077433738	0	0
TCGA-55-7728-01	0	0.126531213	0.014089068	0.075872866	0.076873046	0	0.128465476	0.033443686	0	0.042457001	0.014364633	0.056725952	0.105202108	0.032245774	0.16940961	0.056392155	0.050181072	0.017435795	0	0
TCGA-55-7815-01	0.057593353	0	0.063861778	0.005064522	0.224942317	0.042651513	0.08202029	0	0.031758199	0	0.006664375	0.02639939	0.027501963	0.045727728	0.151757311	0.102311357	0.056473397	0.04780562	0	0
TCGA-55-7816-01	0	0.03407374	0.017884147	0.129486187	0.03358903	0.001841437	0.031462521	0.044547735	0	0	0.082759044	0.094723095	0.058797744	0.075577034	0.280688738	0	0.00179982	0.093257794	0	0
TCGA-55-7903-01	0.011259077	0.016058133	0.048610067	0.083181023	0.113221243	0	0.123987129	0.05945863	0	0.034470342	0.028622558	0.070331746	0.087579386	0.044371385	0.140953066	0.025773536	0.060465025	0.051657654	0	0
TCGA-55-7907-01	0.003740239	0.033871313	0.075242098	0.087130883	0.082854836	0	0.11714206	0.065454153	0	0	0.048214816	0.026052527	0.120516487	0.062186647	0.164084399	0	0.026479967	0.084934242	0	0
TCGA-55-7910-01	0.098519734	0	0.233783868	0	0.156862372	0	0.102320854	0.002247015	0	0.022210654	0	0	0.118735684	0.00713326	0.10167094	0	0.101270431	0	0.026923359	0
TCGA-55-7911-01	0.029891963	0.011052666	0.059672944	0.067017952	0.125027164	0.040619721	0.188091363	0	0	0.055312415	0.009850348	0.058614553	0.058719854	0.046949586	0.136866073	0	0.094070396	0.018243003	0	0
TCGA-55-7913-01	0.047144603	0	0.063861481	0.067627707	0.153145739	0	0.092594319	0.079013215	0	0.057592483	0	0.024778895	0.119232482	0.038592708	0.112321773	0.05799146	0.023561092	0	0.02653567	0
TCGA-55-7914-01	0.052841257	0.009637273	0.085754916	0.027779509	0.167032433	0	0.158462528	0	0	0.026600949	0.019691857	0.065321367	0.029878509	0.067652124	0.148864368	0	0.054800313	0.085682595	0	0
TCGA-55-7994-01	0.108101391	0.015016642	0.117159098	0.112432388	0.074565828	0	0.032545497	0.002520684	0	0.063839517	0.01502462	0	0.020045464	0.055111242	0.194499623	0.084579172	0.071371807	0.003836993	0	0
TCGA-55-7995-01	0	0.104249105	0.034201191	0.128025094	0.105516361	0.040908321	0.080453415	0.025915603	0	0.093870464	0	0.085642804	0.084561854	0.067442943	0.125383514	0	0.02382933	0	0	0
TCGA-55-8085-01	0.06981852	0	0.074005329	0.106141575	0.106855203	0.022744194	0.1153418	0.033093597	0	0.04513817	0	0.050012483	0.137894013	0.042598297	0.116259084	0	0.058986045	0	0.011605257	0
TCGA-55-8087-01	0.007642563	0.011018691	0.011549195	0	0.206835231	0	0.04259498	0.050639421	0	0	0.072913484	0.047106826	0.03884245	0.020709447	0.148092918	0.155319116	0.033663115	0.142632019	0	0
TCGA-55-8089-01	0.048171754	0	0.071166572	0.113534875	0.047051593	0.014540028	0.117827177	0.007717346	0	0	0.09296682	0.047395566	0.100899793	0.06757036	0.151350682	0.012539686	0.03984743	0.067420319	0	0
TCGA-55-8090-01	0	0.063827438	0.044043836	0.016778413	0.194347609	0	0.094026872	0.027514904	0	0.039048323	0	0.070937837	0.046257019	0.042710548	0.181792636	0.095860629	0.044843325	0.019988398	0.006818961	0
TCGA-55-8091-01	0	0.012110389	0.119084644	0.01229469	0.206538822	0	0.038646727	0.050615435	0	0.046134483	0.016498075	0.036402806	0.001331651	0.055039321	0.140287098	0.082258583	0.066595774	0.079155418	0	0
TCGA-55-8092-01	0	0.102579686	0.213067838	0.142180236	0.018092987	0.025883229	0.094759573	0.024753177	0	0	0.051036978	0.024990908	0.10881802	0.059128562	0.124716353	0	0.009992454	0	0	0
TCGA-55-8094-01	0.10542607	0	0.109557018	0.086389044	0	0	0.166916588	0	0	0.02755049	0.0310869	0.009215808	0.187097923	0	0.123526265	0	0.139874906	0.013358988	0	0
TCGA-55-8096-01	0.082430499	0	0.166295366	0.04821578	0.138647181	0.015110022	0.010136171	0.023099257	0	0.037646125	0	0.041574877	0.195232167	0.026172951	0.149648542	0	0.02814773	0.013843559	0.013293603	0
TCGA-55-8097-01	0.041209591	0.046873154	0.06623866	0.031611455	0.134960863	0	0.148574445	0	0	0	0.021792862	0.041986173	0	0.031597964	0.194263101	0.073494929	0.047733568	0.119663235	0	0
TCGA-55-8203-01	0	0.139853065	0.175111029	0.088054502	0.141293075	0.013777786	0.096352745	0	0	0.005580664	0.018089951	0.015892591	0.062423213	0.043582188	0.120255739	0.010728073	0.044377973	0.01369707	0	0
TCGA-55-8204-01	0.044340096	0	0.232900788	0.042940634	0.166056766	0	0.074454291	0	0	0.01358616	0.012165014	0.020748146	0.033725377	0.052985273	0.101538896	0.041773118	0.045380764	0.071000556	0	0

TCGA-55-8205-01	0	0.056037693	0.028153738	0.151320476	0.124478187	0.039870019	0.099662301	0.000571044	0	0.016890005	0.009186417	0.051859593	0.088209246	0.068385138	0.191620647	0.007685221	0.034414127	0.02110979	0	0
TCGA-55-8206-01	0.025402883	0.026241341	0.089145275	0	0.186713153	0	0.067936544	0.017081191	0	0.003657111	0.011997665	0.129578793	0	0.013211353	0.13090606	0.102314466	0.051773575	0.110670965	0	0.023928422
TCGA-55-8207-01	0.052311294	0	0.020645435	0.070290612	0.235508261	0	0.02246536	0	0	0.016232505	0.00284183	0.065621441	0.073298233	0.022993815	0.201677355	0.058341543	0.048943008	0.093983246	0	0
TCGA-55-8208-01	0.07185641	0.007757886	0.132265448	0.064051651	0.116585658	0.008793758	0.121658362	0.007489629	0	0.019659527	0.000479186	0.003932724	0.098200759	0.067237523	0.176971259	0	0.036457749	0.0279644	0	0
TCGA-55-8299-01	0.036264589	0.010809699	0.086163118	0.148071628	0.027074507	0.021787174	0.089449201	0.015751552	0	0.047132978	0.023248558	0.014857291	0.112624715	0.048156389	0.142059553	0	0.076511655	0.078947336	0	0
TCGA-55-8301-01	0.00732671	0.06279047	0.130766532	0.104568587	0.124186821	0.010306265	0.049144069	0.029884097	0	0	0.024233483	0.051868418	0.049744301	0.059939004	0.147788193	0.044648552	0.05980797	0.042996527	0	0
TCGA-55-8302-01	0	0.08632765	0.081356017	0.101036569	0.053248843	0.012071363	0.146970786	0.004336625	0	0.028567621	0.015598379	0.056613667	0.106052178	0.052772624	0.150243298	0.003932083	0.051894147	0.046276765	0	0
TCGA-55-8505-01	0.050065678	0	0.246181641	0	0.138840063	0	0.043979601	0.045519554	0	0.018254578	0.016160646	0	0.162734904	0.02461042	0.153765887	0	0.012852537	0.039745637	0.010318538	0
TCGA-55-8506-01	0	0.021436954	0.123846724	0.109394644	0.091945535	0	0.13736815	0	0	0.003164872	0.038661052	0.079212307	0.04271374	0.046220241	0.121908022	0.055800836	0.06046289	0.049801415	0	0
TCGA-55-8507-01	0	0.058395366	0.048299543	0.061610421	0.098469756	0	0.095193441	0	0	0.041749527	0.040445778	0.026068721	0.145885789	0.012474238	0.162604054	0	0.08611297	0.071135239	0	0
TCGA-55-8508-01	0.086716847	0	0.258201883	0.102361008	0.0415793	0.038749401	0.080180786	0.001446736	0	0.026312983	0.011557366	0.03900386	0.119202911	0.016692273	0.092011261	0	0.032787902	0.000484368	0	0
TCGA-55-8510-01	0.010084925	0.054619655	0.01521306	0.11553794	0.112119911	0.000834123	0.112603236	0.026670447	0	0.041618026	0	0.042587681	0.091290019	0.056338637	0.173164191	0.054454782	0.031805024	0.053592609	0	0
TCGA-55-8511-01	0.080183291	0.01117911	0.090856309	0.108928384	0.084843175	0.037868884	0.154659572	0	0	0.000764644	0.01007153	0.025271367	0.12843909	0.058644708	0.154573191	0	0.027687319	0.026029423	0	0
TCGA-55-8512-01	0.016069919	0	0.085879026	0.153222323	0.001963483	0	0.142150534	0	0	0.063320676	0.050142339	0.077655121	0.097225766	0.008160393	0.13076126	0	0.075578116	0	0.0155886	0
TCGA-55-8513-01	0.008912741	0.107895235	0.029903104	0.005656691	0.131978004	0	0.124216948	0	0	0.051703916	0	0.04296072	0.125928682	0.022535666	0.185101378	0.052292152	0.068920819	0.025104584	0	0
TCGA-55-8514-01	0.056710361	0.005810997	0.078251899	0	0.091175397	0	0.158516535	0.020250405	0	0.041940317	0.007268661	0.044490074	0.141398238	0.048460976	0.201249636	0	0.049781563	0.037406265	0	0
TCGA-55-8614-01	0	0.052089293	0.089769876	0.09108034	0.097873923	0	0.113110482	0.037266731	0	0	0.033927694	0.044311975	0.035806619	0.055995934	0.13812365	0.032705478	0.063022192	0.092047959	0	0
TCGA-55-8615-01	0.019742234	0	0	0.025833687	0.153265935	0	0.160213555	0.070585301	0	0.022702531	0.030917258	0.094523503	0.163477033	0	0.15036089	0	0.093589222	0	0	0
TCGA-55-8616-01	0.160106515	0	0.099306426	0	0.219810678	0	0.121539935	0.012094983	0	0	0.004621197	0.070845656	0.052435665	0.030547391	0.091462019	0	0.061398157	0.061505388	0	0
TCGA-55-8619-01	0.069929917	0.022914034	0.074854604	0.105733357	0.066826304	0	0.10150975	0.009920942	0	0.043003818	0.002714628	0.009183417	0.116524341	0.031810338	0.197416684	0	0.030283281	0.073021422	0	0
TCGA-55-8620-01	0.07708279	0	0.14832855	0.116154594	0.030356568	0.022514476	0.099640131	0.044203116	0	0	0.029388021	0.018733865	0.176727609	0.061869307	0.143089396	0	0.009138228	0.020346064	0	0
TCGA-55-8621-01	0.038512241	0	0.155000185	0.075756086	0.084390496	0	0.056583814	0.015856554	0	0.057427885	0.010828499	0.046250376	0.121504231	0.04256155	0.174238186	0	0.03101366	0.074859544	0	0
TCGA-55-A48X-01	0.056564793	0.116948684	0.069726408	0.083228948	0.118856704	0	0.099933567	0.042217117	0	0.012830122	0.022012152	0.032364591	0.02792865	0.045366204	0.114239612	0.035290119	0.028948069	0.093544262	0	0
TCGA-55-A48Y-01	0.109066168	0	0.177847576	0.045973688	0.117590869	0.011760573	0.081835392	0.016635045	0	0	0.03115674	0.021315003	0.135468979	0.028053199	0.116301754	0	0.071616675	0	0	0
TCGA-55-A48Z-01	0	0.033717333	0.172221956	0.036250658	0.145020646	0	0.087098099	0.074983936	0	0.043767234	0.013523493	0.043297354	0.043355837	0.046820131	0.119894759	0.078784554	0.029181778	0.032082233	0	0
TCGA-55-A490-01	0.082105319	0	0.116814795	0.090254722	0.130973337	0.015403035	0.024127045	0.099781134	0	0.014979654	0	0.056128223	0.138592809	0.024649672	0.104851108	0.063119081	0.02014189	0	0.018078177	0
TCGA-55-A491-01	0.116457849	0	0.181514898	0.035471925	0.084641082	0	0.094533426	0.052705369	0	0.02346989	0.01937089	0.064407888	0.086374774	0.037577133	0.116530492	0	0.033961945	0.052982439	0	0
TCGA-55-A492-01	0.041067011	0	0.199426439	0.098826574	0.047724025	0	0.104938049	0.02372427	0	0.009522495	0.054793779	0.04216409	0.084951271	0.036995457	0.153656065	0	0.04897303	0.053237446	0	0
TCGA-55-A493-01	0.106491711	0	0.086325489	0.102687671	0.089653621	0.018378735	0.141589767	0	0	0.016727802	0.029255446	0.057829406	0.080919923	0.045687226	0.126562841	0.046193975	0.051696388	0	0	0
TCGA-55-A494-01	0.121695443	0	0.001705166	0.109944298	0.07680366	0	0.10949708	0.049034771	0	0.019456716	0	0.090113265	0	0	0.187736997	0	0.1147664	0	0.086122136	0
TCGA-55-A4DF-01	0.018895949	0.032113509	0.088732268	0.063924818	0.115065142	0.041578098	0.124481492	0.007257529	0	0.020512421	0.038053624	0.05736191	0.141481463	0.074942865	0.140145516	0	0.025786777	0.009666618	0	0
TCGA-55-A4DG-01	0.034773606	0.09087451	0.135300779	0.113625871	0.052918149	0	0.123249707	0	0	0	0.049791037	0.025156774	0.086426987	0.043575792	0.124085574	0	0.029155679	0.091065533	0	0
TCGA-55-A57B-01	0.05998225	0	0	0.043061579	0.142908783	0	0.07102608	0.096852745	0	0.010704504	0.015743892	0.065146999	0.013607346	0.022707494	0.169038721	0.101423785	0.084648321	0.074745352	0	0
TCGA-62-8394-01	0.027527169	0.000783845	0.100043263	0.071603162	0.165814486	0	0.086813329	0.067509341	0	0.015116377	0.025302248	0.052635293	0.129682171	0.055392392	0.138805981	0	0.062970944	0	0	0
TCGA-62-8395-01	0.061100065	0	0.112030705	0.039948218	0.204591788	0	0.1018387	0.032235917	0	0.023992726	0.010460883	0.039631387	0.038856985	0.031947137	0.151589301	0.079532675	0.027481892	0.044761619	0	0
TCGA-62-8397-01	0.044984195	0	0.13887916	0.034482048	0.243061622	0	0.023558577	0.013746894	0	0.013894678	0.029172596	0.093691909	0.027435726	0	0.104681543	0.097391948	0.034729903	0.100289201	0	0
TCGA-62-8398-01	0.090870235	0	0.106308349	0.020468001	0.166077135	0	0.121125896	0	0	0.038177046	0.042112786	0.072528873	0.047771279	0.053741931	0.088398593	0.0711483	0.035986292	0.02024686	0	0
TCGA-62-8399-01	0.067188628	0	0.164277524	0	0.179975448	0	0.167205511	0	0	0	0.026367999	0.069387709	0.003376952	0.031285823	0.126112054	0	0.077622767	0.023775221	0	0.028078082
TCGA-62-8402-01	0.010462844	0	0.076116819	0.075111223	0.191472294	0.023798708	0.084795825	0.070613494	0	0	0.052850788	0.040595413	0.095628947	0.087490599	0.127165695	0	0.026426585	0	0	0
TCGA-62-A46O-01	0.086432487	0	0.235807279	0.035884497	0.028423287	0	0.127326541	0.019067769	0	0.036122606	0.030667402	0	0.164323534	0	0.098782942	0	0.120227403	0.016934252	0	0
TCGA-62-A46P-01	0.037544308	0.007133363	0.09644756	0.074529844	0.140949861	0	0.119987122	0.046495565	0	0	0.047984046	0.05423201	0.054783449	0.015753446	0.162236825	0.015279593	0.075545271	0.030776413	0	0
TCGA-62-A46R-01	0.097344288	0	0.057859841	0.110737546	0.098678678	0	0.054920028	0.032965149	0	0.028286488	0.015646801	0.101127025	0.030676857	0.014823897	0.100564132	0.058251319	0.076775211	0.121342741	0	0
TCGA-62-A46S-01	0.013508614	0.01452548	0.234709614	0.023308616	0.160685521	0	0.111635462	0.030675166	0	0.043822832	0.007926203	0.077314677	0.002803494	0.054384008	0.151318965	0.022981514	0.047448347	0.002951487	0	0
TCGA-62-A46U-01	0.018462946	0	0.084209385	0.003765146	0.221552784	0	0.116961426	0.008119664	0	0	0.059152197	0	0.037613765	0.075625561	0.17266778	0.074372785	0.065913929	0	0	0
TCGA-62-A46V-01	0.063967463	0	0.135900868	0	0.160698942	0	0.055505952	0.0730875	0	0	0.021045114	0.042044642	0.01965231	0	0.134014693	0.125678962	0.099274991	0.064232661	0	0
TCGA-62-A46Y-01	0	0.093915638	0.049965012	0.07432178	0.069877767	0	0.155689781	0.019589041	0	0.032143773	0.02452085	0.06602986	0.02701325	0.046125587	0.166619539	0.075605907	0.064691634	0.03203593	0	0
TCGA-62-A470-01	0.07472495	0	0.022561088	0.067823085	0.210233147	0	0.015867687	0.043042932	0	0.017605023	0.002122502	0.024981982	0.143027446	0.03466444	0.250754172	0	0.044955829	0.014588804	0.017792802	0
TCGA-62-A471-01	0.002643344	0.031477469	0.121301576	0.114287799	0.020626504	0	0.204863803	0	0	0.039658872	0.05691946	0	0.07546983	0.01503078	0.132442707	0	0.098178808	0.049461669	0	0
TCGA-62-A472-01	0.037607371	0.020852475	0.046140084	0.087709629	0.081821756	0	0.135021972	0.035408815	0	0.065396764	0.013360887	0.048294583	0.075057915	0.046695866	0.131448935	0.06429735	0.038747394	0.072138205	0	0
TCGA-64-1676-01	0.008800652	0.017012497	0.082046302	0.107380036	0.126221104	0.010165026	0.086235244	0.05659747	0	0	0.011227601	0.094987863	0.08099499	0.053263248	0.171106167	0.059970901	0.020665332	0.007846169	0.001004905	0
TCGA-64-1677-01	0.113468559	0	0.161268713	0.107850524	0.053122939	0.024171323	0.156720337	0.001071499	0	0.017861802	0.029563604	0.046254099	0.066227274	0.052414587	0.076167943	0.04219015	0.051646647	0	0	0
TCGA-64-1678-01	0.101308779	0	0.258861863	0.107876859	0.123777173	0.01915322	0.082786603	0.024561707	0	0.020849344	0.01185427	0	0.009065722	0	0.100805344	0.053986097	0.052809268	0	0.003365482	0
TCGA-64-1679-01	0.011199061	0	0.179572148	0	0.249115007	0	0.042840928	0.011336518	0	0	0.014355885	0.021839292	0.008100428	0.027539671	0.139841111	0.115526066	0.078418546	0.020756661	0.024101367	0
TCGA-64-1680-01	0.056769681	0	0.184273081	0.047411886	0.171769488	0	0.104001036	0	0	0	0.034597143	0.00530902	0.050306506	0.029736855	0.149078359	0.013046641	0.038387414	0.113572243	0	0

TCGA-64-1681-01	0.03558745	0.019912981	0.102016134	0	0.202515683	0	0.071621226	0.022201391	0	0	0.010616425	0.050088744	0.021476786	0.052896249	0.120506693	0.113509705	0.089652974	0.081362895	0	0
TCGA-64-5774-01	0.089977644	0	0.209814035	0	0.215786199	0	0.118295552	0.035754467	0	0	0.064506695	0.015791343	0.038079408	0.00701242	0.117518326	0	0.047177562	0	0	0
TCGA-64-5775-01	0	0.004158649	0.037064448	0.120950084	0.094737984	0	0.091297507	0.072333704	0	0	0.053103146	0.082023368	0.072482967	0.039151663	0.226071909	0	0.042233654	0	0.033020362	0
TCGA-64-5778-01	0.051865619	0.035619063	0.143424194	0.188367115	0.024084468	0.041465573	0.111283792	0.009744491	0	0	0.040643146	0.026934488	0.068901895	0.071286958	0.135733383	0	0.03131775	0.019328062	0	0
TCGA-64-5779-01	0.083254082	0	0.254225253	0.082146696	0.166514635	0.015492009	0.107905708	0	0	0	0.015473634	0.034889964	0.060891671	0.028743679	0.080634491	0	0.043946841	0.025881338	0	0
TCGA-64-5781-01	0.064132934	0	0.189755521	0.177358125	0.049345993	0.010495296	0.143918751	0.022292857	0	0.008893319	0.048591575	0.018387147	0.086208053	0.037069811	0.0878978	0	0.040680751	0.014972067	0	0
TCGA-64-5815-01	0	0.036757331	0.086377177	0.003074904	0.233977423	0.01011333	0.04559621	0.041431494	0	0.027846472	0.003230714	0.065971375	0.050074825	0.030843869	0.129922352	0.102140371	0.072501983	0.041288806	0	0
TCGA-67-3770-01	0.005399322	0	0.113047071	0.058726724	0.189289423	0	0.087324767	0.010766032	0	0.027491758	0.017479096	0.047079624	0	0.051778855	0.179793896	0.059557679	0.046190204	0.080007376	0	0
TCGA-67-3771-01	0.109649051	0	0.155144436	0.080273948	0.150074406	0.02714561	0.029151126	0.031997928	0	0	0.02874649	0.043277915	0.118648704	0.061720431	0.138885106	0	0.010731686	0	0	0
TCGA-67-3772-01	0.090576506	0	0.170073224	0.035001452	0.159221709	0.001890338	0.075861911	0	0	0	0.031745707	0.051586272	0.013653519	0.027574775	0.095297169	0.093779237	0.056265336	0.08808183	0	0
TCGA-67-3773-01	0.022954329	0	0.090064514	0.067065073	0.178350765	0	0.112289107	0.011763727	0	0	0.028116011	0.020175695	0.012879822	0.05234982	0.167211655	0.101843751	0.034123635	0.083265168	0	0
TCGA-67-3774-01	0	0.097743577	0.075838584	0.035588803	0.168474784	0	0.099977588	0.006737962	0	0	0.042309508	0.024075976	0.091373537	0.050208709	0.152499332	0	0.049730717	0.073534692	0	0
TCGA-67-4679-01	0.046704087	0	0	0.076553708	0.219757625	0	0.048955137	0.01164754	0	0.002695056	0.006431817	0.075869811	0	0.029863752	0.162843169	0.100933187	0.040691165	0.146308928	0	0
TCGA-67-6215-01	0	0.125371016	0.034477249	0.010270515	0.21766972	0.001322339	0	0.024452736	0	0.019305526	0	0.034844869	0.091328828	0.026661705	0.18489653	0.076656195	0.038849542	0.098574736	0	0
TCGA-67-6216-01	0	0.115180613	0.051611725	0.082052106	0.147470212	0	0.082241707	0.013833889	0	0.027490708	0.003012642	0.094624697	0	0.029809929	0.112851099	0.101719915	0.048664196	0.08943656	0	0
TCGA-67-6217-01	0.03053296	0.062293875	0.120935106	0.122799611	0.082458282	0	0.097869156	0.008466026	0	0	0.043626215	0.038372393	0	0.043614602	0.145778198	0.070399058	0.045589504	0.087265015	0	0
TCGA-69-7760-01	0.13179666	0	0.14218868	0	0.094358019	0	0.050214127	0.073301198	0	0	0.017172678	0.059700831	0.064045005	0.001434491	0.163722046	0	0.164464389	0	0.030591609	0
TCGA-69-7761-01	0	0.046165623	0.161734859	0.077644447	0.135604738	0.027321034	0.095376628	0.00545216	0	0.004425088	0.002397943	0.018260284	0.102399678	0.080369702	0.123555309	0	0.029182736	0.066346819	0	0
TCGA-69-7763-01	0.091659263	0	0.148707009	0.051961232	0.163895221	0	0.069396622	0.008292169	0	0	0.033717509	0.012424463	0.110816731	0.028397157	0.177521527	0	0.039994193	0.049215082	0	0
TCGA-69-7764-01	0.07975446	0	0.228284042	0.02578514	0.216637897	0	0.123569926	0	0	0	0.032143851	0	0.033705927	0.033172876	0.118828924	0.010447562	0.061440595	0.0362288	0	0
TCGA-69-7765-01	0	0.074061883	0.156902961	0.044872735	0.158112095	0.008414925	0.06575881	0.030521012	0	0.006062983	0.013476427	0.011585899	0.056276376	0.04287189	0.114385236	0.08431853	0.051791143	0.051675772	0	0
TCGA-69-7973-01	0.095923918	0	0.075362165	0.165732388	0.13615176	0.033890378	0.085262906	0	0	0	0.019833227	0.057495235	0.02145056	0.023842019	0.140947704	0	0.048310325	0.071084371	0	0
TCGA-69-7974-01	0	0.055883215	0.037453715	0.064580407	0.173856943	0.006292553	0.097056398	0	0	0.043102827	0.009958398	0.056070146	0.05128402	0.053828795	0.192639173	0.070101724	0.043327583	0.044510878	0	0
TCGA-69-7978-01	0.010153924	0.028416362	0.099711007	0.080385892	0.14336012	0.036186782	0.074172396	0.020796107	0	0	0.047336893	0.039213702	0.03940209	0.050496324	0.12797355	0.096075789	0.042494057	0.050203171	0	0
TCGA-69-7979-01	0.083532767	0	0.18258624	0.026151532	0.089210924	0.013149206	0.102781074	0	0	0	0.046314124	0.042407377	0.075133412	0.052848153	0.132732693	0.021470266	0.048772386	0.071831027	0	0
TCGA-69-7980-01	0.018308356	0.028440277	0.081463629	0.105479362	0.183181653	0.010897879	0.13270661	0.011225536	0	0.006225105	0.011601347	0.038619834	0.051701748	0.051576559	0.187995717	0	0.049008025	0.026150013	0	0
TCGA-69-8253-01	0.046574923	0.021091465	0.199383441	0.007568342	0.094334736	0	0.162623753	0	0	0.028514252	0.038990984	0.016162664	0.003471373	0.008083534	0.146901497	0.074282111	0.075324402	0.036086487	0	0
TCGA-69-8254-01	0.025587716	0.059080844	0.131072226	0.070108088	0.167562267	0	0.129727026	0	0	0	0	0.025453719	0.023333297	0.049705639	0.155653157	0.045299505	0.055094927	0.020487002	0.018626884	0
TCGA-69-8255-01	0.059046025	0	0.152847844	0.140844542	0.081639162	0.059370719	0.131380607	0	0	0.0093973	0.033267932	0.033399133	0.09504617	0.057482399	0.130406781	0	0.014632018	0	0	0
TCGA-69-8453-01	0.013042474	0.054818497	0	0.072536256	0.143081253	0	0.141516821	0.008697556	0	0.038895328	0	0.032308383	0.13429475	0.053480911	0.146568175	0.05255337	0.034008062	0.042698988	0.006337071	0
TCGA-69-A59K-01	0.053245168	0	0.239878061	0.072567857	0.115832446	0.020166093	0.075110254	0.012126273	0	0.038055096	0	0.041977179	0.057210284	0.043083845	0.13687446	0.025987789	0.03296094	0.026714063	0	0
TCGA-71-6725-01	0	0.075055495	0.062376187	0	0.150331078	0	0.117482894	0	0	0.033835781	0.037198226	0.094450042	0.037563822	0.004315061	0.213625609	0.025678419	0.100459086	0	0.017269402	0
TCGA-71-8520-01	0.015357467	0	0.082066968	0.002554144	0.239594314	0	0.088356289	0.034973042	0	0.029320447	0.018814467	0.056903969	0.087328452	0.025438487	0.141822495	0.042596691	0.078852862	0.045995263	0	0
TCGA-73-4658-01	0.002111586	0.013128304	0.105867009	0.11771556	0.134225334	0.012139082	0.103697606	0.003013804	0	0.010892343	0	0.055249535	0.026390561	0.038012579	0.16168612	0.09182552	0.055900582	0.014883424	0	0.014382412
TCGA-73-4659-01	0	0.026832139	0.056084997	0	0.196604709	0	0.064522024	0	0	0.02710594	0.018153812	0.039836008	0.114421747	0.008778991	0.208275309	0.021011956	0.080709859	0.078963279	0	0
TCGA-73-4662-01	0.001579988	0.097496537	0.123483814	0.06553857	0.148661447	0.012205299	0.113385751	0.025574037	0.003387567	0	0.023153107	0.04605418	0.014418486	0.038690853	0.128763973	0.057805152	0.050953159	0.048722099	0	0
TCGA-73-4666-01	0	0.00441005	0.019233689	0.172422546	0.064755986	0.056591419	0.108243649	0.021523076	0	0.007657327	0.029492627	0.027047017	0.09557382	0.067600674	0.217125631	0	0.033307332	0.059582459	0	0
TCGA-73-4668-01	0	0.113468963	0.196686818	0.003590381	0.094097793	0	0.133680847	0.007419514	0	0.040820895	0	0.068914572	0.102286797	0.00374666	0.047188094	0.027611928	0.103304461	0.030465873	0	0
TCGA-73-4670-01	0.061431459	0	0.116361249	0.05436015	0.159568436	0.018962419	0.078910785	0	0	0.027764871	0	0.019918481	0.141532125	0.019382535	0.152293516	0	0.087143183	0	0.014996886	0
TCGA-73-4675-01	0.059273265	0	0.032416035	0.00171205	0.134497263	0	0.053763539	0.050099301	0	0	0.032432749	0.047432164	0.102690707	0.008652522	0.196055699	0.08245888	0.074833057	0.095311708	0	0
TCGA-73-4676-01	0	0.031864511	0.029250333	0.096858816	0.099815615	0.007197588	0.094660719	0	0	0	0.051017694	0.069428088	0.119585387	0.012174694	0.174832527	0.010453446	0.069186335	0.104764125	0	0
TCGA-73-4677-01	0.014596039	0.014308359	0.190805122	0.06184252	0.182967154	0	0.079521364	0	0	0.011676871	0.016278721	0.023542255	0	0.020699479	0.163287483	0.039859979	0.081779652	0.056664206	0	0
TCGA-73-7498-01	0.017239953	0.018110297	0.145864987	0.092312178	0.11387571	0	0.119066627	0.006757232	0	0	0.05739452	0.08685562	0.024412575	0.01946445	0.117918003	0.0116629	0.051242124	0.117822822	0	0
TCGA-73-7499-01	0.02895089	0	0.085911833	0.16970858	0.099561198	0.041734151	0.091823503	0.040956908	0	0	0.031546086	0.031290935	0.051853486	0.069237106	0.116444458	0.054601962	0.038861625	0.046279989	0	0
TCGA-73-A9RS-01	0.111132064	0	0.14966901	0.115880165	0.031342838	0.021307141	0.127949091	0.020929967	0	0.010926108	0.007474079	0	0.125504058	0.032260294	0.144328191	0	0.027849453	0.072091005	0	0
TCGA-75-5125-01	0.012907445	0	0.082348501	0.070055507	0.217776525	0.028803982	0.086707956	0.001221184	0	0	0.03387544	0.052923731	0	0.059543615	0.164367312	0.032273867	0.061515324	0.048331554	0	0
TCGA-75-5146-01	0.059146329	0.040831366	0.177844996	0.044203891	0.153654998	0	0.135917654	0	0	0	0.024819932	0.031526507	0	0.033208958	0.170323313	0.025570505	0.044667874	0.047658801	0	0
TCGA-75-5147-01	0.083691904	0	0.162616797	0.043856994	0.177004613	0.034317233	0.072539167	0.006789726	0	0	0	0.043470282	0.08234547	0.046821521	0.12144037	0.023692873	0.042173155	0.037195895	0	0
TCGA-75-6206-01	0.126015716	0	0.086759598	0.022237588	0.116571644	0	0.098562749	0	0	0.001387755	0.038189588	0.033419031	0.126187535	0.01900239	0.211683866	0	0.05602928	0.047907868	0	0
TCGA-75-6212-01	0.119494559	0	0.131118732	0	0.176220959	0	0.062655498	0.028720386	0	0	0.047401106	0.004045548	0.000128849	0.018582473	0.129750743	0.093300833	0.054353599	0.114929462	0	0
TCGA-75-6214-01	0	0	0.067448088	0.119899999	0.133516488	0	0.104934029	0.030785923	0	0.03144302	0.017010362	0	0.12231442	0.031538006	0.171739742	0	0.092005975	0	0.016603925	0
TCGA-75-7025-01	0.009873009	0.1080002	0.052012173	0.095866711	0.117952455	0	0.118722144	0	0	0.021507009	0	0.071199123	0	0.023295142	0.135500595	0.084313162	0.077766909	0.083991369	0	0
TCGA-75-7027-01	0.145124429	0	0.125967009	0.04731451	0.134316153	0.009846349	0.059263566	0.030531819	0	0.022644746	0.005092968	0.018614547	0.144305623	0.041181728	0.128524419	0	0.021273064	0.061761632	0	0
TCGA-78-7143-01	0.041209724	0.014195843	0.057796565	0.01066337	0.178219527	0	0.120354692	0.030710544	0	0	0.021356654	0.054073442	0.057729666	0.027285913	0.139805037	0.089979573	0.094177524	0.062441925	0	0

TCGA-78-7145-01	0.000781196	0	0.025471002	0	0.311748029	0.01651967	0.042941636	0.000195256	0	0	0.021863169	0.071853033	0.083349628	0.008952428	0.153317114	0.067179503	0.103509125	0.070738848	0.00039607	0
TCGA-78-7146-01	0.04672367	0	0.062220603	0.109302884	0.193004254	0.01859807	0.096744685	0.01036622	0	0	0.030606165	0.039160592	0.13171141	0.048898485	0.143297609	0	0.04284096	0.024625447	0	0
TCGA-78-7147-01	0.019607774	0	0.077380765	0.067256006	0.120789647	0	0.089362173	0.103841173	0	0	0.031581666	0.063736794	0.035308038	0.073697741	0.134654282	0.0287597	0.056444112	0.097580129	0	0
TCGA-78-7148-01	0.064165463	0	0.111609401	0.090105451	0.123918388	0	0.105696855	0.062248073	0	0.018703674	0.0351779	0.056667909	0.055263863	0.043319873	0.131603281	0.001335974	0.040748635	0.058731755	0	0
TCGA-78-7149-01	0.1019725	0	0.176941114	0.08724929	0.094450939	0	0.098624868	0.038636592	0	0.018083434	0.028638156	0.004296939	0.085489621	0.034477105	0.122350794	0	0.03588659	0.071532485	0	0
TCGA-78-7150-01	0.124781935	0	0.115914772	0.075793868	0.120186427	0	0.107941635	0.001618587	0	0	0.056439701	0	0.132245108	0.026175102	0.145216966	0	0.070955507	0	0	0
TCGA-78-7152-01	0.010333598	0.108391636	0.061824226	0.133183502	0.125008116	0.001261163	0.154900462	0.025224163	0	0	0.017294027	0	0.050178323	0.057869524	0.138009474	0.042478174	0.02688118	0.038062825	0	0
TCGA-78-7153-01	0.101055209	0	0.183949236	0.131124765	0.137495465	0	0.098676313	0	0	0.011417813	0.010703635	0.032799315	0.035249796	0.026480353	0.127651315	0.020160135	0.038919255	0.044317396	0	0
TCGA-78-7154-01	0.051542416	0	0.037414929	0.060610489	0.224714641	0	0.126610927	0	0	0.058510554	0	0.083707989	0	0.054788365	0.186786336	0	0.074735453	0.026063209	0	0
TCGA-78-7155-01	0.015249828	0	0.075838797	0.088486271	0.052095865	0	0.212373202	0	0	0	0.10576138	0.080404395	0.011972395	0.064188654	0.22196225	0	0.041579996	0.030086967	0	0
TCGA-78-7156-01	0	0	0.205969871	0.010978909	0.149486999	0	0.114370948	0	0	0	0.073377486	0.071985771	0.022461477	0.056916252	0.131183966	0	0.04360955	0.104666705	0	0
TCGA-78-7158-01	0.005754655	0.018964652	0.034809364	0.01696648	0.255414411	0	0.042709057	0.043399206	0	0	0.013469794	0.06140297	0.063983507	0.032932257	0.165291829	0.069648598	0.054420903	0.116468458	0	0
TCGA-78-7159-01	0.064247757	0	0.164800962	0.063252233	0.145678617	0	0.07759643	0.016266021	0	0	0.036254335	0	0.12318979	0.026552508	0.131119932	0	0.08157694	0.01499067	0	0
TCGA-78-7160-01	0.036692312	0.017808502	0.073842772	0.105758538	0.143455538	0	0.123684804	0	0	0	0.047045967	0.050762362	0.04038227	0.045273615	0.141403788	0.035376724	0.066100133	0.058884408	0	0
TCGA-78-7161-01	0.029037293	0	0.17792412	0	0.154839339	0	0.116780949	0.087740296	0	0.015100938	0.033142025	0.040566712	0.097110485	0.026720047	0.116365956	0	0.048007292	0.016249096	0	0
TCGA-78-7162-01	0	0.086138093	0.063991586	0.107168873	0.073130831	0	0.137027687	0	0	0	0.057173271	0.026623924	0.018563923	0.042569063	0.186921004	0.031257923	0.049396271	0.12003755	0	0
TCGA-78-7163-01	0.10173335	0	0.15710605	0.056829372	0.094398749	0	0.122202188	0.08358454	0	0.041662856	0.028982946	0.041392058	0.01362088	0.037241229	0.115749833	0.005955701	0.049457578	0.050082671	0	0
TCGA-78-7166-01	0.036593451	0.069214195	0.104461486	0.092665415	0.109038275	0	0.135077789	0.053437461	0	0.033632393	0.013267589	0.035774553	0.104656851	0.055612384	0.110706427	0	0.04586173	0	0	0
TCGA-78-7167-01	0.085442393	0	0.110927816	0.068591715	0.116798806	0	0.113089855	0.027095735	0	0.00664147	0.049004323	0.057279476	0.068411461	0.036017638	0.122100951	0.024775129	0.024262715	0.089560516	0	0
TCGA-78-7220-01	0.073043239	0	0.077664209	0.147339322	0.057937032	0.002650656	0.164832236	0	0	0	0.042655438	0	0.131431026	0.031330581	0.176866352	0	0.033313268	0.05001026	0	0
TCGA-78-7535-01	0	0.176193161	0.081031092	0.058200524	0.13195169	0	0.045530061	0.025212779	0	0.069043254	0.006841178	0.074992415	0.00444382	0	0.145801638	0.098798944	0.035931076	0	0.026577055	0.013582219
TCGA-78-7536-01	0.032354249	0	0.145021877	0.116049877	0.134218288	0.080720474	0.092264159	0	0	0.005517116	0.007046993	0.054445044	0.057064673	0.045064401	0.18492858	0	0.014203506	0	0.004890539	0
TCGA-78-7537-01	0.024664421	0.022498565	0.200982547	0.055129502	0.098274711	0	0.083238305	0.067341014	0	0.035053153	0.019044501	0.044393212	0.020721827	0.01146436	0.127099138	0.044755784	0.062856286	0.082482673	0	0
TCGA-78-7539-01	0.031584846	0.040660056	0.078899	0.088338735	0.186997605	0.001187267	0.141444929	0	0	0	0.020273067	0.049981368	0.015949485	0.035663243	0.153456692	0.041588814	0.082818161	0.031156735	0	0
TCGA-78-7540-01	0	0.105182249	0.053693225	0.106364794	0.067702474	0	0.086808069	0.0379109	0	0.058420884	0.009349871	0.033940724	0.119195739	0.02625442	0.143022821	0.098002051	0.041392117	0.012759661	0	0
TCGA-78-7542-01	0.047159502	0	0.082649496	0.050172338	0.075668803	0.011917433	0.235256964	0.002039021	0	0.005016037	0.066352077	0.024157121	0.023186177	0.044072835	0.161143549	0.054995394	0.110664751	0.005548504	0	0
TCGA-78-7633-01	0.115288776	0	0.076031507	0	0.230694669	0	0.074472456	0	0	0	0.070740912	0.066558415	0.045813839	0	0.150794893	0	0.089601802	0.068088125	0	0
TCGA-78-8640-01	0.074597321	0	0.135051407	0.087795086	0.101402039	0	0.102303148	0.022505925	0	0.055683158	0.014488605	0.035204922	0.14394391	0.043259048	0.13667943	0	0.026840125	0.020245874	0	0
TCGA-78-8648-01	0	0.061021273	0.11792259	0.135956139	0.045353259	0.013836992	0.09341686	0.013258745	0	0	0.04045033	0.036703495	0.143595314	0.028487464	0.148676397	0	0.031267742	0.079367675	0	0
TCGA-78-8655-01	0.045582091	0	0.054181076	0.141553667	0.105337496	0.011063372	0.090221397	0.017896308	0	0.001859402	0.01746113	0.059038717	0.066159669	0.056436298	0.201683509	0.024362099	0.034469506	0.065852379	0	0
TCGA-78-8660-01	0.054283842	0	0.093356585	0.191052046	0.041013304	0	0.151478482	0.010089989	0	0	0.045449213	0.052849576	0.03649477	0.049282656	0.158427345	0.043500258	0.04292677	0.029795163	0	0
TCGA-78-8662-01	0.094165151	0	0.063794452	0.092492501	0.149094338	0	0.133953663	0.004970035	0	0	0.0258193	0.029252608	0.117632253	0.055228499	0.156448244	0	0.063108216	0	0.014040741	0
TCGA-80-5608-01	0.054792993	0	0.134090523	0.092453441	0.086019295	0	0.119522237	0.02491206	0	0.047996894	0.03834498	0.039504454	0.063645877	0.043819072	0.133070907	0.016960692	0.027855208	0.024904452	0	0
TCGA-80-5611-01	0.021197376	0	0.067168214	0.034791431	0.161401968	0.000971541	0.086307462	0.040895629	0	0.013676112	0.035778216	0.09703919	0.04111748	0.088740327	0.158826073	0.035234326	0.05194729	0.057919071	0	0
TCGA-83-5908-01	0.081173807	0	0.053495096	0.138055837	0.051158848	0.05338093	0.161220489	0.000409543	0	0	0.075760951	0.022266504	0.023197658	0.071939234	0.150407679	0.027701932	0.041729753	0.048101739	0	0
TCGA-86-6562-01	0.01790553	0.016993239	0.116959252	0	0.169408698	0	0.049431433	0.038229302	0	0.08200395	0	0.017387259	0.123193293	0.025814923	0.175385784	0	0.063289603	0.051919088	0.018564016	0
TCGA-86-6851-01	0	0.062724428	0.175643962	0.116205704	0.080043523	0.014124322	0.118444228	0.02987829	0	0.004482145	0.037004482	0.011229329	0.08622177	0.067747614	0.137161067	0	9.70E-05	0.055411251	0	0
TCGA-86-7701-01	0.070472574	0.005285673	0.182737781	0.076240852	0.100417958	0.020582684	0.136799638	0	0	0.018972152	0.033650231	0.022857809	0.050875268	0.039814906	0.090523599	0.039629721	0.043879241	0.04109897	0	0
TCGA-86-7711-01	0	0.004646464	0.053086416	0.079347513	0.191737385	0	0.081634799	0.002887574	0	0.051573693	0.017359994	0.03951885	0.122777905	0.04895595	0.160961394	0	0.053528486	0.002624358	0.009917024	0
TCGA-86-7713-01	0.094672184	0	0.149083071	0	0.150006531	0.010355385	0.092036415	0	0	0.004216059	0.028270113	0.041357691	0.125797999	0.040960666	0.152259257	0	0.098263583	0	0	0
TCGA-86-7714-01	0.104025858	0	0.04828364	0.011893143	0.201962553	0	0.029790611	0.034388053	0	0	0.034401405	0.039638023	0	0.016251027	0.143442177	0.159724936	0.08095383	0.062855459	0	0
TCGA-86-7953-01	0.007579235	0.04122853	0.011398277	0.054110773	0.240239923	0.018768956	0.092075684	0.037638717	0	0.035062729	0	0.077093846	0.062925136	0.030623728	0.141750496	0.02775363	0.070355759	0	0.025589335	0
TCGA-86-7954-01	0.023672104	0.013963793	0.046096101	0.095659549	0.121537243	0.007814215	0.130375541	0.025809104	0	0	0.033145268	0.03196123	0.085741356	0.084892089	0.17606254	0	0.048606589	0.058263494	0	0
TCGA-86-7955-01	0.113929622	0	0.097493612	0.067387277	0.146523069	0	0.112012323	0.064435436	0	0.028896589	0.048227059	0.029948112	0.092395422	0.026961176	0.095937409	0	0.048882301	0.090970593	0	0
TCGA-86-8054-01	0.196412993	0	0.089730273	0.00846572	0.138459029	0	0.064425107	0.010158984	0	0.01875376	0.029870997	0.012934099	0.11777354	0.011902421	0.135486424	0.015282245	0.035575375	0.087645211	0	0
TCGA-86-8055-01	0.104017707	0	0.174565812	0.00777348	0.221024248	0.005584415	0.043697011	0.010414686	0	0.015335096	0	0.024327247	0.094029534	0.037974615	0.138564035	0.018074746	0.047826523	0.023463107	0.004379155	0
TCGA-86-8056-01	0.007552359	0.052922056	0.047587354	0.038110037	0.151902447	0	0.102258881	0	0	0.039335397	0	0.04393586	0.097153538	0.043047043	0.161842866	0.045496016	0.059968146	0.085363753	0	2.79E-05
TCGA-86-8073-01	0.023081853	0	0.098484609	0.09965582	0.121882101	0	0.099907141	0.017187227	0	0	0.026121938	0.055940095	0.094390215	0.048525482	0.194118436	0	0.040056401	0.074309517	0	0
TCGA-86-8074-01	0.035821601	0.029004575	0.12746936	0	0.190843429	0	0.141619365	0.019291474	0	0.027718737	0.001223094	0.075409073	0.038659127	0.042075111	0.111241443	0.042851703	0.077023327	0	0	0
TCGA-86-8075-01	0.052729904	0	0.165250431	0	0.226950757	0	0.062503666	0.015253822	0	0.042165806	0	0.026224227	0.069866815	0.025325618	0.134512364	0.023878914	0.083470142	0	0.036350756	0
TCGA-86-8076-01	0.025101926	0.046547618	0.136211021	0.128999006	0.09004106	0	0.124669854	0.027608421	0	0	0.040022292	0.02047358	0.064542706	0.03530196	0.099031959	0.039913448	0.045012067	0.04481391	0	0
TCGA-86-8278-01	0	0.073204893	0.132219491	0.020826447	0.253073491	0	0.055601064	0	0	0.040337876	0.024168746	0.010259813	0.016022104	0.039774315	0.126145929	0.074394256	0.060451031	0.063833174	0	0
TCGA-86-8279-01	0.021120111	0	0.070675572	0.062662662	0.104908902	0	0.126647798	0.021481041	0	0	0.07595024	0.071815931	0.050725159	0.017540248	0.121715566	0.105190786	0.109732398	0.039833587	0	0
TCGA-86-8280-01	0.05633599	0	0.113159113	0.030406694	0.142720589	0	0.134472324	0	0	0.002618035	0.023201064	0.065210167	0.037084933	0.054988445	0.108625037	0.090660517	0.052396496	0.088120595	0	0

TCGA-86-8358-01	0.033906973	0.031127248	0.141328577	0.097178693	0.15819125	0	0.165009422	0	0	0	0.006854175	0	0.035893046	0.060823077	0.245499662	0.024187877	0	0	0	0
TCGA-86-8359-01	0.039176578	0	0.162394521	0.115036826	0.146234287	0	0.163889876	0	0	0.012068516	0.041489707	0.065488908	0.058382156	0.044659222	0.019102136	0	0.039749421	0	0.021594774	0
TCGA-86-8585-01	0.010490655	0.064810697	0.050737589	0.119304691	0.073185875	0.04089427	0.14049564	0	0	0.00118198	0.025891891	0.055141455	0.154530852	0.06150168	0.139079952	0	0.036833278	0.025919495	0	0
TCGA-86-8668-01	0.005419091	0.090389439	0.113066355	0.035372275	0.1040407	0	0.127302482	0.030675068	0	0	0.048741532	0.034764643	0.047704555	0.04412612	0.154001487	0.03012227	0.039870868	0.094403114	0	0
TCGA-86-8669-01	0.080513438	0	0.199195321	0.036884144	0.154955981	0	0.115342842	0	0	0.020501564	0.015175252	0.061105805	0.017668424	0.029062567	0.115132861	0.071420574	0.058478985	0.006225196	0	0
TCGA-86-8671-01	0.063483043	0.034990574	0.040073562	0.13290931	0.035413667	0	0.144880821	0.03545567	0	0	0.063964023	0.045263242	0.089591354	0.063151039	0.13085549	0.015006685	0.038576203	0.066385317	0	0
TCGA-86-8672-01	0	0.034899815	0.166767441	0.102136521	0.08103344	0.029648077	0.147898704	0	0	0.029696932	0.036597547	0.049558886	0.095649462	0.045728544	0.140499032	0.008883475	0.031002124	0	0	0
TCGA-86-8673-01	0	0.123460014	0.154054083	0.126448415	0.004620488	0	0.122432868	0.043176577	0	0.036928559	0.03991712	0.038627036	0.084348927	0.033579774	0.13476462	0	0.053624567	0	0	0
TCGA-86-8674-01	0.062420936	0	0.162479687	0.017487159	0.162719812	0	0.186662758	0.017745718	0	0.029600637	0.046223163	0.033112084	0.039526423	0.00715019	0.113398182	0	0.092966595	0	0	0
TCGA-86-A456-01	0	0.050567016	0.062461716	0.024739042	0.206873342	0	0.145190778	0	0	0	0.023073729	0.03095117	0.102569539	0.040671101	0.209736248	0	0.076532513	0.001068813	0.025564993	0
TCGA-86-A4D0-01	0.045560995	0	0.15246169	0.050530526	0.063427797	0	0.199888599	0	0	0.033757306	0.047619801	0.042826603	0	0.041852799	0.198668385	0.07298103	0.05042447	0	0	0
TCGA-86-A4JF-01	0.035510775	0	0.079967221	0.090764024	0.123659309	0	0.131923133	0	0	0.029877142	0.027859361	0.032851512	0.070377506	0.043770964	0.148423682	0.021021443	0.084584204	0.059534246	0	0
TCGA-86-A4P7-01	0.008567148	0.075570421	0.121277765	0.107625197	0.054296082	0	0.134107173	0.014736734	0	0.046401071	0	0.023033242	0.147716797	0.025044268	0.162615026	0	0.018331502	0.060677574	0	0
TCGA-86-A4P8-01	0	0.039761789	0	0.089525072	0.207511205	0	0.075191019	0	0	0.037729059	0.029315239	0.042997654	0.132318461	0.037290817	0.204079687	0	0.026305774	0.077974224	0	0
TCGA-91-6828-01	0	0.050638097	0.083383562	0.105456507	0.167871689	0.029579668	0.099212232	0	0	0	0.00467216	0.04374241	0.055509036	0.050572499	0.182497126	0.029234543	0.032975163	0.022600839	0	0
TCGA-91-6829-01	0.143071849	0	0.027990678	0.050398073	0.175465482	0	0.025706793	0.028274495	0	0.016528104	0.013981478	0.015233445	0.188265595	0.013967285	0.152612746	0	0.027997611	0.099852652	0	0
TCGA-91-6830-01	0.0859326	0	0.074682264	0.073352612	0.127741091	0	0.082616493	0	0	0	0.053208819	0.017965147	0.112813073	0.04069973	0.1500316	0	0.05249747	0.087515453	0	0
TCGA-91-6831-01	0.060251059	0	0.174723476	0.075768249	0.170367496	0.020453583	0.050881158	0.017695586	0	0.005680512	0.023665625	0.033998941	0.095544848	0.053233095	0.131497739	0	0.030134689	0.022485355	0	0
TCGA-91-6835-01	0.121543459	0	0.06378581	0.055004109	0.199673371	0	0.101021987	0	0	0	0.017465126	0.008420468	0.043086837	0.045776069	0.168413396	0.053046358	0.05952834	0.063234669	0	0
TCGA-91-6836-01	0.036899605	0	0.122218807	0.108317626	0.17731523	0.032206	0.078887616	0.015144166	0	0.040206524	0	0.01730079	0.106848035	0.013769738	0.122674724	0	0.074577255	0.002267411	0.018752847	0
TCGA-91-6840-01	0.076000721	0	0.100935268	0.072562139	0.136568232	0.004643405	0.120026246	0	0	0	0.059708417	0.042513804	0.048044026	0.064512367	0.144083976	0	0.04265273	0.087748669	0	0
TCGA-91-6847-01	0.010797486	0	0.011649993	0.069065094	0.050434129	0	0.188571464	0.08296103	0	0.144462488	0.04162228	0.06886285	0.108501989	0.025962577	0	0.018870138	0.061969223	0.073535041	0	0
TCGA-91-6848-01	0	0.035989594	0.116284451	0.077093912	0.187465189	0.047836366	0.075663021	0	0	0.019690841	0.021815592	0.056236983	0.05999469	0.061736466	0.168589282	0.015798308	0.040278133	0	0	0
TCGA-91-6849-01	0.041587366	0.068173371	0.166084378	0.071080159	0.104629285	0.009659665	0.10685338	0	0	0.019594202	0.003298351	0.013283247	0.093162195	0.017347086	0.1343089	0.049164158	0.063109106	0.009807162	0	0
TCGA-91-7771-01	0.030532075	0.021655902	0.073439546	0.091927716	0.166417756	0	0.10405534	0.010755569	0	0	0.061975064	0.02947153	0.055792441	0.047689274	0.179507471	0.01497347	0.044132687	0.055380379	0	0
TCGA-91-8496-01	0.020258259	0	0.001826647	0.060941415	0.140808149	0	0.070219416	0.020999073	0	0.041179108	0.027232087	0.090382858	0.030621982	0.01783554	0.141615661	0.090755735	0.060306831	0.126303176	0	0.012770084
TCGA-91-8497-01	0.07241384	0.04146151	0.181046991	0.011604016	0.078995839	0	0.141183405	0.006780207	0	0	0.029872885	0.003976599	0.05578693	0.020643589	0.156684291	0.041896037	0.057004854	0.095509963	0	0
TCGA-91-8499-01	0	0.018490265	0.152608376	0.057233026	0.166464585	0.01070507	0.132938128	0	0	0.020031511	0.015302143	0.033126425	0.083163024	0.067581879	0.214214627	0	0.012324815	0.008295943	0	0
TCGA-91-A4BC-01	0.037657061	0	0.135242255	0.165064588	0.062844105	0.035198456	0.127579393	0.021256336	0	0	0.057416823	0.031742095	0.106965904	0.046409691	0.130927256	0	0.006946745	0.034749293	0	0
TCGA-91-A4BD-01	0.022634187	0	0.105450841	0.049938817	0.186905263	0	0.068328875	0	0	0.021635993	0.028282759	0.093518614	0.040550923	0.028155094	0.179980411	0.03484033	0.043996782	0.08190239	0	0
TCGA-93-7347-01	0.027639889	0.022057098	0.05947363	0.144995029	0.130645042	0.003515264	0.111995525	0.047837058	0	0	0.01056909	0.035551572	0.030062011	0.056189592	0.138545824	0.060629661	0.045248827	0.075044887	0	0
TCGA-93-7348-01	0.113101163	0	0.197563659	0.062313238	0.126453024	0.002193023	0.117718109	0.022675859	0	0.006345224	0.016061864	0.025939753	0.074508645	0.040188129	0.090078995	0.025501878	0.036233669	0.043123769	0	0
TCGA-93-8067-01	0.065207565	0	0.12078908	0.126542664	0.071888736	0	0.147219623	0.008360269	0	0	0.043115082	0.050451892	0.047110067	0.051526235	0.153032284	0.059875444	0.037471215	0.004144219	0	0
TCGA-93-A4JN-01	0.124767562	0	0.135278046	0.022580494	0.071082029	0	0.163416351	0	0	0	0.041670102	0.064577914	0.04670651	0.033260902	0.13192101	0	0.058609179	0.106129902	0	0
TCGA-93-A4JO-01	0.008249931	0.086571054	0.075812054	0.121387786	0.076649362	0	0.135139395	0	0	0.005465619	0.026721304	0.057143263	0.034181493	0.061778344	0.183287724	0.050781308	0.01553088	0.061300482	0	0
TCGA-93-A4JP-01	0.048112657	0	0.024195531	0.009041876	0.085452633	0	0.127583048	0	0	0.022539383	0.032096043	0.027281082	0.090955069	0.045043102	0.22135938	0	0.113972896	0.122070503	0	0.004201922
TCGA-93-A4JQ-01	0.043235984	0.136264526	0.036962751	0.04228568	0.157685222	0.016674314	0.206506135	0	0	0.05115171	0	0.009714331	0.046226225	0.069165028	0.128145448	0.019863727	0.036118919	0	0	0
TCGA-95-7039-01	0.057332579	0	0.051708454	0.143240355	0.099463086	0.014096382	0.11773311	0.003358716	0	0	0.055002983	0	0.077509959	0.057377681	0.175263815	0.023586985	0.041172348	0.083153545	0	0
TCGA-95-7043-01	0.069689067	0	0.153222631	0.149280482	0.024443189	0	0.139895213	0.03199167	0	0	0.054939365	0.043202851	0.051446989	0.036760368	0.157151964	0	0.049246221	0.038729992	0	0
TCGA-95-7562-01	0.057688698	0	0.117604539	0.045013626	0.182800513	0.016359511	0.075249586	0.029105011	0	0	0.016022637	0.028136061	0.059539263	0.087561758	0.151356675	0.026327032	0.035549931	0.047945102	0	0
TCGA-95-7567-01	0.030666323	0	0.169013852	0.006569457	0.182806929	0	0.096423565	0.052007864	0	0	0.051212276	0.060368633	0.014519515	0.037510102	0.191443921	0.001546451	0.086917633	0	0.01899348	0
TCGA-95-7944-01	0.017629395	0	0.048654377	0.175902714	0.053204225	0.015826459	0.128881799	0.032316013	0	0.02177212	0.049602116	0.034629135	0.128894159	0.060461723	0.168977274	0	0.058801783	0	0	0
TCGA-95-7947-01	0.044190426	0.005767534	0.049108309	0.053298522	0.179231143	0	0.160490911	0	0	0.060173566	0	0.012159523	0.029558833	0.081295924	0.217225937	0	0.054079312	0.031742413	0	0
TCGA-95-7948-01	0.001466376	0.022407949	0.122865277	0.102436704	0.199286252	0	0.097271697	0	0	0	0.037043773	0.053860699	0	0.020070457	0.150882061	0.069606071	0.068317296	0.054485386	0	0
TCGA-95-8039-01	0.006805773	0.042116501	0.031592977	0	0.233775563	0	0.056498403	0.036209138	0	0.011976248	0.032698363	0.048366133	0.01212288	0.041173501	0.147948138	0.102652141	0.069951421	0.113064453	0	0
TCGA-95-8494-01	0	0.053876625	0.080082195	0.002207395	0.200768502	0	0.07280622	0.011343708	0	0.035439087	0.03403647	0.159114236	0.011186747	0	0.098601894	0.099002431	0.073056938	0.068477553	0	0
TCGA-95-A4VK-01	0.11128064	0.03043298	0.158346638	0.008963801	0.192666563	0	0.159770371	0	0	0.024106546	0	0.032202254	0.072033242	0.029514341	0.034627965	0.000204762	0.086093152	0.026576893	0	0
TCGA-95-A4VN-01	0.062959405	0	0.068540251	0.179046956	0.073755404	0.00248232	0.144633138	0	0	0	0.035357892	0.065163538	0.018387934	0.058626379	0.136961615	0.048417408	0.067534371	0	0.004049854	0
TCGA-95-A4VP-01	0.121234746	0	0.076636271	0.169219609	0.000756572	0	0.15981084	0.034850771	0	0.026719353	0.002373046	0	0.123214942	0.038493459	0.196962799	0	0.04760999	0	0	0
TCGA-97-7546-01	0.030731338	0.025700064	0.1144237	0.062872811	0.193046116	0	0.118594669	0	0	0.024581699	0	0.071255395	0	0.02478357	0.117437897	0.06304776	0.071219037	0.055998276	0	0
TCGA-97-7547-01	0.088256181	0	0.133358268	0.114386713	0.112531412	0	0.116637717	0.00418435	0	0	0.020633492	0.083774737	0.031522273	0.02344484	0.116827405	0.023267537	0.050053595	0.081121482	0	0
TCGA-97-7552-01	0.022890958	0.069269796	0.121614154	0.113318064	0.080661741	0	0.121607361	0.002908554	0	0	0.075741994	0.04283534	0.072820476	0.051612413	0.074047209	0.032187208	0.026351346	0.088837367	0	0
TCGA-97-7553-01	0.060743993	0	0.042927854	0.07841414	0.136991509	0	0.110897281	0	0	0.037470525	0.000730307	0.011784426	0.116802081	0.027832431	0.167328224	0.041440479	0.070240914	0.045410243	0.002997491	0
TCGA-97-7554-01	0.051052561	0.000637665	0.186592044	0.048335297	0.198149604	0	0.076371963	0.002181983	0	0	0.025111481	0.026845512	0.089885026	0.040411196	0.145378881	0	0.054936016	0.037447397	0	0

TCGA-97-7937-01	0.071935143	0	0.140037614	0.022124713	0.142424969	0	0.097129908	0.010409947	0	0.025394649	0.008464418	0.038337365	0.082739472	0.030559731	0.135105643	0.041956807	0.071163009	0.050781599	0.014034676	0
TCGA-97-7938-01	0.095048146	0	0.027629513	0.10254485	0.093756044	0	0.120784495	0	0.005405659	0	0.04618319	0.00770555	0.077706134	0.052774952	0.169275527	0.040864476	0.040441675	0.108476609	0	0
TCGA-97-7941-01	0.012623591	0.081402848	0	0.069659907	0.150281555	0	0.11680786	0.033143738	0	0.04236031	0.003749785	0.033755254	0.082262959	0.040581429	0.188594076	0.047031271	0.023416771	0.074328643	0	0
TCGA-97-8171-01	0.098951107	0	0.15914668	0	0.141652088	0	0.113989391	0	0	0.002285061	0.031894796	0.050983981	0.114139412	0.003814136	0.155436898	0.001725294	0.085342064	0.040639092	0	0
TCGA-97-8172-01	0	0.156593401	0.082039101	0.072805384	0.110000005	0	0.132182546	0	0.003331227	0	0.030997404	0.036660022	0.041982705	0.044563077	0.129005912	0.012694188	0.02557114	0.121573887	0	0
TCGA-97-8174-01	0.064139403	0	0.101046041	0.049730173	0.156709709	0	0.100238027	0.000984829	0	0	0.052551743	0.054974816	0.025319834	0.051811337	0.193638791	0	0.040752677	0.108102618	0	0
TCGA-97-8175-01	0	0.083004852	0	0	0.124906139	0	0.158142236	0.004804408	0	0.061314974	0	0.067773503	0.049540559	0.048735032	0.180652612	0.096695739	0.064830938	0	0.010937068	0
TCGA-97-8176-01	0.051741958	0.006290851	0.059681036	0.050496692	0.06838342	0	0.068237902	0.016623662	0	0.05473706	0	0.042569569	0.185821208	0.032205838	0.252327469	0	0.024964666	0.022940977	0	0
TCGA-97-8177-01	0.067847368	0.007259941	0.067944018	0.080717295	0.13733819	0	0.094858145	0.027912039	0	0	0.019534739	0.059843733	0.058257613	0.024016952	0.138901914	0.082757561	0.064509457	0.068301036	0	0
TCGA-97-8179-01	0.029223043	0	0.11777064	0	0.119307637	0	0.179888647	0	0	0.019163153	0.049840005	0.067669537	0.002240952	0.01533531	0.141932291	0.029000903	0.068543065	0.138172649	0	0
TCGA-97-8547-01	0	0	0.045209108	0	0.152586544	0	0.113953312	0.061110103	0	0	0.043813885	0.092409402	0.054805741	0.028472036	0.134494623	0.094949451	0.078008632	0.100187164	0	0
TCGA-97-8552-01	0	0.109699883	0.049237552	0.080206642	0.127756852	0	0.107843718	0	0	0.023960588	0.016151342	0.075681168	0	0.026579398	0.16201931	0.050097171	0.065468419	0.082143412	0	0
TCGA-97-A4LX-01	0.043698398	0.033494872	0.094178332	0.106173634	0.063592294	0	0.124505878	0.041226536	0	0.005841281	0.032840535	0.034145297	0.061305768	0.058025986	0.154322079	0.031980722	0.032726661	0.069277818	0	0
TCGA-97-A4M0-01	0	0.058232566	0.028261893	0.135117733	0.123953315	0	0.075178462	0.076781194	0	0.007156568	0.020283095	0.091561312	0.017543279	0.04018324	0.115197033	0.057747091	0.063071749	0.08973147	0	0
TCGA-97-A4M1-01	0.063721695	0	0.016162565	0.110075922	0.105323085	0	0.08326808	0.004127069	0	0.001327906	0.061491688	0.076423476	0.062166756	0.023662505	0.182659061	0.0331308	0.032112196	0.14339291	0	0.000860914
TCGA-97-A4M2-01	0.002393745	0.051635875	0.066704346	0.102601857	0.107860703	0	0.101243823	0.003791667	0	0.060312348	0.004877889	0.05081337	0.100006132	0.036899263	0.165511917	0.034147115	0.039740258	0.060577689	0	0
TCGA-97-A4M3-01	0.129413305	0	0.034624095	0.043886146	0.192777231	0	0.131829898	0.023288638	0	0	0.038931317	0.079415789	0.056991967	0.020502613	0.157010964	0.017311689	0.042115376	0.018787641	0.013113332	0
TCGA-97-A4M5-01	0.022840779	0.02280091	0.041207987	0.056331137	0.076796611	0	0.129653528	0	0	0.093680336	0.016615289	0.050207917	0.089763159	0.040645446	0.185733021	0	0.058294901	0.094431907	0	0
TCGA-97-A4M6-01	0.10578384	0	0.027734152	0.054780937	0.107930308	0	0.128305312	0.069551001	0	0.013498498	0	0.047801287	0.064478638	0.055680898	0.124792648	0.055726542	0.05696578	0.057850988	0	0
TCGA-97-A4M7-01	0.053815726	0	0.071085022	0.060159159	0.100720643	0.00867761	0.153829751	0.035406531	0	0	0.007085028	0.06498328	0.011928966	0.048749597	0.120304648	0.091770368	0.050673638	0.118581282	0	0
TCGA-99-7458-01	0.009428619	0.022492603	0.037081548	0.081775665	0.191181628	0	0.090230593	0.073582178	0	0	0.032256778	0.044431582	0.028203702	0.039931825	0.111199506	0.112624316	0.053734846	0.071844611	0	0
TCGA-99-8025-01	0.037075595	0.038607764	0.138480452	0	0.162702565	0	0.135529353	0.002520993	0	0.015157902	0.025069958	0.01273659	0.123137472	0.048086877	0.182743659	0	0.041473219	0.024293161	0	0
TCGA-99-8028-01	0.010060115	0.028581916	0.136608994	0.1326822	0.082092251	0.032387752	0.107492751	0.044599426	0	0.014761333	0.012826866	0.019938821	0.101952447	0.056834994	0.154133002	0	0.030622906	0.026906937	0	0
TCGA-99-8032-01	0.075493661	0	0.24049678	0.040218237	0.090516803	0	0.090762435	0.035461584	0	0	0.026714614	0.016281976	0.116904082	0.012220173	0.12728117	0	0.042854622	0.073571953	0	0
TCGA-99-8033-01	0.017091342	0	0.091063302	0.145571327	0.008010409	0.017599885	0.155529651	0.004012578	0	0.041189108	0.05480475	0.032605818	0.13125357	0.030413576	0.119680093	0	0.092059909	0.038713961	0	0
TCGA-99-AA5R-01	0.005776183	0.119008938	0.07471051	0.117790974	0.118939318	0	0.153527347	0	0	0.056910181	0	0.02689176	0.025061148	0.0510194	0.15161566	0.042642606	0.02239632	0.02977726	0.003932395	0
TCGA-J2-8192-01	0.0715198	0	0.074041242	0.011714682	0.220325484	0	0.068738121	0.004985132	0	0.020509239	0	0.032171725	0.051373554	0.032500471	0.159126724	0.084717642	0.061226223	0.078327117	0	0
TCGA-J2-8194-01	0.078427365	0	0.07136789	0	0.256676619	0.003643507	0.054589259	0	0	0	0.021375241	0.033539756	0.045648438	0.049708389	0.168507811	0.066082337	0.04462146	0.030529366	0	0
TCGA-J2-A4AD-01	0.040043147	0	0.17775415	0.045825957	0.082118539	0	0.120803929	0	0	0	0.03902548	0	0.138697729	0	0.151568154	0	0.10308504	0.074914355	0	0
TCGA-J2-A4AE-01	0.006407071	0.070982354	0.163131336	0.067522488	0.080017186	0	0.169347709	0	0	0	0.017787867	0.042832227	0.002422561	0.041499341	0.148057319	0.068441136	0.070280723	0.051270681	0	0
TCGA-J2-A4AG-01	0.058469318	0.087198736	0.05667538	0.039091674	0.125646254	0.027798525	0.128715329	0.023446697	0	0.034071525	0	0.048100075	0.021788126	0.024603362	0.146776033	0.07361545	0.080027994	0.007548487	0.016427034	0
TCGA-L4-A4E5-01	0.032155967	0	0.134722515	0.080664179	0.147770903	0.00874455	0.13882	0.051472763	0	0.004899644	0.034282311	0	0.061995406	0.047784111	0.139422242	0.036309459	0.031882207	0.049073743	0	0
TCGA-L4-A4E6-01	0	0.059575909	0.049795658	0.040586266	0.144076653	0	0.134885466	0	0	0.029786858	0.013825377	0.054185573	0.105673659	0.030649046	0.186884216	0.048406935	0.046813291	0.042181172	0	0
TCGA-L9-A443-01	0.022429813	0.009584481	0.106956963	0.086273028	0.174110078	0	0.116983151	0.034279546	0	0.032878721	0.009586278	0.048598218	0.02264189	0.055079997	0.176495724	0.029850535	0.050423207	0.016727191	0.007101181	0
TCGA-L9-A444-01	0	0.049667099	0.123742752	0.157139103	0.127155203	0.034473917	0.089367775	0.022136293	0	0	0.045082586	0.011240409	0.06457907	0.066302589	0.133253513	0	0.01194045	0.061755291	0	0
TCGA-L9-A50W-01	0.02757734	0	0.18193959	0.040105335	0.074856958	0	0.107907857	0.072139092	0	0	0.072352526	0.04416206	0.006100528	0.041793697	0.118632326	0.066994827	0.042278268	0.079200088	0	0
TCGA-L9-A5IP-01	0	0.051070236	0.09408216	0.109377531	0.104077032	0	0.110018972	0.026055732	0	0.026687652	0.035651769	0	0.181309512	0.039317845	0.14719418	0	0.020783429	0.002971967	0	0
TCGA-L9-A743-01	0.021905249	0.061180993	0.072067847	0.071869518	0.110841081	0.04603518	0.127613184	0.01518397	0	0.047455207	0	0.032607308	0.041468236	0.047324541	0.135721479	0.058985556	0.063443172	0.046297478	0	0
TCGA-L9-A7SV-01	0.034452317	0.047322128	0.105000455	0	0.123052568	0	0.206877742	0	0	0.025230669	0.019882666	0.081259677	0.015465466	0.073707461	0.201561558	0.01175561	0.054431684	0	0	0
TCGA-L9-A8F4-01	0.022402208	0.037200722	0.150274767	0.13325157	0.018782811	0.059541077	0.136848454	0.032409857	0	0	0.010449086	0.018306869	0.15282084	0.058470344	0.141018592	0	0.000522366	0.027700437	0	0
TCGA-MN-A4N1-01	0.089914369	0	0	0.021762799	0.096359533	0	0.124288095	0.111834677	0	0.010280574	0.040579684	0.127825648	0.103165818	0.019637269	0.139672344	0.040379074	0.074300117	0	0	0
TCGA-MN-A4N4-01	0.057507316	0	0.170186362	0.079469246	0.083101993	0.013802705	0.052002482	0.044936783	0	0.01277032	0.008948593	0.038475044	0.202132617	0.034478265	0.137566946	0	0	0.053710216	0	0
TCGA-MN-A4N5-01	0.068995931	0.016425937	0.060351033	0.026212628	0.090861706	0	0.175247231	0	0	0.074316055	0.050104509	0.058081734	0.036506842	0.068633516	0.157819484	0	0.049453713	0.059648422	0	0
TCGA-MP-A4SV-01	0.048101489	0	0.129130445	0.111490597	0.095809838	0.028498865	0.107953352	0.018320965	0	0	0.052858245	0.046618082	0.037617838	0.04886572	0.124805576	0.045212344	0.049287593	0.05542905	0	0
TCGA-MP-A4SW-01	0	0.10757062	0.118766152	0.036999868	0.171072292	0	0.107714929	0	0	0.0570586	0	0.047970262	0.087139502	0.033552665	0.139313724	0.009851484	0.039239042	0.026652522	0.01709834	0
TCGA-MP-A4SY-01	0	0	0.063547539	0.013506327	0.214559053	0	0.04022254	0.115705807	0	0.016795816	0.005112862	0.045324654	0.079258662	0.036694866	0.177267884	0.060387193	0.06228836	0.047262941	0	0
TCGA-MP-A4T4-01	0	0.035220148	0.136382563	0.142831893	0.089209002	0.01110369	0.119102488	0.018676251	0	0.044954371	0.011429735	0.032781721	0.074577422	0.060933079	0.1239338	0.017943524	0.033937125	0.039818426	0	0
TCGA-MP-A4T6-01	0.061696682	0.006553438	0.101688942	0.076146628	0.106608234	0	0.162047047	0.016242615	0	0.021001989	0.017093645	0.029973833	0.07430333	0.054046763	0.121986295	0.042073322	0.038669445	0.06456924	0	0
TCGA-MP-A4T7-01	0.012801287	0.034844066	0.048810839	0.090951018	0.143524695	0	0.130334816	0.014967113	0	0.031867959	0.023841497	0.030985324	0.113175138	0.037015855	0.160025816	0	0.085703339	0.018869566	0	0
TCGA-MP-A4T8-01	0.139360528	0	0.100287536	0	0.11261225	0	0.060345413	0.07866446	0	0.008681063	0.045766667	0.014643451	0.138773731	0	0.1453947	0	0.118090577	0.015730496	0	0
TCGA-MP-A4T9-01	0.022944995	0.01055396	0	0.071403236	0.173853304	0	0.075404797	0.015646777	0	0.07215776	0	0.050770932	0.12203998	0.043850828	0.197978613	0.005929555	0.048201827	0.076771377	0	0
TCGA-MP-A4TA-01	0.083867462	0	0.108727825	0.112452828	0.100100744	0	0.1047683	0.046771858	0	0.030917851	0.016118175	0.051648825	0.099186973	0.042771672	0.14322714	0	0.055695332	0	0.003745015	0
TCGA-MP-A4TC-01	0	0.066953986	0.120835953	0.032832048	0.106872257	0	0.143265826	0.009242245	0	0.053965995	0.009398261	0.03327298	0.05614304	0.068363585	0.194099263	0.01932381	0.03064942	0.007422524	0.026825464	0
TCGA-MP-A4TD-01	0.049242405	0.07595356	0.141814211	0.099346067	0.107746025	0	0.140065406	0	0	0.013159322	0.005368924	0.050948332	0.078512405	0.020378081	0.092969283	0.050091557	0.046856788	0.027547632	0	0

TCGA-MP-A4TE-01	0.149218698	0	0.199874913	0.086578152	0.068774425	0.009680876	0.099146215	0.038693372	0	0	0.015115526	0.004572709	0.094002145	0	0.121644682	0	0.042975865	0	0	0
TCGA-MP-A4TF-01	0	0.069302577	0.244922988	0.077747954	0.065049547	0.018939333	0.091900316	0.003312701	0	0.016648359	0.041727042	0.007345403	0.125835957	0.045491457	0.088905067	0	0.054949679	0.016457227	0	0
TCGA-MP-A4TH-01	0.043876194	0.094291053	0.165680623	0.092396397	0.083797196	0	0.1611702	0	0	0.003594359	0.002019555	0.020671715	0.013969978	0.038842872	0.148706409	0.056406169	0.039255007	0.035322272	0	0
TCGA-MP-A4TI-01	0.031539353	0	0.006727293	0.157082069	0.047664694	0.026381341	0.132063501	0.043918348	0	0.058788299	0.004018137	0.068004583	0.044762132	0.06015594	0.173996482	0.035130273	0.04914509	0.046223586	0	0
TCGA-MP-A4TJ-01	0.006752823	0.081106006	0.11878116	0.120744014	0.080507655	0.039003009	0.147114195	0	0	0.022122267	0.004286605	0.018714443	0.09049159	0.054434386	0.133602302	0	0.030140997	0.033338718	0	0
TCGA-MP-A4TK-01	0.012688417	0.058074376	0.20029105	0.004920596	0.112427171	0	0.11383716	0.003091706	0	0.041657991	0	0.051227631	0.107743432	0.036516838	0.151444678	0.037850824	0.057291125	0	0.010937004	0
TCGA-MP-A5C7-01	0.132906661	0	0.12494728	0.050142846	0.144384043	0	0.140602654	0	0	0	0.052787731	0.018791485	0.025885476	0.032751142	0.128079331	0.067426212	0.046466274	0.034828865	0	0
TCGA-NJ-A4YF-01	0.001558784	0	0.140259817	0.062797153	0.110001812	0.019267286	0.144367776	0.042717311	0	0.035123158	0.010977145	0.051244418	0.169917879	0.046394569	0.149959763	0	0	0	0.008918437	0
TCGA-NJ-A4YG-01	0	0.049195517	0.060658599	0.070878836	0.133068516	0	0.100175019	0.030165065	0	0.002689156	0.015021171	0.044611039	0.065253436	0.032986427	0.156090041	0.08087686	0.056112602	0.084157643	0	0
TCGA-NJ-A4YI-01	0.015166013	0	0.063349931	0	0.221971572	0	0.123634299	0.006935715	0	0.026517592	0.050497342	0.090285126	0.072985608	0.013417292	0.132351596	0.090632521	0.037174996	0.0298152	0	0
TCGA-NJ-A4YP-01	0	0.049609402	0.068882291	0.112236808	0.10541965	0.009003163	0.120343824	0.030232459	0	0.056392589	0	0.018781254	0.077778432	0.044355518	0.152696462	0.00044832	0.083681197	0.04696857	0	0
TCGA-NJ-A4YQ-01	0.014461697	0.083163272	0.129256955	0.140327479	0	0	0.15578639	0.016333172	0	0.008581546	0.033727194	0.011687328	0.106601561	0.064002501	0.155893969	0	0.011565706	0.066909283	0	0
TCGA-NJ-A55A-01	0	0.168413496	0.02578548	0.08498086	0.087709893	0	0.143714752	0.006875299	0.000372695	0	0.041754453	0.023214519	0.008659816	0.058857513	0.138859858	0.051749798	0.036291785	0.122759784	0	0
TCGA-NJ-A55O-01	0.047681254	0.018454757	0.026258072	0.029363759	0.187239795	0	0.093795505	0.016227709	0	0.040190893	0	0.019741643	0.138266621	0.043996898	0.248200423	0.007897143	0.045481153	0.023544633	0.004001354	0
TCGA-NJ-A55R-01	0.065450191	0.090684173	0.245506272	0	0.078681391	0	0.129594748	0	0	0	0.044811806	0	0.091706814	0.024870131	0.11736327	0	0.017509921	0.093821283	0	0
TCGA-NJ-A7XG-01	0	0.033191457	0.117988178	0.001031076	0.221750812	0	0.086446716	0.099846244	0	0.00212994	0.021109986	0.014505672	0	0.015138399	0.159886131	0.021193745	0.094721172	0.111060472	0	0
TCGA-O1-A52J-01	0.001299079	0	0.047764428	0.035382638	0.212165977	0	0.05612814	0.024219377	0	0.038695263	0.033151762	0.131096073	0.022877947	0.022699508	0.20283722	0.050813286	0.063531382	0.04342831	0	0
TCGA-S2-AA1A-01	0.107961102	0.003219463	0.17706972	0.085713112	0.134489157	0	0.117131406	0	0	5.30E-05	0.013212664	0.01573284	0.040172819	0.035503898	0.08274224	0.065398651	0.044463855	0.077136096	0	0

Neutrophils
0.02572446
0.01918684
0.036463928
0.034883016
0
0.044817996
0.026687415
0.0147002
0.024896436
0.047676892
0.026379059
0
0.054592347
0.024030966
0.004928301
0.096841639
0.010507657
0.025430283
0.002652191
0.068475522
0.011930233
0.029234783
0.046352435
0.029766623
0.029077731
0.02782413
0.024129804
0.034223939
0.058396017
0.032717916
0.021424178
0.015581524
0.018085014
0.011279098
0.027842778
0.016586696
0.05222782
0.015503004
0.023194984
0.011034783
0
0.048494703
0.027707314
0.025638814
0.00472647
0
0.01134634
0.009881094
0
0
0.014291371
0.048001273

0.021678061
0.031342691
0.06053729
0.051273345
0.027076939
0.009289548
0.00752497
0.033668157
0
0.047948739
0.010513517
0.042532655
0.011144729
0.035654672
0.005500046
0.030546918
0.020037966
0.002478444
0.024994081
0.018913648
0.030085314
0.002994291
0.021294683
0
0.023260208
0.008081449
0.022125059
0.003466484
0.028710621
0.00527633
0.010283452
0.025045883
0.027241379
0
0.003781399
0
0
0.009389565
0
0
0
0
0
0.004366524
0.000799727
0.027249812
0.033975018
0
0.015428094
0
0
0.014597009
0.02511715
0.015926387

0.008018451
0.014391291
0
0
0
0.014997516
0.010036476
0
0
0
0.01128587
0.011446871
0
0.000836842
0
0
0.004972475
0
0.019468434
0
0.008585733
0
0.013893606
0.000544699
0
0.003635298
0.028706265
0
0.027308899
0.034989296
0.016942238
0.015797544
0.002612055
0.002558391
0
0
0.025518434
0.03165558
0
0.018610737
0
0
0.015822379
0
0.01557853
0
0.003631701
0.038268143
0
0
0.013585645
0.036076647
0
0

0
0.051611827
0.025859725
0
0.006692074
0
0.016817391
0.02367549
0
0.02620357
0.002106856
0.007294527
0
0
0.018111592
0.005888797
0.052426944
0.023995577
0.009948983
0.016062102
0.006501975
0
0.032446596
0.018937715
0
0
0.010219217
0.005431727
0.062929294
0.011191002
0.02438824
0.020962518
0.000310543
0.027466888
0.019511934
0
0.002095333
0.028321829
0
0.036006374
0
0.029350035
0
0.009506434
0.010440543
0
0.011203252
0.037006086
0
0
0.010506171
0
0.010930336
0.046404122

0.010546359
0.009441202
0.014846059
0.038638071
0.021090059
0
0.002701383
0.036970315
0.018062618
0.051555156
0.052711114
0.007465734
0
0.082282443
0.01688936
0.017288676
0.022867854
0.01478885
0.014325991
0.044353163
0.002427284
0.015216692
0
0.035378339
0
0
0
0
0
0.033124068
0
0
0.028402148
0
0
0
0.025038425
0.035346284
0.037470767
0
0.020321323
0
0
0.061582632
0.004895902
0.001854651
0.015254112
0.03763738
0
0.004474495
0
0.02893827
0.05545731
0.001740647

0.006034665
0.040286349
0.031370556
0
0
0
0.018851367
0.026068173
0.014553164
0.009391014
0.017546926
0.031906232
0.030745018
0.015318493
0
0
0.007010267
0.023762951
0.014001822
0
0.028911324
0.024713043
5.32E-05
0.013621836
0.011078819
0.00541835
0.040606038
0.023207703
0.001239368
0.025162106
0.008210191
0.030358898
0.010024645
0.038878638
0.05869923
0.00012598
0.015432699
0.026716404
0.047373906
0.028371061
0.02891012
0.042170796
0
0.001237291
0.001356532
0.047348058
0.010624875
0.022044001
0.016045394
0.019297254
0.060760023
0
0.004237438
0

0.021184292
0.001898946
0
0.000703505
0.001369574
0.022730393
0.009099606
0
0.014514692
0
0.014992065
0.004363858
0.054473807
0.013528266
0.040415451
0
0
0
0
0.010926381
0.005869094
0.026210223
0
0
0
0
0.011914606
0
0.010685723
0.006841886
0
0
0.052106915
0.006988293
0
0.033514628
0.003580922
0.026160944
0.079442194
0.012721045
0.032389284
0.025805246
0.016399787
0
0.027123821
0.028948583
0.023496307
0.006339164
0.039748582
0.035516779
0.031709171
0.00968737
0
0

0
0.070733071
0
0
0.018337046
0
0
0.004016952
0.028506656
0
0
0.01987548
0
0
0.042054467
0.020653715
0.040943649
0.03361859
0
0.032613625
0
0.042734218
0.015527172
0.028857988
0.01229378
0.04594398
0.005139045
0.007520185
0
0.013878721
0
0
0.013265625
0
0
0.026094874
0
0
0
0.023740056
0
0.004446709
0.021677648
0
0.013048367
0
0.033179852
0.034083534
0.002117601
0.026307668
0
0.003296019
0.047988102
0.016663373

0.017400335
0.011403179
0
0
0
0
0.048661941
0.062977691
0
0.021912166
0
0.023154545
0.012663909
0
9.34E-05
0.010882
0
0.020997071
0.029119169
0.002228751
0
0.01238444
0.007517289
0.011221908
0.020400725
0
0.028722844
0.075282561
0.026163519
0
0
0
0.012673922
0
0.002163949
0.023959509
0.051401985
0
0
0
0
0.010911112
0.00734126
0
0
0.022065496
0.00716476
0.005298552
0.022281674
0.021649127
0.012492058
0
0.020533343
0

0.069722421
0.031464394
0
0.014398879
0.018859827
0
0
0.006494693
0.018060074
0.025265197
0.023170063
0.001701946
0
0.009658389
0
0
0.01390961
0
