# Supplementary material for: Insight of a Metabolic Prognostic Model to Identify Tumor Environment and Drug Vulnerability for Lung Adenocarcinoma
Source: Front Immunol. 2022 Jun 23;13:872910. doi: 10.3389/fimmu.2022.872910 (PMC9262104; doi:10.3389/fimmu.2022.872910)
Supplement: Supplementary file 11 [file DataSheet_10.pdf]

Supplementary Table S10: Immune Score, Tumor Purity, and Metabolism Score of LUAD.

ID	ImmuneScore	TumorPurity	olism Score（risk s	risk
TCGA-49-AARQ-01	616.6128766	0.861455485	0.265302333	low
TCGA-91-8497-01	1618.161217	0.613620092	0.293832841	low
TCGA-62-8397-01	894.7539392	0.764057261	0.334459437	low
TCGA-86-A4P8-01	2463.766886	0.452790173	0.360876662	low
TCGA-55-8206-01	1880.877447	0.600148179	0.369985887	low
TCGA-L9-A444-01	2244.971092	0.521291678	0.374923982	low
TCGA-78-7539-01	1513.197316	0.709984156	0.386354372	low
TCGA-67-4679-01	1172.323599	0.6759943	0.388755108	low
TCGA-55-A4DG-01	904.0592	0.788331957	0.396874565	low
TCGA-MP-A4TH-01	1735.206877	0.640604637	0.402765429	low
TCGA-97-8552-01	1423.382516	0.692597271	0.406448335	low
TCGA-97-8172-01	1641.478283	0.5708357	0.415004868	low
TCGA-55-8087-01	284.5686791	0.829940321	0.416361053	low
TCGA-95-7948-01	139.0993081	0.85715607	0.427290035	low
TCGA-86-6851-01	2244.649089	0.557639129	0.447972336	low
TCGA-69-7764-01	331.556862	0.829103328	0.450580046	low
TCGA-86-8056-01	817.2866821	0.757765951	0.46728579	low
TCGA-73-7498-01	686.395262	0.743010152	0.47152296	low
TCGA-MP-A4T6-01	842.5419335	0.848352764	0.488618474	low
TCGA-78-7537-01	244.1612446	0.80305276	0.516169482	low
TCGA-97-A4M1-01	1147.375354	0.721830888	0.538091131	low
TCGA-55-8207-01	1217.734528	0.605407891	0.547204	low
TCGA-91-8496-01	1771.360172	0.649344803	0.548537556	low
TCGA-55-7573-01	1275.155714	0.683532553	0.548709021	low
TCGA-93-7348-01	737.1894109	0.714104765	0.549892172	low
TCGA-93-A4JP-01	1174.471215	0.673471883	0.557512182	low
TCGA-97-A4M0-01	1549.814944	0.710374908	0.558212908	low
TCGA-S2-AA1A-01	1915.037319	0.54525127	0.561354988	low
TCGA-97-A4M2-01	2083.45061	0.576372812	0.569009866	low
TCGA-55-8512-01	215.3732278	0.852556837	0.571996161	low
TCGA-55-8507-01	282.9770984	0.823946209	0.573374342	low
TCGA-MP-A5C7-01	-551.8691914	0.93898939	0.585726768	low
TCGA-86-8671-01	2901.819461	0.32904623	0.586861312	low
TCGA-99-AA5R-01	2311.121879	0.477605271	0.5870845	low
TCGA-55-A492-01	173.608905	0.869887906	0.589456572	low
TCGA-86-8669-01	744.2158511	0.79157876	0.593252125	low
TCGA-55-A491-01	1166.088734	0.700820831	0.594602429	low
TCGA-05-4405-01	845.9714568	0.65435698	0.616744398	low
TCGA-97-A4M7-01	1685.385907	0.618402796	0.621502186	low
TCGA-O1-A52J-01	1264.285122	0.733750964	0.622967252	low
TCGA-55-8097-01	661.1315468	0.769521099	0.623909754	low
TCGA-97-8179-01	238.5343826	0.819514692	0.626753761	low
TCGA-44-5645-01	1679.204353	0.635818315	0.629360642	low
TCGA-NJ-A7XG-01	-281.5743481	0.951378755	0.635502939	low
TCGA-97-A4M6-01	1742.543872	0.615071194	0.637426662	low
TCGA-78-7167-01	-379.3192467	0.905260874	0.639698267	low
TCGA-86-A4P7-01	1482.666095	0.628294725	0.639722261	low
TCGA-97-A4LX-01	2310.112891	0.45226751	0.641152754	low
TCGA-93-A4JQ-01	1753.711799	0.556498845	0.642881522	low
TCGA-75-7025-01	1211.199076	0.670996086	0.64740512	low
TCGA-97-8177-01	1680.916171	0.555006546	0.647637784	low
TCGA-78-8648-01	2469.333591	0.313521773	0.648964975	low

TCGA-L4-A4E6-01	2306.461249	0.470742582	0.649636996	low
TCGA-78-7163-01	-203.8450459	0.957444639	0.652649113	low
TCGA-49-AARR-01	1278.559881	0.654731016	0.663132049	low
TCGA-44-2665-01	1324.71304	0.524237651	0.665884838	low
TCGA-67-6217-01	1250.704901	0.699432361	0.675600565	low
TCGA-91-A4BD-01	1483.403473	0.752395112	0.682473627	low
TCGA-62-8395-01	176.1383354	0.782005334	0.683494129	low
TCGA-05-5715-01	944.4736706	0.688869064	0.692051482	low
TCGA-86-8278-01	731.6595856	0.719043676	0.69880487	low
TCGA-NJ-A4YG-01	1075.412692	0.709775223	0.702109648	low
TCGA-86-8358-01	-261.6933434	0.88874886	0.702384826	low
TCGA-97-7938-01	386.6502297	0.798059022	0.709478814	low
TCGA-55-6972-01	-889.007508	0.982488281	0.714087468	low
TCGA-91-6835-01	2380.674679	0.466881953	0.716522861	low
TCGA-78-7161-01	-558.0660761	0.891687489	0.72035694	low
TCGA-78-8640-01	495.488187	0.850227998	0.725622862	low
TCGA-NJ-A4YF-01	-167.9680754	0.89484384	0.730966538	low
TCGA-78-8662-01	-445.3152291	0.92652844	0.733744207	low
TCGA-50-6673-01	553.4649725	0.748268918	0.738678495	low
TCGA-J2-A4AE-01	1056.886822	0.782884931	0.739467257	low
TCGA-50-5068-01	2161.024512	0.54200384	0.745072043	low
TCGA-50-8457-01	1705.336747	0.579272409	0.745859787	low
TCGA-93-A4JN-01	727.363723	0.740278358	0.747972423	low
TCGA-64-5778-01	1863.114154	0.696849953	0.749476416	low
TCGA-L9-A50W-01	673.0406527	0.802731028	0.751676264	low
TCGA-55-8615-01	-270.794806	0.896609356	0.761054691	low
TCGA-44-3918-01	2302.377501	0.450677943	0.761321577	low
TCGA-44-5643-01	412.621725	0.881151809	0.76395858	low
TCGA-91-A4BC-01	2277.299649	0.470875361	0.764627646	low
TCGA-95-8039-01	1381.611026	0.698490274	0.767363238	low
TCGA-91-6828-01	1558.810143	0.613308171	0.773166421	low
TCGA-55-A48Z-01	791.2113222	0.693987182	0.773236339	low
TCGA-44-7659-01	614.8091455	0.820595255	0.784495626	low
TCGA-55-8208-01	2490.727547	0.407620386	0.785449847	low
TCGA-50-5045-01	2416.629241	0.390468722	0.785650025	low
TCGA-55-6971-01	2186.187502	0.529608774	0.786563262	low
TCGA-97-8547-01	815.9652016	0.651162371	0.790287394	low
TCGA-86-8668-01	1089.449132	0.644752834	0.791645396	low
TCGA-MN-A4N5-01	911.5676427	0.800273454	0.792444954	low
TCGA-97-8174-01	1072.821137	0.641799616	0.794081634	low
TCGA-62-A46V-01	185.82515	0.797552555	0.795799713	low
TCGA-44-A4SU-01	561.0349112	0.792379961	0.796621377	low
TCGA-97-7547-01	617.8741187	0.762933222	0.79835846	low
TCGA-55-7728-01	2422.988506	0.596526718	0.806172878	low
TCGA-78-7155-01	-637.1024655	0.944501939	0.808639941	low
TCGA-97-8175-01	1119.769394	0.753473662	0.814089459	low
TCGA-MP-A4TD-01	764.7152167	0.677269318	0.814593314	low
TCGA-50-5942-01	502.3828508	0.781044127	0.816396064	low
TCGA-NJ-A4YI-01	935.4643302	0.728817136	0.818176612	low
TCGA-97-A4M5-01	1139.786627	0.668016991	0.818223364	low
TCGA-53-A4EZ-01	-158.4426755	0.913708212	0.828506705	low
TCGA-50-5946-01	-557.5692816	0.935201517	0.833308225	low
TCGA-86-8280-01	1599.238171	0.598164094	0.83734788	low
TCGA-55-A48X-01	1495.016203	0.669456163	0.842449588	low

TCGA-55-8614-01	236.7483236	0.80013532	0.846984773	low
TCGA-55-8091-01	1378.119457	0.581586175	0.851264267	low
TCGA-MN-A4N4-01	503.3434071	0.724624001	0.855176649	low
TCGA-93-7347-01	2211.2945	0.490701203	0.85611221	low
TCGA-NJ-A55A-01	1418.459114	0.665770897	0.85622436	low
TCGA-50-5935-01	938.6642648	0.733007487	0.858492179	low
TCGA-55-6642-01	1015.874737	0.582053825	0.859695436	low
TCGA-69-7980-01	1096.087411	0.705154374	0.863515134	low
TCGA-69-7979-01	-180.6665753	0.852858508	0.865011696	low
TCGA-99-8028-01	2753.642201	0.361763058	0.866063969	low
TCGA-69-8254-01	1025.723431	0.782067978	0.8712116	low
TCGA-44-6148-01	689.567341	0.716054589	0.87145809	low
TCGA-97-7546-01	1221.5428	0.636200316	0.877639349	low
TCGA-75-6206-01	648.9895295	0.750188445	0.878850611	low
TCGA-55-8619-01	2148.498275	0.540212535	0.879288706	low
TCGA-86-7954-01	1722.45334	0.614405591	0.879352339	low
TCGA-75-5147-01	1108.130349	0.713661338	0.881844552	low
TCGA-49-AARN-01	560.1621268	0.803129774	0.885169154	low
TCGA-49-4512-01	946.0784259	0.657983474	0.886694718	low
TCGA-93-A4JO-01	1896.872921	0.594544365	0.886708425	low
TCGA-55-8620-01	434.2722772	0.868881816	0.887820706	low
TCGA-69-8453-01	2185.47316	0.550624159	0.888399697	low
TCGA-55-6987-01	2589.957252	0.488525114	0.88921016	low
TCGA-86-7714-01	860.8760101	0.749655852	0.897696605	low
TCGA-97-7552-01	2265.337209	0.554248635	0.898683489	low
TCGA-55-8513-01	1752.755951	0.592819299	0.900405506	low
TCGA-55-7574-01	1928.038363	0.507327407	0.901182436	low
TCGA-L9-A743-01	1925.280977	0.560355517	0.907222132	low
TCGA-L9-A8F4-01	1506.331932	0.668344908	0.916114502	low
TCGA-44-6776-01	-273.2382784	0.907529609	0.925131801	low
TCGA-05-4390-01	77.89915281	0.805171939	0.926323301	low
TCGA-73-7499-01	1676.7718	0.67029735	0.927499917	low
TCGA-05-4417-01	1580.712864	0.517716343	0.931738402	low
TCGA-55-8621-01	2113.280111	0.489725754	0.939745314	low
TCGA-67-6215-01	693.3171108	0.833219708	0.940980598	low
TCGA-MP-A4SW-01	1281.792532	0.707267904	0.943219119	low
TCGA-44-A47B-01	800.3635095	0.771009035	0.950282576	low
TCGA-05-5423-01	1819.144102	0.63299313	0.963559479	low
TCGA-97-7553-01	2373.40177	0.47846611	0.969852465	low
TCGA-86-8054-01	-619.5545564	0.915129829	0.971256356	low
TCGA-78-7143-01	728.595379	0.832892509	0.973989985	low
TCGA-50-5055-01	2733.351906	0.371574461	0.976016831	low
TCGA-80-5608-01	285.0616405	0.865417622	0.976088245	low
TCGA-53-7626-01	2013.52655	0.54311967	0.978817937	low
TCGA-97-7554-01	838.1602453	0.635029853	0.979096952	low
TCGA-50-5944-01	563.1459861	0.708653811	0.979749398	low
TCGA-78-8655-01	1264.401854	0.701982537	0.985431292	low
TCGA-55-8510-01	1828.471473	0.571209923	0.98862852	low
TCGA-91-6840-01	645.4097941	0.797255327	0.993257935	low
TCGA-86-A456-01	1205.631442	0.696041538	0.996046043	low
TCGA-86-7713-01	-566.8093658	0.9365165	0.996172159	low
TCGA-44-5644-01	-639.4158479	0.941873334	1.001736445	low
TCGA-53-7813-01	322.8240889	0.871738824	1.005482687	low
TCGA-55-8506-01	812.0294486	0.756466747	1.005730538	low

TCGA-55-8096-01	1210.423889	0.604774221	1.012389222	low
TCGA-95-7562-01	681.635485	0.775550216	1.01533589	low
TCGA-55-A57B-01	918.2177468	0.713482343	1.015570091	low
TCGA-44-6774-01	1020.334587	0.513253218	1.015854628	low
TCGA-78-7149-01	-146.5122999	0.854537263	1.01843221	low
TCGA-50-5932-01	-20.35561823	0.879113535	1.025218381	low
TCGA-44-A47G-01	2127.504983	0.504592282	1.025904208	low
TCGA-97-8171-01	-403.2278024	0.935661023	1.026684626	low
TCGA-62-A46Y-01	1482.571648	0.71513791	1.027603342	low
TCGA-97-7937-01	-37.44728687	0.849499781	1.029395076	low
TCGA-55-7816-01	1700.195458	0.455916841	1.03459385	low
TCGA-38-A44F-01	1873.995873	0.587529067	1.036541376	low
TCGA-NJ-A55O-01	922.3673257	0.770768155	1.039942903	low
TCGA-50-8459-01	1989.965972	0.399100256	1.044517226	low
TCGA-38-7271-01	2741.133481	0.378878716	1.044866161	low
TCGA-50-8460-01	1474.901165	0.671283039	1.049713686	low
TCGA-05-4384-01	767.8386104	0.760444147	1.052003682	low
TCGA-62-A46U-01	2531.80932	0.560322726	1.066526654	low
TCGA-MP-A4TE-01	-790.667127	0.952898867	1.067298428	low
TCGA-MP-A4TJ-01	2444.295322	0.455632089	1.0736944	low
TCGA-05-4427-01	823.4281048	0.740269774	1.082018148	low
TCGA-J2-8192-01	1557.622844	0.4958827	1.083889971	low
TCGA-44-4112-01	627.3224553	0.712985011	1.085514875	low
TCGA-55-6543-01	1029.789945	0.727183544	1.089932945	low
TCGA-50-6591-01	-1352.992645	0.963231632	1.094873563	low
TCGA-86-8359-01	1165.972559	0.750432887	1.108109713	low
TCGA-44-6777-01	2263.496427	0.335501982	1.110142452	low
TCGA-MP-A4T9-01	1079.986641	0.692102555	1.111876314	low
TCGA-44-2668-01	1860.399745	0.524314274	1.124002314	low
TCGA-NJ-A55R-01	-188.1990045	0.871116987	1.129549198	low
TCGA-55-7910-01	-30.77703876	0.861616589	1.130479683	low
TCGA-50-5066-01	2206.478985	0.486269896	1.140930953	low
TCGA-86-8076-01	1858.114278	0.631220971	1.144734399	low
TCGA-44-A47A-01	1395.106165	0.713852781	1.152837062	low
TCGA-49-AAR0-01	1251.765812	0.723076057	1.15350818	low
TCGA-55-6985-01	1332.433815	0.640858898	1.160208742	low
TCGA-71-6725-01	141.5949404	0.884138912	1.162627335	low
TCGA-97-7941-01	697.5259016	0.759400289	1.167957614	low
TCGA-62-8402-01	1312.524587	0.777956409	1.169492045	low
TCGA-L9-A443-01	474.2962412	0.763143811	1.171200566	low
TCGA-86-8073-01	529.7622104	0.743985285	1.171312422	low
TCGA-44-2666-01	136.2130139	0.867672786	1.171584796	low
TCGA-67-6216-01	1314.82391	0.732668238	1.175297838	low
TCGA-55-8508-01	727.8733327	0.765522865	1.177202528	low
TCGA-55-8301-01	2124.079577	0.532197034	1.188273512	low
TCGA-55-7227-01	1529.237414	0.593297864	1.192157991	low
TCGA-50-7109-01	684.2550201	0.752996593	1.192229565	low
TCGA-35-5375-01	1039.174804	0.748672399	1.192289977	low
TCGA-91-7771-01	1632.790418	0.590087479	1.193445265	low
TCGA-55-8203-01	935.1636775	0.743776242	1.196137401	low
TCGA-55-8616-01	279.2269861	0.833922824	1.197491037	low
TCGA-55-7911-01	1627.717509	0.693451953	1.200239187	low
TCGA-91-6847-01	-973.0139553	0.980385604	1.202532616	low
TCGA-38-6178-01	409.4986572	0.758354274	1.206931688	low

TCGA-44-6778-01	2423.843802	0.492974699	1.208680427	low
TCGA-49-AAQV-01	893.2728647	0.806172871	1.208817328	low
TCGA-49-4487-01	1749.005293	0.591436158	1.210356611	low
TCGA-75-5146-01	1050.95837	0.747860277	1.234511034	low
TCGA-44-6775-01	1480.922779	0.522049847	1.245512288	low
TCGA-95-A4VP-01	984.2602032	0.719049889	1.245879113	low
TCGA-MP-A4TK-01	1477.89584	0.534707762	1.24678786	low
TCGA-55-8090-01	771.8134782	0.756949703	1.258256006	low
TCGA-L9-A7SV-01	-240.1149309	0.915162057	1.2624201	high
TCGA-05-5428-01	831.3463504	0.737818043	1.263676725	high
TCGA-62-A46S-01	642.3378997	0.807182262	1.264402047	high
TCGA-55-8085-01	1306.950242	0.725035516	1.264549038	high
TCGA-49-AAR4-01	1932.081351	0.642018133	1.268964627	high
TCGA-69-8253-01	379.8748265	0.847373752	1.27575063	high
TCGA-91-8499-01	400.3517667	0.829407261	1.275801984	high
TCGA-55-8514-01	639.7516623	0.807696048	1.276774419	high
TCGA-62-A46R-01	1204.542308	0.719133946	1.278189258	high
TCGA-86-8279-01	74.82751241	0.7951917	1.28435347	high
TCGA-05-5425-01	1752.412845	0.63204056	1.287603418	high
TCGA-55-6982-01	1098.041999	0.622483869	1.291281487	high
TCGA-49-AARE-01	487.6244503	0.80112074	1.293505078	high
TCGA-95-7947-01	621.1668174	0.842314609	1.294331385	high
TCGA-73-4658-01	1917.137248	0.469004943	1.296242026	high
TCGA-55-7725-01	1555.71416	0.702168422	1.300636451	high
TCGA-62-8394-01	375.3143937	0.8340733	1.30457973	high
TCGA-86-8673-01	521.2706907	0.789226874	1.306213189	high
TCGA-69-7765-01	1079.231862	0.603548726	1.307270535	high
TCGA-78-8660-01	1660.529277	0.672232062	1.324476888	high
TCGA-J2-8194-01	630.8750584	0.754862791	1.327243488	high
TCGA-55-8092-01	1471.1728	0.664897719	1.329106775	high
TCGA-86-7953-01	1305.887254	0.69560445	1.338559509	high
TCGA-50-5930-01	1170.984159	0.661482343	1.33920875	high
TCGA-86-8075-01	912.1675294	0.622482948	1.340187961	high
TCGA-MP-A4T4-01	1940.171487	0.545434744	1.344789183	high
TCGA-49-AAR2-01	813.2789605	0.821504883	1.348169936	high
TCGA-91-6836-01	-39.89491882	0.90211541	1.354348718	high
TCGA-L4-A4E5-01	27.16523517	0.874958039	1.359093228	high
TCGA-44-A479-01	2043.664338	0.503001498	1.359197645	high
TCGA-69-7763-01	559.8900496	0.709497956	1.362669152	high
TCGA-50-5941-01	2172.288516	0.534036651	1.372915976	high
TCGA-78-7162-01	1048.009348	0.70136613	1.375879149	high
TCGA-78-7633-01	-336.5113047	0.89607068	1.38982139	high
TCGA-J2-A4AG-01	1380.475986	0.654901101	1.394301628	high
TCGA-78-7158-01	70.03087297	0.877823848	1.394304666	high
TCGA-78-7159-01	105.237657	0.821321818	1.415097872	high
TCGA-55-7913-01	-332.5656402	0.944394367	1.419496109	high
TCGA-MN-A4N1-01	-246.9377481	0.891310386	1.423290527	high
TCGA-50-6593-01	1117.06786	0.635944389	1.42766694	high
TCGA-55-7284-01	1150.200853	0.693922392	1.431804767	high
TCGA-99-7458-01	1603.212789	0.583097257	1.437241067	high
TCGA-64-5779-01	726.3606116	0.721564837	1.445561909	high
TCGA-49-AARO-01	1746.629613	0.58518466	1.456841957	high
TCGA-95-A4VN-01	1742.522375	0.634980401	1.457177627	high
TCGA-55-8511-01	1366.481014	0.632308637	1.463519872	high

TCGA-64-1679-01	877.9172642	0.629409269	1.471962642	high
TCGA-55-6980-01	1569.434754	0.587637847	1.472880859	high
TCGA-91-6829-01	334.8486414	0.681938194	1.481783313	high
TCGA-50-5049-01	2843.355929	0.303035036	1.51370855	high
TCGA-55-7995-01	1932.465908	0.638251374	1.52737692	high
TCGA-55-7815-01	1254.993603	0.608491792	1.533659629	high
TCGA-44-A4SS-01	1622.554029	0.608522959	1.547369015	high
TCGA-86-8074-01	866.8716787	0.677762707	1.548047628	high
TCGA-62-8399-01	107.953951	0.819521484	1.548698001	high
TCGA-44-7662-01	1013.489952	0.606475478	1.550082368	high
TCGA-55-6979-01	2372.193508	0.457274485	1.558158104	high
TCGA-MP-A4SV-01	1141.672836	0.719291576	1.567920784	high
TCGA-44-7672-01	2093.703135	0.486886253	1.568253459	high
TCGA-55-6970-01	1165.658987	0.724580264	1.568767094	high
TCGA-44-8117-01	287.1674808	0.784690857	1.570062058	high
TCGA-71-8520-01	310.7684294	0.755591091	1.573496239	high
TCGA-55-8204-01	834.8314437	0.760241535	1.579315074	high
TCGA-78-7153-01	31.77141541	0.879420064	1.586934339	high
TCGA-38-4631-01	255.2941323	0.8672407	1.587539724	high
TCGA-78-7152-01	980.1813663	0.760593582	1.589675081	high
TCGA-4B-A93V-01	387.2695104	0.86274162	1.593937907	high
TCGA-55-7903-01	827.4310918	0.806879647	1.595237721	high
TCGA-05-4424-01	1169.414541	0.658861839	1.602870958	high
TCGA-86-8585-01	1646.545443	0.675325164	1.613855348	high
TCGA-62-A470-01	134.300063	0.866945126	1.619364631	high
TCGA-55-7281-01	1658.899666	0.589447449	1.627599249	high
TCGA-49-4488-01	952.4198798	0.786324377	1.63749656	high
TCGA-49-6744-01	1971.256146	0.498174907	1.640749864	high
TCGA-MP-A4T8-01	-248.8727036	0.847539344	1.644260098	high
TCGA-55-6969-01	1552.935565	0.585468565	1.656511555	high
TCGA-78-7220-01	-428.4891461	0.915944694	1.6667249	high
TCGA-55-7727-01	1010.069681	0.767897402	1.668172546	high
TCGA-MP-A4T7-01	759.4650908	0.816477017	1.674103281	high
TCGA-49-6743-01	706.7509593	0.723671913	1.687584406	high
TCGA-99-8025-01	125.483812	0.812891582	1.688040258	high
TCGA-55-5899-01	547.2618049	0.755101819	1.689619641	high
TCGA-50-5933-01	1452.510687	0.517120581	1.691477798	high
TCGA-55-7907-01	1114.0104	0.694327531	1.691528421	high
TCGA-55-7726-01	645.7454293	0.665887902	1.695455914	high
TCGA-MP-A4TI-01	2585.7854	0.401404806	1.702143511	high
TCGA-69-7978-01	2203.114184	0.483365658	1.707285593	high
TCGA-69-A59K-01	760.3401397	0.786641208	1.713699623	high
TCGA-69-7761-01	1821.87354	0.599936748	1.716645479	high
TCGA-99-8032-01	342.5838107	0.745305496	1.738173043	high
TCGA-50-5051-01	-203.6717623	0.894212676	1.755346569	high
TCGA-69-7760-01	-456.1916214	0.905328887	1.775257784	high
TCGA-49-6767-01	1054.008967	0.771474244	1.777206544	high
TCGA-91-6849-01	1207.267679	0.716082885	1.801206678	high
TCGA-75-6212-01	1538.88502	0.674235587	1.813084207	high
TCGA-62-A46P-01	-46.52780371	0.873954437	1.815895652	high
TCGA-55-6986-01	794.6087414	0.787100037	1.822475769	high
TCGA-NJ-A4YQ-01	2020.119464	0.627641275	1.825071539	high
TCGA-44-7667-01	-312.6773831	0.893785368	1.825544047	high
TCGA-55-6978-01	2102.647664	0.439183246	1.828827655	high

TCGA-80-5611-01	1562.95977	0.708718249	1.84880242	high
TCGA-44-7670-01	-287.5941212	0.927550269	1.851657123	high
TCGA-86-A4D0-01	-278.2663108	0.92741119	1.855622362	high
TCGA-69-7973-01	-141.4734998	0.849981694	1.871404068	high
TCGA-97-A4M3-01	-48.72401126	0.83434793	1.879483274	high
TCGA-44-6145-01	1889.317611	0.554440974	1.885357312	high
TCGA-55-6981-01	377.9075813	0.80701665	1.895927559	high
TCGA-91-6848-01	1539.898687	0.55693368	1.896982089	high
TCGA-55-7283-01	769.2450715	0.784968019	1.900483237	high
TCGA-91-6830-01	1121.709181	0.647144103	1.912816612	high
TCGA-55-A494-01	-1012.869765	0.965795716	1.918814013	high
TCGA-05-4396-01	-203.8758778	0.891347888	1.93069884	high
TCGA-44-7671-01	-184.7049583	0.834332253	1.934928826	high
TCGA-38-4632-01	1530.961139	0.66833257	1.942870351	high
TCGA-55-A490-01	682.9618768	0.738709133	1.947181505	high
TCGA-83-5908-01	1829.413238	0.617883227	1.94894298	high
TCGA-86-8674-01	-505.3691259	0.93300732	1.951567566	high
TCGA-44-2656-01	2173.988287	0.513565199	1.970795892	high
TCGA-50-6590-01	1746.015893	0.612999847	1.978202068	high
TCGA-55-7724-01	1534.546088	0.598423392	1.985431157	high
TCGA-MP-A4TF-01	188.8629076	0.901120954	2.005509984	high
TCGA-69-8255-01	1348.84926	0.790995659	2.011557931	high
TCGA-55-6983-01	1311.236988	0.656804965	2.026190464	high
TCGA-86-6562-01	578.0371914	0.702003433	2.031544227	high
TCGA-44-8120-01	558.4790534	0.737338266	2.031629321	high
TCGA-55-A48Y-01	421.5214831	0.736405277	2.057955627	high
TCGA-55-7914-01	754.9015028	0.767234731	2.06532757	high
TCGA-93-8067-01	45.95444573	0.874397524	2.072454386	high
TCGA-MP-A4SY-01	460.4927302	0.732993904	2.077888976	high
TCGA-95-7039-01	327.2507982	0.795546567	2.092528996	high
TCGA-64-5781-01	1367.470778	0.691791062	2.101352327	high
TCGA-95-7944-01	1834.307837	0.638789448	2.104814471	high
TCGA-91-6831-01	676.0126709	0.699738746	2.11150434	high
TCGA-78-7147-01	401.6592524	0.871310458	2.132832545	high
TCGA-64-5815-01	1464.582529	0.511561433	2.135594705	high
TCGA-95-A4VK-01	707.0142622	0.806407716	2.147399998	high
TCGA-55-A4DF-01	1137.134379	0.758915483	2.151265905	high
TCGA-NJ-A4YP-01	593.9267206	0.695498375	2.165937979	high
TCGA-64-1681-01	947.3190534	0.725736302	2.168230869	high
TCGA-55-7576-01	812.9043594	0.733604655	2.208103349	high
TCGA-62-A472-01	859.4128904	0.807149197	2.217286161	high
TCGA-55-8299-01	2094.790142	0.452608552	2.224878194	high
TCGA-55-8505-01	-272.5239668	0.832809184	2.228057878	high
TCGA-55-8302-01	759.9046941	0.799862035	2.232892133	high
TCGA-49-4490-01	422.3084256	0.779312242	2.24690131	high
TCGA-75-6214-01	-90.45389329	0.909835268	2.26263759	high
TCGA-55-A493-01	1602.714309	0.657899612	2.263477866	high
TCGA-J2-A4AD-01	-398.3267761	0.88545785	2.282424225	high
TCGA-86-8055-01	898.4117765	0.608028156	2.284075637	high
TCGA-05-4425-01	1599.590883	0.618126638	2.289693168	high
TCGA-L9-A5IP-01	503.7836132	0.839516645	2.292889473	high
TCGA-44-6146-01	-207.7146707	0.906413182	2.303683623	high
TCGA-69-7974-01	1577.197892	0.620978173	2.314622346	high
TCGA-64-5774-01	-527.5913826	0.91056715	2.321820556	high

TCGA-78-7156-01	-227.6146851	0.901172449	2.33752114	high
TCGA-95-8494-01	867.3580855	0.797745423	2.347151007	high
TCGA-49-4514-01	736.6144602	0.828355979	2.360874042	high
TCGA-50-6597-01	1237.952025	0.752354336	2.377556972	high
TCGA-86-A4JF-01	1147.462763	0.756299472	2.377925241	high
TCGA-44-7661-01	1667.437405	0.560511495	2.409083212	high
TCGA-78-7145-01	431.9396815	0.786490546	2.434571094	high
TCGA-50-5931-01	-701.4442068	0.915057063	2.44733327	high
TCGA-78-7540-01	401.8298755	0.862501637	2.450986159	high
TCGA-55-6984-01	318.3822553	0.873435245	2.458420221	high
TCGA-49-6761-01	1071.626343	0.742843315	2.47974124	high
TCGA-86-7955-01	-932.7971066	0.978430877	2.500721518	high
TCGA-75-7027-01	-71.86965501	0.802985938	2.508710258	high
TCGA-62-A471-01	-185.8556096	0.933887283	2.514488481	high
TCGA-75-5125-01	1492.833011	0.651108604	2.518437667	high
TCGA-55-7994-01	1673.892425	0.699660582	2.566286828	high
TCGA-78-7148-01	420.8165277	0.834820701	2.594473221	high
TCGA-78-7160-01	1563.242898	0.608952611	2.602101124	high
TCGA-49-AAR3-01	2037.489383	0.54662017	2.671644588	high
TCGA-44-8119-01	726.5599723	0.660169053	2.681938329	high
TCGA-MP-A4TA-01	584.4760705	0.809328579	2.729547734	high
TCGA-78-7154-01	-165.2418135	0.878719933	2.736472386	high
TCGA-05-4433-01	1498.049011	0.71070906	2.782442585	high
TCGA-50-6594-01	378.7100796	0.81734396	2.80841214	high
TCGA-95-7043-01	-623.2100013	0.914396228	2.831648188	high
TCGA-55-8094-01	-1002.191292	0.985454893	2.936423211	high
TCGA-44-7669-01	737.2626391	0.771685187	2.973263261	high
TCGA-44-7660-01	255.7728437	0.865566935	2.994750843	high
TCGA-50-5936-01	578.1374338	0.757346748	2.995759072	high
TCGA-73-A9RS-01	-103.6429901	0.907366302	3.030729317	high
TCGA-55-8205-01	2211.421548	0.519310211	3.098079255	high
TCGA-62-A46O-01	-1096.639874	0.979545673	3.136703948	high
TCGA-86-7711-01	1245.748222	0.646941387	3.180550294	high
TCGA-53-7624-01	135.037548	0.885268846	3.183718859	high
TCGA-MP-A4TC-01	1139.379121	0.620953774	3.331661962	high
TCGA-95-7567-01	754.127197	0.75101549	3.42186807	high
TCGA-62-8398-01	695.1498679	0.782654301	3.450293519	high
TCGA-55-6968-01	1003.9598	0.743999428	3.508082371	high
TCGA-50-5044-01	1010.634151	0.714242331	3.578138766	high
TCGA-78-7536-01	653.6975406	0.810337932	3.661062338	high
TCGA-50-6595-01	1101.595067	0.60487285	3.66545671	high
TCGA-97-8176-01	528.0032627	0.817387234	3.777082193	high
TCGA-55-8089-01	2493.812708	0.493023476	3.804920283	high
TCGA-86-7701-01	1484.829612	0.61732549	3.850390581	high
TCGA-86-8672-01	1379.367933	0.641265372	3.880104086	high
TCGA-49-AAR9-01	-427.2432347	0.920486481	3.888140294	high
TCGA-78-7535-01	1203.362889	0.732617976	4.0383967	high
TCGA-49-6745-01	1535.085563	0.606764052	4.104876467	high
TCGA-05-5429-01	-65.59476111	0.866404634	4.230303045	high
TCGA-78-7166-01	187.6822231	0.891596681	4.296117034	high
TCGA-50-6592-01	1411.025554	0.655445037	4.307778405	high
TCGA-55-6975-01	321.5453521	0.712375813	4.329442771	high
TCGA-78-7146-01	239.6746391	0.86076482	4.354357241	high
TCGA-50-5939-01	1353.834188	0.644117444	4.385692999	high

TCGA-78-7542-01	335.1633686	0.901582379	4.608152604	high
TCGA-99-8033-01	1081.94306	0.75296128	4.668588695	high
TCGA-64-5775-01	407.973451	0.798551671	4.982189339	high
TCGA-55-6712-01	1723.237506	0.614895749	5.102625989	high
TCGA-73-4676-01	538.1780335	0.800644828	5.285918972	high
TCGA-55-7570-01	-759.17315	0.93163394	5.409757661	high
TCGA-78-7150-01	-405.8225332	0.859929821	5.856780989	high
TCGA-50-5072-01	87.48611106	0.829467976	6.003839502	high
TCGA-49-6742-01	-309.741646	0.91980254	7.119846837	high
TCGA-05-4415-01	53.72390672	0.901232667	7.315548522	high
TCGA-44-6779-01	1885.820733	0.563766255	7.914491481	high
