# Supplementary material for: Insight of a Metabolic Prognostic Model to Identify Tumor Environment and Drug Vulnerability for Lung Adenocarcinoma
Source: Front Immunol. 2022 Jun 23;13:872910. doi: 10.3389/fimmu.2022.872910 (PMC9262104; doi:10.3389/fimmu.2022.872910)
Supplement: Supplementary file 12 [file DataSheet_11.pdf]

Supplementary Table S11: The prediction of responder and non-responder to immunotherapy with TIDE and cohort (IMvigor210).

Patient	Responder	TIDE	IFNG	MSI Expr Sig	Merck18	CD274	CD8	CTL.flag	Dysfunction	Exclusion	MDSC	CAF	TAM M2	riskScore	risk
TCGA-55-7728-01	Responder	-4.25	0.04	0.42	0.4	0.31	0.26	FALSE	2.93	-4.25	-0.18	-0.15	-0.04	0.806172878	low
TCGA-97-A4M2-01	Responder	-4.07	0.69	0.72	0.78	0.43	1.12	FALSE	2.81	-4.07	-0.26	-0.09	0	0.569009866	low
TCGA-L4-A4E6-01	Responder	-3.37	0.43	0.5	0.54	0.16	-0.32	FALSE	2.42	-3.37	-0.28	0	-0.02	0.649636996	low
TCGA-55-8513-01	Responder	-3.18	-0.33	0.65	0.08	0.47	-1.24	FALSE	2.36	-3.18	-0.21	-0.05	-0.02	0.900405506	low
TCGA-05-4422-01	Responder	-3.04	1.17	0.86	0.91	-0.25	2.21	FALSE	-0.08	-3.04	-0.1	-0.18	0.01	0.956147237	low
TCGA-97-A4M0-01	Responder	-3.03	0.26	0.44	0.58	0.12	1.16	FALSE	1.19	-3.03	-0.12	-0.11	-0.03	0.558212908	low
TCGA-91-A4BD-01	Responder	-2.93	-0.5	0.96	-0.5	-2.22	-1.16	FALSE	0.12	-2.93	-0.11	-0.16	0.01	0.682473627	low
TCGA-J2-A4AE-01	Responder	-2.91	0.22	0.89	0.29	-0.03	0.25	FALSE	0.32	-2.91	-0.09	-0.14	-0.02	0.739467257	low
TCGA-55-8206-01	Responder	-2.75	-1.32	0.33	-0.36	-0.24	-1.48	FALSE	1.22	-2.75	-0.23	-0.04	0.04	0.369985887	low
TCGA-MP-A4T6-01	Responder	-2.75	-0.27	0.21	0.05	-0.82	-0.36	FALSE	1.54	-2.75	-0.07	-0.17	0	0.488618474	low
TCGA-86-A456-01	Responder	-2.42	0.08	0.65	0.18	0.67	-0.44	FALSE	0.66	-2.42	-0.12	-0.01	-0.08	0.996046043	low
TCGA-86-7954-01	Responder	-2.37	2.67	0.05	1.25	0.8	0.89	FALSE	0.86	-2.37	-0.08	-0.06	-0.07	0.879352339	low
TCGA-62-A46Y-01	Responder	-2.37	0.46	0.83	0.33	1.44	0.48	FALSE	0.41	-2.37	-0.09	-0.13	0.01	1.027603342	low
TCGA-64-1677-01	Responder	-2.29	1.38	0.94	0.55	0.75	0.63	TRUE	-2.29	0.28	0.14	-0.11	-0.01	0.857657006	low
TCGA-MP-A4TH-01	Responder	-2.25	0.12	0.39	0.64	-0.23	0.94	FALSE	2.72	-2.25	-0.18	-0.05	0.04	0.402765429	low
TCGA-69-8254-01	Responder	-2.24	0.26	0.53	0.39	0.45	-0.44	FALSE	1.06	-2.24	-0.1	-0.09	-0.01	0.8712116	low
TCGA-MP-A4SW-01	Responder	-2.22	-0.78	0.66	-0.13	-0.35	-0.6	FALSE	1.56	-2.22	-0.11	-0.08	0	0.943219119	low
TCGA-91-8496-01	Responder	-2.2	-0.95	0.97	-0.2	0.73	-0.09	FALSE	-0.53	-2.2	-0.16	-0.06	0.02	0.548537556	low
TCGA-55-8621-01	Responder	-2.14	1.14	0.7	1.06	1.82	0.21	FALSE	3.25	-2.14	-0.16	0.01	-0.03	0.939745314	low
TCGA-86-8056-01	Responder	-2.11	-0.77	0.44	-0.73	-0.03	-0.72	FALSE	-0.26	-2.11	-0.14	-0.06	0.01	0.46728579	low
TCGA-97-8552-01	Responder	-2.09	-0.68	0.61	0.06	-0.62	-0.74	FALSE	2.19	-2.09	-0.17	-0.05	0.04	0.406448335	low
TCGA-05-5423-01	Responder	-2	-0.01	0.94	0.06	-0.4	0.32	FALSE	-0.67	-2	-0.12	-0.07	0.01	0.963559479	low
TCGA-NJ-A55O-01	Responder	-1.96	-0.63	0.63	-0.35	0.84	-1.22	FALSE	0.88	-1.96	-0.09	-0.09	0.01	1.039942903	low
TCGA-91-8497-01	Responder	-1.94	-0.98	0.48	0.23	0.01	-0.32	FALSE	3.24	-1.94	-0.17	-0.04	0.04	0.293832841	low
TCGA-97-A4M6-01	Responder	-1.82	1.15	0.34	0.84	-0.13	0.15	FALSE	2.41	-1.82	-0.12	0	-0.03	0.637426662	low
TCGA-44-7659-01	Responder	-1.8	-1.46	0.8	-0.8	0.06	-1.15	FALSE	-0.07	-1.8	-0.1	-0.07	0.01	0.784495626	low
TCGA-O1-A52J-01	Responder	-1.76	-1.06	0.23	-0.15	-0.33	-1.33	FALSE	1.17	-1.76	-0.12	-0.04	0	0.622967252	low
TCGA-05-4384-01	Responder	-1.66	-1.46	0.52	-0.99	-0.99	-1.34	FALSE	0.28	-1.66	-0.15	-0.02	0.03	1.052003682	low
TCGA-44-5645-01	Responder	-1.62	-0.17	0.4	0.4	-0.17	-0.21	FALSE	1.53	-1.62	-0.15	-0.03	0.04	0.629360642	low
TCGA-97-A4M1-01	Responder	-1.55	-0.58	0.59	-0.29	-0.76	-0.22	FALSE	1.28	-1.55	-0.13	-0.05	0.05	0.538091131	low
TCGA-67-6216-01	Responder	-1.53	-0.67	0.89	0.11	0.72	0.29	FALSE	0.68	-1.53	-0.07	-0.06	0	1.175297838	low
TCGA-67-6217-01	Responder	-1.53	0.49	0.48	0.45	-0.15	1.09	FALSE	1.24	-1.53	-0.14	-0.02	0.03	0.675600565	low
TCGA-86-8669-01	Responder	-1.52	-0.68	0.16	-0.15	-0.18	-0.36	FALSE	0.03	-1.52	-0.08	-0.07	0.02	0.593252125	low
TCGA-38-4626-01	Responder	-1.48	-0.49	0.66	0.11	1.24	-0.15	FALSE	1.5	-1.48	-0.19	0.11	-0.04	0.795415761	low

TCGA-67-6215-01	Responder	-1.47	-1.34	0.82	-1.23	-1.21	-2.41	FALSE	-1.12	-1.47	-0.05	-0.11	0.03	0.940980598	low
TCGA-97-A4M7-01	Responder	-1.42	0.48	0.2	0.77	-0.04	0.21	FALSE	2.2	-1.42	-0.12	-0.01	0.01	0.621502186	low
TCGA-93-A4JP-01	Responder	-1.36	0.62	0.41	0.18	0.42	-1.29	FALSE	0.72	-1.36	-0.08	-0.03	0	0.557512182	low
TCGA-73-4662-01	Responder	-1.35	-0.17	0.67	0.32	1.33	0.57	FALSE	0.5	-1.35	-0.1	-0.02	0	0.70754073	low
TCGA-67-3774-01	Responder	-1.31	0.38	0.69	0.39	-0.34	-0.17	FALSE	1.22	-1.31	-0.1	0	-0.01	0.967001427	low
TCGA-67-3773-01	Responder	-1.27	1.24	0.98	0.76	0.56	0.88	FALSE	-0.28	-1.27	-0.1	0	-0.01	0.81775047	low
TCGA-97-A4M5-01	Responder	-1.23	-0.35	0.54	-0.02	0.02	-0.94	FALSE	1.96	-1.23	-0.12	0	0.01	0.818223364	low
TCGA-55-7573-01	Responder	-1.23	0.31	0.4	0.16	-0.55	-0.31	FALSE	1.5	-1.23	-0.15	0.02	0.02	0.548709021	low
TCGA-49-AAQV-01	Responder	-1.23	0.13	0.55	0.09	0.35	0.08	FALSE	0.6	-1.23	0.03	-0.12	-0.01	1.208817328	low
TCGA-78-7143-01	Responder	-1.21	-0.59	0.52	-0.63	-0.09	-1.05	FALSE	-0.48	-1.21	0.01	-0.13	0.02	0.973989985	low
TCGA-44-A47A-01	Responder	-1.2	0.02	0.63	0.05	0.11	-0.67	FALSE	0.59	-1.2	-0.05	-0.08	0.03	1.152837062	low
TCGA-95-8039-01	Responder	-1.15	0.1	0.32	0.2	0.56	-0.83	FALSE	1.28	-1.15	-0.08	-0.04	0.02	0.767363238	low
TCGA-67-4679-01	Responder	-1.15	-0.9	0.56	-0.43	0.11	0.09	FALSE	0.22	-1.15	-0.14	-0.01	0.04	0.388755108	low
TCGA-55-8512-01	Responder	-1.14	-2.04	0.72	-1.14	-0.65	0.43	FALSE	1.06	-1.14	-0.08	-0.03	0.02	0.571996161	low
TCGA-50-5066-01	Responder	-1.12	2.2	0.98	1.21	0.41	3	TRUE	-1.12	0.64	0.03	0.04	-0.02	1.140930953	low
TCGA-97-8172-01	Responder	-1.11	-0.35	0.46	0.25	-0.38	0.71	FALSE	2.33	-1.11	-0.16	0.02	0.05	0.415004868	low
TCGA-67-3770-01	Responder	-1.1	0.8	0.98	0.18	-0.64	-0.05	FALSE	-0.86	-1.1	-0.08	-0.04	0.03	0.817386699	low
TCGA-50-8457-01	Responder	-1.06	0.03	0.48	0.47	-0.33	1	FALSE	2.36	-1.06	-0.15	0.02	0.04	0.745859787	low
TCGA-05-4249-01	Responder	-1.05	-0.59	0.34	-0.25	0.05	-0.81	FALSE	0.14	-1.05	-0.11	-0.03	0.05	1.004494489	low
TCGA-44-3919-01	Responder	-1.04	1.46	0.62	1.02	1.38	1.32	FALSE	1.56	-1.04	-0.07	0	-0.02	1.113721345	low
TCGA-55-A4DG-01	Responder	-1.03	-0.42	0.38	-0.07	-1.07	0.66	FALSE	0.06	-1.03	-0.07	-0.06	0.04	0.396874565	low
TCGA-73-4677-01	Responder	-1.02	-1.21	0.91	-0.45	-0.94	0.02	FALSE	-1.14	-1.02	-0.11	-0.03	0.05	1.110528414	low
TCGA-50-5068-01	Responder	-1.01	2.21	0.99	0.91	-0.23	2.1	TRUE	-1.01	-0.58	-0.03	-0.02	0	0.745072043	low
TCGA-78-7163-01	Responder	-0.98	-0.94	0.8	-0.64	-0.27	-0.46	FALSE	-1.25	-0.98	0.01	-0.12	0.01	0.652649113	low
TCGA-50-7109-01	Responder	-0.96	0.98	0.43	0.17	-1.38	0.6	TRUE	-0.96	0.05	0.01	0	0	1.192229565	low
TCGA-97-8175-01	Responder	-0.92	-0.63	0.55	-0.4	1.5	-1.69	FALSE	-0.59	-0.92	0	-0.06	-0.02	0.814089459	low
TCGA-73-7499-01	Responder	-0.9	2.73	0.86	1.37	-0.18	2.6	TRUE	-0.9	-1.01	0.02	-0.09	-0.01	0.927499917	low
TCGA-NJ-A55A-01	Responder	-0.88	-0.17	0.54	0.32	-0.11	0.46	FALSE	1.68	-0.88	-0.09	-0.02	0.03	0.85622436	low
TCGA-55-6543-01	Responder	-0.88	-0.3	0.74	-0.26	0.78	-1.48	FALSE	0.27	-0.88	-0.09	0.04	-0.03	1.089932945	low
TCGA-49-AARQ-01	Responder	-0.84	0.71	0.4	0.3	-1.19	1.13	FALSE	-0.4	-0.84	0.06	-0.13	-0.01	0.265302333	low
TCGA-97-8177-01	Responder	-0.83	-0.26	0.76	0.38	-0.46	0.26	FALSE	1.23	-0.83	-0.12	0.06	-0.01	0.647637784	low
TCGA-97-7941-01	Responder	-0.78	-0.84	0.56	-0.23	-1.28	-0.39	FALSE	1.87	-0.78	-0.07	-0.03	0.04	1.167957614	low
TCGA-55-1592-01	Responder	-0.77	-0.59	0.38	-0.43	-0.13	-1.14	FALSE	-0.6	-0.77	-0.06	-0.04	0.02	0.763754712	low
TCGA-86-8280-01	Responder	-0.73	1.28	0.47	0.86	0.48	0.28	FALSE	2.33	-0.73	-0.12	0.04	0.02	0.83734788	low
TCGA-78-8655-01	Responder	-0.71	0.39	0.43	0.28	-0.37	0.77	FALSE	0.81	-0.71	-0.06	-0.01	0.01	0.985431292	low
TCGA-75-5146-01	Responder	-0.7	-0.71	0.93	-0.35	-1.94	0.35	FALSE	-0.89	-0.7	-0.08	-0.04	0.06	1.234511034	low

TCGA-L9-A50W-01	Responder	-0.66	-0.55	0.07	-0.47	-2.19	-1.98	FALSE	0.99	-0.66	-0.06	-0.03	0.03	0.751676264	low
TCGA-50-8460-01	Responder	-0.58	-0.93	0.72	0.22	-0.27	0.19	FALSE	1.5	-0.58	-0.09	-0.01	0.04	1.049713686	low
TCGA-75-6206-01	Responder	-0.58	-1.24	0.22	-0.8	-1.08	-1.61	FALSE	-0.44	-0.58	-0.15	0.08	0.01	0.878850611	low
TCGA-75-7025-01	Responder	-0.54	-0.4	0.46	0.19	-0.98	0.39	FALSE	2.01	-0.54	-0.12	0.04	0.03	0.64740512	low
TCGA-78-8640-01	Responder	-0.52	0.05	0.32	-0.22	-1.22	-0.27	FALSE	-0.82	-0.52	0.07	-0.11	-0.01	0.725622862	low
TCGA-97-7546-01	Responder	-0.43	-0.43	0.45	-0.04	1.29	0.61	FALSE	0.51	-0.43	-0.1	0.06	0	0.877639349	low
TCGA-53-A4EZ-01	Responder	-0.42	-1.22	0.22	-1.3	-1.45	-0.6	FALSE	-1.65	-0.42	0.03	-0.12	0.05	0.828506705	low
TCGA-62-8397-01	Responder	-0.42	-3.06	0.69	-1.26	-0.94	-2.22	FALSE	0.07	-0.42	-0.09	-0.01	0.06	0.334459437	low
TCGA-55-A492-01	Responder	-0.42	-1.74	0.72	-1.3	-5	-1.41	FALSE	-0.31	-0.42	-0.04	-0.07	0.07	0.589456572	low
TCGA-NJ-A4YG-01	Responder	-0.37	-0.21	0.74	-0.1	-0.39	-0.8	FALSE	0.46	-0.37	-0.07	0.01	0.03	0.702109648	low
TCGA-50-5935-01	Responder	-0.36	0.49	0.3	0.37	-0.46	-0.17	FALSE	0.33	-0.36	-0.05	0.02	0.01	0.858492179	low
TCGA-86-7714-01	Responder	-0.31	-1.44	0.49	-1.27	-1.38	-1.62	FALSE	-0.99	-0.31	-0.09	0.02	0.04	0.897696605	low
TCGA-97-7547-01	Responder	-0.28	-0.88	0.4	-0.43	-0.17	-0.99	FALSE	0.37	-0.28	-0.08	0.02	0.04	0.79835846	low
TCGA-49-4486-01	Responder	-0.26	-1.69	0.98	-1.53	-2.78	-0.9	FALSE	-2.3	-0.26	0	-0.11	0.08	1.16041438	low
TCGA-44-6148-01	Responder	-0.25	-2.04	0.71	-0.47	-0.56	0.86	FALSE	1.17	-0.25	-0.11	0.04	0.05	0.87145809	low
TCGA-80-5608-01	Responder	-0.25	0.08	0.36	-0.31	-0.21	0.45	FALSE	-0.24	-0.25	0.04	-0.07	0	0.976088245	low
TCGA-NJ-A4YI-01	Responder	-0.23	-2.35	0.28	-1.36	1.14	-2.73	FALSE	0.51	-0.23	-0.05	0.02	0.01	0.818176612	low
TCGA-44-6147-01	Responder	-0.22	-0.24	0.3	0.14	-1.68	0.12	FALSE	1.4	-0.22	-0.06	0.02	0.02	0.62973591	low
TCGA-55-8087-01	Responder	-0.18	-2.23	0.53	-1.46	-1.99	-2.27	FALSE	-0.72	-0.18	-0.1	0	0.09	0.416361053	low
TCGA-44-2659-01	Responder	-0.18	0.68	0.46	0.72	-0.27	1.53	FALSE	0.74	-0.18	-0.08	0.06	0.01	0.414642053	low
TCGA-05-5715-01	Responder	-0.18	-1.32	0.34	-1.12	-1.58	-1.21	FALSE	-0.08	-0.18	-0.05	0.03	0.02	0.692051482	low
TCGA-44-5643-01	Responder	-0.17	-0.14	0.65	-0.01	-1.15	0.13	FALSE	-0.75	-0.17	0.07	-0.12	0.02	0.76395858	low
TCGA-73-7498-01	Responder	-0.17	-1.27	0.27	-0.54	-1.4	-0.95	FALSE	1	-0.17	-0.11	0.01	0.08	0.47152296	low
TCGA-69-7764-01	Responder	-0.17	-0.19	0.09	-0.26	-0.79	0.41	FALSE	-0.97	-0.17	-0.01	-0.04	0.03	0.450580046	low
TCGA-55-8506-01	Responder	-0.16	0.09	0.63	-0.17	0.19	-0.56	FALSE	0.34	-0.16	0.02	0.03	-0.05	1.005730538	low
TCGA-55-6972-01	Responder	-0.15	-2.66	0.82	-2.52	-3.3	-2.04	FALSE	-2.89	-0.15	0.04	-0.15	0.09	0.714087468	low
TCGA-64-5778-01	Responder	-0.15	2.79	0.72	1.81	0.83	2.85	TRUE	-0.15	-2.2	-0.02	-0.14	-0.03	0.749476416	low
TCGA-55-7227-01	Responder	-0.15	0.7	0.64	0.44	1.25	0.04	FALSE	1.42	-0.15	-0.09	0.09	0	1.192157991	low
TCGA-44-A47B-01	Responder	-0.14	-0.01	0.64	-0.19	-1.74	0.7	TRUE	-0.14	-0.71	-0.03	-0.02	-0.02	0.950282576	low
TCGA-MP-A5C7-01	Responder	-0.12	-1.33	0.38	-1.28	-2.44	-3.4	FALSE	-1.67	-0.12	-0.01	-0.07	0.06	0.585726768	low
TCGA-97-8174-01	Responder	-0.1	0.2	0.48	0.32	-0.54	-0.4	FALSE	1.16	-0.1	-0.12	0.07	0.04	0.794081634	low
TCGA-55-8203-01	Responder	-0.07	0.51	0.49	0.32	0.05	0.92	FALSE	0.18	-0.07	0.02	-0.04	0.02	1.196137401	low
TCGA-55-8097-01	Responder	-0.05	-1.19	0.29	-0.62	-1.23	-0.85	FALSE	0.77	-0.05	-0.09	0	0.08	0.623909754	low
TCGA-71-6725-01	Responder	-0.01	-2.68	0.14	-2.43	-1.57	-4.86	FALSE	-1.46	-0.01	0.02	-0.06	0.03	1.162627335	low
TCGA-55-A491-01	Responder	0	0.45	0.14	0.22	0.53	0.71	FALSE	0.66	0	0.01	0	0	0.594602429	low
TCGA-97-8171-01	Responder	0	-2.56	0.68	-2.21	-2.85	-3.97	FALSE	-2.36	0	0.02	-0.1	0.08	1.026684626	low

TCGA-50-5942-01	Responder	0	-1.33	0.51	-0.49	-1	-0.07	FALSE	0.32	0	-0.07	0.02	0.05	0.816396064	low
TCGA-86-7713-01	Responder	0	-1.21	0.1	-1.55	-0.91	-1.95	FALSE	-1.89	0	0.06	-0.09	0.02	0.996172159	low
TCGA-05-4426-01	Non-responder	0.01	-1.39	0.88	-0.72	2.15	-1.15	FALSE	-0.09	0.01	0.01	0.01	-0.02	0.881778653	low
TCGA-05-4427-01	Non-responder	0.03	0.23	0.44	-0.06	2.15	0.24	FALSE	-1.18	0.03	0.03	0.01	-0.04	1.082018148	low
TCGA-55-7911-01	Non-responder	0.07	1.86	0.79	1.11	1.81	1.59	TRUE	0.07	-2.53	-0.01	-0.13	-0.08	1.200239187	low
TCGA-91-6828-01	Non-responder	0.08	1.23	0.22	0.5	0.45	0.66	TRUE	0.08	-1.97	-0.11	-0.03	-0.02	0.773166421	low
TCGA-49-AARN-01	Non-responder	0.08	-0.22	0.38	-0.11	0	-0.15	FALSE	1.38	0.08	0.04	-0.03	0	0.885169154	low
TCGA-86-8668-01	Non-responder	0.1	-0.11	0.19	-0.04	-0.16	-0.65	FALSE	1.66	0.1	-0.1	0.09	0.03	0.791645396	low
TCGA-62-8402-01	Non-responder	0.1	2.72	0.36	1.35	1.43	2.19	TRUE	0.1	-1.77	0.03	-0.13	-0.06	1.169492045	low
TCGA-05-4389-01	Non-responder	0.11	1.41	0.24	0.87	0.66	2.36	TRUE	0.11	-1.49	-0.04	-0.13	0.03	1.115736626	low
TCGA-86-8073-01	Non-responder	0.11	-0.85	0.53	-0.71	-1.4	-0.66	FALSE	-0.95	0.11	-0.07	0.04	0.05	1.171312422	low
TCGA-44-6777-01	Non-responder	0.15	0.4	0.5	0.85	1.56	-0.45	FALSE	1.79	0.15	-0.15	0.2	-0.03	1.110142452	low
TCGA-50-5932-01	Non-responder	0.18	-1.84	0.26	-1.34	-1.5	-2.32	FALSE	-2.02	0.18	0	-0.04	0.05	1.025218381	low
TCGA-86-8359-01	Non-responder	0.22	0.37	0.19	0.29	-1.08	2.4	TRUE	0.22	0.12	0.03	0.03	-0.05	1.108109713	low
TCGA-55-1594-01	Non-responder	0.25	0.13	0.16	-0.22	0.17	1.51	FALSE	-0.98	0.25	0.04	-0.05	0.03	0.809124092	low
TCGA-NJ-A7XG-01	Non-responder	0.25	-1.69	0.45	-1.78	-2.12	-2.87	FALSE	-1	0.25	0.05	-0.08	0.04	0.635502939	low
TCGA-67-3771-01	Non-responder	0.25	1.05	0.37	0.47	-0.21	0.53	TRUE	0.25	-0.45	-0.01	-0.03	-0.01	0.832073914	low
TCGA-44-6776-01	Non-responder	0.28	-1.85	0.69	-1.2	-1.8	-1.12	FALSE	-1.18	0.28	-0.03	-0.03	0.09	0.925131801	low
TCGA-44-2666-01	Non-responder	0.28	-1.8	0.9	-0.73	1.05	-0.26	FALSE	-0.81	0.28	0.02	-0.04	0.04	1.171584796	low
TCGA-97-7938-01	Non-responder	0.29	-0.72	0.33	-0.64	0.79	0.15	FALSE	-1.06	0.29	-0.07	0.07	0.02	0.709478814	low
TCGA-NJ-A4YF-01	Non-responder	0.31	-2.01	0.45	-1.57	-2.89	-1.61	FALSE	-1.12	0.31	0.06	-0.05	0.02	0.730966538	low
TCGA-55-8507-01	Non-responder	0.36	-1.17	0.14	-0.84	-1.1	-1.09	FALSE	0.14	0.36	0.04	-0.02	0.01	0.573374342	low
TCGA-55-8620-01	Non-responder	0.38	0.47	0.56	0.09	-0.25	0.68	TRUE	0.38	-0.63	0.03	-0.12	0.03	0.887820706	low
TCGA-53-7813-01	Non-responder	0.38	0.33	0.28	-0.2	-1.25	1.42	FALSE	-2.36	0.38	0.08	-0.04	0	1.005482687	low
TCGA-44-A4SU-01	Non-responder	0.38	0.02	0.22	-0.21	-0.58	-0.94	FALSE	0.92	0.38	0	0.01	0.03	0.796621377	low
TCGA-MP-A4T9-01	Non-responder	0.41	-0.72	0.18	-0.2	0.89	-0.23	FALSE	1.62	0.41	-0.02	0.05	0	1.111876314	low
TCGA-55-8207-01	Non-responder	0.42	-1.12	0.25	-0.7	-0.11	-0.15	FALSE	0.66	0.42	-0.12	0.15	0.01	0.547204	low
TCGA-78-7539-01	Non-responder	0.42	1.26	0.49	0.92	0.07	1.53	TRUE	0.42	-2.67	-0.12	-0.09	-0.02	0.386354372	low
TCGA-55-8096-01	Non-responder	0.42	-0.31	0.11	-0.18	-0.75	-0.24	FALSE	1	0.42	-0.04	0.08	0.01	1.012389222	low
TCGA-49-4501-01	Non-responder	0.44	0.1	0.75	0.26	-0.02	0.29	FALSE	0.69	0.44	-0.05	0.06	0.03	0.85804482	low
TCGA-55-6642-01	Non-responder	0.44	0.07	0.37	0.43	-0.11	1.05	TRUE	0.44	1.84	0.01	0.17	-0.01	0.859695436	low
TCGA-44-2668-01	Non-responder	0.44	1.49	0.46	1.18	2.31	1.3	TRUE	0.44	0.62	0.06	0.12	-0.12	1.124002314	low
TCGA-75-5147-01	Non-responder	0.45	0.29	0.83	0.19	-0.05	0.02	FALSE	-0.55	0.45	0.07	-0.04	0.01	0.881844552	low
TCGA-62-8395-01	Non-responder	0.47	-1.47	0.36	-0.96	-2.58	-1.01	FALSE	0.7	0.47	-0.08	0.06	0.06	0.683494129	low
TCGA-91-6840-01	Non-responder	0.47	0.41	0.02	0.42	-1.21	0.97	TRUE	0.47	-0.25	0.03	-0.06	0	0.993257935	low
TCGA-49-4510-01	Non-responder	0.47	-1.35	0.48	-0.64	-1.09	-0.15	FALSE	0.05	0.47	-0.03	0.02	0.05	0.568618376	low

TCGA-55-A57B-01	Non-responder	0.5	-1.32	0.39	-0.62	0.03	-1.38	FALSE	0.96	0.5	-0.03	0.04	0.03	1.015570091	low
TCGA-49-4487-01	Non-responder	0.55	1.05	0.3	0.99	-0.16	1.66	TRUE	0.55	0.4	0.05	0.03	-0.04	1.210356611	low
TCGA-55-8091-01	Non-responder	0.59	1.69	0.74	0.88	1.96	-1.21	FALSE	1.15	0.59	-0.06	0.17	-0.04	0.851264267	low
TCGA-95-7562-01	Non-responder	0.6	1.5	0.71	0.24	-0.42	-0.34	FALSE	-2.02	0.6	0.07	0	-0.02	1.01533589	low
TCGA-97-8179-01	Non-responder	0.63	-0.69	0.07	-1.08	-0.82	-1.12	FALSE	-1.47	0.63	-0.02	0.01	0.06	0.626753761	low
TCGA-44-3918-01	Non-responder	0.63	2.82	0.89	1.96	3.4	2.28	TRUE	0.63	-1.38	-0.07	0.04	-0.08	0.761321577	low
TCGA-78-7167-01	Non-responder	0.65	-1.69	0.36	-1.71	-3.86	-1.24	FALSE	-1.4	0.65	-0.05	0.01	0.09	0.639698267	low
TCGA-91-7771-01	Non-responder	0.68	0.93	0.44	0.71	-0.05	1.23	TRUE	0.68	-1.82	-0.13	-0.02	-0.01	1.193445265	low
TCGA-55-7910-01	Non-responder	0.69	-1.04	0.18	-1.2	-1.11	-2.02	FALSE	-1.35	0.69	0.09	-0.01	-0.02	1.130479683	low
TCGA-55-8616-01	Non-responder	0.71	-1.61	0.33	-0.89	0.34	-0.19	FALSE	-0.05	0.71	0.02	-0.02	0.07	1.197491037	low
TCGA-44-5644-01	Non-responder	0.75	-2.08	0.04	-1.74	-2.88	-0.79	FALSE	-0.89	0.75	0.1	-0.05	0.01	1.001736445	low
TCGA-55-7816-01	Non-responder	0.77	1.59	0.83	0.96	0.66	1.12	TRUE	0.77	0.25	-0.11	0.14	-0.01	1.03459385	low
TCGA-55-8090-01	Non-responder	0.78	-0.63	0.61	-0.38	1.9	-1.32	FALSE	1.32	0.78	0.03	0.05	-0.01	1.258256006	low
TCGA-MP-A4TE-01	Non-responder	0.78	-4.09	0.08	-2.42	-1.61	-2.33	FALSE	-1.03	0.78	0.07	-0.05	0.04	1.067298428	low
TCGA-62-A46U-01	Non-responder	0.78	2.94	0.78	1.72	2.77	0.72	TRUE	0.78	-4.72	-0.16	-0.14	-0.1	1.066526654	low
TCGA-55-8508-01	Non-responder	0.78	-1.13	0.2	-0.24	-0.86	-0.58	FALSE	0.89	0.78	0.07	0.02	-0.02	1.177202528	low
TCGA-MN-A4N5-01	Non-responder	0.79	0.99	0.48	0.75	1.78	1.17	TRUE	0.79	-1.13	0.01	-0.09	-0.02	0.792444954	low
TCGA-NJ-A55R-01	Non-responder	0.81	-2.66	0.5	-1.08	-1.21	-2.27	FALSE	0.9	0.81	0.02	-0.01	0.05	1.129549198	low
TCGA-86-8278-01	Non-responder	0.83	0.22	0.6	0	0.53	-1.17	FALSE	0.64	0.83	-0.01	0.11	-0.02	0.69880487	low
TCGA-44-3398-01	Non-responder	0.87	0.49	0.99	0.47	1.7	0.51	FALSE	-0.33	0.87	-0.06	0.11	0.02	1.18747188	low
TCGA-86-8358-01	Non-responder	0.89	-0.4	0.4	-0.35	-1.13	0.47	FALSE	-0.28	0.89	0.09	-0.06	0.04	0.702384826	low
TCGA-95-7948-01	Non-responder	0.94	-1.4	0.8	-1.14	-2.48	-0.96	FALSE	-1.1	0.94	0.03	0	0.05	0.427290035	low
TCGA-67-3772-01	Non-responder	0.96	0.02	0.79	0.05	-2.09	-0.35	FALSE	-0.18	0.96	-0.05	0.1	0.04	0.867184064	low
TCGA-05-4417-01	Non-responder	1	0.7	0.39	0.71	0.28	0.88	TRUE	1	0.69	-0.06	0.13	-0.01	0.931738402	low
TCGA-44-6775-01	Non-responder	1.04	-0.09	0.28	0.04	0.24	-0.91	FALSE	0.62	1.04	-0.06	0.16	0	1.245512288	low
TCGA-L9-A443-01	Non-responder	1.09	0.13	0.22	-0.49	-1.57	0.27	FALSE	-0.22	1.09	0.03	0.06	0.01	1.171200566	low
TCGA-35-5375-01	Non-responder	1.1	1.35	0.9	0.6	0.27	1.46	FALSE	-2.11	1.1	0.08	0.01	0.01	1.192289977	low
TCGA-05-4430-01	Non-responder	1.1	0.14	0.52	0.37	0.12	0.25	TRUE	1.1	1.97	-0.01	0.15	0.03	1.224521994	low
TCGA-55-8615-01	Non-responder	1.11	-2.53	0.14	-2.02	-2.26	-3.18	FALSE	-0.78	1.11	0.05	-0.01	0.04	0.761054691	low
TCGA-50-6673-01	Non-responder	1.16	-1.56	0.12	-1.01	-1.24	-2.22	FALSE	0.02	1.16	0.04	0.05	0.01	0.738678495	low
TCGA-55-A48Z-01	Non-responder	1.2	1.05	0.3	0.72	0.58	0.32	FALSE	0.56	1.2	0.05	0.09	-0.03	0.773236339	low
TCGA-93-7348-01	Non-responder	1.21	-0.53	0.38	-0.08	-1.08	-0.58	FALSE	0.9	1.21	-0.01	0.12	0.01	0.549892172	low
TCGA-97-7937-01	Non-responder	1.25	-1.48	0.09	-1.7	-1.92	-2.72	FALSE	-2.02	1.25	0.06	0.04	0.01	1.029395076	low
TCGA-L9-A8F4-01	Non-responder	1.27	1	0.25	0.78	-0.17	1.5	TRUE	1.27	-0.69	0	-0.06	0	0.916114502	low
TCGA-J2-8192-01	Non-responder	1.28	0.11	0.4	0.13	0.97	-0.43	FALSE	0.81	1.28	-0.09	0.22	0	1.083889971	low
TCGA-78-7537-01	Non-responder	1.3	-1.78	0.65	-0.81	-2.02	-0.12	FALSE	0.02	1.3	-0.03	0.07	0.07	0.516169482	low

TCGA-97-7554-01	Non-responder	1.3	-0.29	0.47	0.15	2.94	-0.03	FALSE	0.19	1.3	0	0.14	-0.02	0.979096952	low
TCGA-05-4405-01	Non-responder	1.32	-1.45	0.2	-0.79	0.28	-0.35	FALSE	0.63	1.32	-0.05	0.14	0.02	0.616744398	low
TCGA-69-7980-01	Non-responder	1.36	1.16	0.11	0.47	0.67	0.76	TRUE	1.36	-0.12	0	0.02	-0.03	0.863515134	low
TCGA-78-7149-01	Non-responder	1.37	-1.12	0.38	-1.22	-3.47	-1.24	FALSE	-0.84	1.37	0.02	0	0.1	1.01843221	low
TCGA-38-4628-01	Non-responder	1.4	-2.21	0.71	-1.71	-1.31	0.1	FALSE	-1.6	1.4	0	0.07	0.06	1.124907726	low
TCGA-86-8076-01	Non-responder	1.4	1.27	0.83	1.18	0.08	2.18	TRUE	1.4	-2.34	-0.14	-0.05	-0.01	1.144734399	low
TCGA-44-2661-01	Non-responder	1.43	1.86	0.95	1.53	0.64	2.09	TRUE	1.43	-2.38	-0.2	-0.01	0.01	0.76635256	low
TCGA-50-8459-01	Non-responder	1.43	-0.88	0.31	0.21	-0.76	0	FALSE	3.03	1.43	-0.08	0.22	0	1.044517226	low
TCGA-55-6987-01	Non-responder	1.44	2.45	0.8	1.88	1.89	2.49	TRUE	1.44	-2.95	-0.1	-0.07	-0.1	0.88921016	low
TCGA-78-7155-01	Non-responder	1.45	-0.45	0.12	-1.26	-1.35	-0.35	FALSE	-2.48	1.45	0.12	-0.06	0.06	0.808639941	low
TCGA-93-A4JN-01	Non-responder	1.45	-0.74	0.13	-0.22	-0.32	-1.39	FALSE	1.47	1.45	0.05	0.08	0	0.747972423	low
TCGA-05-4390-01	Non-responder	1.48	-1.64	0.46	-1.34	-0.24	-0.49	FALSE	-1.01	1.48	0.08	0.03	0.02	0.926323301	low
TCGA-55-1595-01	Non-responder	1.48	0.31	0.52	0.03	0.06	0.48	FALSE	-0.45	1.48	0.04	0.07	0.02	0.703773721	low
TCGA-49-4512-01	Non-responder	1.52	-0.92	0.28	-0.7	-1.82	-2.71	FALSE	1.23	1.52	-0.02	0.13	0.03	0.886694718	low
TCGA-78-8662-01	Non-responder	1.58	-1.68	0.37	-1.9	-2.69	-1.4	FALSE	-2.22	1.58	0.1	-0.03	0.06	0.733744207	low
TCGA-55-8301-01	Non-responder	1.63	2.85	0.75	1.96	2.25	1.6	TRUE	1.63	-1.38	-0.04	0	-0.07	1.188273512	low
TCGA-50-5946-01	Non-responder	1.65	-1.05	0.15	-1.31	-1.16	-0.11	FALSE	-1.69	1.65	0.18	-0.08	0.03	0.833308225	low
TCGA-50-5944-01	Non-responder	1.65	-1.17	0.25	-0.93	-0.94	-1.36	FALSE	-0.51	1.65	-0.06	0.16	0.05	0.979749398	low
TCGA-64-1678-01	Non-responder	1.68	-1.96	0.98	-1.63	-2.96	-0.72	FALSE	-3.06	1.68	0.15	-0.06	0.05	1.254294529	low
TCGA-78-7161-01	Non-responder	1.68	-1.55	0.09	-1.61	-2.33	-1.27	FALSE	-0.92	1.68	0.06	0.07	0.01	0.72035694	low
TCGA-MP-A4TD-01	Non-responder	1.74	-1.66	0.3	-0.58	-1.75	-0.85	FALSE	1.58	1.74	0	0.11	0.05	0.814593314	low
TCGA-95-A4VP-01	Non-responder	1.76	-0.47	0.21	0.13	-0.83	0.91	TRUE	1.76	0.1	0.01	0	0	1.245879113	low
TCGA-62-A46V-01	Non-responder	1.87	-1.85	0.73	-1.8	-1.67	-3.56	FALSE	-1.2	1.87	0.03	0.08	0.05	0.795799713	low
TCGA-44-6778-01	Non-responder	1.87	1.97	0.48	1.74	2.22	1.58	TRUE	1.87	-2.55	-0.11	-0.09	-0.04	1.208680427	low
TCGA-55-6985-01	Non-responder	1.88	1.36	0.45	0.92	0.77	0.63	TRUE	1.88	0.11	0	0.07	-0.05	1.160208742	low
TCGA-50-5045-01	Non-responder	1.88	1.34	0.86	1.05	0.87	1.65	TRUE	1.88	0.22	-0.07	0.1	0	0.785650025	low
TCGA-05-4420-01	Non-responder	1.91	-2.34	0.49	-1.76	-1.66	-0.51	FALSE	-1.78	1.91	0.12	0.01	0.03	1.113104752	low
TCGA-91-A4BC-01	Non-responder	1.93	1.54	0.22	1.51	-0.63	3.72	TRUE	1.93	0.07	0.02	0.04	-0.04	0.764627646	low
TCGA-MN-A4N4-01	Non-responder	1.93	-1.31	0.1	-0.76	-1.13	-0.25	FALSE	0.37	1.93	0.05	0.11	0.01	0.855176649	low
TCGA-53-7626-01	Non-responder	2.02	0.84	0.39	0.99	0.45	1.4	TRUE	2.02	-1.67	-0.13	0.02	-0.03	0.978817937	low
TCGA-97-7553-01	Non-responder	2.03	0.68	0.57	0.89	1.34	1.43	TRUE	2.03	-3.02	-0.21	-0.01	-0.04	0.969852465	low
TCGA-69-7979-01	Non-responder	2.05	-0.49	0.37	-1.17	-0.23	-1.84	FALSE	-0.87	2.05	0.1	0.07	0.02	0.865011696	low
TCGA-49-AAR0-01	Non-responder	2.13	0.11	0.47	0.42	-1.18	0.08	TRUE	2.13	-0.99	-0.03	-0.08	0.02	1.15350818	low
TCGA-55-6971-01	Non-responder	2.18	1.59	0.5	1.51	1.18	2.06	TRUE	2.18	-2.03	-0.1	0	-0.07	0.786563262	low
TCGA-38-6178-01	Non-responder	2.18	-0.66	0.14	-0.5	-1.69	-1.9	FALSE	-0.02	2.18	0.05	0.15	-0.01	1.206931688	low
TCGA-97-8547-01	Non-responder	2.19	-0.52	0.39	-0.52	-0.3	-2.76	FALSE	0.84	2.19	0.01	0.17	0.02	0.790287394	low

TCGA-L9-A444-01	Non-responder	2.2	2.69	0.39	2.1	2.26	2.89	TRUE	2.2	-2.4	-0.1	-0.04	-0.06	0.374923982	low
TCGA-93-A4JO-01	Non-responder	2.24	0.8	0.4	0.79	0.31	1.31	TRUE	2.24	-1.63	-0.09	-0.05	0	0.886708425	low
TCGA-44-A47G-01	Non-responder	2.27	1.29	0.66	1.22	1.62	1.52	TRUE	2.27	-1.83	-0.12	0.04	-0.07	1.025904208	low
TCGA-86-6851-01	Non-responder	2.28	2.58	0.29	1.74	1.28	1.71	TRUE	2.28	-3.22	-0.11	-0.1	-0.06	0.447972336	low
TCGA-49-AARR-01	Non-responder	2.29	-0.86	0.64	0.18	0.53	0.81	TRUE	2.29	-1.17	-0.11	0.02	0	0.663132049	low
TCGA-44-4112-01	Non-responder	2.29	-0.25	0.96	-0.33	0.22	0.14	FALSE	-1.54	2.29	0.06	0.12	0.02	1.085514875	low
TCGA-55-A48X-01	Non-responder	2.3	0.03	0.29	0.39	0.07	0.49	TRUE	2.3	-1.11	-0.07	-0.04	0.02	0.842449588	low
TCGA-86-8054-01	Non-responder	2.35	-2.03	0.42	-1.75	-1.03	-1.48	FALSE	-2.53	2.35	0.15	0	0.05	0.971256356	low
TCGA-97-7552-01	Non-responder	2.36	1.84	0.75	1.48	0.81	2.47	TRUE	2.36	-3.38	-0.16	-0.1	-0.03	0.898683489	low
TCGA-91-6835-01	Non-responder	2.43	1.35	0.13	1.4	1.31	2.41	TRUE	2.43	-2.32	-0.15	-0.04	-0.01	0.716522861	low
TCGA-86-A4P7-01	Non-responder	2.44	-0.55	0.4	0.3	-0.31	1.02	TRUE	2.44	-1.65	-0.11	-0.03	-0.01	0.639722261	low
TCGA-S2-AA1A-01	Non-responder	2.45	0.5	0.26	0.95	0.02	0.71	TRUE	2.45	-0.77	-0.1	0.04	0	0.561354988	low
TCGA-55-8208-01	Non-responder	2.48	2.28	0.29	1.69	2.14	1.65	TRUE	2.48	-2.13	-0.13	0.03	-0.08	0.785449847	low
TCGA-93-A4JQ-01	Non-responder	2.52	1.24	0.23	0.98	1.21	0.21	TRUE	2.52	0.82	0.03	0.06	-0.02	0.642881522	low
TCGA-38-A44F-01	Non-responder	2.56	1.01	0.78	0.62	-1.22	0.45	TRUE	2.56	-1.8	-0.12	-0.03	-0.01	1.036541376	low
TCGA-91-6847-01	Non-responder	2.59	-1.33	0.13	-1.99	-1.02	-1.7	FALSE	-3.21	2.59	0.21	-0.03	0.04	1.202532616	low
TCGA-86-A4P8-01	Non-responder	2.61	0.42	0.62	0.73	0.43	1.48	TRUE	2.61	-3.43	-0.24	-0.03	-0.03	0.360876662	low
TCGA-MP-A4TK-01	Non-responder	2.64	0.01	0.38	0.17	-0.55	0.12	FALSE	1.78	2.64	0.03	0.19	0.01	1.24678786	low
TCGA-55-8510-01	Non-responder	2.69	1.1	0.18	0.8	1.11	1.16	TRUE	2.69	-1.2	-0.11	0.02	-0.01	0.98862852	low
TCGA-55-7574-01	Non-responder	2.7	0.13	0.3	0.64	-0.44	1.56	TRUE	2.7	-0.1	-0.07	0.08	-0.02	0.901182436	low
TCGA-69-8453-01	Non-responder	2.74	0.79	0.66	0.98	0.51	0.51	TRUE	2.74	-3.24	-0.17	-0.06	-0.05	0.888399697	low
TCGA-50-5055-01	Non-responder	2.76	0.92	0.67	1.28	-1.02	2.17	TRUE	2.76	-0.6	-0.09	0.04	0.01	0.976016831	low
TCGA-97-A4LX-01	Non-responder	2.78	2.15	0.51	1.74	1.84	1.42	TRUE	2.78	-2.65	-0.19	0.01	-0.04	0.641152754	low
TCGA-55-8614-01	Non-responder	2.81	-0.08	0.11	-0.24	-0.97	-0.6	FALSE	0.53	2.81	0.09	0.14	0.01	0.846984773	low
TCGA-MP-A4TJ-01	Non-responder	2.86	1.93	0.5	1.67	2.02	2.11	TRUE	2.86	-2.17	-0.11	0	-0.06	1.0736944	low
TCGA-44-2665-01	Non-responder	2.88	-0.33	0.8	-0.11	1.31	-1.32	FALSE	-0.07	2.88	0.01	0.25	0	0.665884838	low
TCGA-L9-A743-01	Non-responder	2.99	1.08	0.49	1.01	1.12	1.28	TRUE	2.99	-1.27	-0.08	0.03	-0.05	0.907222132	low
TCGA-38-7271-01	Non-responder	3.03	0.73	0.35	1.51	0.31	2.43	TRUE	3.03	-1.38	-0.14	0.04	-0.01	1.044866161	low
TCGA-44-2657-01	Non-responder	3.1	1.81	0.66	1.59	0	2.57	TRUE	3.1	-2.1	-0.15	-0.04	0.01	0.550161615	low
TCGA-93-7347-01	Non-responder	3.1	1.07	0.64	1.29	-0.03	2.04	TRUE	3.1	-1.62	-0.16	0.03	0	0.85611221	low
TCGA-99-8028-01	Non-responder	3.13	2.49	0.55	2.28	2.16	2.47	TRUE	3.13	-1.27	-0.11	0.1	-0.09	0.866063969	low
TCGA-78-8648-01	Non-responder	3.13	0.62	0.37	1.35	0.74	2.13	TRUE	3.13	0.4	-0.13	0.23	-0.06	0.648964975	low
TCGA-99-AA5R-01	Non-responder	3.16	0.69	0.52	1.08	1.04	1.7	TRUE	3.16	-2.04	-0.16	0	-0.02	0.5870845	low
TCGA-55-8619-01	Non-responder	3.49	-0.03	0.54	0.79	-0.01	1.25	TRUE	3.49	-2.97	-0.18	-0.05	-0.02	0.879288706	low
TCGA-44-6774-01	Non-responder	3.53	-0.49	0.14	-0.29	-0.48	-1.41	FALSE	0.75	3.53	0.03	0.3	-0.01	1.015854628	low
TCGA-50-6591-01	Non-responder	4.21	-3.84	0.03	-4	-3.38	-3.89	FALSE	-2.56	4.21	0.21	0.09	0.06	1.094873563	low

TCGA-86-8671-01	Non-responder	4.72	2.51	0.42	2.14	0.8	2.31	TRUE	4.72	-2.66	-0.2	0.01	-0.04	0.586861312	low
TCGA-05-4433-01	Responder	-3.39	0.39	0.41	0.4	-0.21	0.55	FALSE	1.16	-3.39	-0.12	-0.13	-0.04	2.782442585	high
TCGA-55-7725-01	Responder	-2.56	1.26	0.78	0.47	0.88	0.63	FALSE	-1.59	-2.56	-0.09	-0.13	-0.01	1.300636451	high
TCGA-91-6849-01	Responder	-2.08	-0.88	0.71	-0.02	-1.38	0.21	FALSE	0.18	-2.08	-0.16	-0.01	0	1.801206678	high
TCGA-95-7947-01	Responder	-2.07	0.62	0.46	0.16	0.19	0.04	FALSE	-0.74	-2.07	-0.03	-0.15	-0.01	1.294331385	high
TCGA-05-4415-01	Responder	-2.06	0.17	0.73	-0.2	-0.05	0.4	TRUE	-2.06	1.26	0.15	-0.03	-0.02	7.315548522	high
TCGA-55-7284-01	Responder	-2.04	-0.53	0.4	-0.01	0.14	0.19	FALSE	1.4	-2.04	-0.13	-0.03	-0.01	1.431804767	high
TCGA-78-7540-01	Responder	-1.76	-2.1	0.53	-1.1	-1.13	-1.58	FALSE	0.55	-1.76	-0.06	-0.1	0.01	2.450986159	high
TCGA-55-6970-01	Responder	-1.47	0.95	0.79	0.45	0.74	0.26	FALSE	-1.12	-1.47	-0.05	-0.07	-0.02	1.568767094	high
TCGA-62-A46S-01	Responder	-1.45	-0.19	0.35	-0.43	-0.61	-0.46	FALSE	-0.29	-1.45	-0.07	-0.06	0.01	1.264402047	high
TCGA-69-7974-01	Responder	-1.42	1.06	0.53	0.72	2.6	0.04	FALSE	1.27	-1.42	-0.05	-0.04	-0.03	2.314622346	high
TCGA-05-4403-01	Responder	-1.39	-0.1	0.93	-0.03	-0.45	-0.73	FALSE	1.34	-1.39	-0.09	-0.03	-0.01	1.527415506	high
TCGA-62-A46R-01	Responder	-1.36	-0.76	0.54	-0.46	-0.33	0.92	FALSE	-0.8	-1.36	-0.07	-0.06	0.01	1.278189258	high
TCGA-62-A472-01	Responder	-1.3	0.58	0.8	0.28	1.3	0.87	FALSE	-0.73	-1.3	0.01	-0.11	-0.02	2.217286161	high
TCGA-55-7727-01	Responder	-1.25	0.56	0.73	-0.03	0.17	0.22	FALSE	-2.04	-1.25	-0.02	-0.09	0.01	1.668172546	high
TCGA-80-5611-01	Responder	-1.16	1.83	0.72	0.7	-0.43	-0.79	FALSE	-1.33	-1.16	0.02	-0.08	-0.04	1.84880242	high
TCGA-49-4506-01	Responder	-1.16	1.04	1	0.82	-0.77	2.77	TRUE	-1.16	-0.11	0.07	-0.06	-0.02	6.213258422	high
TCGA-69-A59K-01	Responder	-1.12	0.32	0.67	0.47	3.62	0.45	FALSE	0.3	-1.12	-0.02	-0.06	-0.02	1.713699623	high
TCGA-75-6212-01	Responder	-1.11	-1.04	0.82	-0.26	-0.38	-1.92	FALSE	0.39	-1.11	-0.12	-0.02	0.05	1.813084207	high
TCGA-78-7536-01	Responder	-1.06	0.66	0.38	-0.34	-1.03	-0.32	FALSE	-2.11	-1.06	0.03	-0.11	-0.01	3.661062338	high
TCGA-MP-A4T7-01	Responder	-1.03	-0.56	0.78	-0.3	-0.98	-0.84	FALSE	0.4	-1.03	0.01	-0.09	-0.02	1.674103281	high
TCGA-55-7903-01	Responder	-0.96	1.11	0.81	0.31	1.64	0.46	TRUE	-0.96	-1.3	0.03	-0.11	-0.03	1.595237721	high
TCGA-99-7458-01	Responder	-0.94	0.97	0.74	1.01	2.3	1.49	FALSE	1.25	-0.94	-0.11	0.03	0.01	1.437241067	high
TCGA-78-7148-01	Responder	-0.85	-0.4	0.56	-0.19	-1.38	0.71	TRUE	-0.85	0.24	0.04	-0.03	0.01	2.594473221	high
TCGA-55-8514-01	Responder	-0.82	-1.2	0.31	-0.78	-0.26	-2.38	FALSE	0.64	-0.82	-0.03	-0.05	0	1.276774419	high
TCGA-69-8255-01	Responder	-0.8	1.79	0.85	1.19	0.16	2.38	TRUE	-0.8	-1.64	0.02	-0.18	0	2.011557931	high
TCGA-49-6767-01	Responder	-0.73	2.34	0.26	1.13	1.66	1.02	TRUE	-0.73	0.62	0.12	-0.01	-0.04	1.777206544	high
TCGA-78-7152-01	Responder	-0.72	-0.64	0.8	-0.07	-1.45	0.5	FALSE	0.24	-0.72	-0.03	-0.07	0.03	1.589675081	high
TCGA-55-7724-01	Responder	-0.72	0.98	0.52	0.32	-0.11	0.43	TRUE	-0.72	-0.36	-0.05	0.02	0	1.985431157	high
TCGA-49-AAR2-01	Responder	-0.68	-0.49	0.37	-0.02	-2.01	0.01	FALSE	1.02	-0.68	0.04	-0.14	0.01	1.348169936	high
TCGA-44-6146-01	Responder	-0.66	-1.78	0.46	-1.45	-1.26	-1.28	FALSE	-0.49	-0.66	-0.03	-0.05	0.01	2.303683623	high
TCGA-62-A470-01	Responder	-0.65	-1.24	0.4	-1.37	-1.24	-1.53	FALSE	-1.57	-0.65	-0.02	-0.09	0.05	1.619364631	high
TCGA-05-4398-01	Responder	-0.63	0.96	0.47	0.56	3.3	0.15	FALSE	0.33	-0.63	0	0	-0.06	2.340809891	high
TCGA-99-8033-01	Responder	-0.61	1.71	0.78	1	1.34	1.27	TRUE	-0.61	-0.23	0.08	-0.01	-0.09	4.668588695	high
TCGA-86-7953-01	Responder	-0.59	-0.28	0.29	-0.08	-0.52	-0.25	FALSE	-0.18	-0.59	0	-0.02	-0.02	1.338559509	high
TCGA-50-6597-01	Responder	-0.58	0.46	0.23	0.38	-2.8	0.27	FALSE	0.82	-0.58	-0.01	-0.05	0.01	2.377556972	high

TCGA-35-4122-01	Responder	-0.54	2.18	0.93	1.5	2.04	2.09	TRUE	-0.54	-0.47	0	-0.01	-0.04	2.873221231	high
TCGA-05-5420-01	Responder	-0.53	3.24	0.98	1.71	1.7	2.02	TRUE	-0.53	-2.72	-0.11	-0.08	-0.05	1.640301564	high
TCGA-95-A4VK-01	Responder	-0.49	-0.25	0.26	-0.24	-1.04	0.36	FALSE	0.77	-0.49	0	-0.05	0.02	2.147399998	high
TCGA-86-A4JF-01	Responder	-0.49	0.83	0.67	0.5	1.99	0.13	TRUE	-0.49	-1.25	0.03	-0.08	-0.07	2.377925241	high
TCGA-55-8085-01	Responder	-0.47	1.41	0.62	0.99	-0.45	1.2	TRUE	-0.47	-1.6	-0.01	-0.1	-0.03	1.264549038	high
TCGA-49-4514-01	Responder	-0.46	0.37	0.33	0.17	-2.7	0.98	TRUE	-0.46	-0.28	0.08	-0.12	0.01	2.360874042	high
TCGA-78-7156-01	Responder	-0.44	-0.74	0.75	-1.2	-2.97	-1.48	FALSE	-2.01	-0.44	-0.03	-0.06	0.05	2.33752114	high
TCGA-J2-A4AG-01	Responder	-0.42	-0.1	0.48	0.08	-0.36	0	FALSE	1.59	-0.42	-0.06	0.03	-0.01	1.394301628	high
TCGA-44-7669-01	Responder	-0.41	0.18	0.42	0.14	0.5	0.05	FALSE	-0.39	-0.41	0.03	-0.08	0	2.973263261	high
TCGA-78-7147-01	Responder	-0.4	1.27	0.44	0.24	-1.96	0.52	FALSE	-0.88	-0.4	0.05	-0.1	0.02	2.132832545	high
TCGA-44-2655-01	Responder	-0.39	-1.07	0.91	-0.53	-1.12	-1.01	FALSE	-0.56	-0.39	-0.08	-0.02	0.06	1.810766015	high
TCGA-55-7283-01	Responder	-0.37	0.09	0.6	0.02	-0.52	0.19	FALSE	-0.36	-0.37	0	-0.07	0.04	1.900483237	high
TCGA-95-7567-01	Responder	-0.35	0.53	0.32	0.07	0.43	0.26	TRUE	-0.35	0.48	0.05	-0.01	0	3.42186807	high
TCGA-55-A4DF-01	Responder	-0.34	1.77	0.65	0.87	1.53	1.23	TRUE	-0.34	-1.55	0.01	-0.1	-0.04	2.151265905	high
TCGA-64-1681-01	Responder	-0.33	0.54	0.15	0.13	-0.9	-2.15	FALSE	0.7	-0.33	-0.04	0.03	-0.01	2.168230869	high
TCGA-64-5781-01	Responder	-0.33	1.47	0.89	0.93	0.13	2.6	TRUE	-0.33	1.66	0.12	0.08	-0.06	2.101352327	high
TCGA-4B-A93V-01	Responder	-0.29	0.02	0.6	0.14	3.32	-0.57	FALSE	-0.67	-0.29	0.05	-0.07	0	1.593937907	high
TCGA-NJ-A4YP-01	Responder	-0.29	0.76	0.83	0.16	-1.05	0.13	TRUE	-0.29	1.72	0.06	0.09	0	2.165937979	high
TCGA-35-3615-01	Responder	-0.28	-0.34	0.96	-0.56	-1.54	-0.27	FALSE	-1.79	-0.28	-0.06	-0.04	0.06	1.885552439	high
TCGA-73-A9RS-01	Responder	-0.24	-0.49	0.52	-0.58	0.75	-0.38	FALSE	-2.1	-0.24	0.08	-0.09	-0.01	3.030729317	high
TCGA-73-4666-01	Responder	-0.23	2.27	0.86	1.54	3.38	2.23	TRUE	-0.23	-1.28	0.03	-0.08	-0.06	3.804353588	high
TCGA-MP-A4TA-01	Responder	-0.22	0.76	0.61	0.51	-0.69	0.86	TRUE	-0.22	0.06	0.11	-0.07	-0.04	2.729547734	high
TCGA-55-5899-01	Responder	-0.21	0.55	0.4	0.4	-0.06	0.22	TRUE	-0.21	0.89	0.04	0.01	0.03	1.689619641	high
TCGA-55-6984-01	Responder	-0.21	-2.79	0.72	-1.39	-0.82	-1.47	FALSE	-0.19	-0.21	0.06	-0.08	0	2.458420221	high
TCGA-35-4123-01	Responder	-0.16	2.14	0.96	1.62	3.02	3.88	TRUE	-0.16	0	0.02	0.03	-0.04	2.442337466	high
TCGA-69-8253-01	Responder	-0.15	-1.91	0.75	-0.95	-1.4	-1.35	FALSE	-0.5	-0.15	-0.01	-0.04	0.03	1.27575063	high
TCGA-86-8673-01	Responder	-0.12	-0.65	0.46	-0.43	-1.97	0.51	TRUE	-0.12	0.97	0.07	0.03	-0.02	1.306213189	high
TCGA-05-4418-01	Responder	-0.12	0.98	0.99	0.89	0.98	1.04	TRUE	-0.12	-0.27	0.06	-0.04	-0.05	3.383371427	high
TCGA-49-4488-01	Responder	-0.11	0.58	0.95	0.26	0.63	1.2	FALSE	-1.36	-0.11	0.02	-0.03	0	1.63749656	high
TCGA-05-4425-01	Responder	-0.09	1.23	0.89	0.56	1.07	-0.35	FALSE	0.78	-0.09	-0.04	0.03	0	2.289693168	high
TCGA-05-4250-01	Responder	-0.08	1.38	0.56	0.77	2.61	1.77	TRUE	-0.08	1.58	0.13	0.03	-0.03	3.822172228	high
TCGA-05-5425-01	Responder	-0.06	2.12	0.35	1.36	0.71	2.39	TRUE	-0.06	-1.85	-0.04	-0.08	-0.05	1.287603418	high
TCGA-49-4507-01	Responder	-0.06	1.53	0.83	1.46	-1.52	2.51	TRUE	-0.06	0.21	0.1	-0.04	-0.04	3.916110156	high
TCGA-50-6592-01	Responder	-0.06	1.65	0.32	0.97	0.35	1.55	TRUE	-0.06	-0.16	0.05	0.01	-0.07	4.307778405	high
TCGA-97-8176-01	Responder	-0.05	-2.44	0.24	-1.08	-2.43	-1.03	FALSE	-0.78	-0.05	0.03	-0.08	0.04	3.777082193	high
TCGA-64-1680-01	Responder	-0.05	-0.52	0.97	-0.68	-1.21	-1.27	FALSE	-1.85	-0.05	0.01	-0.06	0.04	1.436947948	high

TCGA-55-1596-01	Responder	-0.04	-0.09	0.32	-0.39	-1.61	-0.43	FALSE	-1.45	-0.04	0.08	-0.09	0	1.390089797	high
TCGA-49-6761-01	Responder	-0.02	1.09	0.7	0.89	2.34	0.64	TRUE	-0.02	0.22	0.07	0	-0.04	2.47974124	high
TCGA-55-7281-01	Non-responder	0.02	1.03	0.31	0.19	-1.14	0.18	FALSE	1.43	0.02	-0.07	0.08	-0.01	1.627599249	high
TCGA-62-A46P-01	Non-responder	0.03	-2.05	0.59	-1.35	-2.34	-1.35	FALSE	-0.61	0.03	0.01	-0.06	0.04	1.815895652	high
TCGA-55-8302-01	Non-responder	0.05	0.76	0.45	0.51	2.96	-0.05	FALSE	0.88	0.05	0.07	-0.03	-0.04	2.232892133	high
TCGA-64-5779-01	Non-responder	0.05	-0.6	0.19	-0.29	-0.87	0.54	TRUE	0.05	0.98	0.04	0.05	0	1.445561909	high
TCGA-L9-A7SV-01	Non-responder	0.07	-0.76	0.12	-1.04	-1.46	-1.12	FALSE	-1.6	0.07	0.06	-0.11	0.05	1.2624201	high
TCGA-55-7815-01	Non-responder	0.08	0.22	0.5	-0.63	0.86	-1.04	FALSE	-2.11	0.08	-0.05	0.06	0	1.533659629	high
TCGA-05-4397-01	Non-responder	0.08	0.38	0.83	-0.14	-0.86	-0.88	FALSE	-1.7	0.08	0.08	-0.06	-0.01	1.463048297	high
TCGA-44-2662-01	Non-responder	0.08	-0.3	0.42	-0.04	2.61	-1.78	FALSE	-0.49	0.08	0.01	0.07	-0.06	1.814947979	high
TCGA-53-7624-01	Non-responder	0.1	1.55	0.64	0.34	1.15	0	FALSE	-1.03	0.1	0.12	-0.05	-0.06	3.183718859	high
TCGA-05-4382-01	Non-responder	0.1	0.74	0.27	0.74	0.84	-0.62	FALSE	0.85	0.1	-0.04	0.11	-0.06	1.543823647	high
TCGA-95-8494-01	Non-responder	0.11	-1.8	0.72	-1.15	0.9	-2.21	FALSE	-0.78	0.11	0.07	-0.05	-0.01	2.347151007	high
TCGA-55-6986-01	Non-responder	0.13	-1.47	0.82	-0.61	-0.46	-0.91	FALSE	-0.27	0.13	-0.02	-0.02	0.05	1.822475769	high
TCGA-55-7914-01	Non-responder	0.13	0.34	0.48	0.15	0.27	0.8	FALSE	-0.85	0.13	0.01	-0.02	0.02	2.06532757	high
TCGA-78-7160-01	Non-responder	0.14	1.14	0.5	0.79	0.27	1.66	TRUE	0.14	-1.22	-0.1	0.02	-0.02	2.602101124	high
TCGA-38-4632-01	Non-responder	0.14	0.51	0.88	0.35	1.17	0.75	FALSE	-1.01	0.14	0.02	-0.02	0	1.942870351	high
TCGA-91-6848-01	Non-responder	0.15	1.24	0.23	0.85	2.94	0.88	TRUE	0.15	2.24	0.13	0.1	-0.03	1.896982089	high
TCGA-55-8092-01	Non-responder	0.16	2.01	0.67	1.33	0.43	2.12	TRUE	0.16	-0.7	0.02	-0.02	-0.06	1.329106775	high
TCGA-75-5125-01	Non-responder	0.17	1.99	0.62	1.25	4.06	0.56	TRUE	0.17	-1.16	-0.01	-0.03	-0.05	2.518437667	high
TCGA-86-A4D0-01	Non-responder	0.18	-0.81	0.61	-0.94	-1.29	-1.16	FALSE	-1.92	0.18	0.09	-0.12	0.04	1.855622362	high
TCGA-38-4629-01	Non-responder	0.2	1.91	0.89	1.5	2.11	1.43	TRUE	0.2	1.56	0.08	0.12	-0.05	4.394383853	high
TCGA-05-4424-01	Non-responder	0.21	-0.07	0.13	0.21	-0.61	0.52	FALSE	0.23	0.21	-0.01	0.01	0.01	1.602870958	high
TCGA-86-8672-01	Non-responder	0.23	1.21	0.67	0.93	0.62	1.42	TRUE	0.23	0.93	0.06	0.07	-0.05	3.880104086	high
TCGA-55-6968-01	Non-responder	0.23	1.46	0.43	0.78	0.87	0.75	TRUE	0.23	-0.63	0.03	-0.07	-0.01	3.508082371	high
TCGA-55-7913-01	Non-responder	0.25	-1.06	0.19	-1.36	-2.19	-1.35	FALSE	-1.88	0.25	0.11	-0.11	0.02	1.419496109	high
TCGA-55-7907-01	Non-responder	0.28	1.02	0.17	0.47	-0.31	0.79	TRUE	0.28	0.01	-0.02	0.01	0.01	1.691528421	high
TCGA-49-AARO-01	Non-responder	0.29	0.26	0.32	0.47	0.64	0.38	FALSE	2.45	0.29	0	0.04	-0.01	1.456841957	high
TCGA-64-1676-01	Non-responder	0.29	1.25	0.96	0.71	1.91	1.3	FALSE	-1.71	0.29	0.01	0.04	-0.01	1.491768447	high
TCGA-78-7158-01	Non-responder	0.32	-1.48	0.51	-1.59	-0.92	-1.98	FALSE	-2.15	0.32	0.03	-0.07	0.07	1.394304666	high
TCGA-05-4396-01	Non-responder	0.34	-0.88	0.45	-1.13	-1.43	-1.99	FALSE	-1.41	0.34	0.02	-0.02	0.03	1.93069884	high
TCGA-50-5930-01	Non-responder	0.35	0.06	0.4	0.47	-0.84	0.88	TRUE	0.35	0.99	0.01	0.09	-0.02	1.33920875	high
TCGA-78-7535-01	Non-responder	0.35	-2.33	0.31	-1.32	-1.24	-1.71	FALSE	0.13	0.35	0.01	-0.03	0.04	4.0383967	high
TCGA-55-7994-01	Non-responder	0.38	1.9	0.71	1.5	3.62	1.79	TRUE	0.38	-2.72	-0.06	-0.14	-0.04	2.566286828	high
TCGA-86-7701-01	Non-responder	0.39	0.79	0.52	0.61	0.24	0.91	TRUE	0.39	-0.35	-0.01	0	-0.01	3.850390581	high
TCGA-J2-8194-01	Non-responder	0.41	-1.15	0.33	-1.14	-0.67	-2.11	FALSE	-1.28	0.41	-0.06	0.07	0.02	1.327243488	high

TCGA-55-8094-01	Non-responder	0.44	-2.17	0.12	-2.33	-2.82	-2.43	FALSE	-1.98	0.44	0.14	-0.12	0.01	2.936423211	high
TCGA-MP-A4SV-01	Non-responder	0.44	1.15	0.47	0.77	1.06	1.2	TRUE	0.44	-0.84	0.05	-0.1	-0.03	1.567920784	high
TCGA-MP-A4TF-01	Non-responder	0.44	0.57	0.61	-0.3	1.76	-0.51	FALSE	-1.44	0.44	0.15	-0.05	-0.07	2.005509984	high
TCGA-55-6969-01	Non-responder	0.44	1.15	0.46	1.2	0.22	1	TRUE	0.44	1.05	0.07	0.05	-0.03	1.656511555	high
TCGA-44-6779-01	Non-responder	0.44	1.96	0.54	1.33	1.06	1.82	TRUE	0.44	-0.2	0.02	0.05	-0.08	7.914491481	high
TCGA-55-7576-01	Non-responder	0.45	-0.49	0.27	-0.03	-0.01	0.31	FALSE	-0.45	0.45	0	0.02	0.01	2.208103349	high
TCGA-62-8398-01	Non-responder	0.47	0.19	0.56	-0.05	0.41	-0.77	FALSE	-0.21	0.47	0.07	-0.01	-0.02	3.450293519	high
TCGA-93-8067-01	Non-responder	0.49	-0.44	0.23	-0.86	-1.07	-1.86	FALSE	-1.34	0.49	0.08	-0.03	-0.01	2.072454386	high
TCGA-62-8394-01	Non-responder	0.49	0.34	0.22	-0.08	-0.85	0.15	FALSE	-0.24	0.49	0.1	-0.05	-0.01	1.30457973	high
TCGA-49-6745-01	Non-responder	0.5	0.77	0.58	0.58	3.68	1.11	TRUE	0.5	0.3	-0.01	0.07	-0.03	4.104876467	high
TCGA-62-A471-01	Non-responder	0.53	-0.89	0.79	-0.82	-1.49	-0.3	FALSE	-1.35	0.53	0.16	-0.09	-0.03	2.514488481	high
TCGA-78-7542-01	Non-responder	0.53	1.13	0.83	0.31	3.1	-0.29	FALSE	-0.97	0.53	0.17	-0.08	-0.06	4.608152604	high
TCGA-78-7166-01	Non-responder	0.53	-0.36	0.79	-0.84	-1.43	-1.22	FALSE	-1.05	0.53	0.11	-0.07	0.01	4.296117034	high
TCGA-78-7162-01	Non-responder	0.55	0.34	0.54	0.41	-0.69	0.61	TRUE	0.55	-0.51	-0.1	-0.01	0.06	1.375879149	high
TCGA-83-5908-01	Non-responder	0.55	3.24	0.34	1.94	4.18	2.76	TRUE	0.55	-0.37	0.08	-0.02	-0.09	1.94894298	high
TCGA-50-6590-01	Non-responder	0.58	2.02	0.59	1.44	2.85	1.15	TRUE	0.58	0.58	0.08	0.03	-0.05	1.978202068	high
TCGA-55-6980-01	Non-responder	0.58	0.5	0.24	0.54	0.35	-0.32	FALSE	1.89	0.58	-0.09	0.16	-0.01	1.472880859	high
TCGA-50-5939-01	Non-responder	0.6	-0.82	0.67	0.05	0.96	-1.78	FALSE	0.44	0.6	0.02	0.09	-0.06	4.385692999	high
TCGA-49-AARE-01	Non-responder	0.6	-0.1	0.34	-0.09	-1.89	-0.13	FALSE	0.33	0.6	0.08	-0.02	0	1.293505078	high
TCGA-50-5044-01	Non-responder	0.61	0.11	0.6	0.63	1.81	0.72	TRUE	0.61	3.18	0.16	0.11	0.01	3.578138766	high
TCGA-78-7633-01	Non-responder	0.61	-2.11	0.43	-1.91	-1.73	-3.05	FALSE	-1.86	0.61	-0.01	-0.02	0.07	1.38982139	high
TCGA-L9-A5IP-01	Non-responder	0.62	-1.32	0.54	-0.53	0.59	-0.11	FALSE	-0.93	0.62	0.11	-0.01	-0.05	2.292889473	high
TCGA-86-7711-01	Non-responder	0.62	-0.22	0.21	-0.13	-0.08	-0.11	FALSE	-1.46	0.62	0.02	0.1	-0.06	3.180550294	high
TCGA-05-4432-01	Non-responder	0.64	0.61	0.31	0.5	-0.14	0.88	TRUE	0.64	-0.23	0.01	-0.03	-0.01	1.483791482	high
TCGA-91-6830-01	Non-responder	0.65	0.82	0.34	0.48	0.29	0.75	TRUE	0.65	0.21	-0.01	0.06	-0.03	1.912816612	high
TCGA-78-7220-01	Non-responder	0.67	-0.63	0.44	-0.92	-0.76	0.57	FALSE	-1.99	0.67	0.1	-0.06	0.01	1.6667249	high
TCGA-86-8585-01	Non-responder	0.71	0.81	0.57	0.76	-0.1	1.22	TRUE	0.71	-0.92	0	-0.05	-0.04	1.613855348	high
TCGA-05-5428-01	Non-responder	0.71	-0.25	0.98	-1.18	-0.05	-0.06	FALSE	-3.12	0.71	0.04	-0.03	0.05	1.263676725	high
TCGA-69-7761-01	Non-responder	0.74	2.39	0.3	1.46	0.69	1.56	TRUE	0.74	-1.83	-0.07	-0.03	-0.05	1.716645479	high
TCGA-49-4494-01	Non-responder	0.74	0.77	0.87	0.16	0.06	0.99	FALSE	-1.13	0.74	0.09	-0.03	0	2.047446373	high
TCGA-91-8499-01	Non-responder	0.75	0.9	0.8	0.2	-0.93	-0.3	FALSE	-1.87	0.75	0.1	-0.07	0.03	1.275801984	high
TCGA-91-6836-01	Non-responder	0.76	-0.72	0.53	-0.69	1.69	-0.45	FALSE	-2.25	0.76	0.14	-0.06	-0.02	1.354348718	high
TCGA-44-A4SS-01	Non-responder	0.82	1.89	0.09	1.13	-0.03	1.11	TRUE	0.82	-0.68	-0.03	0.03	-0.05	1.547369015	high
TCGA-49-6742-01	Non-responder	0.83	-1.96	0.58	-1.36	-1.55	-1.35	FALSE	-0.88	0.83	0.11	-0.07	0.02	7.119846837	high
TCGA-44-7660-01	Non-responder	0.83	-0.1	0.2	-0.31	0.18	0.39	FALSE	-0.75	0.83	0.1	-0.06	0.02	2.994750843	high
TCGA-44-2656-01	Non-responder	0.84	2.36	0.81	1.86	2.76	2.68	TRUE	0.84	-2.75	-0.12	-0.04	-0.07	1.970795892	high

TCGA-95-7944-01	Non-responder	0.86	2.29	0.74	1.9	1.83	2.57	TRUE	0.86	-0.56	0.05	-0.01	-0.09	2.104814471	high
TCGA-78-7146-01	Non-responder	0.88	0.07	0.37	-0.62	0.8	-0.62	FALSE	-2.5	0.88	0.13	-0.02	-0.04	4.354357241	high
TCGA-78-7153-01	Non-responder	0.88	-1.38	0.64	-0.93	-3.64	-0.2	FALSE	-1.55	0.88	0.07	-0.07	0.07	1.586934339	high
TCGA-73-4659-01	Non-responder	0.88	-1.77	0.53	-0.83	-0.04	-1.51	FALSE	-0.06	0.88	-0.03	0.07	0.04	1.869365902	high
TCGA-05-4402-01	Non-responder	0.9	-0.27	0.46	-0.19	-0.5	-1.05	FALSE	0.24	0.9	0.02	0.05	0.01	2.000969417	high
TCGA-95-A4VN-01	Non-responder	0.93	1.67	0.53	1.36	4.32	1.64	TRUE	0.93	-1.73	0	-0.08	-0.07	1.457177627	high
TCGA-L4-A4E5-01	Non-responder	0.96	-0.5	0.25	-0.51	0.68	-0.72	FALSE	-0.87	0.96	0.11	-0.05	0.02	1.359093228	high
TCGA-55-8511-01	Non-responder	1.01	0.47	0.6	0.71	2.44	0.69	TRUE	1.01	0.17	0	0.06	-0.03	1.463519872	high
TCGA-78-7159-01	Non-responder	1.02	-0.11	0.27	-0.63	-0.93	0.7	FALSE	-1.13	1.02	0.08	0.01	0	1.415097872	high
TCGA-62-A46O-01	Non-responder	1.03	-2.06	0.63	-2.46	-1.99	-2.16	FALSE	-3.35	1.03	0.15	-0.08	0.01	3.136703948	high
TCGA-05-5429-01	Non-responder	1.04	-2.06	0.97	-1.55	-3.05	-0.95	FALSE	-1.43	1.04	0.07	-0.01	0.03	4.230303045	high
TCGA-44-8117-01	Non-responder	1.07	0.05	0.34	-0.62	0.74	-0.9	FALSE	-0.96	1.07	0.03	0.05	0.01	1.570062058	high
TCGA-55-8205-01	Non-responder	1.08	2.42	0.73	1.58	3.53	2.08	TRUE	1.08	-1.94	-0.03	-0.05	-0.08	3.098079255	high
TCGA-38-4625-01	Non-responder	1.1	1.01	0.87	0.53	4.4	0.21	FALSE	-1.42	1.1	0.13	0	-0.04	1.552205748	high
TCGA-50-5051-01	Non-responder	1.1	-2.53	0.59	-1.56	-1.65	-0.84	FALSE	-0.49	1.1	0.08	-0.02	0.04	1.755346569	high
TCGA-44-7670-01	Non-responder	1.1	-0.77	0.06	-0.76	-0.61	0.65	FALSE	-0.48	1.1	0.14	-0.07	0.02	1.851657123	high
TCGA-55-6978-01	Non-responder	1.11	2.91	0.4	1.92	3.38	2.56	TRUE	1.11	0.71	0.02	0.14	-0.09	1.828827655	high
TCGA-55-7995-01	Non-responder	1.11	2.18	0.26	1.53	1.26	2.27	TRUE	1.11	-2.68	-0.06	-0.1	-0.06	1.52737692	high
TCGA-44-6145-01	Non-responder	1.11	1.79	0.5	1.45	1.8	2.15	TRUE	1.11	-1.26	-0.06	0.05	-0.09	1.885357312	high
TCGA-49-4505-01	Non-responder	1.14	1.37	0.75	0.87	0.51	1.42	TRUE	1.14	-0.42	-0.06	0.05	-0.02	1.398329536	high
TCGA-50-6594-01	Non-responder	1.15	-1.81	0.54	-0.86	4.64	-2.59	FALSE	-1.44	1.15	0.08	0.01	0.01	2.80841214	high
TCGA-95-7043-01	Non-responder	1.15	-2.09	0.03	-1.49	-2.78	-1.11	FALSE	-2.01	1.15	0.07	-0.04	0.07	2.831648188	high
TCGA-38-4631-01	Non-responder	1.17	-0.1	0.58	-0.49	-2.13	0.14	FALSE	-1.6	1.17	0.15	-0.04	-0.02	1.587539724	high
TCGA-69-7763-01	Non-responder	1.19	-1.39	0.4	-0.58	-0.64	-1.02	FALSE	0.75	1.19	-0.05	0.14	0.02	1.362669152	high
TCGA-55-6712-01	Non-responder	1.19	1.8	0.68	1.52	1.74	2.3	TRUE	1.19	-0.67	-0.02	0.01	-0.05	5.102625989	high
TCGA-73-4675-01	Non-responder	1.2	-2.49	0.8	-1.41	-2.56	-1.52	FALSE	0.36	1.2	-0.03	0.09	0.05	2.789197219	high
TCGA-97-A4M3-01	Non-responder	1.22	-1.86	0.3	-1.43	-1.75	-1.91	FALSE	-0.73	1.22	0	0.07	0.03	1.879483274	high
TCGA-78-7145-01	Non-responder	1.23	-1.73	0.37	-1.52	-0.05	-3.2	FALSE	-2.2	1.23	0.06	0.07	-0.02	2.434571094	high
TCGA-73-4658-01	Non-responder	1.23	0.28	0.56	0.53	3.01	0.28	FALSE	0.99	1.23	-0.05	0.18	-0.02	1.296242026	high
TCGA-86-8674-01	Non-responder	1.26	-1.56	0.63	-1.79	-1.76	-2.22	FALSE	-2.07	1.26	0.06	-0.01	0.05	1.951567566	high
TCGA-55-6982-01	Non-responder	1.28	1.23	0.28	0.52	0.3	-0.37	FALSE	0.25	1.28	0	0.16	-0.03	1.291281487	high
TCGA-55-8204-01	Non-responder	1.31	0.69	0.53	0.1	0.58	-0.1	FALSE	0.01	1.31	0.07	0.06	-0.01	1.579315074	high
TCGA-55-A490-01	Non-responder	1.31	-1.43	0.06	-1.18	-1.26	-1.22	FALSE	0.24	1.31	0.06	0.05	0	1.947181505	high
TCGA-44-A479-01	Non-responder	1.31	1.95	0.27	1.35	0.82	1.59	TRUE	1.31	-0.91	-0.04	-0.01	-0.03	1.359197645	high
TCGA-73-4676-01	Non-responder	1.32	-1.43	0.69	-1.03	1.37	-0.26	FALSE	-2.49	1.32	0.08	0.01	0.02	5.285918972	high
TCGA-78-8660-01	Non-responder	1.33	1.23	0.49	0.94	1.02	1.11	TRUE	1.33	-1.25	0	-0.11	0	1.324476888	high

TCGA-55-A493-01	Non-responder	1.33	1.23	0.29	0.95	2.76	0.79	TRUE	1.33	0.22	0.09	-0.01	-0.06	2.263477866	high
TCGA-NJ-A4YQ-01	Non-responder	1.34	1.44	0.63	1.46	0.33	2.29	TRUE	1.34	-2.27	-0.07	-0.11	-0.02	1.825071539	high
TCGA-44-3396-01	Non-responder	1.34	1.13	0.75	1.05	2.2	1.35	TRUE	1.34	0.93	0.02	0.11	-0.05	1.648012297	high
TCGA-69-7765-01	Non-responder	1.35	0.29	0.38	0.11	-0.03	-0.17	FALSE	-0.43	1.35	-0.03	0.19	-0.03	1.307270535	high
TCGA-86-7955-01	Non-responder	1.36	-1.63	0.65	-1.85	-1.58	-1.92	FALSE	-2.85	1.36	0.17	-0.1	0.04	2.500721518	high
TCGA-50-5933-01	Non-responder	1.39	0.83	0.53	0.12	2.98	-0.64	FALSE	-0.63	1.39	0.02	0.16	-0.05	1.691477798	high
TCGA-50-5936-01	Non-responder	1.41	-1.27	0.43	-0.69	-0.96	-1.62	FALSE	-0.86	1.41	0.05	0.07	0	2.995759072	high
TCGA-49-6743-01	Non-responder	1.43	-0.03	0.26	-0.01	1.13	-0.26	FALSE	-0.63	1.43	0.07	0.07	-0.02	1.687584406	high
TCGA-62-8399-01	Non-responder	1.43	-0.95	0.1	-0.76	0.38	-0.45	FALSE	-0.32	1.43	0.07	0.03	0.01	1.548698001	high
TCGA-44-7667-01	Non-responder	1.45	-0.51	0.31	-1.22	-1.31	-2.01	FALSE	-1.31	1.45	0.14	-0.04	0.03	1.825544047	high
TCGA-78-7154-01	Non-responder	1.47	-0.47	0.17	-0.84	-1.22	-0.97	FALSE	-1.78	1.47	0.12	-0.03	0.03	2.736472386	high
TCGA-91-6831-01	Non-responder	1.49	0.59	0.41	0.13	-0.04	0.46	FALSE	-0.86	1.49	0.09	0.06	-0.02	2.11150434	high
TCGA-55-6983-01	Non-responder	1.57	-0.66	0.49	0.08	-1.02	0.78	TRUE	1.57	0.12	-0.06	0.03	0.04	2.026190464	high
TCGA-55-8089-01	Non-responder	1.58	3.31	0.69	2.17	1.87	2.26	TRUE	1.58	-2.08	-0.06	-0.02	-0.09	3.804920283	high
TCGA-44-7661-01	Non-responder	1.61	0.89	0.48	0.74	3.92	-0.09	FALSE	0.95	1.61	0.08	0.09	-0.03	2.409083212	high
TCGA-99-8025-01	Non-responder	1.67	-0.9	0.3	-0.87	-0.88	-1.83	FALSE	-0.16	1.67	0.07	0.05	0.03	1.688040258	high
TCGA-75-6214-01	Non-responder	1.74	-0.39	0.35	-0.52	0.49	-0.97	FALSE	-1.41	1.74	0.16	-0.01	0	2.26263759	high
TCGA-38-4630-01	Non-responder	1.75	-0.05	0.4	-0.55	-1.19	-0.14	FALSE	-1.41	1.75	0.12	0	0.04	1.614352391	high
TCGA-86-8074-01	Non-responder	1.75	0.62	0.23	0.05	0.27	-0.96	FALSE	-1.1	1.75	0.06	0.13	-0.03	1.548047628	high
TCGA-55-A494-01	Non-responder	1.76	-3.97	0.12	-2.93	-2.81	-0.88	FALSE	-1.45	1.76	0.12	-0.05	0.08	1.918814013	high
TCGA-69-7973-01	Non-responder	1.77	-1.41	0.21	-0.93	-0.79	0.89	FALSE	-0.74	1.77	0.1	0.01	0.04	1.871404068	high
TCGA-50-6593-01	Non-responder	1.78	-1.37	0.3	-0.72	0.89	-1.69	FALSE	0.58	1.78	-0.02	0.13	0.05	1.42766694	high
TCGA-50-5049-01	Non-responder	1.78	2.58	0.61	1.94	1.05	3.11	TRUE	1.78	-0.57	-0.1	0.09	-0.03	1.51370855	high
TCGA-05-4434-01	Non-responder	1.78	1.79	0.79	1.46	1.99	0.6	TRUE	1.78	-1.1	-0.03	-0.01	-0.05	2.03176943	high
TCGA-50-5072-01	Non-responder	1.78	-0.91	0.74	-0.67	-0.52	-1.23	FALSE	-0.96	1.78	0.13	0.02	0.01	6.003839502	high
TCGA-55-8299-01	Non-responder	1.79	2	0.63	1.6	1.32	2.38	TRUE	1.79	0.32	-0.02	0.14	-0.08	2.224878194	high
TCGA-99-8032-01	Non-responder	1.79	-1.44	0.32	-0.74	-0.92	-0.54	FALSE	0.31	1.79	0.06	0.07	0.03	1.738173043	high
TCGA-49-AAR3-01	Non-responder	1.82	2.75	0.28	1.93	2.62	2.44	TRUE	1.82	-0.05	0.04	0.02	-0.06	2.671644588	high
TCGA-55-6981-01	Non-responder	1.86	-1.54	0.12	-1.11	0.16	-2.86	FALSE	-0.59	1.86	0.06	0.06	0.04	1.895927559	high
TCGA-95-7039-01	Non-responder	1.9	-0.33	0.24	-0.15	0.37	0.06	FALSE	-0.38	1.9	0.09	0.05	0.02	2.092528996	high
TCGA-50-5941-01	Non-responder	1.9	2.32	0.29	1.72	1.61	1.78	TRUE	1.9	-2.1	-0.1	-0.03	-0.06	1.372915976	high
TCGA-55-6979-01	Non-responder	1.95	2.8	0.29	1.76	2.23	1.83	TRUE	1.95	-1.34	-0.07	0.02	-0.07	1.558158104	high
TCGA-64-5774-01	Non-responder	1.97	-1.57	0.36	-1.33	-0.65	1.01	FALSE	-1.3	1.97	0.15	0	0.02	2.321820556	high
TCGA-64-5815-01	Non-responder	1.97	-0.61	0.39	-0.16	0.16	-1.12	FALSE	1.19	1.97	-0.02	0.24	-0.04	2.135594705	high
TCGA-55-A48Y-01	Non-responder	2.02	-0.48	0.29	-0.36	-1.52	-0.84	FALSE	-1.59	2.02	0.05	0.12	0.01	2.057955627	high
TCGA-86-6562-01	Non-responder	2.03	-1.46	0.27	-0.77	0.14	-1.94	FALSE	-0.43	2.03	0.02	0.16	0	2.031544227	high

TCGA-44-7671-01	Non-responder	2.06	-2.83	0.48	-1.72	-1.71	-2.11	FALSE	-0.98	2.06	0	0.1	0.08	1.934928826	high
TCGA-86-8075-01	Non-responder	2.06	-0.17	0.11	-0.27	0.67	-2.06	FALSE	0.09	2.06	0.03	0.2	-0.04	1.340187961	high
TCGA-49-6744-01	Non-responder	2.06	0.67	0.49	1.12	0.08	1.37	TRUE	2.06	-0.39	-0.11	0.11	-0.03	1.640749864	high
TCGA-69-7978-01	Non-responder	2.08	2.04	0.28	1.86	3.42	1.66	TRUE	2.08	-1.36	-0.08	0.05	-0.08	1.707285593	high
TCGA-44-8120-01	Non-responder	2.12	-1.75	0.14	-0.88	-0.57	-1.37	FALSE	-0.13	2.12	-0.01	0.13	0.06	2.031629321	high
TCGA-49-4490-01	Non-responder	2.15	-0.86	0.14	-0.78	-2.55	-0.65	FALSE	0.71	2.15	0.09	0.06	0.03	2.24690131	high
TCGA-73-4670-01	Non-responder	2.15	-1.25	0.81	-1.18	-0.49	-1.63	FALSE	-2.1	2.15	0.14	0.07	-0.03	4.283382523	high
TCGA-69-7760-01	Non-responder	2.18	-1.81	0.19	-1.96	-1.28	-3.29	FALSE	-2.55	2.18	0.13	0.06	0	1.775257784	high
TCGA-MN-A4N1-01	Non-responder	2.19	-2.64	0.27	-1.97	-1.42	-3.05	FALSE	-0.8	2.19	0.13	0.01	0.05	1.423290527	high
TCGA-55-7570-01	Non-responder	2.19	-1.14	0.38	-1.12	-1.38	1.52	FALSE	-1.86	2.19	0.15	-0.02	0.06	5.409757661	high
TCGA-MP-A4TC-01	Non-responder	2.23	1.39	0.57	0.78	2.32	-0.13	FALSE	0.77	2.23	0.08	0.19	-0.07	3.331661962	high
TCGA-49-AAR9-01	Non-responder	2.27	-0.18	0.16	-0.58	-1.99	-0.86	FALSE	-1.65	2.27	0.2	0	-0.01	3.888140294	high
TCGA-44-8119-01	Non-responder	2.29	0.21	0.41	0.08	-0.17	-0.14	FALSE	0.19	2.29	0.1	0.11	-0.02	2.681938329	high
TCGA-MP-A4SY-01	Non-responder	2.33	-1.19	0.52	-0.72	1.13	-2.45	FALSE	-0.67	2.33	0.05	0.16	0	2.077888976	high
TCGA-55-8505-01	Non-responder	2.34	-2.52	0.39	-1.57	-2.13	-2.46	FALSE	-0.35	2.34	0.1	0.1	0	2.228057878	high
TCGA-MP-A4T4-01	Non-responder	2.5	2.35	0.63	1.9	3.06	1.98	TRUE	2.5	-1.06	-0.04	0.04	-0.08	1.344789183	high
TCGA-71-8520-01	Non-responder	2.5	-1.15	0.12	-0.91	-0.13	-1.76	FALSE	0.07	2.5	0.07	0.15	0	1.573496239	high
TCGA-44-7662-01	Non-responder	2.52	0.59	0.16	0.29	1.09	-0.1	FALSE	0.31	2.52	0.1	0.18	-0.05	1.550082368	high
TCGA-49-AAR4-01	Non-responder	2.6	0.79	0.45	1.27	0.82	3.53	TRUE	2.6	-0.68	0	-0.05	-0.02	1.268964627	high
TCGA-86-8279-01	Non-responder	2.64	-0.78	0.21	-1.27	-1.82	-2.68	FALSE	-0.97	2.64	0.07	0.13	0.04	1.28435347	high
TCGA-44-7672-01	Non-responder	2.78	1.14	0.44	1.21	1.14	0.72	TRUE	2.78	0.06	-0.03	0.1	-0.05	1.568253459	high
TCGA-MP-A4T8-01	Non-responder	2.81	-2.62	0.26	-2.06	-1.96	-2.91	FALSE	-1.21	2.81	0.13	0.09	0.02	1.644260098	high
TCGA-J2-A4AD-01	Non-responder	2.85	-2.73	0.54	-1.74	-1.48	-1.42	FALSE	-1.92	2.85	0.12	0.06	0.06	2.282424225	high
TCGA-MP-A4TI-01	Non-responder	2.92	2.37	0.54	1.81	2.39	1.56	TRUE	2.92	-0.73	-0.03	0.07	-0.09	1.702143511	high
TCGA-78-7150-01	Non-responder	2.94	-0.9	0.34	-1.14	-1.69	-0.93	FALSE	-1.93	2.94	0.15	0.11	-0.02	5.856780989	high
TCGA-73-4668-01	Non-responder	2.94	-1.43	0.14	-0.93	-0.34	-0.86	FALSE	-1.28	2.94	0.12	0.13	0.01	1.797648307	high
TCGA-50-5931-01	Non-responder	2.98	-1.58	0.44	-1.98	-3.06	-0.45	FALSE	-1.82	2.98	0.18	0.04	0.04	2.44733327	high
TCGA-86-8055-01	Non-responder	2.99	0.06	0.19	-0.05	0.35	-0.85	FALSE	0.17	2.99	0.07	0.22	-0.02	2.284075637	high
TCGA-75-7027-01	Non-responder	3.04	-0.89	0.21	-0.85	-1.47	-1.08	FALSE	-1.6	3.04	0.13	0.11	0.02	2.508710258	high
TCGA-64-1679-01	Non-responder	3.1	-0.58	0.09	-0.63	0.62	-2.62	FALSE	0.5	3.1	0.07	0.21	-0.01	1.471962642	high
TCGA-50-6595-01	Non-responder	3.19	1.94	0.28	1.11	2.94	0.04	FALSE	0.34	3.19	0.17	0.18	-0.07	3.66545671	high
TCGA-91-6829-01	Non-responder	3.22	-1.45	0.05	-0.77	0.24	-0.38	FALSE	-1.15	3.22	0.06	0.21	0.01	1.481783313	high
TCGA-64-5775-01	Non-responder	3.24	-2.34	0.3	-1.47	3.17	-1.94	FALSE	-1.44	3.24	0.17	0.11	0	4.982189339	high
TCGA-38-4627-01	Non-responder	3.46	-0.09	0.52	0.04	-0.62	-0.43	FALSE	1.07	3.46	-0.01	0.3	0.02	1.392060804	high
TCGA-55-6975-01	Non-responder	3.6	-1.71	0.3	-1.01	-2.58	-0.64	FALSE	-0.77	3.6	0.14	0.17	0.01	4.329442771	high
TCGA-55-7726-01	Non-responder	3.63	-0.26	0.13	-0.86	-1.01	-2.08	FALSE	-1.11	3.63	0.12	0.22	-0.02	1.695455914	high

Clinical characteristics of IMvigor210 cohort(Validation cohort 4).

Patient id	Sex	Stage	Respon	Tissue	OS(month	Status	Metabo	Group
SAM025b45c27e05	M	III	SD/PD	bladder	8.77207	1	12.8	low
SAM032c642382a7	F	IV	SD/PD	bladder	2.49692	1	46.61	high
SAM0a0f2bac4b20	M	III	SD/PD	kidney	1.18275	1	84.27	high
SAM0a7c2091dd56	M	III	SD/PD	bladder	2.62834	1	51.12	high
SAM0ce9c983b20f	M	III	SD/PD	ureter	6.6037	1	42.96	high
SAM0d855cff64e6	F	IV	SD/PD	bladder	2.85832	1	29.74	high
SAM14df63a65411	F	III	CR/PR	bladder	21.4209	0	-9.36	low
SAM166a419a4e5a	M	IV	SD/PD	bladder	5.38809	1	12.16	low
SAM181b638b8248	M	IV	SD/PD	kidney	10.8747	1	32.08	high
SAM18b9351e265a	M	III	SD/PD	ureter	15.8686	1	26.85	high
SAM18be5b395318	M	IV	SD/PD	bladder	9.00205	1	50.44	high
SAM19fec8f3b3bd	M	III	SD/PD	bladder	9.88912	1	37.48	high
SAM1a87df750b9d	M	III	SD/PD	bladder	5.38809	1	33.3	high
SAM1c0ecfb3eb63	F	III	SD/PD	bladder	5.84805	1	18.91	high
SAM1f66db567eb5	M	III	CR/PR	bladder	16.5914	0	-12.3	low
SAM25510f300d79	M	III	CR/PR	other	20.6982	0	-22.5	low
SAM2570ff4aae6e	F	III	CR/PR	lymph node	22.3737	0	9.332	low
SAM26104d5adc89	M	III	SD/PD	bladder	5.38809	1	17.15	high
SAM28687037e4ff	M	III	SD/PD	kidney	21.5852	0	30.14	high
SAM297c0301e861	M	IV	SD/PD	bladder	8.08214	1	47.65	high
SAM29da928587ad	F	III	SD/PD	ureter	2.59548	1	7.067	low
SAM2dc578e0165f	M	IV	NA	bladder	1.97125	1	17.18	high
SAM2e9ac0b1b250	M	IV	CR/PR	bladder	22.1437	0	1.673	low
SAM2f228939632f	F	IV	SD/PD	bladder	7.88501	1	17.06	high
SAM30b5c6c54cf7	F	IV	CR/PR	bladder	9.26489	1	25.7	high
SAM3330c03fdf00	M	IV	SD/PD	lymph node	19.1869	0	12.57	low
SAM34430ef08e5b	M	IV	SD/PD	bladder	18.1684	0	18.16	high
SAM36a9225b0222	M	IV	SD/PD	bladder	23.8193	0	33.11	high
SAM36d87392593b	M	III	SD/PD	ureter	23.6879	0	94.69	high
SAM39eb94fa504d	M	III	SD/PD	bladder	17.9055	1	-1.06	low
SAM3b1066e5801b	M	IV	SD/PD	lymph node	15.6386	0	0.484	low
SAM3b15b4c6311d	M	III	CR/PR	bladder	21.4538	0	57.35	high
SAM3cb94b0d5297	M	IV	SD/PD	kidney	5.05955	1	15.98	high
SAM415f36ad349e	M	IV	SD/PD	bladder	17.9713	1	16.61	high

SAM4501e41e4751	M	III	SD/PD	bladder	1.11704	1	33.64	high
SAM45c8e6412c66	M	III	SD/PD	bladder	3.48255	1	29.68	high
SAM468a9e1dc821	M	IV	SD/PD	NA	3.8768	1	-3.01	low
SAM47fc46c3d6be	M	IV	SD/PD	bladder	18.7269	0	8.823	low
SAM4caabd64e7fd	M	III	CR/PR	ureter	15.7043	0	-2.32	low
SAM4edbe45817b3	M	III	NA	bladder	1.05133	1	42.18	high
SAM548551ef782c	M	IV	CR/PR	bladder	17.2813	0	97.34	high
SAM553c3c35b847	F	IV	SD/PD	kidney	6.70226	1	19.42	high
SAM560f23d6a3ad	M	IV	CR/PR	other	21.3881	0	-18	low
SAM563d6233dfa2	M	IV	SD/PD	bladder	2.13552	1	19.17	high
SAM568ce160abd9	F	III	SD/PD	bladder	12.846	1	2.621	low
SAM572f19794c96	M	III	CR/PR	kidney	16.8214	0	64.2	high
SAM5767dd75d142	M	IV	CR/PR	other	18.1684	0	7.1	low
SAM59b825252c0d	M	III	NA	lymph node	0.52567	1	17.23	high
SAM59fda9035d1d	M	IV	SD/PD	NA	3.74538	1	0.029	low
SAM5c139c5c1c4f	M	III	SD/PD	bladder	7.22793	1	26.9	high
SAM5d989c86255e	F	III	SD/PD	bladder	3.44969	1	18.1	high
SAM5fc9ae0aed1f	M	IV	NA	bladder	0.62423	1	55.73	high
SAM62fb1388c871	F	IV	SD/PD	kidney	22.5051	0	18.11	high
SAM65afda25b920	M	III	SD/PD	bladder	8.08214	1	11.42	low
SAM6780ed436b55	F	III	SD/PD	bladder	6.2423	1	31.79	high
SAM698d8d76b934	F	IV	NA	bladder	0.4271	1	121.7	high
SAM6cbc10abddb0	M	III	SD/PD	bladder	5.94661	1	47.46	high
SAM6dd7ad1d797d	M	III	SD/PD	bladder	19.2854	1	35.51	high
SAM714285adf612	M	IV	NA	kidney	0.62423	1	45.49	high
SAM73b653ae20d1	F	III	NA	other	1.80698	1	64.33	high
SAM7538ad9ff524	F	III	SD/PD	bladder	19.1211	0	-5.62	low
SAM75f12d1a55fc	M	IV	SD/PD	lung	14.7515	0	-1.92	low
SAM76a431ba6ce1	M	III	SD/PD	kidney	14.1273	1	-6.02	low
SAM771445e92421	M	III	CR/PR	bladder	19.4168	0	21.33	high
SAM7829a341b9f3	M	IV	SD/PD	bladder	3.8768	1	16.34	high
SAM7893196e0e89	M	III	SD/PD	bladder	2.66119	1	8.864	low
SAM7b40007f4aa4	F	IV	CR/PR	ureter	21.6509	0	15.68	low
SAM7bff231634e9	M	III	SD/PD	ureter	10.2505	1	67.3	high
SAM7d2dfba6cd84	M	IV	SD/PD	lymph node	12.7146	1	-7.43	low
SAM7fb6987514a4	M	III	SD/PD	bladder	22.8337	0	28.01	high

SAM80c6183220e6	M	III	SD/PD	kidney	14.7515	0	12.42	low
SAM85e41e7f33f9	M	IV	NA	bladder	0.62423	1	51.43	high
SAM85f0a3ac1c45	M	III	SD/PD	lung	5.84805	1	23.1	high
SAM8884fe446d20	M	III	CR/PR	bladder	23.1622	0	54.56	high
SAM8a42c0d59187	M	III	NA	NA	2.82546	1	26.88	high
SAM8e8ef2368dfa	M	IV	SD/PD	kidney	4.50103	1	-0.58	low
SAM9410b866974a	M	IV	SD/PD	bladder	6.73511	1	17.57	high
SAM943df5cf15df	M	III	SD/PD	bladder	2.13552	1	25.57	high
SAM9681450bbc90	M	III	NA	bladder	4.96099	1	36.65	high
SAM9cafb905b36a	M	IV	SD/PD	kidney	7.62218	1	18.05	high
SAM9daccafc18db	F	III	SD/PD	kidney	16.46	0	19.72	high
SAM9e11ec6bea80	M	IV	SD/PD	bladder	15.4086	1	54	high
SAM9eebdef2858a	F	IV	NA	bladder	0.82136	1	33.99	high
SAM9fb814c22bdb	F	III	SD/PD	bladder	19.1211	1	7.1	low
SAMa90d73f8d891	M	III	SD/PD	other	1.01848	1	7.209	low
SAMa9ca8536d2b1	M	III	SD/PD	bladder	21.848	0	2.774	low
SAMab8052a03398	M	III	SD/PD	lymph node	13.306	1	-2.77	low
SAMabc151b01ea3	F	IV	SD/PD	bladder	0.55852	1	1.934	low
SAMaec7380f9ab0	M	IV	CR/PR	ureter	20.8624	0	56.36	high
SAMb15ad09d6e24	F	IV	SD/PD	bladder	3.8768	1	19.92	high
SAMb2e4a082541a	F	IV	SD/PD	bladder	4.36961	1	-0.14	low
SAMb2f1d0e54ece	M	IV	CR/PR	bladder	11.4004	1	13.31	low
SAMb419a8fcbfcd	M	IV	CR/PR	bladder	22.7351	0	0.247	low
SAMb8101c538753	M	IV	SD/PD	bladder	13.4045	1	27.34	high
SAMb8f13a0525a6	M	IV	NA	kidney	2.69405	1	89.26	high
SAMb963dda93cfd	M	IV	SD/PD	bladder	2.75975	1	40.15	high
SAMba1a34b5a060	M	III	SD/PD	bladder	12.846	1	7.954	low
SAMbcb07ba81cee	M	III	SD/PD	bladder	2.52977	1	10.42	low
SAMbe25e2c88f3e	M	IV	NA	lymph node	2.92402	1	29.15	high
SAMbfdffb97c446	F	IV	SD/PD	bladder	6.70226	1	56.65	high
SAMc0da5d48686d	M	IV	SD/PD	bladder	9.49487	1	53.63	high
SAMc1251c7bfee2	M	IV	CR/PR	bladder	21.6181	0	66.99	high
SAMc6eff056c89a	M	III	SD/PD	lymph node	1.80698	1	6.79	low
SAMc919aebc7fdd	M	IV	CR/PR	bladder	20.731	0	12.27	low
SAMcabb6d58ff55	F	IV	SD/PD	lung	20.271	0	-12.7	low
SAMce39dd79b441	M	IV	SD/PD	bladder	6.27515	1	28.62	high

SAMd027124354ce	F	III	CR/PR	ureter	23.5236	0	16.53	high
SAMd135d5867fe3	M	IV	SD/PD	bladder	3.61396	1	105.3	high
SAMd1bd63734394	M	III	SD/PD	bladder	1.9384	1	5.125	low
SAMd2492b2a31bb	F	III	SD/PD	lymph node	7.85216	1	19.8	high
SAMd4c0837b0997	M	III	SD/PD	kidney	5.38809	1	22.39	high
SAMd636e3461955	M	IV	SD/PD	bladder	10.4805	1	9.848	low
SAMd7d57ee3a863	M	IV	CR/PR	bladder	20.271	0	2.34	low
SAMd98bac0a070f	M	III	SD/PD	ureter	2.06982	1	6.035	low
SAMda4d892fddc8	M	IV	SD/PD	kidney	14.1273	0	6.677	low
SAMdb3f50c9129c	M	IV	SD/PD	bladder	15.8029	1	36.78	high
SAMdf3e42c8672a	M	III	SD/PD	bladder	7.32649	1	8.731	low
SAMe0c49ea0df5d	F	III	SD/PD	bladder	4.13963	1	11.3	low
SAMe3210d3632b4	F	III	SD/PD	NA	2.16838	1	15.91	high
SAMe41b1e773582	M	III	SD/PD	bladder	0.85421	1	21.78	high
SAMe56c96c51190	F	III	SD/PD	kidney	9.7577	1	7.737	low
SAMe7bf6c015192	M	III	SD/PD	kidney	3.12115	1	16.98	high
SAMe7e4f7c076a7	M	IV	SD/PD	bladder	1.97125	1	39.74	high
SAMe9475f77504b	M	IV	SD/PD	bladder	18.6612	0	70.02	high
SAMe9ae8beb82fa	M	III	SD/PD	bladder	5.48665	1	-18.9	low
SAMef0e3d2415fd	M	IV	SD/PD	lung	10.1191	1	-11.4	low
SAMf2aae1443f67	F	III	SD/PD	bladder	10.3491	1	6.77	low
SAMfb7aec7cb0e2	M	IV	CR/PR	bladder	22.538	0	75.72	high
SAMffa5c7cad0e5	M	IV	CR/PR	other	15.54	1	11.92	low
