# Supplementary material for: Insight of a Metabolic Prognostic Model to Identify Tumor Environment and Drug Vulnerability for Lung Adenocarcinoma
Source: Front Immunol. 2022 Jun 23;13:872910. doi: 10.3389/fimmu.2022.872910 (PMC9262104; doi:10.3389/fimmu.2022.872910)
Supplement: Supplementary file 13 [file DataSheet_12.pdf]

Supplementary Table S12: The DEIRGs between high- and low-Metabolism Score group.

gene	conMean	treatMean	logFC	pValue	fdr
ANGPT4	1.19178559	0.75821918	-0.6524379	2.69E-05	0.00010088
ANGPTL5	1.52361036	0.92174612	-0.7250527	3.93E-08	3.17E-07
ANGPTL7	1.52181982	0.86959406	-0.8073836	6.99E-07	4.02E-06
AZU1	2.53817072	1.38767626	-0.8711181	2.37E-10	3.71E-09
CCL16	1.06497027	0.6289895	-0.7597053	1.45E-05	5.89E-05
CMA1	1.28159009	0.6919758	-0.8891414	1.23E-06	6.55E-06
CMTM5	0.85925721	0.53474018	-0.684252	1.47E-05	5.97E-05
CNTFR	2.64642613	1.4236484	-0.8944525	1.62E-13	6.67E-12
CRHR2	1.45715541	0.95189863	-0.6142749	0.0003763	0.00106866
EDN3	1.36977387	0.72149452	-0.9248774	4.16E-07	2.49E-06
ELANE	2.16741351	1.09306393	-0.9875966	3.77E-11	8.00E-10
FGF10	0.67936892	0.400321	-0.7630379	0.00113245	0.00274346
FGF17	1.93002568	1.28692968	-0.5846868	1.09E-08	9.78E-08
FGF5	0.89813739	1.64555479	0.87356601	2.23E-06	1.10E-05
GAL	2.30533243	3.4765895	0.59269792	1.02E-05	4.33E-05
GDF7	1.0401045	0.59894155	-0.7962414	8.67E-08	6.38E-07
GLP1R	2.15104505	1.25434749	-0.7781007	8.02E-08	6.11E-07
HRG	0.54632162	1.10880594	1.02118446	6.87E-06	3.06E-05
HTR3C	0.89992568	0.55953151	-0.6855865	0.0059187	0.01179379
IL2	0.82558649	0.53705251	-0.6203562	0.00019894	0.00061502
INSL4	0.75995315	2.7633758	1.86244938	3.62E-10	5.44E-09
LBP	1.54214099	2.52213973	0.70971353	0.00159811	0.00371861
LCNL1	1.89202928	1.13252329	-0.7403937	1.15E-10	2.13E-09
NMBR	0.54202342	0.31708904	-0.7734672	0.00064212	0.00170287
NR0B1	1.24195721	2.6188137	1.07629797	4.11E-05	0.00015034
PAK7	1.17005811	0.75367078	-0.6345738	0.00012705	0.00040913
PENK	4.37077162	2.78225434	-0.6516337	8.39E-14	4.32E-12
RAET1L	0.64754144	1.03364932	0.67470238	0.00070329	0.00183807
RXRG	3.66757838	2.40058356	-0.6114426	2.92E-11	6.32E-10
S100A7	0.96999369	1.59375845	0.71638572	0.00100449	0.00247575
SLC10A2	1.45648108	0.66913836	-1.1221105	8.65E-07	4.92E-06
SPINLW1	1.81942793	1.08788721	-0.7419559	4.28E-07	2.54E-06
VIP	1.60573964	1.05210639	-0.6099574	1.15E-07	7.87E-07
