# Supplementary material for: Insight of a Metabolic Prognostic Model to Identify Tumor Environment and Drug Vulnerability for Lung Adenocarcinoma
Source: Front Immunol. 2022 Jun 23;13:872910. doi: 10.3389/fimmu.2022.872910 (PMC9262104; doi:10.3389/fimmu.2022.872910)
Supplement: Supplementary file 14 [file DataSheet_13.pdf]

Supplementary Table S13: The correlations between Metabolism Score, expression of 14 hub

| Gene | Drug | cor | pvalue |
|---|---|---|---|
| GCDH | Nelarabine | 0.567289299 | 5.14E-06 |
| GCDH | Chelerythrine | 0.550877765 | 4.10E-05 |
| GCDH | Cyclophosphamide | 0.544341874 | 7.65E-05 |
| TRDMT1 | Chelerythrine | 0.485474213 | 0.000406562 |
| TRDMT1 | Nelarabine | 0.483735378 | 0.00015877 |
| GCDH | Hydroxyurea | 0.478036126 | 0.000112089 |
| TKFC | SR16157 | 0.449677779 | 0.000398196 |
| DTYMK | Asparaginase | 0.446772245 | 0.000345703 |
| GCDH | Melphalan | 0.437479465 | 0.00047345 |
| DTYMK | Dexamethasone Decadron | 0.433660847 | 0.002920494 |
| UAP1 | Dexamethasone Decadron | 0.424605319 | 0.003647603 |
| TKFC | Fulvestrant | 0.422765395 | 0.000946076 |
| PPOX | Vorinostat | 0.421599198 | 0.000882385 |
| GCDH | Pipobroman | 0.419834738 | 0.00083971 |
| GCDH | Dexamethasone Decadron | 0.417539207 | 0.004320725 |
| GCDH | Cladribine | 0.417249129 | 0.001010002 |
| PPOX | Nelarabine | 0.416597257 | 0.001404316 |
| DTYMK | Nelarabine | 0.41147099 | 0.001629659 |
| GCDH | Raloxifene | 0.411371852 | 0.001208794 |
| DTYMK | Chelerythrine | 0.403262738 | 0.004065044 |
| GCDH | Carmustine | 0.402020412 | 0.001452417 |
| HEMK1 | Nelarabine | 0.401377243 | 0.002169923 |
| NEU1 | Palbociclib | -0.40007327 | 0.00169227 |
| WARS2 | Olaparib | -0.411423228 | 0.001805008 |
| NNT | Lificguat | -0.426618874 | 0.000753331 |
| NEU1 | Oxaliplatin | -0.458196746 | 0.000819566 |
| GCDH | Dasatinib | -0.505227016 | 4.48196E-05 |
| RiskScore | Rapamycin | -0.3643 | 0.0042 |
| | Arsenic trioxide | -0.3596 | 0.0052 |
| | Calusterone | -0.3578 | 0.0054 |
| | Pyrazoloacridine | 0.401 | 0.0057 |
| | Rapamycin | -0.3513 | 0.0074 |
| | Temsirolimus | -0.3463 | 0.0077 |
| | Estramustine | -0.3401 | 0.0084 |
| | Denileukin Diftitox Ontak | -0.337 | 0.0085 |
| | Everolimus | -0.3273 | 0.0114 |
| | Imexon | -0.3204 | 0.0134 |
| | Dacarbazine | -0.3155 | 0.0141 |
| | kahalide f | 0.3416 | 0.0142 |
| | Idelalisib | -0.3116 | 0.0173 |
| | Arsenic trioxide | -0.2993 | 0.0202 |
| | Olaparib | -0.2996 | 0.0263 |
| | Arsenic trioxide | -0.2734 | 0.0362 |
| | Amonafide | 0.2662 | 0.0416 |
| | Nandrolone phenpropionate | -0.2637 | 0.0436 |
| | Raloxifene | -0.2611 | 0.0457 |
| | Cisplatin | -0.2585 | 0.0461 |

ɔ genes,and drug susceptibility.
